# Biological Therapy and Small Molecules for Adults With Crohn's Disease: Systematic Review and Network Meta‐Analysis

**DOI:** 10.1002/phar.70049

**Published:** 2025-08-07

**Authors:** Daniela Gorski, Raul Edison Luna Lazo, Dalton de Assis de Souza, Helena Hiemisch Lobo Borba, Roberto Pontarolo, Fernanda Stumpf Tonin

**Affiliations:** ^1^ Pharmaceutical Sciences Postgraduate Program, Health Sciences Sector Universidade Federal do Paraná Curitiba Brazil; ^2^ Department of Pharmacy Universidade Federal do Paraná Curitiba Brazil; ^3^ Pharmacy and Pharmaceutical Technology Department, Faculty of Pharmacy University of Granada Granada Spain

**Keywords:** CINeMA, interleukin inhibitors, monoclonal antibody, RCT comparison

## Abstract

First‐line therapeutic approaches for Crohn's disease include immunosuppressants, aminosalicylates, and corticosteroids. However, more than one‐third of patients are resistant to these treatments and require second‐line therapies. Our goal was to synthesize the evidence on the efficacy and safety of biologics and small molecules for inducing remission in patients with moderate‐to‐severe Crohn's disease. A systematic review was conducted by searching for randomized controlled trials on the target population in PubMed, Scopus, and Web of Science (March 2025). Data synthesis for the outcomes of remission, health‐related quality of life (HRQoL), and safety was performed using network meta‐analyses and surface under the cumulative rating curve (SUCRA) analyses. The results were presented as risk ratios with 95% credible intervals. We included 55 trials (*n* = 16,113 patients) evaluating 26 biological drugs across 83 doses and six small molecules across 15 doses. Similar results were obtained in the sensitivity analyses conducted across different measurement time points. Alongside infliximab 5 mg/kg (SUCRA 98.6%), 10 mg/kg (92%), and 20 mg/kg intravenous (91.8%), the recently approved drugs guselkumab 1200 mg (83.2%), 600 mg (89.2%), and 200 mg intravenous (90.1%), as well as mirikizumab 600 mg (91.5%) and 1000 mg intravenous (82.4%) presented higher probabilities of disease remission and were associated with increased HRQoL. Drugs such as certolizumab, andecaliximab, fontolizumab, abatacept, and etanercept ranked low for remission (SUCRA < 40%) and presented high probabilities of serious adverse events (over 60%). Small molecules presented an intermediate profile. Inhibitors of interleukin‐23 appear to be promising alternatives for the treatment of moderate‐to‐severe Crohn's disease. Given their safety profile, some anti‐TNF drugs should be avoided in practice.

**Trial Registration:** PROSPERO: CRD42024519150

## 1 Introduction

Crohn's disease is a chronic inflammatory bowel disease (IBD) that is characterized by a course that is remitting–relapsing [1], being highly prevalent in high‐ and middle‐income countries, with incidence rates of 29.3 per 100,000 person‐years in Australia, 23.8–82 per 100,000 person‐years in Canada, and 13.9 per 100,000 person‐years in the United States [2].

The initial therapeutic strategy for managing moderate‐to‐severe Crohn's disease typically involves early introduction of biologic agents, often in combination with immunosuppressants or corticosteroids, depending on disease severity and patient profile [3, 4]. However, approximately 30%–50% of patients experience intolerance, insufficient response, or refractory symptoms or relapse [5]. Studies have demonstrated that only 30% of patients treated with thiopurines respond to therapy [6], while less than half (45%) of those treated with sulfasalazine achieve remission [7]. Moreover, increasing rates of treatment failure with biological agents, drugs such as conventional anti‐TNF agents (e.g., infliximab, adalimumab, and certolizumab), have been reported in 20%–30% of cases in recent years, with an additional 40%–50% experiencing secondary treatment failures [8], including to interleukin (IL)‐23 inhibitors, such as ustekinumab [8]. The use of these drugs, whether as monotherapy or in combination, has also been associated with a heightened risk of serious adverse events (SAE), including infections, which raises concerns regarding therapy safety and patient monitoring [9].

Given this scenario, further therapies beyond the approved and currently recommended biologics (e.g., infliximab, adalimumab, certolizumab, vedolizumab, ustekinumab, and natalizumab), including new IL inhibitors, such as IL‐23 p19 inhibitors (guselkumab, mirikizumab, risankizumab), and some classes of small molecules (low molecular weight [< 1 kDa]; e.g., Janus kinase inhibitors [JAKi], sphingosine 1‐phophate antagonists [S1Pa]) are under investigation [10, 11, 12, 13]. Yet, with this overdue increase in the IBD treatment pipeline, it is essential to establish a robust body of evidence on the efficacy, safety, and impact on the quality of life of patients using these interventions, with the aim of supporting more confident decision‐making processes at both the societal and individual level. However, updated comparative evidence on the effects of biologics and small molecules for Crohn's disease comes primarily from systematic reviews with meta‐analyses limited to patients previously treated with biologics [14], focused only on some selected therapies [14, 15, 16, 17] using limited data for some drugs, [18] or restricted to articles' publication dates [19]. Additionally, assessments of drug doses, routes of administration, and the impact of these therapies on quality of life are lacking [14, 15, 16, 17, 18, 19].

Thus, given these gaps in the literature, we aimed to synthesize and critically appraise the most up‐to‐date evidence on the effects of all available biological and small molecules for managing moderate‐to‐severe Crohn's disease in adult patients by means of a broad systematic review with network meta‐analysis (NMA).

## 2 Methods

This study was conducted according to the recommendations of the Cochrane Collaboration and reported using the PRISMA (Preferred Reporting Items for Systematic Reviews and Meta‐Analyses) checklist [20, 21, 22]. A project has been created on the Open Science Framework (OSF) to make the Supporting Information publicly available under the DOI: https://doi.org/10.17605/OSF.IO/4DKT9. The selection, data extraction, and risk of bias assessment steps of the studies were conducted independently by two authors. A third author was consulted in case of discrepancies.

### 2.1 Search Strategy

Systematic searches were performed in the electronic databases PubMed, Scopus, and Web of Science, with no restrictions on time frame or language, through March 2025. The detailed search strategy is available in Table S1. Trial registration databases (clinicaltrials.gov) and the reference lists of the included studies and other systematic reviews in the field were also consulted as part of the manual searching process.

### 2.2 Inclusion Criteria

After removing duplicates, the titles and abstracts of the retrieved records were examined using the Rayyan web application for systematic reviews [23]. The relevant records were then read in full by the reviewers, and primary studies that met the following criteria (PICOS acronym) were included for data extraction and analyses:

1. Population: Adult patients (≥ 18 years) diagnosed with moderate‐to‐severe Crohn's disease.
2. Intervention: Induction treatment with any biological therapy or small molecules (JAKi, IL Inhibitors, or S1Pa receptor modulator) at any regimen.
3. Comparator: Head‐to‐head comparison to another biological, small molecule, or placebo.
4. Outcomes: Crohn's Disease Activity Index (CDAI) for clinical remission (CDAI ≤ 150), clinical response (CDAI degrees ≥ 70 or ≥ 100), health‐related quality of life (HRQoL), or any safety outcome.
5. Study design: Randomized controlled trials (RCTs).

Studies in which patients continued using prior treatments (e.g., azathioprine and/or corticosteroids) during the study were included, provided that the study reported these treatments were stable before the initiation of the intervention.

### 2.3 Exclusion Criteria

The following studies were excluded: those targeting specific subgroups of patients with complications (e.g., complex perianal fistulas); those targeting postoperative patients; those targeting pregnant women; those targeting patients with compromised immune systems or related comorbidities; those evaluating biosimilars; those evaluating only the maintenance treatment; congress abstracts; and those published in non‐Roman characters.

### 2.4 Data Extraction

Data from each included study were independently extracted by the two researchers into a standardized Microsoft Excel Office 365 spreadsheet (Microsoft Corp, Redmond, WA, USA), comprising information on trial design and participant characteristics (e.g., authors' names, year of publication, country, number of centers, sample size, trials' duration, patient's gender, age, clinical condition, follow‐up); details of the intervention (therapy, dosage, regimen, route of administration); HRQoL (inflammatory bowel disease questionnaire [IBDQ]) and efficacy (clinical remission and response) or safety outcome results (incidence of any adverse event or SAE); and times of measurement. Outcome results reported only using graphs or figures in the original articles were extracted using an online tool PlotDigitizer (https://plotdigitizer.com/).

### 2.5 Quality Assessment

To assess the risk of bias of included studies, the Cochrane Collaboration's tool for assessing the risk of bias in RCTs of interventions (RoB2) was employed [24]. This tool evaluates five main sources of bias per outcome comparison: selection, performance, detection, attrition, and reporting bias. Domains were judged as having a “low risk of bias,” “some concerns,” or “high risk of bias”.

### 2.6 Statistical Analysis

A narrative synthesis of the findings from the included studies, structured around the type of intervention, target population characteristics, and type of outcome, is provided in the tables. Additionally, both pairwise and NMAs were performed for each outcome of interest (clinical remission, clinical response, safety profile considering SAE). All analyses were conducted using the R/Rstudio software (version 4.3.3), built in the packages “network,” “gemtc” [25], and “netmeta” [26].

The NMA was constructed using a Bayesian approach. Transitivity was assessed through visual inspection of patient characteristics and comparison of the intervention, comparator, and outcome definitions across studies, based on the PICOS framework. To preserve transitivity, treatments were considered equivalent for comparison purposes only when both dosage and duration matched.

The geometry of the network was consistent with the complexity level of the primary studies (i.e., arm‐level entry data) to ensure the integrity of the treatment arms provided by the included trials and to avoid potential biases. A conservative analysis of noninformative priors was employed. The convergence of the analysis was determined through the visual assessment of the Brooks–Gelman–Rubin plots and the potential scale reduction factor (PSRF), which was maintained between 1 and 1.05. The results are presented as league tables, with dichotomous data reported as risk ratio (RR) and continuous data as mean difference (MD) with their 95% credible intervals (CrIs). The surface under the cumulative rating curve analysis (SUCRA) was used to establish rank probabilities involving all treatment options for each outcome. To evaluate the robustness of the networks, inconsistency, defined as the difference between the direct and indirect estimates for a particular comparison, was assessed through node‐splitting analysis (values > 0.05 indicate incoherences). Publication bias was assessed by visual inspection of funnel plots. The geometry parameters of the networks was also assessed [27].

The primary meta‐analyses were conducted using data from the final week of outcome reporting in each study. To evaluate the potential impact of varying assessment time points, sensitivity analyses were performed using alternative measurement times. Additional sensitivity analyses were also conducted based on clinical response, approved drugs, and types of adverse reactions to confirm the robustness of the main findings. Subgroup analyses according to patients' prior use of therapies (exclusively used biological drugs vs. naïve patients) were also performed.

### 2.7 Certainty of Evidence Evaluation

The Confidence in Network Meta‐Analysis (CINeMA) web application was used to assess the certainty of the evidence for each outcome of interest in the network. This semiautomated framework is based on the Grading of Recommendations, Assessment, Development, and Evaluations (GRADE) approach for evidence rating [28]. Outcomes for a given comparison were rated as having “no concerns,” “some concerns,” or “major concerns” based on six main criteria: (i) within‐study bias, (ii) reporting bias, (iii) indirectness, (iv) imprecision, (v) heterogeneity, and (vi) inconsistency. The evaluation of the domains was summarized in a single confidence rating to improve data interpretability as (i) high, (ii) moderate, (iii) low, or (iv) very low certainty [28].

## 3 Results

**FIGURE 1:**
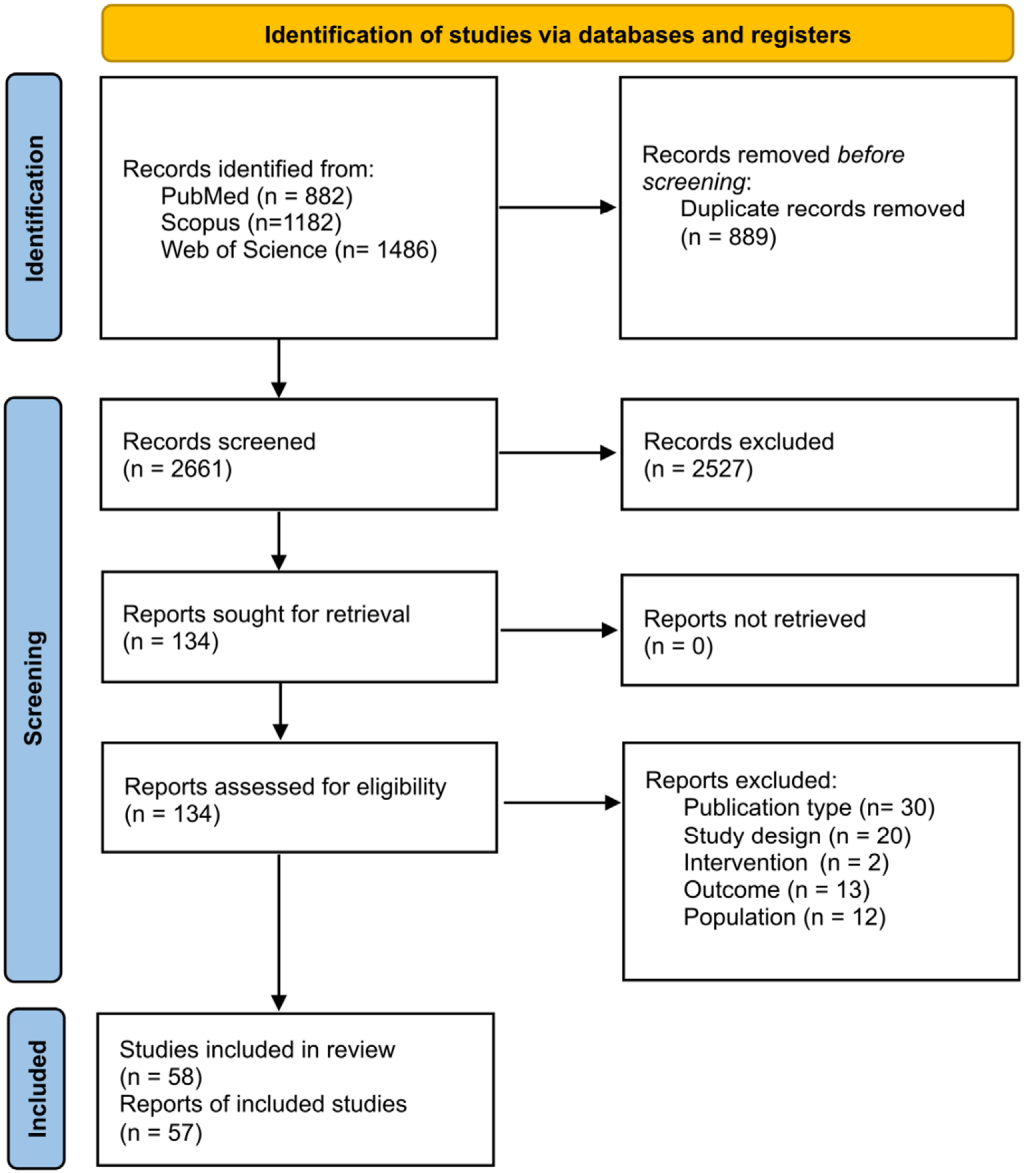
Systematic review flowchart.

The search strategy identified 2661 unique records, of which 2527 were excluded during the screening process, with 134 records being fully appraised. Seventy‐seven records were excluded after full‐text appraisal (see complete list with reasons for exclusion in Table S2), and the remaining 57 records, accounting for 58 RCTs (Figure 1) were selected for data extraction and analyses (see complete list in Table S3). No additional studies were found through manual research.

These 58 RCTs (*n* = 16,113 patients), published between 1997 and 2025, were mostly multinational and multicentric (*n* = 51; 87.9%) trials performed in the United States (*n* = 38), Belgium (*n* = 37), and Canada (*n* = 36). Two studies did not report on their location [29, 30]. The patients' mean age ranged from 27 to 44 years, with a slight predominance of females (54.7%) and biologically prior patients (62.6%) (Table S4).

Overall, 26 biologic drugs distributed across 11 classes were assessed: IL inhibitors (*n* = 16 studies; risankizumab, PF‐04236921, ustekinumab, secukinumab, guselkumab, MEDI2070, mirikizumab, briakinumab, and brodalumab), anti‐TNF (*n* = 17; adalimumab, onercept certolizumab, etanercept, CDP571, and infliximab), natural Killer Group 2D inhibitors (Anti‐NKG2D; *n* = 2; NNC0142‐0002 and tesnatilimab), integrin antagonists (*n* = 8; vedolizumab, natalizumab), anti‐CD3 (*n* = 1; NI‐0401), anti‐IFN‐γ (*n* = 2; fontolizumab), ICAM‐1 inhibitors (*n* = 1; alicaforsen), MMP9 inhibitors (*n* = 1; andecaliximab), IP‐10 inhibitors (*n* = 1; eldelumab), MAdCAM‐1 inhibitors (*n* = 3; ontamalimab and etrolizumab), and inhibitors of T‐cell activation (*n* = 1; abatacept). Regarding the small molecules, six drugs distributed across four classes were assessed: JAKi (*n* = 6 studies; upadacitinib, tofacitinib, filgotinib), IL inhibitor (*n* = 1; apilimod), toll‐like receptor (*n* = 1; semapimod), and S1Pa (*n* = 1; amiselimod). Ninety‐eight different dosages were recorded, with treatment durations ranging from 1 day to 24 weeks (most durations were over 4 weeks). The intravenous route of administration was the most prevalent, accounting for 51.8% of cases. Subcutaneous administration was the second most common, representing 31% of cases, while oral administration accounted for the remaining 13.8%. Only two trials [31] (3.4%) used intravenous therapy for the initial administration, followed by subsequent subcutaneous applications of the drug (see Table S4).

Overall, the risk of bias of the trials was judged as moderate‐to‐high, with some pitfalls regarding study design, outcome evaluation, and reporting for both CDAI, HRQoL, and safety data (57.9%, 50%, and 46.4% of “some concerns,” respectively; Tables S5–S7). All studies were randomized, and 98.2% were double blind (*n* = 56). Thirty studies (52.6%) provided clear descriptions of the blinding process, and 38 studies (66.7%) described the randomization procedures. Thirty‐one studies (53.4%) provided a protocol or registry. Eight trials (13.8%) were considered to have a high risk of bias for safety outcomes, and six (23.1%) studies had a high risk of bias for HRQoL due to the lack of information on the methods used to define and measure the outcomes (Tables S5 and S6). One study had a high risk of bias for the CDAI outcome due to potential deviation from the intended intervention [32] (Table S7).

**FIGURE 2:**
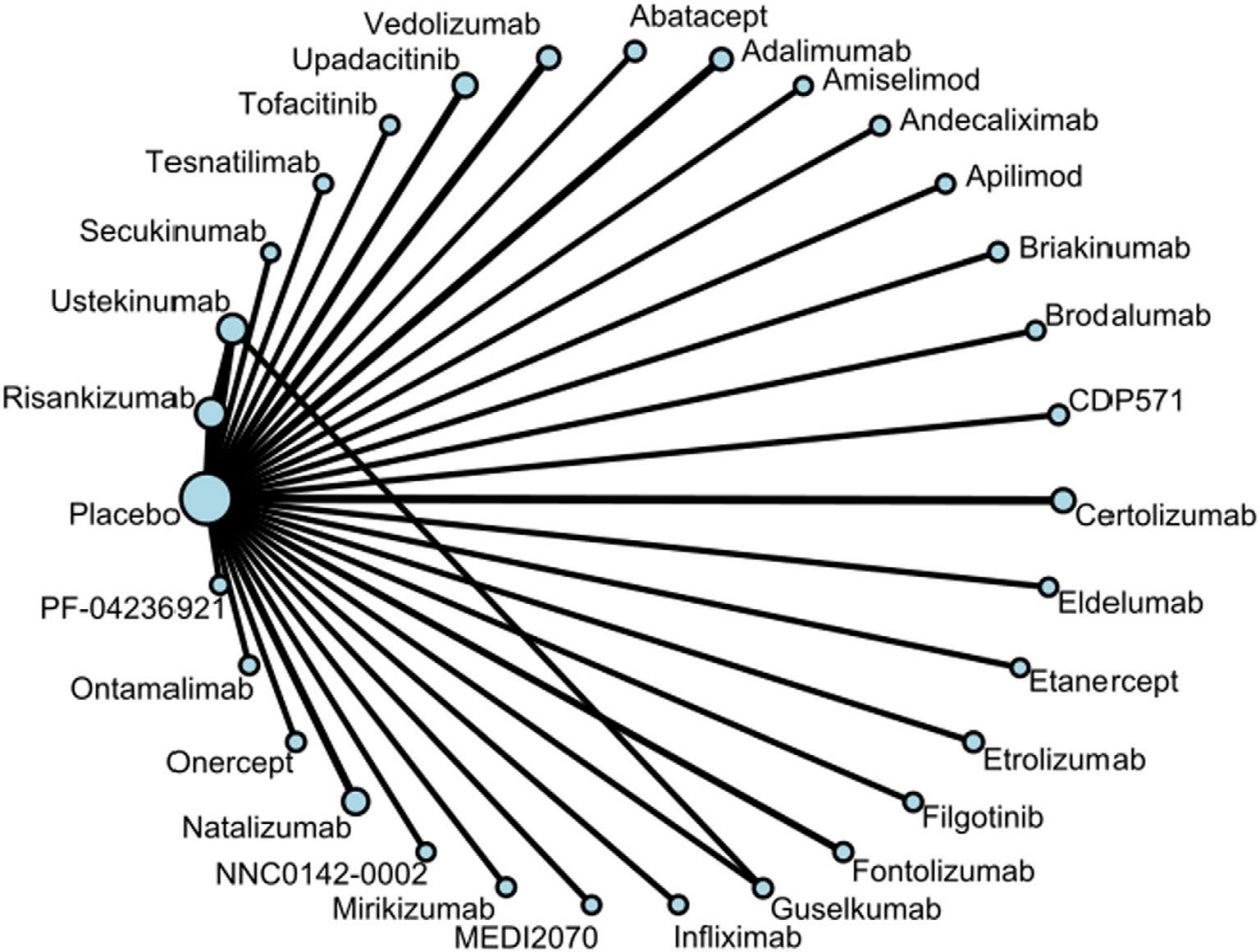
Overall network plot of treatment comparisons.

Four main networks for the outcomes related to CDAI (clinical remission, clinical response), HRQoL (measured by IBDQ), and SAE were built with additional subgroup NMA performed whenever necessary. All the networks converged (Table S8). No major differences in age, gender, geographic location, or disease duration were identified across studies, supporting the assumption of transitivity. The robustness of the NMA was evaluated through node‐splitting analysis, which revealed no inconsistencies within the network (Table S9). The geometry of the networks revealed the limited available evidence for most comparisons between the drugs (Table S10). No important publication bias was detected (Figure S1). A broad representation of the network of comparisons is depicted in Figure 2. The treatment and dosage abbreviations used in the NMAs are listed in Table S11.

Overall, 55 trials were included in the NMA of clinical remission (*n* = 16,467 patients), summarizing the effect of 31 drugs at 91 different doses (Figure S2). The NMA league table revealed that most of the drugs were statistically superior to placebo. The anti‐TNF infliximab, especially the 5 mg/kg dose (RR 15.3 [CrI 2.9–138.05]), and adalimumab 160/80/60 mg intravenous had the highest rates of remission (RR ranging 15.30–5.65), followed by IL inhibitors mirikizumab (600 mg intravenous), guselkumab (200, 600, and 1200 mg), and ustekinumab (6 mg/kg intravenous followed by 90 mg subcutaneous; RR ranging from 4.44 to 2.86; see complete results in Table S12). According to the SUCRA analysis, the top‐performing drugs were as follows: infliximab 5, 10, and 20 mg/kg intravenous (SUCRA probabilities of 98.6%, 92%, and 91.8%, respectively); adalimumab 160/80/60, 160/80, and 160/80/40 mg subcutaneous (95.1%, 82.7%, and 81.3%, respectively); mirikizumab 600 and 1000 mg intravenous (91.5% and 82.4%, respectively); guselkumab 200, 600, and 1200 mg intravenous (90.1%, 89.2%, and 83.2%, respectively); and ustekinumab 6 mg/kg intravenous followed by 90 mg subcutaneous (83.3%). Conversely, the lowest SUCRA probabilities were attributed to certolizumab 20 mg/kg intravenous (7.7%); andecaliximab 300 mg subcutaneous (13.6%); apilimod 50 and 100 mg oral (11.5% and 20%, respectively); fontolizumab 0.1 mg/kg intravenous (15.4%); etanercept 25 mg (14.2%); onercept 25 mg subcutaneous (14.6%); abatacept 10 mg/kg intravenous (15.9%); amiselimod 0.4 mg oral (17%); and brodalumab 210 mg intravenous (16.5%). Drugs such as PF‐04236921 (50 mg subcutaneous), brodalumab (350 mg intravenous), briakinumab (400 mg intravenous), upadacitinib (6 mg twice daily oral), vedolizumab (300 mg intravenous), and filgotinib (200 mg oral) presented intermediate profiles (Table S13). The sensitivity analyses regarding only approved drugs and the timing of measurement at 4, 6, 8, and 12 weeks revealed similar results for clinical remission (see complete analyses in Tables S12 and S13).

The subgroup analyses of clinical remission revealed similar results both for patients who previously used biologics (21 trials [*n* = 5835 patients] evaluating 16 drugs) and for those who were treatment naïve (15 trials [*n* = 3010 patients] evaluating 12 drugs), highlighting positive effects mostly from the IL inhibitors in the first subgroup and from infliximab and mirikizumab in the second subgroup of patients (see complete analyses in Tables S12 and S13).

The meta‐analyses on clinical response (reductions of 100 and 70 points in the CDAI) showed similar trends as those from clinical remission, both in the original and subgroup analyses according to patients' previous use of biologics. The original networks encompassed 40 studies each (*n* = 12,537 patients) on the effects of 25 drugs across 66 doses. Infliximab, adalimumab, guselkumab, and ustekinumab (at different doses and regimens) were significantly superior to the placebo (Tables S12 and S13) and other active drugs, with SUCRA above 80%. Conversely, drugs such as fontolizumab, certolizumab, ontamalimab, onercept, secukinumab, and abatacept presented SUCRA < 20% for at least one dose or regimen (Tables S12 and S13).

For HRQoL analysis, the NMA conducted for the increase in IBDQ score included 12 trials (*n* = 3828 patients), summarizing 10 drugs at 23 different doses. The league table revealed upadacitinib (6 and 24 mg oral twice daily and 45 mg oral), risankizumab (600 mg intravenous), and infliximab (all doses) as statistically superior to placebo (MD ranging 41.10–23.09) with infliximab 5 mg intravenous presenting the highest rates of increased HRQoL (MD −41.10 [CrI 18.45–62.65], SUCRA 95.1%; Tables S14 and S15). Conversely, semapimod (14.4%) and fontolizumab (18.8%–38.6%) had the lowest probabilities of improving patients' HRQoL. For the IBDQ remission (IBDQ score ≥ 170) the NMA included seven studies (*n* = 3050 patients) summarizing six drugs at 12 doses. Only ustekinumab 6 mg/kg intravenous was statistically superior to placebo (RR 4.24 [CrI 1.32–17.72]), with a SUCRA of 83.3%. Other doses of ustekinumab (6 mg/kg intravenous followed by 90 mg by subcutaneous and 130 mg intravenous) as well as upadacitinib 24 mg oral and guselkumab (all doses) presented an intermediate SUCRA profile with probabilities above 45%. Conversely, tofacitinib 15 mg oral twice daily presented the lowest SUCRA (29.3%; Tables S14 and S15). Similar results were obtained in the sensitivity analyses of IBDQ response (improvement of 16 points compared to baseline; see complete analyses in Tables S14 and S15).

Finally, the NMA on SAEs included 49 studies (*n* = 15,057 patients) reporting data on 30 drugs across 83 dosages. Few significant differences between drugs were found. Risankizumab 600 and 1200 mg intravenous were significantly safer than placebo (RR ranging 0.27–0.43), whereas amiselimod 0.4 mg oral, brodalumab 700 and 350 mg intravenous, tesnatilimab 400/200 mg subcutaneous, and upadacitinib 12 mg twice daily were significantly riskier than placebo for this outcome (see complete analyses in Table S16). According to SUCRA, risankizumab 1200 mg intravenous (SUCRA probabilities 12%), mirikizumab 200 and 1000 mg intravenous (14.8% and 14.4%, respectively), and guselkumab 1200 mg (13.7%), demonstrated better safety profiles. The anti‐TNF agent adalimumab 80/40 and 40/20 mg subcutaneous (12.9% and 14%, respectively), the MMP9 inhibitor andecaliximab 150 mg subcutaneous (11.2%), and the JAKi tofacitinib 15 mg oral twice daily (10.5%) were also ranked among the safest options. On the other hand, brodalumab 700 and 350 mg intravenous (89.1% and 87.1%, respectively), upadacitinib 12 mg oral twice daily and 24 mg oral (91% and 85.4%, respectively), amiselimod 0.4 mg oral (90.7%), and tesnatilimab 400/200 mg subcutaneous (94%) were the least safe alternatives (Table S17). Additional sensitivity analyses considering the incidence of any adverse event and infection yielded similar results (see Tables S16 and S17).

Due to the moderate‐to‐high risk of bias of the included studies, a sensitivity analysis was conducted with subgroup analyses for low, some, and high risk for CDAI remission, IBDQ score, and SAE outcome. These analyses showed similar results to those found in the main analysis (Table S18).

**FIGURE 3:**
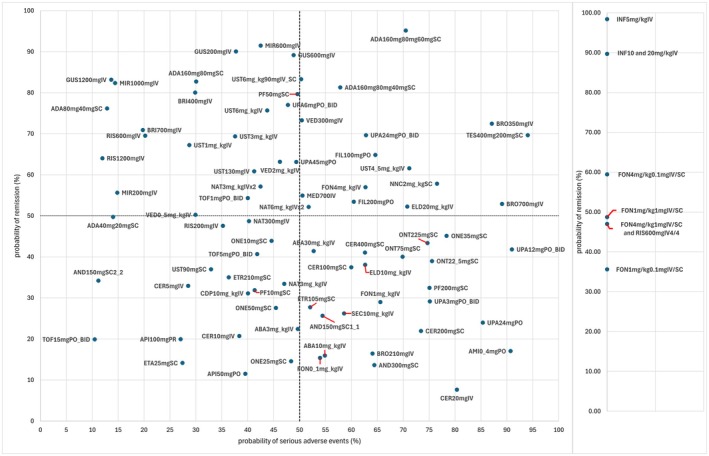
Comparison of clinical remission probability and serious adverse effects probability. Data based on SUCRA from network meta‐analysis. ABA, abatacept; ADA, adalimumab; AMI, amiselimod; AND, andecaliximab; BRI, briakinumab; BRO, brodalumab; CDP, CDP571; CER, certolizumab pegol; ELD, eldelumab; ETA, etanercept; ETR, etrolizumab; FIL, filgotinib; FON, fontolizumab; GUS, guselkumab; INF, infliximab; MED, MEDI2070; MIR, mirikizumab; NAT, natalizumab; NNC, NNC0142‐0002; ONE, onercept; ONT, ontamalimab; PF, PF‐0423921; RIS, risankizumab; SEC, secukinumab; TES, tesnatilimab; TOF, tofacitinib; UPA, upadacitinib; UST, ustekinumab; VED, vedolizumab.

To improve interpretability and decision‐making, Figure 3 correlates the NMA results (SUCRA probabilities) of the two main outcomes of clinical remission and SAE incidence. Drugs displayed in the left upper quadrant, such as infliximab, guselkumab, mirikizumab, adalimumab, upadacitinib, and risankizumab, are associated with better benefit–risk balance, whereas certolizumab, andecaliximab, abatacept, amiselimod, and secukinumab presented the least favorable profiles (lower right quadrant). The main findings for the prior and naïve treatment populations are summarized in Table 1.

**TABLE 1:** Main findings on efficacy and safety by population.

Population	Treatment	Dose	SUCRA efficacy (%)^a^	SUCRA safety (%)^b^	Main findings
Treatment‐naïve	Infliximab	5 mg/kg IV 10 mg/kg IV 15 mg/kg IV	> 70.0%^c^	—	Previously approved for this condition; demonstrated good efficacy. No data available on the safety profile of different dosing regimens. The 5 mg/kg IV dose showed the best efficacy profile (SUCRA 89.5%)
	Adalimumab	160/80/60 mg SC	76.5%	57.9%	Previously approved for this condition; demonstrated a good efficacy profile and an intermediary safety profile
	Mirikizumab	1000 mg IV 600 mg IV	> 70.0%^c^	< 45.0%^c^	Not yet approved for CD; showed promising potential in terms of efficacy and safety
	Filgotinib	200 mg PO	75.5%	60.5%	Best efficacy profile among oral therapies; however, with an unfavorable safety profile
	Certolizumab pegol	400 mg SC	32.9%	62.6%	Previously approved for this condition; demonstrated an unfavorable efficacy and safety profiles
Previously treated	Guselkumab	200 mg IV 600 mg IV 1200 mg IV	> 70.0%^c^	< 50.0%^c^	Not yet approved for CD; showed promising potential in terms of efficacy and safety. The 200 mg IV dose showed the best efficacy profile (93.1%) and an intermediary safety profile (37.7%)
	Mirikizumab	600 mg IV	74.1%	42.5%	Not yet approved for CD; showed promising potential in terms of efficacy and safety
	Adalimumab	80/40 mg SC	88.0%	12.9%	Previously approved for this condition; demonstrated good efficacy and safety profile
	Upadacitinib	45 mg PO	41.3%	49.4%	Previously approved for this condition; showed a low to intermediate efficacy, but stands out as one of the better options among oral treatments

Abbreviations: CD, Crohn disease; IV, intravenous; PO, oral; SC, subcutaneous; SUCRA, surface under the cumulative rating curve analysis.

^a^ SUCRA for clinical remission; higher probabilities indicate better‐performing interventions.

^b^ SUCRA for serious adverse events; lower probabilities indicate better‐performing interventions.

^c^ Mean SUCRA value considering different doses.

The certainty of the evidence (CINeMA approach) for the comparisons of the different treatment regimens within the primary efficacy network (clinical remission) was globally classified as low (73.3% of the cases), with a moderate level in 26.7% of the comparisons (Table S19). Treatment comparisons relating to safety outcomes were also considered to have low (58.6%) or moderate (31%) levels of evidence. Regarding HRQoL (IBDQ), the evidence was classified as very low (60%) or low (40%). The primary reasons for downgrading the certainty of evidence stemmed from the low methodological quality of some studies and imprecision of the results (large CrI in the network; small number of available studies per comparison). The results for each evaluated drug are summarized in Table 2.

**TABLE 2:** Confidence of evidence summary (by drug) in the network meta‐analyses.

Treatment	CDAI^a^	Safety—AE^b^	HRQoL^b^
Abatacept	⊕⊕◯◯	⊕⊕◯◯	—
Adalimumab	⊕⊕⊕◯	⊕⊕◯◯	—
Amiselimod	⊕⊕◯◯	⊕⊕◯◯	—
Andecaliximab	⊕⊕◯◯	⊕⊕◯◯	—
Apilimod	⊕⊕◯◯	⊕⊕◯◯	—
Briakinumab	⊕⊕◯◯	⊕⊕◯◯	—
Brodalumab	⊕⊕◯◯	⊕⊕◯◯	—
CDP571	⊕⊕◯◯	⊕⊕◯◯	—
Certolizumab pegol	⊕⊕◯◯	⊕⊕◯◯	⊕⊕◯◯◯
Eldelumab	⊕⊕◯◯	⊕⊕◯◯	—
Etanercept	⊕⊕◯◯	⊕⊕◯◯	—
Etrolizumab	⊕⊕◯◯	—	
Filgotinib	⊕⊕⊕◯	⊕⊕◯◯	⊕◯◯◯
Fontolizumab	⊕⊕◯◯	⊕⊕⊕◯	⊕◯◯◯
Guselkumab	⊕⊕⊕◯	⊕⊕⊕◯	—
Infliximab	⊕⊕◯◯	—	⊕◯◯◯
MEDI2070	⊕⊕⊕◯	⊕⊕⊕◯	—
Mirikizumab	⊕⊕⊕◯	⊕⊕◯◯	—
Natalizumab	⊕⊕◯◯	⊕⊕◯◯	⊕◯◯◯
NNC0142‐0002	⊕⊕◯◯	⊕⊕◯◯	—
Onercept	⊕⊕◯◯	⊕⊕◯◯	—
Ontamalimab	⊕⊕⊕◯	⊕⊕⊕◯	—
Placebo	⊕⊕⊕◯	⊕⊕⊕◯	⊕◯◯◯
PF‐04236921	⊕⊕◯◯	⊕◯◯◯	—
Risankizumab	⊕⊕◯◯	⊕⊕⊕◯	⊕⊕◯◯
Secukinumab	⊕⊕⊕◯	⊕⊕⊕◯	—
Semapimod	—	—	⊕◯◯◯
Tesnatilimab	⊕⊕◯◯	⊕⊕⊕◯	—
Tofacitinib	⊕⊕⊕◯	⊕⊕⊕◯	—
Upadacitinib	⊕⊕⊕◯	⊕⊕⊕◯	⊕⊕◯◯
Ustekinumab	⊕⊕◯◯	⊕⊕◯◯	⊕⊕◯◯
Vedolizumab	⊕⊕◯◯	⊕⊕⊕◯	—

*Note:* ⨁◯◯◯: critical low certainty of evidence; ⨁⨁◯◯: low certainty of evidence; ⨁⨁⨁◯: moderate certainty of evidence.

Abbreviations: AE, adverse events; CDAI, Crohn's Disease Activity Index; HRQoL, health‐related quality of life.

^a^ Critical outcome.

^b^ Important outcome.

## 4 Discussion

This broad and updated systematic review with NMA synthesized and critically appraised the methodological quality and certainty of evidence of over 58 RCTs published until 2025 on the profile of 26 different biological drugs at 83 doses/regimens and six different small molecules at 15 doses/regimens, both in an overall population of adults with moderate‐to‐severe Crohn's disease, as well as in subgroups of patients with and without prior biologic use. The findings, presented in a unified model, have the potential to provide policymakers, practitioners, and other stakeholders with a more comprehensive understanding of the available evidence concerning the benefit–risk ratio of these drugs, including novel ones. This enhanced knowledge can serve as a foundation for updating treatment guidelines, with the aim of enhancing both clinical and humanistic outcomes. The updated guidelines would also contribute to reducing drug intolerance, lack of response, and safety monitoring issues. Furthermore, these results can serve as a foundation for the development of additional well‐designed, standard‐reporting, and head‐to‐head primary studies in this field, given the moderate methodological quality of most studies.

Although previous systematic reviews and NMAs have highlighted the role of anti‐TNF drugs and IL inhibitors in the clinical remission or response of patients with Crohn's disease, the evidence is still controversial, especially regarding drug doses and routes of administration, and is often limited to a few biologics (adalimumab, natalizumab, vedolizumab, ustekinumab, certolizumab pegol, infliximab, risankizumab) [14, 15]. Our review provides insights into the effects of 15 additional biologics (PF‐04236921, secukinumab, MEDI2070, mirikizumab, brodalumab, onercept, etanercept, CDP571, tesnatilimab, fontolizumab, alicaforsen, andecaliximab, eldelumab, ontamalimab, and abatacept) and five small molecules (semapimod, tofacitinib, filgotinib, apilimod, and amiselimod) for managing this disease that were not previously assessed using NMA. This growing number of clinical trials evaluating new drugs reflects efforts to develop more selective interventions to minimize the risk of off‐target/adverse effects and increase efficacy [33].

Our results suggest that recent second‐generation selective IL inhibitors, in a preference order of guselkumab (especially in patients who failed previous biologics) and mirikizumab (particularly for biologics‐naïve patients), as well as some anti‐TNF drugs (preferably infliximab or adalimumab), should be considered target approaches for this population. However, these treatments are not yet recommended in any published international guidelines [3, 4]. Of previous studies, only one review evaluated guselkumab treatment, showing a high success rate of 79%. Nevertheless, this intervention was only included in the main NMA as a sensitivity analysis [15]. Both guselkumab (200 and 1200 mg intravenous) and mirikizumab (1000 mg intravenous) exhibited more favorable clinical profiles within all evaluated outcomes (moderate certainty of the evidence), including compared to ustekinumab, a first‐generation treatment entailing a mix of IL‐12 and IL‐23 inhibition, which may be attributed to their very specific IL‐23p19 antagonism [10]. In fact, IL‐23 plays a crucial role in the differentiation of Th‐17 cells, promoting the activation of the inflammatory cascade through a pathway mediated by Janus kinase and regulating the gut immune response, which contributes to the development and exacerbation of inflammatory conditions [34, 35]. Moreover, although the evidence on the long‐term safety of these drugs is still limited, especially in the context of IBD (currently no available recommendations in international clinical practice guidelines [3, 4, 36]), previous NMA on moderate‐to‐severe plaque psoriasis and ulcerative colitis highlighted their favorable safety profile [37, 38], with no significant differences compared to placebo for the incidence of SAE [38, 39]. According to the results of the GALAXI‐1 study, 43% of patients treated with guselkumab 1200 mg intravenous versus 60% of those in the placebo group reported at least one adverse event, mostly related to headache, anemia, and nasopharyngitis [10]. Similarly, the rates of overall adverse events are lower than 65.6% with the use of mirikizumab, with < 10% of discontinuations due to these events [11].

Since the late 1990s, the anti‐TNF agents, infliximab and adalimumab, have been recommended as the first approaches to control Crohn's disease symptoms because they have demonstrated efficacy in inducing and maintaining remission, as confirmed by our NMA, preventing disease complications and achieving mucosal healing [33]. Conversely, the literature shows that approximately 50% of patients do not respond to TNF antagonists or experience a decline in response over time [40]. We were not able to build a robust NMA on the safety profile of infliximab given the lack of standardized data from the included primary studies (i.e., no information on SAE, no distinction on doses/regimen), which may hamper further conclusions on its true effects in Crohn's disease. Yet, Targan et al. [41] reported that 76% of patients (*n* = 83) treated with infliximab experienced at least one adverse event compared to 60% of those in the placebo group, whereas rates of at least one adverse event were 68%–75% for adalimumab. Among these two anti‐TNF agents, adalimumab may have fewer adverse effects (odds ratio [OR] 0.62 [95% confidence intervals [CI] 0.42–0.91]) [42], mostly related to infections, musculoskeletal disorders, and gastrointestinal disorders [43]. The 5‐year, prospective, observational ENCORE registry demonstrated that infliximab is significantly associated with an increased risk of serious infections (hazard ratio [HR] 1.64 [95% CI 1.17–2.31]) and hematological conditions (HR 2.91 [95% CI 1.51–5.59]) versus conventional therapy [44]. Sickness‐like reactions, sepsis, and autoimmune disorders have also been reported with the use of these drugs, which warrants the need for further monitoring of these patients [45], with additional changes in therapeutic strategies if necessary (e.g., dose tapering, shortening intervals, or combination with immunomodulators) [40].

In 2008, certolizumab pegol (another anti‐TNF alpha inhibitor) was approved by the US Food and Drug Administration (FDA) for treating patients with Crohn's disease. It is a single antigen‐binding fragment of a humanized IgG1 antigen‐specific monoclonal antibody conjugated to two 20 kDa polyethylene glycol chains, which increases the drug's half‐life [46]. However, compared to other biologics, certolizumab has a poorer clinical profile, as confirmed by our NMA, and is among the least effective and least safer drugs, with SUCRA < 20%. Singh et al. similarly demonstrated that certolizumab was comparatively less effective than infliximab, adalimumab, ustekinumab, risankizumab, and vedolizumab for inducing remission in adult patients [15] and was associated with important SAEs, especially infections [45]. In contrast to other anti‐TNF therapies, certolizumab pegol is a Fab fragment of a monoclonal antibody that lacks the Fc region. This structural difference results in a longer plasma half‐life and a distinct mechanism of action. Although other anti‐TNF agents mediate complement‐dependent and antibody‐dependent cell‐mediated cytotoxicity, certolizumab pegol primarily binds to and neutralizes human TNFα. This immunomodulatory activity may contribute to the higher incidence of adverse events observed with this biological drug. Therefore, certolizumab pegol should be avoided in clinical practice, especially since there are other alternative treatments available for Crohn's disease; if used, patients require close surveillance [46, 47].

In the past 5 years, new drugs, featuring mechanisms distinct from those of currently recommended biologics, have emerged for treating IBD. Although not yet approved by the FDA, the anti‐NKG2D NNC0142‐0002, an antagonizing human immunoglobulin G4 monoclonal antibody that binds to natural killer group 2 member D receptors, has successfully completed phase 2 studies for Crohn's disease and rheumatoid arthritis [48, 49]. It has shown important clinical benefits [49], particularly in biologically‐naïve patients, highlighting the role of therapies targeting T cells. Another biological development, the anti‐CD3 monoclonal antibody foralumab (NI‐0401), has demonstrated significant efficacy in adult patients with moderate‐to‐severe Crohn's disease when administered subcutaneously [50]. Investigations are underway regarding its oral administration, which could enhance patient adherence [51]. Nevertheless, given the limited clinical evidence on these and other new drugs (e.g., ontamalimab and eldelumab), it was not possible to properly compare them with other active treatments in the NMA. The evidence for these comparisons is of very low or low certainty, highlighting the need for further well‐designed clinical trials to confirm these preliminary results [49, 50].

Moreover, in addition to the clinical profile of biological therapies for Crohn's disease (summarized in Figure 3), other factors can influence patient compliance and treatment success, including therapy costs, duration, and route of administration. Recent monoclonal antibody drugs tend to be more expensive and not readily accessible, making interventions with intermediate therapeutic profiles, such as ustekinumab (6 mg/kg intravenous followed by 90 mg subcutaneous, 1 mg/kg, 6 mg/kg, and 130 mg intravenous) and natalizumab (3 mg/kg intravenous in two infusions), potentially more affordable options. A systematic review revealed that although biologic agents incur high costs, particularly for use as maintenance therapy (incremental cost‐effectiveness rates above 100,000 purchasing power parity/quality‐adjusted life years), their cost‐effectiveness may improve as market prices decrease and with the introduction of biosimilars over the years. Moreover, although the evidence on HRQoL is still heterogeneous and poorly reported by RCTs—as confirmed by our review, authors agree that biologics and small molecules can indirectly impact this domain by improving disease control, reducing symptoms, and enhancing overall well‐being [52, 53]. It is imperative that future studies identify optimal treatment strategies that reflect routine clinical practice (in the real world) in a given country or region and prioritize human outcomes [54]. Drugs with less invasive routes of administration, such as subcutaneous adalimumab, and those with shorter treatment courses may improve adherence compared to long intravenous treatments, which also impact patients' HRQoL and treatment costs. A study investigating patient preferences and willingness to switch from intravenous to subcutaneous dosing (*n* = 454 IBD patients) revealed a preference for subcutaneous every 8 weeks, which is comparable to daily oral dosing. However, among intravenous‐treated patients, approximately 55% resisted switching to subcutaneous dosing mostly due to the presence of medical staff, fear of needles, and longer intervals between infusions, suggesting that transitioning to different treatments is still challenging in daily practice and should be carefully addressed in future studies [55].

An alternative to intravenous drug administration would be the utilization of a JAKi, as these agents have favorable oral bioavailability [56]. The clinical profiles of upadacitinib and tofacitinib have been shown to be similar to those of some biologics currently used in clinical practice, such as vedolizumab and certolizumab pegol. Moreover, use of JAKi has been linked to an enhancement in the HRQoL of patients when compared to placebo—as demonstrated by our systematic review of the IBQD score, with better responses in the systemic symptoms and social functioning domains [52, 57]. Nevertheless, some studies of upadacitinib for the treatment of rheumatoid arthritis have demonstrated an increased prevalence of herpes zoster, with an incidence of 3.0 (95% CI 2.6–3.5) per 100 patients for upadacitinib 15 mg, 5.3 (95% CI 4.5–6.2) per 100 patients for upadacitinib 30 mg, and 1.1 (95% CI 0.5–1.9) per 100 patients for treatment with adalimumab + methotrexate [58]. Further studies are, thus, required to confirm the benefits and risks of this treatment for IBD.

Another oral alternative, the selective oral sphingosine‐1‐phosphate receptor modulator (S1PRM), amiselimod, has shown a comparable effect with anti‐TNF in animal models [59]. However, our study revealed an unfavorable efficacy and safety profile for amiselimod. SUCRA values were below 20% for clinical remission and above 90% for SAE, primarily related to lymphopenia, affecting 8.9% of patients. Another S1PRM, ozanimod, demonstrated endoscopic, histological, and clinical improvements in a single‐arm uncontrolled study, with clinical remission and response rates of 39.1% and 56.6%, respectively [60]. However, the lack of comparative randomized studies evaluating this treatment limits the ability to fully assess its efficacy and safety in a more rigorous context.

Our study has some limitations. The included trials exhibited variation in terms of sample size, risk of bias, and external validity. To avoid systematic errors, transitivity and sensitivity analyses were conducted, yielding results that were analogous to those of the original analyses. However, these studies represent the available evidence on the effect of biological drugs for Crohn's disease. Our analyses were limited to the most reported outcomes; other relevant information (e.g., corticosteroid‐free remission and endoscopic remission) was not evaluated given the lack of standardized data reported in the primary studies. Furthermore, inconsistent reporting of clinical response outcomes across studies was noted, with some authors indicating a decrease of 70 points in the CDAI, while others use a more stringent criterion of a 100‐point decrease. Additionally, certain imprecision in the indirect comparisons was found in our analyses, resulting in a low level of confidence in the evidence. This inconsistency may be attributed to variations in the timing of efficacy outcome measurements, ranging from 2 to 28 weeks, as well as differences in treatment duration. As with any method, the NMA has limitations; therefore, the validity of its conclusions is contingent upon the distribution of relative treatment modifiers between the groups of comparators. Treatment rankings should not be interpreted separately from the relative treatment effects. However, these preliminary findings may contribute to the design and conduction of future studies targeting the most promising drugs in the field.

Finally, based on the available evidence on the effect of biological drugs and small molecules for managing moderate‐to‐severe Crohn's disease in adults, we recommend incorporating novel inhibitors of IL‐23 as second‐line alternatives, especially for patients who have previously failed other biologic treatments. Given their safety profile, some anti‐TNF drugs should be closely monitored or avoided in clinical practice. Additionally, therapeutic drug monitoring is also a valuable tool for assessing serum drug concentrations and the presence of antidrug antibodies. Such monitoring can provide crucial insights into treatment efficacy and potential issues with drug resistance or inadequate response. Other important factors, such as drug access and costs, should be considered for this decision. Further well‐designed head‐to‐head studies on the short‐ and long‐term effects of the most promising therapies—including dose, regimen, and drug combinations—should be performed to confirm these findings and better establish treatment algorithms for this disease.

## Author Contributions

**Daniela Gorski:** conceptualization, methodology, data curation, formal analysis, visualization, writing – original draft, writing – review and editing. **Raul Edison Luna Lazo:** conceptualization, validation, formal analysis, methodology, writing – original draft. **Dalton de Assis de Souza:** data curation, formal analysis, methodology, writing – original draft. **Helena Hiemisch Lobo Borba:** writing – original draft, writing – review and editing. **Roberto Pontarolo:** conceptualization, writing – review and editing, supervision, writing – original draft, project administration. **Fernanda Stumpf Tonin:** conceptualization, methodology, writing – original draft, writing – review and editing, supervision.

## Conflicts of Interest

The authors declare no conflicts of interest.

## Supporting information


**Appendix S1:** phar70049‐sup‐0001‐AppendixS1.pdf.

## Data Availability

The datasets generated and/or analyzed during the current study are available on request. A project has been created on the Open Science Framework (OSF) to make the Supporting Information publicly available under the DOI: https://doi.org/10.17605/OSF.IO/4DKT9.
