# Supplementary material for: Biological Therapy and Small Molecules for Adults With Crohn's Disease: Systematic Review and Network Meta‐Analysis
Source: Pharmacotherapy. 2025 Aug 7;45(9):587–99. doi: 10.1002/phar.70049 (PMC12424525; doi:10.1002/phar.70049)
Supplement: Supplementary file 1 — Appendix S1: phar70049‐sup‐0001‐AppendixS1.pdf. [file PHAR-45-587-s001.pdf]

Biological Therapy and Small Molecules for Adults with Crohn's Disease: Systematic Review and Network Meta-analysis

Daniela Gorski, Raul Edison Luna Lazo, Dalton de Assis de Souza, Helena Hiemisch Lobo Borba, Roberto Pontarolo, and Fernanda Stumpf Tonin.

The OSF project, which contains all the files, can be found at the DOI: 10.17605/OSF.IO/4DKT9.

SUMMARY

TABLE S1. SEARCH STRATEGY 2

TABLE S2. EXCLUDED STUDIES, AFTER FULL-TEXT READING, AND JUSTIFICATIONS 3

TABLE S3. LIST OF THE INCLUDED STUDIES 6

TABLE S4. BASELINE DATA EXTRACTION 9

TABLE S5. RISK OF BIAS ANALYSIS OF ADVERSE EFFECTS 20

TABLE S6. RISK OF BIAS ANALYSIS OF QUALITY OF LIFE (IBDQ) 22

TABLE S7. RISK OF BIAS ANALYSIS OF CDAI 23

FIG. S1. FUNNEL PLOT ANALYSIS 25

TABLES S8. GELMAN-RUBIN BROOKS ANALYSIS FOR THE BAYESIAN MODELS. 26

TABLES S9. RESULTS FROM NODE SPLITTING ANALYSIS. 27

FIG. S2. NETWORK META-ANALYSIS PLOT OF CLINICAL REMISSION WITH DOSE AND ROUTE OF ADMINISTRATION 29

TABLE S10. GEOMETRIC METRIC FOR NETWORK META-ANALYSIS 30

TABLE S11. ABBREVIATIONS FOR THE TREATMENTS USED TO CONSTRUCT THE NETWORKS 31

TABLE S12. LEAGUE TABLES FOR CDAI OUTCOMES 36

TABLE S13: SUCRA PROBABILITYS FOR CDAI OUTCOMES 98

TABLE S14. LEAGUE TABLE FOR IBDQ OUTCOMES.. 101

TABLE S15. SUCRA PROBABILITYS FOR IBDQ OUTCOMES 106

TABLE S16: LEAGUE TABLES FOR SAFETY OUTCOMES. 107

TABLE S17. SUCRA PROBABILITYS FOR SAFETY OUTCOMES 149

TABLE S18. SENSITIVE ANALYSIS OF RISK OF BIAS Each table presents the results for 151

TABLE S19. CINEMA ANALYSIS. 192

TABLE S1. SEARCH STRATEGY

Pubmed (03/06/2025)	
**#1** “Crohn Disease”[MH] OR “Crohn*”[TIAB]<br><br>**#2** "monoclonal antibody"[TIAB] OR biologic*[TIAB] OR "Tumor Necrosis Factor Inhibitors"[MH] OR "Tumor Necrosis Factor Inhibitors"[TIAB] OR "TNF Inhibitor*"[TIAB] OR "Tumor Necrosis Factor Antagonists"[TIAB] OR "TNF Antagonist"[TIAB] OR "Interleukin-6 Inhibitors"[MH] OR Anti-IL[TIAB] OR Infliximab[MH] OR infliximab[TIAB] OR Adalimumab[MH] OR adalimumab[TIAB] OR "Certolizumab Pegol"[MH] OR certolizumab[TIAB] OR etrasimod[TIAB] OR risankizumab[TIAB] OR risankizumab[Supplementary Concept] OR Ustekinumab[MH] OR Ustekinumab[TIAB] OR vedolizumab[Supplementary Concept] OR vedolizumab[TIAB] OR mirikizumab[Supplementary Concept] OR mirikizumab[TIAB] OR guselkumab[Supplementary Concept] OR guselkumab[TIAB] OR etrolizumab[Supplementary Concept] OR etrolizumab[TIAB] OR ontamalimab[Supplementary Concept] OR ontamalimab[TIAB] OR abrilumab[Supplementary Concept] OR abrilumab[TIAB] OR Natalizumab[MH] OR Natalizumab[TIAB] OR andecaliximab[Supplementary Concept] OR andecaliximab[TIAB] OR brazikumab[TIAB] OR MEDI2070[TIAB] OR secukinumab[Supplementary Concept] OR secukinumab[TIAB] OR brodalumab[Supplementary Concept] OR brodalumab [TIAB] OR Abatacept[MH] OR Abatacept[TIAB] OR Belatacept[TIAB] OR Sphingosine-1-phosphate[TIAB] OR amiselimod[Supplementary Concept] OR amiselimod[TIAB] OR ozanimod[Supplementary Concept] OR ozanimod[TIAB] OR etrasimod[Supplementary Concept] OR etrasimod[TIAB] OR "Janus Kinase 1"[MH] OR "Janus Kinase"[TIAB] OR Upadacitinib[Supplementary Concept] OR Upadacitinib[TIAB] OR GLPG0634[Supplementary Concept] OR filgotinib[TIAB] OR tofacitinib[Supplementary Concept] OR tofacitinib[TIAB] OR tasocitinib[TIAB]<br><br>**#3** "second line"[All Fields] OR "second-line"[All Fields] OR refractory[TIAB] OR recurrent[TIAB] OR recurrence[TIAB] OR failure[TIAB] OR "after withdrawal"[TIAB] OR "after first-line"[TIAB] OR "after first line"[TIAB] OR "moderate to severe"[TIAB] OR "moderate-to-severe"[TIAB] OR "moderately to severely"[TIAB] OR "moderately-to-severely"[TIAB] OR "active Crohn*"[TIAB] OR "inadequate response"[TIAB] OR previously treated[TIAB]<br><br>**#4** “clinical trial”[TIAB] OR “clinical study”[TIAB] OR “clinical trials as topic”[MH] OR “clinical trial”[PT] OR random*[TIAB] OR “random allocation”[MH] OR “controlled trial”[TIAB] OR “controlled study”[TIAB]<br><br>#1 AND #2 AND #3 AND #4	**845**
**Scopus (03/06/2025)**	
**#1** TITLE-ABS-KEY(“Crohn*”)<br><br>**#2** TITLE-ABS("monoclonal antibody" OR biologic* OR "Tumor Necrosis Factor Inhibitors" OR "TNF Inhibitor*" OR "Tumor Necrosis Factor Antagonists" OR "TNF Antagonist" OR Anti-IL OR OR infliximab OR OR adalimumab OR certolizumab OR etrasimod OR risankizumab OR Ustekinumab OR vedolizumab OR mirikizumab OR guselkumab OR etrolizumab OR ontamalimab OR abrilumab OR Natalizumab OR OR andecaliximab OR brazikumab OR MEDI2070 OR secukinumab OR brodalumab OR Abatacept OR Belatacept OR Sphingosine-1-phosphate OR amiselimod OR ozanimod OR etrasimod OR "Janus Kinase" OR Upadacitinib OR filgotinib OR tofacitinib OR tasocitinib)<br><br>**#3** ALL(“second line” OR “second-line”) OR TITLE-ABS(refractory OR recurrent OR recurrence OR failure OR "after withdrawal" OR "after first-line" OR "after first line" OR "moderate to severe" OR "moderate-to-severe" OR "moderately to severely" OR "moderately-to-severely" OR "active Crohn*" OR "inadequate response" OR previously treated)<br><br>**#4** TITLE-ABS(“clinical trial” OR “clinical study” OR “clinical trial” OR random* OR “controlled trial” OR “controlled study”)<br><br>#1 AND #2 AND #3 AND #4	**1124**
**WoS (03/06/2025)**	
**#1** TS=(“Crohn*”)<br><br>**#2** TS=("monoclonal antibody" OR biologic* OR "Tumor Necrosis Factor Inhibitors" OR "TNF Inhibitor*" OR "Tumor Necrosis Factor Antagonists" OR "TNF Antagonist" OR Anti-IL OR OR infliximab OR OR adalimumab OR certolizumab OR etrasimod OR risankizumab OR Ustekinumab OR vedolizumab OR mirikizumab OR guselkumab OR etrolizumab OR ontamalimab OR abrilumab OR Natalizumab OR OR andecaliximab OR brazikumab OR MEDI2070 OR secukinumab OR brodalumab OR Abatacept OR Belatacept OR Sphingosine-1-phosphate OR amiselimod OR ozanimod OR etrasimod OR "Janus Kinase" OR Upadacitinib OR filgotinib OR tofacitinib OR tasocitinib)<br><br>**#3** ALL=(“second line” OR “second-line”) OR TS=(refractory OR recurrent OR recurrence OR failure OR "after withdrawal" OR "after first-line" OR "after first line" OR "moderate to severe" OR "moderate-to-severe" OR "moderately to severely" OR "moderately-to-severely" OR "active Crohn*" OR "inadequate response" OR previously treated)<br><br>**#4** TS=(“clinical trial” OR “clinical study” OR “clinical trial” OR random* OR “controlled trial” OR “controlled study”)<br><br>#1 AND #2 AND #3 AND #4	**1436**

TABLE S2. EXCLUDED STUDIES, AFTER FULL-TEXT READING, AND JUSTIFICATIONS

Study	Reason
Aguilar D, Revilla L, Garrido-Trigo A, et al. Randomized Controlled Trial Substudy of Cell-specific Mechanisms of Janus Kinase 1 Inhibition With Upadacitinib in the Crohn's Disease Intestinal Mucosa: Analysis From the CELEST Study. Inflamm Bowel Dis 2021;27:1999-2009.	outcome
Colombel J, Lemann M, Bouhnik Y, et al. Endoscopic Mucosal improvement in patients with active Crohn's Disease treated with Certolizumab Pegol: First results of the MUSIC Clinical Trial. INFLAMMATORY BOWEL DISEASES 2008;14:S25-S25.	publication type
Colombel JF, Hébuterne X. Endoscopic mucosal improvement in patients with active Crohn's disease treated with certolizumab pegol:: first results of the music clinical trial. AMERICAN JOURNAL OF GASTROENTEROLOGY 2008;103:S432-S432.	outcome
Colombel JF, Hisamatsu T, Atreya R, et al. Upadacitinib Reduces Crohn's Disease Symptoms Within the First Week of Induction Therapy. Clin Gastroenterol Hepatol 2024;22:1668-1677.	study design: post hoc
Colombel JF, Panes J, Lacerda AP, et al. EFFICACY AND SAFETY OF UPADACITINIB INDUCTION THERAPY IN PATIENTS WITH MODERATELY TO SEVERELY ACTIVE CROHN'S DISEASE WHO FAILED PRIOR BIOLOGICS: RESULTS FROM A RANDOMIZED PHASE 3 U-EXCEED STUDY. GASTROENTEROLOGY 2022;162:S1394-S1394.	publication type
Colombel JF, Rutgeerts PJ, Sandborn WJ, et al. Adalimumab induces deep remission in patients with Crohn's disease. Clin Gastroenterol Hepatol 2014;12:414-22.e5.	study design: induction with biological treatment in both arms
Colombel JF, Sands BE, Gasink C, et al. Evolution of Symptoms After Ustekinumab Induction Therapy in Patients With Crohn's Disease. Clin Gastroenterol Hepatol 2024;22:144-153.e2.	outcome
D'Haens G, Dubinsky MC, Peyrin-Biroulet L, et al. Etrasimod induction therapy in moderately to severely active Crohn's disease: results from a phase 2, randomised, double-blind substudy. JOURNAL OF CROHNS & COLITIS 2023;17:764-765.	publication type
D'Haens G, Dubinsky MC, Peyrin-Biroulet L, et al. ETRASIMOD INDUCTION THERAPY IN MODERATELY TO SEVERELY ACTIVE CROHN'S DISEASE: RESULTS FROM A PHASE 2, RANDOMIZED, DOUBLE-BLIND SUBSTUDY. GASTROENTEROLOGY 2023;164:S223-S223.	publication type
D'Haens G, Sandborn WJ, Colombel JF, et al. A phase II study of laquinimod in Crohn's disease. GUT 2015;64:1227-1235.	population
D'Haens G, Van Deventer S, Van Hogezand R, et al. Endoscopic and histological healing with infliximab anti-tumor necrosis factor antibodies in Crohn's disease: A European multicenter trial. Gastroenterology 1999;116:1029-34.	outcome
D'Haens GR, Sandborn WJ, Loftus EV, Jr., et al. Higher vs Standard Adalimumab Induction Dosing Regimens and Two Maintenance Strategies: Randomized SERENE CD Trial Results. Gastroenterology 2022;162:1876-1890.	intervention
Danese S, Beaton A, Duncan EA, et al. Long-term safety of brazikumab in the open-label period of a randomized phase 2a study of patients with Crohn's disease. BMC Gastroenterol 2023;23:451.	study design: maintenance treatment
El-Hachem S, Regueiro M, Kevin K, et al. Long-term follow-up of patients enrolled in the Randomized Controlled Trial (RCT) of Infliximab for prevention of recurrent Crohn's Disease (CD). INFLAMMATORY BOWEL DISEASES 2009;15:S11-S11.	publication type
Feagan B, Gasink C, Gao L, et al. A Multicenter, Randomized, Double-blind, Placebo-controlled Phase 2b Study of Ustekinumab, a Human Monoclonal Antibody to Il-12/23p40, in Patients with Moderately to Severely Active Crohn's Disease: Results through Week 36 from the CERTIFI Trial. AMERICAN JOURNAL OF GASTROENTEROLOGY 2011;106:S463-S463.	publication type
Feagan BG, Schreiber S, Afzali A, et al. Ozanimod as a novel oral small molecule therapy for the treatment of Crohn's disease: The YELLOWSTONE clinical trial program. Contemp Clin Trials 2022;122:106958.	outcome
Ferrante M, Panaccione R, Baert F, et al. Risankizumab as maintenance therapy for moderately to severely active Crohn's disease: results from the multicentre, randomised, double-blind, placebo-controlled, withdrawal phase 3 FORTIFY maintenance trial. Lancet 2022;399:2031-2046.	study design: maintenance treatment
Gibble TH, Macey J, Makin H, et al. Exit interviews exploring Crohn's disease patients' experience of changes in their bowel urgency during the mirikizumab Phase 3 clinical trial in adult patients with moderate to severe Crohn disease. JOURNAL OF CROHNS & COLITIS 2024;18:I1343-I1344.	publication type
Gordon FH, Lai CW, Hamilton MI, et al. A randomized placebo-controlled trial of a humanized monoclonal antibody to alpha4 integrin in active Crohn's disease. Gastroenterology 2001;121:268-74.	population
Hanauer S, Lukás M, MacIntosh D, et al. A randomized, double-blind, placebo-controlled trial of the human anti-TNF-α monoclonal antibody adalimumab for the induction of remission in patients with moderate to severely active Crohn's disease. GASTROENTEROLOGY 2004;127:332-332.	publication type
Hanauer SB, Feagan BG, MacIntosh DG, et al. Efficacy of Vedolizumab in Crohn's Disease by Prior Treatment Failure in Gemini II, a Randomized, Placebo-Controlled, Double-Blind, Multicenter Study. GASTROENTEROLOGY 2013;144:S772-S772.	publication type
Hanauer SB, Sandborn WJ, Sands BE, et al. A Randomized Placebo-Controlled Trial of Abatacept for Moderately-to-Severely Active Crohn's Disease (CD). GASTROENTEROLOGY 2010;138:S86-S86.	publication type
Hibi T, Imai Y, Murata Y, et al. Efficacy and safety of ustekinumab in Japanese patients with moderately to severely active Crohn's disease: a subpopulation analysis of phase 3 induction and maintenance studies. Intest Res 2017;15:475-486.	publication type
Hibi T, Watanabe M, Camez A, et al. Efficacy and safety of adalimumab for the treatment of Japanese patients with moderately to severely active Crohn's disease: Results from a randomized controlled trial. AMERICAN JOURNAL OF GASTROENTEROLOGY 2008;103:S414-S415.	publication type
Ito H, Takazoe M, Fukuda Y, et al. Effective treatment of active Crohn's disease with humanized monoclonal antibody MRA to interleukin-6 receptor: A randomized placebo-controlled trial. GASTROENTEROLOGY 2003;124:A25-A25.	publication type

Ito H, Takazoe M, Fukuda Y, et al. A pilot randomized trial of a human anti-interleukin-6 receptor monoclonal antibody in active Crohn's disease. Gastroenterology 2004;126:989-96; discussion 947.	publication type
Kamal ME, Werida RH, Radwan MA, et al. Efficacy and safety of infliximab and adalimumab in inflammatory bowel disease patients. Inflammopharmacology 2024.	population
Lam MC, Bressler B. Vedolizumab for ulcerative colitis and Crohn's disease: results and implications of GEMINI studies. Immunotherapy 2014;6:963-71.	study design: post hoc
Li K, Friedman JR, Chan D, et al. Effects of Ustekinumab on Histologic Disease Activity in Patients With Crohn's Disease. Gastroenterology 2019;157:1019-1031.e7.	outcome
Loftus EV, Colombel JF, Lacerda AP, et al. Efficacy and Safety of Upadacitinib Induction Therapy in Patients With Moderately to Severely Active Crohn's Disease: Results From a Randomized Phase 3 U-EXCEL Study. AMERICAN JOURNAL OF GASTROENTEROLOGY 2022;117:S518-S518.	publication type
Mannon PJ, Fuss IJ, Mayer L, et al. Anti-interleukin-12 antibody for active Crohn's disease. N Engl J Med 2004;351:2069-79.	population
Mohamed MF, Klünder B, Lacerda AP, et al. Exposure-Response Analyses for Upadacitinib Efficacy and Safety in the Crohn's Disease CELEST Study and Bridging to the Extended-Release Formulation. Clin Pharmacol Ther 2020;107:639-649.	outcome
Narula N, Kainz S, Petritsch W, et al. The efficacy and safety of either infliximab or adalimumab in 362 patients with anti-TNF-α naïve Crohn's disease. Alimentary Pharmacology and Therapeutics 2016;44:170-180.	study design: observational study
Panés J, Chan G, Maller E, et al. Effects of Oral Tofacitinib on Patient-Reported Outcomes in Patients With Moderate to Severe Crohn's Disease: Results of Two Phase 2B Randomized Placebo-Controlled Trials. GASTROENTEROLOGY 2016;150:S1003-S1003.	publication type
Panés J, Moscariello M, Maller E, et al. Effects of oral tofacitinib on patient-reported outcomes in patients with moderate-to-severe Crohn's disease: results of 2 Phase 2b randomised placebo-controlled trials. JOURNAL OF CROHNS & COLITIS 2016;10:S60-S61.	publication type
Panés J, Sandborn W, Schreiber S, et al. Efficacy and Safety of Tofacitinib for Oral Induction Therapy in Patients With Moderate to Severe Crohn's Disease: Results of a Phase 2B Randomized Placebo-Controlled Trial. GASTROENTEROLOGY 2016;150:S182-S183.	publication type
Panés J, Sandborn WJ, Schreiber S, et al. Tofacitinib for induction and maintenance therapy of Crohn's disease: results of two phase IIb randomised placebo-controlled trials. Gut 2017;66:1049-1059.	population
Pang Y, D'Cunha R, Mohammad AS, et al. Clinical Bridging From Prefilled Syringe to On-body Injector for Risankizumab in Crohn's Disease. Clin Ther 2024;46:30-39.	outcome
Peyrin-Biroulet L, Panaccione R, Louis E, et al. Upadacitinib Achieves Clinical and Endoscopic Outcomes in Crohn's Disease Regardless of Prior Biologic Exposure. Clin Gastroenterol Hepatol 2024.	outcome
Regueiro M, Fischer M, Bossuyt P, et al. Mirikizumab Sustained Impact on Fatigue in Patients with Moderately to Severely Active Crohn's Disease in the Phase 2 AMAG Study. Inflamm Bowel Dis 2024.	population
Reinisch W, Leszczyszyn J, Dudkowiak R, et al. A NOVEL SUBCUTANEOUS INFLIXIMAB (CT-P13) IN PATIENTS WITH ACTIVE CROHN'S DISEASE AND ULCERATIVE COLITIS: WEEK54 AND SWITCHING RESULTS FROM A MULTICENTER, RANDOMISED CONTROLLED PIVOTAL TRIAL. GASTROENTEROLOGY 2020;158:S193-S193.	publication type
Rosario M, French JL, Dirks NL, et al. Exposure-efficacy Relationships for Vedolizumab Induction Therapy in Patients with Ulcerative Colitis or Crohn's Disease. J Crohns Colitis 2017;11:921-929.	outcome
Rosario M, Wyant T, Leach T, et al. Vedolizumab Pharmacokinetics, Pharmacodynamics, Safety, and Tolerability Following Administration of a Single, Ascending, Intravenous Dose to Healthy Volunteers. Clin Drug Investig 2016;36:913-923.	population
Rubin DT, Mulani P, Chao J, et al. Effect of adalimumab on clinical laboratory parameters in patients with Crohn's disease: results from the CHARM trial. Inflamm Bowel Dis 2012;18:818-25.	study design: post hoc
Rutgeerts P, D'Haens G, Targan S, et al. Efficacy and safety of retreatment with anti-tumor necrosis factor antibody (infliximab) to maintain remission in Crohn's disease. Gastroenterology 1999;117:761-9.	study design: maintenance treatment
Rutgeerts P, Gasink C, Chan D, et al. Efficacy of Ustekinumab for Inducing Endoscopic Healing in Patients With Crohn's Disease. Gastroenterology 2018;155:1045-1058.	outcome
Sandborn, Feagan B, Fedorak R, et al. A multicenter, randomized, phase 2A study of human monoclonal antibody to IL-12/23P40 (CNTO 1275) in patients with moderately to severely active Crohn's disease. INFLAMMATORY BOWEL DISEASES 2008;14:S10-S10.	publication type
Sandborn W, Schreiber S, Feagan B, et al. Induction Therapy with Certolizumab Pegol in Patients with Moderate to Severe Crohn's Disease: A Placebo-Controlled Trial. AMERICAN JOURNAL OF GASTROENTEROLOGY 2010;105:S419-S419.	publication type
Sandborn WJ, Colombel JF, Schreiber S, et al. Dosage adjustment during long-term adalimumab treatment for Crohn's disease: clinical efficacy and pharmacoeconomics. Inflamm Bowel Dis 2011;17:141-51.	study design: maintenance treatment
Sandborn WJ, Feagan B, Radford-Smith G, et al. A randomized, placebo-controlled trial of CDP571, a humanized monoclonal antibody to TNF-α, in patients with moderate to severe Crohn's disease. GASTROENTEROLOGY 2003;124:A61-A61.	publication type
Sandborn WJ, Feagan BG, Fedorak R, et al. A multicenter, randomized, phase 2a study of human monoclonal antibody to IL-12/23p40 (Cnto 1275) in patients with moderately to severely active Crohn's disease. GASTROENTEROLOGY 2007;132:A51-A51.	publication type
Sandborn WJ, Gasink C, Gao LL, et al. A Multicenter, Randomized, Double-Blind, Placebo-Controlled Phase2b Study or Ustekinumab, a Human Monoclonal Antibody to IL-12/23p40, in Patients With Moderately to Severely Active Crohn's Disease: Results Through Week 22 From the Certifi Trial. GASTROENTEROLOGY 2011;140:S109-S109.	publication type
Sandborn WJ, Ghosh S, Panes J, et al. Phase 2 Randomized Study of CP-690,550, an Oral Janus Kinase Inhibitor, in Active Crohn's Disease. GASTROENTEROLOGY 2011;140:S124-S124.	publication type
Sandborn WJ, Panes J, Peyrin-Biroulet L, et al. DOSE ESCALATION OF UPADACITINIB IN CROHN'S DISEASE PATIENTS WITH INADEQUATE RESPONSE: DATA FROM THE RANDOMISED CELEST STUDY. GASTROENTEROLOGY 2020;158:S1187-S1187.	publication type

Sandborn WJ, Rutgeerts P, Gasink C, et al. Long-term efficacy and safety of ustekinumab for Crohn's disease through the second year of therapy. Aliment Pharmacol Ther 2018;48:65-77.	study design: maintenance treatment
Sandborn WJ, Vermeire S, D'Haens GR, et al. Welcome: A Randomized, Double-Blind, Controlled Trial Comparing Certolizumab Pegol 400 Mg Every 2 Weeks with Every 4 Weeks for Maintenance of Response and Remission in Patients with Moderate to Severe Crohn's Disease with Secondary Failure to Infliximab. GASTROENTEROLOGY 2009;136:A27-A27.	publication type
Sands B, Feagan B, Rutgeerts P, et al. Vedolizumab Induction Therapy for Patients With Crohn's Disease and Prior Anti-TNF Antagonist Failure: A Randomized, Placebo-controlled, Double-blind, Multicenter Trial. INFLAMMATORY BOWEL DISEASES 2012;18:S24-S25.	publication type
Sands B, Siegel C, Sandborn W, et al. Natalizumab reduces the rate of hospitalization in moderate to severe Crohn's Patients: Evidence from the Clinical Trial Program. INFLAMMATORY BOWEL DISEASES 2009;15:S27-S27.	publication type
Sands BE, Chen JJ, Penney M, et al. Initial Evaluation of MEDI2070 (Specific Anti-IL-23 Antibody) in Patients With Active Crohn's Disease Who Have Failed Anti-TNF Antibody Therapy: A Randomized, Double-Blind Placebo-Controlled Phase 2A Induction Study. GASTROENTEROLOGY 2015;148:S163-S164.	publication type
Sands BE, Gasink C, Gao LL, et al. Health related quality of life results through week 22 from the CERTIFI study, a multicenter, randomized, double-blind, placebo-controlled phase2b study of Ustekinumab in patients with moderately to severely active Crohn's disease. INFLAMMATORY BOWEL DISEASES 2011;17:S24-S24.	study design: congress abstract
Sands BE, Kozarek R, Spainhour J, et al. Safety and tolerability of concurrent natalizumab treatment for patients with Crohn's disease not in remission while receiving infliximab. Inflamm Bowel Dis 2007;13:2-11.	population
Sands BE, Sandborn WJ, Van Assche G, et al. Vedolizumab as Induction and Maintenance Therapy for Crohn's Disease in Patients Naïve to or Who Have Failed Tumor Necrosis Factor Antagonist Therapy. Inflamm Bowel Dis 2017;23:97-106.	study design: post hoc
Schreiber S, Cross RK, Panaccione R, et al. Efficacy and safety of risankizumab by baseline corticosteroid use and achievement of corticosteroid-free clinical and endoscopic outcomes in patients with moderately to severely active Crohn's disease. Aliment Pharmacol Ther 2024.	study design: post hoc
Schreiber S, Feagan B, D'Haens G, et al. Oral p38 mitogen-activated protein kinase inhibition with BIRB 796 for active Crohn's disease: A randomized, double-blind, placebo-controlled trial. CLINICAL GASTROENTEROLOGY AND HEPATOLOGY 2006;4:325-334.	intervention
Schreiber S, Nikolaus S, Malchow H, et al. Absence of efficacy of subcutaneous antisense ICAM-1 treatment of chronic active Crohn's disease. GASTROENTEROLOGY 2001;120:1339-1346.	outcome
Stack WA, Mann SD, Roy AJ, et al. Randomised controlled trial of CDP571 antibody to tumour necrosis factor-alpha in Crohn's disease. LANCET 1997;349:521-524.	population
Steenholdt C, Brynskov J, Thomsen O, et al. Treatment of Secondary Infliximab Failure in Crohn's Disease Based on Serum Levels of Infliximab and Antibodies Against Infliximab: The Danish Study of Optimizing Infliximab Therapy in Crohn's Disease (Do It Crohn) Randomized Clinical Trial. GASTROENTEROLOGY 2013;144:S22-S22.	study design: congress abstract
Toedter GP, Blank M, Lang Y, et al. Relationship of C-reactive protein with clinical response after therapy with ustekinumab in Crohn's disease. Am J Gastroenterol 2009;104:2768-73.	outcome
Travis S, Hisamatsu T, Fischer M, et al. Mirikizumab improves fatigue, bowel urgency, and quality of life in patients with moderately to severely active Crohn's Disease: Results from a phase 3 clinical trial. JOURNAL OF CROHNS & COLITIS 2024;18:I21-I23.	study design: congress abstract
van der Woude CJ, Stokkers P, van Bodegraven AA, et al. Phase I, double-blind, randomized, placebo-controlled, dose-escalation study of NI-0401 (a fully human anti-CD3 monoclonal antibody) in patients with moderate to severe active Crohn's disease. Inflamm Bowel Dis 2010;16:1708-16.	outcome
van der Woude CJ, Stokkers PC, van Bodegraven AA, et al. Safety profile and signal of efficacy of a non-fcr-binding, fully human anti-Cd3 monoclonal antibody in patients with moderate to severe Crohn's disease- results of a phase I/Iia, double blind, placebo-controlled, randomized, dose escalation study. GASTROENTEROLOGY 2008;134:A67-A67.	study design: congress abstract
Vermeire S, Danese S, Sandborn WJ, et al. Efficacy and Safety of the Anti-mucosal Addressin Cell Adhesion Molecule-1 Antibody Ontamalimab in Patients with Moderate-to-Severe Ulcerative Colitis or Crohn's Disease. J Crohns Colitis 2024;18:708-719.	population
Ferrante M, D'Haens G, Jairath V, Danese S, Chen M, Ghosh S, Hisamatsu T, Kierkus J, Siegmund B, Bragg SM, Crandall W, Durand F, Hon E, Lin Z, Lopes MU, Morris N, Protic M, Carlier H, Sands BE; VIVID Study Group. Efficacy and safety of mirikizumab in patients with moderately-to-severely active Crohn's disease: a phase 3, multicentre, randomised, double-blind, placebo-controlled and active-controlled, treat-through study. Lancet. 2024 Dec 14;404(10470):2423-2436. doi: 10.1016/S0140-6736(24)01762-8. Epub 2024 Nov 21. PMID: 39581202.	population
Watanabe M, Hibi T, Mostafa NM, et al. Long-term safety and efficacy of adalimumab in Japanese patients with moderate to severe Crohn's disease. J Crohns Colitis 2014;8:1407-16.	study design: maintenancetherapy
Yacyshyn BR, Chey WY, Goff J, et al. Double blind, placebo controlled trial of the remission inducing and steroid sparing properties of an ICAM-1 antisense oligodeoxynucleotide, alicaforsen (ISIS 2302), in active steroid dependent Crohn's disease. GUT 2002;51:30-36.	population
Zhang N, Chan ML, Li J, et al. Combining pharmacometric models with predictive and prognostic biomarkers for precision therapy in Crohn's disease: A case study of brazikumab. CPT Pharmacometrics Syst Pharmacol 2023;12:1945-1959.	study design: pharmacokincts
Zhou J, Xu Y, Chen Y, et al. Model-Based Investigation of Inadequate Efficacy of Tesnatilimab, an Anti-Natural Killer Group 2 Member D Monoclonal Antibody, in Moderately to Severely Active Crohn Disease. J Clin Pharmacol 2023;63:928-942.	study design: post hoc

TABLE S3. LIST OF THE INCLUDED STUDIES

	Study
1	Allez M, s BE, Feagan BG, et al. A Phase 2b, Randomised, Double-blind, Placebo-controlled, Parallel-arm, Multicenter Study Evaluating the Safety and Efficacy of Tesnatilimab in Patients with Moderately to Severely Active Crohn's Disease. J Crohns Colitis. 2023;17:1235-51. doi:10.1093/ecco-jcc/jjad047
2	Allez M, Skolnick BE, Wisniewska-Jarosinska M, et al. Anti-NKG2D monoclonal antibody (NNC0142-0002) in active Crohn's disease: a randomised controlled trial. Gut. 2017;66:1918-25. doi:10.1136/gutjnl-2016-311824
3	Chen B, Gao X, Zhong J, et al. Efficacy and safety of adalimumab in Chinese patients with moderately to severely active Crohn's disease: results from a randomized trial. Therap Adv Gastroenterol. 2020;13:1756284820938960-. doi:10.1177/1756284820938960
4	Danese S, Vermeire S, Hellstern P, et al. Randomised trial and open-label extension study of an anti-interleukin-6 antibody in Crohn’s disease (ANDANTE I and II). Gut. 2019;68:40-8. doi:10.1136/gutjnl-2017-314562
5	D'Haens G, Danese S, Davies M, et al. A phase II, Multicentre, Randomised, Double-Blind, Placebo-controlled Study to Evaluate Safety, Tolerability, and Efficacy of Amiselimod in Patients with Moderate to Severe Active Crohn's Disease. Journal of Crohns & Colitis. 2022;16:746-56. doi:10.1093/ecco-jcc/jjab201
6	D'Haens G, Panaccione R, Baert F, et al. Risankizumab as induction therapy for Crohn's disease: results from the phase 3 ADVANCE and MOTIVATE induction trials. Lancet. 2022;399:2015-30. doi:10.1016/s0140-6736(22)00467-6
7	Dotan I, Rachmilewitz D, Schreiber S, et al. A randomised placebo-controlled multicentre trial of intravenous semapimod HCl for moderate to severe Crohn's disease. Gut. 2010;59:760-6. doi:10.1136/gut.2009.179994
8	Fang Y, Li JP, Wu J, et al. Study of the Effects of Risankizumab in Moderate to Severe Crohn's Disease. Indian Journal of Pharmaceutical Sciences. 2023;85:230-5.
9	Feagan BG, born WJ, D'Haens G, et al. Induction therapy with the selective interleukin-23 inhibitor risankizumab in patients with moderate-to-severe Crohn's disease: a randomised, double-blind, placebo-controlled phase 2 study. Lancet. 2017;389:1699-709. doi:10.1016/s0140-6736(17)30570-6
10	Feagan BG, born WJ, Gasink C, et al. Ustekinumab as Induction and Maintenance Therapy for Crohn's Disease. N Engl J Med. 2016;375:1946-60. doi:10.1056/NEJMoa1602773
11	Feagan BG, Greenberg GR, Wild G, et al. Treatment of Active Crohn's Disease With MLN0002, a Humanized Antibody to the α4β7 Integrin. Clinical Gastroenterology and Hepatology. 2008;6:1370-7. doi:10.1016/j.cgh.2008.06.007
12	Ghosh S, Feagan BG, Parra RS, et al. Impact of Upadacitinib Induction and Maintenance Therapy on Health-related Quality of Life, Fatigue, and Work Productivity in Patients with Moderately-to-severely Active Crohn's Disease. J Crohns Colitis. 2024;18:1804-18. doi:10.1093/ecco-jcc/jjae083
13	Ghosh S, Goldin E, Gordon FH, et al. Natalizumab for active Crohn's disease. N Engl J Med. 2003;348:24-32. doi:10.1056/NEJMoa020732
14	Hanauer SB, born WJ, Rutgeerts P, et al. Human anti-tumor necrosis factor monoclonal antibody (adalimumab) in Crohn's disease: the CLASSIC-I trial. Gastroenterology. 2006;130:323-33; quiz 591. doi:10.1053/j.gastro.2005.11.030
15	Hueber W, s BE, Lewitzky S, et al. Secukinumab, a human anti-IL-17A monoclonal antibody, for moderate to severe Crohn's disease: unexpected results of a randomised, double-blind placebo-controlled trial. Gut. 2012;61:1693-700. doi:10.1136/gutjnl-2011-301668
16	Loftus EV, Jr., Panes J, Lacerda AP, et al. Upadacitinib Induction and Maintenance Therapy for Crohn's Disease. N Engl J Med. 2023;388:1966-80. doi:10.1056/NEJMoa2212728
17	Panaccione R, Sandborn WJ, Gordon GL, et al. Briakinumab for treatment of Crohn's disease: results of a randomized trial. Inflamm Bowel Dis. 2015;21:1329-40. doi:10.1097/MIB.0000000000000366
18	Reinisch W, de Villiers W, Bene L, et al. Fontolizumab in moderate to severe Crohn's disease: a phase 2, randomized, double-blind, placebo-controlled, multiple-dose study. Inflamm Bowel Dis. 2010;16:233-42. doi:10.1002/ibd.21038
19	Reinisch W, Hommes DW, Van Assche G, et al. A dose escalating, placebo controlled, double blind, single dose and multidose, safety and tolerability study of fontolizumab, a humanised anti-interferon gamma antibody, in patients with moderate to severe Crohn's disease. Gut. 2006;55:1138-44. doi:10.1136/gut.2005.079434
20	Rutgeerts P, born WJ, Fedorak RN, et al. Onercept for moderate-to-severe Crohn's disease: A randomized, double-blind, placebo-controlled trial. Clinical Gastroenterology and Hepatology. 2006;4:888-93. doi:10.1016/j.cgh.2006.04.022
21	Rutgeerts P, Schreiber S, Feagan B, et al. Certolizumab pegol, a monthly subcutaneously administered Fc-free anti-TNFalpha, improves health-related quality of life in patients with moderate to severe Crohn's disease. Int J Colorectal Dis. 2008;23:289-96. doi:10.1007/s00384-007-0395-7
22	s BE, Irving PM, Hoops T, et al. Ustekinumab versus adalimumab for induction and maintenance therapy in biologic-naive patients with moderately to severely active Crohn's disease: a multicentre, randomised, double-blind, parallel-group, phase 3b trial. Lancet. 2022;399:2200-11. doi:10.1016/s0140-6736(22)00688-2
23	s BE, Jacobson EW, Sylwestrowicz T, et al. Randomized, Double-Blind, Placebo-Controlled Trial of the Oral Interleukin-12/23 Inhibitor Apilimod Mesylate for Treatment of Active Crohn's Disease. Inflammatory Bowel Diseases. 2010;16:1209-18. doi:10.1002/ibd.21159
24	Sandborn WJ, Colombel JF, Enns R, et al. Natalizumab induction and maintenance therapy for Crohn's disease. N Engl J Med. 2005;353:1912-25. doi:10.1056/NEJMoa043335

25	Sandborn WJ, Colombel JF, Sands BE, et al. Abatacept for Crohn's disease and ulcerative colitis. Gastroenterology. 2012;143. doi:10.1053/j.gastro.2012.04.010
26	Sandborn WJ, D'Haens GR, Reinisch W, et al. Guselkumab for the Treatment of Crohn's Disease: Induction Results From the Phase 2 GALAXI-1 Study. Gastroenterology. 2022;162:1650-64.e8. doi:10.1053/j.gastro.2022.01.047
27	Sandborn WJ, Feagan BG, Fedorak RN, et al. A randomized trial of Ustekinumab, a human interleukin-12/23 monoclonal antibody, in patients with moderate-to-severe Crohn's disease. Gastroenterology. 2008;135:1130-41. doi:10.1053/j.gastro.2008.07.014
28	Sandborn WJ, Feagan BG, Hanauer SB, et al. An engineered human antibody to TNF (CDP571) for active Crohn's disease: A randomized double-blind placebo-controlled trial. Gastroenterology. 2001;120:1330-8. doi:10.1053/gast.2001.24042
29	Sandborn WJ, Feagan BG, Loftus EV, Jr., et al. Efficacy and Safety of Upadacitinib in a Randomized Trial of Patients With Crohn's Disease. Gastroenterology. 2020;158:2123-38 e8. doi:10.1053/j.gastro.2020.01.047
30	Sandborn WJ, Feagan BG, Radford-Smith G, et al. CDP571, a humanised monoclonal antibody to tumour necrosis factor alpha, for moderate to severe Crohn's disease: a randomised, double blind, placebo controlled trial. Gut. 2004;53:1485-93. doi:10.1136/gut.2003.035253
31	Sandborn WJ, Feagan BG, Rutgeerts P, et al. Vedolizumab as Induction and Maintenance Therapy for Crohn's Disease. New England Journal of Medicine. 2013;369:711-21. doi:10.1056/NEJMoa1215739
32	Sandborn WJ, Feagan BG, Stoinov S, et al. Certolizumab pegol for the treatment of Crohn's disease. N Engl J Med. 2007;357:228-38. doi:10.1056/NEJMoa067594
33	Sandborn WJ, Gasink C, Gao LL, et al. Ustekinumab induction and maintenance therapy in refractory Crohn's disease. N Engl J Med. 2012;367:1519-28. doi:10.1056/NEJMoa1203572
34	Sandborn WJ, Ghosh S, Panes J, et al. A phase 2 study of tofacitinib, an oral Janus kinase inhibitor, in patients with Crohn's disease. Clin Gastroenterol Hepatol. 2014;12:1485-93 e2. doi:10.1016/j.cgh.2014.01.029
35	Sandborn WJ, Hanauer SB, Katz S, et al. Etanercept for active Crohn's disease: a randomized, double-blind, placebo-controlled trial. Gastroenterology. 2001;121:1088-94. doi:10.1053/gast.2001.28674
36	Sandborn WJ, Lee SD, Tarabar D, et al. Phase II evaluation of anti-MAdCAM antibody PF-00547659 in the treatment of Crohn's disease: report of the OPERA study. Gut. 2018;67:1824-35. doi:10.1136/gutjnl-2016-313457
37	Sandborn WJ, Rutgeerts P, Colombel JF, et al. Eldelumab [anti-interferon-gamma-inducible protein-10 antibody] Induction Therapy for Active Crohn's Disease: a Randomised, Double-blind, Placebo-controlled Phase IIa Study. J Crohns Colitis. 2017;11:811-9. doi:10.1093/ecco-jcc/jjx005
38	Sandborn WJ, Rutgeerts P, Colombel JF, et al. Eldelumab [anti-interferon-γ-inducible protein-10 antibody] Induction Therapy for Active Crohn's Disease: a Randomised, Double-blind, Placebo-controlled Phase IIa Study. Journal of Crohns & Colitis. 2017;11:811-9. doi:10.1093/ecco-jcc/jjx005
39	Sandborn WJ, Rutgeerts P, Enns R, et al. Adalimumab Induction Therapy for Crohn Disease Previously Treated with Infliximab. Annals of Internal Medicine. 2007;146:829-. doi:10.7326/0003-4819-146-12-200706190-00159
40	Sandborn WJ, Schreiber S, Feagan BG, et al. Certolizumab pegol for active Crohn's disease: a placebo-controlled, randomized trial. Clin Gastroenterol Hepatol. 2011;9:670-8.e3. doi:10.1016/j.cgh.2011.04.031
41	Sands BE, Chen J, Feagan BG, et al. Efficacy and Safety of MEDI2070, an Antibody Against Interleukin 23, in Patients With Moderate to Severe Crohn's Disease: A Phase 2a Study. Gastroenterology. 2017;153:77-86.e6. doi:10.1053/j.gastro.2017.03.049
42	Sands BE, Feagan BG, Rutgeerts P, et al. Effects of Vedolizumab Induction Therapy for Patients With Crohn’s Disease in Whom Tumor Necrosis Factor Antagonist Treatment Failed. Gastroenterology. 2014;147:618-27.e3. doi:10.1053/j.gastro.2014.05.008
43	Saruta M, Park DI, Kim YH, et al. Anti-MAdCAM-1 antibody (PF-00547659) for active refractory Crohn's disease in Japanese and Korean patients: the OPERA study. Intest Res. 2020;18:45-55. doi:10.5217/ir.2019.00039
44	Schreiber S, Nikolaus S, Malchow H, et al. Absence of efficacy of subcutaneous antisense ICAM-1 treatment of chronic active Crohn's disease. Gastroenterology. 2001;120:1339-46. doi:10.1053/gast.2001.24015
45	Schreiber S, Rutgeerts P, Fedorak RN, et al. A randomized, placebo-controlled trial of certolizumab pegol (CDP870) for treatment of Crohn's disease. Gastroenterology. 2005;129:807-18. doi:10.1053/j.gastro.2005.06.064
46	Schreiber S, Siegel CA, Friedenberg KA, et al. A Phase 2, Randomized, Placebo-Controlled Study Evaluating Matrix Metalloproteinase-9 Inhibitor, Andecaliximab, in Patients With Moderately to Severely Active Crohn's Disease. J Crohns Colitis. 2018;12:1014-20. doi:10.1093/ecco-jcc/jjy070
47	Targan SR, Feagan B, Vermeire S, et al. A Randomized, Double-Blind, Placebo-Controlled Phase 2 Study of Brodalumab in Patients With Moderate-to-Severe Crohn's Disease. Am J Gastroenterol. 2016;111:1599-607. doi:10.1038/ajg.2016.298
48	Targan SR, Feagan BG, Fedorak RN, et al. Natalizumab for the treatment of active Crohn's disease: results of the ENCORE Trial. Gastroenterology. 2007;132:1672-83. doi:10.1053/j.gastro.2007.03.024
49	Targan SR, Hanauer SB, van Deventer SJ, et al. A short-term study of chimeric monoclonal antibody cA2 to tumor necrosis factor alpha for Crohn's disease. Crohn's Disease cA2 Study Group. N Engl J Med. 1997;337:1029-35. doi:10.1056/nejm199710093371502
50	van der Woude CJ, Stokkers P, van Bodegraven AA, et al. Phase I, double-blind, randomized, placebo-controlled, dose-escalation study of NI-0401 (a fully human anti-CD3 monoclonal antibody) in patients with moderate to severe active Crohn's disease. Inflamm Bowel Dis. 2010;16:1708-16. doi:10.1002/ibd.21252

51	Vermeire S, Schreiber S, Petryka R, et al. Clinical remission in patients with moderate-to-severe Crohn's disease treated with filgotinib (the FITZROY study): results from a phase 2, double-blind, randomised, placebo-controlled trial. Lancet. 2017;389:266-75. doi:10.1016/S0140-6736(16)32537-5
52	Vermeire S, Schreiber S, Rubin DT, D'Haens G, Reinisch W, Watanabe M, Mehta R, Roblin X, Beales I, Gietka P, Hibi T, Hospodarskyy I, Ritter T, Genovese MC, Kwon P, Santermans E, Le Brun FO, Barron R, Masior T, Danese S. Efficacy and safety of filgotinib as induction and maintenance therapy for Crohn's disease (DIVERSITY): a phase 3, double-blind, randomised, placebo-controlled trial. Lancet Gastroenterol Hepatol. 2025 Feb;10(2):138-153. doi: 10.1016/S2468-1253(24)00272-3
53	Watanabe K, Motoya S, Ogata H, et al. Effects of vedolizumab in Japanese patients with Crohn's disease: a prospective, multicenter, randomized, placebo-controlled Phase 3 trial with exploratory analyses. J Gastroenterol. 2020;55:291-306. doi:10.1007/s00535-019-01647-w
54	Watanabe M, Hibi T, Lomax KG, et al. Adalimumab for the induction and maintenance of clinical remission in Japanese patients with Crohn's disease. J Crohns Colitis. 2012;6:160-73. doi:10.1016/j.crohns.2011.07.013
55	Winter TA, Wright J, Ghosh S, et al. Intravenous CDP870, a PEGylated Fab' fragment of a humanized antitumour necrosis factor antibody, in patients with moderate-to-severe Crohn's disease: an exploratory study. Aliment Pharmacol Ther. 2004;20:1337-46. doi:10.1111/j.1365-2036.2004.02285.x
56	Peyrin-Biroulet L, Chapman JC, Colombel JF, et al. Risankizumab versus Ustekinumab for Moderate-to-Severe Crohn's Disease. N Engl J Med. 2024;391(3):213-223. doi:10.1056/NEJMoa2314585
57	Sandborn WJ, Panés J, Danese S, et al. Etrolizumab as induction and maintenance therapy in patients with moderately to severely active Crohn's disease (BERGAMOT): a randomised, placebo-controlled, double-blind, phase 3 trial. *Lancet Gastroenterol Hepatol*. 2023;8(1):43-55. doi:10.1016/S2468-1253(22)00303-X

TABLE S4. BASELINE DATA EXTRACTION. All data input, conversion, or assumptions data from the studies are highlighted and explained in the "obs" column. For better visualization access the excel version using the LINK

Author, year	Trial	Design	Country	Number of centers	Sample size (n)	Male (n)	Mean age (y; SD)	Disease localization (n)	Therapies	Class	dosage (dose; dosage)	route of administration	Follow up (weeks)	Concomitant medications (n, Drug)	prior use of biologics (n)	Duration of the disease (years; SD)	CDAI baseline (Index; SD)	Obs
Allez et al., 2017	NCT01203631	Double-blind, randomized	BE; CA; FR; HU; IS; PL; RU; US	52	40	20	36.5; 14.0	13 C; 10 I; 17 IC	NNC0142-0002	Anti -Natural Killer Group 2D	2 mg/kg; single dose	SC	24	25 ASA, 14 CTD, 19 IMD	12	NF	335.1; 66.5	
Allez et al., 2017	NCT01203631	Double-blind, randomized	BE; CA; FR; HU; IS; PL; RU; US	52	38	16	34.1; 11.6	12 C; 6 I; 18 IC	Placebo	-	Placebo	SC	24	27 ASA, 12 CTD, 20 IMD	11	NF	325.7; 64.0	
Allez et al., 2023	NCT02877134	Double-blind, randomized	BE, BG, CA, DE, FR, HU, JP, KO, PL, RO, RU, UA, UK, US	74	73	40	38.0; 13.3	NF	Tesnatilimab	Anti -Natural Killer Group 2D	400 mg, week 0, 200 mg, every 2 weeks from Week 2–22	SC	38	NF	73	NF	316.2; 63.5	In red: data obtained from the protocol
Allez et al., 2023	NCT02877134	Double-blind, randomized	BE, BG, CA, DE, FR, HU, JP, KO, PL, RO, RU, UA, UK, US	74	72	41	38.9; 13.3;	NF	Placebo	-	Placebo	SC	38	NF	72	NF	309.3; 56.5	
Chen et al., 2020	NCT02499783	Double-blind, randomized	CN	15	103	67	33.2; 10.2	19 C, 22 I, 60 IC, 9 UP	Adalimumab	Anti-TNF	160 mg, week 0, 80 mg, week 2, 60 mg week 4	SC	4	60 AZA; 30 CTD; 0 MCP; 1 MTX	103	3.1; 3.2	272.1; 48.1	It was only considered up to 4 weeks into the study
Chen et al., 2020	NCT02499783	Double-blind, randomized	CN	15	102	73	32.6; 9.5	24 C, 19 I, 60 IC, 10 UP	Placebo	-	Placebo	SC	4	59 AZA, 61 CTD, 2 MCP, 4 MTX	102	2.3; 2.7	274.7; 49.1	
D'Haens, Danese et al., 2022	NF	Double-blind, randomized	BE, CZ, FR, HU, IT, IS, JP, NL, PL, SK, UA	NF	37	24	31; 20-56	11 C, 5 I, 16 IC	Placebo	-	Placebo	OR	28	13 ASA, 19 CTD	25	6.86; 0.33-21.79	307; 63.2	
D'Haens, Danese et al., 2022	NF	Double-blind, randomized	BE, CZ, FR, HU, IT, IS, JP, NL, PL, SK, UA	NF	39	23	34; 20-58	9 C, 5 I, 22IC	Amiselimod	sphingosine-1-phosphate	0.4mg daily	OR	28	8 ATB, 12 ASA, 22 CTD	23	8.25; 0.32-29.15	35.1; 50.7	
D'Haens, Panaccione et al., 2022 a	NCT03105128	Double-blind, randomized	AR, AT, AU, BA, BE, BG, BR, BY, CA, CL, CH, CN, CO, CZ, DE, EE, ES, GR, HR, IL, IR, IT, JP, KO, LT, LV, MX, MY, NL, NO, NZ, PL, PT, RO, RS, RU,SE, SG, SK, UA, UK, US, ZA	297	336	189	38.3; 13.3	115 C, 52 I, 169 IC	Risankizumab	Inhibitor of interleukin-23	600 mg, week 0,2 and 4	IV	12	112 CTD, 88 IMD	195	9.0; 8.8	311.2; 62.4	In red: data obtained from the protocol
D'Haens, Panaccione et al., 2022 a	NCT03105128	Double-blind, randomized	AR, AT, AU, BA, BE, BG, BR, BY, CA, CL, CH, CN, CO, CZ, DE, EE, ES, GR, HR, IL, IR, IT, JP, KO, LT, LV, MX, MY, NL, NO, NZ, PL, PT, RO, RS, RU, SE, SG, SK, UA, UK, US, ZA	297	339	183	37.0; 13.2	118 C, 54 I, 167 IC	Risankizumab	Inhibitor of interleukin-23	1200 mg, week 0,2 and 4	IV	12	101 CTD, 73 IMD	199	8.9; 8.4	311.5; 68.4	
D'Haens, Panaccione et al., 2022 a	NCT03105128	Double-blind, randomized	AR, AT, AU, BA, BE, BG, BR, BY, CA, CL, CH, CN, CO, CZ, DE, EE, ES, GR, HR, IL, IR, IT, JP, KO, LT, LV, MX, MY, NL, NO, NZ, PL, PT, RO, RS, RU, SE, SG, SK, UA, UK, US, ZA	297	175	88	37·1;13. 4	70 C, 19 I, 86 IC	Placebo	-	Placebo	IV	12	50 CTD, 42 IMD	97	8.2; 7.1	319.2; 59.4	
D'Haens, Panaccione et al., 2022 b	NCT03104413	Double-blind, randomized	AR, AT, AU, BA, BE,BU, BY, CA, CH, CL, CN, CO, CZ, DE, DK, EE, ES, EG, FR, GR, HR, IR, IL, IT, KO, LV, LT, MX, MY, NL, NZ, PL, RO, RS, RU, SG, SK, TW, UA, UK, US, ZA	214	191	92	40.2; 13.6	75 C, 33 I, 83 I/C	Risankizumab	Inhibitor of interleukin-23	600 mg, week 0,2 and 4	IV	12	65 CTD, 36 IMD	191	10.9; 7.7	310.7; 63.6	In red: data obtained from the protocol
D'Haens, Panaccione et al., 2022 b	NCT03104413	Double-blind, randomized	AR, AT, AU, BA, BE,BU, BY, CA, CH, CL, CN, CO, CZ, DE, DK, EE, ES, EG, FR, GR, HR, IR, IL, IT, KO, LV, LT, MX, MY, NL, NZ, PL, RO, RS, RU, SG, SK, TW, UA, UK, US, ZA	214	191	102	39.3; 12.9	74 C, 21 I,, 96 I/C	Risankizumab	Inhibitor of interleukin-23	1200 mg, week 0,2 and 4	IV	12	62 CTD, 53 IMD	191	11.8; 9.1	312.5; 61.2	
D'Haens, Panaccione et al., 2022 b	NCT03104413	Double-blind, randomized	AR, AT, AU, BA, BE,BU, BY, CA, CH, CL, CN, CO, CZ, DE, DK, EE, ES, EG, FR, GR, HR, IR, IL, IT, KO, LV, LT, MX, MY, NL, NZ, PL, RO, RS, RU, SG, SK, TW, UA, UK, US, ZA	214	187	99	39.3; 13.5	73 C, 26 I , 88 I/C	Placebo	-	Placebo	IV	12	68 CTD, 40 IMD	187	12.5; 9.7	319.6; 69.8	
Danese et al., 2019	NCT01287897	Double-blind, randomized	AU, BE, BR, CA, CH, CZ, DE, DK, FR, GR, HU, IR, IL, IT, NZ, RO, UK, US,	128	67	33	38.9; 12.9	48 I, 32 LC, 30 R, 25 RC, 23 TC	PF-04236921	Inhibitor of interleukin-6	10 mg, day 1 and 28	SC	12	10 AZA, 17 CTD, 2 MCP, 8 MTX	67	11.0; 0.7–49.0	319.9; 61.9	In red: The data is presented in range, not in standard deviation.
Danese et al., 2019	NCT01287897	Double-blind, randomized	AU, BE, BR, CA, CH, CZ, DE, DK, FR, GR, HU, IR, IL, IT, NZ, RO, UK, US,	128	71	27	38.9; 13.1	33 I, 50 LC, 47 R, 28 RC, 34 TC	PF-04236921	Inhibitor of interleukin-6	50 mg, day 1 and 28	SC	12	13 AZA, 29 CTD, 3 MCP, 7 MTX	71	9.8; 0.3–43.0	296.7; 63.3	In blue: obtained by subtraction total patients and women
Danese et al., 2019	NCT01287897	Double-blind, randomized	AU, BE, BR, CA, CH, CZ, DE, DK, FR, GR, HU, IR, IL, IT, NZ, RO, UK, US,	128	40	15	42.2; 13.2	27 I, 29 LC, 23 R, 19 RC, 21 TC	PF-04236921	Inhibitor of interleukin-6	200 mg, day 1 and 28	SC	12	5 AZA, 1 CTD, 2 MCP, 19 MTX	40	11.5; 0.3–39.0	337.4; 73.4	

Author, year	Trial	Design	Country	Number of centers	Sample size (n)	Male (n)	Mean age (y; SD)	Disease localization (n)	Therapies	Class	dosage (dose; dosage)	route of administration	Follow up (weeks)	Concomitant medications (n, Drug)	prior use of biologics (n)	Duration of the disease (years; SD)	CDAI baseline (Index; SD)	Obs
Danese et al., 2019	NCT01287897	Double-blind, randomized	AU, BE, BR, CA, CH, CZ, DE, DK, FR, GR, HU, IR, IL, IT, NZ, RO, UK, US,	128	69	31	38.4; 13.6	40 I, 46 LC, 38 R, 34 RC, 30 TC	Placebo	-	Placebo	SC	12	10 AZA, 36 CTD, 6 MCP, 4 MTX	69	10.0; 1.2–36.0	320.7; 64.2	
Dotan et al. 2010	NF	Double-blind, randomized	Five countries	28	46	18	38; 14.1	NF	Semapimod	inhibit TLR	60mg, on day 1	IV	8	10 none, 22 ASA, 4 ATB, 19 CTD, 14 PUR, 2 MTX, 4 other	30	9; 6.8	330; 49	
Dotan et al. 2010	NF	Double-blind, randomized	Five countries	28	51	27	37; 37; 12.5	NF	Semapimod	inhibit TLR	60mg, on day 1-3	IV	8	5 none, 26 ASA, 6 ATB, 20 CTD, 17 PUR, 3 MTX, 11 other	26	10.3; 8.4	311; 47	
Dotan et al. 2010	NF	Double-blind, randomized	Five countries	28	55	24	35; 11.9	NF	Placebo	-	Placebo	IV	8	7 none, 24 ASA, 6 ATB, 23 CTD, 22 PUR, 6 MTX, 14 other	27	11; 9	324; 55	Red: data obtained by convert days to weeks
Fang et al., 2023	NF	Double-blind, randomized	CN	NF	40	17	37.13; 13.42	18 C, 6 I, 16 IC	Risankuzumab	Inhibitor of interleukin-23	600mg, every four weeks	IV	12	NF	NF	7.24; 1.11	295.49; 14.39	This study focuses on hospitalized patients
Fang et al., 2023	NF	Double-blind, randomized	CN	NF	40	18	37.22; 13.21	18 C, 5 I, 17 IC	Placebo	-	Placebo	IV	12	NF	NF	7.28; 1.21	296.08; 14.41	
Feagan et al., 2008	NCT00655135	Double-blind, randomized	CA	21	62	25	36.0; 12.67	NF	Vedolizumab	Integrin antagonist	0.5 mg/ kg, day 1 and 28	IV	8	55 MSL	62	105; 99.2	288.1; 48.63	This study refers to vedolizumab as MLN0002.
Feagan et al., 2008	NCT00655135	Double-blind, randomized	CA	21	65	31	38.5; 13.07	NF	Vedolizumab	Integrin antagonist	2 mg/ kg, day 1 and 28	IV	8	42 MSL	65	96; 94.8	296.6; 55.37	In red: The results are expressed in months, not years.
Feagan et al., 2008	NCT00655135	Double-blind, randomized	CA	21	58	30	34.5; 11.26	NF	Placebo	-	Placebo	IV	8	47 MSL	58	109; 99.3	288.0 ; 45.83	In blue: data obtained by converting values reported in the article (% to n; days to weeks)
Feagan et al., 2016 A	NCT01369329	Double-blind, randomized	AU, AT, BE, BR, CA, CZ, DE, DK, ES, FR, HU, IL, IR, IS, JP, KO, NL, NZ, PL, RS, UK, US, ZA	178	245	98	37.3; 12.5	35 C, 36 I, 166 I/C, 107 PE, 57 PR	Ustekinumab	Inhibitor of interleukin-12 and 23	130 mg, single dose	IV	8	50 ASA, 121 CTD, 74 IMD	245	11.8; 8.3	321.0; 64.7	In blue: Glucocorticoids have been classified as corticosteroids
Feagan et al., 2016 A	NCT01369329	Double-blind, randomized	AU, AT, BE, BR, CA, CZ, DE, DK, ES, FR, HU, IL, IR, IS, JP, KO, NL, NZ, PL, RS, UK, US, ZA	178	249	101	37.4; 11.8	40 C, 38 I, 171 I/C, 107 PE, 54 PR	Ustekinumab	Inhibitor of interleukin-12 and 23	6 mg/kg, single dose	IV	8	50 ASA, 108 CTD, 78 IMD	249	12.7; 9.2	327.6; 62.0	In red: data obtained from the protocol
Feagan et al., 2016 A	NCT01369329	Double-blind, randomized	AU, AT, BE, BR, CA, CZ, DE, DK, ES, FR, HU, IL, IR, IS, JP, KO, NL, NZ, PL, RS, UK, US, ZA	178	247	118	37.3; 11.8	48 C, 28 I, 166 I/C, 107 PE, 45 PR	Placebo	-	Placebo	IV	8	54 ASA, 111 CTD, 81 IMD	247	12.1; 8.4	319.0; 59.7	
Feagan et al., 2016 B	NCT01369342	Double-blind, randomized	AT, BE, BR, BG, CA, DE, ES, FR, HR HG, IL, IS, JP, KO, NL, NZ, PL, RS, RU, UK, US, ZA	175	209	104	38.4; 13.1	44 C, 53 I, 109 I/C, 60 PE, 34 PR	Ustekinumab	Inhibitor of interleukin-12 and 23	130 mg, single dose	IV	8	89 ASA, 80 CTD, 74 IMD	57	8.7; 8.4	304.1; 57.0	In red: data obtained from the protocol
Feagan et al., 2016 B	NCT01369342	Double-blind, randomized	AT, BE, BR, BG, CA, DE, ES, FR, HR HG, IL, IS, JP, KO, NL, NZ, PL, RS, RU, UK, US, ZA	175	209	90	39.1; 13.8	49 C, 43 I, 117 I/C, 61 PE, 29 PR	Ustekinumab	Inhibitor of interleukin-12 and 23	6 mg/kg, single dose	IV	8	93 ASA, 92 CTD, 72 IMD	65	8.7; 8.5	302.2; 58.9	In blue: Glucocorticoids have been classified as corticosteroids
Feagan et al., 2016 B	NCT01369342	Double-blind, randomized	AT, BE, BR, BG, CA, DE, ES, FR, HR HG, IL, IS, JP, KO, NL, NZ, PL, RS, RU, UK, US, ZA	175	210	99	40.2; 13.1	37 C, 44 I, 129 I/C, 57 PE, 32 PR	Placebo	-	Placebo	IV	8	89 ASA, 75 CTD, 73 IMD	79	10.4; 9.8	302.2; 61.7	
Feagan et al., 2017	NCT02031276.	Double-blind, randomized	AT, BE, CA, DE, KR, NL, ES, UK and US	36	41	15	39;13	10 C, 6 I, 25 I/C	Risankizumab	Inhibitor of interleukin-23	200 mg, week 0,2 and 4	IV	12	10 CTD; 10 IMD	37	14; 9	311; (247-375)	In red: supplementary material could not be retrieved

Author, year	Trial	Design	Country	Number of centers	Sample size (n)	Male (n)	Mean age (y; SD)	Disease localization (n)	Therapies	Class	dosage (dose; dosage)	route of administration	Follow up (weeks)	Concomitant medications (n, Drug)	prior use of biologics (n)	Duration of the disease (years; SD)	CDAI baseline (Index; SD)	Obs
Feagan et al., 2017	NCT02031276.	Double-blind, randomized	AT, BE, CA, DE, KR, NL, ES, UK and US	36	41	16	40; 13	14 C, 10 I, 16 I/C	Risankizumab	Inhibitor of interleukin-23	600 mg, week 0,2 and 4	IV	12	12 CTD; 10 IMD	39	14; 10	298; (259-330)	in green: The data is presented in range, not in standard deviation.
Feagan et al., 2017	NCT02031276.	Double-blind, randomized	AT, BE, CA, DE, KR, NL, ES, UK and US	36	39	16	36; 14	18 C, 5 I, 16 I/C	Placebo	-	Placebo	IV	12	11 CTD; 13 IMD	37	12; 9	295; (237-386)	
Ghosh et al., 2003	NF	Double-blind, randomized	BE, CZ, DE, DK, IL, SE, UK	35	68	27	36; 18–66	16 C, 9 I, 43 I/C	Natalizumab	Integrin antagonist	3 mg/kg, 1 infusion	IV	12	41 ASA, 26 IMD, 31 CTD	68	8.4; 0.5–27.6	288; 211–427	In red: The data is presented in range, not in standard deviation.
Ghosh et al., 2003	NF	Double-blind, randomized	BE, CZ, DE, DK, IL, SE, UK	35	66	30	36; 19–64	16 C, 17 I, 33 I/C	Natalizumab	Integrin antagonist	3 mg/kg, 2 infusion	IV	12	41 ASA, 17 IMD, 37 CTD	66	8.1; 0.5–21.9	300; 219–449	In blue: data obtained by subtracting the total number from the number of women
Ghosh et al., 2003	NF	Double-blind, randomized	BE, CZ, DE, DK, IL, SE, UK	35	51	25	35; 19–62	16 C, 12 I, 23 I/C	Natalizumab	Integrin antagonist	6 mg/kg, 2 infusion	IV	12	30 ASA, 9 IMD, 32 CTD	51	7.8; 0.6–29.0	298; 210–429	
Ghosh et al., 2003	NF	Double-blind, randomized	BE, CZ, DE, DK, IL, SE, UK	35	63	30	34; 18–68	11 C, 15 I, 37 I/C	Placebo	-	Placebo	IV	12	30 ASA, 22 IMD, 31 CTD	63	8.9; 0.3–64.3	300; 186–449	
Ghosh et al., 2024A,Loftus et al., 2023	NCT03345836	Double-blind, randomized	AR, AT, AU, BA, BE, BG, BR, CA, CH, CL, CO, CZ, DE, DK, EG, EE, ES, GR, HK, HR, HU, IR, IL, IT, JP, KO, LV, LT, MY, MX, NL, PL, PR, PT, RO, RU, SE, SG, SK, US, ZA, UA, UK	277	176	94	39.3; 13.6	57 C, 27 I, 92 IC	Placebo	-	Placebo	OR	12	3 IMS, 64 CTD	NF	5.7; 0.3-46.3	293.9; 85.4	in red: The data is presented in range, not in standard deviation.
Ghosh et al., 2024A,Loftus et al., 2023	NCT03345836	Double-blind, randomized	AR, AT, AU, BA, BE, BG, BR, CA, CH, CL, CO, CZ, DE, DK, EG, EE, ES, GR, HK, HR, HU, IR, IL, IT, JP, KO, LV, LT, MY, MX, NL, PL, PR, PT, RO, RU, SE, SG, SK, US, ZA, UA, UK	277	350	189	39.7; 13.7	121 C, 58, I, 171 IC	Upadacitinib	Janus kinase 1 inhibitor	45 mg, daily	OR	12	13 IMS, 126 CTD	NF	6.7; 0.1-52.1	292.4; 81.3	
Ghosh et al., 2024B, Loftus et al., 2023	NCT03345849	Double-blind, randomized	AR, AT, AU, BA, BE, BG, BR, CA, CH, CL, CO, CZ, DE, DK, EG, EE, ES, GR, HK, HR, HU, IR, IL, IT, JP, KO, LV, LT, MY, MX, NL, PL, PR, PT, RO, RU, SE, SG, SK, US, ZA, UA, UK	277	171	96	37.5; 12.1	68 C, 23 I, 80 IC	Placebo	-	Placebo	OR	12	13 IMS, 60 CTD	NF	9.8; 0.6-46.1	308..1; 84.3	
Ghosh et al., 2024B, Loftus et al., 2023	NCT03345849	Double-blind, randomized	AR, AT, AU, BA, BE, BG, BR, CA, CH, CL, CO, CZ, DE, DK, EG, EE, ES, GR, HK, HR, HU, IR, IL, IT, JP, KO, LV, LT, MY, MX, NL, PL, PR, PT, RO, RU, SE, SG, SK, US, ZA, UA, UK	277	324	169	38.4; 13.7	112 C, 48 I, 164 IC	Upadacitinib	Janus kinase 1 inhibitor	45 mg, daily	OR	12	24 IMS, 108 CTD	NF	9.3; 0.5-55.2	306.6; 89.4	
Hanauer et al., 2006	NF	Double-blind, randomized	BE, CA, CZ, NL, PL and US	55	74	39	39; 13	23 C, 45 I, 4 IC, 0 PE, 1 SB	Adalimumab	Anti-TNF	40 mg, week 0, 20 mg, week 2	SC	4	37 ASA, 10 ATB, 17 CTD, 22 IMD	74	NF	299; 57	
Hanauer et al., 2006	NF	Double-blind, randomized	BE, CA, CZ, NL, PL and US	55	75	25	38; 12	17 C, 47 I, 7 IC, 1 PE, 0 SB	Adalimumab	Anti-TNF	80 mg, week 0, 40 mg, week 2	SC	4	40 ASA, 7 ATB, 32 CTD, 21 IMD	75	NF	301; 61	
Hanauer et al., 2006	NF	Double-blind, randomized	BE, CA, CZ, NL, PL and US	55	76	36	39; 11	22 C, 40 I, 8 IC, 1 PE, 2 SB	Adalimumab	Anti-TNF	160 mg, week 0, 80 mg, week 2	SC	4	39 ASA, 4 ATB, 24 CTD, 22 IMD	76	NF	295; 52	
Hanauer et al., 2006	NF	Double-blind, randomized	BE, CA, CZ, NL, PL and US	55	74	37	37; 13	14 C, 50 I, 7IC, 0 PE, 0 SB	Placebo	-	Placebo	SC	4	37 ASA, 5 ATB, 25 CTD, 22 IMD	74	NF	296; 60	
Hueber et al., 2012	NCT01009281	Double-blind, randomized	AU, CA, DE, PL, US	25	39	24	37.3; 12.0	NF	Secukinumab	Inhibitor of interleukin-17A	10 mg/kg, day 1 and 28	IV	18	14 ASA, 7 ATB, 9 THP, 1 MTX	7	12.2; 9.4	307	
Hueber et al., 2012	NCT01009281	Double-blind, randomized	AU, CA, DE, PL, US	25	20	11	38.3;14.3	NF	Placebo	-	Placebo	IV	18	7 ASA, 1 ATB, 8 THP, 1 MTX	0	10.3; 6.5	301	
Panaccione et al., 2015	NCT00562887	Double-blind, randomized	AT, AU, BE, CA, DK,PR, US	60	45	22	35.5; 10.8	6A, 9R, 6GD, 3C, 3J, 33I, 5 other	Briakinumab	Inhibitor of interleukin-12 and 23	400 mg, every 4 weeks	IV	12	5 AZA, 16CTD, 3 MTX	34	11.75; 9.65	326.2; 59.6	

Author, year	Trial	Design	Country	Number of centers	Sample size (n)	Male (n)	Mean age (y; SD)	Disease localization (n)	Therapies	Class	dosage (dose; dosage)	route of administration	Follow up (weeks)	Concomitant medications (n, Drug)	prior use of biologics (n)	Duration of the disease (years; SD)	CDAI baseline (Index; SD)	Obs
Panaccione et al., 2015	NCT00562887	Double-blind, randomized	AT, AU, BE, CA, DK,PR, US	60	139	75	38.9; 12.3	25A, 31R, 3GD, 86C, 6J, 99I, 13 other	Briakinumab	Inhibitor of interleukin-12 and 23	700 mg, every 4 weeks	IV	12	20 AZA, 69 CTD, 9 MTX	105	11.53; 9.42	320.7; 65	
Panaccione et al., 2015	NCT00562887	Double-blind, randomized	AT, AU, BE, CA, DK,PR, US	60	46	25	41.8; 14.5	6A, 10 R, 27C, 2J, 31IL, 6 other	Placebo		Placebo	IV	12	7 AZA, 22CTD, 3MTX	34	11.07; 7.81	307.1; 55.3	
Reinisch et al., 2006	NF	Double-blind, randomized	AT, BE, FR and NL	7	6	0	39; 19-63	1 C, 0 I, 5 IC	Fontolizumab	Anti-IFN-γ	0.1 mg/kg, single dose	IV	4	0 ATB, 4 ASA, 1 AZA, 1 CTD, 0 MTX	NF	16.2; 1-21	340; NF	In red: The data is presented in range, not in standard deviation.
Reinisch et al., 2006	NF	Double-blind, randomized	AT, BE, FR and NL	7	14	7	37; 23-49	3 C, 3 I, 8 IC	Fontolizumab	Anti-IFN-γ	1 mg/kg, single dose	IV	4	1 ATB, 5 ASA, 4 AZA, 7 CTD, 2 MTX	NF	8.0; 1-19	289; NF	In blue:data obtained by convert days to weeks
Reinisch et al., 2006	NF	Double-blind, randomized	AT, BE, FR and NL	7	15	3	33; 26-42	1 C, 9 I, 5 IC	Fontolizumab	Anti-IFN-γ	4 mg/kg, single dose	IV	4	2 ATB, 3 ASA, 5 AZA, 7 CTD, 0 MTX	NF	5.1; 2-13	304; NF	
Reinisch et al., 2006	NF	Double-blind, randomized	AT, BE, FR and NL	7	10	4	30.5; 22-67	1 C, 3 I, 6 IC	Placebo	-	Placebo	IV	4	0 ATB, 3 ASA, 2 AZA, 4 CTD, 0 MTX	NF	7.3; 2-20	304; NF	
Reinisch et al., 2010	NF	Double-blind, randomized	AT, BE, CA, FR, HU, NL and US	31	41	20	33.0; 20–56	13 C, 18 I, 19 I/C, 4 UP	Fontolizumab	Anti-IFN-γ	1 mg/kg, day 1, 0.1 mg, day 29 every 4 weeks	IV/SC	16	39 ASA, 15 AZA, 39 CTD, 2 MTX	NF	9.1; NF	315.0; 241-427	
Reinisch et al., 2010	NF	Double-blind, randomized	AT, BE, CA, FR, HU, NL and US	31	40	19	34.5; 18–62	12 C, 13 I, 21 I/C, 4 UP	Fontolizumab	Anti-IFN-γ	1 mg/kg, day 1, 1 mg, day 29 every 4 weeks	IV/SC	16	50 ASA, 28 AZA, 32 CTD, 0 MTX	NF	10.8; NF	319.5; 211-478	
Reinisch et al., 2010	NF	Double-blind, randomized	AT, BE, CA, FR, HU, NL and US	31	40	20	34.5; 19–66	12 C, 14 I, 22 I/C, 12 UP	Fontolizumab	Anti-IFN-γ	4 mg/kg, day 1, 0.1 mg, day 29 every 4 weeks	IV/SC	16	50 ASA, 35 AZA, 28 CTD, 0 MTX	NF	10.0; NF	300.5; 224-429	in green: data obtained by sum treatment months plus follow up months
Reinisch et al., 2010	NF	Double-blind, randomized	AT, BE, CA, FR, HU, NL and US	31	40	17	34.0; 18–68	17 C, 15 I, 15 I/C, 0 UP	Fontolizumab	Anti-IFN-γ	4 mg/kg, day 1, 1 mg, day 29 every 4 weeks	IV/SC	16	55 ASA, 22 AZA, 38 CTD, 2 MTX	NF	8.4; NF	315.0; 234-457	
Reinisch et al., 2010	NF	Double-blind, randomized	AT, BE, CA, FR, HU, NL and US	31	40	15	36.0; 19–67	30C, 18 I, 21 I/C, 3 UP	Placebo	-	Placebo	IV/SC	16	42 ASA, 30 AZA, 45 CTD, 5 MTX	NF	7.9; NF	306.0; 246-509	
Rutgeerts et al., 2006	NF	Double-blind, randomized	North America, Europe, and Asia	27	44	15	34.0; 11.0	19 CLS, 22 CRS, 1 D, 31 I, 1 J	Onercept	Anti-TNF	10 mg, 3 times weekly for 8 weeks	SC	10	21 AI, 9 ATB, 9 AZA, 12 CTD	44	93.5; 69.3	307.2; 48.2/ 297.8; 46.2	In red: data of acute active Crohn's disease /chronic active Crohn's disease
Rutgeerts et al., 2006	NF	Double-blind, randomized	North America, Europe, and Asia	27	42	21	35.7; 11.5	20 CLS, 19 CRS, 0 D, 30 I, 1 J	Onercept	Anti-TNF	25 mg, 3 times weekly for 8 weeks	SC	10	18 AI, 8 ATB, 11 AZA, 14 CTD	42	146.3; 101.9	312.2; 52.4/ 341.0; 64.0	In green: The results are expressed in months, not years.
Rutgeerts et al., 2006	NF	Double-blind, randomized	North America, Europe, and Asia	27	40	17	39.2; 13.2	22 CLS, 19 CRS, 0 D, 30 I, 3 J	Onercept	Anti-TNF	35 mg, 3 times weekly for 8 weeks	SC	10	18 AI, 12 ATB, 11 AZA, 13 CTD	40	125.2; 87.1	325.3; 44.0/ 324.7; 51.9	
Rutgeerts et al., 2006	NF	Double-blind, randomized	North America, Europe, and Asia	27	43	18	35.6; 10.8	34 CLS, 14 CRS, 1 D, 34 I, 1 J	Onercept	Anti-TNF	50 mg, 3 times weekly for 8 weeks	SC	10	20 AI, 11 ATB, 10 AZA, 21 CTD	43	112.4; 102.6	315.8; 49.6/ 308.8; 47.3	
Rutgeerts et al., 2006	NF	Double-blind, randomized	North America, Europe, and Asia	27	38	22	39.1; 11.3	14CLS, 13 CRS, 31 D, 29 I, 2 J	Placebo	-	Placebo	SC	10	19 AI, 5 ATB, 9 AZA, 13 CTD	38	150.4; 94.8	311.9; 51.4/ 300.5; 37.7	
Rutgeerts et al., 2008	NF	Double-blind, randomized	NF	58	74	NF	NF	NF	Certolizumab pegol	Anti-TNF	100 mg, week 0, 4 and 8	SC	12	37 ASA, 13 AZA, 24 CTD, 9 MCT	NF	NF	299.2; 194–520	
Rutgeerts et al., 2008	NF	Double-blind, randomized	NF	58	72	NF	NF	NF	Certolizumab pegol	Anti-TNF	200 mg, week 0, 4 and 8	SC	12	32 ASA, 23 AZA, 29 CTD, 2 MCT	NF	NF	310.7; 184–446	In red: The data is presented in range, not in

Author, year	Trial	Design	Country	Number of centers	Sample size (n)	Male (n)	Mean age (y; SD)	Disease localization (n)	Therapies	Class	dosage (dose; dosage)	route of administration	Follow up (weeks)	Concomitant medications (n, Drug)	prior use of biologics (n)	Duration of the disease (years; SD)	CDAI baseline (Index; SD)	Obs
																		standard deviation.
Rutgeerts et al., 2008	NF	Double-blind, randomized	NF	58	72	NF	NF	NF	Certolizumab pegol	Anti-TNF	400 mg, week 0,4 and 8	SC	12	28 ASA, 22 AZA, 22 CTD, 2 MCT	NF	NF	304.5; 204–461	
Rutgeerts et al., 2008	NF	Double-blind, randomized	NF	58	73	NF	NF	NF	Placebo	-	Placebo	SC	12	29 ASA, 17 AZA, 29 CTD, 4 MCT	NF	NF	291.5; 206–448	
Sandborn, Hanauer et al., 2001	NF	Double-blind, randomized	US	6	23	16	37.4; 20–69	2 C, 3 I, 18 IC	Etanercept	Anti-TNF	25 mg, twice weekly for 8 weeks	SC	8	11 ASA, 4 ATB, 10 CTD, 1 MTX, 10 THP	12	NF	303; 226–499	In red: The data is presented in range, not in standard deviation.
Sandborn, Hanauer et al., 2001	NF	Double-blind, randomized	US	6	20	10	39.3; 22–60	5 C, 5 I, 10 IC	Placebo	-	NF	SC	8	9 ASA, 5 ATB, 7 CTD, 1 MTX, 7 THP	8	NF	265; 115–453	
Sandborn, Feagan et al., 2001	NF	Double-blind, randomized	CA, GB, US	21	29	16	28; 18-71	8 C, 4 I, 17 IC	CDP571	Anti-TNF	10 mg/kg, week 0 and 8	IV	24	17 ASA, 5 AZA/ MCT, 18 CTD, 0 MTX	29	4.7; 0-32	327; 226-473	In red: The data is presented in range, not in standard deviation.
Sandborn, Feagan et al., 2001	NF	Double-blind, randomized	CA, GB, US	21	28	12	33; 22-76	10 C, 8 I, 10 IC	CDP571	Anti-TNF	20 mg/kg, week 0 and 8	IV	24	17 ASA, 7 AZA/ MCT, 14 CTD, 1 MTX	28	8.2; 0-24	289; 222-438	
Sandborn, Feagan et al., 2001	NF	Double-blind, randomized	CA, GB, US	21	27	14	34; 20-66	9 C, 6 I, 11 IC	Placebo	-	Placebo 8	IV	24	17 ASA, 10 AZA/ MCT, 9 CTD, 2 MTX	27	11.3, 1-32	320; 223-449	
Sandborn, Feagan et al., 2001	NF	Double-blind, randomized	CA, GB, US	21	25	13	38; 24-57	6 C, 7 I, 12 IC	CDP571	Anti-TNF	10 mg/kg, week 0 and 12	IV	24	13 ASA, 8 AZA/ MCT, 13 CTD, 1 MTX	25	13.8; 1-31	319; 240-462	
Sandborn, Feagan et al., 2001	NF	Double-blind, randomized	CA, GB, US	21	29	16	32; 23-67	6 C, 8 I, 15 IC	CDP571	Anti-TNF	20 mg/kg, week 0 and 12	IV	24	12 ASA, 7 AZA/ MCT, 17 CTD, 2 MTX	29	7.7; 0-26	304; 217-486	
Sandborn, Feagan et al., 2001	NF	Double-blind, randomized	CA, GB, US	21	31	15	31; 18-62	3 C, 7 I, 21 IC	Placebo	-	Placebo 12	IV	24	16 ASA, 10 AZA/ MCT, 18 CTD, 3 MTX	31	7.9; 0-33	343; 216-466	
Sandborn et al., 2004	NF	Double-blind, randomized	AT, BU, CA, CZ, HG, PL, US	68	263	105	37.9; 12.4	72 C, 45 I, 145 IC	CDP571	Anti-TNF	10 mg/kg, week 0, 8, 16 and 24	IV	28	157 ASA, 39 ATB, 81 AZA/MCT, 109 CTD, 9 MTX	63	9.1; 8.1	297; 29	
Sandborn et al., 2004	NF	Double-blind, randomized	AT, BU, CA, CZ, HG, PL, US	68	132	43	37.7; 12.5	35 C, 25 I, 71 IC	Placebo	-	Placebo	IV	28	76 ASA, 18 ATB, 36 AZA/MCT, 54 CTD, 4 MTX	28	8.4; 7.6	301; 61	
Sandborn et al., 2005	NCT00032786	Double-blind, randomized	AT, AU, BE, CA, CZ, DE, DK, ES, FR, IE, IL, IT, NL, NZ, NZ, SE, UK, US and ZA	142	724	311	38; 12	157 C, 194 I, 373 I/C	Natalizumab	Integrin antagonist	300 mg, week 1, 4, 8 and 12	IV	12	343 ASA, 43 ATB, 271 CTD, 247 IMS	291	10.08; 7.67	302; 60	
Sandborn et al., 2005	NCT00032786	Double-blind, randomized	AT, AU, BE, CA, CZ, DE, DK, ES, FR, IE, IL, IT, NL, NZ, NZ, SE, UK, US and ZA	142	181	73	39; 14	49 C, 47 I, 84 I/C	Placebo	-	Placebo	IV	12	80 ASA, 12 ATB, 70 CTD, 52 IMS	69	9.17; 7.75	303; 65	
Sandborn, Rutgeerts et al., 2007	NCT00105300	Double-blind, randomized	BE, CA, FR, US	52	159	50	39; 12	105 C, 5 GD, 6 J, 112 I, 27 PE/A, 36 R	Adalimumab	Anti-TNF	160 mg, week 0, 80 mg week 2	SC	4	45 ASA, 55 CTD, 73 IMS	159	NF	313; 58	
Sandborn, Rutgeerts et al., 2007	NCT00105300	Double-blind, randomized	BE, CA, FR, US	52	166	65	37; 12	113C, 16 GD, 4 J, 124 I, 31 PE/A, 37 R	Placebo	-	Placebo	SC	4	60 ASA, 73 CTD, 85 IMS	166	NF	313; 66	
Sandborn, Feagan et al., 2007	NCT00152490	Double-blind, randomized	AT, AU, BE, BG, BY, CA, CZ, DE, EE, GE, HK, HU, IT, LV, NO, PL, RU, SE, SI, UK, US and ZA	171	331	157	37; 12	87 C, 149 IC, 95 TI	Certolizumab pegol	Anti-TNF	400 mg, week 0, 2, 4 and every four weeks	SC	26	72 CTD, 69 IMS, 57 CTD/IMS	100	7; <1–44	300; 64	In red: The data is presented in range, not in standard deviation.
Sandborn, Feagan et al., 2007	NCT00152490	Double-blind, randomized	AT, AU, BE, BG, BY, CA, CZ, DE, EE, GE, HK, HU, IT, LV, NO, PL, RU, SE, SI, UK, US and ZA	171	328	131	38; 12	74 C, 167 IC, 87 TI	Placebo		Placebo	SC	26	75 CTD, 66 IMS, 55 CTD/IMS	85	8; <1–40	297; 62	

Author, year	Trial	Design	Country	Number of centers	Sample size (n)	Male (n)	Mean age (y; SD)	Disease localization (n)	Therapies	Class	dosage (dose; dosage)	route of administration	Follow up (weeks)	Concomitant medications (n, Drug)	prior use of biologics (n)	Duration of the disease (years; SD)	CDAI baseline (Index; SD)	Obs
Sandborn et al., 2008A	NCT00265122	Double-blind, randomized	BE, CA, US	49	25	15	37; 13	17 C, 18 I, 4 PR	Ustekinumab	Inhibitor of interleukin-12 and 23	90 mg, week 0, 1, 2 and 3	SC	8	9 ASA, 2 ATB, 10 CTD, 5 IMS	14	12; 10	311; 80	cross-over study
Sandborn et al., 2008A	NCT00265122	Double-blind, randomized	BE, CA, US	49	26	15	37; 14	14 C, 21 I, 1 PR	Placebo	-	Placebo	SC	8	13 ASA, 2 ATB, 8 CTD, 10 IMS	16	13; 11	292; 40	
Sandborn et al., 2008B	NCT00265122	Double-blind, randomized	BE, CA, US	49	26	14	43; 12	11 C, 22 I, 0 PR	Ustekinumab	Inhibitor of interleukin-12 and 23	4.5 mg/kg, week 0, 1, 2 and 3	IV	8	10 ASA, 1 ATB, 7 CTD, 10 IMS	8	11; 9	325; 66	
Sandborn et al., 2008B	NCT00265122	Double-blind, randomized	BE, CA, US	49	27	13	44; 11	20 C, 19 I, 2 PR	Placebo	-	Placebo	IV	8	14 ASA, 2 ATB, 8 CTD, 10 IMS	11	13; 13	316; 56	
Sandborn et al., 2011	NF	Double-blind, randomized	AT, AU, BE, BR, CA, CL, CZ, DE, EE, FI, HU, IL, IT, LV, NZ, PL, RO, RU, UA, US	120	223	105	36.3; 12.6	65 C, 90 IC, 5 IP, 63 TI	Certolizumab pegol	Anti-TNF	400 mg, weeks 0, 2 and 4	SC	6	97 CTD, 77 IMS	223	7.5; 8.2	262.1; 59.0	
Sandborn et al., 2011	NF	Double-blind, randomized	AT, AU, BE, BR, CA, CL, CZ, DE, EE, FI, HU, IL, IT, LV, NZ, PL, RO, RU, UA, US	120	215	90	38.8; 12.8	61 C, 89 IC, 57 TI, 8 UP	Placebo	-	Placebo	SC	6	98 CTD, 67 IMS	215	7.0; 8.5	292.7; 61.1	
Sandborn, Colombel et al., 2012	NCT00406653	Double-blind, randomized	AU, BE, BR, CA, CH, CZ, DE, DK, FR, IN, IT, MX, NL, PL, PR, US, ZA	142	65	27	36.0; 11.1	NF	Abatacept	Inhibits T cell activation	30 mg/kg, week 0, 2 and 4	IV	12	24 CTD, 24 IMS	42	8.4; 7.5	320.6; 61.6	In blue: data obtained by convert % to n
Sandborn, Colombel et al., 2012	NCT00406653	Double-blind, randomized	AU, BE, BR, CA, CH, CZ, DE, DK, FR, IN, IT, MX, NL, PL, PR, US, ZA	142	128	50	38.6; 12.9	NF	Abatacept	Inhibits T cell activation	10 mg/kg, week 0, 2 and 4	IV	12	49 CTD, 53 IMS	86	9.9; 8.7	318.9; 65.1	
Sandborn, Colombel et al., 2012	NCT00406653	Double-blind, randomized	AU, BE, BR, CA, CH, CZ, DE, DK, FR, IN, IT, MX, NL, PL, PR, US, ZA	142	130	52	36.9; 13.4	NF	Abatacept	Inhibits T cell activation	3 mg/kg, week 0, 2 and 4	IV	12	43 CTD, 48 IMS	77	9.2; 8.0	317.9; 59.9	
Sandborn, Colombel et al., 2012	NCT00406653	Double-blind, randomized	AU, BE, BR, CA, CH, CZ, DE, DK, FR, IN, IT, MX, NL, PL, PR, US, ZA	142	128	45	38.0; 13.0	NF	Placebo	-	Placebo	IV	12	44 CTD, 54 IMS	77	9.8; 8.3	320.7; 72.1	
Sandborn, Gasink et al., 2012	NCT00771667	Double-blind, randomized	AT, AU, BE, CA, DE, ES, FR, IL, IT, NL, UK, US	153	131	48	38.8; 12.0	NF	Ustekinumab	Inhibitor of interleukin-12 and 23	1 mg, week 0	IV	8	20 ASA, 59 CTD, 33 IMD	131	12.2; 7.4	318.5; 62.4	In red: data obtained from the protocol
Sandborn, Gasink et al., 2012	NCT00771667	Double-blind, randomized	AT, AU, BE, CA, DE, ES, FR, IL, IT, NL, UK, US	153	132	57	38.2; 12.6	NF	Ustekinumab	Inhibitor of interleukin-12 and 23	3 mg, week 0	IV	8	22 ASA, 71 CTD, 28 IMD	132	12.0; 9.1	326.8; 63.1	
Sandborn, Gasink et al., 2012	NCT00771667	Double-blind, randomized	AT, AU, BE, CA, DE, ES, FR, IL, IT, NL, UK, US	153	131	48	39.4; 13.2	NF	Ustekinumab	Inhibitor of interleukin-12 and 23	6 mg, week 0	IV	8	25 ASA, 59 CTD, 35 IMD	131	12.7; 8.9	338.0; 67.3	
Sandborn, Gasink et al., 2012	NCT00771667	Double-blind, randomized	AT, AU, BE, CA, DE, ES, FR, IL, IT, NL, UK, US	153	132	68	39.5; 13.1	NF	Placebo	-	Placebo	IV	8	24 ASA, 73 CTD, 30 IMD	132	12.4; 9.1	312.4; 64.2	
Sandborn et al., 2013	NCT00783692	Double-blind, randomized	AT, AU, BE, BG, CA, CN, CZ, DE, DK, EE, ES, FR, GR, HG, IS, IN, IL, IR, IT, KO, LV, MY, NL, NO, NZ, PL, RO, RU, RS, SG, SK, SE, TW, UA, UK, US, ZA	285	220	105	36.3; 11.6	62 C, 37 I, 121 I/C	Vedolizumab	Integrin antagonist	300 mg, week 0 and 2	IV	6	67 CTD, 37 IMS, 38 CTD/IMS	111	9.2; 8.2	327; 71	In red: data obtained from the protocol
Sandborn et al., 2013	NCT00783692	Double-blind, randomized	AT, AU, BE, BG, CA, CN, CZ, DE, DK, EE, ES, FR, GR, HG, IS, IN, IL, IR, IT, KO, LV, MY, NL, NO, NZ, PL, RO, RU, RS, SG, SK, SE, TW, UA, UK, US, ZA	285	148	69	38.6; 13.2	43 C, 21 I, 84 I/C	Placebo	-	Placebo	IV	6	45 CTD, 25 IMS, 26 CTD/IMS	72	8.2; 7.8	325; 78	
Sandborn et al., 2014	NCT00615199	Double-blind, randomized	BE, CZ, FR, ES, HU, IT, NL, PL, SK, UK, US, ZA	48	34	12	35.7; 12.7	14C, 9I, 16IC	Placebo	-	Placebo	OR	4	NF	NF	8.2; 0.1-35.6	306.4; 62.6	
Sandborn et al., 2014	NCT00615199	Double-blind, randomized	BE, CZ, FR, ES, HU, IT, NL, PL, SK, UK, US, ZA	48	36	25	36.6; 12.2	20C, 6I, 15IC	Tofacitinib	Janus kinase 1 inhibitor	1mg, twice for day	OR	4	NF	NF	11.1; 0.1-28.5	300.3; 76.7	
Sandborn et al., 2014	NCT00615199	Double-blind, randomized	BE, CZ, FR, ES, HU, IT, NL, PL, SK, UK, US, ZA	48	34	14	38.7; 10.2	18C, 3I, 11IC	Tofacitinib	Janus kinase 1 inhibitor	5mg, twice for day	OR	4	NF	NF	10.9; 0.3-29.3	297.7; 63.7	
Sandborn et al., 2014	NCT00615199	Double-blind, randomized	BE, CZ, FR, ES, HU, IT, NL, PL, SK, UK, US, ZA	48	35	18	38.1; 11.7	17C, 10I, 17IC	Tofacitinib	Janus kinase 1 inhibitor	15mg, twice for day	OR	4	NF	NF	11.2; 1.5-36.3	308; 50.8	
Sandborn et al., 2017	NCT01466374	Double-blind, randomized	BE, FR, HG, IL, PL, US, ZA	28	40	23	35.8; 13.0	NF	Eldelumab	inhibitor of IP-10	10 mg/kg, days 1 and 8, and every otherweek thereafter to Day 64	IV	11	21 CTD, 11 IMS	25	8.7; 8.4	310; 59	In blue: data obtained by subtracting the total number of patients from the number of women
Sandborn et al., 2017	NCT01466374	Double-blind, randomized	BE, FR, HG, IL, PL, US, ZA	28	41	21	35.4; 13.1	NF	Eldelumab	inhibitor of IP-10	20 mg/kg, days 1 and 8, and every other week	IV	11	19 CTD, 7 IMS	24	9.3; 8.3	317; 57	

Author, year	Trial	Design	Country	Number of centers	Sample size (n)	Male (n)	Mean age (y; SD)	Disease localization (n)	Therapies	Class	dosage (dose; dosage)	route of administration	Follow up (weeks)	Concomitant medications (n, Drug)	prior use of biologics (n)	Duration of the disease (years; SD)	CDAI baseline (Index; SD)	Obs
											thereafter to Day 64							
Sandborn et al., 2017	NCT01466374	Double-blind, randomized	BE, FR, HG, IL, PL, US, ZA	28	40	18	37.3; 13.1	NF	Placebo	-	Placebo	IV	11	22 CTD, 11 IMS	27	9.5; 8.8	323; 67	
Sandborn et al., 2018; Saruta et al., 2020	NCT01276509	Double-blind, randomized	AT, BE, CA, DE, ES, FR, JP, KO, NL, NO, PL, SE, SV, US, ZA	103	66	18	37.3; 13.0	38 I, 49 LC, 49 R, 34 RC, 37 TC	Ontamalimab	inhibitor of MAdCAM-1	22.5 mg, week 0, 4 and 8	SC	12	31 CTD, 27 IMS	60	12.7; NF	307.4; 71.1	In blue: data obtained by subtracting the total number of patients from the number of women
Sandborn et al., 2018; Saruta et al., 2020	NCT01276509	Double-blind, randomized	AT, BE, CA, DE, ES, FR, JP, KO, NL, NO, PL, SE, SV, US, ZA	103	65	30	34.4; 10.7	40 I, 45 LC, 40 R, 36 RC, 34 TC	Ontamalimab	inhibitor of MAdCAM-1	75 mg, week 0, 4 and 8	SC	12	36 CTD, 28 IMS	60	11.4; NF	324.1; 63.1	in red: data obtained by the sum of the prior treatment strata (Relapsed, intolerance or no response to ≥1 anti-TNF-α)
Sandborn et al., 2018; Saruta et al., 2020	NCT01276509	Double-blind, randomized	AT, BE, CA, DE, ES, FR, JP, KO, NL, NO, PL, SE, SV, US, ZA	103	68	25	35.9; 11.0	43 I, 42 LC, 44 R, 25 RC, 27 TC	Ontamalimab	inhibitor of MAdCAM-1	225 mg, week 0, 4 and 8	SC	12	35 CTD, 26 IMS	63	12.0; NF	316.4; 64.6	
Sandborn et al., 2018; Saruta et al., 2020	NCT01276509	Double-blind, randomized	AT, BE, CA, DE, ES, FR, JP, KO, NL, NO, PL, SE, SV, US, ZA	103	63	33	34.4;11.1	40 I, 43 LC, 41 R, 40 RC, 36 TC	Placebo	-	Placebo	SC	12	29 CTD, 21 IMS	58	11.5; NF	313.1; 61.4	
Sandborn, Feagan et al., 2020	NCT02365649	Double-blind, randomized	IL, AU, NZ, europe	93	38	20	39.2; 13.9	NF	Upadacitinib	Janus kinase 1 inhibitor	3mg, daily, twice for day	OR	16	NF	37	10.7; 0.1-44.7	288; 180-445	in red: The data is presented in range, not in standard deviation.
Sandborn, Feagan et al., 2020	NCT02365649	Double-blind, randomized	IL, AU, NZ, europe	93	36	16	40.8; 13.5	NF	Upadacitinib	Janus kinase 1 inhibitor	6mg, daily, twice for day	OR	16	NF	36	8.8; 2.2-46.4	296; 230-599	
Sandborn, Feagan et al., 2020	NCT02365649	Double-blind, randomized	IL, AU, NZ, europe	93	36	19	40.8; 15.2	NF	Upadacitinib	Janus kinase 1 inhibitor	12mg, daily, twice for day	OR	16	NF	34	9.1; 1.2-38.8	280; 224-446	
Sandborn, Feagan et al., 2020	NCT02365649	Double-blind, randomized	IL, AU, NZ, europe	93	35	10	43.1; 9.64	NF	Upadacitinib	Janus kinase 1 inhibitor	24mg, daily, twice for day	OR	16	NF	36	14.1; 1.1-35.5	277; 162-556	
Sandborn, Feagan et al., 2020	NCT02365649	Double-blind, randomized	IL, AU, NZ, europe	93	34	15	40.4; 12.9	NF	Upadacitinib	Janus kinase 1 inhibitor	24mg, daily	OR	16	NF	33	10.8; 0.7-36.3	305; 231-421	
Sandborn, Feagan et al., 2020	NCT02365649	Double-blind, randomized	IL, AU, NZ, europe	93	37	13	40.5; 12.1	NF	Placebo		Placebo	OR	16	NF	35	8.7; 1.2-41.6	276; 188-447	
Sandborn et al., 2022	NCT03466411	Double-blind, randomized	AT, AU, BA, BE, BR, BY, CA, CN, CO, CZ, DE, ES, FR, GE, GR, HU, HR, IN, IT, JP, JO, KO, LB, LV, MK, MY, NL, NZ, PL, PR, PT, RS, SA, SK, TN, TR, TW, UA, UK, US	128	61	38	40.3; 13.67	27 C, 22 I, 12 I/C	Guselkumab	Inhibitor of interleukin-23	200 mg, week 0, 4 and 8	IV	12	24 CTD, 15 IMD	36	10.7; 12.17	304.6; 57.24	In red: data obtained from the protocol
Sandborn et al., 2022	NCT03466411	Double-blind, randomized	AT, AU, BA, BE, BR, BY, CA, CN, CO, CZ, DE, ES, FR, GE, GR, HU, HR, IN, IT, JP, JO, KO, LB, LV, MK, MY, NL, NZ, PL, PR, PT, RS, SA, SK, TN, TR, TW, UA, UK, US	128	63	36	39.0; 14.35	18 C, 23 I, 22 I/C	Guselkumab	Inhibitor of interleukin-23	600 mg, week 0, 4 and 8	IV	12	19 CTD, 18 IMD	39	10.4; 9.74	305.8; 58.77	
Sandborn et al., 2022	NCT03466411	Double-blind, randomized	AT, AU, BA, BE, BR, BY, CA, CN, CO, CZ, DE, ES, FR, GE, GR, HU, HR, IN, IT, JP, JO, KO, LB, LV, MK, MY, NL, NZ, PL, PR, PT, RS, SA, SK, TN, TR, TW, UA, UK, US	128	61	31	39.6; 13.72	31 C, 15 I, 15 I/C	Guselkumab	Inhibitor of interleukin-23	1200 mg, week 0, 4 and 8	IV	12	20 CTD, 25 IMD	36	6.7; 6.91	305.8; 54.46	
Sandborn et al., 2022	NCT03466411	Double-blind, randomized	AT, AU, BA, BE, BR, BY, CA, CN, CO, CZ, DE, ES, FR, GE, GR, HU, HR, IN, IT, JP, JO, KO, LB, LV, MK, MY, NL, NZ, PL, PR, PT, RS, SA, SK, TN, TR, TW, UA, UK, US	128	63	41	36.1; 12.02	29 C, 12 I, 22 I/C	Ustekinumab	Inhibitor of interleukin-12 and 23	6 mg/ kg, week 0, 90 mg, week 8	IV/SC	12	26 CTD, 26 IMD	44	7.4; 6.17	313.3; 61.30	

Author, year	Trial	Design	Country	Number of centers	Sample size (n)	Male (n)	Mean age (y; SD)	Disease localization (n)	Therapies	Class	dosage (dose; dosage)	route of administration	Follow up (weeks)	Concomitant medications (n, Drug)	prior use of biologics (n)	Duration of the disease (years; SD)	CDAI baseline (Index; SD)	Obs
Sandborn et al., 2022	NCT03466411	Double-blind, randomized	AT, AU, BA, BE, BR, BY, CA, CN, CO, CZ, DE, ES, FR, GE, GR, HU, HR, IN, IT, JP, JO, KO, LB, LV, MK, MY, NL, NZ, PL, PR, PT, RS, SA, SK, TN, TR, TW, UA, UK, US	128	61	37	38.9; 12.95	26 C, 16 I, 19 I/C	Placebo	-	Placebo	IV	12	24 CTD, 26 IMD	42	8.7; 6.54	300.8; 49.91	
Sands, Jacobson et al., 2010	NF	Double-blind, randomized	North America and Europe	60	73	27	40.6; 12.59	NF	Apilimod	inhibitor of IL-12/IL-23	50 mg, daily for 43 days	OR	53	2 ASA, 6 ATB, 2 BIO, 8 CTD, 2 MCP/AZA, 1 MTX	47	10.82; 68.64	304; 661	These studies included patients with concomitant biological treatment, but at a low percentage.
Sands, Jacobson et al., 2010	NF	Double-blind, randomized	North America and Europe	60	74	30	41.4; 11.94	NF	Apilimod	inhibitor of IL-12/IL-23	100 mg, daily for 43 days	OR	53	1 ASA, 9 ATB, 1 BIO, 9 CTD, 4 MCP/AZA, 2 MTX	45	11.35; 68.98	300; 659	
Sands, Jacobson et al., 2010	NF	Double-blind, randomized	North America and Europe	60	73	30	42.6; 13.07	NF	Placebo	inhibitor of IL-12/IL-23	Placebo	OR	53	4 ASA, 5 ATB, 2 BIO, 14 CTD, 1 MCP/AZA, 0 MTX	38	11.52; 69.72	305; 665	
Sands et al., 2014	NCT01224171/ EudraCT 2009-016488-12	Double-blind, randomized	AU, CA, US, NL, SK, HU, DE, AT, BE, CZ, IT	107	209	91	36.9; 20–69	48 C, 33 I, 128 IC	Vedolizumab	Integrin antagonist	300 mg, weeks 0, 2 and 6	IV	10	33 ASA, 53 CTD, 34 IMD	84	8.4, 0.3–41.8	313.9; 53.2	In blue: data obtained by subtracting the total number of patients from the number of women
Sands et al., 2014	NCT01224171/ EudraCT 2009-016488-12	Double-blind, randomized	AU, CA, US, NL, SK, HU, DE, AT, BE, CZ, IT	107	207	39	34.8; 19–77	52 C, 29 I, 126 IC	Placebo	-	Placebo	IV	10	29 ASA, 52 CTD, 33 IMD	93	8.0, 0.3–42.9	301.3; 55.0	In red: The data is presented in range, not in standard deviation.
Sands et al., 2017	NCT01714726	Double-blind, randomized	CA, CZ, DE, ES, FR, HU, IT, PL, US	49	59	22	34.9; 11.2	16 C, 14 I, 28 IC	MEDI2070	Inhibitor of interleukin-23	700 mg, week 0 and 4	IV	12	18 ASA, 24 CTD, 18 IMD	59	13.1; 9.4	325.0; 59.2	In blue: data obtained by subtracting the total number of patients from the number of women
Sands et al., 2017	NCT01714726	Double-blind, randomized	CA, CZ, DE, ES, FR, HU, IT, PL, US	49	60	23	38.1; 10.7	18 C, 18 I, 24 IC	Placebo	-	Placebo	IV	12	18 ASA, 24 CTD, 14 IMD	60	11.2 ; 8.5	312.4; 56.3	
Sands, Irving et al., 2022	NCT03464136/ EudraCT, 2017-004209-41	Double-blind, randomized	AU, BE, BR, BG, CA, CZ, DE, ES, FR, HU, IT, KO, NL, PL, RS, RU, UK, US	121	191	90	37.0;13.23	26 C, 60 I, 102 IC, 50 PE, 30 PR	Ustekinumab	Inhibitor of interleukin-12 and 23	6 mg/kg, week 0 and 2, 90 mg, every 8 weeks for 54 weeks	IV/SC	52	112 CTD	191	5.4; 8.36	301.6; 61.58	only the induction period was considered (week 16)
Sands, Irving et al., 2022	NCT03464136/ EudraCT, 2017-004209-41	Double-blind, randomized	AU, BE, BR, BG, CA, CZ, DE, ES, FR, HU, IT, KO, NL, PL, RS, RU, UK, US	121	195	95	37.4; 12.99	34 C, 55 I, 103 IC, 41 PE, 17 PR	Adalimumab	Anti-TNF	160 mg, week 0, 80 mg, week 2, 40 mg, every 2 weeks for 54 weeks	SC	52	121 CTD	195	5.8; 7.09	300.0; 55.99	In red: data obtained from the protocol
Sands, Peyrinn-Biroulet et al., 2022	NCT02891226	Double-blind, randomized	AU, BE, CA, CH, CZ, HU, JP, NL, PL, RO, UA, UK, US	80	31	17	38.1; 11.8	14 C, 6 I, 11 IC	Mirikizumab	Inhibitor of interleukin-23	200 mg, every four weeks for 12 weeks	IV	12	14 CTD, 12 IMD	19	8.9; 7.4	348.3; 92.1	
Sands, Peyrinn-Biroulet et al., 2022	NCT02891226	Double-blind, randomized	AU, BE, CA, CH, CZ, HU, JP, NL, PL, RO, UA, UK, US	80	32	14	40.4; 13.3	10 C, 5 I, 17 IC	Mirikizumab	Inhibitor of interleukin-23	600 mg, every four weeks for 12 weeks	IV	12	7 CTD, 10 IMD	19	10.8, 9.7	298.2; 103.7	In red: data obtained from the protocol
Sands, Peyrinn-Biroulet et al., 2022	NCT02891226	Double-blind, randomized	AU, BE, CA, CH, CZ, HU, JP, NL, PL, RO, UA, UK, US	80	64	34	37.7; 13.1	26 C, 11 I, 27 IC	Mirikizumab	Inhibitor of interleukin-23	1000 mg, every four weeks for 12 weeks	IV	12	15 CTD, 21 IMD	39	8.6, 6.7	304.5; 94.4	

Author, year	Trial	Design	Country	Number of centers	Sample size (n)	Male (n)	Mean age (y; SD)	Disease localization (n)	Therapies	Class	dosage (dose; dosage)	route of administration	Follow up (weeks)	Concomitant medications (n, Drug)	prior use of biologics (n)	Duration of the disease (years; SD)	CDAI baseline (Index; SD)	Obs
Sands, Peyrinn-Biroulet et al., 2022	NCT02891226	Double-blind, randomized	AU, BE, CA, CH, CZ, HU, JP, NL, PL, RO, UA, UK, US	80	64	28	39.0; 13.0	25 C, 11 I, 28 IC	Placebo	-	Placebo	IV	12	21 CTD, 19 IMD	43	10.2, 9.8	304.7; 93.1	
Schreiber et al., 2005	NF	Double-blind, randomized	BE, CA, DE, DK, IL, RU, RS, SE, UK, ZA	58	74	35	33.5; 18–56	35 AC, 45 CE, 5 D, 35 DC, 57 I, 20 P, 36 R, 32 TC	Certolizumab pegol	Anti-TNF	100 mg, week 0, 4 and 8	SC	12	37 ASA, 24 CTD, 26 IMD	18	7.73; 0.0–31.8	NF	In red: The data is presented in range, not in standard deviation.
Schreiber et al., 2005	NF	Double-blind, randomized	BE, CA, DE, DK, IL, RU, RS, SE, UK, ZA	58	72	22	40.1; 19–71	35 AC, 32 CE, 2 D, 40 DC, 51 I, 23 P, 33 R, 33 TC	Certolizumab pegol	Anti-TNF	200 mg, week 0, 4 and 8	SC	12	32 ASA, 29 CTD, 29 IMD	17	8.84; 0.0–30.7	NF	
Schreiber et al., 2005	NF	Double-blind, randomized	BE, CA, DE, DK, IL, RU, RS, SE, UK, ZA	58	72	32	35.9; 18–67	36 AC, 41 CE, 3 D, 47 DC, 56 I, 21 P, 32 R, 37 TC	Certolizumab pegol	Anti-TNF	400 mg, week 0, 4 and 8	SC	12	8 ASA, 22 CTD, 27 IMD	12	8.43; 0.2–26.5	NF	
Schreiber et al., 2005	NF	Double-blind, randomized	BE, CA, DE, DK, IL, RU, RS, SE, UK, ZA	58	73	24	35.8; 19–64	31 AC, 37 CE, 1 D, 42 DC, 54 I, 23 P, 36 R, 33 TC	Placebo	-	Placebo	SC	12	29 ASA, 29 CTD, 26 IMD	16	7.95; 0.1–27.6	NF	
Schreiber et al., 2018	NCT02405442	Double-blind, randomized	AU, DE, CA, CZ, ES, FR, HU, IT, NZ, PL, UK, US, ZA	73	53	28	38; 12.8	10 C, 7 I, 36 IC	Andecaliximab	inhibiting MMP9	150 mg, every 2 weeks for 8 weeks	SC	8	24 CTD, 19 IMD	46	12.7; 8.60	320; 54.8	In red: data obtained from the protocol
Schreiber et al., 2018	NCT02405442	Double-blind, randomized	AU, DE, CA, CZ, ES, FR, HU, IT, NZ, PL, UK, US, ZA	73	53	25	39; 13.5	13 C, 5 I, 35 IC	Andecaliximab	inhibiting MMP9	150 mg, every weeks for 8 weeks	SC	8	26 CTD, 17 IMD	45	11.4; 9.23	335; 61.4	
Schreiber et al., 2018	NCT02405442	Double-blind, randomized	AU, DE, CA, CZ, ES, FR, HU, IT, NZ, PL, UK, US, ZA	73	53	31	42; 11.7	11 C, 9 I, 33 IC	Andecaliximab	inhibiting MMP9	300mg, every weeks for 8 weeks	SC	8	24 CTD, 8 IMD	43	12.6; 10.80	329; 60.7	
Schreiber et al., 2018	NCT02405442	Double-blind, randomized	AU, DE, CA, CZ, ES, FR, HU, IT, NZ, PL, UK, US, ZA	73	28	13	38; 13.5	4 C, 4 I, 20 IC	Placebo	-	Placebo	SC	8	9 CTD, 8 IMD	24	13.4; 9.30	298; 61.6	
Targan et al., 1997	NF	Double-blind, randomized	BE, NL, US	18	27	14	37.0; 11.8	9 C, 3 I, 15 IC	Infliximab	Anti-TNF	5 mg/kg, week 0	IV	12	16 ASA, 5 AZA, 15 CTD, 4 MCP	27	12.5; 10.3	312; 56	in blue: data obtained of the sum of patients with <20mg/kg and >20mg/kh of prednisone
Targan et al., 1997	NF	Double-blind, randomized	BE, NL, US	18	28	13	39.3; 10.6	10 C, 4 I, 14 IC	Infliximab	Anti-TNF	10 mg/kg, week 0	IV	12	18 ASA, 4 AZA, 16 CTD, 4 MCP	28	11.5; 9.6	318; 59	
Targan et al., 1997	NF	Double-blind, randomized	BE, NL, US	18	28	13	36.0; 9.7	7 C, 2 I, 19 IC	Infliximab	Anti-TNF	20 mg/kg, week 0	IV	12	13 ASA, 8 AZA, 17 CTD, 4 MCP	28	13.5; 8.8	307; 50	
Targan et al., 1997	NF	Double-blind, randomized	BE, NL, US	18	25	15	38.5; 11.0	7 C, 8 I, 10 IC	Placebo	-	Placebo	IV	12	17 ASA, 7 AZA, 16 CTD, 4 MCP	25	10.4; 7.7	288; 54	
Targan et al., 2007	NF	Double-blind, randomized	NF	114	259	105	38.1	69 C, 56 I, 134 IC	Natalizumab	Integrin antagonist	300 mg, week 0, 4 and 8	IV	12	128 ASA, 17 ATB, 109 CTD, 97 IMD	130	121.4	303.9; 64.80	
Targan et al., 2007	NF	Double-blind, randomized	NF	114	250	102	37.7	65 C, 65 I, 120 IC	Placebo	-	Placebo	IV	12	120 ASA, 13 ATB, 94 CTD, 96 IMD	112	120.3	299.5; 63.19	
Targan et al., 2016	NF	Double-blind, randomized	AU, BE, CA, ES, FR, NL, PL, US	39	32	13	32.8; 10.2	7 C, 5 I, 19 IC	Brodalumab	Inhibitor of interleukin-17	210 mg, week 0 and 4	IV	12	18	29	9.6; 7.6	333; 62	
Targan et al., 2016	NF	Double-blind, randomized	AU, BE, CA, ES, FR, NL, PL, US	39	33	12	36.8; 12.6	11 C, 4 I, 17 IC	Brodalumab	Inhibitor of interleukin-17	350 mg, week 0 and 4	IV	12	19	29	14.2; 10.4	334; 60	
Targan et al., 2016	NF	Double-blind, randomized	AU, BE, CA, ES, FR, NL, PL, US	39	33	12	36.7; 10.0	13 C, 3 I, 16 IC	Brodalumab	Inhibitor of interleukin-17	700 mg, week 0 and 4	IV	12	21	28	11.7; 7.4	315; 54	
Targan et al., 2016	NF	Double-blind, randomized	AU, BE, CA, ES, FR, NL, PL, US	39	32	15	36.8; 13.0	8 C, 6 I, 17 IC	Placebo	-	Placebo	IV	12	18	25	11.4; 9.4	328; 63	
Vermeire et al., 2017	NCT02048618	Double-blind, randomized	BE, CZ, DE, FR, HU, PL, RO, RU,UK	52	130	59	37.4; 11.6	29C, 24I, 77IC	Filgotinib	Janus kinase 1 inhibitor	200mg, daily	PO	10	65CTD	73	8.8; 8.5	291.3; 53.8	

Author, year	Trial	Design	Country	Number of centers	Sample size (n)	Male (n)	Mean age (y; SD)	Disease localization (n)	Therapies	Class	dosage (dose; dosage)	route of administration	Follow up (weeks)	Concomitant medications (n, Drug)	prior use of biologics (n)	Duration of the disease (years; SD)	CDAI baseline (Index; SD)	Obs
Vermeire et al., 2017	NCT02048618	Double-blind, randomized	BE, CZ, DE, FR, HU, PL, RO, RU,UK	52	44	18	35.1; 11.8	6C, 7I, 31IC	Placebo		Placebo	PO	10	23CTD	28	6.8; 5.7	298.6; 56.8	
Watanabe et al., 2012	NCT00445939	Double-blind, randomized	JP	25	34	16	30.6; 9.3	NF	Adalimumab	Anti-TNF	160 mg, week 0, 80 mg, week 2	SC	4	27 ASA, 1 ATB, 6 CTD, 11 IMD	19	9.2; 6.6	302.7; 66.6	
Watanabe et al., 2012	NCT00445939	Double-blind, randomized	JP	25	33	20	32.0; 9.6	NF	Adalimumab	Anti-TNF	80 mg, week 0, 40 mg, week 2	SC	4	32 ASA, 2 ATB, 8 CTD, 10 IMD	20	11.0; 7.1	300.5; 66.5	
Watanabe et al., 2012	NCT00445939	Double-blind, randomized	JP	25	23	16	30.4; 6.9	NF	Placebo		Placebo	SC	4	23 ASA, 2 ATB, 5 CTD, 8 IMD	13	7.9; 4.7	308.1; 63.8	
Watanabe et al., 2020	NCT02038920	Double-blind, randomized	JP	77	79	51	33.9; 12.3	11 C, 13 I, 55 IC	Vedolizumab	Integrin antagonist	300 mg, weeks 0, 2 and 6	IV	10	64 ASA, 13 CTD, 9 CTD/IMD	61	9.0; 6.2	303.9; 63.2	In red: Obtained from the supplementary material
Watanabe et al., 2020	NCT02038920	Double-blind, randomized	JP	77	78	52	32.6; 10.9	19 C, 9 I, 50 IC	Placebo	-	Placebo	IV	10	59 ASA, 7 CTD, 11 CTD/IMD	62	9.1; 6.5	295.0; 64.8	
Winter et al., 2004	NF	Double-blind, randomized	ES, IL, NO, RS, UK and ZA	24	2	0	36.5; 31–42	NF	Certolizumab Pegol	Anti-TNF	1.25 mg, week 0	IV	12	0 ASA, 1 CTD, 0 IMD	1	9.28; 7.7–10.9	NF	In red: The data is presented in range, not in standard deviation.
Winter et al., 2004	NF	Double-blind, randomized	ES, IL, NO, RS, UK and ZA	24	25	12	36.4; 21–61	NF	Certolizumab Pegol	Anti-TNF	5 mg, week 0	IV	12	12 ASA, 6 CTD, 11 IMD	6	7.52; 0.6–17.0	NF	
Winter et al., 2004	NF	Double-blind, randomized	ES, IL, NO, RS, UK and ZA	24	17	6	40.3; 18–64	NF	Certolizumab Pegol	Anti-TNF	10 mg, week 0	IV	12	9 ASA, 6 CTD, 9 IMD	5	10.20; 0.9–26.0	NF	
Winter et al., 2004	NF	Double-blind, randomized	ES, IL, NO, RS, UK and ZA	24	23	10	33.3; 19–60	NF	Certolizumab Pegol	Anti-TNF	20 mg, week 0	IV	12	9 ASA, 6 CTD, 10 IMD	7	7.94; 1.3–18.9	NF	
Winter et al., 2004	NF	Double-blind, randomized	ES, IL, NO, RS, UK and ZA	24	25	6	32.1; 18–56	NF	Placebo	-	Placebo	IV	12	10 ASA, 7 CTD, 11 IMD	3	7.74; 0.1–21.9	NF	
Vermeire et al., 2025	NCT03046056	Double-blind, randomized	AT, BE, CA,CZ, DE, , FR, HU, IT, UA, UK, US	38	237	107	38; 14	33 I, 98 C, 106 IC	Placebo	-	Placebo	PO	11	96 ASA, 76 CTD	125	NF	320;59.4	
Vermeire et al., 2025	NCT03046056	Double-blind, randomized	AT, BE, CA,CZ, DE, , FR, HU, IT, UA, UK, US	38	245	139	39; 14.1	31 I, 104 C, 110 IC	Filgotinib	Janus kinase 1 inhibitor	100 mg , daily	PO	11	102 ASA, 84 CTD	134	NF	322; 55.5	
Vermeire et al., 2025	NCT03046056	Double-blind, randomized	AT, BE, CA,CZ, DE, , FR, HU, IT, UA, UK, US	38	222	112	39; 13.8	90 C, 31 C, 101 IC	Filgotinib	Janus kinase 1 inhibitor	200 mg, daily	PO	11	101 ASA, 74 CTD	121	NF	323; 55.6	
Peyrin-Biroulet et al., 2024	NCT04524611	Open label, randomized	AR, AT, AU, BE, BR, CA, CH, CL, CN, CZ, DE, ES, FR, GR, HU, IL, IT, KO,MX, NL, PL, RO, RU, SE, TR, UA, UK, US, ZA	187	255	136	38; 13.1	102 C, 42 I, 111 IL	Risankizumab	Inhibitor of interleukin-23	600 mg, week 0, 4 and 8	IV	8	58 CTD, 34 IMD	255	7.3; 0.3-40.6	306; 265.9-344.8	In red: The data is presented in range, not in standard deviation.
Peyrin-Biroulet et al., 2024	NCT04524611	Open label, randomized	AR, AT, AU, BE, BR, CA, CH, CL, CN, CZ, DE, ES, FR, GR, HU, IL, IT, KO,MX, NL, PL, RO, RU, SE, TR, UA, UK, US, ZA	187	265	131	38.3; 13.8	106 C, 45 I, 114 IL	Ustekinumab	Inhibitor of interleukin-12 and 23	4.5 mg/kg, week 0	IV	8	71 CTD, 47 IMD	265	7.3; 0.3–51.9	307.8; 260.8-347.9	
Sandborn et al., 2023	NCT02394028	Double-blind, randomized	AR, AT, AU, BR, BG, CA, CH, CZ, DE, EE, ES, FR, HR, HU, IL, IT, KO, LT, LV, MX, NL, NZ, PL, RO, RU, RS, SK, TK, UA, UK, US, ZA	326	97	59	37.4; 13.7	17 C, 23 C, 57 IC	Placebo	-	Placebo	SC	14	37 CTD, 24 IMD	40	7.9; 3.7–14.3	329.4; 64	
Sandborn et al., 2023	NCT02394028	Double-blind, randomized	AR, AT, AU, BR, BG, CA, CH, CZ, DE, EE, ES, FR, HR, HU, IL, IT, KO, LT, LV, MX, NL, NZ, PL, RO, RU, RS, SK, TK, UA, UK, US, ZA	326	143	74	38.3; 13.4	28 C, 27 I, 91 IC	Etrolizumab		105 mg, week 0, 4, 8, 12	SC	14	54 CTD, 39 IMD	67	6.1; 2–11.7	326.3; 60.4	In red: The data is presented in range, not in standard deviation.
Sandborn et al., 2023	NCT02394028	Double-blind, randomized	AR, AT, AU, BR, BG, CA, CH, CZ, DE, EE, ES, FR, HR, HU, IL, IT, KO, LT, LV, MX, NL, NZ, PL, RO, RU, RS, SK, TK, UA, UK, US, ZA	326	145	76	36.5; 13.1	37 C, 25 I, 83 IC	Etrolizumab		210 mg, week 0, 2, 4, 8, 12	SC	14	53 CTD, 36 IMD	72	6.9; 3–14.4	328; 61	

Legend: NF- not found, A- Anus, AC- Ascending colon, I- Ileal, IC- Ileocolonic, C-Colonic, CE- Cecum, CLS- Colon left side, CRS- Colon, right side, D- Duodenum, DC- Descending colon, GD- Gastroduodenal, J- Jejunum, LB- arge bowel, LC- Left colon, R- Rectum, RC- Right colon, SB- Small bowel, TC- Transverse colon, TI- Terminal ileum, PR- Proximal , PE- Perianal, IV- intrevenous, OR- oral, SB- small bowel , SC- subcutaneous, UP-Upper diasese; AR-argentina, AT- Austria AU- Australia, BA- Bosnia and Herzegovina, BE- belgium, BG- Bulgaria, BR- Brazil, BY- Belarus, CA- Canada, CH- Switzerland, CL- Chile, CN- China, CO- Colombia, CZ-Czech Republi, DE- Germany, DK- Denmark, EE- Estonia, EG- Egypt, ES- Spain, FI- Finland, FR- France, GB- Great Britain, GE- Georgia, GR- Greece, HK- Hong kong, HR- Croatia, HU- Hungary, IL- Israel, IN- India, IR- Ireland, IT- italy, IS- Iceland, JP- Japan, JO- Jordan, KO- Republic of Korea, LB- Lebanon, LT- Lithuania, LV- Latvia, MK- North Macedonia, MX, Mexico, MY- Malaysia, NL- Netherlands, LT- Lithuania, NO- Norway, NZ- New Zealand, PL- Poland, PT- Portugal, PR- Puerto Rico, RO- Romania, RU- Russian federation, RS- Serbia, SA- Saudi Arabia, SE- Sweden, SG- Singapore, SK- Slovakia, TN-Tunisia, TR- Turkey, TW-

Taiwan, UA- Ukraine, UK- United Kingdom, US- United States, ZA- South Africa, AI- anti-inflammatory, ATB- antibiotics, ASA- aminosalicylates,AZA- Azathioprine, BIO- Biological, CTD- Corticosteroids, IMD- Immunomodulator, IMS- Immunosuppressant , MCP- Mercaptopurine, PUR- Purine, MTX-methotrexate

TABLE S5. RISK OF BIAS ANALYSIS OF ADVERSE EFFECTS

Study	D1	D2	D3	D4	D5	Overall
Allez, 2017	Low	Low	Low	Low	Low	Low
Allez, 2022	Low	Low	Low	Low	Low	Low
Chen, 2020	Low	Low	Low	Low	Low	Low
D'haens, 1999	Low	Low	Low	Low	Some concerns[3]	Some concerns
D'Haens, Danese, 2022	Low	Low	Low	Some concerns[2]	Some concerns[3]	Some concerns
Danese, 2019	Low	Low	Low	Some concerns[2]	Low	Some concerns
D'Haens, Panaccione, 2022	Some concerns[1]	Low	Low	Low	Some concerns[6]	Some concerns
D'Haens, Panaccione, 2022	Some concerns[7]	Low	Low	Low	Some concerns[3]	Some concerns
Dotan, 2010	Some concerns[1]	Low	Low	Some concerns[2]	Some concerns[3]	High
Fang, 2023	Some concerns[1]	Low	Low	Some concerns[2]	Some concerns[3]	High
Feagan, 2008	Some concerns[1]	Low	Low	Some concerns[2]	Some concerns[3]	High
Feagan, 2016	Low	Low	Low	Some concerns[2]	Low	Some concerns
Feagan, 2016B	Some concerns[1]	Low	Low	Some concerns[2]	Low	Some concerns
Feagan, 2017	Low	Low	Low	Low	Low	Low
Ghosh, 2003	Low	Low	Low	Some concerns[2]	Some concerns[3]	Some concerns
Ghosh, 2024; Loftus, 2023 (U-EXCEED)	Low	Low	Low	Low	Low	Low
Ghosh, 2024; Loftus, 2023 (U-EXCEL)	Low	Low	Low	Low	Low	Low
Hanauer, 2006	Low	Low	Low	Low	Some concerns[3]	Some concerns
Hueber, 2012	Low	Low	Low	Some concerns[2]	Some concerns[3]	Some concerns
Panaccione, 2015	Low	Low	Low	Low	Some concerns[6]	Some concerns
Reinisch, 2006	Some concerns[1]	Low	Low	Some concerns[2]	Some concerns[3]	High
Reinisch, 2010	Some concerns[1]	Low	Low	Some concerns[2]	Some concerns[3]	High
Rutgeerts, 2008	Some concerns[1]	Low	Low	Some concerns[2]	Some concerns[3]	High
Rutgeerts, 2006	Low	Low	Low	Some concerns[2]	Some concerns[3]	Some concerns
Sandborn, 2001	Some concerns[1]	Low	Low	Some concerns[2]	Some concerns[3]	High
Sandborn, 2004	Low	Low	Low	Some concerns[2]	Some concerns[3]	Some concerns
Sandborn, 2005	Low	Low	Low	Low	Some concerns[6]	Some concerns
Sandborn, 2008	Low	Low	Low	Some concerns[2]	Low	Some concerns
Sandborn, 2011	Low	Low	Low	Some concerns[2]	Some concerns[3]	Some concerns
Sandborn, 2013	Low	Low	Low	Low	Low	Low
Sandborn, 2014	Low	Low	Low	Low	Some concerns[3]	Some concerns
Sandborn, 2017	Some concerns[1]	Low	Low	Some concerns[2]	Low	Some concerns
Sandborn, 2018	Low	Low	Low	Low	Low	Low
Sandborn, 2020	Low	Low	Low	Low	Low	Low
Sandborn, 2022	Low	Low	Low	Low	Low	Low
Sandborn, Colombel, 2012	Some concerns[1]	Low	Low	Some concerns[2]	Low	Some concerns
Sandborn, Feagan, 2007	Some concerns[1]	Low	Low	Some concerns[2]	Some concerns[3]	High
Sandborn, Gasink, 2012	Some concerns[1]	Low	Low	Some concerns[2]	Low	Some concerns
Sandborn, Hanauer, 2001	Some concerns[4]	Low	Some concerns	Some concerns[5]	Some concerns[3]	High
Sandborn, Rutgeerts, 2007	Low	Low	Low	Low	Low	Low
Sands, 2010	Some concerns[4]	Low	Low	Low	Some concerns[3]	Some concerns

Study	D1	D2	D3	D4	D5	Overall
**Sands, 2014**	Low	Low	Low	Low	Low	Low
**Sands, 2017**	Low	Low	Low	Low	Low	Low
**Sandborn, 2020**	Low	Low	Low	Low	Low	Low
**Sands, Irving et al., 2022**	Low	Low	Low	Low	Low	Low
**Sands, Peyrinn-Biroulet, 2022**	Low	Low	Low	Low	Low	Low
**Schreiber, 2005**	Low	Low	Low	Low	Some concerns[3]	Some concerns
**Schreiber, 2018**	Some concerns[1]	Low	Low	Some concerns[2]	Some concerns[3]	High
**Targan, 1997**	Low	Low	Low	Low	Some concerns[3]	Some concerns
**Targan, 2007**	Some concerns[1]	Low	Low	Low	Some concerns[3]	Some concerns
**Targan, 2016**	Low	Low	Low	Some concerns[2]	Some concerns[3]	Some concerns
**Vermeire, 2017**	Low	Low	Low	Low	Low	Low
**Vermeire, 2025**	Low	Low	Low	Low	Low	Low
**Watanabe, 2012**	Some concerns[1]	Low	Low	Some concerns[2]	Some concerns[3]	High
**Watanabe, 2020**	Some concerns[1]	Low	Low	Low	Low	Some concerns
**Winter, 2004**	Some concerns[1]	Low	Low	Some concerns[2]	Some concerns[3]	High
**Peyrin-Biroulet, 2024**	Low	Some concerns[8]	Low	High[2,8]	low	High
**Sandborn, 2023**	Low	Low	Low	Low	Low	Low

D1: Randomization process; D2 Deviations from intended interventions; D3: missing outcome data; D4: measurement of the outcome; D5: selection of the reported result; [1]: did not explain how the randomization was done; [2]: Adverse effect considers subjective data; [3]: protocol is not available; [4]: Placebo group has lower average CDAI and patients who did not meet the inclusion criteria (CDAI 150-250); [5]: ~50% n total study patients discontinue treatment; [6]: Safety outcomes were not clearly defined in the protocol or methods. As they were not well defined, it is not possible to know if they were all reported; [7]: Population difference in use of antibiotics;

TABLE S6. RISK OF BIAS ANALYSIS OF QUALITY OF LIFE (IBDQ)

Study	D1	D2	D3	D4	D5	Overall
Allez, 2017	Low	Low	Low	Low	Low	Low
Dotan, 2010	Some concerns[1]	Low	Low	Some concerns[1]	Some concerns[2]	High
Feagan, 2008	Some concerns[3]	Low	Low	Some concerns[1]	Some concerns[6]	High
Feagan, 2016; Sands, 2018	Low	Low	Low	Some concerns[1]	Some concerns[2]	Some concerns
Feagan, 2017	Low	Low	Low	Low	Some concerns[2]	Some concerns
Ghosh, 2003	Low	Low	Low	Some concerns[1]	Some concerns[6]	Some concerns
Ghosh, 2023A; Loftus, 2023 (U-EXCEED)	Low	Low	Low	Low	Low	Low
Ghosh, 2023A; Loftus, 2023 (U-EXCEL)	Low	Low	Low	Low	Low	Low
Hanauer, 2006	Low	Low	Low	Low	Some concerns[2]	Some concerns
Reinisch, 2010	Some concerns[3]	Low	Low	Some concerns[1]	Some concerns[2]	High
Rugteerts, 2008	Some concerns[1]	Low	Low	Some concerns[1]	Some concerns[2]	High
Rutgeerts, 2006	Low	Low	Low	Some concerns[1]	Some concerns[2]	Some concerns
Sandborn, 2004	Low	Low	Low	Some concerns[1]	Some concerns[2]	Some concerns
Sandborn, 2005	Low	Low	Low	Low	Some concerns[2]	Some concerns
Sandborn, 2014	Low	Low	Low	Low	Some concerns[2]	Some concerns
Sandborn, 2020	Low	Low	Low	Low	Low	Low
Sandborn, 2022	Low	Low	Low	Low	Some concerns[2]	Some concerns
Sandborn, hanauer, 2001	Some concerns[4]	Low	Low	Some concerns[5]	Some concerns[2]	High
Sandborn, Rutgeerts, 2007	Low	Low	Low	Low	Low	Low
Sands, 2010	Some concerns[4]	Low	Low	Low	Some concerns[6]	Some concerns
Sands, Peyrinn-Biroulet, 2022	Low	Low	Low	Low	Low	Low
Schreiber, 2005	Low	Low	Low	Low	Some concerns[2]	Some concerns
Targan, 1997	Low	Low	Low	Low	Some concerns[2]	Some concerns
Targan, 2007	Some concerns[3]	Low	Low	Low	Some concerns[2]	Some concerns
Vermeire, 2017	Low	Low	Low	Low	Low	Low
Watanabe, 2012	Some concerns[3]	Low	Low	Some concerns[1]	Some concerns[2]	High

D1: Randomization process; D2 Deviations from intended interventions; D3: missing outcome data; D4: measurement of the outcome; D5: selection of the reported result; [1]: IBDQ considers subjective data; [2]: IBDQ was not mentioned in the protocol; [3]: did not explain how the randomization was done; [4]: Placebo group has lower average CDAI and patients who did not meet the inclusion criteria (CDAI 150-250); [5]: >20% n total study patients discontinue treatment; [6]: protocol is not available

TABLE S7. RISK OF BIAS ANALYSIS OF CDAI

Study	D1	D2	D3	D4	D5	Overall
Allez, 2017	Low	Low	Low	Low	Low	Low
Allez, 2022	Low	Low	Low	Low	Low	Low
Chen, 2020	Low	Low	Low	Low	Low	Low
D'haens, 1999	Low	Low	Low	Low	Some concerns[2]	Some concerns
D'Haens, Danese, 2022	Low	Low	Low	Low	Some concerns[2]	Some concerns
Danese, 2019	Low	Low	Low	Low	Low	Low
D'Haens, Panaccione, 2022A	Some concerns[1]	Low	Low	Low	Low	Some concerns
D'Haens, Panaccione, 2022B	Some concerns[7]	Low	Low	Low	Low	Some concerns
Dotan, 2010	Some concerns[1]	Low	Low	Low	Some concerns[2]	Some concerns
Fang, 2023	Some concerns[1]	Low	Low	Low	Some concerns[2]	Some concerns
Feagan, 2008	Some concerns[1]	Low	Low	Low	Some concerns[2]	Some concerns
Feagan, 2016	Low	Low	Low	Low	Low	Low
Feagan, 2016B	Low	Low	Low	Low	Low	Low
Feagan, 2017	Low	Low	Low	Low	Low	Low
Ghosh, 2003	Low	Low	Low	Low	Some concerns[2]	Some concerns
Ghosh, 2024; Loftus, 2023 (U-EXCEED)	Low	Low	Low	Low	Low	Low
Ghosh, 2024; Loftus, 2023 (U-EXCEED)	Low	Low	Low	Low	Low	Low
Hanauer, 2006	Low	Low	Low	Low	Some concerns[2]	Some concerns
Hueber, 2012	Low	Low	Low	Low	Low	Low
Panaccione, 2015	Low	Low	Low	Low	Some concerns[2]	Some concerns
Reinisch, 2006	Some concerns[1]	Low	Low	Low	Some concerns[2]	Some concerns
Reinisch, 2010	Some concerns[1]	Low	Low	Low	Some concerns[2]	Some concerns
Rutgeerts, 2006	Low	Low	Low	Low	Some concerns[2]	Some concerns
Rutgeerts, 2008	Low	Low	Low	Low	Some concerns[2]	Some concerns
Sandborn, Feagan, 2001	Some concerns[1]	Low	Low	Low	Some concerns[2]	Some concerns
Sandborn, 2004	Low	Low	Low	Low	Some concerns[2]	Some concerns
Sandborn, 2005	Low	Low	Low	Low	Some concerns[5]	Some concerns
Sandborn, 2008	Low	Low	Some concerns[4]	Low	Low	Some concerns
Sandborn, 2011	Low	Low	Low	Low	Some concerns[2]	Some concerns
Sandborn, 2013	Low	Low	Low	Low	Low	Low
Sandborn, 2014	Low	Low	Low	Low	Low	Low
Sandborn, 2017	Some concerns[1]	Low	Low	Low	Low	Some concerns
Sandborn, 2018	Low	Low	Low	Low	Low	Low
Sandborn, 2020	Low	Low	Low	Low	Low	Low
Sandborn, 2022	Low	Low	Low	Low	Low	Low
Sandborn, Colombel, 2012	Some concerns[1]	Low	Low	Low	Low	Some concerns
Sandborn, Feagan, 2007	Some concerns[1]	Low	Low	Low	Low	Some concerns
Sandborn, Gasink, 2012	Some concerns[1]	Low	Low	Low	Low	Some concerns
Sandborn, Hanauer, 2001	Some concerns[6]	Low	Some concerns[4]	Low	Some concerns[2]	High
Sandborn, Rutgeerts, 2007	Low	Low	Low	Low	Low	Low
Sands, 2010	Some concerns[6]	Low	Low	Low	Some concerns[2]	Some concerns
Sands, 2014	Low	Low	Low	Low	Low	Low
Sands, 2017	Low	Low	Low	Low	Low	Low
Sands, Irving et al., 2022	Low	Low	Low	Low	Low	Low
Sands, Peyrinn-Biroulet, 2022	Low	Low	Low	Low	Low	Low
Schreiber, 2005	Low	Low	Low	Low	Some concerns[2]	Some concerns
Schreiber, 2018	Some concerns[1]	Low	Low	Low	Low	Some concerns
Targan, 1997	Low	Low	Some concerns[3]	Low	Some concerns[2]	Some concerns
Targan, 2007	Some concerns[1]	Low	Low	Low	Some concerns[2]	Some concerns
Targan, 2016	Low	Low	Low	Low	Some concerns[2]	Some concerns
Vermeire, 2017	Low	Low	Low	Low	Low	Low
Vermeire, 2025	Low	Low	Low	Low	Low	Low
Watanabe, 2012	Some concerns[1]	Low	Low	Low	Low	Some concerns
Watanabe, 2020	Some concerns[1]	Low	Low	Low	Some concerns[2]	Some concerns
Winter, 2004	Some concerns[1]	Low	Low	Low	Some concerns[2]	Some concerns
Peyrin-Biroulet, 2024	Low	Some concerns[8]	Low	Some concerns[8]	low	Some concerns
Sandborn, 2023	Low	Low	Low	Low	Low	Low

D1: Randomization process; D2 Deviations from intended interventions; D3: missing outcome data; D4: measurement of the outcome; D5: selection of the reported result; [1]: did not explain how the randomization was done; [2]: protocol is not available; [3]: n (~20% in 10 mg group) of patients was not assessed at week 2 but was assessed at week 4, and no explanation as to why was given in the article; [4]: >20%

n total study patients discontinue treatment; [5]: Does not inform what will be measured in the protocol; [6]: Some patients did not meet the inclusion criteria (CDAI >250); [7]: Population diference in use of antibiotics; [8]: open label study

FIG. S1. FUNNEL PLOT ANALYSIS

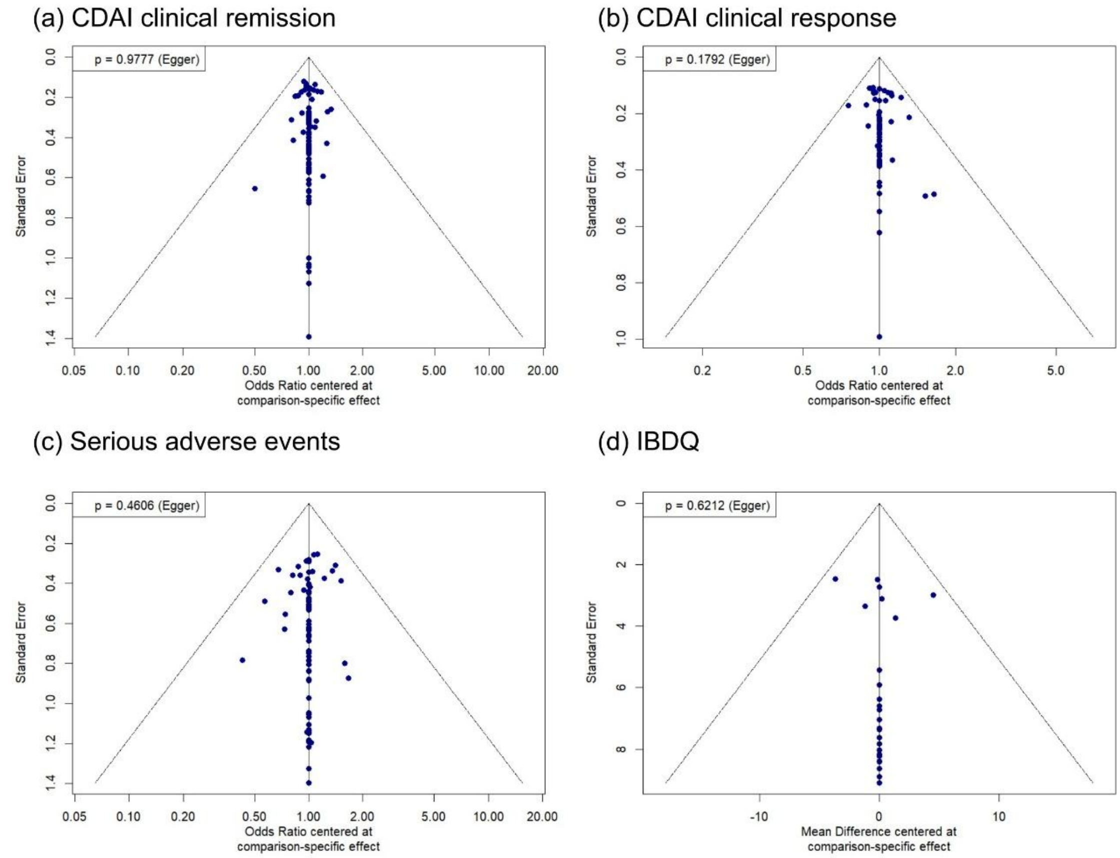

TABLES S8. GELMAN-RUBIN BROOKS ANALYSIS FOR THE BAYESIAN MODELS.

Outcome	PSRF Point est. (SD)*	PSRF Upper C.I. (SD)*
**Remission**	1.01	1.03
**Remission in the naive population**	1.00	1.00
**Remission in the prior population**	1.01	1.01
**Remission in 4 weeks**	1.00	1.00
**Remission in 6 weeks**	1.00	1.01
**Remission in 8 weeks**	1.00	1.01
**Remission in 12 weeks**	1.00	1.01
**Response with 100 points degree**	1.00	1.00
**Response with 70 points degree**	1.01	1.02
**IBDQ score**	1.00	1.00
**IBDQ remission**	1.00	1.00
**IBDQ response**	1.01	1.01
**SAE**	1.00	1.00
**AE**	1.00	1.01
**Infections**	1.00	1.01

*A potential scale reduction factor (PSRF) between 1 and 1.05 indicates convergence of the Bayesian model; IBDQ: inflammatory bowel disease questionnaire

TABLES S9. RESULTS FROM NODE SPLITTING ANALYSIS.

REMISSION		
comparison	p.value*	CrI
d.PBO.RIS600mgIV	0.613125	-
-> direct	-	0.96 (0.67, 1.2)
-> indirect	-	1.4 (-0.18, 3.1)
-> network	-	0.97 (0.69, 1.3)
d.PBO.UST4_5mg_kgIV	0.591875	-
-> direct	-	1.2 (-0.29, 2.8)
-> indirect	-	0.74 (0.24, 1.2)
-> network	-	0.78 (0.33, 1.3)
d.RIS600mgIV.UST4_5mg_kgIV	0.606042	-
-> direct	-	-0.20 (-0.62, 0.20)
-> indirect	-	0.20 (-1.2, 2.0)
-> network	-	-0.19 (-0.57, 0.22)
**REMISSION APPROVED**		
comparison	p.value*	CrI
d.PBO.RIS600mgIV	0.56	-
-> direct	-	0.96 (0.68, 1.2)
-> indirect	-	1.4 (-0.069, 3.1)
-> network	-	0.97 (0.70, 1.2)
d.PBO.UST4_5mg_kgIV	0.624792	-
-> direct	-	1.1 (-0.37, 2.7)
-> indirect	-	0.75 (0.25, 1.2)
-> network	-	0.78 (0.33, 1.2)
d.RIS600mgIV.UST4_5mg_kgIV	0.600625	-
-> direct	-	-0.22 (-0.61, 0.19)
-> indirect	-	0.25 (-1.4, 1.8)
-> network	-	-0.19 (-0.57, 0.20)
**REMISSION 8 WEEKS**		
Comparison	p.value*	CrI
d.PBO.RIS600mgIV	0.294792	-
-> direct	-	2.9 (0.78, 6.2)
-> indirect	-	1.4 (-0.44, 3.3)
-> network	-	1.9 (0.71, 3.5)
d.PBO.UST4_5mg_kgIV	0.288542	-
-> direct	-	1.2 (-0.43, 3.)
-> indirect	-	2.7 (0.51, 6.4)
-> network	-	1.6 (0.41, 3.1)
d.RIS600mgIV.UST4_5mg_kgIV	0.290833	-
-> direct	-	-0.21 (-0.89, 0.50)
-> indirect	-	-1.8 (-5.3, 0.94)
-> network	-	-0.27 (-1.1, 0.36)
**RESPONSE 100**		
comparison	p.value*	CrI
d.PBO.RIS600mgIV	0.12125	-
-> direct	-	0.96 (0.48, 1.5)
-> indirect	-	-0.40 (-2.2, 1.3)
-> network	-	0.89 (0.44, 1.3)
d.PBO.UST4_5mg_kgIV	0.0975	-
-> direct	-	-0.57 (-2.2, 0.88)
-> indirect	-	0.78 (-0.0023, 1.5)
-> network	-	0.56 (-0.24, 1.2)
d.RIS600mgIV.UST4_5mg_kgIV	0.112708	-
-> direct	-	-0.21 (-0.83, 0.40)
-> indirect	-	-1.6 (-3.2, 0.034)
-> network	-	-0.34 (-1.0, 0.17)
**REMISSION PRIOR**		
comparison	p.value*	CrI

d.PBO.RIS600mgIV	0.12375	-
-> direct	-	0.97 (0.48, 1.5)
-> indirect	-	-0.37 (-2.2, 1.3)
-> network	-	0.90 (0.44, 1.3)
d.PBO.UST4_5mg_kgIV	0.104583	-
-> direct	-	-0.57 (-2.2, 0.90)
-> indirect	-	0.77 (0.045, 1.5)
-> network	-	0.55 (-0.26, 1.1)
d.RIS600mgIV.UST4_5mg_kgIV	0.102917	-
-> direct	-	-0.22 (-0.81, 0.41)
-> indirect	-	-1.6 (-3.3, -0.022)
-> network	-	-0.34 (-1.0, 0.14)

*P values <0.05 indicate inconsistency in the networks

FIG. S2. NETWORK META-ANALYSIS PLOT OF CLINICAL REMISSION WITH DOSE AND ROUTE OF ADMINISTRATION:

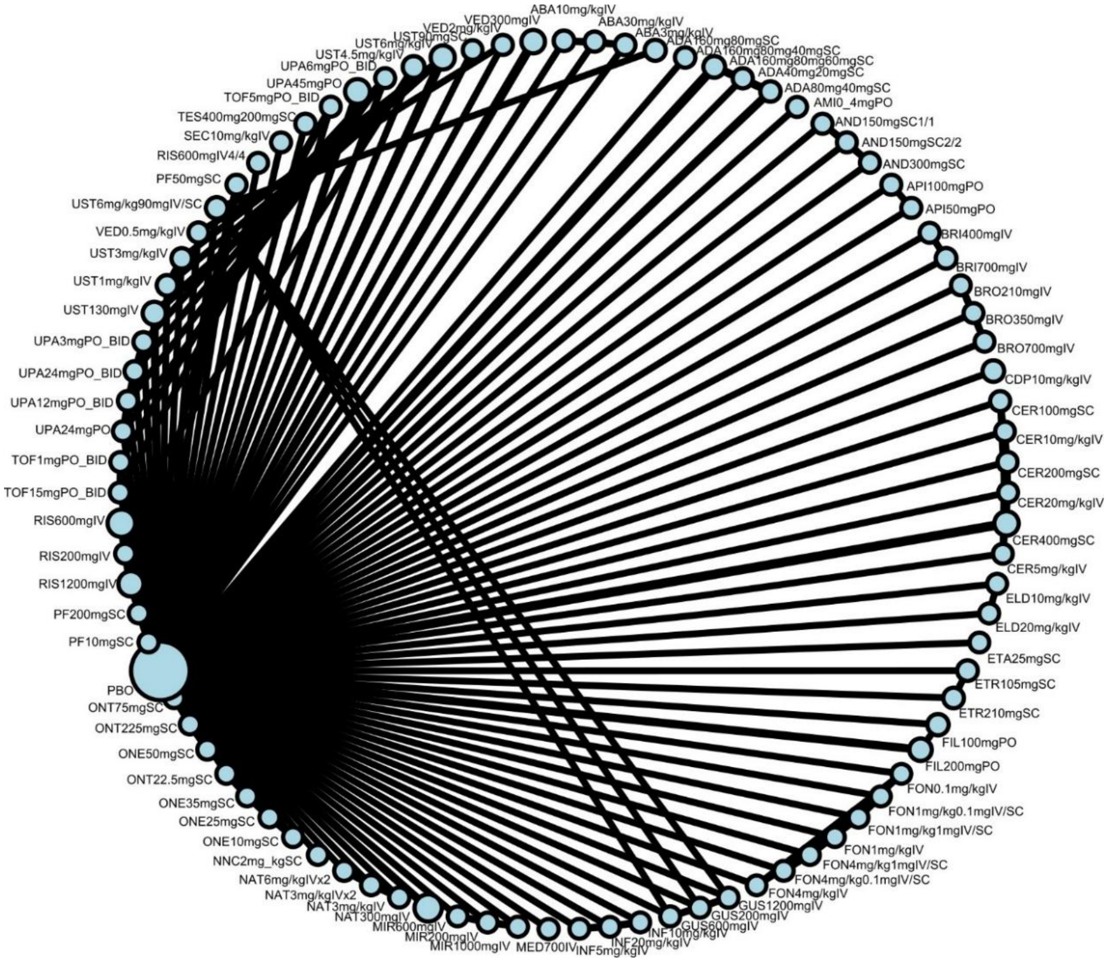

ABA: abatacept; ADA: adalimumab; AMI: Amiselimod; AND: andecaliximab; BRI: briakinumab; BRO: brodalumab; CDP: CDP571; CER: certolizumab pegol; ELD: eldelumab; ETA: etanercept; ETR: etrolizumb; FIL: filgotinib; FON: fontolizumab; GUS: guselkumab; INF: infliximab; MED: MEDI2070; MIR: mirikizumab; NAT: natalizumab; NNC: NNC0142-0002; ONE: onercept; ONT: ontamalimab; PF: PF-0423921; RIS: risankizumab; SEC: secukinumab; TES: tesnatilimab; TOF: tofacitinib; UPA: Upadacitinib; UST: Ustekinumab; VED: vedolizumab

TABLE S10. GEOMETRIC METRIC FOR NETWORK META-ANALYSIS. Nodes represent the number of interventions in the network. Edges represent direct comparisons, and "Edges more than 1study" represent comparisons with more than one study. Density measures the connectedness of a graph. The mean thickness represents the total number of studies divided by the total number of edges, and the percentage of common comparators represents the directly connected nodes that can be considered "common comparators." The percentage of strong edges represents the number of studies on an edge, which is proportional to the direct evidence between two nodes. The greater the number of studies in a comparison, the more robust the evidence. For better visualization access the excel version using the LINK.

Outcome	Nodes	Edges	Studies	Edges more than 1study	Density	Mean thickness	Common comparator	strong edges
Remission	91	170	55	14	0.0	0.3	84.6	8.2
Remission in the naive population	27	46	15	4	0.1	0.3	77.8	8.7
Remission in the prior population	29	49	21	7	0.1	0.4	79.3	14.3
Remission in 4 weeks	53	95	30	8	0.1	0.3	81.1	8.4
Remission in 6 weeks	30	51	19	5	0.1	0.4	80.0	9.8
Remission in 8 weeks	44	80	24	4	0.1	0.3	81.8	5.0
Remission in 12 weeks	55	107	28	5	0.1	0.3	83.6	4.7
Remission approved	58	109	40	14	0.1	0.4	84.5	12.8
Response with 100 points degree	29	49	21	7	0.1	0.4	79.3	14.3
Response with 70 points degree	45	84	24	7	0.1	0.3	82.2	8.3
IBDQ score	23	44	12	4	0.2	0.3	82.6	9.1
IBDQ remission	13	21	7	4	0.3	0.3	76.9	19.0
IBDQ response	12	21	7	4	0.3	0.3	83.3	19.0
SAE	83	152	49	14	0.0	0.3	83.1	9.2
AE	88	164	50	13	0.0	0.3	83.0	7.9
Infections	67	121	41	13	0.1	0.3	80.6	10.7

TABLE S11. ABBREVIATIONS FOR THE TREATMENTS USED TO CONSTRUCT THE NETWORKS. For better visualization access the excel version using the LINK

Biologics					
**Therapies**	**Class**	**Route of administration**	**Posology**	**Abbreviation**	**Author, year**
NNC0142-0002	Anti -Natural Killer Group 2D	SC	2 mg/kg; single dose	NNC2mg/kgSC	Allez et al., 2017
Tesnatilimab	Anti -Natural Killer Group 2D	SC	400 mg, week 0, 200 mg, every 2 weeks from Week 2–22	TES400mgSC	Allez et al., 2023
NI-0401	anti-CD3	IV	0,05 mg, for 5 days	NI0.05mgIV	Woude et al., 2010
			0,1 mg, for 5 days	NI0.1mgIV	
			0,5 mg, for 5 days	NI0.5mgIV	
			1 mg, for 5 days	NI1mgIV	
			2 mg, for 5 days	NI2mgIV	
			10 mg, for 5 days	NI10mgIV	
Fontolizumab	Anti-IFN-γ	IV	0.1 mg/kg, single dose	FON0.1mg/kgIV	Reinisch et al., 2006
			1 mg/kg, single dose	FON1mg/kgIV	
			4 mg/kg, single dose	FON4mg/kgIV	
		IV/SC	1 mg/kg, day 1, 0.1 mg, day 29 every 4 weeks	FON1mg/kg0.1mg/kgIV/SC	Reinisch et al., 2010
			1 mg/kg, day 1, 1 mg, day 29 every 4 weeks	FON1mg/kg1mg/kgIV/SC	
			4 mg/kg, day 1, 0.1 mg, day 29 every 4 weeks	FONmg/kg1mg/kgIV/SC	
			4 mg/kg, day 1, 1 mg, day 29 every 4 weeks	FON4mg/kg1mg/kgIV/SC	
Infliximab	Anti-TNF	IV	5 mg/kg, single dose	INF5mg/kgIV	Targan et al., 1997 and D'Haens et al., 1999
			10 mg/kg, single dose	INF10mg/kgIV	
			20 mg/kg, single dose	INF20mg/kgIV	
Adalimumab	Anti-TNF	SC	160 mg, week 0, 80 mg, week 2, 60 mg week 4	ADA160mg80mg60mgSC	Chen et al., 2020
			40 mg, week 0, 20 mg, week 2	ADA40mg20mgSC	Hanauer et al., 2006
			80 mg, week 0, 40 mg, week 2	ADA80mg40mgSC	
			160 mg, week 0, 80 mg week 2	ADA160mg80mgSC	Sandborn, Rutgeerts et al., 2007; Watanabe et

					al., 2012 and Hanauer et al., 2006
			160 mg, week 0, 80 mg, week 2, 40 mg, every 2 weeks for 54 weeks	ADA160mg80mg40mgSC	Sands, Irving et al., 2022
Onercept	Anti-TNF	SC	10 mg, 3 times weekly for 8 weeks	ONE10mgSC	Rutgeerts et al., 2006
			25 mg, 3 times weekly for 8 weeks	ONE25mgSC	
			35 mg, 3 times weekly for 8 weeks	ONE35mgSC	
			50 mg, 3 times weekly for 8 weeks	ONE50mgSC	
Etanercept	Anti-TNF	SC	25 mg, twice weekly for 8 weeks	ETA25mgSC	Sandborn, Hanauer et al., 2001
CDP571	Anti-TNF	IV	10 mg/kg, week 0 and 8 or week 0 and 12	CDP10mg/kgIV	Sandborn, Feagan et al., 2001
			20 mg/kg, week 0 and 8 or week 0 and 12	CDP20mg/kgIV	
			10 mg/kg, week 0, 8, 16 and 24	CDP10mg/kgx4IV	Sandborn et al., 2004
Certolizumab pegol	Anti-TNF	SC	100 mg, week 0, 4 and 8	CER100mgSC	Rutgeerts et al., 2008
			200 mg, week 0, 4 and 8	CER200mgSC	Rutgeerts et al., 2008 and Schreiber et al., 2005
			400 mg, week 0, 2, 4 or week 0, 4, 8	CER400mgSC	Sandborn, Feagan et al., 2007; Schreiber et al., 2005, Rutgeerts et al., 2008 and Sandborn et al., 2011
		IV	1.25 mg, week 0	CER1.25mgIV	Winter et al., 2004
			5 mg, week 0	CER5mgIV	
			10 mg, week 0	CER10mgIV	
			20 mg, week 0	CER20mgIV	
Andecaliximab	inhibiting MMP9	SC	150 mg, every 2 weeks for 8 weeks	AND150mgx4SC	Schreiber et al., 2018
			150 mg, every weeks for 8 weeks	AND150mgx8SC	
			300mg, every weeks for 8 weeks	AND300mgSC	
Ustekinumab	Inhibitor of interleukin-12 and 23	SC	90 mg, week 0, 1, 2 and 3	UST90mgSC	Sandborn et al., 2008
		IV	4.5 mg/kg, week 0	UST4.5mg/kgIV	Sandborn et al., 2008, Peyrin-Biroulet et al., 2024
			1 mg, week 0	UST1mgIV	Sandborn, Gasink et al., 2012
			130 mg, single dose	UST130mgIV	Feagan et al., 2016

		IV/SC	6 mg/ kg, week 0, 90 mg, week 8	UST6mg/kg	Sandborn et al., 2022 and Sands, Irving et al., 2022
Briakinumab	Inhibitor of interleukin-12 and 23	IV	400 mg, every 4 weeks	BRI400mgIV	Panaccione et al., 2015
			700 mg, every 4 weeks	BRI700mgIV	
Guselkumab	Inhibitor of interleukin-23	IV	200 mg, week 0, 4 and 8	GUS200mgIV	Sandborn et al., 2022
MEDI2070	Inhibitor of interleukin-23	IV	600 mg, week 0, 4 and 8	GUS600mgIV	Sands et al., 2017
			1200 mg, week 0, 4 and 8	GUS1200mgIV	
			700 mg, week 0 and 4	MED700mgIV	
Mirikizumab	Inhibitor of interleukin-23	IV	200 mg, every four weeks for 12 weeks	MIR200mgIV	Sands, Peyrinn-Biroulet et al., 2022
			600 mg, every four weeks for 12 weeks	MIR600mgIV	
			1000 mg, every four weeks for 12 weeks	MIR1000mgIV	
Brodalumab	Inhibitor of interleukin-17	IV	210 mg, week 0 and 4	BRO210mgIV	Targan et al., 2016
			350 mg, week 0 and 4	BRO350mgIV	
			700 mg, week 0 and 4	BRO700mgIV	
PF-04236921	Inhibitor of interleukin-6	SC	10 mg, day 1 and 28	PF10mgSC	Danese et al., 2019
			50 mg, day 1 and 28	PF50mgSC	
			200 mg, day 1 and 28	PF200mgSC	
Risankizumab	Inhibitor of interleukin-23	IV	600 mg, week 0,2 and 4	RIS600mgIV	D'Haens, Panaccione et al., 2022 and Feagan et al., 2017, Peyrin-Biroulet et al., 2024
			1200 mg, week 0,2 and 4	RIS1200mgIV	D'Haens, Panaccione et al., 2022
			200 mg, week 0,2 and 4	RIS200mgIV	Feagan et al., 2017
			600mg, every four weeks	RIS400mgIV4/4	
Secukinumab	Inhibitor of interleukin-17A	IV	10 mg/kg, day 1 and 28	SEC10mg/kgIV	Hueber et al., 2012
Eldelumab	inhibitor of IP-10	IV	10 mg/kg, days 1 and 8, and every other week there after to Day 64	ELD10mg/kgIV	Sandborn et al., 2017
			20 mg/kg, days 1 and 8, and every other week there after to Day 64	ELD20mg/kgIV	
Ontamalimab	inhibitor of MAdCAM-1	SC	22.5 mg, week 0, 4 and 8	ONT22.5mgSC	Sandborn et al., 2018; Saruta et al., 2020
			75 mg, week 0, 4 and 8	ONT75mgSC	
			225 mg, week 0, 4 and 8	ONT225mgSC	

Etrolizumab	inhibitor of MAdCAM-1	SC	105 mg, week 0, 4, 8, 12	ETR105mgSC	Sandborn et al. 2023
			210 mg, week 0, 2, 4, 8, 12	ETR210mgSC	
Abatacept	Inhibits T cell activation	IV	30 mg/kg, week 0, 2 and 4	ABA30mg/kgIV	Sandborn, Colombel et al., 2012
			10 mg/kg, week 0, 2 and 4	ABA10mg/kgIV	
			3 mg/kg, week 0, 2 and 4	ABA3mg/kgIV	
Vedolizumab	Integrin antagonist	IV	0.5 mg/ kg, day 1 and 28	VED0.5mg/kgIV	Feagan et al., 2008
			2 mg/ kg, day 1 and 28	VED2mg/kgIV	
			300 mg, week 0 and 2	VED300mgIV	Sandborn et al., 2013; Watanabe et al., 2020 and Sands et al., 2014
Natalizumab	Integrin antagonist	IV	3 mg/kg, 1 infusion	NAT3mg/kgIV	Ghosh et al., 2003
			3 mg/kg, 2 infusion	NAT3mg/kgx2IV	
			6 mg/kg, 2 infusion	NAT6mg/kgIV	
			300 mg, week 1, 4, 8 and 12 or week 0, 4 and 8	NAT300mgIV	Sandborn et al., 2005 and Targan et al., 2007
**Small molecules**					
Therapies	Class	route of administration	Posology	Abbreviation	Author, year
Semapimod	Toll-like receptor 4	IV	60mg, on day 1	SEM60mgIV	Dotan et al. 2010
			60mg, on day 1-3	SEM60mgIVx3	Dotan et al. 2010
Upadacitinib	Janus kinase 1 inhibitor	OR	3mg, daily, twice for day	UPA3mgOR_BID	Sandborn, Feagan et al., 2020
			6mg, daily, twice for day	UPA6mgOR_BID	Sandborn, Feagan et al., 2020
			12mg, daily, twice for day	UPA12mgOR_BID	Sandborn, Feagan et al., 2020
			24mg, daily, twice for day	UPA24mgOR_BID	Sandborn, Feagan et al., 2020
			24mg, daily	UPA24mgOR	Sandborn, Feagan et al., 2020
			45 mg, daily	UPA45mgOR	Ghosh et al., 2023A,Loftus et al., 2023
Tofacitinib	Janus kinase 1 inhibitor	OR	1mg, twice for day	TOF1mgOR_BID	Sandborn et al., 2014
			5mg, twice for day	TOF5mgOR_BID	Sandborn et al., 2014
			15mg, twice for day	TOF15mgOR_BID	Sandborn et al., 2014
Filgotinib	Janus kinase 1 inhibitor	OR	200mg, daily	FIL200mgOR	Vermeire et al., 2017

Apilimod	inhibitor of IL-12/IL-23	OR	50 mg, daily for 43 days	API50mgOR	Sands, Jacobson et al., 2010
			100 mg, daily for 43 days	API100mgOR	Sands, Jacobson et al., 2010
Amiselimod	sphingosine-1-phosphate	OR	0.4mg daily	AMI0.4mgOR	D'Haens, Danese et al., 2022

TABLE S12. LEAGUE TABLES FOR CDAI OUTCOMES. Each table present the results for main network (remission), subgroups by population (those who have or have not received biologic treatment), the week when the endpoint was reported, clinical response results (with decreases of 100 and 70 points), and FDA-approved drugs. The table presented the multiple treatment comparisons based on consistency analysis of the networks. Treatments are depicted alphabetically. The upper quadrant is the inverse of the lower quadrant, i.e., the mirror image of the results. Values are presented as relative risk (RR) with 95% credible interval (CrI). For all comparisons an RR <1 favors the occurrence of the event for the row-defining treatment, while an RR>1 favors the column-defining treatment. Statistically significant results are represented in bold. Multiple treatment comparisons based on consistency analysis of the networks. Treatments are depicted alphabetically. Values are presented as relative risk (RR) with 95% credible interval (CrI). For all comparisons an RR <1 favors the occurrence of the event for the row-defining treatment, while an RR>1 favors the column-defining treatment. Statistically significant results are represented in bold. For better visualization access the excel version using the LINK

Table S12A: League table for clinical remission (main network).

	ABA10mg/kgIV	ABA30mg/kgIV	ABA3mg/kgIV	ADA160mg80mg40mgSC	ADA160mg80mg60mgSC	ADA160mg80mgSC	ADA40mg20mgSC	ADA80mg40mgSC	AMI0.4mgPO	AND150mgSC1.1	AND150mgSC2.2	AND300mgSC	API100mgPO	API50mgPO	BRI400mgIV	BRI700mgIV	BRO210mgIV	BRO350mgIV	BRO700mgIV	CDP10mg/kgIV	CER100mgSC	CER10mg/kgIV	CER200mgSC
ABA10mg/kgIV	ABA10mg/kgIV	2.03 (0.56, 6.54)	1.25 (0.39, 3.86)	**4.68 (1.39, 18.19)**	**9.79 (2.76, 39.91)**	**4.57 (1.53, 15.36)**	2.32 (0.72, 8.45)	**3.93 (1.29, 13.23)**	1.17 (0.34, 4.24)	1.36 (0.35, 6.14)	1.72 (0.44, 7.30)	0.91 (0.19, 4.14)	1.26 (0.33, 4.63)	0.90 (0.24, 3.59)	**4.65 (1.19, 23.08)**	3.72 (0.96, 17.49)	0.71 (0.05, 6.86)	4.48 (0.74, 40.43)	2.57 (0.26, 25.50)	1.72 (0.58, 5.48)	1.94 (0.60, 6.50)	1.18 (0.22, 5.48)	1.38 (0.41, 4.88)
ABA30mg/kgIV	0.49 (0.15, 1.79)	ABA30mg/kgIV	0.63 (0.18, 2.18)	2.40 (0.70, 8.86)	**5.06 (1.38, 17.56)**	2.35 (0.75, 7.56)	1.22 (0.35, 4.12)	1.99 (0.64, 6.71)	0.59 (0.17, 2.05)	0.68 (0.16, 3.24)	0.86 (0.20, 3.81)	0.46 (0.09, 2.24)	0.64 (0.17, 2.43)	0.46 (0.11, 1.83)	2.45 (0.58, 9.85)	1.89 (0.50, 7.88)	0.35 (0.03, 3.42)	2.23 (0.38, 18.13)	1.32 (0.13, 12.55)	0.87 (0.29, 2.67)	0.98 (0.30, 3.25)	0.60 (0.12, 2.63)	0.68 (0.20, 2.34)
ABA3mg/kgIV	0.80 (0.26, 2.58)	1.60 (0.46, 5.46)	ABA3mg/kgIV	**3.81 (1.12, 13.07)**	**7.91 (2.43, 29.67)**	**3.60 (1.28, 11.18)**	1.92 (0.52, 6.84)	3.15 (0.98, 10.59)	0.93 (0.28, 3.35)	1.10 (0.30, 4.79)	1.37 (0.37, 6.17)	0.72 (0.16, 3.38)	0.99 (0.30, 3.60)	0.72 (0.17, 2.75)	3.87 (0.97, 17.29)	3.04 (0.81, 13.32)	0.54 (0.04, 5.71)	3.49 (0.60, 35.16)	2.10 (0.24, 21.73)	1.37 (0.49, 4.35)	1.54 (0.54, 4.98)	0.94 (0.18, 4.17)	1.09 (0.35, 3.62)
ADA160mg80mg40mgSC	**0.21 (0.05, 0.72)**	0.42 (0.11, 1.43)	**0.26 (0.08, 0.89)**	ADA160mg80mg40mgSC	2.09 (0.75, 6.42)	0.97 (0.41, 2.16)	0.51 (0.18, 1.32)	0.83 (0.35, 2.04)	**0.24 (0.09, 0.65)**	**0.29 (0.09, 0.96)**	0.36 (0.11, 1.22)	**0.20 (0.05, 0.67)**	**0.26 (0.09, 0.75)**	**0.19 (0.06, 0.58)**	1.00 (0.33, 3.28)	0.79 (0.27, 2.78)	0.14 (0.01, 1.26)	0.89 (0.20, 7.56)	0.54 (0.07, 4.81)	**0.37 (0.16, 0.80)**	**0.40 (0.17, 0.94)**	**0.25 (0.06, 0.86)**	**0.29 (0.12, 0.69)**
ADA160mg80mg60mgSC	**0.10 (0.03, 0.36)**	**0.20 (0.06, 0.72)**	**0.13 (0.03, 0.41)**	0.48 (0.16, 1.33)	ADA160mg80mg60mgSC	0.47 (0.17, 1.08)	**0.24 (0.08, 0.66)**	0.41 (0.14, 1.03)	**0.12 (0.04, 0.32)**	**0.15 (0.04, 0.47)**	**0.17 (0.05, 0.56)**	**0.09 (0.02, 0.34)**	**0.13 (0.04, 0.36)**	**0.09 (0.03, 0.29)**	0.49 (0.14, 1.70)	0.37 (0.12, 1.33)	**0.07 (0.01, 0.66)**	0.44 (0.08, 4.20)	0.26 (0.03, 2.28)	**0.18 (0.07, 0.40)**	**0.20 (0.07, 0.47)**	**0.12 (0.03, 0.41)**	**0.14 (0.05, 0.35)**
ADA160mg80mgSC	**0.22 (0.07, 0.65)**	0.43 (0.13, 1.33)	**0.28 (0.09, 0.78)**	1.03 (0.46, 2.42)	2.13 (0.92, 5.73)	ADA160mg80mgSC	**0.53 (0.26, 0.94)**	0.87 (0.54, 1.32)	**0.25 (0.11, 0.56)**	**0.31 (0.10, 0.88)**	0.38 (0.13, 1.09)	**0.20 (0.06, 0.62)**	**0.27 (0.11, 0.64)**	**0.20 (0.07, 0.48)**	1.03 (0.38, 3.09)	0.82 (0.32, 2.39)	0.15 (0.01, 1.18)	0.93 (0.22, 6.85)	0.55 (0.09, 4.49)	**0.38 (0.21, 0.68)**	**0.43 (0.22, 0.78)**	**0.26 (0.08, 0.75)**	**0.30 (0.14, 0.60)**
ADA40mg20mgSC	0.43 (0.12, 1.38)	0.82 (0.24, 2.89)	0.52 (0.15, 1.92)	1.98 (0.76, 5.48)	**4.13 (1.51, 12.11)**	**1.88 (1.07, 3.85)**	ADA40mg20mgSC	1.64 (0.86, 3.33)	0.49 (0.18, 1.22)	0.58 (0.18, 1.90)	0.71 (0.23, 2.29)	0.38 (0.10, 1.37)	0.51 (0.18, 1.44)	0.37 (0.12, 1.05)	1.99 (0.64, 6.49)	1.56 (0.53, 5.15)	0.30 (0.02, 2.42)	1.80 (0.36, 14.89)	1.06 (0.16, 9.11)	0.71 (0.33, 1.63)	0.80 (0.34, 1.97)	0.50 (0.13, 1.60)	0.57 (0.24, 1.35)
ADA80mg40mgSC	**0.25 (0.08, 0.78)**	0.50 (0.15, 1.57)	0.32 (0.09, 1.02)	1.20 (0.49, 2.87)	2.43 (0.97, 7.22)	1.15 (0.76, 1.84)	0.61 (0.30, 1.16)	ADA80mg40mgSC	**0.30 (0.12, 0.70)**	0.35 (0.12, 1.08)	0.43 (0.15, 1.34)	**0.23 (0.07, 0.72)**	**0.32 (0.13, 0.77)**	**0.23 (0.08, 0.62)**	1.18 (0.42, 4.00)	0.92 (0.36, 3.06)	0.17 (0.01, 1.43)	1.10 (0.24, 8.20)	0.64 (0.09, 5.21)	**0.43 (0.22, 0.88)**	0.49 (0.23, 1.03)	**0.29 (0.09, 0.94)**	**0.35 (0.15, 0.75)**
AMI0.4mgPO	0.86 (0.24, 2.98)	1.68 (0.49, 5.97)	1.08 (0.30, 3.55)	**4.13 (1.55, 10.79)**	**8.44 (3.13, 25.14)**	**3.95 (1.80, 8.87)**	2.05 (0.82, 5.43)	**3.36 (1.42, 8.36)**	AMI0.4mgPO	1.21 (0.38, 3.86)	1.47 (0.47, 4.76)	0.78 (0.20, 2.76)	1.09 (0.38, 2.78)	0.78 (0.26, 2.14)	**4.01 (1.34, 13.85)**	**3.19 (1.16, 11.01)**	0.58 (0.05, 5.40)	3.69 (0.80, 31.79)	2.25 (0.31, 19.52)	1.48 (0.69, 3.23)	1.67 (0.72, 3.77)	1.01 (0.26, 3.45)	1.16 (0.50, 2.78)
AND150mgSC1.1	0.74 (0.16, 2.83)	1.46 (0.31, 6.20)	0.91 (0.21, 3.35)	**3.44 (1.04, 11.57)**	**6.84 (2.14, 26.07)**	**3.28 (1.14, 9.65)**	1.73 (0.53, 5.55)	2.82 (0.92, 8.67)	0.83 (0.26, 2.64)	AND150mgSC1.1	1.24 (0.53, 2.80)	0.66 (0.23, 1.74)	0.89 (0.27, 3.20)	0.65 (0.18, 2.13)	3.37 (0.88, 13.88)	2.70 (0.72, 10.68)	0.47 (0.04, 3.96)	3.18 (0.60, 25.12)	1.92 (0.18, 15.66)	1.24 (0.45, 3.46)	1.40 (0.46, 3.93)	0.86 (0.18, 3.49)	0.99 (0.32, 2.81)
AND150mgSC2.2	0.58 (0.14, 2.29)	1.16 (0.26, 4.90)	0.73 (0.16, 2.71)	2.79 (0.82, 8.76)	**5.73 (1.80, 20.75)**	2.65 (0.92, 7.42)	1.40 (0.44, 4.41)	2.30 (0.75, 6.71)	0.68 (0.21, 2.14)	0.80 (0.36, 1.88)	AND150mgSC2.2	0.53 (0.19, 1.37)	0.72 (0.21, 2.35)	0.53 (0.15, 1.81)	2.72 (0.75, 11.51)	2.15 (0.64, 8.50)	0.39 (0.03, 3.54)	2.57 (0.48, 20.78)	1.52 (0.18, 12.37)	1.01 (0.36, 2.63)	1.14 (0.39, 3.02)	0.69 (0.15, 2.63)	0.79 (0.27, 2.22)
AND300mgSC	1.10 (0.24, 5.27)	2.20 (0.45, 10.81)	1.39 (0.30, 6.34)	**5.09 (1.50, 19.82)**	**10.86 (2.96, 46.59)**	**5.03 (1.61, 16.98)**	2.61 (0.73, 9.57)	**4.33 (1.38, 15.10)**	1.29 (0.36, 4.93)	1.52 (0.57, 4.35)	1.89 (0.73, 5.33)	AND300mgSC	1.39 (0.37, 5.06)	1.00 (0.25, 3.83)	**5.22 (1.22, 26.22)**	**4.15 (1.04, 20.07)**	0.73 (0.06, 7.73)	5.03 (0.83, 42.42)	2.88 (0.31, 27.19)	1.93 (0.60, 6.12)	2.11 (0.65, 7.10)	1.35 (0.26, 5.71)	1.51 (0.48, 5.19)
API100mgPO	0.80 (0.22, 3.07)	1.57 (0.41, 5.90)	1.01 (0.28, 3.38)	**3.82 (1.33, 11.06)**	**7.73 (2.77, 23.40)**	**3.65 (1.55, 8.94)**	1.94 (0.70, 5.43)	**3.17 (1.30, 7.99)**	0.92 (0.36, 2.62)	1.12 (0.31, 3.64)	1.39 (0.43, 4.82)	0.72 (0.20, 2.69)	API100mgPO	0.72 (0.29, 1.80)	**3.79 (1.20, 14.18)**	2.97 (0.94, 11.33)	0.54 (0.05, 5.56)	3.39 (0.74, 29.90)	2.05 (0.28, 18.29)	1.38 (0.60, 3.43)	1.54 (0.63, 3.83)	0.97 (0.26, 3.10)	1.11 (0.43, 2.79)
API50mgPO	1.11 (0.28, 4.21)	2.17 (0.55, 8.76)	1.38 (0.36, 5.78)	**5.32 (1.72, 16.47)**	**11.21 (3.43, 35.07)**	**5.06 (2.07, 13.40)**	2.70 (0.95, 8.08)	**4.42 (1.62, 12.42)**	1.29 (0.47, 3.85)	1.55 (0.47, 5.41)	1.90 (0.55, 6.80)	1.00 (0.26, 3.94)	1.39 (0.56, 3.48)	API50mgPO	**5.37 (1.56, 19.98)**	**4.21 (1.31, 14.84)**	0.78 (0.06, 6.82)	**4.89 (1.01, 42.49)**	2.88 (0.46, 24.81)	1.93 (0.76, 4.94)	2.14 (0.79, 6.42)	1.32 (0.32, 4.92)	1.53 (0.56, 4.32)
BRI400mgIV	**0.21 (0.04, 0.84)**	0.41 (0.10, 1.72)	0.26 (0.06, 1.04)	1.00 (0.31, 3.04)	2.05 (0.59, 7.22)	0.97 (0.32, 2.66)	0.50 (0.15, 1.57)	0.84 (0.25, 2.37)	**0.25 (0.07, 0.74)**	0.30 (0.07, 1.14)	0.37 (0.09, 1.33)	**0.19 (0.04, 0.82)**	**0.26 (0.07, 0.83)**	**0.19 (0.05, 0.64)**	BRI400mgIV	0.77 (0.46, 1.42)	0.14 (0.01, 1.31)	0.92 (0.16, 8.33)	0.53 (0.06, 4.98)	0.37 (0.12, 1.00)	0.41 (0.13, 1.15)	0.24 (0.05, 1.02)	**0.29 (0.09, 0.86)**
BRI700mgIV	0.27 (0.06, 1.04)	0.53 (0.13, 2.00)	0.33 (0.08, 1.24)	1.27 (0.36, 3.73)	2.67 (0.75, 8.37)	1.22 (0.42, 3.12)	0.64 (0.19, 1.90)	1.09 (0.33, 2.79)	**0.31 (0.09, 0.86)**	0.37 (0.09, 1.39)	0.46 (0.12, 1.57)	**0.24 (0.05, 0.96)**	0.34 (0.09, 1.06)	**0.24 (0.07, 0.76)**	1.29 (0.70, 2.19)	BRI700mgIV	0.18 (0.01, 1.77)	1.17 (0.19, 9.85)	0.69 (0.08, 6.02)	0.46 (0.15, 1.16)	0.52 (0.16, 1.38)	0.32 (0.07, 1.20)	0.37 (0.11, 1.03)
BRO210mgIV	1.40 (0.15, 18.80)	2.88 (0.29, 39.09)	1.84 (0.18, 22.60)	7.00 (0.79, 81.98)	**14.44 (1.53, 192.58)**	6.72 (0.85, 79.72)	3.28 (0.41, 44.14)	5.93 (0.70, 71.71)	1.73 (0.19, 18.66)	2.13 (0.25, 26.37)	2.58 (0.28, 33.80)	1.36 (0.13, 17.99)	1.86 (0.18, 21.77)	1.28 (0.15, 17.13)	7.20 (0.76, 87.07)	5.68 (0.56, 67.43)	BRO210mgIV	**7.26 (1.12, 62.48)**	3.99 (0.46, 40.68)	2.56 (0.33, 28.58)	2.88 (0.31, 31.75)	1.72 (0.15, 22.47)	2.09 (0.24, 21.58)
BRO350mgIV	0.22 (0.02, 1.35)	0.45 (0.06, 2.60)	0.29 (0.03, 1.67)	1.12 (0.13, 4.88)	2.27 (0.24, 11.86)	1.08 (0.15, 4.60)	0.55 (0.07, 2.76)	0.91 (0.12, 4.17)	0.27 (0.03, 1.26)	0.31 (0.04, 1.66)	0.39 (0.05, 2.10)	0.20 (0.02, 1.20)	0.29 (0.03, 1.35)	**0.20 (0.02, 0.99)**	1.09 (0.12, 6.43)	0.85 (0.10, 5.23)	**0.14 (0.02, 0.89)**	BRO350mgIV	0.59 (0.12, 2.18)	0.40 (0.05, 1.66)	0.45 (0.05, 1.84)	0.27 (0.03, 1.44)	0.31 (0.04, 1.35)
BRO700mgIV	0.39 (0.04, 3.89)	0.76 (0.08, 7.48)	0.48 (0.05, 4.16)	1.85 (0.21, 13.62)	3.89 (0.44, 35.12)	1.83 (0.22, 11.71)	0.95 (0.11, 6.12)	1.56 (0.19, 10.77)	0.44 (0.05, 3.18)	0.52 (0.06, 5.51)	0.66 (0.08, 5.62)	0.35 (0.04, 3.18)	0.49 (0.05, 3.57)	0.35 (0.04, 2.17)	1.88 (0.20, 15.39)	1.45 (0.17, 12.12)	0.25 (0.02, 2.19)	1.70 (0.46, 8.52)	BRO700mgIV	0.67 (0.09, 4.63)	0.77 (0.09, 5.32)	0.46 (0.04, 3.62)	0.54 (0.06, 3.77)
CDP10mg/kgIV	0.58 (0.18, 1.73)	1.15 (0.37, 3.44)	0.73 (0.23, 2.05)	**2.71 (1.25, 6.18)**	**5.59 (2.48, 15.21)**	**2.67 (1.48, 4.79)**	1.41 (0.61, 3.02)	**2.30 (1.14, 4.46)**	0.68 (0.31, 1.46)	0.81 (0.29, 2.22)	0.99 (0.38, 2.76)	0.52 (0.16, 1.66)	0.72 (0.29, 1.67)	0.52 (0.20, 1.32)	2.73 (1.00, 8.30)	2.15 (0.86, 6.46)	0.39 (0.03, 3.01)	2.49 (0.60, 19.06)	1.49 (0.22, 11.10)	CDP10mg/kgIV	1.12 (0.58, 2.07)	0.69 (0.20, 1.98)	0.80 (0.39, 1.58)
CER100mgSC	0.52 (0.15, 1.67)	1.02 (0.31, 3.35)	0.65 (0.20, 1.87)	**2.47 (1.06, 5.73)**	**5.09 (2.13, 14.18)**	**2.33 (1.28, 4.65)**	1.25 (0.51, 2.93)	2.04 (0.97, 4.32)	0.60 (0.27, 1.38)	0.71 (0.25, 2.17)	0.87 (0.33, 2.59)	0.47 (0.14, 1.54)	0.65 (0.26, 1.58)	0.47 (0.16, 1.27)	2.45 (0.87, 7.97)	1.91 (0.73, 6.33)	0.35 (0.03, 3.26)	2.25 (0.54, 19.40)	1.31 (0.19, 11.46)	0.89 (0.48, 1.73)	CER100mgSC	0.61 (0.17, 2.00)	0.71 (0.37, 1.35)
CER10mg/kgIV	0.85 (0.18, 4.58)	1.66 (0.38, 8.50)	1.07 (0.24, 5.47)	**4.00 (1.17, 15.89)**	**8.26 (2.44, 39.11)**	**3.89 (1.33, 13.11)**	2.00 (0.62, 7.75)	**3.40 (1.06, 11.68)**	0.99 (0.29, 3.79)	1.17 (0.29, 5.62)	1.45 (0.38, 6.80)	0.74 (0.18, 3.78)	1.03 (0.32, 3.88)	0.76 (0.20, 3.10)	4.17 (0.98, 18.34)	3.17 (0.83, 13.76)	0.58 (0.04, 6.68)	3.70 (0.69, 38.55)	2.15 (0.28, 23.42)	1.45 (0.50, 5.06)	1.64 (0.50, 5.75)	CER10mg/kgIV	1.14 (0.37, 4.38)
CER200mgSC	0.72 (0.20, 2.42)	1.46 (0.43, 4.97)	0.92 (0.28, 2.84)	**3.48 (1.45, 8.38)**	**7.11 (2.83, 21.28)**	**3.30 (1.68, 6.95)**	1.75 (0.74, 4.24)	**2.84 (1.34, 6.53)**	0.86 (0.36, 2.01)	1.01 (0.36, 3.09)	1.26 (0.45, 3.67)	0.66 (0.19, 2.09)	0.90 (0.36, 2.33)	0.65 (0.23, 1.78)	**3.46 (1.16, 11.46)**	2.70 (0.97, 9.01)	0.48 (0.05, 4.18)	3.27 (0.74, 27.83)	1.86 (0.27, 15.76)	1.25 (0.63, 2.54)	1.41 (0.74, 2.68)	0.87 (0.23, 2.71)	CER200mgSC

	ABA10mg/kgIV	ABA30mg/kgIV	ABA3mg/kgIV	ADA160mg80mg40mgSC	ADA160mg80mg60mgSC	ADA160mg80mgSC	ADA40mg20mgSC	ADA80mg40mgSC	AMI0.4mgPO	AND150mgSC1.1	AND150mgSC2.2	AND300mgSC	API100mgPO	API50mgPO	BRI400mgIV	BRI700mgIV	BRO210mgIV	BRO350mgIV	BRO700mgIV	CDP10mg/kgIV	CER100mgSC	CER10mg/kgIV	CER200mgSC
CER20mg/kgIV	1.55 (0.32, 10.11)	3.06 (0.64, 20.24)	1.92 (0.43, 11.68)	**7.46 (1.96, 36.28)**	**14.98 (4.01, 106.75)**	**7.08 (2.19, 35.62)**	3.69 (1.00, 20.29)	**6.18 (1.78, 32.04)**	1.76 (0.51, 10.06)	2.22 (0.47, 11.17)	2.71 (0.64, 13.94)	1.44 (0.30, 8.64)	1.93 (0.48, 11.17)	1.40 (0.35, 8.30)	**7.48 (1.73, 46.49)**	**5.74 (1.47, 36.80)**	1.07 (0.07, 11.67)	**6.94 (1.23, 77.86)**	4.03 (0.44, 51.76)	2.66 (0.85, 13.35)	2.93 (0.88, 15.73)	1.84 (0.43, 10.42)	2.09 (0.57, 11.10)
CER400mgSC	0.48 (0.15, 1.35)	0.94 (0.31, 2.77)	0.60 (0.20, 1.62)	**2.27 (1.12, 4.79)**	**4.63 (2.19, 11.73)**	**2.19 (1.37, 3.60)**	1.16 (0.55, 2.32)	**1.89 (1.05, 3.42)**	0.56 (0.28, 1.11)	0.66 (0.26, 1.79)	0.82 (0.32, 2.16)	0.43 (0.14, 1.29)	0.60 (0.26, 1.29)	**0.44 (0.17, 0.99)**	2.27 (0.90, 6.60)	1.76 (0.76, 5.18)	0.32 (0.03, 2.53)	2.05 (0.52, 15.66)	1.21 (0.18, 9.24)	0.83 (0.53, 1.29)	0.92 (0.56, 1.48)	0.58 (0.17, 1.60)	0.66 (0.37, 1.13)
CER5mg/kgIV	0.59 (0.15, 2.17)	1.18 (0.30, 4.41)	0.73 (0.19, 2.66)	2.78 (0.92, 8.31)	**5.93 (1.74, 18.92)**	2.71 (1.00, 6.77)	1.42 (0.48, 4.08)	2.36 (0.77, 6.24)	0.70 (0.22, 1.97)	0.85 (0.22, 2.75)	1.05 (0.28, 3.31)	0.54 (0.13, 2.01)	0.75 (0.23, 2.15)	0.53 (0.16, 1.66)	2.92 (0.77, 9.75)	2.30 (0.65, 7.81)	0.41 (0.03, 3.88)	2.52 (0.55, 24.15)	1.48 (0.21, 13.29)	1.03 (0.36, 2.51)	1.16 (0.40, 2.86)	0.71 (0.20, 1.98)	0.81 (0.28, 2.13)
ELD10mg/kgIV	0.54 (0.13, 2.13)	1.06 (0.27, 4.33)	0.68 (0.16, 2.59)	2.52 (0.80, 7.72)	**5.18 (1.62, 18.90)**	2.44 (0.86, 6.50)	1.28 (0.40, 3.81)	2.12 (0.73, 5.78)	0.63 (0.20, 1.81)	0.73 (0.19, 2.74)	0.92 (0.26, 3.44)	0.48 (0.12, 1.95)	0.67 (0.20, 2.02)	0.48 (0.14, 1.62)	2.53 (0.70, 10.35)	1.98 (0.57, 7.70)	0.35 (0.03, 2.97)	2.38 (0.45, 19.32)	1.36 (0.16, 11.52)	0.92 (0.34, 2.42)	1.03 (0.36, 2.95)	0.62 (0.14, 2.36)	0.74 (0.25, 2.09)
ELD20mg/kgIV	0.40 (0.10, 1.51)	0.79 (0.20, 3.05)	0.51 (0.12, 1.82)	1.91 (0.60, 5.58)	**3.92 (1.29, 13.20)**	1.85 (0.70, 4.40)	0.95 (0.34, 2.59)	1.59 (0.59, 3.96)	0.47 (0.16, 1.30)	0.57 (0.15, 1.93)	0.69 (0.20, 2.38)	0.36 (0.09, 1.37)	0.51 (0.16, 1.37)	0.36 (0.11, 1.08)	1.94 (0.52, 7.11)	1.50 (0.45, 5.62)	0.27 (0.02, 2.24)	1.79 (0.35, 15.36)	1.03 (0.15, 9.16)	0.70 (0.26, 1.63)	0.78 (0.29, 1.86)	0.48 (0.11, 1.73)	0.56 (0.20, 1.41)
ETA25mgSC	1.15 (0.21, 6.85)	2.26 (0.45, 15.50)	1.44 (0.26, 9.78)	**5.57 (1.27, 28.81)**	**11.26 (2.74, 63.59)**	**5.24 (1.40, 24.06)**	2.71 (0.72, 14.36)	**4.50 (1.19, 22.94)**	1.37 (0.31, 6.61)	1.59 (0.32, 10.49)	2.03 (0.43, 11.25)	1.04 (0.19, 6.78)	1.48 (0.32, 7.59)	1.07 (0.22, 5.35)	**5.54 (1.20, 33.10)**	4.33 (1.00, 25.40)	0.83 (0.05, 10.54)	5.22 (0.79, 58.20)	3.00 (0.41, 36.87)	1.98 (0.52, 9.51)	2.20 (0.57, 11.68)	1.37 (0.25, 7.39)	1.55 (0.40, 8.00)
ETR105mgSC	0.62 (0.19, 1.84)	1.21 (0.40, 3.75)	0.79 (0.24, 2.20)	**2.93 (1.34, 6.56)**	**6.01 (2.67, 16.10)**	**2.83 (1.62, 5.07)**	1.48 (0.69, 3.21)	**2.43 (1.24, 4.81)**	0.72 (0.33, 1.54)	0.86 (0.32, 2.43)	1.07 (0.40, 2.94)	0.56 (0.17, 1.75)	0.77 (0.32, 1.78)	0.56 (0.21, 1.33)	**2.98 (1.09, 8.73)**	2.28 (0.96, 6.94)	0.42 (0.04, 3.41)	2.63 (0.65, 20.83)	1.56 (0.22, 12.10)	1.06 (0.62, 1.86)	1.19 (0.64, 2.17)	0.73 (0.21, 2.12)	0.85 (0.42, 1.62)
ETR210mgSC	0.54 (0.17, 1.55)	1.05 (0.34, 3.22)	0.68 (0.22, 1.87)	**2.55 (1.18, 5.73)**	**5.28 (2.34, 14.06)**	**2.46 (1.42, 4.29)**	1.30 (0.62, 2.76)	**2.13 (1.12, 4.06)**	0.63 (0.29, 1.31)	0.75 (0.28, 2.15)	0.91 (0.35, 2.68)	0.48 (0.15, 1.52)	0.67 (0.27, 1.57)	0.49 (0.18, 1.17)	2.57 (0.93, 7.72)	1.97 (0.82, 5.96)	0.36 (0.03, 3.00)	2.33 (0.56, 17.02)	1.38 (0.19, 10.76)	0.92 (0.55, 1.62)	1.04 (0.55, 1.89)	0.64 (0.19, 1.84)	0.74 (0.37, 1.44)
FIL100mgPO	**0.33 (0.10, 0.95)**	0.64 (0.21, 1.93)	0.42 (0.13, 1.14)	1.54 (0.74, 3.39)	**3.13 (1.43, 8.22)**	1.48 (0.88, 2.53)	0.79 (0.36, 1.64)	1.29 (0.69, 2.41)	**0.38 (0.18, 0.80)**	0.45 (0.16, 1.24)	0.56 (0.22, 1.56)	**0.29 (0.09, 0.91)**	**0.41 (0.17, 0.94)**	**0.30 (0.12, 0.71)**	1.53 (0.58, 4.46)	1.20 (0.51, 3.57)	0.22 (0.02, 1.74)	1.39 (0.36, 10.71)	0.83 (0.12, 6.28)	**0.56 (0.34, 0.92)**	0.63 (0.35, 1.14)	0.39 (0.11, 1.09)	**0.45 (0.23, 0.83)**
FIL200mgPO	0.39 (0.12, 1.11)	0.77 (0.26, 2.36)	0.50 (0.16, 1.35)	1.85 (0.89, 3.98)	**3.78 (1.74, 9.61)**	**1.78 (1.08, 3.01)**	0.96 (0.43, 1.95)	1.55 (0.83, 2.82)	**0.46 (0.22, 0.93)**	0.54 (0.21, 1.50)	0.67 (0.26, 1.83)	0.35 (0.11, 1.08)	0.49 (0.20, 1.10)	**0.36 (0.13, 0.84)**	1.86 (0.71, 5.47)	1.43 (0.62, 4.27)	0.27 (0.02, 2.12)	1.66 (0.44, 13.02)	1.00 (0.15, 7.51)	0.68 (0.41, 1.10)	0.76 (0.42, 1.34)	0.46 (0.13, 1.30)	**0.54 (0.28, 0.99)**
FON0.1mg/kgIV	1.52 (0.17, 30.09)	3.00 (0.32, 55.63)	1.90 (0.21, 33.92)	6.97 (0.98, 113.69)	**14.60 (1.86, 258.43)**	**7.01 (1.03, 113.65)**	3.65 (0.51, 57.33)	5.99 (0.88, 97.39)	1.77 (0.21, 27.70)	2.05 (0.22, 36.42)	2.61 (0.31, 44.00)	1.35 (0.14, 26.47)	1.85 (0.27, 28.01)	1.37 (0.16, 21.38)	7.04 (0.94, 126.37)	5.61 (0.70, 96.34)	0.98 (0.03, 46.71)	7.25 (0.51, 229.82)	3.93 (0.26, 140.65)	2.62 (0.38, 37.61)	2.80 (0.43, 43.44)	1.73 (0.20, 27.95)	2.05 (0.26, 32.30)
FON1mg/kg0.1mgIV/SC	0.54 (0.13, 2.07)	1.07 (0.28, 4.39)	0.67 (0.18, 2.55)	2.56 (0.89, 7.72)	**5.16 (1.72, 19.41)**	2.42 (0.98, 6.85)	1.30 (0.44, 3.89)	2.10 (0.83, 5.89)	0.62 (0.22, 1.88)	0.73 (0.22, 2.74)	0.92 (0.27, 3.31)	0.47 (0.12, 1.90)	0.67 (0.21, 1.93)	0.48 (0.14, 1.50)	2.52 (0.71, 9.39)	1.99 (0.61, 7.52)	0.37 (0.03, 3.35)	2.37 (0.45, 18.00)	1.42 (0.17, 12.08)	0.92 (0.37, 2.36)	1.04 (0.40, 2.86)	0.63 (0.16, 2.41)	0.73 (0.26, 1.96)
FON1mg/kg1mgIV/SC	0.42 (0.10, 1.48)	0.83 (0.22, 3.15)	0.53 (0.13, 1.84)	2.02 (0.66, 5.46)	**4.17 (1.35, 12.88)**	1.93 (0.78, 4.58)	1.01 (0.35, 2.73)	1.66 (0.64, 4.23)	0.50 (0.17, 1.31)	0.59 (0.17, 2.03)	0.73 (0.21, 2.46)	0.38 (0.09, 1.35)	0.53 (0.17, 1.55)	0.38 (0.12, 1.12)	2.05 (0.58, 6.85)	1.57 (0.51, 5.33)	0.29 (0.02, 2.64)	1.87 (0.36, 15.07)	1.12 (0.13, 9.31)	0.73 (0.30, 1.70)	0.82 (0.30, 1.97)	0.50 (0.13, 1.78)	0.58 (0.20, 1.49)
FON1mg/kgIV	0.70 (0.12, 4.39)	1.41 (0.26, 8.39)	0.92 (0.14, 4.95)	3.40 (0.74, 16.76)	**7.08 (1.50, 35.13)**	3.27 (0.76, 14.69)	1.72 (0.38, 8.57)	2.82 (0.67, 12.80)	0.82 (0.17, 4.32)	1.01 (0.19, 5.31)	1.23 (0.24, 6.79)	0.65 (0.11, 4.03)	0.87 (0.19, 4.30)	0.64 (0.13, 3.17)	3.23 (0.68, 21.31)	2.54 (0.54, 16.46)	0.45 (0.03, 7.56)	3.33 (0.41, 34.60)	1.91 (0.18, 21.71)	1.23 (0.30, 5.40)	1.38 (0.34, 6.20)	0.84 (0.14, 5.35)	0.98 (0.22, 4.50)
FON4mg/kg0.1mgIV/SC	0.35 (0.09, 1.19)	0.68 (0.18, 2.49)	0.43 (0.11, 1.46)	1.61 (0.59, 4.37)	**3.32 (1.18, 10.61)**	1.54 (0.68, 3.71)	0.83 (0.30, 2.19)	1.33 (0.55, 3.34)	0.40 (0.15, 1.05)	0.46 (0.15, 1.58)	0.58 (0.19, 1.95)	0.31 (0.08, 1.09)	0.42 (0.15, 1.20)	**0.30 (0.10, 0.90)**	1.62 (0.50, 5.48)	1.27 (0.42, 4.37)	0.23 (0.02, 1.93)	1.48 (0.30, 11.76)	0.89 (0.11, 7.96)	0.59 (0.25, 1.34)	0.66 (0.26, 1.61)	0.40 (0.10, 1.43)	0.47 (0.18, 1.19)
FON4mg/kg1mgIV/SC	0.43 (0.10, 1.45)	0.83 (0.22, 2.91)	0.52 (0.13, 1.87)	1.95 (0.69, 5.48)	**4.10 (1.37, 12.95)**	1.91 (0.77, 4.59)	0.99 (0.35, 2.70)	1.65 (0.65, 4.19)	0.49 (0.17, 1.35)	0.57 (0.17, 1.93)	0.72 (0.22, 2.31)	0.37 (0.09, 1.38)	0.51 (0.17, 1.53)	0.37 (0.11, 1.18)	2.04 (0.58, 6.83)	1.56 (0.50, 5.32)	0.28 (0.02, 2.43)	1.85 (0.36, 15.16)	1.07 (0.13, 8.99)	0.73 (0.29, 1.69)	0.81 (0.30, 2.00)	0.49 (0.12, 1.88)	0.58 (0.20, 1.48)
FON4mg/kgIV	0.35 (0.07, 1.68)	0.70 (0.13, 3.21)	0.46 (0.08, 1.85)	1.68 (0.39, 7.46)	3.50 (0.81, 14.03)	1.69 (0.41, 5.67)	0.88 (0.19, 3.05)	1.43 (0.35, 5.17)	0.42 (0.09, 1.60)	0.49 (0.10, 2.26)	0.60 (0.13, 2.69)	0.32 (0.06, 1.48)	0.45 (0.10, 1.81)	0.32 (0.07, 1.33)	1.71 (0.37, 7.73)	1.35 (0.28, 6.23)	0.23 (0.02, 2.98)	1.64 (0.24, 14.23)	0.97 (0.10, 8.74)	0.63 (0.15, 2.11)	0.69 (0.17, 2.42)	0.42 (0.08, 1.76)	0.50 (0.11, 1.72)
GUS1200mgIV	**0.21 (0.05, 0.67)**	0.40 (0.11, 1.35)	**0.25 (0.07, 0.80)**	0.95 (0.58, 1.59)	1.99 (0.69, 5.95)	0.92 (0.41, 2.04)	0.49 (0.17, 1.20)	0.80 (0.34, 1.85)	**0.23 (0.09, 0.61)**	**0.28 (0.09, 0.87)**	0.34 (0.11, 1.14)	**0.18 (0.05, 0.63)**	**0.25 (0.09, 0.70)**	**0.18 (0.06, 0.53)**	0.96 (0.32, 3.16)	0.74 (0.27, 2.59)	0.14 (0.01, 1.20)	0.86 (0.21, 7.23)	0.52 (0.07, 4.47)	**0.36 (0.16, 0.73)**	**0.39 (0.17, 0.86)**	**0.24 (0.06, 0.83)**	**0.28 (0.12, 0.66)**
GUS200mgIV	**0.16 (0.04, 0.54)**	0.32 (0.09, 1.04)	**0.20 (0.06, 0.63)**	0.76 (0.48, 1.19)	1.58 (0.59, 4.66)	0.73 (0.34, 1.59)	**0.39 (0.15, 0.96)**	0.64 (0.28, 1.48)	**0.19 (0.07, 0.48)**	**0.22 (0.07, 0.69)**	**0.27 (0.09, 0.89)**	**0.15 (0.04, 0.50)**	**0.20 (0.07, 0.55)**	**0.15 (0.05, 0.42)**	0.76 (0.26, 2.53)	0.58 (0.21, 2.05)	**0.11 (0.01, 0.93)**	0.69 (0.16, 5.65)	0.41 (0.06, 3.52)	**0.28 (0.13, 0.57)**	**0.32 (0.14, 0.66)**	**0.19 (0.05, 0.66)**	**0.22 (0.10, 0.50)**
GUS600mgIV	**0.17 (0.04, 0.55)**	0.33 (0.09, 1.10)	**0.21 (0.06, 0.67)**	0.79 (0.50, 1.26)	1.63 (0.61, 4.89)	0.76 (0.35, 1.63)	**0.41 (0.15, 0.98)**	0.66 (0.29, 1.52)	**0.19 (0.08, 0.48)**	**0.23 (0.07, 0.72)**	**0.29 (0.09, 0.91)**	**0.15 (0.04, 0.51)**	**0.21 (0.08, 0.57)**	**0.15 (0.05, 0.43)**	0.78 (0.26, 2.51)	0.62 (0.22, 2.08)	**0.11 (0.01, 0.97)**	0.72 (0.16, 5.72)	0.44 (0.06, 3.56)	**0.29 (0.13, 0.58)**	**0.32 (0.14, 0.70)**	**0.20 (0.05, 0.67)**	**0.23 (0.10, 0.52)**
INF10mg/kgIV	**0.07 (0.01, 0.56)**	0.14 (0.01, 1.24)	**0.08 (0.01, 0.78)**	0.34 (0.03, 2.52)	0.68 (0.07, 5.47)	0.32 (0.03, 2.18)	0.17 (0.02, 1.25)	0.28 (0.03, 2.10)	**0.09 (0.01, 0.57)**	**0.10 (0.01, 0.80)**	**0.12 (0.01, 0.98)**	**0.06 (0.00, 0.55)**	**0.09 (0.01, 0.65)**	**0.06 (0.01, 0.45)**	0.35 (0.03, 2.76)	0.27 (0.02, 2.20)	**0.04 (0.00, 0.64)**	0.32 (0.02, 3.90)	0.19 (0.01, 2.36)	**0.12 (0.01, 0.79)**	**0.14 (0.01, 0.91)**	**0.09 (0.01, 0.64)**	**0.10 (0.01, 0.65)**
INF20mg/kgIV	**0.07 (0.01, 0.56)**	0.14 (0.01, 1.30)	**0.09 (0.01, 0.79)**	0.33 (0.03, 2.82)	0.69 (0.07, 5.75)	0.34 (0.04, 2.30)	0.18 (0.02, 1.27)	0.29 (0.03, 2.03)	**0.08 (0.01, 0.61)**	**0.10 (0.01, 0.89)**	0.12 (0.01, 1.08)	**0.06 (0.01, 0.58)**	**0.09 (0.01, 0.67)**	**0.06 (0.01, 0.47)**	0.34 (0.03, 2.83)	0.27 (0.02, 2.28)	**0.04 (0.00, 0.77)**	0.33 (0.02, 4.31)	0.18 (0.01, 2.57)	**0.13 (0.01, 0.90)**	**0.14 (0.01, 0.95)**	**0.08 (0.01, 0.69)**	**0.10 (0.01, 0.67)**
INF5mg/kgIV	**0.04 (0.00, 0.26)**	**0.07 (0.01, 0.62)**	**0.04 (0.00, 0.36)**	0.17 (0.02, 1.21)	0.36 (0.04, 2.56)	**0.18 (0.02, 0.94)**	**0.09 (0.01, 0.55)**	**0.15 (0.02, 0.91)**	**0.04 (0.00, 0.26)**	**0.05 (0.00, 0.40)**	**0.06 (0.01, 0.47)**	**0.03 (0.00, 0.27)**	**0.05 (0.00, 0.30)**	**0.03 (0.00, 0.22)**	0.17 (0.02, 1.33)	0.14 (0.01, 1.08)	**0.02 (0.00, 0.31)**	0.16 (0.01, 2.01)	0.10 (0.00, 1.22)	**0.07 (0.01, 0.39)**	**0.07 (0.01, 0.42)**	**0.04 (0.00, 0.29)**	**0.05 (0.01, 0.31)**

(TABLE S12A. continued)

	ABA10mg/kgIV	ABA30mg/kgIV	ABA3mg/kgIV	ADA160mg80mg40mgSC	ADA160mg80mg60mgSC	ADA160mg80mgSC	ADA40mg20mgSC	ADA80mg40mgSC	AMI0.4mgPO	AND150mgSC1.1	AND150mgSC2.2	AND300mgSC	API100mgPO	API50mgPO	BRI400mgIV	BRI700mgIV	BRO210mgIV	BRO350mgIV	BRO700mgIV	CDP10mg/kgIV	CER100mgSC	CER10mg/kgIV	CER200mgSC
MED700mgIV	0.38 (0.08, 1.49)	0.76 (0.17, 3.09)	0.48 (0.11, 1.67)	1.79 (0.56, 5.39)	**3.69 (1.13, 12.55)**	1.75 (0.62, 4.47)	0.90 (0.30, 2.66)	1.48 (0.53, 4.28)	0.44 (0.15, 1.24)	0.51 (0.14, 2.05)	0.63 (0.19, 2.38)	0.34 (0.08, 1.39)	0.48 (0.14, 1.52)	0.34 (0.10, 1.10)	1.79 (0.52, 6.48)	1.39 (0.42, 5.23)	0.25 (0.02, 2.31)	1.69 (0.31, 12.87)	0.98 (0.12, 8.33)	0.66 (0.24, 1.63)	0.74 (0.27, 1.93)	0.44 (0.10, 1.78)	0.52 (0.18, 1.46)
MIR1000mgIV	**0.20 (0.05, 0.70)**	0.39 (0.10, 1.52)	**0.26 (0.06, 0.84)**	0.92 (0.31, 2.70)	2.00 (0.59, 6.24)	0.92 (0.32, 2.31)	0.47 (0.16, 1.43)	0.80 (0.28, 2.10)	**0.23 (0.08, 0.65)**	0.27 (0.07, 1.01)	0.33 (0.10, 1.21)	**0.18 (0.04, 0.70)**	**0.25 (0.07, 0.72)**	**0.18 (0.05, 0.55)**	0.97 (0.26, 3.55)	0.74 (0.21, 2.82)	0.13 (0.01, 1.37)	0.88 (0.16, 7.01)	0.50 (0.06, 4.21)	**0.35 (0.13, 0.84)**	**0.39 (0.14, 0.97)**	**0.24 (0.05, 0.87)**	**0.27 (0.09, 0.74)**
MIR200mgIV	0.37 (0.08, 1.65)	0.75 (0.16, 3.41)	0.47 (0.10, 2.12)	1.74 (0.48, 7.50)	3.57 (0.90, 16.17)	1.68 (0.48, 6.63)	0.86 (0.24, 3.80)	1.47 (0.42, 5.67)	0.43 (0.12, 1.73)	0.53 (0.12, 2.36)	0.64 (0.15, 3.04)	0.34 (0.06, 1.64)	0.46 (0.11, 1.92)	0.33 (0.08, 1.44)	1.80 (0.41, 8.38)	1.40 (0.33, 6.32)	0.23 (0.02, 2.94)	1.70 (0.26, 14.81)	0.96 (0.10, 8.52)	0.63 (0.19, 2.34)	0.71 (0.20, 2.82)	0.44 (0.08, 2.01)	0.51 (0.14, 1.86)
MIR600mgIV	**0.14 (0.03, 0.48)**	0.26 (0.07, 1.06)	**0.17 (0.04, 0.68)**	0.63 (0.21, 1.82)	1.33 (0.39, 4.22)	0.60 (0.22, 1.55)	**0.32 (0.10, 0.95)**	0.53 (0.18, 1.42)	**0.15 (0.05, 0.44)**	**0.19 (0.05, 0.67)**	**0.23 (0.06, 0.81)**	**0.12 (0.03, 0.49)**	**0.17 (0.04, 0.48)**	**0.12 (0.03, 0.38)**	0.63 (0.18, 2.45)	0.49 (0.14, 1.89)	**0.09 (0.01, 0.84)**	0.59 (0.11, 4.76)	0.34 (0.05, 2.61)	**0.23 (0.09, 0.57)**	**0.26 (0.09, 0.73)**	**0.16 (0.03, 0.59)**	**0.18 (0.06, 0.51)**
NAT300mgIV	0.42 (0.13, 1.20)	0.83 (0.28, 2.42)	0.54 (0.17, 1.42)	1.99 (0.98, 4.18)	**4.06 (1.95, 10.26)**	**1.93 (1.21, 3.18)**	1.02 (0.49, 2.04)	1.67 (0.92, 3.03)	**0.49 (0.24, 0.97)**	0.58 (0.22, 1.57)	0.73 (0.29, 1.87)	0.38 (0.12, 1.16)	0.52 (0.23, 1.15)	**0.38 (0.16, 0.88)**	2.00 (0.78, 5.78)	1.56 (0.68, 4.44)	0.29 (0.03, 2.27)	1.77 (0.47, 13.62)	1.08 (0.16, 8.38)	0.72 (0.47, 1.15)	0.81 (0.47, 1.36)	0.51 (0.15, 1.39)	0.59 (0.31, 1.03)
NAT3mg/kgIV	0.57 (0.16, 1.79)	1.11 (0.33, 3.55)	0.71 (0.21, 2.22)	**2.64 (1.12, 6.57)**	**5.52 (2.19, 15.68)**	**2.57 (1.27, 5.45)**	1.34 (0.57, 3.39)	2.21 (0.99, 5.06)	0.66 (0.27, 1.69)	0.79 (0.26, 2.52)	0.95 (0.33, 3.27)	0.51 (0.14, 1.82)	0.71 (0.27, 1.75)	0.52 (0.17, 1.32)	2.62 (0.89, 9.24)	2.04 (0.75, 7.07)	0.39 (0.03, 3.13)	2.46 (0.55, 18.74)	1.43 (0.19, 11.64)	0.98 (0.47, 1.98)	1.08 (0.52, 2.36)	0.66 (0.16, 2.14)	0.77 (0.34, 1.84)
NAT3mg/kgIVx2	0.37 (0.10, 1.20)	0.73 (0.22, 2.45)	0.46 (0.14, 1.38)	1.74 (0.76, 4.09)	**3.59 (1.55, 9.88)**	1.69 (0.87, 3.32)	0.88 (0.38, 2.10)	1.45 (0.68, 3.11)	0.43 (0.18, 1.02)	0.50 (0.17, 1.58)	0.62 (0.22, 1.99)	0.33 (0.10, 1.09)	0.45 (0.18, 1.12)	**0.33 (0.12, 0.84)**	1.74 (0.58, 5.62)	1.36 (0.52, 4.25)	0.25 (0.02, 2.19)	1.58 (0.38, 12.61)	0.93 (0.13, 7.91)	0.64 (0.33, 1.23)	0.71 (0.34, 1.41)	0.43 (0.12, 1.40)	0.50 (0.23, 1.10)
NAT6mg/kgIVx2	0.41 (0.11, 1.29)	0.79 (0.23, 2.66)	0.50 (0.15, 1.53)	1.89 (0.82, 4.51)	**3.91 (1.57, 11.03)**	1.83 (0.91, 3.77)	0.95 (0.40, 2.31)	1.58 (0.72, 3.50)	0.47 (0.19, 1.10)	0.56 (0.18, 1.71)	0.69 (0.23, 2.12)	0.36 (0.10, 1.19)	0.49 (0.19, 1.27)	**0.37 (0.12, 0.92)**	1.89 (0.63, 6.05)	1.49 (0.53, 4.76)	0.28 (0.02, 2.26)	1.74 (0.40, 14.40)	1.00 (0.14, 8.76)	0.70 (0.33, 1.36)	0.77 (0.37, 1.59)	0.48 (0.12, 1.53)	0.55 (0.24, 1.20)
NNC2mg/kgSC	0.36 (0.08, 1.35)	0.71 (0.18, 2.79)	0.45 (0.11, 1.66)	1.69 (0.50, 5.63)	**3.48 (1.07, 12.17)**	1.63 (0.57, 4.50)	0.86 (0.27, 2.63)	1.43 (0.48, 4.14)	0.41 (0.13, 1.24)	0.50 (0.13, 1.86)	0.61 (0.17, 2.18)	0.32 (0.08, 1.31)	0.45 (0.14, 1.42)	0.32 (0.09, 1.08)	1.72 (0.45, 6.09)	1.37 (0.37, 4.81)	0.23 (0.02, 2.58)	1.53 (0.28, 14.37)	0.90 (0.12, 8.57)	0.61 (0.22, 1.65)	0.68 (0.23, 2.01)	0.43 (0.10, 1.66)	0.48 (0.17, 1.46)
ONE10mgSC	0.48 (0.12, 1.66)	0.94 (0.24, 3.45)	0.61 (0.16, 1.91)	2.21 (0.81, 6.57)	**4.62 (1.56, 15.22)**	2.14 (0.89, 5.12)	1.12 (0.39, 3.07)	1.85 (0.74, 4.69)	0.55 (0.20, 1.43)	0.66 (0.20, 2.15)	0.80 (0.25, 2.59)	0.43 (0.11, 1.51)	0.59 (0.19, 1.66)	0.42 (0.14, 1.22)	2.24 (0.69, 7.68)	1.74 (0.59, 6.15)	0.31 (0.03, 2.89)	2.08 (0.41, 16.24)	1.21 (0.14, 9.57)	0.81 (0.34, 1.91)	0.91 (0.36, 2.23)	0.56 (0.14, 1.97)	0.65 (0.22, 1.61)
ONE25mgSC	1.04 (0.23, 3.76)	2.00 (0.48, 8.18)	1.29 (0.29, 5.34)	**4.76 (1.56, 15.92)**	**9.84 (3.04, 34.82)**	**4.62 (1.71, 12.47)**	2.40 (0.75, 7.56)	**4.00 (1.40, 11.68)**	1.17 (0.39, 3.53)	1.37 (0.37, 5.47)	1.74 (0.48, 6.29)	0.91 (0.22, 3.61)	1.24 (0.37, 4.32)	0.89 (0.26, 2.90)	**4.88 (1.29, 18.23)**	**3.78 (1.14, 14.08)**	0.71 (0.06, 6.94)	4.41 (0.83, 34.32)	2.55 (0.36, 21.90)	1.72 (0.64, 5.18)	1.92 (0.69, 5.69)	1.23 (0.26, 4.39)	1.39 (0.47, 3.90)
ONE35mgSC	0.46 (0.12, 1.66)	0.91 (0.23, 3.35)	0.60 (0.15, 1.96)	2.14 (0.77, 6.32)	**4.52 (1.51, 14.73)**	2.10 (0.82, 5.09)	1.10 (0.37, 3.06)	1.82 (0.65, 4.62)	0.54 (0.18, 1.44)	0.63 (0.19, 2.32)	0.77 (0.24, 2.70)	0.41 (0.11, 1.58)	0.57 (0.18, 1.73)	0.41 (0.13, 1.20)	2.19 (0.66, 7.47)	1.69 (0.55, 5.78)	0.32 (0.03, 2.82)	2.02 (0.40, 14.97)	1.22 (0.14, 9.69)	0.79 (0.33, 1.94)	0.89 (0.33, 2.30)	0.54 (0.13, 1.90)	0.64 (0.23, 1.61)
ONE50mgSC	0.66 (0.17, 2.40)	1.31 (0.32, 5.22)	0.86 (0.21, 2.99)	**3.16 (1.05, 9.41)**	**6.68 (2.04, 21.03)**	**3.07 (1.22, 7.54)**	1.61 (0.53, 4.54)	**2.61 (1.01, 7.10)**	0.79 (0.26, 2.14)	0.91 (0.27, 3.49)	1.14 (0.34, 4.02)	0.61 (0.16, 2.40)	0.83 (0.25, 2.46)	0.60 (0.18, 1.86)	3.17 (0.92, 11.49)	2.47 (0.78, 8.99)	0.44 (0.04, 4.31)	2.92 (0.58, 24.06)	1.73 (0.20, 13.73)	1.15 (0.45, 2.93)	1.33 (0.46, 3.26)	0.82 (0.19, 2.83)	0.91 (0.29, 2.49)
ONT22_5mgSC	0.52 (0.14, 1.71)	1.01 (0.29, 3.49)	0.64 (0.19, 2.12)	2.42 (0.95, 6.18)	**5.02 (1.87, 14.81)**	**2.33 (1.09, 5.41)**	1.21 (0.50, 3.12)	2.03 (0.85, 4.74)	0.59 (0.23, 1.53)	0.72 (0.23, 2.36)	0.88 (0.28, 2.89)	0.46 (0.13, 1.66)	0.63 (0.24, 1.70)	0.46 (0.16, 1.25)	2.42 (0.83, 8.25)	1.91 (0.68, 6.30)	0.35 (0.03, 3.18)	2.29 (0.46, 18.81)	1.27 (0.19, 12.04)	0.88 (0.41, 1.96)	0.99 (0.42, 2.33)	0.61 (0.16, 2.04)	0.70 (0.29, 1.73)
ONT225mgSC	0.47 (0.13, 1.53)	0.94 (0.27, 3.18)	0.59 (0.17, 1.88)	2.22 (0.87, 5.74)	**4.57 (1.80, 13.04)**	**2.14 (1.01, 4.67)**	1.12 (0.48, 2.93)	1.84 (0.80, 4.37)	0.54 (0.22, 1.37)	0.66 (0.21, 2.10)	0.82 (0.27, 2.53)	0.42 (0.12, 1.52)	0.58 (0.22, 1.52)	0.42 (0.15, 1.13)	2.27 (0.75, 7.16)	1.80 (0.62, 5.55)	0.31 (0.03, 2.62)	2.01 (0.45, 15.76)	1.16 (0.18, 10.58)	0.81 (0.39, 1.78)	0.91 (0.41, 2.00)	0.56 (0.15, 1.87)	0.64 (0.27, 1.52)
ONT75mgSC	0.50 (0.13, 1.70)	0.98 (0.28, 3.36)	0.63 (0.17, 2.37)	2.41 (0.89, 6.47)	**4.94 (1.77, 14.11)**	**2.28 (1.04, 5.08)**	1.21 (0.48, 3.11)	1.99 (0.84, 4.58)	0.59 (0.22, 1.41)	0.70 (0.22, 2.32)	0.86 (0.28, 2.90)	0.46 (0.12, 1.62)	0.63 (0.22, 1.65)	0.44 (0.16, 1.25)	2.43 (0.78, 7.56)	1.92 (0.66, 5.93)	0.34 (0.03, 2.80)	2.16 (0.45, 16.75)	1.28 (0.20, 11.15)	0.86 (0.40, 1.93)	0.97 (0.42, 2.43)	0.59 (0.16, 2.01)	0.68 (0.29, 1.64)
PBO	0.59 (0.19, 1.63)	1.16 (0.39, 3.33)	0.74 (0.25, 1.94)	**2.76 (1.42, 5.65)**	**5.65 (2.76, 14.16)**	**2.68 (1.77, 4.22)**	1.43 (0.70, 2.76)	**2.33 (1.34, 4.01)**	0.68 (0.34, 1.32)	0.82 (0.31, 2.13)	1.01 (0.41, 2.60)	0.53 (0.17, 1.57)	0.73 (0.32, 1.55)	0.53 (0.22, 1.18)	**2.77 (1.12, 7.87)**	2.15 (0.97, 6.25)	0.41 (0.04, 2.99)	2.47 (0.66, 18.47)	1.50 (0.22, 11.44)	1.01 (0.68, 1.52)	1.14 (0.68, 1.84)	0.70 (0.21, 1.90)	0.82 (0.45, 1.39)
PF10mgSC	0.65 (0.12, 3.05)	1.25 (0.27, 5.95)	0.80 (0.16, 3.69)	3.07 (0.68, 12.74)	**6.39 (1.27, 26.24)**	3.01 (0.71, 10.92)	1.59 (0.30, 5.97)	2.55 (0.57, 9.62)	0.77 (0.18, 2.81)	0.89 (0.17, 4.25)	1.11 (0.23, 5.25)	0.58 (0.10, 3.08)	0.80 (0.17, 3.22)	0.58 (0.12, 2.25)	3.12 (0.53, 14.83)	2.43 (0.48, 11.08)	0.44 (0.04, 3.89)	2.93 (0.41, 23.03)	1.69 (0.17, 14.29)	1.12 (0.27, 4.01)	1.27 (0.32, 4.39)	0.77 (0.12, 3.48)	0.87 (0.20, 3.46)
PF200mgSC	0.65 (0.12, 4.10)	1.24 (0.26, 8.73)	0.79 (0.16, 5.25)	3.03 (0.74, 17.00)	**6.34 (1.52, 36.26)**	2.96 (0.82, 14.44)	1.56 (0.31, 8.42)	2.51 (0.67, 13.73)	0.74 (0.19, 3.85)	0.89 (0.20, 4.93)	1.09 (0.23, 6.55)	0.59 (0.11, 3.63)	0.81 (0.20, 4.25)	0.60 (0.11, 3.14)	3.12 (0.70, 19.90)	2.40 (0.57, 15.20)	0.45 (0.04, 4.79)	2.96 (0.43, 27.14)	1.69 (0.15, 19.27)	1.11 (0.31, 5.25)	1.24 (0.34, 6.20)	0.76 (0.14, 4.86)	0.88 (0.22, 4.30)
PF50mgSC	**0.21 (0.05, 0.87)**	0.43 (0.10, 1.72)	0.27 (0.06, 1.01)	1.01 (0.31, 3.11)	2.13 (0.61, 7.09)	0.99 (0.33, 2.60)	0.53 (0.13, 1.52)	0.85 (0.27, 2.32)	**0.25 (0.07, 0.70)**	0.29 (0.08, 1.14)	0.37 (0.10, 1.36)	**0.19 (0.05, 0.83)**	**0.27 (0.08, 0.83)**	**0.19 (0.05, 0.62)**	1.04 (0.26, 3.90)	0.80 (0.22, 2.99)	0.14 (0.01, 1.26)	0.96 (0.18, 7.01)	0.57 (0.07, 4.24)	**0.38 (0.13, 0.92)**	0.42 (0.14, 1.05)	**0.26 (0.06, 0.93)**	**0.30 (0.10, 0.81)**
RIS1200mgIV	**0.33 (0.11, 0.94)**	0.65 (0.22, 1.93)	0.42 (0.13, 1.11)	1.56 (0.76, 3.25)	**3.18 (1.52, 8.33)**	1.51 (0.96, 2.45)	0.80 (0.37, 1.62)	1.31 (0.73, 2.34)	**0.39 (0.19, 0.77)**	0.46 (0.17, 1.23)	0.56 (0.23, 1.49)	**0.30 (0.10, 0.91)**	**0.41 (0.18, 0.89)**	**0.30 (0.12, 0.69)**	1.55 (0.61, 4.49)	1.22 (0.53, 3.57)	0.23 (0.02, 1.72)	1.40 (0.38, 10.69)	0.84 (0.12, 6.38)	**0.57 (0.37, 0.89)**	0.64 (0.38, 1.08)	0.39 (0.12, 1.07)	**0.46 (0.24, 0.82)**
RIS200mgIV	0.43 (0.11, 1.63)	0.86 (0.25, 3.21)	0.55 (0.16, 1.84)	2.06 (0.72, 5.74)	**4.23 (1.47, 14.66)**	1.98 (0.87, 4.79)	1.04 (0.33, 2.99)	1.70 (0.72, 4.49)	0.50 (0.20, 1.40)	0.60 (0.21, 1.98)	0.75 (0.25, 2.29)	0.40 (0.11, 1.45)	0.54 (0.19, 1.49)	0.40 (0.11, 1.14)	2.05 (0.69, 7.53)	1.63 (0.58, 5.76)	0.30 (0.03, 2.52)	1.94 (0.38, 14.11)	1.11 (0.17, 8.76)	0.75 (0.32, 1.73)	0.84 (0.37, 1.97)	0.52 (0.13, 1.77)	0.60 (0.24, 1.47)
RIS600mgIV	**0.30 (0.10, 0.87)**	0.60 (0.20, 1.76)	0.38 (0.13, 1.02)	1.43 (0.71, 3.01)	**2.92 (1.38, 7.58)**	1.38 (0.88, 2.26)	0.74 (0.34, 1.46)	1.20 (0.67, 2.15)	**0.36 (0.17, 0.70)**	0.42 (0.16, 1.13)	0.52 (0.21, 1.36)	**0.27 (0.09, 0.83)**	**0.38 (0.17, 0.82)**	**0.27 (0.11, 0.64)**	1.43 (0.56, 4.09)	1.12 (0.49, 3.24)	0.21 (0.02, 1.58)	1.29 (0.34, 9.97)	0.77 (0.12, 5.97)	**0.52 (0.34, 0.83)**	**0.59 (0.34, 0.99)**	**0.36 (0.11, 0.99)**	**0.42 (0.22, 0.75)**

(TABLE S12A. continued)

	ABA10mg/kgIV	ABA30mg/kgIV	ABA3mg/kgIV	ADA160mg80mg40mgSC	ADA160mg80mg60mgSC	ADA160mg80mgSC	ADA40mg20mgSC	ADA80mg40mgSC	AMI0.4mgPO	AND150mgSC1.1	AND150mgSC2.2	AND300mgSC	API100mgPO	API50mgPO	BRI400mgIV	BRI700mgIV	BRO210mgIV	BRO350mgIV	BRO700mgIV	CDP10mg/kgIV	CER100mgSC	CER10mg/kgIV	CER200mgSC
RIS600mgIV4_4	0.42 (0.13, 1.26)	0.83 (0.28, 2.53)	0.53 (0.17, 1.47)	1.97 (0.93, 4.44)	**4.08 (1.81, 10.71)**	**1.92 (1.10, 3.35)**	1.02 (0.46, 2.20)	1.67 (0.85, 3.20)	0.49 (0.23, 1.02)	0.58 (0.21, 1.61)	0.73 (0.28, 1.94)	0.38 (0.12, 1.17)	0.52 (0.22, 1.19)	**0.38 (0.15, 0.95)**	2.00 (0.75, 6.05)	1.55 (0.63, 4.54)	0.28 (0.03, 2.18)	1.78 (0.45, 13.26)	1.09 (0.16, 8.31)	0.73 (0.43, 1.23)	0.81 (0.44, 1.49)	0.50 (0.15, 1.42)	0.58 (0.29, 1.12)
SEC10mg/kgIV	0.78 (0.12, 4.92)	1.58 (0.27, 9.63)	1.00 (0.16, 6.17)	3.79 (0.79, 17.67)	**7.78 (1.66, 41.76)**	3.54 (0.80, 16.65)	1.93 (0.33, 9.27)	3.05 (0.65, 15.56)	0.90 (0.19, 5.21)	1.11 (0.19, 6.36)	1.40 (0.22, 7.64)	0.74 (0.10, 4.39)	0.98 (0.19, 5.15)	0.69 (0.13, 3.71)	3.84 (0.68, 19.77)	3.00 (0.58, 15.58)	0.53 (0.03, 6.61)	3.60 (0.48, 33.10)	2.06 (0.20, 20.69)	1.35 (0.30, 6.09)	1.54 (0.33, 8.19)	0.90 (0.16, 5.50)	1.08 (0.23, 5.55)
TES400mg200mgSC	**0.29 (0.08, 0.97)**	0.56 (0.17, 2.04)	0.37 (0.10, 1.11)	1.37 (0.55, 3.52)	**2.80 (1.03, 8.65)**	1.34 (0.65, 2.70)	0.70 (0.28, 1.69)	1.14 (0.49, 2.50)	**0.34 (0.13, 0.80)**	0.40 (0.13, 1.22)	0.49 (0.17, 1.45)	**0.26 (0.07, 0.87)**	**0.36 (0.13, 0.94)**	**0.26 (0.08, 0.73)**	1.40 (0.44, 4.40)	1.07 (0.39, 3.31)	0.19 (0.02, 1.69)	1.26 (0.27, 9.97)	0.74 (0.10, 6.13)	**0.50 (0.24, 0.99)**	0.56 (0.24, 1.16)	0.34 (0.09, 1.12)	**0.40 (0.16, 0.88)**
TOF15mgPO_BID	0.91 (0.15, 4.21)	1.81 (0.35, 8.86)	1.12 (0.23, 5.12)	**4.10 (1.09, 18.66)**	**8.60 (2.17, 38.64)**	**4.07 (1.15, 15.62)**	2.13 (0.57, 8.58)	3.50 (0.85, 13.67)	1.04 (0.27, 3.88)	1.23 (0.28, 5.94)	1.52 (0.37, 7.11)	0.81 (0.15, 4.01)	1.11 (0.29, 4.55)	0.80 (0.16, 3.31)	4.20 (0.97, 21.55)	3.34 (0.75, 16.29)	0.63 (0.04, 6.16)	4.10 (0.55, 35.05)	2.45 (0.22, 18.98)	1.55 (0.44, 5.81)	1.69 (0.47, 6.76)	1.04 (0.17, 5.03)	1.23 (0.31, 4.79)
TOF1mgPO_BID	0.39 (0.09, 1.47)	0.77 (0.18, 2.98)	0.48 (0.11, 1.75)	1.81 (0.60, 5.49)	**3.69 (1.10, 13.98)**	1.78 (0.59, 4.70)	0.92 (0.27, 2.89)	1.54 (0.48, 4.33)	0.45 (0.14, 1.35)	0.53 (0.15, 1.95)	0.66 (0.20, 2.41)	0.35 (0.08, 1.43)	0.47 (0.14, 1.45)	0.35 (0.09, 1.13)	1.77 (0.53, 8.07)	1.39 (0.44, 5.96)	0.27 (0.02, 2.60)	1.71 (0.30, 13.21)	1.00 (0.09, 8.63)	0.66 (0.22, 1.74)	0.75 (0.26, 2.01)	0.44 (0.11, 1.79)	0.53 (0.16, 1.55)
TOF5mgPO_BID	0.51 (0.11, 1.96)	1.00 (0.24, 4.00)	0.64 (0.15, 2.31)	2.39 (0.80, 7.57)	**4.84 (1.48, 17.76)**	2.31 (0.83, 6.31)	1.21 (0.36, 3.83)	2.03 (0.63, 5.75)	0.59 (0.19, 1.74)	0.69 (0.20, 2.44)	0.88 (0.25, 3.13)	0.47 (0.11, 1.75)	0.64 (0.18, 1.98)	0.46 (0.12, 1.48)	2.39 (0.68, 9.96)	1.87 (0.56, 7.74)	0.33 (0.03, 3.55)	2.25 (0.43, 17.98)	1.31 (0.12, 11.29)	0.86 (0.29, 2.29)	0.97 (0.35, 2.63)	0.60 (0.14, 2.45)	0.71 (0.22, 1.97)
UPA12mgPO_BID	0.50 (0.08, 2.52)	0.97 (0.19, 5.41)	0.64 (0.11, 3.00)	2.39 (0.55, 9.11)	**4.84 (1.16, 22.98)**	2.36 (0.58, 8.21)	1.21 (0.29, 4.70)	1.98 (0.48, 7.78)	0.58 (0.13, 2.30)	0.70 (0.14, 3.76)	0.88 (0.17, 4.12)	0.46 (0.09, 2.42)	0.62 (0.15, 2.71)	0.46 (0.09, 2.08)	2.40 (0.52, 11.15)	1.89 (0.41, 8.53)	0.32 (0.03, 4.40)	2.25 (0.32, 24.98)	1.25 (0.14, 15.21)	0.89 (0.22, 3.16)	0.97 (0.25, 3.81)	0.60 (0.10, 2.63)	0.68 (0.17, 2.68)
UPA24mgPO	0.88 (0.11, 5.69)	1.68 (0.29, 10.86)	1.03 (0.18, 7.00)	3.99 (0.79, 20.57)	**8.39 (1.78, 50.28)**	4.00 (0.83, 18.21)	2.07 (0.41, 9.96)	3.46 (0.65, 15.53)	1.04 (0.20, 5.55)	1.22 (0.21, 6.31)	1.52 (0.25, 7.67)	0.81 (0.11, 4.37)	1.08 (0.21, 6.04)	0.76 (0.13, 4.46)	4.11 (0.74, 26.53)	3.19 (0.60, 20.60)	0.54 (0.04, 10.03)	3.78 (0.51, 50.55)	2.19 (0.25, 28.47)	1.50 (0.31, 7.30)	1.67 (0.35, 7.76)	1.03 (0.16, 6.35)	1.21 (0.23, 5.52)
UPA24mgPO_BID	0.27 (0.05, 1.26)	0.53 (0.12, 2.32)	0.34 (0.06, 1.44)	1.26 (0.33, 4.38)	2.64 (0.69, 10.18)	1.22 (0.37, 3.76)	0.63 (0.18, 2.27)	1.07 (0.31, 3.31)	0.31 (0.08, 1.10)	0.37 (0.09, 1.60)	0.48 (0.10, 1.79)	0.25 (0.05, 1.10)	0.33 (0.09, 1.18)	0.23 (0.06, 1.06)	1.27 (0.30, 5.59)	1.01 (0.24, 4.17)	0.18 (0.02, 1.84)	1.16 (0.19, 11.20)	0.67 (0.08, 7.31)	0.47 (0.14, 1.39)	0.52 (0.15, 1.65)	0.33 (0.06, 1.30)	0.37 (0.10, 1.19)
UPA3mgPO_BID	0.68 (0.12, 4.55)	1.38 (0.27, 8.08)	0.87 (0.16, 5.55)	3.27 (0.81, 15.64)	**6.80 (1.62, 40.63)**	3.20 (0.83, 14.82)	1.69 (0.40, 7.94)	2.79 (0.65, 12.83)	0.82 (0.19, 3.98)	0.92 (0.21, 6.21)	1.21 (0.25, 7.11)	0.63 (0.11, 4.00)	0.84 (0.21, 4.57)	0.62 (0.13, 3.33)	3.38 (0.69, 18.74)	2.60 (0.57, 14.71)	0.46 (0.04, 5.86)	3.17 (0.51, 30.24)	1.81 (0.18, 17.17)	1.20 (0.31, 5.61)	1.34 (0.35, 5.93)	0.83 (0.13, 5.18)	0.96 (0.25, 4.33)
UPA45mgPO	**0.34 (0.11, 0.95)**	0.66 (0.22, 1.96)	0.43 (0.14, 1.14)	1.58 (0.78, 3.34)	**3.26 (1.52, 8.19)**	1.53 (0.96, 2.55)	0.81 (0.39, 1.62)	1.33 (0.73, 2.42)	**0.39 (0.19, 0.77)**	0.47 (0.18, 1.25)	0.58 (0.23, 1.53)	**0.30 (0.10, 0.91)**	**0.42 (0.18, 0.91)**	**0.30 (0.12, 0.70)**	1.58 (0.63, 4.76)	1.24 (0.54, 3.59)	0.23 (0.02, 1.73)	1.42 (0.37, 10.51)	0.85 (0.13, 6.52)	**0.58 (0.37, 0.92)**	0.65 (0.37, 1.11)	0.40 (0.12, 1.11)	**0.47 (0.25, 0.83)**
UPA6mgPO_BID	**0.22 (0.04, 0.93)**	0.43 (0.10, 1.90)	0.28 (0.06, 1.13)	1.07 (0.28, 3.30)	2.22 (0.59, 8.13)	1.04 (0.31, 2.84)	0.54 (0.15, 1.69)	0.90 (0.24, 2.57)	**0.26 (0.07, 0.84)**	0.30 (0.07, 1.35)	0.38 (0.09, 1.49)	**0.20 (0.04, 0.90)**	**0.28 (0.07, 0.92)**	**0.19 (0.04, 0.76)**	1.06 (0.25, 4.33)	0.84 (0.21, 3.25)	0.14 (0.01, 1.62)	0.95 (0.17, 10.05)	0.54 (0.08, 5.67)	0.40 (0.11, 1.02)	0.43 (0.12, 1.22)	0.27 (0.05, 1.04)	**0.31 (0.08, 0.92)**
UST130mgIV	**0.35 (0.11, 0.99)**	0.68 (0.23, 2.06)	0.44 (0.14, 1.17)	1.65 (0.77, 3.53)	**3.38 (1.52, 8.56)**	1.58 (0.95, 2.68)	0.84 (0.38, 1.73)	1.36 (0.73, 2.55)	**0.40 (0.19, 0.84)**	0.48 (0.18, 1.34)	0.60 (0.23, 1.62)	**0.32 (0.10, 0.98)**	**0.43 (0.19, 0.93)**	**0.31 (0.12, 0.74)**	1.67 (0.62, 4.77)	1.27 (0.54, 3.85)	0.23 (0.02, 1.87)	1.45 (0.39, 11.18)	0.86 (0.13, 6.88)	0.60 (0.37, 1.00)	0.67 (0.38, 1.19)	0.42 (0.12, 1.16)	**0.48 (0.24, 0.90)**
UST1mg/kgIV	**0.31 (0.08, 0.96)**	0.59 (0.19, 2.00)	0.39 (0.11, 1.14)	1.46 (0.59, 3.40)	**2.96 (1.23, 8.39)**	1.42 (0.69, 2.86)	0.73 (0.29, 1.73)	1.22 (0.53, 2.59)	**0.36 (0.15, 0.81)**	0.43 (0.14, 1.23)	0.52 (0.19, 1.48)	**0.28 (0.08, 0.91)**	**0.39 (0.14, 0.93)**	**0.28 (0.09, 0.71)**	1.46 (0.50, 4.59)	1.14 (0.44, 3.57)	0.20 (0.02, 1.75)	1.30 (0.32, 10.18)	0.76 (0.11, 6.00)	0.53 (0.27, 1.02)	0.59 (0.29, 1.20)	0.37 (0.10, 1.11)	**0.42 (0.19, 0.88)**
UST3mg/kgIV	**0.29 (0.08, 0.93)**	0.58 (0.18, 1.95)	0.37 (0.11, 1.08)	1.40 (0.60, 3.22)	**2.84 (1.17, 7.98)**	1.35 (0.69, 2.61)	0.72 (0.29, 1.63)	1.17 (0.52, 2.45)	**0.34 (0.15, 0.77)**	0.40 (0.14, 1.20)	0.51 (0.18, 1.44)	**0.27 (0.08, 0.87)**	**0.37 (0.14, 0.88)**	**0.26 (0.10, 0.71)**	1.39 (0.47, 4.50)	1.08 (0.41, 3.50)	0.20 (0.02, 1.67)	1.25 (0.30, 10.50)	0.73 (0.11, 5.94)	**0.50 (0.27, 0.97)**	0.57 (0.28, 1.14)	0.35 (0.10, 1.09)	**0.40 (0.18, 0.85)**
UST4.5mg/kgIV	0.35 (0.11, 1.01)	0.68 (0.22, 2.03)	0.43 (0.14, 1.17)	1.61 (0.77, 3.54)	**3.31 (1.46, 8.83)**	1.58 (0.93, 2.73)	0.83 (0.38, 1.74)	1.35 (0.71, 2.58)	**0.40 (0.19, 0.80)**	0.48 (0.18, 1.32)	0.59 (0.23, 1.60)	**0.31 (0.09, 0.96)**	**0.43 (0.18, 0.94)**	**0.31 (0.12, 0.73)**	1.62 (0.63, 4.68)	1.28 (0.52, 3.74)	0.23 (0.02, 1.83)	1.46 (0.38, 11.63)	0.87 (0.13, 6.99)	**0.59 (0.36, 0.99)**	0.66 (0.36, 1.18)	0.41 (0.12, 1.19)	**0.47 (0.24, 0.88)**
UST6mg/kg90mgIV/SC	**0.21 (0.05, 0.67)**	0.40 (0.11, 1.33)	**0.25 (0.08, 0.82)**	0.95 (0.75, 1.22)	1.99 (0.74, 6.04)	0.93 (0.41, 1.97)	0.49 (0.18, 1.21)	0.80 (0.34, 1.85)	**0.23 (0.09, 0.60)**	**0.28 (0.08, 0.89)**	0.34 (0.11, 1.11)	**0.19 (0.05, 0.64)**	**0.25 (0.09, 0.69)**	**0.18 (0.06, 0.54)**	0.97 (0.33, 3.07)	0.75 (0.27, 2.55)	0.14 (0.01, 1.18)	0.86 (0.20, 7.05)	0.52 (0.07, 4.57)	**0.35 (0.16, 0.72)**	**0.39 (0.17, 0.87)**	**0.24 (0.06, 0.80)**	**0.28 (0.12, 0.65)**
UST6mg/kgIV	**0.27 (0.08, 0.76)**	0.52 (0.18, 1.59)	**0.34 (0.11, 0.89)**	1.27 (0.61, 2.69)	**2.59 (1.21, 6.67)**	1.22 (0.75, 2.02)	0.65 (0.31, 1.33)	1.06 (0.57, 1.94)	**0.31 (0.15, 0.63)**	0.37 (0.14, 1.01)	0.46 (0.18, 1.21)	**0.24 (0.08, 0.75)**	**0.34 (0.14, 0.74)**	**0.24 (0.10, 0.57)**	1.28 (0.49, 3.63)	0.99 (0.43, 2.92)	0.18 (0.02, 1.42)	1.11 (0.30, 8.97)	0.67 (0.10, 5.40)	**0.46 (0.29, 0.76)**	**0.52 (0.29, 0.89)**	**0.32 (0.09, 0.89)**	**0.37 (0.19, 0.67)**
UST90mgSC	0.57 (0.12, 2.37)	1.10 (0.25, 4.77)	0.70 (0.16, 2.95)	2.61 (0.76, 8.70)	**5.49 (1.47, 20.14)**	2.56 (0.79, 7.62)	1.32 (0.35, 4.61)	2.23 (0.61, 6.90)	0.65 (0.17, 2.12)	0.78 (0.18, 3.22)	0.96 (0.23, 3.54)	0.51 (0.11, 2.21)	0.70 (0.18, 2.40)	0.49 (0.12, 1.83)	2.70 (0.68, 10.14)	2.13 (0.54, 7.97)	0.39 (0.03, 3.72)	2.37 (0.40, 21.18)	1.36 (0.21, 13.45)	0.96 (0.30, 2.81)	1.08 (0.32, 3.42)	0.67 (0.13, 2.65)	0.75 (0.24, 2.53)
VED0_5mg/kgIV	0.42 (0.11, 1.32)	0.82 (0.24, 2.77)	0.52 (0.15, 1.61)	1.92 (0.77, 5.03)	**4.06 (1.45, 11.84)**	1.90 (0.83, 4.11)	1.00 (0.36, 2.51)	1.64 (0.68, 3.89)	0.48 (0.18, 1.19)	0.58 (0.18, 1.85)	0.70 (0.22, 2.19)	0.37 (0.10, 1.34)	0.51 (0.18, 1.46)	0.36 (0.12, 1.12)	1.97 (0.62, 6.21)	1.52 (0.52, 5.22)	0.28 (0.02, 2.57)	1.85 (0.39, 14.32)	1.07 (0.14, 8.66)	0.72 (0.32, 1.52)	0.81 (0.31, 1.75)	0.48 (0.12, 1.70)	0.57 (0.24, 1.32)
VED2mg/kgIV	0.33 (0.09, 1.04)	0.65 (0.18, 2.10)	0.41 (0.11, 1.45)	1.56 (0.62, 3.85)	**3.19 (1.21, 9.52)**	1.51 (0.65, 3.12)	0.79 (0.29, 1.96)	1.30 (0.54, 2.97)	**0.38 (0.15, 0.95)**	0.46 (0.15, 1.51)	0.56 (0.18, 1.80)	0.30 (0.08, 1.03)	0.41 (0.14, 1.11)	**0.29 (0.10, 0.87)**	1.56 (0.49, 4.94)	1.22 (0.42, 3.87)	0.23 (0.02, 2.00)	1.41 (0.32, 11.55)	0.85 (0.12, 6.96)	0.57 (0.25, 1.19)	0.64 (0.26, 1.47)	0.39 (0.10, 1.27)	0.45 (0.19, 1.01)
VED300mgIV	**0.28 (0.09, 0.83)**	0.55 (0.18, 1.66)	**0.35 (0.12, 0.95)**	1.30 (0.63, 2.87)	**2.68 (1.22, 7.12)**	1.27 (0.74, 2.28)	0.67 (0.31, 1.40)	1.10 (0.57, 2.08)	**0.32 (0.15, 0.68)**	0.38 (0.15, 1.06)	0.47 (0.19, 1.27)	**0.25 (0.08, 0.77)**	**0.35 (0.14, 0.78)**	**0.25 (0.10, 0.60)**	1.32 (0.50, 3.90)	1.02 (0.43, 3.09)	0.19 (0.02, 1.52)	1.19 (0.30, 9.19)	0.70 (0.10, 5.73)	**0.48 (0.29, 0.80)**	**0.54 (0.29, 0.95)**	**0.33 (0.10, 0.94)**	**0.39 (0.19, 0.72)**

	CER20mg/kgIV	CER400mgSC	CER5mg/kgIV	ELD10mg/kgIV	ELD20mg/kgIV	ETA25mgSC	ETR105mgSC	ETR210mgSC	FIL100mgPO	FIL200mgPO	FON0.1mg/kgIV	FON1mg/kg0.1mgIV/SC	FON1mg/kg1mgIV/SC	FON1mg/kgIV	FON4mg/kg0.1mgIV/SC	FON4mg/kg1mgIV/SC	FON4mg/kgIV	GUS1200mgIV	GUS200mgIV	GUS600mgIV	INF10mg/kgIV	INF20mg/kgIV	INF5mg/kgIV
ABA10mg/kgIV	0.64 (0.10, 3.12)	2.08 (0.74, 6.58)	1.68 (0.46, 6.87)	1.85 (0.47, 7.81)	2.51 (0.66, 9.61)	0.87 (0.15, 4.73)	1.62 (0.54, 5.40)	1.84 (0.64, 6.06)	**3.06 (1.06, 9.98)**	2.55 (0.90, 8.30)	0.66 (0.03, 5.75)	1.85 (0.48, 7.61)	2.39 (0.68, 9.63)	1.42 (0.23, 8.44)	2.85 (0.84, 11.66)	2.31 (0.69, 9.85)	2.87 (0.59, 15.28)	**4.79 (1.49, 18.71)**	**6.17 (1.85, 22.77)**	**5.93 (1.80, 22.83)**	**14.71 (1.77, 143.02)**	**14.42 (1.78, 141.34)**	**27.56 (3.79, 269.00)**
ABA30mg/kgIV	0.33 (0.05, 1.57)	1.06 (0.36, 3.18)	0.85 (0.23, 3.38)	0.94 (0.23, 3.76)	1.27 (0.33, 4.94)	0.44 (0.06, 2.23)	0.83 (0.27, 2.53)	0.95 (0.31, 2.95)	1.55 (0.52, 4.72)	1.29 (0.42, 3.91)	0.33 (0.02, 3.09)	0.93 (0.23, 3.62)	1.21 (0.32, 4.48)	0.71 (0.12, 3.83)	1.47 (0.40, 5.53)	1.20 (0.34, 4.60)	1.43 (0.31, 7.44)	2.47 (0.74, 8.99)	3.11 (0.96, 11.00)	2.99 (0.91, 10.78)	7.18 (0.81, 87.84)	7.15 (0.77, 81.46)	**13.99 (1.61, 159.67)**
ABA3mg/kgIV	0.52 (0.09, 2.31)	1.65 (0.62, 4.89)	1.37 (0.38, 5.28)	1.47 (0.39, 6.24)	1.95 (0.55, 8.06)	0.70 (0.10, 3.79)	1.27 (0.46, 4.16)	1.46 (0.54, 4.65)	2.40 (0.88, 7.64)	2.00 (0.74, 6.22)	0.53 (0.03, 4.72)	1.50 (0.39, 5.48)	1.87 (0.54, 7.47)	1.09 (0.20, 6.98)	2.32 (0.69, 8.71)	1.92 (0.53, 7.41)	2.16 (0.54, 12.35)	**3.94 (1.25, 13.42)**	**4.94 (1.57, 16.72)**	**4.81 (1.50, 16.17)**	**11.77 (1.28, 138.72)**	**11.56 (1.26, 124.52)**	**23.50 (2.76, 239.27)**
ADA160mg80mg40mgSC	**0.13 (0.03, 0.51)**	**0.44 (0.21, 0.89)**	0.36 (0.12, 1.08)	0.40 (0.13, 1.24)	0.52 (0.18, 1.66)	**0.18 (0.03, 0.79)**	**0.34 (0.15, 0.75)**	**0.39 (0.17, 0.85)**	0.65 (0.30, 1.35)	0.54 (0.25, 1.13)	0.14 (0.01, 1.02)	0.39 (0.13, 1.13)	0.50 (0.18, 1.50)	0.29 (0.06, 1.34)	0.62 (0.23, 1.68)	0.51 (0.18, 1.45)	0.60 (0.13, 2.54)	1.05 (0.63, 1.71)	1.31 (0.84, 2.08)	1.26 (0.80, 2.01)	2.96 (0.40, 32.14)	3.01 (0.35, 30.80)	5.85 (0.83, 57.55)
ADA160mg80mg60mgSC	**0.07 (0.01, 0.25)**	**0.22 (0.09, 0.46)**	**0.17 (0.05, 0.57)**	**0.19 (0.05, 0.62)**	**0.26 (0.08, 0.77)**	**0.09 (0.02, 0.36)**	**0.17 (0.06, 0.37)**	**0.19 (0.07, 0.43)**	**0.32 (0.12, 0.70)**	**0.26 (0.10, 0.57)**	**0.07 (0.00, 0.54)**	**0.19 (0.05, 0.58)**	**0.24 (0.08, 0.74)**	**0.14 (0.03, 0.67)**	**0.30 (0.09, 0.85)**	**0.24 (0.08, 0.73)**	0.29 (0.07, 1.23)	0.50 (0.17, 1.44)	0.63 (0.21, 1.71)	0.61 (0.20, 1.63)	1.46 (0.18, 14.62)	1.44 (0.17, 13.44)	2.79 (0.39, 25.58)
ADA160mg80mgSC	**0.14 (0.03, 0.46)**	**0.46 (0.28, 0.73)**	0.37 (0.15, 1.00)	0.41 (0.15, 1.16)	0.54 (0.23, 1.43)	**0.19 (0.04, 0.72)**	**0.35 (0.20, 0.62)**	**0.41 (0.23, 0.71)**	0.67 (0.40, 1.14)	**0.56 (0.33, 0.93)**	**0.14 (0.01, 0.97)**	0.41 (0.15, 1.02)	0.52 (0.22, 1.28)	0.31 (0.07, 1.32)	0.65 (0.27, 1.46)	0.52 (0.22, 1.30)	0.59 (0.18, 2.41)	1.08 (0.49, 2.46)	1.36 (0.63, 2.92)	1.31 (0.62, 2.88)	3.09 (0.46, 30.11)	2.97 (0.44, 27.93)	**5.64 (1.07, 51.33)**
ADA40mg20mgSC	0.27 (0.05, 1.00)	0.86 (0.43, 1.81)	0.70 (0.25, 2.09)	0.78 (0.26, 2.48)	1.06 (0.39, 2.96)	0.37 (0.07, 1.39)	0.68 (0.31, 1.46)	0.77 (0.36, 1.62)	1.26 (0.61, 2.77)	1.04 (0.51, 2.32)	0.27 (0.02, 1.95)	0.77 (0.26, 2.28)	0.99 (0.37, 2.82)	0.58 (0.12, 2.66)	1.20 (0.46, 3.34)	1.01 (0.37, 2.88)	1.13 (0.33, 5.26)	2.05 (0.83, 5.74)	**2.56 (1.04, 6.85)**	**2.47 (1.02, 6.67)**	5.77 (0.80, 65.69)	5.59 (0.79, 60.24)	**11.14 (1.81, 130.25)**
ADA80mg40mgSC	**0.16 (0.03, 0.56)**	**0.53 (0.29, 0.96)**	0.42 (0.16, 1.29)	0.47 (0.17, 1.36)	0.63 (0.25, 1.69)	**0.22 (0.04, 0.84)**	**0.41 (0.21, 0.81)**	**0.47 (0.25, 0.89)**	0.78 (0.42, 1.46)	0.64 (0.35, 1.20)	0.17 (0.01, 1.13)	0.48 (0.17, 1.21)	0.60 (0.24, 1.57)	0.35 (0.08, 1.50)	0.75 (0.30, 1.81)	0.60 (0.24, 1.53)	0.70 (0.19, 2.86)	1.25 (0.54, 2.97)	1.57 (0.68, 3.63)	1.52 (0.66, 3.47)	3.56 (0.48, 39.29)	3.47 (0.49, 34.27)	**6.69 (1.10, 62.63)**
AMI0.4mgPO	0.57 (0.10, 1.95)	1.79 (0.90, 3.63)	1.44 (0.51, 4.59)	1.60 (0.55, 5.03)	2.14 (0.77, 6.37)	0.73 (0.15, 3.19)	1.38 (0.65, 3.00)	1.58 (0.76, 3.45)	**2.63 (1.25, 5.63)**	**2.20 (1.07, 4.61)**	0.56 (0.04, 4.71)	1.60 (0.53, 4.57)	2.02 (0.76, 5.90)	1.21 (0.23, 5.78)	2.52 (0.95, 6.83)	2.06 (0.74, 5.97)	2.37 (0.63, 11.07)	**4.29 (1.64, 11.18)**	**5.35 (2.09, 13.68)**	**5.19 (2.07, 13.12)**	**11.75 (1.75, 128.05)**	**11.89 (1.64, 118.02)**	**23.20 (3.78, 223.20)**
AND150mgSC1.1	0.45 (0.09, 2.12)	1.51 (0.56, 3.89)	1.18 (0.36, 4.49)	1.36 (0.36, 5.33)	1.76 (0.52, 6.67)	0.63 (0.10, 3.09)	1.16 (0.41, 3.12)	1.34 (0.46, 3.62)	2.20 (0.80, 6.08)	1.85 (0.67, 4.88)	0.49 (0.03, 4.47)	1.37 (0.37, 4.52)	1.71 (0.49, 5.82)	0.99 (0.19, 5.32)	2.18 (0.63, 6.85)	1.76 (0.52, 5.77)	2.06 (0.44, 9.63)	**3.54 (1.15, 11.54)**	**4.46 (1.44, 14.29)**	**4.29 (1.39, 13.96)**	**9.92 (1.25, 133.52)**	**10.08 (1.12, 124.60)**	**19.09 (2.51, 223.03)**
AND150mgSC2.2	0.37 (0.07, 1.57)	1.22 (0.46, 3.09)	0.95 (0.30, 3.60)	1.08 (0.29, 3.90)	1.45 (0.42, 5.07)	0.49 (0.09, 2.33)	0.94 (0.34, 2.53)	1.10 (0.37, 2.82)	1.78 (0.64, 4.62)	1.49 (0.55, 3.86)	0.38 (0.02, 3.25)	1.09 (0.30, 3.70)	1.37 (0.41, 4.77)	0.81 (0.15, 4.10)	1.73 (0.51, 5.27)	1.39 (0.43, 4.64)	1.65 (0.37, 7.97)	2.93 (0.88, 9.26)	**3.65 (1.12, 11.13)**	**3.48 (1.10, 10.65)**	**8.03 (1.02, 93.69)**	8.42 (0.93, 93.68)	**16.38 (2.12, 175.90)**
AND300mgSC	0.70 (0.12, 3.35)	2.31 (0.77, 7.14)	1.84 (0.50, 7.65)	2.09 (0.51, 8.22)	2.75 (0.73, 11.27)	0.96 (0.15, 5.24)	1.78 (0.57, 5.79)	2.07 (0.66, 6.69)	**3.42 (1.09, 10.85)**	2.83 (0.92, 8.86)	0.74 (0.04, 7.16)	2.11 (0.53, 8.21)	2.63 (0.74, 10.63)	1.54 (0.25, 9.00)	3.26 (0.92, 12.44)	2.67 (0.73, 10.72)	3.12 (0.68, 16.51)	**5.48 (1.58, 20.11)**	**6.79 (2.00, 25.21)**	**6.58 (1.95, 24.08)**	**15.47 (1.83, 201.32)**	**15.90 (1.73, 194.41)**	**30.18 (3.67, 381.61)**
API100mgPO	0.52 (0.09, 2.09)	1.68 (0.78, 3.88)	1.34 (0.46, 4.33)	1.49 (0.49, 5.09)	1.96 (0.73, 6.32)	0.68 (0.13, 3.12)	1.30 (0.56, 3.16)	1.48 (0.64, 3.72)	**2.45 (1.07, 5.76)**	2.05 (0.91, 4.92)	0.54 (0.04, 3.69)	1.49 (0.52, 4.70)	1.88 (0.64, 6.03)	1.15 (0.23, 5.24)	2.36 (0.83, 6.83)	1.95 (0.65, 5.95)	2.25 (0.55, 10.18)	**3.94 (1.43, 10.83)**	**5.01 (1.82, 13.77)**	**4.84 (1.76, 13.33)**	**11.66 (1.55, 116.92)**	**11.47 (1.49, 114.38)**	**22.20 (3.39, 218.61)**
API50mgPO	0.72 (0.12, 2.85)	**2.28 (1.01, 5.97)**	1.88 (0.60, 6.19)	2.08 (0.62, 7.19)	2.80 (0.92, 9.05)	0.94 (0.19, 4.46)	1.79 (0.75, 4.66)	2.05 (0.85, 5.51)	**3.38 (1.42, 8.66)**	**2.82 (1.19, 7.53)**	0.73 (0.05, 6.29)	2.07 (0.67, 7.20)	2.64 (0.90, 8.64)	1.57 (0.32, 7.95)	**3.28 (1.11, 10.23)**	2.72 (0.85, 8.78)	3.10 (0.75, 14.30)	**5.60 (1.89, 16.59)**	**6.88 (2.37, 20.83)**	**6.74 (2.33, 19.74)**	**15.88 (2.21, 172.49)**	**15.57 (2.11, 162.03)**	**31.17 (4.51, 317.99)**
BRI400mgIV	**0.13 (0.02, 0.58)**	0.44 (0.15, 1.11)	0.34 (0.10, 1.30)	0.39 (0.10, 1.43)	0.52 (0.14, 1.91)	**0.18 (0.03, 0.83)**	**0.34 (0.11, 0.91)**	0.39 (0.13, 1.08)	0.65 (0.22, 1.74)	0.54 (0.18, 1.41)	0.14 (0.01, 1.06)	0.40 (0.11, 1.41)	0.49 (0.15, 1.73)	0.31 (0.05, 1.47)	0.62 (0.18, 1.98)	0.49 (0.15, 1.74)	0.59 (0.13, 2.72)	1.04 (0.32, 3.13)	1.31 (0.39, 3.90)	1.28 (0.40, 3.79)	2.89 (0.36, 32.45)	2.96 (0.35, 32.01)	5.77 (0.75, 60.41)
BRI700mgIV	**0.17 (0.03, 0.68)**	0.57 (0.19, 1.32)	0.44 (0.13, 1.54)	0.51 (0.13, 1.75)	0.67 (0.18, 2.23)	0.23 (0.04, 1.00)	0.44 (0.14, 1.05)	0.51 (0.17, 1.22)	0.83 (0.28, 1.95)	0.70 (0.23, 1.61)	0.18 (0.01, 1.44)	0.50 (0.13, 1.65)	0.64 (0.19, 1.96)	0.39 (0.06, 1.86)	0.79 (0.23, 2.41)	0.64 (0.19, 2.00)	0.74 (0.16, 3.58)	1.35 (0.39, 3.69)	1.71 (0.49, 4.72)	1.63 (0.48, 4.53)	3.71 (0.46, 43.35)	3.69 (0.44, 40.80)	7.18 (0.93, 76.90)
BRO210mgIV	0.94 (0.09, 13.68)	3.09 (0.39, 34.56)	2.44 (0.26, 32.25)	2.86 (0.34, 33.43)	3.77 (0.45, 43.88)	1.20 (0.09, 19.98)	2.38 (0.29, 27.74)	2.77 (0.33, 31.10)	4.48 (0.58, 51.07)	3.74 (0.47, 42.62)	1.02 (0.02, 31.80)	2.70 (0.30, 35.49)	3.49 (0.38, 45.53)	2.20 (0.13, 32.79)	4.38 (0.52, 52.79)	3.63 (0.41, 46.02)	4.42 (0.34, 59.10)	7.36 (0.83, 87.92)	**9.04 (1.08, 107.75)**	**8.96 (1.03, 103.83)**	**22.89 (1.56, 650.63)**	**22.37 (1.29, 635.90)**	**43.22 (3.18, 1201.76)**
BRO350mgIV	**0.14 (0.01, 0.82)**	0.49 (0.06, 1.91)	0.40 (0.04, 1.82)	0.42 (0.05, 2.20)	0.56 (0.07, 2.84)	0.19 (0.02, 1.27)	0.38 (0.05, 1.55)	0.43 (0.06, 1.79)	0.72 (0.09, 2.75)	0.60 (0.08, 2.27)	0.14 (0.00, 1.96)	0.42 (0.06, 2.25)	0.54 (0.07, 2.77)	0.30 (0.03, 2.42)	0.67 (0.09, 3.31)	0.54 (0.07, 2.78)	0.61 (0.07, 4.13)	1.16 (0.14, 4.81)	1.45 (0.18, 6.20)	1.39 (0.17, 6.11)	3.08 (0.26, 53.51)	3.06 (0.23, 49.10)	6.18 (0.50, 94.36)
BRO700mgIV	0.25 (0.02, 2.25)	0.83 (0.11, 5.54)	0.67 (0.08, 4.87)	0.74 (0.09, 6.18)	0.97 (0.11, 6.80)	0.33 (0.03, 2.45)	0.64 (0.08, 4.62)	0.73 (0.09, 5.23)	1.20 (0.16, 8.51)	1.00 (0.13, 6.66)	0.25 (0.01, 3.77)	0.70 (0.08, 5.92)	0.89 (0.11, 7.70)	0.52 (0.05, 5.50)	1.12 (0.13, 8.86)	0.93 (0.11, 7.78)	1.04 (0.11, 10.32)	1.94 (0.22, 13.78)	2.41 (0.28, 17.18)	2.30 (0.28, 16.65)	5.38 (0.42, 92.86)	5.50 (0.39, 91.96)	10.02 (0.82, 222.05)
CDP10mg/kgIV	0.38 (0.07, 1.17)	1.21 (0.77, 1.89)	0.97 (0.40, 2.74)	1.08 (0.41, 2.93)	1.42 (0.61, 3.81)	0.51 (0.11, 1.92)	0.94 (0.54, 1.60)	1.08 (0.62, 1.82)	**1.78 (1.08, 2.97)**	1.48 (0.91, 2.42)	0.38 (0.03, 2.66)	1.09 (0.42, 2.73)	1.36 (0.59, 3.36)	0.82 (0.19, 3.38)	1.69 (0.75, 4.00)	1.38 (0.59, 3.46)	1.60 (0.47, 6.46)	**2.82 (1.37, 6.31)**	**3.53 (1.75, 7.53)**	**3.45 (1.71, 7.43)**	**8.06 (1.26, 82.85)**	**7.84 (1.11, 75.22)**	**15.23 (2.54, 144.14)**
CER100mgSC	0.34 (0.06, 1.14)	1.08 (0.67, 1.79)	0.86 (0.35, 2.50)	0.97 (0.34, 2.80)	1.28 (0.54, 3.49)	0.45 (0.09, 1.74)	0.84 (0.46, 1.57)	0.96 (0.53, 1.80)	1.58 (0.88, 2.88)	1.32 (0.75, 2.39)	0.36 (0.02, 2.33)	0.96 (0.35, 2.53)	1.22 (0.51, 3.30)	0.73 (0.16, 2.97)	1.52 (0.62, 3.79)	1.24 (0.50, 3.30)	1.44 (0.41, 5.85)	**2.55 (1.17, 5.84)**	**3.16 (1.52, 7.31)**	**3.09 (1.44, 7.08)**	**7.18 (1.10, 73.76)**	**7.26 (1.05, 69.42)**	**14.23 (2.41, 130.01)**
CER10mg/kgIV	0.54 (0.10, 2.32)	1.73 (0.62, 6.04)	1.41 (0.51, 5.12)	1.60 (0.42, 7.29)	2.10 (0.58, 9.01)	0.73 (0.14, 4.05)	1.36 (0.47, 4.73)	1.55 (0.54, 5.26)	2.57 (0.91, 8.88)	2.15 (0.77, 7.44)	0.58 (0.04, 4.94)	1.59 (0.41, 6.42)	2.01 (0.56, 7.82)	1.20 (0.19, 7.16)	2.50 (0.70, 9.71)	2.04 (0.53, 8.63)	2.40 (0.57, 12.96)	**4.14 (1.21, 16.36)**	**5.30 (1.51, 19.88)**	**5.09 (1.50, 19.40)**	**11.74 (1.56, 185.70)**	**11.82 (1.45, 181.31)**	**22.98 (3.42, 323.38)**
CER200mgSC	0.48 (0.09, 1.76)	1.50 (0.88, 2.72)	1.23 (0.47, 3.57)	1.36 (0.48, 4.05)	1.80 (0.71, 5.03)	0.65 (0.13, 2.53)	1.18 (0.62, 2.36)	1.35 (0.70, 2.71)	**2.23 (1.20, 4.36)**	**1.84 (1.01, 3.59)**	0.49 (0.03, 3.89)	1.37 (0.51, 3.78)	1.74 (0.67, 4.98)	1.02 (0.22, 4.61)	2.13 (0.84, 5.68)	1.73 (0.68, 5.05)	2.01 (0.58, 8.76)	**3.58 (1.53, 8.68)**	**4.46 (2.00, 10.51)**	**4.29 (1.91, 10.39)**	**10.13 (1.54, 107.98)**	**10.14 (1.49, 104.10)**	**19.25 (3.20, 195.39)**

(TABLE S12A. continued)

	CER20mg/kgIV	CER400mgSC	CER5mg/kgIV	ELD10mg/kgIV	ELD20mg/kgIV	ETA25mgSC	ETR105mgSC	ETR210mgSC	FIL100mgPO	FIL200mgPO	FON0.1mg/kgIV	FON1mg/kg0.1mgIV/SC	FON1mg/kg1mgIV/SC	FON1mg/kgIV	FON4mg/kg0.1mgIV/SC	FON4mg/kg1mgIV/SC	FON4mg/kgIV	GUS1200mgIV	GUS200mgIV	GUS600mgIV	INF10mg/kgIV	INF20mg/kgIV	INF5mg/kgIV
CER20mg/kgIV	CER20mg/kgIV	**3.16 (1.02, 15.12)**	2.59 (0.90, 11.72)	2.83 (0.72, 17.00)	**3.79 (1.04, 23.04)**	1.34 (0.19, 11.14)	2.48 (0.80, 12.49)	2.82 (0.91, 14.37)	**4.70 (1.50, 22.87)**	**3.89 (1.26, 18.87)**	1.08 (0.06, 12.21)	2.95 (0.62, 16.31)	3.60 (0.94, 21.17)	2.21 (0.35, 15.75)	**4.50 (1.19, 24.88)**	3.73 (0.97, 20.59)	4.46 (0.88, 31.61)	**7.82 (2.14, 35.53)**	**9.63 (2.63, 46.10)**	**9.41 (2.54, 45.12)**	**22.63 (2.46, 353.77)**	**22.42 (2.34, 341.38)**	**44.29 (5.47, 612.65)**
CER400mgSC	**0.32 (0.07, 0.98)**	CER400mgSC	0.80 (0.36, 2.01)	0.90 (0.36, 2.23)	1.19 (0.55, 2.89)	0.42 (0.09, 1.49)	0.78 (0.51, 1.18)	0.89 (0.58, 1.37)	1.47 (0.98, 2.19)	1.22 (0.86, 1.80)	0.31 (0.02, 2.18)	0.89 (0.37, 2.08)	1.13 (0.52, 2.69)	0.67 (0.16, 2.75)	1.40 (0.67, 3.07)	1.15 (0.52, 2.71)	1.31 (0.41, 5.23)	**2.35 (1.21, 4.72)**	**2.95 (1.58, 5.88)**	**2.84 (1.52, 5.77)**	**6.82 (1.05, 65.99)**	**6.46 (1.02, 64.33)**	**12.55 (2.35, 116.28)**
CER5mg/kgIV	0.39 (0.09, 1.11)	1.24 (0.50, 2.81)	CER5mg/kgIV	1.11 (0.35, 3.94)	1.47 (0.44, 4.76)	0.51 (0.09, 2.45)	0.97 (0.36, 2.31)	1.12 (0.41, 2.68)	1.83 (0.68, 4.21)	1.52 (0.56, 3.52)	0.41 (0.02, 3.06)	1.13 (0.27, 3.54)	1.38 (0.45, 4.63)	0.82 (0.15, 4.09)	1.74 (0.55, 5.45)	1.45 (0.40, 4.58)	1.65 (0.41, 7.56)	**2.91 (1.01, 7.95)**	**3.69 (1.29, 10.03)**	**3.56 (1.23, 9.46)**	**8.16 (1.23, 100.80)**	**8.24 (1.15, 94.55)**	**16.49 (2.40, 185.06)**
ELD10mg/kgIV	0.35 (0.06, 1.39)	1.11 (0.45, 2.78)	0.90 (0.25, 2.89)	ELD10mg/kgIV	1.31 (0.63, 3.30)	0.45 (0.08, 2.10)	0.87 (0.33, 2.32)	1.00 (0.38, 2.57)	1.64 (0.62, 4.11)	1.37 (0.52, 3.39)	0.34 (0.02, 2.74)	0.98 (0.30, 3.23)	1.26 (0.38, 4.28)	0.77 (0.13, 3.61)	1.55 (0.50, 5.08)	1.30 (0.38, 4.38)	1.50 (0.32, 6.90)	2.64 (0.85, 8.00)	**3.36 (1.07, 10.06)**	**3.25 (1.01, 9.19)**	7.30 (0.94, 80.95)	7.68 (0.94, 74.04)	**14.58 (2.08, 138.90)**
ELD20mg/kgIV	**0.26 (0.04, 0.97)**	0.84 (0.35, 1.82)	0.68 (0.21, 2.29)	0.76 (0.30, 1.59)	ELD20mg/kgIV	0.34 (0.06, 1.50)	0.65 (0.26, 1.49)	0.75 (0.30, 1.70)	1.24 (0.48, 2.76)	1.03 (0.41, 2.26)	0.27 (0.02, 2.01)	0.76 (0.21, 2.28)	0.95 (0.30, 3.14)	0.56 (0.11, 2.60)	1.17 (0.39, 3.51)	0.97 (0.29, 2.95)	1.10 (0.28, 5.25)	1.99 (0.67, 5.80)	2.47 (0.85, 7.06)	2.39 (0.80, 6.74)	5.33 (0.77, 57.45)	5.55 (0.71, 56.12)	**10.97 (1.63, 102.18)**
ETA25mgSC	0.75 (0.09, 5.31)	2.39 (0.67, 11.41)	1.97 (0.41, 11.39)	2.21 (0.48, 12.40)	2.92 (0.66, 16.46)	ETA25mgSC	1.87 (0.50, 8.95)	2.13 (0.58, 9.97)	3.50 (0.97, 17.01)	2.93 (0.82, 14.08)	0.77 (0.04, 9.07)	2.16 (0.50, 11.41)	2.65 (0.67, 13.89)	1.67 (0.24, 11.88)	3.41 (0.85, 16.43)	2.77 (0.67, 15.63)	3.27 (0.62, 22.81)	**5.72 (1.37, 31.54)**	**7.16 (1.73, 38.25)**	**6.89 (1.64, 37.45)**	**17.36 (1.82, 206.98)**	**17.31 (1.82, 195.52)**	**33.47 (3.85, 389.86)**
ETR105mgSC	0.40 (0.08, 1.26)	1.29 (0.85, 1.98)	1.03 (0.43, 2.78)	1.15 (0.43, 3.01)	1.54 (0.67, 3.85)	0.53 (0.11, 2.02)	ETR105mgSC	1.15 (0.82, 1.60)	**1.90 (1.15, 3.07)**	1.58 (0.98, 2.53)	0.41 (0.03, 2.87)	1.16 (0.45, 2.82)	1.45 (0.63, 3.63)	0.87 (0.19, 3.63)	1.80 (0.82, 4.13)	1.49 (0.62, 3.60)	1.71 (0.48, 7.04)	**3.04 (1.49, 6.58)**	**3.81 (1.88, 8.21)**	**3.69 (1.84, 7.85)**	**8.61 (1.35, 88.05)**	**8.37 (1.24, 81.56)**	**16.08 (2.98, 151.44)**
ETR210mgSC	0.36 (0.07, 1.10)	1.12 (0.73, 1.71)	0.90 (0.37, 2.43)	1.00 (0.39, 2.65)	1.34 (0.59, 3.38)	0.47 (0.10, 1.72)	0.87 (0.62, 1.22)	ETR210mgSC	1.65 (1.00, 2.71)	1.37 (0.86, 2.21)	0.35 (0.02, 2.48)	1.01 (0.39, 2.46)	1.26 (0.56, 3.08)	0.76 (0.17, 3.07)	1.58 (0.72, 3.62)	1.29 (0.55, 3.13)	1.49 (0.43, 5.98)	**2.66 (1.30, 5.79)**	**3.31 (1.63, 7.11)**	**3.21 (1.59, 6.88)**	**7.50 (1.18, 74.79)**	**7.31 (1.09, 70.04)**	**14.01 (2.58, 130.03)**
FIL100mgPO	**0.21 (0.04, 0.67)**	0.68 (0.46, 1.02)	0.55 (0.24, 1.48)	0.61 (0.24, 1.61)	0.80 (0.36, 2.07)	0.29 (0.06, 1.03)	**0.53 (0.33, 0.87)**	0.60 (0.37, 1.00)	FIL100mgPO	0.83 (0.62, 1.12)	0.22 (0.01, 1.47)	0.61 (0.24, 1.46)	0.77 (0.35, 1.91)	0.45 (0.10, 1.92)	0.95 (0.44, 2.17)	0.78 (0.33, 1.90)	0.90 (0.26, 3.56)	1.59 (0.78, 3.55)	**2.00 (1.02, 4.33)**	1.93 (0.98, 4.04)	4.55 (0.73, 43.09)	4.47 (0.69, 41.26)	**8.48 (1.56, 78.91)**
FIL200mgPO	**0.26 (0.05, 0.79)**	0.82 (0.56, 1.17)	0.66 (0.28, 1.79)	0.73 (0.30, 1.94)	0.97 (0.44, 2.44)	0.34 (0.07, 1.22)	0.63 (0.39, 1.02)	0.73 (0.45, 1.17)	1.20 (0.89, 1.60)	FIL200mgPO	0.26 (0.02, 1.80)	0.73 (0.30, 1.75)	0.91 (0.42, 2.29)	0.55 (0.13, 2.27)	1.13 (0.54, 2.59)	0.94 (0.40, 2.24)	1.09 (0.32, 4.25)	1.92 (0.96, 4.16)	**2.39 (1.24, 4.96)**	**2.32 (1.21, 4.81)**	5.52 (0.88, 54.67)	5.35 (0.82, 50.69)	**10.28 (1.88, 95.46)**
FON0.1mg/kgIV	0.92 (0.08, 17.78)	3.18 (0.46, 45.75)	2.46 (0.33, 41.80)	2.93 (0.37, 43.60)	3.76 (0.50, 64.05)	1.30 (0.11, 22.87)	2.47 (0.35, 36.51)	2.82 (0.40, 41.36)	4.59 (0.68, 67.28)	3.89 (0.56, 55.89)	FON0.1mg/kgIV	2.70 (0.38, 45.83)	3.60 (0.48, 59.73)	1.92 (0.30, 35.29)	4.38 (0.57, 72.81)	3.60 (0.49, 56.74)	4.06 (0.78, 64.97)	7.22 (0.99, 116.94)	**9.13 (1.24, 146.14)**	**8.79 (1.24, 141.25)**	**23.22 (1.38, 813.93)**	**23.21 (1.19, 855.84)**	**43.77 (2.63, 1699.27)**
FON1mg/kg0.1mgIV/SC	0.34 (0.06, 1.62)	1.12 (0.48, 2.73)	0.89 (0.28, 3.69)	1.02 (0.31, 3.38)	1.32 (0.44, 4.75)	0.46 (0.09, 1.99)	0.86 (0.35, 2.20)	0.99 (0.41, 2.53)	1.65 (0.68, 4.18)	1.38 (0.57, 3.36)	0.37 (0.02, 2.66)	FON1mg/kg0.1mgIV/SC	1.27 (0.63, 2.88)	0.78 (0.15, 3.53)	1.58 (0.80, 3.38)	1.29 (0.59, 2.94)	1.51 (0.33, 6.54)	2.60 (0.94, 9.03)	**3.25 (1.21, 10.62)**	**3.14 (1.16, 10.42)**	7.78 (0.93, 82.62)	7.71 (0.93, 76.11)	**14.76 (2.11, 150.68)**
FON1mg/kg1mgIV/SC	0.28 (0.05, 1.07)	0.89 (0.37, 1.92)	0.72 (0.22, 2.21)	0.79 (0.23, 2.60)	1.06 (0.32, 3.32)	0.38 (0.07, 1.48)	0.69 (0.28, 1.58)	0.79 (0.32, 1.79)	1.31 (0.52, 2.86)	1.10 (0.44, 2.36)	0.28 (0.02, 2.10)	0.79 (0.35, 1.60)	FON1mg/kg1mgIV/SC	0.59 (0.11, 2.79)	1.23 (0.65, 2.37)	1.01 (0.46, 2.14)	1.14 (0.32, 5.31)	2.07 (0.73, 5.66)	2.61 (0.95, 6.96)	2.51 (0.90, 6.77)	5.83 (0.78, 62.66)	5.78 (0.75, 59.04)	**11.15 (1.77, 111.70)**
FON1mg/kgIV	0.45 (0.06, 2.88)	1.49 (0.36, 6.30)	1.22 (0.24, 6.79)	1.30 (0.28, 7.51)	1.80 (0.38, 9.51)	0.60 (0.08, 4.22)	1.14 (0.28, 5.34)	1.32 (0.33, 5.88)	2.20 (0.52, 10.05)	1.82 (0.44, 7.99)	0.52 (0.03, 3.29)	1.28 (0.28, 6.74)	1.69 (0.36, 8.80)	FON1mg/kgIV	2.14 (0.46, 11.02)	1.73 (0.37, 8.82)	2.02 (0.60, 8.14)	3.52 (0.82, 17.33)	4.46 (0.99, 21.94)	4.29 (0.97, 21.05)	**10.16 (1.03, 144.98)**	10.95 (0.94, 139.26)	**20.96 (2.11, 267.04)**
FON4mg/kg0.1mgIV/SC	**0.22 (0.04, 0.84)**	0.71 (0.33, 1.50)	0.58 (0.18, 1.82)	0.65 (0.20, 2.01)	0.86 (0.28, 2.59)	0.29 (0.06, 1.17)	0.56 (0.24, 1.22)	0.63 (0.28, 1.40)	1.05 (0.46, 2.28)	0.88 (0.39, 1.86)	0.23 (0.01, 1.76)	0.63 (0.30, 1.25)	0.81 (0.42, 1.53)	0.47 (0.09, 2.17)	FON4mg/kg0.1mgIV/SC	0.83 (0.39, 1.58)	0.93 (0.23, 4.10)	1.68 (0.64, 4.50)	2.07 (0.82, 5.50)	2.02 (0.80, 5.38)	4.65 (0.68, 52.91)	4.78 (0.62, 51.26)	**9.01 (1.44, 95.01)**
FON4mg/kg1mgIV/SC	0.27 (0.05, 1.04)	0.87 (0.37, 1.93)	0.69 (0.22, 2.48)	0.77 (0.23, 2.61)	1.03 (0.34, 3.48)	0.36 (0.06, 1.49)	0.67 (0.28, 1.60)	0.78 (0.32, 1.83)	1.28 (0.53, 3.02)	1.06 (0.45, 2.51)	0.28 (0.02, 2.04)	0.78 (0.34, 1.70)	0.99 (0.47, 2.15)	0.58 (0.11, 2.74)	1.20 (0.63, 2.57)	FON4mg/kg1mgIV/SC	1.14 (0.30, 5.25)	2.01 (0.76, 5.92)	2.54 (0.95, 7.08)	2.45 (0.91, 6.78)	5.58 (0.82, 68.17)	5.57 (0.75, 64.54)	**10.88 (1.70, 127.42)**
FON4mg/kgIV	0.22 (0.03, 1.14)	0.76 (0.19, 2.43)	0.61 (0.13, 2.46)	0.67 (0.15, 3.13)	0.91 (0.19, 3.59)	0.31 (0.04, 1.62)	0.58 (0.14, 2.07)	0.67 (0.17, 2.32)	1.11 (0.28, 3.79)	0.91 (0.24, 3.16)	0.25 (0.02, 1.27)	0.66 (0.15, 3.01)	0.87 (0.19, 3.12)	0.49 (0.12, 1.68)	1.07 (0.24, 4.43)	0.88 (0.19, 3.36)	FON4mg/kgIV	1.75 (0.43, 7.72)	2.20 (0.52, 9.54)	2.13 (0.51, 9.32)	5.08 (0.54, 80.72)	4.99 (0.44, 74.31)	**9.58 (1.02, 146.45)**
GUS1200mgIV	**0.13 (0.03, 0.47)**	**0.43 (0.21, 0.82)**	**0.34 (0.13, 0.99)**	0.38 (0.12, 1.17)	0.50 (0.17, 1.50)	**0.17 (0.03, 0.73)**	**0.33 (0.15, 0.67)**	**0.38 (0.17, 0.77)**	0.63 (0.28, 1.27)	0.52 (0.24, 1.05)	0.14 (0.01, 1.01)	0.38 (0.11, 1.07)	0.48 (0.18, 1.36)	0.28 (0.06, 1.23)	0.59 (0.22, 1.57)	0.50 (0.17, 1.32)	0.57 (0.13, 2.35)	GUS1200mgIV	1.25 (0.87, 1.84)	1.21 (0.81, 1.80)	2.71 (0.40, 30.62)	2.79 (0.37, 28.56)	5.44 (0.87, 55.44)
GUS200mgIV	**0.10 (0.02, 0.38)**	**0.34 (0.17, 0.63)**	**0.27 (0.10, 0.78)**	**0.30 (0.10, 0.93)**	0.41 (0.14, 1.18)	**0.14 (0.03, 0.58)**	**0.26 (0.12, 0.53)**	**0.30 (0.14, 0.61)**	**0.50 (0.23, 0.98)**	**0.42 (0.20, 0.81)**	**0.11 (0.01, 0.80)**	**0.31 (0.09, 0.82)**	0.38 (0.14, 1.06)	0.22 (0.05, 1.01)	0.48 (0.18, 1.22)	0.39 (0.14, 1.06)	0.45 (0.10, 1.91)	0.80 (0.54, 1.15)	GUS200mgIV	0.97 (0.67, 1.37)	2.22 (0.32, 23.77)	2.26 (0.31, 22.33)	4.43 (0.71, 42.79)
GUS600mgIV	**0.11 (0.02, 0.39)**	**0.35 (0.17, 0.66)**	**0.28 (0.11, 0.81)**	**0.31 (0.11, 0.99)**	0.42 (0.15, 1.25)	**0.15 (0.03, 0.61)**	**0.27 (0.13, 0.54)**	**0.31 (0.15, 0.63)**	0.52 (0.25, 1.02)	**0.43 (0.21, 0.82)**	**0.11 (0.01, 0.81)**	**0.32 (0.10, 0.86)**	0.40 (0.15, 1.11)	0.23 (0.05, 1.04)	0.50 (0.19, 1.25)	0.41 (0.15, 1.09)	0.47 (0.11, 1.95)	0.83 (0.56, 1.23)	1.04 (0.73, 1.49)	GUS600mgIV	2.28 (0.33, 25.55)	2.33 (0.32, 24.42)	4.52 (0.74, 46.37)
INF10mg/kgIV	**0.04 (0.00, 0.41)**	**0.15 (0.02, 0.96)**	**0.12 (0.01, 0.81)**	0.14 (0.01, 1.06)	0.19 (0.02, 1.30)	**0.06 (0.00, 0.55)**	**0.12 (0.01, 0.74)**	**0.13 (0.01, 0.85)**	0.22 (0.02, 1.37)	0.18 (0.02, 1.13)	**0.04 (0.00, 0.72)**	0.13 (0.01, 1.07)	0.17 (0.02, 1.29)	**0.10 (0.01, 0.97)**	0.21 (0.02, 1.48)	0.18 (0.01, 1.22)	0.20 (0.01, 1.87)	0.37 (0.03, 2.50)	0.45 (0.04, 3.15)	0.44 (0.04, 2.99)	INF10mg/kgIV	0.98 (0.40, 2.45)	1.92 (0.94, 4.34)
INF20mg/kgIV	**0.04 (0.00, 0.43)**	**0.15 (0.02, 0.98)**	**0.12 (0.01, 0.87)**	0.13 (0.01, 1.06)	0.18 (0.02, 1.42)	**0.06 (0.01, 0.55)**	**0.12 (0.01, 0.81)**	**0.14 (0.01, 0.91)**	0.22 (0.02, 1.46)	0.19 (0.02, 1.22)	**0.04 (0.00, 0.84)**	0.13 (0.01, 1.08)	0.17 (0.02, 1.33)	0.09 (0.01, 1.06)	0.21 (0.02, 1.61)	0.18 (0.02, 1.33)	0.20 (0.01, 2.28)	0.36 (0.04, 2.70)	0.44 (0.04, 3.28)	0.43 (0.04, 3.14)	1.02 (0.41, 2.52)	INF20mg/kgIV	1.94 (0.94, 4.54)
INF5mg/kgIV	**0.02 (0.00, 0.18)**	**0.08 (0.01, 0.43)**	**0.06 (0.01, 0.42)**	**0.07 (0.01, 0.48)**	**0.09 (0.01, 0.61)**	**0.03 (0.00, 0.26)**	**0.06 (0.01, 0.34)**	**0.07 (0.01, 0.39)**	**0.12 (0.01, 0.64)**	**0.10 (0.01, 0.53)**	**0.02 (0.00, 0.38)**	**0.07 (0.01, 0.47)**	**0.09 (0.01, 0.56)**	**0.05 (0.00, 0.47)**	**0.11 (0.01, 0.69)**	**0.09 (0.01, 0.59)**	**0.10 (0.01, 0.98)**	0.18 (0.02, 1.15)	0.23 (0.02, 1.40)	0.22 (0.02, 1.36)	0.52 (0.23, 1.07)	0.51 (0.22, 1.07)	INF5mg/kgIV

(TABLE S12A. continued)

	CER20mg/kgIV	CER400mgSC	CER5mg/kgIV	ELD10mg/kgIV	ELD20mg/kgIV	ETA25mgSC	ETR105mgSC	ETR210mgSC	FIL100mgPO	FIL200mgPO	FON0.1mg/kgIV	FON1mg/kg0.1mgIV/SC	FON1mg/kg1mgIV/SC	FON1mg/kgIV	FON4mg/kg0.1mgIV/SC	FON4mg/kg1mgIV/SC	FON4mg/kgIV	GUS1200mgIV	GUS200mgIV	GUS600mgIV	INF10mg/kgIV	INF20mg/kgIV	INF5mg/kgIV
MED700mgIV	0.24 (0.04, 1.03)	0.80 (0.31, 1.84)	0.63 (0.18, 2.22)	0.71 (0.21, 2.37)	0.94 (0.30, 3.02)	0.33 (0.06, 1.49)	0.61 (0.23, 1.47)	0.71 (0.26, 1.72)	1.18 (0.43, 2.85)	0.99 (0.37, 2.31)	0.25 (0.02, 1.93)	0.71 (0.20, 2.27)	0.89 (0.29, 2.88)	0.53 (0.08, 2.65)	1.13 (0.35, 3.49)	0.91 (0.27, 3.01)	1.07 (0.24, 4.87)	1.84 (0.61, 5.49)	2.33 (0.75, 6.82)	2.25 (0.74, 6.57)	5.29 (0.69, 63.41)	5.26 (0.64, 56.21)	**10.08 (1.46, 106.64)**
MIR1000mgIV	**0.13 (0.02, 0.54)**	**0.42 (0.17, 0.96)**	0.34 (0.10, 1.15)	0.38 (0.10, 1.24)	0.50 (0.16, 1.55)	**0.17 (0.03, 0.82)**	**0.32 (0.12, 0.81)**	**0.37 (0.14, 0.91)**	0.62 (0.24, 1.47)	0.51 (0.19, 1.23)	0.12 (0.01, 1.07)	0.36 (0.12, 1.22)	0.47 (0.16, 1.51)	0.27 (0.05, 1.39)	0.58 (0.19, 1.82)	0.48 (0.15, 1.51)	0.55 (0.13, 2.51)	0.99 (0.34, 2.83)	1.24 (0.41, 3.37)	1.20 (0.40, 3.33)	2.74 (0.37, 33.45)	2.75 (0.35, 31.54)	5.20 (0.80, 60.53)
MIR200mgIV	0.23 (0.04, 1.22)	0.76 (0.24, 2.63)	0.60 (0.16, 2.93)	0.68 (0.17, 3.04)	0.93 (0.25, 3.70)	0.31 (0.05, 1.94)	0.58 (0.18, 2.16)	0.68 (0.21, 2.44)	1.13 (0.35, 4.29)	0.93 (0.29, 3.67)	0.25 (0.01, 2.11)	0.67 (0.18, 3.01)	0.86 (0.24, 3.55)	0.51 (0.08, 3.15)	1.08 (0.30, 4.80)	0.89 (0.23, 3.72)	1.01 (0.21, 5.92)	1.80 (0.51, 7.96)	2.30 (0.63, 9.41)	2.23 (0.61, 9.31)	5.22 (0.57, 80.08)	5.14 (0.52, 86.54)	**9.68 (1.21, 160.73)**
MIR600mgIV	**0.09 (0.02, 0.35)**	**0.27 (0.11, 0.65)**	**0.22 (0.07, 0.75)**	**0.26 (0.06, 0.84)**	0.34 (0.10, 1.06)	**0.11 (0.02, 0.55)**	**0.21 (0.08, 0.53)**	**0.25 (0.09, 0.63)**	**0.41 (0.16, 0.98)**	**0.34 (0.13, 0.84)**	**0.08 (0.01, 0.71)**	**0.24 (0.08, 0.82)**	0.31 (0.10, 1.05)	**0.18 (0.03, 0.96)**	0.39 (0.13, 1.19)	0.32 (0.10, 1.01)	0.37 (0.08, 1.72)	0.66 (0.22, 1.86)	0.83 (0.28, 2.31)	0.81 (0.27, 2.26)	1.83 (0.22, 21.85)	1.83 (0.23, 20.48)	3.50 (0.50, 37.76)
NAT300mgIV	**0.28 (0.06, 0.84)**	0.88 (0.65, 1.18)	0.70 (0.31, 1.87)	0.79 (0.32, 1.99)	1.04 (0.48, 2.50)	0.37 (0.08, 1.30)	0.69 (0.45, 1.03)	0.78 (0.51, 1.18)	1.30 (0.89, 1.90)	1.07 (0.75, 1.54)	0.27 (0.02, 1.89)	0.79 (0.33, 1.80)	0.99 (0.46, 2.36)	0.59 (0.14, 2.39)	1.22 (0.60, 2.73)	1.01 (0.45, 2.37)	1.17 (0.36, 4.59)	**2.06 (1.08, 4.28)**	**2.59 (1.40, 5.17)**	**2.51 (1.35, 4.99)**	5.86 (0.94, 57.81)	5.76 (0.90, 56.23)	**10.93 (2.09, 101.71)**
NAT3mg/kgIV	0.37 (0.07, 1.26)	1.17 (0.63, 2.24)	0.95 (0.33, 2.75)	1.06 (0.35, 3.04)	1.41 (0.53, 3.93)	0.49 (0.09, 1.98)	0.91 (0.47, 1.87)	1.04 (0.52, 2.14)	1.72 (0.90, 3.44)	1.42 (0.76, 2.85)	0.37 (0.03, 2.69)	1.06 (0.35, 2.85)	1.33 (0.52, 3.87)	0.78 (0.18, 3.58)	1.65 (0.65, 4.34)	1.34 (0.51, 3.80)	1.58 (0.43, 6.93)	**2.75 (1.20, 6.68)**	**3.44 (1.53, 8.19)**	**3.37 (1.49, 7.80)**	**7.76 (1.20, 79.01)**	**7.86 (1.17, 74.39)**	**14.86 (2.53, 141.81)**
NAT3mg/kgIVx2	**0.24 (0.05, 0.77)**	0.77 (0.44, 1.36)	0.63 (0.23, 1.77)	0.69 (0.24, 1.88)	0.92 (0.36, 2.49)	0.32 (0.06, 1.23)	0.60 (0.31, 1.14)	0.69 (0.36, 1.31)	1.14 (0.60, 2.06)	0.94 (0.51, 1.68)	0.25 (0.02, 1.83)	0.69 (0.25, 1.82)	0.88 (0.35, 2.36)	0.52 (0.11, 2.23)	1.08 (0.44, 2.78)	0.89 (0.34, 2.39)	1.03 (0.29, 4.33)	1.82 (0.82, 4.13)	**2.28 (1.05, 5.11)**	**2.22 (1.01, 4.89)**	5.06 (0.74, 49.92)	5.10 (0.72, 47.53)	**9.89 (1.70, 90.23)**
NAT6mg/kgIVx2	**0.26 (0.05, 0.89)**	0.84 (0.45, 1.50)	0.67 (0.25, 1.97)	0.75 (0.26, 2.08)	1.00 (0.39, 2.76)	0.35 (0.07, 1.36)	0.65 (0.33, 1.25)	0.75 (0.37, 1.44)	1.24 (0.64, 2.30)	1.02 (0.54, 1.90)	0.27 (0.02, 2.03)	0.75 (0.26, 2.03)	0.97 (0.37, 2.54)	0.57 (0.12, 2.54)	1.18 (0.47, 3.10)	0.97 (0.37, 2.73)	1.12 (0.31, 4.97)	1.96 (0.89, 4.73)	**2.48 (1.12, 5.78)**	**2.39 (1.08, 5.58)**	5.46 (0.81, 54.81)	5.43 (0.79, 50.87)	**10.64 (1.83, 106.06)**
NNC2mg/kgSC	**0.23 (0.04, 0.94)**	0.75 (0.28, 1.91)	0.60 (0.17, 2.21)	0.69 (0.18, 2.27)	0.90 (0.28, 3.00)	0.31 (0.06, 1.45)	0.58 (0.20, 1.52)	0.67 (0.24, 1.76)	1.09 (0.40, 2.81)	0.91 (0.33, 2.36)	0.24 (0.01, 1.88)	0.66 (0.19, 2.21)	0.85 (0.25, 2.76)	0.50 (0.08, 2.65)	1.04 (0.31, 3.34)	0.85 (0.25, 2.77)	0.97 (0.22, 4.98)	1.75 (0.56, 5.60)	2.22 (0.71, 7.08)	2.12 (0.67, 6.56)	4.82 (0.63, 64.25)	5.05 (0.57, 53.49)	**9.92 (1.34, 102.38)**
ONE10mgSC	0.30 (0.05, 1.11)	0.97 (0.42, 2.11)	0.79 (0.27, 2.54)	0.89 (0.27, 2.79)	1.19 (0.38, 3.48)	0.40 (0.08, 1.84)	0.76 (0.32, 1.70)	0.87 (0.36, 1.97)	1.44 (0.63, 3.23)	1.19 (0.54, 2.66)	0.32 (0.02, 2.37)	0.85 (0.29, 2.64)	1.11 (0.38, 3.45)	0.66 (0.13, 3.03)	1.38 (0.49, 3.95)	1.13 (0.38, 3.25)	1.32 (0.32, 5.94)	2.27 (0.88, 6.62)	**2.83 (1.09, 8.02)**	**2.73 (1.09, 7.69)**	**6.33 (1.01, 72.67)**	6.30 (0.91, 73.22)	**12.07 (2.09, 132.28)**
ONE25mgSC	0.65 (0.10, 2.57)	2.10 (0.82, 5.48)	1.71 (0.49, 5.88)	1.89 (0.53, 7.23)	2.53 (0.79, 8.75)	0.84 (0.16, 4.34)	1.62 (0.61, 4.55)	1.88 (0.73, 5.14)	**3.07 (1.19, 8.40)**	2.55 (1.00, 6.84)	0.68 (0.04, 6.28)	1.82 (0.55, 6.89)	2.37 (0.75, 8.47)	1.39 (0.23, 7.50)	2.91 (0.90, 9.92)	2.41 (0.71, 8.53)	2.79 (0.64, 13.62)	**4.94 (1.64, 16.44)**	**6.18 (2.09, 19.71)**	**5.98 (2.03, 19.36)**	**13.63 (1.93, 179.32)**	**13.68 (2.05, 180.61)**	**27.26 (4.25, 332.23)**
ONE35mgSC	0.30 (0.05, 1.16)	0.96 (0.40, 2.17)	0.78 (0.23, 2.73)	0.87 (0.24, 2.81)	1.16 (0.35, 3.52)	0.39 (0.07, 1.70)	0.74 (0.31, 1.76)	0.86 (0.35, 2.02)	1.42 (0.58, 3.26)	1.18 (0.50, 2.69)	0.31 (0.02, 2.50)	0.86 (0.26, 2.56)	1.08 (0.36, 3.23)	0.63 (0.12, 3.08)	1.36 (0.45, 3.98)	1.10 (0.35, 3.32)	1.29 (0.31, 5.92)	2.24 (0.83, 6.47)	**2.79 (1.07, 8.01)**	**2.70 (1.05, 7.71)**	6.22 (0.92, 73.49)	5.99 (0.93, 71.33)	**11.90 (2.06, 135.24)**
ONE50mgSC	0.44 (0.07, 1.71)	1.42 (0.54, 3.24)	1.15 (0.34, 3.43)	1.26 (0.35, 4.25)	1.70 (0.50, 5.31)	0.57 (0.12, 2.68)	1.10 (0.42, 2.64)	1.25 (0.48, 3.02)	2.05 (0.82, 4.97)	1.73 (0.69, 4.02)	0.45 (0.03, 3.54)	1.24 (0.37, 3.92)	1.58 (0.52, 4.89)	0.94 (0.17, 4.43)	1.97 (0.65, 5.81)	1.62 (0.48, 5.01)	1.89 (0.43, 8.88)	**3.28 (1.14, 9.58)**	**4.15 (1.41, 11.48)**	**3.97 (1.40, 11.10)**	**9.63 (1.29, 111.65)**	**9.30 (1.26, 105.77)**	**18.06 (2.86, 200.73)**
ONT22_5mgSC	0.33 (0.06, 1.20)	1.06 (0.53, 2.18)	0.88 (0.31, 2.74)	0.99 (0.31, 2.90)	1.27 (0.46, 3.68)	0.44 (0.09, 1.80)	0.82 (0.39, 1.78)	0.94 (0.45, 2.04)	1.56 (0.75, 3.26)	1.30 (0.64, 2.70)	0.34 (0.02, 2.41)	0.96 (0.31, 2.88)	1.21 (0.44, 3.44)	0.70 (0.14, 3.47)	1.50 (0.55, 4.15)	1.24 (0.44, 3.43)	1.42 (0.40, 5.87)	**2.57 (1.02, 6.43)**	**3.17 (1.27, 8.28)**	**3.09 (1.25, 7.69)**	**7.15 (1.03, 76.57)**	7.08 (0.99, 70.69)	**14.06 (2.22, 129.79)**
ONT225mgSC	0.31 (0.06, 1.09)	0.97 (0.51, 1.95)	0.79 (0.28, 2.46)	0.89 (0.30, 2.70)	1.17 (0.43, 3.38)	0.41 (0.08, 1.61)	0.76 (0.37, 1.59)	0.87 (0.43, 1.82)	1.43 (0.72, 3.00)	1.19 (0.61, 2.46)	0.31 (0.02, 2.32)	0.88 (0.29, 2.44)	1.12 (0.42, 3.03)	0.65 (0.13, 3.03)	1.38 (0.51, 3.62)	1.13 (0.40, 3.10)	1.31 (0.35, 5.54)	2.30 (0.96, 5.72)	**2.92 (1.22, 7.05)**	**2.84 (1.16, 6.71)**	6.40 (0.96, 73.32)	6.44 (0.93, 67.86)	**12.30 (2.06, 131.58)**
ONT75mgSC	0.32 (0.06, 1.19)	1.04 (0.52, 2.16)	0.86 (0.29, 2.69)	0.96 (0.30, 2.80)	1.26 (0.43, 3.65)	0.43 (0.08, 1.75)	0.81 (0.39, 1.72)	0.93 (0.43, 1.99)	1.54 (0.71, 3.26)	1.28 (0.62, 2.73)	0.33 (0.02, 2.50)	0.94 (0.32, 2.71)	1.19 (0.42, 3.36)	0.69 (0.14, 3.36)	1.46 (0.54, 4.13)	1.19 (0.45, 3.58)	1.35 (0.36, 6.28)	2.50 (0.98, 6.49)	**3.13 (1.27, 7.82)**	**3.04 (1.17, 7.52)**	6.90 (0.97, 79.36)	6.75 (0.95, 73.14)	**13.71 (2.08, 133.54)**
PBO	0.38 (0.08, 1.15)	1.22 (0.99, 1.53)	0.98 (0.44, 2.58)	1.10 (0.45, 2.72)	1.45 (0.70, 3.44)	0.51 (0.11, 1.80)	0.95 (0.66, 1.37)	1.09 (0.76, 1.58)	**1.80 (1.31, 2.51)**	**1.49 (1.13, 2.03)**	0.39 (0.03, 2.61)	1.10 (0.46, 2.47)	1.37 (0.67, 3.22)	0.83 (0.20, 3.28)	1.70 (0.85, 3.65)	1.41 (0.64, 3.20)	1.63 (0.51, 6.19)	**2.86 (1.56, 5.83)**	**3.60 (2.01, 7.02)**	**3.47 (1.94, 6.83)**	**8.30 (1.33, 79.36)**	**7.98 (1.26, 77.48)**	**15.30 (2.90, 138.05)**
PF10mgSC	0.39 (0.06, 2.23)	1.34 (0.34, 4.53)	1.10 (0.20, 5.08)	1.22 (0.22, 5.10)	1.66 (0.31, 6.58)	0.55 (0.05, 3.40)	1.05 (0.26, 3.80)	1.19 (0.29, 4.19)	1.98 (0.49, 6.98)	1.65 (0.41, 5.69)	0.38 (0.03, 4.39)	1.18 (0.26, 4.99)	1.50 (0.33, 6.58)	0.90 (0.15, 5.50)	1.87 (0.46, 7.92)	1.55 (0.30, 6.24)	1.77 (0.39, 10.24)	3.17 (0.74, 13.90)	4.00 (0.92, 16.62)	3.86 (0.90, 15.66)	9.24 (0.84, 115.89)	9.36 (0.77, 113.03)	**17.87 (1.95, 224.83)**
PF200mgSC	0.42 (0.06, 2.74)	1.34 (0.38, 6.33)	1.12 (0.25, 6.90)	1.21 (0.26, 6.99)	1.62 (0.35, 8.59)	0.55 (0.07, 4.68)	1.03 (0.30, 5.24)	1.19 (0.33, 5.86)	1.95 (0.58, 9.60)	1.64 (0.49, 7.70)	0.41 (0.03, 5.55)	1.19 (0.30, 6.55)	1.50 (0.38, 9.06)	0.90 (0.13, 7.42)	1.89 (0.47, 9.29)	1.59 (0.33, 8.18)	1.84 (0.31, 13.57)	3.17 (0.81, 18.84)	**3.93 (1.06, 22.18)**	**3.83 (1.04, 21.23)**	**9.00 (1.10, 161.08)**	**9.17 (1.03, 151.48)**	**17.68 (2.33, 301.61)**
PF50mgSC	**0.14 (0.02, 0.57)**	0.46 (0.16, 1.04)	0.37 (0.09, 1.30)	0.41 (0.11, 1.38)	0.54 (0.15, 1.72)	**0.19 (0.03, 0.86)**	**0.36 (0.12, 0.86)**	**0.41 (0.14, 0.98)**	0.67 (0.23, 1.62)	0.56 (0.19, 1.31)	0.14 (0.01, 1.22)	0.39 (0.12, 1.33)	0.50 (0.16, 1.61)	0.30 (0.06, 1.58)	0.62 (0.20, 1.92)	0.52 (0.15, 1.61)	0.60 (0.15, 2.81)	1.06 (0.34, 3.37)	1.32 (0.42, 4.03)	1.27 (0.42, 3.79)	2.96 (0.39, 34.26)	2.97 (0.34, 32.18)	5.67 (0.76, 60.21)
RIS1200mgIV	**0.22 (0.05, 0.65)**	**0.69 (0.51, 0.92)**	0.56 (0.24, 1.42)	0.62 (0.26, 1.60)	0.82 (0.38, 2.00)	0.29 (0.06, 1.03)	**0.53 (0.35, 0.82)**	**0.61 (0.40, 0.94)**	1.02 (0.70, 1.49)	0.84 (0.60, 1.20)	0.22 (0.02, 1.51)	0.62 (0.25, 1.42)	0.78 (0.35, 1.85)	0.47 (0.11, 1.87)	0.96 (0.47, 2.13)	0.79 (0.35, 1.87)	0.91 (0.28, 3.52)	1.61 (0.85, 3.27)	**2.01 (1.08, 4.03)**	**1.94 (1.05, 3.97)**	4.64 (0.74, 45.53)	4.51 (0.69, 43.41)	**8.67 (1.58, 81.29)**
RIS200mgIV	0.28 (0.06, 1.10)	0.90 (0.42, 1.99)	0.74 (0.24, 2.31)	0.83 (0.27, 2.54)	1.09 (0.34, 3.34)	0.38 (0.06, 1.68)	0.70 (0.32, 1.61)	0.80 (0.38, 1.86)	1.33 (0.61, 3.00)	1.11 (0.53, 2.49)	0.29 (0.02, 2.22)	0.80 (0.30, 2.52)	1.03 (0.39, 2.99)	0.59 (0.12, 2.90)	1.27 (0.52, 3.47)	1.05 (0.36, 3.07)	1.23 (0.31, 5.45)	2.12 (0.79, 5.98)	**2.68 (1.10, 7.23)**	**2.57 (1.03, 7.02)**	6.52 (0.85, 65.60)	6.49 (0.77, 63.38)	**12.61 (1.79, 114.37)**
RIS600mgIV	**0.20 (0.04, 0.59)**	**0.63 (0.47, 0.85)**	0.51 (0.22, 1.30)	0.57 (0.23, 1.44)	0.75 (0.35, 1.83)	**0.26 (0.06, 0.95)**	**0.49 (0.32, 0.76)**	**0.56 (0.37, 0.86)**	0.93 (0.64, 1.36)	0.77 (0.55, 1.09)	0.20 (0.01, 1.39)	0.57 (0.23, 1.31)	0.72 (0.32, 1.70)	0.43 (0.10, 1.72)	0.88 (0.43, 1.96)	0.73 (0.33, 1.70)	0.84 (0.26, 3.23)	1.48 (0.77, 2.99)	1.85 (1.00, 3.70)	1.79 (0.96, 3.60)	4.22 (0.68, 41.59)	4.14 (0.63, 40.74)	**7.97 (1.45, 73.55)**

	CER20mg/kgIV	CER400mgSC	CER5mg/kgIV	ELD10mg/kgIV	ELD20mg/kgIV	ETA25mgSC	ETR105mgSC	ETR210mgSC	FIL100mgPO	FIL200mgPO	FON0.1mg/kgIV	FON1mg/kg0.1mgIV/SC	FON1mg/kg1mgIV/SC	FON1mg/kgIV	FON4mg/kg0.1mgIV/SC	FON4mg/kg1mgIV/SC	FON4mg/kgIV	GUS1200mgIV	GUS200mgIV	GUS600mgIV	INF10mg/kgIV	INF20mg/kgIV	INF5mg/kgIV
RIS600mgIV4_4	**0.28 (0.06, 0.85)**	0.88 (0.57, 1.33)	0.70 (0.29, 2.01)	0.79 (0.30, 2.08)	1.04 (0.47, 2.62)	0.37 (0.08, 1.35)	0.68 (0.41, 1.12)	0.78 (0.47, 1.29)	1.30 (0.80, 2.07)	1.07 (0.67, 1.70)	0.28 (0.02, 1.87)	0.79 (0.30, 1.92)	0.99 (0.42, 2.52)	0.60 (0.13, 2.41)	1.22 (0.56, 2.90)	1.00 (0.43, 2.56)	1.18 (0.34, 4.51)	2.06 (1.00, 4.49)	**2.58 (1.29, 5.41)**	**2.49 (1.25, 5.22)**	5.73 (0.91, 62.94)	5.64 (0.86, 56.17)	**11.11 (1.97, 103.18)**
SEC10mg/kgIV	0.51 (0.07, 3.90)	1.65 (0.39, 8.16)	1.35 (0.26, 7.38)	1.50 (0.27, 8.17)	1.93 (0.38, 11.27)	0.69 (0.08, 4.59)	1.28 (0.30, 6.46)	1.45 (0.35, 7.59)	2.38 (0.55, 11.99)	2.01 (0.48, 10.21)	0.56 (0.02, 6.23)	1.45 (0.27, 8.00)	1.91 (0.38, 10.10)	1.09 (0.13, 8.13)	2.30 (0.45, 13.68)	1.92 (0.34, 9.90)	2.28 (0.27, 15.76)	3.79 (0.86, 18.11)	**4.82 (1.09, 24.44)**	**4.67 (1.07, 22.61)**	11.50 (0.97, 168.95)	11.72 (0.91, 160.44)	**22.33 (2.01, 300.41)**
TES400mg200mgSC	**0.19 (0.04, 0.65)**	0.61 (0.31, 1.10)	0.49 (0.18, 1.37)	0.54 (0.18, 1.62)	0.72 (0.27, 2.05)	0.25 (0.05, 1.05)	**0.47 (0.22, 0.91)**	0.54 (0.26, 1.05)	0.90 (0.44, 1.72)	0.75 (0.37, 1.39)	0.19 (0.01, 1.46)	0.54 (0.19, 1.48)	0.68 (0.26, 1.89)	0.40 (0.08, 1.86)	0.84 (0.33, 2.17)	0.69 (0.26, 1.89)	0.79 (0.21, 3.57)	1.42 (0.59, 3.55)	1.79 (0.75, 4.49)	1.73 (0.73, 4.19)	4.07 (0.59, 42.63)	4.09 (0.58, 42.26)	**7.84 (1.32, 78.29)**
TOF15mgPO_BID	0.57 (0.08, 2.91)	1.87 (0.56, 6.65)	1.52 (0.34, 6.54)	1.72 (0.32, 7.24)	2.27 (0.46, 9.14)	0.75 (0.12, 4.55)	1.45 (0.42, 5.26)	1.67 (0.48, 5.95)	2.75 (0.80, 9.81)	2.28 (0.68, 8.10)	0.61 (0.03, 5.31)	1.67 (0.39, 7.44)	2.17 (0.49, 9.23)	1.29 (0.19, 7.73)	2.55 (0.61, 11.96)	2.12 (0.49, 9.74)	2.59 (0.41, 15.33)	**4.28 (1.17, 18.72)**	**5.47 (1.47, 22.10)**	**5.29 (1.46, 21.45)**	**12.51 (1.39, 158.36)**	**12.28 (1.45, 157.47)**	**24.64 (3.04, 287.40)**
TOF1mgPO_BID	0.25 (0.04, 1.02)	0.80 (0.28, 2.04)	0.65 (0.18, 2.30)	0.72 (0.20, 2.42)	0.97 (0.24, 3.18)	0.33 (0.06, 1.56)	0.63 (0.21, 1.65)	0.72 (0.25, 1.85)	1.20 (0.40, 2.94)	0.99 (0.36, 2.44)	0.26 (0.02, 1.98)	0.72 (0.22, 2.36)	0.92 (0.24, 2.96)	0.53 (0.09, 2.80)	1.12 (0.35, 3.57)	0.93 (0.23, 2.96)	1.09 (0.20, 5.34)	1.91 (0.66, 5.68)	2.38 (0.83, 6.88)	2.30 (0.76, 6.69)	5.53 (0.68, 60.91)	5.41 (0.60, 57.12)	**10.54 (1.45, 119.60)**
TOF5mgPO_BID	0.32 (0.06, 1.42)	1.05 (0.39, 2.63)	0.83 (0.25, 3.09)	0.96 (0.26, 3.28)	1.27 (0.32, 4.31)	0.44 (0.08, 2.14)	0.83 (0.28, 2.14)	0.95 (0.33, 2.47)	1.56 (0.55, 3.93)	1.29 (0.48, 3.27)	0.33 (0.02, 2.62)	0.93 (0.28, 3.35)	1.19 (0.33, 4.04)	0.70 (0.13, 3.60)	1.47 (0.45, 5.09)	1.19 (0.35, 4.47)	1.42 (0.29, 6.75)	2.47 (0.86, 7.46)	**3.13 (1.09, 9.24)**	2.99 (1.00, 8.97)	7.25 (0.92, 80.94)	7.08 (0.81, 75.80)	**13.76 (1.90, 146.63)**
UPA12mgPO_BID	0.32 (0.04, 1.65)	1.05 (0.29, 3.73)	0.87 (0.18, 3.71)	0.97 (0.18, 4.34)	1.26 (0.27, 5.59)	0.44 (0.06, 2.32)	0.83 (0.21, 2.95)	0.95 (0.24, 3.34)	1.54 (0.41, 5.48)	1.29 (0.34, 4.54)	0.33 (0.02, 2.98)	0.93 (0.22, 3.96)	1.19 (0.29, 5.03)	0.71 (0.10, 4.02)	1.46 (0.35, 5.81)	1.21 (0.29, 5.04)	1.41 (0.25, 7.99)	2.50 (0.57, 9.42)	3.08 (0.73, 11.47)	3.02 (0.72, 10.94)	7.22 (0.71, 106.31)	7.27 (0.72, 93.84)	**14.06 (1.55, 181.27)**
UPA24mgPO	0.56 (0.06, 3.61)	1.83 (0.40, 8.37)	1.48 (0.25, 8.16)	1.65 (0.21, 10.77)	2.17 (0.38, 11.90)	0.75 (0.09, 5.03)	1.42 (0.30, 6.61)	1.62 (0.35, 7.61)	2.70 (0.58, 12.46)	2.25 (0.48, 10.06)	0.54 (0.03, 5.36)	1.62 (0.28, 8.16)	2.00 (0.35, 11.61)	1.18 (0.15, 7.75)	2.55 (0.47, 13.09)	2.07 (0.41, 11.06)	2.35 (0.35, 14.72)	4.27 (0.87, 22.37)	**5.35 (1.10, 26.74)**	**5.17 (1.08, 25.12)**	11.87 (0.96, 179.32)	**12.26 (1.08, 181.37)**	**23.06 (2.24, 335.08)**
UPA24mgPO_BID	**0.18 (0.02, 0.76)**	0.57 (0.18, 1.59)	0.46 (0.11, 1.93)	0.52 (0.11, 2.00)	0.68 (0.17, 2.35)	0.23 (0.03, 1.13)	0.43 (0.13, 1.28)	0.50 (0.15, 1.48)	0.83 (0.26, 2.45)	0.69 (0.21, 2.08)	0.18 (0.01, 1.50)	0.51 (0.13, 1.76)	0.64 (0.16, 2.48)	0.38 (0.06, 1.81)	0.79 (0.21, 2.88)	0.65 (0.17, 2.41)	0.75 (0.16, 3.71)	1.34 (0.36, 4.61)	1.65 (0.44, 5.62)	1.62 (0.44, 5.41)	3.85 (0.43, 53.61)	3.83 (0.45, 49.18)	7.55 (1.00, 90.66)
UPA3mgPO_BID	0.46 (0.07, 3.21)	1.46 (0.40, 6.43)	1.17 (0.25, 6.81)	1.30 (0.23, 6.81)	1.75 (0.38, 8.50)	0.59 (0.08, 4.05)	1.13 (0.30, 4.91)	1.31 (0.35, 5.61)	2.15 (0.57, 9.30)	1.80 (0.47, 7.88)	0.45 (0.03, 4.95)	1.30 (0.30, 6.64)	1.62 (0.40, 8.74)	0.99 (0.15, 5.90)	2.00 (0.49, 10.40)	1.69 (0.40, 8.59)	1.95 (0.38, 12.36)	3.42 (0.87, 16.62)	**4.26 (1.09, 21.04)**	**4.09 (1.05, 20.55)**	9.81 (1.00, 179.29)	**9.68 (1.04, 182.31)**	**18.58 (2.21, 388.20)**
UPA45mgPO	**0.22 (0.05, 0.68)**	**0.70 (0.51, 0.94)**	0.56 (0.24, 1.50)	0.63 (0.25, 1.61)	0.82 (0.38, 2.01)	0.29 (0.06, 1.06)	**0.54 (0.35, 0.83)**	**0.62 (0.40, 0.96)**	1.03 (0.70, 1.53)	0.86 (0.59, 1.24)	0.22 (0.02, 1.48)	0.63 (0.26, 1.43)	0.79 (0.37, 1.88)	0.47 (0.11, 1.87)	0.98 (0.48, 2.16)	0.80 (0.36, 1.83)	0.93 (0.28, 3.65)	1.63 (0.85, 3.43)	**2.06 (1.10, 4.11)**	**1.98 (1.06, 3.98)**	4.73 (0.73, 47.27)	4.57 (0.70, 44.30)	**8.80 (1.62, 81.70)**
UPA6mgPO_BID	**0.15 (0.02, 0.60)**	0.47 (0.15, 1.20)	0.39 (0.09, 1.37)	0.43 (0.08, 1.54)	0.58 (0.13, 1.85)	**0.19 (0.03, 0.91)**	0.36 (0.11, 1.01)	0.42 (0.12, 1.15)	0.70 (0.21, 1.79)	0.58 (0.17, 1.50)	0.14 (0.01, 1.21)	0.42 (0.10, 1.48)	0.55 (0.12, 1.95)	0.31 (0.05, 1.48)	0.66 (0.17, 2.18)	0.53 (0.14, 1.87)	0.61 (0.12, 3.14)	1.11 (0.30, 3.38)	1.39 (0.38, 4.19)	1.34 (0.37, 4.06)	3.12 (0.33, 36.94)	3.14 (0.35, 35.08)	6.28 (0.76, 67.84)
UST130mgIV	**0.23 (0.05, 0.72)**	0.72 (0.50, 1.05)	0.58 (0.25, 1.65)	0.64 (0.25, 1.72)	0.86 (0.39, 2.13)	0.30 (0.06, 1.12)	**0.56 (0.35, 0.89)**	0.65 (0.41, 1.02)	1.07 (0.68, 1.64)	0.89 (0.58, 1.35)	0.23 (0.02, 1.58)	0.65 (0.27, 1.51)	0.82 (0.37, 1.95)	0.49 (0.11, 1.98)	1.02 (0.46, 2.24)	0.84 (0.36, 1.93)	0.96 (0.28, 3.62)	1.70 (0.86, 3.73)	**2.14 (1.08, 4.48)**	**2.08 (1.01, 4.27)**	4.84 (0.76, 49.15)	4.75 (0.73, 45.88)	**9.23 (1.68, 82.95)**
UST1mg/kgIV	**0.20 (0.04, 0.71)**	0.64 (0.36, 1.13)	0.52 (0.20, 1.55)	0.58 (0.19, 1.64)	0.76 (0.28, 2.13)	0.27 (0.05, 1.06)	**0.50 (0.26, 0.92)**	0.57 (0.30, 1.07)	0.94 (0.50, 1.73)	0.78 (0.43, 1.42)	0.20 (0.01, 1.47)	0.58 (0.21, 1.46)	0.71 (0.29, 1.96)	0.43 (0.09, 1.85)	0.90 (0.36, 2.20)	0.74 (0.29, 1.96)	0.84 (0.24, 3.52)	1.51 (0.67, 3.60)	1.89 (0.84, 4.24)	1.84 (0.84, 4.07)	4.38 (0.63, 43.84)	4.35 (0.61, 40.53)	**8.24 (1.35, 76.38)**
UST3mg/kgIV	**0.19 (0.04, 0.69)**	0.62 (0.35, 1.06)	0.49 (0.19, 1.42)	0.54 (0.18, 1.61)	0.72 (0.29, 2.01)	**0.26 (0.05, 0.99)**	**0.48 (0.25, 0.89)**	0.55 (0.29, 1.02)	0.90 (0.49, 1.65)	0.75 (0.41, 1.36)	0.20 (0.01, 1.41)	0.56 (0.21, 1.41)	0.69 (0.28, 1.89)	0.41 (0.08, 1.83)	0.87 (0.35, 2.11)	0.71 (0.28, 1.84)	0.82 (0.24, 3.39)	1.43 (0.66, 3.46)	1.81 (0.83, 4.16)	1.74 (0.79, 3.95)	4.23 (0.61, 41.95)	4.22 (0.61, 38.86)	**8.04 (1.28, 74.24)**
UST4.5mg/kgIV	**0.22 (0.04, 0.68)**	0.72 (0.48, 1.04)	0.58 (0.25, 1.51)	0.65 (0.25, 1.67)	0.85 (0.38, 2.06)	0.30 (0.06, 1.08)	**0.55 (0.34, 0.90)**	0.63 (0.39, 1.04)	1.05 (0.67, 1.64)	0.87 (0.58, 1.33)	0.23 (0.02, 1.60)	0.64 (0.26, 1.53)	0.80 (0.36, 2.01)	0.48 (0.11, 1.95)	0.99 (0.46, 2.33)	0.82 (0.36, 1.98)	0.94 (0.28, 3.86)	1.69 (0.82, 3.49)	**2.12 (1.05, 4.40)**	**2.05 (1.02, 4.25)**	4.75 (0.75, 47.91)	4.65 (0.70, 46.07)	**8.96 (1.61, 87.57)**
UST6mg/kg90mgIV/SC	**0.13 (0.03, 0.48)**	**0.42 (0.21, 0.81)**	**0.34 (0.12, 0.98)**	0.38 (0.12, 1.15)	0.50 (0.18, 1.55)	**0.17 (0.03, 0.73)**	**0.33 (0.15, 0.68)**	**0.38 (0.17, 0.78)**	0.62 (0.29, 1.23)	0.52 (0.25, 1.01)	**0.14 (0.01, 0.98)**	0.38 (0.12, 1.06)	0.48 (0.18, 1.37)	0.28 (0.06, 1.23)	0.59 (0.22, 1.53)	0.49 (0.18, 1.33)	0.57 (0.13, 2.41)	1.00 (0.65, 1.50)	1.25 (0.86, 1.85)	1.21 (0.81, 1.79)	2.82 (0.40, 29.79)	2.86 (0.35, 28.88)	5.51 (0.81, 54.67)
UST6mg/kgIV	**0.17 (0.04, 0.54)**	**0.56 (0.40, 0.79)**	0.45 (0.20, 1.23)	0.50 (0.19, 1.29)	0.67 (0.31, 1.62)	**0.24 (0.05, 0.85)**	**0.43 (0.28, 0.68)**	**0.50 (0.32, 0.78)**	0.82 (0.54, 1.26)	0.68 (0.46, 1.03)	0.18 (0.01, 1.23)	0.50 (0.20, 1.16)	0.63 (0.29, 1.49)	0.38 (0.08, 1.54)	0.79 (0.36, 1.75)	0.65 (0.29, 1.51)	0.74 (0.22, 2.87)	1.31 (0.67, 2.82)	1.65 (0.86, 3.41)	1.60 (0.82, 3.29)	3.77 (0.60, 36.45)	3.71 (0.57, 35.94)	**7.18 (1.29, 64.45)**
UST90mgSC	0.35 (0.05, 1.66)	1.15 (0.39, 3.29)	0.94 (0.24, 3.67)	1.06 (0.25, 3.99)	1.41 (0.37, 5.16)	0.47 (0.09, 2.32)	0.90 (0.28, 2.64)	1.05 (0.31, 2.98)	1.71 (0.53, 4.79)	1.41 (0.45, 3.93)	0.35 (0.02, 3.37)	1.05 (0.24, 3.74)	1.32 (0.36, 4.59)	0.75 (0.12, 4.40)	1.61 (0.44, 5.75)	1.36 (0.36, 4.95)	1.48 (0.35, 8.07)	2.74 (0.77, 8.86)	3.44 (0.99, 10.93)	3.31 (0.98, 10.61)	8.10 (0.84, 90.43)	8.03 (0.80, 84.10)	**15.99 (1.90, 162.62)**
VED0_5mg/kgIV	**0.26 (0.05, 0.96)**	0.87 (0.40, 1.69)	0.69 (0.24, 2.33)	0.76 (0.26, 2.37)	1.01 (0.37, 3.12)	0.35 (0.07, 1.50)	0.67 (0.30, 1.41)	0.77 (0.35, 1.61)	1.28 (0.59, 2.61)	1.06 (0.49, 2.14)	0.27 (0.02, 2.19)	0.77 (0.26, 2.20)	0.99 (0.35, 2.75)	0.59 (0.11, 2.57)	1.21 (0.43, 3.23)	1.00 (0.36, 2.79)	1.16 (0.26, 4.91)	2.02 (0.83, 5.25)	**2.55 (1.04, 6.51)**	2.48 (1.00, 6.12)	5.57 (0.83, 59.70)	5.54 (0.70, 56.18)	**10.77 (1.71, 106.67)**
VED2mg/kgIV	**0.21 (0.04, 0.78)**	0.68 (0.33, 1.29)	0.55 (0.18, 1.69)	0.62 (0.21, 1.87)	0.81 (0.30, 2.46)	0.28 (0.06, 1.13)	0.53 (0.24, 1.08)	0.61 (0.28, 1.24)	1.01 (0.48, 1.98)	0.84 (0.40, 1.64)	0.21 (0.02, 1.70)	0.61 (0.21, 1.70)	0.78 (0.28, 2.18)	0.46 (0.09, 2.02)	0.96 (0.36, 2.48)	0.79 (0.28, 2.10)	0.92 (0.22, 3.74)	1.61 (0.66, 3.96)	2.03 (0.84, 4.78)	1.97 (0.82, 4.63)	4.57 (0.65, 48.01)	4.47 (0.53, 46.34)	**8.95 (1.27, 83.53)**
ED300mgIV	**0.18 (0.04, 0.56)**	**0.58 (0.39, 0.85)**	0.47 (0.20, 1.23)	0.52 (0.20, 1.38)	0.69 (0.31, 1.74)	**0.24 (0.05, 0.90)**	**0.45 (0.27, 0.75)**	**0.52 (0.31, 0.84)**	0.85 (0.53, 1.35)	0.71 (0.46, 1.11)	0.19 (0.01, 1.28)	0.52 (0.20, 1.26)	0.66 (0.30, 1.66)	0.39 (0.09, 1.57)	0.80 (0.38, 1.83)	0.66 (0.29, 1.60)	0.78 (0.25, 2.92)	1.35 (0.69, 2.86)	1.72 (0.87, 3.57)	1.66 (0.84, 3.46)	3.87 (0.59, 39.48)	3.84 (0.58, 37.53)	**7.35 (1.29, 69.69)**

	MED700mgIV	MIR1000mgIV	MIR200mgIV	MIR600mgIV	NAT300mgIV	NAT3mg/kgIV	NAT3mg/kgIVx2	NAT6mg/kgIVx2	NNC2mg/kgSC	ONE10mgSC	ONE25mgSC	ONE35mgSC	ONE50mgSC	ONT22_5mgSC	ONT225mgSC	ONT75mgSC	PBO	PF10mgSC	PF200mgSC	PF50mgSC	RIS1200mgIV	RIS200mgIV	RIS600mgIV
ABA10mg/kgIV	2.63 (0.67, 12.89)	**4.98 (1.43, 20.36)**	2.71 (0.61, 12.90)	**7.35 (2.08, 31.73)**	2.35 (0.84, 7.59)	1.76 (0.56, 6.26)	2.69 (0.83, 9.56)	2.41 (0.78, 8.76)	2.81 (0.74, 12.20)	2.10 (0.60, 8.26)	0.96 (0.27, 4.37)	2.18 (0.60, 8.39)	1.50 (0.42, 6.03)	1.91 (0.59, 6.97)	2.14 (0.66, 7.92)	1.98 (0.59, 7.46)	1.69 (0.61, 5.27)	1.55 (0.33, 8.44)	1.54 (0.24, 8.45)	**4.68 (1.15, 19.64)**	**3.04 (1.06, 9.27)**	2.32 (0.61, 8.70)	**3.28 (1.15, 10.14)**
ABA30mg/kgIV	1.32 (0.32, 5.98)	2.56 (0.66, 10.12)	1.34 (0.29, 6.41)	3.83 (0.94, 15.13)	1.20 (0.41, 3.61)	0.90 (0.28, 3.00)	1.37 (0.41, 4.49)	1.26 (0.38, 4.39)	1.40 (0.36, 5.71)	1.06 (0.29, 4.18)	0.50 (0.12, 2.07)	1.09 (0.30, 4.33)	0.76 (0.19, 3.08)	0.99 (0.29, 3.43)	1.07 (0.31, 3.76)	1.02 (0.30, 3.57)	0.86 (0.30, 2.55)	0.80 (0.17, 3.70)	0.81 (0.11, 3.92)	2.35 (0.58, 9.75)	1.54 (0.52, 4.60)	1.17 (0.31, 4.01)	1.67 (0.57, 5.00)
ABA3mg/kgIV	2.09 (0.60, 9.08)	**3.81 (1.19, 16.22)**	2.13 (0.47, 10.33)	**5.93 (1.47, 25.54)**	1.86 (0.71, 5.75)	1.42 (0.45, 4.80)	2.17 (0.72, 7.22)	2.00 (0.65, 6.62)	2.23 (0.60, 9.30)	1.65 (0.52, 6.40)	0.78 (0.19, 3.41)	1.67 (0.51, 6.75)	1.17 (0.33, 4.88)	1.56 (0.47, 5.25)	1.69 (0.53, 5.79)	1.59 (0.42, 5.79)	1.34 (0.52, 4.00)	1.25 (0.27, 6.26)	1.27 (0.19, 6.16)	3.73 (0.99, 15.78)	2.39 (0.90, 7.41)	1.83 (0.54, 6.32)	2.60 (0.98, 7.92)
ADA160mg80mg40mgSC	0.56 (0.19, 1.80)	1.08 (0.37, 3.24)	0.57 (0.13, 2.07)	1.58 (0.55, 4.76)	0.50 (0.24, 1.02)	**0.38 (0.15, 0.89)**	0.57 (0.24, 1.32)	0.53 (0.22, 1.21)	0.59 (0.18, 1.98)	0.45 (0.15, 1.24)	**0.21 (0.06, 0.64)**	0.47 (0.16, 1.30)	**0.32 (0.11, 0.96)**	0.41 (0.16, 1.05)	0.45 (0.17, 1.15)	0.41 (0.15, 1.13)	**0.36 (0.18, 0.70)**	0.33 (0.08, 1.47)	0.33 (0.06, 1.36)	0.99 (0.32, 3.21)	0.64 (0.31, 1.31)	0.49 (0.17, 1.39)	0.70 (0.33, 1.41)
ADA160mg80mg60mgSC	**0.27 (0.08, 0.88)**	0.50 (0.16, 1.70)	0.28 (0.06, 1.11)	0.75 (0.24, 2.55)	**0.25 (0.10, 0.51)**	**0.18 (0.06, 0.46)**	**0.28 (0.10, 0.65)**	**0.26 (0.09, 0.64)**	**0.29 (0.08, 0.93)**	**0.22 (0.07, 0.64)**	**0.10 (0.03, 0.33)**	**0.22 (0.07, 0.66)**	**0.15 (0.05, 0.49)**	**0.20 (0.07, 0.53)**	**0.22 (0.08, 0.55)**	**0.20 (0.07, 0.56)**	**0.18 (0.07, 0.36)**	**0.16 (0.04, 0.79)**	**0.16 (0.03, 0.66)**	0.47 (0.14, 1.65)	**0.31 (0.12, 0.66)**	**0.24 (0.07, 0.68)**	**0.34 (0.13, 0.73)**
ADA160mg80mgSC	0.57 (0.22, 1.62)	1.08 (0.43, 3.11)	0.60 (0.15, 2.08)	1.66 (0.65, 4.60)	**0.52 (0.31, 0.83)**	**0.39 (0.18, 0.78)**	0.59 (0.30, 1.15)	0.55 (0.27, 1.10)	0.61 (0.22, 1.74)	0.47 (0.20, 1.12)	**0.22 (0.08, 0.58)**	0.48 (0.20, 1.22)	**0.33 (0.13, 0.82)**	**0.43 (0.18, 0.92)**	**0.47 (0.21, 0.99)**	**0.44 (0.20, 0.96)**	**0.37 (0.24, 0.57)**	0.33 (0.09, 1.40)	0.34 (0.07, 1.22)	1.01 (0.39, 3.06)	0.66 (0.41, 1.04)	0.50 (0.21, 1.15)	0.72 (0.44, 1.14)
ADA40mg20mgSC	1.11 (0.38, 3.31)	2.13 (0.70, 6.39)	1.16 (0.26, 4.19)	**3.17 (1.06, 9.70)**	0.98 (0.49, 2.05)	0.75 (0.29, 1.76)	1.14 (0.48, 2.62)	1.05 (0.43, 2.49)	1.16 (0.38, 3.69)	0.89 (0.33, 2.55)	0.42 (0.13, 1.33)	0.91 (0.33, 2.72)	0.62 (0.22, 1.90)	0.83 (0.32, 1.99)	0.89 (0.34, 2.10)	0.83 (0.32, 2.08)	0.70 (0.36, 1.44)	0.63 (0.17, 3.31)	0.64 (0.12, 3.24)	1.87 (0.66, 7.58)	1.25 (0.62, 2.71)	0.96 (0.33, 3.06)	1.35 (0.68, 2.98)
ADA80mg40mgSC	0.67 (0.23, 1.90)	1.25 (0.48, 3.53)	0.68 (0.18, 2.40)	1.89 (0.70, 5.69)	0.60 (0.33, 1.09)	0.45 (0.20, 1.01)	0.69 (0.32, 1.48)	0.63 (0.29, 1.40)	0.70 (0.24, 2.09)	0.54 (0.21, 1.34)	**0.25 (0.09, 0.72)**	0.55 (0.22, 1.53)	**0.38 (0.14, 0.99)**	0.49 (0.21, 1.17)	0.54 (0.23, 1.24)	0.50 (0.22, 1.19)	**0.43 (0.25, 0.75)**	0.39 (0.10, 1.75)	0.40 (0.07, 1.49)	1.18 (0.43, 3.71)	0.76 (0.43, 1.37)	0.59 (0.22, 1.38)	0.83 (0.47, 1.49)
AMI0.4mgPO	2.25 (0.81, 6.89)	**4.30 (1.55, 12.94)**	2.34 (0.58, 8.51)	**6.46 (2.28, 19.77)**	**2.04 (1.03, 4.19)**	1.53 (0.59, 3.74)	2.34 (0.98, 5.41)	2.15 (0.91, 5.18)	2.41 (0.80, 7.88)	1.81 (0.70, 5.12)	0.86 (0.28, 2.58)	1.85 (0.70, 5.44)	1.26 (0.47, 3.85)	1.69 (0.65, 4.38)	1.84 (0.73, 4.57)	1.70 (0.71, 4.46)	1.46 (0.76, 2.90)	1.29 (0.36, 5.52)	1.35 (0.26, 5.31)	**3.96 (1.42, 13.50)**	**2.59 (1.31, 5.34)**	1.99 (0.71, 4.93)	**2.82 (1.43, 5.81)**
AND150mgSC1.1	1.95 (0.49, 6.93)	3.66 (0.99, 13.67)	1.88 (0.42, 8.50)	**5.40 (1.49, 20.77)**	1.71 (0.64, 4.49)	1.27 (0.40, 3.92)	1.98 (0.63, 5.85)	1.80 (0.59, 5.55)	2.00 (0.54, 7.60)	1.52 (0.46, 5.09)	0.73 (0.18, 2.69)	1.59 (0.43, 5.19)	1.09 (0.29, 3.72)	1.39 (0.42, 4.41)	1.51 (0.48, 4.70)	1.42 (0.43, 4.53)	1.22 (0.47, 3.18)	1.12 (0.24, 5.97)	1.12 (0.20, 4.98)	3.39 (0.88, 12.00)	2.18 (0.81, 5.75)	1.67 (0.50, 4.81)	2.38 (0.89, 6.31)
AND150mgSC2.2	1.58 (0.42, 5.36)	3.03 (0.83, 10.08)	1.56 (0.33, 6.81)	**4.42 (1.24, 15.44)**	1.37 (0.53, 3.43)	1.05 (0.31, 3.05)	1.60 (0.50, 4.47)	1.46 (0.47, 4.26)	1.63 (0.46, 5.84)	1.25 (0.39, 3.96)	0.57 (0.16, 2.08)	1.29 (0.37, 4.15)	0.88 (0.25, 2.98)	1.14 (0.35, 3.57)	1.22 (0.39, 3.76)	1.16 (0.35, 3.54)	0.99 (0.38, 2.42)	0.90 (0.19, 4.27)	0.91 (0.15, 4.26)	2.69 (0.74, 10.19)	1.78 (0.67, 4.39)	1.33 (0.44, 4.00)	1.93 (0.73, 4.77)
AND300mgSC	2.96 (0.72, 11.83)	**5.54 (1.42, 23.93)**	2.94 (0.61, 15.78)	**8.23 (2.03, 36.62)**	2.62 (0.87, 8.15)	1.95 (0.55, 7.04)	3.01 (0.92, 10.40)	2.75 (0.84, 9.92)	3.15 (0.76, 13.07)	2.31 (0.66, 8.84)	1.10 (0.28, 4.62)	2.45 (0.63, 9.43)	1.65 (0.42, 6.44)	2.16 (0.60, 7.80)	2.37 (0.66, 8.31)	2.18 (0.62, 8.04)	1.89 (0.64, 5.72)	1.71 (0.32, 10.17)	1.71 (0.28, 8.80)	**5.27 (1.21, 21.50)**	**3.36 (1.10, 10.39)**	2.48 (0.69, 9.41)	**3.67 (1.20, 11.36)**
API100mgPO	2.10 (0.66, 7.33)	**3.98 (1.39, 14.73)**	2.18 (0.52, 8.97)	**5.94 (2.09, 22.29)**	1.90 (0.87, 4.35)	1.42 (0.57, 3.73)	2.23 (0.89, 5.59)	2.02 (0.79, 5.26)	2.24 (0.70, 7.31)	1.70 (0.60, 5.23)	0.81 (0.23, 2.72)	1.77 (0.58, 5.43)	1.20 (0.41, 3.96)	1.60 (0.59, 4.12)	1.71 (0.66, 4.64)	1.59 (0.60, 4.56)	1.37 (0.64, 3.08)	1.25 (0.31, 5.76)	1.23 (0.24, 5.03)	**3.74 (1.21, 12.20)**	**2.42 (1.12, 5.48)**	1.85 (0.67, 5.34)	**2.64 (1.22, 5.97)**
API50mgPO	2.93 (0.91, 9.86)	**5.53 (1.82, 19.94)**	3.07 (0.69, 12.52)	**8.34 (2.62, 29.03)**	**2.61 (1.14, 6.30)**	1.92 (0.76, 5.76)	**2.99 (1.19, 8.40)**	**2.74 (1.09, 8.05)**	3.13 (0.93, 11.14)	2.38 (0.82, 7.27)	1.12 (0.34, 3.84)	2.43 (0.83, 7.50)	1.67 (0.54, 5.51)	2.19 (0.80, 6.30)	2.40 (0.88, 6.65)	2.25 (0.80, 6.31)	1.87 (0.84, 4.50)	1.73 (0.44, 8.21)	1.68 (0.32, 8.99)	**5.14 (1.62, 18.73)**	**3.37 (1.45, 8.04)**	2.52 (0.88, 8.88)	**3.69 (1.56, 8.86)**
BRI400mgIV	0.56 (0.15, 1.92)	1.04 (0.28, 3.88)	0.56 (0.12, 2.41)	1.60 (0.41, 5.65)	0.50 (0.17, 1.28)	0.38 (0.11, 1.12)	0.58 (0.18, 1.72)	0.53 (0.17, 1.60)	0.58 (0.16, 2.24)	0.45 (0.13, 1.46)	**0.20 (0.05, 0.78)**	0.46 (0.13, 1.52)	0.32 (0.09, 1.09)	0.41 (0.12, 1.20)	0.44 (0.14, 1.33)	0.41 (0.13, 1.28)	**0.36 (0.13, 0.90)**	0.32 (0.07, 1.89)	0.32 (0.05, 1.44)	0.96 (0.26, 3.84)	0.64 (0.22, 1.63)	0.49 (0.13, 1.46)	0.70 (0.24, 1.77)
BRI700mgIV	0.72 (0.19, 2.38)	1.35 (0.35, 4.67)	0.71 (0.16, 3.03)	2.04 (0.53, 7.00)	0.64 (0.23, 1.48)	0.49 (0.14, 1.34)	0.74 (0.24, 1.93)	0.67 (0.21, 1.88)	0.73 (0.21, 2.68)	0.58 (0.16, 1.70)	**0.26 (0.07, 0.87)**	0.59 (0.17, 1.81)	0.40 (0.11, 1.28)	0.52 (0.16, 1.47)	0.56 (0.18, 1.62)	0.52 (0.17, 1.52)	0.46 (0.16, 1.03)	0.41 (0.09, 2.10)	0.42 (0.07, 1.74)	1.24 (0.33, 4.48)	0.82 (0.28, 1.90)	0.61 (0.17, 1.73)	0.90 (0.31, 2.04)
BRO210mgIV	3.98 (0.43, 51.94)	7.46 (0.73, 90.62)	4.27 (0.34, 54.09)	**10.95 (1.20, 136.70)**	3.44 (0.44, 39.49)	2.59 (0.32, 33.04)	4.03 (0.46, 47.34)	3.59 (0.44, 44.22)	4.27 (0.39, 46.33)	3.21 (0.35, 37.60)	1.41 (0.14, 18.17)	3.12 (0.35, 39.99)	2.26 (0.23, 28.00)	2.84 (0.31, 33.93)	3.22 (0.38, 36.25)	2.92 (0.36, 34.41)	2.47 (0.33, 28.15)	2.26 (0.26, 25.21)	2.25 (0.21, 28.46)	7.15 (0.79, 74.68)	4.44 (0.58, 48.86)	3.30 (0.40, 36.63)	4.82 (0.63, 53.80)
BRO350mgIV	0.59 (0.08, 3.20)	1.14 (0.14, 6.08)	0.59 (0.07, 3.85)	1.71 (0.21, 8.84)	0.57 (0.07, 2.12)	0.41 (0.05, 1.81)	0.63 (0.08, 2.64)	0.57 (0.07, 2.48)	0.66 (0.07, 3.53)	0.48 (0.06, 2.43)	0.23 (0.03, 1.20)	0.50 (0.07, 2.47)	0.34 (0.04, 1.73)	0.44 (0.05, 2.17)	0.50 (0.06, 2.24)	0.46 (0.06, 2.22)	0.41 (0.05, 1.51)	0.34 (0.04, 2.41)	0.34 (0.04, 2.30)	1.04 (0.14, 5.46)	0.71 (0.09, 2.64)	0.51 (0.07, 2.66)	0.78 (0.10, 2.91)
BRO700mgIV	1.02 (0.12, 8.23)	2.01 (0.24, 16.69)	1.04 (0.12, 10.04)	2.93 (0.38, 19.70)	0.93 (0.12, 6.41)	0.70 (0.09, 5.25)	1.08 (0.13, 7.66)	1.00 (0.11, 7.24)	1.11 (0.12, 8.28)	0.82 (0.10, 7.22)	0.39 (0.05, 2.74)	0.82 (0.10, 6.97)	0.58 (0.07, 4.90)	0.79 (0.08, 5.30)	0.86 (0.09, 5.68)	0.78 (0.09, 5.01)	0.67 (0.09, 4.45)	0.59 (0.07, 5.73)	0.59 (0.05, 6.69)	1.75 (0.24, 14.80)	1.19 (0.16, 8.00)	0.90 (0.11, 5.93)	1.30 (0.17, 8.65)
CDP10mg/kgIV	1.52 (0.61, 4.23)	**2.89 (1.19, 7.82)**	1.58 (0.43, 5.18)	**4.39 (1.77, 11.74)**	1.38 (0.87, 2.14)	1.02 (0.51, 2.12)	1.56 (0.81, 3.06)	1.43 (0.74, 2.99)	1.63 (0.61, 4.60)	1.24 (0.52, 2.90)	0.58 (0.19, 1.56)	1.26 (0.52, 3.02)	0.87 (0.34, 2.24)	1.14 (0.51, 2.43)	1.23 (0.56, 2.55)	1.16 (0.52, 2.48)	0.99 (0.66, 1.46)	0.89 (0.25, 3.64)	0.90 (0.19, 3.19)	**2.66 (1.08, 7.85)**	**1.76 (1.13, 2.70)**	1.33 (0.58, 3.08)	**1.92 (1.21, 2.93)**
CER100mgSC	1.35 (0.52, 3.74)	**2.59 (1.03, 7.27)**	1.42 (0.35, 5.09)	**3.92 (1.37, 11.31)**	1.23 (0.73, 2.12)	0.92 (0.42, 1.91)	1.40 (0.71, 2.91)	1.29 (0.63, 2.67)	1.46 (0.50, 4.28)	1.10 (0.45, 2.80)	0.52 (0.18, 1.45)	1.12 (0.43, 3.07)	0.75 (0.31, 2.17)	1.01 (0.43, 2.36)	1.10 (0.50, 2.45)	1.03 (0.41, 2.37)	0.88 (0.54, 1.46)	0.79 (0.23, 3.11)	0.80 (0.16, 2.90)	2.35 (0.95, 7.22)	1.57 (0.93, 2.66)	1.20 (0.51, 2.69)	**1.71 (1.02, 2.92)**
CER10mg/kgIV	2.26 (0.56, 10.24)	**4.18 (1.15, 18.73)**	2.27 (0.50, 12.03)	**6.31 (1.70, 28.59)**	1.97 (0.72, 6.68)	1.51 (0.47, 6.42)	2.32 (0.72, 8.47)	2.09 (0.65, 8.39)	2.33 (0.60, 10.05)	1.80 (0.51, 6.91)	0.81 (0.23, 3.87)	1.85 (0.53, 7.84)	1.23 (0.35, 5.36)	1.63 (0.49, 6.14)	1.79 (0.53, 6.49)	1.69 (0.50, 6.29)	1.42 (0.53, 4.73)	1.29 (0.29, 8.35)	1.31 (0.21, 7.32)	**3.88 (1.08, 17.27)**	2.56 (0.93, 8.57)	1.94 (0.56, 7.52)	**2.78 (1.01, 9.36)**
CER200mgSC	1.91 (0.68, 5.59)	**3.65 (1.36, 10.57)**	1.97 (0.54, 7.04)	**5.51 (1.98, 16.06)**	1.70 (0.98, 3.21)	1.30 (0.54, 2.98)	1.99 (0.91, 4.34)	1.80 (0.84, 4.24)	2.08 (0.68, 6.03)	1.55 (0.62, 4.47)	0.72 (0.26, 2.13)	1.56 (0.62, 4.40)	1.10 (0.40, 3.42)	1.43 (0.58, 3.46)	1.55 (0.66, 3.75)	1.47 (0.61, 3.45)	1.22 (0.72, 2.21)	1.15 (0.29, 5.01)	1.14 (0.23, 4.62)	**3.38 (1.24, 10.16)**	**2.19 (1.23, 4.14)**	1.67 (0.68, 4.11)	**2.37 (1.33, 4.54)**

	MED700mgIV	MIR1000mgIV	MIR200mgIV	MIR600mgIV	NAT300mgIV	NAT3mg/kgIV	NAT3mg/kgIVx2	NAT6mg/kgIVx2	NNC2mg/kgSC	ONE10mgSC	ONE25mgSC	ONE35mgSC	ONE50mgSC	ONT22_5mgSC	ONT225mgSC	ONT75mgSC	PBO	PF10mgSC	PF200mgSC	PF50mgSC	RIS1200mgIV	RIS200mgIV	RIS600mgIV
CER20mg/kgIV	4.23 (0.97, 23.95)	**7.67 (1.87, 44.11)**	4.34 (0.82, 27.03)	**11.58 (2.88, 64.14)**	**3.63 (1.19, 17.35)**	2.72 (0.79, 13.93)	**4.12 (1.30, 21.14)**	**3.82 (1.12, 20.15)**	**4.44 (1.06, 25.93)**	3.29 (0.90, 18.84)	1.54 (0.39, 9.59)	3.30 (0.86, 19.85)	2.28 (0.59, 14.51)	2.99 (0.84, 16.25)	3.21 (0.92, 17.84)	3.12 (0.84, 16.71)	2.60 (0.87, 12.40)	2.55 (0.45, 15.65)	2.37 (0.37, 18.15)	**7.13 (1.76, 44.29)**	**4.64 (1.55, 22.12)**	3.61 (0.91, 18.08)	**5.04 (1.69, 23.79)**
CER400mgSC	1.25 (0.54, 3.26)	**2.39 (1.04, 5.94)**	1.32 (0.38, 4.16)	**3.65 (1.53, 9.20)**	1.14 (0.84, 1.54)	0.86 (0.45, 1.58)	1.30 (0.74, 2.30)	1.19 (0.67, 2.22)	1.34 (0.52, 3.63)	1.03 (0.47, 2.36)	0.48 (0.18, 1.21)	1.04 (0.46, 2.48)	0.71 (0.31, 1.84)	0.94 (0.46, 1.89)	1.03 (0.51, 1.96)	0.96 (0.46, 1.91)	0.82 (0.65, 1.01)	0.75 (0.22, 2.93)	0.75 (0.16, 2.62)	2.18 (0.96, 6.20)	**1.45 (1.08, 1.97)**	1.11 (0.50, 2.36)	**1.58 (1.18, 2.15)**
CER5mg/kgIV	1.58 (0.45, 5.42)	2.93 (0.87, 9.93)	1.66 (0.34, 6.40)	**4.49 (1.33, 14.85)**	1.42 (0.54, 3.20)	1.06 (0.36, 2.99)	1.60 (0.57, 4.43)	1.49 (0.51, 3.98)	1.65 (0.45, 5.79)	1.27 (0.39, 3.71)	0.59 (0.17, 2.02)	1.28 (0.37, 4.28)	0.87 (0.29, 2.97)	1.14 (0.36, 3.20)	1.27 (0.41, 3.53)	1.16 (0.37, 3.42)	1.02 (0.39, 2.25)	0.91 (0.20, 4.99)	0.90 (0.14, 4.00)	2.72 (0.77, 10.78)	1.80 (0.71, 4.12)	1.36 (0.43, 4.11)	1.96 (0.77, 4.50)
ELD10mg/kgIV	1.41 (0.42, 4.80)	2.63 (0.80, 9.77)	1.46 (0.33, 5.93)	**3.91 (1.19, 15.51)**	1.26 (0.50, 3.17)	0.94 (0.33, 2.88)	1.45 (0.53, 4.24)	1.33 (0.48, 3.82)	1.45 (0.44, 5.43)	1.12 (0.36, 3.75)	0.53 (0.14, 1.89)	1.15 (0.36, 4.15)	0.79 (0.24, 2.82)	1.02 (0.34, 3.19)	1.12 (0.37, 3.38)	1.04 (0.36, 3.34)	0.91 (0.37, 2.24)	0.82 (0.20, 4.46)	0.83 (0.14, 3.84)	2.46 (0.72, 9.14)	1.61 (0.62, 3.91)	1.20 (0.39, 3.77)	1.76 (0.69, 4.26)
ELD20mg/kgIV	1.07 (0.33, 3.36)	1.98 (0.64, 6.20)	1.08 (0.27, 3.99)	2.97 (0.94, 9.72)	0.96 (0.40, 2.09)	0.71 (0.25, 1.87)	1.09 (0.40, 2.81)	1.00 (0.36, 2.59)	1.11 (0.33, 3.63)	0.84 (0.29, 2.65)	0.40 (0.11, 1.27)	0.86 (0.28, 2.87)	0.59 (0.19, 2.01)	0.79 (0.27, 2.19)	0.86 (0.30, 2.32)	0.79 (0.27, 2.33)	0.69 (0.29, 1.43)	0.60 (0.15, 3.18)	0.62 (0.12, 2.86)	1.85 (0.58, 6.70)	1.22 (0.50, 2.61)	0.92 (0.30, 2.95)	1.34 (0.55, 2.84)
ETA25mgSC	3.05 (0.67, 17.35)	**5.88 (1.22, 31.01)**	3.22 (0.52, 20.14)	**8.96 (1.81, 47.69)**	2.72 (0.77, 12.54)	2.04 (0.51, 10.98)	3.12 (0.81, 15.92)	2.83 (0.73, 14.88)	3.27 (0.69, 17.98)	2.49 (0.54, 12.20)	1.19 (0.23, 6.18)	2.53 (0.59, 13.67)	1.76 (0.37, 8.47)	2.25 (0.56, 11.70)	2.42 (0.62, 12.39)	2.31 (0.57, 12.25)	1.96 (0.56, 8.84)	1.81 (0.29, 20.05)	1.81 (0.21, 14.94)	**5.33 (1.16, 31.88)**	3.47 (0.97, 16.06)	2.61 (0.59, 18.08)	**3.78 (1.05, 17.41)**
ETR105mgSC	1.63 (0.68, 4.31)	**3.10 (1.24, 8.29)**	1.71 (0.46, 5.50)	**4.71 (1.89, 12.48)**	1.46 (0.97, 2.24)	1.10 (0.53, 2.14)	1.68 (0.88, 3.21)	1.54 (0.80, 3.03)	1.72 (0.66, 4.89)	1.32 (0.59, 3.12)	0.62 (0.22, 1.64)	1.34 (0.57, 3.26)	0.91 (0.38, 2.36)	1.22 (0.56, 2.53)	1.32 (0.63, 2.71)	1.24 (0.58, 2.59)	1.05 (0.73, 1.52)	0.95 (0.26, 3.82)	0.97 (0.19, 3.33)	**2.82 (1.17, 8.24)**	**1.87 (1.22, 2.87)**	1.43 (0.62, 3.10)	**2.04 (1.32, 3.11)**
ETR210mgSC	1.41 (0.58, 3.80)	**2.70 (1.10, 6.94)**	1.48 (0.41, 4.74)	**4.06 (1.60, 11.10)**	1.28 (0.84, 1.96)	0.96 (0.47, 1.91)	1.46 (0.76, 2.75)	1.34 (0.69, 2.69)	1.49 (0.57, 4.22)	1.16 (0.51, 2.80)	0.53 (0.19, 1.37)	1.16 (0.49, 2.82)	0.80 (0.33, 2.09)	1.06 (0.49, 2.22)	1.15 (0.55, 2.32)	1.08 (0.50, 2.35)	0.92 (0.63, 1.32)	0.84 (0.24, 3.45)	0.84 (0.17, 3.03)	**2.47 (1.02, 7.40)**	**1.63 (1.07, 2.47)**	1.24 (0.54, 2.63)	**1.78 (1.16, 2.69)**
FIL100mgPO	0.85 (0.35, 2.33)	1.62 (0.68, 4.23)	0.89 (0.23, 2.85)	**2.44 (1.02, 6.36)**	0.77 (0.53, 1.12)	0.58 (0.29, 1.11)	0.88 (0.49, 1.66)	0.81 (0.43, 1.56)	0.92 (0.36, 2.48)	0.69 (0.31, 1.59)	**0.33 (0.12, 0.84)**	0.71 (0.31, 1.72)	0.49 (0.20, 1.22)	0.64 (0.31, 1.33)	0.70 (0.33, 1.39)	0.65 (0.31, 1.40)	**0.56 (0.40, 0.76)**	0.50 (0.14, 2.03)	0.51 (0.10, 1.71)	1.49 (0.62, 4.33)	0.98 (0.67, 1.44)	0.75 (0.33, 1.63)	1.07 (0.74, 1.57)
FIL200mgPO	1.01 (0.43, 2.71)	1.95 (0.81, 5.15)	1.07 (0.27, 3.39)	**2.98 (1.19, 7.70)**	0.93 (0.65, 1.33)	0.70 (0.35, 1.32)	1.07 (0.59, 1.94)	0.98 (0.53, 1.84)	1.10 (0.42, 3.01)	0.84 (0.38, 1.86)	0.39 (0.15, 1.00)	0.85 (0.37, 2.02)	0.58 (0.25, 1.45)	0.77 (0.37, 1.56)	0.84 (0.41, 1.65)	0.78 (0.37, 1.62)	**0.67 (0.49, 0.89)**	0.61 (0.18, 2.44)	0.61 (0.13, 2.06)	1.78 (0.76, 5.16)	1.19 (0.84, 1.68)	0.90 (0.40, 1.88)	1.29 (0.91, 1.82)
FON0.1mg/kgIV	3.94 (0.52, 64.44)	8.02 (0.94, 126.13)	3.97 (0.47, 73.94)	**12.21 (1.41, 192.59)**	3.64 (0.53, 51.38)	2.72 (0.37, 39.70)	4.08 (0.55, 63.55)	3.73 (0.49, 60.03)	4.23 (0.53, 81.50)	3.10 (0.42, 52.63)	1.46 (0.16, 27.47)	3.25 (0.40, 58.09)	2.21 (0.28, 37.34)	2.92 (0.42, 46.84)	3.25 (0.43, 48.15)	3.01 (0.40, 49.39)	2.59 (0.38, 35.61)	2.60 (0.23, 38.47)	2.46 (0.18, 38.30)	7.24 (0.82, 108.29)	4.64 (0.66, 65.45)	3.49 (0.45, 52.74)	5.07 (0.72, 70.37)
FON1mg/kg0.1mgIV/SC	1.41 (0.44, 5.05)	2.75 (0.82, 8.43)	1.48 (0.33, 5.65)	**4.09 (1.22, 12.87)**	1.27 (0.56, 3.06)	0.95 (0.35, 2.85)	1.44 (0.55, 4.05)	1.32 (0.49, 3.90)	1.51 (0.45, 5.37)	1.17 (0.38, 3.43)	0.55 (0.15, 1.81)	1.17 (0.39, 3.80)	0.81 (0.26, 2.67)	1.05 (0.35, 3.19)	1.13 (0.41, 3.40)	1.06 (0.37, 3.16)	0.91 (0.40, 2.17)	0.85 (0.20, 3.84)	0.84 (0.15, 3.30)	2.57 (0.75, 8.46)	1.62 (0.70, 4.01)	1.26 (0.40, 3.38)	1.76 (0.76, 4.42)
FON1mg/kg1mgIV/SC	1.12 (0.35, 3.48)	2.11 (0.66, 6.44)	1.16 (0.28, 4.23)	3.22 (0.95, 10.22)	1.02 (0.42, 2.17)	0.75 (0.26, 1.93)	1.14 (0.42, 2.84)	1.03 (0.39, 2.71)	1.17 (0.36, 3.99)	0.90 (0.29, 2.66)	0.42 (0.12, 1.33)	0.92 (0.31, 2.75)	0.63 (0.20, 1.92)	0.83 (0.29, 2.27)	0.89 (0.33, 2.40)	0.84 (0.30, 2.36)	0.73 (0.31, 1.50)	0.67 (0.15, 3.02)	0.67 (0.11, 2.64)	1.99 (0.62, 6.34)	1.28 (0.54, 2.82)	0.97 (0.33, 2.57)	1.40 (0.59, 3.09)
FON1mg/kgIV	1.89 (0.38, 11.99)	3.69 (0.72, 20.39)	1.95 (0.32, 13.01)	**5.49 (1.04, 32.85)**	1.69 (0.42, 7.31)	1.28 (0.28, 5.68)	1.91 (0.45, 8.92)	1.77 (0.39, 8.51)	2.02 (0.38, 12.19)	1.52 (0.33, 7.75)	0.72 (0.13, 4.43)	1.58 (0.33, 8.31)	1.06 (0.23, 5.72)	1.42 (0.29, 7.08)	1.53 (0.33, 7.57)	1.45 (0.30, 7.24)	1.20 (0.30, 5.11)	1.11 (0.18, 6.66)	1.12 (0.13, 7.41)	3.30 (0.63, 17.36)	2.15 (0.54, 9.33)	1.69 (0.34, 8.01)	2.33 (0.58, 10.20)
FON4mg/kg0.1mgIV/SC	0.88 (0.29, 2.84)	1.73 (0.55, 5.36)	0.93 (0.21, 3.37)	2.59 (0.84, 7.88)	0.82 (0.37, 1.66)	0.61 (0.23, 1.53)	0.93 (0.36, 2.25)	0.85 (0.32, 2.13)	0.96 (0.30, 3.26)	0.72 (0.25, 2.05)	0.34 (0.10, 1.11)	0.73 (0.25, 2.23)	0.51 (0.17, 1.55)	0.67 (0.24, 1.83)	0.72 (0.28, 1.94)	0.69 (0.24, 1.85)	0.59 (0.27, 1.17)	0.54 (0.13, 2.19)	0.53 (0.11, 2.14)	1.61 (0.52, 5.10)	1.04 (0.47, 2.13)	0.79 (0.29, 1.93)	1.13 (0.51, 2.32)
FON4mg/kg1mgIV/SC	1.10 (0.33, 3.64)	2.08 (0.66, 6.66)	1.12 (0.27, 4.33)	3.14 (0.99, 10.08)	0.99 (0.42, 2.23)	0.75 (0.26, 1.98)	1.13 (0.42, 2.93)	1.03 (0.37, 2.70)	1.17 (0.36, 4.01)	0.88 (0.31, 2.64)	0.41 (0.12, 1.41)	0.91 (0.30, 2.85)	0.62 (0.20, 2.08)	0.81 (0.29, 2.29)	0.89 (0.32, 2.49)	0.84 (0.28, 2.23)	0.71 (0.31, 1.56)	0.64 (0.16, 3.32)	0.63 (0.12, 3.05)	1.94 (0.62, 6.73)	1.27 (0.54, 2.82)	0.96 (0.33, 2.79)	1.37 (0.59, 3.05)
FON4mg/kgIV	0.94 (0.21, 4.23)	1.83 (0.40, 7.86)	0.99 (0.17, 4.83)	2.74 (0.58, 11.82)	0.86 (0.22, 2.81)	0.63 (0.14, 2.33)	0.97 (0.23, 3.39)	0.89 (0.20, 3.26)	1.03 (0.20, 4.45)	0.76 (0.17, 3.16)	0.36 (0.07, 1.57)	0.77 (0.17, 3.19)	0.53 (0.11, 2.31)	0.70 (0.17, 2.50)	0.76 (0.18, 2.85)	0.74 (0.16, 2.78)	0.61 (0.16, 1.97)	0.56 (0.10, 2.55)	0.54 (0.07, 3.26)	1.67 (0.36, 6.77)	1.10 (0.28, 3.62)	0.81 (0.18, 3.21)	1.20 (0.31, 3.88)
GUS1200mgIV	0.54 (0.18, 1.63)	1.01 (0.35, 2.98)	0.56 (0.13, 1.97)	1.50 (0.54, 4.50)	**0.48 (0.23, 0.93)**	**0.36 (0.15, 0.83)**	0.55 (0.24, 1.23)	0.51 (0.21, 1.13)	0.57 (0.18, 1.79)	0.44 (0.15, 1.14)	**0.20 (0.06, 0.61)**	0.45 (0.15, 1.21)	**0.31 (0.10, 0.88)**	**0.39 (0.16, 0.98)**	0.44 (0.17, 1.04)	0.40 (0.15, 1.02)	**0.35 (0.17, 0.64)**	0.32 (0.07, 1.36)	0.32 (0.05, 1.24)	0.95 (0.30, 2.98)	0.62 (0.31, 1.18)	0.47 (0.17, 1.27)	0.67 (0.33, 1.30)
GUS200mgIV	0.43 (0.15, 1.34)	0.81 (0.30, 2.41)	0.43 (0.11, 1.59)	1.21 (0.43, 3.57)	**0.39 (0.19, 0.72)**	**0.29 (0.12, 0.65)**	**0.44 (0.20, 0.96)**	**0.40 (0.17, 0.89)**	0.45 (0.14, 1.41)	**0.35 (0.12, 0.92)**	**0.16 (0.05, 0.48)**	**0.36 (0.12, 0.93)**	**0.24 (0.09, 0.71)**	**0.32 (0.12, 0.78)**	**0.34 (0.14, 0.82)**	**0.32 (0.13, 0.79)**	**0.28 (0.14, 0.50)**	0.25 (0.06, 1.09)	**0.25 (0.05, 0.94)**	0.76 (0.25, 2.35)	**0.50 (0.25, 0.92)**	**0.37 (0.14, 0.91)**	0.54 (0.27, 1.00)
GUS600mgIV	0.45 (0.15, 1.35)	0.83 (0.30, 2.50)	0.45 (0.11, 1.64)	1.24 (0.44, 3.69)	**0.40 (0.20, 0.74)**	**0.30 (0.13, 0.67)**	**0.45 (0.20, 0.99)**	**0.42 (0.18, 0.92)**	0.47 (0.15, 1.48)	**0.37 (0.13, 0.92)**	**0.17 (0.05, 0.49)**	**0.37 (0.13, 0.95)**	**0.25 (0.09, 0.72)**	**0.32 (0.13, 0.80)**	**0.35 (0.15, 0.86)**	**0.33 (0.13, 0.86)**	**0.29 (0.15, 0.52)**	0.26 (0.06, 1.11)	**0.26 (0.05, 0.96)**	0.79 (0.26, 2.40)	**0.52 (0.25, 0.95)**	**0.39 (0.14, 0.97)**	0.56 (0.28, 1.04)
INF10mg/kgIV	0.19 (0.02, 1.45)	0.37 (0.03, 2.72)	0.19 (0.01, 1.76)	0.55 (0.05, 4.51)	0.17 (0.02, 1.06)	**0.13 (0.01, 0.84)**	0.20 (0.02, 1.35)	0.18 (0.02, 1.23)	0.21 (0.02, 1.58)	**0.16 (0.01, 0.99)**	**0.07 (0.01, 0.52)**	0.16 (0.01, 1.09)	**0.10 (0.01, 0.77)**	**0.14 (0.01, 0.97)**	0.16 (0.01, 1.04)	0.14 (0.01, 1.03)	**0.12 (0.01, 0.75)**	0.11 (0.01, 1.19)	**0.11 (0.01, 0.91)**	0.34 (0.03, 2.57)	0.22 (0.02, 1.35)	0.15 (0.02, 1.18)	0.24 (0.02, 1.48)
INF20mg/kgIV	0.19 (0.02, 1.57)	0.36 (0.03, 2.85)	0.19 (0.01, 1.93)	0.55 (0.05, 4.44)	0.17 (0.02, 1.11)	**0.13 (0.01, 0.86)**	0.20 (0.02, 1.38)	0.18 (0.02, 1.27)	0.20 (0.02, 1.77)	0.16 (0.01, 1.10)	**0.07 (0.01, 0.49)**	0.17 (0.01, 1.08)	**0.11 (0.01, 0.80)**	0.14 (0.01, 1.01)	0.16 (0.01, 1.07)	0.15 (0.01, 1.05)	**0.13 (0.01, 0.79)**	0.11 (0.01, 1.30)	**0.11 (0.01, 0.97)**	0.34 (0.03, 2.95)	0.22 (0.02, 1.45)	0.15 (0.02, 1.30)	0.24 (0.02, 1.58)
INF5mg/kgIV	**0.10 (0.01, 0.68)**	0.19 (0.02, 1.26)	**0.10 (0.01, 0.83)**	0.29 (0.03, 2.00)	**0.09 (0.01, 0.48)**	**0.07 (0.01, 0.39)**	**0.10 (0.01, 0.59)**	**0.09 (0.01, 0.55)**	**0.10 (0.01, 0.74)**	**0.08 (0.01, 0.48)**	**0.04 (0.00, 0.24)**	**0.08 (0.01, 0.49)**	**0.06 (0.00, 0.35)**	**0.07 (0.01, 0.45)**	**0.08 (0.01, 0.48)**	**0.07 (0.01, 0.48)**	**0.07 (0.01, 0.34)**	**0.06 (0.00, 0.51)**	**0.06 (0.00, 0.43)**	0.18 (0.02, 1.31)	**0.12 (0.01, 0.63)**	**0.08 (0.01, 0.56)**	**0.13 (0.01, 0.69)**

(TABLE S12A. continued)

	MED700mgIV	MIR1000mgIV	MIR200mgIV	MIR600mgIV	NAT300mgIV	NAT3mg/kgIV	NAT3mg/kgIVx2	NAT6mg/kgIVx2	NNC2mg/kgSC	ONE10mgSC	ONE25mgSC	ONE35mgSC	ONE50mgSC	ONT22_5mgSC	ONT225mgSC	ONT75mgSC	PBO	PF10mgSC	PF200mgSC	PF50mgSC	RIS1200mgIV	RIS200mgIV	RIS600mgIV
MED700mgIV	MED700mgIV	1.92 (0.59, 6.18)	1.02 (0.24, 4.05)	2.83 (0.88, 9.43)	0.90 (0.35, 2.04)	0.68 (0.22, 1.87)	1.01 (0.36, 2.74)	0.95 (0.32, 2.51)	1.05 (0.30, 3.70)	0.80 (0.25, 2.47)	0.38 (0.10, 1.25)	0.84 (0.24, 2.64)	0.57 (0.16, 1.87)	0.75 (0.23, 2.05)	0.81 (0.26, 2.21)	0.77 (0.25, 2.16)	0.65 (0.26, 1.45)	0.58 (0.13, 2.90)	0.60 (0.08, 2.74)	1.76 (0.50, 6.57)	1.15 (0.44, 2.68)	0.87 (0.26, 2.48)	1.26 (0.48, 2.91)
MIR1000mgIV	0.52 (0.16, 1.70)	MIR1000mgIV	0.57 (0.17, 1.29)	1.50 (0.83, 2.70)	0.48 (0.19, 1.09)	**0.36 (0.12, 0.95)**	0.54 (0.19, 1.41)	0.50 (0.17, 1.34)	0.55 (0.16, 2.09)	0.42 (0.14, 1.42)	**0.19 (0.06, 0.69)**	0.44 (0.14, 1.44)	0.30 (0.09, 1.02)	0.40 (0.12, 1.11)	0.43 (0.14, 1.13)	0.40 (0.13, 1.13)	**0.34 (0.14, 0.76)**	0.31 (0.07, 1.47)	0.31 (0.05, 1.47)	0.93 (0.28, 3.11)	0.61 (0.24, 1.38)	0.45 (0.14, 1.46)	0.67 (0.27, 1.50)
MIR200mgIV	0.98 (0.25, 4.14)	1.77 (0.78, 5.77)	MIR200mgIV	**2.69 (1.13, 9.65)**	0.87 (0.27, 3.25)	0.65 (0.19, 2.45)	1.00 (0.30, 3.61)	0.92 (0.27, 3.42)	1.02 (0.23, 4.78)	0.78 (0.21, 3.57)	0.35 (0.08, 2.07)	0.79 (0.20, 4.19)	0.54 (0.14, 2.74)	0.72 (0.19, 2.71)	0.78 (0.22, 2.87)	0.73 (0.20, 2.92)	0.62 (0.20, 2.20)	0.57 (0.11, 3.57)	0.56 (0.09, 3.73)	1.71 (0.41, 7.49)	1.11 (0.34, 4.10)	0.82 (0.22, 3.61)	1.20 (0.37, 4.54)
MIR600mgIV	0.35 (0.11, 1.13)	0.67 (0.37, 1.21)	**0.37 (0.10, 0.88)**	MIR600mgIV	**0.31 (0.12, 0.73)**	**0.24 (0.08, 0.63)**	**0.37 (0.13, 0.94)**	**0.34 (0.11, 0.87)**	0.37 (0.10, 1.39)	**0.28 (0.08, 0.93)**	**0.13 (0.04, 0.45)**	**0.29 (0.09, 0.91)**	**0.20 (0.06, 0.70)**	**0.27 (0.08, 0.75)**	**0.28 (0.09, 0.79)**	**0.27 (0.09, 0.76)**	**0.23 (0.09, 0.51)**	**0.20 (0.05, 0.96)**	0.21 (0.03, 1.03)	0.63 (0.19, 2.05)	**0.40 (0.16, 0.93)**	**0.30 (0.09, 0.97)**	0.44 (0.17, 1.01)
NAT300mgIV	1.11 (0.49, 2.83)	2.09 (0.92, 5.28)	1.15 (0.31, 3.66)	**3.19 (1.36, 8.03)**	NAT300mgIV	0.75 (0.39, 1.38)	1.15 (0.65, 2.03)	1.05 (0.59, 1.90)	1.17 (0.47, 3.19)	0.90 (0.42, 2.06)	0.42 (0.16, 1.06)	0.91 (0.42, 2.18)	0.62 (0.27, 1.54)	0.84 (0.41, 1.64)	0.90 (0.46, 1.73)	0.84 (0.42, 1.68)	**0.72 (0.58, 0.87)**	0.65 (0.19, 2.54)	0.66 (0.14, 2.23)	1.89 (0.86, 5.47)	1.28 (0.96, 1.70)	0.97 (0.44, 2.06)	**1.39 (1.05, 1.84)**
NAT3mg/kgIV	1.48 (0.54, 4.51)	**2.81 (1.05, 8.18)**	1.54 (0.41, 5.23)	**4.17 (1.59, 12.69)**	1.33 (0.73, 2.58)	NAT3mg/kgIV	1.52 (0.94, 2.61)	1.40 (0.83, 2.44)	1.58 (0.54, 4.80)	1.20 (0.43, 3.36)	0.56 (0.18, 1.76)	1.21 (0.43, 3.47)	0.83 (0.31, 2.67)	1.11 (0.43, 2.74)	1.20 (0.52, 2.91)	1.13 (0.46, 2.87)	0.95 (0.54, 1.80)	0.89 (0.24, 3.64)	0.89 (0.17, 3.52)	2.61 (0.95, 7.93)	1.69 (0.91, 3.28)	1.30 (0.50, 3.27)	1.84 (1.00, 3.52)
NAT3mg/kgIVx2	0.99 (0.37, 2.77)	1.84 (0.71, 5.24)	1.00 (0.28, 3.31)	**2.72 (1.06, 7.99)**	0.87 (0.49, 1.53)	0.66 (0.38, 1.07)	NAT3mg/kgIVx2	0.92 (0.57, 1.46)	1.04 (0.36, 3.08)	0.78 (0.30, 2.17)	0.37 (0.12, 1.08)	0.80 (0.31, 2.22)	0.54 (0.21, 1.66)	0.72 (0.31, 1.69)	0.78 (0.34, 1.79)	0.74 (0.30, 1.75)	0.63 (0.37, 1.06)	0.58 (0.15, 2.41)	0.57 (0.11, 2.20)	1.69 (0.63, 5.25)	1.12 (0.62, 1.94)	0.84 (0.33, 2.13)	1.22 (0.68, 2.11)
NAT6mg/kgIVx2	1.05 (0.40, 3.11)	1.99 (0.75, 5.73)	1.09 (0.29, 3.74)	**2.96 (1.15, 8.75)**	0.95 (0.53, 1.68)	0.71 (0.41, 1.21)	1.09 (0.68, 1.76)	NAT6mg/kgIVx2	1.11 (0.39, 3.42)	0.86 (0.33, 2.43)	0.40 (0.13, 1.27)	0.87 (0.32, 2.56)	0.60 (0.21, 1.88)	0.79 (0.33, 1.85)	0.85 (0.38, 1.98)	0.80 (0.33, 1.89)	0.68 (0.39, 1.17)	0.63 (0.17, 2.66)	0.62 (0.12, 2.51)	1.85 (0.69, 5.64)	1.22 (0.66, 2.19)	0.92 (0.36, 2.49)	1.33 (0.71, 2.37)
NNC2mg/kgSC	0.95 (0.27, 3.31)	1.83 (0.48, 6.34)	0.98 (0.21, 4.36)	2.73 (0.72, 9.63)	0.85 (0.31, 2.14)	0.63 (0.21, 1.85)	0.96 (0.32, 2.77)	0.90 (0.29, 2.57)	NNC2mg/kgSC	0.75 (0.23, 2.42)	0.36 (0.09, 1.27)	0.78 (0.24, 2.51)	0.53 (0.16, 1.81)	0.69 (0.23, 2.12)	0.77 (0.24, 2.25)	0.70 (0.23, 2.21)	0.61 (0.23, 1.50)	0.55 (0.12, 2.40)	0.54 (0.09, 2.62)	1.65 (0.46, 6.37)	1.09 (0.40, 2.73)	0.83 (0.24, 2.51)	1.18 (0.44, 2.94)
ONE10mgSC	1.25 (0.40, 4.04)	2.40 (0.71, 7.34)	1.28 (0.28, 4.83)	**3.56 (1.08, 12.03)**	1.11 (0.49, 2.38)	0.83 (0.30, 2.32)	1.27 (0.46, 3.29)	1.17 (0.41, 3.04)	1.33 (0.41, 4.28)	ONE10mgSC	0.47 (0.18, 1.07)	1.02 (0.51, 1.99)	0.70 (0.33, 1.43)	0.91 (0.33, 2.46)	1.00 (0.36, 2.64)	0.94 (0.35, 2.48)	0.80 (0.36, 1.67)	0.71 (0.18, 3.05)	0.73 (0.14, 3.16)	2.15 (0.70, 7.64)	1.42 (0.63, 3.01)	1.08 (0.38, 2.73)	1.55 (0.69, 3.31)
ONE25mgSC	2.63 (0.80, 9.67)	**5.14 (1.45, 17.28)**	2.83 (0.48, 11.90)	**7.57 (2.22, 26.27)**	2.39 (0.95, 6.25)	1.77 (0.57, 5.66)	2.72 (0.93, 8.38)	2.50 (0.79, 7.73)	2.80 (0.79, 11.13)	2.11 (0.93, 5.60)	ONE25mgSC	2.15 (0.95, 6.14)	1.48 (0.61, 4.05)	2.01 (0.63, 5.94)	2.15 (0.69, 6.47)	2.03 (0.66, 5.99)	1.71 (0.70, 4.45)	1.52 (0.37, 8.76)	1.52 (0.27, 8.39)	**4.56 (1.37, 20.43)**	**3.03 (1.19, 8.01)**	2.28 (0.68, 7.92)	**3.29 (1.29, 8.77)**
ONE35mgSC	1.19 (0.38, 4.09)	2.27 (0.70, 7.35)	1.27 (0.24, 5.06)	**3.42 (1.09, 11.64)**	1.10 (0.46, 2.41)	0.83 (0.29, 2.32)	1.26 (0.45, 3.27)	1.15 (0.39, 3.13)	1.29 (0.40, 4.21)	0.98 (0.50, 1.97)	0.47 (0.16, 1.06)	ONE35mgSC	0.69 (0.32, 1.45)	0.91 (0.34, 2.45)	0.98 (0.35, 2.61)	0.92 (0.34, 2.46)	0.79 (0.34, 1.69)	0.70 (0.18, 3.18)	0.71 (0.14, 3.05)	2.12 (0.70, 7.52)	1.40 (0.59, 3.08)	1.06 (0.34, 2.91)	1.52 (0.64, 3.32)
ONE50mgSC	1.76 (0.53, 6.08)	3.38 (0.98, 11.11)	1.84 (0.36, 7.39)	**5.01 (1.44, 17.16)**	1.62 (0.65, 3.67)	1.21 (0.38, 3.27)	1.85 (0.60, 4.75)	1.68 (0.53, 4.66)	1.90 (0.55, 6.41)	1.42 (0.70, 3.00)	0.68 (0.25, 1.65)	1.46 (0.69, 3.11)	ONE50mgSC	1.32 (0.43, 3.60)	1.45 (0.49, 3.95)	1.34 (0.47, 3.70)	1.16 (0.46, 2.56)	1.03 (0.24, 4.81)	1.02 (0.19, 5.04)	3.10 (0.96, 12.53)	2.04 (0.83, 4.66)	1.55 (0.49, 4.35)	2.23 (0.92, 5.07)
ONT22_5mgSC	1.33 (0.49, 4.41)	2.52 (0.90, 8.15)	1.39 (0.37, 5.37)	**3.75 (1.34, 12.77)**	1.20 (0.61, 2.45)	0.90 (0.36, 2.31)	1.39 (0.59, 3.28)	1.27 (0.54, 3.05)	1.45 (0.47, 4.37)	1.09 (0.41, 3.05)	0.50 (0.17, 1.58)	1.09 (0.41, 2.98)	0.76 (0.28, 2.35)	ONT22_5mgSC	1.08 (0.60, 2.01)	1.01 (0.53, 2.01)	0.87 (0.45, 1.69)	0.79 (0.19, 3.39)	0.76 (0.16, 3.39)	2.36 (0.77, 7.77)	1.55 (0.76, 3.12)	1.18 (0.43, 3.28)	1.69 (0.83, 3.41)
ONT225mgSC	1.24 (0.45, 3.82)	2.31 (0.89, 7.06)	1.27 (0.35, 4.65)	**3.53 (1.27, 10.77)**	1.11 (0.58, 2.16)	0.83 (0.34, 1.94)	1.28 (0.56, 2.91)	1.18 (0.50, 2.66)	1.31 (0.45, 4.09)	1.00 (0.38, 2.74)	0.47 (0.15, 1.46)	1.02 (0.38, 2.83)	0.69 (0.25, 2.04)	0.92 (0.50, 1.68)	ONT225mgSC	0.94 (0.51, 1.73)	0.80 (0.43, 1.52)	0.72 (0.19, 3.16)	0.72 (0.16, 2.79)	2.15 (0.77, 7.50)	1.42 (0.74, 2.83)	1.08 (0.41, 2.85)	1.55 (0.81, 3.11)
ONT75mgSC	1.30 (0.46, 4.02)	2.53 (0.88, 7.50)	1.38 (0.34, 4.95)	**3.75 (1.32, 11.59)**	1.19 (0.59, 2.40)	0.89 (0.35, 2.19)	1.36 (0.57, 3.28)	1.24 (0.53, 3.05)	1.42 (0.45, 4.39)	1.06 (0.40, 2.82)	0.49 (0.17, 1.52)	1.08 (0.41, 2.96)	0.75 (0.27, 2.14)	0.99 (0.50, 1.90)	1.07 (0.58, 1.96)	ONT75mgSC	0.86 (0.44, 1.70)	0.78 (0.19, 3.72)	0.77 (0.15, 3.46)	2.29 (0.77, 7.99)	1.52 (0.76, 3.08)	1.14 (0.43, 3.36)	1.65 (0.82, 3.35)
PBO	1.55 (0.69, 3.88)	**2.91 (1.31, 7.24)**	1.60 (0.45, 4.93)	**4.44 (1.94, 10.81)**	**1.39 (1.15, 1.71)**	1.05 (0.56, 1.87)	1.59 (0.94, 2.71)	1.46 (0.85, 2.59)	1.63 (0.67, 4.35)	1.25 (0.60, 2.75)	0.58 (0.22, 1.43)	1.27 (0.59, 2.94)	0.87 (0.39, 2.18)	1.16 (0.59, 2.23)	1.25 (0.66, 2.32)	1.17 (0.59, 2.27)	PBO	0.91 (0.27, 3.51)	0.92 (0.20, 3.03)	**2.66 (1.19, 7.55)**	**1.78 (1.45, 2.18)**	1.36 (0.63, 2.76)	**1.94 (1.59, 2.36)**
PF10mgSC	1.72 (0.35, 7.56)	3.26 (0.68, 14.26)	1.76 (0.28, 8.90)	**4.89 (1.04, 20.86)**	1.55 (0.39, 5.19)	1.13 (0.27, 4.17)	1.74 (0.41, 6.66)	1.59 (0.38, 5.98)	1.81 (0.42, 8.22)	1.40 (0.33, 5.44)	0.66 (0.11, 2.67)	1.42 (0.31, 5.46)	0.97 (0.21, 4.22)	1.27 (0.30, 5.17)	1.38 (0.32, 5.32)	1.29 (0.27, 5.26)	1.10 (0.28, 3.72)	PF10mgSC	0.99 (0.20, 4.13)	**2.92 (1.13, 8.60)**	1.97 (0.49, 6.78)	1.46 (0.35, 5.58)	2.14 (0.53, 7.46)
PF200mgSC	1.68 (0.36, 12.68)	3.24 (0.68, 19.43)	1.77 (0.27, 10.95)	4.73 (0.97, 29.37)	1.53 (0.45, 6.99)	1.12 (0.28, 5.89)	1.76 (0.45, 8.87)	1.62 (0.40, 8.48)	1.84 (0.38, 10.53)	1.37 (0.32, 6.95)	0.66 (0.12, 3.69)	1.42 (0.33, 7.16)	0.98 (0.20, 5.20)	1.31 (0.29, 6.22)	1.40 (0.36, 6.43)	1.30 (0.29, 6.57)	1.09 (0.33, 4.94)	1.01 (0.24, 5.10)	PF200mgSC	2.98 (0.92, 13.48)	1.97 (0.59, 9.34)	1.47 (0.35, 7.50)	2.14 (0.64, 10.09)
PF50mgSC	0.57 (0.15, 1.99)	1.07 (0.32, 3.61)	0.59 (0.13, 2.41)	1.60 (0.49, 5.31)	0.53 (0.18, 1.17)	0.38 (0.13, 1.05)	0.59 (0.19, 1.59)	0.54 (0.18, 1.46)	0.61 (0.16, 2.19)	0.46 (0.13, 1.43)	**0.22 (0.05, 0.73)**	0.47 (0.13, 1.43)	0.32 (0.08, 1.04)	0.42 (0.13, 1.30)	0.47 (0.13, 1.29)	0.44 (0.13, 1.31)	**0.38 (0.13, 0.84)**	**0.34 (0.12, 0.89)**	0.34 (0.07, 1.08)	PF50mgSC	0.66 (0.24, 1.50)	0.51 (0.14, 1.38)	0.72 (0.26, 1.66)
RIS1200mgIV	0.87 (0.37, 2.26)	1.63 (0.72, 4.12)	0.90 (0.24, 2.92)	**2.49 (1.08, 6.37)**	0.78 (0.59, 1.04)	0.59 (0.30, 1.10)	0.89 (0.52, 1.62)	0.82 (0.46, 1.53)	0.92 (0.37, 2.47)	0.70 (0.33, 1.60)	**0.33 (0.12, 0.84)**	0.72 (0.32, 1.70)	0.49 (0.21, 1.21)	0.65 (0.32, 1.31)	0.70 (0.35, 1.36)	0.66 (0.33, 1.32)	**0.56 (0.46, 0.69)**	0.51 (0.15, 2.05)	0.51 (0.11, 1.69)	1.51 (0.67, 4.25)	RIS1200mgIV	0.76 (0.35, 1.55)	1.09 (0.94, 1.26)
RIS200mgIV	1.15 (0.40, 3.78)	2.22 (0.69, 7.14)	1.22 (0.28, 4.55)	**3.32 (1.03, 11.03)**	1.03 (0.49, 2.26)	0.77 (0.31, 2.01)	1.19 (0.47, 3.01)	1.09 (0.40, 2.77)	1.20 (0.40, 4.11)	0.93 (0.37, 2.65)	0.44 (0.13, 1.46)	0.95 (0.34, 2.93)	0.65 (0.23, 2.05)	0.85 (0.31, 2.34)	0.93 (0.35, 2.46)	0.88 (0.30, 2.35)	0.74 (0.36, 1.58)	0.68 (0.18, 2.84)	0.68 (0.13, 2.83)	1.97 (0.73, 7.04)	1.31 (0.65, 2.82)	RIS200mgIV	1.42 (0.72, 3.04)
RIS600mgIV	0.79 (0.34, 2.07)	1.50 (0.67, 3.74)	0.83 (0.22, 2.70)	2.29 (0.99, 5.79)	**0.72 (0.54, 0.95)**	0.54 (0.28, 1.00)	0.82 (0.47, 1.46)	0.75 (0.42, 1.40)	0.85 (0.34, 2.27)	0.65 (0.30, 1.45)	**0.30 (0.11, 0.77)**	0.66 (0.30, 1.55)	0.45 (0.20, 1.09)	0.59 (0.29, 1.20)	0.65 (0.32, 1.23)	0.61 (0.30, 1.21)	**0.52 (0.42, 0.63)**	0.47 (0.13, 1.90)	0.47 (0.10, 1.56)	1.38 (0.60, 3.92)	0.92 (0.79, 1.06)	0.70 (0.33, 1.40)	RIS600mgIV

	MED700mgIV	MIR1000mgIV	MIR200mgIV	MIR600mgIV	NAT300mgIV	NAT3mg/kgIV	NAT3mg/kgIVx2	NAT6mg/kgIVx2	NNC2mg/kgSC	ONE10mgSC	ONE25mgSC	ONE35mgSC	ONE50mgSC	ONT22_5mgSC	ONT225mgSC	ONT75mgSC	PBO	PF10mgSC	PF200mgSC	PF50mgSC	RIS1200mgIV	RIS200mgIV	RIS600mgIV
RIS600mgIV4_4	1.11 (0.45, 2.96)	2.10 (0.85, 5.51)	1.15 (0.31, 3.77)	**3.17 (1.29, 8.36)**	1.00 (0.65, 1.50)	0.75 (0.37, 1.50)	1.14 (0.60, 2.17)	1.04 (0.54, 2.08)	1.17 (0.44, 3.27)	0.90 (0.40, 2.08)	0.42 (0.16, 1.09)	0.91 (0.39, 2.18)	0.62 (0.26, 1.61)	0.82 (0.39, 1.79)	0.90 (0.43, 1.82)	0.84 (0.39, 1.79)	0.72 (0.49, 1.02)	0.65 (0.19, 2.68)	0.66 (0.14, 2.31)	1.90 (0.82, 5.59)	1.27 (0.84, 1.91)	0.97 (0.41, 2.22)	1.39 (0.91, 2.07)
SEC10mg/kgIV	2.08 (0.40, 10.87)	4.00 (0.76, 21.16)	2.20 (0.28, 13.39)	**5.92 (1.14, 30.31)**	1.86 (0.44, 8.53)	1.41 (0.30, 7.87)	2.10 (0.47, 9.87)	1.97 (0.41, 9.65)	2.25 (0.41, 13.69)	1.64 (0.31, 9.78)	0.78 (0.15, 4.37)	1.70 (0.34, 9.49)	1.17 (0.23, 6.57)	1.54 (0.31, 7.08)	1.71 (0.36, 7.79)	1.57 (0.33, 7.19)	1.33 (0.33, 6.34)	1.28 (0.17, 9.22)	1.20 (0.16, 9.57)	3.71 (0.67, 23.99)	2.39 (0.56, 11.43)	1.83 (0.34, 9.97)	2.61 (0.61, 12.43)
TES400mg200mgSC	0.76 (0.27, 2.12)	1.43 (0.54, 4.34)	0.80 (0.20, 2.84)	2.19 (0.78, 6.39)	0.69 (0.36, 1.25)	0.51 (0.21, 1.16)	0.79 (0.34, 1.70)	0.73 (0.31, 1.61)	0.81 (0.26, 2.38)	0.61 (0.23, 1.60)	**0.29 (0.09, 0.85)**	0.62 (0.24, 1.73)	0.43 (0.15, 1.24)	0.57 (0.22, 1.36)	0.62 (0.25, 1.44)	0.58 (0.23, 1.37)	**0.50 (0.27, 0.87)**	0.45 (0.11, 1.78)	0.46 (0.08, 1.79)	1.33 (0.46, 4.19)	0.88 (0.45, 1.61)	0.67 (0.25, 1.53)	0.96 (0.50, 1.73)
TOF15mgPO_BID	2.39 (0.47, 10.47)	**4.45 (1.08, 21.26)**	2.45 (0.39, 13.02)	**6.76 (1.57, 33.79)**	2.11 (0.65, 7.64)	1.58 (0.43, 6.31)	2.42 (0.68, 9.29)	2.23 (0.62, 8.60)	2.51 (0.51, 11.14)	1.96 (0.48, 7.57)	0.90 (0.22, 4.29)	1.95 (0.46, 8.79)	1.33 (0.34, 5.97)	1.72 (0.38, 7.21)	1.89 (0.47, 7.89)	1.78 (0.42, 7.57)	1.52 (0.47, 5.35)	1.34 (0.26, 8.27)	1.39 (0.21, 7.99)	**4.12 (1.01, 22.30)**	2.71 (0.82, 9.68)	1.99 (0.55, 8.55)	2.94 (0.88, 10.60)
TOF1mgPO_BID	1.02 (0.29, 3.61)	1.95 (0.54, 7.09)	1.05 (0.18, 4.59)	2.89 (0.77, 11.29)	0.91 (0.32, 2.31)	0.69 (0.22, 1.85)	1.05 (0.36, 2.82)	0.95 (0.30, 2.65)	1.09 (0.28, 3.91)	0.84 (0.23, 2.56)	0.38 (0.10, 1.41)	0.84 (0.24, 2.81)	0.58 (0.16, 1.93)	0.76 (0.20, 2.35)	0.81 (0.24, 2.51)	0.77 (0.23, 2.41)	0.65 (0.24, 1.62)	0.59 (0.14, 2.84)	0.58 (0.10, 2.86)	1.79 (0.51, 6.84)	1.17 (0.40, 2.96)	0.87 (0.27, 2.76)	1.27 (0.44, 3.18)
TOF5mgPO_BID	1.33 (0.36, 5.01)	2.56 (0.69, 9.18)	1.40 (0.25, 6.03)	3.77 (0.99, 14.80)	1.19 (0.44, 2.99)	0.89 (0.29, 2.52)	1.37 (0.45, 3.88)	1.26 (0.41, 3.60)	1.44 (0.37, 5.23)	1.08 (0.30, 3.46)	0.49 (0.14, 1.90)	1.10 (0.31, 3.62)	0.75 (0.22, 2.61)	0.99 (0.26, 3.00)	1.08 (0.34, 3.21)	1.01 (0.30, 3.16)	0.86 (0.32, 2.08)	0.79 (0.18, 3.82)	0.77 (0.14, 3.61)	2.38 (0.71, 8.73)	1.52 (0.54, 3.74)	1.14 (0.37, 3.45)	1.66 (0.60, 4.08)
UPA12mgPO_BID	1.31 (0.28, 5.85)	2.57 (0.57, 10.91)	1.33 (0.26, 7.03)	3.89 (0.84, 16.79)	1.20 (0.32, 4.11)	0.89 (0.21, 3.53)	1.38 (0.34, 5.33)	1.25 (0.30, 5.01)	1.42 (0.32, 6.31)	1.07 (0.24, 4.26)	0.51 (0.11, 2.15)	1.08 (0.24, 4.30)	0.74 (0.19, 3.03)	1.00 (0.23, 3.82)	1.07 (0.27, 4.51)	0.99 (0.24, 4.18)	0.86 (0.23, 2.93)	0.78 (0.12, 4.49)	0.77 (0.09, 4.74)	2.34 (0.46, 11.51)	1.54 (0.41, 5.31)	1.16 (0.26, 4.82)	1.68 (0.44, 5.81)
UPA24mgPO	2.30 (0.39, 12.42)	4.36 (0.81, 22.78)	2.42 (0.36, 13.66)	**6.45 (1.21, 34.39)**	2.08 (0.46, 9.84)	1.54 (0.33, 7.49)	2.38 (0.49, 11.14)	2.16 (0.47, 11.01)	2.45 (0.44, 16.49)	1.92 (0.31, 8.81)	0.84 (0.17, 4.79)	1.92 (0.32, 9.65)	1.29 (0.25, 6.46)	1.68 (0.31, 9.28)	1.84 (0.37, 10.16)	1.71 (0.34, 9.31)	1.50 (0.34, 6.91)	1.29 (0.18, 9.92)	1.31 (0.13, 10.15)	4.05 (0.71, 25.83)	2.66 (0.57, 12.30)	1.94 (0.40, 9.45)	2.88 (0.63, 13.21)
UPA24mgPO_BID	0.72 (0.18, 2.89)	1.34 (0.34, 5.19)	0.74 (0.14, 3.48)	2.03 (0.51, 7.80)	0.64 (0.20, 1.85)	0.48 (0.13, 1.54)	0.74 (0.21, 2.32)	0.68 (0.18, 2.24)	0.76 (0.19, 3.00)	0.57 (0.14, 2.28)	0.27 (0.07, 1.01)	0.59 (0.14, 2.23)	0.40 (0.11, 1.67)	0.52 (0.14, 1.88)	0.57 (0.15, 1.94)	0.53 (0.14, 1.89)	0.46 (0.15, 1.28)	0.42 (0.07, 2.61)	0.41 (0.05, 2.13)	1.26 (0.29, 5.20)	0.82 (0.26, 2.33)	0.62 (0.16, 2.04)	0.89 (0.28, 2.51)
UPA3mgPO_BID	1.83 (0.38, 10.01)	3.52 (0.82, 20.06)	1.87 (0.35, 10.06)	**5.36 (1.20, 28.43)**	1.63 (0.46, 7.28)	1.27 (0.29, 5.72)	1.89 (0.48, 8.75)	1.73 (0.43, 8.23)	2.00 (0.43, 10.56)	1.52 (0.32, 8.67)	0.69 (0.15, 4.55)	1.54 (0.32, 9.40)	1.02 (0.25, 6.44)	1.38 (0.30, 6.81)	1.48 (0.36, 7.75)	1.39 (0.33, 6.85)	1.18 (0.33, 5.13)	1.12 (0.18, 6.49)	1.05 (0.13, 8.24)	3.33 (0.70, 18.45)	2.11 (0.57, 9.18)	1.61 (0.36, 8.73)	2.28 (0.63, 10.26)
UPA45mgPO	0.89 (0.37, 2.27)	1.67 (0.73, 4.23)	0.92 (0.26, 2.87)	**2.54 (1.06, 6.34)**	0.80 (0.59, 1.06)	0.60 (0.31, 1.10)	0.91 (0.51, 1.61)	0.84 (0.46, 1.54)	0.93 (0.38, 2.51)	0.72 (0.33, 1.65)	**0.33 (0.13, 0.85)**	0.72 (0.33, 1.71)	0.50 (0.22, 1.28)	0.66 (0.32, 1.31)	0.72 (0.36, 1.39)	0.67 (0.33, 1.35)	**0.57 (0.45, 0.71)**	0.52 (0.15, 2.07)	0.52 (0.11, 1.77)	1.53 (0.66, 4.37)	1.02 (0.75, 1.36)	0.77 (0.35, 1.64)	1.11 (0.82, 1.48)
UPA6mgPO_BID	0.60 (0.15, 2.27)	1.13 (0.28, 4.12)	0.61 (0.12, 2.74)	1.70 (0.44, 6.04)	0.53 (0.17, 1.37)	0.40 (0.11, 1.20)	0.61 (0.17, 1.78)	0.57 (0.15, 1.69)	0.61 (0.15, 2.28)	0.48 (0.12, 1.70)	**0.22 (0.06, 0.83)**	0.49 (0.11, 1.71)	0.33 (0.09, 1.34)	0.45 (0.12, 1.36)	0.48 (0.13, 1.47)	0.45 (0.12, 1.38)	**0.38 (0.12, 0.97)**	0.35 (0.06, 2.04)	0.34 (0.04, 1.78)	1.06 (0.24, 4.11)	0.68 (0.21, 1.73)	0.51 (0.13, 1.86)	0.74 (0.23, 1.90)
UST130mgIV	0.91 (0.38, 2.39)	1.74 (0.73, 4.46)	0.95 (0.23, 3.04)	**2.66 (1.09, 6.78)**	0.82 (0.58, 1.19)	0.62 (0.31, 1.17)	0.94 (0.52, 1.69)	0.86 (0.46, 1.61)	0.96 (0.37, 2.72)	0.74 (0.33, 1.65)	**0.35 (0.13, 0.88)**	0.76 (0.33, 1.82)	0.51 (0.22, 1.27)	0.68 (0.33, 1.38)	0.74 (0.37, 1.45)	0.69 (0.34, 1.46)	**0.59 (0.44, 0.79)**	0.54 (0.15, 2.17)	0.54 (0.12, 1.80)	1.57 (0.66, 4.58)	1.05 (0.73, 1.50)	0.80 (0.36, 1.71)	1.14 (0.79, 1.64)
UST1mg/kgIV	0.79 (0.29, 2.26)	1.54 (0.59, 4.35)	0.85 (0.22, 2.86)	2.35 (0.79, 6.51)	0.73 (0.41, 1.26)	0.54 (0.24, 1.15)	0.83 (0.40, 1.69)	0.76 (0.36, 1.63)	0.85 (0.30, 2.54)	0.65 (0.25, 1.74)	**0.30 (0.09, 0.86)**	0.67 (0.25, 1.73)	0.45 (0.17, 1.30)	0.60 (0.25, 1.36)	0.67 (0.28, 1.43)	0.61 (0.24, 1.43)	**0.52 (0.30, 0.88)**	0.47 (0.13, 1.76)	0.48 (0.10, 1.85)	1.39 (0.54, 4.43)	0.93 (0.51, 1.63)	0.71 (0.28, 1.55)	1.01 (0.55, 1.75)
UST3mg/kgIV	0.77 (0.28, 2.19)	1.45 (0.58, 4.03)	0.80 (0.21, 2.72)	2.20 (0.85, 6.17)	0.69 (0.40, 1.20)	0.53 (0.23, 1.11)	0.79 (0.39, 1.63)	0.73 (0.35, 1.56)	0.82 (0.29, 2.52)	0.63 (0.25, 1.70)	**0.29 (0.10, 0.85)**	0.63 (0.24, 1.65)	0.44 (0.16, 1.25)	0.58 (0.24, 1.29)	0.63 (0.27, 1.36)	0.59 (0.25, 1.35)	**0.50 (0.30, 0.83)**	0.46 (0.12, 2.11)	0.46 (0.10, 1.77)	1.36 (0.52, 4.13)	0.89 (0.51, 1.55)	0.68 (0.28, 1.60)	0.97 (0.55, 1.68)
UST4.5mg/kgIV	0.91 (0.37, 2.38)	1.70 (0.73, 4.36)	0.93 (0.26, 3.08)	**2.57 (1.07, 6.62)**	0.82 (0.56, 1.18)	0.61 (0.30, 1.17)	0.93 (0.50, 1.72)	0.86 (0.45, 1.63)	0.95 (0.37, 2.63)	0.73 (0.33, 1.70)	**0.34 (0.13, 0.89)**	0.74 (0.33, 1.80)	0.50 (0.21, 1.29)	0.67 (0.31, 1.41)	0.73 (0.35, 1.45)	0.68 (0.32, 1.42)	**0.58 (0.43, 0.79)**	0.53 (0.15, 2.19)	0.53 (0.11, 1.84)	1.55 (0.65, 4.74)	1.04 (0.77, 1.39)	0.79 (0.36, 1.68)	1.13 (0.87, 1.46)
UST6mg/kg90mgIV/SC	0.54 (0.18, 1.69)	1.03 (0.36, 3.04)	0.55 (0.13, 1.94)	1.51 (0.54, 4.42)	**0.48 (0.24, 0.91)**	**0.37 (0.15, 0.83)**	0.55 (0.25, 1.20)	0.50 (0.22, 1.13)	0.57 (0.17, 1.84)	0.44 (0.15, 1.14)	**0.20 (0.06, 0.59)**	0.45 (0.16, 1.22)	**0.30 (0.10, 0.87)**	0.39 (0.16, 1.01)	0.44 (0.17, 1.05)	0.40 (0.15, 1.02)	**0.35 (0.18, 0.64)**	0.31 (0.08, 1.35)	0.32 (0.05, 1.21)	0.94 (0.32, 2.95)	0.62 (0.30, 1.16)	0.47 (0.17, 1.29)	0.67 (0.33, 1.26)
UST6mg/kgIV	0.70 (0.30, 1.84)	1.34 (0.57, 3.37)	0.73 (0.20, 2.40)	2.04 (0.84, 5.05)	**0.64 (0.46, 0.89)**	**0.48 (0.24, 0.90)**	0.72 (0.41, 1.31)	0.67 (0.37, 1.24)	0.75 (0.29, 2.04)	0.57 (0.26, 1.34)	**0.27 (0.10, 0.69)**	0.58 (0.26, 1.39)	0.40 (0.17, 1.00)	0.53 (0.26, 1.04)	0.57 (0.29, 1.10)	0.54 (0.26, 1.10)	**0.46 (0.35, 0.59)**	0.41 (0.12, 1.68)	0.42 (0.09, 1.41)	1.22 (0.52, 3.57)	0.81 (0.58, 1.12)	0.62 (0.28, 1.33)	0.88 (0.64, 1.23)
UST90mgSC	1.49 (0.35, 5.64)	2.80 (0.74, 10.67)	1.50 (0.33, 6.95)	**4.14 (1.09, 15.17)**	1.32 (0.45, 3.65)	1.00 (0.27, 3.05)	1.52 (0.44, 4.58)	1.40 (0.40, 4.27)	1.56 (0.39, 5.88)	1.19 (0.30, 4.39)	0.54 (0.14, 2.25)	1.21 (0.33, 4.36)	0.80 (0.21, 3.21)	1.09 (0.31, 3.56)	1.17 (0.36, 3.80)	1.09 (0.34, 3.59)	0.95 (0.32, 2.59)	0.85 (0.16, 5.11)	0.84 (0.12, 4.70)	2.58 (0.61, 10.04)	1.69 (0.56, 4.58)	1.26 (0.34, 4.69)	1.85 (0.61, 5.10)
VED0_5mg/kgIV	1.08 (0.36, 3.43)	2.10 (0.70, 5.90)	1.12 (0.26, 3.96)	**3.12 (1.07, 9.21)**	0.99 (0.47, 1.90)	0.74 (0.29, 1.80)	1.13 (0.47, 2.58)	1.04 (0.42, 2.52)	1.17 (0.35, 3.69)	0.89 (0.31, 2.32)	0.42 (0.12, 1.28)	0.91 (0.32, 2.51)	0.62 (0.20, 1.89)	0.82 (0.29, 2.01)	0.89 (0.33, 2.08)	0.82 (0.30, 2.08)	0.71 (0.35, 1.32)	0.64 (0.15, 2.93)	0.63 (0.11, 2.54)	1.88 (0.61, 6.66)	1.27 (0.59, 2.42)	0.96 (0.34, 2.61)	1.38 (0.65, 2.65)
VED2mg/kgIV	0.87 (0.30, 2.59)	1.66 (0.57, 4.54)	0.88 (0.23, 3.08)	2.43 (0.86, 7.71)	0.78 (0.38, 1.49)	0.58 (0.23, 1.42)	0.88 (0.39, 2.00)	0.82 (0.35, 1.89)	0.93 (0.28, 2.75)	0.70 (0.24, 1.77)	**0.33 (0.10, 0.98)**	0.72 (0.26, 1.90)	0.49 (0.16, 1.42)	0.65 (0.25, 1.52)	0.70 (0.27, 1.64)	0.66 (0.25, 1.57)	0.56 (0.28, 1.01)	0.51 (0.13, 2.32)	0.51 (0.09, 2.21)	1.50 (0.51, 4.99)	1.00 (0.49, 1.86)	0.75 (0.27, 2.00)	1.09 (0.53, 2.01)
VED300mgIV	0.74 (0.30, 1.93)	1.38 (0.59, 3.63)	0.77 (0.22, 2.34)	2.08 (0.86, 5.45)	**0.66 (0.44, 0.96)**	**0.50 (0.25, 0.94)**	0.76 (0.40, 1.40)	0.69 (0.36, 1.31)	0.77 (0.29, 2.20)	0.60 (0.26, 1.47)	**0.28 (0.10, 0.74)**	0.60 (0.26, 1.52)	0.41 (0.18, 1.14)	0.54 (0.25, 1.14)	0.60 (0.29, 1.19)	0.55 (0.26, 1.17)	**0.47 (0.34, 0.65)**	0.43 (0.12, 1.82)	0.43 (0.09, 1.62)	1.29 (0.52, 3.63)	0.84 (0.56, 1.24)	0.65 (0.28, 1.47)	0.92 (0.62, 1.35)

	RIS600mgIV4_4	SEC10mg/kgIV	TES400mg200mgSC	TOF15mgPO_BID	TOF1mgPO_BID	TOF5mgPO_BID	UPA12mgPO_BID	UPA24mgPO	UPA24mgPO_BID	UPA3mgPO_BID	UPA45mgPO	UPA6mgPO_BID	UST130mgIV	UST1mg/kgIV	UST3mg/kgIV	UST4.5mg/kgIV	UST6mg/kg90mgIV/SC	UST6mg/kgIV	UST90mgSC	VED0_5mg/kgIV	VED2mg/kgIV	VED300mgIV
ABA10mg/kgIV	2.39 (0.80, 7.69)	1.28 (0.20, 8.36)	**3.48 (1.03, 12.76)**	1.09 (0.24, 6.68)	2.58 (0.68, 11.57)	1.95 (0.51, 9.23)	2.02 (0.40, 11.87)	1.14 (0.18, 8.72)	3.66 (0.79, 18.65)	1.48 (0.22, 8.46)	**2.97 (1.05, 9.51)**	**4.55 (1.08, 24.17)**	**2.89 (1.01, 9.23)**	**3.27 (1.04, 11.92)**	**3.42 (1.08, 11.83)**	2.88 (0.99, 9.19)	**4.85 (1.50, 18.68)**	**3.73 (1.31, 11.79)**	1.77 (0.42, 8.07)	2.38 (0.76, 8.82)	3.04 (0.96, 10.79)	**3.59 (1.21, 11.64)**
ABA30mg/kgIV	1.21 (0.40, 3.60)	0.63 (0.10, 3.75)	1.79 (0.49, 5.83)	0.55 (0.11, 2.85)	1.31 (0.34, 5.63)	1.00 (0.25, 4.24)	1.03 (0.18, 5.33)	0.60 (0.09, 3.48)	1.89 (0.43, 8.52)	0.72 (0.12, 3.68)	1.51 (0.51, 4.49)	2.30 (0.53, 9.97)	1.47 (0.49, 4.41)	1.69 (0.50, 5.30)	1.73 (0.51, 5.67)	1.47 (0.49, 4.54)	2.52 (0.75, 9.03)	1.91 (0.63, 5.61)	0.91 (0.21, 4.02)	1.21 (0.36, 4.25)	1.55 (0.48, 5.54)	1.83 (0.60, 5.53)
ABA3mg/kgIV	1.88 (0.68, 5.77)	1.00 (0.16, 6.33)	2.69 (0.90, 9.83)	0.90 (0.20, 4.34)	2.08 (0.57, 9.14)	1.56 (0.43, 6.46)	1.57 (0.33, 9.13)	0.97 (0.14, 5.66)	2.98 (0.69, 15.62)	1.15 (0.18, 6.09)	2.35 (0.88, 7.18)	3.60 (0.89, 18.01)	2.25 (0.85, 7.30)	2.54 (0.88, 9.39)	2.70 (0.93, 9.36)	2.30 (0.85, 7.17)	**3.94 (1.22, 13.14)**	**2.93 (1.13, 9.25)**	1.43 (0.34, 6.43)	1.92 (0.62, 6.86)	2.46 (0.69, 8.72)	**2.84 (1.05, 8.55)**
ADA160mg80mg40mgSC	0.51 (0.23, 1.08)	0.26 (0.06, 1.27)	0.73 (0.28, 1.82)	**0.24 (0.05, 0.92)**	0.55 (0.18, 1.65)	0.42 (0.13, 1.26)	0.42 (0.11, 1.82)	0.25 (0.05, 1.26)	0.79 (0.23, 3.00)	0.31 (0.06, 1.23)	0.63 (0.30, 1.28)	0.93 (0.30, 3.54)	0.61 (0.28, 1.31)	0.69 (0.29, 1.69)	0.71 (0.31, 1.66)	0.62 (0.28, 1.30)	1.05 (0.82, 1.34)	0.79 (0.37, 1.63)	0.38 (0.11, 1.31)	0.52 (0.20, 1.29)	0.64 (0.26, 1.61)	0.77 (0.35, 1.58)
ADA160mg80mg60mgSC	**0.25 (0.09, 0.55)**	**0.13 (0.02, 0.60)**	**0.36 (0.12, 0.97)**	**0.12 (0.03, 0.46)**	**0.27 (0.07, 0.91)**	**0.21 (0.06, 0.68)**	**0.21 (0.04, 0.86)**	**0.12 (0.02, 0.56)**	0.38 (0.10, 1.45)	**0.15 (0.02, 0.62)**	**0.31 (0.12, 0.66)**	0.45 (0.12, 1.70)	**0.30 (0.12, 0.66)**	**0.34 (0.12, 0.81)**	**0.35 (0.13, 0.86)**	**0.30 (0.11, 0.69)**	0.50 (0.17, 1.36)	**0.39 (0.15, 0.83)**	**0.18 (0.05, 0.68)**	**0.25 (0.08, 0.69)**	**0.31 (0.11, 0.82)**	**0.37 (0.14, 0.82)**
ADA160mg80mgSC	**0.52 (0.30, 0.91)**	0.28 (0.06, 1.25)	0.75 (0.37, 1.54)	**0.25 (0.06, 0.87)**	0.56 (0.21, 1.68)	0.43 (0.16, 1.21)	0.42 (0.12, 1.71)	0.25 (0.05, 1.21)	0.82 (0.27, 2.72)	0.31 (0.07, 1.20)	0.65 (0.39, 1.04)	0.97 (0.35, 3.21)	0.63 (0.37, 1.05)	0.71 (0.35, 1.44)	0.74 (0.38, 1.45)	0.63 (0.37, 1.08)	1.07 (0.51, 2.41)	0.82 (0.49, 1.33)	0.39 (0.13, 1.27)	0.53 (0.24, 1.21)	0.66 (0.32, 1.53)	0.79 (0.44, 1.34)
ADA40mg20mgSC	0.98 (0.45, 2.16)	0.52 (0.11, 3.00)	1.43 (0.59, 3.60)	0.47 (0.12, 1.76)	1.09 (0.35, 3.74)	0.83 (0.26, 2.77)	0.83 (0.21, 3.43)	0.48 (0.10, 2.42)	1.58 (0.44, 5.64)	0.59 (0.13, 2.49)	1.23 (0.62, 2.57)	1.86 (0.59, 6.78)	1.20 (0.58, 2.64)	1.36 (0.58, 3.45)	1.39 (0.61, 3.45)	1.21 (0.57, 2.60)	2.05 (0.82, 5.64)	1.54 (0.75, 3.26)	0.76 (0.22, 2.87)	1.00 (0.40, 2.77)	1.27 (0.51, 3.41)	1.48 (0.71, 3.25)
ADA80mg40mgSC	0.60 (0.31, 1.17)	0.33 (0.06, 1.55)	0.87 (0.40, 2.04)	0.29 (0.07, 1.18)	0.65 (0.23, 2.09)	0.49 (0.17, 1.60)	0.51 (0.13, 2.07)	0.29 (0.06, 1.55)	0.93 (0.30, 3.27)	0.36 (0.08, 1.53)	0.75 (0.41, 1.37)	1.11 (0.39, 4.22)	0.74 (0.39, 1.37)	0.82 (0.39, 1.90)	0.85 (0.41, 1.94)	0.74 (0.39, 1.41)	1.25 (0.54, 2.94)	0.94 (0.52, 1.75)	0.45 (0.14, 1.64)	0.61 (0.26, 1.46)	0.77 (0.34, 1.85)	0.91 (0.48, 1.76)
AMI0.4mgPO	2.03 (0.98, 4.44)	1.11 (0.19, 5.20)	**2.96 (1.24, 7.66)**	0.96 (0.26, 3.71)	2.21 (0.74, 7.35)	1.68 (0.57, 5.19)	1.71 (0.44, 7.48)	0.97 (0.18, 5.08)	3.22 (0.91, 12.19)	1.23 (0.25, 5.23)	**2.56 (1.31, 5.27)**	**3.82 (1.19, 14.80)**	**2.48 (1.19, 5.18)**	**2.79 (1.23, 6.54)**	**2.91 (1.29, 6.89)**	**2.50 (1.25, 5.30)**	**4.31 (1.68, 11.08)**	**3.19 (1.59, 6.66)**	1.55 (0.47, 5.89)	2.07 (0.84, 5.41)	**2.62 (1.05, 6.76)**	**3.09 (1.47, 6.64)**
AND150mgSC1.1	1.72 (0.62, 4.70)	0.90 (0.16, 5.14)	2.49 (0.82, 7.61)	0.81 (0.17, 3.57)	1.89 (0.51, 6.62)	1.44 (0.41, 5.01)	1.43 (0.27, 6.91)	0.82 (0.16, 4.67)	2.69 (0.62, 11.29)	1.09 (0.16, 4.71)	2.15 (0.80, 5.64)	3.31 (0.74, 14.00)	2.10 (0.74, 5.60)	2.33 (0.81, 6.93)	2.47 (0.83, 7.22)	2.08 (0.76, 5.71)	**3.55 (1.12, 11.87)**	2.69 (0.99, 7.09)	1.29 (0.31, 5.53)	1.73 (0.54, 5.60)	2.18 (0.66, 6.74)	2.61 (0.94, 6.82)
AND150mgSC2.2	1.37 (0.52, 3.62)	0.72 (0.13, 4.58)	2.03 (0.69, 5.90)	0.66 (0.14, 2.70)	1.52 (0.41, 5.08)	1.13 (0.32, 3.93)	1.14 (0.24, 5.76)	0.66 (0.13, 3.93)	2.10 (0.56, 9.53)	0.83 (0.14, 3.96)	1.73 (0.65, 4.39)	2.66 (0.67, 11.16)	1.67 (0.62, 4.33)	1.91 (0.67, 5.20)	1.98 (0.70, 5.64)	1.70 (0.62, 4.43)	2.91 (0.90, 8.95)	2.17 (0.83, 5.56)	1.04 (0.28, 4.38)	1.44 (0.46, 4.52)	1.79 (0.55, 5.44)	2.11 (0.79, 5.39)
AND300mgSC	2.65 (0.86, 8.25)	1.35 (0.23, 9.69)	**3.81 (1.15, 14.30)**	1.24 (0.25, 6.61)	2.89 (0.70, 12.86)	2.14 (0.57, 9.30)	2.16 (0.41, 11.66)	1.23 (0.23, 8.99)	4.04 (0.91, 20.52)	1.60 (0.25, 8.81)	**3.34 (1.09, 10.11)**	**4.96 (1.11, 25.53)**	**3.17 (1.02, 9.77)**	**3.58 (1.10, 12.51)**	**3.76 (1.15, 12.73)**	**3.25 (1.04, 10.58)**	**5.34 (1.57, 20.43)**	**4.14 (1.34, 12.57)**	1.97 (0.45, 9.29)	2.69 (0.75, 10.28)	3.30 (0.97, 12.68)	**4.02 (1.30, 12.35)**
API100mgPO	1.91 (0.84, 4.64)	1.02 (0.19, 5.20)	**2.77 (1.06, 7.79)**	0.90 (0.22, 3.43)	2.11 (0.69, 7.32)	1.57 (0.51, 5.58)	1.62 (0.37, 6.65)	0.93 (0.17, 4.86)	3.06 (0.85, 11.18)	1.18 (0.22, 4.86)	**2.39 (1.10, 5.57)**	**3.63 (1.09, 13.94)**	**2.32 (1.08, 5.33)**	**2.57 (1.07, 6.92)**	**2.71 (1.14, 7.29)**	**2.34 (1.06, 5.55)**	**3.97 (1.44, 11.13)**	**2.98 (1.35, 6.91)**	1.42 (0.42, 5.61)	1.95 (0.68, 5.59)	2.43 (0.90, 7.10)	**2.88 (1.29, 6.90)**
API50mgPO	**2.65 (1.06, 6.59)**	1.44 (0.27, 7.74)	**3.84 (1.38, 11.81)**	1.25 (0.30, 6.34)	2.90 (0.88, 10.62)	2.19 (0.67, 8.56)	2.18 (0.48, 11.20)	1.32 (0.22, 7.96)	4.28 (0.95, 17.69)	1.62 (0.30, 7.86)	**3.29 (1.42, 8.10)**	**5.13 (1.31, 22.89)**	**3.19 (1.36, 8.07)**	**3.58 (1.40, 11.14)**	**3.80 (1.41, 10.35)**	**3.26 (1.36, 8.05)**	**5.58 (1.87, 16.88)**	**4.13 (1.76, 10.27)**	2.03 (0.55, 8.08)	2.76 (0.90, 8.14)	**3.44 (1.15, 10.36)**	**4.01 (1.67, 9.80)**
BRI400mgIV	0.50 (0.17, 1.33)	0.26 (0.05, 1.47)	0.72 (0.23, 2.26)	0.24 (0.05, 1.03)	0.56 (0.12, 1.88)	0.42 (0.10, 1.46)	0.42 (0.09, 1.91)	0.24 (0.04, 1.35)	0.78 (0.18, 3.35)	0.30 (0.05, 1.45)	0.63 (0.21, 1.60)	0.95 (0.23, 3.95)	0.60 (0.21, 1.61)	0.69 (0.22, 2.01)	0.72 (0.22, 2.12)	0.62 (0.21, 1.58)	1.03 (0.33, 3.07)	0.78 (0.28, 2.03)	0.37 (0.10, 1.46)	0.51 (0.16, 1.60)	0.64 (0.20, 2.06)	0.76 (0.26, 2.01)
BRI700mgIV	0.64 (0.22, 1.59)	0.33 (0.06, 1.72)	0.94 (0.30, 2.59)	0.30 (0.06, 1.33)	0.72 (0.17, 2.25)	0.54 (0.13, 1.77)	0.53 (0.12, 2.45)	0.31 (0.05, 1.66)	0.99 (0.24, 4.13)	0.38 (0.07, 1.75)	0.81 (0.28, 1.87)	1.19 (0.31, 4.70)	0.79 (0.26, 1.86)	0.88 (0.28, 2.29)	0.92 (0.29, 2.43)	0.78 (0.27, 1.91)	1.33 (0.39, 3.75)	1.01 (0.34, 2.32)	0.47 (0.13, 1.86)	0.66 (0.19, 1.91)	0.82 (0.26, 2.38)	0.98 (0.32, 2.31)
BRO210mgIV	3.56 (0.46, 39.35)	1.88 (0.15, 30.34)	5.26 (0.59, 61.45)	1.58 (0.16, 23.14)	3.77 (0.39, 49.82)	3.06 (0.28, 38.38)	3.15 (0.23, 36.42)	1.85 (0.10, 26.76)	5.70 (0.54, 62.35)	2.16 (0.17, 25.38)	4.38 (0.58, 48.92)	7.07 (0.62, 76.77)	4.34 (0.53, 50.27)	4.89 (0.57, 53.94)	5.07 (0.60, 58.07)	4.34 (0.55, 47.50)	7.35 (0.85, 84.30)	5.54 (0.70, 64.08)	2.57 (0.27, 39.31)	3.61 (0.39, 44.51)	4.43 (0.50, 55.39)	5.32 (0.66, 58.40)
BRO350mgIV	0.56 (0.08, 2.21)	0.28 (0.03, 2.07)	0.79 (0.10, 3.73)	0.24 (0.03, 1.82)	0.58 (0.08, 3.30)	0.44 (0.06, 2.34)	0.44 (0.04, 3.13)	0.26 (0.02, 1.96)	0.86 (0.09, 5.14)	0.32 (0.03, 1.96)	0.70 (0.10, 2.73)	1.05 (0.10, 5.85)	0.69 (0.09, 2.59)	0.77 (0.10, 3.17)	0.80 (0.10, 3.30)	0.69 (0.09, 2.66)	1.17 (0.14, 5.05)	0.90 (0.11, 3.33)	0.42 (0.05, 2.50)	0.54 (0.07, 2.58)	0.71 (0.09, 3.15)	0.84 (0.11, 3.39)
BRO700mgIV	0.92 (0.12, 6.35)	0.49 (0.05, 4.92)	1.35 (0.16, 10.36)	0.41 (0.05, 4.54)	1.00 (0.12, 11.12)	0.76 (0.09, 8.15)	0.80 (0.07, 7.18)	0.46 (0.04, 4.03)	1.48 (0.14, 12.59)	0.55 (0.06, 5.52)	1.17 (0.15, 7.87)	1.85 (0.18, 11.98)	1.17 (0.15, 7.70)	1.31 (0.17, 9.41)	1.36 (0.17, 9.48)	1.15 (0.14, 7.71)	1.93 (0.22, 13.81)	1.49 (0.19, 10.16)	0.74 (0.07, 4.79)	0.93 (0.12, 7.27)	1.18 (0.14, 8.36)	1.43 (0.17, 9.63)
CDP10mg/kgIV	1.38 (0.81, 2.33)	0.74 (0.16, 3.31)	**2.00 (1.01, 4.18)**	0.65 (0.17, 2.28)	1.51 (0.58, 4.52)	1.16 (0.44, 3.40)	1.12 (0.32, 4.52)	0.67 (0.14, 3.22)	2.13 (0.72, 7.13)	0.83 (0.18, 3.18)	**1.73 (1.09, 2.71)**	2.53 (0.98, 8.80)	1.68 (1.00, 2.72)	1.90 (0.98, 3.68)	**1.98 (1.03, 3.73)**	**1.69 (1.01, 2.80)**	**2.83 (1.38, 6.30)**	**2.16 (1.32, 3.44)**	1.04 (0.36, 3.33)	1.38 (0.66, 3.17)	1.75 (0.84, 3.95)	**2.09 (1.25, 3.44)**
CER100mgSC	1.24 (0.67, 2.29)	0.65 (0.12, 3.04)	1.78 (0.86, 4.14)	0.59 (0.15, 2.15)	1.34 (0.50, 3.88)	1.03 (0.38, 2.85)	1.03 (0.26, 3.95)	0.60 (0.13, 2.85)	1.93 (0.61, 6.52)	0.74 (0.17, 2.90)	1.54 (0.90, 2.70)	2.33 (0.82, 8.06)	1.50 (0.84, 2.65)	1.70 (0.83, 3.48)	1.77 (0.88, 3.58)	1.51 (0.84, 2.76)	**2.56 (1.15, 5.85)**	**1.93 (1.12, 3.40)**	0.93 (0.29, 3.16)	1.24 (0.57, 3.18)	1.57 (0.68, 3.80)	**1.86 (1.05, 3.43)**
CER10mg/kgIV	2.01 (0.70, 6.79)	1.11 (0.18, 6.35)	2.93 (0.90, 11.16)	0.96 (0.20, 5.73)	2.25 (0.56, 9.14)	1.68 (0.41, 7.31)	1.66 (0.38, 9.93)	0.97 (0.16, 6.33)	3.07 (0.77, 17.14)	1.21 (0.19, 7.53)	2.48 (0.90, 8.44)	3.73 (0.96, 19.55)	2.41 (0.86, 8.19)	2.72 (0.90, 9.81)	2.88 (0.92, 10.31)	2.45 (0.84, 8.28)	**4.21 (1.25, 16.25)**	**3.15 (1.12, 10.60)**	1.50 (0.38, 7.49)	2.07 (0.59, 8.32)	2.59 (0.79, 10.52)	**3.02 (1.07, 10.31)**
CER200mgSC	1.73 (0.89, 3.49)	0.92 (0.18, 4.28)	**2.50 (1.14, 6.10)**	0.82 (0.21, 3.21)	1.90 (0.65, 6.31)	1.41 (0.51, 4.61)	1.47 (0.37, 5.78)	0.83 (0.18, 4.30)	2.69 (0.84, 10.06)	1.04 (0.23, 4.07)	**2.15 (1.20, 4.04)**	**3.20 (1.09, 12.35)**	**2.09 (1.11, 4.08)**	**2.37 (1.14, 5.24)**	**2.47 (1.18, 5.51)**	**2.11 (1.14, 4.16)**	**3.62 (1.55, 8.44)**	**2.70 (1.48, 5.20)**	1.33 (0.40, 4.24)	1.76 (0.76, 4.24)	2.24 (0.99, 5.17)	**2.59 (1.40, 5.21)**

(TABLE S12A. continued)

	RIS600mgIV4_4	SEC10mg/kgIV	TES400mg200mgSC	TOF15mgPO_BID	TOF1mgPO_BID	TOF5mgPO_BID	UPA12mgPO_BID	UPA24mgPO	UPA24mgPO_BID	UPA3mgPO_BID	UPA45mgPO	UPA6mgPO_BID	UST130mgIV	UST1mg/kgIV	UST3mg/kgIV	UST4.5mg/kgIV	UST6mg/kg90mgIV/SC	UST6mg/kgIV	UST90mgSC	VED0_5mg/kgIV	VED2mg/kgIV	VED300mgIV
CER20mg/kgIV	**3.62 (1.18, 18.14)**	1.97 (0.26, 15.02)	**5.35 (1.53, 28.55)**	1.74 (0.34, 13.20)	4.06 (0.98, 25.81)	3.12 (0.70, 16.78)	3.10 (0.61, 25.43)	1.79 (0.28, 16.51)	**5.54 (1.31, 44.89)**	2.19 (0.31, 15.10)	**4.55 (1.47, 21.25)**	**6.79 (1.67, 55.34)**	**4.43 (1.39, 21.39)**	**5.09 (1.41, 26.23)**	**5.15 (1.46, 26.70)**	**4.47 (1.47, 22.32)**	**7.73 (2.07, 38.15)**	**5.73 (1.86, 27.46)**	2.87 (0.60, 18.67)	**3.77 (1.04, 20.03)**	**4.80 (1.28, 24.60)**	**5.49 (1.78, 27.41)**
CER400mgSC	1.14 (0.75, 1.76)	0.61 (0.12, 2.58)	1.65 (0.91, 3.19)	0.54 (0.15, 1.77)	1.25 (0.49, 3.55)	0.95 (0.38, 2.58)	0.95 (0.27, 3.51)	0.55 (0.12, 2.50)	1.77 (0.63, 5.62)	0.69 (0.16, 2.49)	**1.43 (1.06, 1.95)**	2.12 (0.83, 6.74)	1.38 (0.95, 1.99)	1.56 (0.89, 2.81)	1.62 (0.95, 2.86)	1.40 (0.96, 2.07)	**2.36 (1.23, 4.86)**	**1.79 (1.26, 2.52)**	0.87 (0.30, 2.55)	1.15 (0.59, 2.51)	1.46 (0.77, 3.08)	**1.72 (1.17, 2.55)**
CER5mg/kgIV	1.42 (0.50, 3.42)	0.74 (0.14, 3.90)	2.04 (0.73, 5.59)	0.66 (0.15, 2.94)	1.54 (0.43, 5.48)	1.20 (0.32, 4.07)	1.15 (0.27, 5.52)	0.68 (0.12, 3.97)	2.20 (0.52, 9.30)	0.85 (0.15, 4.01)	1.79 (0.66, 4.13)	2.59 (0.73, 10.98)	1.74 (0.61, 4.01)	1.94 (0.65, 4.93)	2.04 (0.70, 5.13)	1.74 (0.66, 4.07)	**2.90 (1.02, 8.40)**	2.24 (0.81, 5.09)	1.06 (0.27, 4.12)	1.45 (0.43, 4.21)	1.81 (0.59, 5.52)	2.12 (0.82, 5.07)
ELD10mg/kgIV	1.27 (0.48, 3.33)	0.67 (0.12, 3.66)	1.86 (0.62, 5.50)	0.58 (0.14, 3.11)	1.39 (0.41, 5.09)	1.04 (0.30, 3.90)	1.04 (0.23, 5.57)	0.61 (0.09, 4.72)	1.93 (0.50, 9.13)	0.77 (0.15, 4.36)	1.58 (0.62, 4.08)	2.35 (0.65, 12.03)	1.55 (0.58, 3.99)	1.73 (0.61, 5.36)	1.84 (0.62, 5.55)	1.54 (0.60, 3.96)	2.65 (0.87, 8.05)	2.00 (0.77, 5.17)	0.95 (0.25, 4.02)	1.31 (0.42, 3.83)	1.62 (0.53, 4.85)	1.92 (0.72, 5.02)
ELD20mg/kgIV	0.96 (0.38, 2.14)	0.52 (0.09, 2.62)	1.39 (0.49, 3.72)	0.44 (0.11, 2.17)	1.03 (0.31, 4.20)	0.79 (0.23, 3.17)	0.79 (0.18, 3.68)	0.46 (0.08, 2.66)	1.47 (0.42, 5.92)	0.57 (0.12, 2.62)	1.21 (0.50, 2.61)	1.74 (0.54, 7.50)	1.16 (0.47, 2.57)	1.31 (0.47, 3.54)	1.39 (0.50, 3.46)	1.18 (0.48, 2.63)	1.99 (0.64, 5.69)	1.50 (0.62, 3.27)	0.71 (0.19, 2.73)	0.99 (0.32, 2.68)	1.23 (0.41, 3.34)	1.45 (0.57, 3.24)
ETA25mgSC	2.72 (0.74, 13.11)	1.44 (0.22, 11.82)	3.94 (0.96, 20.59)	1.34 (0.22, 8.35)	3.00 (0.64, 17.33)	2.27 (0.47, 12.83)	2.30 (0.43, 15.51)	1.33 (0.20, 10.69)	4.36 (0.89, 29.10)	1.69 (0.25, 11.86)	3.44 (0.94, 15.82)	**5.34 (1.10, 33.70)**	3.34 (0.89, 15.52)	3.76 (0.94, 19.28)	**3.90 (1.01, 19.93)**	3.38 (0.93, 15.96)	**5.79 (1.37, 29.72)**	**4.25 (1.18, 19.93)**	2.12 (0.43, 11.07)	2.84 (0.66, 13.88)	3.58 (0.89, 17.28)	**4.17 (1.11, 19.23)**
ETR105mgSC	1.46 (0.89, 2.45)	0.78 (0.15, 3.37)	**2.13 (1.09, 4.46)**	0.69 (0.19, 2.39)	1.58 (0.60, 4.83)	1.20 (0.47, 3.54)	1.21 (0.34, 4.65)	0.71 (0.15, 3.28)	2.31 (0.78, 7.68)	0.89 (0.20, 3.28)	**1.84 (1.20, 2.85)**	2.75 (0.99, 9.25)	**1.77 (1.12, 2.85)**	**2.00 (1.09, 3.86)**	**2.09 (1.12, 3.92)**	**1.81 (1.11, 2.92)**	**3.05 (1.48, 6.57)**	**2.31 (1.46, 3.59)**	1.11 (0.38, 3.58)	1.48 (0.71, 3.33)	1.88 (0.93, 4.12)	**2.21 (1.33, 3.71)**
ETR210mgSC	1.28 (0.77, 2.14)	0.69 (0.13, 2.89)	1.86 (0.95, 3.83)	0.60 (0.17, 2.07)	1.38 (0.54, 4.05)	1.05 (0.40, 3.06)	1.06 (0.30, 4.12)	0.62 (0.13, 2.89)	2.01 (0.67, 6.66)	0.76 (0.18, 2.89)	**1.61 (1.04, 2.47)**	2.40 (0.87, 8.06)	1.55 (0.98, 2.46)	1.75 (0.94, 3.31)	1.82 (0.98, 3.43)	1.58 (0.96, 2.55)	**2.65 (1.28, 5.73)**	**2.00 (1.29, 3.12)**	0.95 (0.34, 3.19)	1.30 (0.62, 2.89)	1.64 (0.81, 3.61)	**1.93 (1.19, 3.20)**
FIL100mgPO	0.77 (0.48, 1.25)	0.42 (0.08, 1.82)	1.11 (0.58, 2.25)	0.36 (0.10, 1.25)	0.84 (0.34, 2.49)	0.64 (0.25, 1.82)	0.65 (0.18, 2.41)	0.37 (0.08, 1.73)	1.21 (0.41, 3.90)	0.47 (0.11, 1.75)	0.97 (0.65, 1.44)	1.43 (0.56, 4.69)	0.94 (0.61, 1.46)	1.06 (0.58, 2.00)	1.11 (0.61, 2.03)	0.95 (0.61, 1.50)	1.60 (0.81, 3.39)	1.21 (0.79, 1.86)	0.59 (0.21, 1.87)	0.78 (0.38, 1.68)	0.99 (0.51, 2.10)	1.17 (0.74, 1.90)
FIL200mgPO	0.93 (0.59, 1.48)	0.50 (0.10, 2.10)	1.34 (0.72, 2.72)	0.44 (0.12, 1.48)	1.01 (0.41, 2.77)	0.77 (0.31, 2.08)	0.78 (0.22, 2.97)	0.45 (0.10, 2.10)	1.45 (0.48, 4.73)	0.56 (0.13, 2.12)	1.17 (0.80, 1.69)	1.73 (0.67, 5.72)	1.13 (0.74, 1.73)	1.28 (0.70, 2.35)	1.33 (0.74, 2.42)	1.15 (0.75, 1.73)	1.92 (0.99, 4.01)	1.46 (0.97, 2.16)	0.71 (0.25, 2.20)	0.94 (0.47, 2.03)	1.19 (0.61, 2.50)	1.41 (0.90, 2.19)
FON0.1mg/kgIV	3.60 (0.54, 54.18)	1.79 (0.16, 43.73)	5.18 (0.69, 79.15)	1.63 (0.19, 31.07)	3.90 (0.50, 62.76)	3.04 (0.38, 49.05)	3.06 (0.34, 53.89)	1.85 (0.19, 33.00)	5.66 (0.67, 87.00)	2.22 (0.20, 31.65)	4.59 (0.68, 65.20)	7.04 (0.83, 111.28)	4.36 (0.63, 61.28)	4.93 (0.68, 72.45)	5.10 (0.71, 76.07)	4.42 (0.63, 65.97)	**7.31 (1.02, 119.18)**	5.63 (0.81, 81.06)	2.89 (0.30, 49.40)	3.72 (0.46, 52.53)	4.79 (0.59, 62.91)	5.40 (0.78, 80.96)
FON1mg/kg0.1mgIV/SC	1.27 (0.52, 3.31)	0.69 (0.13, 3.67)	1.85 (0.68, 5.25)	0.60 (0.13, 2.56)	1.38 (0.42, 4.62)	1.07 (0.30, 3.55)	1.08 (0.25, 4.54)	0.62 (0.12, 3.61)	1.97 (0.57, 7.87)	0.77 (0.15, 3.35)	1.60 (0.70, 3.81)	2.36 (0.68, 9.86)	1.55 (0.66, 3.75)	1.72 (0.68, 4.71)	1.79 (0.71, 4.83)	1.57 (0.65, 3.89)	2.64 (0.94, 8.09)	1.99 (0.87, 5.02)	0.96 (0.27, 4.21)	1.30 (0.46, 3.86)	1.65 (0.59, 4.73)	1.93 (0.79, 5.05)
FON1mg/kg1mgIV/SC	1.01 (0.40, 2.37)	0.52 (0.10, 2.64)	1.46 (0.53, 3.90)	0.46 (0.11, 2.04)	1.09 (0.34, 4.12)	0.84 (0.25, 3.03)	0.84 (0.20, 3.48)	0.50 (0.09, 2.83)	1.56 (0.40, 6.07)	0.62 (0.11, 2.49)	1.27 (0.53, 2.73)	1.83 (0.51, 8.03)	1.22 (0.51, 2.73)	1.41 (0.51, 3.40)	1.45 (0.53, 3.53)	1.24 (0.50, 2.79)	2.10 (0.73, 5.59)	1.58 (0.67, 3.48)	0.76 (0.22, 2.79)	1.01 (0.36, 2.83)	1.28 (0.46, 3.52)	1.53 (0.60, 3.35)
FON1mg/kgIV	1.67 (0.41, 7.56)	0.92 (0.12, 7.73)	2.52 (0.54, 11.94)	0.78 (0.13, 5.21)	1.90 (0.36, 10.89)	1.42 (0.28, 7.65)	1.42 (0.25, 10.05)	0.84 (0.13, 6.71)	2.65 (0.55, 16.32)	1.01 (0.17, 6.71)	2.12 (0.53, 9.11)	3.19 (0.68, 20.13)	2.02 (0.51, 9.38)	2.34 (0.54, 11.62)	2.42 (0.55, 11.88)	2.07 (0.51, 9.30)	3.55 (0.81, 17.49)	2.63 (0.65, 11.88)	1.33 (0.23, 8.03)	1.71 (0.39, 9.07)	2.17 (0.49, 11.58)	2.55 (0.64, 10.94)
FON4mg/kg0.1mgIV/SC	0.82 (0.35, 1.77)	0.44 (0.07, 2.23)	1.19 (0.46, 2.99)	0.39 (0.08, 1.63)	0.89 (0.28, 2.89)	0.68 (0.20, 2.21)	0.69 (0.17, 2.88)	0.39 (0.08, 2.13)	1.27 (0.35, 4.67)	0.50 (0.10, 2.05)	1.02 (0.46, 2.09)	1.52 (0.46, 5.92)	0.98 (0.45, 2.18)	1.11 (0.45, 2.77)	1.15 (0.47, 2.88)	1.01 (0.43, 2.16)	1.69 (0.65, 4.48)	1.27 (0.57, 2.74)	0.62 (0.17, 2.26)	0.83 (0.31, 2.32)	1.04 (0.40, 2.80)	1.25 (0.55, 2.62)
FON4mg/kg1mgIV/SC	1.00 (0.39, 2.34)	0.52 (0.10, 2.91)	1.44 (0.53, 3.78)	0.47 (0.10, 2.05)	1.08 (0.34, 4.30)	0.84 (0.22, 2.87)	0.83 (0.20, 3.43)	0.48 (0.09, 2.42)	1.55 (0.42, 5.95)	0.59 (0.12, 2.49)	1.25 (0.55, 2.81)	1.87 (0.53, 7.07)	1.19 (0.52, 2.77)	1.36 (0.51, 3.42)	1.42 (0.54, 3.53)	1.22 (0.50, 2.80)	2.03 (0.75, 5.60)	1.55 (0.66, 3.46)	0.74 (0.20, 2.78)	1.00 (0.36, 2.79)	1.26 (0.48, 3.53)	1.51 (0.62, 3.48)
FON4mg/kgIV	0.85 (0.22, 2.98)	0.44 (0.06, 3.77)	1.26 (0.28, 4.79)	0.39 (0.07, 2.45)	0.92 (0.19, 4.95)	0.70 (0.15, 3.45)	0.71 (0.13, 4.02)	0.42 (0.07, 2.84)	1.33 (0.27, 6.32)	0.51 (0.08, 2.63)	1.07 (0.27, 3.51)	1.63 (0.32, 8.10)	1.05 (0.28, 3.55)	1.18 (0.28, 4.21)	1.22 (0.30, 4.12)	1.07 (0.26, 3.63)	1.76 (0.42, 7.84)	1.36 (0.35, 4.54)	0.68 (0.12, 2.89)	0.86 (0.20, 3.78)	1.08 (0.27, 4.46)	1.29 (0.34, 4.07)
GUS1200mgIV	0.49 (0.22, 1.00)	0.26 (0.06, 1.16)	0.70 (0.28, 1.69)	**0.23 (0.05, 0.85)**	0.52 (0.18, 1.51)	0.41 (0.13, 1.16)	0.40 (0.11, 1.75)	0.23 (0.04, 1.15)	0.74 (0.22, 2.81)	0.29 (0.06, 1.14)	0.61 (0.29, 1.18)	0.90 (0.30, 3.36)	0.59 (0.27, 1.16)	0.66 (0.28, 1.50)	0.70 (0.29, 1.52)	0.59 (0.29, 1.22)	1.00 (0.67, 1.54)	0.76 (0.35, 1.49)	0.37 (0.11, 1.31)	0.50 (0.19, 1.21)	0.62 (0.25, 1.51)	0.74 (0.35, 1.46)
GUS200mgIV	**0.39 (0.18, 0.77)**	**0.21 (0.04, 0.92)**	0.56 (0.22, 1.33)	**0.18 (0.05, 0.68)**	0.42 (0.15, 1.21)	**0.32 (0.11, 0.92)**	0.32 (0.09, 1.36)	**0.19 (0.04, 0.91)**	0.60 (0.18, 2.26)	**0.23 (0.05, 0.91)**	**0.49 (0.24, 0.91)**	0.72 (0.24, 2.62)	**0.47 (0.22, 0.93)**	0.53 (0.24, 1.18)	0.55 (0.24, 1.20)	**0.47 (0.23, 0.95)**	0.80 (0.54, 1.17)	0.61 (0.29, 1.17)	0.29 (0.09, 1.01)	**0.39 (0.15, 0.96)**	0.49 (0.21, 1.19)	0.58 (0.28, 1.14)
GUS600mgIV	**0.40 (0.19, 0.80)**	**0.21 (0.04, 0.94)**	0.58 (0.24, 1.38)	**0.19 (0.05, 0.68)**	0.43 (0.15, 1.32)	0.33 (0.11, 1.00)	0.33 (0.09, 1.39)	**0.19 (0.04, 0.93)**	0.62 (0.18, 2.25)	**0.24 (0.05, 0.96)**	**0.50 (0.25, 0.95)**	0.74 (0.25, 2.67)	**0.48 (0.23, 0.99)**	0.54 (0.25, 1.19)	0.58 (0.25, 1.27)	**0.49 (0.24, 0.98)**	0.83 (0.56, 1.23)	0.62 (0.30, 1.21)	0.30 (0.09, 1.02)	0.40 (0.16, 1.00)	0.51 (0.22, 1.21)	0.60 (0.29, 1.20)
INF10mg/kgIV	0.17 (0.02, 1.09)	0.09 (0.01, 1.03)	0.25 (0.02, 1.69)	**0.08 (0.01, 0.72)**	0.18 (0.02, 1.48)	0.14 (0.01, 1.09)	0.14 (0.01, 1.40)	0.08 (0.01, 1.04)	0.26 (0.02, 2.33)	0.10 (0.01, 1.00)	0.21 (0.02, 1.37)	0.32 (0.03, 3.00)	0.21 (0.02, 1.32)	0.23 (0.02, 1.58)	0.24 (0.02, 1.65)	0.21 (0.02, 1.33)	0.35 (0.03, 2.52)	0.27 (0.03, 1.68)	0.12 (0.01, 1.19)	0.18 (0.02, 1.20)	0.22 (0.02, 1.54)	0.26 (0.03, 1.69)
INF20mg/kgIV	0.18 (0.02, 1.17)	0.09 (0.01, 1.10)	0.24 (0.02, 1.72)	**0.08 (0.01, 0.69)**	0.18 (0.02, 1.66)	0.14 (0.01, 1.24)	0.14 (0.01, 1.38)	**0.08 (0.01, 0.93)**	0.26 (0.02, 2.24)	**0.10 (0.01, 0.96)**	0.22 (0.02, 1.44)	0.32 (0.03, 2.82)	0.21 (0.02, 1.37)	0.23 (0.02, 1.64)	0.24 (0.03, 1.63)	0.21 (0.02, 1.43)	0.35 (0.03, 2.89)	0.27 (0.03, 1.76)	0.12 (0.01, 1.26)	0.18 (0.02, 1.43)	0.22 (0.02, 1.90)	0.26 (0.03, 1.72)
INF5mg/kgIV	**0.09 (0.01, 0.51)**	**0.04 (0.00, 0.50)**	**0.13 (0.01, 0.76)**	**0.04 (0.00, 0.33)**	**0.09 (0.01, 0.69)**	**0.07 (0.01, 0.53)**	**0.07 (0.01, 0.65)**	**0.04 (0.00, 0.45)**	0.13 (0.01, 1.00)	**0.05 (0.00, 0.45)**	**0.11 (0.01, 0.62)**	0.16 (0.01, 1.31)	**0.11 (0.01, 0.60)**	**0.12 (0.01, 0.74)**	**0.12 (0.01, 0.78)**	**0.11 (0.01, 0.62)**	0.18 (0.02, 1.23)	**0.14 (0.02, 0.77)**	**0.06 (0.01, 0.53)**	**0.09 (0.01, 0.58)**	**0.11 (0.01, 0.79)**	**0.14 (0.01, 0.77)**

	RIS600mgIV4_4	SEC10mg/kgIV	TES400mg200mgSC	TOF15mgPO_BID	TOF1mgPO_BID	TOF5mgPO_BID	UPA12mgPO_BID	UPA24mgPO	UPA24mgPO_BID	UPA3mgPO_BID	UPA45mgPO	UPA6mgPO_BID	UST130mgIV	UST1mg/kgIV	UST3mg/kgIV	UST4.5mg/kgIV	UST6mg/kg90mgIV/SC	UST6mg/kgIV	UST90mgSC	VED0_5mg/kgIV	VED2mg/kgIV	VED300mgIV
MED700mgIV	0.90 (0.34, 2.21)	0.48 (0.09, 2.52)	1.31 (0.47, 3.70)	0.42 (0.10, 2.12)	0.98 (0.28, 3.43)	0.75 (0.20, 2.79)	0.76 (0.17, 3.57)	0.44 (0.08, 2.57)	1.39 (0.35, 5.64)	0.55 (0.10, 2.62)	1.13 (0.44, 2.69)	1.67 (0.44, 6.86)	1.10 (0.42, 2.62)	1.26 (0.44, 3.39)	1.29 (0.46, 3.51)	1.10 (0.42, 2.69)	1.86 (0.59, 5.47)	1.42 (0.54, 3.30)	0.67 (0.18, 2.86)	0.93 (0.29, 2.76)	1.15 (0.39, 3.36)	1.36 (0.52, 3.36)
MIR1000mgIV	0.48 (0.18, 1.17)	0.25 (0.05, 1.31)	0.70 (0.23, 1.86)	**0.22 (0.05, 0.93)**	0.51 (0.14, 1.84)	0.39 (0.11, 1.45)	0.39 (0.09, 1.76)	0.23 (0.04, 1.24)	0.74 (0.19, 2.95)	0.28 (0.05, 1.22)	0.60 (0.24, 1.38)	0.88 (0.24, 3.52)	0.58 (0.22, 1.37)	0.65 (0.23, 1.69)	0.69 (0.25, 1.72)	0.59 (0.23, 1.37)	0.97 (0.33, 2.76)	0.75 (0.30, 1.75)	0.36 (0.09, 1.34)	0.48 (0.17, 1.42)	0.60 (0.22, 1.76)	0.72 (0.28, 1.68)
MIR200mgIV	0.87 (0.27, 3.18)	0.45 (0.07, 3.54)	1.25 (0.35, 5.05)	0.41 (0.08, 2.58)	0.95 (0.22, 5.71)	0.72 (0.17, 3.94)	0.75 (0.14, 3.90)	0.41 (0.07, 2.75)	1.35 (0.29, 7.23)	0.53 (0.10, 2.87)	1.09 (0.35, 3.82)	1.65 (0.36, 8.22)	1.05 (0.33, 4.35)	1.17 (0.35, 4.45)	1.25 (0.37, 4.80)	1.07 (0.32, 3.91)	1.82 (0.52, 7.59)	1.37 (0.42, 5.04)	0.67 (0.14, 2.99)	0.89 (0.25, 3.91)	1.13 (0.32, 4.32)	1.30 (0.43, 4.60)
MIR600mgIV	**0.32 (0.12, 0.78)**	**0.17 (0.03, 0.88)**	0.46 (0.16, 1.28)	**0.15 (0.03, 0.64)**	0.35 (0.09, 1.30)	0.27 (0.07, 1.01)	0.26 (0.06, 1.19)	**0.15 (0.03, 0.83)**	0.49 (0.13, 1.98)	**0.19 (0.04, 0.83)**	**0.39 (0.16, 0.94)**	0.59 (0.17, 2.30)	**0.38 (0.15, 0.92)**	0.43 (0.15, 1.27)	0.45 (0.16, 1.18)	**0.39 (0.15, 0.93)**	0.66 (0.23, 1.84)	0.49 (0.20, 1.20)	**0.24 (0.07, 0.92)**	**0.32 (0.11, 0.93)**	0.41 (0.13, 1.17)	0.48 (0.18, 1.16)
NAT300mgIV	1.00 (0.67, 1.54)	0.54 (0.12, 2.27)	1.45 (0.80, 2.80)	0.47 (0.13, 1.54)	1.10 (0.43, 3.09)	0.84 (0.33, 2.26)	0.83 (0.24, 3.14)	0.48 (0.10, 2.20)	1.56 (0.54, 5.04)	0.61 (0.14, 2.17)	1.26 (0.94, 1.70)	1.88 (0.73, 5.99)	1.22 (0.84, 1.74)	1.36 (0.79, 2.43)	1.44 (0.83, 2.49)	1.23 (0.85, 1.78)	**2.08 (1.10, 4.22)**	**1.57 (1.12, 2.18)**	0.76 (0.27, 2.23)	1.01 (0.53, 2.15)	1.28 (0.67, 2.64)	**1.52 (1.04, 2.25)**
NAT3mg/kgIV	1.34 (0.66, 2.72)	0.71 (0.13, 3.34)	1.95 (0.86, 4.67)	0.63 (0.16, 2.33)	1.45 (0.54, 4.50)	1.12 (0.40, 3.44)	1.12 (0.28, 4.74)	0.65 (0.13, 3.03)	2.07 (0.65, 7.93)	0.79 (0.17, 3.40)	1.66 (0.91, 3.24)	2.53 (0.83, 9.26)	1.61 (0.86, 3.20)	1.85 (0.87, 4.11)	1.90 (0.90, 4.30)	1.63 (0.85, 3.31)	**2.74 (1.21, 6.65)**	**2.08 (1.11, 4.11)**	1.00 (0.33, 3.72)	1.36 (0.56, 3.45)	1.72 (0.71, 4.39)	**2.02 (1.06, 4.07)**
NAT3mg/kgIVx2	0.88 (0.46, 1.66)	0.48 (0.10, 2.13)	1.26 (0.59, 2.94)	0.41 (0.11, 1.47)	0.96 (0.35, 2.81)	0.73 (0.26, 2.22)	0.72 (0.19, 2.94)	0.42 (0.09, 2.03)	1.36 (0.43, 4.84)	0.53 (0.11, 2.07)	1.09 (0.62, 1.96)	1.63 (0.56, 5.83)	1.06 (0.59, 1.93)	1.20 (0.59, 2.47)	1.26 (0.61, 2.58)	1.07 (0.58, 1.99)	1.82 (0.83, 4.06)	1.38 (0.76, 2.44)	0.66 (0.22, 2.25)	0.89 (0.39, 2.12)	1.13 (0.50, 2.55)	1.32 (0.72, 2.48)
NAT6mg/kgIVx2	0.96 (0.48, 1.85)	0.51 (0.10, 2.41)	1.38 (0.62, 3.24)	0.45 (0.12, 1.62)	1.05 (0.38, 3.29)	0.80 (0.28, 2.43)	0.80 (0.20, 3.36)	0.46 (0.09, 2.12)	1.48 (0.45, 5.58)	0.58 (0.12, 2.33)	1.19 (0.65, 2.18)	1.74 (0.59, 6.49)	1.16 (0.62, 2.18)	1.31 (0.61, 2.81)	1.38 (0.64, 2.87)	1.17 (0.62, 2.21)	1.98 (0.88, 4.56)	1.50 (0.80, 2.71)	0.71 (0.23, 2.52)	0.97 (0.40, 2.36)	1.22 (0.53, 2.87)	1.45 (0.77, 2.79)
NNC2mg/kgSC	0.85 (0.31, 2.27)	0.44 (0.07, 2.44)	1.23 (0.42, 3.84)	0.40 (0.09, 1.97)	0.92 (0.26, 3.62)	0.69 (0.19, 2.69)	0.71 (0.16, 3.15)	0.41 (0.06, 2.26)	1.31 (0.33, 5.29)	0.50 (0.09, 2.34)	1.07 (0.40, 2.67)	1.65 (0.44, 6.70)	1.04 (0.37, 2.71)	1.17 (0.39, 3.31)	1.22 (0.40, 3.45)	1.05 (0.38, 2.72)	1.76 (0.54, 5.75)	1.34 (0.49, 3.50)	0.64 (0.17, 2.56)	0.85 (0.27, 2.86)	1.07 (0.36, 3.54)	1.29 (0.45, 3.48)
ONE10mgSC	1.11 (0.48, 2.51)	0.61 (0.10, 3.26)	1.64 (0.62, 4.28)	0.51 (0.13, 2.07)	1.19 (0.39, 4.28)	0.93 (0.29, 3.30)	0.93 (0.23, 4.25)	0.52 (0.11, 3.20)	1.74 (0.44, 6.91)	0.66 (0.12, 3.16)	1.39 (0.61, 3.03)	2.09 (0.59, 8.48)	1.36 (0.61, 3.04)	1.55 (0.58, 4.02)	1.60 (0.59, 4.05)	1.38 (0.59, 3.07)	2.28 (0.88, 6.80)	1.74 (0.75, 3.89)	0.84 (0.23, 3.30)	1.12 (0.43, 3.21)	1.42 (0.57, 4.24)	1.67 (0.68, 3.88)
ONE25mgSC	2.39 (0.92, 6.44)	1.28 (0.23, 6.87)	**3.46 (1.18, 10.55)**	1.11 (0.23, 4.54)	2.67 (0.71, 9.95)	2.04 (0.53, 7.36)	1.98 (0.46, 8.94)	1.19 (0.21, 5.98)	3.71 (0.99, 14.85)	1.45 (0.22, 6.56)	**3.00 (1.18, 7.93)**	**4.54 (1.21, 17.87)**	**2.88 (1.13, 7.71)**	**3.30 (1.16, 10.64)**	**3.41 (1.18, 9.71)**	**2.94 (1.13, 7.94)**	**4.98 (1.68, 16.29)**	**3.75 (1.45, 9.60)**	1.87 (0.44, 7.33)	2.40 (0.78, 8.23)	**3.07 (1.02, 10.48)**	**3.63 (1.36, 9.78)**
ONE35mgSC	1.10 (0.46, 2.54)	0.59 (0.11, 2.96)	1.60 (0.58, 4.24)	0.51 (0.11, 2.16)	1.20 (0.36, 4.22)	0.91 (0.28, 3.27)	0.93 (0.23, 4.11)	0.52 (0.10, 3.11)	1.70 (0.45, 7.00)	0.65 (0.11, 3.14)	1.38 (0.58, 3.06)	2.05 (0.58, 9.03)	1.32 (0.55, 3.00)	1.49 (0.58, 3.99)	1.57 (0.61, 4.17)	1.35 (0.56, 3.05)	2.23 (0.82, 6.38)	1.72 (0.72, 3.83)	0.83 (0.23, 3.05)	1.10 (0.40, 3.08)	1.39 (0.53, 3.88)	1.66 (0.66, 3.81)
ONE50mgSC	1.61 (0.62, 3.82)	0.86 (0.15, 4.32)	2.35 (0.81, 6.52)	0.75 (0.17, 2.92)	1.73 (0.52, 6.11)	1.33 (0.38, 4.55)	1.34 (0.33, 5.36)	0.77 (0.15, 4.05)	2.53 (0.60, 9.52)	0.98 (0.16, 4.08)	2.01 (0.78, 4.64)	3.01 (0.75, 11.69)	1.94 (0.79, 4.61)	2.21 (0.77, 5.72)	2.27 (0.80, 6.17)	2.00 (0.78, 4.66)	**3.31 (1.15, 9.66)**	2.52 (1.00, 5.87)	1.25 (0.31, 4.68)	1.61 (0.53, 4.92)	2.05 (0.70, 6.30)	2.45 (0.88, 5.66)
ONT22_5mgSC	1.22 (0.56, 2.56)	0.65 (0.14, 3.25)	1.77 (0.74, 4.50)	0.58 (0.14, 2.63)	1.32 (0.43, 5.08)	1.01 (0.33, 3.78)	1.00 (0.26, 4.44)	0.60 (0.11, 3.26)	1.93 (0.53, 7.31)	0.72 (0.15, 3.28)	1.52 (0.76, 3.08)	2.23 (0.74, 8.54)	1.46 (0.72, 3.07)	1.66 (0.74, 4.01)	1.73 (0.78, 4.19)	1.48 (0.71, 3.19)	2.55 (0.99, 6.32)	1.90 (0.96, 3.86)	0.92 (0.28, 3.19)	1.23 (0.50, 3.50)	1.53 (0.66, 4.04)	1.84 (0.88, 4.04)
ONT225mgSC	1.11 (0.55, 2.33)	0.59 (0.13, 2.79)	1.61 (0.70, 3.94)	0.53 (0.13, 2.12)	1.23 (0.40, 4.22)	0.93 (0.31, 2.98)	0.93 (0.22, 3.70)	0.54 (0.10, 2.72)	1.75 (0.52, 6.52)	0.68 (0.13, 2.77)	1.40 (0.72, 2.75)	2.08 (0.68, 7.48)	1.35 (0.69, 2.72)	1.50 (0.70, 3.53)	1.58 (0.74, 3.68)	1.38 (0.69, 2.83)	2.30 (0.95, 5.87)	1.75 (0.91, 3.49)	0.85 (0.26, 2.81)	1.13 (0.48, 3.01)	1.42 (0.61, 3.74)	1.67 (0.84, 3.50)
ONT75mgSC	1.19 (0.56, 2.57)	0.64 (0.14, 3.05)	1.72 (0.73, 4.30)	0.56 (0.13, 2.36)	1.30 (0.41, 4.38)	0.99 (0.32, 3.33)	1.01 (0.24, 4.14)	0.58 (0.11, 2.93)	1.87 (0.53, 7.30)	0.72 (0.15, 3.06)	1.50 (0.74, 3.00)	2.23 (0.72, 8.62)	1.45 (0.68, 2.98)	1.63 (0.70, 4.19)	1.69 (0.74, 4.00)	1.47 (0.70, 3.12)	2.52 (0.98, 6.65)	1.86 (0.91, 3.82)	0.91 (0.28, 2.96)	1.22 (0.48, 3.28)	1.52 (0.64, 4.03)	1.81 (0.85, 3.90)
PBO	1.39 (0.98, 2.05)	0.75 (0.16, 3.07)	**2.01 (1.14, 3.74)**	0.66 (0.19, 2.15)	1.53 (0.62, 4.24)	1.17 (0.48, 3.12)	1.16 (0.34, 4.28)	0.67 (0.14, 2.97)	2.17 (0.78, 6.73)	0.85 (0.20, 3.01)	**1.75 (1.41, 2.21)**	**2.61 (1.03, 8.12)**	**1.69 (1.27, 2.29)**	**1.91 (1.14, 3.31)**	**1.99 (1.20, 3.33)**	**1.72 (1.26, 2.35)**	**2.87 (1.57, 5.71)**	**2.19 (1.69, 2.84)**	1.05 (0.39, 3.15)	1.40 (0.76, 2.89)	1.79 (0.99, 3.57)	**2.11 (1.53, 2.94)**
PF10mgSC	1.55 (0.37, 5.38)	0.78 (0.11, 6.02)	2.23 (0.56, 8.76)	0.74 (0.12, 3.90)	1.69 (0.35, 7.13)	1.27 (0.26, 5.46)	1.28 (0.22, 8.62)	0.78 (0.10, 5.46)	2.41 (0.38, 14.26)	0.89 (0.15, 5.41)	1.93 (0.48, 6.73)	2.83 (0.49, 16.38)	1.86 (0.46, 6.53)	2.11 (0.57, 7.78)	2.18 (0.47, 8.05)	1.90 (0.46, 6.62)	3.20 (0.74, 12.99)	2.42 (0.59, 8.28)	1.18 (0.20, 6.44)	1.57 (0.34, 6.58)	1.97 (0.43, 7.92)	2.31 (0.55, 8.12)
PF200mgSC	1.52 (0.43, 7.12)	0.83 (0.10, 6.18)	2.18 (0.56, 12.70)	0.72 (0.13, 4.80)	1.73 (0.35, 9.80)	1.29 (0.28, 7.23)	1.29 (0.21, 10.60)	0.76 (0.10, 7.79)	2.45 (0.47, 18.48)	0.95 (0.12, 7.44)	1.92 (0.56, 8.71)	2.95 (0.56, 23.06)	1.85 (0.56, 8.27)	2.09 (0.54, 10.09)	2.18 (0.56, 10.33)	1.88 (0.54, 9.12)	3.16 (0.83, 18.49)	2.38 (0.71, 10.68)	1.18 (0.21, 8.37)	1.59 (0.39, 8.94)	1.98 (0.45, 11.38)	2.32 (0.62, 11.41)
PF50mgSC	0.53 (0.18, 1.23)	0.27 (0.04, 1.48)	0.75 (0.24, 2.17)	**0.24 (0.04, 0.99)**	0.56 (0.15, 1.96)	0.42 (0.11, 1.42)	0.43 (0.09, 2.17)	0.25 (0.04, 1.41)	0.79 (0.19, 3.45)	0.30 (0.05, 1.43)	0.65 (0.23, 1.51)	0.94 (0.24, 4.16)	0.64 (0.22, 1.51)	0.72 (0.23, 1.85)	0.74 (0.24, 1.94)	0.64 (0.21, 1.54)	1.06 (0.34, 3.12)	0.82 (0.28, 1.93)	0.39 (0.10, 1.63)	0.53 (0.15, 1.65)	0.67 (0.20, 1.96)	0.78 (0.28, 1.91)
RIS1200mgIV	0.79 (0.52, 1.20)	0.42 (0.09, 1.79)	1.14 (0.62, 2.20)	0.37 (0.10, 1.23)	0.86 (0.34, 2.49)	0.66 (0.27, 1.84)	0.65 (0.19, 2.46)	0.38 (0.08, 1.75)	1.22 (0.43, 3.83)	0.47 (0.11, 1.74)	0.98 (0.73, 1.34)	1.47 (0.58, 4.70)	0.95 (0.67, 1.38)	1.08 (0.62, 1.97)	1.12 (0.64, 1.98)	0.96 (0.72, 1.30)	1.61 (0.86, 3.29)	1.23 (0.89, 1.73)	0.59 (0.22, 1.80)	0.79 (0.41, 1.70)	1.00 (0.54, 2.06)	1.19 (0.80, 1.77)
RIS200mgIV	1.03 (0.45, 2.45)	0.55 (0.10, 2.97)	1.50 (0.65, 4.02)	0.50 (0.12, 1.82)	1.15 (0.36, 3.74)	0.88 (0.29, 2.71)	0.86 (0.21, 3.90)	0.51 (0.11, 2.50)	1.63 (0.49, 6.21)	0.62 (0.11, 2.81)	1.29 (0.61, 2.84)	1.96 (0.54, 7.61)	1.24 (0.58, 2.77)	1.41 (0.65, 3.57)	1.47 (0.63, 3.60)	1.26 (0.60, 2.80)	2.15 (0.78, 5.83)	1.62 (0.75, 3.56)	0.79 (0.21, 2.97)	1.04 (0.38, 2.98)	1.34 (0.50, 3.69)	1.55 (0.68, 3.60)
RIS600mgIV	0.72 (0.48, 1.10)	0.38 (0.08, 1.63)	1.04 (0.58, 2.02)	0.34 (0.09, 1.13)	0.79 (0.31, 2.28)	0.60 (0.25, 1.68)	0.59 (0.17, 2.26)	0.35 (0.08, 1.58)	1.12 (0.40, 3.54)	0.44 (0.10, 1.59)	0.90 (0.68, 1.22)	1.36 (0.53, 4.28)	0.88 (0.61, 1.26)	0.99 (0.57, 1.81)	1.03 (0.59, 1.81)	0.89 (0.68, 1.15)	1.49 (0.79, 3.04)	1.13 (0.82, 1.57)	0.54 (0.20, 1.65)	0.72 (0.38, 1.54)	0.92 (0.50, 1.90)	1.09 (0.74, 1.62)

	RIS600mgIV4_4	SEC10mg/kgIV	TES400mg200mgSC	TOF15mgPO_BID	TOF1mgPO_BID	TOF5mgPO_BID	UPA12mgPO_BID	UPA24mgPO	UPA24mgPO_BID	UPA3mgPO_BID	UPA45mgPO	UPA6mgPO_BID	UST130mgIV	UST1mg/kgIV	UST3mg/kgIV	UST4.5mg/kgIV	UST6mg/kg90mgIV/SC	UST6mg/kgIV	UST90mgSC	VED0_5mg/kgIV	VED2mg/kgIV	VED300mgIV
RIS600mgIV4_4	RIS600mgIV4_4	0.54 (0.11, 2.28)	1.44 (0.74, 2.99)	0.47 (0.13, 1.65)	1.09 (0.40, 3.20)	0.83 (0.31, 2.36)	0.83 (0.23, 3.26)	0.48 (0.10, 2.22)	1.54 (0.52, 5.19)	0.61 (0.13, 2.25)	1.25 (0.81, 1.92)	1.86 (0.68, 6.09)	1.21 (0.75, 1.93)	1.37 (0.73, 2.60)	1.42 (0.76, 2.65)	1.23 (0.76, 1.98)	**2.05 (1.03, 4.48)**	**1.58 (1.02, 2.40)**	0.75 (0.26, 2.33)	1.02 (0.50, 2.26)	1.28 (0.64, 2.76)	1.51 (0.94, 2.51)
SEC10mg/kgIV	1.85 (0.44, 9.34)	SEC10mg/kgIV	2.76 (0.58, 15.25)	0.91 (0.13, 5.22)	2.10 (0.35, 10.99)	1.62 (0.27, 8.34)	1.60 (0.23, 11.66)	0.95 (0.11, 7.43)	2.97 (0.50, 17.51)	1.16 (0.12, 7.77)	2.34 (0.55, 11.18)	3.71 (0.64, 21.33)	2.24 (0.54, 11.36)	2.58 (0.54, 14.27)	2.69 (0.57, 13.14)	2.29 (0.53, 9.77)	3.92 (0.86, 17.94)	2.91 (0.69, 14.26)	1.48 (0.23, 9.19)	1.88 (0.41, 9.03)	2.43 (0.53, 11.14)	2.84 (0.64, 13.79)
TES400mg200mgSC	0.69 (0.33, 1.36)	0.36 (0.07, 1.72)	TES400mg200mgSC	0.32 (0.08, 1.18)	0.75 (0.25, 2.40)	0.57 (0.19, 1.73)	0.57 (0.14, 2.44)	0.33 (0.07, 1.65)	1.07 (0.33, 4.03)	0.42 (0.09, 1.72)	0.86 (0.45, 1.60)	1.29 (0.41, 4.79)	0.84 (0.42, 1.60)	0.95 (0.43, 2.05)	0.97 (0.44, 2.15)	0.85 (0.42, 1.62)	1.43 (0.60, 3.57)	1.08 (0.56, 2.06)	0.51 (0.16, 1.77)	0.70 (0.29, 1.78)	0.89 (0.38, 2.19)	1.04 (0.52, 2.05)
TOF15mgPO_BID	2.13 (0.61, 7.87)	1.10 (0.19, 7.72)	3.13 (0.85, 12.43)	TOF15mgPO_BID	2.29 (0.87, 8.11)	1.75 (0.63, 6.11)	1.75 (0.30, 10.97)	1.04 (0.14, 8.59)	3.30 (0.59, 19.95)	1.29 (0.15, 8.05)	2.65 (0.81, 9.60)	4.05 (0.79, 24.94)	2.59 (0.77, 9.13)	2.89 (0.84, 10.86)	3.06 (0.82, 11.73)	2.58 (0.78, 9.16)	**4.27 (1.18, 18.98)**	3.39 (1.00, 11.81)	1.60 (0.30, 8.90)	2.13 (0.52, 9.27)	2.69 (0.61, 11.92)	3.18 (0.95, 12.32)
TOF1mgPO_BID	0.91 (0.31, 2.47)	0.48 (0.09, 2.86)	1.32 (0.42, 4.02)	0.44 (0.12, 1.15)	TOF1mgPO_BID	0.76 (0.33, 1.65)	0.75 (0.17, 3.47)	0.45 (0.08, 2.52)	1.41 (0.31, 6.15)	0.56 (0.08, 2.60)	1.14 (0.40, 2.92)	1.72 (0.42, 7.37)	1.11 (0.41, 2.85)	1.25 (0.41, 3.54)	1.30 (0.43, 3.74)	1.12 (0.37, 2.87)	1.90 (0.66, 5.65)	1.45 (0.49, 3.63)	0.68 (0.17, 3.06)	0.93 (0.29, 2.92)	1.17 (0.36, 3.81)	1.38 (0.46, 3.62)
TOF5mgPO_BID	1.20 (0.42, 3.21)	0.62 (0.12, 3.67)	1.75 (0.58, 5.36)	0.57 (0.16, 1.59)	1.32 (0.61, 2.99)	TOF5mgPO_BID	1.00 (0.22, 4.78)	0.58 (0.11, 3.33)	1.82 (0.42, 8.12)	0.72 (0.10, 3.61)	1.50 (0.56, 3.78)	2.22 (0.57, 9.77)	1.47 (0.56, 3.81)	1.66 (0.55, 4.66)	1.72 (0.58, 4.75)	1.48 (0.52, 3.70)	2.49 (0.86, 7.59)	1.89 (0.70, 4.78)	0.90 (0.22, 3.87)	1.20 (0.38, 3.97)	1.53 (0.48, 4.92)	1.81 (0.65, 4.78)
UPA12mgPO_BID	1.21 (0.31, 4.29)	0.63 (0.09, 4.30)	1.75 (0.41, 6.96)	0.57 (0.09, 3.39)	1.33 (0.29, 6.03)	1.00 (0.21, 4.61)	UPA12mgPO_BID	0.60 (0.13, 2.30)	1.83 (0.69, 5.83)	0.71 (0.17, 2.40)	1.51 (0.41, 5.08)	2.27 (0.79, 6.64)	1.47 (0.38, 5.03)	1.67 (0.39, 6.47)	1.71 (0.42, 6.69)	1.47 (0.38, 5.28)	2.50 (0.59, 9.32)	1.91 (0.50, 6.56)	0.91 (0.19, 4.10)	1.23 (0.28, 5.23)	1.58 (0.35, 6.80)	1.82 (0.47, 6.53)
UPA24mgPO	2.07 (0.45, 9.85)	1.05 (0.13, 8.84)	3.00 (0.61, 14.49)	0.96 (0.12, 7.05)	2.22 (0.40, 12.16)	1.73 (0.30, 9.20)	1.67 (0.44, 7.49)	UPA24mgPO	3.12 (0.98, 13.51)	1.20 (0.28, 5.55)	2.63 (0.58, 12.35)	**3.67 (1.27, 15.56)**	2.51 (0.56, 11.39)	2.85 (0.58, 13.11)	2.96 (0.61, 13.37)	2.57 (0.54, 11.77)	4.19 (0.86, 21.05)	3.23 (0.75, 14.76)	1.53 (0.27, 9.39)	2.11 (0.41, 11.39)	2.63 (0.51, 13.59)	3.16 (0.69, 14.58)
UPA24mgPO_BID	0.65 (0.19, 1.92)	0.34 (0.06, 1.99)	0.94 (0.25, 3.08)	0.30 (0.05, 1.68)	0.71 (0.16, 3.21)	0.55 (0.12, 2.36)	0.55 (0.17, 1.46)	0.32 (0.07, 1.02)	UPA24mgPO_BID	0.39 (0.11, 1.03)	0.80 (0.25, 2.28)	1.21 (0.56, 2.65)	0.77 (0.24, 2.23)	0.88 (0.24, 2.80)	0.91 (0.26, 2.97)	0.79 (0.24, 2.31)	1.31 (0.36, 4.53)	1.01 (0.31, 2.89)	0.48 (0.11, 2.15)	0.66 (0.18, 2.23)	0.82 (0.23, 2.85)	0.97 (0.30, 2.84)
UPA3mgPO_BID	1.64 (0.44, 7.51)	0.86 (0.13, 8.49)	2.38 (0.58, 11.38)	0.77 (0.12, 6.52)	1.80 (0.38, 13.06)	1.38 (0.28, 9.94)	1.40 (0.42, 5.80)	0.84 (0.18, 3.58)	2.59 (0.97, 9.46)	UPA3mgPO_BID	2.05 (0.57, 8.89)	**3.19 (1.15, 10.68)**	2.01 (0.54, 9.04)	2.29 (0.56, 10.23)	2.32 (0.60, 11.16)	2.02 (0.55, 9.37)	3.41 (0.87, 15.88)	2.61 (0.70, 11.52)	1.26 (0.26, 7.24)	1.73 (0.41, 8.37)	2.18 (0.49, 10.43)	2.48 (0.69, 11.11)
UPA45mgPO	0.80 (0.52, 1.23)	0.43 (0.09, 1.81)	1.16 (0.63, 2.22)	0.38 (0.10, 1.23)	0.87 (0.34, 2.47)	0.66 (0.26, 1.80)	0.66 (0.20, 2.45)	0.38 (0.08, 1.73)	1.25 (0.44, 3.95)	0.49 (0.11, 1.76)	UPA45mgPO	1.48 (0.57, 4.71)	0.97 (0.67, 1.40)	1.09 (0.62, 1.95)	1.14 (0.66, 2.00)	0.98 (0.67, 1.43)	1.65 (0.86, 3.37)	1.25 (0.89, 1.75)	0.60 (0.22, 1.81)	0.80 (0.41, 1.69)	1.02 (0.53, 2.10)	1.21 (0.81, 1.79)
UPA6mgPO_BID	0.54 (0.16, 1.48)	0.27 (0.05, 1.57)	0.78 (0.21, 2.43)	0.25 (0.04, 1.26)	0.58 (0.14, 2.41)	0.45 (0.10, 1.74)	0.44 (0.15, 1.27)	**0.27 (0.06, 0.79)**	0.83 (0.38, 1.77)	**0.31 (0.09, 0.87)**	0.67 (0.21, 1.76)	UPA6mgPO_BID	0.65 (0.20, 1.74)	0.73 (0.20, 2.19)	0.77 (0.22, 2.28)	0.66 (0.20, 1.77)	1.12 (0.30, 3.42)	0.84 (0.26, 2.21)	0.40 (0.09, 1.62)	0.55 (0.14, 1.75)	0.70 (0.18, 2.21)	0.80 (0.25, 2.21)
UST130mgIV	0.82 (0.52, 1.33)	0.45 (0.09, 1.87)	1.19 (0.63, 2.36)	0.39 (0.11, 1.30)	0.90 (0.35, 2.46)	0.68 (0.26, 1.78)	0.68 (0.20, 2.61)	0.40 (0.09, 1.80)	1.29 (0.45, 4.24)	0.50 (0.11, 1.87)	1.03 (0.71, 1.49)	1.54 (0.57, 4.97)	UST130mgIV	1.13 (0.66, 1.93)	1.18 (0.70, 1.99)	1.01 (0.65, 1.56)	1.72 (0.84, 3.55)	**1.30 (1.01, 1.63)**	0.62 (0.21, 1.99)	0.84 (0.42, 1.80)	1.06 (0.53, 2.29)	1.24 (0.79, 2.00)
UST1mg/kgIV	0.73 (0.38, 1.37)	0.39 (0.07, 1.84)	1.06 (0.49, 2.31)	0.35 (0.09, 1.19)	0.80 (0.28, 2.42)	0.60 (0.21, 1.83)	0.60 (0.15, 2.57)	0.35 (0.08, 1.73)	1.14 (0.36, 4.16)	0.44 (0.10, 1.79)	0.92 (0.51, 1.60)	1.36 (0.46, 4.93)	0.88 (0.52, 1.52)	UST1mg/kgIV	1.03 (0.62, 1.80)	0.90 (0.48, 1.64)	1.51 (0.67, 3.42)	1.14 (0.70, 1.87)	0.55 (0.17, 1.86)	0.73 (0.32, 1.85)	0.94 (0.36, 2.29)	1.10 (0.59, 2.10)
UST3mg/kgIV	0.70 (0.38, 1.32)	0.37 (0.08, 1.75)	1.03 (0.46, 2.26)	0.33 (0.09, 1.23)	0.77 (0.27, 2.31)	0.58 (0.21, 1.74)	0.58 (0.15, 2.38)	0.34 (0.07, 1.65)	1.10 (0.34, 3.88)	0.43 (0.09, 1.68)	0.87 (0.50, 1.52)	1.31 (0.44, 4.58)	0.85 (0.50, 1.43)	0.97 (0.55, 1.61)	UST3mg/kgIV	0.86 (0.46, 1.59)	1.46 (0.65, 3.29)	1.10 (0.68, 1.77)	0.52 (0.17, 1.75)	0.70 (0.31, 1.75)	0.90 (0.39, 2.13)	1.05 (0.58, 1.94)
UST4.5mg/kgIV	0.81 (0.51, 1.32)	0.44 (0.10, 1.88)	1.18 (0.62, 2.35)	0.39 (0.11, 1.29)	0.89 (0.35, 2.67)	0.68 (0.27, 1.93)	0.68 (0.19, 2.61)	0.39 (0.08, 1.86)	1.27 (0.43, 4.18)	0.50 (0.11, 1.81)	1.02 (0.70, 1.50)	1.51 (0.57, 5.08)	0.99 (0.64, 1.54)	1.11 (0.61, 2.10)	1.17 (0.63, 2.16)	UST4.5mg/kgIV	1.68 (0.85, 3.60)	1.28 (0.85, 1.92)	0.61 (0.21, 1.87)	0.82 (0.41, 1.79)	1.04 (0.54, 2.18)	1.24 (0.77, 1.95)
UST6mg/kg90mgIV/SC	**0.49 (0.22, 0.97)**	0.26 (0.06, 1.16)	0.70 (0.28, 1.67)	**0.23 (0.05, 0.85)**	0.53 (0.18, 1.51)	0.40 (0.13, 1.17)	0.40 (0.11, 1.70)	0.24 (0.05, 1.17)	0.76 (0.22, 2.79)	0.29 (0.06, 1.16)	0.61 (0.30, 1.16)	0.89 (0.29, 3.29)	0.58 (0.28, 1.19)	0.66 (0.29, 1.50)	0.69 (0.30, 1.53)	0.59 (0.28, 1.18)	UST6mg/kg90mgIV/SC	0.76 (0.37, 1.49)	0.37 (0.11, 1.25)	0.49 (0.20, 1.24)	0.61 (0.26, 1.49)	0.74 (0.34, 1.45)
UST6mg/kgIV	**0.63 (0.42, 0.98)**	0.34 (0.07, 1.46)	0.92 (0.49, 1.80)	0.30 (0.08, 1.00)	0.69 (0.28, 2.03)	0.53 (0.21, 1.42)	0.52 (0.15, 2.00)	0.31 (0.07, 1.34)	0.99 (0.35, 3.27)	0.38 (0.09, 1.44)	0.80 (0.57, 1.12)	1.19 (0.45, 3.81)	**0.77 (0.61, 0.99)**	0.88 (0.54, 1.42)	0.91 (0.57, 1.46)	0.78 (0.52, 1.18)	1.32 (0.67, 2.71)	UST6mg/kgIV	0.48 (0.17, 1.49)	0.64 (0.33, 1.38)	0.81 (0.42, 1.76)	0.96 (0.64, 1.50)
UST90mgSC	1.33 (0.43, 3.85)	0.68 (0.11, 4.40)	1.95 (0.57, 6.24)	0.62 (0.11, 3.29)	1.47 (0.33, 5.98)	1.11 (0.26, 4.61)	1.10 (0.24, 5.26)	0.65 (0.11, 3.70)	2.08 (0.47, 9.35)	0.79 (0.14, 3.88)	1.67 (0.55, 4.54)	2.50 (0.62, 10.76)	1.62 (0.50, 4.68)	1.83 (0.54, 5.83)	1.92 (0.57, 5.75)	1.63 (0.53, 4.72)	2.73 (0.80, 8.85)	2.08 (0.67, 5.86)	UST90mgSC	1.33 (0.38, 4.76)	1.68 (0.51, 5.73)	2.01 (0.65, 5.77)
VED0_5mg/kgIV	0.99 (0.44, 2.02)	0.53 (0.11, 2.47)	1.43 (0.56, 3.49)	0.47 (0.11, 1.91)	1.08 (0.34, 3.40)	0.83 (0.25, 2.65)	0.82 (0.19, 3.55)	0.47 (0.09, 2.46)	1.51 (0.45, 5.70)	0.58 (0.12, 2.43)	1.25 (0.59, 2.42)	1.81 (0.57, 7.21)	1.20 (0.56, 2.40)	1.37 (0.54, 3.13)	1.42 (0.57, 3.24)	1.21 (0.56, 2.46)	2.02 (0.80, 5.10)	1.57 (0.72, 3.08)	0.75 (0.21, 2.66)	VED0_5mg/kgIV	1.26 (0.76, 2.17)	1.51 (0.67, 3.05)
VED2mg/kgIV	0.78 (0.36, 1.56)	0.41 (0.09, 1.90)	1.13 (0.46, 2.64)	0.37 (0.08, 1.64)	0.86 (0.26, 2.77)	0.65 (0.20, 2.07)	0.63 (0.15, 2.83)	0.38 (0.07, 1.97)	1.22 (0.35, 4.42)	0.46 (0.10, 2.05)	0.98 (0.48, 1.87)	1.43 (0.45, 5.51)	0.95 (0.44, 1.87)	1.06 (0.44, 2.79)	1.11 (0.47, 2.59)	0.97 (0.46, 1.84)	1.63 (0.67, 3.87)	1.23 (0.57, 2.37)	0.60 (0.17, 1.94)	0.80 (0.46, 1.32)	VED2mg/kgIV	1.19 (0.56, 2.35)
VED300mgIV	0.66 (0.40, 1.07)	0.35 (0.07, 1.55)	0.97 (0.49, 1.91)	0.31 (0.08, 1.06)	0.73 (0.28, 2.17)	0.55 (0.21, 1.54)	0.55 (0.15, 2.12)	0.32 (0.07, 1.44)	1.03 (0.35, 3.34)	0.40 (0.09, 1.46)	0.83 (0.56, 1.23)	1.25 (0.45, 3.98)	0.81 (0.50, 1.26)	0.91 (0.48, 1.69)	0.95 (0.52, 1.73)	0.81 (0.51, 1.30)	1.36 (0.69, 2.92)	1.05 (0.67, 1.57)	0.50 (0.17, 1.53)	0.66 (0.33, 1.49)	0.84 (0.43, 1.79)	VED300mgIV

Table S12B: League table for clinical remission for FDA-approved drugs only.

	ABA10mg/kgIV	ABA30mg/kgIV	ABA3mg/kgIV	ADA160mg80mg40mgSC	ADA160mg80mg60mgSC	ADA160mg80mgSC	ADA40mg20mgSC	ADA80mg40mgSC	BRO210mgIV	BRO350mgIV	BRO700mgIV	CER100mgSC	CER10mg/kgIV	CER200mgSC	CER20mg/kgIV	CER400mgSC	CER5mg/kgIV	ETA25mgSC	FIL100mgPO	FIL200mgPO
ABA10mg/kgIV	ABA10mg/kgIV	1.92 (0.49, 7.47)	1.19 (0.34, 4.22)	**4.68 (1.40, 17.65)**	**10.14 (2.70, 42.87)**	**4.78 (1.53, 15.69)**	2.54 (0.77, 9.60)	**4.07 (1.29, 14.24)**	0.52 (0.01, 9.47)	4.14 (0.84, 41.51)	2.75 (0.38, 23.36)	2.02 (0.58, 7.34)	1.18 (0.24, 5.82)	1.42 (0.40, 4.90)	0.59 (0.12, 3.27)	2.13 (0.73, 6.94)	1.71 (0.45, 7.29)	0.85 (0.13, 5.02)	1.98 (0.65, 6.86)	**3.30 (1.14, 11.12)**
ABA30mg/kgIV	0.52 (0.13, 2.05)	ABA30mg/kgIV	0.61 (0.19, 2.13)	2.32 (0.71, 9.13)	**5.17 (1.43, 21.65)**	2.38 (0.75, 9.04)	1.31 (0.37, 5.15)	2.06 (0.62, 7.45)	0.27 (0.00, 4.38)	2.26 (0.40, 19.45)	1.38 (0.17, 13.99)	0.99 (0.32, 3.68)	0.60 (0.12, 2.93)	0.70 (0.22, 2.77)	0.29 (0.06, 1.76)	1.06 (0.37, 3.68)	0.86 (0.24, 3.63)	0.43 (0.07, 2.62)	0.98 (0.33, 3.83)	1.65 (0.57, 5.88)
ABA3mg/kgIV	0.84 (0.24, 2.94)	1.64 (0.47, 5.28)	ABA3mg/kgIV	**3.93 (1.09, 14.15)**	**8.85 (2.30, 32.16)**	**3.95 (1.24, 13.29)**	2.20 (0.61, 7.28)	**3.46 (1.03, 12.01)**	0.45 (0.01, 6.47)	3.68 (0.64, 28.90)	2.38 (0.29, 17.69)	1.66 (0.48, 5.17)	1.05 (0.19, 4.14)	1.17 (0.32, 3.91)	0.51 (0.08, 2.64)	1.77 (0.57, 5.23)	1.44 (0.38, 5.64)	0.71 (0.11, 4.44)	1.68 (0.51, 5.22)	2.75 (0.89, 8.41)
ADA160mg80mg40mgSC	**0.21 (0.06, 0.72)**	0.43 (0.11, 1.40)	**0.25 (0.07, 0.91)**	ADA160mg80mg40mgSC	2.22 (0.77, 6.25)	1.01 (0.44, 2.26)	0.54 (0.21, 1.44)	0.87 (0.37, 2.14)	0.12 (0.00, 1.48)	0.90 (0.20, 7.16)	0.58 (0.09, 4.26)	**0.43 (0.17, 0.95)**	**0.25 (0.06, 0.86)**	**0.30 (0.12, 0.71)**	**0.13 (0.03, 0.49)**	**0.46 (0.21, 0.88)**	0.37 (0.12, 1.11)	**0.18 (0.03, 0.79)**	**0.43 (0.19, 0.91)**	0.71 (0.33, 1.45)
ADA160mg80mg60mgSC	**0.10 (0.02, 0.37)**	**0.19 (0.05, 0.70)**	**0.11 (0.03, 0.43)**	0.45 (0.16, 1.29)	ADA160mg80mg60mgSC	0.47 (0.18, 1.10)	**0.26 (0.08, 0.66)**	**0.40 (0.15, 0.98)**	**0.06 (0.00, 0.71)**	0.42 (0.09, 3.30)	0.27 (0.04, 1.91)	**0.19 (0.07, 0.47)**	**0.11 (0.03, 0.42)**	**0.14 (0.05, 0.33)**	**0.06 (0.01, 0.24)**	**0.21 (0.09, 0.43)**	**0.17 (0.05, 0.52)**	**0.08 (0.02, 0.42)**	**0.20 (0.08, 0.45)**	**0.32 (0.13, 0.72)**
ADA160mg80mgSC	**0.21 (0.06, 0.65)**	0.42 (0.11, 1.34)	**0.25 (0.08, 0.81)**	0.99 (0.44, 2.28)	2.14 (0.91, 5.69)	ADA160mg80mgSC	**0.54 (0.30, 0.95)**	0.86 (0.56, 1.33)	0.11 (0.00, 1.53)	0.89 (0.20, 7.24)	0.57 (0.09, 4.34)	**0.42 (0.21, 0.82)**	**0.25 (0.08, 0.80)**	**0.30 (0.14, 0.59)**	**0.13 (0.03, 0.44)**	**0.44 (0.28, 0.75)**	**0.36 (0.13, 0.99)**	**0.18 (0.04, 0.71)**	**0.42 (0.22, 0.77)**	0.69 (0.40, 1.21)
ADA40mg20mgSC	0.39 (0.10, 1.30)	0.76 (0.19, 2.69)	0.45 (0.14, 1.63)	1.84 (0.70, 4.79)	**3.90 (1.51, 12.00)**	**1.84 (1.05, 3.38)**	ADA40mg20mgSC	1.58 (0.90, 2.93)	0.21 (0.00, 2.94)	1.64 (0.36, 13.18)	1.04 (0.15, 7.57)	0.77 (0.34, 1.74)	0.47 (0.13, 1.59)	0.54 (0.23, 1.26)	**0.24 (0.05, 0.86)**	0.82 (0.42, 1.64)	0.67 (0.22, 2.00)	0.33 (0.06, 1.40)	0.77 (0.35, 1.70)	1.26 (0.62, 2.68)
ADA80mg40mgSC	**0.25 (0.07, 0.77)**	0.49 (0.13, 1.60)	**0.29 (0.08, 0.97)**	1.15 (0.47, 2.74)	**2.49 (1.02, 6.63)**	1.17 (0.75, 1.77)	0.63 (0.34, 1.11)	ADA80mg40mgSC	0.13 (0.00, 1.73)	1.04 (0.23, 7.69)	0.65 (0.10, 4.74)	0.48 (0.23, 1.03)	**0.29 (0.09, 0.94)**	**0.34 (0.15, 0.74)**	**0.15 (0.03, 0.53)**	**0.51 (0.29, 0.95)**	0.42 (0.15, 1.18)	**0.21 (0.04, 0.86)**	0.48 (0.24, 1.00)	0.80 (0.42, 1.53)
BRO210mgIV	1.92 (0.11, 122.25)	3.67 (0.23, 213.84)	2.24 (0.15, 129.83)	8.17 (0.68, 484.19)	**17.74 (1.41, 1154.82)**	8.84 (0.65, 467.46)	4.71 (0.34, 273.02)	7.69 (0.58, 432.15)	BRO210mgIV	7.73 (0.98, 301.37)	4.72 (0.53, 233.96)	3.44 (0.30, 236.84)	2.08 (0.16, 142.87)	2.51 (0.21, 163.65)	1.11 (0.05, 86.89)	3.80 (0.32, 248.82)	3.17 (0.23, 216.10)	1.41 (0.08, 121.23)	3.53 (0.29, 227.03)	5.95 (0.49, 359.10)
BRO350mgIV	0.24 (0.02, 1.19)	0.44 (0.05, 2.53)	0.27 (0.03, 1.55)	1.12 (0.14, 4.97)	2.37 (0.30, 11.41)	1.13 (0.14, 5.08)	0.61 (0.08, 2.77)	0.96 (0.13, 4.33)	0.13 (0.00, 1.02)	BRO350mgIV	0.61 (0.14, 2.67)	0.47 (0.06, 2.00)	0.27 (0.03, 1.42)	0.33 (0.04, 1.55)	**0.14 (0.01, 0.93)**	0.51 (0.07, 2.10)	0.41 (0.05, 2.08)	0.19 (0.02, 1.55)	0.48 (0.06, 2.11)	0.78 (0.10, 3.36)
BRO700mgIV	0.36 (0.04, 2.62)	0.72 (0.07, 5.76)	0.42 (0.06, 3.39)	1.72 (0.23, 11.66)	3.75 (0.52, 25.12)	1.75 (0.23, 10.57)	0.96 (0.13, 6.54)	1.53 (0.21, 9.66)	0.21 (0.00, 1.88)	1.63 (0.37, 7.14)	BRO700mgIV	0.75 (0.09, 4.36)	0.43 (0.05, 3.30)	0.51 (0.07, 3.21)	0.23 (0.02, 1.84)	0.79 (0.11, 4.54)	0.65 (0.08, 4.00)	0.31 (0.03, 2.71)	0.76 (0.09, 3.97)	1.23 (0.17, 6.77)
CER100mgSC	0.50 (0.14, 1.72)	1.01 (0.27, 3.12)	0.60 (0.19, 2.10)	**2.34 (1.05, 5.81)**	**5.17 (2.13, 14.55)**	**2.39 (1.22, 4.65)**	1.30 (0.57, 2.96)	2.06 (0.97, 4.43)	0.29 (0.00, 3.37)	2.15 (0.50, 17.67)	1.34 (0.23, 10.84)	CER100mgSC	0.58 (0.17, 1.86)	0.70 (0.36, 1.32)	0.30 (0.07, 1.09)	1.05 (0.67, 1.78)	0.86 (0.30, 2.40)	0.42 (0.08, 1.78)	1.00 (0.53, 1.97)	1.64 (0.92, 3.07)
CER10mg/kgIV	0.85 (0.17, 4.17)	1.65 (0.34, 8.10)	0.96 (0.24, 5.22)	**3.97 (1.16, 15.47)**	**8.71 (2.37, 36.39)**	**3.94 (1.24, 13.26)**	2.11 (0.63, 7.90)	**3.39 (1.06, 11.70)**	0.48 (0.01, 6.41)	3.66 (0.70, 28.71)	2.30 (0.30, 19.30)	1.71 (0.54, 5.74)	CER10mg/kgIV	1.16 (0.34, 4.00)	0.50 (0.12, 2.46)	1.76 (0.65, 5.53)	1.40 (0.51, 4.59)	0.70 (0.10, 4.55)	1.62 (0.57, 5.74)	2.67 (0.95, 8.76)
CER200mgSC	0.70 (0.20, 2.48)	1.42 (0.36, 4.56)	0.85 (0.26, 3.17)	**3.36 (1.40, 8.61)**	**7.25 (3.02, 20.32)**	**3.38 (1.70, 6.97)**	1.84 (0.79, 4.35)	**2.94 (1.36, 6.59)**	0.40 (0.01, 4.83)	3.02 (0.65, 24.84)	1.94 (0.31, 15.24)	1.42 (0.76, 2.75)	0.86 (0.25, 2.91)	CER200mgSC	0.42 (0.10, 1.58)	1.50 (0.89, 2.86)	1.20 (0.45, 3.48)	0.61 (0.12, 2.88)	1.40 (0.71, 3.01)	**2.31 (1.25, 4.67)**
CER20mg/kgIV	1.70 (0.31, 8.50)	3.40 (0.57, 16.25)	1.98 (0.38, 12.13)	**7.71 (2.05, 33.34)**	**17.29 (4.24, 86.21)**	**7.88 (2.26, 31.61)**	**4.25 (1.17, 18.24)**	**6.83 (1.89, 28.67)**	0.90 (0.01, 18.87)	**7.05 (1.08, 84.31)**	4.41 (0.54, 56.03)	3.33 (0.92, 14.34)	2.01 (0.41, 8.63)	2.39 (0.63, 9.84)	CER20mg/kgIV	**3.54 (1.09, 13.63)**	2.84 (0.91, 11.12)	1.39 (0.19, 9.77)	3.35 (0.96, 14.06)	**5.49 (1.62, 20.82)**
CER400mgSC	0.47 (0.14, 1.37)	0.94 (0.27, 2.68)	0.56 (0.19, 1.77)	**2.19 (1.13, 4.71)**	**4.77 (2.31, 11.36)**	**2.26 (1.33, 3.63)**	1.21 (0.61, 2.36)	**1.95 (1.05, 3.48)**	0.26 (0.00, 3.08)	1.98 (0.48, 15.23)	1.27 (0.22, 8.83)	0.95 (0.56, 1.49)	0.57 (0.18, 1.55)	0.67 (0.35, 1.12)	**0.28 (0.07, 0.92)**	CER400mgSC	0.81 (0.33, 2.00)	0.41 (0.09, 1.54)	0.93 (0.59, 1.50)	**1.54 (1.04, 2.29)**
CER5mg/kgIV	0.59 (0.14, 2.20)	1.16 (0.28, 4.17)	0.69 (0.18, 2.64)	2.68 (0.90, 8.59)	**5.88 (1.93, 19.17)**	**2.77 (1.01, 7.76)**	1.50 (0.50, 4.56)	2.36 (0.85, 6.77)	0.32 (0.00, 4.26)	2.44 (0.48, 20.26)	1.53 (0.25, 12.86)	1.16 (0.42, 3.34)	0.72 (0.22, 1.95)	0.83 (0.29, 2.22)	0.35 (0.09, 1.09)	1.23 (0.50, 3.00)	CER5mg/kgIV	0.50 (0.08, 2.58)	1.16 (0.44, 3.09)	1.92 (0.75, 4.84)
ETA25mgSC	1.18 (0.20, 7.81)	2.34 (0.38, 14.84)	1.41 (0.23, 9.26)	**5.58 (1.26, 28.86)**	**12.15 (2.37, 59.79)**	**5.53 (1.41, 25.62)**	3.04 (0.71, 15.46)	**4.87 (1.16, 23.41)**	0.71 (0.01, 13.26)	5.30 (0.65, 55.94)	3.28 (0.37, 29.72)	2.36 (0.56, 11.93)	1.43 (0.22, 9.58)	1.63 (0.35, 8.43)	0.72 (0.10, 5.16)	2.46 (0.65, 11.58)	2.00 (0.39, 12.20)	ETA25mgSC	2.28 (0.60, 11.96)	3.80 (1.00, 18.98)
FIL100mgPO	0.50 (0.15, 1.53)	1.02 (0.26, 2.99)	0.60 (0.19, 1.95)	**2.33 (1.10, 5.24)**	**5.08 (2.22, 13.27)**	**2.37 (1.30, 4.51)**	1.30 (0.59, 2.84)	2.08 (1.00, 4.16)	0.28 (0.00, 3.41)	2.07 (0.47, 17.21)	1.32 (0.25, 10.59)	1.00 (0.51, 1.90)	0.62 (0.17, 1.76)	0.72 (0.33, 1.40)	0.30 (0.07, 1.04)	1.07 (0.66, 1.71)	0.86 (0.32, 2.27)	0.44 (0.08, 1.67)	FIL100mgPO	**1.64 (1.14, 2.45)**
FIL200mgPO	**0.30 (0.09, 0.88)**	0.60 (0.17, 1.76)	0.36 (0.12, 1.12)	1.41 (0.69, 3.07)	**3.10 (1.39, 7.72)**	1.45 (0.83, 2.48)	0.79 (0.37, 1.61)	1.25 (0.66, 2.36)	0.17 (0.00, 2.03)	1.28 (0.30, 9.70)	0.81 (0.15, 6.05)	0.61 (0.33, 1.09)	0.37 (0.11, 1.06)	**0.43 (0.21, 0.80)**	**0.18 (0.05, 0.62)**	**0.65 (0.44, 0.96)**	0.52 (0.21, 1.33)	0.26 (0.05, 1.00)	**0.61 (0.41, 0.88)**	FIL200mgPO

(TABLE S12B. continued)

	ABA10mg/kgIV	ABA30mg/kgIV	ABA3mg/kgIV	ADA160mg80mg40mgSC	ADA160mg80mg60mgSC	ADA160mg80mgSC	ADA40mg20mgSC	ADA80mg40mgSC	BRO210mgIV	BRO350mgIV	BRO700mgIV	CER100mgSC	CER10mg/kgIV	CER200mgSC	CER20mg/kgIV	CER400mgSC	CER5mg/kgIV	ETA25mgSC	FIL100mgPO	FIL200mgPO
GUS1200mgIV	**0.21 (0.06, 0.68)**	0.41 (0.11, 1.34)	**0.24 (0.07, 0.85)**	0.96 (0.59, 1.55)	2.12 (0.74, 5.85)	0.99 (0.44, 2.09)	0.53 (0.20, 1.29)	0.83 (0.37, 1.93)	0.12 (0.00, 1.37)	0.88 (0.19, 6.79)	0.56 (0.09, 4.04)	**0.41 (0.17, 0.89)**	**0.24 (0.06, 0.83)**	**0.29 (0.12, 0.66)**	**0.12 (0.03, 0.47)**	**0.44 (0.21, 0.84)**	0.36 (0.12, 1.07)	**0.17 (0.03, 0.74)**	**0.41 (0.19, 0.85)**	0.68 (0.32, 1.34)
GUS200mgIV	**0.16 (0.04, 0.52)**	0.33 (0.08, 1.00)	**0.20 (0.06, 0.65)**	0.76 (0.48, 1.19)	1.69 (0.58, 4.44)	0.78 (0.35, 1.64)	0.42 (0.17, 1.04)	0.66 (0.29, 1.51)	0.09 (0.00, 1.13)	0.68 (0.15, 5.33)	0.45 (0.07, 3.29)	**0.33 (0.14, 0.70)**	**0.20 (0.05, 0.62)**	**0.23 (0.09, 0.51)**	**0.10 (0.02, 0.37)**	**0.35 (0.17, 0.63)**	**0.29 (0.09, 0.81)**	**0.14 (0.03, 0.58)**	**0.33 (0.15, 0.66)**	0.54 (0.25, 1.03)
GUS600mgIV	**0.17 (0.05, 0.55)**	0.34 (0.08, 1.03)	**0.20 (0.06, 0.67)**	0.79 (0.49, 1.24)	1.72 (0.62, 4.66)	0.80 (0.36, 1.68)	0.43 (0.17, 1.05)	0.68 (0.29, 1.55)	0.09 (0.00, 1.14)	0.70 (0.16, 5.48)	0.46 (0.07, 3.31)	**0.33 (0.14, 0.70)**	**0.20 (0.05, 0.66)**	**0.24 (0.09, 0.52)**	**0.10 (0.02, 0.39)**	**0.36 (0.17, 0.64)**	**0.29 (0.09, 0.81)**	**0.14 (0.03, 0.60)**	**0.34 (0.15, 0.67)**	0.56 (0.26, 1.06)
INF10mg/kgIV	**0.07 (0.00, 0.49)**	0.13 (0.01, 1.07)	**0.08 (0.00, 0.58)**	0.32 (0.02, 2.15)	0.66 (0.04, 4.72)	0.32 (0.02, 1.98)	0.17 (0.01, 1.12)	0.27 (0.02, 1.75)	**0.04 (0.00, 0.78)**	0.30 (0.01, 4.28)	0.18 (0.01, 2.64)	**0.13 (0.01, 0.84)**	**0.08 (0.00, 0.59)**	**0.10 (0.01, 0.59)**	**0.04 (0.00, 0.33)**	**0.14 (0.01, 0.82)**	**0.11 (0.01, 0.78)**	**0.05 (0.00, 0.54)**	**0.14 (0.01, 0.82)**	0.23 (0.01, 1.33)
INF20mg/kgIV	**0.07 (0.00, 0.48)**	**0.13 (0.01, 0.98)**	**0.08 (0.00, 0.59)**	0.33 (0.02, 2.07)	0.71 (0.03, 4.77)	0.33 (0.02, 1.97)	0.18 (0.01, 1.13)	0.28 (0.02, 1.72)	**0.04 (0.00, 0.69)**	0.30 (0.01, 3.76)	0.18 (0.01, 2.24)	**0.14 (0.01, 0.78)**	**0.08 (0.00, 0.55)**	**0.10 (0.01, 0.56)**	**0.04 (0.00, 0.34)**	**0.15 (0.01, 0.80)**	**0.12 (0.01, 0.76)**	**0.06 (0.00, 0.53)**	**0.14 (0.01, 0.78)**	0.23 (0.01, 1.29)
INF5mg/kgIV	**0.04 (0.00, 0.23)**	**0.07 (0.00, 0.47)**	**0.04 (0.00, 0.28)**	**0.17 (0.01, 0.99)**	0.35 (0.02, 2.30)	**0.17 (0.01, 0.94)**	**0.09 (0.01, 0.55)**	**0.14 (0.01, 0.83)**	**0.02 (0.00, 0.36)**	0.15 (0.00, 1.83)	0.09 (0.00, 1.11)	**0.07 (0.00, 0.37)**	**0.04 (0.00, 0.28)**	**0.05 (0.00, 0.28)**	**0.02 (0.00, 0.16)**	**0.08 (0.00, 0.39)**	**0.06 (0.00, 0.38)**	**0.03 (0.00, 0.26)**	**0.07 (0.00, 0.39)**	**0.12 (0.01, 0.61)**
MIR1000mgIV	**0.20 (0.04, 0.70)**	0.37 (0.09, 1.59)	**0.23 (0.06, 0.89)**	0.93 (0.31, 2.64)	2.00 (0.59, 6.86)	0.94 (0.33, 2.28)	0.53 (0.15, 1.46)	0.83 (0.26, 2.08)	0.11 (0.00, 1.49)	0.84 (0.14, 7.04)	0.51 (0.08, 4.27)	0.39 (0.13, 1.09)	**0.23 (0.05, 0.86)**	**0.28 (0.09, 0.74)**	**0.12 (0.03, 0.50)**	**0.43 (0.16, 0.98)**	0.35 (0.10, 1.13)	**0.16 (0.03, 0.86)**	0.40 (0.14, 1.01)	0.66 (0.24, 1.56)
MIR200mgIV	0.36 (0.07, 1.56)	0.68 (0.13, 3.47)	0.42 (0.09, 2.06)	1.60 (0.46, 6.76)	3.54 (0.87, 15.56)	1.67 (0.49, 5.99)	0.91 (0.25, 3.37)	1.43 (0.41, 5.29)	0.20 (0.00, 3.22)	1.52 (0.21, 15.14)	0.89 (0.13, 8.78)	0.69 (0.20, 2.51)	0.42 (0.08, 1.93)	0.49 (0.13, 1.82)	**0.21 (0.04, 0.99)**	0.75 (0.23, 2.51)	0.61 (0.15, 2.30)	0.29 (0.05, 1.94)	0.69 (0.20, 2.51)	1.16 (0.34, 3.92)
MIR600mgIV	**0.14 (0.03, 0.48)**	0.26 (0.06, 1.02)	**0.15 (0.04, 0.65)**	0.62 (0.20, 1.86)	1.32 (0.39, 4.45)	0.62 (0.21, 1.64)	0.34 (0.10, 1.04)	0.54 (0.18, 1.47)	0.08 (0.00, 1.04)	0.57 (0.10, 4.44)	0.35 (0.05, 2.70)	**0.26 (0.08, 0.78)**	**0.16 (0.03, 0.60)**	**0.18 (0.06, 0.51)**	**0.08 (0.02, 0.34)**	**0.28 (0.10, 0.70)**	**0.22 (0.06, 0.79)**	**0.11 (0.02, 0.54)**	**0.26 (0.09, 0.71)**	0.43 (0.15, 1.09)
NAT300mgIV	0.42 (0.12, 1.18)	0.82 (0.24, 2.27)	0.49 (0.16, 1.52)	1.91 (1.00, 4.05)	**4.20 (2.03, 9.92)**	**1.97 (1.22, 3.20)**	1.06 (0.54, 2.09)	1.70 (0.95, 3.09)	0.23 (0.00, 2.70)	1.74 (0.42, 13.28)	1.11 (0.19, 7.91)	0.82 (0.48, 1.40)	0.50 (0.16, 1.40)	0.58 (0.30, 1.03)	**0.25 (0.07, 0.82)**	0.87 (0.65, 1.18)	0.71 (0.29, 1.78)	0.35 (0.08, 1.34)	0.82 (0.52, 1.30)	1.35 (0.93, 2.03)
NAT3mg/kgIV	0.57 (0.15, 1.81)	1.12 (0.27, 3.68)	0.66 (0.20, 2.36)	**2.59 (1.09, 6.65)**	**5.70 (2.27, 15.97)**	**2.68 (1.27, 5.52)**	1.43 (0.61, 3.55)	**2.29 (1.04, 5.05)**	0.32 (0.01, 4.27)	2.42 (0.51, 19.52)	1.51 (0.23, 11.84)	1.13 (0.51, 2.38)	0.67 (0.19, 2.14)	0.78 (0.33, 1.75)	0.34 (0.07, 1.29)	1.20 (0.64, 2.24)	0.97 (0.33, 2.79)	0.47 (0.10, 2.03)	1.12 (0.54, 2.35)	1.84 (0.93, 3.65)
NAT3mg/kgIVx2	0.37 (0.10, 1.15)	0.72 (0.19, 2.34)	0.43 (0.13, 1.51)	1.72 (0.75, 4.19)	**3.73 (1.58, 9.71)**	1.76 (0.87, 3.49)	0.94 (0.41, 2.21)	1.50 (0.72, 3.22)	0.21 (0.00, 2.76)	1.56 (0.34, 13.65)	0.98 (0.17, 7.43)	0.74 (0.34, 1.44)	0.44 (0.13, 1.34)	0.51 (0.22, 1.09)	**0.22 (0.05, 0.76)**	0.78 (0.45, 1.33)	0.62 (0.22, 1.73)	0.31 (0.06, 1.35)	0.73 (0.37, 1.39)	1.20 (0.65, 2.20)
NAT6mg/kgIVx2	0.40 (0.11, 1.35)	0.80 (0.20, 2.85)	0.48 (0.14, 1.72)	1.88 (0.78, 4.57)	**4.10 (1.63, 11.47)**	1.94 (0.92, 3.88)	1.04 (0.45, 2.42)	1.67 (0.77, 3.58)	0.23 (0.00, 2.90)	1.72 (0.39, 13.74)	1.08 (0.18, 8.21)	0.80 (0.37, 1.72)	0.49 (0.13, 1.68)	0.56 (0.24, 1.25)	**0.25 (0.05, 0.87)**	0.86 (0.47, 1.57)	0.69 (0.23, 1.96)	0.33 (0.07, 1.41)	0.81 (0.39, 1.60)	1.32 (0.69, 2.54)
PBO	0.57 (0.18, 1.58)	1.14 (0.33, 3.17)	0.69 (0.24, 2.07)	**2.67 (1.42, 5.51)**	**5.80 (2.92, 13.38)**	**2.74 (1.74, 4.24)**	1.46 (0.76, 2.80)	**2.37 (1.36, 4.13)**	0.32 (0.00, 3.69)	2.41 (0.59, 18.32)	1.54 (0.28, 10.97)	1.15 (0.68, 1.87)	0.70 (0.23, 1.90)	0.82 (0.43, 1.37)	0.34 (0.09, 1.11)	1.22 (0.99, 1.52)	0.99 (0.40, 2.44)	0.49 (0.11, 1.89)	1.14 (0.76, 1.71)	**1.88 (1.38, 2.65)**
RIS1200mgIV	**0.32 (0.10, 0.93)**	0.65 (0.19, 1.84)	0.39 (0.13, 1.20)	1.51 (0.79, 3.22)	**3.30 (1.59, 7.90)**	1.56 (0.93, 2.50)	0.84 (0.41, 1.62)	1.34 (0.73, 2.42)	0.18 (0.00, 2.17)	1.35 (0.32, 10.71)	0.87 (0.16, 6.39)	0.65 (0.37, 1.09)	0.40 (0.13, 1.10)	**0.46 (0.23, 0.81)**	**0.20 (0.05, 0.64)**	**0.69 (0.51, 0.92)**	0.56 (0.22, 1.38)	0.28 (0.06, 1.07)	0.64 (0.41, 1.02)	1.06 (0.73, 1.56)
RIS200mgIV	0.43 (0.12, 1.49)	0.84 (0.21, 2.94)	0.51 (0.14, 1.83)	1.98 (0.81, 5.38)	**4.29 (1.68, 12.92)**	2.05 (0.95, 4.45)	1.11 (0.44, 2.77)	1.75 (0.77, 4.24)	0.25 (0.00, 3.34)	1.84 (0.38, 15.64)	1.19 (0.19, 9.54)	0.84 (0.38, 1.97)	0.51 (0.13, 1.74)	0.60 (0.23, 1.48)	**0.26 (0.06, 0.96)**	0.91 (0.46, 1.91)	0.73 (0.25, 2.19)	0.36 (0.07, 1.67)	0.85 (0.39, 1.92)	1.41 (0.69, 3.04)
RIS600mgIV	**0.30 (0.09, 0.86)**	0.60 (0.17, 1.71)	0.36 (0.12, 1.10)	1.39 (0.73, 2.91)	**3.02 (1.47, 7.41)**	1.43 (0.86, 2.31)	0.77 (0.39, 1.49)	1.23 (0.67, 2.24)	0.17 (0.00, 1.99)	1.25 (0.31, 9.88)	0.80 (0.15, 5.81)	**0.60 (0.34, 0.99)**	0.36 (0.11, 1.00)	**0.42 (0.22, 0.74)**	**0.18 (0.05, 0.58)**	**0.63 (0.47, 0.84)**	0.52 (0.21, 1.27)	0.25 (0.05, 1.00)	**0.59 (0.38, 0.94)**	0.98 (0.68, 1.43)
RIS600mgIV4_4	0.41 (0.12, 1.21)	0.81 (0.25, 2.37)	0.49 (0.16, 1.60)	1.93 (0.94, 4.23)	**4.15 (1.88, 10.29)**	**1.96 (1.12, 3.43)**	1.07 (0.50, 2.26)	1.70 (0.90, 3.20)	0.23 (0.00, 2.83)	1.75 (0.41, 12.92)	1.10 (0.19, 8.10)	0.82 (0.43, 1.51)	0.49 (0.15, 1.45)	0.58 (0.28, 1.10)	**0.25 (0.07, 0.85)**	0.88 (0.56, 1.31)	0.72 (0.28, 1.81)	0.35 (0.08, 1.42)	0.83 (0.47, 1.39)	1.36 (0.81, 2.17)
SEC10mg/kgIV	0.80 (0.12, 5.11)	1.54 (0.24, 11.06)	0.96 (0.15, 6.07)	3.74 (0.77, 19.77)	**8.28 (1.39, 44.30)**	3.88 (0.79, 17.60)	2.13 (0.39, 10.53)	3.29 (0.70, 16.52)	0.43 (0.01, 8.32)	3.54 (0.42, 38.87)	2.23 (0.20, 23.28)	1.61 (0.34, 7.48)	0.94 (0.15, 7.06)	1.14 (0.21, 5.64)	0.50 (0.06, 3.14)	1.72 (0.37, 7.91)	1.41 (0.23, 8.03)	0.70 (0.09, 4.35)	1.60 (0.35, 7.92)	2.65 (0.59, 12.91)
TOF15mgPO_BID	0.81 (0.16, 5.11)	1.64 (0.32, 8.61)	1.02 (0.22, 5.73)	**3.77 (1.16, 18.10)**	**8.46 (2.32, 40.54)**	**3.89 (1.18, 14.86)**	2.08 (0.57, 8.74)	3.38 (1.00, 13.48)	0.50 (0.01, 7.96)	3.73 (0.61, 32.03)	2.25 (0.32, 19.17)	1.67 (0.49, 6.66)	1.00 (0.20, 4.66)	1.15 (0.34, 4.63)	0.51 (0.09, 2.74)	1.74 (0.58, 6.33)	1.41 (0.37, 6.58)	0.68 (0.11, 4.56)	1.62 (0.53, 6.37)	2.66 (0.88, 10.58)

	ABA10mg/kgIV	ABA30mg/kgIV	ABA3mg/kgIV	ADA160mg80mg40mgSC	ADA160mg80mg60mgSC	ADA160mg80mgSC	ADA40mg20mgSC	ADA80mg40mgSC	BRO210mgIV	BRO350mgIV	BRO700mgIV	CER100mgSC	CER10mg/kgIV	CER200mgSC	CER20mg/kgIV	CER400mgSC	CER5mg/kgIV	ETA25mgSC	FIL100mgPO	FIL200mgPO
TOF1mgPO_BID	0.37 (0.08, 1.45)	0.74 (0.16, 2.71)	0.44 (0.11, 1.84)	1.75 (0.58, 5.28)	**3.79 (1.22, 12.35)**	1.78 (0.64, 4.88)	0.97 (0.31, 2.86)	1.54 (0.54, 4.30)	0.21 (0.00, 2.84)	1.60 (0.31, 14.04)	1.01 (0.15, 8.61)	0.76 (0.26, 1.92)	0.44 (0.10, 1.77)	0.53 (0.18, 1.37)	**0.23 (0.04, 0.93)**	0.82 (0.31, 1.92)	0.65 (0.19, 2.04)	0.30 (0.06, 1.75)	0.75 (0.27, 1.95)	1.25 (0.46, 3.08)
TOF5mgPO_BID	0.49 (0.10, 1.97)	1.00 (0.21, 3.62)	0.57 (0.15, 2.60)	2.30 (0.72, 7.27)	**5.05 (1.49, 17.40)**	2.36 (0.78, 6.24)	1.29 (0.38, 3.89)	2.03 (0.66, 5.77)	0.27 (0.00, 4.26)	2.07 (0.36, 18.59)	1.30 (0.19, 11.53)	0.98 (0.33, 2.65)	0.60 (0.13, 2.40)	0.70 (0.22, 1.96)	0.30 (0.05, 1.24)	1.05 (0.39, 2.56)	0.87 (0.23, 2.74)	0.41 (0.06, 2.32)	0.98 (0.35, 2.66)	1.63 (0.58, 4.13)
UPA12mgPO_BID	0.42 (0.08, 2.10)	0.82 (0.14, 4.18)	0.51 (0.09, 2.40)	1.92 (0.46, 7.93)	4.23 (1.00, 18.95)	1.97 (0.52, 6.64)	1.08 (0.25, 4.25)	1.68 (0.45, 6.26)	0.20 (0.00, 4.88)	1.66 (0.26, 21.57)	1.06 (0.16, 12.30)	0.83 (0.20, 3.07)	0.49 (0.10, 2.31)	0.59 (0.14, 2.10)	0.26 (0.04, 1.22)	0.89 (0.24, 2.87)	0.71 (0.14, 3.07)	0.35 (0.05, 2.08)	0.83 (0.22, 2.94)	1.37 (0.38, 4.61)
UPA24mgPO	0.75 (0.11, 4.14)	1.50 (0.19, 7.32)	0.87 (0.13, 6.09)	3.42 (0.65, 16.45)	**7.60 (1.42, 37.46)**	3.48 (0.68, 16.44)	1.92 (0.35, 9.84)	3.02 (0.59, 15.05)	0.38 (0.01, 9.63)	2.87 (0.42, 41.87)	1.91 (0.21, 24.52)	1.45 (0.27, 7.09)	0.90 (0.15, 5.59)	1.03 (0.19, 4.83)	0.45 (0.05, 2.73)	1.59 (0.32, 6.93)	1.28 (0.22, 6.66)	0.64 (0.07, 4.12)	1.48 (0.29, 6.77)	2.46 (0.48, 11.08)
UPA24mgPO_BID	**0.23 (0.04, 0.94)**	0.47 (0.07, 1.85)	0.28 (0.04, 1.24)	1.09 (0.24, 3.50)	2.33 (0.55, 8.02)	1.10 (0.29, 3.17)	0.61 (0.14, 1.90)	0.97 (0.24, 2.84)	0.12 (0.00, 2.27)	0.90 (0.15, 10.89)	0.58 (0.08, 6.42)	0.46 (0.11, 1.44)	0.27 (0.06, 1.12)	0.33 (0.08, 1.04)	**0.14 (0.02, 0.69)**	0.50 (0.13, 1.35)	0.41 (0.08, 1.40)	0.19 (0.03, 1.01)	0.45 (0.11, 1.50)	0.77 (0.19, 2.18)
UPA3mgPO_BID	0.60 (0.10, 3.19)	1.19 (0.18, 6.18)	0.73 (0.11, 3.66)	2.80 (0.55, 12.08)	**6.11 (1.25, 27.34)**	2.77 (0.64, 11.03)	1.55 (0.32, 6.51)	2.40 (0.52, 10.03)	0.30 (0.01, 6.15)	2.51 (0.35, 27.07)	1.55 (0.19, 16.97)	1.21 (0.25, 4.41)	0.70 (0.14, 3.61)	0.84 (0.18, 3.24)	0.37 (0.05, 2.14)	1.29 (0.29, 4.56)	1.02 (0.21, 4.88)	0.50 (0.06, 3.45)	1.20 (0.27, 4.65)	1.97 (0.43, 7.47)
UPA45mgPO	**0.33 (0.10, 0.92)**	0.66 (0.18, 1.86)	0.39 (0.13, 1.21)	1.53 (0.78, 3.23)	**3.31 (1.62, 7.86)**	1.56 (0.95, 2.56)	0.85 (0.42, 1.65)	1.37 (0.75, 2.41)	0.18 (0.00, 2.15)	1.38 (0.35, 10.58)	0.88 (0.16, 6.30)	0.65 (0.37, 1.11)	0.40 (0.12, 1.12)	**0.47 (0.23, 0.81)**	**0.20 (0.05, 0.64)**	**0.70 (0.51, 0.95)**	0.57 (0.23, 1.38)	0.28 (0.06, 1.14)	0.66 (0.41, 1.03)	1.08 (0.73, 1.59)
UPA6mgPO_BID	**0.19 (0.04, 0.82)**	0.37 (0.06, 1.59)	**0.23 (0.04, 0.96)**	0.90 (0.20, 3.18)	1.92 (0.48, 7.05)	0.93 (0.23, 2.70)	0.50 (0.11, 1.64)	0.79 (0.19, 2.50)	0.10 (0.00, 2.00)	0.75 (0.14, 9.68)	0.46 (0.07, 5.52)	0.38 (0.10, 1.17)	**0.22 (0.05, 0.92)**	**0.27 (0.07, 0.84)**	**0.11 (0.02, 0.51)**	0.41 (0.11, 1.13)	0.33 (0.07, 1.22)	**0.16 (0.02, 0.90)**	0.38 (0.10, 1.12)	0.64 (0.17, 1.78)
UST130mgIV	**0.34 (0.10, 0.98)**	0.69 (0.19, 1.91)	0.41 (0.14, 1.26)	1.60 (0.79, 3.41)	**3.49 (1.63, 8.55)**	1.64 (0.94, 2.83)	0.89 (0.43, 1.84)	1.43 (0.74, 2.63)	0.19 (0.00, 2.30)	1.44 (0.36, 10.92)	0.93 (0.17, 6.55)	0.69 (0.38, 1.20)	0.42 (0.13, 1.20)	**0.49 (0.24, 0.90)**	**0.20 (0.05, 0.69)**	0.73 (0.51, 1.04)	0.59 (0.23, 1.52)	0.29 (0.07, 1.20)	0.69 (0.41, 1.11)	1.14 (0.74, 1.71)
UST1mg/kgIV	0.30 (0.08, 1.00)	0.59 (0.17, 1.94)	0.36 (0.11, 1.31)	1.39 (0.59, 3.49)	**3.04 (1.30, 8.03)**	1.42 (0.72, 2.78)	0.78 (0.33, 1.77)	1.24 (0.58, 2.58)	0.17 (0.00, 2.07)	1.28 (0.31, 9.89)	0.82 (0.14, 5.79)	0.59 (0.29, 1.22)	0.36 (0.11, 1.15)	**0.42 (0.19, 0.89)**	**0.18 (0.04, 0.70)**	0.64 (0.37, 1.12)	0.51 (0.19, 1.42)	0.26 (0.05, 1.07)	0.59 (0.31, 1.16)	0.99 (0.53, 1.83)
UST3mg/kgIV	**0.29 (0.08, 0.94)**	0.57 (0.15, 1.87)	0.33 (0.11, 1.23)	1.33 (0.59, 3.21)	**2.91 (1.18, 7.45)**	1.36 (0.69, 2.71)	0.72 (0.32, 1.69)	1.17 (0.56, 2.52)	0.15 (0.00, 2.03)	1.20 (0.27, 9.58)	0.78 (0.12, 5.47)	0.57 (0.28, 1.11)	0.34 (0.10, 1.08)	**0.39 (0.18, 0.86)**	**0.17 (0.04, 0.63)**	0.60 (0.36, 1.05)	0.49 (0.18, 1.32)	**0.24 (0.05, 0.98)**	0.56 (0.29, 1.10)	0.93 (0.51, 1.67)
UST4.5mg/kgIV	0.21 (0.04, 1.19)	0.41 (0.07, 2.29)	0.24 (0.05, 1.54)	0.97 (0.21, 4.20)	2.20 (0.42, 9.34)	1.01 (0.21, 3.97)	0.54 (0.11, 2.39)	0.86 (0.18, 3.48)	0.12 (0.00, 1.98)	0.88 (0.13, 10.24)	0.54 (0.07, 6.02)	0.41 (0.09, 1.59)	0.25 (0.04, 1.24)	0.29 (0.06, 1.20)	**0.12 (0.02, 0.68)**	0.44 (0.11, 1.61)	0.35 (0.07, 1.70)	0.18 (0.02, 1.06)	0.41 (0.10, 1.61)	0.68 (0.17, 2.58)
UST6mg/kg90mgIV/SC	**0.20 (0.06, 0.67)**	0.41 (0.11, 1.29)	**0.24 (0.07, 0.85)**	0.95 (0.75, 1.22)	2.11 (0.74, 5.81)	0.96 (0.43, 2.08)	0.52 (0.20, 1.31)	0.82 (0.35, 1.96)	0.11 (0.00, 1.42)	0.86 (0.20, 6.82)	0.56 (0.09, 4.05)	**0.41 (0.17, 0.88)**	**0.24 (0.06, 0.81)**	**0.29 (0.12, 0.66)**	**0.12 (0.03, 0.46)**	**0.44 (0.21, 0.80)**	0.36 (0.12, 1.03)	**0.17 (0.03, 0.74)**	**0.41 (0.19, 0.84)**	0.68 (0.32, 1.32)
UST6mg/kgIV	**0.26 (0.08, 0.75)**	0.52 (0.15, 1.47)	**0.31 (0.10, 0.98)**	1.22 (0.62, 2.62)	**2.69 (1.25, 6.32)**	1.26 (0.74, 2.13)	0.68 (0.33, 1.37)	1.10 (0.58, 2.01)	0.15 (0.00, 1.78)	1.12 (0.28, 8.38)	0.71 (0.13, 4.96)	**0.53 (0.30, 0.92)**	**0.32 (0.10, 0.89)**	**0.38 (0.19, 0.69)**	**0.16 (0.04, 0.52)**	**0.56 (0.40, 0.78)**	0.46 (0.18, 1.14)	**0.23 (0.05, 0.89)**	**0.52 (0.32, 0.85)**	0.87 (0.57, 1.30)
UST90mgSC	0.58 (0.13, 2.44)	1.14 (0.24, 4.67)	0.67 (0.14, 3.16)	2.69 (0.80, 9.21)	**5.81 (1.61, 22.18)**	2.74 (0.86, 8.79)	1.49 (0.43, 5.22)	2.35 (0.74, 8.09)	0.33 (0.00, 4.68)	2.41 (0.40, 23.78)	1.49 (0.20, 15.30)	1.15 (0.35, 3.63)	0.65 (0.15, 3.34)	0.81 (0.23, 2.72)	0.34 (0.06, 1.72)	1.22 (0.41, 3.54)	0.98 (0.25, 4.17)	0.48 (0.08, 2.58)	1.15 (0.37, 3.49)	1.89 (0.64, 5.54)
VED0_5mg/kgIV	0.41 (0.11, 1.39)	0.81 (0.22, 2.67)	0.49 (0.14, 1.71)	1.90 (0.76, 5.05)	**4.13 (1.63, 11.42)**	1.95 (0.88, 4.30)	1.06 (0.41, 2.55)	1.69 (0.72, 3.97)	0.23 (0.00, 3.26)	1.80 (0.38, 14.13)	1.11 (0.18, 8.45)	0.80 (0.36, 1.89)	0.50 (0.14, 1.49)	0.57 (0.25, 1.37)	**0.25 (0.06, 0.93)**	0.87 (0.44, 1.71)	0.71 (0.24, 1.94)	0.34 (0.06, 1.52)	0.81 (0.38, 1.76)	1.35 (0.65, 2.77)
VED2mg/kgIV	0.32 (0.09, 1.05)	0.63 (0.16, 2.09)	0.38 (0.11, 1.32)	1.47 (0.61, 3.74)	**3.19 (1.31, 9.05)**	1.50 (0.70, 3.20)	0.82 (0.32, 1.99)	1.31 (0.57, 2.96)	0.18 (0.00, 2.45)	1.37 (0.29, 10.97)	0.87 (0.14, 6.48)	0.62 (0.29, 1.35)	0.38 (0.11, 1.21)	0.44 (0.19, 1.03)	**0.19 (0.04, 0.79)**	0.68 (0.36, 1.25)	0.55 (0.20, 1.52)	0.27 (0.05, 1.19)	0.63 (0.30, 1.32)	1.05 (0.52, 2.04)
VED300mgIV	**0.27 (0.08, 0.78)**	0.55 (0.16, 1.59)	0.33 (0.11, 1.00)	1.26 (0.61, 2.78)	**2.73 (1.27, 6.76)**	1.29 (0.74, 2.24)	0.70 (0.33, 1.46)	1.11 (0.59, 2.09)	0.15 (0.00, 1.77)	1.13 (0.28, 8.70)	0.72 (0.13, 5.27)	**0.54 (0.29, 0.96)**	**0.32 (0.10, 0.94)**	**0.38 (0.19, 0.72)**	**0.16 (0.04, 0.54)**	**0.58 (0.39, 0.85)**	0.47 (0.19, 1.23)	**0.23 (0.05, 0.92)**	**0.54 (0.32, 0.90)**	0.90 (0.56, 1.39)
VED300mgIV	**0.30 (0.08, 0.88)**	0.56 (0.15, 1.70)	0.35 (0.12, 1.15)	1.29 (0.62, 2.97)	**2.72 (1.25, 6.34)**	1.30 (0.75, 2.33)	0.70 (0.33, 1.51)	1.13 (0.58, 2.14)	0.31 (0.02, 3.19)	1.28 (0.28, 11.33)	0.79 (0.11, 6.48)	**0.48 (0.29, 0.81)**	**0.54 (0.29, 0.98)**	**0.32 (0.08, 0.99)**	**0.39 (0.19, 0.71)**	**0.19 (0.04, 0.60)**	**0.58 (0.39, 0.87)**	0.47 (0.18, 1.26)	**0.23 (0.04, 0.99)**	**0.55 (0.32, 0.94)**

(TABLE S12B. continued)

	GUS1200mgIV	GUS200mgIV	GUS600mgIV	INF10mg/kgIV	INF20mg/kgIV	INF5mg/kgIV	MIR1000mgIV	MIR200mgIV	MIR600mgIV	NAT300mgIV	NAT3mg/kgIV	NAT3mg/kgIVx2	NAT6mg/kgIVx2	PBO	RIS1200mgIV	RIS200mgIV	RIS600mgIV	RIS600mgIV4_4	SEC10mg/kgIV	TOF15mgPO_BID
ABA10mg/kgIV	**4.83 (1.47, 17.78)**	**6.15 (1.91, 22.57)**	**5.95 (1.82, 21.46)**	**13.65 (2.05, 362.18)**	**13.54 (2.07, 353.26)**	**27.16 (4.34, 656.21)**	**5.00 (1.42, 23.17)**	2.81 (0.64, 14.72)	**7.33 (2.07, 34.84)**	2.40 (0.85, 8.07)	1.77 (0.55, 6.55)	2.67 (0.87, 9.72)	2.49 (0.74, 9.15)	1.75 (0.63, 5.69)	**3.09 (1.08, 10.20)**	2.33 (0.67, 8.31)	**3.39 (1.16, 11.29)**	2.43 (0.83, 8.36)	1.26 (0.20, 8.23)	1.23 (0.20, 6.14)
ABA30mg/kgIV	2.41 (0.74, 9.37)	3.06 (1.00, 11.86)	2.95 (0.97, 11.77)	7.56 (0.94, 137.76)	**7.50 (1.02, 142.20)**	**14.93 (2.11, 259.22)**	2.67 (0.63, 11.55)	1.47 (0.29, 7.65)	3.88 (0.98, 17.94)	1.21 (0.44, 4.20)	0.89 (0.27, 3.70)	1.38 (0.43, 5.35)	1.26 (0.35, 5.07)	0.88 (0.32, 3.02)	1.54 (0.54, 5.38)	1.19 (0.34, 4.74)	1.68 (0.58, 5.91)	1.23 (0.42, 4.07)	0.65 (0.09, 4.19)	0.61 (0.12, 3.14)
ABA3mg/kgIV	**4.09 (1.18, 13.58)**	**5.09 (1.53, 17.86)**	**5.00 (1.48, 17.13)**	**12.14 (1.73, 254.23)**	**12.48 (1.68, 306.08)**	**23.81 (3.54, 540.84)**	**4.30 (1.12, 18.02)**	2.41 (0.48, 11.61)	**6.49 (1.54, 28.31)**	2.04 (0.66, 6.12)	1.52 (0.42, 5.09)	2.30 (0.66, 7.56)	2.10 (0.58, 7.07)	1.45 (0.48, 4.21)	2.59 (0.83, 7.67)	1.95 (0.55, 7.05)	2.81 (0.91, 8.48)	2.05 (0.63, 6.29)	1.04 (0.16, 6.50)	0.98 (0.17, 4.63)
ADA160mg80mg40mgSC	1.04 (0.64, 1.69)	1.31 (0.84, 2.08)	1.27 (0.81, 2.02)	3.17 (0.47, 60.92)	3.06 (0.48, 61.80)	**6.03 (1.01, 117.30)**	1.07 (0.38, 3.27)	0.62 (0.15, 2.19)	1.62 (0.54, 5.11)	0.52 (0.25, 1.00)	**0.39 (0.15, 0.92)**	0.58 (0.24, 1.34)	0.53 (0.22, 1.29)	**0.37 (0.18, 0.70)**	0.66 (0.31, 1.26)	0.50 (0.19, 1.23)	0.72 (0.34, 1.37)	0.52 (0.24, 1.07)	0.27 (0.05, 1.30)	**0.27 (0.06, 0.86)**
ADA160mg80mg60mgSC	0.47 (0.17, 1.36)	0.59 (0.23, 1.71)	0.58 (0.21, 1.60)	1.52 (0.21, 25.40)	1.42 (0.21, 28.59)	2.86 (0.44, 59.39)	0.50 (0.15, 1.69)	0.28 (0.06, 1.15)	0.76 (0.22, 2.59)	**0.24 (0.10, 0.49)**	**0.18 (0.06, 0.44)**	**0.27 (0.10, 0.63)**	**0.24 (0.09, 0.61)**	**0.17 (0.07, 0.34)**	**0.30 (0.13, 0.63)**	**0.23 (0.08, 0.60)**	**0.33 (0.13, 0.68)**	**0.24 (0.10, 0.53)**	**0.12 (0.02, 0.72)**	**0.12 (0.02, 0.43)**
ADA160mg80mgSC	1.01 (0.48, 2.27)	1.28 (0.61, 2.87)	1.25 (0.60, 2.80)	3.11 (0.50, 59.57)	3.02 (0.51, 55.56)	**5.96 (1.06, 104.82)**	1.06 (0.44, 3.06)	0.60 (0.17, 2.06)	1.60 (0.61, 4.72)	**0.51 (0.31, 0.82)**	**0.37 (0.18, 0.79)**	0.57 (0.29, 1.16)	0.52 (0.26, 1.08)	**0.36 (0.24, 0.57)**	0.64 (0.40, 1.08)	0.49 (0.22, 1.06)	0.70 (0.43, 1.17)	**0.51 (0.29, 0.89)**	0.26 (0.06, 1.27)	**0.26 (0.07, 0.85)**
ADA40mg20mgSC	1.90 (0.78, 4.89)	2.39 (0.96, 6.01)	2.33 (0.95, 5.77)	5.85 (0.89, 109.90)	5.67 (0.88, 105.56)	**11.39 (1.81, 196.00)**	1.90 (0.68, 6.61)	1.09 (0.30, 4.08)	2.93 (0.96, 9.79)	0.94 (0.48, 1.85)	0.70 (0.28, 1.65)	1.06 (0.45, 2.42)	0.96 (0.41, 2.24)	0.68 (0.36, 1.31)	1.20 (0.62, 2.42)	0.90 (0.36, 2.29)	1.30 (0.67, 2.59)	0.94 (0.44, 1.99)	0.47 (0.09, 2.55)	0.48 (0.11, 1.74)
ADA80mg40mgSC	1.20 (0.52, 2.70)	1.51 (0.66, 3.48)	1.46 (0.64, 3.39)	3.67 (0.57, 64.93)	3.51 (0.58, 62.45)	**6.96 (1.20, 117.72)**	1.21 (0.48, 3.92)	0.70 (0.19, 2.42)	1.85 (0.68, 5.69)	0.59 (0.32, 1.05)	**0.44 (0.20, 0.96)**	0.67 (0.31, 1.39)	0.60 (0.28, 1.30)	**0.42 (0.24, 0.73)**	0.75 (0.41, 1.37)	0.57 (0.24, 1.30)	0.81 (0.45, 1.48)	0.59 (0.31, 1.11)	0.30 (0.06, 1.44)	0.30 (0.07, 1.00)
BRO210mgIV	8.55 (0.73, 518.45)	11.06 (0.89, 644.98)	10.53 (0.87, 632.58)	**25.56 (1.28, 7401.80)**	**25.64 (1.45, 8975.64)**	**49.98 (2.80, 16087.33)**	8.76 (0.67, 521.71)	5.02 (0.31, 374.24)	13.17 (0.96, 853.80)	4.38 (0.37, 269.99)	3.16 (0.23, 174.44)	4.81 (0.36, 271.60)	4.37 (0.34, 244.73)	3.12 (0.27, 201.72)	5.50 (0.46, 364.81)	3.98 (0.30, 279.03)	5.97 (0.50, 381.97)	4.36 (0.35, 274.91)	2.32 (0.12, 152.94)	2.00 (0.13, 162.63)
BRO350mgIV	1.13 (0.15, 5.29)	1.47 (0.19, 6.72)	1.42 (0.18, 6.35)	3.30 (0.23, 97.96)	3.30 (0.27, 123.69)	6.54 (0.55, 218.21)	1.19 (0.14, 6.92)	0.66 (0.07, 4.71)	1.77 (0.23, 10.26)	0.57 (0.08, 2.40)	0.41 (0.05, 1.96)	0.64 (0.07, 2.97)	0.58 (0.07, 2.58)	0.42 (0.05, 1.69)	0.74 (0.09, 3.08)	0.54 (0.06, 2.63)	0.80 (0.10, 3.25)	0.57 (0.08, 2.42)	0.28 (0.03, 2.36)	0.27 (0.03, 1.64)
BRO700mgIV	1.79 (0.25, 11.75)	2.25 (0.30, 14.30)	2.19 (0.30, 13.94)	5.55 (0.38, 130.97)	5.45 (0.45, 160.27)	11.02 (0.90, 320.51)	1.95 (0.23, 12.26)	1.12 (0.11, 7.67)	2.87 (0.37, 18.51)	0.90 (0.13, 5.21)	0.66 (0.08, 4.29)	1.02 (0.13, 6.04)	0.93 (0.12, 5.64)	0.65 (0.09, 3.54)	1.14 (0.16, 6.29)	0.84 (0.10, 5.20)	1.25 (0.17, 6.67)	0.91 (0.12, 5.14)	0.45 (0.04, 5.04)	0.44 (0.05, 3.13)
CER100mgSC	**2.42 (1.12, 5.93)**	**3.04 (1.43, 7.37)**	**2.99 (1.43, 7.35)**	**7.59 (1.20, 136.04)**	**7.05 (1.29, 142.04)**	**14.58 (2.70, 268.79)**	2.59 (0.91, 7.86)	1.46 (0.40, 5.02)	**3.88 (1.29, 11.85)**	1.21 (0.71, 2.08)	0.89 (0.42, 1.96)	1.35 (0.69, 2.92)	1.25 (0.58, 2.68)	0.87 (0.53, 1.46)	1.53 (0.92, 2.72)	1.19 (0.51, 2.65)	**1.67 (1.01, 2.91)**	1.21 (0.66, 2.35)	0.62 (0.13, 2.96)	0.60 (0.15, 2.03)
CER10mg/kgIV	**4.12 (1.20, 16.38)**	**5.10 (1.61, 20.05)**	**4.95 (1.52, 19.88)**	**12.84 (1.70, 232.80)**	**11.79 (1.81, 308.78)**	**24.35 (3.54, 532.68)**	**4.29 (1.17, 19.33)**	2.36 (0.52, 11.88)	**6.41 (1.66, 30.38)**	2.01 (0.71, 6.22)	1.50 (0.47, 5.14)	2.29 (0.75, 7.79)	2.06 (0.60, 7.62)	1.43 (0.53, 4.43)	2.52 (0.91, 8.00)	1.95 (0.57, 7.70)	2.77 (1.00, 8.72)	2.02 (0.69, 6.58)	1.06 (0.14, 6.81)	1.00 (0.21, 4.96)
CER200mgSC	**3.48 (1.53, 8.67)**	**4.38 (1.95, 10.86)**	**4.20 (1.91, 10.60)**	**10.38 (1.70, 196.63)**	**10.04 (1.77, 186.43)**	**20.23 (3.56, 346.87)**	**3.61 (1.36, 10.93)**	2.03 (0.55, 7.44)	**5.45 (1.96, 17.26)**	1.71 (0.97, 3.36)	1.28 (0.57, 2.99)	1.95 (0.92, 4.46)	1.78 (0.80, 4.19)	1.23 (0.73, 2.32)	**2.17 (1.23, 4.28)**	1.67 (0.67, 4.29)	**2.36 (1.35, 4.60)**	1.71 (0.91, 3.51)	0.88 (0.18, 4.66)	0.87 (0.22, 2.95)
CER20mg/kgIV	**8.08 (2.11, 33.64)**	**10.17 (2.71, 43.36)**	**9.77 (2.57, 40.61)**	**26.07 (3.05, 531.08)**	**25.10 (2.90, 604.20)**	**49.88 (6.10, 1230.68)**	**8.60 (2.00, 39.50)**	**4.68 (1.01, 26.26)**	**13.06 (2.93, 60.54)**	**4.06 (1.22, 15.01)**	2.94 (0.77, 13.77)	**4.46 (1.31, 19.75)**	**4.03 (1.15, 18.43)**	2.92 (0.90, 10.72)	**5.11 (1.56, 19.39)**	**3.86 (1.04, 17.59)**	**5.60 (1.72, 21.30)**	**4.05 (1.18, 15.29)**	2.01 (0.32, 15.85)	1.96 (0.36, 11.57)
CER400mgSC	**2.27 (1.19, 4.74)**	**2.84 (1.58, 6.02)**	**2.77 (1.55, 5.89)**	**7.00 (1.22, 122.77)**	**6.65 (1.25, 118.55)**	**13.29 (2.59, 221.65)**	**2.35 (1.02, 6.34)**	1.34 (0.40, 4.43)	**3.55 (1.42, 9.72)**	1.14 (0.85, 1.54)	0.84 (0.45, 1.56)	1.28 (0.75, 2.22)	1.16 (0.64, 2.13)	0.82 (0.66, 1.01)	**1.46 (1.09, 1.96)**	1.10 (0.52, 2.17)	**1.58 (1.18, 2.11)**	1.13 (0.76, 1.79)	0.58 (0.13, 2.69)	0.58 (0.16, 1.72)
CER5mg/kgIV	2.78 (0.93, 8.64)	**3.47 (1.23, 10.66)**	**3.40 (1.24, 10.58)**	**8.71 (1.29, 177.85)**	**8.58 (1.31, 175.86)**	**16.81 (2.63, 331.83)**	2.90 (0.89, 10.51)	1.64 (0.43, 6.78)	**4.50 (1.27, 15.56)**	1.41 (0.56, 3.48)	1.04 (0.36, 3.03)	1.61 (0.58, 4.48)	1.44 (0.51, 4.32)	1.01 (0.41, 2.47)	1.77 (0.73, 4.45)	1.37 (0.46, 4.07)	1.92 (0.79, 4.85)	1.39 (0.55, 3.57)	0.71 (0.12, 4.39)	0.71 (0.15, 2.71)
ETA25mgSC	**5.86 (1.34, 29.57)**	**7.33 (1.72, 38.30)**	**7.13 (1.66, 36.10)**	**18.58 (1.86, 411.39)**	**17.42 (1.89, 440.46)**	**35.73 (3.82, 791.51)**	**6.18 (1.16, 37.59)**	3.50 (0.51, 22.20)	**9.22 (1.86, 54.61)**	2.86 (0.75, 13.17)	2.13 (0.49, 10.46)	3.27 (0.74, 15.69)	3.00 (0.71, 13.97)	2.06 (0.53, 9.29)	3.64 (0.93, 17.09)	2.77 (0.60, 13.69)	3.97 (1.00, 18.64)	2.88 (0.70, 12.74)	1.42 (0.23, 11.07)	1.47 (0.22, 9.12)
FIL100mgPO	**2.45 (1.17, 5.27)**	**3.08 (1.50, 6.77)**	**2.97 (1.49, 6.57)**	**7.30 (1.22, 138.07)**	**6.98 (1.28, 125.33)**	**14.17 (2.59, 233.19)**	2.50 (0.99, 6.99)	1.44 (0.40, 4.93)	**3.83 (1.40, 11.03)**	1.22 (0.77, 1.91)	0.90 (0.43, 1.86)	1.37 (0.72, 2.70)	1.24 (0.63, 2.56)	0.88 (0.58, 1.32)	1.55 (0.98, 2.46)	1.18 (0.52, 2.54)	**1.68 (1.07, 2.66)**	1.21 (0.72, 2.13)	0.63 (0.13, 2.83)	0.62 (0.16, 1.90)
FIL200mgPO	1.47 (0.75, 3.15)	1.84 (0.97, 3.97)	1.79 (0.94, 3.87)	4.42 (0.75, 75.37)	4.33 (0.78, 76.24)	**8.52 (1.65, 140.22)**	1.52 (0.64, 4.20)	0.86 (0.26, 2.94)	2.31 (0.92, 6.48)	0.74 (0.49, 1.07)	0.54 (0.27, 1.08)	0.83 (0.45, 1.54)	0.76 (0.39, 1.44)	**0.53 (0.38, 0.73)**	0.94 (0.64, 1.37)	0.71 (0.33, 1.46)	1.02 (0.70, 1.48)	0.73 (0.46, 1.24)	0.38 (0.08, 1.70)	0.38 (0.09, 1.13)

(TABLE S12B. continued)

	GUS1200mgIV	GUS200mgIV	GUS600mgIV	INF10mg/kgIV	INF20mg/kgIV	INF5mg/kgIV	MIR1000mgIV	MIR200mgIV	MIR600mgIV	NAT300mgIV	NAT3mg/kgIV	NAT3mg/kgIVx2	NAT6mg/kgIVx2	PBO	RIS1200mgIV	RIS200mgIV	RIS600mgIV	RIS600mgIV4_4	SEC10mg/kgIV	TOF15mgPO_BID
GUS1200mgIV	GUS1200mgIV	1.26 (0.88, 1.84)	1.22 (0.85, 1.79)	3.07 (0.43, 62.23)	2.99 (0.45, 57.15)	5.84 (0.92, 108.93)	1.03 (0.36, 3.11)	0.60 (0.15, 1.97)	1.59 (0.54, 4.82)	**0.50 (0.25, 0.94)**	**0.37 (0.15, 0.83)**	0.56 (0.24, 1.28)	0.51 (0.22, 1.16)	**0.36 (0.18, 0.66)**	0.64 (0.31, 1.22)	0.49 (0.18, 1.14)	0.69 (0.34, 1.32)	0.50 (0.23, 1.02)	0.25 (0.05, 1.26)	**0.26 (0.05, 0.81)**
GUS200mgIV	0.79 (0.54, 1.14)	GUS200mgIV	0.97 (0.69, 1.36)	2.40 (0.36, 48.69)	2.32 (0.36, 48.12)	4.57 (0.75, 85.68)	0.82 (0.29, 2.42)	0.48 (0.12, 1.56)	1.26 (0.43, 3.83)	**0.40 (0.19, 0.72)**	**0.29 (0.12, 0.65)**	**0.44 (0.19, 0.99)**	**0.40 (0.17, 0.94)**	**0.29 (0.14, 0.50)**	**0.51 (0.25, 0.91)**	**0.39 (0.14, 0.89)**	**0.55 (0.27, 0.99)**	**0.40 (0.19, 0.78)**	**0.20 (0.04, 0.99)**	**0.21 (0.04, 0.64)**
GUS600mgIV	0.82 (0.56, 1.18)	1.03 (0.74, 1.46)	GUS600mgIV	2.47 (0.37, 47.66)	2.43 (0.38, 46.73)	4.77 (0.79, 86.91)	0.85 (0.29, 2.56)	0.49 (0.12, 1.63)	1.28 (0.45, 3.84)	**0.41 (0.19, 0.74)**	**0.30 (0.12, 0.67)**	0.46 (0.20, 1.03)	**0.42 (0.18, 0.96)**	**0.29 (0.15, 0.52)**	**0.52 (0.26, 0.92)**	**0.40 (0.15, 0.90)**	0.57 (0.28, 1.01)	**0.41 (0.19, 0.81)**	0.21 (0.04, 1.00)	**0.21 (0.04, 0.67)**
INF10mg/kgIV	0.33 (0.02, 2.33)	0.42 (0.02, 2.78)	0.41 (0.02, 2.74)	INF10mg/kgIV	0.99 (0.40, 2.78)	1.93 (0.96, 4.63)	0.37 (0.02, 2.34)	0.20 (0.01, 1.51)	0.53 (0.03, 3.74)	**0.17 (0.01, 0.92)**	**0.12 (0.01, 0.78)**	0.19 (0.01, 1.11)	0.17 (0.01, 1.04)	**0.12 (0.01, 0.65)**	0.21 (0.01, 1.15)	**0.16 (0.01, 0.96)**	0.23 (0.01, 1.25)	**0.17 (0.01, 0.95)**	**0.08 (0.00, 0.86)**	**0.08 (0.00, 0.59)**
INF20mg/kgIV	0.33 (0.02, 2.23)	0.43 (0.02, 2.75)	0.41 (0.02, 2.61)	1.01 (0.36, 2.52)	INF20mg/kgIV	1.95 (0.85, 4.65)	0.36 (0.02, 2.32)	0.19 (0.01, 1.54)	0.53 (0.03, 3.62)	**0.17 (0.01, 0.90)**	**0.12 (0.01, 0.83)**	0.19 (0.01, 1.15)	0.18 (0.01, 1.08)	**0.12 (0.01, 0.64)**	0.22 (0.01, 1.13)	**0.16 (0.01, 0.92)**	0.24 (0.01, 1.24)	**0.17 (0.01, 0.91)**	**0.09 (0.00, 0.75)**	**0.08 (0.00, 0.64)**
INF5mg/kgIV	0.17 (0.01, 1.09)	0.22 (0.01, 1.32)	0.21 (0.01, 1.27)	0.52 (0.22, 1.04)	0.51 (0.22, 1.17)	INF5mg/kgIV	0.18 (0.01, 1.18)	**0.10 (0.01, 0.79)**	0.27 (0.02, 1.82)	**0.09 (0.01, 0.44)**	**0.06 (0.00, 0.38)**	**0.09 (0.01, 0.53)**	**0.09 (0.00, 0.51)**	**0.06 (0.00, 0.31)**	**0.11 (0.01, 0.55)**	**0.08 (0.00, 0.45)**	**0.12 (0.01, 0.61)**	**0.09 (0.01, 0.46)**	**0.04 (0.00, 0.39)**	**0.04 (0.00, 0.30)**
MIR1000mgIV	0.97 (0.32, 2.77)	1.22 (0.41, 3.42)	1.18 (0.39, 3.41)	2.72 (0.43, 47.52)	2.80 (0.43, 54.51)	5.47 (0.85, 104.29)	MIR1000mgIV	0.58 (0.21, 1.28)	1.50 (0.81, 2.76)	0.49 (0.19, 1.12)	**0.35 (0.11, 0.95)**	0.55 (0.18, 1.43)	0.49 (0.16, 1.33)	**0.35 (0.14, 0.77)**	0.62 (0.24, 1.39)	0.46 (0.15, 1.28)	0.67 (0.26, 1.52)	0.48 (0.18, 1.19)	0.25 (0.04, 1.42)	**0.24 (0.05, 0.85)**
MIR200mgIV	1.67 (0.51, 6.80)	2.07 (0.64, 8.44)	2.06 (0.61, 8.39)	4.92 (0.66, 94.47)	5.18 (0.65, 92.10)	**9.57 (1.27, 181.08)**	1.73 (0.78, 4.83)	MIR200mgIV	**2.60 (1.12, 8.00)**	0.85 (0.26, 2.76)	0.62 (0.17, 2.37)	0.98 (0.27, 3.43)	0.87 (0.24, 3.29)	0.61 (0.19, 1.96)	1.09 (0.34, 3.66)	0.82 (0.21, 3.14)	1.17 (0.37, 3.96)	0.83 (0.26, 2.80)	0.43 (0.05, 3.41)	0.43 (0.08, 1.87)
MIR600mgIV	0.63 (0.21, 1.84)	0.80 (0.26, 2.32)	0.78 (0.26, 2.24)	1.87 (0.27, 32.37)	1.89 (0.28, 33.67)	3.69 (0.55, 60.26)	0.67 (0.36, 1.24)	**0.38 (0.12, 0.89)**	MIR600mgIV	**0.32 (0.12, 0.78)**	**0.23 (0.07, 0.67)**	0.36 (0.12, 1.03)	**0.32 (0.11, 0.98)**	**0.23 (0.09, 0.56)**	0.41 (0.15, 1.01)	**0.30 (0.10, 0.92)**	0.44 (0.17, 1.12)	**0.32 (0.11, 0.82)**	**0.16 (0.03, 0.97)**	**0.16 (0.03, 0.58)**
NAT300mgIV	**1.99 (1.07, 4.05)**	**2.50 (1.39, 5.25)**	**2.42 (1.35, 5.18)**	**6.01 (1.09, 104.39)**	**5.84 (1.11, 99.37)**	**11.64 (2.26, 186.17)**	2.06 (0.89, 5.36)	1.18 (0.36, 3.84)	**3.12 (1.28, 8.46)**	NAT300mgIV	0.74 (0.39, 1.38)	1.12 (0.66, 1.96)	1.03 (0.57, 1.90)	**0.72 (0.58, 0.88)**	1.27 (0.96, 1.69)	0.96 (0.47, 1.92)	**1.38 (1.04, 1.83)**	0.99 (0.68, 1.49)	0.51 (0.11, 2.31)	0.51 (0.14, 1.51)
NAT3mg/kgIV	**2.67 (1.20, 6.55)**	**3.42 (1.54, 8.25)**	**3.34 (1.48, 8.10)**	**8.35 (1.28, 142.72)**	**8.05 (1.21, 134.73)**	**16.21 (2.65, 259.45)**	**2.85 (1.05, 8.72)**	1.62 (0.42, 5.76)	**4.33 (1.48, 13.47)**	1.36 (0.72, 2.55)	NAT3mg/kgIV	1.53 (0.94, 2.54)	1.39 (0.79, 2.48)	0.98 (0.54, 1.75)	1.73 (0.91, 3.22)	1.32 (0.51, 3.21)	1.88 (1.00, 3.47)	1.37 (0.69, 2.67)	0.71 (0.14, 3.52)	0.68 (0.17, 2.31)
NAT3mg/kgIVx2	1.79 (0.78, 4.11)	**2.25 (1.01, 5.14)**	2.19 (0.97, 4.91)	5.27 (0.90, 100.45)	5.28 (0.87, 95.76)	**10.57 (1.87, 173.46)**	1.83 (0.70, 5.52)	1.02 (0.29, 3.71)	2.81 (0.97, 8.57)	0.89 (0.51, 1.51)	0.65 (0.39, 1.07)	NAT3mg/kgIVx2	0.91 (0.54, 1.44)	0.64 (0.38, 1.05)	1.13 (0.64, 1.96)	0.86 (0.36, 1.96)	1.23 (0.70, 2.11)	0.89 (0.48, 1.62)	0.46 (0.10, 2.16)	0.44 (0.11, 1.51)
NAT6mg/kgIVx2	1.94 (0.86, 4.61)	**2.48 (1.07, 5.76)**	**2.41 (1.05, 5.64)**	5.89 (0.96, 110.32)	5.69 (0.93, 101.27)	**11.69 (1.96, 203.43)**	2.05 (0.75, 6.16)	1.15 (0.30, 4.20)	**3.13 (1.02, 9.48)**	0.97 (0.53, 1.77)	0.72 (0.40, 1.26)	1.09 (0.69, 1.84)	NAT6mg/kgIVx2	0.70 (0.40, 1.23)	1.24 (0.67, 2.25)	0.96 (0.38, 2.17)	1.34 (0.73, 2.43)	0.98 (0.50, 1.93)	0.50 (0.11, 2.45)	0.49 (0.12, 1.64)
PBO	**2.78 (1.51, 5.52)**	**3.47 (1.98, 7.01)**	**3.39 (1.93, 6.80)**	**8.35 (1.53, 145.48)**	**8.07 (1.57, 138.23)**	**16.19 (3.20, 261.44)**	**2.86 (1.29, 7.33)**	1.64 (0.51, 5.22)	**4.33 (1.77, 11.47)**	**1.39 (1.14, 1.71)**	1.02 (0.57, 1.84)	1.57 (0.95, 2.63)	1.43 (0.81, 2.53)	PBO	**1.77 (1.46, 2.18)**	1.35 (0.66, 2.58)	**1.92 (1.59, 2.35)**	**1.38 (1.01, 2.01)**	0.71 (0.16, 3.25)	0.70 (0.20, 2.07)
RIS1200mgIV	1.57 (0.82, 3.18)	**1.96 (1.10, 3.98)**	**1.91 (1.08, 3.89)**	4.76 (0.87, 81.96)	4.56 (0.89, 76.95)	**9.17 (1.83, 147.08)**	1.62 (0.72, 4.21)	0.92 (0.27, 2.93)	2.43 (0.99, 6.47)	0.79 (0.59, 1.05)	0.58 (0.31, 1.10)	0.88 (0.51, 1.55)	0.81 (0.44, 1.48)	**0.57 (0.46, 0.69)**	RIS1200mgIV	0.76 (0.38, 1.45)	1.09 (0.95, 1.25)	0.78 (0.54, 1.19)	0.40 (0.09, 1.83)	0.40 (0.11, 1.16)
RIS200mgIV	2.06 (0.88, 5.42)	**2.59 (1.13, 7.02)**	**2.51 (1.11, 6.65)**	**6.29 (1.05, 102.48)**	**6.12 (1.09, 104.34)**	**12.14 (2.22, 203.50)**	2.19 (0.78, 6.84)	1.23 (0.32, 4.72)	**3.29 (1.09, 10.46)**	1.04 (0.52, 2.12)	0.76 (0.31, 1.97)	1.17 (0.51, 2.78)	1.04 (0.46, 2.62)	0.74 (0.39, 1.52)	1.32 (0.69, 2.66)	RIS200mgIV	1.43 (0.76, 2.90)	1.04 (0.50, 2.29)	0.54 (0.11, 3.02)	0.53 (0.11, 1.83)
RIS600mgIV	1.45 (0.76, 2.92)	**1.81 (1.01, 3.68)**	1.77 (0.99, 3.59)	4.34 (0.80, 74.79)	4.22 (0.81, 71.60)	**8.47 (1.65, 134.07)**	1.50 (0.66, 3.87)	0.85 (0.25, 2.70)	2.27 (0.89, 5.93)	**0.72 (0.55, 0.96)**	0.53 (0.29, 1.00)	0.81 (0.47, 1.42)	0.75 (0.41, 1.36)	**0.52 (0.43, 0.63)**	0.92 (0.80, 1.06)	0.70 (0.34, 1.32)	RIS600mgIV	0.72 (0.50, 1.09)	0.38 (0.08, 1.73)	0.37 (0.10, 1.08)
RIS600mgIV4_4	2.01 (0.98, 4.34)	**2.51 (1.27, 5.35)**	**2.45 (1.24, 5.25)**	**5.94 (1.05, 102.17)**	**5.89 (1.10, 99.88)**	**11.55 (2.19, 194.72)**	2.07 (0.84, 5.50)	1.20 (0.36, 3.87)	**3.13 (1.23, 8.87)**	1.01 (0.67, 1.47)	0.73 (0.37, 1.44)	1.13 (0.62, 2.08)	1.02 (0.52, 1.99)	**0.73 (0.50, 0.99)**	1.29 (0.84, 1.86)	0.96 (0.44, 2.01)	1.39 (0.92, 2.00)	RIS600mgIV4_4	0.52 (0.11, 2.40)	0.51 (0.13, 1.57)
SEC10mg/kgIV	3.97 (0.79, 20.06)	**4.92 (1.01, 25.18)**	4.80 (1.00, 25.17)	**11.83 (1.17, 357.85)**	**11.67 (1.33, 343.50)**	**22.77 (2.59, 615.36)**	4.03 (0.71, 24.69)	2.35 (0.29, 18.43)	**6.28 (1.03, 34.09)**	1.95 (0.43, 9.09)	1.42 (0.28, 7.11)	2.18 (0.46, 10.43)	2.00 (0.41, 9.27)	1.41 (0.31, 6.39)	2.50 (0.55, 11.23)	1.87 (0.33, 9.51)	2.66 (0.58, 12.53)	1.93 (0.42, 9.36)	SEC10mg/kgIV	0.95 (0.12, 6.89)
TOF15mgPO_BID	**3.92 (1.24, 19.02)**	**4.87 (1.55, 23.27)**	**4.75 (1.49, 22.56)**	**11.99 (1.69, 247.11)**	**12.17 (1.57, 294.46)**	**23.00 (3.33, 443.80)**	**4.10 (1.18, 20.36)**	2.33 (0.53, 12.79)	**6.36 (1.71, 29.93)**	1.97 (0.66, 7.08)	1.47 (0.43, 6.03)	2.27 (0.66, 9.42)	2.04 (0.61, 8.12)	1.42 (0.48, 5.04)	2.50 (0.86, 9.15)	1.89 (0.55, 8.72)	2.72 (0.92, 9.71)	1.97 (0.64, 7.49)	1.06 (0.15, 8.03)	TOF15mgPO_BID

(TABLE S12B. continued)

	GUS1200mgIV	GUS200mgIV	GUS600mgIV	INF10mg/kgIV	INF20mg/kgIV	INF5mg/kgIV	MIR1000mgIV	MIR200mgIV	MIR600mgIV	NAT300mgIV	NAT3mg/kgIV	NAT3mg/kgIVx2	NAT6mg/kgIVx2	PBO	RIS1200mgIV	RIS200mgIV	RIS600mgIV	RIS600mgIV4_4	SEC10mg/kgIV	TOF15mgPO_BID
TOF1mgPO_BID	1.83 (0.60, 5.42)	2.29 (0.79, 6.77)	2.22 (0.74, 6.43)	5.44 (0.81, 99.78)	5.63 (0.82, 93.95)	**10.47 (1.67, 192.18)**	1.91 (0.60, 6.63)	1.08 (0.26, 4.35)	2.85 (0.90, 10.49)	0.92 (0.36, 2.16)	0.68 (0.22, 1.85)	1.04 (0.35, 2.60)	0.95 (0.32, 2.49)	0.66 (0.26, 1.50)	1.17 (0.45, 2.76)	0.88 (0.28, 2.76)	1.28 (0.50, 2.98)	0.92 (0.34, 2.23)	0.48 (0.08, 2.71)	0.47 (0.13, 1.10)
TOF5mgPO_BID	2.37 (0.76, 7.64)	3.00 (0.98, 9.37)	2.91 (0.93, 9.24)	**7.69 (1.04, 138.16)**	7.47 (0.95, 128.31)	**14.35 (2.01, 255.08)**	2.44 (0.69, 9.12)	1.42 (0.31, 5.79)	3.72 (1.00, 14.82)	1.20 (0.45, 2.88)	0.87 (0.27, 2.47)	1.35 (0.45, 3.76)	1.21 (0.39, 3.47)	0.87 (0.33, 2.02)	1.53 (0.57, 3.67)	1.13 (0.33, 3.58)	1.65 (0.62, 3.95)	1.19 (0.42, 3.01)	0.61 (0.11, 3.42)	0.58 (0.17, 1.85)
UPA12mgPO_BID	2.00 (0.47, 8.11)	2.51 (0.62, 10.10)	2.43 (0.60, 9.86)	6.09 (0.72, 142.22)	6.33 (0.69, 127.86)	**12.62 (1.36, 220.73)**	2.12 (0.45, 9.06)	1.17 (0.22, 7.50)	3.24 (0.69, 13.96)	1.01 (0.28, 3.27)	0.73 (0.18, 2.79)	1.11 (0.29, 3.97)	1.03 (0.26, 3.74)	0.73 (0.20, 2.24)	1.27 (0.35, 4.03)	0.97 (0.24, 3.35)	1.39 (0.39, 4.42)	1.00 (0.27, 3.35)	0.52 (0.07, 3.50)	0.51 (0.07, 2.61)
UPA24mgPO	3.59 (0.66, 17.46)	4.51 (0.87, 21.27)	4.44 (0.79, 20.55)	**10.85 (1.08, 272.26)**	**11.23 (1.04, 311.36)**	**20.79 (2.09, 585.74)**	3.66 (0.62, 20.10)	2.15 (0.30, 11.85)	5.62 (0.94, 29.88)	1.80 (0.37, 7.69)	1.33 (0.25, 5.99)	2.02 (0.40, 9.28)	1.82 (0.36, 8.78)	1.30 (0.27, 5.64)	2.29 (0.48, 10.10)	1.75 (0.32, 8.52)	2.48 (0.52, 10.94)	1.79 (0.37, 8.00)	0.91 (0.12, 7.79)	0.89 (0.11, 5.01)
UPA24mgPO_BID	1.13 (0.24, 3.63)	1.43 (0.31, 4.49)	1.39 (0.31, 4.27)	3.38 (0.38, 72.49)	3.36 (0.37, 69.91)	6.43 (0.71, 123.47)	1.19 (0.23, 4.31)	0.66 (0.12, 3.23)	1.82 (0.34, 6.98)	0.57 (0.15, 1.55)	0.41 (0.09, 1.28)	0.64 (0.15, 1.93)	0.57 (0.13, 1.83)	0.41 (0.11, 1.10)	0.71 (0.19, 2.01)	0.54 (0.13, 1.72)	0.77 (0.21, 2.23)	0.56 (0.15, 1.67)	0.28 (0.04, 1.78)	0.27 (0.04, 1.27)
UPA3mgPO_BID	2.91 (0.54, 12.58)	3.72 (0.73, 15.71)	3.54 (0.73, 15.00)	8.71 (0.86, 216.56)	8.88 (0.95, 215.15)	**17.41 (1.73, 393.42)**	3.10 (0.52, 15.07)	1.69 (0.29, 12.41)	4.72 (0.82, 21.75)	1.46 (0.33, 5.21)	1.08 (0.21, 4.26)	1.62 (0.33, 6.21)	1.48 (0.29, 6.02)	1.06 (0.23, 3.74)	1.82 (0.41, 6.76)	1.38 (0.27, 5.76)	1.99 (0.45, 7.54)	1.45 (0.32, 5.59)	0.72 (0.10, 5.20)	0.71 (0.09, 4.36)
UPA45mgPO	1.59 (0.83, 3.25)	**1.98 (1.10, 4.12)**	**1.94 (1.06, 3.97)**	4.80 (0.86, 85.41)	4.66 (0.86, 79.61)	**9.29 (1.79, 163.79)**	1.65 (0.72, 4.29)	0.94 (0.29, 3.29)	2.52 (0.98, 6.76)	0.80 (0.60, 1.08)	0.59 (0.32, 1.09)	0.90 (0.52, 1.57)	0.82 (0.44, 1.50)	**0.57 (0.46, 0.71)**	1.01 (0.76, 1.36)	0.77 (0.37, 1.51)	1.10 (0.81, 1.46)	0.79 (0.54, 1.29)	0.41 (0.09, 1.90)	0.40 (0.11, 1.20)
UPA6mgPO_BID	0.95 (0.20, 3.22)	1.18 (0.27, 4.06)	1.15 (0.26, 3.87)	2.78 (0.33, 58.19)	2.83 (0.32, 56.84)	5.24 (0.61, 99.80)	0.97 (0.19, 3.76)	0.52 (0.10, 2.57)	1.48 (0.30, 5.61)	0.47 (0.12, 1.23)	0.34 (0.08, 1.03)	0.52 (0.12, 1.59)	0.47 (0.11, 1.46)	**0.34 (0.09, 0.89)**	0.60 (0.16, 1.59)	0.45 (0.10, 1.42)	0.65 (0.17, 1.73)	0.46 (0.12, 1.34)	0.22 (0.04, 1.52)	0.22 (0.03, 1.03)
UST130mgIV	1.67 (0.83, 3.43)	**2.09 (1.09, 4.39)**	**2.03 (1.08, 4.27)**	5.07 (0.89, 85.55)	4.79 (0.93, 83.11)	**9.78 (1.89, 160.85)**	1.73 (0.71, 4.60)	0.98 (0.29, 3.38)	2.61 (0.99, 7.19)	0.84 (0.58, 1.16)	0.61 (0.32, 1.18)	0.93 (0.53, 1.69)	0.85 (0.46, 1.61)	**0.60 (0.45, 0.79)**	1.07 (0.76, 1.50)	0.80 (0.38, 1.60)	1.15 (0.82, 1.62)	0.83 (0.54, 1.40)	0.42 (0.09, 1.96)	0.42 (0.12, 1.27)
UST1mg/kgIV	1.45 (0.63, 3.54)	1.81 (0.81, 4.39)	1.76 (0.79, 4.28)	4.44 (0.73, 79.47)	4.34 (0.76, 78.74)	**8.69 (1.55, 154.08)**	1.52 (0.56, 4.63)	0.86 (0.24, 2.98)	2.26 (0.78, 7.06)	0.72 (0.42, 1.26)	0.52 (0.24, 1.23)	0.81 (0.40, 1.78)	0.73 (0.35, 1.62)	**0.52 (0.31, 0.87)**	0.92 (0.54, 1.60)	0.70 (0.30, 1.67)	1.00 (0.58, 1.75)	0.73 (0.40, 1.35)	0.37 (0.07, 1.83)	0.37 (0.09, 1.19)
UST3mg/kgIV	1.37 (0.62, 3.34)	1.73 (0.80, 4.19)	1.68 (0.79, 4.08)	4.15 (0.69, 72.29)	3.92 (0.73, 77.38)	**8.09 (1.48, 139.70)**	1.43 (0.53, 4.29)	0.80 (0.22, 2.89)	2.17 (0.73, 6.84)	0.69 (0.40, 1.19)	0.50 (0.23, 1.08)	0.77 (0.39, 1.58)	0.70 (0.34, 1.52)	**0.49 (0.30, 0.82)**	0.86 (0.51, 1.53)	0.65 (0.29, 1.53)	0.94 (0.56, 1.64)	0.68 (0.37, 1.29)	0.35 (0.07, 1.71)	0.34 (0.09, 1.18)
UST4.5mg/kgIV	1.01 (0.22, 4.30)	1.30 (0.27, 5.48)	1.27 (0.27, 5.20)	3.44 (0.33, 71.29)	3.02 (0.34, 80.70)	6.31 (0.68, 144.59)	1.08 (0.20, 5.34)	0.60 (0.09, 3.32)	1.63 (0.28, 8.26)	0.51 (0.12, 1.77)	0.38 (0.08, 1.43)	0.57 (0.12, 2.16)	0.53 (0.12, 2.02)	0.36 (0.09, 1.29)	0.64 (0.15, 2.29)	0.49 (0.10, 2.07)	0.70 (0.17, 2.52)	0.51 (0.12, 1.90)	0.26 (0.03, 1.99)	0.24 (0.04, 1.44)
UST6mg/kg90mgIV/SC	0.99 (0.66, 1.50)	1.25 (0.87, 1.84)	1.22 (0.83, 1.80)	3.04 (0.46, 56.39)	2.95 (0.47, 59.17)	5.76 (0.99, 110.59)	1.04 (0.37, 3.08)	0.60 (0.14, 2.04)	1.56 (0.53, 4.74)	**0.50 (0.24, 0.91)**	**0.36 (0.15, 0.84)**	0.56 (0.24, 1.24)	0.51 (0.22, 1.18)	**0.36 (0.18, 0.64)**	0.64 (0.31, 1.15)	0.48 (0.18, 1.13)	0.69 (0.34, 1.25)	**0.50 (0.23, 0.98)**	0.25 (0.05, 1.23)	**0.25 (0.05, 0.81)**
UST6mg/kgIV	1.29 (0.64, 2.65)	1.61 (0.85, 3.34)	1.56 (0.83, 3.25)	3.89 (0.68, 65.17)	3.75 (0.70, 63.13)	**7.46 (1.49, 121.18)**	1.32 (0.54, 3.51)	0.76 (0.23, 2.46)	2.03 (0.78, 5.45)	**0.64 (0.46, 0.88)**	**0.47 (0.25, 0.90)**	0.72 (0.41, 1.28)	0.65 (0.36, 1.23)	**0.46 (0.36, 0.59)**	0.82 (0.59, 1.12)	0.62 (0.30, 1.26)	0.89 (0.64, 1.21)	0.64 (0.42, 1.01)	0.33 (0.07, 1.51)	**0.32 (0.09, 0.97)**
UST90mgSC	2.77 (0.85, 9.41)	**3.53 (1.07, 11.75)**	**3.37 (1.03, 11.50)**	**8.92 (1.11, 158.44)**	**8.43 (1.17, 159.30)**	**17.22 (2.36, 296.71)**	2.90 (0.71, 12.42)	1.65 (0.33, 7.91)	**4.38 (1.07, 19.48)**	1.40 (0.47, 4.04)	1.02 (0.32, 3.44)	1.56 (0.50, 5.19)	1.41 (0.46, 4.70)	1.01 (0.34, 2.85)	1.80 (0.60, 5.17)	1.32 (0.38, 4.57)	1.95 (0.66, 5.63)	1.38 (0.46, 4.15)	0.73 (0.12, 3.96)	0.68 (0.13, 3.16)
VED0_5mg/kgIV	1.98 (0.80, 5.23)	**2.49 (1.05, 6.29)**	**2.39 (1.02, 6.20)**	6.13 (1.00, 118.05)	6.00 (1.00, 107.79)	**11.65 (2.08, 202.69)**	2.05 (0.73, 6.40)	1.18 (0.32, 4.39)	**3.12 (1.03, 9.67)**	0.99 (0.50, 1.90)	0.73 (0.30, 1.76)	1.12 (0.48, 2.59)	1.02 (0.43, 2.36)	0.71 (0.37, 1.34)	1.26 (0.64, 2.42)	0.96 (0.37, 2.30)	1.37 (0.69, 2.65)	1.00 (0.49, 2.05)	0.52 (0.10, 2.51)	0.50 (0.13, 1.63)
VED2mg/kgIV	1.53 (0.64, 3.78)	1.92 (0.83, 4.84)	1.85 (0.81, 4.64)	4.62 (0.75, 88.20)	4.63 (0.79, 85.28)	**8.73 (1.63, 158.69)**	1.62 (0.57, 4.97)	0.90 (0.25, 3.35)	2.42 (0.83, 7.63)	0.77 (0.41, 1.43)	0.56 (0.24, 1.34)	0.86 (0.40, 1.96)	0.78 (0.35, 1.85)	0.55 (0.30, 1.00)	0.98 (0.52, 1.81)	0.73 (0.29, 1.81)	1.06 (0.56, 2.01)	0.76 (0.38, 1.54)	0.41 (0.07, 1.87)	0.39 (0.10, 1.27)
VED300mgIV	1.31 (0.65, 2.78)	1.64 (0.85, 3.55)	1.62 (0.82, 3.42)	3.93 (0.69, 73.31)	3.87 (0.69, 66.17)	**7.65 (1.43, 132.16)**	1.37 (0.58, 3.62)	0.79 (0.24, 2.53)	2.07 (0.81, 5.45)	**0.66 (0.44, 0.96)**	**0.48 (0.25, 0.96)**	0.74 (0.40, 1.37)	0.67 (0.34, 1.31)	**0.48 (0.34, 0.65)**	0.84 (0.57, 1.22)	0.64 (0.30, 1.29)	0.91 (0.61, 1.33)	0.66 (0.41, 1.06)	0.34 (0.07, 1.59)	0.33 (0.09, 1.01)
VED300mgIV	0.89 (0.55, 1.44)	1.36 (0.66, 2.86)	1.68 (0.86, 3.56)	1.63 (0.83, 3.53)	5.50 (0.64, 621.68)	5.70 (0.60, 640.31)	**11.22 (1.35, 1184.11)**	1.35 (0.55, 3.89)	0.77 (0.22, 2.82)	2.08 (0.81, 6.27)	**0.66 (0.44, 0.98)**	**0.50 (0.26, 0.95)**	0.75 (0.42, 1.37)	0.69 (0.36, 1.30)	**0.47 (0.33, 0.66)**	0.83 (0.56, 1.22)	0.63 (0.29, 1.28)	0.90 (0.61, 1.33)	0.66 (0.41, 1.07)	0.34 (0.07, 1.57)

(TABLE S12B. continued)

	TOF1mgPO_BID	TOF5mgPO_BID	UPA12mgPO_BID	UPA24mgPO	UPA24mgPO_BID	UPA3mgPO_BID	UPA45mgPO	UPA6mgPO_BID	UST130mgIV	UST1mg/kgIV	UST3mg/kgIV	UST4.5mg/kgIV	UST6mg/kg90mgIV/SC	UST6mg/kgIV	UST90mgSC	VED0_5mg/kgIV	VED2mg/kgIV	VED300mgIV	VED300mgIV
ABA10mg/kgIV	2.72 (0.69, 12.59)	2.03 (0.51, 9.88)	2.39 (0.48, 13.27)	1.34 (0.24, 9.28)	**4.31 (1.06, 24.39)**	1.68 (0.31, 10.06)	**3.08 (1.08, 10.11)**	**5.17 (1.22, 28.20)**	**2.93 (1.02, 9.80)**	3.35 (1.00, 11.87)	**3.48 (1.07, 12.01)**	4.68 (0.84, 26.19)	**4.91 (1.50, 17.73)**	**3.82 (1.34, 12.84)**	1.74 (0.41, 7.80)	2.43 (0.72, 8.89)	3.17 (0.95, 11.08)	**3.70 (1.28, 12.53)**	**3.32 (1.14, 12.10)**
ABA30mg/kgIV	1.35 (0.37, 6.28)	1.00 (0.28, 4.77)	1.23 (0.24, 7.37)	0.67 (0.14, 5.16)	2.13 (0.54, 13.68)	0.84 (0.16, 5.68)	1.52 (0.54, 5.42)	2.69 (0.63, 15.71)	1.46 (0.52, 5.18)	1.71 (0.52, 6.01)	1.76 (0.54, 6.56)	2.42 (0.44, 13.43)	2.42 (0.78, 9.33)	1.91 (0.68, 6.67)	0.88 (0.21, 4.23)	1.24 (0.37, 4.63)	1.60 (0.48, 6.21)	1.82 (0.63, 6.22)	1.79 (0.59, 6.52)
ABA3mg/kgIV	2.26 (0.54, 8.87)	1.76 (0.38, 6.73)	1.95 (0.42, 11.00)	1.15 (0.16, 7.71)	3.55 (0.81, 22.74)	1.36 (0.27, 9.43)	2.55 (0.82, 7.57)	**4.27 (1.05, 25.06)**	2.46 (0.79, 7.38)	2.81 (0.76, 8.97)	2.99 (0.82, 9.18)	4.22 (0.65, 21.55)	**4.11 (1.18, 14.05)**	**3.18 (1.02, 9.56)**	1.49 (0.32, 7.35)	2.04 (0.59, 7.25)	2.64 (0.76, 9.32)	3.07 (1.00, 9.34)	2.89 (0.87, 8.65)
ADA160mg80mg40mgSC	0.57 (0.19, 1.72)	0.43 (0.14, 1.39)	0.52 (0.13, 2.19)	0.29 (0.06, 1.55)	0.92 (0.29, 4.18)	0.36 (0.08, 1.80)	0.65 (0.31, 1.29)	1.11 (0.31, 5.02)	0.62 (0.29, 1.27)	0.72 (0.29, 1.69)	0.75 (0.31, 1.70)	1.03 (0.24, 4.72)	1.05 (0.82, 1.33)	0.82 (0.38, 1.62)	0.37 (0.11, 1.25)	0.53 (0.20, 1.31)	0.68 (0.27, 1.63)	0.80 (0.36, 1.64)	0.78 (0.34, 1.61)
ADA160mg80mg60mgSC	**0.26 (0.08, 0.82)**	**0.20 (0.06, 0.67)**	0.24 (0.05, 1.00)	**0.13 (0.03, 0.71)**	0.43 (0.12, 1.83)	**0.16 (0.04, 0.80)**	**0.30 (0.13, 0.62)**	0.52 (0.14, 2.10)	**0.29 (0.12, 0.61)**	**0.33 (0.12, 0.77)**	**0.34 (0.13, 0.85)**	0.46 (0.11, 2.40)	0.47 (0.17, 1.35)	**0.37 (0.16, 0.80)**	**0.17 (0.05, 0.62)**	**0.24 (0.09, 0.61)**	**0.31 (0.11, 0.76)**	**0.37 (0.15, 0.79)**	**0.37 (0.16, 0.80)**
ADA160mg80mgSC	0.56 (0.20, 1.56)	0.42 (0.16, 1.28)	0.51 (0.15, 1.94)	0.29 (0.06, 1.47)	0.91 (0.32, 3.51)	0.36 (0.09, 1.57)	0.64 (0.39, 1.05)	1.08 (0.37, 4.31)	0.61 (0.35, 1.06)	0.70 (0.36, 1.39)	0.74 (0.37, 1.45)	0.99 (0.25, 4.67)	1.04 (0.48, 2.33)	0.80 (0.47, 1.35)	0.37 (0.11, 1.17)	0.51 (0.23, 1.14)	0.66 (0.31, 1.44)	0.77 (0.45, 1.36)	0.77 (0.43, 1.33)
ADA40mg20mgSC	1.03 (0.35, 3.19)	0.78 (0.26, 2.63)	0.92 (0.24, 4.05)	0.52 (0.10, 2.85)	1.64 (0.53, 7.23)	0.65 (0.15, 3.16)	1.17 (0.61, 2.38)	2.01 (0.61, 8.78)	1.12 (0.54, 2.34)	1.29 (0.56, 3.03)	1.38 (0.59, 3.10)	1.85 (0.42, 8.72)	1.91 (0.76, 4.94)	1.46 (0.73, 3.02)	0.67 (0.19, 2.32)	0.94 (0.39, 2.42)	1.22 (0.50, 3.08)	1.42 (0.69, 3.03)	1.43 (0.66, 3.08)
ADA80mg40mgSC	0.65 (0.23, 1.84)	0.49 (0.17, 1.53)	0.60 (0.16, 2.23)	0.33 (0.07, 1.69)	1.03 (0.35, 4.16)	0.42 (0.10, 1.93)	0.73 (0.41, 1.34)	1.27 (0.40, 5.14)	0.70 (0.38, 1.35)	0.81 (0.39, 1.72)	0.85 (0.40, 1.80)	1.16 (0.29, 5.46)	1.21 (0.51, 2.83)	0.91 (0.50, 1.74)	0.42 (0.12, 1.36)	0.59 (0.25, 1.38)	0.76 (0.34, 1.75)	0.90 (0.48, 1.70)	0.88 (0.47, 1.72)
BRO210mgIV	4.75 (0.35, 343.87)	3.74 (0.23, 303.25)	4.94 (0.21, 228.47)	2.64 (0.10, 156.36)	8.60 (0.44, 411.99)	3.35 (0.16, 168.32)	5.53 (0.47, 358.70)	10.37 (0.50, 497.77)	5.19 (0.44, 346.74)	5.87 (0.48, 346.53)	6.47 (0.49, 338.61)	8.66 (0.50, 602.21)	8.73 (0.70, 511.23)	6.82 (0.56, 446.40)	3.01 (0.21, 211.82)	4.36 (0.31, 279.13)	5.64 (0.41, 366.22)	6.53 (0.57, 439.59)	3.26 (0.31, 57.32)
BRO350mgIV	0.62 (0.07, 3.23)	0.48 (0.05, 2.76)	0.60 (0.05, 3.85)	0.35 (0.02, 2.40)	1.11 (0.09, 6.68)	0.40 (0.04, 2.89)	0.73 (0.09, 2.82)	1.34 (0.10, 7.16)	0.69 (0.09, 2.79)	0.78 (0.10, 3.27)	0.83 (0.10, 3.66)	1.13 (0.10, 7.72)	1.17 (0.15, 5.12)	0.89 (0.12, 3.59)	0.42 (0.04, 2.52)	0.56 (0.07, 2.65)	0.73 (0.09, 3.44)	0.89 (0.11, 3.57)	0.78 (0.09, 3.52)
BRO700mgIV	0.99 (0.12, 6.50)	0.77 (0.09, 5.29)	0.94 (0.08, 6.33)	0.52 (0.04, 4.79)	1.73 (0.16, 11.99)	0.65 (0.06, 5.39)	1.14 (0.16, 6.39)	2.16 (0.18, 13.87)	1.08 (0.15, 5.97)	1.22 (0.17, 7.24)	1.29 (0.18, 8.40)	1.84 (0.17, 14.95)	1.80 (0.25, 11.76)	1.41 (0.20, 7.64)	0.67 (0.07, 5.02)	0.90 (0.12, 5.70)	1.15 (0.15, 6.94)	1.38 (0.19, 7.43)	1.27 (0.15, 9.36)
CER100mgSC	1.32 (0.52, 3.82)	1.02 (0.38, 3.01)	1.20 (0.33, 4.92)	0.69 (0.14, 3.64)	2.15 (0.69, 8.98)	0.83 (0.23, 4.02)	1.53 (0.90, 2.71)	2.61 (0.85, 10.40)	1.46 (0.83, 2.62)	1.69 (0.82, 3.42)	1.76 (0.90, 3.55)	2.43 (0.63, 10.59)	**2.46 (1.14, 5.82)**	**1.90 (1.08, 3.34)**	0.87 (0.28, 2.86)	1.25 (0.53, 2.75)	1.61 (0.74, 3.48)	**1.85 (1.05, 3.44)**	**2.09 (1.24, 3.50)**
CER10mg/kgIV	2.25 (0.56, 9.75)	1.65 (0.42, 7.97)	2.03 (0.43, 10.39)	1.11 (0.18, 6.64)	3.71 (0.89, 16.77)	1.43 (0.28, 7.06)	2.49 (0.89, 8.00)	**4.45 (1.09, 19.67)**	2.37 (0.83, 7.77)	2.79 (0.87, 9.20)	2.98 (0.92, 9.74)	4.03 (0.81, 24.73)	**4.17 (1.23, 15.54)**	**3.10 (1.12, 9.82)**	1.54 (0.30, 6.89)	1.99 (0.67, 7.31)	2.64 (0.83, 9.26)	**3.14 (1.06, 9.90)**	**1.86 (1.02, 3.47)**
CER200mgSC	1.89 (0.73, 5.61)	1.42 (0.51, 4.46)	1.70 (0.48, 6.92)	0.97 (0.21, 5.18)	3.04 (0.96, 12.29)	1.20 (0.31, 5.56)	**2.14 (1.23, 4.28)**	**3.66 (1.19, 15.06)**	**2.05 (1.11, 4.14)**	**2.39 (1.12, 5.30)**	**2.54 (1.17, 5.69)**	3.44 (0.83, 17.16)	**3.51 (1.51, 8.60)**	**2.67 (1.45, 5.26)**	1.24 (0.37, 4.33)	1.76 (0.73, 4.02)	2.27 (0.97, 5.15)	**2.62 (1.39, 5.39)**	**3.14 (1.01, 12.62)**
CER20mg/kgIV	**4.35 (1.07, 23.21)**	3.37 (0.80, 19.32)	3.85 (0.82, 28.03)	2.24 (0.37, 18.93)	**7.19 (1.45, 50.00)**	2.69 (0.47, 19.68)	**5.10 (1.56, 19.14)**	**8.73 (1.94, 59.73)**	**4.90 (1.46, 19.14)**	**5.60 (1.44, 23.20)**	**5.81 (1.59, 25.79)**	**8.05 (1.47, 58.82)**	**8.10 (2.15, 35.07)**	**6.34 (1.93, 25.12)**	2.98 (0.58, 16.36)	**4.02 (1.08, 17.60)**	**5.25 (1.27, 22.68)**	**6.27 (1.84, 24.30)**	**2.58 (1.41, 5.15)**
CER400mgSC	1.23 (0.52, 3.24)	0.95 (0.39, 2.59)	1.13 (0.35, 4.19)	0.63 (0.14, 3.08)	2.02 (0.74, 7.79)	0.78 (0.22, 3.44)	**1.43 (1.05, 1.95)**	2.42 (0.89, 9.22)	1.37 (0.97, 1.94)	1.57 (0.89, 2.69)	1.67 (0.95, 2.78)	2.28 (0.62, 9.46)	**2.29 (1.24, 4.72)**	**1.78 (1.28, 2.49)**	0.82 (0.28, 2.42)	1.15 (0.59, 2.26)	1.48 (0.80, 2.77)	**1.73 (1.17, 2.60)**	**5.28 (1.66, 25.74)**
CER5mg/kgIV	1.54 (0.49, 5.15)	1.15 (0.37, 4.40)	1.41 (0.33, 7.20)	0.78 (0.15, 4.45)	2.47 (0.71, 11.99)	0.98 (0.20, 4.88)	1.76 (0.72, 4.30)	3.03 (0.82, 13.70)	1.68 (0.66, 4.28)	1.96 (0.70, 5.35)	2.06 (0.76, 5.62)	2.84 (0.59, 13.73)	2.78 (0.97, 8.58)	2.18 (0.88, 5.54)	1.02 (0.24, 4.03)	1.41 (0.52, 4.15)	1.81 (0.66, 5.10)	2.13 (0.81, 5.33)	**1.73 (1.15, 2.57)**
ETA25mgSC	3.32 (0.57, 18.09)	2.44 (0.43, 15.50)	2.82 (0.48, 18.59)	1.56 (0.24, 14.31)	5.35 (0.99, 34.19)	2.00 (0.29, 15.43)	3.54 (0.88, 16.13)	**6.34 (1.11, 41.89)**	3.39 (0.83, 15.21)	3.92 (0.94, 18.40)	**4.14 (1.02, 20.14)**	5.62 (0.95, 43.18)	**5.84 (1.35, 28.87)**	**4.41 (1.12, 20.75)**	2.06 (0.39, 13.02)	2.93 (0.66, 15.54)	3.75 (0.84, 19.39)	**4.39 (1.09, 20.67)**	2.11 (0.79, 5.42)
FIL100mgPO	1.33 (0.51, 3.72)	1.02 (0.38, 2.88)	1.21 (0.34, 4.57)	0.68 (0.15, 3.43)	2.21 (0.67, 8.74)	0.83 (0.22, 3.75)	1.53 (0.97, 2.46)	2.61 (0.89, 10.20)	1.45 (0.90, 2.42)	1.68 (0.86, 3.26)	1.77 (0.91, 3.46)	2.43 (0.62, 9.98)	**2.45 (1.20, 5.29)**	**1.91 (1.18, 3.09)**	0.87 (0.29, 2.68)	1.23 (0.57, 2.62)	1.59 (0.76, 3.30)	**1.86 (1.11, 3.09)**	**4.28 (1.01, 25.01)**
FIL200mgPO	0.80 (0.32, 2.18)	0.61 (0.24, 1.72)	0.73 (0.22, 2.66)	0.41 (0.09, 2.08)	1.30 (0.46, 5.24)	0.51 (0.13, 2.32)	0.93 (0.63, 1.37)	1.57 (0.56, 6.03)	0.88 (0.58, 1.36)	1.02 (0.55, 1.89)	1.07 (0.60, 1.95)	1.47 (0.39, 6.05)	1.47 (0.76, 3.16)	1.15 (0.77, 1.74)	0.53 (0.18, 1.57)	0.74 (0.36, 1.53)	0.95 (0.49, 1.92)	1.12 (0.72, 1.78)	**1.83 (1.07, 3.12)**

	TOF1mgPO_BID	TOF5mgPO_BID	UPA12mgPO_BID	UPA24mgPO	UPA24mgPO_BID	UPA3mgPO_BID	UPA45mgPO	UPA6mgPO_BID	UST130mgIV	UST1mg/kgIV	UST3mg/kgIV	UST4.5mg/kgIV	UST6mg/kg90mgIV/SC	UST6mg/kgIV	UST90mgSC	VED0_5mg/kgIV	VED2mg/kgIV	VED300mgIV	VED300mgIV
GUS1200mgIV	0.55 (0.18, 1.65)	0.42 (0.13, 1.32)	0.50 (0.12, 2.13)	0.28 (0.06, 1.50)	0.88 (0.28, 4.15)	0.34 (0.08, 1.84)	0.63 (0.31, 1.21)	1.06 (0.31, 4.91)	0.60 (0.29, 1.21)	0.69 (0.28, 1.58)	0.73 (0.30, 1.62)	0.99 (0.23, 4.63)	1.01 (0.67, 1.51)	0.78 (0.38, 1.55)	0.36 (0.11, 1.17)	0.50 (0.19, 1.26)	0.65 (0.26, 1.55)	0.76 (0.36, 1.54)	1.13 (0.70, 1.82)
GUS200mgIV	0.44 (0.15, 1.27)	0.33 (0.11, 1.02)	0.40 (0.10, 1.60)	0.22 (0.05, 1.15)	0.70 (0.22, 3.22)	0.27 (0.06, 1.38)	**0.50 (0.24, 0.91)**	0.85 (0.25, 3.77)	**0.48 (0.23, 0.92)**	0.55 (0.23, 1.23)	0.58 (0.24, 1.24)	0.77 (0.18, 3.71)	0.80 (0.54, 1.15)	0.62 (0.30, 1.18)	**0.28 (0.09, 0.93)**	**0.40 (0.16, 0.95)**	0.52 (0.21, 1.21)	0.61 (0.28, 1.17)	0.74 (0.35, 1.51)
GUS600mgIV	0.45 (0.16, 1.35)	0.34 (0.11, 1.08)	0.41 (0.10, 1.66)	0.23 (0.05, 1.27)	0.72 (0.23, 3.24)	0.28 (0.07, 1.38)	**0.52 (0.25, 0.95)**	0.87 (0.26, 3.90)	**0.49 (0.23, 0.93)**	0.57 (0.23, 1.26)	0.60 (0.24, 1.26)	0.79 (0.19, 3.73)	0.82 (0.56, 1.20)	0.64 (0.31, 1.21)	**0.30 (0.09, 0.97)**	**0.42 (0.16, 0.98)**	0.54 (0.22, 1.23)	0.62 (0.29, 1.22)	0.60 (0.28, 1.16)
INF10mg/kgIV	0.18 (0.01, 1.23)	**0.13 (0.01, 0.96)**	0.16 (0.01, 1.40)	**0.09 (0.00, 0.93)**	0.30 (0.01, 2.63)	0.11 (0.00, 1.16)	0.21 (0.01, 1.16)	0.36 (0.02, 3.06)	0.20 (0.01, 1.12)	0.23 (0.01, 1.36)	0.24 (0.01, 1.46)	0.29 (0.01, 3.04)	0.33 (0.02, 2.19)	0.26 (0.02, 1.48)	**0.11 (0.01, 0.90)**	0.16 (0.01, 1.00)	0.22 (0.01, 1.33)	0.25 (0.01, 1.46)	0.61 (0.28, 1.21)
INF20mg/kgIV	0.18 (0.01, 1.21)	0.13 (0.01, 1.05)	0.16 (0.01, 1.46)	**0.09 (0.00, 0.96)**	0.30 (0.01, 2.71)	0.11 (0.00, 1.05)	0.21 (0.01, 1.16)	0.35 (0.02, 3.12)	0.21 (0.01, 1.08)	0.23 (0.01, 1.31)	0.26 (0.01, 1.38)	0.33 (0.01, 2.92)	0.34 (0.02, 2.15)	0.27 (0.02, 1.42)	**0.12 (0.01, 0.85)**	0.17 (0.01, 1.00)	0.22 (0.01, 1.27)	0.26 (0.02, 1.44)	0.18 (0.00, 1.57)
INF5mg/kgIV	**0.10 (0.01, 0.60)**	**0.07 (0.00, 0.50)**	**0.08 (0.00, 0.73)**	**0.05 (0.00, 0.48)**	0.16 (0.01, 1.41)	**0.06 (0.00, 0.58)**	**0.11 (0.01, 0.56)**	0.19 (0.01, 1.65)	**0.10 (0.01, 0.53)**	**0.12 (0.01, 0.64)**	**0.12 (0.01, 0.68)**	0.16 (0.01, 1.47)	0.17 (0.01, 1.01)	**0.13 (0.01, 0.67)**	**0.06 (0.00, 0.42)**	**0.09 (0.00, 0.48)**	**0.11 (0.01, 0.61)**	**0.13 (0.01, 0.70)**	0.18 (0.00, 1.66)
MIR1000mgIV	0.52 (0.15, 1.66)	0.41 (0.11, 1.44)	0.47 (0.11, 2.23)	0.27 (0.05, 1.61)	0.84 (0.23, 4.32)	0.32 (0.07, 1.91)	0.61 (0.23, 1.38)	1.04 (0.27, 5.19)	0.58 (0.22, 1.40)	0.66 (0.22, 1.79)	0.70 (0.23, 1.87)	0.93 (0.19, 5.04)	0.97 (0.33, 2.73)	0.76 (0.29, 1.84)	0.34 (0.08, 1.41)	0.49 (0.16, 1.38)	0.62 (0.20, 1.74)	0.73 (0.28, 1.74)	**0.09 (0.00, 0.74)**
MIR200mgIV	0.92 (0.23, 3.85)	0.70 (0.17, 3.18)	0.85 (0.13, 4.54)	0.47 (0.08, 3.31)	1.51 (0.31, 8.18)	0.59 (0.08, 3.45)	1.06 (0.30, 3.48)	1.91 (0.39, 9.56)	1.02 (0.30, 3.42)	1.17 (0.34, 4.25)	1.24 (0.35, 4.57)	1.66 (0.30, 11.24)	1.68 (0.49, 7.08)	1.31 (0.41, 4.42)	0.61 (0.13, 3.06)	0.85 (0.23, 3.10)	1.11 (0.30, 3.94)	1.27 (0.40, 4.22)	0.74 (0.26, 1.83)
MIR600mgIV	0.35 (0.10, 1.12)	0.27 (0.07, 1.00)	0.31 (0.07, 1.46)	0.18 (0.03, 1.06)	0.55 (0.14, 2.90)	0.21 (0.05, 1.22)	0.40 (0.15, 1.02)	0.67 (0.18, 3.28)	0.38 (0.14, 1.01)	0.44 (0.14, 1.28)	0.46 (0.15, 1.38)	0.61 (0.12, 3.56)	0.64 (0.21, 1.87)	0.49 (0.18, 1.29)	**0.23 (0.05, 0.93)**	**0.32 (0.10, 0.97)**	0.41 (0.13, 1.20)	0.48 (0.18, 1.24)	1.30 (0.36, 4.52)
NAT300mgIV	1.08 (0.46, 2.77)	0.83 (0.35, 2.21)	0.99 (0.31, 3.60)	0.55 (0.13, 2.71)	1.76 (0.64, 6.78)	0.69 (0.19, 3.04)	1.25 (0.93, 1.68)	2.14 (0.81, 8.04)	1.19 (0.86, 1.71)	1.38 (0.79, 2.38)	1.46 (0.84, 2.50)	1.98 (0.56, 8.19)	**2.00 (1.09, 4.09)**	**1.56 (1.13, 2.15)**	0.71 (0.25, 2.11)	1.01 (0.53, 1.99)	1.30 (0.70, 2.45)	**1.52 (1.04, 2.26)**	0.48 (0.16, 1.24)
NAT3mg/kgIV	1.47 (0.54, 4.51)	1.14 (0.40, 3.76)	1.36 (0.36, 5.70)	0.75 (0.17, 4.08)	2.41 (0.78, 11.18)	0.93 (0.23, 4.69)	1.70 (0.92, 3.15)	2.97 (0.97, 12.50)	1.64 (0.85, 3.15)	1.91 (0.81, 4.09)	1.99 (0.92, 4.37)	2.65 (0.70, 12.67)	**2.76 (1.19, 6.70)**	**2.13 (1.11, 4.04)**	0.98 (0.29, 3.17)	1.38 (0.57, 3.31)	1.77 (0.74, 4.14)	**2.08 (1.04, 4.08)**	**1.52 (1.02, 2.26)**
NAT3mg/kgIVx2	0.96 (0.38, 2.87)	0.74 (0.27, 2.23)	0.90 (0.25, 3.50)	0.49 (0.11, 2.52)	1.57 (0.52, 6.85)	0.62 (0.16, 3.00)	1.12 (0.64, 1.91)	1.93 (0.63, 8.01)	1.07 (0.59, 1.89)	1.24 (0.56, 2.51)	1.30 (0.63, 2.56)	1.75 (0.46, 8.06)	1.80 (0.80, 4.23)	1.40 (0.78, 2.42)	0.64 (0.19, 2.01)	0.89 (0.39, 2.08)	1.17 (0.51, 2.49)	1.36 (0.73, 2.48)	**2.00 (1.06, 3.88)**
NAT6mg/kgIVx2	1.05 (0.40, 3.16)	0.82 (0.29, 2.58)	0.97 (0.27, 3.90)	0.55 (0.11, 2.80)	1.75 (0.55, 7.52)	0.67 (0.17, 3.43)	1.22 (0.67, 2.26)	2.11 (0.69, 8.71)	1.17 (0.62, 2.18)	1.36 (0.62, 2.89)	1.43 (0.66, 2.94)	1.88 (0.50, 8.56)	1.98 (0.84, 4.64)	1.53 (0.81, 2.81)	0.71 (0.21, 2.18)	0.98 (0.42, 2.35)	1.28 (0.54, 2.89)	1.50 (0.76, 2.92)	1.33 (0.73, 2.39)
PBO	1.51 (0.66, 3.85)	1.15 (0.50, 3.06)	1.38 (0.45, 4.90)	0.77 (0.18, 3.72)	2.46 (0.91, 9.30)	0.94 (0.27, 4.26)	**1.74 (1.41, 2.18)**	**2.95 (1.13, 11.14)**	**1.67 (1.27, 2.21)**	**1.92 (1.15, 3.22)**	**2.03 (1.22, 3.32)**	2.74 (0.78, 11.52)	**2.77 (1.57, 5.56)**	**2.16 (1.70, 2.81)**	0.99 (0.35, 2.91)	1.40 (0.75, 2.72)	1.81 (1.00, 3.28)	**2.10 (1.55, 2.98)**	1.44 (0.77, 2.77)
RIS1200mgIV	0.86 (0.36, 2.21)	0.65 (0.27, 1.75)	0.79 (0.25, 2.83)	0.44 (0.10, 2.10)	1.40 (0.50, 5.27)	0.55 (0.15, 2.42)	0.99 (0.73, 1.32)	1.66 (0.63, 6.37)	0.94 (0.67, 1.32)	1.08 (0.62, 1.87)	1.16 (0.65, 1.94)	1.57 (0.44, 6.46)	1.57 (0.87, 3.23)	1.23 (0.89, 1.70)	0.56 (0.19, 1.68)	0.79 (0.41, 1.57)	1.02 (0.55, 1.93)	1.19 (0.82, 1.76)	**2.11 (1.52, 3.00)**
RIS200mgIV	1.14 (0.36, 3.61)	0.88 (0.28, 3.01)	1.04 (0.30, 4.23)	0.57 (0.12, 3.16)	1.87 (0.58, 7.88)	0.73 (0.17, 3.77)	1.30 (0.66, 2.69)	2.23 (0.71, 9.66)	1.25 (0.62, 2.61)	1.44 (0.60, 3.39)	1.53 (0.65, 3.42)	2.03 (0.48, 9.69)	2.08 (0.89, 5.44)	1.62 (0.79, 3.37)	0.76 (0.22, 2.63)	1.05 (0.43, 2.70)	1.37 (0.55, 3.41)	1.57 (0.77, 3.37)	1.20 (0.82, 1.78)
RIS600mgIV	0.78 (0.34, 2.00)	0.60 (0.25, 1.62)	0.72 (0.23, 2.58)	0.40 (0.09, 1.92)	1.29 (0.45, 4.82)	0.50 (0.13, 2.23)	0.91 (0.68, 1.23)	1.53 (0.58, 5.85)	0.87 (0.62, 1.22)	1.00 (0.57, 1.71)	1.06 (0.61, 1.79)	1.43 (0.40, 6.02)	1.45 (0.80, 2.97)	1.13 (0.82, 1.56)	0.51 (0.18, 1.52)	0.73 (0.38, 1.45)	0.94 (0.50, 1.77)	1.10 (0.75, 1.63)	1.60 (0.78, 3.46)
RIS600mgIV4_4	1.09 (0.45, 2.91)	0.84 (0.33, 2.36)	1.00 (0.30, 3.77)	0.56 (0.12, 2.71)	1.79 (0.60, 6.89)	0.69 (0.18, 3.14)	1.27 (0.77, 1.86)	2.16 (0.74, 8.35)	1.21 (0.72, 1.86)	1.37 (0.74, 2.52)	1.46 (0.77, 2.69)	1.96 (0.53, 8.49)	**2.00 (1.02, 4.27)**	1.57 (0.99, 2.35)	0.73 (0.24, 2.17)	1.00 (0.49, 2.05)	1.31 (0.65, 2.60)	1.52 (0.94, 2.44)	1.11 (0.75, 1.64)
SEC10mg/kgIV	2.10 (0.37, 11.82)	1.65 (0.29, 9.20)	1.94 (0.29, 13.76)	1.10 (0.13, 8.51)	3.53 (0.56, 23.11)	1.38 (0.19, 10.13)	2.46 (0.53, 11.46)	4.52 (0.66, 28.34)	2.36 (0.51, 11.06)	2.70 (0.55, 13.48)	2.85 (0.59, 13.51)	3.92 (0.50, 31.49)	3.93 (0.81, 20.37)	3.07 (0.66, 14.28)	1.38 (0.25, 8.61)	1.93 (0.40, 10.33)	2.45 (0.53, 13.44)	2.96 (0.63, 13.73)	1.51 (0.94, 2.42)
TOF15mgPO_BID	2.15 (0.91, 7.54)	1.71 (0.54, 5.75)	1.96 (0.38, 13.37)	1.12 (0.20, 8.72)	3.73 (0.79, 24.16)	1.41 (0.23, 10.71)	2.49 (0.83, 8.90)	4.46 (0.97, 29.14)	2.37 (0.79, 8.46)	2.73 (0.84, 10.71)	2.92 (0.85, 11.60)	4.10 (0.70, 25.70)	**3.95 (1.24, 18.40)**	**3.10 (1.03, 11.01)**	1.47 (0.32, 7.94)	2.00 (0.61, 7.98)	2.57 (0.79, 10.52)	3.02 (0.99, 11.40)	2.97 (0.64, 14.46)

	TOF1mgPO_BID	TOF5mgPO_BID	UPA12mgPO_BID	UPA24mgPO	UPA24mgPO_BID	UPA3mgPO_BID	UPA45mgPO	UPA6mgPO_BID	UST130mgIV	UST1mg/kgIV	UST3mg/kgIV	UST4.5mg/kgIV	UST6mg/kg90mgIV/SC	UST6mg/kgIV	UST90mgSC	VED0_5mg/kgIV	VED2mg/kgIV	VED300mgIV	VED300mgIV
TOF1mgPO_BID	TOF1mgPO_BID	0.78 (0.33, 1.64)	0.89 (0.21, 4.08)	0.51 (0.10, 2.98)	1.67 (0.42, 6.98)	0.62 (0.14, 3.08)	1.17 (0.45, 2.74)	2.01 (0.50, 8.61)	1.11 (0.42, 2.62)	1.25 (0.43, 3.46)	1.34 (0.46, 3.59)	1.81 (0.33, 9.89)	1.82 (0.63, 5.49)	1.45 (0.55, 3.36)	0.65 (0.16, 2.38)	0.91 (0.31, 2.48)	1.17 (0.41, 3.15)	1.38 (0.53, 3.48)	**3.15 (1.08, 10.96)**
TOF5mgPO_BID	1.28 (0.61, 2.99)	TOF5mgPO_BID	1.16 (0.26, 5.80)	0.66 (0.12, 3.88)	2.13 (0.51, 10.24)	0.81 (0.17, 4.65)	1.51 (0.56, 3.64)	2.59 (0.65, 11.87)	1.44 (0.52, 3.52)	1.67 (0.55, 4.36)	1.76 (0.58, 4.61)	2.38 (0.45, 12.74)	2.38 (0.77, 7.51)	1.87 (0.68, 4.56)	0.84 (0.21, 3.37)	1.19 (0.39, 3.45)	1.56 (0.51, 4.24)	1.80 (0.67, 4.63)	1.41 (0.55, 3.45)
UPA12mgPO_BID	1.12 (0.25, 4.69)	0.86 (0.17, 3.87)	UPA12mgPO_BID	0.59 (0.13, 2.30)	1.86 (0.64, 5.61)	0.72 (0.18, 2.34)	1.27 (0.35, 4.02)	2.17 (0.91, 6.60)	1.21 (0.34, 3.95)	1.38 (0.35, 5.00)	1.48 (0.38, 5.19)	2.05 (0.34, 11.92)	2.01 (0.48, 8.16)	1.59 (0.44, 5.05)	0.77 (0.13, 3.57)	1.02 (0.24, 3.84)	1.29 (0.32, 4.92)	1.56 (0.40, 5.06)	1.92 (0.69, 5.13)
UPA24mgPO	1.95 (0.34, 10.07)	1.51 (0.26, 8.39)	1.69 (0.43, 7.50)	UPA24mgPO	3.14 (0.99, 12.72)	1.22 (0.29, 6.47)	2.25 (0.47, 9.91)	**3.75 (1.28, 15.05)**	2.20 (0.44, 9.79)	2.40 (0.47, 11.85)	2.52 (0.50, 12.18)	3.43 (0.47, 28.84)	3.60 (0.68, 16.82)	2.82 (0.58, 12.73)	1.29 (0.19, 8.89)	1.74 (0.34, 9.75)	2.28 (0.43, 12.10)	2.69 (0.54, 12.71)	1.52 (0.39, 6.20)
UPA24mgPO_BID	0.60 (0.14, 2.39)	0.47 (0.10, 1.96)	0.54 (0.18, 1.56)	0.32 (0.08, 1.01)	UPA24mgPO_BID	0.39 (0.11, 1.13)	0.71 (0.19, 2.03)	1.19 (0.56, 2.69)	0.67 (0.17, 1.95)	0.77 (0.19, 2.53)	0.81 (0.20, 2.56)	1.11 (0.19, 6.37)	1.14 (0.25, 3.58)	0.87 (0.23, 2.48)	0.41 (0.07, 1.69)	0.56 (0.14, 1.76)	0.70 (0.18, 2.26)	0.86 (0.22, 2.49)	2.71 (0.64, 14.10)
UPA3mgPO_BID	1.61 (0.32, 7.12)	1.24 (0.22, 5.96)	1.39 (0.43, 5.56)	0.82 (0.15, 3.47)	2.58 (0.89, 9.11)	UPA3mgPO_BID	1.85 (0.42, 6.83)	**3.10 (1.11, 10.21)**	1.76 (0.38, 6.40)	1.98 (0.41, 7.89)	2.13 (0.46, 8.05)	2.81 (0.43, 17.83)	2.94 (0.57, 12.29)	2.31 (0.50, 8.27)	1.06 (0.17, 5.23)	1.47 (0.31, 5.83)	1.88 (0.39, 7.65)	2.26 (0.48, 8.13)	0.82 (0.22, 2.56)
UPA45mgPO	0.86 (0.36, 2.22)	0.66 (0.27, 1.77)	0.79 (0.25, 2.86)	0.44 (0.10, 2.12)	1.42 (0.49, 5.37)	0.54 (0.15, 2.40)	UPA45mgPO	1.70 (0.62, 6.35)	0.96 (0.67, 1.36)	1.10 (0.62, 1.92)	1.16 (0.67, 1.96)	1.58 (0.44, 6.70)	1.59 (0.86, 3.29)	1.24 (0.89, 1.75)	0.57 (0.19, 1.71)	0.80 (0.41, 1.58)	1.03 (0.54, 1.96)	1.21 (0.82, 1.80)	2.13 (0.42, 10.96)
UPA6mgPO_BID	0.50 (0.12, 2.01)	0.39 (0.08, 1.54)	0.46 (0.15, 1.10)	**0.27 (0.07, 0.78)**	0.84 (0.37, 1.78)	**0.32 (0.10, 0.90)**	0.59 (0.16, 1.60)	UPA6mgPO_BID	0.56 (0.14, 1.56)	0.64 (0.16, 1.94)	0.68 (0.17, 1.97)	0.90 (0.16, 5.18)	0.94 (0.21, 3.18)	0.73 (0.20, 1.99)	0.34 (0.06, 1.38)	0.47 (0.12, 1.49)	0.60 (0.15, 1.90)	0.71 (0.18, 2.05)	1.21 (0.81, 1.83)
UST130mgIV	0.90 (0.38, 2.40)	0.69 (0.28, 1.92)	0.83 (0.25, 2.98)	0.46 (0.10, 2.28)	1.49 (0.51, 5.81)	0.57 (0.16, 2.61)	1.04 (0.73, 1.48)	1.78 (0.64, 6.98)	UST130mgIV	1.16 (0.65, 1.91)	1.22 (0.71, 2.05)	1.63 (0.46, 6.91)	1.67 (0.86, 3.43)	**1.30 (1.04, 1.64)**	0.60 (0.20, 1.78)	0.84 (0.42, 1.68)	1.08 (0.56, 2.07)	1.26 (0.82, 2.00)	0.69 (0.19, 2.11)
UST1mg/kgIV	0.80 (0.29, 2.35)	0.60 (0.23, 1.82)	0.72 (0.20, 2.85)	0.42 (0.08, 2.13)	1.30 (0.39, 5.32)	0.50 (0.13, 2.42)	0.91 (0.52, 1.60)	1.57 (0.52, 6.37)	0.86 (0.52, 1.54)	UST1mg/kgIV	1.06 (0.62, 1.79)	1.43 (0.38, 6.26)	1.45 (0.65, 3.51)	1.13 (0.70, 1.92)	0.53 (0.16, 1.71)	0.73 (0.32, 1.65)	0.95 (0.43, 2.05)	1.10 (0.62, 2.04)	1.27 (0.82, 1.99)
UST3mg/kgIV	0.75 (0.28, 2.19)	0.57 (0.22, 1.74)	0.68 (0.19, 2.64)	0.40 (0.08, 2.02)	1.24 (0.39, 4.92)	0.47 (0.12, 2.18)	0.86 (0.51, 1.50)	1.47 (0.51, 5.71)	0.82 (0.49, 1.41)	0.94 (0.56, 1.61)	UST3mg/kgIV	1.35 (0.36, 6.02)	1.39 (0.64, 3.27)	1.07 (0.67, 1.76)	0.49 (0.16, 1.56)	0.70 (0.31, 1.55)	0.90 (0.42, 1.96)	1.05 (0.58, 1.93)	1.10 (0.60, 2.07)
UST4.5mg/kgIV	0.55 (0.10, 3.04)	0.42 (0.08, 2.22)	0.49 (0.08, 2.91)	0.29 (0.03, 2.12)	0.90 (0.16, 5.40)	0.36 (0.06, 2.34)	0.63 (0.15, 2.29)	1.11 (0.19, 6.43)	0.61 (0.14, 2.18)	0.70 (0.16, 2.64)	0.74 (0.17, 2.78)	UST4.5mg/kgIV	1.05 (0.22, 4.30)	0.80 (0.19, 2.88)	0.36 (0.07, 1.95)	0.51 (0.11, 2.07)	0.67 (0.15, 2.67)	0.78 (0.18, 2.79)	1.05 (0.57, 2.05)
UST6mg/kg90mgIV/SC	0.55 (0.18, 1.60)	0.42 (0.13, 1.30)	0.50 (0.12, 2.10)	0.28 (0.06, 1.47)	0.88 (0.28, 3.92)	0.34 (0.08, 1.75)	0.63 (0.30, 1.16)	1.06 (0.31, 4.72)	0.60 (0.29, 1.16)	0.69 (0.28, 1.54)	0.72 (0.31, 1.56)	0.95 (0.23, 4.46)	UST6mg/kg90mgIV/SC	0.78 (0.38, 1.49)	0.36 (0.11, 1.14)	0.50 (0.19, 1.21)	0.65 (0.26, 1.52)	0.76 (0.36, 1.52)	0.85 (0.19, 2.88)
UST6mg/kgIV	0.69 (0.30, 1.83)	0.53 (0.22, 1.46)	0.63 (0.20, 2.27)	0.35 (0.08, 1.73)	1.15 (0.40, 4.32)	0.43 (0.12, 2.00)	0.80 (0.57, 1.12)	1.37 (0.50, 5.12)	**0.77 (0.61, 0.97)**	0.89 (0.52, 1.43)	0.94 (0.57, 1.49)	1.25 (0.35, 5.34)	1.28 (0.67, 2.65)	UST6mg/kgIV	0.46 (0.16, 1.35)	0.65 (0.32, 1.30)	0.83 (0.44, 1.60)	0.97 (0.65, 1.49)	0.74 (0.33, 1.50)
UST90mgSC	1.54 (0.42, 6.11)	1.19 (0.30, 4.76)	1.30 (0.28, 7.56)	0.78 (0.11, 5.31)	2.45 (0.59, 13.59)	0.95 (0.19, 5.95)	1.77 (0.59, 5.17)	2.93 (0.73, 16.36)	1.67 (0.56, 4.94)	1.90 (0.59, 6.11)	2.04 (0.64, 6.38)	2.77 (0.51, 15.01)	2.78 (0.88, 9.41)	2.18 (0.74, 6.25)	UST90mgSC	1.40 (0.44, 4.86)	1.78 (0.56, 6.29)	2.13 (0.70, 6.35)	0.97 (0.63, 1.52)
VED0_5mg/kgIV	1.10 (0.40, 3.24)	0.84 (0.29, 2.58)	0.98 (0.26, 4.21)	0.57 (0.10, 2.93)	1.80 (0.57, 7.01)	0.68 (0.17, 3.18)	1.24 (0.63, 2.43)	2.15 (0.67, 8.31)	1.19 (0.60, 2.37)	1.37 (0.61, 3.08)	1.44 (0.65, 3.19)	1.95 (0.48, 9.00)	1.99 (0.83, 5.23)	1.55 (0.77, 3.08)	0.72 (0.21, 2.27)	VED0_5mg/kgIV	1.29 (0.75, 2.17)	1.50 (0.74, 3.09)	1.92 (0.65, 5.66)
VED2mg/kgIV	0.85 (0.32, 2.46)	0.64 (0.24, 1.94)	0.77 (0.20, 3.11)	0.44 (0.08, 2.31)	1.43 (0.44, 5.53)	0.53 (0.13, 2.56)	0.97 (0.51, 1.86)	1.67 (0.53, 6.60)	0.93 (0.48, 1.78)	1.05 (0.49, 2.30)	1.11 (0.51, 2.39)	1.49 (0.37, 6.88)	1.53 (0.66, 3.80)	1.20 (0.63, 2.26)	0.56 (0.16, 1.77)	0.78 (0.46, 1.33)	VED2mg/kgIV	1.16 (0.59, 2.35)	1.49 (0.72, 3.19)
VED300mgIV	0.72 (0.29, 1.88)	0.56 (0.22, 1.50)	0.64 (0.20, 2.48)	0.37 (0.08, 1.87)	1.17 (0.40, 4.61)	0.44 (0.12, 2.07)	0.83 (0.56, 1.22)	1.42 (0.49, 5.63)	0.79 (0.50, 1.22)	0.91 (0.49, 1.62)	0.95 (0.52, 1.73)	1.28 (0.36, 5.53)	1.32 (0.66, 2.79)	1.03 (0.67, 1.54)	0.47 (0.16, 1.44)	0.67 (0.32, 1.35)	0.86 (0.43, 1.68)	VED300mgIV	1.17 (0.56, 2.35)
VED300mgIV	**0.32 (0.09, 0.93)**	0.71 (0.29, 1.82)	0.52 (0.19, 1.45)	0.66 (0.16, 2.57)	0.37 (0.07, 1.56)	1.21 (0.39, 4.46)	0.47 (0.09, 2.38)	0.83 (0.55, 1.24)	1.46 (0.47, 5.24)	0.79 (0.50, 1.22)	0.91 (0.48, 1.67)	0.95 (0.49, 1.76)	1.18 (0.35, 5.22)	1.34 (0.67, 2.99)	1.03 (0.66, 1.59)	0.52 (0.18, 1.53)	0.67 (0.31, 1.38)	0.85 (0.43, 1.79)	VED300mgIV

Table S12C: League table for clinical remission for FDA-approved drugs only.

	ADA160mg80mg60mgSC	ADA160mg80mgSC	ADA40mg20mgSC	ADA80mg40mgSC	BRI400mgIV	CER400mgSC	FIL200mgPO	INF10mg/kgIV	INF20mg/kgIV	INF5mg/kgIV	MIR1000mgIV	MIR200mgIV	MIR600mgIV	NAT3mg/kgIV
ADA160mg80mg60mgSC	ADA160mg80mg60mgSC	0.43 (0.04, 3.74)	0.24 (0.03, 2.50)	0.37 (0.04, 3.73)	0.19 (0.01, 4.28)	0.22 (0.02, 2.20)	1.02 (0.07, 18.12)	1.51 (0.08, 67.76)	1.46 (0.07, 64.94)	3.00 (0.17, 121.61)	1.88 (0.11, 93.28)	1.16 (0.06, 67.05)	1.54 (0.07, 73.29)	0.18 (0.02, 2.16)
ADA160mg80mgSC	2.35 (0.27, 22.81)	ADA160mg80mgSC	0.56 (0.13, 2.78)	0.88 (0.28, 3.27)	0.45 (0.02, 8.89)	0.50 (0.05, 5.15)	2.36 (0.25, 38.49)	3.55 (0.25, 131.12)	3.46 (0.24, 142.55)	6.81 (0.51, 291.46)	4.41 (0.29, 224.60)	2.75 (0.16, 141.04)	3.56 (0.19, 164.29)	0.42 (0.05, 3.85)
ADA40mg20mgSC	4.19 (0.40, 39.68)	1.79 (0.36, 7.44)	ADA40mg20mgSC	1.54 (0.34, 7.72)	0.79 (0.04, 18.53)	0.91 (0.08, 9.15)	4.19 (0.32, 76.56)	6.03 (0.37, 273.38)	6.11 (0.37, 250.66)	11.84 (0.72, 484.74)	7.69 (0.49, 448.64)	4.85 (0.25, 235.47)	6.44 (0.32, 266.09)	0.75 (0.08, 8.16)
ADA80mg40mgSC	2.68 (0.27, 23.45)	1.14 (0.31, 3.60)	0.65 (0.13, 2.93)	ADA80mg40mgSC	0.50 (0.03, 8.75)	0.58 (0.06, 4.87)	2.73 (0.26, 40.59)	3.93 (0.24, 133.27)	3.89 (0.24, 167.98)	7.84 (0.50, 273.26)	4.91 (0.32, 240.81)	3.17 (0.16, 152.40)	4.10 (0.20, 164.61)	0.48 (0.06, 3.40)
BRI400mgIV	5.22 (0.23, 114.74)	2.24 (0.11, 43.58)	1.27 (0.05, 27.32)	1.98 (0.11, 36.47)	BRI400mgIV	1.13 (0.05, 23.50)	5.47 (0.20, 212.93)	7.86 (0.22, 588.93)	7.64 (0.22, 549.20)	15.78 (0.49, 1217.68)	10.09 (0.32, 919.03)	6.33 (0.16, 506.69)	7.87 (0.23, 695.04)	0.96 (0.04, 21.62)
CER400mgSC	4.61 (0.46, 66.51)	1.98 (0.19, 19.34)	1.10 (0.11, 13.13)	1.73 (0.21, 17.00)	0.88 (0.04, 21.35)	CER400mgSC	4.83 (0.37, 86.96)	6.88 (0.44, 324.98)	6.75 (0.38, 293.47)	13.69 (0.75, 583.39)	8.60 (0.62, 515.75)	5.23 (0.29, 312.30)	7.21 (0.38, 351.47)	0.82 (0.10, 10.18)
FIL200mgPO	0.98 (0.06, 14.02)	0.42 (0.03, 4.07)	0.24 (0.01, 3.15)	0.37 (0.02, 3.81)	0.18 (0.00, 4.96)	0.21 (0.01, 2.68)	FIL200mgPO	1.44 (0.05, 70.93)	1.41 (0.05, 73.51)	2.76 (0.11, 128.13)	1.79 (0.07, 120.48)	1.10 (0.04, 80.95)	1.39 (0.05, 93.89)	0.18 (0.01, 2.47)
INF10mg/kgIV	0.66 (0.01, 12.92)	0.28 (0.01, 4.04)	0.17 (0.00, 2.69)	0.25 (0.01, 4.13)	0.13 (0.00, 4.50)	0.15 (0.00, 2.25)	0.69 (0.01, 19.37)	INF10mg/kgIV	1.00 (0.16, 5.68)	1.98 (0.38, 11.37)	1.25 (0.02, 95.53)	0.81 (0.01, 65.02)	1.00 (0.02, 67.31)	0.12 (0.00, 2.50)
INF20mg/kgIV	0.68 (0.02, 13.54)	0.29 (0.01, 4.20)	0.16 (0.00, 2.73)	0.26 (0.01, 4.14)	0.13 (0.00, 4.63)	0.15 (0.00, 2.64)	0.71 (0.01, 20.06)	1.00 (0.18, 6.40)	INF20mg/kgIV	1.99 (0.35, 12.65)	1.32 (0.02, 82.09)	0.78 (0.01, 59.38)	1.01 (0.02, 66.48)	0.12 (0.00, 2.31)
INF5mg/kgIV	0.33 (0.01, 6.04)	0.15 (0.00, 1.96)	0.08 (0.00, 1.39)	0.13 (0.00, 2.00)	0.06 (0.00, 2.03)	0.07 (0.00, 1.33)	0.36 (0.01, 9.49)	0.51 (0.09, 2.62)	0.50 (0.08, 2.86)	INF5mg/kgIV	0.62 (0.01, 46.05)	0.38 (0.01, 30.90)	0.49 (0.01, 33.13)	0.06 (0.00, 1.11)
MIR1000mgIV	0.53 (0.01, 9.12)	0.23 (0.00, 3.44)	0.13 (0.00, 2.02)	0.20 (0.00, 3.14)	0.10 (0.00, 3.15)	0.12 (0.00, 1.62)	0.56 (0.01, 14.27)	0.80 (0.01, 48.48)	0.76 (0.01, 44.85)	1.62 (0.02, 83.50)	MIR1000mgIV	0.62 (0.08, 4.09)	0.79 (0.11, 4.54)	0.10 (0.00, 1.56)
MIR200mgIV	0.86 (0.01, 15.70)	0.36 (0.01, 6.42)	0.21 (0.00, 4.00)	0.32 (0.01, 6.41)	0.16 (0.00, 6.29)	0.19 (0.00, 3.41)	0.91 (0.01, 26.18)	1.24 (0.02, 98.96)	1.29 (0.02, 78.41)	2.65 (0.03, 157.29)	1.61 (0.24, 13.14)	MIR200mgIV	1.28 (0.17, 9.36)	0.15 (0.00, 3.69)
MIR600mgIV	0.65 (0.01, 14.19)	0.28 (0.01, 5.16)	0.16 (0.00, 3.12)	0.24 (0.01, 4.91)	0.13 (0.00, 4.40)	0.14 (0.00, 2.65)	0.72 (0.01, 19.54)	1.00 (0.01, 63.02)	0.99 (0.02, 61.67)	2.05 (0.03, 124.74)	1.26 (0.22, 9.33)	0.78 (0.11, 5.81)	MIR600mgIV	0.12 (0.00, 2.64)
NAT3mg/kgIV	5.51 (0.46, 63.21)	2.38 (0.26, 19.03)	1.34 (0.12, 12.81)	2.07 (0.29, 16.86)	1.05 (0.05, 23.64)	1.22 (0.10, 10.02)	5.67 (0.40, 106.95)	8.29 (0.40, 352.70)	8.01 (0.43, 360.98)	15.90 (0.90, 672.84)	10.36 (0.64, 503.28)	6.58 (0.27, 357.52)	8.19 (0.38, 394.27)	NAT3mg/kgIV
NAT3mg/kgIVx2	3.63 (0.30, 47.47)	1.58 (0.18, 13.07)	0.87 (0.08, 9.74)	1.36 (0.17, 12.37)	0.71 (0.03, 15.94)	0.81 (0.08, 8.79)	3.75 (0.28, 77.29)	5.56 (0.28, 249.85)	5.43 (0.33, 249.70)	10.71 (0.68, 473.67)	7.15 (0.42, 368.77)	4.17 (0.22, 279.95)	5.56 (0.26, 301.29)	0.67 (0.14, 3.60)
NAT6mg/kgIVx2	3.91 (0.35, 45.95)	1.70 (0.19, 15.70)	0.96 (0.10, 10.68)	1.51 (0.20, 13.96)	0.78 (0.04, 18.65)	0.86 (0.08, 8.85)	4.09 (0.32, 84.54)	5.93 (0.29, 285.51)	5.88 (0.33, 263.31)	11.82 (0.77, 509.79)	7.53 (0.48, 442.46)	4.51 (0.25, 337.13)	6.17 (0.32, 353.21)	0.72 (0.15, 4.41)
NNC2mg/kgSC	2.76 (0.22, 42.13)	1.17 (0.10, 12.79)	0.68 (0.05, 8.83)	1.06 (0.11, 11.98)	0.53 (0.02, 12.47)	0.61 (0.05, 7.65)	2.91 (0.20, 66.74)	4.15 (0.18, 236.89)	4.12 (0.18, 218.70)	7.93 (0.37, 401.42)	5.59 (0.23, 304.38)	3.36 (0.12, 186.85)	4.25 (0.16, 244.24)	0.51 (0.03, 6.51)
ONE10mgSC	4.37 (0.40, 55.29)	1.87 (0.21, 16.76)	1.07 (0.12, 12.74)	1.63 (0.21, 14.76)	0.83 (0.04, 18.14)	0.96 (0.09, 10.33)	4.57 (0.38, 90.74)	6.55 (0.35, 297.69)	6.50 (0.39, 322.03)	12.78 (0.71, 549.66)	8.24 (0.57, 438.76)	5.15 (0.26, 315.11)	6.78 (0.33, 311.54)	0.80 (0.08, 8.18)
ONE25mgSC	9.63 (0.79, 152.66)	4.11 (0.43, 40.96)	2.37 (0.21, 32.88)	3.63 (0.41, 43.42)	1.93 (0.08, 42.66)	2.12 (0.17, 25.87)	10.04 (0.67, 215.08)	14.26 (0.68, 770.80)	14.26 (0.70, 733.75)	**28.24 (1.33, 1330.76)**	18.42 (1.00, 1158.52)	11.64 (0.48, 727.90)	14.80 (0.68, 731.24)	1.76 (0.15, 22.11)
ONE34mgSC	4.37 (0.41, 49.41)	1.85 (0.20, 15.75)	1.04 (0.10, 12.86)	1.61 (0.20, 15.23)	0.84 (0.03, 19.02)	0.96 (0.08, 9.20)	4.55 (0.34, 84.41)	6.56 (0.35, 331.38)	6.41 (0.32, 331.43)	12.86 (0.69, 605.95)	8.17 (0.47, 431.90)	5.10 (0.25, 311.49)	6.99 (0.32, 323.65)	0.80 (0.07, 9.23)
ONE50mgSC	6.35 (0.50, 81.15)	2.81 (0.24, 25.14)	1.56 (0.13, 16.53)	2.43 (0.23, 23.42)	1.22 (0.05, 25.69)	1.42 (0.11, 14.50)	6.69 (0.40, 126.99)	9.60 (0.47, 461.93)	9.50 (0.48, 461.99)	**18.65 (1.05, 968.19)**	12.00 (0.62, 635.47)	7.52 (0.29, 540.32)	9.82 (0.47, 502.31)	1.18 (0.09, 12.24)
PBO	**5.65 (1.01, 34.91)**	2.42 (0.55, 9.86)	1.39 (0.28, 7.44)	2.13 (0.62, 8.27)	1.08 (0.08, 15.51)	1.24 (0.21, 6.35)	5.86 (0.78, 68.66)	8.30 (0.74, 264.81)	8.41 (0.81, 242.22)	**16.19 (1.52, 518.97)**	**10.49 (1.09, 376.51)**	6.41 (0.52, 240.00)	8.61 (0.69, 257.83)	1.02 (0.20, 5.56)
RIS1200mgIV	2.71 (0.23, 36.06)	1.20 (0.12, 9.21)	0.67 (0.06, 7.17)	1.03 (0.13, 9.57)	0.53 (0.02, 11.30)	0.59 (0.05, 6.17)	2.80 (0.20, 52.67)	4.07 (0.25, 192.80)	4.13 (0.24, 194.00)	7.93 (0.46, 397.88)	5.07 (0.31, 317.98)	3.24 (0.17, 215.07)	4.22 (0.21, 201.07)	0.50 (0.05, 6.42)
RIS600mgIV	2.64 (0.22, 34.11)	1.15 (0.10, 11.31)	0.64 (0.06, 7.40)	0.99 (0.11, 11.43)	0.50 (0.02, 10.90)	0.57 (0.05, 6.25)	2.73 (0.20, 59.13)	3.87 (0.22, 188.51)	3.78 (0.24, 214.79)	7.65 (0.44, 400.48)	4.88 (0.30, 288.76)	3.04 (0.16, 174.36)	3.96 (0.21, 190.14)	0.47 (0.05, 5.53)
VED0.5mg/kgIV	4.02 (0.37, 50.58)	1.73 (0.19, 15.26)	0.98 (0.09, 9.68)	1.52 (0.19, 14.00)	0.79 (0.03, 17.99)	0.87 (0.07, 9.74)	4.09 (0.29, 89.97)	5.90 (0.31, 283.75)	5.86 (0.33, 296.74)	12.07 (0.65, 597.66)	7.69 (0.46, 446.44)	4.70 (0.24, 275.00)	6.20 (0.30, 288.01)	0.73 (0.08, 8.27)
VED2mg/kgIV	3.11 (0.26, 35.40)	1.34 (0.12, 11.96)	0.75 (0.07, 8.85)	1.17 (0.13, 11.57)	0.60 (0.03, 12.45)	0.68 (0.06, 7.33)	3.24 (0.22, 57.80)	4.49 (0.22, 218.26)	4.56 (0.24, 224.42)	9.13 (0.46, 407.98)	5.85 (0.31, 361.51)	3.57 (0.17, 212.83)	4.58 (0.22, 232.03)	0.55 (0.05, 6.12)
VED300mgIV	3.69 (0.42, 35.91)	1.54 (0.22, 11.97)	0.86 (0.12, 7.00)	1.36 (0.23, 9.79)	0.71 (0.03, 13.43)	0.78 (0.10, 6.61)	3.81 (0.32, 59.21)	5.57 (0.37, 210.39)	5.45 (0.37, 205.90)	10.79 (0.72, 380.08)	7.01 (0.52, 336.32)	4.20 (0.24, 212.57)	5.61 (0.35, 226.23)	0.65 (0.08, 5.88)

	NAT3mg/kgIVx2	NAT6mg/kgIVx2	NNC2mg/kgSC	ONE10mgSC	ONE25mgSC	ONE34mgSC	ONE50mgSC	PBO	RIS1200mgIV	RIS600mgIV	VED0.5mg/kgIV	VED2mg/kgIV	VED300mgIV
ADA160mg80mg60mgSC	0.28 (0.02, 3.32)	0.26 (0.02, 2.85)	0.36 (0.02, 4.54)	0.23 (0.02, 2.52)	0.10 (0.01, 1.27)	0.23 (0.02, 2.47)	0.16 (0.01, 1.98)	**0.18 (0.03, 0.99)**	0.37 (0.03, 4.27)	0.38 (0.03, 4.47)	0.25 (0.02, 2.71)	0.32 (0.03, 3.87)	0.27 (0.03, 2.41)
ADA160mg80mgSC	0.63 (0.08, 5.62)	0.59 (0.06, 5.30)	0.86 (0.08, 9.65)	0.54 (0.06, 4.79)	0.24 (0.02, 2.32)	0.54 (0.06, 5.12)	0.36 (0.04, 4.23)	0.41 (0.10, 1.81)	0.83 (0.11, 8.21)	0.87 (0.09, 10.16)	0.58 (0.07, 5.29)	0.74 (0.08, 8.21)	0.65 (0.08, 4.47)
ADA40mg20mgSC	1.15 (0.10, 12.43)	1.04 (0.09, 10.24)	1.47 (0.11, 19.17)	0.94 (0.08, 8.57)	0.42 (0.03, 4.78)	0.96 (0.08, 10.15)	0.64 (0.06, 7.81)	0.72 (0.13, 3.52)	1.50 (0.14, 15.44)	1.56 (0.14, 17.24)	1.02 (0.10, 11.21)	1.34 (0.11, 15.12)	1.16 (0.14, 8.51)
ADA80mg40mgSC	0.73 (0.08, 5.93)	0.66 (0.07, 4.99)	0.94 (0.08, 9.05)	0.61 (0.07, 4.78)	0.28 (0.02, 2.45)	0.62 (0.07, 5.05)	0.41 (0.04, 4.29)	0.47 (0.12, 1.63)	0.97 (0.10, 7.84)	1.01 (0.09, 9.30)	0.66 (0.07, 5.33)	0.86 (0.09, 7.74)	0.74 (0.10, 4.31)
BRI400mgIV	1.42 (0.06, 30.63)	1.29 (0.05, 27.55)	1.88 (0.08, 51.75)	1.21 (0.06, 23.89)	0.52 (0.02, 12.97)	1.20 (0.05, 28.90)	0.82 (0.04, 20.55)	0.92 (0.06, 12.56)	1.90 (0.09, 46.71)	2.01 (0.09, 42.98)	1.26 (0.06, 30.64)	1.65 (0.08, 39.56)	1.40 (0.07, 29.89)
CER400mgSC	1.24 (0.11, 12.85)	1.16 (0.11, 13.07)	1.65 (0.13, 21.86)	1.04 (0.10, 11.14)	0.47 (0.04, 5.87)	1.05 (0.11, 12.95)	0.70 (0.07, 8.93)	0.81 (0.16, 4.75)	1.68 (0.16, 19.25)	1.75 (0.16, 20.59)	1.15 (0.10, 13.33)	1.47 (0.14, 17.70)	1.28 (0.15, 10.35)
FIL200mgPO	0.27 (0.01, 3.54)	0.24 (0.01, 3.11)	0.34 (0.01, 5.02)	0.22 (0.01, 2.67)	0.10 (0.00, 1.49)	0.22 (0.01, 2.96)	0.15 (0.01, 2.51)	0.17 (0.01, 1.28)	0.36 (0.02, 5.08)	0.37 (0.02, 5.06)	0.24 (0.01, 3.46)	0.31 (0.02, 4.47)	0.26 (0.02, 3.08)
INF10mg/kgIV	0.18 (0.00, 3.55)	0.17 (0.00, 3.39)	0.24 (0.00, 5.51)	0.15 (0.00, 2.86)	0.07 (0.00, 1.48)	0.15 (0.00, 2.89)	0.10 (0.00, 2.13)	0.12 (0.00, 1.36)	0.25 (0.01, 3.95)	0.26 (0.01, 4.46)	0.17 (0.00, 3.27)	0.22 (0.00, 4.62)	0.18 (0.00, 2.70)
INF20mg/kgIV	0.18 (0.00, 3.05)	0.17 (0.00, 3.05)	0.24 (0.00, 5.41)	0.15 (0.00, 2.55)	0.07 (0.00, 1.42)	0.16 (0.00, 3.16)	0.11 (0.00, 2.09)	0.12 (0.00, 1.23)	0.24 (0.01, 4.19)	0.26 (0.00, 4.21)	0.17 (0.00, 3.03)	0.22 (0.00, 4.21)	0.18 (0.00, 2.69)
INF5mg/kgIV	0.09 (0.00, 1.48)	0.08 (0.00, 1.30)	0.13 (0.00, 2.70)	0.08 (0.00, 1.41)	**0.04 (0.00, 0.75)**	0.08 (0.00, 1.45)	**0.05 (0.00, 0.96)**	**0.06 (0.00, 0.66)**	0.13 (0.00, 2.19)	0.13 (0.00, 2.30)	0.08 (0.00, 1.53)	0.11 (0.00, 2.17)	0.09 (0.00, 1.39)
MIR1000mgIV	0.14 (0.00, 2.36)	0.13 (0.00, 2.07)	0.18 (0.00, 4.39)	0.12 (0.00, 1.76)	0.05 (0.00, 1.00)	0.12 (0.00, 2.12)	0.08 (0.00, 1.61)	**0.10 (0.00, 0.91)**	0.20 (0.00, 3.21)	0.20 (0.00, 3.29)	0.13 (0.00, 2.18)	0.17 (0.00, 3.28)	0.14 (0.00, 1.92)
MIR200mgIV	0.24 (0.00, 4.57)	0.22 (0.00, 4.05)	0.30 (0.01, 8.08)	0.19 (0.00, 3.84)	0.09 (0.00, 2.10)	0.20 (0.00, 3.94)	0.13 (0.00, 3.42)	0.16 (0.00, 1.91)	0.31 (0.00, 6.02)	0.33 (0.01, 6.35)	0.21 (0.00, 4.25)	0.28 (0.00, 5.85)	0.24 (0.00, 4.09)
MIR600mgIV	0.18 (0.00, 3.78)	0.16 (0.00, 3.17)	0.24 (0.00, 6.30)	0.15 (0.00, 3.03)	0.07 (0.00, 1.46)	0.14 (0.00, 3.15)	0.10 (0.00, 2.15)	0.12 (0.00, 1.45)	0.24 (0.00, 4.78)	0.25 (0.01, 4.71)	0.16 (0.00, 3.30)	0.22 (0.00, 4.63)	0.18 (0.00, 2.87)
NAT3mg/kgIV	1.50 (0.28, 6.90)	1.39 (0.23, 6.81)	1.96 (0.15, 28.82)	1.26 (0.12, 12.13)	0.57 (0.05, 6.58)	1.26 (0.11, 13.96)	0.85 (0.08, 10.60)	0.98 (0.18, 4.96)	2.01 (0.16, 19.38)	2.11 (0.18, 21.26)	1.37 (0.12, 13.33)	1.81 (0.16, 19.00)	1.53 (0.17, 12.09)
NAT3mg/kgIVx2	NAT3mg/kgIVx2	0.92 (0.13, 5.37)	1.30 (0.11, 18.96)	0.83 (0.07, 9.51)	0.38 (0.03, 4.46)	0.83 (0.08, 10.12)	0.57 (0.05, 8.50)	0.64 (0.13, 3.68)	1.33 (0.14, 16.73)	1.38 (0.11, 18.30)	0.91 (0.08, 10.18)	1.18 (0.10, 14.42)	1.00 (0.11, 9.42)
NAT6mg/kgIVx2	1.09 (0.19, 7.60)	NAT6mg/kgIVx2	1.46 (0.11, 19.06)	0.92 (0.08, 10.06)	0.42 (0.03, 5.10)	0.91 (0.08, 10.87)	0.62 (0.06, 8.06)	0.69 (0.14, 4.07)	1.46 (0.15, 18.78)	1.51 (0.14, 17.46)	0.98 (0.08, 11.22)	1.30 (0.11, 15.35)	1.11 (0.13, 9.23)
NNC2mg/kgSC	0.77 (0.05, 8.78)	0.69 (0.05, 9.30)	NNC2mg/kgSC	0.62 (0.04, 9.15)	0.28 (0.02, 4.23)	0.62 (0.05, 8.20)	0.42 (0.03, 6.44)	0.49 (0.07, 3.29)	1.01 (0.08, 13.53)	1.05 (0.07, 15.11)	0.71 (0.05, 9.11)	0.92 (0.07, 12.81)	0.76 (0.06, 7.71)
ONE10mgSC	1.21 (0.11, 13.98)	1.09 (0.10, 13.04)	1.61 (0.11, 22.97)	ONE10mgSC	0.46 (0.07, 2.80)	1.01 (0.18, 6.14)	0.68 (0.13, 4.67)	0.78 (0.14, 4.51)	1.60 (0.14, 18.24)	1.67 (0.15, 17.22)	1.13 (0.10, 12.48)	1.39 (0.14, 16.96)	1.21 (0.14, 9.13)
ONE25mgSC	2.66 (0.22, 36.76)	2.39 (0.20, 31.67)	3.55 (0.24, 49.47)	2.16 (0.36, 14.14)	ONE25mgSC	2.18 (0.36, 15.32)	1.49 (0.23, 11.32)	1.68 (0.25, 12.15)	3.57 (0.25, 51.27)	3.67 (0.29, 46.67)	2.39 (0.17, 31.83)	3.10 (0.23, 41.07)	2.66 (0.28, 25.91)
ONE34mgSC	1.20 (0.10, 12.65)	1.10 (0.09, 12.30)	1.61 (0.12, 22.02)	0.99 (0.16, 5.66)	0.46 (0.07, 2.77)	ONE34mgSC	0.68 (0.11, 4.22)	0.75 (0.15, 4.39)	1.57 (0.14, 19.37)	1.66 (0.14, 18.68)	1.07 (0.10, 11.86)	1.39 (0.13, 16.00)	1.21 (0.14, 10.92)
ONE50mgSC	1.76 (0.12, 19.57)	1.61 (0.12, 17.55)	2.38 (0.16, 32.55)	1.48 (0.21, 7.79)	0.67 (0.09, 4.30)	1.47 (0.24, 9.13)	ONE50mgSC	1.15 (0.17, 7.11)	2.33 (0.16, 27.73)	2.48 (0.17, 26.85)	1.61 (0.12, 17.18)	2.04 (0.18, 26.80)	1.78 (0.15, 15.68)
PBO	1.56 (0.27, 7.63)	1.45 (0.25, 7.06)	2.02 (0.30, 14.92)	1.29 (0.22, 7.15)	0.60 (0.08, 3.93)	1.33 (0.23, 6.89)	0.87 (0.14, 5.95)	PBO	2.07 (0.35, 10.43)	2.15 (0.34, 11.56)	1.42 (0.23, 7.97)	1.82 (0.31, 11.98)	1.58 (0.38, 5.48)
RIS1200mgIV	0.75 (0.06, 7.20)	0.68 (0.05, 6.88)	0.99 (0.07, 12.68)	0.63 (0.05, 7.26)	0.28 (0.02, 3.95)	0.64 (0.05, 7.25)	0.43 (0.04, 6.42)	0.48 (0.10, 2.86)	RIS1200mgIV	1.04 (0.20, 5.34)	0.68 (0.06, 7.44)	0.89 (0.08, 10.45)	0.77 (0.09, 5.68)
RIS600mgIV	0.72 (0.05, 9.07)	0.66 (0.06, 7.25)	0.95 (0.07, 13.95)	0.60 (0.06, 6.58)	0.27 (0.02, 3.41)	0.60 (0.05, 7.00)	0.40 (0.04, 5.76)	0.47 (0.09, 2.96)	0.96 (0.19, 5.05)	RIS600mgIV	0.66 (0.06, 7.98)	0.84 (0.09, 11.57)	0.73 (0.08, 6.82)
VED0.5mg/kgIV	1.10 (0.10, 13.31)	1.02 (0.09, 12.18)	1.42 (0.11, 19.31)	0.89 (0.08, 10.43)	0.42 (0.03, 5.77)	0.93 (0.08, 10.09)	0.62 (0.06, 8.52)	0.70 (0.13, 4.41)	1.48 (0.13, 16.15)	1.51 (0.13, 17.31)	VED0.5mg/kgIV	1.30 (0.26, 7.38)	1.13 (0.10, 9.98)
VED2mg/kgIV	0.85 (0.07, 9.66)	0.77 (0.07, 9.40)	1.09 (0.08, 13.41)	0.72 (0.06, 7.34)	0.32 (0.02, 4.34)	0.72 (0.06, 7.54)	0.49 (0.04, 5.42)	0.55 (0.08, 3.21)	1.12 (0.10, 12.55)	1.19 (0.09, 11.09)	0.77 (0.14, 3.90)	VED2mg/kgIV	0.87 (0.08, 7.99)
VED300mgIV	1.00 (0.11, 9.37)	0.90 (0.11, 7.50)	1.31 (0.13, 16.48)	0.83 (0.11, 7.23)	0.38 (0.04, 3.62)	0.83 (0.09, 7.35)	0.56 (0.06, 6.46)	0.63 (0.18, 2.65)	1.30 (0.18, 11.66)	1.36 (0.15, 12.71)	0.88 (0.10, 10.05)	1.15 (0.13, 12.53)	VED300mgIV

Table S12D: League table for clinical remission for FDA-approved drugs only.

	ADA160mg80mgSC	ADA80mg40mgSC	BRI400mgIV	BRI700mgIV	FIL200mgPO	GUS1200mgIV	GUS200mgIV	GUS600mgIV	MED700IV	MIR1000mgIV	MIR200mgIV	MIR600mgIV	PBO	PF10mgSC
ADA160mg80mgSC	ADA160mg80mgSC	2.18 (0.58, 11.05)	0.82 (0.14, 5.72)	1.07 (0.23, 6.26)	0.47 (0.19, 1.37)	1.45 (0.36, 8.20)	2.41 (0.64, 14.48)	2.01 (0.50, 11.73)	0.54 (0.17, 1.72)	0.53 (0.15, 1.89)	0.24 (0.03, 1.22)	1.21 (0.39, 4.22)	**0.35 (0.17, 0.72)**	0.31 (0.08, 1.34)
ADA80mg40mgSC	0.46 (0.09, 1.74)	ADA80mg40mgSC	0.36 (0.04, 4.02)	0.49 (0.05, 4.99)	0.22 (0.04, 1.19)	0.65 (0.08, 5.36)	1.12 (0.15, 9.11)	0.89 (0.12, 7.45)	0.24 (0.04, 1.44)	0.24 (0.03, 1.48)	**0.10 (0.01, 0.90)**	0.55 (0.08, 3.74)	**0.16 (0.03, 0.73)**	0.14 (0.02, 1.00)
BRI400mgIV	1.23 (0.17, 7.24)	2.81 (0.25, 25.97)	BRI400mgIV	1.29 (0.48, 4.57)	0.59 (0.08, 3.82)	1.86 (0.21, 15.90)	3.07 (0.35, 28.46)	2.56 (0.28, 23.72)	0.66 (0.09, 4.47)	0.62 (0.08, 5.24)	0.28 (0.02, 2.92)	1.49 (0.19, 11.38)	0.44 (0.07, 2.24)	0.37 (0.04, 3.13)
BRI700mgIV	0.93 (0.16, 4.41)	2.05 (0.20, 19.29)	0.77 (0.22, 2.09)	BRI700mgIV	0.46 (0.07, 2.22)	1.42 (0.17, 10.29)	2.37 (0.31, 16.93)	1.89 (0.23, 13.93)	0.50 (0.08, 2.58)	0.48 (0.07, 3.09)	0.21 (0.01, 1.95)	1.11 (0.17, 6.94)	0.34 (0.06, 1.29)	0.30 (0.04, 1.93)
FIL200mgPO	2.14 (0.73, 5.40)	4.60 (0.84, 28.02)	1.69 (0.26, 12.57)	2.19 (0.45, 15.20)	FIL200mgPO	3.03 (0.73, 16.96)	**5.07 (1.28, 29.02)**	**4.09 (1.04, 22.60)**	1.14 (0.33, 3.60)	1.11 (0.29, 4.07)	0.48 (0.06, 2.68)	2.53 (0.71, 8.73)	0.75 (0.32, 1.49)	0.65 (0.14, 2.80)
GUS1200mgIV	0.69 (0.12, 2.78)	1.53 (0.19, 12.24)	0.54 (0.06, 4.86)	0.70 (0.10, 5.87)	0.33 (0.06, 1.37)	GUS1200mgIV	1.67 (0.87, 3.26)	1.36 (0.69, 2.91)	0.37 (0.06, 1.66)	0.36 (0.06, 1.94)	0.15 (0.01, 1.22)	0.83 (0.14, 4.03)	**0.25 (0.05, 0.78)**	0.21 (0.03, 1.17)
GUS200mgIV	0.41 (0.07, 1.57)	0.89 (0.11, 6.87)	0.33 (0.04, 2.87)	0.42 (0.06, 3.25)	**0.20 (0.03, 0.78)**	0.60 (0.31, 1.15)	GUS200mgIV	0.81 (0.45, 1.48)	0.22 (0.04, 1.00)	0.22 (0.03, 1.01)	**0.09 (0.01, 0.68)**	0.50 (0.08, 2.32)	**0.15 (0.03, 0.43)**	**0.13 (0.02, 0.68)**
GUS600mgIV	0.50 (0.09, 1.99)	1.12 (0.13, 8.34)	0.39 (0.04, 3.52)	0.53 (0.07, 4.31)	**0.24 (0.04, 0.96)**	0.73 (0.34, 1.44)	1.23 (0.67, 2.25)	GUS600mgIV	0.28 (0.05, 1.20)	0.26 (0.04, 1.28)	**0.11 (0.01, 0.88)**	0.61 (0.10, 2.77)	**0.18 (0.04, 0.54)**	**0.16 (0.02, 0.84)**
MED700IV	1.85 (0.58, 5.79)	4.18 (0.69, 27.89)	1.51 (0.22, 11.63)	1.99 (0.39, 12.20)	0.88 (0.28, 2.99)	2.67 (0.60, 15.98)	4.47 (1.00, 27.74)	3.60 (0.84, 21.78)	MED700IV	0.97 (0.26, 4.20)	0.42 (0.05, 2.53)	2.22 (0.59, 8.69)	0.65 (0.26, 1.58)	0.58 (0.12, 2.74)
MIR1000mgIV	1.88 (0.53, 6.75)	4.19 (0.68, 29.28)	1.62 (0.19, 12.52)	2.08 (0.32, 15.09)	0.90 (0.25, 3.44)	2.78 (0.52, 16.75)	4.63 (0.99, 29.00)	3.79 (0.78, 24.60)	1.03 (0.24, 3.89)	MIR1000mgIV	0.44 (0.07, 1.97)	2.25 (0.96, 5.85)	0.67 (0.23, 1.90)	0.61 (0.11, 2.70)
MIR200mgIV	4.21 (0.82, 35.35)	**9.85 (1.11, 114.70)**	3.56 (0.34, 57.24)	4.73 (0.51, 75.10)	2.09 (0.37, 16.80)	6.49 (0.82, 82.76)	**10.70 (1.47, 134.89)**	**8.93 (1.14, 104.50)**	2.36 (0.39, 20.22)	2.30 (0.51, 15.26)	MIR200mgIV	**5.13 (1.39, 33.95)**	1.51 (0.33, 11.20)	1.35 (0.19, 13.31)
MIR600mgIV	0.83 (0.24, 2.58)	1.83 (0.27, 11.83)	0.67 (0.09, 5.28)	0.90 (0.14, 5.85)	0.40 (0.11, 1.41)	1.20 (0.25, 7.18)	1.99 (0.43, 12.26)	1.65 (0.36, 9.85)	0.45 (0.12, 1.68)	0.44 (0.17, 1.04)	**0.19 (0.03, 0.72)**	MIR600mgIV	**0.29 (0.10, 0.73)**	0.26 (0.05, 1.17)
PBO	**2.82 (1.38, 5.82)**	**6.30 (1.37, 34.19)**	2.27 (0.45, 14.32)	2.95 (0.78, 16.57)	1.34 (0.67, 3.17)	**4.08 (1.27, 18.52)**	**6.79 (2.34, 33.34)**	**5.49 (1.87, 26.88)**	1.53 (0.63, 3.91)	1.49 (0.53, 4.43)	0.66 (0.09, 3.01)	**3.43 (1.37, 9.68)**	PBO	0.90 (0.26, 3.02)
PF10mgSC	3.20 (0.75, 13.26)	7.09 (1.00, 53.61)	2.67 (0.32, 22.48)	3.33 (0.52, 27.59)	1.53 (0.36, 7.06)	4.83 (0.85, 32.91)	**7.89 (1.46, 59.16)**	**6.37 (1.19, 44.50)**	1.71 (0.37, 8.51)	1.64 (0.37, 9.17)	0.74 (0.08, 5.14)	3.82 (0.85, 19.09)	1.11 (0.33, 3.82)	PF10mgSC
PF200mgSC	3.09 (0.65, 17.41)	6.92 (0.93, 61.02)	2.56 (0.30, 26.79)	3.34 (0.47, 35.26)	1.47 (0.31, 9.11)	4.62 (0.73, 39.65)	**7.78 (1.37, 69.17)**	**6.34 (1.09, 55.50)**	1.64 (0.34, 10.28)	1.66 (0.32, 10.18)	0.74 (0.08, 5.48)	3.85 (0.71, 24.09)	1.09 (0.28, 5.68)	0.98 (0.24, 4.90)
PF50mgSC	1.05 (0.28, 3.32)	2.29 (0.39, 14.97)	0.84 (0.11, 5.95)	1.11 (0.19, 7.20)	0.50 (0.13, 1.71)	1.52 (0.30, 8.61)	2.53 (0.56, 14.32)	2.06 (0.45, 11.77)	0.56 (0.14, 2.14)	0.54 (0.13, 2.34)	0.24 (0.03, 1.32)	1.25 (0.31, 4.88)	**0.37 (0.13, 0.88)**	**0.33 (0.10, 0.84)**
RIS1200mgIV	1.55 (0.70, 3.55)	3.47 (0.72, 19.75)	1.27 (0.23, 8.28)	1.61 (0.41, 9.34)	0.74 (0.34, 1.87)	2.27 (0.70, 10.93)	**3.81 (1.24, 18.07)**	3.06 (0.99, 15.42)	0.85 (0.32, 2.32)	0.84 (0.27, 2.57)	0.36 (0.05, 1.72)	1.87 (0.70, 5.81)	**0.55 (0.38, 0.79)**	0.50 (0.13, 1.80)
RIS200mgIV	2.09 (0.72, 6.21)	4.61 (0.92, 28.72)	1.70 (0.29, 12.71)	2.19 (0.46, 13.69)	0.98 (0.35, 3.18)	3.06 (0.76, 16.85)	**5.09 (1.36, 28.56)**	**4.22 (1.13, 23.23)**	1.12 (0.36, 4.00)	1.10 (0.31, 4.24)	0.49 (0.06, 2.66)	2.48 (0.74, 9.71)	0.73 (0.34, 1.67)	0.68 (0.15, 2.84)
RIS600mgIV	1.43 (0.65, 3.24)	3.17 (0.69, 18.32)	1.16 (0.22, 7.34)	1.47 (0.38, 8.37)	0.67 (0.32, 1.72)	2.09 (0.62, 9.90)	**3.46 (1.12, 17.05)**	2.79 (0.88, 13.83)	0.78 (0.29, 2.13)	0.77 (0.25, 2.24)	0.33 (0.04, 1.56)	1.73 (0.63, 5.16)	**0.50 (0.36, 0.70)**	0.45 (0.12, 1.62)
TES400mg200mgSC	1.40 (0.51, 3.78)	3.18 (0.57, 19.68)	1.08 (0.18, 7.99)	1.47 (0.32, 8.97)	0.66 (0.24, 1.94)	2.03 (0.53, 11.24)	3.43 (0.91, 18.59)	2.75 (0.78, 15.00)	0.75 (0.24, 2.48)	0.75 (0.22, 2.59)	0.32 (0.04, 1.74)	1.68 (0.52, 5.75)	**0.50 (0.24, 0.97)**	0.43 (0.11, 1.86)
UPA45mgPO	1.61 (0.73, 3.52)	3.54 (0.75, 19.75)	1.30 (0.25, 8.07)	1.69 (0.42, 9.72)	0.76 (0.36, 1.90)	2.30 (0.70, 10.74)	**3.89 (1.25, 18.75)**	**3.15 (1.02, 15.28)**	0.88 (0.32, 2.42)	0.86 (0.28, 2.59)	0.38 (0.05, 1.77)	1.94 (0.72, 5.63)	**0.57 (0.41, 0.77)**	0.52 (0.14, 1.80)
UST130mgIV	1.48 (0.61, 3.76)	3.38 (0.68, 19.66)	1.22 (0.22, 8.53)	1.57 (0.36, 9.48)	0.71 (0.29, 1.93)	2.15 (0.59, 11.01)	**3.62 (1.05, 18.38)**	2.97 (0.85, 15.84)	0.81 (0.27, 2.40)	0.78 (0.23, 2.68)	0.35 (0.04, 1.80)	1.80 (0.58, 6.07)	**0.53 (0.30, 0.93)**	0.48 (0.13, 1.82)
UST1mg/kgIV	1.38 (0.55, 3.59)	3.11 (0.63, 18.79)	1.12 (0.20, 7.79)	1.46 (0.33, 8.85)	0.66 (0.26, 1.95)	2.02 (0.52, 10.64)	3.40 (0.94, 18.74)	2.80 (0.76, 15.27)	0.76 (0.24, 2.34)	0.74 (0.21, 2.66)	0.32 (0.04, 1.72)	1.69 (0.52, 5.66)	**0.49 (0.26, 0.97)**	0.44 (0.11, 1.89)
UST3mg/kgIV	1.34 (0.51, 3.45)	2.99 (0.59, 17.47)	1.08 (0.18, 7.90)	1.39 (0.33, 8.97)	0.65 (0.25, 1.87)	1.96 (0.51, 9.81)	3.28 (0.92, 16.61)	2.68 (0.75, 13.77)	0.73 (0.23, 2.29)	0.71 (0.21, 2.47)	0.31 (0.04, 1.55)	1.64 (0.53, 5.42)	**0.47 (0.25, 0.89)**	0.43 (0.11, 1.80)
UST4.5mg/kgIV	**4.38 (1.04, 20.34)**	**9.75 (1.38, 74.02)**	3.54 (0.50, 31.73)	4.79 (0.80, 38.32)	2.10 (0.51, 10.46)	**6.53 (1.20, 46.09)**	**11.01 (1.96, 80.31)**	**8.95 (1.57, 66.85)**	2.36 (0.54, 12.25)	2.33 (0.47, 12.87)	1.01 (0.11, 7.75)	**5.37 (1.11, 31.24)**	1.56 (0.47, 5.76)	1.41 (0.24, 8.70)
UST6mg/kg90mgIV/SC	0.60 (0.10, 2.44)	1.34 (0.16, 10.66)	0.48 (0.05, 4.39)	0.62 (0.09, 5.14)	0.29 (0.05, 1.22)	0.88 (0.43, 1.74)	1.46 (0.81, 2.85)	1.20 (0.62, 2.31)	0.33 (0.06, 1.44)	0.31 (0.05, 1.57)	**0.13 (0.01, 0.98)**	0.72 (0.12, 3.43)	**0.21 (0.05, 0.67)**	0.18 (0.03, 1.01)
UST6mg/kgIV	1.20 (0.51, 2.70)	2.66 (0.56, 15.49)	0.96 (0.18, 6.71)	1.25 (0.30, 7.48)	0.56 (0.25, 1.59)	1.74 (0.50, 8.73)	2.88 (0.89, 15.28)	2.36 (0.70, 12.55)	0.65 (0.23, 1.88)	0.63 (0.20, 2.17)	0.28 (0.04, 1.44)	1.43 (0.50, 4.84)	**0.42 (0.27, 0.66)**	0.38 (0.10, 1.37)
UST90mgSC	2.47 (0.64, 10.52)	5.31 (0.78, 46.90)	1.99 (0.23, 17.48)	2.64 (0.40, 21.28)	1.18 (0.30, 4.89)	3.48 (0.65, 28.16)	**6.05 (1.17, 48.33)**	4.88 (0.96, 40.47)	1.36 (0.30, 6.14)	1.30 (0.27, 6.25)	0.56 (0.05, 4.00)	3.00 (0.63, 14.13)	0.87 (0.28, 2.97)	0.77 (0.15, 4.24)
VED300mgIV	1.16 (0.45, 2.84)	2.54 (0.52, 14.59)	0.94 (0.16, 5.86)	1.21 (0.28, 6.96)	0.55 (0.22, 1.54)	1.64 (0.45, 8.20)	2.74 (0.80, 14.80)	2.25 (0.65, 11.52)	0.63 (0.22, 1.81)	0.60 (0.19, 2.07)	0.27 (0.03, 1.31)	1.40 (0.45, 4.50)	**0.41 (0.23, 0.69)**	0.36 (0.09, 1.37)

	PF200mgSC	PF50mgSC	RIS1200mgIV	RIS200mgIV	RIS600mgIV	TES400mg200mgSC	UPA45mgPO	UST130mgIV	UST1mg/kgIV	UST3mg/kgIV	UST4.5mg/kgIV	UST6mg/kg90mgIV/SC	UST6mg/kgIV	UST90mgSC	VED300mgIV
ADA160mg80mgSC	0.32 (0.06, 1.53)	0.95 (0.30, 3.57)	0.64 (0.28, 1.43)	0.48 (0.16, 1.39)	0.70 (0.31, 1.55)	0.72 (0.26, 1.97)	0.62 (0.28, 1.37)	0.68 (0.27, 1.64)	0.73 (0.28, 1.83)	0.74 (0.29, 1.97)	**0.23 (0.05, 0.96)**	1.68 (0.41, 9.81)	0.84 (0.37, 1.95)	0.41 (0.10, 1.56)	0.86 (0.35, 2.23)
ADA80mg40mgSC	0.14 (0.02, 1.08)	0.44 (0.07, 2.57)	0.29 (0.05, 1.38)	0.22 (0.03, 1.08)	0.32 (0.05, 1.46)	0.31 (0.05, 1.74)	0.28 (0.05, 1.33)	0.30 (0.05, 1.48)	0.32 (0.05, 1.58)	0.33 (0.06, 1.69)	**0.10 (0.01, 0.72)**	0.75 (0.09, 6.44)	0.38 (0.06, 1.78)	0.19 (0.02, 1.29)	0.39 (0.07, 1.93)
BRI400mgIV	0.39 (0.04, 3.32)	1.19 (0.17, 9.06)	0.79 (0.12, 4.40)	0.59 (0.08, 3.48)	0.86 (0.14, 4.64)	0.92 (0.13, 5.46)	0.77 (0.12, 4.00)	0.82 (0.12, 4.47)	0.90 (0.13, 5.08)	0.92 (0.13, 5.67)	0.28 (0.03, 2.00)	2.10 (0.23, 20.19)	1.05 (0.15, 5.63)	0.50 (0.06, 4.44)	1.07 (0.17, 6.16)
BRI700mgIV	0.30 (0.03, 2.13)	0.90 (0.14, 5.21)	0.62 (0.11, 2.46)	0.46 (0.07, 2.19)	0.68 (0.12, 2.66)	0.68 (0.11, 3.10)	0.59 (0.10, 2.38)	0.64 (0.11, 2.77)	0.68 (0.11, 3.06)	0.72 (0.11, 3.07)	0.21 (0.03, 1.25)	1.61 (0.19, 11.43)	0.80 (0.13, 3.28)	0.38 (0.05, 2.53)	0.83 (0.14, 3.60)
FIL200mgPO	0.68 (0.11, 3.27)	2.02 (0.58, 7.67)	1.35 (0.54, 2.94)	1.02 (0.31, 2.87)	1.49 (0.58, 3.17)	1.52 (0.51, 4.10)	1.32 (0.53, 2.81)	1.41 (0.52, 3.47)	1.51 (0.51, 3.79)	1.54 (0.54, 4.07)	0.48 (0.10, 1.96)	3.48 (0.82, 18.59)	1.78 (0.63, 3.98)	0.85 (0.20, 3.28)	1.82 (0.65, 4.61)
GUS1200mgIV	0.22 (0.03, 1.37)	0.66 (0.12, 3.32)	0.44 (0.09, 1.42)	0.33 (0.06, 1.31)	0.48 (0.10, 1.61)	0.49 (0.09, 1.88)	0.44 (0.09, 1.42)	0.46 (0.09, 1.71)	0.50 (0.09, 1.92)	0.51 (0.10, 1.96)	**0.15 (0.02, 0.83)**	1.14 (0.57, 2.35)	0.57 (0.11, 2.00)	0.29 (0.04, 1.54)	0.61 (0.12, 2.21)
GUS200mgIV	**0.13 (0.01, 0.73)**	0.40 (0.07, 1.79)	**0.26 (0.06, 0.81)**	**0.20 (0.04, 0.74)**	**0.29 (0.06, 0.89)**	0.29 (0.05, 1.10)	**0.26 (0.05, 0.80)**	**0.28 (0.05, 0.96)**	0.29 (0.05, 1.07)	0.30 (0.06, 1.09)	**0.09 (0.01, 0.51)**	0.69 (0.35, 1.23)	0.35 (0.07, 1.12)	**0.17 (0.02, 0.86)**	0.36 (0.07, 1.25)
GUS600mgIV	**0.16 (0.02, 0.91)**	0.49 (0.08, 2.21)	0.33 (0.06, 1.01)	**0.24 (0.04, 0.89)**	0.36 (0.07, 1.13)	0.36 (0.07, 1.28)	**0.32 (0.07, 0.98)**	0.34 (0.06, 1.18)	0.36 (0.07, 1.31)	0.37 (0.07, 1.34)	**0.11 (0.01, 0.64)**	0.83 (0.43, 1.61)	0.42 (0.08, 1.43)	0.20 (0.02, 1.04)	0.44 (0.09, 1.53)
MED700IV	0.61 (0.10, 2.91)	1.79 (0.47, 7.36)	1.18 (0.43, 3.11)	0.89 (0.25, 2.78)	1.28 (0.47, 3.41)	1.33 (0.40, 4.19)	1.14 (0.41, 3.12)	1.23 (0.42, 3.69)	1.31 (0.43, 4.11)	1.36 (0.44, 4.26)	0.42 (0.08, 1.86)	3.06 (0.69, 17.99)	1.53 (0.53, 4.31)	0.74 (0.16, 3.38)	1.59 (0.55, 4.57)
MIR1000mgIV	0.60 (0.10, 3.13)	1.85 (0.43, 7.62)	1.19 (0.39, 3.73)	0.91 (0.24, 3.25)	1.31 (0.45, 4.00)	1.34 (0.39, 4.62)	1.17 (0.39, 3.57)	1.27 (0.37, 4.30)	1.35 (0.38, 4.77)	1.41 (0.40, 4.84)	0.43 (0.08, 2.13)	3.18 (0.64, 19.42)	1.59 (0.46, 4.95)	0.77 (0.16, 3.70)	1.65 (0.48, 5.39)
MIR200mgIV	1.35 (0.18, 12.88)	4.13 (0.76, 34.52)	2.75 (0.58, 21.64)	2.03 (0.38, 16.93)	3.00 (0.64, 22.82)	3.09 (0.57, 25.03)	2.66 (0.57, 19.54)	2.85 (0.56, 23.13)	3.10 (0.58, 26.28)	3.28 (0.64, 25.30)	0.99 (0.13, 9.39)	**7.44 (1.02, 90.91)**	3.58 (0.70, 28.21)	1.79 (0.25, 18.71)	3.66 (0.76, 31.08)
MIR600mgIV	0.26 (0.04, 1.42)	0.80 (0.21, 3.22)	0.53 (0.17, 1.43)	0.40 (0.10, 1.35)	0.58 (0.19, 1.58)	0.59 (0.17, 1.92)	0.51 (0.18, 1.38)	0.56 (0.16, 1.73)	0.59 (0.18, 1.93)	0.61 (0.18, 1.89)	**0.19 (0.03, 0.90)**	1.40 (0.29, 8.10)	0.70 (0.21, 1.99)	0.33 (0.07, 1.58)	0.72 (0.22, 2.22)
PBO	0.92 (0.18, 3.62)	**2.69 (1.13, 7.83)**	**1.82 (1.27, 2.64)**	1.36 (0.60, 2.90)	**1.99 (1.44, 2.77)**	**2.01 (1.04, 4.11)**	**1.76 (1.30, 2.46)**	**1.89 (1.07, 3.38)**	**2.05 (1.03, 3.87)**	**2.12 (1.13, 3.93)**	0.64 (0.17, 2.14)	**4.69 (1.48, 21.62)**	**2.36 (1.51, 3.76)**	1.16 (0.34, 3.63)	**2.44 (1.45, 4.38)**
PF10mgSC	1.02 (0.20, 4.17)	**3.02 (1.18, 10.11)**	2.02 (0.56, 7.42)	1.48 (0.35, 6.68)	2.20 (0.62, 8.15)	2.31 (0.54, 9.31)	1.93 (0.56, 7.00)	2.08 (0.55, 7.99)	2.26 (0.53, 9.10)	2.33 (0.56, 9.28)	0.71 (0.11, 4.22)	5.48 (0.99, 38.38)	2.61 (0.73, 9.94)	1.30 (0.24, 6.74)	2.74 (0.73, 10.88)
PF200mgSC	PF200mgSC	2.93 (0.93, 13.73)	2.00 (0.50, 10.80)	1.44 (0.32, 9.09)	2.17 (0.55, 11.58)	2.21 (0.47, 13.36)	1.93 (0.49, 10.24)	2.08 (0.47, 11.56)	2.21 (0.48, 12.67)	2.30 (0.50, 13.41)	0.70 (0.11, 5.68)	5.29 (0.80, 44.09)	2.57 (0.63, 14.17)	1.27 (0.21, 9.23)	2.66 (0.62, 15.10)
PF50mgSC	0.34 (0.07, 1.07)	PF50mgSC	0.67 (0.22, 1.72)	0.50 (0.12, 1.57)	0.74 (0.25, 1.85)	0.75 (0.20, 2.33)	0.65 (0.21, 1.65)	0.69 (0.21, 2.04)	0.75 (0.20, 2.24)	0.78 (0.22, 2.28)	0.23 (0.04, 1.07)	1.74 (0.35, 9.58)	0.87 (0.26, 2.43)	0.43 (0.08, 1.81)	0.91 (0.28, 2.66)
RIS1200mgIV	0.50 (0.09, 2.02)	1.48 (0.58, 4.61)	RIS1200mgIV	0.75 (0.32, 1.59)	1.09 (0.83, 1.49)	1.11 (0.50, 2.44)	0.96 (0.62, 1.57)	1.04 (0.54, 2.11)	1.13 (0.52, 2.35)	1.16 (0.57, 2.42)	0.36 (0.09, 1.23)	2.61 (0.78, 12.69)	1.30 (0.72, 2.36)	0.64 (0.18, 2.12)	1.34 (0.73, 2.71)
RIS200mgIV	0.69 (0.11, 3.15)	2.01 (0.64, 8.36)	1.33 (0.63, 3.11)	RIS200mgIV	1.45 (0.73, 3.25)	1.50 (0.54, 4.39)	1.29 (0.58, 3.09)	1.39 (0.54, 3.86)	1.49 (0.55, 4.19)	1.55 (0.59, 4.26)	0.48 (0.11, 1.95)	3.45 (0.90, 20.23)	1.74 (0.74, 4.40)	0.85 (0.22, 3.32)	1.79 (0.74, 4.86)
RIS600mgIV	0.46 (0.09, 1.83)	1.35 (0.54, 4.06)	0.92 (0.67, 1.20)	0.69 (0.31, 1.38)	RIS600mgIV	1.03 (0.46, 2.22)	0.89 (0.56, 1.40)	0.96 (0.51, 1.81)	1.03 (0.49, 2.08)	1.06 (0.52, 2.13)	0.33 (0.08, 1.12)	2.39 (0.70, 11.43)	1.19 (0.68, 2.09)	0.59 (0.16, 1.90)	1.23 (0.69, 2.39)
TES400mg200mgSC	0.45 (0.07, 2.11)	1.34 (0.43, 4.89)	0.90 (0.41, 2.00)	0.67 (0.23, 1.87)	0.97 (0.45, 2.15)	TES400mg200mgSC	0.87 (0.40, 1.86)	0.94 (0.39, 2.29)	1.00 (0.36, 2.59)	1.04 (0.40, 2.61)	0.31 (0.07, 1.32)	2.37 (0.60, 12.29)	1.18 (0.50, 2.72)	0.57 (0.14, 2.30)	1.22 (0.50, 2.95)
UPA45mgPO	0.52 (0.10, 2.06)	1.54 (0.61, 4.78)	1.04 (0.63, 1.62)	0.78 (0.32, 1.73)	1.12 (0.72, 1.78)	1.15 (0.54, 2.53)	UPA45mgPO	1.08 (0.55, 2.14)	1.17 (0.55, 2.34)	1.19 (0.59, 2.41)	0.37 (0.09, 1.29)	2.68 (0.79, 13.04)	1.35 (0.75, 2.34)	0.66 (0.19, 2.18)	1.38 (0.75, 2.67)
UST130mgIV	0.48 (0.09, 2.13)	1.45 (0.49, 4.71)	0.96 (0.47, 1.84)	0.72 (0.26, 1.84)	1.04 (0.55, 1.96)	1.06 (0.44, 2.56)	0.93 (0.47, 1.81)	UST130mgIV	1.09 (0.49, 2.22)	1.13 (0.51, 2.29)	0.34 (0.08, 1.36)	2.47 (0.66, 12.43)	1.25 (0.73, 2.03)	0.61 (0.15, 2.28)	1.29 (0.60, 2.86)
UST1mg/kgIV	0.45 (0.08, 2.09)	1.33 (0.45, 4.97)	0.89 (0.43, 1.93)	0.67 (0.24, 1.83)	0.97 (0.48, 2.04)	1.00 (0.39, 2.76)	0.86 (0.43, 1.83)	0.92 (0.45, 2.03)	UST1mg/kgIV	1.04 (0.54, 1.96)	0.32 (0.07, 1.24)	2.34 (0.61, 11.95)	1.17 (0.66, 2.17)	0.57 (0.14, 2.11)	1.21 (0.52, 2.93)
UST3mg/kgIV	0.44 (0.07, 2.00)	1.29 (0.44, 4.64)	0.86 (0.41, 1.77)	0.65 (0.23, 1.69)	0.94 (0.47, 1.94)	0.96 (0.38, 2.48)	0.84 (0.42, 1.69)	0.89 (0.44, 1.97)	0.97 (0.51, 1.84)	UST3mg/kgIV	0.31 (0.07, 1.19)	2.25 (0.59, 11.29)	1.13 (0.63, 1.99)	0.55 (0.14, 2.06)	1.16 (0.52, 2.82)
UST4.5mg/kgIV	1.42 (0.18, 8.82)	4.31 (0.93, 22.91)	2.80 (0.81, 11.11)	2.08 (0.51, 9.42)	3.06 (0.89, 12.00)	3.21 (0.76, 14.05)	2.73 (0.78, 10.97)	2.95 (0.74, 12.57)	3.13 (0.80, 14.17)	3.26 (0.84, 13.87)	UST4.5mg/kgIV	**7.30 (1.25, 52.09)**	3.64 (0.96, 14.92)	1.79 (0.35, 10.97)	3.82 (0.96, 15.86)
UST6mg/kg90mgIV/SC	0.19 (0.02, 1.24)	0.58 (0.10, 2.89)	0.38 (0.08, 1.28)	0.29 (0.05, 1.12)	0.42 (0.09, 1.43)	0.42 (0.08, 1.67)	0.37 (0.08, 1.27)	0.40 (0.08, 1.52)	0.43 (0.08, 1.65)	0.44 (0.09, 1.69)	**0.14 (0.02, 0.80)**	UST6mg/kg90mgIV/SC	0.50 (0.10, 1.80)	0.24 (0.03, 1.29)	0.53 (0.11, 1.92)
UST6mg/kgIV	0.39 (0.07, 1.58)	1.14 (0.41, 3.87)	0.77 (0.42, 1.38)	0.58 (0.23, 1.36)	0.84 (0.48, 1.48)	0.85 (0.37, 1.99)	0.74 (0.43, 1.33)	0.80 (0.49, 1.37)	0.86 (0.46, 1.51)	0.89 (0.50, 1.58)	0.27 (0.07, 1.04)	2.00 (0.56, 9.98)	UST6mg/kgIV	0.49 (0.14, 1.73)	1.03 (0.53, 2.18)
UST90mgSC	0.79 (0.11, 4.71)	2.34 (0.55, 12.17)	1.55 (0.47, 5.52)	1.17 (0.30, 4.62)	1.69 (0.53, 6.11)	1.76 (0.44, 7.22)	1.51 (0.46, 5.23)	1.65 (0.44, 6.47)	1.76 (0.47, 7.03)	1.82 (0.49, 7.20)	0.56 (0.09, 2.89)	4.19 (0.78, 34.09)	2.04 (0.58, 7.39)	UST90mgSC	2.10 (0.61, 7.99)
VED300mgIV	0.38 (0.07, 1.60)	1.10 (0.38, 3.56)	0.74 (0.37, 1.36)	0.56 (0.21, 1.35)	0.81 (0.42, 1.46)	0.82 (0.34, 1.99)	0.72 (0.37, 1.34)	0.78 (0.35, 1.66)	0.83 (0.34, 1.92)	0.86 (0.35, 1.93)	0.26 (0.06, 1.04)	1.89 (0.52, 9.44)	0.97 (0.46, 1.89)	0.48 (0.13, 1.65)	VED300mgIV

Table S12E: League table for clinical response with reductions of 100 points.

	ADA160mg80mgSC	ADA80mg40mgSC	BRI400mgIV	BRI700mgIV	FIL200mgPO	GUS1200mgIV	GUS200mgIV	GUS600mgIV	MED700IV	MIR1000mgIV	MIR200mgIV	MIR600mgIV	PBO	PF10mgSC	PF200mgSC	PF50mgSC
ADA160mg80mgSC	ADA160mg80mgSC	2.25 (0.60, 10.97)	0.77 (0.13, 6.02)	1.04 (0.21, 10.24)	0.47 (0.17, 1.45)	1.37 (0.37, 6.72)	2.31 (0.66, 11.26)	1.89 (0.52, 8.99)	0.54 (0.18, 1.72)	0.53 (0.15, 1.95)	0.23 (0.03, 1.32)	1.19 (0.35, 4.45)	**0.36 (0.17, 0.69)**	0.31 (0.07, 1.34)	0.32 (0.05, 1.61)	0.97 (0.30, 3.33)
ADA80mg40mgSC	0.45 (0.09, 1.67)	ADA80mg40mgSC	0.34 (0.03, 3.67)	0.47 (0.05, 4.91)	0.21 (0.03, 1.05)	0.63 (0.08, 4.35)	1.06 (0.13, 7.07)	0.86 (0.10, 5.95)	0.24 (0.04, 1.32)	0.23 (0.03, 1.42)	**0.10 (0.01, 0.79)**	0.54 (0.07, 2.97)	**0.15 (0.03, 0.66)**	**0.14 (0.02, 0.94)**	0.14 (0.01, 1.01)	0.43 (0.06, 2.46)
BRI400mgIV	1.29 (0.17, 7.65)	2.92 (0.27, 29.71)	BRI400mgIV	1.33 (0.45, 4.63)	0.61 (0.07, 3.87)	1.81 (0.18, 16.12)	3.05 (0.31, 25.97)	2.49 (0.25, 21.30)	0.69 (0.08, 4.63)	0.68 (0.07, 4.73)	0.29 (0.02, 2.88)	1.53 (0.13, 10.60)	0.45 (0.06, 2.28)	0.39 (0.04, 3.52)	0.39 (0.03, 3.49)	1.22 (0.14, 9.10)
BRI700mgIV	0.96 (0.10, 4.72)	2.15 (0.20, 20.22)	0.75 (0.22, 2.21)	BRI700mgIV	0.46 (0.05, 2.35)	1.33 (0.15, 9.66)	2.29 (0.23, 15.98)	1.87 (0.16, 13.28)	0.52 (0.06, 2.89)	0.51 (0.05, 2.98)	0.22 (0.01, 1.74)	1.18 (0.08, 6.29)	0.34 (0.04, 1.37)	0.30 (0.03, 1.93)	0.30 (0.03, 2.15)	0.94 (0.11, 5.36)
FIL200mgPO	2.11 (0.69, 5.91)	4.73 (0.95, 30.37)	1.65 (0.26, 13.79)	2.16 (0.43, 19.93)	FIL200mgPO	2.89 (0.74, 15.21)	**4.88 (1.24, 24.08)**	**4.00 (1.04, 20.21)**	1.16 (0.35, 3.76)	1.12 (0.29, 4.15)	0.49 (0.06, 2.69)	2.50 (0.71, 9.45)	0.75 (0.31, 1.57)	0.66 (0.15, 2.75)	0.67 (0.11, 3.50)	2.02 (0.59, 7.29)
GUS1200mgIV	0.73 (0.15, 2.72)	1.59 (0.23, 12.70)	0.55 (0.06, 5.53)	0.75 (0.10, 6.74)	0.35 (0.07, 1.36)	GUS1200mgIV	1.69 (0.88, 3.34)	1.38 (0.67, 2.85)	0.41 (0.07, 1.71)	0.38 (0.06, 1.89)	0.16 (0.01, 1.16)	0.86 (0.15, 4.15)	**0.26 (0.06, 0.77)**	0.23 (0.04, 1.27)	0.23 (0.03, 1.39)	0.70 (0.13, 3.22)
GUS200mgIV	0.43 (0.09, 1.51)	0.94 (0.14, 7.85)	0.33 (0.04, 3.25)	0.44 (0.06, 4.41)	**0.20 (0.04, 0.80)**	0.59 (0.30, 1.14)	GUS200mgIV	0.81 (0.44, 1.48)	**0.24 (0.04, 0.97)**	0.22 (0.04, 1.09)	**0.10 (0.01, 0.67)**	0.50 (0.10, 2.43)	**0.15 (0.04, 0.42)**	**0.13 (0.02, 0.71)**	**0.13 (0.02, 0.83)**	0.41 (0.07, 1.80)
GUS600mgIV	0.53 (0.11, 1.93)	1.16 (0.17, 9.65)	0.40 (0.05, 3.99)	0.54 (0.08, 6.26)	**0.25 (0.05, 0.96)**	0.73 (0.35, 1.48)	1.23 (0.68, 2.25)	GUS600mgIV	0.29 (0.05, 1.20)	0.28 (0.05, 1.31)	**0.12 (0.01, 0.84)**	0.62 (0.11, 2.81)	**0.19 (0.05, 0.53)**	**0.17 (0.03, 0.88)**	0.16 (0.02, 1.11)	0.51 (0.09, 2.31)
MED700IV	1.85 (0.58, 5.45)	4.14 (0.76, 27.81)	1.45 (0.22, 12.19)	1.91 (0.35, 16.99)	0.87 (0.27, 2.86)	2.47 (0.58, 13.55)	**4.24 (1.03, 23.19)**	3.45 (0.84, 19.39)	MED700IV	0.99 (0.24, 3.84)	0.42 (0.05, 2.60)	2.20 (0.57, 8.56)	0.65 (0.25, 1.52)	0.57 (0.12, 2.81)	0.60 (0.09, 3.02)	1.79 (0.47, 6.91)
MIR1000mgIV	1.89 (0.51, 6.72)	4.30 (0.70, 29.72)	1.48 (0.21, 14.58)	1.97 (0.34, 21.27)	0.90 (0.24, 3.41)	2.65 (0.53, 15.43)	4.49 (0.92, 25.26)	3.60 (0.76, 21.40)	1.01 (0.26, 4.23)	MIR1000mgIV	0.43 (0.06, 1.79)	2.21 (0.93, 5.61)	0.66 (0.22, 1.87)	0.59 (0.11, 2.98)	0.60 (0.09, 3.81)	1.82 (0.42, 7.92)
MIR200mgIV	4.27 (0.76, 37.25)	**9.72 (1.27, 127.49)**	3.48 (0.35, 57.65)	4.52 (0.58, 69.65)	2.04 (0.37, 17.19)	6.30 (0.86, 68.70)	**10.52 (1.50, 114.68)**	**8.42 (1.19, 97.16)**	2.37 (0.38, 21.97)	2.32 (0.56, 15.84)	MIR200mgIV	**5.08 (1.40, 35.41)**	1.49 (0.32, 11.68)	1.41 (0.18, 14.08)	1.36 (0.15, 16.24)	4.24 (0.72, 39.34)
MIR600mgIV	0.84 (0.22, 2.85)	1.84 (0.34, 13.54)	0.65 (0.09, 7.53)	0.85 (0.16, 11.98)	0.40 (0.11, 1.42)	1.17 (0.24, 6.47)	1.99 (0.41, 10.43)	1.62 (0.36, 8.78)	0.45 (0.12, 1.76)	0.45 (0.18, 1.07)	**0.20 (0.03, 0.72)**	MIR600mgIV	**0.29 (0.10, 0.77)**	0.26 (0.05, 1.25)	0.26 (0.04, 1.64)	0.80 (0.20, 3.86)
PBO	**2.81 (1.45, 5.81)**	**6.46 (1.51, 35.66)**	2.22 (0.44, 15.98)	2.91 (0.73, 25.66)	1.34 (0.64, 3.25)	**3.85 (1.31, 16.82)**	**6.54 (2.37, 27.26)**	**5.35 (1.87, 22.05)**	1.54 (0.66, 4.02)	1.51 (0.53, 4.56)	0.67 (0.09, 3.15)	**3.39 (1.29, 10.03)**	PBO	0.89 (0.25, 3.16)	0.92 (0.18, 3.89)	**2.76 (1.06, 7.80)**
PF10mgSC	3.19 (0.74, 13.73)	**7.10 (1.06, 55.94)**	2.60 (0.28, 25.12)	3.35 (0.52, 32.06)	1.51 (0.36, 6.72)	4.36 (0.79, 28.46)	**7.44 (1.40, 47.20)**	**5.99 (1.14, 38.85)**	1.75 (0.36, 8.20)	1.70 (0.34, 9.28)	0.71 (0.07, 5.69)	3.82 (0.80, 20.62)	1.12 (0.32, 3.97)	PF10mgSC	1.04 (0.20, 4.19)	**3.06 (1.16, 9.47)**
PF200mgSC	3.14 (0.62, 18.35)	6.99 (0.99, 70.81)	2.54 (0.29, 29.46)	3.28 (0.46, 37.62)	1.48 (0.29, 8.93)	4.42 (0.72, 35.67)	**7.60 (1.21, 57.43)**	6.13 (0.90, 47.89)	1.66 (0.33, 10.72)	1.66 (0.26, 11.70)	0.74 (0.06, 6.49)	3.81 (0.61, 25.08)	1.08 (0.26, 5.65)	0.96 (0.24, 5.08)	PF200mgSC	**2.93 (1.01, 13.16)**
PF50mgSC	1.03 (0.30, 3.38)	2.32 (0.41, 15.62)	0.82 (0.11, 7.09)	1.06 (0.19, 9.01)	0.49 (0.14, 1.69)	1.42 (0.31, 7.96)	2.46 (0.55, 13.50)	1.98 (0.43, 11.08)	0.56 (0.14, 2.12)	0.55 (0.13, 2.36)	0.24 (0.03, 1.39)	1.25 (0.26, 5.05)	**0.36 (0.13, 0.94)**	**0.33 (0.11, 0.86)**	**0.34 (0.08, 0.99)**	PF50mgSC
RIS1200mgIV	1.64 (0.78, 3.55)	3.68 (0.85, 21.23)	1.28 (0.24, 9.58)	1.68 (0.42, 16.81)	0.77 (0.34, 2.01)	2.22 (0.70, 9.89)	**3.84 (1.27, 16.50)**	3.07 (1.00, 13.56)	0.89 (0.35, 2.38)	0.88 (0.29, 2.75)	0.38 (0.05, 1.90)	1.97 (0.68, 6.05)	**0.57 (0.40, 0.82)**	0.51 (0.14, 1.93)	0.53 (0.10, 2.48)	1.58 (0.58, 5.05)
RIS200mgIV	2.15 (0.78, 5.90)	4.89 (0.97, 30.68)	1.69 (0.28, 17.03)	2.24 (0.46, 25.55)	1.02 (0.35, 3.25)	2.95 (0.79, 14.96)	**5.05 (1.38, 24.43)**	**4.14 (1.08, 20.16)**	1.17 (0.38, 4.17)	1.16 (0.31, 4.41)	0.50 (0.05, 2.88)	2.55 (0.74, 9.62)	0.75 (0.36, 1.70)	0.67 (0.16, 2.85)	0.69 (0.12, 3.67)	2.10 (0.60, 8.27)
RIS600mgIV	1.52 (0.73, 3.33)	3.43 (0.80, 19.56)	1.20 (0.23, 8.82)	1.57 (0.38, 14.95)	0.72 (0.32, 1.85)	2.07 (0.66, 9.45)	**3.53 (1.21, 15.30)**	2.88 (0.95, 12.60)	0.83 (0.33, 2.24)	0.81 (0.27, 2.56)	0.36 (0.05, 1.72)	1.82 (0.67, 5.58)	**0.54 (0.39, 0.74)**	0.48 (0.13, 1.72)	0.50 (0.09, 2.24)	1.48 (0.55, 4.43)
TES400mg200mgSC	1.42 (0.52, 3.88)	3.19 (0.67, 20.76)	1.12 (0.18, 8.79)	1.46 (0.30, 12.79)	0.67 (0.24, 1.95)	1.97 (0.51, 9.32)	3.33 (0.92, 15.27)	2.69 (0.73, 12.18)	0.77 (0.25, 2.38)	0.75 (0.21, 2.83)	0.33 (0.04, 1.85)	1.70 (0.50, 6.17)	**0.50 (0.24, 0.98)**	0.45 (0.11, 1.93)	0.46 (0.08, 2.13)	1.39 (0.41, 4.81)
UPA45mgPO	1.61 (0.76, 3.48)	3.65 (0.87, 20.92)	1.26 (0.24, 9.15)	1.65 (0.40, 14.39)	0.76 (0.34, 1.89)	2.20 (0.70, 9.95)	**3.73 (1.24, 16.20)**	3.02 (0.99, 13.35)	0.88 (0.35, 2.41)	0.85 (0.28, 2.69)	0.38 (0.05, 1.92)	1.93 (0.70, 5.96)	**0.57 (0.40, 0.79)**	0.51 (0.14, 1.89)	0.53 (0.10, 2.35)	1.57 (0.58, 4.67)
UST130mgIV	1.50 (0.60, 3.62)	3.36 (0.71, 20.15)	1.18 (0.20, 9.42)	1.53 (0.34, 14.16)	0.71 (0.28, 1.91)	2.04 (0.58, 9.93)	**3.50 (1.05, 15.88)**	2.87 (0.83, 12.96)	0.81 (0.29, 2.40)	0.79 (0.24, 2.79)	0.35 (0.04, 1.89)	1.79 (0.58, 5.96)	**0.52 (0.29, 0.93)**	0.47 (0.12, 1.84)	0.48 (0.09, 2.24)	1.44 (0.47, 4.69)
UST1mg/kgIV	1.40 (0.55, 3.72)	3.12 (0.66, 20.53)	1.11 (0.19, 8.63)	1.46 (0.32, 12.02)	0.66 (0.25, 1.95)	1.94 (0.53, 9.37)	3.28 (0.93, 15.29)	2.68 (0.76, 12.57)	0.77 (0.27, 2.40)	0.75 (0.22, 2.74)	0.33 (0.04, 1.81)	1.68 (0.53, 5.88)	**0.49 (0.26, 0.96)**	0.44 (0.10, 1.82)	0.46 (0.08, 2.09)	1.37 (0.43, 4.61)
UST3mg/kgIV	1.38 (0.53, 3.52)	3.09 (0.63, 18.36)	1.10 (0.19, 8.44)	1.41 (0.31, 13.17)	0.65 (0.24, 1.96)	1.91 (0.52, 9.31)	3.26 (0.91, 14.85)	2.66 (0.74, 12.22)	0.75 (0.26, 2.35)	0.73 (0.21, 2.70)	0.32 (0.04, 1.69)	1.64 (0.52, 5.95)	**0.48 (0.25, 0.94)**	0.42 (0.10, 1.77)	0.45 (0.08, 2.22)	1.33 (0.42, 4.78)
UST4.5mg/kgIV	1.89 (0.87, 4.71)	4.34 (0.98, 25.71)	1.51 (0.29, 11.41)	1.95 (0.46, 18.72)	0.90 (0.39, 2.64)	2.61 (0.79, 12.76)	**4.45 (1.44, 21.20)**	**3.60 (1.13, 16.99)**	1.04 (0.40, 3.11)	1.00 (0.33, 3.46)	0.45 (0.06, 2.31)	2.26 (0.80, 7.88)	0.66 (0.44, 1.17)	0.60 (0.16, 2.33)	0.62 (0.12, 3.01)	1.83 (0.66, 6.06)
UST6mg/kg90mgIV/SC	0.63 (0.13, 2.30)	1.41 (0.19, 11.31)	0.48 (0.06, 4.54)	0.65 (0.09, 6.38)	0.30 (0.06, 1.22)	0.87 (0.42, 1.75)	1.47 (0.80, 2.76)	1.20 (0.63, 2.33)	0.34 (0.06, 1.49)	0.33 (0.05, 1.63)	**0.14 (0.01, 0.98)**	0.74 (0.14, 3.70)	**0.23 (0.05, 0.64)**	0.20 (0.03, 1.09)	0.20 (0.02, 1.23)	0.61 (0.11, 2.79)
UST6mg/kgIV	1.21 (0.52, 2.81)	2.68 (0.59, 16.42)	0.95 (0.17, 7.14)	1.24 (0.29, 10.53)	0.57 (0.24, 1.56)	1.66 (0.50, 7.82)	2.82 (0.89, 12.21)	2.30 (0.70, 10.17)	0.66 (0.25, 1.88)	0.64 (0.20, 2.17)	0.29 (0.03, 1.43)	1.45 (0.50, 4.59)	**0.42 (0.26, 0.69)**	0.38 (0.10, 1.43)	0.39 (0.07, 1.76)	1.17 (0.41, 3.66)
UST90mgSC	2.40 (0.59, 9.65)	5.46 (0.83, 43.70)	1.94 (0.24, 17.49)	2.54 (0.36, 27.04)	1.14 (0.27, 4.88)	3.35 (0.64, 20.07)	**5.71 (1.13, 33.15)**	4.54 (0.95, 26.63)	1.31 (0.28, 5.88)	1.28 (0.25, 6.52)	0.56 (0.05, 4.27)	2.87 (0.58, 14.24)	0.86 (0.24, 2.76)	0.77 (0.13, 4.04)	0.78 (0.11, 4.71)	2.33 (0.50, 10.94)
VED300mgIV	1.17 (0.47, 2.73)	2.59 (0.58, 15.38)	0.90 (0.16, 7.29)	1.19 (0.26, 12.21)	0.55 (0.21, 1.45)	1.57 (0.44, 7.42)	2.71 (0.79, 12.11)	2.20 (0.64, 9.95)	0.63 (0.22, 1.81)	0.62 (0.19, 2.05)	0.27 (0.03, 1.39)	1.39 (0.44, 4.54)	**0.41 (0.22, 0.68)**	0.36 (0.09, 1.37)	0.38 (0.07, 1.73)	1.12 (0.36, 3.71)

	RIS1200mgIV	RIS200mgIV	RIS600mgIV	TES400mg200mgSC	UPA45mgPO	UST130mgIV	UST1mg/kgIV	UST3mg/kgIV	UST4.5mg/kgIV	UST6mg/kg90mgIV/SC	UST6mg/kgIV	UST90mgSC	VED300mgIV
ADA160mg80mgSC	0.61 (0.28, 1.29)	0.47 (0.17, 1.28)	0.66 (0.30, 1.37)	0.71 (0.26, 1.92)	0.62 (0.29, 1.32)	0.67 (0.28, 1.66)	0.71 (0.27, 1.83)	0.72 (0.28, 1.89)	0.53 (0.21, 1.15)	1.58 (0.43, 7.97)	0.82 (0.36, 1.93)	0.42 (0.10, 1.70)	0.85 (0.37, 2.14)
ADA80mg40mgSC	0.27 (0.05, 1.18)	0.20 (0.03, 1.03)	0.29 (0.05, 1.25)	0.31 (0.05, 1.49)	0.27 (0.05, 1.15)	0.30 (0.05, 1.41)	0.32 (0.05, 1.52)	0.32 (0.05, 1.59)	0.23 (0.04, 1.02)	0.71 (0.09, 5.19)	0.37 (0.06, 1.69)	0.18 (0.02, 1.20)	0.39 (0.07, 1.74)
BRI400mgIV	0.78 (0.10, 4.10)	0.59 (0.06, 3.60)	0.84 (0.11, 4.32)	0.89 (0.11, 5.43)	0.79 (0.11, 4.16)	0.85 (0.11, 5.03)	0.90 (0.12, 5.22)	0.91 (0.12, 5.40)	0.66 (0.09, 3.50)	2.10 (0.22, 17.79)	1.05 (0.14, 5.83)	0.52 (0.06, 4.24)	1.11 (0.14, 6.33)
BRI700mgIV	0.60 (0.06, 2.38)	0.45 (0.04, 2.18)	0.64 (0.07, 2.64)	0.68 (0.08, 3.28)	0.61 (0.07, 2.51)	0.65 (0.07, 2.96)	0.68 (0.08, 3.08)	0.71 (0.08, 3.18)	0.51 (0.05, 2.17)	1.55 (0.16, 11.25)	0.80 (0.09, 3.47)	0.39 (0.04, 2.78)	0.84 (0.08, 3.92)
FIL200mgPO	1.30 (0.50, 2.91)	0.98 (0.31, 2.87)	1.40 (0.54, 3.12)	1.48 (0.51, 4.21)	1.31 (0.53, 2.94)	1.41 (0.52, 3.56)	1.52 (0.51, 3.94)	1.53 (0.51, 4.14)	1.12 (0.38, 2.59)	3.32 (0.82, 17.14)	1.76 (0.64, 4.14)	0.87 (0.21, 3.76)	1.81 (0.69, 4.80)
GUS1200mgIV	0.45 (0.10, 1.44)	0.34 (0.07, 1.26)	0.48 (0.11, 1.51)	0.51 (0.11, 1.95)	0.45 (0.10, 1.42)	0.49 (0.10, 1.72)	0.52 (0.11, 1.90)	0.52 (0.11, 1.91)	0.38 (0.08, 1.27)	1.15 (0.57, 2.40)	0.60 (0.13, 2.01)	0.30 (0.05, 1.57)	0.64 (0.13, 2.27)
GUS200mgIV	**0.26 (0.06, 0.79)**	**0.20 (0.04, 0.73)**	**0.28 (0.07, 0.82)**	0.30 (0.07, 1.09)	**0.27 (0.06, 0.80)**	**0.29 (0.06, 0.95)**	0.31 (0.07, 1.08)	0.31 (0.07, 1.10)	**0.22 (0.05, 0.70)**	0.68 (0.36, 1.26)	0.36 (0.08, 1.12)	**0.18 (0.03, 0.89)**	0.37 (0.08, 1.26)
GUS600mgIV	0.33 (0.07, 1.00)	**0.24 (0.05, 0.93)**	0.35 (0.08, 1.05)	0.37 (0.08, 1.37)	0.33 (0.07, 1.01)	0.35 (0.08, 1.20)	0.37 (0.08, 1.31)	0.38 (0.08, 1.35)	**0.28 (0.06, 0.89)**	0.84 (0.43, 1.59)	0.43 (0.10, 1.42)	0.22 (0.04, 1.06)	0.45 (0.10, 1.57)
MED700IV	1.13 (0.42, 2.86)	0.86 (0.24, 2.65)	1.21 (0.45, 3.01)	1.29 (0.42, 4.02)	1.13 (0.41, 2.85)	1.23 (0.42, 3.43)	1.30 (0.42, 3.77)	1.34 (0.42, 3.89)	0.96 (0.32, 2.52)	2.90 (0.67, 15.78)	1.52 (0.53, 4.05)	0.76 (0.17, 3.53)	1.60 (0.55, 4.53)
MIR1000mgIV	1.13 (0.36, 3.50)	0.87 (0.23, 3.21)	1.24 (0.39, 3.68)	1.34 (0.35, 4.71)	1.17 (0.37, 3.53)	1.26 (0.36, 4.21)	1.33 (0.36, 4.54)	1.37 (0.37, 4.69)	1.00 (0.29, 3.05)	3.01 (0.61, 18.55)	1.56 (0.46, 4.96)	0.78 (0.15, 4.00)	1.62 (0.49, 5.34)
MIR200mgIV	2.61 (0.53, 20.39)	2.01 (0.35, 18.87)	2.78 (0.58, 21.89)	2.99 (0.54, 26.36)	2.62 (0.52, 21.66)	2.86 (0.53, 25.17)	3.03 (0.55, 24.78)	3.14 (0.59, 24.92)	2.24 (0.43, 18.18)	**7.04 (1.02, 86.43)**	3.50 (0.70, 30.18)	1.80 (0.23, 19.59)	3.69 (0.72, 31.02)
MIR600mgIV	0.51 (0.17, 1.46)	0.39 (0.10, 1.36)	0.55 (0.18, 1.50)	0.59 (0.16, 2.01)	0.52 (0.17, 1.44)	0.56 (0.17, 1.73)	0.60 (0.17, 1.90)	0.61 (0.17, 1.92)	0.44 (0.13, 1.25)	1.34 (0.27, 7.31)	0.69 (0.22, 2.01)	0.35 (0.07, 1.73)	0.72 (0.22, 2.26)
PBO	**1.74 (1.21, 2.50)**	1.33 (0.59, 2.79)	**1.87 (1.35, 2.58)**	**2.00 (1.02, 4.23)**	**1.76 (1.27, 2.50)**	**1.92 (1.08, 3.43)**	**2.04 (1.05, 3.86)**	**2.08 (1.06, 3.95)**	1.51 (0.86, 2.29)	**4.42 (1.56, 18.97)**	**2.36 (1.46, 3.81)**	1.17 (0.36, 4.09)	**2.44 (1.47, 4.49)**
PF10mgSC	1.94 (0.52, 7.40)	1.49 (0.35, 6.23)	2.10 (0.58, 7.55)	2.22 (0.52, 9.34)	1.97 (0.53, 7.17)	2.11 (0.54, 8.63)	2.27 (0.55, 9.73)	2.36 (0.56, 9.68)	1.67 (0.43, 6.23)	5.01 (0.92, 33.92)	2.63 (0.70, 10.52)	1.31 (0.25, 7.47)	2.75 (0.73, 11.46)
PF200mgSC	1.87 (0.40, 10.09)	1.45 (0.27, 8.16)	2.01 (0.45, 10.92)	2.17 (0.47, 12.55)	1.90 (0.43, 10.47)	2.07 (0.45, 11.75)	2.20 (0.48, 12.54)	2.23 (0.45, 12.80)	1.62 (0.33, 8.50)	5.12 (0.82, 40.12)	2.57 (0.57, 13.87)	1.28 (0.21, 9.49)	2.65 (0.58, 15.25)
PF50mgSC	0.63 (0.20, 1.73)	0.48 (0.12, 1.65)	0.67 (0.23, 1.82)	0.72 (0.21, 2.44)	0.64 (0.21, 1.72)	0.69 (0.21, 2.11)	0.73 (0.22, 2.31)	0.75 (0.21, 2.37)	0.55 (0.16, 1.52)	1.65 (0.36, 9.30)	0.85 (0.27, 2.46)	0.43 (0.09, 1.98)	0.89 (0.27, 2.80)
RIS1200mgIV	RIS1200mgIV	0.76 (0.34, 1.66)	1.07 (0.77, 1.46)	1.15 (0.54, 2.65)	1.01 (0.63, 1.68)	1.10 (0.56, 2.19)	1.17 (0.55, 2.44)	1.20 (0.57, 2.50)	0.87 (0.47, 1.34)	2.60 (0.83, 11.71)	1.35 (0.76, 2.47)	0.68 (0.20, 2.46)	1.40 (0.77, 2.87)
RIS200mgIV	1.31 (0.60, 2.94)	RIS200mgIV	1.41 (0.69, 3.04)	1.51 (0.55, 4.55)	1.33 (0.59, 3.15)	1.46 (0.56, 3.78)	1.53 (0.54, 4.14)	1.56 (0.57, 4.35)	1.14 (0.47, 2.60)	3.42 (0.92, 16.96)	1.78 (0.74, 4.47)	0.91 (0.21, 3.68)	1.85 (0.75, 5.15)
RIS600mgIV	0.93 (0.68, 1.29)	0.71 (0.33, 1.46)	RIS600mgIV	1.08 (0.51, 2.43)	0.94 (0.60, 1.50)	1.02 (0.53, 2.04)	1.09 (0.51, 2.25)	1.12 (0.54, 2.33)	0.81 (0.49, 1.15)	2.40 (0.79, 10.64)	1.25 (0.71, 2.27)	0.63 (0.18, 2.24)	1.30 (0.71, 2.68)
TES400mg200mgSC	0.87 (0.38, 1.86)	0.66 (0.22, 1.82)	0.93 (0.41, 1.96)	TES400mg200mgSC	0.87 (0.39, 1.88)	0.95 (0.38, 2.33)	1.01 (0.38, 2.61)	1.03 (0.38, 2.67)	0.75 (0.29, 1.62)	2.25 (0.61, 10.90)	1.17 (0.49, 2.69)	0.58 (0.15, 2.45)	1.21 (0.50, 3.09)
UPA45mgPO	0.99 (0.59, 1.59)	0.75 (0.32, 1.68)	1.06 (0.67, 1.66)	1.14 (0.53, 2.56)	UPA45mgPO	1.08 (0.55, 2.14)	1.15 (0.54, 2.37)	1.17 (0.54, 2.45)	0.86 (0.43, 1.43)	2.52 (0.83, 11.60)	1.33 (0.75, 2.41)	0.67 (0.20, 2.42)	1.39 (0.76, 2.77)
UST130mgIV	0.91 (0.46, 1.78)	0.69 (0.26, 1.80)	0.98 (0.49, 1.89)	1.05 (0.43, 2.64)	0.92 (0.47, 1.83)	UST130mgIV	1.07 (0.49, 2.19)	1.10 (0.49, 2.33)	0.79 (0.34, 1.59)	2.35 (0.69, 11.21)	1.23 (0.74, 2.15)	0.62 (0.16, 2.45)	1.28 (0.59, 3.05)
UST1mg/kgIV	0.85 (0.41, 1.83)	0.65 (0.24, 1.84)	0.92 (0.44, 1.95)	0.99 (0.38, 2.66)	0.87 (0.42, 1.84)	0.94 (0.46, 2.04)	UST1mg/kgIV	1.02 (0.54, 1.98)	0.74 (0.31, 1.61)	2.22 (0.62, 10.71)	1.16 (0.65, 2.13)	0.59 (0.15, 2.35)	1.21 (0.54, 3.04)
UST3mg/kgIV	0.84 (0.40, 1.76)	0.64 (0.23, 1.74)	0.89 (0.43, 1.87)	0.97 (0.37, 2.65)	0.85 (0.41, 1.83)	0.91 (0.43, 2.03)	0.98 (0.50, 1.85)	UST3mg/kgIV	0.73 (0.29, 1.56)	2.20 (0.59, 10.52)	1.14 (0.61, 2.06)	0.57 (0.15, 2.26)	1.17 (0.52, 2.91)
UST4.5mg/kgIV	1.14 (0.75, 2.15)	0.88 (0.38, 2.15)	1.23 (0.87, 2.05)	1.33 (0.62, 3.42)	1.16 (0.70, 2.31)	1.26 (0.63, 2.95)	1.35 (0.62, 3.23)	1.37 (0.64, 3.40)	UST4.5mg/kgIV	3.00 (0.94, 14.82)	1.56 (0.84, 3.31)	0.79 (0.22, 3.04)	1.62 (0.84, 3.94)
UST6mg/kg90mgIV/SC	0.38 (0.09, 1.20)	0.29 (0.06, 1.09)	0.42 (0.09, 1.26)	0.44 (0.09, 1.65)	0.40 (0.09, 1.20)	0.43 (0.09, 1.44)	0.45 (0.09, 1.63)	0.45 (0.10, 1.68)	0.33 (0.07, 1.07)	UST6mg/kg90mgIV/SC	0.52 (0.12, 1.69)	0.26 (0.04, 1.30)	0.55 (0.12, 1.88)
UST6mg/kgIV	0.74 (0.40, 1.32)	0.56 (0.22, 1.35)	0.80 (0.44, 1.42)	0.85 (0.37, 2.04)	0.75 (0.42, 1.33)	0.81 (0.46, 1.36)	0.86 (0.47, 1.53)	0.88 (0.49, 1.63)	0.64 (0.30, 1.19)	1.91 (0.59, 8.55)	UST6mg/kgIV	0.50 (0.14, 1.87)	1.04 (0.52, 2.31)
UST90mgSC	1.47 (0.41, 5.06)	1.10 (0.27, 4.66)	1.58 (0.45, 5.42)	1.72 (0.41, 6.81)	1.50 (0.41, 5.13)	1.62 (0.41, 6.08)	1.69 (0.43, 6.72)	1.77 (0.44, 6.64)	1.26 (0.33, 4.54)	3.84 (0.77, 22.94)	1.99 (0.54, 7.25)	UST90mgSC	2.10 (0.55, 7.94)
VED300mgIV	0.71 (0.35, 1.30)	0.54 (0.19, 1.34)	0.77 (0.37, 1.41)	0.83 (0.32, 2.01)	0.72 (0.36, 1.31)	0.78 (0.33, 1.70)	0.83 (0.33, 1.87)	0.85 (0.34, 1.94)	0.62 (0.25, 1.20)	1.83 (0.53, 8.53)	0.96 (0.43, 1.93)	0.48 (0.13, 1.83)	VED300mgIV

Table S12F: League table for clinical response with reductions of 100 points.

	ADA160mg80mg60mgSC	ADA160mg80mgSC	ADA40mg20mgSC	ADA80mg40mgSC	AMI0.4mgPO	CDP10mg/kgIV	CER400mgSC	ETA25mgSC	FON0.1mg/kgIV	FON1mg/kgIV	FON4mg/kgIV	INF10mg/kgIV	INF20mg/kgIV	INF5mg/kgIV	NAT300mgIV	NAT3mg/kgIV	NAT3mg/kgIVx2	NAT6mg/kgIVx2	ONE10mgSC	ONE25mgSC	ONE35mgSC	ONE50mgSC
ADA160mg80mg60mgSC	ADA160mg80mg60mgSC	0.65 (0.40, 1.01)	0.61 (0.36, 1.02)	0.69 (0.43, 1.12)	**0.33 (0.18, 0.58)**	**0.47 (0.28, 0.76)**	0.90 (0.42, 2.14)	0.40 (0.13, 1.12)	**0.32 (0.08, 0.87)**	0.46 (0.19, 1.11)	0.54 (0.25, 1.33)	0.97 (0.42, 3.23)	1.27 (0.55, 3.92)	1.61 (0.73, 5.02)	**0.51 (0.32, 0.76)**	**0.47 (0.26, 0.85)**	0.57 (0.33, 1.02)	0.60 (0.34, 1.08)	0.51 (0.21, 1.23)	**0.24 (0.08, 0.69)**	0.51 (0.21, 1.27)	**0.35 (0.13, 0.94)**
ADA160mg80mgSC	1.55 (0.99, 2.49)	ADA160mg80mgSC	0.94 (0.68, 1.30)	1.08 (0.84, 1.39)	**0.51 (0.31, 0.82)**	0.72 (0.49, 1.10)	1.41 (0.72, 3.09)	0.62 (0.23, 1.70)	0.50 (0.13, 1.32)	0.71 (0.33, 1.67)	0.82 (0.41, 1.92)	1.51 (0.69, 4.75)	1.97 (0.89, 5.93)	**2.52 (1.21, 7.47)**	0.79 (0.58, 1.07)	0.73 (0.44, 1.19)	0.88 (0.57, 1.43)	0.94 (0.60, 1.51)	0.78 (0.34, 1.78)	0.37 (0.13, 1.03)	0.80 (0.34, 1.83)	0.55 (0.22, 1.40)
ADA40mg20mgSC	1.65 (0.98, 2.77)	1.06 (0.77, 1.48)	ADA40mg20mgSC	1.15 (0.82, 1.59)	**0.55 (0.31, 0.95)**	0.77 (0.48, 1.22)	1.49 (0.70, 3.53)	0.66 (0.23, 1.87)	0.52 (0.14, 1.44)	0.75 (0.34, 1.82)	0.87 (0.41, 2.14)	1.61 (0.71, 5.29)	2.09 (0.91, 6.48)	**2.64 (1.25, 8.35)**	0.83 (0.56, 1.23)	0.77 (0.45, 1.31)	0.94 (0.57, 1.63)	1.00 (0.59, 1.70)	0.84 (0.36, 1.94)	0.39 (0.13, 1.10)	0.84 (0.35, 2.06)	0.58 (0.22, 1.52)
ADA80mg40mgSC	1.44 (0.90, 2.34)	0.93 (0.72, 1.19)	0.87 (0.63, 1.22)	ADA80mg40mgSC	**0.48 (0.27, 0.79)**	0.67 (0.43, 1.06)	1.31 (0.64, 2.91)	0.57 (0.21, 1.61)	0.46 (0.13, 1.24)	0.66 (0.30, 1.52)	0.77 (0.38, 1.79)	1.41 (0.63, 4.41)	1.82 (0.80, 5.47)	**2.32 (1.10, 7.02)**	0.73 (0.52, 1.03)	0.68 (0.40, 1.13)	0.82 (0.52, 1.35)	0.87 (0.53, 1.44)	0.73 (0.32, 1.69)	**0.35 (0.12, 0.96)**	0.74 (0.31, 1.75)	0.51 (0.20, 1.29)
AMI0.4mgPO	**3.03 (1.74, 5.57)**	**1.95 (1.22, 3.26)**	**1.83 (1.05, 3.24)**	**2.10 (1.27, 3.68)**	AMI0.4mgPO	1.41 (0.83, 2.43)	**2.73 (1.25, 6.71)**	1.21 (0.41, 3.52)	0.96 (0.25, 2.67)	1.39 (0.61, 3.28)	1.61 (0.75, 3.88)	**3.01 (1.26, 9.91)**	**3.84 (1.63, 12.52)**	**4.89 (2.21, 15.83)**	1.53 (0.96, 2.47)	1.43 (0.78, 2.70)	1.73 (0.96, 3.22)	1.84 (1.00, 3.37)	1.53 (0.63, 3.97)	0.72 (0.23, 2.11)	1.55 (0.63, 3.99)	1.07 (0.39, 3.02)
CDP10mg/kgIV	**2.14 (1.31, 3.63)**	1.38 (0.91, 2.06)	1.30 (0.82, 2.09)	1.49 (0.94, 2.31)	0.71 (0.41, 1.20)	CDP10mg/kgIV	1.91 (0.94, 4.50)	0.84 (0.30, 2.37)	0.68 (0.18, 1.88)	0.98 (0.44, 2.33)	1.13 (0.54, 2.79)	2.07 (0.92, 6.71)	**2.68 (1.21, 8.25)**	**3.43 (1.60, 10.55)**	1.08 (0.74, 1.59)	1.01 (0.59, 1.72)	1.22 (0.75, 2.06)	1.30 (0.77, 2.17)	1.08 (0.47, 2.56)	0.51 (0.17, 1.48)	1.10 (0.44, 2.63)	0.76 (0.28, 1.97)
CER400mgSC	1.11 (0.47, 2.41)	0.71 (0.32, 1.40)	0.67 (0.28, 1.43)	0.76 (0.34, 1.55)	**0.37 (0.15, 0.80)**	0.52 (0.22, 1.06)	CER400mgSC	0.44 (0.13, 1.40)	0.34 (0.08, 1.10)	0.51 (0.17, 1.39)	0.59 (0.21, 1.65)	1.10 (0.38, 3.86)	1.42 (0.49, 4.73)	1.81 (0.64, 5.97)	0.56 (0.25, 1.10)	0.52 (0.22, 1.16)	0.64 (0.27, 1.34)	0.68 (0.28, 1.44)	0.56 (0.19, 1.58)	**0.27 (0.07, 0.89)**	0.56 (0.20, 1.70)	0.39 (0.12, 1.14)
ETA25mgSC	2.51 (0.89, 7.45)	1.62 (0.59, 4.39)	1.52 (0.53, 4.32)	1.77 (0.62, 4.66)	0.83 (0.28, 2.44)	1.19 (0.42, 3.31)	2.25 (0.71, 7.97)	ETA25mgSC	0.79 (0.15, 3.28)	1.15 (0.34, 4.35)	1.34 (0.41, 4.76)	2.55 (0.70, 10.49)	3.24 (0.91, 12.91)	**4.15 (1.15, 16.35)**	1.27 (0.48, 3.24)	1.20 (0.40, 3.41)	1.45 (0.51, 4.07)	1.54 (0.53, 4.27)	1.28 (0.37, 4.48)	0.58 (0.15, 2.45)	1.29 (0.38, 4.71)	0.88 (0.25, 3.34)
FON0.1mg/kgIV	**3.14 (1.15, 12.11)**	2.02 (0.76, 7.42)	1.92 (0.69, 7.34)	2.19 (0.80, 7.96)	1.04 (0.37, 3.99)	1.47 (0.53, 5.69)	2.91 (0.91, 12.23)	1.26 (0.30, 6.50)	FON0.1mg/kgIV	1.46 (0.64, 4.91)	1.68 (0.76, 5.70)	3.19 (0.92, 15.82)	**4.17 (1.25, 18.92)**	**5.32 (1.62, 23.87)**	1.59 (0.60, 5.83)	1.50 (0.51, 5.73)	1.81 (0.62, 7.07)	1.93 (0.66, 7.34)	1.60 (0.49, 7.24)	0.77 (0.19, 3.70)	1.65 (0.49, 8.13)	1.12 (0.31, 5.32)
FON1mg/kgIV	2.18 (0.90, 5.20)	1.40 (0.60, 3.06)	1.33 (0.55, 2.94)	1.51 (0.66, 3.34)	0.72 (0.30, 1.65)	1.02 (0.43, 2.28)	1.95 (0.72, 5.75)	0.87 (0.23, 2.92)	0.69 (0.20, 1.57)	FON1mg/kgIV	1.16 (0.65, 2.18)	2.14 (0.68, 8.17)	2.77 (0.91, 9.94)	**3.55 (1.20, 12.53)**	1.11 (0.48, 2.38)	1.03 (0.41, 2.41)	1.25 (0.51, 2.85)	1.32 (0.52, 3.10)	1.10 (0.36, 3.31)	0.52 (0.15, 1.78)	1.11 (0.34, 3.49)	0.76 (0.24, 2.46)
FON4mg/kgIV	1.87 (0.75, 3.99)	1.22 (0.52, 2.44)	1.14 (0.47, 2.43)	1.30 (0.56, 2.66)	0.62 (0.26, 1.33)	0.89 (0.36, 1.84)	1.69 (0.60, 4.72)	0.74 (0.21, 2.42)	0.59 (0.18, 1.31)	0.86 (0.46, 1.54)	FON4mg/kgIV	1.83 (0.58, 6.72)	2.39 (0.78, 8.59)	**3.06 (1.03, 10.63)**	0.95 (0.42, 1.89)	0.89 (0.35, 1.94)	1.07 (0.43, 2.33)	1.15 (0.44, 2.44)	0.96 (0.31, 2.67)	0.45 (0.13, 1.49)	0.95 (0.32, 2.78)	0.66 (0.20, 2.04)
INF10mg/kgIV	1.03 (0.31, 2.38)	0.66 (0.21, 1.44)	0.62 (0.19, 1.40)	0.71 (0.23, 1.58)	**0.33 (0.10, 0.80)**	0.48 (0.15, 1.09)	0.91 (0.26, 2.66)	0.39 (0.10, 1.42)	0.31 (0.06, 1.09)	0.47 (0.12, 1.47)	0.55 (0.15, 1.73)	INF10mg/kgIV	1.28 (0.77, 2.20)	**1.64 (1.05, 2.67)**	0.52 (0.16, 1.11)	0.48 (0.15, 1.16)	0.59 (0.17, 1.38)	0.62 (0.19, 1.50)	0.51 (0.14, 1.52)	**0.24 (0.05, 0.85)**	0.52 (0.14, 1.60)	0.35 (0.08, 1.13)
INF20mg/kgIV	0.79 (0.26, 1.83)	0.51 (0.17, 1.12)	0.48 (0.15, 1.10)	0.55 (0.18, 1.24)	**0.26 (0.08, 0.61)**	**0.37 (0.12, 0.83)**	0.70 (0.21, 2.06)	0.31 (0.08, 1.09)	**0.24 (0.05, 0.80)**	0.36 (0.10, 1.10)	0.42 (0.12, 1.28)	0.78 (0.45, 1.30)	INF20mg/kgIV	1.27 (0.86, 1.96)	**0.40 (0.13, 0.87)**	**0.37 (0.12, 0.92)**	0.45 (0.14, 1.06)	0.47 (0.15, 1.16)	0.39 (0.11, 1.19)	**0.19 (0.04, 0.68)**	0.39 (0.11, 1.25)	**0.27 (0.07, 0.90)**
INF5mg/kgIV	0.62 (0.20, 1.38)	**0.40 (0.13, 0.83)**	**0.38 (0.12, 0.80)**	**0.43 (0.14, 0.91)**	**0.20 (0.06, 0.45)**	**0.29 (0.09, 0.63)**	0.55 (0.17, 1.56)	**0.24 (0.06, 0.87)**	**0.19 (0.04, 0.62)**	**0.28 (0.08, 0.83)**	**0.33 (0.09, 0.97)**	**0.61 (0.37, 0.95)**	0.79 (0.51, 1.16)	INF5mg/kgIV	**0.31 (0.11, 0.64)**	**0.29 (0.10, 0.68)**	**0.35 (0.11, 0.80)**	**0.37 (0.12, 0.87)**	**0.31 (0.09, 0.90)**	**0.15 (0.03, 0.51)**	**0.31 (0.09, 0.93)**	**0.21 (0.05, 0.69)**
NAT300mgIV	**1.97 (1.32, 3.09)**	1.27 (0.94, 1.73)	1.20 (0.81, 1.80)	1.37 (0.97, 1.93)	0.65 (0.41, 1.04)	0.92 (0.63, 1.35)	1.78 (0.91, 4.00)	0.79 (0.31, 2.09)	0.63 (0.17, 1.67)	0.90 (0.42, 2.08)	1.05 (0.53, 2.39)	1.93 (0.90, 6.12)	**2.49 (1.15, 7.51)**	**3.17 (1.55, 9.35)**	NAT300mgIV	0.93 (0.60, 1.50)	1.13 (0.73, 1.78)	1.20 (0.78, 1.88)	1.00 (0.44, 2.33)	0.47 (0.16, 1.31)	1.01 (0.45, 2.37)	0.70 (0.28, 1.79)
NAT3mg/kgIV	**2.13 (1.18, 3.82)**	1.36 (0.84, 2.26)	1.29 (0.77, 2.23)	1.47 (0.89, 2.47)	0.70 (0.37, 1.29)	0.99 (0.58, 1.69)	1.92 (0.86, 4.61)	0.83 (0.29, 2.50)	0.67 (0.17, 1.97)	0.97 (0.42, 2.43)	1.13 (0.52, 2.82)	2.08 (0.86, 6.57)	**2.72 (1.09, 8.25)**	**3.47 (1.47, 10.49)**	1.08 (0.67, 1.68)	NAT3mg/kgIV	1.22 (0.85, 1.77)	1.29 (0.88, 1.87)	1.09 (0.43, 2.70)	0.50 (0.16, 1.51)	1.08 (0.42, 2.68)	0.75 (0.27, 2.05)
NAT3mg/kgIVx2	1.75 (0.98, 3.04)	1.13 (0.70, 1.76)	1.06 (0.61, 1.74)	1.22 (0.74, 1.92)	0.58 (0.31, 1.05)	0.82 (0.49, 1.34)	1.56 (0.75, 3.77)	0.69 (0.25, 1.98)	0.55 (0.14, 1.61)	0.80 (0.35, 1.97)	0.93 (0.43, 2.30)	1.69 (0.72, 5.76)	2.23 (0.95, 6.96)	**2.85 (1.24, 8.80)**	0.89 (0.56, 1.36)	0.82 (0.56, 1.17)	NAT3mg/kgIVx2	1.06 (0.74, 1.48)	0.89 (0.37, 2.12)	0.42 (0.14, 1.20)	0.89 (0.37, 2.20)	0.61 (0.23, 1.67)
NAT6mg/kgIVx2	1.66 (0.93, 2.92)	1.06 (0.66, 1.66)	1.00 (0.59, 1.69)	1.15 (0.69, 1.87)	0.54 (0.30, 1.00)	0.77 (0.46, 1.30)	1.48 (0.69, 3.53)	0.65 (0.23, 1.88)	0.52 (0.14, 1.52)	0.76 (0.32, 1.91)	0.87 (0.41, 2.25)	1.62 (0.67, 5.35)	2.11 (0.86, 6.69)	**2.70 (1.15, 8.27)**	0.83 (0.53, 1.28)	0.78 (0.54, 1.14)	0.94 (0.67, 1.35)	NAT6mg/kgIVx2	0.84 (0.34, 2.06)	0.39 (0.13, 1.12)	0.84 (0.35, 2.03)	0.58 (0.22, 1.58)
ONE10mgSC	1.98 (0.81, 4.74)	1.28 (0.56, 2.91)	1.19 (0.52, 2.80)	1.37 (0.59, 3.14)	0.65 (0.25, 1.58)	0.92 (0.39, 2.15)	1.78 (0.63, 5.16)	0.78 (0.22, 2.73)	0.63 (0.14, 2.03)	0.91 (0.30, 2.78)	1.04 (0.37, 3.19)	1.96 (0.66, 7.25)	2.54 (0.84, 9.28)	**3.25 (1.11, 11.36)**	1.00 (0.43, 2.25)	0.92 (0.37, 2.31)	1.13 (0.47, 2.74)	1.19 (0.49, 2.91)	ONE10mgSC	0.48 (0.17, 1.10)	1.01 (0.49, 2.04)	0.70 (0.29, 1.49)
ONE25mgSC	**4.14 (1.44, 12.61)**	2.71 (0.97, 7.67)	2.54 (0.91, 7.42)	**2.89 (1.04, 8.41)**	1.39 (0.47, 4.31)	1.97 (0.67, 5.85)	**3.76 (1.12, 14.55)**	1.71 (0.41, 6.86)	1.29 (0.27, 5.23)	1.92 (0.56, 6.87)	2.24 (0.67, 7.94)	**4.18 (1.18, 19.73)**	**5.34 (1.48, 25.28)**	**6.83 (1.98, 31.37)**	2.11 (0.76, 6.10)	1.99 (0.66, 6.14)	2.40 (0.84, 7.26)	2.56 (0.89, 7.65)	2.07 (0.91, 5.74)	ONE25mgSC	2.09 (0.88, 5.79)	1.47 (0.56, 4.14)
ONE35mgSC	1.96 (0.79, 4.71)	1.26 (0.55, 2.96)	1.18 (0.48, 2.88)	1.35 (0.57, 3.25)	0.64 (0.25, 1.58)	0.91 (0.38, 2.27)	1.79 (0.59, 5.06)	0.78 (0.21, 2.62)	0.61 (0.12, 2.04)	0.90 (0.29, 2.91)	1.05 (0.36, 3.17)	1.93 (0.62, 7.34)	2.54 (0.80, 9.14)	**3.22 (1.07, 11.69)**	0.99 (0.42, 2.20)	0.92 (0.37, 2.37)	1.12 (0.45, 2.70)	1.18 (0.49, 2.85)	0.99 (0.49, 2.04)	0.48 (0.17, 1.14)	ONE35mgSC	0.69 (0.29, 1.51)
ONE50mgSC	**2.84 (1.07, 7.70)**	1.82 (0.72, 4.55)	1.71 (0.66, 4.48)	1.96 (0.78, 4.95)	0.93 (0.33, 2.55)	1.32 (0.51, 3.56)	2.56 (0.88, 8.05)	1.13 (0.30, 4.08)	0.89 (0.19, 3.27)	1.31 (0.41, 4.13)	1.52 (0.49, 4.97)	2.85 (0.88, 11.80)	**3.68 (1.11, 14.89)**	**4.69 (1.45, 18.41)**	1.43 (0.56, 3.60)	1.34 (0.49, 3.65)	1.63 (0.60, 4.30)	1.72 (0.63, 4.56)	1.42 (0.67, 3.40)	0.68 (0.24, 1.79)	1.44 (0.66, 3.42)	ONE50mgSC

	ADA160mg80mg60mgSC	ADA160mg80mgSC	ADA40mg20mgSC	ADA80mg40mgSC	AMI0.4mgPO	CDP10mg/kgIV	CER400mgSC	ETA25mgSC	FON0.1mg/kgIV	FON1mg/kgIV	FON4mg/kgIV	INF10mg/kgIV	INF20mg/kgIV	INF5mg/kgIV	NAT300mgIV	NAT3mg/kgIV	NAT3mg/kgIVx2	NAT6mg/kgIVx2	ONE10mgSC	ONE25mgSC	ONE35mgSC	ONE50mgSC
ONT22.5mgSC	**2.38 (1.27, 4.61)**	1.53 (0.88, 2.63)	1.45 (0.78, 2.67)	1.64 (0.94, 2.95)	0.78 (0.41, 1.57)	1.11 (0.61, 2.03)	2.16 (0.93, 5.19)	0.94 (0.33, 2.91)	0.75 (0.19, 2.18)	1.09 (0.45, 2.80)	1.27 (0.56, 3.31)	2.36 (0.96, 7.95)	**3.02 (1.28, 9.54)**	**3.90 (1.64, 12.20)**	1.20 (0.70, 2.04)	1.12 (0.58, 2.18)	1.36 (0.71, 2.56)	1.44 (0.75, 2.76)	1.20 (0.48, 3.06)	0.56 (0.18, 1.73)	1.21 (0.51, 3.08)	0.83 (0.31, 2.31)
ONT225mgSC	**2.54 (1.32, 4.69)**	1.63 (0.93, 2.74)	1.55 (0.83, 2.75)	1.76 (1.00, 3.06)	0.84 (0.44, 1.58)	1.19 (0.63, 2.08)	2.29 (1.00, 5.72)	1.01 (0.35, 2.96)	0.80 (0.20, 2.23)	1.16 (0.48, 2.95)	1.36 (0.59, 3.27)	**2.48 (1.03, 8.22)**	**3.24 (1.36, 10.01)**	**4.16 (1.79, 12.52)**	1.28 (0.75, 2.13)	1.20 (0.62, 2.24)	1.45 (0.76, 2.69)	1.54 (0.81, 2.88)	1.28 (0.52, 3.41)	0.61 (0.19, 1.92)	1.29 (0.54, 3.36)	0.89 (0.33, 2.52)
ONT75mgSC	**2.36 (1.25, 4.59)**	1.52 (0.88, 2.72)	1.43 (0.79, 2.66)	1.63 (0.94, 2.98)	0.78 (0.42, 1.52)	1.11 (0.62, 2.01)	2.15 (0.92, 5.40)	0.95 (0.33, 2.85)	0.76 (0.19, 2.13)	1.09 (0.45, 2.82)	1.26 (0.56, 3.18)	2.32 (0.95, 7.72)	**3.01 (1.25, 9.36)**	**3.84 (1.67, 12.06)**	1.19 (0.71, 2.03)	1.11 (0.59, 2.17)	1.35 (0.74, 2.58)	1.44 (0.76, 2.72)	1.19 (0.49, 3.24)	0.57 (0.18, 1.83)	1.21 (0.48, 3.23)	0.83 (0.31, 2.39)
PBO	**2.48 (1.72, 3.78)**	**1.61 (1.27, 2.03)**	**1.51 (1.07, 2.17)**	**1.73 (1.30, 2.32)**	0.82 (0.53, 1.25)	1.16 (0.84, 1.64)	**2.24 (1.18, 4.87)**	0.99 (0.38, 2.60)	0.79 (0.22, 2.07)	1.14 (0.55, 2.57)	1.32 (0.69, 2.98)	**2.43 (1.16, 7.44)**	**3.13 (1.46, 9.36)**	**3.99 (1.98, 11.51)**	**1.26 (1.04, 1.53)**	1.18 (0.77, 1.80)	1.42 (0.97, 2.17)	**1.51 (1.02, 2.26)**	1.25 (0.58, 2.81)	0.60 (0.21, 1.61)	1.28 (0.58, 2.89)	0.88 (0.36, 2.18)
PF10mgSC	2.03 (0.96, 4.15)	1.31 (0.66, 2.46)	1.23 (0.59, 2.47)	1.41 (0.70, 2.73)	0.67 (0.31, 1.45)	0.95 (0.46, 1.87)	1.83 (0.71, 4.68)	0.79 (0.27, 2.49)	0.64 (0.15, 2.06)	0.94 (0.34, 2.64)	1.08 (0.43, 3.04)	1.99 (0.74, 7.16)	2.60 (0.95, 9.01)	**3.30 (1.28, 11.46)**	1.03 (0.52, 1.90)	0.96 (0.44, 1.96)	1.16 (0.56, 2.37)	1.22 (0.57, 2.50)	1.02 (0.37, 2.83)	0.48 (0.14, 1.58)	1.03 (0.37, 2.88)	0.72 (0.23, 2.06)
PF200mgSC	1.68 (0.80, 3.80)	1.08 (0.55, 2.23)	1.02 (0.49, 2.20)	1.18 (0.59, 2.43)	0.56 (0.25, 1.26)	0.79 (0.38, 1.68)	1.55 (0.60, 4.14)	0.66 (0.22, 2.20)	0.54 (0.12, 1.73)	0.78 (0.28, 2.28)	0.89 (0.36, 2.54)	1.67 (0.63, 6.23)	2.14 (0.84, 7.81)	**2.75 (1.10, 9.57)**	0.85 (0.44, 1.78)	0.80 (0.37, 1.81)	0.97 (0.46, 2.11)	1.02 (0.48, 2.26)	0.85 (0.32, 2.42)	0.40 (0.12, 1.35)	0.87 (0.31, 2.54)	0.60 (0.20, 1.88)
PF50mgSC	1.43 (0.72, 2.79)	0.92 (0.49, 1.64)	0.87 (0.44, 1.66)	0.99 (0.52, 1.82)	**0.47 (0.23, 0.95)**	0.67 (0.34, 1.23)	1.30 (0.53, 3.27)	0.56 (0.20, 1.71)	0.45 (0.11, 1.35)	0.66 (0.24, 1.73)	0.76 (0.32, 1.97)	1.42 (0.56, 4.99)	1.82 (0.73, 5.92)	2.32 (0.95, 7.59)	0.72 (0.39, 1.29)	0.67 (0.32, 1.31)	0.81 (0.41, 1.58)	0.86 (0.42, 1.71)	0.73 (0.27, 1.89)	0.34 (0.10, 1.05)	0.73 (0.28, 1.89)	0.51 (0.17, 1.40)
SEM60mgIV	**2.94 (1.52, 5.76)**	**1.89 (1.03, 3.44)**	1.77 (0.93, 3.41)	**2.02 (1.11, 3.74)**	0.97 (0.47, 1.94)	1.36 (0.71, 2.61)	**2.62 (1.14, 6.79)**	1.14 (0.40, 3.57)	0.92 (0.23, 2.68)	1.36 (0.52, 3.60)	1.56 (0.66, 4.18)	**2.89 (1.13, 9.28)**	**3.82 (1.45, 11.54)**	**4.81 (1.90, 14.67)**	1.48 (0.83, 2.66)	1.37 (0.69, 2.79)	1.67 (0.85, 3.34)	1.76 (0.91, 3.59)	1.47 (0.58, 3.68)	0.70 (0.22, 2.15)	1.49 (0.57, 4.02)	1.04 (0.37, 2.89)
SEM60mgIVx3	**2.47 (1.27, 4.71)**	1.59 (0.88, 2.76)	1.49 (0.80, 2.77)	1.70 (0.94, 3.06)	0.81 (0.41, 1.58)	1.15 (0.61, 2.16)	2.19 (0.99, 5.54)	0.98 (0.34, 2.90)	0.77 (0.20, 2.33)	1.12 (0.46, 2.89)	1.31 (0.57, 3.49)	2.47 (0.94, 7.96)	**3.17 (1.21, 10.12)**	**4.04 (1.64, 13.02)**	1.24 (0.71, 2.13)	1.15 (0.58, 2.24)	1.42 (0.72, 2.75)	1.49 (0.75, 2.87)	1.25 (0.49, 3.19)	0.58 (0.19, 1.79)	1.26 (0.50, 3.30)	0.86 (0.32, 2.31)
TOF15mgPO_BID	**2.60 (1.29, 4.97)**	1.67 (0.89, 3.06)	1.57 (0.79, 3.03)	1.79 (0.93, 3.39)	0.85 (0.43, 1.70)	1.21 (0.62, 2.25)	2.33 (0.98, 6.06)	1.02 (0.35, 3.18)	0.82 (0.20, 2.34)	1.19 (0.46, 3.02)	1.38 (0.56, 3.45)	2.54 (0.98, 8.81)	**3.26 (1.28, 10.70)**	**4.16 (1.69, 13.54)**	1.31 (0.71, 2.35)	1.21 (0.60, 2.50)	1.48 (0.74, 3.01)	1.56 (0.77, 3.18)	1.31 (0.49, 3.42)	0.62 (0.19, 1.94)	1.31 (0.50, 3.54)	0.90 (0.32, 2.58)
TOF1mgPO_BID	**3.26 (1.61, 7.03)**	**2.10 (1.12, 4.08)**	1.97 (0.99, 4.11)	**2.26 (1.18, 4.54)**	1.07 (0.52, 2.27)	1.53 (0.77, 3.10)	**2.96 (1.22, 8.05)**	1.29 (0.44, 4.07)	1.05 (0.25, 3.19)	1.50 (0.56, 4.26)	1.76 (0.71, 4.58)	**3.22 (1.23, 11.94)**	**4.15 (1.60, 14.17)**	**5.26 (2.06, 17.98)**	1.64 (0.89, 3.18)	1.55 (0.75, 3.32)	1.86 (0.93, 4.04)	1.97 (0.98, 4.26)	1.65 (0.60, 4.45)	0.78 (0.24, 2.50)	1.69 (0.62, 4.64)	1.16 (0.38, 3.50)
TOF5mgPO_BID	**2.09 (1.08, 4.02)**	1.34 (0.75, 2.34)	1.27 (0.67, 2.31)	1.45 (0.79, 2.60)	0.69 (0.34, 1.32)	0.98 (0.52, 1.79)	1.90 (0.82, 4.75)	0.82 (0.28, 2.47)	0.66 (0.17, 1.89)	0.96 (0.38, 2.48)	1.10 (0.47, 2.77)	2.06 (0.81, 7.09)	**2.66 (1.07, 8.76)**	**3.36 (1.38, 10.93)**	1.06 (0.59, 1.81)	0.99 (0.49, 1.92)	1.20 (0.62, 2.28)	1.27 (0.64, 2.42)	1.06 (0.40, 2.76)	0.49 (0.15, 1.51)	1.07 (0.42, 2.82)	0.74 (0.26, 2.01)
UPA12mgPO_BID	1.96 (0.91, 4.00)	1.26 (0.64, 2.37)	1.18 (0.57, 2.33)	1.36 (0.67, 2.65)	0.65 (0.29, 1.37)	0.91 (0.44, 1.80)	1.77 (0.68, 4.69)	0.78 (0.25, 2.34)	0.61 (0.15, 1.81)	0.89 (0.35, 2.44)	1.03 (0.42, 2.87)	1.91 (0.67, 6.93)	2.47 (0.88, 8.32)	**3.18 (1.17, 10.78)**	1.00 (0.51, 1.82)	0.91 (0.42, 1.91)	1.13 (0.52, 2.34)	1.19 (0.56, 2.41)	0.99 (0.35, 2.62)	0.47 (0.13, 1.52)	1.01 (0.34, 2.78)	0.69 (0.24, 2.07)
UPA24mgPO	1.79 (0.86, 3.64)	1.15 (0.57, 2.16)	1.09 (0.52, 2.12)	1.24 (0.61, 2.35)	0.59 (0.27, 1.22)	0.84 (0.40, 1.62)	1.61 (0.63, 4.25)	0.71 (0.23, 2.17)	0.56 (0.14, 1.72)	0.81 (0.31, 2.21)	0.95 (0.38, 2.51)	1.76 (0.62, 6.20)	2.29 (0.82, 7.78)	**2.91 (1.07, 10.08)**	0.91 (0.46, 1.68)	0.84 (0.40, 1.73)	1.02 (0.49, 2.08)	1.08 (0.52, 2.16)	0.90 (0.34, 2.39)	0.43 (0.13, 1.33)	0.92 (0.31, 2.48)	0.63 (0.21, 1.83)
UPA24mgPO_BID	1.41 (0.68, 2.64)	0.91 (0.47, 1.59)	0.85 (0.43, 1.58)	0.98 (0.50, 1.75)	**0.47 (0.22, 0.93)**	0.66 (0.33, 1.23)	1.27 (0.51, 3.20)	0.56 (0.19, 1.63)	0.44 (0.11, 1.30)	0.65 (0.25, 1.68)	0.74 (0.31, 1.95)	1.38 (0.51, 4.60)	1.79 (0.67, 5.90)	2.30 (0.89, 7.50)	0.71 (0.38, 1.23)	0.66 (0.33, 1.29)	0.80 (0.40, 1.57)	0.85 (0.43, 1.65)	0.71 (0.26, 1.83)	0.34 (0.10, 1.06)	0.73 (0.26, 1.92)	0.50 (0.17, 1.42)
UPA3mgPO_BID	1.88 (0.88, 3.82)	1.21 (0.62, 2.24)	1.15 (0.55, 2.19)	1.31 (0.65, 2.48)	0.62 (0.28, 1.26)	0.88 (0.43, 1.68)	1.70 (0.69, 4.65)	0.75 (0.24, 2.29)	0.59 (0.15, 1.75)	0.86 (0.33, 2.38)	1.00 (0.41, 2.68)	1.86 (0.67, 6.42)	2.39 (0.89, 7.95)	**3.09 (1.17, 10.02)**	0.96 (0.49, 1.70)	0.88 (0.42, 1.74)	1.07 (0.53, 2.14)	1.14 (0.55, 2.24)	0.95 (0.35, 2.52)	0.45 (0.13, 1.38)	0.96 (0.32, 2.60)	0.67 (0.22, 1.89)
UPA6mgPO_BID	1.60 (0.76, 3.25)	1.03 (0.52, 1.86)	0.97 (0.47, 1.87)	1.11 (0.56, 2.04)	0.52 (0.24, 1.09)	0.74 (0.35, 1.45)	1.44 (0.56, 3.78)	0.63 (0.21, 1.85)	0.50 (0.12, 1.45)	0.73 (0.28, 1.98)	0.84 (0.35, 2.28)	1.57 (0.57, 5.37)	2.05 (0.75, 6.73)	2.60 (0.99, 8.44)	0.81 (0.42, 1.44)	0.75 (0.36, 1.50)	0.91 (0.45, 1.83)	0.96 (0.46, 1.94)	0.80 (0.30, 2.14)	0.38 (0.12, 1.18)	0.82 (0.28, 2.20)	0.56 (0.20, 1.62)
UST130mgIV	**1.82 (1.20, 2.93)**	1.18 (0.86, 1.63)	1.11 (0.75, 1.66)	1.27 (0.90, 1.80)	**0.60 (0.37, 0.97)**	0.86 (0.58, 1.26)	1.63 (0.83, 3.80)	0.73 (0.28, 1.98)	0.59 (0.16, 1.47)	0.83 (0.38, 1.92)	0.97 (0.49, 2.24)	1.79 (0.81, 5.51)	**2.30 (1.05, 6.98)**	**2.93 (1.43, 8.48)**	0.93 (0.70, 1.23)	0.86 (0.54, 1.37)	1.04 (0.67, 1.67)	1.11 (0.71, 1.74)	0.93 (0.42, 2.17)	0.44 (0.15, 1.26)	0.94 (0.42, 2.22)	0.65 (0.26, 1.63)
UST4.5mg/kgIV	1.47 (0.62, 3.43)	0.94 (0.41, 2.05)	0.89 (0.36, 2.05)	1.02 (0.44, 2.27)	0.48 (0.20, 1.16)	0.69 (0.29, 1.53)	1.33 (0.50, 3.94)	0.59 (0.17, 2.00)	0.46 (0.11, 1.50)	0.67 (0.23, 2.08)	0.78 (0.29, 2.28)	1.46 (0.50, 5.56)	1.88 (0.66, 6.80)	2.36 (0.85, 8.60)	0.74 (0.33, 1.61)	0.69 (0.28, 1.65)	0.84 (0.35, 1.98)	0.89 (0.37, 2.12)	0.75 (0.25, 2.23)	0.35 (0.09, 1.17)	0.74 (0.24, 2.29)	0.52 (0.16, 1.66)
UST6mg/kgIV	**1.60 (1.06, 2.51)**	1.04 (0.76, 1.41)	0.98 (0.66, 1.46)	1.12 (0.78, 1.59)	**0.53 (0.32, 0.84)**	0.75 (0.50, 1.12)	1.44 (0.74, 3.24)	0.64 (0.24, 1.70)	0.51 (0.14, 1.28)	0.74 (0.34, 1.70)	0.85 (0.43, 1.96)	1.57 (0.72, 4.78)	2.03 (0.93, 6.09)	**2.59 (1.26, 7.55)**	0.81 (0.63, 1.06)	0.76 (0.48, 1.21)	0.92 (0.60, 1.47)	0.97 (0.63, 1.55)	0.81 (0.36, 1.91)	0.39 (0.13, 1.09)	0.82 (0.36, 1.91)	0.56 (0.23, 1.47)
UST90mgSC	**2.59 (1.25, 5.50)**	1.67 (0.85, 3.18)	1.59 (0.77, 3.13)	1.82 (0.92, 3.54)	0.86 (0.40, 1.81)	1.21 (0.59, 2.46)	2.35 (0.93, 6.39)	1.04 (0.30, 3.18)	0.81 (0.20, 2.50)	1.19 (0.46, 3.31)	1.38 (0.57, 3.73)	2.55 (0.98, 9.44)	**3.34 (1.23, 11.53)**	**4.20 (1.65, 14.59)**	1.31 (0.67, 2.50)	1.24 (0.58, 2.62)	1.49 (0.69, 3.15)	1.59 (0.75, 3.36)	1.31 (0.52, 3.57)	0.62 (0.19, 1.93)	1.35 (0.49, 3.65)	0.91 (0.32, 2.69)
VED0.5mg/kgIV	**2.15 (1.17, 3.88)**	1.38 (0.83, 2.28)	1.30 (0.72, 2.27)	1.48 (0.87, 2.53)	0.71 (0.36, 1.31)	1.00 (0.57, 1.75)	1.94 (0.88, 4.61)	0.86 (0.30, 2.42)	0.67 (0.17, 1.94)	0.98 (0.42, 2.45)	1.15 (0.53, 2.86)	2.09 (0.85, 7.26)	**2.73 (1.13, 8.91)**	**3.47 (1.49, 11.08)**	1.08 (0.65, 1.74)	1.02 (0.54, 1.83)	1.22 (0.67, 2.24)	1.29 (0.70, 2.36)	1.10 (0.43, 2.63)	0.51 (0.16, 1.52)	1.11 (0.44, 2.75)	0.76 (0.27, 2.04)
VED2mg/kgIV	**1.98 (1.07, 3.69)**	1.27 (0.76, 2.10)	1.20 (0.67, 2.10)	1.37 (0.80, 2.34)	0.66 (0.34, 1.25)	0.93 (0.53, 1.60)	1.78 (0.82, 4.33)	0.79 (0.26, 2.32)	0.63 (0.16, 1.79)	0.91 (0.40, 2.26)	1.05 (0.49, 2.62)	1.92 (0.80, 6.85)	**2.50 (1.05, 8.46)**	**3.18 (1.37, 10.22)**	1.00 (0.61, 1.60)	0.94 (0.50, 1.71)	1.13 (0.64, 2.09)	1.20 (0.64, 2.21)	1.01 (0.40, 2.52)	0.47 (0.15, 1.44)	1.02 (0.39, 2.60)	0.70 (0.25, 1.90)

	ONT22.5mgSC	ONT225mgSC	ONT75mgSC	PBO	PF10mgSC	PF200mgSC	PF50mgSC	SEM60mgIV	SEM60mgIVx3	TOF15mgPO_BID	TOF1mgPO_BID	TOF5mgPO_BID	UPA12mgPO_BID	UPA24mgPO	UPA24mgPO_BID	UPA3mgPO_BID	UPA6mgPO_BID	UST130mgIV	UST4.5mg/kgIV	UST6mg/kgIV	UST90mgSC	VED0.5mg/kgIV	VED2mg/kgIV
ADA160mg80mg60mgSC	**0.42 (0.22, 0.78)**	**0.39 (0.21, 0.76)**	**0.42 (0.22, 0.80)**	**0.40 (0.26, 0.58)**	0.49 (0.24, 1.05)	0.59 (0.26, 1.25)	0.70 (0.36, 1.40)	**0.34 (0.17, 0.66)**	**0.41 (0.21, 0.79)**	**0.38 (0.20, 0.78)**	**0.31 (0.14, 0.62)**	**0.48 (0.25, 0.92)**	0.51 (0.25, 1.10)	0.56 (0.27, 1.17)	0.71 (0.38, 1.47)	0.53 (0.26, 1.13)	0.62 (0.31, 1.32)	**0.55 (0.34, 0.84)**	0.68 (0.29, 1.61)	**0.62 (0.40, 0.94)**	**0.39 (0.18, 0.80)**	**0.46 (0.26, 0.86)**	**0.51 (0.27, 0.93)**
ADA160mg80mgSC	0.65 (0.38, 1.13)	0.61 (0.37, 1.07)	0.66 (0.37, 1.13)	**0.62 (0.49, 0.79)**	0.76 (0.41, 1.52)	0.93 (0.45, 1.83)	1.09 (0.61, 2.03)	**0.53 (0.29, 0.97)**	0.63 (0.36, 1.13)	0.60 (0.33, 1.12)	**0.48 (0.24, 0.89)**	0.75 (0.43, 1.32)	0.79 (0.42, 1.57)	0.87 (0.46, 1.76)	1.10 (0.63, 2.12)	0.82 (0.45, 1.62)	0.97 (0.54, 1.91)	0.85 (0.61, 1.16)	1.06 (0.49, 2.46)	0.97 (0.71, 1.32)	0.60 (0.31, 1.17)	0.72 (0.44, 1.21)	0.78 (0.48, 1.31)
ADA40mg20mgSC	0.69 (0.37, 1.28)	0.64 (0.36, 1.21)	0.70 (0.38, 1.27)	**0.66 (0.46, 0.93)**	0.81 (0.40, 1.68)	0.98 (0.45, 2.04)	1.16 (0.60, 2.26)	0.56 (0.29, 1.08)	0.67 (0.36, 1.25)	0.64 (0.33, 1.27)	0.51 (0.24, 1.01)	0.79 (0.43, 1.50)	0.84 (0.43, 1.75)	0.92 (0.47, 1.91)	1.17 (0.63, 2.35)	0.87 (0.46, 1.80)	1.03 (0.53, 2.13)	0.90 (0.60, 1.34)	1.13 (0.49, 2.77)	1.02 (0.69, 1.51)	0.63 (0.32, 1.29)	0.77 (0.44, 1.39)	0.83 (0.48, 1.49)
ADA80mg40mgSC	0.61 (0.34, 1.06)	0.57 (0.33, 1.00)	0.61 (0.34, 1.07)	**0.58 (0.43, 0.77)**	0.71 (0.37, 1.43)	0.85 (0.41, 1.70)	1.01 (0.55, 1.92)	**0.49 (0.27, 0.90)**	0.59 (0.33, 1.06)	0.56 (0.30, 1.07)	**0.44 (0.22, 0.85)**	0.69 (0.38, 1.27)	0.74 (0.38, 1.48)	0.80 (0.43, 1.63)	1.02 (0.57, 2.00)	0.76 (0.40, 1.54)	0.90 (0.49, 1.80)	0.79 (0.56, 1.11)	0.98 (0.44, 2.27)	0.89 (0.63, 1.28)	0.55 (0.28, 1.09)	0.67 (0.40, 1.15)	0.73 (0.43, 1.25)
AMI0.4mgPO	1.28 (0.64, 2.42)	1.20 (0.63, 2.29)	1.29 (0.66, 2.40)	1.21 (0.80, 1.89)	1.49 (0.69, 3.23)	1.79 (0.79, 4.00)	**2.13 (1.05, 4.39)**	1.04 (0.52, 2.11)	1.23 (0.63, 2.45)	1.18 (0.59, 2.35)	0.94 (0.44, 1.91)	1.45 (0.76, 2.93)	1.55 (0.73, 3.49)	1.71 (0.82, 3.66)	**2.15 (1.08, 4.49)**	1.60 (0.80, 3.56)	1.90 (0.92, 4.20)	**1.66 (1.03, 2.68)**	2.06 (0.86, 5.10)	**1.87 (1.19, 3.08)**	1.16 (0.55, 2.47)	1.41 (0.76, 2.77)	1.53 (0.80, 2.92)
CDP10mg/kgIV	0.90 (0.49, 1.64)	0.84 (0.48, 1.58)	0.90 (0.50, 1.63)	0.86 (0.61, 1.19)	1.05 (0.54, 2.19)	1.26 (0.59, 2.61)	1.50 (0.81, 2.92)	0.73 (0.38, 1.41)	0.87 (0.46, 1.63)	0.82 (0.44, 1.62)	0.65 (0.32, 1.31)	1.02 (0.56, 1.92)	1.09 (0.56, 2.28)	1.19 (0.62, 2.49)	1.52 (0.81, 3.01)	1.14 (0.60, 2.32)	1.34 (0.69, 2.83)	1.17 (0.79, 1.73)	1.45 (0.65, 3.47)	1.33 (0.89, 1.98)	0.83 (0.41, 1.70)	1.00 (0.57, 1.74)	1.08 (0.63, 1.88)
CER400mgSC	0.46 (0.19, 1.08)	0.44 (0.17, 1.00)	0.47 (0.19, 1.08)	**0.45 (0.21, 0.84)**	0.55 (0.21, 1.41)	0.64 (0.24, 1.66)	0.77 (0.31, 1.88)	**0.38 (0.15, 0.87)**	0.46 (0.18, 1.01)	0.43 (0.16, 1.02)	**0.34 (0.12, 0.82)**	0.53 (0.21, 1.22)	0.56 (0.21, 1.47)	0.62 (0.24, 1.58)	0.79 (0.31, 1.95)	0.59 (0.21, 1.44)	0.70 (0.26, 1.78)	0.61 (0.26, 1.20)	0.75 (0.25, 1.99)	0.69 (0.31, 1.35)	0.43 (0.16, 1.08)	0.52 (0.22, 1.14)	0.56 (0.23, 1.22)
ETA25mgSC	1.07 (0.34, 3.04)	0.99 (0.34, 2.87)	1.05 (0.35, 3.02)	1.01 (0.38, 2.63)	1.26 (0.40, 3.74)	1.51 (0.46, 4.47)	1.79 (0.58, 5.06)	0.87 (0.28, 2.51)	1.02 (0.34, 2.95)	0.98 (0.31, 2.87)	0.77 (0.25, 2.29)	1.22 (0.40, 3.53)	1.29 (0.43, 3.98)	1.41 (0.46, 4.37)	1.78 (0.61, 5.30)	1.34 (0.44, 4.15)	1.58 (0.54, 4.80)	1.37 (0.50, 3.62)	1.70 (0.50, 5.94)	1.55 (0.59, 4.22)	0.96 (0.31, 3.37)	1.17 (0.41, 3.33)	1.26 (0.43, 3.80)
FON0.1mg/kgIV	1.33 (0.46, 5.32)	1.25 (0.45, 4.89)	1.32 (0.47, 5.27)	1.26 (0.48, 4.58)	1.55 (0.49, 6.82)	1.85 (0.58, 8.10)	2.20 (0.74, 9.07)	1.09 (0.37, 4.41)	1.29 (0.43, 5.07)	1.23 (0.43, 4.90)	0.96 (0.31, 3.99)	1.53 (0.53, 6.04)	1.65 (0.55, 6.64)	1.78 (0.58, 7.15)	2.27 (0.77, 9.26)	1.69 (0.57, 6.89)	2.01 (0.69, 8.43)	1.71 (0.68, 6.34)	2.18 (0.66, 9.06)	1.95 (0.78, 7.26)	1.23 (0.40, 5.13)	1.48 (0.52, 5.99)	1.60 (0.56, 6.34)
FON1mg/kgIV	0.92 (0.36, 2.23)	0.86 (0.34, 2.10)	0.92 (0.35, 2.22)	0.88 (0.39, 1.81)	1.06 (0.38, 2.97)	1.28 (0.44, 3.56)	1.51 (0.58, 4.09)	0.74 (0.28, 1.91)	0.89 (0.35, 2.17)	0.84 (0.33, 2.18)	0.67 (0.23, 1.80)	1.04 (0.40, 2.61)	1.13 (0.41, 2.84)	1.23 (0.45, 3.19)	1.54 (0.60, 3.94)	1.17 (0.42, 3.03)	1.36 (0.50, 3.53)	1.20 (0.52, 2.63)	1.48 (0.48, 4.28)	1.36 (0.59, 2.94)	0.84 (0.30, 2.18)	1.02 (0.41, 2.37)	1.10 (0.44, 2.52)
FON4mg/kgIV	0.79 (0.30, 1.78)	0.74 (0.31, 1.70)	0.79 (0.31, 1.79)	0.76 (0.34, 1.45)	0.92 (0.33, 2.31)	1.12 (0.39, 2.76)	1.32 (0.51, 3.15)	0.64 (0.24, 1.52)	0.76 (0.29, 1.75)	0.72 (0.29, 1.80)	0.57 (0.22, 1.40)	0.91 (0.36, 2.11)	0.97 (0.35, 2.41)	1.05 (0.40, 2.64)	1.34 (0.51, 3.24)	1.00 (0.37, 2.46)	1.20 (0.44, 2.85)	1.03 (0.45, 2.02)	1.28 (0.44, 3.50)	1.17 (0.51, 2.31)	0.72 (0.27, 1.76)	0.87 (0.35, 1.88)	0.95 (0.38, 2.04)
INF10mg/kgIV	0.42 (0.13, 1.04)	**0.40 (0.12, 0.97)**	0.43 (0.13, 1.05)	**0.41 (0.13, 0.86)**	0.50 (0.14, 1.36)	0.60 (0.16, 1.60)	0.71 (0.20, 1.79)	**0.35 (0.11, 0.89)**	0.40 (0.13, 1.06)	0.39 (0.11, 1.02)	**0.31 (0.08, 0.81)**	0.49 (0.14, 1.24)	0.52 (0.14, 1.49)	0.57 (0.16, 1.61)	0.72 (0.22, 1.95)	0.54 (0.16, 1.49)	0.64 (0.19, 1.75)	0.56 (0.18, 1.23)	0.69 (0.18, 2.00)	0.64 (0.21, 1.39)	0.39 (0.11, 1.02)	0.48 (0.14, 1.18)	0.52 (0.15, 1.26)
INF20mg/kgIV	**0.33 (0.10, 0.78)**	**0.31 (0.10, 0.73)**	**0.33 (0.11, 0.80)**	**0.32 (0.11, 0.69)**	0.38 (0.11, 1.05)	0.47 (0.13, 1.19)	0.55 (0.17, 1.38)	**0.26 (0.09, 0.69)**	**0.32 (0.10, 0.83)**	**0.31 (0.09, 0.78)**	**0.24 (0.07, 0.62)**	**0.38 (0.11, 0.93)**	0.41 (0.12, 1.13)	0.44 (0.13, 1.22)	0.56 (0.17, 1.49)	0.42 (0.13, 1.12)	0.49 (0.15, 1.33)	**0.44 (0.14, 0.96)**	0.53 (0.15, 1.52)	0.49 (0.16, 1.07)	**0.30 (0.09, 0.81)**	**0.37 (0.11, 0.88)**	**0.40 (0.12, 0.96)**
INF5mg/kgIV	**0.26 (0.08, 0.61)**	**0.24 (0.08, 0.56)**	**0.26 (0.08, 0.60)**	**0.25 (0.09, 0.51)**	**0.30 (0.09, 0.78)**	**0.36 (0.10, 0.91)**	0.43 (0.13, 1.05)	**0.21 (0.07, 0.53)**	**0.25 (0.08, 0.61)**	**0.24 (0.07, 0.59)**	**0.19 (0.06, 0.49)**	**0.30 (0.09, 0.72)**	**0.31 (0.09, 0.85)**	**0.34 (0.10, 0.93)**	0.44 (0.13, 1.13)	**0.32 (0.10, 0.85)**	0.39 (0.12, 1.01)	**0.34 (0.12, 0.70)**	0.42 (0.12, 1.18)	**0.39 (0.13, 0.79)**	**0.24 (0.07, 0.61)**	**0.29 (0.09, 0.67)**	**0.31 (0.10, 0.73)**
NAT300mgIV	0.83 (0.49, 1.42)	0.78 (0.47, 1.34)	0.84 (0.49, 1.40)	**0.79 (0.65, 0.96)**	0.98 (0.53, 1.92)	1.18 (0.56, 2.29)	1.39 (0.78, 2.55)	0.67 (0.38, 1.21)	0.81 (0.47, 1.40)	0.77 (0.43, 1.41)	0.61 (0.31, 1.13)	0.95 (0.55, 1.69)	1.00 (0.55, 1.97)	1.10 (0.60, 2.17)	1.40 (0.81, 2.61)	1.04 (0.59, 2.03)	1.24 (0.69, 2.39)	1.08 (0.82, 1.43)	1.34 (0.62, 3.03)	1.23 (0.94, 1.60)	0.76 (0.40, 1.49)	0.92 (0.58, 1.54)	1.00 (0.62, 1.63)
NAT3mg/kgIV	0.89 (0.46, 1.71)	0.83 (0.45, 1.60)	0.90 (0.46, 1.70)	0.85 (0.55, 1.30)	1.05 (0.51, 2.27)	1.25 (0.55, 2.69)	1.50 (0.76, 3.11)	0.73 (0.36, 1.44)	0.87 (0.45, 1.73)	0.83 (0.40, 1.66)	0.65 (0.30, 1.34)	1.01 (0.52, 2.03)	1.10 (0.52, 2.36)	1.19 (0.58, 2.49)	1.50 (0.77, 3.03)	1.14 (0.57, 2.38)	1.34 (0.66, 2.76)	1.16 (0.73, 1.85)	1.46 (0.61, 3.56)	1.32 (0.83, 2.09)	0.81 (0.38, 1.72)	0.98 (0.55, 1.86)	1.07 (0.58, 2.01)
NAT3mg/kgIVx2	0.73 (0.39, 1.40)	0.69 (0.37, 1.32)	0.74 (0.39, 1.36)	0.70 (0.46, 1.03)	0.86 (0.42, 1.79)	1.03 (0.47, 2.17)	1.24 (0.63, 2.45)	0.60 (0.30, 1.17)	0.71 (0.36, 1.38)	0.68 (0.33, 1.35)	0.54 (0.25, 1.07)	0.84 (0.44, 1.62)	0.89 (0.43, 1.92)	0.98 (0.48, 2.04)	1.25 (0.64, 2.50)	0.94 (0.47, 1.88)	1.10 (0.55, 2.24)	0.96 (0.60, 1.48)	1.19 (0.50, 2.86)	1.09 (0.68, 1.68)	0.67 (0.32, 1.44)	0.82 (0.45, 1.48)	0.88 (0.48, 1.57)
NAT6mg/kgIVx2	0.69 (0.36, 1.33)	0.65 (0.35, 1.23)	0.70 (0.37, 1.31)	**0.66 (0.44, 0.98)**	0.82 (0.40, 1.75)	0.98 (0.44, 2.09)	1.16 (0.58, 2.36)	0.57 (0.28, 1.10)	0.67 (0.35, 1.34)	0.64 (0.31, 1.30)	0.51 (0.23, 1.02)	0.79 (0.41, 1.57)	0.84 (0.42, 1.79)	0.93 (0.46, 1.94)	1.18 (0.61, 2.35)	0.88 (0.45, 1.81)	1.04 (0.52, 2.16)	0.90 (0.57, 1.41)	1.12 (0.47, 2.72)	1.03 (0.65, 1.59)	0.63 (0.30, 1.33)	0.77 (0.42, 1.43)	0.83 (0.45, 1.56)
ONE10mgSC	0.83 (0.33, 2.08)	0.78 (0.29, 1.94)	0.84 (0.31, 2.05)	0.80 (0.36, 1.73)	0.98 (0.35, 2.73)	1.17 (0.41, 3.12)	1.37 (0.53, 3.76)	0.68 (0.27, 1.72)	0.80 (0.31, 2.04)	0.76 (0.29, 2.02)	0.61 (0.22, 1.68)	0.94 (0.36, 2.47)	1.01 (0.38, 2.87)	1.11 (0.42, 2.94)	1.41 (0.55, 3.82)	1.05 (0.40, 2.86)	1.25 (0.47, 3.36)	1.08 (0.46, 2.40)	1.33 (0.45, 3.95)	1.23 (0.52, 2.74)	0.76 (0.28, 1.92)	0.91 (0.38, 2.30)	0.99 (0.40, 2.48)
ONE25mgSC	1.78 (0.58, 5.64)	1.65 (0.52, 5.21)	1.77 (0.55, 5.56)	1.67 (0.62, 4.77)	2.09 (0.63, 7.04)	2.50 (0.74, 8.33)	2.93 (0.95, 9.55)	1.43 (0.47, 4.65)	1.72 (0.56, 5.29)	1.62 (0.52, 5.39)	1.29 (0.40, 4.25)	2.02 (0.66, 6.57)	2.14 (0.66, 7.47)	2.32 (0.75, 7.94)	2.97 (0.95, 10.33)	2.21 (0.72, 7.55)	2.61 (0.85, 8.54)	2.28 (0.79, 6.61)	2.89 (0.86, 10.66)	2.60 (0.92, 7.53)	1.62 (0.52, 5.24)	1.97 (0.66, 6.33)	2.14 (0.70, 6.81)
ONE35mgSC	0.83 (0.32, 1.95)	0.77 (0.30, 1.86)	0.83 (0.31, 2.08)	0.78 (0.35, 1.74)	0.97 (0.35, 2.70)	1.15 (0.39, 3.20)	1.36 (0.53, 3.57)	0.67 (0.25, 1.75)	0.79 (0.30, 2.02)	0.76 (0.28, 2.02)	0.59 (0.22, 1.62)	0.93 (0.36, 2.39)	0.99 (0.36, 2.98)	1.09 (0.40, 3.21)	1.37 (0.52, 3.89)	1.04 (0.38, 3.15)	1.22 (0.46, 3.55)	1.06 (0.45, 2.41)	1.35 (0.44, 4.09)	1.22 (0.52, 2.76)	0.74 (0.27, 2.05)	0.90 (0.36, 2.25)	0.98 (0.38, 2.56)
ONE50mgSC	1.21 (0.43, 3.18)	1.13 (0.40, 3.00)	1.20 (0.42, 3.22)	1.13 (0.46, 2.81)	1.40 (0.49, 4.28)	1.68 (0.53, 5.06)	1.97 (0.71, 5.88)	0.96 (0.35, 2.69)	1.16 (0.43, 3.14)	1.11 (0.39, 3.17)	0.86 (0.29, 2.66)	1.35 (0.50, 3.84)	1.45 (0.48, 4.24)	1.59 (0.55, 4.71)	1.99 (0.70, 6.03)	1.50 (0.53, 4.46)	1.79 (0.62, 5.11)	1.55 (0.61, 3.86)	1.91 (0.60, 6.33)	1.77 (0.68, 4.35)	1.09 (0.37, 3.16)	1.32 (0.49, 3.68)	1.43 (0.53, 3.94)

	ONT22.5mgSC	ONT225mgSC	ONT75mgSC	PBO	PF10mgSC	PF200mgSC	PF50mgSC	SEM60mgIV	SEM60mgIVx3	TOF15mgPO_BID	TOF1mgPO_BID	TOF5mgPO_BID	UPA12mgPO_BID	UPA24mgPO	UPA24mgPO_BID	UPA3mgPO_BID	UPA6mgPO_BID	UST130mgIV
ONT22.5mgSC	ONT22.5mgSC	0.94 (0.59, 1.53)	1.01 (0.62, 1.61)	0.96 (0.59, 1.57)	1.20 (0.53, 2.61)	1.42 (0.62, 3.09)	1.67 (0.81, 3.55)	0.81 (0.39, 1.70)	0.97 (0.48, 1.93)	0.93 (0.43, 2.04)	0.73 (0.31, 1.58)	1.14 (0.55, 2.42)	1.22 (0.55, 2.72)	1.34 (0.61, 2.88)	1.69 (0.81, 3.65)	1.28 (0.60, 2.73)	1.50 (0.70, 3.23)	1.30 (0.76, 2.25)
ONT225mgSC	1.07 (0.65, 1.69)	ONT225mgSC	1.07 (0.66, 1.72)	1.02 (0.61, 1.63)	1.26 (0.57, 2.77)	1.51 (0.63, 3.40)	1.78 (0.86, 3.74)	0.87 (0.42, 1.84)	1.03 (0.50, 2.07)	0.99 (0.45, 2.05)	0.78 (0.34, 1.65)	1.23 (0.58, 2.50)	1.29 (0.60, 2.94)	1.41 (0.66, 3.10)	1.80 (0.89, 3.99)	1.35 (0.64, 2.96)	1.58 (0.74, 3.54)	1.39 (0.80, 2.34)
ONT75mgSC	0.99 (0.62, 1.61)	0.93 (0.58, 1.53)	ONT75mgSC	0.95 (0.58, 1.57)	1.17 (0.55, 2.60)	1.40 (0.61, 3.17)	1.66 (0.80, 3.58)	0.81 (0.40, 1.68)	0.97 (0.47, 1.98)	0.92 (0.43, 1.99)	0.72 (0.33, 1.59)	1.13 (0.55, 2.42)	1.21 (0.56, 2.72)	1.33 (0.62, 2.99)	1.69 (0.81, 3.71)	1.25 (0.61, 2.80)	1.49 (0.70, 3.32)	1.30 (0.76, 2.23)
PBO	1.05 (0.64, 1.69)	0.98 (0.61, 1.64)	1.06 (0.64, 1.73)	PBO	1.22 (0.69, 2.34)	1.48 (0.75, 2.83)	**1.75 (1.03, 3.11)**	0.85 (0.50, 1.49)	1.02 (0.61, 1.72)	0.96 (0.56, 1.71)	0.76 (0.41, 1.39)	1.19 (0.73, 2.06)	1.27 (0.71, 2.45)	1.39 (0.79, 2.71)	**1.77 (1.07, 3.30)**	1.31 (0.76, 2.52)	1.56 (0.91, 2.96)	**1.36 (1.11, 1.69)**
PF10mgSC	0.84 (0.38, 1.87)	0.79 (0.36, 1.77)	0.86 (0.39, 1.83)	0.82 (0.43, 1.45)	PF10mgSC	1.20 (0.63, 2.16)	1.41 (0.84, 2.45)	0.69 (0.30, 1.56)	0.82 (0.36, 1.80)	0.80 (0.34, 1.76)	0.62 (0.26, 1.40)	0.97 (0.43, 2.19)	1.04 (0.42, 2.55)	1.14 (0.47, 2.73)	1.44 (0.61, 3.36)	1.09 (0.44, 2.54)	1.27 (0.54, 3.10)	1.10 (0.57, 2.07)
PF200mgSC	0.71 (0.32, 1.62)	0.66 (0.29, 1.58)	0.71 (0.32, 1.64)	0.67 (0.35, 1.34)	0.83 (0.46, 1.59)	PF200mgSC	1.18 (0.68, 2.17)	0.58 (0.25, 1.40)	0.68 (0.30, 1.64)	0.66 (0.28, 1.57)	0.51 (0.22, 1.22)	0.81 (0.36, 1.93)	0.87 (0.36, 2.22)	0.95 (0.39, 2.38)	1.21 (0.51, 2.95)	0.90 (0.38, 2.25)	1.07 (0.44, 2.73)	0.92 (0.47, 1.89)
PF50mgSC	0.60 (0.28, 1.23)	0.56 (0.27, 1.17)	0.60 (0.28, 1.25)	**0.57 (0.32, 0.97)**	0.71 (0.41, 1.19)	0.85 (0.46, 1.46)	PF50mgSC	0.49 (0.23, 1.07)	0.58 (0.27, 1.25)	0.55 (0.25, 1.24)	**0.43 (0.19, 0.97)**	0.68 (0.32, 1.47)	0.73 (0.31, 1.68)	0.80 (0.34, 1.79)	1.02 (0.46, 2.22)	0.75 (0.34, 1.72)	0.90 (0.40, 2.04)	0.78 (0.42, 1.38)
SEM60mgIV	1.23 (0.59, 2.55)	1.15 (0.54, 2.41)	1.24 (0.60, 2.52)	1.18 (0.67, 2.02)	1.44 (0.64, 3.37)	1.72 (0.72, 4.05)	2.05 (0.94, 4.44)	SEM60mgIV	1.19 (0.68, 2.13)	1.15 (0.51, 2.46)	0.91 (0.39, 1.99)	1.41 (0.66, 2.95)	1.50 (0.65, 3.49)	1.65 (0.74, 3.69)	2.08 (0.98, 4.57)	1.57 (0.70, 3.55)	1.85 (0.83, 4.12)	1.61 (0.88, 2.83)
SEM60mgIVx3	1.03 (0.52, 2.08)	0.97 (0.48, 2.00)	1.04 (0.50, 2.11)	0.98 (0.58, 1.63)	1.22 (0.55, 2.79)	1.46 (0.61, 3.32)	1.72 (0.80, 3.72)	0.84 (0.47, 1.47)	SEM60mgIVx3	0.96 (0.44, 2.06)	0.75 (0.33, 1.63)	1.17 (0.58, 2.42)	1.25 (0.56, 2.85)	1.38 (0.62, 3.06)	1.76 (0.83, 3.76)	1.31 (0.62, 2.91)	1.54 (0.72, 3.41)	1.35 (0.76, 2.30)
TOF15mgPO_BID	1.08 (0.49, 2.32)	1.01 (0.49, 2.20)	1.08 (0.50, 2.33)	1.04 (0.58, 1.80)	1.25 (0.57, 2.95)	1.53 (0.64, 3.53)	1.80 (0.81, 3.97)	0.87 (0.41, 1.95)	1.05 (0.49, 2.26)	TOF15mgPO_BID	0.79 (0.42, 1.44)	1.24 (0.74, 2.10)	1.32 (0.56, 3.03)	1.45 (0.62, 3.43)	1.83 (0.83, 4.14)	1.38 (0.60, 3.02)	1.62 (0.71, 3.75)	1.42 (0.75, 2.56)
TOF1mgPO_BID	1.36 (0.63, 3.20)	1.29 (0.60, 2.94)	1.39 (0.63, 3.03)	1.31 (0.72, 2.45)	1.62 (0.71, 3.83)	1.94 (0.82, 4.65)	**2.31 (1.03, 5.22)**	1.10 (0.50, 2.58)	1.33 (0.61, 3.02)	1.27 (0.69, 2.39)	TOF1mgPO_BID	1.56 (0.90, 2.86)	1.68 (0.71, 3.83)	1.84 (0.79, 4.37)	**2.33 (1.07, 5.38)**	1.75 (0.76, 3.99)	2.07 (0.89, 4.79)	1.79 (0.98, 3.49)
TOF5mgPO_BID	0.88 (0.41, 1.82)	0.81 (0.40, 1.73)	0.88 (0.41, 1.81)	0.84 (0.49, 1.37)	1.03 (0.46, 2.32)	1.24 (0.52, 2.74)	1.48 (0.68, 3.10)	0.71 (0.34, 1.51)	0.85 (0.41, 1.74)	0.81 (0.48, 1.35)	0.64 (0.35, 1.12)	TOF5mgPO_BID	1.08 (0.46, 2.34)	1.18 (0.52, 2.57)	1.49 (0.69, 3.17)	1.11 (0.50, 2.39)	1.31 (0.59, 2.85)	1.14 (0.64, 1.92)
UPA12mgPO_BID	0.82 (0.37, 1.83)	0.78 (0.34, 1.67)	0.83 (0.37, 1.79)	0.79 (0.41, 1.41)	0.96 (0.39, 2.37)	1.16 (0.45, 2.74)	1.38 (0.59, 3.19)	0.67 (0.29, 1.53)	0.80 (0.35, 1.77)	0.76 (0.33, 1.78)	0.59 (0.26, 1.42)	0.93 (0.43, 2.15)	UPA12mgPO_BID	1.09 (0.63, 1.91)	1.39 (0.84, 2.30)	1.04 (0.60, 1.79)	1.22 (0.73, 2.08)	1.07 (0.55, 1.99)
UPA24mgPO	0.74 (0.35, 1.63)	0.71 (0.32, 1.51)	0.75 (0.33, 1.62)	0.72 (0.37, 1.26)	0.88 (0.37, 2.12)	1.05 (0.42, 2.54)	1.25 (0.56, 2.92)	0.61 (0.27, 1.36)	0.72 (0.33, 1.61)	0.69 (0.29, 1.62)	0.54 (0.23, 1.26)	0.85 (0.39, 1.94)	0.91 (0.52, 1.58)	UPA24mgPO	1.26 (0.78, 2.09)	0.94 (0.56, 1.64)	1.12 (0.67, 1.87)	0.99 (0.49, 1.79)
UPA24mgPO_BID	0.59 (0.27, 1.24)	0.56 (0.25, 1.13)	0.59 (0.27, 1.24)	**0.56 (0.30, 0.93)**	0.69 (0.30, 1.64)	0.83 (0.34, 1.94)	0.98 (0.45, 2.18)	0.48 (0.22, 1.02)	0.57 (0.27, 1.21)	0.55 (0.24, 1.20)	**0.43 (0.19, 0.94)**	0.67 (0.32, 1.45)	0.72 (0.43, 1.20)	0.79 (0.48, 1.28)	UPA24mgPO_BID	0.75 (0.46, 1.20)	0.88 (0.56, 1.40)	0.77 (0.40, 1.32)
UPA3mgPO_BID	0.78 (0.37, 1.66)	0.74 (0.34, 1.55)	0.80 (0.36, 1.64)	0.76 (0.40, 1.32)	0.92 (0.39, 2.30)	1.11 (0.45, 2.63)	1.33 (0.58, 2.97)	0.64 (0.28, 1.43)	0.76 (0.34, 1.63)	0.72 (0.33, 1.66)	0.57 (0.25, 1.32)	0.90 (0.42, 2.01)	0.96 (0.56, 1.66)	1.06 (0.61, 1.78)	1.34 (0.83, 2.16)	UPA3mgPO_BID	1.18 (0.71, 1.97)	1.03 (0.52, 1.84)
UPA6mgPO_BID	0.67 (0.31, 1.44)	0.63 (0.28, 1.35)	0.67 (0.30, 1.44)	0.64 (0.34, 1.10)	0.79 (0.32, 1.84)	0.93 (0.37, 2.29)	1.11 (0.49, 2.51)	0.54 (0.24, 1.21)	0.65 (0.29, 1.38)	0.62 (0.27, 1.41)	0.48 (0.21, 1.13)	0.76 (0.35, 1.70)	0.82 (0.48, 1.38)	0.89 (0.53, 1.48)	1.13 (0.71, 1.79)	0.85 (0.51, 1.40)	UPA6mgPO_BID	0.87 (0.45, 1.53)
UST130mgIV	0.77 (0.44, 1.31)	0.72 (0.43, 1.26)	0.77 (0.45, 1.32)	**0.73 (0.59, 0.90)**	0.91 (0.48, 1.76)	1.09 (0.53, 2.13)	1.28 (0.72, 2.38)	0.62 (0.35, 1.13)	0.74 (0.43, 1.31)	0.71 (0.39, 1.33)	0.56 (0.29, 1.02)	0.88 (0.52, 1.56)	0.93 (0.50, 1.83)	1.01 (0.56, 2.05)	1.30 (0.76, 2.52)	0.97 (0.54, 1.92)	1.14 (0.65, 2.22)	UST130mgIV
UST4.5mg/kgIV	0.63 (0.25, 1.45)	0.58 (0.22, 1.41)	0.63 (0.23, 1.51)	0.59 (0.27, 1.25)	0.73 (0.27, 1.89)	0.86 (0.31, 2.32)	1.04 (0.39, 2.60)	0.50 (0.19, 1.24)	0.60 (0.24, 1.49)	0.57 (0.22, 1.46)	0.45 (0.17, 1.13)	0.71 (0.28, 1.76)	0.75 (0.28, 1.90)	0.83 (0.31, 2.12)	1.04 (0.41, 2.67)	0.77 (0.31, 2.07)	0.93 (0.36, 2.35)	0.81 (0.33, 1.75)
UST6mg/kgIV	0.68 (0.39, 1.16)	0.63 (0.37, 1.09)	0.68 (0.39, 1.17)	**0.65 (0.53, 0.78)**	0.79 (0.43, 1.55)	0.95 (0.46, 1.86)	1.13 (0.64, 2.09)	**0.55 (0.31, 0.99)**	0.65 (0.38, 1.15)	0.62 (0.34, 1.17)	**0.49 (0.25, 0.91)**	0.77 (0.45, 1.37)	0.81 (0.44, 1.61)	0.89 (0.49, 1.78)	1.14 (0.66, 2.20)	0.85 (0.47, 1.69)	1.01 (0.56, 1.96)	0.88 (0.73, 1.06)
UST90mgSC	1.10 (0.49, 2.41)	1.02 (0.47, 2.28)	1.10 (0.49, 2.41)	1.04 (0.55, 1.92)	1.29 (0.53, 3.13)	1.56 (0.61, 3.73)	1.84 (0.80, 4.21)	0.89 (0.38, 2.11)	1.06 (0.46, 2.43)	1.01 (0.42, 2.41)	0.79 (0.34, 1.84)	1.24 (0.54, 2.86)	1.34 (0.57, 3.26)	1.46 (0.63, 3.58)	1.85 (0.84, 4.39)	1.38 (0.60, 3.34)	1.62 (0.73, 3.96)	1.43 (0.72, 2.75)
VED0.5mg/kgIV	0.90 (0.45, 1.74)	0.84 (0.44, 1.68)	0.91 (0.46, 1.77)	0.86 (0.54, 1.35)	1.05 (0.50, 2.36)	1.26 (0.58, 2.77)	1.50 (0.77, 3.12)	0.74 (0.35, 1.50)	0.87 (0.43, 1.75)	0.83 (0.40, 1.70)	0.65 (0.31, 1.36)	1.02 (0.53, 2.07)	1.09 (0.54, 2.42)	1.20 (0.57, 2.57)	1.52 (0.77, 3.16)	1.14 (0.55, 2.35)	1.35 (0.67, 2.88)	1.17 (0.71, 1.90)
VED2mg/kgIV	0.83 (0.42, 1.65)	0.77 (0.41, 1.57)	0.84 (0.42, 1.64)	0.79 (0.50, 1.22)	0.97 (0.46, 2.22)	1.17 (0.53, 2.61)	1.38 (0.70, 2.92)	0.68 (0.33, 1.38)	0.80 (0.41, 1.61)	0.76 (0.38, 1.57)	0.61 (0.28, 1.25)	0.95 (0.48, 1.89)	1.02 (0.49, 2.28)	1.11 (0.53, 2.43)	1.40 (0.71, 2.93)	1.05 (0.50, 2.25)	1.24 (0.60, 2.67)	1.08 (0.65, 1.77)

Table S12G: League table for clinical responses at 4 weeks of treatment.

	ADA160mg80mg60mgSC	ADA160mg80mgSC	ADA40mg20mgSC	ADA80mg40mgSC	API100mgPO	API50mgPO	CDP10mg/kgIV	CER100mgSC	CER10mgIV	CER200mgSC	CER20mgIV	CER400mgSC	CER5mgIV	ETA25mgSC	FON0.1mg/kgIV	FON1mg/kg0.1mgIV/SC	FON1mg/kg1mgIV/SC	FON1mg/kgIV	FON4mg/kg0.1mgIV/SC	FON4mg/kg1mgIV/SC
ADA160mg80mg60mgSC	ADA160mg80mg60mgSC	0.47 (0.15, 1.20)	**0.25 (0.07, 0.81)**	0.41 (0.13, 1.22)	**0.13 (0.04, 0.45)**	**0.12 (0.03, 0.39)**	**0.23 (0.07, 0.66)**	0.35 (0.10, 1.18)	0.41 (0.10, 1.69)	**0.30 (0.09, 0.97)**	0.25 (0.06, 1.13)	**0.29 (0.10, 0.82)**	0.35 (0.09, 1.41)	**0.07 (0.01, 0.45)**	**0.06 (0.00, 0.72)**	**0.17 (0.04, 0.71)**	**0.20 (0.05, 0.79)**	**0.14 (0.02, 0.88)**	**0.20 (0.05, 0.83)**	**0.04 (0.00, 0.26)**
ADA160mg80mgSC	2.14 (0.83, 6.69)	ADA160mg80mgSC	0.55 (0.26, 1.12)	0.90 (0.53, 1.60)	**0.28 (0.10, 0.78)**	**0.26 (0.09, 0.67)**	0.49 (0.21, 1.13)	0.76 (0.30, 1.97)	0.86 (0.28, 3.21)	0.65 (0.25, 1.73)	0.56 (0.16, 2.12)	0.61 (0.31, 1.36)	0.74 (0.24, 2.57)	**0.14 (0.02, 0.86)**	0.14 (0.00, 1.27)	0.37 (0.11, 1.27)	0.44 (0.13, 1.46)	0.32 (0.06, 1.77)	0.44 (0.14, 1.43)	**0.09 (0.01, 0.48)**
ADA40mg20mgSC	**3.96 (1.24, 13.52)**	1.82 (0.89, 3.86)	ADA40mg20mgSC	1.62 (0.79, 3.73)	0.52 (0.16, 1.57)	0.47 (0.14, 1.45)	0.90 (0.32, 2.53)	1.38 (0.46, 4.35)	1.57 (0.46, 6.54)	1.18 (0.38, 3.83)	0.99 (0.26, 4.33)	1.13 (0.45, 2.93)	1.36 (0.38, 5.53)	0.26 (0.03, 1.79)	0.26 (0.00, 2.47)	0.68 (0.18, 2.67)	0.79 (0.22, 3.13)	0.58 (0.10, 3.40)	0.79 (0.21, 3.13)	**0.17 (0.02, 0.97)**
ADA80mg40mgSC	2.41 (0.82, 7.44)	1.11 (0.63, 1.88)	0.62 (0.27, 1.27)	ADA80mg40mgSC	**0.32 (0.10, 0.92)**	**0.29 (0.09, 0.80)**	0.55 (0.21, 1.37)	0.85 (0.31, 2.34)	0.96 (0.29, 3.55)	0.72 (0.26, 2.04)	0.62 (0.17, 2.40)	0.68 (0.32, 1.60)	0.83 (0.24, 2.94)	**0.16 (0.02, 0.95)**	0.16 (0.00, 1.55)	0.41 (0.11, 1.50)	0.49 (0.14, 1.69)	0.35 (0.06, 2.02)	0.49 (0.14, 1.64)	**0.11 (0.01, 0.56)**
API100mgPO	**7.69 (2.22, 27.60)**	**3.51 (1.28, 10.15)**	1.92 (0.64, 6.29)	**3.16 (1.08, 9.55)**	API100mgPO	0.90 (0.35, 2.28)	1.74 (0.59, 5.32)	2.64 (0.88, 9.12)	3.06 (0.81, 13.71)	2.28 (0.73, 7.74)	2.00 (0.46, 8.65)	2.17 (0.81, 6.10)	2.68 (0.70, 11.17)	0.51 (0.05, 3.42)	0.51 (0.01, 5.16)	1.32 (0.33, 5.37)	1.53 (0.40, 6.14)	1.10 (0.18, 7.27)	1.54 (0.40, 6.29)	0.33 (0.04, 1.83)
API50mgPO	**8.35 (2.57, 31.91)**	**3.86 (1.49, 11.22)**	2.14 (0.69, 7.00)	**3.50 (1.24, 10.59)**	1.11 (0.44, 2.87)	API50mgPO	1.91 (0.67, 5.89)	2.96 (0.98, 9.73)	3.41 (0.89, 15.19)	2.52 (0.84, 8.73)	2.19 (0.53, 10.20)	2.40 (0.92, 6.83)	2.87 (0.78, 12.71)	0.56 (0.06, 4.03)	0.55 (0.01, 5.50)	1.46 (0.37, 6.28)	1.71 (0.46, 7.12)	1.23 (0.21, 7.59)	1.71 (0.45, 7.34)	0.36 (0.04, 2.20)
CDP10mg/kgIV	**4.36 (1.52, 14.12)**	2.04 (0.89, 4.67)	1.11 (0.40, 3.15)	1.81 (0.73, 4.70)	0.58 (0.19, 1.69)	0.52 (0.17, 1.49)	CDP10mg/kgIV	1.53 (0.56, 4.45)	1.74 (0.49, 6.97)	1.32 (0.48, 3.76)	1.13 (0.30, 4.64)	1.24 (0.55, 3.01)	1.51 (0.45, 5.69)	0.29 (0.04, 1.81)	0.29 (0.01, 2.66)	0.75 (0.22, 2.81)	0.89 (0.26, 3.05)	0.63 (0.12, 3.60)	0.90 (0.26, 3.10)	0.19 (0.02, 1.02)
CER100mgSC	2.84 (0.85, 9.89)	1.32 (0.51, 3.31)	0.72 (0.23, 2.18)	1.18 (0.43, 3.25)	0.38 (0.11, 1.14)	0.34 (0.10, 1.02)	0.65 (0.22, 1.79)	CER100mgSC	1.14 (0.32, 4.50)	0.86 (0.39, 1.82)	0.73 (0.18, 3.11)	0.81 (0.41, 1.70)	0.98 (0.28, 3.60)	0.19 (0.02, 1.19)	0.19 (0.00, 1.77)	0.50 (0.13, 1.82)	0.58 (0.16, 2.13)	0.42 (0.07, 2.46)	0.58 (0.16, 2.10)	**0.12 (0.01, 0.70)**
CER10mgIV	2.44 (0.59, 10.16)	1.16 (0.31, 3.63)	0.64 (0.15, 2.19)	1.04 (0.28, 3.50)	0.33 (0.07, 1.23)	0.29 (0.07, 1.12)	0.57 (0.14, 2.03)	0.88 (0.22, 3.14)	CER10mgIV	0.75 (0.19, 2.75)	0.65 (0.24, 1.62)	0.70 (0.21, 2.37)	0.85 (0.36, 2.04)	0.16 (0.02, 1.17)	0.16 (0.00, 1.78)	0.43 (0.09, 1.89)	0.50 (0.11, 2.24)	0.36 (0.05, 2.49)	0.50 (0.11, 2.25)	**0.10 (0.01, 0.68)**
CER200mgSC	**3.32 (1.03, 11.49)**	1.53 (0.58, 3.99)	0.84 (0.26, 2.60)	1.40 (0.49, 3.84)	0.44 (0.13, 1.38)	0.40 (0.11, 1.19)	0.76 (0.27, 2.09)	1.17 (0.55, 2.54)	1.33 (0.36, 5.14)	CER200mgSC	0.87 (0.22, 3.44)	0.95 (0.45, 2.04)	1.13 (0.32, 4.30)	0.22 (0.02, 1.46)	0.22 (0.00, 2.07)	0.58 (0.15, 2.17)	0.67 (0.19, 2.62)	0.48 (0.08, 2.79)	0.67 (0.17, 2.45)	**0.14 (0.02, 0.78)**
CER20mgIV	3.98 (0.88, 17.18)	1.79 (0.47, 6.17)	1.01 (0.23, 3.79)	1.62 (0.42, 5.89)	0.50 (0.12, 2.16)	0.46 (0.10, 1.88)	0.89 (0.22, 3.32)	1.36 (0.32, 5.42)	1.55 (0.62, 4.24)	1.16 (0.29, 4.61)	CER20mgIV	1.11 (0.32, 3.96)	1.32 (0.55, 3.61)	0.25 (0.02, 1.96)	0.26 (0.00, 3.42)	0.67 (0.14, 3.30)	0.77 (0.16, 3.77)	0.57 (0.08, 3.98)	0.78 (0.16, 3.91)	0.17 (0.01, 1.19)
CER400mgSC	**3.50 (1.22, 10.14)**	1.64 (0.74, 3.20)	0.89 (0.34, 2.21)	1.46 (0.63, 3.16)	0.46 (0.16, 1.23)	0.42 (0.15, 1.09)	0.81 (0.33, 1.81)	1.23 (0.59, 2.46)	1.42 (0.42, 4.86)	1.05 (0.49, 2.22)	0.90 (0.25, 3.11)	CER400mgSC	1.21 (0.38, 3.87)	0.23 (0.03, 1.36)	0.23 (0.00, 2.07)	0.60 (0.18, 1.97)	0.71 (0.22, 2.29)	0.52 (0.09, 2.77)	0.70 (0.21, 2.21)	**0.15 (0.02, 0.76)**
CER5mgIV	2.85 (0.71, 11.61)	1.35 (0.39, 4.10)	0.73 (0.18, 2.62)	1.20 (0.34, 4.09)	0.37 (0.09, 1.44)	0.35 (0.08, 1.28)	0.66 (0.18, 2.25)	1.02 (0.28, 3.56)	1.18 (0.49, 2.76)	0.88 (0.23, 3.12)	0.76 (0.28, 1.83)	0.83 (0.26, 2.64)	CER5mgIV	0.19 (0.02, 1.32)	0.19 (0.00, 2.10)	0.51 (0.11, 2.13)	0.58 (0.13, 2.62)	0.42 (0.06, 2.88)	0.58 (0.13, 2.57)	**0.12 (0.01, 0.79)**
ETA25mgSC	**15.07 (2.25, 133.63)**	**6.97 (1.16, 55.32)**	3.85 (0.56, 32.90)	**6.17 (1.05, 51.66)**	1.98 (0.29, 18.51)	1.79 (0.25, 15.80)	3.44 (0.55, 27.03)	5.28 (0.84, 44.04)	6.20 (0.85, 62.90)	4.47 (0.68, 40.77)	3.99 (0.51, 42.01)	4.36 (0.73, 35.62)	5.35 (0.76, 54.95)	ETA25mgSC	0.98 (0.01, 16.39)	2.60 (0.34, 26.62)	3.06 (0.41, 30.45)	2.23 (0.21, 27.98)	3.01 (0.39, 32.33)	0.66 (0.05, 7.68)
FON0.1mg/kgIV	**15.44 (1.39, 1246.78)**	6.95 (0.79, 382.01)	3.89 (0.41, 241.00)	6.18 (0.64, 349.07)	1.96 (0.19, 109.99)	1.82 (0.18, 109.17)	3.43 (0.38, 196.39)	5.29 (0.56, 355.16)	6.10 (0.56, 393.73)	4.60 (0.48, 315.25)	3.92 (0.29, 252.45)	4.30 (0.48, 261.71)	5.25 (0.48, 342.56)	1.02 (0.06, 86.29)	FON0.1mg/kgIV	2.59 (0.25, 170.30)	3.07 (0.28, 219.51)	2.14 (0.26, 135.58)	3.11 (0.31, 234.05)	0.65 (0.03, 46.35)
FON1mg/kg0.1mgIV/SC	**5.85 (1.41, 24.22)**	2.72 (0.79, 8.76)	1.48 (0.37, 5.43)	2.44 (0.67, 8.77)	0.76 (0.19, 3.02)	0.69 (0.16, 2.69)	1.34 (0.36, 4.62)	2.01 (0.55, 7.78)	2.32 (0.53, 11.39)	1.71 (0.46, 6.83)	1.49 (0.30, 7.22)	1.66 (0.51, 5.71)	1.98 (0.47, 9.28)	0.38 (0.04, 2.98)	0.39 (0.01, 4.06)	FON1mg/kg0.1mgIV/SC	1.16 (0.42, 3.33)	0.83 (0.12, 5.86)	1.17 (0.40, 3.44)	0.25 (0.03, 1.09)
FON1mg/kg1mgIV/SC	**4.98 (1.27, 19.88)**	2.29 (0.69, 7.44)	1.26 (0.32, 4.63)	2.05 (0.59, 7.03)	0.65 (0.16, 2.50)	0.58 (0.14, 2.19)	1.12 (0.33, 3.84)	1.72 (0.47, 6.19)	2.00 (0.45, 9.27)	1.49 (0.38, 5.37)	1.29 (0.27, 6.19)	1.41 (0.44, 4.45)	1.71 (0.38, 7.82)	0.33 (0.03, 2.42)	0.33 (0.00, 3.55)	0.86 (0.30, 2.38)	FON1mg/kg1mgIV/SC	0.72 (0.10, 4.63)	1.01 (0.36, 2.80)	**0.21 (0.03, 0.93)**
FON1mg/kgIV	**6.97 (1.13, 45.58)**	3.17 (0.56, 17.53)	1.73 (0.29, 10.11)	2.84 (0.49, 15.96)	0.91 (0.14, 5.43)	0.81 (0.13, 4.72)	1.58 (0.28, 8.63)	2.39 (0.41, 14.77)	2.78 (0.40, 20.12)	2.06 (0.36, 13.10)	1.76 (0.25, 12.87)	1.93 (0.36, 11.08)	2.38 (0.35, 16.94)	0.45 (0.04, 4.73)	0.47 (0.01, 3.80)	1.20 (0.17, 8.15)	1.38 (0.22, 9.87)	FON1mg/kgIV	1.38 (0.22, 9.26)	0.30 (0.02, 2.69)
FON4mg/kg0.1mgIV/SC	**5.00 (1.20, 20.17)**	2.26 (0.70, 7.18)	1.27 (0.32, 4.69)	2.04 (0.61, 7.19)	0.65 (0.16, 2.50)	0.59 (0.14, 2.23)	1.11 (0.32, 3.89)	1.73 (0.48, 6.33)	1.99 (0.44, 9.29)	1.48 (0.41, 5.82)	1.29 (0.26, 6.29)	1.42 (0.45, 4.81)	1.74 (0.39, 7.81)	0.33 (0.03, 2.57)	0.32 (0.00, 3.19)	0.85 (0.29, 2.49)	0.99 (0.36, 2.79)	0.72 (0.11, 4.64)	FON4mg/kg0.1mgIV/SC	**0.21 (0.03, 0.93)**
FON4mg/kg1mgIV/SC	**23.53 (3.88, 223.09)**	**10.67 (2.09, 90.48)**	**5.81 (1.03, 51.68)**	**9.52 (1.79, 84.10)**	3.06 (0.54, 27.54)	2.80 (0.45, 23.37)	5.37 (0.98, 46.63)	**8.15 (1.42, 77.02)**	**9.53 (1.46, 101.16)**	**7.05 (1.28, 65.43)**	6.01 (0.84, 71.06)	**6.58 (1.31, 57.11)**	**8.29 (1.26, 83.14)**	1.52 (0.13, 20.44)	1.53 (0.02, 32.10)	3.98 (0.92, 32.23)	**4.66 (1.08, 36.23)**	3.32 (0.37, 50.40)	**4.70 (1.08, 37.58)**	FON4mg/kg1mgIV/SC

(TABLE S12G. continued)

	ADA160mg80mg60mgSC	ADA160mg80mgSC	ADA40mg20mgSC	ADA80mg40mgSC	API100mgPO	API50mgPO	CDP10mg/kgIV	CER100mgSC	CER10mgIV	CER200mgSC	CER20mgIV	CER400mgSC	CER5mgIV	ETA25mgSC	FON0.1mg/kgIV	FON1mg/kg0.1mgIV/SC	FON1mg/kg1mgIV/SC	FON1mg/kgIV	FON4mg/kg0.1mgIV/SC	FON4mg/kg1mgIV/SC
FON4mg/kgIV	3.63 (0.63, 16.92)	1.67 (0.33, 5.99)	0.91 (0.17, 3.80)	1.50 (0.30, 5.68)	0.47 (0.08, 2.02)	0.42 (0.08, 1.79)	0.82 (0.16, 3.16)	1.25 (0.23, 5.40)	1.44 (0.24, 7.73)	1.07 (0.19, 4.78)	0.91 (0.14, 5.18)	1.02 (0.22, 3.85)	1.24 (0.20, 6.39)	0.23 (0.02, 1.86)	0.24 (0.00, 1.57)	0.62 (0.10, 3.04)	0.73 (0.12, 3.51)	0.53 (0.11, 1.73)	0.72 (0.12, 3.38)	0.15 (0.01, 1.14)
GUS1200mgIV	3.39 (0.78, 13.42)	1.57 (0.49, 4.49)	0.86 (0.24, 2.87)	1.39 (0.42, 4.43)	0.44 (0.11, 1.62)	0.40 (0.10, 1.43)	0.78 (0.22, 2.38)	1.17 (0.33, 3.98)	1.36 (0.32, 6.09)	1.01 (0.28, 3.69)	0.88 (0.16, 3.98)	0.97 (0.30, 2.99)	1.16 (0.27, 5.00)	0.22 (0.02, 1.61)	0.23 (0.00, 2.34)	0.58 (0.13, 2.58)	0.68 (0.16, 2.96)	0.48 (0.07, 3.33)	0.69 (0.15, 2.82)	**0.14 (0.02, 0.90)**
GUS200mgIV	3.46 (0.86, 13.27)	1.57 (0.49, 4.74)	0.85 (0.24, 3.04)	1.40 (0.43, 4.62)	0.44 (0.11, 1.66)	0.40 (0.10, 1.53)	0.78 (0.23, 2.46)	1.17 (0.33, 4.39)	1.37 (0.31, 6.27)	1.01 (0.28, 3.79)	0.88 (0.19, 4.13)	0.97 (0.31, 3.00)	1.16 (0.28, 5.12)	0.23 (0.02, 1.55)	0.22 (0.00, 2.26)	0.59 (0.14, 2.49)	0.68 (0.16, 2.89)	0.50 (0.07, 3.19)	0.70 (0.16, 2.86)	**0.14 (0.02, 0.89)**
GUS600mgIV	3.22 (0.81, 12.51)	1.48 (0.48, 4.44)	0.81 (0.23, 2.73)	1.32 (0.41, 4.33)	0.42 (0.11, 1.52)	0.38 (0.09, 1.42)	0.73 (0.22, 2.35)	1.11 (0.31, 4.04)	1.28 (0.32, 5.69)	0.95 (0.27, 3.60)	0.82 (0.17, 3.89)	0.90 (0.30, 2.85)	1.10 (0.28, 4.77)	0.21 (0.02, 1.56)	0.21 (0.00, 2.07)	0.55 (0.12, 2.47)	0.65 (0.15, 2.77)	0.47 (0.07, 3.19)	0.65 (0.15, 2.82)	**0.14 (0.01, 0.91)**
INF10mg/kgIV	0.70 (0.03, 5.84)	0.33 (0.02, 2.31)	0.17 (0.01, 1.43)	0.29 (0.01, 2.25)	**0.09 (0.00, 0.73)**	**0.08 (0.00, 0.67)**	0.16 (0.01, 1.15)	0.24 (0.01, 1.86)	0.27 (0.01, 2.65)	0.21 (0.01, 1.59)	0.18 (0.01, 1.84)	0.20 (0.01, 1.38)	0.23 (0.01, 2.24)	**0.04 (0.00, 0.58)**	**0.04 (0.00, 0.75)**	0.12 (0.00, 1.18)	0.14 (0.01, 1.28)	0.10 (0.00, 1.20)	0.14 (0.01, 1.39)	**0.03 (0.00, 0.34)**
INF20mg/kgIV	0.68 (0.04, 5.80)	0.31 (0.02, 2.20)	0.17 (0.01, 1.33)	0.28 (0.02, 2.10)	**0.09 (0.00, 0.76)**	**0.08 (0.00, 0.65)**	0.16 (0.01, 1.15)	0.23 (0.01, 1.82)	0.27 (0.01, 2.49)	0.20 (0.01, 1.62)	0.18 (0.01, 1.73)	0.19 (0.01, 1.38)	0.23 (0.01, 2.24)	**0.04 (0.00, 0.55)**	**0.04 (0.00, 0.75)**	0.12 (0.00, 1.19)	0.13 (0.01, 1.32)	0.09 (0.00, 1.33)	0.14 (0.01, 1.18)	**0.03 (0.00, 0.35)**
INF5mg/kgIV	0.35 (0.02, 2.76)	0.16 (0.01, 1.08)	**0.09 (0.00, 0.64)**	0.14 (0.01, 1.00)	**0.05 (0.00, 0.35)**	**0.04 (0.00, 0.31)**	**0.08 (0.00, 0.54)**	**0.12 (0.01, 0.88)**	0.13 (0.01, 1.29)	**0.10 (0.01, 0.78)**	**0.09 (0.00, 0.84)**	**0.10 (0.01, 0.67)**	0.12 (0.01, 1.05)	**0.02 (0.00, 0.28)**	**0.02 (0.00, 0.36)**	**0.06 (0.00, 0.55)**	**0.07 (0.00, 0.61)**	**0.05 (0.00, 0.62)**	**0.07 (0.00, 0.58)**	**0.01 (0.00, 0.18)**
NAT300mgIV	**4.24 (1.66, 12.76)**	1.98 (0.99, 3.81)	1.10 (0.44, 2.67)	1.79 (0.83, 3.86)	0.56 (0.21, 1.45)	0.51 (0.19, 1.25)	0.97 (0.44, 2.20)	1.48 (0.61, 3.83)	1.71 (0.57, 6.10)	1.28 (0.53, 3.44)	1.11 (0.33, 3.93)	1.21 (0.67, 2.52)	1.46 (0.50, 4.97)	0.29 (0.03, 1.64)	0.28 (0.00, 2.49)	0.72 (0.23, 2.39)	0.87 (0.28, 2.77)	0.63 (0.12, 3.30)	0.87 (0.28, 2.73)	**0.18 (0.02, 0.92)**
NAT3mg/kgIV	2.64 (0.79, 9.46)	1.24 (0.44, 3.48)	0.68 (0.20, 2.27)	1.12 (0.37, 3.40)	0.35 (0.09, 1.23)	0.32 (0.09, 1.10)	0.60 (0.21, 1.93)	0.93 (0.30, 3.02)	1.09 (0.26, 4.25)	0.79 (0.25, 2.64)	0.69 (0.16, 2.92)	0.76 (0.29, 2.26)	0.92 (0.24, 3.67)	0.17 (0.02, 1.21)	0.18 (0.00, 1.94)	0.46 (0.11, 2.07)	0.54 (0.14, 2.34)	0.38 (0.06, 2.48)	0.55 (0.14, 2.10)	**0.11 (0.01, 0.77)**
NAT3mg/kgIVx2	2.80 (0.84, 10.50)	1.32 (0.46, 3.47)	0.71 (0.21, 2.30)	1.18 (0.39, 3.47)	0.37 (0.10, 1.27)	0.34 (0.10, 1.11)	0.65 (0.21, 1.90)	0.98 (0.31, 3.24)	1.15 (0.28, 4.44)	0.84 (0.25, 2.84)	0.74 (0.17, 3.05)	0.81 (0.30, 2.17)	0.99 (0.26, 3.82)	0.19 (0.02, 1.24)	0.19 (0.00, 2.06)	0.48 (0.12, 2.14)	0.57 (0.15, 2.22)	0.40 (0.07, 2.56)	0.57 (0.14, 2.41)	**0.12 (0.01, 0.73)**
NAT6mg/kgIVx2	2.79 (0.82, 10.75)	1.31 (0.46, 3.57)	0.71 (0.22, 2.33)	1.18 (0.38, 3.57)	0.37 (0.10, 1.26)	0.33 (0.09, 1.13)	0.64 (0.21, 1.94)	0.98 (0.31, 3.30)	1.15 (0.29, 4.59)	0.83 (0.26, 2.94)	0.73 (0.16, 3.16)	0.80 (0.29, 2.24)	0.97 (0.24, 3.90)	0.18 (0.02, 1.21)	0.18 (0.00, 2.06)	0.48 (0.12, 2.10)	0.56 (0.15, 2.22)	0.40 (0.07, 2.66)	0.56 (0.14, 2.34)	**0.12 (0.01, 0.75)**
NNC2mg/kgSC	**5.16 (1.29, 23.09)**	2.38 (0.73, 7.82)	1.31 (0.37, 5.05)	2.15 (0.63, 7.70)	0.68 (0.18, 2.63)	0.61 (0.15, 2.31)	1.18 (0.34, 4.08)	1.81 (0.49, 6.78)	2.08 (0.48, 9.57)	1.54 (0.42, 6.14)	1.32 (0.29, 6.75)	1.48 (0.48, 4.92)	1.77 (0.42, 8.14)	0.34 (0.03, 2.56)	0.34 (0.00, 3.34)	0.88 (0.21, 4.15)	1.04 (0.24, 4.90)	0.75 (0.12, 5.16)	1.04 (0.23, 4.65)	0.22 (0.02, 1.43)
ONT22.5mgSC	3.33 (0.98, 11.84)	1.56 (0.54, 4.09)	0.86 (0.25, 2.59)	1.40 (0.46, 4.07)	0.44 (0.13, 1.41)	0.40 (0.11, 1.33)	0.77 (0.25, 2.17)	1.18 (0.37, 3.85)	1.35 (0.34, 5.55)	0.99 (0.31, 3.25)	0.86 (0.20, 4.02)	0.95 (0.36, 2.56)	1.15 (0.31, 4.60)	0.23 (0.02, 1.58)	0.21 (0.00, 2.45)	0.58 (0.14, 2.33)	0.68 (0.18, 2.68)	0.49 (0.07, 3.10)	0.68 (0.18, 2.64)	**0.14 (0.02, 0.84)**
ONT225mgSC	**3.65 (1.08, 13.57)**	1.70 (0.57, 4.50)	0.92 (0.27, 2.79)	1.52 (0.49, 4.37)	0.48 (0.14, 1.64)	0.44 (0.12, 1.42)	0.83 (0.28, 2.35)	1.28 (0.39, 3.94)	1.49 (0.35, 5.99)	1.09 (0.33, 3.44)	0.95 (0.22, 3.99)	1.04 (0.39, 2.88)	1.26 (0.33, 4.97)	0.24 (0.03, 1.59)	0.23 (0.00, 2.49)	0.63 (0.15, 2.61)	0.74 (0.19, 2.84)	0.53 (0.09, 3.26)	0.75 (0.18, 2.98)	**0.16 (0.02, 0.93)**
ONT75mgSC	**4.37 (1.23, 15.83)**	2.03 (0.69, 5.48)	1.10 (0.33, 3.53)	1.79 (0.59, 5.50)	0.57 (0.15, 1.97)	0.52 (0.14, 1.75)	0.98 (0.32, 2.95)	1.53 (0.46, 5.09)	1.75 (0.44, 7.68)	1.30 (0.39, 4.54)	1.12 (0.26, 4.99)	1.25 (0.46, 3.52)	1.49 (0.39, 6.31)	0.29 (0.03, 2.02)	0.28 (0.00, 2.91)	0.75 (0.18, 2.95)	0.88 (0.22, 3.37)	0.64 (0.09, 4.05)	0.88 (0.22, 3.39)	0.19 (0.02, 1.11)
PBO	**5.75 (2.52, 15.80)**	**2.69 (1.63, 4.54)**	1.48 (0.66, 3.18)	**2.43 (1.29, 4.62)**	0.76 (0.31, 1.83)	0.69 (0.28, 1.58)	1.32 (0.69, 2.58)	2.03 (0.94, 4.56)	2.32 (0.82, 7.39)	1.74 (0.80, 4.04)	1.50 (0.48, 4.97)	**1.64 (1.04, 2.95)**	1.98 (0.73, 6.27)	0.39 (0.05, 2.14)	0.39 (0.01, 3.26)	0.99 (0.34, 3.10)	1.16 (0.43, 3.53)	0.84 (0.17, 4.33)	1.17 (0.42, 3.47)	0.25 (0.03, 1.19)
RIS1200mgIV	**3.15 (1.23, 9.32)**	1.47 (0.71, 2.92)	0.81 (0.31, 2.03)	1.31 (0.61, 3.04)	0.42 (0.15, 1.13)	**0.37 (0.14, 0.97)**	0.72 (0.32, 1.61)	1.12 (0.45, 2.76)	1.27 (0.40, 4.41)	0.94 (0.39, 2.45)	0.83 (0.24, 2.99)	0.90 (0.46, 1.90)	1.09 (0.34, 3.74)	0.21 (0.03, 1.27)	0.21 (0.00, 1.93)	0.54 (0.17, 1.75)	0.63 (0.21, 2.09)	0.46 (0.09, 2.57)	0.64 (0.20, 2.07)	**0.14 (0.02, 0.69)**
RIS200mgIV	**5.99 (1.28, 34.47)**	2.72 (0.73, 12.45)	1.50 (0.36, 7.39)	2.47 (0.65, 11.47)	0.79 (0.18, 3.94)	0.69 (0.16, 3.62)	1.33 (0.34, 6.39)	2.06 (0.55, 10.05)	2.40 (0.50, 13.83)	1.79 (0.44, 9.31)	1.58 (0.26, 9.46)	1.65 (0.51, 7.60)	2.08 (0.42, 11.53)	0.38 (0.04, 3.49)	0.40 (0.01, 4.40)	1.05 (0.21, 5.77)	1.21 (0.25, 6.57)	0.88 (0.12, 6.79)	1.21 (0.26, 6.61)	0.26 (0.02, 2.05)
RIS600mgIV	**3.05 (1.19, 8.89)**	1.43 (0.69, 2.78)	0.79 (0.30, 1.92)	1.28 (0.59, 2.88)	0.41 (0.15, 1.06)	**0.36 (0.13, 0.92)**	0.70 (0.30, 1.52)	1.08 (0.44, 2.60)	1.23 (0.39, 4.13)	0.92 (0.38, 2.37)	0.79 (0.23, 2.85)	0.87 (0.45, 1.81)	1.05 (0.34, 3.43)	0.20 (0.02, 1.19)	0.20 (0.00, 1.89)	0.53 (0.16, 1.68)	0.62 (0.20, 2.01)	0.45 (0.08, 2.45)	0.62 (0.20, 1.97)	**0.13 (0.02, 0.65)**

	ADA160mg80mg60mgSC	ADA160mg80mgSC	ADA40mg20mgSC	ADA80mg40mgSC	API100mgPO	API50mgPO	CDP10mg/kgIV	CER100mgSC	CER10mgIV	CER200mgSC	CER20mgIV	CER400mgSC	CER5mgIV	ETA25mgSC	FON0.1mg/kgIV	FON1mg/kg0.1mgIV/SC	FON1mg/kg1mgIV/SC	FON1mg/kgIV	FON4mg/kg0.1mgIV/SC	FON4mg/kg1mgIV/SC
SEC10mg/kgIV	**11.18 (1.59, 79.64)**	5.06 (0.85, 33.34)	2.81 (0.41, 19.27)	4.62 (0.72, 30.09)	1.46 (0.20, 9.89)	1.28 (0.19, 9.20)	2.50 (0.41, 16.04)	3.95 (0.62, 24.33)	4.51 (0.55, 38.09)	3.31 (0.51, 23.31)	2.91 (0.35, 23.85)	3.17 (0.55, 20.07)	3.91 (0.49, 29.78)	0.74 (0.05, 7.78)	0.68 (0.01, 12.05)	1.93 (0.26, 14.90)	2.27 (0.30, 17.21)	1.53 (0.17, 18.97)	2.28 (0.29, 17.65)	0.47 (0.03, 5.58)
TES400mg200mgSC	1.88 (0.51, 7.34)	0.86 (0.28, 2.45)	0.47 (0.13, 1.68)	0.78 (0.24, 2.39)	**0.24 (0.06, 0.90)**	**0.22 (0.06, 0.76)**	0.43 (0.12, 1.32)	0.66 (0.17, 2.33)	0.76 (0.17, 3.42)	0.57 (0.15, 2.01)	0.49 (0.10, 2.25)	0.53 (0.18, 1.62)	0.65 (0.15, 2.84)	**0.13 (0.01, 0.86)**	0.12 (0.00, 1.27)	0.32 (0.07, 1.42)	0.38 (0.09, 1.58)	0.27 (0.04, 1.78)	0.38 (0.09, 1.58)	**0.08 (0.01, 0.52)**
TOF15mgPO_BID	**8.46 (2.00, 41.50)**	**3.94 (1.12, 15.08)**	2.16 (0.51, 9.49)	3.57 (0.96, 14.37)	1.12 (0.27, 5.06)	1.00 (0.23, 4.56)	1.95 (0.51, 8.02)	2.95 (0.76, 13.86)	3.39 (0.73, 18.01)	2.55 (0.64, 11.78)	2.17 (0.44, 12.79)	2.42 (0.72, 9.43)	2.93 (0.65, 14.69)	0.56 (0.06, 5.40)	0.56 (0.01, 6.21)	1.46 (0.32, 7.87)	1.70 (0.36, 9.15)	1.22 (0.19, 9.27)	1.71 (0.36, 9.54)	0.36 (0.04, 2.54)
TOF1mgPO_BID	**3.83 (1.05, 15.12)**	1.78 (0.59, 5.07)	0.99 (0.28, 3.38)	1.62 (0.49, 5.00)	0.50 (0.13, 1.80)	0.45 (0.12, 1.67)	0.88 (0.27, 2.82)	1.35 (0.39, 4.78)	1.57 (0.36, 6.74)	1.17 (0.31, 4.14)	1.01 (0.22, 4.44)	1.11 (0.37, 3.33)	1.33 (0.31, 5.64)	0.25 (0.03, 1.90)	0.26 (0.00, 2.52)	0.66 (0.16, 2.71)	0.78 (0.17, 3.16)	0.55 (0.09, 3.50)	0.78 (0.18, 3.31)	**0.16 (0.02, 0.98)**
TOF5mgPO_BID	**5.06 (1.28, 20.04)**	2.33 (0.70, 7.37)	1.28 (0.34, 4.70)	2.08 (0.59, 7.11)	0.66 (0.17, 2.53)	0.60 (0.15, 2.34)	1.15 (0.32, 3.90)	1.75 (0.49, 6.57)	2.03 (0.46, 10.40)	1.51 (0.39, 5.75)	1.30 (0.27, 6.20)	1.43 (0.46, 4.63)	1.75 (0.40, 7.99)	0.33 (0.03, 2.51)	0.34 (0.00, 3.34)	0.87 (0.20, 3.98)	1.02 (0.22, 4.50)	0.73 (0.11, 4.69)	1.02 (0.24, 4.73)	0.21 (0.02, 1.39)
UPA45mgPO	**4.14 (1.49, 13.21)**	1.92 (0.89, 4.12)	1.06 (0.41, 2.79)	1.72 (0.74, 4.10)	0.55 (0.19, 1.50)	0.49 (0.17, 1.36)	0.95 (0.39, 2.29)	1.45 (0.57, 4.02)	1.65 (0.53, 5.98)	1.24 (0.49, 3.53)	1.08 (0.29, 4.10)	1.17 (0.59, 2.76)	1.43 (0.47, 5.24)	0.28 (0.03, 1.71)	0.28 (0.00, 2.52)	0.71 (0.21, 2.44)	0.84 (0.25, 2.83)	0.61 (0.11, 3.36)	0.85 (0.26, 2.83)	**0.18 (0.02, 0.95)**
UST1mgIV	**4.87 (1.46, 19.47)**	2.28 (0.78, 6.60)	1.24 (0.38, 4.11)	2.04 (0.67, 6.37)	0.65 (0.18, 2.23)	0.58 (0.16, 2.00)	1.11 (0.36, 3.33)	1.72 (0.50, 5.83)	2.00 (0.48, 8.52)	1.49 (0.44, 4.84)	1.28 (0.29, 5.80)	1.42 (0.50, 4.08)	1.70 (0.44, 7.18)	0.32 (0.04, 2.30)	0.32 (0.01, 3.14)	0.85 (0.21, 3.47)	0.99 (0.25, 3.99)	0.71 (0.11, 4.62)	1.01 (0.24, 3.88)	0.21 (0.02, 1.29)
UST3mgIV	2.72 (0.86, 9.60)	1.28 (0.47, 3.25)	0.70 (0.23, 2.08)	1.14 (0.39, 3.16)	0.36 (0.11, 1.14)	0.32 (0.10, 1.04)	0.62 (0.22, 1.71)	0.97 (0.29, 2.93)	1.10 (0.30, 4.50)	0.82 (0.25, 2.57)	0.71 (0.17, 3.10)	0.78 (0.30, 2.06)	0.94 (0.25, 3.65)	0.18 (0.02, 1.19)	0.18 (0.00, 1.64)	0.48 (0.13, 1.83)	0.55 (0.15, 2.13)	0.40 (0.06, 2.41)	0.55 (0.14, 2.06)	**0.12 (0.01, 0.64)**
UST4.5mg/kgIV	3.09 (0.64, 14.97)	1.44 (0.34, 5.33)	0.79 (0.17, 3.33)	1.31 (0.28, 5.02)	0.41 (0.08, 1.77)	0.37 (0.08, 1.54)	0.71 (0.16, 2.75)	1.10 (0.23, 4.57)	1.26 (0.24, 6.85)	0.94 (0.21, 3.84)	0.78 (0.15, 4.46)	0.89 (0.22, 3.37)	1.09 (0.21, 5.45)	0.21 (0.02, 1.65)	0.20 (0.00, 2.46)	0.54 (0.09, 2.70)	0.63 (0.12, 3.01)	0.45 (0.05, 3.28)	0.64 (0.11, 3.18)	**0.13 (0.01, 0.96)**
UST6mg/kg90mgIV/SC	2.16 (0.56, 8.33)	1.00 (0.33, 2.90)	0.55 (0.16, 1.80)	0.90 (0.29, 2.83)	0.28 (0.08, 1.02)	**0.25 (0.07, 0.92)**	0.50 (0.15, 1.48)	0.76 (0.22, 2.55)	0.87 (0.21, 3.77)	0.65 (0.18, 2.24)	0.56 (0.12, 2.51)	0.62 (0.21, 1.79)	0.74 (0.19, 3.17)	0.14 (0.01, 1.00)	0.14 (0.00, 1.29)	0.38 (0.09, 1.59)	0.43 (0.11, 1.71)	0.31 (0.05, 1.99)	0.44 (0.11, 1.76)	**0.09 (0.01, 0.54)**
UST6mgIV	**4.15 (1.28, 17.16)**	1.97 (0.69, 5.48)	1.08 (0.33, 3.55)	1.76 (0.59, 5.29)	0.56 (0.16, 1.86)	0.50 (0.14, 1.73)	0.97 (0.32, 2.86)	1.48 (0.45, 4.86)	1.72 (0.44, 7.28)	1.26 (0.38, 4.26)	1.11 (0.26, 4.77)	1.21 (0.44, 3.31)	1.45 (0.38, 6.09)	0.28 (0.03, 1.93)	0.28 (0.01, 2.67)	0.73 (0.18, 3.06)	0.85 (0.22, 3.55)	0.62 (0.10, 4.00)	0.86 (0.21, 3.45)	0.18 (0.02, 1.07)
UST90mgSC	3.51 (0.69, 17.69)	1.65 (0.37, 6.63)	0.88 (0.18, 4.16)	1.47 (0.31, 6.47)	0.47 (0.09, 2.28)	0.41 (0.08, 1.98)	0.79 (0.18, 3.38)	1.24 (0.26, 5.71)	1.40 (0.25, 7.86)	1.06 (0.22, 4.97)	0.89 (0.15, 5.25)	1.02 (0.23, 4.09)	1.21 (0.22, 6.44)	0.23 (0.02, 1.90)	0.22 (0.00, 2.89)	0.61 (0.11, 3.24)	0.70 (0.12, 3.82)	0.51 (0.06, 4.12)	0.71 (0.13, 3.83)	0.15 (0.01, 1.15)
VED0.5mg/kgIV	3.25 (0.85, 12.48)	1.51 (0.48, 4.50)	0.81 (0.22, 2.78)	1.34 (0.41, 4.35)	0.43 (0.11, 1.55)	0.39 (0.10, 1.40)	0.74 (0.21, 2.39)	1.15 (0.33, 3.97)	1.31 (0.29, 5.98)	0.97 (0.28, 3.54)	0.84 (0.17, 3.77)	0.93 (0.31, 2.78)	1.12 (0.27, 4.93)	0.21 (0.02, 1.59)	0.20 (0.00, 2.13)	0.55 (0.14, 2.35)	0.66 (0.16, 2.71)	0.48 (0.07, 3.08)	0.65 (0.15, 2.74)	**0.14 (0.01, 0.92)**
VED2mg/kgIV	2.45 (0.67, 9.06)	1.14 (0.38, 2.98)	0.62 (0.17, 1.96)	1.02 (0.32, 2.88)	0.32 (0.08, 1.06)	**0.29 (0.08, 0.94)**	0.56 (0.17, 1.68)	0.86 (0.25, 2.79)	0.97 (0.22, 4.24)	0.74 (0.21, 2.39)	0.62 (0.14, 2.72)	0.70 (0.24, 2.00)	0.84 (0.21, 3.59)	0.16 (0.02, 1.11)	0.15 (0.00, 1.50)	0.42 (0.10, 1.69)	0.49 (0.12, 1.95)	0.35 (0.05, 2.24)	0.49 (0.12, 1.90)	**0.10 (0.01, 0.65)**

	FON4mg/kgIV	GUS1200mgIV	GUS200mgIV	GUS600mgIV	INF10mg/kgIV	INF20mg/kgIV	INF5mg/kgIV	NAT300mgIV	NAT3mg/kgIV	NAT3mg/kgIVx2	NAT6mg/kgIVx2	NNC2mg/kgSC	ONT22.5mgSC	ONT225mgSC	ONT75mgSC	PBO	RIS1200mgIV	RIS200mgIV	RIS600mgIV	SEC10mg/kgIV
ADA160mg80mg60mgSC	0.28 (0.06, 1.58)	0.29 (0.07, 1.28)	0.29 (0.08, 1.16)	0.31 (0.08, 1.23)	1.43 (0.17, 32.50)	1.48 (0.17, 27.55)	2.87 (0.36, 59.71)	**0.24 (0.08, 0.60)**	0.38 (0.11, 1.26)	0.36 (0.10, 1.18)	0.36 (0.09, 1.23)	**0.19 (0.04, 0.77)**	0.30 (0.08, 1.02)	**0.27 (0.07, 0.93)**	**0.23 (0.06, 0.81)**	**0.17 (0.06, 0.40)**	**0.32 (0.11, 0.82)**	**0.17 (0.03, 0.78)**	**0.33 (0.11, 0.84)**	**0.09 (0.01, 0.63)**
ADA160mg80mgSC	0.60 (0.17, 2.99)	0.64 (0.22, 2.04)	0.64 (0.21, 2.04)	0.67 (0.23, 2.07)	3.03 (0.43, 59.06)	3.22 (0.45, 55.41)	6.22 (0.93, 115.60)	0.51 (0.26, 1.01)	0.81 (0.29, 2.25)	0.76 (0.29, 2.18)	0.76 (0.28, 2.18)	0.42 (0.13, 1.37)	0.64 (0.24, 1.84)	0.59 (0.22, 1.77)	0.49 (0.18, 1.45)	**0.37 (0.22, 0.61)**	0.68 (0.34, 1.42)	0.37 (0.08, 1.37)	0.70 (0.36, 1.44)	0.20 (0.03, 1.18)
ADA40mg20mgSC	1.10 (0.26, 5.77)	1.17 (0.35, 4.21)	1.18 (0.33, 4.15)	1.23 (0.37, 4.43)	5.77 (0.70, 123.57)	5.85 (0.75, 108.08)	**11.51 (1.56, 250.25)**	0.91 (0.37, 2.28)	1.48 (0.44, 4.98)	1.40 (0.44, 4.68)	1.41 (0.43, 4.64)	0.76 (0.20, 2.73)	1.17 (0.39, 3.93)	1.08 (0.36, 3.70)	0.91 (0.28, 3.02)	0.67 (0.31, 1.52)	1.23 (0.49, 3.17)	0.67 (0.14, 2.79)	1.27 (0.52, 3.29)	0.36 (0.05, 2.42)
ADA80mg40mgSC	0.67 (0.18, 3.31)	0.72 (0.23, 2.39)	0.71 (0.22, 2.35)	0.76 (0.23, 2.46)	3.43 (0.44, 69.30)	3.59 (0.48, 64.37)	6.96 (1.00, 133.53)	0.56 (0.26, 1.21)	0.90 (0.29, 2.73)	0.85 (0.29, 2.58)	0.84 (0.28, 2.65)	0.47 (0.13, 1.58)	0.71 (0.25, 2.16)	0.66 (0.23, 2.03)	0.56 (0.18, 1.71)	**0.41 (0.22, 0.78)**	0.76 (0.33, 1.65)	0.40 (0.09, 1.53)	0.78 (0.35, 1.70)	0.22 (0.03, 1.40)
API100mgPO	2.13 (0.50, 12.10)	2.28 (0.62, 8.92)	2.27 (0.60, 8.84)	2.41 (0.66, 9.25)	**11.23 (1.36, 247.33)**	**11.60 (1.31, 212.36)**	**22.00 (2.84, 461.64)**	1.77 (0.69, 4.66)	2.84 (0.81, 10.70)	2.70 (0.79, 9.78)	2.72 (0.79, 9.86)	1.48 (0.38, 5.71)	2.29 (0.71, 7.87)	2.07 (0.61, 7.22)	1.76 (0.51, 6.67)	1.31 (0.55, 3.21)	2.41 (0.89, 6.63)	1.27 (0.25, 5.69)	2.45 (0.94, 6.73)	0.69 (0.10, 4.95)
API50mgPO	2.36 (0.56, 12.56)	2.52 (0.70, 10.13)	2.52 (0.65, 10.26)	2.64 (0.71, 10.71)	**12.40 (1.50, 297.52)**	**12.66 (1.54, 222.74)**	**24.30 (3.26, 528.13)**	1.97 (0.80, 5.37)	3.15 (0.91, 11.54)	2.98 (0.90, 10.49)	3.04 (0.89, 11.62)	1.64 (0.43, 6.67)	2.51 (0.75, 8.88)	2.30 (0.71, 8.65)	1.94 (0.57, 7.05)	1.45 (0.63, 3.58)	**2.68 (1.03, 7.36)**	1.45 (0.28, 6.14)	**2.75 (1.08, 7.46)**	0.78 (0.11, 5.32)
CDP10mg/kgIV	1.22 (0.32, 6.06)	1.29 (0.42, 4.51)	1.28 (0.41, 4.27)	1.37 (0.42, 4.52)	6.20 (0.87, 129.17)	6.44 (0.87, 113.38)	**12.43 (1.84, 238.44)**	1.03 (0.45, 2.28)	1.66 (0.52, 4.86)	1.55 (0.53, 4.84)	1.57 (0.52, 4.82)	0.85 (0.25, 2.90)	1.30 (0.46, 4.01)	1.21 (0.43, 3.61)	1.02 (0.34, 3.15)	0.76 (0.39, 1.46)	1.39 (0.62, 3.17)	0.75 (0.16, 2.93)	1.43 (0.66, 3.30)	0.40 (0.06, 2.43)
CER100mgSC	0.80 (0.19, 4.35)	0.85 (0.25, 3.06)	0.86 (0.23, 2.99)	0.90 (0.25, 3.19)	4.12 (0.54, 81.92)	4.34 (0.55, 75.58)	**8.19 (1.14, 160.07)**	0.67 (0.26, 1.63)	1.07 (0.33, 3.32)	1.02 (0.31, 3.20)	1.02 (0.30, 3.23)	0.55 (0.15, 2.04)	0.85 (0.26, 2.72)	0.78 (0.25, 2.58)	0.65 (0.20, 2.17)	0.49 (0.22, 1.06)	0.89 (0.36, 2.24)	0.48 (0.10, 1.80)	0.92 (0.38, 2.28)	0.25 (0.04, 1.62)
CER10mgIV	0.69 (0.13, 4.24)	0.74 (0.16, 3.11)	0.73 (0.16, 3.23)	0.78 (0.18, 3.16)	3.68 (0.38, 86.88)	3.73 (0.40, 70.75)	7.42 (0.77, 164.77)	0.58 (0.16, 1.75)	0.92 (0.24, 3.80)	0.87 (0.23, 3.52)	0.87 (0.22, 3.46)	0.48 (0.10, 2.10)	0.74 (0.18, 2.91)	0.67 (0.17, 2.86)	0.57 (0.13, 2.28)	0.43 (0.14, 1.23)	0.79 (0.23, 2.53)	0.42 (0.07, 2.02)	0.81 (0.24, 2.58)	0.22 (0.03, 1.81)
CER200mgSC	0.93 (0.21, 5.15)	0.99 (0.27, 3.56)	0.99 (0.26, 3.59)	1.05 (0.28, 3.76)	4.85 (0.63, 89.45)	5.02 (0.62, 79.14)	**9.63 (1.28, 178.65)**	0.78 (0.29, 1.90)	1.27 (0.38, 4.07)	1.19 (0.35, 3.97)	1.21 (0.34, 3.89)	0.65 (0.16, 2.36)	1.01 (0.31, 3.20)	0.92 (0.29, 3.00)	0.77 (0.22, 2.53)	0.58 (0.25, 1.24)	1.06 (0.41, 2.56)	0.56 (0.11, 2.25)	1.08 (0.42, 2.67)	0.30 (0.04, 1.96)
CER20mgIV	1.09 (0.19, 7.08)	1.14 (0.25, 6.25)	1.14 (0.24, 5.36)	1.21 (0.26, 5.87)	5.69 (0.54, 123.16)	5.71 (0.58, 111.62)	**11.37 (1.20, 250.18)**	0.90 (0.25, 2.99)	1.44 (0.34, 6.18)	1.36 (0.33, 6.01)	1.36 (0.32, 6.10)	0.76 (0.15, 3.41)	1.17 (0.25, 5.00)	1.05 (0.25, 4.64)	0.89 (0.20, 3.90)	0.67 (0.20, 2.10)	1.21 (0.33, 4.23)	0.63 (0.11, 3.79)	1.26 (0.35, 4.40)	0.34 (0.04, 2.86)
CER400mgSC	0.98 (0.26, 4.49)	1.03 (0.33, 3.30)	1.03 (0.33, 3.25)	1.11 (0.35, 3.36)	5.09 (0.73, 95.02)	5.17 (0.73, 85.67)	**10.06 (1.49, 185.41)**	0.83 (0.40, 1.49)	1.32 (0.44, 3.42)	1.24 (0.46, 3.30)	1.25 (0.45, 3.45)	0.68 (0.20, 2.10)	1.05 (0.39, 2.79)	0.96 (0.35, 2.59)	0.80 (0.28, 2.19)	**0.61 (0.34, 0.96)**	1.11 (0.53, 2.16)	0.60 (0.13, 1.98)	1.15 (0.55, 2.23)	0.32 (0.05, 1.81)
CER5mgIV	0.80 (0.16, 4.89)	0.86 (0.20, 3.71)	0.86 (0.20, 3.59)	0.91 (0.21, 3.63)	4.31 (0.45, 97.62)	4.32 (0.45, 79.15)	8.69 (0.95, 187.09)	0.68 (0.20, 1.99)	1.09 (0.27, 4.23)	1.01 (0.26, 3.91)	1.03 (0.26, 4.16)	0.57 (0.12, 2.38)	0.87 (0.22, 3.21)	0.79 (0.20, 3.07)	0.67 (0.16, 2.55)	0.51 (0.16, 1.38)	0.92 (0.27, 2.91)	0.48 (0.09, 2.40)	0.95 (0.29, 2.94)	0.26 (0.03, 2.06)
ETA25mgSC	4.33 (0.54, 52.91)	4.56 (0.62, 42.38)	4.41 (0.65, 44.21)	4.74 (0.64, 47.26)	**22.31 (1.71, 893.12)**	**22.72 (1.81, 791.89)**	**44.58 (3.56, 1763.22)**	3.49 (0.61, 28.82)	5.76 (0.82, 49.28)	5.28 (0.81, 45.75)	5.42 (0.83, 47.82)	2.94 (0.39, 30.23)	4.44 (0.63, 45.33)	4.09 (0.63, 39.90)	3.50 (0.50, 36.83)	2.58 (0.47, 20.21)	4.70 (0.79, 38.61)	2.63 (0.29, 26.45)	4.88 (0.84, 41.08)	1.35 (0.13, 18.63)
FON0.1mg/kgIV	4.21 (0.64, 253.77)	4.39 (0.43, 280.59)	4.50 (0.44, 274.36)	4.76 (0.48, 284.17)	**24.84 (1.33, 2526.16)**	**24.70 (1.33, 2424.32)**	**49.26 (2.77, 4617.21)**	3.53 (0.40, 205.16)	5.71 (0.51, 412.38)	5.37 (0.49, 386.23)	5.44 (0.48, 376.82)	2.94 (0.30, 228.07)	4.66 (0.41, 287.34)	4.27 (0.40, 287.16)	3.59 (0.34, 240.12)	2.57 (0.31, 144.67)	4.81 (0.52, 268.93)	2.52 (0.23, 157.59)	4.93 (0.53, 280.90)	1.47 (0.08, 107.75)
FON1mg/kg0.1mgIV/SC	1.60 (0.33, 9.78)	1.72 (0.39, 7.60)	1.70 (0.40, 7.40)	1.83 (0.41, 8.01)	8.27 (0.85, 226.67)	8.61 (0.84, 205.74)	**16.91 (1.80, 444.29)**	1.38 (0.42, 4.32)	2.19 (0.48, 8.75)	2.08 (0.47, 8.39)	2.10 (0.48, 8.41)	1.13 (0.24, 4.85)	1.72 (0.43, 7.12)	1.58 (0.38, 6.82)	1.33 (0.34, 5.70)	1.01 (0.32, 2.92)	1.85 (0.57, 5.80)	0.95 (0.17, 4.75)	1.88 (0.59, 6.12)	0.52 (0.07, 3.79)
FON1mg/kg1mgIV/SC	1.38 (0.28, 8.14)	1.48 (0.34, 6.44)	1.47 (0.35, 6.12)	1.55 (0.36, 6.47)	7.21 (0.78, 170.43)	7.45 (0.76, 145.57)	**14.49 (1.63, 344.88)**	1.15 (0.36, 3.53)	1.86 (0.43, 7.14)	1.76 (0.45, 6.63)	1.78 (0.45, 6.83)	0.96 (0.20, 4.17)	1.48 (0.37, 5.64)	1.35 (0.35, 5.36)	1.14 (0.30, 4.63)	0.86 (0.28, 2.34)	1.58 (0.48, 4.80)	0.82 (0.15, 4.04)	1.61 (0.50, 4.97)	0.44 (0.06, 3.29)
FON1mg/kgIV	1.90 (0.58, 8.90)	2.07 (0.30, 14.03)	1.99 (0.31, 13.61)	2.13 (0.31, 14.08)	10.19 (0.83, 262.77)	10.59 (0.75, 249.84)	**20.54 (1.61, 530.91)**	1.60 (0.30, 8.57)	2.63 (0.40, 15.71)	2.47 (0.39, 14.77)	2.48 (0.38, 15.07)	1.33 (0.19, 8.63)	2.04 (0.32, 13.60)	1.89 (0.31, 11.29)	1.56 (0.25, 10.54)	1.19 (0.23, 5.95)	2.19 (0.39, 11.23)	1.14 (0.15, 8.28)	2.25 (0.41, 11.97)	0.65 (0.05, 5.93)
FON4mg/kg0.1mgIV/SC	1.38 (0.30, 8.12)	1.46 (0.35, 6.46)	1.44 (0.35, 6.30)	1.54 (0.35, 6.67)	7.09 (0.72, 174.81)	7.38 (0.85, 162.06)	**14.58 (1.71, 340.16)**	1.15 (0.37, 3.54)	1.83 (0.48, 7.20)	1.75 (0.41, 7.05)	1.79 (0.43, 7.03)	0.96 (0.21, 4.34)	1.48 (0.38, 5.69)	1.34 (0.34, 5.44)	1.14 (0.30, 4.63)	0.85 (0.29, 2.40)	1.56 (0.48, 4.94)	0.82 (0.15, 3.87)	1.61 (0.51, 5.06)	0.44 (0.06, 3.40)
FON4mg/kg1mgIV/SC	6.70 (0.88, 92.84)	**7.01 (1.11, 65.58)**	**6.94 (1.12, 64.12)**	**7.32 (1.10, 69.30)**	**37.05 (2.92, 1133.39)**	**37.56 (2.87, 968.80)**	**74.74 (5.49, 2172.11)**	**5.41 (1.09, 44.38)**	**9.13 (1.29, 80.25)**	**8.43 (1.36, 71.68)**	**8.48 (1.33, 72.93)**	4.58 (0.70, 43.77)	**6.93 (1.20, 63.94)**	**6.29 (1.08, 60.46)**	5.31 (0.90, 50.99)	3.94 (0.84, 31.34)	**7.34 (1.45, 61.98)**	3.85 (0.49, 42.97)	**7.44 (1.54, 64.62)**	2.13 (0.18, 30.21)

(TABLE S12F. continued)

	FON4mg/kgIV	GUS1200mgIV	GUS200mgIV	GUS600mgIV	INF10mg/kgIV	INF20mg/kgIV	INF5mg/kgIV	NAT300mgIV	NAT3mg/kgIV	NAT3mg/kgIVx2	NAT6mg/kgIVx2	NNC2mg/kgSC	ONT22.5mgSC	ONT225mgSC	ONT75mgSC	PBO	RIS1200mgIV	RIS200mgIV	RIS600mgIV	SEC10mg/kgIV
FON4mg/kgIV	FON4mg/kgIV	1.06 (0.18, 5.11)	1.05 (0.18, 5.01)	1.11 (0.19, 5.23)	5.07 (0.51, 122.04)	5.26 (0.48, 111.58)	10.18 (0.98, 252.33)	0.84 (0.18, 2.92)	1.35 (0.24, 5.59)	1.26 (0.24, 5.59)	1.27 (0.23, 5.58)	0.68 (0.11, 3.41)	1.09 (0.19, 4.51)	0.99 (0.17, 4.34)	0.83 (0.15, 3.65)	0.62 (0.14, 2.00)	1.13 (0.24, 4.23)	0.58 (0.09, 3.24)	1.17 (0.25, 4.23)	0.32 (0.03, 2.58)
GUS1200mgIV	0.95 (0.20, 5.44)	GUS1200mgIV	1.00 (0.39, 2.37)	1.06 (0.44, 2.40)	4.73 (0.54, 103.42)	4.94 (0.57, 99.02)	**9.65 (1.19, 219.89)**	0.80 (0.25, 2.26)	1.26 (0.31, 4.55)	1.18 (0.30, 4.37)	1.19 (0.28, 4.48)	0.65 (0.15, 2.71)	0.99 (0.27, 3.65)	0.93 (0.24, 3.27)	0.78 (0.19, 2.83)	0.59 (0.19, 1.48)	1.08 (0.32, 3.03)	0.55 (0.10, 2.67)	1.13 (0.34, 3.14)	0.30 (0.04, 2.23)
GUS200mgIV	0.96 (0.20, 5.47)	1.00 (0.42, 2.56)	GUS200mgIV	1.06 (0.44, 2.49)	4.95 (0.56, 99.59)	5.10 (0.56, 90.05)	**10.00 (1.19, 188.34)**	0.79 (0.26, 2.39)	1.26 (0.31, 4.74)	1.18 (0.30, 4.59)	1.21 (0.29, 4.52)	0.65 (0.16, 2.78)	1.00 (0.28, 3.87)	0.92 (0.25, 3.56)	0.78 (0.21, 2.97)	0.58 (0.21, 1.57)	1.07 (0.34, 3.30)	0.56 (0.10, 2.81)	1.10 (0.36, 3.41)	0.30 (0.04, 2.36)
GUS600mgIV	0.90 (0.19, 5.16)	0.95 (0.42, 2.27)	0.94 (0.40, 2.27)	GUS600mgIV	4.62 (0.54, 97.09)	4.79 (0.55, 95.24)	**9.29 (1.13, 180.94)**	0.76 (0.25, 2.12)	1.22 (0.28, 4.37)	1.13 (0.29, 4.02)	1.14 (0.28, 4.46)	0.63 (0.15, 2.52)	0.95 (0.27, 3.48)	0.88 (0.24, 3.23)	0.74 (0.19, 2.79)	0.56 (0.20, 1.43)	1.02 (0.33, 2.98)	0.53 (0.10, 2.53)	1.05 (0.34, 3.09)	0.29 (0.04, 2.09)
INF10mg/kgIV	0.20 (0.01, 1.97)	0.21 (0.01, 1.86)	0.20 (0.01, 1.79)	0.22 (0.01, 1.85)	INF10mg/kgIV	1.00 (0.35, 2.92)	1.96 (0.84, 5.21)	0.16 (0.01, 1.13)	0.26 (0.01, 2.28)	0.25 (0.01, 2.04)	0.25 (0.01, 2.06)	0.13 (0.01, 1.15)	0.21 (0.01, 1.64)	0.19 (0.01, 1.58)	0.15 (0.01, 1.35)	**0.12 (0.01, 0.79)**	0.22 (0.01, 1.56)	0.11 (0.00, 1.08)	0.23 (0.01, 1.64)	**0.06 (0.00, 0.84)**
INF20mg/kgIV	0.19 (0.01, 2.10)	0.20 (0.01, 1.75)	0.20 (0.01, 1.77)	0.21 (0.01, 1.81)	1.00 (0.34, 2.88)	INF20mg/kgIV	1.95 (0.83, 5.17)	0.16 (0.01, 1.11)	0.25 (0.01, 2.19)	0.24 (0.01, 2.07)	0.24 (0.01, 2.05)	0.13 (0.01, 1.19)	0.20 (0.01, 1.61)	0.18 (0.01, 1.42)	0.16 (0.01, 1.33)	**0.12 (0.01, 0.77)**	0.21 (0.01, 1.52)	0.11 (0.01, 1.10)	0.22 (0.01, 1.59)	**0.06 (0.00, 0.82)**
INF5mg/kgIV	0.10 (0.00, 1.02)	**0.10 (0.00, 0.84)**	**0.10 (0.01, 0.84)**	**0.11 (0.01, 0.88)**	0.51 (0.19, 1.20)	0.51 (0.19, 1.21)	INF5mg/kgIV	**0.08 (0.00, 0.53)**	0.13 (0.01, 1.02)	0.12 (0.01, 1.00)	**0.12 (0.01, 0.99)**	**0.06 (0.00, 0.57)**	**0.10 (0.01, 0.78)**	**0.09 (0.00, 0.73)**	**0.08 (0.00, 0.62)**	**0.06 (0.00, 0.36)**	**0.11 (0.01, 0.71)**	**0.06 (0.00, 0.54)**	**0.11 (0.01, 0.75)**	**0.03 (0.00, 0.45)**
NAT300mgIV	1.19 (0.34, 5.47)	1.26 (0.44, 4.07)	1.27 (0.42, 3.84)	1.31 (0.47, 4.06)	6.20 (0.88, 118.67)	6.28 (0.90, 108.02)	**12.31 (1.88, 233.35)**	NAT300mgIV	1.60 (0.59, 4.28)	1.50 (0.58, 4.06)	1.52 (0.57, 4.22)	0.83 (0.26, 2.67)	1.27 (0.52, 3.50)	1.17 (0.45, 3.30)	0.98 (0.38, 2.80)	0.74 (0.48, 1.13)	1.35 (0.70, 2.60)	0.73 (0.16, 2.54)	1.39 (0.74, 2.67)	0.39 (0.07, 2.27)
NAT3mg/kgIV	0.74 (0.18, 4.11)	0.79 (0.22, 3.26)	0.79 (0.21, 3.25)	0.82 (0.23, 3.51)	3.80 (0.44, 86.44)	4.01 (0.46, 68.67)	7.77 (0.98, 154.54)	0.63 (0.23, 1.69)	NAT3mg/kgIV	0.94 (0.46, 1.89)	0.96 (0.45, 1.96)	0.51 (0.13, 2.01)	0.78 (0.23, 2.94)	0.73 (0.22, 3.01)	0.61 (0.17, 2.47)	0.46 (0.19, 1.09)	0.84 (0.31, 2.28)	0.44 (0.09, 2.11)	0.87 (0.32, 2.37)	0.23 (0.04, 1.77)
NAT3mg/kgIVx2	0.79 (0.18, 4.25)	0.85 (0.23, 3.37)	0.84 (0.22, 3.36)	0.89 (0.25, 3.50)	4.07 (0.49, 87.02)	4.19 (0.48, 72.08)	8.05 (1.00, 164.76)	0.66 (0.25, 1.73)	1.06 (0.53, 2.16)	NAT3mg/kgIVx2	1.01 (0.46, 2.17)	0.54 (0.14, 2.14)	0.83 (0.23, 3.04)	0.78 (0.22, 2.82)	0.65 (0.18, 2.38)	0.49 (0.19, 1.12)	0.90 (0.33, 2.37)	0.47 (0.09, 2.23)	0.93 (0.32, 2.41)	0.25 (0.04, 1.73)
NAT6mg/kgIVx2	0.79 (0.18, 4.37)	0.84 (0.22, 3.63)	0.82 (0.22, 3.41)	0.88 (0.22, 3.57)	4.03 (0.49, 88.29)	4.20 (0.49, 71.56)	**8.15 (1.01, 159.68)**	0.66 (0.24, 1.75)	1.04 (0.51, 2.24)	0.99 (0.46, 2.17)	NAT6mg/kgIVx2	0.54 (0.14, 2.14)	0.84 (0.24, 2.99)	0.77 (0.22, 2.71)	0.64 (0.18, 2.37)	0.48 (0.19, 1.16)	0.89 (0.32, 2.40)	0.46 (0.09, 2.36)	0.92 (0.32, 2.45)	0.25 (0.04, 1.74)
NNC2mg/kgSC	1.46 (0.29, 8.88)	1.53 (0.37, 6.65)	1.54 (0.36, 6.22)	1.60 (0.40, 6.82)	7.69 (0.87, 159.03)	7.74 (0.84, 147.71)	**15.49 (1.75, 321.10)**	1.20 (0.38, 3.83)	1.96 (0.50, 7.53)	1.85 (0.47, 7.38)	1.87 (0.47, 7.29)	NNC2mg/kgSC	1.55 (0.39, 6.02)	1.43 (0.36, 5.77)	1.21 (0.29, 4.68)	0.88 (0.31, 2.51)	1.64 (0.52, 5.11)	0.86 (0.16, 4.21)	1.68 (0.55, 5.38)	0.47 (0.06, 3.26)
ONT22.5mgSC	0.92 (0.22, 5.18)	1.01 (0.27, 3.71)	1.00 (0.26, 3.54)	1.05 (0.29, 3.76)	4.85 (0.61, 100.34)	4.98 (0.62, 97.17)	**9.81 (1.28, 188.71)**	0.79 (0.29, 1.94)	1.28 (0.34, 4.43)	1.20 (0.33, 4.37)	1.19 (0.33, 4.18)	0.64 (0.17, 2.54)	ONT22.5mgSC	0.92 (0.43, 1.94)	0.77 (0.35, 1.66)	0.58 (0.23, 1.33)	1.06 (0.39, 2.76)	0.57 (0.11, 2.40)	1.09 (0.39, 2.90)	0.30 (0.04, 2.10)
ONT225mgSC	1.01 (0.23, 5.75)	1.07 (0.31, 4.10)	1.08 (0.28, 3.99)	1.14 (0.31, 4.11)	5.26 (0.63, 116.39)	5.43 (0.70, 109.56)	**10.57 (1.37, 215.89)**	0.85 (0.30, 2.21)	1.38 (0.33, 4.60)	1.28 (0.35, 4.64)	1.30 (0.37, 4.57)	0.70 (0.17, 2.80)	1.09 (0.52, 2.32)	ONT225mgSC	0.84 (0.38, 1.84)	0.63 (0.25, 1.48)	1.16 (0.42, 3.14)	0.62 (0.11, 2.46)	1.18 (0.43, 3.23)	0.33 (0.05, 2.40)
ONT75mgSC	1.20 (0.27, 6.75)	1.28 (0.35, 5.22)	1.28 (0.34, 4.83)	1.35 (0.36, 5.17)	6.46 (0.74, 128.19)	6.37 (0.75, 122.18)	**12.72 (1.60, 268.04)**	1.02 (0.36, 2.67)	1.63 (0.41, 5.72)	1.54 (0.42, 5.45)	1.55 (0.42, 5.57)	0.82 (0.21, 3.48)	1.29 (0.60, 2.86)	1.19 (0.54, 2.66)	ONT75mgSC	0.75 (0.30, 1.77)	1.38 (0.48, 3.69)	0.74 (0.13, 3.12)	1.41 (0.51, 3.91)	0.39 (0.06, 2.70)
PBO	1.62 (0.50, 7.11)	1.69 (0.68, 5.18)	1.72 (0.64, 4.84)	1.79 (0.70, 5.02)	**8.35 (1.27, 162.61)**	**8.62 (1.29, 141.87)**	**16.59 (2.75, 297.61)**	1.35 (0.88, 2.09)	2.16 (0.92, 5.33)	2.04 (0.89, 5.18)	2.07 (0.86, 5.15)	1.13 (0.40, 3.24)	1.73 (0.75, 4.29)	1.58 (0.68, 3.97)	1.34 (0.56, 3.38)	PBO	**1.83 (1.14, 2.99)**	0.99 (0.24, 3.22)	**1.87 (1.21, 3.09)**	0.52 (0.09, 2.88)
RIS1200mgIV	0.88 (0.24, 4.23)	0.92 (0.33, 3.11)	0.94 (0.30, 2.93)	0.98 (0.34, 3.05)	4.54 (0.64, 90.43)	4.77 (0.66, 82.82)	**9.13 (1.41, 167.07)**	0.74 (0.39, 1.43)	1.19 (0.44, 3.20)	1.11 (0.42, 3.03)	1.12 (0.42, 3.16)	0.61 (0.20, 1.94)	0.94 (0.36, 2.60)	0.86 (0.32, 2.39)	0.73 (0.27, 2.10)	**0.55 (0.33, 0.88)**	RIS1200mgIV	0.54 (0.13, 1.78)	1.03 (0.68, 1.62)	0.29 (0.05, 1.70)
RIS200mgIV	1.71 (0.31, 11.13)	1.82 (0.37, 10.11)	1.79 (0.36, 9.80)	1.88 (0.40, 9.92)	8.86 (0.93, 238.81)	9.04 (0.91, 188.61)	**17.85 (1.84, 442.23)**	1.36 (0.39, 6.15)	2.27 (0.47, 11.62)	2.14 (0.45, 10.88)	2.19 (0.42, 11.11)	1.17 (0.24, 6.38)	1.76 (0.42, 9.43)	1.61 (0.41, 8.81)	1.35 (0.32, 7.69)	1.01 (0.31, 4.17)	1.85 (0.56, 7.86)	RIS200mgIV	1.89 (0.59, 8.12)	0.55 (0.07, 4.59)
RIS600mgIV	0.85 (0.24, 4.06)	0.89 (0.32, 2.92)	0.91 (0.29, 2.78)	0.95 (0.32, 2.91)	4.40 (0.61, 85.58)	4.56 (0.63, 81.89)	**8.81 (1.33, 164.17)**	0.72 (0.37, 1.36)	1.15 (0.42, 3.09)	1.08 (0.42, 3.08)	1.09 (0.41, 3.10)	0.59 (0.19, 1.82)	0.92 (0.34, 2.53)	0.85 (0.31, 2.32)	0.71 (0.26, 1.97)	**0.53 (0.32, 0.82)**	0.97 (0.62, 1.46)	0.53 (0.12, 1.69)	RIS600mgIV	0.28 (0.05, 1.66)

	FON4mg/kgIV	GUS1200mgIV	GUS200mgIV	GUS600mgIV	INF10mg/kgIV	INF20mg/kgIV	INF5mg/kgIV	NAT300mgIV	NAT3mg/kgIV	NAT3mg/kgIVx2	NAT6mg/kgIVx2	NNC2mg/kgSC	ONT22.5mgSC	ONT225mgSC	ONT75mgSC	PBO	RIS1200mgIV	RIS200mgIV	RIS600mgIV	SEC10mg/kgIV
SEC10mg/kgIV	3.10 (0.39, 34.15)	3.36 (0.45, 23.86)	3.34 (0.42, 23.34)	3.49 (0.48, 24.98)	**16.44 (1.19, 539.37)**	**17.26 (1.21, 485.59)**	**33.63 (2.23, 1041.02)**	2.58 (0.44, 14.82)	4.32 (0.56, 28.39)	4.01 (0.58, 27.45)	4.04 (0.58, 28.24)	2.11 (0.31, 16.53)	3.38 (0.48, 22.76)	3.04 (0.42, 21.17)	2.55 (0.37, 17.65)	1.91 (0.35, 10.81)	3.48 (0.59, 20.98)	1.82 (0.22, 14.96)	3.61 (0.60, 21.93)	SEC10mg/kgIV
TES400mg200mgSC	0.52 (0.10, 2.94)	0.55 (0.14, 2.37)	0.55 (0.14, 2.26)	0.58 (0.15, 2.33)	2.74 (0.28, 58.47)	2.87 (0.32, 55.23)	5.57 (0.65, 114.24)	0.44 (0.14, 1.24)	0.70 (0.18, 2.46)	0.67 (0.18, 2.38)	0.66 (0.18, 2.44)	0.37 (0.08, 1.45)	0.57 (0.16, 2.06)	0.52 (0.13, 1.86)	0.44 (0.11, 1.54)	**0.33 (0.11, 0.81)**	0.60 (0.19, 1.68)	0.31 (0.06, 1.48)	0.62 (0.20, 1.71)	0.17 (0.02, 1.22)
TOF15mgPO_BID	2.39 (0.45, 15.72)	2.51 (0.57, 12.82)	2.49 (0.59, 12.16)	2.64 (0.60, 12.73)	**12.60 (1.33, 298.30)**	**13.14 (1.40, 238.06)**	**25.40 (2.86, 561.02)**	1.99 (0.62, 7.71)	3.16 (0.81, 14.22)	3.01 (0.77, 13.80)	3.02 (0.73, 14.67)	1.62 (0.35, 8.87)	2.56 (0.60, 11.87)	2.35 (0.54, 11.43)	1.98 (0.45, 9.29)	1.47 (0.48, 5.08)	2.72 (0.79, 10.34)	1.42 (0.24, 8.26)	2.80 (0.82, 10.93)	0.76 (0.10, 6.10)
TOF1mgPO_BID	1.11 (0.23, 6.06)	1.13 (0.29, 4.90)	1.13 (0.29, 4.72)	1.20 (0.30, 5.05)	5.59 (0.62, 123.32)	5.93 (0.65, 99.57)	**11.37 (1.36, 230.10)**	0.90 (0.31, 2.50)	1.44 (0.41, 5.25)	1.35 (0.38, 4.92)	1.37 (0.37, 4.90)	0.75 (0.17, 3.01)	1.15 (0.32, 4.23)	1.06 (0.30, 3.99)	0.88 (0.24, 3.34)	0.67 (0.24, 1.73)	1.24 (0.40, 3.55)	0.64 (0.12, 3.03)	1.26 (0.42, 3.67)	0.34 (0.05, 2.62)
TOF5mgPO_BID	1.41 (0.29, 8.02)	1.49 (0.36, 6.37)	1.47 (0.35, 6.33)	1.56 (0.37, 6.64)	7.25 (0.82, 166.39)	7.68 (0.84, 151.08)	**14.75 (1.73, 295.12)**	1.17 (0.38, 3.75)	1.87 (0.48, 7.17)	1.77 (0.46, 6.94)	1.79 (0.45, 7.15)	0.97 (0.22, 4.18)	1.50 (0.39, 5.96)	1.39 (0.35, 5.53)	1.15 (0.30, 4.58)	0.86 (0.30, 2.49)	1.58 (0.49, 5.03)	0.84 (0.14, 4.05)	1.63 (0.53, 5.20)	0.45 (0.06, 3.17)
UPA45mgPO	1.16 (0.31, 5.42)	1.22 (0.40, 4.24)	1.22 (0.40, 3.92)	1.30 (0.43, 4.13)	6.00 (0.86, 116.03)	6.23 (0.84, 105.54)	**11.88 (1.82, 227.73)**	0.98 (0.47, 2.02)	1.56 (0.52, 4.46)	1.46 (0.55, 4.25)	1.50 (0.53, 4.42)	0.80 (0.25, 2.65)	1.24 (0.47, 3.65)	1.14 (0.42, 3.33)	0.96 (0.34, 2.90)	0.72 (0.41, 1.28)	1.32 (0.63, 2.80)	0.71 (0.15, 2.64)	1.34 (0.68, 2.85)	0.38 (0.06, 2.32)
UST1mgIV	1.39 (0.32, 8.09)	1.47 (0.39, 5.63)	1.46 (0.36, 5.54)	1.57 (0.40, 5.58)	7.14 (0.86, 165.90)	7.37 (0.91, 134.03)	**14.12 (1.80, 308.75)**	1.16 (0.41, 3.04)	1.85 (0.53, 6.46)	1.74 (0.49, 5.82)	1.76 (0.48, 6.00)	0.96 (0.23, 3.80)	1.49 (0.43, 5.37)	1.35 (0.39, 4.81)	1.13 (0.33, 4.13)	0.86 (0.34, 2.05)	1.56 (0.56, 4.23)	0.81 (0.16, 3.56)	1.61 (0.57, 4.41)	0.45 (0.06, 3.15)
UST3mgIV	0.76 (0.18, 4.06)	0.80 (0.23, 2.97)	0.80 (0.22, 2.96)	0.85 (0.24, 3.00)	3.94 (0.50, 86.50)	4.07 (0.51, 71.56)	**8.03 (1.01, 153.30)**	0.64 (0.25, 1.56)	1.03 (0.31, 3.27)	0.97 (0.28, 3.16)	0.97 (0.28, 3.23)	0.53 (0.14, 1.99)	0.82 (0.24, 2.74)	0.74 (0.22, 2.62)	0.63 (0.18, 2.14)	0.47 (0.21, 1.03)	0.87 (0.32, 2.17)	0.46 (0.09, 1.84)	0.89 (0.35, 2.26)	0.25 (0.04, 1.62)
UST4.5mg/kgIV	0.88 (0.14, 5.80)	0.91 (0.17, 4.87)	0.91 (0.16, 4.55)	0.96 (0.18, 4.77)	4.61 (0.44, 99.60)	4.58 (0.46, 88.63)	9.02 (0.92, 195.17)	0.73 (0.17, 2.59)	1.15 (0.25, 5.22)	1.07 (0.24, 4.87)	1.09 (0.23, 4.99)	0.61 (0.12, 3.01)	0.94 (0.19, 4.12)	0.84 (0.16, 3.94)	0.72 (0.14, 3.12)	0.54 (0.14, 1.74)	1.00 (0.23, 3.72)	0.52 (0.08, 2.83)	1.02 (0.24, 3.61)	0.28 (0.03, 2.13)
UST6mg/kg90mgIV/SC	0.60 (0.13, 3.53)	0.64 (0.29, 1.43)	0.64 (0.27, 1.43)	0.68 (0.30, 1.46)	3.12 (0.35, 68.45)	3.26 (0.38, 58.08)	6.27 (0.75, 121.21)	0.51 (0.17, 1.39)	0.82 (0.20, 2.89)	0.77 (0.21, 2.84)	0.78 (0.19, 2.75)	0.42 (0.10, 1.76)	0.64 (0.19, 2.23)	0.59 (0.17, 2.13)	0.50 (0.13, 1.74)	**0.38 (0.14, 0.92)**	0.69 (0.23, 1.91)	0.36 (0.07, 1.66)	0.71 (0.24, 2.00)	0.19 (0.03, 1.41)
UST6mgIV	1.19 (0.27, 6.79)	1.27 (0.35, 4.80)	1.25 (0.33, 4.84)	1.34 (0.38, 4.80)	6.33 (0.74, 133.07)	6.24 (0.77, 116.05)	**12.71 (1.58, 257.27)**	1.00 (0.37, 2.62)	1.59 (0.45, 5.73)	1.50 (0.42, 5.19)	1.52 (0.41, 5.18)	0.82 (0.20, 3.32)	1.26 (0.38, 4.62)	1.16 (0.35, 4.24)	0.97 (0.27, 3.63)	0.74 (0.30, 1.74)	1.34 (0.51, 3.58)	0.72 (0.13, 2.98)	1.39 (0.52, 3.59)	0.38 (0.06, 2.66)
UST90mgSC	0.98 (0.16, 6.68)	1.05 (0.19, 5.38)	1.05 (0.18, 5.22)	1.11 (0.19, 5.55)	5.20 (0.49, 106.13)	5.12 (0.52, 109.04)	10.56 (1.00, 222.07)	0.83 (0.19, 3.20)	1.30 (0.26, 6.30)	1.23 (0.24, 5.99)	1.28 (0.23, 5.96)	0.67 (0.13, 3.61)	1.06 (0.20, 5.14)	0.97 (0.19, 4.74)	0.81 (0.16, 4.03)	0.62 (0.15, 2.26)	1.13 (0.26, 4.40)	0.59 (0.08, 3.38)	1.15 (0.26, 4.57)	0.31 (0.03, 2.85)
VED0.5mg/kgIV	0.91 (0.18, 5.34)	0.97 (0.23, 4.21)	0.96 (0.23, 3.93)	1.02 (0.23, 4.09)	4.71 (0.52, 93.67)	4.82 (0.54, 91.09)	**9.33 (1.03, 182.17)**	0.77 (0.25, 2.14)	1.21 (0.31, 4.52)	1.14 (0.32, 4.24)	1.15 (0.32, 4.35)	0.63 (0.15, 2.63)	0.97 (0.25, 3.50)	0.89 (0.23, 3.37)	0.76 (0.20, 2.83)	0.56 (0.20, 1.46)	1.02 (0.34, 3.03)	0.54 (0.10, 2.57)	1.05 (0.35, 3.16)	0.30 (0.04, 2.07)
VED2mg/kgIV	0.68 (0.15, 3.63)	0.73 (0.18, 2.82)	0.72 (0.18, 2.83)	0.76 (0.19, 2.84)	3.54 (0.39, 68.64)	3.70 (0.41, 58.47)	6.94 (0.78, 135.14)	0.58 (0.20, 1.46)	0.91 (0.26, 3.20)	0.85 (0.24, 3.05)	0.87 (0.24, 3.09)	0.47 (0.11, 1.92)	0.74 (0.20, 2.46)	0.67 (0.18, 2.32)	0.57 (0.15, 2.04)	**0.42 (0.16, 0.98)**	0.77 (0.26, 2.08)	0.40 (0.07, 1.86)	0.80 (0.27, 2.23)	0.22 (0.03, 1.48)

	TES400mg200mgSC	TOF15mgPO_BID	TOF1mgPO_BID	TOF5mgPO_BID	UPA45mgPO	UST1mgIV	UST3mgIV	UST4.5mg/kgIV	UST6mg/kg90mgIV/SC	UST6mgIV	UST90mgSC	VED0.5mg/kgIV	VED2mg/kgIV
ADA160mg80mg60mgSC	0.53 (0.14, 1.98)	**0.12 (0.02, 0.50)**	**0.26 (0.07, 0.95)**	**0.20 (0.05, 0.78)**	**0.24 (0.08, 0.67)**	**0.21 (0.05, 0.68)**	0.37 (0.10, 1.16)	0.32 (0.07, 1.57)	0.46 (0.12, 1.79)	**0.24 (0.06, 0.78)**	0.28 (0.06, 1.45)	0.31 (0.08, 1.18)	0.41 (0.11, 1.48)
ADA160mg80mgSC	1.16 (0.41, 3.61)	**0.25 (0.07, 0.89)**	0.56 (0.20, 1.71)	0.43 (0.14, 1.42)	0.52 (0.24, 1.13)	0.44 (0.15, 1.29)	0.78 (0.31, 2.14)	0.69 (0.19, 2.97)	1.00 (0.35, 3.01)	0.51 (0.18, 1.45)	0.61 (0.15, 2.68)	0.66 (0.22, 2.06)	0.88 (0.34, 2.65)
ADA40mg20mgSC	2.11 (0.60, 7.76)	0.46 (0.11, 1.95)	1.01 (0.30, 3.63)	0.78 (0.21, 2.96)	0.94 (0.36, 2.45)	0.81 (0.24, 2.66)	1.43 (0.48, 4.44)	1.26 (0.30, 6.03)	1.82 (0.56, 6.39)	0.93 (0.28, 3.04)	1.14 (0.24, 5.44)	1.23 (0.36, 4.46)	1.61 (0.51, 5.77)
ADA80mg40mgSC	1.28 (0.42, 4.18)	0.28 (0.07, 1.04)	0.62 (0.20, 2.03)	0.48 (0.14, 1.68)	0.58 (0.24, 1.35)	0.49 (0.16, 1.49)	0.88 (0.32, 2.54)	0.76 (0.20, 3.53)	1.11 (0.35, 3.44)	0.57 (0.19, 1.69)	0.68 (0.15, 3.18)	0.75 (0.23, 2.43)	0.98 (0.35, 3.10)
API100mgPO	**4.09 (1.11, 15.98)**	0.89 (0.20, 3.70)	1.99 (0.56, 7.63)	1.51 (0.40, 5.93)	1.83 (0.66, 5.20)	1.55 (0.45, 5.58)	2.81 (0.87, 9.25)	2.43 (0.57, 12.14)	3.53 (0.98, 12.81)	1.80 (0.54, 6.17)	2.15 (0.44, 11.61)	2.34 (0.65, 8.83)	3.13 (0.94, 11.82)
API50mgPO	**4.45 (1.32, 18.15)**	1.00 (0.22, 4.27)	2.22 (0.60, 8.57)	1.67 (0.43, 6.89)	2.03 (0.74, 5.73)	1.71 (0.50, 6.10)	3.11 (0.96, 10.17)	2.70 (0.65, 13.33)	**3.94 (1.09, 15.31)**	2.00 (0.58, 6.96)	2.43 (0.50, 12.10)	2.58 (0.72, 10.05)	**3.47 (1.06, 13.16)**
CDP10mg/kgIV	2.33 (0.76, 8.12)	0.51 (0.12, 1.96)	1.14 (0.36, 3.77)	0.87 (0.26, 3.14)	1.05 (0.44, 2.58)	0.90 (0.30, 2.81)	1.60 (0.59, 4.53)	1.41 (0.36, 6.44)	2.01 (0.68, 6.80)	1.03 (0.35, 3.14)	1.26 (0.30, 5.65)	1.35 (0.42, 4.74)	1.80 (0.60, 5.89)
CER100mgSC	1.51 (0.43, 5.77)	0.34 (0.07, 1.32)	0.74 (0.21, 2.54)	0.57 (0.15, 2.06)	0.69 (0.25, 1.76)	0.58 (0.17, 2.01)	1.04 (0.34, 3.46)	0.91 (0.22, 4.37)	1.32 (0.39, 4.55)	0.68 (0.21, 2.22)	0.81 (0.18, 3.81)	0.87 (0.25, 3.02)	1.16 (0.36, 3.93)
CER10mgIV	1.31 (0.29, 5.90)	0.30 (0.06, 1.38)	0.64 (0.15, 2.79)	0.49 (0.10, 2.20)	0.60 (0.17, 1.90)	0.50 (0.12, 2.07)	0.91 (0.22, 3.34)	0.79 (0.15, 4.10)	1.15 (0.26, 4.83)	0.58 (0.14, 2.26)	0.71 (0.13, 3.95)	0.76 (0.17, 3.43)	1.03 (0.24, 4.49)
CER200mgSC	1.75 (0.50, 6.57)	0.39 (0.08, 1.56)	0.85 (0.24, 3.21)	0.66 (0.17, 2.54)	0.80 (0.28, 2.05)	0.67 (0.21, 2.25)	1.22 (0.39, 3.95)	1.07 (0.26, 4.81)	1.55 (0.45, 5.44)	0.79 (0.23, 2.63)	0.94 (0.20, 4.49)	1.03 (0.28, 3.61)	1.36 (0.42, 4.67)
CER20mgIV	2.06 (0.44, 9.53)	0.46 (0.08, 2.27)	0.99 (0.23, 4.63)	0.77 (0.16, 3.66)	0.92 (0.24, 3.40)	0.78 (0.17, 3.49)	1.41 (0.32, 5.97)	1.28 (0.22, 6.73)	1.78 (0.40, 8.53)	0.90 (0.21, 3.85)	1.13 (0.19, 6.65)	1.19 (0.27, 5.86)	1.62 (0.37, 7.09)
CER400mgSC	1.87 (0.62, 5.71)	0.41 (0.11, 1.39)	0.90 (0.30, 2.68)	0.70 (0.22, 2.18)	0.86 (0.36, 1.69)	0.71 (0.24, 1.98)	1.28 (0.48, 3.39)	1.12 (0.30, 4.59)	1.62 (0.56, 4.87)	0.83 (0.30, 2.29)	0.98 (0.24, 4.32)	1.08 (0.36, 3.24)	1.43 (0.50, 4.17)
CER5mgIV	1.53 (0.35, 6.72)	0.34 (0.07, 1.54)	0.75 (0.18, 3.17)	0.57 (0.13, 2.50)	0.70 (0.19, 2.12)	0.59 (0.14, 2.27)	1.06 (0.27, 4.07)	0.92 (0.18, 4.74)	1.35 (0.32, 5.32)	0.69 (0.16, 2.63)	0.83 (0.16, 4.49)	0.90 (0.20, 3.71)	1.19 (0.28, 4.69)
ETA25mgSC	**7.99 (1.16, 77.44)**	1.79 (0.19, 16.98)	4.04 (0.53, 36.17)	3.01 (0.40, 29.61)	3.57 (0.58, 30.34)	3.12 (0.43, 27.60)	5.64 (0.84, 49.63)	4.84 (0.61, 51.74)	6.93 (1.00, 67.96)	3.58 (0.52, 32.22)	4.40 (0.53, 48.12)	4.71 (0.63, 47.37)	6.25 (0.90, 63.98)
FON0.1mg/kgIV	8.22 (0.79, 488.61)	1.78 (0.16, 116.76)	3.81 (0.40, 252.39)	2.94 (0.30, 212.44)	3.62 (0.40, 220.76)	3.14 (0.32, 178.99)	5.66 (0.61, 281.13)	4.98 (0.41, 364.37)	6.98 (0.78, 436.88)	3.60 (0.37, 182.44)	4.56 (0.35, 373.78)	4.95 (0.47, 294.77)	6.48 (0.67, 419.98)
FON1mg/kg0.1mgIV/SC	3.09 (0.70, 13.59)	0.68 (0.13, 3.13)	1.52 (0.37, 6.14)	1.15 (0.25, 5.11)	1.40 (0.41, 4.66)	1.18 (0.29, 4.73)	2.10 (0.55, 7.83)	1.84 (0.37, 11.06)	2.65 (0.63, 11.71)	1.37 (0.33, 5.41)	1.64 (0.31, 9.31)	1.81 (0.42, 7.37)	2.39 (0.59, 9.74)
FON1mg/kg1mgIV/SC	2.62 (0.63, 11.25)	0.59 (0.11, 2.77)	1.28 (0.32, 5.73)	0.98 (0.22, 4.58)	1.19 (0.35, 3.98)	1.01 (0.25, 3.98)	1.83 (0.47, 6.83)	1.59 (0.33, 8.31)	2.31 (0.58, 9.33)	1.17 (0.28, 4.49)	1.43 (0.26, 8.07)	1.51 (0.37, 6.25)	2.04 (0.51, 8.26)
FON1mg/kgIV	3.68 (0.56, 24.44)	0.82 (0.11, 5.40)	1.80 (0.29, 11.46)	1.36 (0.21, 9.24)	1.65 (0.30, 9.24)	1.40 (0.22, 8.75)	2.52 (0.41, 15.57)	2.22 (0.31, 18.39)	3.21 (0.50, 20.43)	1.62 (0.25, 10.32)	1.98 (0.24, 16.51)	2.09 (0.32, 14.62)	2.82 (0.45, 19.27)
FON4mg/kg0.1mgIV/SC	2.62 (0.63, 10.91)	0.59 (0.10, 2.74)	1.29 (0.30, 5.45)	0.98 (0.21, 4.22)	1.18 (0.35, 3.81)	0.99 (0.26, 4.16)	1.83 (0.48, 7.06)	1.56 (0.31, 8.78)	2.27 (0.57, 9.42)	1.17 (0.29, 4.72)	1.41 (0.26, 7.74)	1.55 (0.36, 6.70)	2.05 (0.53, 8.43)
FON4mg/kg1mgIV/SC	**12.35 (1.91, 123.36)**	2.79 (0.39, 26.80)	**6.17 (1.02, 58.52)**	4.68 (0.72, 44.91)	**5.49 (1.05, 44.80)**	4.71 (0.77, 48.04)	**8.57 (1.56, 83.27)**	**7.76 (1.05, 82.33)**	**10.75 (1.86, 95.16)**	5.46 (0.94, 54.25)	6.76 (0.87, 81.84)	**7.19 (1.08, 69.79)**	**9.84 (1.55, 90.72)**
FON4mg/kgIV	1.91 (0.34, 9.54)	0.42 (0.06, 2.23)	0.90 (0.17, 4.32)	0.71 (0.12, 3.42)	0.86 (0.18, 3.20)	0.72 (0.12, 3.15)	1.32 (0.25, 5.63)	1.13 (0.17, 7.11)	1.67 (0.28, 7.55)	0.84 (0.15, 3.66)	1.02 (0.15, 6.20)	1.10 (0.19, 5.53)	1.47 (0.28, 6.82)
GUS1200mgIV	1.83 (0.42, 7.04)	0.40 (0.08, 1.76)	0.88 (0.20, 3.47)	0.67 (0.16, 2.80)	0.82 (0.24, 2.48)	0.68 (0.18, 2.56)	1.25 (0.34, 4.28)	1.10 (0.21, 5.81)	1.56 (0.70, 3.48)	0.79 (0.21, 2.86)	0.95 (0.19, 5.37)	1.03 (0.24, 4.34)	1.38 (0.35, 5.55)
GUS200mgIV	1.81 (0.42, 6.83)	0.40 (0.08, 1.69)	0.88 (0.21, 3.48)	0.68 (0.16, 2.89)	0.82 (0.26, 2.50)	0.69 (0.18, 2.74)	1.25 (0.34, 4.51)	1.10 (0.22, 6.17)	1.57 (0.70, 3.66)	0.80 (0.21, 3.06)	0.96 (0.19, 5.53)	1.04 (0.25, 4.41)	1.39 (0.35, 5.54)
GUS600mgIV	1.72 (0.43, 6.72)	0.38 (0.08, 1.67)	0.83 (0.20, 3.29)	0.64 (0.15, 2.67)	0.77 (0.24, 2.32)	0.64 (0.18, 2.48)	1.17 (0.33, 4.23)	1.04 (0.21, 5.63)	1.47 (0.68, 3.30)	0.75 (0.21, 2.66)	0.90 (0.18, 5.23)	0.98 (0.24, 4.34)	1.31 (0.35, 5.31)
INF10mg/kgIV	0.37 (0.02, 3.53)	**0.08 (0.00, 0.75)**	0.18 (0.01, 1.63)	0.14 (0.01, 1.22)	0.17 (0.01, 1.16)	0.14 (0.01, 1.17)	0.25 (0.01, 1.99)	0.22 (0.01, 2.27)	0.32 (0.01, 2.88)	0.16 (0.01, 1.36)	0.19 (0.01, 2.03)	0.21 (0.01, 1.94)	0.28 (0.01, 2.54)
INF20mg/kgIV	0.35 (0.02, 3.16)	**0.08 (0.00, 0.72)**	0.17 (0.01, 1.54)	0.13 (0.01, 1.20)	0.16 (0.01, 1.19)	0.14 (0.01, 1.10)	0.25 (0.01, 1.97)	0.22 (0.01, 2.20)	0.31 (0.02, 2.60)	0.16 (0.01, 1.30)	0.20 (0.01, 1.94)	0.21 (0.01, 1.86)	0.27 (0.02, 2.43)
INF5mg/kgIV	0.18 (0.01, 1.53)	**0.04 (0.00, 0.35)**	**0.09 (0.00, 0.73)**	**0.07 (0.00, 0.58)**	**0.08 (0.00, 0.55)**	**0.07 (0.00, 0.56)**	**0.12 (0.01, 0.99)**	0.11 (0.01, 1.09)	0.16 (0.01, 1.33)	**0.08 (0.00, 0.63)**	0.09 (0.00, 1.00)	**0.11 (0.01, 0.97)**	0.14 (0.01, 1.28)
NAT300mgIV	2.27 (0.80, 7.01)	0.50 (0.13, 1.62)	1.11 (0.40, 3.25)	0.86 (0.27, 2.65)	1.02 (0.49, 2.13)	0.86 (0.33, 2.41)	1.56 (0.64, 4.01)	1.38 (0.39, 5.82)	1.96 (0.72, 5.76)	1.00 (0.38, 2.68)	1.21 (0.31, 5.20)	1.30 (0.47, 3.99)	1.72 (0.68, 5.02)
NAT3mg/kgIV	1.42 (0.41, 5.61)	0.32 (0.07, 1.24)	0.69 (0.19, 2.45)	0.53 (0.14, 2.06)	0.64 (0.22, 1.91)	0.54 (0.15, 1.88)	0.97 (0.31, 3.27)	0.87 (0.19, 4.02)	1.22 (0.35, 4.93)	0.63 (0.17, 2.20)	0.77 (0.16, 3.78)	0.82 (0.22, 3.23)	1.10 (0.31, 3.81)
NAT3mg/kgIVx2	1.50 (0.42, 5.65)	0.33 (0.07, 1.30)	0.74 (0.20, 2.61)	0.56 (0.14, 2.19)	0.68 (0.24, 1.81)	0.58 (0.17, 2.06)	1.03 (0.32, 3.51)	0.93 (0.21, 4.24)	1.30 (0.35, 4.87)	0.67 (0.19, 2.38)	0.82 (0.17, 4.14)	0.88 (0.24, 3.12)	1.17 (0.33, 4.13)
NAT6mg/kgIVx2	1.51 (0.41, 5.69)	0.33 (0.07, 1.37)	0.73 (0.20, 2.74)	0.56 (0.14, 2.21)	0.67 (0.23, 1.90)	0.57 (0.17, 2.10)	1.03 (0.31, 3.64)	0.92 (0.20, 4.30)	1.28 (0.36, 5.16)	0.66 (0.19, 2.46)	0.78 (0.17, 4.27)	0.87 (0.23, 3.17)	1.15 (0.32, 4.25)
NNC2mg/kgSC	2.74 (0.69, 12.00)	0.62 (0.11, 2.83)	1.34 (0.33, 5.96)	1.04 (0.24, 4.51)	1.25 (0.38, 4.03)	1.04 (0.26, 4.36)	1.90 (0.50, 7.19)	1.65 (0.33, 8.69)	2.39 (0.57, 9.95)	1.22 (0.30, 4.94)	1.49 (0.28, 7.98)	1.60 (0.38, 6.83)	2.15 (0.52, 9.07)
ONT22.5mgSC	1.77 (0.49, 6.39)	0.39 (0.08, 1.67)	0.87 (0.24, 3.09)	0.66 (0.17, 2.55)	0.80 (0.27, 2.11)	0.67 (0.19, 2.33)	1.22 (0.36, 4.10)	1.07 (0.24, 5.40)	1.56 (0.45, 5.26)	0.80 (0.22, 2.62)	0.94 (0.19, 4.90)	1.03 (0.29, 3.98)	1.35 (0.41, 5.00)
ONT225mgSC	1.93 (0.54, 7.49)	0.43 (0.09, 1.85)	0.94 (0.25, 3.32)	0.72 (0.18, 2.83)	0.88 (0.30, 2.37)	0.74 (0.21, 2.59)	1.35 (0.38, 4.47)	1.18 (0.25, 6.18)	1.69 (0.47, 5.94)	0.86 (0.24, 2.87)	1.03 (0.21, 5.39)	1.12 (0.30, 4.27)	1.50 (0.43, 5.54)
ONT75mgSC	2.28 (0.65, 9.03)	0.51 (0.11, 2.22)	1.14 (0.30, 4.18)	0.87 (0.22, 3.30)	1.04 (0.34, 2.94)	0.88 (0.24, 3.03)	1.59 (0.47, 5.44)	1.38 (0.32, 7.03)	1.98 (0.58, 7.63)	1.03 (0.28, 3.77)	1.23 (0.25, 6.42)	1.32 (0.35, 5.05)	1.76 (0.49, 6.50)
PBO	**3.07 (1.23, 8.90)**	0.68 (0.20, 2.08)	1.49 (0.58, 4.13)	1.16 (0.40, 3.32)	1.39 (0.78, 2.45)	1.17 (0.49, 2.93)	2.13 (0.97, 4.86)	1.86 (0.57, 7.16)	**2.65 (1.08, 7.30)**	1.36 (0.58, 3.36)	1.62 (0.44, 6.66)	1.78 (0.69, 4.88)	**2.39 (1.02, 6.20)**
RIS1200mgIV	1.68 (0.59, 5.18)	0.37 (0.10, 1.26)	0.81 (0.28, 2.51)	0.63 (0.20, 2.03)	0.76 (0.36, 1.58)	0.64 (0.24, 1.79)	1.15 (0.46, 3.08)	1.00 (0.27, 4.43)	1.46 (0.52, 4.39)	0.75 (0.28, 1.97)	0.89 (0.23, 3.92)	0.98 (0.33, 2.96)	1.30 (0.48, 3.90)
RIS200mgIV	3.19 (0.68, 18.01)	0.70 (0.12, 4.18)	1.56 (0.33, 8.50)	1.18 (0.25, 7.03)	1.41 (0.38, 6.66)	1.23 (0.28, 6.10)	2.18 (0.54, 10.91)	1.94 (0.35, 12.70)	2.81 (0.60, 14.74)	1.40 (0.34, 7.43)	1.71 (0.30, 13.17)	1.86 (0.39, 10.30)	2.48 (0.54, 14.02)
RIS600mgIV	1.62 (0.58, 5.10)	0.36 (0.09, 1.22)	0.79 (0.27, 2.40)	0.61 (0.19, 1.90)	0.74 (0.35, 1.47)	0.62 (0.23, 1.76)	1.12 (0.44, 2.90)	0.98 (0.28, 4.24)	1.41 (0.50, 4.21)	0.72 (0.28, 1.91)	0.87 (0.22, 3.86)	0.95 (0.32, 2.85)	1.26 (0.45, 3.74)
SEC10mg/kgIV	6.00 (0.82, 45.66)	1.31 (0.16, 10.21)	2.95 (0.38, 21.67)	2.24 (0.32, 17.82)	2.66 (0.43, 16.46)	2.25 (0.32, 15.89)	4.08 (0.62, 27.00)	3.57 (0.47, 30.98)	5.23 (0.71, 37.11)	2.65 (0.38, 18.09)	3.23 (0.35, 30.14)	3.35 (0.48, 25.64)	4.55 (0.67, 31.92)
TES400mg200mgSC	TES400mg200mgSC	**0.22 (0.04, 0.98)**	0.48 (0.11, 2.00)	0.37 (0.08, 1.59)	0.46 (0.14, 1.32)	0.38 (0.10, 1.40)	0.68 (0.19, 2.49)	0.60 (0.12, 3.23)	0.86 (0.22, 3.39)	0.44 (0.12, 1.70)	0.54 (0.10, 2.90)	0.58 (0.14, 2.35)	0.77 (0.20, 2.95)
TOF15mgPO_BID	**4.55 (1.02, 23.22)**	TOF15mgPO_BID	2.20 (0.77, 7.08)	1.69 (0.55, 5.69)	2.03 (0.60, 8.07)	1.72 (0.42, 8.25)	3.11 (0.79, 14.20)	2.79 (0.52, 16.16)	3.96 (0.93, 19.37)	2.00 (0.50, 9.15)	2.44 (0.43, 16.02)	2.66 (0.58, 12.06)	3.54 (0.80, 15.72)
TOF1mgPO_BID	2.08 (0.50, 8.74)	0.45 (0.14, 1.30)	TOF1mgPO_BID	0.78 (0.29, 1.84)	0.93 (0.30, 2.79)	0.78 (0.22, 2.85)	1.42 (0.40, 5.01)	1.24 (0.25, 6.13)	1.76 (0.48, 7.24)	0.91 (0.24, 3.42)	1.09 (0.22, 6.42)	1.18 (0.30, 4.61)	1.61 (0.42, 6.04)
TOF5mgPO_BID	2.68 (0.63, 11.89)	0.59 (0.18, 1.80)	1.29 (0.54, 3.49)	TOF5mgPO_BID	1.21 (0.36, 3.88)	1.01 (0.26, 4.21)	1.81 (0.51, 6.90)	1.59 (0.33, 8.74)	2.30 (0.58, 10.00)	1.17 (0.30, 4.92)	1.45 (0.27, 8.24)	1.58 (0.38, 6.43)	2.09 (0.53, 8.46)
UPA45mgPO	2.18 (0.76, 7.04)	0.49 (0.12, 1.67)	1.08 (0.36, 3.34)	0.82 (0.26, 2.75)	UPA45mgPO	0.84 (0.30, 2.51)	1.51 (0.58, 4.33)	1.34 (0.37, 6.04)	1.92 (0.65, 5.88)	0.98 (0.35, 2.95)	1.17 (0.29, 5.44)	1.28 (0.43, 4.18)	1.70 (0.62, 5.33)
UST1mgIV	2.65 (0.71, 9.81)	0.58 (0.12, 2.38)	1.29 (0.35, 4.65)	0.99 (0.24, 3.80)	1.19 (0.40, 3.35)	UST1mgIV	1.80 (0.84, 4.04)	1.57 (0.36, 7.92)	2.28 (0.64, 7.99)	1.16 (0.51, 2.69)	1.39 (0.28, 7.40)	1.53 (0.41, 5.75)	2.02 (0.57, 7.60)
UST3mgIV	1.48 (0.40, 5.36)	0.32 (0.07, 1.27)	0.70 (0.20, 2.48)	0.55 (0.14, 1.94)	0.66 (0.23, 1.73)	0.55 (0.25, 1.19)	UST3mgIV	0.87 (0.21, 3.98)	1.26 (0.37, 4.32)	0.64 (0.30, 1.34)	0.77 (0.17, 4.00)	0.86 (0.23, 3.10)	1.12 (0.34, 3.95)
UST4.5mg/kgIV	1.67 (0.31, 8.11)	0.36 (0.06, 1.94)	0.81 (0.16, 3.94)	0.63 (0.11, 3.05)	0.74 (0.17, 2.72)	0.64 (0.13, 2.75)	1.14 (0.25, 4.66)	UST4.5mg/kgIV	1.44 (0.26, 6.77)	0.73 (0.15, 3.10)	0.90 (0.15, 5.13)	0.98 (0.19, 4.58)	1.30 (0.26, 5.74)
UST6mg/kg90mgIV/SC	1.16 (0.30, 4.47)	0.25 (0.05, 1.07)	0.57 (0.14, 2.09)	0.43 (0.10, 1.74)	0.52 (0.17, 1.54)	0.44 (0.13, 1.56)	0.79 (0.23, 2.68)	0.69 (0.15, 3.81)	UST6mg/kg90mgIV/SC	0.50 (0.15, 1.82)	0.60 (0.13, 3.36)	0.67 (0.17, 2.65)	0.88 (0.24, 3.44)
UST6mgIV	2.27 (0.59, 8.43)	0.50 (0.11, 1.99)	1.10 (0.29, 4.13)	0.86 (0.20, 3.28)	1.02 (0.34, 2.88)	0.86 (0.37, 1.97)	1.56 (0.74, 3.30)	1.36 (0.32, 6.59)	1.99 (0.55, 6.66)	UST6mgIV	1.20 (0.25, 6.77)	1.33 (0.35, 5.13)	1.74 (0.50, 6.29)
UST90mgSC	1.85 (0.34, 9.99)	0.41 (0.06, 2.33)	0.91 (0.16, 4.61)	0.69 (0.12, 3.72)	0.85 (0.18, 3.41)	0.72 (0.14, 3.52)	1.29 (0.25, 5.93)	1.11 (0.20, 6.78)	1.67 (0.30, 7.61)	0.83 (0.15, 3.97)	UST90mgSC	1.08 (0.19, 5.44)	1.43 (0.27, 7.44)
VED0.5mg/kgIV	1.72 (0.43, 7.15)	0.38 (0.08, 1.71)	0.85 (0.22, 3.32)	0.63 (0.16, 2.65)	0.78 (0.24, 2.31)	0.65 (0.17, 2.47)	1.17 (0.32, 4.27)	1.02 (0.22, 5.40)	1.49 (0.38, 6.04)	0.75 (0.19, 2.87)	0.93 (0.18, 5.27)	VED0.5mg/kgIV	1.32 (0.63, 3.00)
VED2mg/kgIV	1.31 (0.34, 5.07)	0.28 (0.06, 1.24)	0.62 (0.17, 2.40)	0.48 (0.12, 1.87)	0.59 (0.19, 1.61)	0.49 (0.13, 1.75)	0.89 (0.25, 2.95)	0.77 (0.17, 3.92)	1.13 (0.29, 4.15)	0.58 (0.16, 2.02)	0.70 (0.13, 3.73)	0.76 (0.33, 1.59)	VED2mg/kgIV

Table S12H: League table for clinical responses at 6 weeks of treatment.

	API100mgPO	API50mgPO	BRI400mgIV	BRI700mgIV	BRO210mgIV	BRO350mgIV	BRO700mgIV	CER100mgSC	CER200mgSC	CER400mgSC	FON1mg/kg0.1mgIV/SC	FON1mg/kg1mgIV/SC	FON4mg/kg0.1mgIV/SC	FON4mg/kg1mgIV/SC	NAT300mgIV	NAT3mg/kgIV
API100mgPO	API100mgPO	0.75 (0.28, 1.83)	2.23 (0.50, 11.29)	3.06 (0.82, 13.64)	1.53 (0.03, 40.27)	**10.01 (1.08, 217.45)**	5.74 (0.50, 129.99)	1.88 (0.70, 5.37)	1.31 (0.46, 3.91)	1.82 (0.78, 4.53)	1.73 (0.52, 6.28)	2.53 (0.79, 9.09)	2.62 (0.82, 8.79)	1.97 (0.60, 7.15)	2.01 (0.78, 5.23)	1.51 (0.52, 4.35)
API50mgPO	1.34 (0.55, 3.51)	API50mgPO	3.01 (0.60, 16.49)	**4.16 (1.03, 19.00)**	2.04 (0.04, 61.13)	**13.76 (1.47, 307.75)**	7.83 (0.71, 169.19)	2.56 (0.92, 7.96)	1.78 (0.61, 5.69)	**2.46 (1.02, 6.54)**	2.32 (0.65, 9.08)	**3.42 (1.03, 12.98)**	**3.51 (1.07, 12.70)**	2.64 (0.79, 9.73)	**2.67 (1.04, 7.45)**	2.04 (0.70, 6.16)
BRI400mgIV	0.45 (0.09, 2.02)	0.33 (0.06, 1.66)	BRI400mgIV	1.34 (0.58, 3.89)	0.65 (0.01, 22.72)	4.41 (0.38, 124.93)	2.53 (0.18, 72.01)	0.84 (0.18, 3.71)	0.59 (0.12, 2.64)	0.82 (0.20, 3.16)	0.78 (0.14, 3.92)	1.13 (0.23, 5.71)	1.17 (0.24, 5.49)	0.88 (0.17, 4.72)	0.88 (0.20, 3.61)	0.68 (0.14, 2.97)
BRI700mgIV	0.33 (0.07, 1.22)	**0.24 (0.05, 0.97)**	0.75 (0.26, 1.73)	BRI700mgIV	0.49 (0.01, 15.30)	3.21 (0.28, 75.87)	1.86 (0.13, 45.39)	0.62 (0.15, 2.11)	0.44 (0.10, 1.54)	0.60 (0.16, 1.84)	0.58 (0.12, 2.25)	0.84 (0.19, 3.30)	0.86 (0.19, 3.39)	0.64 (0.14, 2.60)	0.66 (0.16, 2.07)	0.50 (0.12, 1.72)
BRO210mgIV	0.66 (0.02, 30.99)	0.49 (0.02, 24.06)	1.54 (0.04, 77.16)	2.05 (0.07, 102.47)	BRO210mgIV	6.71 (0.83, 229.93)	3.81 (0.41, 164.33)	1.28 (0.05, 56.82)	0.90 (0.03, 35.93)	1.22 (0.05, 51.68)	1.14 (0.04, 55.02)	1.70 (0.06, 78.04)	1.73 (0.06, 86.48)	1.32 (0.05, 66.94)	1.31 (0.05, 59.81)	1.01 (0.04, 45.02)
BRO350mgIV	**0.10 (0.00, 0.92)**	**0.07 (0.00, 0.68)**	0.23 (0.01, 2.64)	0.31 (0.01, 3.51)	0.15 (0.00, 1.21)	BRO350mgIV	0.58 (0.11, 2.53)	0.20 (0.01, 1.55)	0.14 (0.01, 1.12)	0.19 (0.01, 1.42)	0.17 (0.01, 1.60)	0.26 (0.01, 2.53)	0.27 (0.01, 2.48)	0.19 (0.01, 1.95)	0.21 (0.01, 1.61)	0.16 (0.01, 1.33)
BRO700mgIV	0.17 (0.01, 2.01)	0.13 (0.01, 1.42)	0.40 (0.01, 5.49)	0.54 (0.02, 7.58)	0.26 (0.01, 2.44)	1.73 (0.40, 9.00)	BRO700mgIV	0.33 (0.01, 3.43)	0.23 (0.01, 2.46)	0.32 (0.02, 3.23)	0.30 (0.01, 3.55)	0.45 (0.02, 5.65)	0.46 (0.02, 5.24)	0.35 (0.02, 4.10)	0.35 (0.02, 3.78)	0.26 (0.01, 2.99)
CER100mgSC	0.53 (0.19, 1.44)	0.39 (0.13, 1.08)	1.19 (0.27, 5.48)	1.62 (0.47, 6.68)	0.78 (0.02, 21.81)	5.11 (0.65, 116.27)	3.05 (0.29, 70.38)	CER100mgSC	0.70 (0.32, 1.44)	0.96 (0.53, 1.85)	0.91 (0.31, 2.82)	1.36 (0.45, 4.11)	1.38 (0.49, 4.12)	1.04 (0.34, 3.23)	1.05 (0.46, 2.33)	0.80 (0.31, 2.00)
CER200mgSC	0.76 (0.26, 2.17)	0.56 (0.18, 1.65)	1.69 (0.38, 8.16)	2.30 (0.65, 9.77)	1.11 (0.03, 31.45)	7.40 (0.90, 153.45)	4.42 (0.41, 101.02)	1.43 (0.69, 3.10)	CER200mgSC	1.38 (0.71, 2.81)	1.33 (0.41, 4.13)	1.93 (0.63, 5.92)	2.00 (0.69, 6.01)	1.49 (0.48, 4.91)	1.51 (0.66, 3.42)	1.15 (0.43, 2.98)
CER400mgSC	0.55 (0.22, 1.28)	**0.41 (0.15, 0.98)**	1.22 (0.32, 4.95)	1.67 (0.54, 6.38)	0.82 (0.02, 20.90)	5.23 (0.70, 106.67)	3.16 (0.31, 63.33)	1.05 (0.54, 1.87)	0.73 (0.36, 1.41)	CER400mgSC	0.94 (0.36, 2.50)	1.39 (0.53, 3.66)	1.42 (0.60, 3.66)	1.08 (0.41, 3.05)	1.10 (0.59, 1.90)	0.83 (0.38, 1.71)
FON1mg/kg0.1mgIV/SC	0.58 (0.16, 1.91)	0.43 (0.11, 1.53)	1.29 (0.26, 7.01)	1.74 (0.45, 8.51)	0.88 (0.02, 27.68)	5.77 (0.63, 143.81)	3.32 (0.28, 80.67)	1.10 (0.35, 3.25)	0.75 (0.24, 2.43)	1.07 (0.40, 2.79)	FON1mg/kg0.1mgIV/SC	1.46 (0.67, 3.46)	1.51 (0.73, 3.41)	1.14 (0.47, 2.72)	1.15 (0.41, 3.23)	0.88 (0.28, 2.74)
FON1mg/kg1mgIV/SC	0.39 (0.11, 1.26)	**0.29 (0.08, 0.98)**	0.88 (0.18, 4.33)	1.19 (0.30, 5.17)	0.59 (0.01, 17.50)	3.90 (0.40, 94.81)	2.24 (0.18, 50.24)	0.74 (0.24, 2.21)	0.52 (0.17, 1.60)	0.72 (0.27, 1.89)	0.68 (0.29, 1.48)	FON1mg/kg1mgIV/SC	1.02 (0.51, 2.14)	0.77 (0.35, 1.66)	0.78 (0.28, 2.06)	0.59 (0.19, 1.81)
FON4mg/kg0.1mgIV/SC	0.38 (0.11, 1.22)	**0.29 (0.08, 0.93)**	0.85 (0.18, 4.13)	1.16 (0.30, 5.29)	0.58 (0.01, 16.59)	3.73 (0.40, 91.06)	2.17 (0.19, 48.16)	0.73 (0.24, 2.03)	0.50 (0.17, 1.46)	0.70 (0.27, 1.67)	0.66 (0.29, 1.37)	0.98 (0.47, 1.94)	FON4mg/kg0.1mgIV/SC	0.76 (0.34, 1.60)	0.76 (0.28, 1.94)	0.57 (0.19, 1.67)
FON4mg/kg1mgIV/SC	0.51 (0.14, 1.68)	0.38 (0.10, 1.27)	1.13 (0.21, 5.98)	1.56 (0.38, 7.14)	0.76 (0.01, 21.27)	5.19 (0.51, 114.36)	2.89 (0.24, 64.58)	0.96 (0.31, 2.93)	0.67 (0.20, 2.08)	0.93 (0.33, 2.43)	0.88 (0.37, 2.11)	1.30 (0.60, 2.85)	1.32 (0.63, 2.94)	FON4mg/kg1mgIV/SC	1.02 (0.36, 2.77)	0.77 (0.25, 2.38)
NAT300mgIV	0.50 (0.19, 1.28)	**0.37 (0.13, 0.96)**	1.13 (0.28, 5.06)	1.52 (0.48, 6.09)	0.76 (0.02, 19.54)	4.85 (0.62, 101.43)	2.84 (0.26, 60.68)	0.95 (0.43, 2.17)	0.66 (0.29, 1.53)	0.91 (0.53, 1.68)	0.87 (0.31, 2.45)	1.29 (0.49, 3.61)	1.32 (0.51, 3.54)	0.99 (0.36, 2.80)	NAT300mgIV	0.76 (0.33, 1.69)
NAT3mg/kgIV	0.66 (0.23, 1.92)	0.49 (0.16, 1.43)	1.48 (0.34, 7.14)	2.00 (0.58, 8.62)	0.99 (0.02, 27.35)	6.39 (0.75, 142.71)	3.77 (0.33, 86.11)	1.24 (0.50, 3.20)	0.87 (0.34, 2.33)	1.21 (0.58, 2.62)	1.14 (0.37, 3.52)	1.70 (0.55, 5.18)	1.74 (0.60, 5.28)	1.31 (0.42, 4.01)	1.31 (0.59, 2.99)	NAT3mg/kgIV
NAT3mg/kgIVx2	0.44 (0.16, 1.25)	**0.33 (0.11, 0.91)**	0.98 (0.23, 4.56)	1.35 (0.40, 5.68)	0.65 (0.02, 18.83)	4.25 (0.50, 96.06)	2.47 (0.22, 57.25)	0.83 (0.34, 2.09)	0.58 (0.23, 1.47)	0.81 (0.41, 1.64)	0.76 (0.25, 2.34)	1.12 (0.38, 3.37)	1.15 (0.42, 3.40)	0.87 (0.29, 2.71)	0.88 (0.39, 1.87)	0.67 (0.37, 1.21)
NAT6mg/kgIVx2	0.63 (0.21, 1.77)	0.47 (0.15, 1.35)	1.41 (0.32, 6.97)	1.89 (0.55, 8.73)	0.94 (0.02, 26.45)	6.05 (0.69, 140.40)	3.62 (0.31, 81.09)	1.18 (0.47, 3.01)	0.83 (0.31, 2.21)	1.15 (0.55, 2.52)	1.09 (0.36, 3.39)	1.60 (0.52, 5.02)	1.65 (0.55, 4.84)	1.24 (0.41, 3.89)	1.25 (0.54, 2.95)	0.95 (0.49, 1.88)
PBO	0.73 (0.31, 1.62)	0.53 (0.22, 1.23)	1.62 (0.44, 6.53)	2.19 (0.77, 7.85)	1.08 (0.03, 26.50)	6.92 (0.93, 142.86)	4.09 (0.41, 81.89)	1.37 (0.70, 2.60)	0.95 (0.47, 1.91)	1.31 (0.98, 1.88)	1.24 (0.51, 3.16)	1.83 (0.77, 4.71)	1.88 (0.84, 4.62)	1.42 (0.58, 3.72)	1.43 (0.92, 2.31)	1.10 (0.56, 2.12)
SEC10mg/kgIV	0.97 (0.16, 5.53)	0.71 (0.12, 4.63)	2.13 (0.27, 17.40)	3.04 (0.46, 22.35)	1.43 (0.03, 48.46)	9.74 (0.79, 268.36)	5.77 (0.36, 158.39)	1.84 (0.35, 10.10)	1.30 (0.22, 7.12)	1.79 (0.35, 9.03)	1.68 (0.26, 11.36)	2.47 (0.39, 15.85)	2.54 (0.41, 15.63)	1.95 (0.30, 12.95)	1.94 (0.37, 10.14)	1.50 (0.28, 8.30)
TES400mg200mgSC	**0.20 (0.05, 0.70)**	**0.15 (0.04, 0.55)**	0.46 (0.08, 2.32)	0.62 (0.14, 3.08)	0.30 (0.01, 9.59)	2.02 (0.20, 46.74)	1.18 (0.10, 27.31)	0.39 (0.12, 1.22)	**0.27 (0.07, 0.88)**	0.38 (0.12, 1.01)	0.35 (0.10, 1.26)	0.52 (0.14, 1.88)	0.54 (0.15, 1.84)	0.40 (0.10, 1.45)	0.42 (0.13, 1.15)	**0.31 (0.09, 0.96)**
UST130mgIV	0.45 (0.18, 1.07)	**0.33 (0.12, 0.84)**	1.00 (0.25, 4.20)	1.35 (0.46, 4.92)	0.67 (0.02, 17.20)	4.28 (0.56, 93.84)	2.56 (0.25, 52.45)	0.84 (0.39, 1.78)	0.59 (0.26, 1.35)	0.81 (0.50, 1.38)	0.77 (0.29, 2.17)	1.12 (0.44, 3.16)	1.17 (0.47, 3.03)	0.87 (0.33, 2.51)	0.88 (0.50, 1.65)	0.68 (0.31, 1.47)
UST1mg/kgIV	0.36 (0.13, 1.00)	**0.27 (0.09, 0.79)**	0.81 (0.19, 3.67)	1.11 (0.32, 4.43)	0.55 (0.01, 15.08)	3.60 (0.43, 78.40)	2.06 (0.19, 47.11)	0.68 (0.28, 1.79)	0.48 (0.19, 1.26)	0.66 (0.32, 1.44)	0.63 (0.21, 2.01)	0.92 (0.32, 2.94)	0.95 (0.34, 2.90)	0.71 (0.25, 2.29)	0.72 (0.34, 1.60)	0.55 (0.22, 1.43)
UST3mg/kgIV	0.36 (0.13, 1.04)	**0.27 (0.09, 0.78)**	0.81 (0.19, 3.91)	1.11 (0.32, 4.68)	0.53 (0.01, 15.55)	3.56 (0.43, 78.51)	2.07 (0.19, 46.97)	0.68 (0.28, 1.75)	0.48 (0.19, 1.26)	0.65 (0.34, 1.45)	0.63 (0.21, 1.99)	0.92 (0.32, 2.92)	0.95 (0.34, 2.86)	0.72 (0.24, 2.31)	0.72 (0.34, 1.66)	0.55 (0.22, 1.43)
UST4.5mg/kgIV	0.33 (0.06, 1.66)	0.25 (0.04, 1.24)	0.73 (0.09, 5.56)	1.01 (0.15, 6.90)	0.49 (0.01, 17.07)	3.23 (0.25, 80.07)	1.92 (0.12, 50.17)	0.62 (0.11, 2.73)	0.44 (0.08, 1.99)	0.61 (0.12, 2.46)	0.57 (0.10, 2.91)	0.84 (0.14, 4.19)	0.87 (0.15, 4.37)	0.66 (0.11, 3.39)	0.66 (0.13, 2.79)	0.50 (0.09, 2.34)
UST6mg/kgIV	**0.39 (0.16, 0.93)**	**0.29 (0.11, 0.72)**	0.87 (0.22, 3.71)	1.18 (0.39, 4.30)	0.58 (0.01, 15.50)	3.77 (0.49, 79.05)	2.20 (0.22, 46.85)	0.74 (0.35, 1.55)	0.51 (0.24, 1.11)	0.70 (0.45, 1.22)	0.67 (0.25, 1.92)	0.98 (0.38, 2.75)	1.01 (0.42, 2.71)	0.76 (0.29, 2.18)	0.77 (0.44, 1.38)	0.59 (0.28, 1.25)
UST90mgSC	0.58 (0.14, 2.24)	0.43 (0.10, 1.63)	1.29 (0.24, 7.05)	1.78 (0.36, 9.43)	0.86 (0.02, 26.81)	5.82 (0.59, 132.04)	3.29 (0.28, 82.29)	1.10 (0.30, 3.94)	0.78 (0.19, 2.85)	1.07 (0.32, 3.26)	1.02 (0.25, 4.04)	1.50 (0.36, 6.12)	1.53 (0.38, 5.61)	1.16 (0.28, 4.52)	1.18 (0.33, 3.82)	0.89 (0.23, 3.09)
VED0.5mg/kgIV	0.47 (0.15, 1.40)	0.35 (0.10, 1.07)	1.05 (0.23, 5.03)	1.43 (0.38, 6.11)	0.71 (0.02, 19.46)	4.66 (0.50, 103.56)	2.71 (0.24, 64.56)	0.89 (0.32, 2.42)	0.62 (0.22, 1.73)	0.87 (0.36, 1.97)	0.81 (0.24, 2.72)	1.20 (0.38, 4.03)	1.22 (0.39, 3.95)	0.92 (0.28, 3.02)	0.95 (0.36, 2.24)	0.71 (0.25, 1.92)
VED2mg/kgIV	0.46 (0.15, 1.37)	0.34 (0.11, 1.06)	1.04 (0.23, 4.87)	1.40 (0.40, 6.37)	0.69 (0.02, 19.78)	4.55 (0.52, 99.11)	2.69 (0.23, 62.68)	0.88 (0.34, 2.38)	0.61 (0.22, 1.76)	0.85 (0.38, 1.95)	0.81 (0.25, 2.68)	1.18 (0.37, 3.72)	1.22 (0.40, 3.85)	0.92 (0.28, 3.06)	0.93 (0.38, 2.27)	0.70 (0.26, 1.86)
VED300mgIV	0.51 (0.19, 1.29)	0.37 (0.13, 1.01)	1.10 (0.28, 4.92)	1.54 (0.48, 5.93)	0.75 (0.02, 20.61)	4.87 (0.60, 106.05)	2.86 (0.27, 62.71)	0.95 (0.43, 2.24)	0.67 (0.28, 1.59)	0.92 (0.52, 1.79)	0.86 (0.32, 2.63)	1.28 (0.47, 3.69)	1.31 (0.52, 3.72)	0.98 (0.35, 3.00)	0.99 (0.53, 2.08)	0.76 (0.33, 1.76)

	NAT3mg/kgIVx2	NAT6mg/kgIVx2	PBO	SEC10mg/kgIV	TES400mg200mgSC	UST130mgIV	UST1mg/kgIV	UST3mg/kgIV	UST4.5mg/kgIV	UST6mg/kgIV	UST90mgSC	VED0.5mg/kgIV	VED2mg/kgIV	VED300mgIV
API100mgPO	2.25 (0.80, 6.43)	1.58 (0.57, 4.72)	1.37 (0.62, 3.20)	1.03 (0.18, 6.27)	**4.94 (1.43, 18.47)**	2.24 (0.94, 5.56)	2.76 (1.00, 7.66)	2.74 (0.96, 7.75)	3.05 (0.60, 17.18)	**2.56 (1.08, 6.26)**	1.71 (0.45, 7.00)	2.13 (0.71, 6.62)	2.16 (0.73, 6.65)	1.98 (0.78, 5.16)
API50mgPO	**3.06 (1.10, 8.92)**	2.12 (0.74, 6.75)	1.87 (0.81, 4.62)	1.41 (0.22, 8.10)	**6.70 (1.81, 26.47)**	**3.01 (1.20, 8.19)**	**3.72 (1.27, 10.88)**	**3.70 (1.27, 11.09)**	4.08 (0.80, 25.08)	**3.48 (1.40, 9.22)**	2.33 (0.61, 9.74)	2.87 (0.93, 9.83)	2.91 (0.94, 9.47)	2.69 (0.99, 7.45)
BRI400mgIV	1.02 (0.22, 4.43)	0.71 (0.14, 3.11)	0.62 (0.15, 2.30)	0.47 (0.06, 3.68)	2.18 (0.43, 12.33)	1.00 (0.24, 4.04)	1.23 (0.27, 5.36)	1.23 (0.26, 5.36)	1.36 (0.18, 10.78)	1.14 (0.27, 4.52)	0.78 (0.14, 4.18)	0.95 (0.20, 4.29)	0.96 (0.21, 4.26)	0.91 (0.20, 3.56)
BRI700mgIV	0.74 (0.18, 2.49)	0.53 (0.11, 1.82)	0.46 (0.13, 1.29)	0.33 (0.04, 2.17)	1.62 (0.32, 7.33)	0.74 (0.20, 2.20)	0.90 (0.23, 3.10)	0.90 (0.21, 3.14)	0.99 (0.14, 6.88)	0.85 (0.23, 2.59)	0.56 (0.11, 2.75)	0.70 (0.16, 2.64)	0.71 (0.16, 2.51)	0.65 (0.17, 2.07)
BRO210mgIV	1.53 (0.05, 65.17)	1.06 (0.04, 46.50)	0.92 (0.04, 38.14)	0.70 (0.02, 36.85)	3.36 (0.10, 160.52)	1.49 (0.06, 64.77)	1.82 (0.07, 78.92)	1.87 (0.06, 81.17)	2.05 (0.06, 97.20)	1.74 (0.06, 74.14)	1.17 (0.04, 54.22)	1.41 (0.05, 62.53)	1.44 (0.05, 66.64)	1.33 (0.05, 57.18)
BRO350mgIV	0.24 (0.01, 1.99)	0.17 (0.01, 1.45)	0.14 (0.01, 1.07)	0.10 (0.00, 1.27)	0.49 (0.02, 4.99)	0.23 (0.01, 1.80)	0.28 (0.01, 2.31)	0.28 (0.01, 2.33)	0.31 (0.01, 4.01)	0.27 (0.01, 2.05)	0.17 (0.01, 1.70)	0.21 (0.01, 1.99)	0.22 (0.01, 1.91)	0.21 (0.01, 1.66)
BRO700mgIV	0.40 (0.02, 4.53)	0.28 (0.01, 3.21)	0.24 (0.01, 2.42)	0.17 (0.01, 2.77)	0.85 (0.04, 9.95)	0.39 (0.02, 4.08)	0.48 (0.02, 5.23)	0.48 (0.02, 5.24)	0.52 (0.02, 8.28)	0.45 (0.02, 4.64)	0.30 (0.01, 3.64)	0.37 (0.02, 4.09)	0.37 (0.02, 4.35)	0.35 (0.02, 3.68)
CER100mgSC	1.21 (0.48, 2.91)	0.85 (0.33, 2.13)	0.73 (0.39, 1.43)	0.54 (0.10, 2.85)	2.60 (0.82, 8.68)	1.19 (0.56, 2.53)	1.46 (0.56, 3.61)	1.46 (0.57, 3.58)	1.60 (0.37, 8.85)	1.36 (0.65, 2.89)	0.91 (0.25, 3.37)	1.12 (0.41, 3.10)	1.14 (0.42, 2.98)	1.05 (0.45, 2.35)
CER200mgSC	1.72 (0.68, 4.38)	1.20 (0.45, 3.27)	1.05 (0.52, 2.13)	0.77 (0.14, 4.45)	**3.70 (1.14, 13.45)**	1.70 (0.74, 3.79)	2.07 (0.79, 5.37)	2.08 (0.79, 5.37)	2.29 (0.50, 12.84)	1.96 (0.90, 4.24)	1.28 (0.35, 5.19)	1.60 (0.58, 4.62)	1.63 (0.57, 4.53)	1.50 (0.63, 3.57)
CER400mgSC	1.24 (0.61, 2.47)	0.87 (0.40, 1.82)	0.76 (0.53, 1.02)	0.56 (0.11, 2.88)	2.63 (0.99, 8.10)	1.23 (0.72, 1.99)	1.52 (0.70, 3.08)	1.53 (0.69, 2.94)	1.64 (0.41, 8.44)	1.42 (0.82, 2.21)	0.93 (0.31, 3.15)	1.16 (0.51, 2.78)	1.18 (0.51, 2.65)	1.09 (0.56, 1.91)
FON1mg/kg0.1mgIV/SC	1.31 (0.43, 3.95)	0.92 (0.30, 2.81)	0.81 (0.32, 1.95)	0.59 (0.09, 3.78)	2.83 (0.80, 10.43)	1.30 (0.46, 3.43)	1.58 (0.50, 4.69)	1.59 (0.50, 4.70)	1.74 (0.34, 10.40)	1.50 (0.52, 3.96)	0.99 (0.25, 4.05)	1.24 (0.37, 4.11)	1.24 (0.37, 4.01)	1.16 (0.38, 3.13)
FON1mg/kg1mgIV/SC	0.89 (0.30, 2.64)	0.63 (0.20, 1.93)	0.55 (0.21, 1.30)	0.41 (0.06, 2.53)	1.91 (0.53, 7.28)	0.89 (0.32, 2.26)	1.09 (0.34, 3.11)	1.08 (0.34, 3.15)	1.19 (0.24, 7.16)	1.02 (0.36, 2.60)	0.67 (0.16, 2.77)	0.84 (0.25, 2.67)	0.85 (0.27, 2.68)	0.78 (0.27, 2.15)
FON4mg/kg0.1mgIV/SC	0.87 (0.29, 2.37)	0.61 (0.21, 1.81)	0.53 (0.22, 1.19)	0.39 (0.06, 2.43)	1.87 (0.54, 6.85)	0.86 (0.33, 2.12)	1.05 (0.35, 2.91)	1.06 (0.35, 2.91)	1.15 (0.23, 6.55)	0.99 (0.37, 2.37)	0.66 (0.18, 2.62)	0.82 (0.25, 2.54)	0.82 (0.26, 2.52)	0.76 (0.27, 1.94)
FON4mg/kg1mgIV/SC	1.15 (0.37, 3.47)	0.81 (0.26, 2.47)	0.70 (0.27, 1.72)	0.51 (0.08, 3.36)	2.49 (0.69, 9.80)	1.15 (0.40, 3.06)	1.40 (0.44, 4.08)	1.39 (0.43, 4.10)	1.51 (0.30, 9.16)	1.32 (0.46, 3.50)	0.86 (0.22, 3.52)	1.09 (0.33, 3.51)	1.09 (0.33, 3.51)	1.02 (0.33, 2.82)
NAT300mgIV	1.14 (0.53, 2.57)	0.80 (0.34, 1.85)	0.70 (0.43, 1.09)	0.51 (0.10, 2.70)	2.40 (0.87, 7.82)	1.13 (0.60, 2.00)	1.39 (0.62, 2.93)	1.38 (0.60, 2.91)	1.52 (0.36, 7.72)	1.30 (0.72, 2.25)	0.85 (0.26, 3.05)	1.05 (0.45, 2.76)	1.08 (0.44, 2.60)	1.01 (0.48, 1.90)
NAT3mg/kgIV	1.50 (0.83, 2.72)	1.06 (0.53, 2.06)	0.91 (0.47, 1.78)	0.67 (0.12, 3.59)	**3.19 (1.04, 10.94)**	1.48 (0.68, 3.18)	1.83 (0.70, 4.59)	1.82 (0.70, 4.53)	2.01 (0.43, 10.59)	1.70 (0.80, 3.52)	1.12 (0.32, 4.38)	1.40 (0.52, 3.99)	1.42 (0.54, 3.81)	1.31 (0.57, 3.01)
NAT3mg/kgIVx2	NAT3mg/kgIVx2	0.71 (0.37, 1.31)	0.62 (0.33, 1.11)	0.45 (0.08, 2.61)	2.12 (0.72, 7.12)	1.00 (0.47, 2.01)	1.22 (0.48, 2.87)	1.23 (0.46, 2.89)	1.34 (0.29, 7.23)	1.14 (0.55, 2.21)	0.75 (0.21, 2.88)	0.94 (0.36, 2.53)	0.95 (0.36, 2.48)	0.88 (0.39, 1.90)
NAT6mg/kgIVx2	1.41 (0.76, 2.72)	NAT6mg/kgIVx2	0.87 (0.45, 1.75)	0.65 (0.11, 3.58)	3.03 (1.00, 10.32)	1.41 (0.65, 3.04)	1.72 (0.66, 4.45)	1.73 (0.64, 4.31)	1.91 (0.41, 10.51)	1.62 (0.77, 3.45)	1.08 (0.30, 4.15)	1.33 (0.48, 3.85)	1.35 (0.50, 3.75)	1.25 (0.53, 2.88)
PBO	1.62 (0.90, 3.03)	1.15 (0.57, 2.24)	PBO	0.73 (0.16, 3.65)	**3.49 (1.39, 10.05)**	**1.62 (1.12, 2.39)**	**2.00 (1.03, 3.58)**	**2.01 (1.01, 3.70)**	2.17 (0.56, 10.67)	**1.86 (1.29, 2.60)**	1.23 (0.43, 3.92)	1.51 (0.74, 3.47)	1.55 (0.74, 3.36)	1.44 (0.85, 2.31)
SEC10mg/kgIV	2.24 (0.38, 12.40)	1.54 (0.28, 9.05)	1.36 (0.27, 6.43)	SEC10mg/kgIV	4.82 (0.79, 30.36)	2.20 (0.41, 10.82)	2.67 (0.49, 14.54)	2.66 (0.48, 14.30)	3.03 (0.35, 25.98)	2.53 (0.50, 12.53)	1.71 (0.25, 11.21)	2.10 (0.36, 11.41)	2.08 (0.37, 12.31)	1.93 (0.37, 9.53)
TES400mg200mgSC	0.47 (0.14, 1.39)	0.33 (0.10, 1.00)	**0.29 (0.10, 0.72)**	0.21 (0.03, 1.27)	TES400mg200mgSC	0.47 (0.15, 1.26)	0.57 (0.16, 1.68)	0.57 (0.16, 1.71)	0.61 (0.11, 3.71)	0.53 (0.17, 1.39)	0.35 (0.08, 1.47)	0.44 (0.12, 1.46)	0.44 (0.12, 1.48)	0.41 (0.12, 1.11)
UST130mgIV	1.00 (0.50, 2.13)	0.71 (0.33, 1.53)	**0.62 (0.42, 0.90)**	0.46 (0.09, 2.41)	2.15 (0.80, 6.69)	UST130mgIV	1.23 (0.59, 2.34)	1.23 (0.58, 2.37)	1.33 (0.33, 6.92)	1.15 (0.78, 1.60)	0.76 (0.25, 2.72)	0.94 (0.42, 2.27)	0.94 (0.42, 2.24)	0.89 (0.47, 1.65)
UST1mg/kgIV	0.82 (0.35, 2.10)	0.58 (0.22, 1.52)	**0.50 (0.28, 0.97)**	0.37 (0.07, 2.03)	1.75 (0.59, 6.17)	0.81 (0.43, 1.69)	UST1mg/kgIV	1.00 (0.52, 1.94)	1.09 (0.25, 6.16)	0.93 (0.52, 1.78)	0.63 (0.18, 2.34)	0.77 (0.30, 2.20)	0.78 (0.30, 2.18)	0.72 (0.33, 1.67)
UST3mg/kgIV	0.82 (0.35, 2.18)	0.58 (0.23, 1.56)	**0.50 (0.27, 0.99)**	0.38 (0.07, 2.10)	1.75 (0.59, 6.39)	0.81 (0.42, 1.71)	1.00 (0.52, 1.93)	UST3mg/kgIV	1.09 (0.25, 6.33)	0.93 (0.51, 1.79)	0.62 (0.18, 2.36)	0.76 (0.30, 2.14)	0.79 (0.29, 2.16)	0.72 (0.32, 1.61)
UST4.5mg/kgIV	0.75 (0.14, 3.45)	0.52 (0.10, 2.47)	0.46 (0.09, 1.78)	0.33 (0.04, 2.87)	1.63 (0.27, 9.27)	0.75 (0.14, 3.03)	0.92 (0.16, 3.98)	0.91 (0.16, 4.01)	UST4.5mg/kgIV	0.85 (0.16, 3.39)	0.55 (0.09, 3.51)	0.71 (0.12, 3.41)	0.71 (0.12, 3.28)	0.65 (0.12, 2.79)
UST6mg/kgIV	0.88 (0.45, 1.83)	0.62 (0.29, 1.30)	**0.54 (0.39, 0.77)**	0.40 (0.08, 1.98)	1.88 (0.72, 5.82)	0.87 (0.62, 1.28)	1.07 (0.56, 1.93)	1.08 (0.56, 1.95)	1.17 (0.29, 6.10)	UST6mg/kgIV	0.66 (0.21, 2.30)	0.82 (0.38, 1.95)	0.83 (0.38, 1.89)	0.78 (0.42, 1.42)
UST90mgSC	1.33 (0.35, 4.67)	0.93 (0.24, 3.32)	0.81 (0.26, 2.35)	0.58 (0.09, 4.02)	2.84 (0.68, 12.22)	1.32 (0.37, 4.07)	1.60 (0.43, 5.58)	1.61 (0.42, 5.47)	1.81 (0.29, 11.13)	1.51 (0.43, 4.65)	UST90mgSC	1.24 (0.31, 4.96)	1.26 (0.33, 4.71)	1.17 (0.33, 3.74)
VED0.5mg/kgIV	1.07 (0.39, 2.78)	0.75 (0.26, 2.08)	0.66 (0.29, 1.36)	0.48 (0.09, 2.77)	2.28 (0.69, 8.57)	1.06 (0.44, 2.36)	1.30 (0.46, 3.34)	1.31 (0.47, 3.32)	1.41 (0.29, 8.18)	1.22 (0.51, 2.64)	0.81 (0.20, 3.21)	VED0.5mg/kgIV	1.02 (0.50, 1.96)	0.95 (0.37, 2.23)
VED2mg/kgIV	1.05 (0.40, 2.76)	0.74 (0.27, 2.01)	0.64 (0.30, 1.35)	0.48 (0.08, 2.70)	2.26 (0.68, 8.02)	1.06 (0.45, 2.36)	1.29 (0.46, 3.31)	1.27 (0.46, 3.44)	1.40 (0.30, 8.28)	1.21 (0.53, 2.63)	0.79 (0.21, 3.01)	0.98 (0.51, 1.98)	VED2mg/kgIV	0.93 (0.37, 2.21)
VED300mgIV	1.14 (0.53, 2.57)	0.80 (0.35, 1.88)	0.70 (0.43, 1.18)	0.52 (0.10, 2.71)	2.43 (0.90, 8.08)	1.13 (0.61, 2.13)	1.39 (0.60, 3.01)	1.40 (0.62, 3.08)	1.54 (0.36, 8.17)	1.29 (0.70, 2.41)	0.86 (0.27, 3.07)	1.05 (0.45, 2.73)	1.08 (0.45, 2.70)	VED300mgIV

Table S12I: League table for clinical responses at 8 weeks of treatment.

	ABA10mg/kgIV	ABA30mg/kgIV	ABA3mg/kgIV	AND150mgSC1/1	AND150mgSC2.2	AND300mgSC	CDP10mg/kgIV	CER100mgSC	CER10mgIV	CER200mgSC	CER20mgIV	CER400mgSC	CER5mgIV	ETA25mgSC	FON1mg/kg0.1mgIV/SC	FON1mg/kg1mgIV/SC	FON4mg/kg0.1mgIV/SC	FON4mg/kg1mgIV/SC	GUS1200mgIV	GUS200mgIV	GUS600mgIV	MED700IV	NAT300mgIV
ABA10mg/kgIV	ABA10mg/kgIV	2.01 (0.51, 8.02)	1.20 (0.33, 4.70)	1.42 (0.27, 7.45)	1.70 (0.34, 9.20)	0.91 (0.16, 5.44)	1.44 (0.38, 5.99)	4.25 (0.93, 20.15)	1.83 (0.39, 9.40)	2.92 (0.61, 14.24)	1.37 (0.26, 7.00)	4.52 (0.99, 21.12)	1.96 (0.42, 9.47)	0.86 (0.12, 5.43)	2.80 (0.59, 17.02)	4.40 (0.95, 27.56)	**5.35 (1.16, 29.10)**	2.76 (0.55, 17.09)	3.95 (0.92, 18.05)	**4.54 (1.03, 20.65)**	**4.90 (1.15, 22.49)**	3.19 (0.70, 14.80)	2.31 (0.64, 8.79)
ABA30mg/kgIV	0.50 (0.12, 1.97)	ABA30mg/kgIV	0.60 (0.17, 2.36)	0.69 (0.14, 3.91)	0.85 (0.18, 4.44)	0.46 (0.08, 2.57)	0.71 (0.19, 3.00)	2.11 (0.48, 10.13)	0.90 (0.20, 4.72)	1.47 (0.33, 7.10)	0.68 (0.14, 3.52)	2.27 (0.53, 11.03)	0.96 (0.21, 4.81)	0.43 (0.06, 2.92)	1.43 (0.31, 8.91)	2.20 (0.48, 12.87)	2.70 (0.60, 14.25)	1.36 (0.29, 8.73)	1.97 (0.48, 9.13)	2.24 (0.55, 10.25)	2.45 (0.58, 10.98)	1.59 (0.36, 7.78)	1.12 (0.33, 4.42)
ABA3mg/kgIV	0.84 (0.21, 3.02)	1.66 (0.42, 6.00)	ABA3mg/kgIV	1.13 (0.24, 6.35)	1.39 (0.29, 8.00)	0.74 (0.14, 4.31)	1.19 (0.32, 4.74)	3.49 (0.81, 16.19)	1.49 (0.29, 7.57)	2.44 (0.53, 11.81)	1.11 (0.21, 5.73)	3.74 (0.87, 16.89)	1.61 (0.34, 7.97)	0.71 (0.10, 4.38)	2.30 (0.49, 13.23)	3.65 (0.82, 21.66)	**4.42 (1.01, 23.09)**	2.27 (0.49, 13.53)	3.32 (0.79, 13.98)	3.77 (0.90, 16.13)	4.08 (0.97, 17.30)	2.61 (0.61, 12.28)	1.89 (0.56, 6.93)
AND150mgSC1/1	0.71 (0.13, 3.75)	1.45 (0.26, 7.17)	0.88 (0.16, 4.21)	AND150mgSC1/1	1.22 (0.48, 3.14)	0.66 (0.21, 1.90)	1.04 (0.29, 3.44)	3.02 (0.74, 11.93)	1.29 (0.28, 5.29)	2.12 (0.47, 8.60)	0.96 (0.21, 4.21)	3.27 (0.78, 12.97)	1.38 (0.33, 5.54)	0.62 (0.08, 3.40)	2.05 (0.45, 9.16)	3.19 (0.71, 14.46)	3.91 (0.89, 17.25)	1.96 (0.41, 9.23)	2.83 (0.69, 10.92)	3.21 (0.80, 12.57)	3.49 (0.87, 13.21)	2.29 (0.54, 9.29)	1.66 (0.50, 5.13)
AND150mgSC2/2	0.59 (0.11, 2.92)	1.18 (0.23, 5.64)	0.72 (0.12, 3.40)	0.82 (0.32, 2.09)	AND150mgSC2.2	0.54 (0.18, 1.48)	0.85 (0.24, 2.71)	2.46 (0.61, 9.60)	1.06 (0.23, 4.53)	1.72 (0.41, 6.59)	0.78 (0.17, 3.41)	2.66 (0.66, 10.34)	1.15 (0.27, 4.37)	0.50 (0.07, 2.91)	1.69 (0.37, 7.55)	2.63 (0.59, 11.10)	3.25 (0.75, 13.58)	1.63 (0.34, 7.51)	2.32 (0.57, 8.56)	2.67 (0.67, 9.47)	2.88 (0.74, 10.08)	1.88 (0.43, 7.47)	1.37 (0.43, 3.96)
AND300mgSC	1.10 (0.18, 6.24)	2.19 (0.39, 11.97)	1.34 (0.23, 7.07)	1.52 (0.53, 4.70)	1.87 (0.67, 5.64)	AND300mgSC	1.60 (0.41, 6.42)	**4.70 (1.08, 20.99)**	2.01 (0.40, 9.53)	3.22 (0.70, 14.83)	1.47 (0.29, 7.36)	**5.01 (1.14, 23.39)**	2.12 (0.46, 9.57)	0.94 (0.12, 6.16)	3.13 (0.63, 15.65)	**4.95 (1.01, 24.22)**	**5.94 (1.28, 28.99)**	3.08 (0.61, 15.69)	4.34 (1.00, 18.91)	**4.96 (1.15, 21.35)**	**5.33 (1.26, 23.57)**	3.50 (0.77, 16.07)	2.54 (0.71, 8.91)
CDP10mg/kgIV	0.69 (0.17, 2.66)	1.41 (0.33, 5.17)	0.84 (0.21, 3.08)	0.96 (0.29, 3.48)	1.17 (0.37, 4.18)	0.63 (0.16, 2.43)	CDP10mg/kgIV	**2.91 (1.03, 8.84)**	1.25 (0.38, 4.14)	2.03 (0.68, 6.15)	0.93 (0.27, 3.20)	**3.16 (1.11, 9.27)**	1.35 (0.41, 4.33)	0.60 (0.10, 2.92)	1.99 (0.57, 6.86)	3.08 (0.96, 10.54)	**3.78 (1.24, 12.37)**	1.89 (0.55, 7.70)	**2.72 (1.01, 7.83)**	**3.13 (1.15, 8.65)**	**3.38 (1.26, 9.54)**	2.19 (0.73, 6.87)	1.59 (0.77, 3.31)
CER100mgSC	0.24 (0.05, 1.07)	0.47 (0.10, 2.08)	0.29 (0.06, 1.24)	0.33 (0.08, 1.36)	0.41 (0.10, 1.65)	**0.21 (0.05, 0.93)**	**0.34 (0.11, 0.98)**	CER100mgSC	0.43 (0.11, 1.59)	0.70 (0.32, 1.46)	0.32 (0.08, 1.25)	1.08 (0.54, 2.18)	0.46 (0.12, 1.65)	0.20 (0.03, 1.08)	0.68 (0.17, 2.64)	1.04 (0.28, 4.32)	1.28 (0.36, 4.79)	0.65 (0.17, 2.90)	0.94 (0.29, 3.04)	1.06 (0.33, 3.41)	1.16 (0.36, 3.68)	0.75 (0.22, 2.68)	0.54 (0.20, 1.38)
CER10mgIV	0.55 (0.11, 2.56)	1.11 (0.21, 4.90)	0.67 (0.13, 3.41)	0.77 (0.19, 3.52)	0.95 (0.22, 4.34)	0.50 (0.10, 2.52)	0.80 (0.24, 2.64)	2.35 (0.63, 9.36)	CER10mgIV	1.62 (0.43, 6.60)	0.75 (0.25, 2.29)	2.50 (0.66, 9.70)	1.07 (0.40, 3.05)	0.48 (0.07, 2.77)	1.58 (0.36, 7.43)	2.43 (0.60, 11.67)	2.93 (0.76, 12.95)	1.51 (0.36, 7.45)	2.16 (0.62, 8.07)	2.51 (0.70, 9.18)	2.68 (0.76, 9.98)	1.75 (0.47, 6.89)	1.26 (0.43, 3.74)
CER200mgSC	0.34 (0.07, 1.63)	0.68 (0.14, 3.07)	0.41 (0.08, 1.87)	0.47 (0.12, 2.11)	0.58 (0.15, 2.44)	0.31 (0.07, 1.44)	0.49 (0.16, 1.46)	1.43 (0.69, 3.09)	0.62 (0.15, 2.35)	CER200mgSC	0.46 (0.11, 1.87)	1.54 (0.76, 3.30)	0.66 (0.18, 2.42)	0.29 (0.05, 1.59)	0.97 (0.24, 3.98)	1.51 (0.38, 6.14)	1.83 (0.51, 7.05)	0.93 (0.23, 4.09)	1.34 (0.41, 4.44)	1.53 (0.46, 5.10)	1.65 (0.51, 5.41)	1.07 (0.31, 4.15)	0.78 (0.29, 2.06)
CER20mgIV	0.73 (0.14, 3.80)	1.46 (0.28, 7.20)	0.90 (0.17, 4.76)	1.04 (0.24, 4.85)	1.28 (0.29, 5.74)	0.68 (0.14, 3.40)	1.07 (0.31, 3.76)	3.12 (0.80, 13.22)	1.33 (0.44, 4.07)	2.19 (0.53, 9.19)	CER20mgIV	3.36 (0.84, 14.10)	1.41 (0.53, 4.18)	0.64 (0.09, 3.82)	2.11 (0.48, 10.08)	3.30 (0.74, 14.63)	4.05 (0.99, 17.05)	2.03 (0.47, 10.43)	2.92 (0.81, 11.36)	3.34 (0.91, 12.53)	3.58 (0.98, 13.37)	2.35 (0.61, 9.59)	1.70 (0.54, 5.35)
CER400mgSC	0.22 (0.05, 1.01)	0.44 (0.09, 1.87)	0.27 (0.06, 1.15)	0.31 (0.08, 1.29)	0.38 (0.10, 1.51)	**0.20 (0.04, 0.87)**	**0.32 (0.11, 0.90)**	0.93 (0.46, 1.87)	0.40 (0.10, 1.51)	0.65 (0.30, 1.32)	0.30 (0.07, 1.19)	CER400mgSC	0.43 (0.11, 1.52)	0.19 (0.03, 1.03)	0.63 (0.16, 2.39)	0.97 (0.26, 3.85)	1.20 (0.33, 4.38)	0.61 (0.15, 2.68)	0.87 (0.27, 2.76)	0.99 (0.32, 3.19)	1.07 (0.34, 3.37)	0.69 (0.21, 2.44)	0.50 (0.19, 1.29)
CER5mgIV	0.51 (0.11, 2.38)	1.04 (0.21, 4.78)	0.62 (0.13, 2.96)	0.72 (0.18, 3.03)	0.87 (0.23, 3.70)	0.47 (0.10, 2.17)	0.74 (0.23, 2.46)	2.18 (0.61, 8.21)	0.94 (0.33, 2.49)	1.52 (0.41, 5.61)	0.71 (0.24, 1.90)	2.33 (0.66, 8.76)	CER5mgIV	0.45 (0.06, 2.64)	1.45 (0.37, 6.95)	2.26 (0.58, 10.30)	2.77 (0.74, 11.66)	1.41 (0.34, 7.22)	2.03 (0.59, 7.55)	2.33 (0.67, 8.50)	2.49 (0.73, 8.96)	1.64 (0.45, 6.46)	1.18 (0.43, 3.54)
ETA25mgSC	1.17 (0.18, 8.41)	2.30 (0.34, 16.73)	1.40 (0.23, 9.88)	1.62 (0.29, 11.97)	1.99 (0.34, 14.89)	1.07 (0.16, 8.35)	1.66 (0.34, 9.57)	4.98 (0.93, 30.59)	2.08 (0.36, 14.82)	3.41 (0.63, 21.26)	1.56 (0.26, 11.35)	5.27 (0.97, 33.82)	2.25 (0.38, 15.80)	ETA25mgSC	3.30 (0.58, 23.74)	5.11 (0.91, 34.67)	**6.28 (1.19, 41.73)**	3.25 (0.54, 23.82)	4.61 (0.85, 28.05)	**5.26 (1.02, 31.25)**	**5.67 (1.11, 34.53)**	3.69 (0.67, 23.51)	2.63 (0.60, 14.77)
FON1mg/kg0.1mgIV/SC	0.36 (0.06, 1.70)	0.70 (0.11, 3.25)	0.43 (0.08, 2.03)	0.49 (0.11, 2.24)	0.59 (0.13, 2.74)	0.32 (0.06, 1.58)	0.50 (0.15, 1.76)	1.48 (0.38, 5.97)	0.63 (0.13, 2.77)	1.03 (0.25, 4.14)	0.47 (0.10, 2.10)	1.58 (0.42, 6.12)	0.69 (0.14, 2.73)	0.30 (0.04, 1.73)	FON1mg/kg0.1mgIV/SC	1.56 (0.68, 3.75)	1.90 (0.84, 4.37)	0.99 (0.38, 2.43)	1.39 (0.35, 5.31)	1.59 (0.41, 5.99)	1.71 (0.43, 6.44)	1.11 (0.28, 4.51)	0.81 (0.24, 2.53)
FON1mg/kg1mgIV/SC	0.23 (0.04, 1.06)	0.45 (0.08, 2.07)	0.27 (0.05, 1.22)	0.31 (0.07, 1.41)	0.38 (0.09, 1.69)	**0.20 (0.04, 0.99)**	0.32 (0.09, 1.04)	0.96 (0.23, 3.54)	0.41 (0.09, 1.67)	0.66 (0.16, 2.60)	0.30 (0.07, 1.34)	1.03 (0.26, 3.83)	0.44 (0.10, 1.73)	0.20 (0.03, 1.10)	0.64 (0.27, 1.48)	FON1mg/kg1mgIV/SC	1.22 (0.59, 2.56)	0.63 (0.25, 1.45)	0.90 (0.23, 3.22)	1.03 (0.26, 3.66)	1.11 (0.29, 3.96)	0.71 (0.18, 2.66)	0.52 (0.15, 1.50)
FON4mg/kg0.1mgIV/SC	**0.19 (0.03, 0.86)**	0.37 (0.07, 1.67)	**0.23 (0.04, 0.99)**	0.26 (0.06, 1.12)	0.31 (0.07, 1.34)	**0.17 (0.03, 0.78)**	**0.26 (0.08, 0.81)**	0.78 (0.21, 2.79)	0.34 (0.08, 1.31)	0.55 (0.14, 1.98)	0.25 (0.06, 1.01)	0.83 (0.23, 3.01)	0.36 (0.09, 1.34)	**0.16 (0.02, 0.84)**	0.53 (0.23, 1.19)	0.82 (0.39, 1.70)	FON4mg/kg0.1mgIV/SC	0.52 (0.22, 1.12)	0.74 (0.20, 2.51)	0.83 (0.23, 2.87)	0.90 (0.25, 3.05)	0.59 (0.15, 2.15)	0.43 (0.13, 1.13)
FON4mg/kg1mgIV/SC	0.36 (0.06, 1.82)	0.73 (0.11, 3.40)	0.44 (0.07, 2.04)	0.51 (0.11, 2.41)	0.61 (0.13, 2.94)	0.32 (0.06, 1.65)	0.53 (0.13, 1.83)	1.53 (0.35, 5.97)	0.66 (0.13, 2.77)	1.07 (0.24, 4.31)	0.49 (0.10, 2.11)	1.64 (0.37, 6.57)	0.71 (0.14, 2.98)	0.31 (0.04, 1.86)	1.01 (0.41, 2.65)	1.58 (0.69, 3.98)	1.93 (0.89, 4.57)	FON4mg/kg1mgIV/SC	1.45 (0.34, 5.48)	1.66 (0.40, 6.23)	1.78 (0.43, 6.78)	1.14 (0.26, 4.54)	0.84 (0.22, 2.58)
GUS1200mgIV	0.25 (0.06, 1.09)	0.51 (0.11, 2.08)	0.30 (0.07, 1.26)	0.35 (0.09, 1.46)	0.43 (0.12, 1.74)	0.23 (0.05, 1.00)	**0.37 (0.13, 0.99)**	1.06 (0.33, 3.49)	0.46 (0.12, 1.63)	0.74 (0.23, 2.46)	0.34 (0.09, 1.24)	1.15 (0.36, 3.74)	0.49 (0.13, 1.68)	0.22 (0.04, 1.17)	0.72 (0.19, 2.84)	1.11 (0.31, 4.29)	1.36 (0.40, 5.01)	0.69 (0.18, 2.90)	GUS1200mgIV	1.14 (0.58, 2.19)	1.23 (0.64, 2.30)	0.80 (0.25, 2.70)	0.59 (0.23, 1.38)
GUS200mgIV	**0.22 (0.05, 0.97)**	0.45 (0.10, 1.83)	0.27 (0.06, 1.11)	0.31 (0.08, 1.25)	0.37 (0.11, 1.49)	**0.20 (0.05, 0.87)**	**0.32 (0.12, 0.87)**	0.94 (0.29, 3.07)	0.40 (0.11, 1.42)	0.65 (0.20, 2.19)	0.30 (0.08, 1.10)	1.01 (0.31, 3.17)	0.43 (0.12, 1.49)	**0.19 (0.03, 0.98)**	0.63 (0.17, 2.41)	0.97 (0.27, 3.79)	1.20 (0.35, 4.36)	0.60 (0.16, 2.50)	0.88 (0.46, 1.71)	GUS200mgIV	1.08 (0.58, 2.07)	0.70 (0.22, 2.36)	0.51 (0.20, 1.21)
GUS600mgIV	**0.20 (0.04, 0.87)**	0.41 (0.09, 1.72)	0.25 (0.06, 1.04)	0.29 (0.08, 1.15)	0.35 (0.10, 1.34)	**0.19 (0.04, 0.80)**	**0.30 (0.10, 0.79)**	0.86 (0.27, 2.81)	0.37 (0.10, 1.31)	0.61 (0.18, 1.97)	0.28 (0.07, 1.02)	0.93 (0.30, 2.97)	0.40 (0.11, 1.37)	**0.18 (0.03, 0.90)**	0.58 (0.16, 2.30)	0.90 (0.25, 3.46)	1.11 (0.33, 4.04)	0.56 (0.15, 2.30)	0.81 (0.44, 1.55)	0.93 (0.48, 1.74)	GUS600mgIV	0.65 (0.20, 2.19)	0.47 (0.19, 1.10)
MED700IV	0.31 (0.07, 1.43)	0.63 (0.13, 2.75)	0.38 (0.08, 1.63)	0.44 (0.11, 1.84)	0.53 (0.13, 2.32)	0.29 (0.06, 1.30)	0.46 (0.15, 1.36)	1.34 (0.37, 4.60)	0.57 (0.15, 2.13)	0.93 (0.24, 3.27)	0.43 (0.10, 1.65)	1.44 (0.41, 4.82)	0.61 (0.15, 2.23)	0.27 (0.04, 1.49)	0.90 (0.22, 3.63)	1.40 (0.38, 5.71)	1.69 (0.47, 6.53)	0.87 (0.22, 3.82)	1.26 (0.37, 4.02)	1.43 (0.42, 4.62)	1.55 (0.46, 5.04)	MED700IV	0.72 (0.26, 1.92)
NAT300mgIV	0.43 (0.11, 1.56)	0.89 (0.23, 3.02)	0.53 (0.14, 1.78)	0.60 (0.19, 1.99)	0.73 (0.25, 2.31)	0.39 (0.11, 1.41)	0.63 (0.30, 1.29)	1.85 (0.72, 4.95)	0.79 (0.27, 2.33)	1.29 (0.48, 3.44)	0.59 (0.19, 1.84)	1.98 (0.78, 5.28)	0.85 (0.28, 2.35)	0.38 (0.07, 1.67)	1.24 (0.40, 4.23)	1.92 (0.67, 6.88)	2.35 (0.88, 7.48)	1.20 (0.39, 4.49)	1.71 (0.72, 4.41)	1.96 (0.83, 4.90)	2.12 (0.91, 5.27)	1.39 (0.52, 3.81)	NAT300mgIV

(TABLE S12I. continued)

	ABA10mg/kgIV	ABA30mg/kgIV	ABA3mg/kgIV	AND150mgSC1/1	AND150mgSC2.2	AND300mgSC	CDP10mg/kgIV	CER100mgSC	CER10mgIV	CER200mgSC	CER20mgIV	CER400mgSC	CER5mgIV	ETA25mgSC	FON1mg/kg0.1mgIV/SC	FON1mg/kg1mgIV/SC	FON4mg/kg0.1mgIV/SC	FON4mg/kg1mgIV/SC	GUS1200mgIV	GUS200mgIV	GUS600mgIV	MED700IV	NAT300mgIV
NAT3mg/kgIV	0.33 (0.07, 1.41)	0.66 (0.14, 2.73)	0.40 (0.09, 1.58)	0.46 (0.12, 1.82)	0.56 (0.15, 2.24)	0.30 (0.07, 1.34)	0.48 (0.16, 1.31)	1.40 (0.41, 4.57)	0.60 (0.16, 2.14)	0.98 (0.28, 3.22)	0.44 (0.11, 1.66)	1.50 (0.45, 4.89)	0.64 (0.17, 2.19)	0.29 (0.04, 1.49)	0.93 (0.25, 3.58)	1.47 (0.40, 6.06)	1.79 (0.52, 6.53)	0.91 (0.23, 3.84)	1.31 (0.41, 4.19)	1.50 (0.45, 4.61)	1.62 (0.49, 4.92)	1.05 (0.30, 3.63)	0.76 (0.29, 1.86)
NAT3mg/kgIVx2	**0.23 (0.05, 0.95)**	0.45 (0.10, 1.79)	0.27 (0.06, 1.07)	0.32 (0.08, 1.22)	0.38 (0.10, 1.44)	**0.20 (0.05, 0.87)**	**0.33 (0.11, 0.87)**	0.95 (0.29, 3.00)	0.41 (0.11, 1.41)	0.66 (0.20, 2.18)	0.31 (0.08, 1.09)	1.02 (0.32, 3.20)	0.43 (0.12, 1.43)	0.19 (0.03, 1.01)	0.64 (0.17, 2.40)	0.99 (0.29, 3.87)	1.21 (0.36, 4.21)	0.62 (0.17, 2.60)	0.89 (0.29, 2.67)	1.00 (0.33, 3.08)	1.08 (0.35, 3.32)	0.71 (0.21, 2.40)	0.51 (0.20, 1.22)
NAT6mg/kgIVx2	**0.21 (0.05, 0.91)**	0.43 (0.09, 1.77)	0.26 (0.06, 1.02)	0.30 (0.08, 1.17)	0.37 (0.10, 1.41)	**0.20 (0.04, 0.84)**	**0.31 (0.11, 0.84)**	0.91 (0.28, 2.99)	0.39 (0.10, 1.38)	0.64 (0.18, 2.05)	0.29 (0.07, 1.06)	0.98 (0.30, 3.12)	0.41 (0.11, 1.44)	**0.19 (0.03, 0.98)**	0.61 (0.17, 2.31)	0.95 (0.27, 3.79)	1.16 (0.34, 4.10)	0.59 (0.16, 2.48)	0.86 (0.27, 2.67)	0.97 (0.31, 2.95)	1.04 (0.33, 3.18)	0.69 (0.20, 2.32)	0.49 (0.19, 1.18)
NNC2mg/kgSC	0.36 (0.06, 1.79)	0.72 (0.13, 3.28)	0.44 (0.08, 1.96)	0.50 (0.11, 2.22)	0.61 (0.14, 2.55)	0.33 (0.06, 1.56)	0.53 (0.14, 1.68)	1.53 (0.39, 5.66)	0.65 (0.15, 2.62)	1.07 (0.27, 4.09)	0.49 (0.11, 2.06)	1.62 (0.42, 6.04)	0.70 (0.15, 2.78)	0.30 (0.04, 1.86)	1.03 (0.22, 4.46)	1.60 (0.35, 7.16)	1.93 (0.45, 8.16)	1.01 (0.22, 4.94)	1.40 (0.36, 5.40)	1.62 (0.42, 6.14)	1.75 (0.45, 6.48)	1.15 (0.28, 4.57)	0.83 (0.26, 2.50)
PBO	0.58 (0.16, 1.97)	1.20 (0.32, 3.88)	0.71 (0.21, 2.23)	0.82 (0.29, 2.51)	0.98 (0.38, 3.00)	0.53 (0.16, 1.76)	0.85 (0.45, 1.60)	**2.48 (1.08, 6.10)**	1.07 (0.38, 2.84)	1.73 (0.73, 4.30)	0.80 (0.26, 2.30)	**2.68 (1.16, 6.48)**	1.14 (0.42, 3.00)	0.51 (0.10, 2.17)	1.66 (0.59, 5.34)	2.59 (0.98, 8.43)	**3.14 (1.30, 9.14)**	1.61 (0.56, 5.58)	**2.30 (1.04, 5.53)**	**2.63 (1.22, 6.07)**	**2.84 (1.37, 6.47)**	1.86 (0.77, 4.75)	1.35 (0.92, 2.05)
PF10mgSC	0.93 (0.17, 5.05)	1.86 (0.34, 9.31)	1.10 (0.23, 5.56)	1.29 (0.28, 6.35)	1.56 (0.34, 7.56)	0.84 (0.16, 4.24)	1.34 (0.38, 4.97)	3.90 (1.00, 17.33)	1.65 (0.38, 7.79)	2.70 (0.69, 11.66)	1.26 (0.26, 6.17)	**4.20 (1.09, 17.55)**	1.79 (0.40, 7.89)	0.79 (0.11, 4.95)	2.66 (0.58, 12.15)	4.10 (0.96, 19.79)	**5.00 (1.19, 23.29)**	2.56 (0.57, 12.01)	3.62 (0.94, 15.07)	**4.13 (1.08, 17.52)**	**4.45 (1.16, 18.45)**	2.86 (0.73, 13.14)	2.11 (0.67, 7.30)
PF200mgSC	1.68 (0.26, 20.56)	3.40 (0.49, 36.63)	2.02 (0.30, 22.07)	2.35 (0.41, 22.33)	2.84 (0.48, 27.60)	1.51 (0.24, 16.23)	2.38 (0.52, 22.19)	**6.95 (1.38, 69.34)**	3.05 (0.51, 27.66)	4.81 (0.92, 50.60)	2.32 (0.36, 22.34)	**7.48 (1.48, 74.69)**	3.34 (0.56, 29.78)	1.45 (0.17, 16.89)	4.79 (0.86, 43.86)	**7.63 (1.37, 71.64)**	**9.26 (1.76, 84.16)**	4.75 (0.81, 44.42)	**6.65 (1.24, 65.84)**	**7.56 (1.45, 74.22)**	**8.25 (1.55, 81.40)**	**5.23 (1.01, 52.93)**	3.73 (0.88, 32.18)
PF50mgSC	0.40 (0.08, 1.81)	0.80 (0.17, 3.42)	0.49 (0.11, 2.06)	0.56 (0.14, 2.35)	0.68 (0.18, 2.84)	0.36 (0.08, 1.62)	0.58 (0.19, 1.66)	1.70 (0.51, 6.00)	0.73 (0.20, 2.72)	1.17 (0.35, 4.10)	0.55 (0.14, 2.07)	1.82 (0.56, 6.23)	0.78 (0.20, 2.76)	0.35 (0.05, 1.88)	1.15 (0.29, 4.35)	1.77 (0.49, 6.99)	2.16 (0.60, 8.01)	1.12 (0.27, 4.57)	1.57 (0.47, 5.24)	1.81 (0.54, 6.00)	1.96 (0.59, 6.28)	1.28 (0.36, 4.40)	0.92 (0.34, 2.37)
RIS200mgIV	**0.15 (0.02, 0.94)**	0.31 (0.04, 1.77)	0.19 (0.03, 1.12)	0.22 (0.03, 1.21)	0.27 (0.04, 1.47)	**0.14 (0.02, 0.86)**	**0.23 (0.04, 0.95)**	0.67 (0.11, 3.03)	0.28 (0.05, 1.41)	0.47 (0.07, 2.19)	0.21 (0.03, 1.11)	0.72 (0.12, 3.28)	0.30 (0.05, 1.51)	**0.14 (0.01, 0.96)**	0.45 (0.07, 2.34)	0.70 (0.10, 3.79)	0.87 (0.13, 4.37)	0.44 (0.06, 2.53)	0.62 (0.11, 2.81)	0.70 (0.12, 3.28)	0.77 (0.14, 3.57)	0.51 (0.08, 2.47)	0.36 (0.07, 1.37)
RIS600mgIV	**0.12 (0.02, 0.58)**	0.24 (0.04, 1.15)	**0.14 (0.02, 0.69)**	**0.17 (0.03, 0.76)**	**0.20 (0.04, 0.91)**	**0.11 (0.02, 0.56)**	**0.17 (0.04, 0.57)**	0.51 (0.10, 1.94)	**0.21 (0.04, 0.95)**	0.35 (0.06, 1.33)	**0.16 (0.03, 0.73)**	0.54 (0.11, 2.04)	0.23 (0.05, 1.01)	**0.10 (0.01, 0.60)**	0.33 (0.06, 1.67)	0.52 (0.10, 2.66)	0.64 (0.12, 3.05)	0.33 (0.06, 1.77)	0.48 (0.10, 1.87)	0.54 (0.11, 2.23)	0.58 (0.13, 2.29)	0.38 (0.08, 1.52)	**0.28 (0.07, 0.83)**
TES400mg200mgSC	0.24 (0.05, 1.10)	0.49 (0.10, 2.17)	0.30 (0.06, 1.29)	0.34 (0.08, 1.44)	0.41 (0.11, 1.69)	0.22 (0.05, 1.01)	0.35 (0.11, 1.05)	1.03 (0.29, 3.75)	0.44 (0.11, 1.69)	0.72 (0.19, 2.57)	0.33 (0.08, 1.33)	1.10 (0.32, 3.93)	0.47 (0.12, 1.70)	0.21 (0.03, 1.14)	0.70 (0.17, 2.74)	1.08 (0.27, 4.45)	1.31 (0.36, 5.08)	0.67 (0.17, 2.97)	0.97 (0.28, 3.30)	1.10 (0.32, 3.63)	1.19 (0.35, 3.99)	0.77 (0.21, 2.78)	0.56 (0.20, 1.49)
UST130mgIV	0.35 (0.09, 1.25)	0.70 (0.18, 2.44)	0.42 (0.11, 1.41)	0.48 (0.16, 1.61)	0.58 (0.20, 1.90)	0.31 (0.09, 1.12)	0.50 (0.23, 1.04)	1.46 (0.57, 4.01)	0.63 (0.21, 1.82)	1.03 (0.38, 2.75)	0.47 (0.15, 1.44)	1.57 (0.60, 4.17)	0.67 (0.22, 1.91)	0.30 (0.05, 1.32)	0.99 (0.31, 3.21)	1.54 (0.52, 5.41)	1.88 (0.68, 5.89)	0.96 (0.31, 3.61)	1.35 (0.57, 3.49)	1.55 (0.64, 3.87)	1.68 (0.71, 4.18)	1.10 (0.41, 3.06)	0.80 (0.43, 1.39)
UST1mg/kgIV	0.31 (0.07, 1.24)	0.61 (0.15, 2.41)	0.38 (0.09, 1.42)	0.43 (0.12, 1.63)	0.53 (0.16, 1.94)	0.28 (0.07, 1.10)	0.45 (0.17, 1.10)	1.30 (0.44, 4.04)	0.56 (0.16, 1.87)	0.91 (0.30, 2.85)	0.42 (0.12, 1.45)	1.40 (0.47, 4.17)	0.59 (0.17, 1.97)	0.27 (0.04, 1.29)	0.89 (0.25, 3.26)	1.37 (0.41, 5.12)	1.67 (0.53, 5.73)	0.85 (0.24, 3.65)	1.20 (0.42, 3.69)	1.38 (0.49, 4.07)	1.50 (0.55, 4.46)	0.98 (0.32, 3.07)	0.71 (0.32, 1.57)
UST3mg/kgIV	0.30 (0.07, 1.18)	0.60 (0.14, 2.31)	0.36 (0.09, 1.35)	0.41 (0.12, 1.55)	0.50 (0.16, 1.83)	0.27 (0.07, 1.06)	0.43 (0.17, 1.09)	1.25 (0.43, 3.78)	0.53 (0.16, 1.83)	0.87 (0.29, 2.75)	0.40 (0.11, 1.40)	1.34 (0.46, 4.00)	0.58 (0.17, 1.84)	0.26 (0.04, 1.21)	0.85 (0.24, 3.06)	1.32 (0.40, 4.70)	1.61 (0.51, 5.40)	0.82 (0.23, 3.36)	1.16 (0.42, 3.42)	1.33 (0.48, 3.81)	1.44 (0.52, 4.15)	0.94 (0.31, 3.01)	0.68 (0.32, 1.50)
UST4.5mg/kgIV	**0.14 (0.02, 0.71)**	0.28 (0.04, 1.39)	**0.17 (0.03, 0.84)**	**0.20 (0.04, 0.91)**	0.25 (0.05, 1.13)	**0.13 (0.02, 0.69)**	**0.21 (0.05, 0.68)**	0.61 (0.13, 2.33)	0.26 (0.05, 1.13)	0.42 (0.08, 1.60)	**0.19 (0.04, 0.86)**	0.65 (0.14, 2.48)	0.28 (0.06, 1.15)	**0.12 (0.02, 0.71)**	0.40 (0.08, 1.95)	0.62 (0.12, 3.25)	0.77 (0.15, 3.71)	0.39 (0.07, 2.12)	0.57 (0.13, 2.13)	0.64 (0.14, 2.56)	0.70 (0.16, 2.58)	0.46 (0.10, 1.81)	**0.33 (0.08, 0.97)**
UST6mg/kg90mgIV/SC	**0.18 (0.04, 0.79)**	0.37 (0.08, 1.52)	**0.22 (0.05, 0.92)**	0.26 (0.07, 1.03)	0.31 (0.09, 1.25)	**0.17 (0.04, 0.72)**	**0.27 (0.10, 0.71)**	0.78 (0.24, 2.48)	0.34 (0.09, 1.15)	0.56 (0.17, 1.77)	**0.25 (0.07, 0.91)**	0.85 (0.27, 2.62)	0.36 (0.10, 1.22)	**0.16 (0.03, 0.80)**	0.54 (0.14, 2.02)	0.82 (0.23, 3.15)	1.00 (0.29, 3.62)	0.51 (0.13, 2.20)	0.74 (0.39, 1.39)	0.84 (0.45, 1.52)	0.91 (0.49, 1.65)	0.58 (0.18, 1.94)	0.43 (0.17, 1.00)
UST6mg/kgIV	**0.27 (0.07, 0.95)**	0.55 (0.14, 1.86)	0.33 (0.09, 1.08)	0.37 (0.12, 1.21)	0.45 (0.16, 1.48)	**0.24 (0.07, 0.85)**	**0.39 (0.19, 0.81)**	1.13 (0.45, 3.03)	0.49 (0.17, 1.42)	0.80 (0.30, 2.14)	0.36 (0.12, 1.12)	1.22 (0.48, 3.16)	0.52 (0.18, 1.48)	0.23 (0.04, 1.00)	0.76 (0.25, 2.48)	1.19 (0.40, 4.18)	1.45 (0.54, 4.46)	0.74 (0.24, 2.71)	1.05 (0.44, 2.73)	1.20 (0.50, 3.00)	1.31 (0.56, 3.19)	0.85 (0.32, 2.33)	0.62 (0.35, 1.06)
UST90mgSC	0.56 (0.10, 2.89)	1.13 (0.20, 5.74)	0.68 (0.13, 3.52)	0.79 (0.17, 3.90)	0.98 (0.20, 4.71)	0.52 (0.09, 2.67)	0.82 (0.22, 3.03)	2.37 (0.56, 10.30)	1.03 (0.21, 4.69)	1.66 (0.38, 7.24)	0.76 (0.15, 3.54)	2.57 (0.61, 10.65)	1.10 (0.23, 4.87)	0.48 (0.07, 3.05)	1.63 (0.34, 7.76)	2.51 (0.55, 11.99)	3.07 (0.71, 14.04)	1.58 (0.33, 7.66)	2.21 (0.56, 9.24)	2.56 (0.64, 10.81)	2.76 (0.69, 11.29)	1.77 (0.44, 7.94)	1.28 (0.38, 4.47)
VED0_5mg/kgIV	0.42 (0.09, 1.78)	0.85 (0.19, 3.42)	0.51 (0.12, 2.02)	0.59 (0.15, 2.35)	0.71 (0.20, 2.71)	0.38 (0.09, 1.60)	0.61 (0.22, 1.68)	1.79 (0.55, 5.83)	0.77 (0.20, 2.71)	1.23 (0.38, 4.12)	0.57 (0.15, 2.07)	1.93 (0.60, 6.06)	0.83 (0.21, 2.85)	0.36 (0.06, 1.94)	1.18 (0.32, 5.11)	1.86 (0.52, 8.25)	2.24 (0.66, 9.06)	1.15 (0.30, 5.42)	1.67 (0.54, 5.15)	1.90 (0.61, 5.87)	2.06 (0.66, 6.22)	1.33 (0.40, 4.56)	0.97 (0.39, 2.39)
VED2mg/kgIV	0.33 (0.07, 1.37)	0.66 (0.15, 2.67)	0.40 (0.09, 1.56)	0.45 (0.13, 1.78)	0.56 (0.16, 2.21)	0.30 (0.07, 1.22)	0.48 (0.17, 1.28)	1.39 (0.45, 4.40)	0.61 (0.17, 2.10)	0.97 (0.29, 3.09)	0.44 (0.12, 1.67)	1.51 (0.48, 4.60)	0.64 (0.18, 2.14)	0.29 (0.05, 1.49)	0.94 (0.26, 3.74)	1.45 (0.42, 6.09)	1.75 (0.55, 6.88)	0.90 (0.24, 4.01)	1.31 (0.44, 3.88)	1.49 (0.49, 4.51)	1.61 (0.54, 4.80)	1.05 (0.32, 3.44)	0.76 (0.31, 1.76)
VED300mgIV	0.27 (0.06, 1.16)	0.53 (0.12, 2.22)	0.32 (0.07, 1.37)	0.37 (0.09, 1.44)	0.46 (0.12, 1.78)	0.24 (0.05, 1.05)	0.39 (0.13, 1.07)	1.13 (0.34, 3.78)	0.49 (0.12, 1.79)	0.79 (0.22, 2.69)	0.36 (0.09, 1.38)	1.22 (0.36, 4.01)	0.52 (0.14, 1.83)	0.23 (0.04, 1.26)	0.76 (0.19, 2.97)	1.17 (0.33, 4.62)	1.44 (0.41, 5.35)	0.74 (0.19, 3.17)	1.06 (0.32, 3.41)	1.21 (0.36, 3.86)	1.31 (0.41, 4.14)	0.85 (0.24, 2.94)	0.62 (0.23, 1.54)
SEC10mg/kgIV	**0.07 (0.00, 0.38)**	**0.05 (0.00, 0.29)**	**0.06 (0.00, 0.34)**	**0.06 (0.00, 0.39)**	**0.07 (0.00, 0.51)**	**0.08 (0.00, 0.43)**	**0.04 (0.00, 0.28)**	**0.07 (0.00, 0.44)**	0.05 (0.00, 7.53)	0.05 (0.00, 4.90)	0.04 (0.00, 3.39)	**0.08 (0.00, 0.49)**	**0.08 (0.00, 0.46)**	**0.07 (0.00, 0.46)**	**0.07 (0.00, 0.45)**	**0.10 (0.01, 0.63)**	**0.08 (0.00, 0.47)**	0.19 (0.01, 2.07)	**0.05 (0.00, 0.33)**	**0.06 (0.00, 0.39)**	**0.14 (0.01, 0.82)**	**0.15 (0.01, 0.87)**	**0.08 (0.00, 0.41)**

	ABA10mg/kgIV	ABA30mg/kgIV	ABA3mg/kgIV	AND150mgSC1/1	AND150mgSC2.2	AND300mgSC	CDP10mg/kgIV	CER100mgSC	CER10mgIV	CER200mgSC	CER20mgIV	CER400mgSC	CER5mgIV	ETA25mgSC	FON1mg/kg0.1mgIV/SC	FON1mg/kg1mgIV/SC	FON4mg/kg0.1mgIV/SC	FON4mg/kg1mgIV/SC	GUS1200mgIV	GUS200mgIV	GUS600mgIV	MED700IV	NAT300mgIV
SEM60mgIV	0.71 (0.28, 1.79)	0.54 (0.19, 1.49)	0.65 (0.26, 1.68)	0.63 (0.19, 2.07)	0.84 (0.23, 2.71)	0.82 (0.35, 2.03)	0.48 (0.15, 1.44)	0.80 (0.31, 2.20)	0.56 (0.02, 59.92)	0.64 (0.02, 37.32)	0.50 (0.02, 26.89)	0.86 (0.32, 2.43)	0.85 (0.32, 2.23)	0.80 (0.27, 2.44)	0.79 (0.27, 2.43)	1.12 (0.40, 3.16)	0.91 (0.38, 2.26)	2.19 (0.49, 12.00)	0.57 (0.18, 1.66)	0.69 (0.24, 1.98)	1.51 (0.59, 4.17)	1.58 (0.62, 4.30)	0.80 (0.36, 1.95)
SEM60mgIVx3	1.21 (0.45, 3.44)	0.93 (0.31, 2.79)	1.12 (0.38, 3.14)	1.05 (0.30, 3.71)	1.41 (0.36, 5.16)	1.38 (0.54, 3.94)	0.81 (0.25, 2.77)	1.36 (0.49, 4.29)	0.97 (0.03, 110.83)	1.09 (0.03, 70.36)	0.87 (0.03, 47.23)	1.48 (0.49, 4.49)	1.43 (0.51, 4.20)	1.37 (0.42, 4.55)	1.35 (0.42, 4.61)	1.93 (0.61, 5.97)	1.53 (0.60, 4.30)	3.74 (0.78, 22.92)	0.97 (0.29, 3.24)	1.17 (0.38, 3.71)	2.61 (0.91, 7.79)	2.71 (0.90, 8.13)	1.37 (0.55, 3.70)
TES400mg200mgSC	**0.28 (0.11, 0.70)**	**0.22 (0.07, 0.56)**	**0.26 (0.10, 0.65)**	**0.25 (0.07, 0.82)**	0.32 (0.09, 1.14)	**0.33 (0.13, 0.78)**	**0.19 (0.06, 0.56)**	**0.32 (0.11, 0.86)**	0.22 (0.01, 23.83)	0.25 (0.01, 14.62)	0.20 (0.01, 10.30)	**0.34 (0.12, 0.92)**	**0.34 (0.12, 0.87)**	0.32 (0.10, 1.00)	0.32 (0.10, 1.02)	0.45 (0.14, 1.30)	**0.37 (0.14, 0.87)**	0.86 (0.18, 4.94)	**0.23 (0.07, 0.63)**	**0.27 (0.09, 0.78)**	0.61 (0.21, 1.65)	0.63 (0.22, 1.67)	**0.32 (0.13, 0.75)**
TOF15mgOR_BID	1.34 (0.42, 4.71)	0.98 (0.29, 3.88)	1.22 (0.37, 4.40)	1.16 (0.28, 5.32)	1.52 (0.34, 7.14)	1.54 (0.49, 5.45)	0.89 (0.23, 3.67)	1.49 (0.44, 5.79)	1.05 (0.03, 100.51)	1.15 (0.04, 78.91)	0.92 (0.03, 51.02)	1.59 (0.46, 6.15)	1.58 (0.47, 5.77)	1.56 (0.39, 6.01)	1.50 (0.39, 5.76)	2.13 (0.58, 8.03)	1.70 (0.54, 5.97)	4.11 (0.73, 28.93)	1.07 (0.29, 4.12)	1.28 (0.35, 5.14)	2.88 (0.84, 10.53)	2.96 (0.87, 11.07)	1.52 (0.50, 5.19)
TOF1mgOR_BID	1.38 (0.43, 5.22)	1.04 (0.30, 4.00)	1.26 (0.39, 4.79)	1.24 (0.30, 5.50)	1.60 (0.35, 7.71)	1.61 (0.51, 5.68)	0.93 (0.25, 3.83)	1.58 (0.45, 5.90)	1.13 (0.03, 130.26)	1.21 (0.04, 82.30)	0.96 (0.03, 51.99)	1.67 (0.46, 6.87)	1.64 (0.48, 6.49)	1.62 (0.41, 6.68)	1.57 (0.40, 6.34)	2.19 (0.59, 8.96)	1.78 (0.54, 6.39)	4.34 (0.76, 27.94)	1.12 (0.29, 4.69)	1.34 (0.37, 5.48)	3.04 (0.85, 11.81)	3.13 (0.88, 11.91)	1.59 (0.52, 5.52)
TOF5mgOR_BID	0.79 (0.28, 2.31)	0.59 (0.19, 1.86)	0.72 (0.26, 2.10)	0.69 (0.18, 2.73)	0.91 (0.23, 3.74)	0.90 (0.34, 2.62)	0.52 (0.16, 1.92)	0.90 (0.29, 2.82)	0.63 (0.02, 63.76)	0.68 (0.02, 48.29)	0.54 (0.02, 29.78)	0.96 (0.31, 3.10)	0.94 (0.31, 2.88)	0.91 (0.27, 3.13)	0.89 (0.25, 2.99)	1.26 (0.39, 4.06)	1.01 (0.36, 2.98)	2.48 (0.49, 14.59)	0.63 (0.19, 2.11)	0.78 (0.23, 2.50)	1.70 (0.55, 5.26)	1.77 (0.58, 5.45)	0.89 (0.34, 2.47)
UPA12mgOR_BID	0.57 (0.23, 1.31)	0.43 (0.16, 1.08)	0.52 (0.21, 1.20)	0.50 (0.15, 1.52)	0.64 (0.19, 2.19)	0.65 (0.28, 1.48)	0.38 (0.12, 1.09)	0.65 (0.24, 1.61)	0.45 (0.01, 48.31)	0.50 (0.01, 29.31)	0.39 (0.01, 21.04)	0.68 (0.26, 1.81)	0.67 (0.26, 1.65)	0.64 (0.21, 1.88)	0.64 (0.21, 1.79)	0.89 (0.33, 2.37)	0.73 (0.30, 1.67)	1.71 (0.40, 9.59)	0.45 (0.16, 1.27)	0.56 (0.19, 1.51)	1.22 (0.47, 3.07)	1.27 (0.49, 3.18)	0.65 (0.28, 1.41)
UPA24mgOR	0.74 (0.29, 1.84)	0.56 (0.19, 1.49)	0.67 (0.26, 1.69)	0.65 (0.18, 2.02)	0.85 (0.25, 2.87)	0.85 (0.36, 2.01)	0.50 (0.16, 1.48)	0.83 (0.30, 2.20)	0.59 (0.02, 58.69)	0.64 (0.02, 36.25)	0.51 (0.02, 26.98)	0.89 (0.33, 2.44)	0.87 (0.34, 2.25)	0.84 (0.27, 2.61)	0.83 (0.27, 2.41)	1.15 (0.41, 3.24)	0.94 (0.37, 2.32)	2.24 (0.52, 12.66)	0.59 (0.19, 1.72)	0.71 (0.24, 2.09)	1.59 (0.61, 4.24)	1.64 (0.60, 4.43)	0.84 (0.36, 1.97)
UPA24mgOR_BID	0.45 (0.19, 1.02)	**0.34 (0.13, 0.83)**	**0.41 (0.17, 0.95)**	0.40 (0.12, 1.21)	0.51 (0.16, 1.70)	0.52 (0.23, 1.13)	**0.31 (0.10, 0.83)**	0.51 (0.20, 1.24)	0.36 (0.01, 38.90)	0.40 (0.01, 23.67)	0.31 (0.01, 16.56)	0.54 (0.21, 1.39)	0.53 (0.22, 1.30)	0.51 (0.17, 1.46)	0.50 (0.18, 1.37)	0.70 (0.27, 1.84)	0.58 (0.25, 1.26)	1.35 (0.33, 7.74)	**0.36 (0.13, 0.96)**	0.44 (0.16, 1.16)	0.96 (0.38, 2.41)	1.00 (0.40, 2.50)	0.51 (0.23, 1.10)
UPA3mgOR_BID	0.62 (0.24, 1.47)	0.46 (0.16, 1.18)	0.57 (0.22, 1.32)	0.54 (0.16, 1.68)	0.71 (0.21, 2.35)	0.70 (0.31, 1.61)	0.41 (0.13, 1.16)	0.69 (0.26, 1.76)	0.49 (0.02, 52.80)	0.55 (0.02, 30.41)	0.42 (0.01, 22.84)	0.73 (0.28, 1.90)	0.72 (0.29, 1.77)	0.69 (0.23, 2.09)	0.69 (0.23, 1.93)	0.96 (0.35, 2.59)	0.78 (0.32, 1.87)	1.85 (0.45, 10.43)	0.49 (0.17, 1.36)	0.59 (0.20, 1.71)	1.33 (0.50, 3.31)	1.36 (0.52, 3.48)	0.70 (0.30, 1.53)
UPA45mgOR	0.58 (0.18, 1.78)	0.44 (0.13, 1.46)	0.53 (0.16, 1.63)	0.51 (0.12, 1.90)	0.67 (0.16, 2.68)	0.67 (0.21, 1.95)	0.39 (0.10, 1.39)	0.66 (0.19, 2.13)	0.48 (0.01, 42.49)	0.52 (0.02, 27.10)	0.42 (0.01, 19.57)	0.71 (0.20, 2.37)	0.69 (0.20, 2.18)	0.66 (0.18, 2.29)	0.65 (0.17, 2.25)	0.92 (0.25, 3.09)	0.75 (0.23, 2.18)	1.79 (0.33, 11.55)	0.46 (0.12, 1.66)	0.57 (0.15, 1.93)	1.26 (0.37, 4.08)	1.29 (0.38, 4.13)	0.66 (0.21, 1.88)
UPA6mgOR_BID	0.49 (0.21, 1.10)	**0.37 (0.14, 0.92)**	0.45 (0.18, 1.02)	0.44 (0.13, 1.30)	0.57 (0.17, 1.82)	0.57 (0.25, 1.23)	**0.33 (0.11, 0.93)**	0.55 (0.22, 1.36)	0.39 (0.01, 40.91)	0.44 (0.01, 25.60)	0.34 (0.01, 17.57)	0.59 (0.23, 1.53)	0.58 (0.23, 1.41)	0.56 (0.19, 1.60)	0.55 (0.19, 1.48)	0.77 (0.29, 2.06)	0.63 (0.27, 1.40)	1.49 (0.36, 8.04)	0.39 (0.14, 1.09)	0.48 (0.17, 1.31)	1.06 (0.43, 2.62)	1.10 (0.43, 2.68)	0.56 (0.25, 1.16)
UST130mgIV	0.97 (0.52, 1.82)	0.73 (0.34, 1.50)	0.89 (0.48, 1.69)	0.85 (0.31, 2.26)	1.12 (0.37, 3.20)	1.10 (0.67, 1.99)	0.65 (0.26, 1.54)	1.09 (0.55, 2.29)	0.76 (0.03, 79.96)	0.85 (0.03, 45.70)	0.67 (0.02, 33.77)	1.16 (0.56, 2.52)	1.14 (0.60, 2.32)	1.11 (0.45, 2.67)	1.08 (0.46, 2.57)	1.53 (0.71, 3.40)	1.23 (0.71, 2.21)	2.93 (0.78, 15.10)	0.78 (0.33, 1.75)	0.95 (0.41, 2.11)	**2.08 (1.06, 4.37)**	**2.15 (1.10, 4.52)**	1.09 (0.68, 1.91)
UST1mg/kgIV	0.72 (0.36, 1.46)	0.55 (0.24, 1.20)	0.66 (0.33, 1.36)	0.64 (0.22, 1.82)	0.83 (0.27, 2.44)	0.83 (0.44, 1.62)	0.49 (0.18, 1.22)	0.81 (0.38, 1.83)	0.56 (0.02, 60.91)	0.63 (0.02, 36.13)	0.50 (0.02, 26.11)	0.87 (0.39, 2.00)	0.86 (0.41, 1.84)	0.82 (0.32, 2.15)	0.81 (0.32, 2.02)	1.15 (0.49, 2.68)	0.92 (0.48, 1.84)	2.19 (0.55, 11.51)	0.58 (0.23, 1.45)	0.71 (0.29, 1.71)	1.55 (0.72, 3.56)	1.61 (0.75, 3.68)	0.82 (0.45, 1.56)
UST3mg/kgIV	0.82 (0.40, 1.70)	0.62 (0.27, 1.42)	0.75 (0.36, 1.60)	0.72 (0.24, 2.11)	0.95 (0.31, 2.90)	0.93 (0.50, 1.88)	0.55 (0.20, 1.42)	0.92 (0.42, 2.13)	0.64 (0.02, 72.02)	0.71 (0.02, 41.62)	0.57 (0.02, 29.55)	0.99 (0.43, 2.32)	0.97 (0.46, 2.12)	0.94 (0.36, 2.47)	0.91 (0.36, 2.34)	1.29 (0.55, 3.16)	1.04 (0.53, 2.12)	2.48 (0.62, 13.31)	0.66 (0.26, 1.66)	0.80 (0.32, 2.00)	1.75 (0.81, 4.04)	1.81 (0.84, 4.26)	0.92 (0.51, 1.84)
UST4.5mg/kgIV	1.41 (0.40, 6.00)	1.06 (0.27, 4.74)	1.29 (0.34, 5.38)	1.26 (0.27, 5.87)	1.61 (0.34, 9.17)	1.60 (0.47, 6.77)	0.95 (0.23, 4.73)	1.61 (0.41, 6.84)	1.17 (0.03, 122.92)	1.29 (0.04, 86.16)	1.00 (0.03, 53.05)	1.70 (0.44, 7.43)	1.66 (0.45, 7.24)	1.63 (0.38, 7.23)	1.58 (0.36, 7.04)	2.24 (0.58, 9.98)	1.82 (0.50, 7.87)	4.29 (0.75, 34.44)	1.13 (0.27, 5.30)	1.36 (0.33, 6.16)	3.09 (0.81, 12.95)	3.17 (0.83, 13.70)	1.61 (0.47, 6.25)
UST6mg/kg90mgIV/SC	1.35 (0.53, 3.77)	1.03 (0.36, 2.96)	1.24 (0.47, 3.40)	1.20 (0.78, 1.82)	1.56 (0.43, 5.96)	1.57 (0.64, 4.21)	0.92 (0.28, 2.98)	1.53 (0.56, 4.77)	1.08 (0.03, 115.59)	1.22 (0.03, 67.25)	0.94 (0.03, 49.46)	1.64 (0.57, 4.87)	1.61 (0.60, 4.64)	1.56 (0.47, 5.23)	1.52 (0.48, 5.05)	2.11 (0.73, 6.65)	1.75 (0.69, 4.72)	4.14 (0.90, 23.63)	1.10 (0.35, 3.35)	1.32 (0.43, 4.00)	**2.97 (1.04, 8.62)**	**3.09 (1.07, 8.80)**	1.55 (0.63, 4.08)
UST6mg/kgIV	0.79 (0.43, 1.45)	0.60 (0.28, 1.19)	0.72 (0.40, 1.32)	0.69 (0.26, 1.82)	0.91 (0.33, 2.57)	0.90 (0.56, 1.57)	0.54 (0.22, 1.24)	0.90 (0.46, 1.81)	0.61 (0.02, 67.32)	0.69 (0.02, 39.24)	0.55 (0.02, 28.15)	0.95 (0.46, 2.01)	0.93 (0.50, 1.85)	0.91 (0.38, 2.18)	0.89 (0.38, 2.07)	1.24 (0.59, 2.70)	1.01 (0.58, 1.78)	2.38 (0.64, 12.18)	0.64 (0.27, 1.42)	0.78 (0.34, 1.66)	1.70 (0.88, 3.47)	1.76 (0.93, 3.56)	0.89 (0.57, 1.48)
UST90mgSC	0.93 (0.24, 3.97)	0.71 (0.17, 3.17)	0.87 (0.21, 3.78)	0.83 (0.16, 4.17)	1.07 (0.20, 5.78)	1.09 (0.29, 4.48)	0.63 (0.14, 3.02)	1.07 (0.26, 4.91)	0.77 (0.02, 84.27)	0.81 (0.02, 61.81)	0.65 (0.02, 43.89)	1.17 (0.27, 4.99)	1.15 (0.27, 4.86)	1.05 (0.24, 5.84)	1.05 (0.23, 5.30)	1.48 (0.34, 7.13)	1.20 (0.31, 5.01)	2.95 (0.42, 23.74)	0.75 (0.17, 3.58)	0.93 (0.20, 4.19)	2.05 (0.47, 9.01)	2.13 (0.50, 9.23)	1.07 (0.28, 4.41)
VED0_5mg/kgIV	0.63 (0.02, 19.64)	0.48 (0.02, 14.82)	0.59 (0.02, 17.39)	0.56 (0.02, 19.54)	0.71 (0.02, 25.34)	0.74 (0.02, 22.98)	0.43 (0.02, 14.72)	0.73 (0.02, 21.68)	0.55 (0.00, 92.46)	0.56 (0.00, 73.69)	0.46 (0.00, 59.01)	0.79 (0.02, 25.57)	0.75 (0.02, 24.28)	0.73 (0.02, 23.19)	0.69 (0.02, 23.12)	1.00 (0.03, 30.81)	0.82 (0.03, 25.24)	2.06 (0.05, 75.67)	0.51 (0.02, 16.22)	0.63 (0.02, 20.80)	1.39 (0.04, 41.75)	1.43 (0.05, 44.01)	0.72 (0.02, 21.98)
VED2mg/kgIV	0.86 (0.03, 21.92)	0.65 (0.02, 18.06)	0.77 (0.03, 21.32)	0.75 (0.02, 22.73)	0.99 (0.03, 30.14)	0.96 (0.03, 27.90)	0.55 (0.02, 17.44)	0.98 (0.03, 26.62)	0.70 (0.01, 195.50)	0.71 (0.01, 160.30)	0.58 (0.01, 135.31)	1.03 (0.03, 28.46)	1.02 (0.03, 27.76)	0.97 (0.03, 27.85)	0.94 (0.03, 27.56)	1.36 (0.04, 37.89)	1.09 (0.04, 30.99)	2.52 (0.07, 112.67)	0.69 (0.02, 21.06)	0.85 (0.03, 23.96)	1.83 (0.06, 52.76)	1.89 (0.06, 57.18)	0.96 (0.04, 27.28)
VED300mgIV	0.85 (0.39, 1.75)	0.65 (0.26, 1.47)	0.78 (0.36, 1.64)	0.75 (0.24, 2.19)	0.98 (0.30, 2.98)	0.97 (0.48, 1.99)	0.57 (0.20, 1.49)	0.95 (0.42, 2.22)	0.68 (0.02, 69.39)	0.73 (0.02, 44.69)	0.59 (0.02, 31.94)	1.03 (0.43, 2.54)	1.01 (0.44, 2.29)	0.96 (0.36, 2.63)	0.94 (0.35, 2.49)	1.33 (0.53, 3.37)	1.09 (0.52, 2.21)	2.61 (0.60, 13.72)	0.67 (0.26, 1.73)	0.83 (0.31, 2.07)	1.83 (0.79, 4.23)	1.88 (0.82, 4.40)	0.96 (0.50, 1.87)

	NAT3mg/kgIV	NAT3mg/kgIVx2	NAT6mg/kgIVx2	NNC2mg/kgSC	PBO	PF10mgSC	PF200mgSC	PF50mgSC	RIS200mgIV	RIS600mgIV	TES400mg200mgSC	UST130mgIV	UST1mg/kgIV	UST3mg/kgIV	UST4.5mg/kgIV	UST6mg/kg90mgIV/SC	UST6mg/kgIV	UST90mgSC	VED0_5mg/kgIV	VED2mg/kgIV	VED300mgIV	SEC10mg/kgIV
ABA10mg/kgIV	3.03 (0.71, 14.49)	**4.44 (1.05, 20.68)**	**4.66 (1.10, 21.65)**	2.81 (0.56, 15.62)	1.72 (0.51, 6.17)	1.08 (0.20, 5.82)	0.60 (0.05, 3.91)	2.49 (0.55, 11.90)	**6.46 (1.06, 47.81)**	**8.48 (1.73, 56.33)**	4.19 (0.91, 20.29)	2.88 (0.80, 11.19)	3.23 (0.81, 13.93)	3.37 (0.84, 14.77)	**6.96 (1.41, 45.71)**	**5.42 (1.26, 24.92)**	**3.73 (1.05, 14.26)**	1.78 (0.35, 10.50)	2.40 (0.56, 11.09)	3.06 (0.73, 14.00)	3.73 (0.86, 17.82)	**15.20 (2.61, 281.66)**
ABA30mg/kgIV	1.50 (0.37, 6.97)	2.21 (0.56, 10.23)	2.33 (0.57, 10.58)	1.38 (0.30, 7.65)	0.84 (0.26, 3.09)	0.54 (0.11, 2.95)	0.29 (0.03, 2.05)	1.25 (0.29, 5.90)	3.25 (0.57, 25.05)	4.22 (0.87, 27.25)	2.03 (0.46, 10.37)	1.43 (0.41, 5.64)	1.63 (0.41, 6.89)	1.66 (0.43, 7.19)	3.52 (0.72, 22.57)	2.70 (0.66, 12.15)	1.83 (0.54, 7.18)	0.88 (0.17, 4.89)	1.18 (0.29, 5.29)	1.52 (0.37, 6.57)	1.87 (0.45, 8.67)	**20.65 (3.40, 364.41)**
ABA3mg/kgIV	2.48 (0.63, 11.21)	3.67 (0.93, 16.00)	3.80 (0.98, 17.27)	2.30 (0.51, 11.96)	1.40 (0.45, 4.69)	0.91 (0.18, 4.44)	0.49 (0.05, 3.29)	2.05 (0.49, 9.02)	5.32 (0.89, 36.44)	**6.98 (1.45, 41.39)**	3.36 (0.77, 16.18)	2.37 (0.71, 8.79)	2.66 (0.70, 10.96)	2.77 (0.74, 10.97)	**5.81 (1.19, 33.22)**	**4.53 (1.09, 18.75)**	3.05 (0.92, 10.98)	1.46 (0.28, 7.98)	1.94 (0.50, 8.68)	2.51 (0.64, 10.66)	3.08 (0.73, 13.76)	**16.61 (2.92, 279.31)**
AND150mgSC1/1	2.16 (0.55, 8.36)	3.17 (0.82, 12.40)	3.32 (0.86, 13.03)	1.99 (0.45, 9.29)	1.22 (0.40, 3.46)	0.78 (0.16, 3.51)	0.42 (0.04, 2.46)	1.80 (0.42, 6.95)	4.55 (0.83, 29.59)	**5.94 (1.31, 34.79)**	2.95 (0.70, 12.38)	2.07 (0.62, 6.43)	2.34 (0.61, 8.14)	2.43 (0.65, 8.28)	**4.89 (1.09, 26.73)**	3.87 (0.97, 14.59)	2.69 (0.82, 8.12)	1.26 (0.26, 5.89)	1.70 (0.42, 6.46)	2.20 (0.56, 7.94)	2.70 (0.69, 10.54)	**17.57 (2.53, 342.98)**
AND150mgSC2/2	1.77 (0.45, 6.74)	2.61 (0.69, 9.84)	2.72 (0.71, 10.16)	1.65 (0.39, 7.17)	1.02 (0.33, 2.66)	0.64 (0.13, 2.91)	0.35 (0.04, 2.09)	1.46 (0.35, 5.71)	3.72 (0.68, 23.53)	**4.90 (1.10, 25.98)**	2.41 (0.59, 9.50)	1.72 (0.53, 5.00)	1.90 (0.52, 6.16)	1.99 (0.55, 6.38)	4.06 (0.88, 20.67)	3.19 (0.80, 11.27)	2.22 (0.67, 6.26)	1.03 (0.21, 4.94)	1.40 (0.37, 4.90)	1.80 (0.45, 6.38)	2.19 (0.56, 8.20)	**13.79 (1.94, 262.70)**
AND300mgSC	3.34 (0.74, 14.76)	**4.91 (1.15, 20.86)**	**5.13 (1.19, 22.57)**	3.07 (0.64, 15.42)	1.88 (0.57, 6.34)	1.19 (0.24, 6.14)	0.66 (0.06, 4.13)	2.74 (0.62, 11.90)	**7.04 (1.17, 50.47)**	**9.21 (1.79, 58.35)**	4.55 (0.99, 20.55)	3.19 (0.89, 11.71)	3.58 (0.91, 14.31)	3.71 (0.94, 15.15)	**7.72 (1.46, 45.80)**	**5.94 (1.40, 24.42)**	**4.14 (1.18, 14.72)**	1.93 (0.37, 10.70)	2.61 (0.62, 11.52)	3.29 (0.82, 14.69)	4.09 (0.95, 18.66)	**13.11 (2.32, 249.59)**
CDP10mg/kgIV	2.09 (0.76, 6.28)	**3.08 (1.15, 8.94)**	**3.20 (1.18, 9.32)**	1.90 (0.59, 7.17)	1.18 (0.62, 2.21)	0.75 (0.20, 2.66)	0.42 (0.05, 1.92)	1.72 (0.60, 5.20)	**4.34 (1.05, 23.46)**	**5.72 (1.75, 25.65)**	2.83 (0.95, 8.71)	1.98 (0.96, 4.36)	2.24 (0.91, 5.72)	2.34 (0.92, 5.96)	**4.80 (1.47, 20.58)**	**3.70 (1.41, 10.47)**	**2.57 (1.24, 5.39)**	1.22 (0.33, 4.63)	1.64 (0.60, 4.65)	2.08 (0.78, 5.82)	2.55 (0.93, 7.68)	**23.47 (3.55, 512.98)**
CER100mgSC	0.71 (0.22, 2.41)	1.05 (0.33, 3.49)	1.10 (0.33, 3.60)	0.65 (0.18, 2.58)	**0.40 (0.16, 0.93)**	0.26 (0.06, 1.00)	**0.14 (0.01, 0.72)**	0.59 (0.17, 1.97)	1.49 (0.33, 8.92)	1.97 (0.52, 9.82)	0.98 (0.27, 3.45)	0.69 (0.25, 1.75)	0.77 (0.25, 2.28)	0.80 (0.26, 2.34)	1.63 (0.43, 7.43)	1.28 (0.40, 4.12)	0.89 (0.33, 2.23)	0.42 (0.10, 1.78)	0.56 (0.17, 1.80)	0.72 (0.23, 2.22)	0.89 (0.26, 2.97)	**13.54 (2.25, 254.21)**
CER10mgIV	1.67 (0.47, 6.42)	2.44 (0.71, 8.98)	2.56 (0.72, 9.72)	1.53 (0.38, 6.69)	0.93 (0.35, 2.62)	0.61 (0.13, 2.65)	0.33 (0.04, 1.95)	1.37 (0.37, 5.07)	3.56 (0.71, 21.76)	**4.72 (1.05, 25.78)**	2.27 (0.59, 9.23)	1.58 (0.55, 4.74)	1.79 (0.53, 6.15)	1.87 (0.55, 6.17)	3.90 (0.88, 20.27)	2.98 (0.87, 11.03)	2.04 (0.70, 5.99)	0.97 (0.21, 4.76)	1.29 (0.37, 4.88)	1.65 (0.48, 6.01)	2.05 (0.56, 8.09)	21.56 (0.13, 1120.81)
CER200mgSC	1.03 (0.31, 3.63)	1.52 (0.46, 5.06)	1.57 (0.49, 5.52)	0.94 (0.24, 3.70)	0.58 (0.23, 1.37)	0.37 (0.09, 1.45)	0.21 (0.02, 1.09)	0.86 (0.24, 2.88)	2.13 (0.46, 13.83)	2.87 (0.75, 15.49)	1.39 (0.39, 5.15)	0.97 (0.36, 2.62)	1.10 (0.35, 3.37)	1.14 (0.36, 3.45)	2.39 (0.62, 12.28)	1.80 (0.57, 6.06)	1.26 (0.47, 3.33)	0.60 (0.14, 2.65)	0.81 (0.24, 2.64)	1.03 (0.32, 3.40)	1.26 (0.37, 4.50)	18.70 (0.20, 1477.20)
CER20mgIV	2.25 (0.60, 9.32)	3.24 (0.92, 13.04)	3.42 (0.94, 13.56)	2.05 (0.48, 9.51)	1.25 (0.43, 3.79)	0.80 (0.16, 3.90)	0.43 (0.04, 2.76)	1.82 (0.48, 7.25)	4.68 (0.90, 30.73)	**6.17 (1.37, 35.01)**	3.04 (0.75, 13.14)	2.14 (0.69, 6.86)	2.40 (0.69, 8.60)	2.49 (0.71, 8.79)	**5.15 (1.17, 28.41)**	**3.98 (1.10, 15.24)**	2.76 (0.89, 8.68)	1.31 (0.28, 6.58)	1.75 (0.48, 6.78)	2.27 (0.60, 8.39)	2.79 (0.72, 11.31)	25.18 (0.30, 1402.27)
CER400mgSC	0.67 (0.20, 2.21)	0.98 (0.31, 3.14)	1.02 (0.32, 3.33)	0.62 (0.17, 2.38)	**0.37 (0.15, 0.86)**	**0.24 (0.06, 0.92)**	**0.13 (0.01, 0.68)**	0.55 (0.16, 1.77)	1.39 (0.30, 8.10)	1.84 (0.49, 8.82)	0.91 (0.25, 3.17)	0.64 (0.24, 1.66)	0.71 (0.24, 2.12)	0.75 (0.25, 2.20)	1.53 (0.40, 6.99)	1.18 (0.38, 3.74)	0.82 (0.32, 2.07)	0.39 (0.09, 1.63)	0.52 (0.17, 1.67)	0.66 (0.22, 2.06)	0.82 (0.25, 2.80)	**13.03 (2.04, 231.76)**
CER5mgIV	1.56 (0.46, 5.90)	2.32 (0.70, 8.60)	2.43 (0.70, 8.81)	1.43 (0.36, 6.59)	0.87 (0.33, 2.38)	0.56 (0.13, 2.50)	0.30 (0.03, 1.80)	1.28 (0.36, 4.97)	3.30 (0.66, 19.80)	4.32 (0.99, 21.69)	2.14 (0.59, 8.58)	1.50 (0.52, 4.47)	1.68 (0.51, 5.81)	1.74 (0.54, 5.92)	3.64 (0.87, 17.40)	2.79 (0.82, 9.60)	1.93 (0.67, 5.71)	0.91 (0.21, 4.43)	1.21 (0.35, 4.68)	1.56 (0.47, 5.59)	1.93 (0.55, 7.06)	**13.01 (2.17, 224.21)**
ETA25mgSC	3.50 (0.67, 22.39)	5.17 (0.99, 31.95)	**5.36 (1.02, 32.90)**	3.28 (0.54, 23.29)	1.96 (0.46, 10.13)	1.27 (0.20, 8.73)	0.69 (0.06, 5.87)	2.89 (0.53, 18.82)	**7.38 (1.04, 68.63)**	**9.81 (1.66, 81.83)**	4.72 (0.88, 32.79)	3.33 (0.76, 18.20)	3.76 (0.78, 22.41)	3.89 (0.83, 22.87)	**8.18 (1.40, 65.42)**	**6.31 (1.25, 37.69)**	4.32 (1.00, 23.41)	2.07 (0.33, 14.75)	2.74 (0.52, 17.61)	3.49 (0.67, 21.39)	4.37 (0.79, 28.57)	**13.50 (2.16, 230.39)**
FON1mg/kg0.1mgIV/SC	1.07 (0.28, 3.97)	1.56 (0.42, 5.75)	1.64 (0.43, 6.02)	0.97 (0.22, 4.62)	0.60 (0.19, 1.69)	0.38 (0.08, 1.72)	0.21 (0.02, 1.16)	0.87 (0.23, 3.41)	2.21 (0.43, 14.56)	2.99 (0.60, 16.87)	1.44 (0.36, 5.84)	1.01 (0.31, 3.20)	1.13 (0.31, 3.97)	1.17 (0.33, 4.13)	2.50 (0.51, 13.08)	1.87 (0.50, 7.27)	1.32 (0.40, 4.04)	0.61 (0.13, 2.94)	0.85 (0.20, 3.16)	1.07 (0.27, 3.84)	1.32 (0.34, 5.23)	**13.60 (2.24, 242.03)**
FON1mg/kg1mgIV/SC	0.68 (0.16, 2.52)	1.01 (0.26, 3.45)	1.05 (0.26, 3.76)	0.63 (0.14, 2.89)	0.39 (0.12, 1.02)	0.24 (0.05, 1.04)	**0.13 (0.01, 0.73)**	0.57 (0.14, 2.06)	1.44 (0.26, 9.56)	1.94 (0.38, 10.50)	0.93 (0.22, 3.72)	0.65 (0.18, 1.93)	0.73 (0.20, 2.43)	0.76 (0.21, 2.48)	1.62 (0.31, 8.51)	1.22 (0.32, 4.39)	0.84 (0.24, 2.48)	0.40 (0.08, 1.80)	0.54 (0.12, 1.91)	0.69 (0.16, 2.38)	0.85 (0.22, 3.07)	**9.65 (1.59, 169.23)**
FON4mg/kg0.1mgIV/SC	0.56 (0.15, 1.93)	0.82 (0.24, 2.79)	0.86 (0.24, 2.94)	0.52 (0.12, 2.22)	**0.32 (0.11, 0.77)**	**0.20 (0.04, 0.84)**	**0.11 (0.01, 0.57)**	0.46 (0.12, 1.66)	1.15 (0.23, 7.57)	1.57 (0.33, 8.32)	0.77 (0.20, 2.81)	0.53 (0.17, 1.47)	0.60 (0.17, 1.89)	0.62 (0.19, 1.96)	1.30 (0.27, 6.73)	1.00 (0.28, 3.45)	0.69 (0.22, 1.85)	0.33 (0.07, 1.40)	0.45 (0.11, 1.51)	0.57 (0.15, 1.83)	0.69 (0.19, 2.43)	**12.09 (2.15, 202.36)**
FON4mg/kg1mgIV/SC	1.09 (0.26, 4.30)	1.61 (0.38, 6.05)	1.68 (0.40, 6.41)	0.99 (0.20, 4.61)	0.62 (0.18, 1.77)	0.39 (0.08, 1.75)	0.21 (0.02, 1.23)	0.90 (0.22, 3.65)	2.28 (0.39, 15.93)	3.05 (0.56, 17.35)	1.48 (0.34, 6.02)	1.04 (0.28, 3.28)	1.18 (0.27, 4.24)	1.22 (0.30, 4.30)	2.55 (0.47, 14.05)	1.96 (0.45, 7.42)	1.35 (0.37, 4.14)	0.63 (0.13, 3.00)	0.87 (0.18, 3.32)	1.11 (0.25, 4.09)	1.35 (0.32, 5.38)	5.14 (0.48, 109.50)
GUS1200mgIV	0.76 (0.24, 2.45)	1.12 (0.37, 3.50)	1.17 (0.37, 3.68)	0.71 (0.19, 2.75)	**0.43 (0.18, 0.96)**	0.28 (0.07, 1.06)	**0.15 (0.02, 0.81)**	0.64 (0.19, 2.12)	1.62 (0.36, 9.03)	2.09 (0.53, 9.82)	1.04 (0.30, 3.61)	0.74 (0.29, 1.76)	0.83 (0.27, 2.35)	0.86 (0.29, 2.39)	1.76 (0.47, 7.97)	1.35 (0.72, 2.59)	0.95 (0.37, 2.25)	0.45 (0.11, 1.77)	0.60 (0.19, 1.84)	0.76 (0.26, 2.30)	0.94 (0.29, 3.13)	**19.34 (3.02, 346.79)**
GUS200mgIV	0.67 (0.22, 2.21)	1.00 (0.32, 3.05)	1.03 (0.34, 3.27)	0.62 (0.16, 2.39)	**0.38 (0.16, 0.82)**	**0.24 (0.06, 0.93)**	**0.13 (0.01, 0.69)**	0.55 (0.17, 1.85)	1.42 (0.30, 8.13)	1.87 (0.45, 8.96)	0.91 (0.28, 3.12)	0.64 (0.26, 1.56)	0.73 (0.25, 2.04)	0.75 (0.26, 2.10)	1.56 (0.39, 7.04)	1.19 (0.66, 2.24)	0.83 (0.33, 1.99)	0.39 (0.09, 1.57)	0.52 (0.17, 1.63)	0.67 (0.22, 2.03)	0.83 (0.26, 2.75)	**16.14 (2.58, 289.24)**
GUS600mgIV	0.62 (0.20, 2.05)	0.92 (0.30, 2.84)	0.96 (0.31, 3.05)	0.57 (0.15, 2.21)	**0.35 (0.15, 0.73)**	**0.22 (0.05, 0.86)**	**0.12 (0.01, 0.65)**	0.51 (0.16, 1.70)	1.30 (0.28, 7.24)	1.72 (0.44, 7.87)	0.84 (0.25, 2.87)	0.59 (0.24, 1.41)	0.67 (0.22, 1.83)	0.69 (0.24, 1.93)	1.44 (0.39, 6.31)	1.10 (0.61, 2.05)	0.76 (0.31, 1.79)	0.36 (0.09, 1.45)	0.49 (0.16, 1.51)	0.62 (0.21, 1.86)	0.76 (0.24, 2.47)	**7.06 (1.22, 123.89)**
MED700IV	0.96 (0.28, 3.37)	1.41 (0.42, 4.76)	1.45 (0.43, 5.01)	0.87 (0.22, 3.63)	0.54 (0.21, 1.30)	0.35 (0.08, 1.37)	**0.19 (0.02, 0.99)**	0.78 (0.23, 2.76)	1.98 (0.40, 12.24)	2.61 (0.66, 13.06)	1.30 (0.36, 4.76)	0.91 (0.33, 2.43)	1.02 (0.33, 3.12)	1.06 (0.33, 3.21)	2.18 (0.55, 10.42)	1.72 (0.52, 5.56)	1.17 (0.43, 3.09)	0.56 (0.13, 2.28)	0.75 (0.22, 2.52)	0.95 (0.29, 3.17)	1.17 (0.34, 4.19)	**6.71 (1.14, 121.35)**
NAT300mgIV	1.31 (0.54, 3.45)	1.95 (0.82, 4.97)	2.03 (0.85, 5.24)	1.21 (0.40, 3.89)	0.74 (0.49, 1.09)	0.47 (0.14, 1.49)	0.27 (0.03, 1.13)	1.08 (0.42, 2.94)	2.74 (0.73, 13.37)	**3.59 (1.20, 14.99)**	1.79 (0.67, 4.97)	1.26 (0.72, 2.30)	1.42 (0.64, 3.10)	1.47 (0.67, 3.16)	**2.99 (1.04, 11.78)**	2.34 (1.00, 5.91)	1.62 (0.94, 2.83)	0.78 (0.22, 2.64)	1.03 (0.42, 2.55)	1.31 (0.57, 3.20)	1.61 (0.65, 4.29)	**13.26 (2.43, 239.54)**

(TABLE S12I. continued)

	NAT3mg/kgIV	NAT3mg/kgIVx2	NAT6mg/kgIVx2	NNC2mg/kgSC	PBO	PF10mgSC	PF200mgSC	PF50mgSC	RIS200mgIV	RIS600mgIV	TES400mg200mgSC	UST130mgIV	UST1mg/kgIV	UST3mg/kgIV	UST4.5mg/kgIV	UST6mg/kg90mgIV/SC	UST6mg/kgIV	UST90mgSC	VED0_5mg/kgIV	VED2mg/kgIV	VED300mgIV	SEC10mg/kgIV
NAT3mg/kgIV	NAT3mg/kgIV	1.46 (0.75, 2.92)	1.54 (0.77, 3.13)	0.91 (0.24, 3.67)	0.57 (0.23, 1.25)	0.36 (0.09, 1.42)	**0.20 (0.02, 0.95)**	0.83 (0.24, 2.78)	2.07 (0.47, 11.97)	2.74 (0.72, 12.91)	1.39 (0.38, 4.68)	0.96 (0.36, 2.44)	1.08 (0.35, 3.09)	1.11 (0.37, 3.12)	2.29 (0.61, 10.53)	1.79 (0.55, 5.35)	1.25 (0.47, 3.05)	0.58 (0.14, 2.38)	0.79 (0.24, 2.54)	1.00 (0.31, 3.09)	1.23 (0.38, 4.11)	**12.19 (2.20, 219.82)**
NAT3mg/kgIVx2	0.68 (0.34, 1.34)	NAT3mg/kgIVx2	1.05 (0.56, 1.97)	0.62 (0.17, 2.41)	**0.38 (0.17, 0.82)**	**0.24 (0.06, 0.92)**	**0.13 (0.01, 0.63)**	0.56 (0.17, 1.83)	1.39 (0.34, 7.64)	1.86 (0.51, 8.68)	0.94 (0.27, 3.14)	0.65 (0.26, 1.57)	0.73 (0.25, 2.06)	0.76 (0.26, 2.16)	1.55 (0.43, 6.96)	1.20 (0.39, 3.66)	0.84 (0.33, 1.98)	0.40 (0.10, 1.65)	0.53 (0.17, 1.66)	0.67 (0.23, 2.06)	0.83 (0.26, 2.68)	12.93 (0.22, 1365.26)
NAT6mg/kgIVx2	0.65 (0.32, 1.30)	0.95 (0.51, 1.80)	NAT6mg/kgIVx2	0.59 (0.16, 2.31)	**0.37 (0.16, 0.81)**	**0.23 (0.06, 0.89)**	**0.13 (0.01, 0.61)**	0.54 (0.16, 1.83)	1.34 (0.31, 7.84)	1.79 (0.47, 8.57)	0.90 (0.26, 3.01)	0.62 (0.24, 1.53)	0.70 (0.23, 1.93)	0.73 (0.24, 2.00)	1.49 (0.39, 6.90)	1.15 (0.37, 3.49)	0.80 (0.32, 1.97)	0.39 (0.09, 1.49)	0.51 (0.16, 1.56)	0.65 (0.22, 1.98)	0.80 (0.25, 2.65)	13.74 (0.18, 1064.90)
NNC2mg/kgSC	1.10 (0.27, 4.09)	1.62 (0.41, 5.85)	1.70 (0.43, 6.14)	NNC2mg/kgSC	0.62 (0.20, 1.70)	0.39 (0.08, 1.81)	0.22 (0.02, 1.20)	0.88 (0.21, 3.60)	2.29 (0.43, 13.86)	2.99 (0.67, 16.11)	1.49 (0.35, 6.09)	1.04 (0.32, 3.11)	1.16 (0.33, 4.11)	1.21 (0.34, 4.08)	2.49 (0.56, 12.98)	1.94 (0.48, 7.32)	1.34 (0.41, 4.01)	0.64 (0.13, 2.92)	0.86 (0.21, 3.13)	1.09 (0.28, 3.96)	1.34 (0.34, 5.15)	13.42 (0.21, 1207.63)
PBO	1.76 (0.80, 4.32)	**2.61 (1.21, 6.05)**	**2.72 (1.23, 6.40)**	1.62 (0.59, 5.04)	PBO	0.64 (0.20, 1.87)	0.36 (0.04, 1.39)	1.47 (0.62, 3.57)	**3.69 (1.04, 17.58)**	**4.82 (1.72, 18.94)**	2.41 (0.99, 6.37)	**1.69 (1.12, 2.65)**	1.91 (0.97, 3.81)	1.98 (0.99, 3.91)	**4.02 (1.52, 15.13)**	**3.15 (1.51, 7.17)**	**2.18 (1.49, 3.23)**	1.05 (0.33, 3.29)	1.39 (0.63, 3.13)	1.76 (0.85, 3.94)	2.18 (0.96, 5.38)	12.53 (0.17, 921.15)
PF10mgSC	2.78 (0.70, 11.39)	**4.11 (1.09, 16.01)**	**4.31 (1.13, 16.90)**	2.58 (0.55, 12.32)	1.57 (0.53, 4.96)	PF10mgSC	0.55 (0.06, 2.58)	2.23 (0.86, 7.06)	**5.92 (1.15, 42.40)**	**7.64 (1.65, 47.61)**	3.79 (0.92, 16.73)	2.64 (0.83, 9.11)	3.01 (0.85, 11.34)	3.10 (0.85, 11.80)	**6.35 (1.40, 37.69)**	**4.95 (1.30, 20.43)**	**3.43 (1.08, 11.58)**	1.60 (0.33, 8.35)	2.17 (0.57, 8.85)	2.79 (0.76, 11.20)	3.42 (0.87, 14.62)	**17.96 (2.79, 301.28)**
PF200mgSC	**5.11 (1.05, 46.98)**	**7.45 (1.58, 68.59)**	**7.71 (1.63, 72.31)**	4.62 (0.83, 47.17)	2.77 (0.72, 23.45)	1.82 (0.39, 15.55)	PF200mgSC	**4.07 (1.08, 33.29)**	**10.71 (1.56, 152.78)**	**14.39 (2.46, 164.20)**	**6.80 (1.31, 70.02)**	**4.69 (1.13, 41.35)**	**5.25 (1.16, 50.68)**	**5.44 (1.21, 52.50)**	**12.00 (2.09, 131.63)**	**8.97 (1.74, 90.84)**	**6.00 (1.48, 53.20)**	2.89 (0.49, 33.94)	3.92 (0.77, 38.12)	**4.97 (1.05, 46.47)**	**6.22 (1.18, 57.40)**	**22.55 (3.34, 421.81)**
PF50mgSC	1.21 (0.36, 4.19)	1.79 (0.55, 5.84)	1.86 (0.55, 6.39)	1.13 (0.28, 4.69)	0.68 (0.28, 1.60)	0.45 (0.14, 1.16)	**0.25 (0.03, 0.93)**	PF50mgSC	2.58 (0.54, 15.77)	3.37 (0.87, 17.19)	1.65 (0.47, 6.02)	1.16 (0.44, 3.14)	1.30 (0.42, 4.09)	1.35 (0.43, 4.20)	2.82 (0.73, 13.48)	2.16 (0.66, 7.05)	1.50 (0.57, 3.90)	0.71 (0.17, 3.13)	0.95 (0.28, 3.11)	1.21 (0.38, 3.88)	1.50 (0.45, 5.22)	**14.70 (2.36, 263.26)**
RIS200mgIV	0.48 (0.08, 2.12)	0.72 (0.13, 2.95)	0.74 (0.13, 3.28)	0.44 (0.07, 2.32)	**0.27 (0.06, 0.96)**	**0.17 (0.02, 0.87)**	**0.09 (0.01, 0.64)**	0.39 (0.06, 1.85)	RIS200mgIV	1.32 (0.49, 3.68)	0.66 (0.10, 3.24)	0.46 (0.09, 1.75)	0.52 (0.09, 2.18)	0.54 (0.10, 2.15)	1.10 (0.35, 3.30)	0.85 (0.15, 3.88)	0.59 (0.12, 2.25)	0.28 (0.04, 1.58)	0.38 (0.06, 1.67)	0.47 (0.08, 2.13)	0.60 (0.10, 2.74)	**35.91 (4.24, 678.06)**
RIS600mgIV	0.37 (0.08, 1.38)	0.54 (0.12, 1.97)	0.56 (0.12, 2.14)	0.33 (0.06, 1.49)	**0.21 (0.05, 0.58)**	**0.13 (0.02, 0.60)**	**0.07 (0.01, 0.41)**	0.30 (0.06, 1.15)	0.75 (0.27, 2.05)	RIS600mgIV	0.50 (0.09, 2.02)	0.35 (0.09, 1.03)	0.39 (0.08, 1.29)	0.41 (0.09, 1.37)	0.84 (0.46, 1.33)	0.64 (0.14, 2.56)	0.45 (0.11, 1.34)	**0.21 (0.04, 0.99)**	0.29 (0.06, 1.04)	0.37 (0.07, 1.30)	0.45 (0.09, 1.74)	**10.39 (1.93, 179.52)**
TES400mg200mgSC	0.72 (0.21, 2.63)	1.07 (0.32, 3.73)	1.12 (0.33, 3.90)	0.67 (0.16, 2.85)	0.41 (0.16, 1.01)	0.26 (0.06, 1.08)	**0.15 (0.01, 0.76)**	0.61 (0.17, 2.11)	1.51 (0.31, 10.09)	2.01 (0.50, 10.76)	TES400mg200mgSC	0.70 (0.24, 1.94)	0.80 (0.24, 2.38)	0.82 (0.25, 2.55)	1.67 (0.42, 8.47)	1.32 (0.38, 4.39)	0.92 (0.32, 2.41)	0.43 (0.10, 1.77)	0.57 (0.17, 1.92)	0.74 (0.21, 2.47)	0.91 (0.25, 3.28)	**17.15 (2.11, 306.93)**
UST130mgIV	1.04 (0.41, 2.80)	1.54 (0.64, 3.90)	1.60 (0.65, 4.12)	0.96 (0.32, 3.14)	**0.59 (0.38, 0.89)**	0.38 (0.11, 1.21)	**0.21 (0.02, 0.88)**	0.87 (0.32, 2.29)	2.16 (0.57, 10.82)	2.86 (0.97, 11.74)	1.42 (0.51, 4.14)	UST130mgIV	1.13 (0.54, 2.33)	1.17 (0.55, 2.41)	2.39 (0.81, 9.52)	1.86 (0.79, 4.53)	1.29 (0.85, 1.94)	0.62 (0.18, 2.11)	0.82 (0.33, 2.04)	1.05 (0.44, 2.52)	1.30 (0.51, 3.50)	**13.89 (1.73, 249.76)**
UST1mg/kgIV	0.93 (0.32, 2.89)	1.37 (0.49, 4.07)	1.43 (0.52, 4.35)	0.87 (0.24, 3.06)	0.52 (0.26, 1.03)	0.33 (0.09, 1.18)	**0.19 (0.02, 0.86)**	0.77 (0.24, 2.36)	1.92 (0.46, 10.75)	2.53 (0.77, 11.85)	1.26 (0.42, 4.16)	0.89 (0.43, 1.86)	UST1mg/kgIV	1.04 (0.50, 2.11)	2.11 (0.64, 9.35)	1.64 (0.61, 4.77)	1.15 (0.60, 2.21)	0.54 (0.14, 2.17)	0.73 (0.25, 2.05)	0.93 (0.33, 2.62)	1.15 (0.40, 3.57)	**22.28 (2.55, 566.57)**
UST3mg/kgIV	0.90 (0.32, 2.70)	1.31 (0.46, 3.90)	1.37 (0.50, 4.11)	0.83 (0.24, 2.97)	0.51 (0.26, 1.01)	0.32 (0.08, 1.17)	**0.18 (0.02, 0.83)**	0.74 (0.24, 2.33)	1.86 (0.47, 10.13)	2.43 (0.73, 11.50)	1.22 (0.39, 3.94)	0.86 (0.41, 1.80)	0.97 (0.47, 2.00)	UST3mg/kgIV	2.03 (0.61, 9.12)	1.58 (0.58, 4.49)	1.10 (0.59, 2.13)	0.52 (0.14, 1.95)	0.70 (0.25, 2.08)	0.89 (0.33, 2.62)	1.10 (0.39, 3.51)	**27.51 (4.01, 518.65)**
UST4.5mg/kgIV	0.44 (0.10, 1.64)	0.64 (0.14, 2.32)	0.67 (0.14, 2.56)	0.40 (0.08, 1.79)	**0.25 (0.07, 0.66)**	**0.16 (0.03, 0.71)**	**0.08 (0.01, 0.48)**	0.36 (0.07, 1.38)	0.91 (0.30, 2.88)	1.19 (0.75, 2.19)	0.60 (0.12, 2.41)	0.42 (0.11, 1.23)	0.47 (0.11, 1.56)	0.49 (0.11, 1.64)	UST4.5mg/kgIV	0.77 (0.17, 2.89)	0.54 (0.14, 1.59)	0.25 (0.04, 1.16)	0.34 (0.07, 1.26)	0.44 (0.09, 1.56)	0.54 (0.11, 2.07)	**11.79 (2.25, 201.65)**
UST6mg/kg90mgIV/SC	0.56 (0.19, 1.81)	0.83 (0.27, 2.56)	0.87 (0.29, 2.73)	0.52 (0.14, 2.08)	**0.32 (0.14, 0.66)**	**0.20 (0.05, 0.77)**	**0.11 (0.01, 0.58)**	0.46 (0.14, 1.52)	1.18 (0.26, 6.61)	1.56 (0.39, 7.16)	0.75 (0.23, 2.64)	0.54 (0.22, 1.26)	0.61 (0.21, 1.63)	0.63 (0.22, 1.71)	1.30 (0.35, 5.78)	UST6mg/kg90mgIV/SC	0.70 (0.28, 1.63)	0.33 (0.08, 1.32)	0.44 (0.15, 1.34)	0.56 (0.19, 1.68)	0.69 (0.22, 2.23)	5.39 (0.05, 180.69)
UST6mg/kgIV	0.80 (0.33, 2.11)	1.19 (0.51, 2.99)	1.25 (0.51, 3.15)	0.74 (0.25, 2.44)	**0.46 (0.31, 0.67)**	**0.29 (0.09, 0.92)**	**0.17 (0.02, 0.68)**	0.67 (0.26, 1.76)	1.69 (0.44, 8.51)	2.22 (0.74, 9.24)	1.09 (0.41, 3.11)	0.77 (0.52, 1.17)	0.87 (0.45, 1.66)	0.91 (0.47, 1.69)	1.84 (0.63, 7.28)	1.44 (0.61, 3.56)	UST6mg/kgIV	0.47 (0.14, 1.63)	0.63 (0.26, 1.59)	0.81 (0.35, 1.98)	1.00 (0.41, 2.67)	3.07 (0.04, 110.04)
UST90mgSC	1.71 (0.42, 7.25)	2.51 (0.60, 10.39)	2.59 (0.67, 10.54)	1.57 (0.34, 7.52)	0.95 (0.30, 3.04)	0.62 (0.12, 3.01)	0.35 (0.03, 2.04)	1.41 (0.32, 5.90)	3.57 (0.63, 25.68)	**4.70 (1.01, 28.42)**	2.32 (0.56, 10.20)	1.61 (0.47, 5.66)	1.84 (0.46, 7.16)	1.91 (0.51, 7.31)	3.95 (0.86, 23.12)	3.04 (0.76, 12.30)	2.11 (0.61, 7.03)	UST90mgSC	1.34 (0.31, 5.45)	1.71 (0.43, 6.84)	2.10 (0.52, 9.03)	5.58 (0.07, 216.99)
VED0_5mg/kgIV	1.27 (0.39, 4.14)	1.89 (0.60, 5.98)	1.96 (0.64, 6.24)	1.17 (0.32, 4.71)	0.72 (0.32, 1.58)	0.46 (0.11, 1.75)	0.25 (0.03, 1.29)	1.05 (0.32, 3.53)	2.64 (0.60, 16.21)	3.46 (0.96, 17.61)	1.74 (0.52, 5.91)	1.22 (0.49, 3.03)	1.37 (0.49, 3.98)	1.43 (0.48, 4.00)	2.90 (0.80, 13.63)	2.26 (0.75, 6.75)	1.58 (0.63, 3.80)	0.75 (0.18, 3.21)	VED0_5mg/kgIV	1.28 (0.64, 2.59)	1.58 (0.50, 5.02)	**67.40 (9.65, 1432.49)**
VED2mg/kgIV	1.00 (0.32, 3.20)	1.49 (0.49, 4.40)	1.54 (0.51, 4.60)	0.92 (0.25, 3.54)	0.57 (0.25, 1.17)	0.36 (0.09, 1.32)	**0.20 (0.02, 0.96)**	0.82 (0.26, 2.62)	2.11 (0.47, 12.05)	2.72 (0.77, 13.46)	1.35 (0.40, 4.67)	0.96 (0.40, 2.25)	1.08 (0.38, 3.03)	1.12 (0.38, 3.05)	2.27 (0.64, 10.80)	1.78 (0.59, 5.19)	1.23 (0.50, 2.84)	0.58 (0.15, 2.30)	0.78 (0.39, 1.57)	VED2mg/kgIV	1.23 (0.40, 3.96)	**57.30 (3.65, 5969.84)**
VED300mgIV	0.81 (0.24, 2.66)	1.20 (0.37, 3.83)	1.25 (0.38, 3.96)	0.74 (0.19, 2.96)	0.46 (0.19, 1.04)	0.29 (0.07, 1.15)	**0.16 (0.02, 0.85)**	0.67 (0.19, 2.22)	1.68 (0.37, 10.23)	2.23 (0.58, 11.26)	1.10 (0.31, 3.92)	0.77 (0.29, 1.97)	0.87 (0.28, 2.49)	0.91 (0.29, 2.56)	1.86 (0.48, 8.95)	1.45 (0.45, 4.60)	1.00 (0.37, 2.42)	0.48 (0.11, 1.94)	0.63 (0.20, 1.99)	0.81 (0.25, 2.47)	VED300mgIV	**48.84 (7.67, 936.50)**
SEC10mg/kgIV	**0.08 (0.00, 0.45)**	0.08 (0.00, 4.63)	0.07 (0.00, 5.67)	0.07 (0.00, 4.69)	0.08 (0.00, 5.92)	**0.06 (0.00, 0.36)**	**0.04 (0.00, 0.30)**	**0.07 (0.00, 0.42)**	**0.03 (0.00, 0.24)**	**0.10 (0.01, 0.52)**	**0.06 (0.00, 0.47)**	**0.07 (0.00, 0.58)**	**0.04 (0.00, 0.39)**	**0.04 (0.00, 0.25)**	**0.08 (0.00, 0.45)**	0.19 (0.01, 18.74)	0.33 (0.01, 26.62)	0.18 (0.00, 13.33)	**0.01 (0.00, 0.10)**	**0.02 (0.00, 0.27)**	**0.02 (0.00, 0.13)**	SEC10mg/kgIV

(TABLE S12I. continued)

	NAT3mg/kgIV	NAT3mg/kgIVx2	NAT6mg/kgIVx2	NNC2mg/kgSC	PBO	PF10mgSC	PF200mgSC	PF50mgSC	RIS200mgIV	RIS600mgIV	TES400mg200mgSC	UST130mgIV	UST1mg/kgIV	UST3mg/kgIV	UST4.5mg/kgIV	UST6mg/kg90mgIV/SC	UST6mg/kgIV	UST90mgSC	VED0_5mg/kgIV	VED2mg/kgIV	VED300mgIV	SEC10mg/kgIV
SEM60mgIV	0.88 (0.39, 2.13)	1.04 (0.02, 41.08)	0.88 (0.03, 52.78)	0.88 (0.03, 43.39)	1.01 (0.02, 59.63)	0.63 (0.20, 1.86)	0.51 (0.16, 1.58)	0.74 (0.26, 2.14)	0.31 (0.07, 1.24)	1.03 (0.47, 2.43)	0.68 (0.19, 2.55)	0.83 (0.25, 3.15)	0.52 (0.12, 2.19)	0.42 (0.12, 1.37)	0.90 (0.43, 1.99)	2.12 (0.18, 144.20)	3.58 (0.29, 221.30)	1.96 (0.17, 108.53)	**0.17 (0.05, 0.55)**	0.21 (0.01, 1.67)	**0.23 (0.07, 0.66)**	**11.16 (1.68, 191.40)**
SEM60mgIVx3	1.49 (0.61, 4.08)	1.76 (0.03, 69.15)	1.45 (0.05, 86.48)	1.53 (0.04, 78.29)	1.70 (0.04, 102.95)	1.07 (0.31, 3.44)	0.85 (0.26, 2.95)	1.27 (0.40, 3.91)	0.54 (0.11, 2.36)	1.76 (0.73, 4.63)	1.18 (0.30, 4.76)	1.43 (0.40, 5.63)	0.91 (0.19, 4.00)	0.71 (0.19, 2.50)	1.54 (0.66, 3.81)	3.80 (0.30, 277.17)	6.26 (0.46, 384.50)	3.38 (0.27, 176.49)	0.28 (0.07, 1.04)	0.35 (0.01, 3.05)	0.39 (0.11, 1.29)	**19.31 (2.77, 372.88)**
TES400mg200mgSC	**0.35 (0.14, 0.81)**	0.41 (0.01, 17.10)	0.35 (0.01, 21.01)	0.35 (0.01, 19.05)	0.38 (0.01, 25.41)	**0.25 (0.07, 0.75)**	**0.20 (0.06, 0.62)**	**0.29 (0.09, 0.85)**	**0.13 (0.03, 0.48)**	**0.42 (0.17, 0.92)**	0.28 (0.07, 1.01)	0.33 (0.10, 1.21)	**0.21 (0.04, 0.84)**	**0.17 (0.04, 0.53)**	**0.36 (0.15, 0.76)**	0.84 (0.07, 60.82)	1.39 (0.11, 91.38)	0.80 (0.06, 39.90)	**0.07 (0.02, 0.21)**	**0.08 (0.00, 0.73)**	**0.09 (0.03, 0.27)**	4.51 (0.65, 80.81)
TOF15mgOR_BID	1.65 (0.55, 5.64)	1.94 (0.03, 89.77)	1.58 (0.05, 112.65)	1.63 (0.04, 91.72)	1.74 (0.04, 121.51)	1.18 (0.27, 4.84)	0.94 (0.24, 4.05)	1.36 (0.37, 5.78)	0.59 (0.12, 2.97)	1.93 (0.65, 6.40)	1.28 (0.27, 6.42)	1.54 (0.38, 7.38)	1.01 (0.17, 5.14)	0.78 (0.17, 3.30)	1.69 (0.59, 5.40)	4.04 (0.32, 299.52)	6.93 (0.50, 406.98)	3.88 (0.26, 211.63)	0.31 (0.07, 1.32)	0.39 (0.01, 3.82)	0.41 (0.11, 1.73)	**21.08 (2.71, 432.60)**
TOF1mgOR_BID	1.73 (0.56, 5.99)	2.10 (0.03, 97.05)	1.68 (0.05, 106.74)	1.67 (0.05, 92.61)	1.82 (0.04, 122.51)	1.25 (0.30, 4.91)	0.98 (0.24, 4.24)	1.44 (0.40, 6.02)	0.62 (0.12, 3.18)	2.01 (0.66, 6.91)	1.38 (0.27, 6.63)	1.62 (0.40, 8.15)	1.04 (0.20, 5.34)	0.81 (0.18, 3.61)	1.75 (0.60, 5.83)	4.09 (0.32, 303.09)	7.14 (0.49, 442.67)	3.94 (0.28, 261.21)	0.33 (0.07, 1.42)	0.39 (0.01, 3.89)	0.44 (0.11, 1.83)	**21.55 (2.77, 502.13)**
TOF5mgOR_BID	0.97 (0.37, 2.73)	1.15 (0.02, 46.15)	1.00 (0.03, 62.21)	0.96 (0.03, 52.98)	1.03 (0.02, 71.73)	0.70 (0.19, 2.48)	0.56 (0.15, 2.02)	0.81 (0.25, 2.89)	0.35 (0.07, 1.46)	1.14 (0.45, 3.11)	0.77 (0.18, 3.20)	0.92 (0.24, 3.76)	0.59 (0.12, 2.72)	0.47 (0.12, 1.71)	1.00 (0.40, 2.60)	2.42 (0.19, 171.80)	4.06 (0.30, 245.23)	2.26 (0.16, 143.79)	**0.18 (0.05, 0.72)**	0.23 (0.01, 2.07)	**0.25 (0.07, 0.85)**	**12.31 (1.73, 278.46)**
UPA12mgOR_BID	0.71 (0.31, 1.52)	0.80 (0.01, 34.45)	0.69 (0.02, 45.88)	0.70 (0.02, 36.46)	0.78 (0.02, 50.85)	0.50 (0.17, 1.40)	0.40 (0.13, 1.16)	0.58 (0.21, 1.63)	**0.25 (0.06, 0.94)**	0.83 (0.37, 1.73)	0.55 (0.16, 1.89)	0.67 (0.19, 2.26)	0.42 (0.10, 1.63)	0.33 (0.09, 1.01)	0.72 (0.34, 1.43)	1.68 (0.15, 119.01)	2.79 (0.24, 173.01)	1.57 (0.13, 83.80)	**0.13 (0.04, 0.40)**	0.17 (0.01, 1.33)	**0.18 (0.06, 0.50)**	**8.62 (1.41, 159.63)**
UPA24mgOR	0.91 (0.39, 2.14)	1.02 (0.02, 44.41)	0.90 (0.03, 61.63)	0.92 (0.02, 46.29)	1.02 (0.02, 71.65)	0.65 (0.21, 1.90)	0.52 (0.16, 1.61)	0.76 (0.27, 2.25)	0.33 (0.07, 1.23)	1.07 (0.47, 2.42)	0.72 (0.20, 2.65)	0.87 (0.23, 3.16)	0.53 (0.12, 2.29)	0.43 (0.12, 1.41)	0.94 (0.43, 2.01)	2.22 (0.20, 154.04)	3.66 (0.31, 229.16)	2.06 (0.17, 105.29)	**0.17 (0.05, 0.56)**	0.21 (0.01, 1.90)	**0.24 (0.07, 0.68)**	**11.38 (1.80, 202.39)**
UPA24mgOR_BID	0.56 (0.26, 1.18)	0.65 (0.01, 26.07)	0.56 (0.02, 34.58)	0.54 (0.02, 29.93)	0.60 (0.01, 39.48)	0.40 (0.13, 1.10)	**0.32 (0.10, 0.91)**	0.46 (0.17, 1.24)	**0.20 (0.05, 0.72)**	0.65 (0.30, 1.33)	0.44 (0.13, 1.49)	0.53 (0.16, 1.80)	0.33 (0.07, 1.27)	**0.27 (0.07, 0.78)**	0.57 (0.28, 1.09)	1.34 (0.13, 95.70)	2.19 (0.20, 135.47)	1.26 (0.10, 66.76)	**0.11 (0.03, 0.32)**	0.13 (0.00, 1.03)	**0.15 (0.05, 0.38)**	**6.86 (1.16, 128.47)**
UPA3mgOR_BID	0.76 (0.34, 1.67)	0.88 (0.02, 36.51)	0.75 (0.02, 48.86)	0.75 (0.02, 38.62)	0.85 (0.02, 55.14)	0.54 (0.18, 1.51)	0.44 (0.14, 1.27)	0.63 (0.23, 1.77)	**0.28 (0.06, 0.98)**	0.89 (0.40, 1.90)	0.59 (0.17, 2.05)	0.72 (0.20, 2.49)	0.44 (0.10, 1.81)	0.36 (0.10, 1.11)	0.78 (0.37, 1.57)	1.81 (0.16, 125.44)	3.07 (0.25, 179.60)	1.73 (0.14, 89.05)	**0.14 (0.04, 0.45)**	0.18 (0.01, 1.55)	**0.20 (0.06, 0.55)**	**9.36 (1.53, 165.24)**
UPA45mgOR	0.72 (0.24, 2.05)	0.81 (0.01, 35.54)	0.74 (0.02, 45.17)	0.73 (0.02, 36.65)	0.81 (0.02, 49.24)	0.51 (0.13, 1.82)	0.41 (0.11, 1.56)	0.61 (0.16, 2.09)	0.26 (0.05, 1.11)	0.86 (0.28, 2.33)	0.57 (0.12, 2.32)	0.69 (0.16, 2.71)	0.43 (0.08, 1.94)	0.34 (0.08, 1.29)	0.74 (0.25, 1.92)	1.74 (0.12, 115.40)	2.96 (0.19, 194.95)	1.72 (0.12, 105.29)	**0.14 (0.03, 0.51)**	0.16 (0.01, 1.63)	**0.19 (0.05, 0.64)**	**9.28 (1.20, 177.84)**
UPA6mgOR_BID	0.61 (0.28, 1.28)	0.72 (0.01, 29.33)	0.60 (0.02, 40.60)	0.59 (0.02, 30.49)	0.67 (0.02, 46.54)	0.43 (0.14, 1.18)	**0.35 (0.11, 0.97)**	0.51 (0.19, 1.39)	**0.22 (0.05, 0.77)**	0.71 (0.33, 1.43)	0.48 (0.14, 1.65)	0.58 (0.17, 1.99)	0.36 (0.08, 1.39)	**0.29 (0.08, 0.87)**	0.63 (0.31, 1.18)	1.46 (0.13, 101.12)	2.39 (0.21, 149.75)	1.39 (0.12, 78.15)	**0.11 (0.03, 0.34)**	0.14 (0.00, 1.20)	**0.16 (0.05, 0.42)**	**7.46 (1.24, 129.66)**
UST130mgIV	1.19 (0.76, 2.08)	1.45 (0.03, 53.27)	1.20 (0.04, 72.83)	1.18 (0.04, 58.75)	1.30 (0.03, 84.66)	0.86 (0.33, 1.98)	0.68 (0.27, 1.73)	1.01 (0.45, 2.31)	0.44 (0.11, 1.38)	1.40 (0.93, 2.20)	0.95 (0.31, 2.82)	1.13 (0.40, 3.43)	0.73 (0.18, 2.47)	0.57 (0.19, 1.46)	1.22 (0.89, 1.74)	2.86 (0.28, 191.08)	4.71 (0.42, 284.07)	2.74 (0.24, 135.01)	**0.23 (0.07, 0.58)**	0.29 (0.01, 2.13)	**0.31 (0.12, 0.73)**	**14.67 (2.71, 254.46)**
UST1mg/kgIV	0.89 (0.50, 1.70)	1.03 (0.02, 41.15)	0.88 (0.03, 54.56)	0.88 (0.03, 43.22)	0.97 (0.02, 63.95)	0.64 (0.24, 1.64)	0.51 (0.18, 1.38)	0.75 (0.30, 1.91)	0.33 (0.08, 1.07)	1.04 (0.60, 1.84)	0.71 (0.21, 2.24)	0.85 (0.29, 2.73)	0.54 (0.13, 1.97)	0.42 (0.14, 1.17)	0.91 (0.56, 1.51)	2.13 (0.20, 142.35)	3.56 (0.31, 213.11)	2.07 (0.17, 103.50)	**0.17 (0.05, 0.47)**	0.22 (0.01, 1.63)	**0.23 (0.09, 0.57)**	**11.14 (1.91, 188.75)**
UST3mg/kgIV	1.01 (0.56, 2.01)	1.19 (0.02, 44.24)	1.01 (0.04, 63.40)	0.99 (0.03, 50.89)	1.12 (0.03, 71.18)	0.72 (0.26, 1.84)	0.57 (0.21, 1.58)	0.85 (0.35, 2.18)	0.37 (0.09, 1.23)	1.19 (0.67, 2.20)	0.80 (0.24, 2.53)	0.96 (0.31, 3.10)	0.61 (0.15, 2.19)	0.48 (0.15, 1.34)	1.03 (0.64, 1.78)	2.40 (0.22, 164.93)	4.05 (0.36, 241.20)	2.30 (0.20, 115.73)	**0.20 (0.06, 0.53)**	0.24 (0.01, 1.98)	**0.27 (0.10, 0.66)**	**12.43 (2.17, 219.02)**
UST4.5mg/kgIV	1.76 (0.52, 7.07)	2.03 (0.03, 87.32)	1.70 (0.06, 112.44)	1.69 (0.04, 86.20)	1.89 (0.04, 123.43)	1.23 (0.28, 5.56)	0.99 (0.24, 4.87)	1.47 (0.36, 6.39)	0.64 (0.10, 3.41)	2.05 (0.61, 7.96)	1.37 (0.28, 7.19)	1.64 (0.36, 8.67)	1.06 (0.19, 5.75)	0.82 (0.17, 4.03)	1.78 (0.56, 6.81)	4.29 (0.28, 408.76)	7.28 (0.46, 516.69)	4.10 (0.27, 273.96)	0.33 (0.07, 1.68)	0.41 (0.01, 4.46)	0.45 (0.11, 2.28)	**22.37 (2.63, 548.36)**
UST6mg/kg90mgIV/SC	1.68 (0.70, 4.46)	1.93 (0.03, 87.96)	1.68 (0.05, 101.07)	1.69 (0.05, 82.07)	1.85 (0.04, 128.98)	1.20 (0.46, 3.17)	0.96 (0.38, 2.50)	1.39 (0.61, 3.56)	0.61 (0.13, 2.44)	1.97 (0.83, 4.99)	1.35 (0.34, 5.12)	1.62 (0.46, 6.13)	0.99 (0.21, 4.08)	0.80 (0.21, 2.73)	1.73 (0.77, 4.09)	4.22 (0.35, 274.97)	6.98 (0.53, 405.40)	3.83 (0.30, 199.42)	0.32 (0.09, 1.10)	0.40 (0.01, 3.66)	0.44 (0.14, 1.49)	**21.18 (3.22, 402.00)**
UST6mg/kgIV	0.98 (0.65, 1.63)	1.13 (0.02, 44.42)	0.99 (0.03, 60.95)	0.97 (0.03, 47.17)	1.06 (0.03, 66.61)	0.71 (0.28, 1.61)	0.56 (0.23, 1.39)	0.82 (0.37, 1.85)	0.36 (0.09, 1.09)	1.15 (0.78, 1.75)	0.78 (0.26, 2.28)	0.93 (0.32, 2.75)	0.59 (0.15, 1.99)	0.46 (0.16, 1.17)	1.00 (0.75, 1.34)	2.35 (0.23, 161.10)	3.87 (0.34, 233.64)	2.27 (0.19, 111.93)	**0.19 (0.06, 0.47)**	0.24 (0.01, 1.70)	**0.25 (0.10, 0.58)**	**11.91 (2.22, 204.61)**
UST90mgSC	1.18 (0.31, 4.88)	1.32 (0.02, 65.69)	1.15 (0.03, 68.09)	1.18 (0.02, 65.57)	1.31 (0.03, 86.84)	0.86 (0.17, 3.77)	0.66 (0.15, 3.18)	0.99 (0.23, 4.39)	0.42 (0.07, 2.30)	1.39 (0.36, 5.57)	0.93 (0.17, 5.14)	1.12 (0.20, 6.00)	0.70 (0.11, 4.25)	0.55 (0.10, 2.75)	1.20 (0.33, 4.70)	3.01 (0.18, 203.03)	5.06 (0.28, 323.04)	2.86 (0.15, 166.30)	0.22 (0.04, 1.09)	0.27 (0.01, 3.01)	0.30 (0.06, 1.58)	**15.12 (1.75, 325.75)**
VED0_5mg/kgIV	0.79 (0.03, 24.14)	0.84 (0.00, 163.75)	0.79 (0.00, 164.34)	0.87 (0.01, 104.92)	0.94 (0.01, 196.02)	0.56 (0.02, 17.24)	0.45 (0.01, 13.22)	0.67 (0.02, 20.61)	0.27 (0.01, 10.09)	0.93 (0.03, 27.32)	0.64 (0.02, 20.60)	0.77 (0.02, 27.50)	0.46 (0.01, 16.72)	0.37 (0.01, 12.71)	0.81 (0.03, 23.59)	2.54 (0.04, 249.86)	4.13 (0.07, 413.35)	2.31 (0.03, 187.95)	0.15 (0.00, 4.83)	0.18 (0.00, 10.41)	0.20 (0.01, 6.35)	11.22 (0.19, 1114.72)
VED2mg/kgIV	1.07 (0.04, 29.66)	0.99 (0.01, 154.42)	1.04 (0.01, 163.08)	1.05 (0.01, 131.42)	1.22 (0.01, 172.06)	0.77 (0.02, 20.46)	0.61 (0.02, 18.41)	0.91 (0.03, 25.06)	0.37 (0.01, 12.16)	1.27 (0.04, 34.37)	0.89 (0.02, 28.39)	1.00 (0.03, 34.99)	0.62 (0.02, 23.16)	0.49 (0.01, 16.07)	1.08 (0.04, 29.93)	3.30 (0.04, 318.36)	5.56 (0.08, 624.98)	2.89 (0.03, 300.08)	0.21 (0.01, 5.53)	0.23 (0.00, 10.16)	0.28 (0.01, 8.15)	15.08 (0.30, 1058.74)
VED300mgIV	1.05 (0.55, 2.07)	1.24 (0.02, 48.68)	1.03 (0.04, 62.83)	0.99 (0.03, 52.86)	1.13 (0.03, 75.15)	0.75 (0.24, 1.91)	0.60 (0.21, 1.63)	0.89 (0.34, 2.20)	0.38 (0.09, 1.29)	1.24 (0.64, 2.29)	0.82 (0.25, 2.72)	0.98 (0.33, 3.25)	0.63 (0.15, 2.31)	0.49 (0.15, 1.40)	1.08 (0.60, 1.84)	2.51 (0.23, 175.46)	4.17 (0.35, 241.48)	2.42 (0.20, 117.27)	**0.20 (0.06, 0.56)**	0.25 (0.01, 1.96)	**0.27 (0.09, 0.70)**	**13.18 (2.30, 223.31)**

	SEM60mgIV	SEM60mgIVx3	TES400mg200mgSC	TOF15mgOR_BID	TOF1mgOR_BID	TOF5mgOR_BID	UPA12mgOR_BID	UPA24mgOR	UPA24mgOR_BID	UPA3mgOR_BID	UPA45mgOR	UPA6mgOR_BID	UST130mgIV	UST1mg/kgIV	UST3mg/kgIV	UST4.5mg/kgIV	UST6mg/kg90mgIV/SC	UST6mg/kgIV	UST90mgSC	VED0_5mg/kgIV	VED2mg/kgIV	VED300mgIV
ABA10mg/kgIV	1.40 (0.56, 3.52)	0.83 (0.29, 2.22)	**3.52 (1.43, 9.49)**	0.75 (0.21, 2.39)	0.72 (0.19, 2.32)	1.27 (0.43, 3.55)	1.76 (0.76, 4.30)	1.35 (0.54, 3.45)	2.24 (0.98, 5.28)	1.63 (0.68, 4.09)	1.72 (0.56, 5.60)	2.03 (0.91, 4.86)	1.03 (0.55, 1.91)	1.39 (0.69, 2.81)	1.23 (0.59, 2.49)	0.71 (0.17, 2.51)	0.74 (0.27, 1.89)	1.27 (0.69, 2.30)	1.07 (0.25, 4.15)	1.59 (0.05, 46.31)	1.17 (0.05, 36.58)	1.18 (0.57, 2.55)
ABA30mg/kgIV	1.86 (0.67, 5.16)	1.08 (0.36, 3.26)	**4.60 (1.77, 14.18)**	1.02 (0.26, 3.47)	0.96 (0.25, 3.29)	1.70 (0.54, 5.18)	2.33 (0.93, 6.35)	1.79 (0.67, 5.16)	**2.95 (1.20, 7.75)**	2.15 (0.85, 6.08)	2.29 (0.69, 7.92)	**2.69 (1.08, 7.27)**	1.37 (0.66, 2.94)	1.83 (0.84, 4.20)	1.62 (0.70, 3.71)	0.94 (0.21, 3.75)	0.98 (0.34, 2.75)	1.66 (0.84, 3.58)	1.41 (0.32, 5.86)	2.09 (0.07, 62.53)	1.53 (0.06, 45.70)	1.55 (0.68, 3.88)
ABA3mg/kgIV	1.53 (0.59, 3.78)	0.90 (0.32, 2.62)	**3.85 (1.55, 10.29)**	0.82 (0.23, 2.70)	0.79 (0.21, 2.59)	1.38 (0.48, 3.92)	1.91 (0.84, 4.81)	1.49 (0.59, 3.79)	**2.42 (1.05, 5.76)**	1.77 (0.76, 4.53)	1.87 (0.61, 6.19)	2.22 (0.98, 5.52)	1.13 (0.59, 2.09)	1.51 (0.73, 3.07)	1.33 (0.63, 2.77)	0.78 (0.19, 2.91)	0.81 (0.29, 2.12)	1.38 (0.76, 2.51)	1.15 (0.26, 4.78)	1.71 (0.06, 52.38)	1.29 (0.05, 37.84)	1.28 (0.61, 2.82)
AND150mgSC1/1	1.60 (0.48, 5.34)	0.96 (0.27, 3.30)	**4.07 (1.22, 14.06)**	0.86 (0.19, 3.55)	0.81 (0.18, 3.38)	1.45 (0.37, 5.51)	2.01 (0.66, 6.77)	1.54 (0.49, 5.44)	2.53 (0.83, 8.43)	1.86 (0.59, 6.40)	1.97 (0.53, 8.29)	2.30 (0.77, 7.76)	1.18 (0.44, 3.25)	1.57 (0.55, 4.62)	1.39 (0.47, 4.10)	0.79 (0.17, 3.73)	0.84 (0.55, 1.28)	1.45 (0.55, 3.88)	1.20 (0.24, 6.23)	1.78 (0.05, 63.84)	1.33 (0.04, 41.60)	1.34 (0.46, 4.22)
AND150mgSC2/2	1.20 (0.37, 4.35)	0.71 (0.19, 2.80)	3.09 (0.88, 11.32)	0.66 (0.14, 2.91)	0.63 (0.13, 2.88)	1.09 (0.27, 4.38)	1.55 (0.46, 5.30)	1.18 (0.35, 3.95)	1.95 (0.59, 6.40)	1.41 (0.43, 4.86)	1.50 (0.37, 6.15)	1.76 (0.55, 5.83)	0.89 (0.31, 2.67)	1.20 (0.41, 3.72)	1.05 (0.35, 3.26)	0.62 (0.11, 2.97)	0.64 (0.17, 2.33)	1.10 (0.39, 3.06)	0.93 (0.17, 4.94)	1.41 (0.04, 43.87)	1.01 (0.03, 32.32)	1.02 (0.34, 3.39)
AND300mgSC	1.22 (0.49, 2.85)	0.72 (0.25, 1.84)	**3.06 (1.28, 7.90)**	0.65 (0.18, 2.03)	0.62 (0.18, 1.97)	1.11 (0.38, 2.97)	1.53 (0.67, 3.53)	1.18 (0.50, 2.80)	1.94 (0.89, 4.29)	1.42 (0.62, 3.26)	1.49 (0.51, 4.74)	1.77 (0.81, 3.99)	0.91 (0.50, 1.50)	1.21 (0.62, 2.26)	1.07 (0.53, 1.99)	0.62 (0.15, 2.14)	0.64 (0.24, 1.57)	1.11 (0.64, 1.79)	0.92 (0.22, 3.47)	1.36 (0.04, 40.59)	1.05 (0.04, 31.01)	1.03 (0.50, 2.07)
CDP10mg/kgIV	2.08 (0.69, 6.66)	1.23 (0.36, 3.95)	**5.21 (1.79, 16.90)**	1.13 (0.27, 4.27)	1.08 (0.26, 4.00)	1.91 (0.52, 6.23)	2.61 (0.91, 8.11)	2.01 (0.68, 6.44)	**3.27 (1.21, 10.06)**	2.43 (0.86, 7.46)	2.57 (0.72, 9.78)	**3.02 (1.08, 9.09)**	1.54 (0.65, 3.86)	2.05 (0.82, 5.61)	1.81 (0.70, 4.91)	1.05 (0.21, 4.39)	1.09 (0.34, 3.58)	1.86 (0.81, 4.65)	1.59 (0.33, 7.32)	2.30 (0.07, 66.46)	1.82 (0.06, 55.25)	1.76 (0.67, 4.90)
CER100mgSC	1.25 (0.45, 3.21)	0.74 (0.23, 2.04)	**3.10 (1.17, 8.80)**	0.67 (0.17, 2.25)	0.63 (0.17, 2.21)	1.11 (0.35, 3.41)	1.55 (0.62, 4.10)	1.20 (0.45, 3.30)	1.96 (0.81, 5.03)	1.45 (0.57, 3.80)	1.51 (0.47, 5.38)	1.80 (0.74, 4.64)	0.91 (0.44, 1.82)	1.23 (0.55, 2.65)	1.08 (0.47, 2.40)	0.62 (0.15, 2.42)	0.65 (0.21, 1.79)	1.12 (0.55, 2.16)	0.93 (0.20, 3.87)	1.37 (0.05, 43.35)	1.02 (0.04, 32.85)	1.05 (0.45, 2.38)
CER10mgIV	1.80 (0.02, 57.89)	1.03 (0.01, 35.76)	4.51 (0.04, 146.70)	0.96 (0.01, 36.57)	0.88 (0.01, 30.50)	1.59 (0.02, 52.31)	2.24 (0.02, 70.09)	1.70 (0.02, 52.52)	2.77 (0.03, 82.15)	2.05 (0.02, 64.80)	2.10 (0.02, 73.93)	2.54 (0.02, 77.78)	1.31 (0.01, 38.94)	1.79 (0.02, 53.01)	1.56 (0.01, 43.87)	0.85 (0.01, 32.69)	0.92 (0.01, 33.29)	1.63 (0.01, 47.32)	1.30 (0.01, 46.91)	1.82 (0.01, 233.58)	1.43 (0.01, 177.55)	1.46 (0.01, 46.74)
CER200mgSC	1.56 (0.03, 48.88)	0.92 (0.01, 30.23)	4.05 (0.07, 133.27)	0.87 (0.01, 28.57)	0.83 (0.01, 28.01)	1.46 (0.02, 43.77)	1.98 (0.03, 68.65)	1.55 (0.03, 55.97)	2.51 (0.04, 86.10)	1.83 (0.03, 64.58)	1.94 (0.04, 66.42)	2.25 (0.04, 76.77)	1.17 (0.02, 34.90)	1.58 (0.03, 46.49)	1.40 (0.02, 42.66)	0.78 (0.01, 27.98)	0.82 (0.01, 28.66)	1.44 (0.03, 42.48)	1.24 (0.02, 43.33)	1.77 (0.01, 221.60)	1.41 (0.01, 197.56)	1.38 (0.02, 40.74)
CER20mgIV	2.01 (0.04, 61.25)	1.15 (0.02, 38.15)	5.09 (0.10, 153.95)	1.08 (0.02, 36.33)	1.04 (0.02, 36.65)	1.84 (0.03, 57.88)	2.54 (0.05, 74.47)	1.97 (0.04, 56.85)	3.18 (0.06, 90.60)	2.37 (0.04, 69.31)	2.37 (0.05, 83.26)	2.95 (0.06, 82.51)	1.50 (0.03, 43.67)	1.99 (0.04, 56.33)	1.77 (0.03, 50.03)	1.00 (0.02, 34.82)	1.07 (0.02, 30.60)	1.81 (0.04, 53.04)	1.55 (0.02, 58.05)	2.15 (0.02, 293.11)	1.74 (0.01, 194.26)	1.70 (0.03, 50.11)
CER400mgSC	1.17 (0.41, 3.16)	0.68 (0.22, 2.05)	**2.92 (1.08, 8.58)**	0.63 (0.16, 2.19)	0.60 (0.15, 2.16)	1.04 (0.32, 3.27)	1.46 (0.55, 3.87)	1.12 (0.41, 3.04)	1.86 (0.72, 4.67)	1.37 (0.53, 3.58)	1.42 (0.42, 4.90)	1.70 (0.66, 4.41)	0.86 (0.40, 1.78)	1.15 (0.50, 2.56)	1.01 (0.43, 2.34)	0.59 (0.13, 2.29)	0.61 (0.21, 1.75)	1.06 (0.50, 2.16)	0.86 (0.20, 3.72)	1.27 (0.04, 40.34)	0.97 (0.04, 29.82)	0.97 (0.39, 2.35)
CER5mgIV	1.18 (0.45, 3.13)	0.70 (0.24, 1.96)	**2.98 (1.14, 8.33)**	0.63 (0.17, 2.12)	0.61 (0.15, 2.09)	1.06 (0.35, 3.23)	1.48 (0.60, 3.78)	1.14 (0.44, 2.93)	1.87 (0.77, 4.59)	1.38 (0.56, 3.50)	1.44 (0.46, 4.91)	1.72 (0.71, 4.28)	0.88 (0.43, 1.66)	1.17 (0.54, 2.46)	1.04 (0.47, 2.20)	0.60 (0.14, 2.22)	0.62 (0.22, 1.68)	1.07 (0.54, 2.00)	0.87 (0.21, 3.75)	1.33 (0.04, 40.02)	0.98 (0.04, 29.83)	0.99 (0.44, 2.26)
ETA25mgSC	1.24 (0.41, 3.74)	0.73 (0.22, 2.39)	3.10 (1.00, 9.98)	0.64 (0.17, 2.59)	0.62 (0.15, 2.43)	1.10 (0.32, 3.76)	1.55 (0.53, 4.78)	1.19 (0.38, 3.65)	1.96 (0.69, 5.73)	1.45 (0.48, 4.35)	1.51 (0.44, 5.59)	1.77 (0.63, 5.32)	0.90 (0.37, 2.21)	1.21 (0.47, 3.14)	1.06 (0.41, 2.78)	0.61 (0.14, 2.65)	0.64 (0.19, 2.13)	1.10 (0.46, 2.63)	0.95 (0.17, 4.25)	1.36 (0.04, 48.18)	1.03 (0.04, 31.41)	1.04 (0.38, 2.80)
FON1mg/kg0.1mgIV/SC	1.27 (0.41, 3.72)	0.74 (0.22, 2.39)	3.16 (0.98, 10.31)	0.67 (0.17, 2.59)	0.64 (0.16, 2.52)	1.13 (0.33, 3.98)	1.57 (0.56, 4.75)	1.21 (0.41, 3.65)	1.99 (0.73, 5.71)	1.46 (0.52, 4.36)	1.54 (0.45, 5.80)	1.81 (0.68, 5.21)	0.93 (0.39, 2.18)	1.24 (0.49, 3.16)	1.10 (0.43, 2.81)	0.63 (0.14, 2.76)	0.66 (0.20, 2.10)	1.13 (0.48, 2.64)	0.95 (0.19, 4.43)	1.44 (0.04, 47.49)	1.06 (0.04, 32.78)	1.06 (0.40, 2.82)
FON1mg/kg1mgIV/SC	0.90 (0.32, 2.51)	0.52 (0.17, 1.63)	2.24 (0.77, 7.00)	0.47 (0.12, 1.73)	0.46 (0.11, 1.69)	0.80 (0.25, 2.55)	1.13 (0.42, 3.07)	0.87 (0.31, 2.46)	1.42 (0.54, 3.76)	1.05 (0.39, 2.85)	1.09 (0.32, 3.94)	1.30 (0.49, 3.48)	0.66 (0.29, 1.41)	0.87 (0.37, 2.04)	0.78 (0.32, 1.83)	0.45 (0.10, 1.73)	0.47 (0.15, 1.36)	0.80 (0.37, 1.71)	0.68 (0.14, 2.97)	1.00 (0.03, 33.69)	0.73 (0.03, 22.63)	0.75 (0.30, 1.88)
FON4mg/kg0.1mgIV/SC	1.10 (0.44, 2.66)	0.65 (0.23, 1.66)	**2.73 (1.14, 6.99)**	0.59 (0.17, 1.86)	0.56 (0.16, 1.84)	0.99 (0.34, 2.81)	1.37 (0.60, 3.32)	1.06 (0.43, 2.67)	1.73 (0.79, 3.99)	1.28 (0.54, 3.12)	1.34 (0.46, 4.32)	1.58 (0.72, 3.73)	0.81 (0.45, 1.40)	1.09 (0.54, 2.09)	0.96 (0.47, 1.88)	0.55 (0.13, 2.00)	0.57 (0.21, 1.45)	0.99 (0.56, 1.71)	0.83 (0.20, 3.24)	1.22 (0.04, 36.10)	0.92 (0.03, 27.09)	0.92 (0.45, 1.94)
FON4mg/kg1mgIV/SC	0.46 (0.08, 2.05)	0.27 (0.04, 1.28)	1.16 (0.20, 5.56)	0.24 (0.03, 1.38)	0.23 (0.04, 1.32)	0.40 (0.07, 2.05)	0.59 (0.10, 2.50)	0.45 (0.08, 1.94)	0.74 (0.13, 3.08)	0.54 (0.10, 2.24)	0.56 (0.09, 3.05)	0.67 (0.12, 2.77)	0.34 (0.07, 1.28)	0.46 (0.09, 1.81)	0.40 (0.08, 1.60)	0.23 (0.03, 1.34)	0.24 (0.04, 1.11)	0.42 (0.08, 1.57)	0.34 (0.04, 2.38)	0.49 (0.01, 19.51)	0.40 (0.01, 15.10)	0.38 (0.07, 1.66)
GUS1200mgIV	1.76 (0.60, 5.45)	1.03 (0.31, 3.46)	**4.40 (1.58, 13.97)**	0.93 (0.24, 3.45)	0.89 (0.21, 3.47)	1.59 (0.47, 5.32)	2.21 (0.79, 6.42)	1.70 (0.58, 5.14)	**2.78 (1.05, 7.92)**	2.06 (0.73, 5.99)	2.17 (0.60, 8.48)	2.55 (0.92, 7.32)	1.28 (0.57, 3.04)	1.73 (0.69, 4.38)	1.52 (0.60, 3.91)	0.88 (0.19, 3.71)	0.91 (0.30, 2.88)	1.57 (0.70, 3.65)	1.34 (0.28, 5.87)	1.97 (0.06, 62.88)	1.45 (0.05, 45.68)	1.49 (0.58, 3.91)
GUS200mgIV	1.44 (0.50, 4.23)	0.85 (0.27, 2.66)	**3.64 (1.28, 11.41)**	0.78 (0.19, 2.87)	0.75 (0.18, 2.72)	1.29 (0.40, 4.34)	1.79 (0.66, 5.30)	1.40 (0.48, 4.19)	2.28 (0.86, 6.33)	1.68 (0.58, 4.97)	1.77 (0.52, 6.60)	2.09 (0.76, 5.87)	1.05 (0.47, 2.43)	1.42 (0.58, 3.49)	1.26 (0.50, 3.15)	0.73 (0.16, 3.04)	0.76 (0.25, 2.32)	1.29 (0.60, 2.96)	1.08 (0.24, 4.88)	1.60 (0.05, 50.19)	1.18 (0.04, 36.93)	1.21 (0.48, 3.26)
GUS600mgIV	0.66 (0.24, 1.69)	0.38 (0.13, 1.10)	1.64 (0.61, 4.75)	0.35 (0.09, 1.19)	0.33 (0.08, 1.17)	0.59 (0.19, 1.80)	0.82 (0.33, 2.12)	0.63 (0.24, 1.65)	1.04 (0.42, 2.62)	0.75 (0.30, 1.98)	0.79 (0.25, 2.69)	0.94 (0.38, 2.34)	**0.48 (0.23, 0.94)**	0.65 (0.28, 1.40)	0.57 (0.25, 1.24)	0.32 (0.08, 1.23)	**0.34 (0.12, 0.96)**	0.59 (0.29, 1.13)	0.49 (0.11, 2.13)	0.72 (0.02, 22.81)	0.55 (0.02, 16.29)	0.55 (0.24, 1.26)
MED700IV	0.63 (0.23, 1.62)	0.37 (0.12, 1.11)	1.58 (0.60, 4.54)	0.34 (0.09, 1.15)	0.32 (0.08, 1.14)	0.57 (0.18, 1.72)	0.79 (0.31, 2.04)	0.61 (0.23, 1.66)	1.00 (0.40, 2.53)	0.73 (0.29, 1.93)	0.77 (0.24, 2.65)	0.91 (0.37, 2.33)	**0.46 (0.22, 0.91)**	0.62 (0.27, 1.34)	0.55 (0.23, 1.19)	0.32 (0.07, 1.20)	**0.32 (0.11, 0.94)**	0.57 (0.28, 1.08)	0.47 (0.11, 2.01)	0.70 (0.02, 21.29)	0.53 (0.02, 15.67)	0.53 (0.23, 1.22)
NAT300mgIV	1.24 (0.51, 2.80)	0.73 (0.27, 1.82)	**3.11 (1.34, 7.80)**	0.66 (0.19, 2.01)	0.63 (0.18, 1.93)	1.12 (0.40, 2.95)	1.54 (0.71, 3.60)	1.19 (0.51, 2.76)	1.95 (0.91, 4.30)	1.43 (0.65, 3.32)	1.51 (0.53, 4.66)	1.79 (0.86, 3.94)	0.92 (0.52, 1.47)	1.23 (0.64, 2.24)	1.09 (0.54, 1.95)	0.62 (0.16, 2.13)	0.65 (0.25, 1.58)	1.12 (0.67, 1.76)	0.93 (0.23, 3.53)	1.39 (0.05, 40.07)	1.04 (0.04, 28.09)	1.04 (0.53, 2.01)

	SEM60mgIV	SEM60mgIVx3	TES400mg200mgSC	TOF15mgOR_BID	TOF1mgOR_BID	TOF5mgOR_BID	UPA12mgOR_BID	UPA24mgOR	UPA24mgOR_BID	UPA3mgOR_BID	UPA45mgOR	UPA6mgOR_BID	UST130mgIV	UST1mg/kgIV	UST3mg/kgIV	UST4.5mg/kgIV	UST6mg/kg90mgIV/SC	UST6mg/kgIV	UST90mgSC	VED0_5mg/kgIV	VED2mg/kgIV	VED300mgIV
NAT3mg/kgIV	1.14 (0.47, 2.55)	0.67 (0.25, 1.64)	**2.83 (1.23, 6.96)**	0.61 (0.18, 1.82)	0.58 (0.17, 1.78)	1.03 (0.37, 2.69)	1.41 (0.66, 3.22)	1.10 (0.47, 2.55)	1.80 (0.85, 3.80)	1.32 (0.60, 2.96)	1.38 (0.49, 4.22)	1.64 (0.78, 3.61)	0.84 (0.48, 1.32)	1.12 (0.59, 2.00)	0.99 (0.50, 1.78)	0.57 (0.14, 1.93)	0.59 (0.22, 1.43)	1.02 (0.61, 1.53)	0.85 (0.20, 3.22)	1.26 (0.04, 35.96)	0.94 (0.03, 27.60)	0.96 (0.48, 1.82)
NAT3mg/kgIVx2	0.96 (0.02, 57.78)	0.57 (0.01, 33.48)	2.45 (0.06, 136.44)	0.51 (0.01, 32.61)	0.48 (0.01, 30.88)	0.87 (0.02, 53.27)	1.25 (0.03, 70.89)	0.98 (0.02, 51.66)	1.54 (0.04, 83.66)	1.14 (0.03, 65.34)	1.24 (0.03, 74.24)	1.39 (0.03, 77.60)	0.69 (0.02, 39.10)	0.97 (0.02, 52.74)	0.84 (0.02, 49.69)	0.49 (0.01, 36.22)	0.52 (0.01, 28.85)	0.88 (0.02, 49.13)	0.76 (0.02, 42.52)	1.19 (0.01, 306.48)	1.01 (0.01, 118.41)	0.81 (0.02, 47.43)
NAT6mg/kgIVx2	1.14 (0.02, 34.00)	0.69 (0.01, 20.34)	2.82 (0.05, 88.17)	0.63 (0.01, 19.80)	0.60 (0.01, 21.26)	1.00 (0.02, 35.58)	1.45 (0.02, 44.62)	1.11 (0.02, 34.19)	1.80 (0.03, 53.80)	1.33 (0.02, 41.66)	1.35 (0.02, 47.53)	1.66 (0.02, 49.46)	0.83 (0.01, 24.35)	1.14 (0.02, 30.31)	0.99 (0.02, 27.45)	0.59 (0.01, 17.78)	0.59 (0.01, 19.54)	1.01 (0.02, 29.06)	0.87 (0.01, 31.59)	1.26 (0.01, 252.40)	0.96 (0.01, 119.19)	0.97 (0.02, 28.27)
NNC2mg/kgSC	1.14 (0.02, 37.01)	0.65 (0.01, 22.62)	2.89 (0.05, 100.43)	0.61 (0.01, 22.88)	0.60 (0.01, 21.50)	1.04 (0.02, 36.71)	1.43 (0.03, 55.76)	1.09 (0.02, 41.90)	1.84 (0.03, 63.56)	1.34 (0.03, 51.24)	1.38 (0.03, 49.03)	1.69 (0.03, 59.69)	0.85 (0.02, 27.35)	1.14 (0.02, 36.07)	1.01 (0.02, 31.07)	0.59 (0.01, 23.21)	0.59 (0.01, 21.83)	1.03 (0.02, 33.66)	0.85 (0.02, 41.73)	1.15 (0.01, 151.83)	0.95 (0.01, 106.00)	1.01 (0.02, 32.19)
PBO	0.99 (0.02, 47.58)	0.59 (0.01, 28.55)	2.62 (0.04, 101.35)	0.57 (0.01, 25.30)	0.55 (0.01, 23.64)	0.97 (0.01, 41.67)	1.28 (0.02, 54.15)	0.98 (0.01, 45.68)	1.66 (0.03, 70.51)	1.17 (0.02, 53.76)	1.23 (0.02, 63.46)	1.49 (0.02, 64.64)	0.77 (0.01, 31.03)	1.04 (0.02, 40.78)	0.90 (0.01, 34.43)	0.53 (0.01, 23.27)	0.54 (0.01, 26.62)	0.94 (0.02, 38.16)	0.77 (0.01, 32.51)	1.07 (0.01, 198.79)	0.82 (0.01, 151.95)	0.88 (0.01, 38.53)
PF10mgSC	1.58 (0.54, 4.97)	0.94 (0.29, 3.18)	**3.93 (1.34, 13.70)**	0.85 (0.21, 3.64)	0.80 (0.20, 3.34)	1.43 (0.40, 5.29)	2.01 (0.71, 6.02)	1.55 (0.53, 4.84)	2.52 (0.91, 7.62)	1.86 (0.66, 5.69)	1.98 (0.55, 7.70)	2.30 (0.85, 7.00)	1.16 (0.51, 3.04)	1.56 (0.61, 4.20)	1.39 (0.54, 3.86)	0.81 (0.18, 3.53)	0.83 (0.32, 2.18)	1.41 (0.62, 3.60)	1.17 (0.26, 5.97)	1.79 (0.06, 59.45)	1.30 (0.05, 41.33)	1.33 (0.52, 4.11)
PF200mgSC	1.96 (0.63, 6.26)	1.17 (0.34, 3.89)	**5.03 (1.62, 16.49)**	1.07 (0.25, 4.22)	1.02 (0.24, 4.16)	1.78 (0.50, 6.52)	2.50 (0.86, 7.71)	1.91 (0.62, 6.16)	**3.16 (1.10, 9.57)**	2.29 (0.79, 7.15)	2.43 (0.64, 9.03)	**2.85 (1.03, 8.96)**	1.47 (0.58, 3.69)	1.97 (0.72, 5.49)	1.74 (0.63, 4.82)	1.01 (0.21, 4.19)	1.04 (0.40, 2.65)	1.80 (0.72, 4.40)	1.51 (0.31, 6.71)	2.21 (0.08, 68.33)	1.65 (0.05, 51.72)	1.66 (0.62, 4.84)
PF50mgSC	1.35 (0.47, 3.79)	0.79 (0.26, 2.51)	**3.40 (1.18, 10.79)**	0.73 (0.17, 2.67)	0.70 (0.17, 2.52)	1.23 (0.35, 4.01)	1.71 (0.61, 4.86)	1.31 (0.44, 3.70)	2.16 (0.80, 5.85)	1.59 (0.56, 4.35)	1.64 (0.48, 6.09)	1.97 (0.72, 5.35)	0.99 (0.43, 2.23)	1.33 (0.52, 3.29)	1.18 (0.46, 2.83)	0.68 (0.16, 2.75)	0.72 (0.28, 1.64)	1.22 (0.54, 2.67)	1.01 (0.23, 4.43)	1.50 (0.05, 46.93)	1.09 (0.04, 31.75)	1.12 (0.45, 2.95)
RIS200mgIV	3.18 (0.80, 14.81)	1.86 (0.42, 8.92)	**7.90 (2.08, 37.11)**	1.71 (0.34, 8.43)	1.62 (0.31, 8.38)	2.87 (0.69, 13.70)	**3.95 (1.07, 17.95)**	3.07 (0.81, 13.86)	**5.01 (1.38, 22.12)**	**3.63 (1.02, 16.63)**	3.80 (0.90, 21.57)	**4.57 (1.30, 20.00)**	2.29 (0.73, 8.79)	3.05 (0.93, 12.52)	2.74 (0.81, 11.52)	1.56 (0.29, 10.04)	1.64 (0.41, 7.45)	2.77 (0.92, 10.79)	2.38 (0.44, 14.91)	3.72 (0.10, 118.62)	2.71 (0.08, 97.38)	2.65 (0.77, 11.27)
RIS600mgIV	0.97 (0.41, 2.13)	0.57 (0.22, 1.38)	**2.41 (1.08, 5.87)**	0.52 (0.16, 1.53)	0.50 (0.14, 1.51)	0.88 (0.32, 2.24)	1.21 (0.58, 2.70)	0.93 (0.41, 2.14)	1.53 (0.75, 3.30)	1.12 (0.53, 2.50)	1.17 (0.43, 3.59)	1.40 (0.70, 3.01)	0.71 (0.46, 1.07)	0.96 (0.54, 1.66)	0.84 (0.46, 1.49)	0.49 (0.13, 1.63)	0.51 (0.20, 1.20)	0.87 (0.57, 1.29)	0.72 (0.18, 2.74)	1.07 (0.04, 31.02)	0.79 (0.03, 24.37)	0.81 (0.44, 1.55)
TES400mg200mgSC	1.46 (0.39, 5.21)	0.85 (0.21, 3.31)	3.64 (0.99, 13.63)	0.78 (0.16, 3.71)	0.73 (0.15, 3.68)	1.29 (0.31, 5.56)	1.82 (0.53, 6.27)	1.40 (0.38, 5.00)	2.27 (0.67, 7.64)	1.69 (0.49, 5.80)	1.75 (0.43, 8.04)	2.10 (0.61, 7.03)	1.05 (0.35, 3.21)	1.40 (0.45, 4.77)	1.25 (0.40, 4.09)	0.73 (0.14, 3.52)	0.74 (0.20, 2.90)	1.28 (0.44, 3.88)	1.07 (0.19, 5.99)	1.57 (0.05, 65.72)	1.13 (0.04, 49.44)	1.22 (0.37, 3.98)
UST130mgIV	1.21 (0.32, 4.04)	0.70 (0.18, 2.50)	3.05 (0.83, 10.31)	0.65 (0.14, 2.60)	0.62 (0.12, 2.53)	1.09 (0.27, 4.09)	1.49 (0.44, 5.29)	1.15 (0.32, 4.26)	1.89 (0.55, 6.28)	1.38 (0.40, 4.96)	1.45 (0.37, 6.16)	1.74 (0.50, 5.73)	0.88 (0.29, 2.52)	1.18 (0.37, 3.48)	1.04 (0.32, 3.18)	0.61 (0.12, 2.81)	0.62 (0.16, 2.19)	1.08 (0.36, 3.14)	0.89 (0.17, 5.07)	1.29 (0.04, 42.92)	1.00 (0.03, 31.20)	1.02 (0.31, 3.03)
UST1mg/kgIV	1.91 (0.46, 8.56)	1.10 (0.25, 5.31)	**4.73 (1.19, 22.81)**	0.99 (0.19, 5.75)	0.96 (0.19, 5.10)	1.70 (0.37, 8.65)	2.41 (0.61, 10.49)	1.88 (0.44, 8.41)	3.07 (0.79, 13.41)	2.26 (0.55, 9.64)	2.30 (0.52, 11.88)	2.79 (0.72, 12.17)	1.37 (0.41, 5.45)	1.86 (0.51, 7.95)	1.64 (0.46, 6.85)	0.94 (0.17, 5.36)	1.01 (0.24, 4.74)	1.69 (0.50, 6.57)	1.43 (0.24, 9.25)	2.18 (0.06, 82.11)	1.61 (0.04, 58.34)	1.58 (0.43, 6.88)
UST3mg/kgIV	2.40 (0.73, 8.36)	1.42 (0.40, 5.30)	**6.04 (1.87, 22.48)**	1.27 (0.30, 5.73)	1.23 (0.28, 5.63)	2.14 (0.59, 8.60)	3.01 (0.99, 10.75)	2.35 (0.71, 8.21)	**3.77 (1.27, 13.44)**	2.77 (0.90, 9.93)	2.94 (0.78, 12.75)	**3.46 (1.15, 11.93)**	1.75 (0.68, 5.19)	2.37 (0.86, 7.34)	2.08 (0.75, 6.63)	1.21 (0.25, 5.73)	1.25 (0.37, 4.67)	2.15 (0.85, 6.22)	1.83 (0.36, 9.87)	2.72 (0.08, 88.85)	2.02 (0.06, 67.17)	2.03 (0.71, 6.74)
UST4.5mg/kgIV	1.11 (0.50, 2.32)	0.65 (0.26, 1.53)	**2.75 (1.32, 6.47)**	0.59 (0.19, 1.70)	0.57 (0.17, 1.65)	1.00 (0.38, 2.47)	1.38 (0.70, 2.97)	1.07 (0.50, 2.32)	1.75 (0.92, 3.59)	1.28 (0.64, 2.73)	1.35 (0.52, 3.98)	1.60 (0.85, 3.26)	0.82 (0.58, 1.12)	1.10 (0.66, 1.77)	0.97 (0.56, 1.57)	0.56 (0.15, 1.79)	0.58 (0.24, 1.31)	1.00 (0.74, 1.33)	0.83 (0.21, 2.99)	1.23 (0.04, 35.48)	0.92 (0.03, 25.84)	0.92 (0.54, 1.66)
UST6mg/kg90mgIV/SC	0.47 (0.01, 5.62)	0.26 (0.00, 3.37)	1.20 (0.02, 14.61)	0.25 (0.00, 3.17)	0.24 (0.00, 3.09)	0.41 (0.01, 5.22)	0.59 (0.01, 6.71)	0.45 (0.01, 5.05)	0.75 (0.01, 7.90)	0.55 (0.01, 6.20)	0.58 (0.01, 8.03)	0.69 (0.01, 7.41)	0.35 (0.01, 3.57)	0.47 (0.01, 5.04)	0.42 (0.01, 4.57)	0.23 (0.00, 3.58)	0.24 (0.00, 2.87)	0.43 (0.01, 4.40)	0.33 (0.00, 5.58)	0.39 (0.00, 22.32)	0.30 (0.00, 22.76)	0.40 (0.01, 4.27)
UST6mg/kgIV	0.28 (0.00, 3.41)	0.16 (0.00, 2.18)	0.72 (0.01, 8.83)	0.14 (0.00, 2.00)	0.14 (0.00, 2.05)	0.25 (0.00, 3.34)	0.36 (0.01, 4.22)	0.27 (0.00, 3.21)	0.46 (0.01, 5.07)	0.33 (0.01, 4.01)	0.34 (0.01, 5.16)	0.42 (0.01, 4.80)	0.21 (0.00, 2.37)	0.28 (0.00, 3.20)	0.25 (0.00, 2.81)	0.14 (0.00, 2.17)	0.14 (0.00, 1.90)	0.26 (0.00, 2.92)	0.20 (0.00, 3.54)	0.24 (0.00, 14.79)	0.18 (0.00, 12.28)	0.24 (0.00, 2.82)
UST90mgSC	0.51 (0.01, 6.01)	0.30 (0.01, 3.64)	1.25 (0.03, 16.93)	0.26 (0.00, 3.80)	0.25 (0.00, 3.54)	0.44 (0.01, 6.42)	0.64 (0.01, 7.64)	0.48 (0.01, 5.93)	0.79 (0.01, 10.12)	0.58 (0.01, 7.36)	0.58 (0.01, 8.31)	0.72 (0.01, 8.55)	0.36 (0.01, 4.21)	0.48 (0.01, 5.85)	0.43 (0.01, 4.91)	0.24 (0.00, 3.76)	0.26 (0.01, 3.33)	0.44 (0.01, 5.14)	0.35 (0.01, 6.78)	0.43 (0.01, 33.35)	0.35 (0.00, 28.71)	0.41 (0.01, 5.06)
VED0_5mg/kgIV	**5.97 (1.82, 22.20)**	3.55 (0.97, 13.49)	**15.03 (4.76, 58.35)**	3.24 (0.76, 13.78)	3.04 (0.70, 13.56)	**5.50 (1.40, 20.17)**	**7.59 (2.50, 27.05)**	**5.85 (1.79, 21.95)**	**9.46 (3.16, 34.17)**	**7.07 (2.25, 25.59)**	**7.39 (1.97, 33.48)**	**8.70 (2.91, 30.69)**	**4.35 (1.73, 13.81)**	**5.83 (2.14, 19.41)**	**5.13 (1.88, 17.16)**	3.03 (0.59, 14.32)	3.13 (0.91, 11.65)	**5.35 (2.15, 16.62)**	4.48 (0.92, 23.61)	6.71 (0.21, 210.31)	4.79 (0.18, 152.20)	**5.02 (1.78, 16.24)**
VED2mg/kgIV	4.76 (0.60, 148.98)	2.89 (0.33, 90.73)	**12.23 (1.36, 415.84)**	2.56 (0.26, 89.95)	2.55 (0.26, 69.51)	4.36 (0.48, 127.14)	6.05 (0.75, 186.63)	4.71 (0.53, 136.71)	7.56 (0.97, 234.06)	5.58 (0.64, 161.87)	6.12 (0.62, 165.63)	6.95 (0.83, 202.06)	3.45 (0.47, 106.53)	4.62 (0.61, 140.48)	4.11 (0.51, 132.92)	2.43 (0.22, 89.67)	2.49 (0.27, 79.23)	4.21 (0.59, 129.94)	3.72 (0.33, 133.92)	5.69 (0.10, 788.92)	4.35 (0.10, 554.41)	4.02 (0.51, 129.44)
VED300mgIV	**4.34 (1.52, 13.55)**	2.56 (0.78, 9.44)	**10.91 (3.75, 36.84)**	2.41 (0.58, 9.46)	2.28 (0.55, 8.96)	**3.94 (1.17, 13.85)**	**5.45 (1.98, 16.83)**	**4.20 (1.48, 13.50)**	**6.85 (2.61, 21.50)**	**5.05 (1.82, 16.22)**	**5.29 (1.57, 20.97)**	**6.25 (2.36, 19.65)**	**3.21 (1.37, 8.10)**	**4.30 (1.75, 11.37)**	**3.76 (1.52, 10.47)**	2.25 (0.44, 9.51)	2.27 (0.67, 7.40)	**3.93 (1.74, 9.76)**	3.34 (0.63, 16.80)	4.98 (0.16, 153.35)	3.61 (0.12, 121.47)	**3.64 (1.43, 10.53)**
SEC10mg/kgIV	**0.09 (0.01, 0.59)**	**0.05 (0.00, 0.36)**	0.22 (0.01, 1.54)	**0.05 (0.00, 0.37)**	**0.05 (0.00, 0.36)**	**0.08 (0.00, 0.58)**	**0.12 (0.01, 0.71)**	**0.09 (0.00, 0.56)**	**0.15 (0.01, 0.87)**	**0.11 (0.01, 0.65)**	**0.11 (0.01, 0.83)**	**0.13 (0.01, 0.80)**	**0.07 (0.00, 0.37)**	**0.09 (0.01, 0.52)**	**0.08 (0.00, 0.46)**	**0.04 (0.00, 0.38)**	**0.05 (0.00, 0.31)**	**0.08 (0.00, 0.45)**	**0.07 (0.00, 0.57)**	0.09 (0.00, 5.40)	0.07 (0.00, 3.30)	**0.08 (0.00, 0.43)**

(TABLE S12I. continued)

	SEM60mgIV	SEM60mgIVx3	TES400mg200mgSC	TOF15mgOR_BID	TOF1mgOR_BID	TOF5mgOR_BID	UPA12mgOR_BID	UPA24mgOR	UPA24mgOR_BID	UPA3mgOR_BID	UPA45mgOR	UPA6mgOR_BID	UST130mgIV	UST1mg/kgIV	UST3mg/kgIV	UST4.5mg/kgIV	UST6mg/kg90mgIV/SC	UST6mg/kgIV	UST90mgSC	VED0_5mg/kgIV	VED2mg/kgIV	VED300mgIV
SEM60mgIV	SEM60mgIV	0.59 (0.24, 1.42)	2.48 (0.87, 8.22)	0.53 (0.13, 2.01)	0.51 (0.12, 2.04)	0.89 (0.27, 3.16)	1.24 (0.46, 3.68)	0.97 (0.33, 2.93)	1.57 (0.59, 4.51)	1.16 (0.42, 3.44)	1.21 (0.35, 4.44)	1.44 (0.54, 4.15)	0.73 (0.32, 1.72)	0.99 (0.40, 2.48)	0.87 (0.35, 2.22)	0.51 (0.11, 1.99)	0.52 (0.17, 1.59)	0.89 (0.41, 2.07)	0.75 (0.16, 3.36)	1.15 (0.04, 33.98)	0.82 (0.03, 24.89)	0.84 (0.33, 2.23)
SEM60mgIVx3	1.69 (0.70, 4.20)	SEM60mgIVx3	**4.22 (1.36, 15.30)**	0.92 (0.22, 3.60)	0.87 (0.20, 3.46)	1.53 (0.43, 5.52)	2.13 (0.73, 6.96)	1.65 (0.52, 5.36)	2.67 (0.95, 8.44)	1.97 (0.66, 6.39)	2.11 (0.57, 8.35)	2.46 (0.86, 7.61)	1.25 (0.49, 3.27)	1.67 (0.62, 4.70)	1.48 (0.54, 4.13)	0.87 (0.19, 3.78)	0.87 (0.28, 2.91)	1.52 (0.63, 3.97)	1.26 (0.25, 6.24)	1.96 (0.06, 64.11)	1.38 (0.05, 48.20)	1.42 (0.52, 4.14)
TES400mg200mgSC	0.40 (0.12, 1.16)	**0.24 (0.07, 0.73)**	TES400mg200mgSC	**0.21 (0.05, 0.81)**	**0.20 (0.05, 0.75)**	0.36 (0.10, 1.21)	0.50 (0.17, 1.49)	0.38 (0.12, 1.17)	0.63 (0.22, 1.81)	0.46 (0.15, 1.40)	0.48 (0.14, 1.82)	0.58 (0.20, 1.61)	**0.30 (0.12, 0.66)**	**0.40 (0.15, 0.96)**	**0.35 (0.13, 0.86)**	**0.20 (0.04, 0.80)**	**0.21 (0.06, 0.64)**	**0.36 (0.15, 0.82)**	0.30 (0.06, 1.34)	0.44 (0.01, 13.18)	0.33 (0.01, 9.91)	**0.34 (0.12, 0.87)**
TOF15mgOR_BID	1.87 (0.50, 7.47)	1.09 (0.28, 4.64)	**4.67 (1.23, 20.07)**	TOF15mgOR_BID	0.95 (0.27, 3.24)	1.64 (0.57, 5.51)	2.32 (0.66, 9.08)	1.79 (0.49, 7.07)	2.96 (0.85, 11.31)	2.16 (0.62, 8.84)	2.27 (0.53, 10.73)	2.70 (0.79, 10.72)	1.37 (0.46, 4.58)	1.85 (0.57, 6.41)	1.63 (0.51, 5.68)	0.93 (0.18, 4.75)	0.97 (0.25, 4.27)	1.68 (0.56, 5.52)	1.41 (0.24, 7.92)	2.05 (0.07, 78.94)	1.57 (0.05, 51.45)	1.57 (0.49, 5.62)
TOF1mgOR_BID	1.95 (0.49, 8.09)	1.15 (0.29, 4.99)	**4.89 (1.33, 20.39)**	1.05 (0.31, 3.71)	TOF1mgOR_BID	1.72 (0.59, 5.62)	2.48 (0.65, 9.82)	1.90 (0.50, 7.93)	3.10 (0.88, 11.98)	2.28 (0.62, 9.19)	2.40 (0.59, 11.45)	2.83 (0.79, 11.64)	1.44 (0.47, 5.02)	1.93 (0.59, 7.11)	1.71 (0.51, 6.08)	0.98 (0.19, 5.31)	1.03 (0.27, 4.32)	1.76 (0.58, 6.04)	1.49 (0.26, 8.21)	2.15 (0.06, 75.74)	1.60 (0.05, 52.75)	1.64 (0.49, 6.00)
TOF5mgOR_BID	1.13 (0.32, 3.72)	0.65 (0.18, 2.33)	2.79 (0.82, 10.30)	0.61 (0.18, 1.74)	0.58 (0.18, 1.69)	TOF5mgOR_BID	1.40 (0.44, 4.69)	1.07 (0.32, 3.71)	1.77 (0.56, 5.60)	1.29 (0.41, 4.25)	1.35 (0.36, 5.51)	1.62 (0.50, 5.25)	0.81 (0.31, 2.26)	1.09 (0.38, 3.22)	0.96 (0.34, 2.82)	0.56 (0.11, 2.57)	0.58 (0.17, 2.04)	0.99 (0.39, 2.69)	0.85 (0.16, 4.14)	1.23 (0.04, 39.84)	0.90 (0.03, 29.14)	0.94 (0.31, 2.85)
UPA12mgOR_BID	0.81 (0.27, 2.16)	0.47 (0.14, 1.36)	2.00 (0.67, 5.90)	0.43 (0.11, 1.52)	0.40 (0.10, 1.55)	0.71 (0.21, 2.30)	UPA12mgOR_BID	0.77 (0.38, 1.55)	1.25 (0.70, 2.34)	0.92 (0.49, 1.77)	0.96 (0.29, 3.49)	1.14 (0.64, 2.12)	0.59 (0.26, 1.26)	0.79 (0.32, 1.83)	0.69 (0.28, 1.64)	0.40 (0.09, 1.60)	0.42 (0.14, 1.17)	0.72 (0.32, 1.51)	0.60 (0.13, 2.55)	0.89 (0.03, 27.17)	0.65 (0.02, 20.81)	0.68 (0.26, 1.60)
UPA24mgOR	1.03 (0.34, 3.01)	0.60 (0.19, 1.91)	2.60 (0.86, 8.26)	0.56 (0.14, 2.04)	0.53 (0.13, 2.02)	0.94 (0.27, 3.11)	1.30 (0.64, 2.62)	UPA24mgOR	1.62 (0.87, 3.25)	1.20 (0.61, 2.46)	1.25 (0.36, 4.65)	1.48 (0.79, 3.01)	0.76 (0.32, 1.73)	1.03 (0.40, 2.47)	0.89 (0.36, 2.26)	0.52 (0.11, 2.06)	0.54 (0.17, 1.56)	0.93 (0.40, 2.11)	0.78 (0.17, 3.46)	1.18 (0.04, 36.89)	0.85 (0.03, 25.52)	0.87 (0.34, 2.23)
UPA24mgOR_BID	0.64 (0.22, 1.68)	0.37 (0.12, 1.06)	1.58 (0.55, 4.64)	0.34 (0.09, 1.18)	0.32 (0.08, 1.13)	0.56 (0.18, 1.78)	0.80 (0.43, 1.43)	0.62 (0.31, 1.15)	UPA24mgOR_BID	0.74 (0.40, 1.32)	0.76 (0.24, 2.69)	0.92 (0.52, 1.59)	**0.46 (0.21, 0.96)**	0.62 (0.26, 1.42)	0.55 (0.24, 1.25)	0.32 (0.07, 1.21)	**0.33 (0.11, 0.92)**	0.57 (0.27, 1.13)	0.47 (0.11, 1.97)	0.70 (0.02, 20.59)	0.52 (0.02, 15.02)	0.53 (0.21, 1.28)
UPA3mgOR_BID	0.86 (0.29, 2.39)	0.51 (0.16, 1.52)	2.16 (0.71, 6.82)	0.46 (0.11, 1.60)	0.44 (0.11, 1.61)	0.77 (0.24, 2.47)	1.08 (0.57, 2.03)	0.83 (0.41, 1.63)	1.36 (0.76, 2.49)	UPA3mgOR_BID	1.04 (0.32, 3.92)	1.23 (0.68, 2.32)	0.64 (0.27, 1.37)	0.85 (0.34, 2.03)	0.74 (0.30, 1.78)	0.43 (0.10, 1.73)	0.45 (0.14, 1.25)	0.77 (0.34, 1.63)	0.64 (0.14, 2.71)	0.95 (0.03, 30.04)	0.71 (0.03, 20.65)	0.73 (0.29, 1.82)
UPA45mgOR	0.83 (0.23, 2.84)	0.47 (0.12, 1.75)	2.07 (0.55, 7.28)	0.44 (0.09, 1.87)	0.42 (0.09, 1.70)	0.74 (0.18, 2.77)	1.04 (0.29, 3.43)	0.80 (0.22, 2.74)	1.31 (0.37, 4.24)	0.96 (0.25, 3.12)	UPA45mgOR	1.19 (0.34, 3.78)	0.61 (0.20, 1.65)	0.81 (0.25, 2.34)	0.72 (0.21, 2.05)	0.41 (0.07, 1.89)	0.42 (0.11, 1.48)	0.74 (0.24, 1.95)	0.63 (0.11, 3.18)	0.91 (0.03, 30.40)	0.66 (0.02, 19.97)	0.69 (0.21, 2.18)
UPA6mgOR_BID	0.69 (0.24, 1.86)	0.41 (0.13, 1.17)	1.74 (0.62, 5.08)	0.37 (0.09, 1.27)	0.35 (0.09, 1.26)	0.62 (0.19, 1.98)	0.87 (0.47, 1.56)	0.68 (0.33, 1.27)	1.09 (0.63, 1.91)	0.81 (0.43, 1.47)	0.84 (0.26, 2.95)	UPA6mgOR_BID	0.51 (0.23, 1.02)	0.69 (0.28, 1.53)	0.60 (0.25, 1.38)	0.35 (0.08, 1.34)	**0.37 (0.11, 0.99)**	0.62 (0.29, 1.25)	0.52 (0.12, 2.16)	0.77 (0.02, 24.10)	0.57 (0.02, 17.15)	0.58 (0.23, 1.39)
UST130mgIV	1.37 (0.58, 3.10)	0.80 (0.31, 2.02)	**3.38 (1.51, 8.51)**	0.73 (0.22, 2.18)	0.69 (0.20, 2.13)	1.23 (0.44, 3.26)	1.70 (0.79, 3.89)	1.32 (0.58, 3.10)	**2.15 (1.05, 4.72)**	1.57 (0.73, 3.65)	1.65 (0.61, 5.09)	1.96 (0.98, 4.39)	UST130mgIV	1.34 (0.77, 2.36)	1.18 (0.65, 2.09)	0.69 (0.17, 2.38)	0.71 (0.29, 1.71)	1.22 (0.88, 1.72)	1.03 (0.25, 3.92)	1.52 (0.05, 43.46)	1.15 (0.04, 32.97)	1.14 (0.60, 2.25)
UST1mg/kgIV	1.01 (0.40, 2.51)	0.60 (0.21, 1.61)	**2.53 (1.05, 6.69)**	0.54 (0.16, 1.75)	0.52 (0.14, 1.71)	0.92 (0.31, 2.61)	1.27 (0.55, 3.11)	0.97 (0.40, 2.47)	1.60 (0.70, 3.84)	1.17 (0.49, 2.91)	1.24 (0.43, 4.01)	1.46 (0.66, 3.54)	0.75 (0.42, 1.30)	UST1mg/kgIV	0.88 (0.50, 1.54)	0.51 (0.12, 1.82)	0.53 (0.20, 1.38)	0.91 (0.56, 1.51)	0.77 (0.18, 3.11)	1.13 (0.04, 34.47)	0.84 (0.03, 24.22)	0.85 (0.41, 1.81)
UST3mg/kgIV	1.15 (0.45, 2.87)	0.67 (0.24, 1.86)	**2.89 (1.17, 7.82)**	0.61 (0.18, 1.97)	0.58 (0.16, 1.95)	1.05 (0.35, 2.97)	1.44 (0.61, 3.62)	1.12 (0.44, 2.79)	1.83 (0.80, 4.23)	1.35 (0.56, 3.31)	1.39 (0.49, 4.70)	1.67 (0.72, 3.94)	0.85 (0.48, 1.53)	1.13 (0.65, 1.99)	UST3mg/kgIV	0.57 (0.14, 2.12)	0.60 (0.22, 1.59)	1.03 (0.63, 1.76)	0.87 (0.20, 3.55)	1.28 (0.04, 39.23)	0.95 (0.03, 29.43)	0.97 (0.46, 2.08)
UST4.5mg/kgIV	1.96 (0.50, 8.94)	1.15 (0.26, 5.37)	**5.05 (1.24, 22.98)**	1.07 (0.21, 5.54)	1.02 (0.19, 5.20)	1.78 (0.39, 8.79)	2.50 (0.62, 11.30)	1.92 (0.48, 8.90)	3.13 (0.83, 14.53)	2.31 (0.58, 10.20)	2.42 (0.53, 13.46)	2.87 (0.75, 12.98)	1.45 (0.42, 5.83)	1.96 (0.55, 8.25)	1.74 (0.47, 7.00)	UST4.5mg/kgIV	1.05 (0.24, 4.58)	1.79 (0.52, 6.99)	1.53 (0.23, 9.29)	2.25 (0.06, 94.47)	1.71 (0.05, 52.06)	1.64 (0.46, 7.25)
UST6mg/kg90mgIV/SC	1.91 (0.63, 5.88)	1.15 (0.34, 3.59)	**4.83 (1.57, 15.70)**	1.03 (0.23, 3.97)	0.97 (0.23, 3.73)	1.72 (0.49, 6.00)	2.38 (0.86, 7.37)	1.84 (0.64, 5.88)	**3.02 (1.09, 9.31)**	2.20 (0.80, 7.03)	2.35 (0.67, 9.26)	**2.73 (1.01, 8.71)**	1.41 (0.58, 3.47)	1.88 (0.73, 4.93)	1.65 (0.63, 4.45)	0.95 (0.22, 4.23)	UST6mg/kg90mgIV/SC	1.73 (0.72, 4.22)	1.42 (0.31, 6.90)	2.11 (0.07, 73.75)	1.59 (0.05, 47.12)	1.61 (0.59, 4.72)
UST6mg/kgIV	1.12 (0.48, 2.46)	0.66 (0.25, 1.60)	**2.79 (1.21, 6.82)**	0.60 (0.18, 1.78)	0.57 (0.17, 1.72)	1.01 (0.37, 2.57)	1.39 (0.66, 3.15)	1.08 (0.47, 2.49)	1.76 (0.88, 3.76)	1.29 (0.61, 2.91)	1.35 (0.51, 4.14)	1.61 (0.80, 3.49)	0.82 (0.58, 1.13)	1.10 (0.66, 1.79)	0.97 (0.57, 1.58)	0.56 (0.14, 1.91)	0.58 (0.24, 1.38)	UST6mg/kgIV	0.84 (0.21, 3.16)	1.24 (0.04, 34.78)	0.94 (0.03, 27.97)	0.93 (0.50, 1.81)
UST90mgSC	1.33 (0.30, 6.38)	0.79 (0.16, 3.94)	3.35 (0.75, 17.50)	0.71 (0.13, 4.10)	0.67 (0.12, 3.90)	1.18 (0.24, 6.29)	1.67 (0.39, 7.76)	1.29 (0.29, 5.96)	2.11 (0.51, 9.18)	1.56 (0.37, 6.91)	1.59 (0.31, 9.42)	1.93 (0.46, 8.52)	0.97 (0.26, 3.93)	1.29 (0.32, 5.64)	1.15 (0.28, 5.11)	0.65 (0.11, 4.36)	0.70 (0.14, 3.27)	1.19 (0.32, 4.78)	UST90mgSC	1.50 (0.04, 49.08)	1.07 (0.03, 37.73)	1.14 (0.27, 5.14)
VED0_5mg/kgIV	0.87 (0.03, 26.67)	0.51 (0.02, 17.32)	2.25 (0.08, 79.50)	0.49 (0.01, 14.65)	0.46 (0.01, 17.46)	0.81 (0.03, 28.23)	1.13 (0.04, 35.01)	0.85 (0.03, 28.44)	1.42 (0.05, 44.48)	1.05 (0.03, 32.47)	1.10 (0.03, 36.73)	1.30 (0.04, 40.48)	0.66 (0.02, 19.15)	0.89 (0.03, 26.96)	0.78 (0.03, 22.87)	0.44 (0.01, 16.03)	0.47 (0.01, 15.08)	0.81 (0.03, 22.84)	0.67 (0.02, 24.69)	VED0_5mg/kgIV	0.73 (0.03, 23.64)	0.74 (0.03, 22.30)
VED2mg/kgIV	1.23 (0.04, 33.63)	0.73 (0.02, 21.22)	3.02 (0.10, 99.82)	0.64 (0.02, 20.49)	0.63 (0.02, 19.69)	1.12 (0.03, 33.95)	1.54 (0.05, 44.97)	1.18 (0.04, 32.41)	1.93 (0.07, 52.56)	1.41 (0.05, 38.58)	1.51 (0.05, 44.11)	1.76 (0.06, 50.59)	0.87 (0.03, 24.70)	1.19 (0.04, 33.14)	1.05 (0.03, 29.25)	0.59 (0.02, 18.74)	0.63 (0.02, 18.34)	1.06 (0.04, 29.19)	0.94 (0.03, 29.53)	1.37 (0.04, 37.22)	VED2mg/kgIV	1.01 (0.03, 27.76)
VED300mgIV	1.20 (0.45, 3.07)	0.70 (0.24, 1.93)	**2.97 (1.15, 8.19)**	0.64 (0.18, 2.05)	0.61 (0.17, 2.03)	1.07 (0.35, 3.21)	1.47 (0.62, 3.84)	1.15 (0.45, 2.97)	1.88 (0.78, 4.66)	1.37 (0.55, 3.51)	1.45 (0.46, 4.87)	1.72 (0.72, 4.31)	0.88 (0.44, 1.66)	1.17 (0.55, 2.43)	1.03 (0.48, 2.17)	0.61 (0.14, 2.16)	0.62 (0.21, 1.69)	1.08 (0.55, 1.99)	0.88 (0.19, 3.67)	1.34 (0.04, 37.28)	0.99 (0.04, 28.97)	VED300mgIV

Table S12J: League table for clinical responses at 6 weeks of treatment.

	ABA10mg/kgIV	ABA30mg/kgIV	ABA3mg/kgIV	AMI0.4mgOR	BRI400mgIV	BRI700mgIV	CDP10mg/kgIV	CER100mgSC	CER10mgIV	CER200mgSC	CER20mgIV	CER400mgSC	CER5mgIV	ELD10mg/kgIV	ELD20mg/kgIV	FON1mg/kg0.1mgIV/SC	FON1mg/kg1mgIV/SC	FON4mg/kg0.1mgIV/SC	FON4mg/kg1mgIV/SC	GUS1200mgIV
ABA10mg/kgIV	ABA10mg/kgIV	1.82 (0.48, 7.75)	1.15 (0.37, 3.85)	1.15 (0.28, 4.23)	**4.70 (1.02, 30.87)**	3.73 (0.85, 24.15)	1.68 (0.50, 6.18)	1.83 (0.47, 8.05)	1.19 (0.21, 5.92)	1.31 (0.31, 6.08)	0.67 (0.10, 3.40)	1.89 (0.48, 8.81)	1.68 (0.38, 8.66)	1.91 (0.45, 9.49)	2.49 (0.60, 11.67)	1.90 (0.46, 10.53)	2.38 (0.58, 9.79)	2.89 (0.78, 11.41)	2.40 (0.62, 12.19)	**4.81 (1.27, 18.42)**
ABA30mg/kgIV	0.55 (0.13, 2.06)	ABA30mg/kgIV	0.62 (0.19, 2.56)	0.60 (0.16, 2.54)	2.58 (0.62, 12.52)	2.08 (0.51, 9.15)	0.89 (0.30, 3.59)	0.98 (0.26, 3.82)	0.63 (0.11, 2.98)	0.70 (0.19, 2.68)	0.35 (0.06, 2.10)	1.01 (0.28, 4.16)	0.92 (0.23, 4.21)	1.05 (0.27, 4.41)	1.33 (0.35, 5.98)	1.04 (0.26, 4.66)	1.27 (0.34, 5.40)	1.57 (0.44, 6.57)	1.34 (0.35, 5.76)	2.55 (0.73, 11.04)
ABA3mg/kgIV	0.87 (0.26, 2.73)	1.60 (0.39, 5.30)	ABA3mg/kgIV	0.97 (0.26, 3.74)	3.95 (0.97, 18.64)	3.17 (0.81, 14.60)	1.45 (0.48, 4.80)	1.56 (0.45, 5.43)	1.03 (0.19, 4.58)	1.12 (0.32, 4.09)	0.56 (0.09, 3.01)	1.62 (0.46, 5.57)	1.40 (0.36, 6.15)	1.63 (0.43, 6.31)	2.17 (0.58, 8.24)	1.64 (0.41, 6.44)	2.02 (0.55, 7.84)	2.49 (0.69, 9.41)	2.10 (0.55, 7.76)	**4.06 (1.18, 15.42)**
AMI0.4mgPO	0.87 (0.24, 3.56)	1.67 (0.39, 6.09)	1.03 (0.27, 3.86)	AMI0.4mgPO	**4.22 (1.27, 14.45)**	**3.30 (1.06, 12.01)**	1.49 (0.68, 3.53)	1.58 (0.66, 4.13)	1.04 (0.23, 3.72)	1.15 (0.44, 3.07)	0.59 (0.11, 2.35)	1.65 (0.68, 4.20)	1.47 (0.50, 4.60)	1.69 (0.54, 5.17)	2.21 (0.78, 6.74)	1.68 (0.58, 5.22)	2.06 (0.76, 6.07)	2.55 (0.99, 7.62)	2.13 (0.75, 6.25)	**4.23 (1.65, 10.88)**
BRI400mgIV	**0.21 (0.03, 0.98)**	0.39 (0.08, 1.61)	0.25 (0.05, 1.03)	**0.24 (0.07, 0.79)**	BRI400mgIV	0.80 (0.45, 1.49)	**0.36 (0.12, 0.99)**	0.38 (0.11, 1.17)	**0.25 (0.05, 0.96)**	**0.27 (0.08, 0.87)**	**0.14 (0.02, 0.60)**	0.39 (0.12, 1.20)	0.35 (0.09, 1.21)	0.40 (0.10, 1.44)	0.53 (0.15, 1.81)	0.39 (0.11, 1.47)	0.50 (0.14, 1.79)	0.61 (0.18, 2.21)	0.51 (0.14, 1.89)	1.00 (0.30, 3.08)
BRI700mgIV	0.27 (0.04, 1.18)	0.48 (0.11, 1.97)	0.32 (0.07, 1.24)	**0.30 (0.08, 0.94)**	1.26 (0.67, 2.23)	BRI700mgIV	0.46 (0.15, 1.13)	0.48 (0.15, 1.32)	0.31 (0.06, 1.17)	0.35 (0.10, 1.02)	**0.18 (0.03, 0.72)**	0.50 (0.15, 1.40)	0.44 (0.11, 1.47)	0.51 (0.13, 1.75)	0.67 (0.18, 2.18)	0.50 (0.14, 1.76)	0.63 (0.18, 2.14)	0.78 (0.23, 2.55)	0.65 (0.18, 2.15)	1.30 (0.38, 3.75)
CDP10mg/kgIV	0.60 (0.16, 2.00)	1.13 (0.28, 3.36)	0.69 (0.21, 2.08)	0.67 (0.28, 1.46)	**2.81 (1.01, 8.33)**	2.17 (0.89, 6.54)	CDP10mg/kgIV	1.07 (0.53, 2.13)	0.70 (0.18, 2.08)	0.78 (0.35, 1.60)	0.40 (0.09, 1.33)	1.11 (0.55, 2.20)	1.00 (0.37, 2.56)	1.13 (0.42, 2.94)	1.49 (0.59, 3.80)	1.14 (0.45, 2.80)	1.39 (0.60, 3.22)	1.70 (0.79, 4.12)	1.45 (0.57, 3.52)	**2.77 (1.35, 6.25)**
CER100mgSC	0.55 (0.12, 2.12)	1.02 (0.26, 3.82)	0.64 (0.18, 2.22)	0.63 (0.24, 1.52)	2.60 (0.86, 8.81)	2.06 (0.76, 6.77)	0.93 (0.47, 1.87)	CER100mgSC	0.66 (0.16, 2.23)	0.72 (0.37, 1.39)	0.37 (0.08, 1.34)	1.03 (0.59, 1.85)	0.93 (0.31, 2.71)	1.04 (0.36, 3.17)	1.37 (0.51, 3.90)	1.05 (0.40, 2.95)	1.30 (0.50, 3.59)	1.58 (0.66, 4.20)	1.34 (0.48, 3.58)	**2.64 (1.13, 6.83)**
CER10mgIV	0.84 (0.17, 4.80)	1.58 (0.34, 8.96)	0.97 (0.22, 5.40)	0.96 (0.27, 4.35)	**4.05 (1.05, 20.18)**	3.26 (0.86, 17.63)	1.43 (0.48, 5.71)	1.52 (0.45, 6.31)	CER10mgIV	1.11 (0.31, 5.03)	0.57 (0.13, 2.90)	1.60 (0.49, 6.50)	1.39 (0.53, 5.64)	1.65 (0.40, 8.19)	2.15 (0.54, 9.96)	1.62 (0.44, 7.83)	1.99 (0.52, 9.37)	2.44 (0.71, 12.02)	2.07 (0.52, 9.79)	**4.04 (1.22, 17.49)**
CER200mgSC	0.76 (0.16, 3.22)	1.42 (0.37, 5.33)	0.89 (0.24, 3.17)	0.87 (0.33, 2.25)	**3.64 (1.14, 12.47)**	2.88 (0.98, 10.01)	1.28 (0.63, 2.87)	1.39 (0.72, 2.72)	0.90 (0.20, 3.26)	CER200mgSC	0.51 (0.10, 1.93)	1.43 (0.76, 2.82)	1.30 (0.39, 4.08)	1.48 (0.48, 4.57)	1.90 (0.65, 5.90)	1.47 (0.52, 4.43)	1.78 (0.67, 5.21)	2.17 (0.90, 6.52)	1.83 (0.66, 5.30)	**3.63 (1.46, 9.42)**
CER20mgIV	1.50 (0.29, 9.78)	2.82 (0.48, 16.87)	1.78 (0.33, 10.95)	1.70 (0.43, 9.05)	**7.17 (1.66, 41.63)**	**5.65 (1.39, 30.91)**	2.51 (0.75, 11.09)	2.71 (0.75, 13.25)	1.76 (0.34, 7.93)	1.97 (0.52, 9.69)	CER20mgIV	2.82 (0.76, 13.32)	2.55 (0.74, 10.49)	2.87 (0.69, 16.87)	3.72 (0.94, 19.97)	2.91 (0.67, 16.63)	3.52 (0.91, 19.59)	**4.37 (1.14, 24.19)**	3.68 (0.89, 19.40)	**7.18 (1.91, 33.89)**
CER400mgSC	0.53 (0.11, 2.09)	0.99 (0.24, 3.53)	0.62 (0.18, 2.18)	0.61 (0.24, 1.47)	2.56 (0.84, 8.26)	1.99 (0.71, 6.61)	0.90 (0.46, 1.80)	0.97 (0.54, 1.69)	0.63 (0.15, 2.05)	0.70 (0.35, 1.31)	0.35 (0.08, 1.32)	CER400mgSC	0.90 (0.31, 2.58)	1.02 (0.36, 3.03)	1.32 (0.49, 3.66)	1.03 (0.40, 2.84)	1.27 (0.50, 3.26)	1.54 (0.65, 4.12)	1.28 (0.50, 3.40)	**2.55 (1.09, 6.27)**
CER5mgIV	0.60 (0.12, 2.66)	1.09 (0.24, 4.41)	0.71 (0.16, 2.76)	0.68 (0.22, 2.01)	2.82 (0.83, 10.93)	2.26 (0.68, 8.78)	1.00 (0.39, 2.71)	1.08 (0.37, 3.22)	0.72 (0.18, 1.89)	0.77 (0.25, 2.56)	0.39 (0.10, 1.35)	1.11 (0.39, 3.21)	CER5mgIV	1.12 (0.32, 4.16)	1.48 (0.45, 4.99)	1.13 (0.34, 3.84)	1.41 (0.43, 4.74)	1.73 (0.56, 5.91)	1.43 (0.44, 4.86)	2.85 (0.97, 8.70)
ELD10mg/kgIV	0.52 (0.11, 2.21)	0.95 (0.23, 3.75)	0.61 (0.16, 2.32)	0.59 (0.19, 1.85)	2.50 (0.69, 9.69)	1.95 (0.57, 7.67)	0.89 (0.34, 2.39)	0.97 (0.32, 2.75)	0.61 (0.12, 2.49)	0.67 (0.22, 2.10)	0.35 (0.06, 1.44)	0.98 (0.33, 2.77)	0.89 (0.24, 3.10)	ELD10mg/kgIV	1.30 (0.59, 2.88)	1.01 (0.31, 3.22)	1.23 (0.41, 3.94)	1.52 (0.52, 4.90)	1.25 (0.39, 4.28)	2.49 (0.84, 7.44)
ELD20mg/kgIV	0.40 (0.09, 1.67)	0.75 (0.17, 2.82)	0.46 (0.12, 1.74)	0.45 (0.15, 1.28)	1.88 (0.55, 6.75)	1.49 (0.46, 5.45)	0.67 (0.26, 1.69)	0.73 (0.26, 1.98)	0.47 (0.10, 1.87)	0.53 (0.17, 1.53)	0.27 (0.05, 1.07)	0.76 (0.27, 2.06)	0.68 (0.20, 2.22)	0.77 (0.35, 1.69)	ELD20mg/kgIV	0.77 (0.25, 2.29)	0.93 (0.31, 2.80)	1.16 (0.40, 3.43)	0.97 (0.32, 2.92)	1.90 (0.64, 5.55)
FON1mg/kg0.1mgIV/SC	0.53 (0.09, 2.18)	0.96 (0.21, 3.87)	0.61 (0.16, 2.42)	0.59 (0.19, 1.72)	2.53 (0.68, 8.83)	1.98 (0.57, 6.94)	0.88 (0.36, 2.22)	0.95 (0.34, 2.50)	0.62 (0.13, 2.30)	0.68 (0.23, 1.91)	0.34 (0.06, 1.49)	0.97 (0.35, 2.51)	0.88 (0.26, 2.92)	0.99 (0.31, 3.27)	1.29 (0.44, 3.98)	FON1mg/kg0.1mgIV/SC	1.24 (0.56, 2.65)	1.51 (0.78, 3.21)	1.26 (0.62, 2.66)	2.45 (0.86, 7.01)
FON1mg/kg1mgIV/SC	0.42 (0.10, 1.72)	0.79 (0.19, 2.95)	0.50 (0.13, 1.81)	0.49 (0.16, 1.32)	2.00 (0.56, 7.02)	1.59 (0.47, 5.70)	0.72 (0.31, 1.67)	0.77 (0.28, 2.00)	0.50 (0.11, 1.91)	0.56 (0.19, 1.50)	0.28 (0.05, 1.10)	0.79 (0.31, 1.99)	0.71 (0.21, 2.31)	0.81 (0.25, 2.45)	1.07 (0.36, 3.20)	0.81 (0.38, 1.78)	FON1mg/kg1mgIV/SC	1.22 (0.65, 2.41)	1.02 (0.48, 2.08)	2.02 (0.74, 5.35)
FON4mg/kg0.1mgIV/SC	0.35 (0.09, 1.28)	0.64 (0.15, 2.27)	0.40 (0.11, 1.45)	0.39 (0.13, 1.01)	1.65 (0.45, 5.54)	1.29 (0.39, 4.41)	0.59 (0.24, 1.27)	0.63 (0.24, 1.51)	0.41 (0.08, 1.41)	0.46 (0.15, 1.12)	**0.23 (0.04, 0.87)**	0.65 (0.24, 1.53)	0.58 (0.17, 1.77)	0.66 (0.20, 1.94)	0.87 (0.29, 2.51)	0.66 (0.31, 1.29)	0.82 (0.41, 1.53)	FON4mg/kg0.1mgIV/SC	0.83 (0.41, 1.61)	1.63 (0.59, 4.27)
FON4mg/kg1mgIV/SC	0.42 (0.08, 1.61)	0.75 (0.17, 2.87)	0.48 (0.13, 1.82)	0.47 (0.16, 1.33)	1.97 (0.53, 7.21)	1.55 (0.46, 5.58)	0.69 (0.28, 1.75)	0.75 (0.28, 2.07)	0.48 (0.10, 1.92)	0.55 (0.19, 1.51)	0.27 (0.05, 1.13)	0.78 (0.29, 2.01)	0.70 (0.21, 2.26)	0.80 (0.23, 2.54)	1.03 (0.34, 3.10)	0.80 (0.38, 1.60)	0.98 (0.48, 2.06)	1.20 (0.62, 2.41)	FON4mg/kg1mgIV/SC	1.95 (0.73, 5.56)
GUS1200mgIV	**0.21 (0.05, 0.79)**	0.39 (0.09, 1.38)	**0.25 (0.06, 0.85)**	**0.24 (0.09, 0.60)**	1.00 (0.32, 3.35)	0.77 (0.27, 2.67)	**0.36 (0.16, 0.74)**	**0.38 (0.15, 0.88)**	**0.25 (0.06, 0.82)**	**0.28 (0.11, 0.69)**	**0.14 (0.03, 0.52)**	**0.39 (0.16, 0.91)**	0.35 (0.11, 1.03)	0.40 (0.13, 1.19)	0.53 (0.18, 1.56)	0.41 (0.14, 1.16)	0.50 (0.19, 1.36)	0.61 (0.23, 1.71)	0.51 (0.18, 1.38)	GUS1200mgIV

(TABLE S12j. continued)

	ABA10mg/kgIV	ABA30mg/kgIV	ABA3mg/kgIV	AMI0.4mgOR	BRI400mgIV	BRI700mgIV	CDP10mg/kgIV	CER100mgSC	CER10mgIV	CER200mgSC	CER20mgIV	CER400mgSC	CER5mgIV	ELD10mg/kgIV	ELD20mg/kgIV	FON1mg/kg0.1mgIV/SC	FON1mg/kg1mgIV/SC	FON4mg/kg0.1mgIV/SC	FON4mg/kg1mgIV/SC	GUS1200mgIV
GUS200mgIV	**0.16 (0.04, 0.64)**	0.32 (0.07, 1.12)	**0.20 (0.05, 0.66)**	**0.19 (0.07, 0.47)**	0.80 (0.25, 2.65)	0.61 (0.21, 2.09)	**0.29 (0.13, 0.57)**	**0.31 (0.12, 0.69)**	**0.20 (0.05, 0.66)**	**0.22 (0.08, 0.53)**	**0.11 (0.02, 0.42)**	**0.32 (0.13, 0.71)**	**0.28 (0.10, 0.82)**	**0.32 (0.10, 0.91)**	0.42 (0.15, 1.22)	**0.32 (0.11, 0.89)**	0.40 (0.14, 1.06)	0.49 (0.19, 1.30)	0.41 (0.15, 1.08)	0.80 (0.53, 1.19)
GUS600mgIV	**0.17 (0.04, 0.66)**	0.32 (0.07, 1.14)	**0.20 (0.06, 0.68)**	**0.20 (0.08, 0.48)**	0.81 (0.26, 2.80)	0.63 (0.23, 2.15)	**0.30 (0.13, 0.60)**	**0.31 (0.12, 0.70)**	**0.21 (0.05, 0.68)**	**0.23 (0.09, 0.54)**	**0.12 (0.02, 0.41)**	**0.33 (0.13, 0.73)**	**0.29 (0.10, 0.84)**	**0.33 (0.11, 0.97)**	0.44 (0.15, 1.22)	**0.34 (0.11, 0.92)**	0.41 (0.15, 1.09)	0.50 (0.20, 1.35)	0.42 (0.15, 1.12)	0.83 (0.55, 1.22)
MED700IV	0.37 (0.08, 1.57)	0.69 (0.15, 3.01)	0.44 (0.10, 1.76)	0.42 (0.14, 1.32)	1.80 (0.48, 7.13)	1.42 (0.39, 5.42)	0.64 (0.24, 1.66)	0.68 (0.24, 1.91)	0.43 (0.10, 1.75)	0.49 (0.16, 1.41)	0.25 (0.05, 1.02)	0.71 (0.25, 1.99)	0.64 (0.18, 2.07)	0.73 (0.20, 2.52)	0.94 (0.27, 3.16)	0.73 (0.20, 2.34)	0.89 (0.28, 2.87)	1.11 (0.35, 3.41)	0.94 (0.26, 2.77)	1.81 (0.59, 5.08)
MIR1000mgIV	**0.21 (0.04, 0.94)**	0.39 (0.08, 1.62)	0.25 (0.05, 1.04)	**0.24 (0.07, 0.68)**	0.99 (0.25, 4.14)	0.77 (0.22, 3.58)	**0.36 (0.12, 0.91)**	0.38 (0.12, 1.18)	**0.25 (0.05, 0.93)**	**0.27 (0.08, 0.77)**	**0.14 (0.02, 0.57)**	0.39 (0.13, 1.13)	0.35 (0.10, 1.30)	0.40 (0.11, 1.38)	0.52 (0.15, 1.76)	0.39 (0.12, 1.34)	0.49 (0.15, 1.58)	0.60 (0.18, 1.92)	0.50 (0.15, 1.64)	1.00 (0.29, 3.18)
MIR200mgIV	0.36 (0.07, 1.96)	0.67 (0.12, 3.53)	0.42 (0.08, 2.20)	0.41 (0.10, 1.63)	1.75 (0.38, 7.61)	1.40 (0.31, 6.20)	0.62 (0.18, 2.22)	0.66 (0.17, 2.51)	0.44 (0.08, 1.92)	0.48 (0.12, 1.77)	0.24 (0.04, 1.39)	0.69 (0.18, 2.49)	0.61 (0.15, 2.49)	0.71 (0.16, 2.99)	0.91 (0.23, 3.73)	0.70 (0.17, 2.85)	0.88 (0.20, 3.69)	1.07 (0.28, 4.49)	0.88 (0.22, 3.72)	1.72 (0.43, 7.00)
MIR600mgIV	**0.13 (0.03, 0.59)**	0.25 (0.05, 1.04)	**0.16 (0.03, 0.66)**	**0.16 (0.04, 0.48)**	0.65 (0.17, 2.48)	0.51 (0.14, 1.87)	**0.23 (0.07, 0.60)**	**0.25 (0.08, 0.73)**	**0.16 (0.03, 0.62)**	**0.18 (0.05, 0.54)**	**0.09 (0.01, 0.38)**	**0.25 (0.08, 0.73)**	**0.22 (0.05, 0.78)**	**0.26 (0.07, 0.85)**	0.34 (0.09, 1.12)	**0.25 (0.08, 0.84)**	0.32 (0.10, 1.05)	0.40 (0.11, 1.26)	0.33 (0.09, 1.08)	0.65 (0.18, 2.01)
NAT300mgIV	0.43 (0.12, 1.40)	0.81 (0.21, 2.35)	0.50 (0.15, 1.48)	0.49 (0.22, 1.02)	2.03 (0.76, 5.69)	1.58 (0.68, 4.71)	0.73 (0.46, 1.15)	0.78 (0.40, 1.44)	0.51 (0.13, 1.42)	0.57 (0.27, 1.08)	**0.29 (0.07, 0.90)**	0.80 (0.42, 1.50)	0.72 (0.29, 1.75)	0.82 (0.33, 2.02)	1.08 (0.46, 2.55)	0.83 (0.36, 1.92)	1.01 (0.46, 2.23)	1.23 (0.61, 2.81)	1.04 (0.46, 2.32)	**2.04 (1.07, 4.28)**
NAT3mg/kgIV	0.58 (0.15, 2.13)	1.09 (0.27, 3.54)	0.68 (0.18, 2.32)	0.65 (0.25, 1.61)	2.75 (0.85, 8.95)	2.12 (0.75, 7.43)	0.97 (0.47, 1.95)	1.05 (0.44, 2.41)	0.68 (0.15, 2.25)	0.76 (0.29, 1.81)	0.38 (0.08, 1.44)	1.09 (0.46, 2.50)	0.97 (0.33, 2.84)	1.11 (0.37, 3.12)	1.46 (0.52, 4.04)	1.12 (0.40, 3.00)	1.35 (0.52, 3.49)	1.68 (0.66, 4.51)	1.40 (0.51, 3.87)	**2.73 (1.12, 6.91)**
NAT3mg/kgIVx2	0.37 (0.09, 1.35)	0.69 (0.17, 2.31)	0.44 (0.12, 1.44)	0.42 (0.17, 1.01)	1.78 (0.58, 5.77)	1.40 (0.49, 4.31)	0.63 (0.32, 1.21)	0.68 (0.29, 1.54)	0.44 (0.11, 1.42)	0.49 (0.20, 1.13)	**0.25 (0.05, 0.86)**	0.71 (0.31, 1.57)	0.63 (0.22, 1.79)	0.71 (0.25, 1.94)	0.95 (0.35, 2.48)	0.72 (0.27, 1.90)	0.88 (0.35, 2.23)	1.08 (0.45, 2.79)	0.92 (0.34, 2.39)	1.79 (0.79, 4.13)
NAT6mg/kgIVx2	0.40 (0.09, 1.55)	0.77 (0.18, 2.52)	0.48 (0.13, 1.62)	0.46 (0.19, 1.13)	1.96 (0.64, 6.15)	1.54 (0.55, 4.86)	0.69 (0.35, 1.36)	0.74 (0.32, 1.67)	0.49 (0.11, 1.57)	0.53 (0.22, 1.26)	**0.27 (0.06, 0.96)**	0.78 (0.33, 1.73)	0.69 (0.24, 1.94)	0.79 (0.27, 2.27)	1.02 (0.37, 2.84)	0.79 (0.29, 2.18)	0.96 (0.38, 2.45)	1.17 (0.50, 3.26)	0.99 (0.36, 2.64)	1.94 (0.82, 4.46)
NNC2mg/kgSC	0.36 (0.08, 1.62)	0.68 (0.15, 2.98)	0.42 (0.10, 1.83)	0.42 (0.12, 1.39)	1.73 (0.43, 7.28)	1.39 (0.36, 5.72)	0.62 (0.21, 1.80)	0.66 (0.21, 1.97)	0.44 (0.08, 1.74)	0.49 (0.14, 1.47)	0.25 (0.05, 1.05)	0.70 (0.22, 2.01)	0.61 (0.17, 2.29)	0.72 (0.17, 2.58)	0.94 (0.25, 3.08)	0.71 (0.20, 2.44)	0.88 (0.25, 2.91)	1.09 (0.31, 3.59)	0.88 (0.27, 2.96)	1.78 (0.52, 5.58)
ONE10mgSC	0.45 (0.12, 1.84)	0.89 (0.19, 3.17)	0.54 (0.14, 2.06)	0.54 (0.18, 1.41)	2.22 (0.62, 8.01)	1.73 (0.55, 6.50)	0.80 (0.32, 1.92)	0.86 (0.31, 2.19)	0.56 (0.12, 2.01)	0.62 (0.21, 1.66)	0.32 (0.06, 1.22)	0.88 (0.32, 2.22)	0.80 (0.23, 2.49)	0.88 (0.27, 2.87)	1.17 (0.39, 3.63)	0.90 (0.28, 2.74)	1.10 (0.38, 3.21)	1.36 (0.48, 3.97)	1.15 (0.39, 3.27)	2.20 (0.81, 5.96)
ONE25mgSC	0.99 (0.21, 4.45)	1.87 (0.40, 8.50)	1.17 (0.27, 5.40)	1.12 (0.33, 3.82)	**4.74 (1.20, 20.49)**	**3.71 (1.04, 15.94)**	1.65 (0.60, 4.90)	1.79 (0.60, 5.85)	1.15 (0.22, 5.03)	1.31 (0.40, 4.19)	0.67 (0.12, 2.98)	1.86 (0.61, 5.87)	1.70 (0.45, 6.10)	1.91 (0.53, 7.08)	2.49 (0.70, 9.49)	1.91 (0.57, 6.55)	2.34 (0.72, 8.14)	2.89 (0.91, 9.67)	2.42 (0.72, 8.80)	**4.67 (1.52, 15.18)**
ONE35mgSC	0.47 (0.11, 1.86)	0.88 (0.19, 3.49)	0.54 (0.14, 2.17)	0.53 (0.17, 1.55)	2.22 (0.65, 8.40)	1.74 (0.56, 6.63)	0.79 (0.31, 1.98)	0.86 (0.31, 2.30)	0.56 (0.12, 2.08)	0.62 (0.22, 1.69)	0.31 (0.06, 1.34)	0.88 (0.33, 2.31)	0.79 (0.22, 2.52)	0.91 (0.27, 2.96)	1.18 (0.37, 3.75)	0.91 (0.29, 2.79)	1.11 (0.37, 3.38)	1.37 (0.47, 4.02)	1.15 (0.37, 3.46)	2.23 (0.79, 6.06)
ONE50mgSC	0.67 (0.15, 3.02)	1.27 (0.27, 5.11)	0.80 (0.18, 3.14)	0.75 (0.27, 2.39)	3.19 (0.83, 12.38)	2.53 (0.75, 10.11)	1.12 (0.45, 3.19)	1.24 (0.43, 3.46)	0.77 (0.16, 3.22)	0.88 (0.29, 2.64)	0.46 (0.08, 1.83)	1.27 (0.44, 3.68)	1.14 (0.33, 3.94)	1.31 (0.37, 4.57)	1.68 (0.53, 5.65)	1.28 (0.41, 4.61)	1.57 (0.53, 5.28)	1.93 (0.67, 6.25)	1.63 (0.53, 5.43)	**3.18 (1.15, 9.94)**
ONT22.5mgSC	0.53 (0.14, 2.01)	1.00 (0.24, 3.67)	0.62 (0.17, 2.17)	0.61 (0.23, 1.57)	2.53 (0.75, 8.88)	2.00 (0.64, 7.32)	0.91 (0.41, 2.06)	0.96 (0.38, 2.30)	0.63 (0.14, 2.17)	0.70 (0.26, 1.75)	0.36 (0.08, 1.37)	1.01 (0.40, 2.47)	0.89 (0.29, 2.88)	1.05 (0.32, 3.06)	1.36 (0.45, 3.82)	1.03 (0.34, 3.17)	1.27 (0.44, 3.64)	1.54 (0.56, 4.53)	1.29 (0.44, 3.80)	**2.54 (1.01, 6.93)**
ONT225mgSC	0.48 (0.13, 1.98)	0.91 (0.22, 3.20)	0.58 (0.15, 2.01)	0.56 (0.21, 1.41)	2.30 (0.71, 9.16)	1.80 (0.61, 7.38)	0.82 (0.37, 1.83)	0.89 (0.36, 2.43)	0.58 (0.13, 1.90)	0.64 (0.24, 1.61)	0.33 (0.07, 1.23)	0.92 (0.37, 2.40)	0.81 (0.27, 2.80)	0.95 (0.30, 2.81)	1.23 (0.42, 3.47)	0.95 (0.31, 2.94)	1.17 (0.42, 3.33)	1.43 (0.54, 3.98)	1.18 (0.42, 3.29)	2.30 (0.95, 6.12)
ONT75mgSC	0.49 (0.12, 1.90)	0.94 (0.23, 3.44)	0.59 (0.16, 2.05)	0.56 (0.20, 1.49)	2.35 (0.70, 7.96)	1.89 (0.62, 6.25)	0.83 (0.38, 1.93)	0.91 (0.36, 2.22)	0.59 (0.13, 1.99)	0.66 (0.24, 1.66)	0.33 (0.07, 1.22)	0.93 (0.38, 2.27)	0.83 (0.28, 2.49)	0.97 (0.29, 2.85)	1.28 (0.42, 3.61)	0.96 (0.31, 2.93)	1.18 (0.42, 3.33)	1.43 (0.54, 4.13)	1.20 (0.43, 3.50)	2.32 (0.98, 6.34)
PBO	0.60 (0.18, 1.93)	1.13 (0.31, 3.28)	0.71 (0.22, 2.06)	0.68 (0.32, 1.35)	**2.81 (1.11, 7.80)**	2.19 (0.99, 6.43)	1.01 (0.68, 1.52)	1.09 (0.59, 2.02)	0.72 (0.19, 1.93)	0.79 (0.38, 1.46)	0.40 (0.10, 1.24)	1.12 (0.62, 2.00)	1.00 (0.41, 2.34)	1.14 (0.47, 2.76)	1.50 (0.66, 3.42)	1.14 (0.51, 2.61)	1.40 (0.67, 2.99)	1.72 (0.89, 3.81)	1.45 (0.66, 3.13)	**2.85 (1.55, 5.74)**
PF10mgSC	0.66 (0.12, 3.79)	1.19 (0.22, 6.53)	0.76 (0.16, 4.07)	0.73 (0.19, 3.26)	3.13 (0.68, 15.70)	2.44 (0.57, 11.80)	1.09 (0.32, 4.55)	1.19 (0.30, 5.10)	0.77 (0.12, 4.03)	0.84 (0.22, 3.66)	0.42 (0.07, 2.69)	1.21 (0.33, 5.09)	1.11 (0.23, 5.25)	1.24 (0.29, 5.92)	1.59 (0.42, 7.68)	1.25 (0.30, 5.91)	1.53 (0.40, 7.33)	1.92 (0.51, 8.94)	1.56 (0.40, 7.24)	3.08 (0.86, 13.74)

	ABA10mg/kgIV	ABA30mg/kgIV	ABA3mg/kgIV	AMI0.4mgOR	BRI400mgIV	BRI700mgIV	CDP10mg/kgIV	CER100mgSC	CER10mgIV	CER200mgSC	CER20mgIV	CER400mgSC	CER5mgIV	ELD10mg/kgIV	ELD20mg/kgIV	FON1mg/kg0.1mgIV/SC	FON1mg/kg1mgIV/SC	FON4mg/kg0.1mgIV/SC	FON4mg/kg1mgIV/SC	GUS1200mgIV
PF200mgSC	0.67 (0.12, 4.40)	1.26 (0.21, 9.19)	0.79 (0.14, 5.32)	0.75 (0.16, 4.60)	3.13 (0.62, 20.28)	2.46 (0.52, 16.66)	1.13 (0.27, 6.11)	1.21 (0.24, 6.87)	0.80 (0.11, 4.96)	0.87 (0.19, 4.94)	0.45 (0.06, 3.04)	1.26 (0.28, 7.23)	1.13 (0.22, 6.49)	1.28 (0.26, 7.75)	1.71 (0.36, 9.80)	1.29 (0.25, 7.49)	1.62 (0.34, 9.89)	1.98 (0.43, 11.21)	1.61 (0.34, 9.27)	3.13 (0.72, 18.30)
PF50mgSC	**0.21 (0.04, 0.88)**	0.39 (0.08, 1.55)	0.25 (0.06, 1.01)	**0.24 (0.07, 0.72)**	1.03 (0.26, 3.71)	0.80 (0.22, 2.94)	**0.36 (0.13, 0.96)**	0.39 (0.12, 1.16)	0.25 (0.05, 1.01)	**0.28 (0.09, 0.82)**	**0.14 (0.02, 0.61)**	0.40 (0.13, 1.11)	0.36 (0.09, 1.25)	0.41 (0.11, 1.35)	0.52 (0.15, 1.75)	0.41 (0.12, 1.37)	0.50 (0.14, 1.69)	0.62 (0.20, 1.98)	0.50 (0.16, 1.64)	1.00 (0.34, 3.06)
RIS1200mgIV	0.34 (0.11, 1.06)	0.64 (0.17, 1.87)	0.40 (0.12, 1.18)	**0.39 (0.18, 0.78)**	1.59 (0.59, 4.55)	1.24 (0.53, 3.75)	**0.57 (0.36, 0.90)**	0.61 (0.32, 1.23)	0.41 (0.10, 1.11)	**0.44 (0.22, 0.88)**	**0.23 (0.05, 0.73)**	0.63 (0.34, 1.17)	0.56 (0.22, 1.38)	0.65 (0.25, 1.58)	0.85 (0.37, 2.00)	0.64 (0.28, 1.51)	0.79 (0.36, 1.77)	0.97 (0.48, 2.19)	0.82 (0.36, 1.87)	1.60 (0.85, 3.29)
RIS200mgIV	0.44 (0.12, 1.81)	0.86 (0.18, 3.28)	0.53 (0.15, 1.88)	0.51 (0.18, 1.37)	2.17 (0.59, 7.71)	1.69 (0.52, 6.30)	0.76 (0.33, 1.83)	0.81 (0.33, 2.16)	0.52 (0.11, 1.96)	0.59 (0.22, 1.65)	0.30 (0.06, 1.19)	0.84 (0.35, 2.14)	0.76 (0.24, 2.51)	0.87 (0.25, 2.73)	1.13 (0.39, 3.52)	0.85 (0.30, 2.88)	1.06 (0.38, 3.25)	1.29 (0.48, 4.04)	1.08 (0.38, 3.46)	2.17 (0.78, 5.74)
RIS600mgIV	**0.31 (0.10, 0.98)**	0.59 (0.15, 1.75)	0.36 (0.11, 1.09)	**0.36 (0.16, 0.71)**	1.45 (0.55, 4.17)	1.14 (0.48, 3.41)	**0.53 (0.33, 0.82)**	0.56 (0.30, 1.15)	0.37 (0.10, 1.03)	**0.41 (0.20, 0.80)**	**0.21 (0.05, 0.67)**	0.58 (0.31, 1.07)	0.52 (0.21, 1.26)	0.60 (0.23, 1.45)	0.78 (0.34, 1.83)	0.59 (0.26, 1.38)	0.73 (0.33, 1.64)	0.90 (0.45, 2.00)	0.75 (0.34, 1.66)	1.48 (0.78, 3.05)
RIS600mgIV4/4	0.43 (0.13, 1.45)	0.81 (0.21, 2.49)	0.50 (0.15, 1.56)	0.48 (0.21, 1.05)	2.01 (0.73, 6.06)	1.57 (0.63, 4.78)	0.72 (0.42, 1.26)	0.77 (0.38, 1.55)	0.51 (0.12, 1.53)	0.56 (0.25, 1.15)	**0.29 (0.07, 0.94)**	0.81 (0.40, 1.58)	0.71 (0.28, 1.83)	0.81 (0.31, 2.12)	1.08 (0.43, 2.59)	0.83 (0.33, 2.00)	1.01 (0.43, 2.35)	1.22 (0.57, 2.93)	1.04 (0.44, 2.43)	2.04 (0.97, 4.43)
TES400mg200mgSC	0.29 (0.08, 1.08)	0.56 (0.13, 1.84)	0.35 (0.09, 1.16)	**0.34 (0.13, 0.83)**	1.40 (0.44, 4.63)	1.10 (0.37, 3.72)	0.51 (0.23, 1.05)	0.54 (0.22, 1.24)	0.35 (0.08, 1.16)	**0.39 (0.14, 0.94)**	**0.20 (0.04, 0.70)**	0.56 (0.23, 1.26)	0.50 (0.16, 1.49)	0.56 (0.19, 1.63)	0.74 (0.27, 2.08)	0.56 (0.20, 1.61)	0.70 (0.25, 1.84)	0.86 (0.34, 2.26)	0.71 (0.26, 1.90)	1.42 (0.57, 3.61)
UPA12mgPO_BID	0.46 (0.06, 2.59)	0.84 (0.14, 4.86)	0.53 (0.10, 2.93)	0.52 (0.11, 2.34)	2.17 (0.40, 12.21)	1.71 (0.35, 9.61)	0.78 (0.19, 3.07)	0.83 (0.18, 3.65)	0.53 (0.09, 2.88)	0.60 (0.13, 2.57)	0.31 (0.04, 1.90)	0.86 (0.20, 3.65)	0.77 (0.16, 3.66)	0.86 (0.16, 4.49)	1.15 (0.24, 5.59)	0.86 (0.21, 3.78)	1.09 (0.26, 4.87)	1.34 (0.32, 5.79)	1.11 (0.26, 4.89)	2.24 (0.47, 9.45)
UPA24mgPO	0.77 (0.09, 8.03)	1.44 (0.20, 11.37)	0.89 (0.16, 7.30)	0.88 (0.16, 6.18)	3.78 (0.57, 25.63)	2.96 (0.47, 23.70)	1.32 (0.26, 8.64)	1.42 (0.25, 10.54)	0.92 (0.12, 6.77)	1.02 (0.18, 7.06)	0.52 (0.06, 4.89)	1.47 (0.27, 10.21)	1.30 (0.22, 8.71)	1.51 (0.23, 11.27)	2.01 (0.35, 14.83)	1.51 (0.25, 9.14)	1.88 (0.35, 12.99)	2.26 (0.43, 15.13)	1.89 (0.35, 12.53)	3.68 (0.71, 25.39)
UPA24mgPO_BID	0.24 (0.03, 1.21)	0.45 (0.07, 2.25)	0.28 (0.06, 1.31)	0.28 (0.06, 1.04)	1.12 (0.23, 5.27)	0.90 (0.18, 4.33)	0.42 (0.10, 1.36)	0.44 (0.09, 1.54)	0.29 (0.05, 1.28)	0.32 (0.07, 1.10)	**0.16 (0.02, 0.75)**	0.46 (0.10, 1.51)	0.41 (0.08, 1.64)	0.47 (0.09, 2.00)	0.61 (0.13, 2.52)	0.46 (0.11, 1.79)	0.57 (0.14, 2.17)	0.72 (0.17, 2.78)	0.58 (0.14, 2.26)	1.15 (0.26, 4.41)
UPA3mgPO_BID	0.62 (0.11, 4.36)	1.16 (0.19, 7.82)	0.74 (0.14, 4.76)	0.71 (0.15, 3.92)	3.10 (0.56, 15.66)	2.47 (0.44, 13.06)	1.06 (0.24, 4.92)	1.16 (0.23, 5.65)	0.74 (0.11, 3.98)	0.81 (0.17, 4.06)	0.42 (0.06, 2.52)	1.17 (0.25, 5.69)	1.05 (0.22, 5.55)	1.24 (0.21, 6.43)	1.60 (0.32, 8.31)	1.24 (0.26, 6.28)	1.47 (0.33, 7.96)	1.85 (0.40, 9.34)	1.52 (0.34, 7.86)	3.01 (0.64, 15.31)
UPA45mgPO	0.34 (0.10, 1.14)	0.65 (0.17, 1.87)	0.40 (0.12, 1.23)	**0.39 (0.17, 0.80)**	1.62 (0.59, 4.57)	1.26 (0.52, 3.68)	**0.58 (0.37, 0.93)**	0.62 (0.32, 1.16)	0.41 (0.11, 1.13)	**0.45 (0.21, 0.88)**	**0.23 (0.05, 0.75)**	0.64 (0.33, 1.20)	0.57 (0.23, 1.38)	0.65 (0.25, 1.62)	0.86 (0.37, 2.01)	0.66 (0.29, 1.54)	0.80 (0.37, 1.80)	0.98 (0.48, 2.28)	0.83 (0.37, 1.88)	1.62 (0.84, 3.34)
UPA6mgPO_BID	0.20 (0.03, 1.07)	0.38 (0.07, 1.82)	0.23 (0.05, 1.16)	**0.23 (0.06, 0.86)**	0.99 (0.21, 4.06)	0.78 (0.17, 3.34)	0.35 (0.09, 1.10)	0.37 (0.09, 1.24)	0.23 (0.04, 1.04)	**0.27 (0.06, 0.96)**	**0.13 (0.02, 0.66)**	0.38 (0.10, 1.28)	0.35 (0.08, 1.37)	0.39 (0.08, 1.68)	0.52 (0.12, 2.11)	0.39 (0.10, 1.43)	0.48 (0.13, 1.81)	0.59 (0.16, 2.14)	0.48 (0.12, 1.86)	0.97 (0.25, 3.52)
UST6mg/kg90mgIV/SC	**0.21 (0.05, 0.78)**	0.39 (0.09, 1.36)	**0.25 (0.07, 0.83)**	**0.24 (0.09, 0.59)**	0.99 (0.32, 3.29)	0.78 (0.27, 2.66)	**0.36 (0.16, 0.75)**	**0.38 (0.14, 0.88)**	**0.24 (0.05, 0.86)**	**0.27 (0.11, 0.68)**	**0.14 (0.03, 0.52)**	**0.39 (0.16, 0.93)**	0.35 (0.12, 1.07)	0.41 (0.13, 1.18)	0.52 (0.18, 1.53)	0.40 (0.13, 1.14)	0.49 (0.18, 1.34)	0.60 (0.23, 1.61)	0.51 (0.18, 1.38)	1.00 (0.65, 1.52)
VED300mgIV	0.33 (0.08, 1.30)	0.62 (0.14, 2.34)	0.39 (0.10, 1.37)	0.39 (0.12, 1.06)	1.58 (0.44, 5.99)	1.24 (0.36, 4.72)	0.57 (0.22, 1.38)	0.61 (0.20, 1.56)	0.41 (0.08, 1.40)	0.44 (0.14, 1.21)	**0.23 (0.04, 0.86)**	0.63 (0.22, 1.60)	0.56 (0.17, 1.81)	0.63 (0.19, 2.17)	0.84 (0.26, 2.63)	0.64 (0.18, 1.98)	0.80 (0.24, 2.34)	0.99 (0.32, 2.82)	0.81 (0.25, 2.41)	1.61 (0.57, 4.49)

	GUS200mgIV	GUS600mgIV	MED700IV	MIR1000mgIV	MIR200mgIV	MIR600mgIV	NAT300mgIV	NAT3mg/kgIV	NAT3mg/kgIVx2	NAT6mg/kgIVx2	NNC2mg/kgSC	ONE10mgSC	ONE25mgSC	ONE35mgSC	ONE50mgSC	ONT22.5mgSC	ONT225mgSC	ONT75mgSC	PBO	PF10mgSC
ABA10mg/kgIV	**6.13 (1.56, 22.78)**	**5.83 (1.52, 22.23)**	2.69 (0.64, 13.08)	**4.79 (1.07, 24.34)**	2.76 (0.51, 14.92)	**7.54 (1.69, 37.39)**	2.35 (0.71, 8.20)	1.73 (0.47, 6.63)	2.71 (0.74, 11.36)	2.48 (0.64, 10.69)	2.75 (0.62, 12.24)	2.20 (0.54, 8.59)	1.01 (0.22, 4.69)	2.15 (0.54, 9.25)	1.50 (0.33, 6.59)	1.88 (0.50, 7.05)	2.08 (0.51, 7.62)	2.05 (0.53, 8.22)	1.67 (0.52, 5.59)	1.51 (0.26, 8.60)
ABA30mg/kgIV	3.17 (0.89, 13.96)	3.09 (0.88, 13.56)	1.45 (0.33, 6.48)	2.58 (0.62, 12.93)	1.49 (0.28, 8.11)	3.97 (0.96, 20.52)	1.24 (0.43, 4.66)	0.92 (0.28, 3.77)	1.44 (0.43, 5.75)	1.30 (0.40, 5.46)	1.47 (0.34, 6.76)	1.12 (0.32, 5.31)	0.53 (0.12, 2.49)	1.14 (0.29, 5.29)	0.79 (0.20, 3.75)	1.00 (0.27, 4.12)	1.10 (0.31, 4.50)	1.06 (0.29, 4.43)	0.89 (0.30, 3.27)	0.84 (0.15, 4.45)
ABA3mg/kgIV	**5.05 (1.52, 19.32)**	**5.00 (1.46, 18.06)**	2.28 (0.57, 9.75)	4.03 (0.96, 18.67)	2.38 (0.45, 12.69)	**6.35 (1.51, 29.63)**	1.99 (0.68, 6.45)	1.46 (0.43, 5.51)	2.26 (0.70, 8.30)	2.08 (0.62, 7.56)	2.37 (0.55, 10.24)	1.84 (0.48, 7.24)	0.86 (0.19, 3.77)	1.85 (0.46, 7.26)	1.26 (0.32, 5.42)	1.61 (0.46, 6.03)	1.72 (0.50, 6.50)	1.71 (0.49, 6.34)	1.42 (0.49, 4.53)	1.32 (0.25, 6.43)
AMI0.4mgPO	**5.24 (2.14, 13.55)**	**5.07 (2.08, 12.91)**	2.39 (0.76, 7.09)	**4.17 (1.47, 14.80)**	2.43 (0.61, 9.60)	**6.40 (2.06, 24.81)**	2.05 (0.98, 4.58)	1.54 (0.62, 4.02)	2.38 (0.99, 5.77)	2.17 (0.88, 5.32)	2.40 (0.72, 8.61)	1.86 (0.71, 5.51)	0.89 (0.26, 3.04)	1.88 (0.65, 5.79)	1.33 (0.42, 3.67)	1.63 (0.64, 4.39)	1.80 (0.71, 4.78)	1.78 (0.67, 4.89)	1.48 (0.74, 3.13)	1.38 (0.31, 5.17)
BRI400mgIV	1.25 (0.38, 3.97)	1.23 (0.36, 3.79)	0.56 (0.14, 2.08)	1.01 (0.24, 3.94)	0.57 (0.13, 2.65)	1.55 (0.40, 5.94)	0.49 (0.18, 1.31)	0.36 (0.11, 1.17)	0.56 (0.17, 1.73)	0.51 (0.16, 1.55)	0.58 (0.14, 2.31)	0.45 (0.12, 1.61)	**0.21 (0.05, 0.83)**	0.45 (0.12, 1.54)	0.31 (0.08, 1.20)	0.39 (0.11, 1.33)	0.44 (0.11, 1.42)	0.42 (0.13, 1.42)	**0.36 (0.13, 0.90)**	0.32 (0.06, 1.48)
BRI700mgIV	1.63 (0.48, 4.75)	1.58 (0.46, 4.29)	0.70 (0.18, 2.56)	1.29 (0.28, 4.53)	0.71 (0.16, 3.22)	1.98 (0.53, 7.37)	0.63 (0.21, 1.47)	0.47 (0.13, 1.34)	0.72 (0.23, 2.02)	0.65 (0.21, 1.81)	0.72 (0.17, 2.77)	0.58 (0.15, 1.83)	**0.27 (0.06, 0.96)**	0.58 (0.15, 1.77)	0.40 (0.10, 1.34)	0.50 (0.14, 1.57)	0.55 (0.14, 1.64)	0.53 (0.16, 1.62)	0.46 (0.16, 1.01)	0.41 (0.08, 1.76)
CDP10mg/kgIV	**3.47 (1.74, 7.90)**	**3.37 (1.66, 7.54)**	1.57 (0.60, 4.11)	**2.79 (1.10, 8.32)**	1.62 (0.45, 5.50)	**4.33 (1.65, 13.77)**	1.38 (0.87, 2.18)	1.03 (0.51, 2.11)	1.58 (0.83, 3.08)	1.45 (0.74, 2.89)	1.61 (0.55, 4.76)	1.25 (0.52, 3.12)	0.61 (0.20, 1.67)	1.26 (0.51, 3.19)	0.89 (0.31, 2.21)	1.10 (0.48, 2.45)	1.22 (0.55, 2.68)	1.21 (0.52, 2.62)	0.99 (0.66, 1.47)	0.91 (0.22, 3.08)
CER100mgSC	**3.27 (1.45, 8.42)**	**3.19 (1.42, 8.13)**	1.46 (0.52, 4.15)	2.62 (0.85, 8.14)	1.52 (0.40, 5.84)	**4.08 (1.38, 13.11)**	1.28 (0.69, 2.50)	0.96 (0.41, 2.25)	1.47 (0.65, 3.47)	1.35 (0.60, 3.16)	1.50 (0.51, 4.65)	1.17 (0.46, 3.21)	0.56 (0.17, 1.65)	1.17 (0.44, 3.21)	0.80 (0.29, 2.31)	1.04 (0.43, 2.62)	1.13 (0.41, 2.80)	1.10 (0.45, 2.80)	0.92 (0.49, 1.69)	0.84 (0.20, 3.33)
CER10mgIV	**5.12 (1.51, 21.73)**	**4.84 (1.46, 21.15)**	2.32 (0.57, 10.26)	**4.03 (1.07, 19.71)**	2.30 (0.52, 13.17)	**6.24 (1.61, 32.05)**	1.96 (0.71, 7.65)	1.46 (0.44, 6.46)	2.26 (0.70, 9.25)	2.04 (0.64, 8.85)	2.27 (0.58, 13.29)	1.80 (0.50, 8.64)	0.87 (0.20, 4.65)	1.77 (0.48, 8.12)	1.29 (0.31, 6.18)	1.59 (0.46, 6.95)	1.72 (0.53, 7.64)	1.70 (0.50, 7.62)	1.40 (0.52, 5.33)	1.30 (0.25, 8.66)
CER200mgSC	**4.55 (1.87, 12.09)**	**4.41 (1.84, 11.46)**	2.05 (0.71, 6.40)	**3.65 (1.29, 11.94)**	2.10 (0.56, 8.29)	**5.64 (1.85, 19.38)**	1.76 (0.92, 3.74)	1.31 (0.55, 3.42)	2.03 (0.89, 5.06)	1.88 (0.79, 4.61)	2.05 (0.68, 6.92)	1.60 (0.60, 4.79)	0.76 (0.24, 2.50)	1.62 (0.59, 4.63)	1.14 (0.38, 3.40)	1.44 (0.57, 3.90)	1.56 (0.62, 4.19)	1.53 (0.60, 4.23)	1.26 (0.68, 2.60)	1.19 (0.27, 4.52)
CER20mgIV	**8.88 (2.41, 43.03)**	**8.60 (2.43, 40.26)**	4.07 (0.98, 21.62)	**7.17 (1.75, 42.27)**	4.14 (0.72, 25.53)	**11.13 (2.65, 67.40)**	**3.48 (1.11, 14.72)**	2.62 (0.70, 12.44)	**4.02 (1.16, 18.96)**	**3.72 (1.04, 17.26)**	4.07 (0.95, 22.12)	3.15 (0.82, 16.77)	1.49 (0.34, 8.45)	3.19 (0.75, 16.12)	2.18 (0.55, 12.38)	2.78 (0.73, 13.33)	3.07 (0.81, 14.28)	3.02 (0.82, 14.31)	2.50 (0.81, 10.37)	2.39 (0.37, 15.17)
CER400mgSC	**3.16 (1.41, 7.79)**	**3.06 (1.37, 7.60)**	1.41 (0.50, 3.97)	2.54 (0.88, 7.83)	1.45 (0.40, 5.66)	**3.94 (1.38, 12.86)**	1.24 (0.67, 2.36)	0.91 (0.40, 2.17)	1.41 (0.64, 3.27)	1.28 (0.58, 3.01)	1.42 (0.50, 4.46)	1.14 (0.45, 3.10)	0.54 (0.17, 1.64)	1.14 (0.43, 3.06)	0.79 (0.27, 2.25)	0.99 (0.40, 2.52)	1.09 (0.42, 2.69)	1.07 (0.44, 2.64)	0.89 (0.50, 1.61)	0.83 (0.20, 3.02)
CER5mgIV	**3.58 (1.22, 10.49)**	**3.48 (1.19, 10.30)**	1.57 (0.48, 5.54)	2.85 (0.77, 10.49)	1.64 (0.40, 6.81)	**4.45 (1.29, 18.47)**	1.38 (0.57, 3.48)	1.03 (0.35, 3.04)	1.60 (0.56, 4.63)	1.45 (0.52, 4.14)	1.64 (0.44, 5.79)	1.26 (0.40, 4.39)	0.59 (0.16, 2.21)	1.26 (0.40, 4.53)	0.88 (0.25, 3.06)	1.12 (0.35, 3.42)	1.23 (0.36, 3.65)	1.20 (0.40, 3.53)	1.00 (0.43, 2.42)	0.90 (0.19, 4.35)
ELD10mg/kgIV	**3.09 (1.09, 9.68)**	**2.99 (1.03, 8.98)**	1.37 (0.40, 5.04)	2.47 (0.73, 9.39)	1.40 (0.33, 6.24)	**3.87 (1.17, 14.98)**	1.22 (0.49, 3.03)	0.90 (0.32, 2.69)	1.40 (0.52, 4.00)	1.27 (0.44, 3.64)	1.40 (0.39, 5.84)	1.13 (0.35, 3.67)	0.52 (0.14, 1.87)	1.10 (0.34, 3.67)	0.77 (0.22, 2.70)	0.95 (0.33, 3.16)	1.06 (0.36, 3.30)	1.03 (0.35, 3.48)	0.87 (0.36, 2.13)	0.80 (0.17, 3.40)
ELD20mg/kgIV	2.39 (0.82, 6.84)	2.30 (0.82, 6.53)	1.07 (0.32, 3.66)	1.92 (0.57, 6.71)	1.10 (0.27, 4.38)	2.95 (0.90, 10.93)	0.92 (0.39, 2.17)	0.69 (0.25, 1.91)	1.05 (0.40, 2.84)	0.98 (0.35, 2.69)	1.06 (0.32, 4.00)	0.85 (0.28, 2.59)	0.40 (0.11, 1.43)	0.85 (0.27, 2.67)	0.60 (0.18, 1.87)	0.73 (0.26, 2.23)	0.81 (0.29, 2.39)	0.78 (0.28, 2.35)	0.67 (0.29, 1.51)	0.63 (0.13, 2.38)
FON1mg/kg0.1mgIV/SC	**3.08 (1.13, 9.13)**	**2.97 (1.09, 8.75)**	1.37 (0.43, 4.88)	2.55 (0.75, 8.40)	1.44 (0.35, 5.80)	**3.92 (1.20, 13.12)**	1.21 (0.52, 2.77)	0.89 (0.33, 2.47)	1.39 (0.53, 3.65)	1.27 (0.46, 3.47)	1.40 (0.41, 5.07)	1.11 (0.37, 3.58)	0.52 (0.15, 1.74)	1.09 (0.36, 3.48)	0.78 (0.22, 2.44)	0.97 (0.32, 2.96)	1.06 (0.34, 3.24)	1.04 (0.34, 3.25)	0.87 (0.38, 1.95)	0.80 (0.17, 3.30)
FON1mg/kg1mgIV/SC	2.51 (0.94, 6.93)	2.45 (0.92, 6.65)	1.12 (0.35, 3.54)	2.03 (0.63, 6.65)	1.14 (0.27, 4.88)	3.13 (0.95, 10.42)	0.99 (0.45, 2.16)	0.74 (0.29, 1.92)	1.14 (0.45, 2.87)	1.04 (0.41, 2.63)	1.13 (0.34, 4.07)	0.91 (0.31, 2.64)	0.43 (0.12, 1.39)	0.90 (0.30, 2.67)	0.64 (0.19, 1.89)	0.79 (0.27, 2.25)	0.86 (0.30, 2.36)	0.85 (0.30, 2.39)	0.72 (0.33, 1.50)	0.65 (0.14, 2.53)
FON4mg/kg0.1mgIV/SC	2.06 (0.77, 5.23)	1.99 (0.74, 5.02)	0.90 (0.29, 2.88)	1.65 (0.52, 5.55)	0.94 (0.22, 3.62)	2.52 (0.79, 8.93)	0.82 (0.36, 1.64)	0.60 (0.22, 1.51)	0.93 (0.36, 2.20)	0.85 (0.31, 2.02)	0.92 (0.28, 3.25)	0.74 (0.25, 2.07)	0.35 (0.10, 1.10)	0.73 (0.25, 2.11)	0.52 (0.16, 1.49)	0.65 (0.22, 1.78)	0.70 (0.25, 1.86)	0.70 (0.24, 1.85)	0.58 (0.26, 1.12)	0.52 (0.11, 1.97)
FON4mg/kg1mgIV/SC	2.44 (0.92, 6.89)	2.37 (0.89, 6.66)	1.07 (0.36, 3.85)	2.00 (0.61, 6.75)	1.13 (0.27, 4.63)	3.07 (0.93, 10.53)	0.96 (0.43, 2.17)	0.71 (0.26, 1.95)	1.09 (0.42, 2.94)	1.01 (0.38, 2.80)	1.13 (0.34, 3.72)	0.87 (0.31, 2.57)	0.41 (0.11, 1.39)	0.87 (0.29, 2.69)	0.61 (0.18, 1.87)	0.78 (0.26, 2.27)	0.85 (0.30, 2.37)	0.83 (0.29, 2.31)	0.69 (0.32, 1.51)	0.64 (0.14, 2.50)
GUS1200mgIV	1.25 (0.84, 1.88)	1.20 (0.82, 1.82)	0.55 (0.20, 1.70)	1.00 (0.31, 3.40)	0.58 (0.14, 2.30)	1.55 (0.50, 5.71)	**0.49 (0.23, 0.94)**	**0.37 (0.14, 0.89)**	0.56 (0.24, 1.27)	0.52 (0.22, 1.22)	0.56 (0.18, 1.93)	0.46 (0.17, 1.24)	**0.21 (0.07, 0.66)**	0.45 (0.16, 1.26)	**0.31 (0.10, 0.87)**	**0.39 (0.14, 0.99)**	0.43 (0.16, 1.05)	0.43 (0.16, 1.03)	**0.35 (0.17, 0.64)**	0.32 (0.07, 1.16)

(TABLE S12j. continued)

	GUS200mgIV	GUS600mgIV	MED700IV	MIR1000mgIV	MIR200mgIV	MIR600mgIV	NAT300mgIV	NAT3mg/kgIV	NAT3mg/kgIVx2	NAT6mg/kgIVx2	NNC2mg/kgSC	ONE10mgSC	ONE25mgSC	ONE35mgSC	ONE50mgSC	ONT22.5mgSC	ONT225mgSC	ONT75mgSC	PBO	PF10mgSC
GUS200mgIV	GUS200mgIV	0.97 (0.68, 1.40)	0.45 (0.16, 1.32)	0.79 (0.25, 2.67)	0.46 (0.11, 1.82)	1.23 (0.39, 4.49)	**0.39 (0.19, 0.73)**	**0.29 (0.12, 0.70)**	**0.45 (0.19, 0.99)**	**0.41 (0.18, 0.93)**	0.45 (0.14, 1.47)	**0.36 (0.13, 0.95)**	**0.17 (0.05, 0.50)**	**0.36 (0.13, 0.98)**	**0.25 (0.08, 0.68)**	**0.32 (0.12, 0.77)**	**0.35 (0.13, 0.82)**	**0.35 (0.13, 0.82)**	**0.28 (0.14, 0.51)**	**0.26 (0.06, 0.98)**
GUS600mgIV	1.03 (0.71, 1.47)	GUS600mgIV	0.45 (0.16, 1.36)	0.83 (0.26, 2.81)	0.48 (0.12, 1.85)	1.28 (0.42, 4.59)	**0.41 (0.20, 0.76)**	**0.30 (0.12, 0.73)**	0.46 (0.20, 1.01)	**0.43 (0.19, 0.97)**	0.47 (0.15, 1.55)	0.37 (0.13, 1.00)	**0.18 (0.06, 0.54)**	**0.37 (0.13, 0.99)**	**0.26 (0.09, 0.71)**	**0.33 (0.12, 0.82)**	**0.36 (0.14, 0.84)**	**0.36 (0.13, 0.86)**	**0.29 (0.15, 0.52)**	**0.27 (0.06, 0.98)**
MED700IV	2.25 (0.76, 6.44)	2.20 (0.73, 6.24)	MED700IV	1.78 (0.52, 7.35)	1.02 (0.25, 4.51)	2.79 (0.81, 10.96)	0.88 (0.35, 2.11)	0.64 (0.22, 1.91)	1.01 (0.35, 2.83)	0.91 (0.32, 2.61)	1.01 (0.29, 3.99)	0.82 (0.24, 2.57)	0.37 (0.10, 1.39)	0.80 (0.23, 2.56)	0.56 (0.15, 1.84)	0.71 (0.22, 2.26)	0.78 (0.24, 2.34)	0.76 (0.24, 2.36)	0.63 (0.25, 1.49)	0.59 (0.12, 2.51)
MIR1000mgIV	1.26 (0.37, 3.98)	1.20 (0.36, 3.83)	0.56 (0.14, 1.91)	MIR1000mgIV	0.59 (0.20, 1.35)	1.57 (0.83, 2.81)	0.49 (0.17, 1.20)	0.37 (0.12, 1.03)	0.56 (0.18, 1.58)	0.52 (0.16, 1.44)	0.57 (0.14, 2.08)	0.44 (0.13, 1.49)	**0.20 (0.05, 0.75)**	0.45 (0.13, 1.41)	0.31 (0.08, 1.02)	0.40 (0.12, 1.11)	0.43 (0.13, 1.20)	0.43 (0.13, 1.21)	**0.35 (0.12, 0.83)**	0.31 (0.07, 1.43)
MIR200mgIV	2.15 (0.55, 8.80)	2.08 (0.54, 8.20)	0.98 (0.22, 4.08)	1.69 (0.74, 4.94)	MIR200mgIV	**2.69 (1.15, 7.96)**	0.85 (0.26, 2.78)	0.63 (0.18, 2.44)	0.98 (0.28, 3.48)	0.90 (0.24, 3.20)	0.99 (0.22, 4.62)	0.78 (0.19, 3.52)	0.37 (0.08, 1.58)	0.79 (0.19, 3.34)	0.54 (0.13, 2.38)	0.68 (0.18, 2.73)	0.73 (0.20, 2.91)	0.73 (0.20, 2.95)	0.61 (0.19, 1.91)	0.56 (0.10, 3.00)
MIR600mgIV	0.81 (0.22, 2.57)	0.78 (0.22, 2.40)	0.36 (0.09, 1.23)	0.64 (0.36, 1.20)	**0.37 (0.13, 0.87)**	MIR600mgIV	**0.32 (0.10, 0.77)**	**0.24 (0.07, 0.67)**	0.37 (0.11, 1.03)	**0.34 (0.10, 0.93)**	0.37 (0.10, 1.49)	0.28 (0.08, 1.04)	**0.13 (0.03, 0.51)**	0.29 (0.08, 1.01)	**0.20 (0.05, 0.73)**	**0.25 (0.07, 0.82)**	**0.27 (0.08, 0.82)**	**0.28 (0.08, 0.83)**	**0.23 (0.08, 0.54)**	**0.20 (0.04, 0.93)**
NAT300mgIV	**2.54 (1.37, 5.32)**	**2.44 (1.32, 5.11)**	1.14 (0.47, 2.90)	2.03 (0.83, 5.86)	1.18 (0.36, 3.88)	**3.15 (1.29, 9.56)**	NAT300mgIV	0.74 (0.39, 1.43)	1.15 (0.65, 2.07)	1.05 (0.58, 1.92)	1.16 (0.46, 3.24)	0.91 (0.41, 2.11)	0.44 (0.15, 1.16)	0.91 (0.40, 2.16)	0.65 (0.25, 1.51)	0.80 (0.38, 1.68)	0.87 (0.44, 1.79)	0.86 (0.41, 1.79)	**0.72 (0.57, 0.90)**	0.67 (0.17, 2.05)
NAT3mg/kgIV	**3.42 (1.43, 8.49)**	**3.31 (1.38, 8.10)**	1.55 (0.52, 4.56)	2.72 (0.97, 8.39)	1.58 (0.41, 5.67)	**4.23 (1.49, 14.37)**	1.34 (0.70, 2.54)	NAT3mg/kgIV	1.54 (0.93, 2.61)	1.42 (0.80, 2.50)	1.59 (0.51, 4.94)	1.21 (0.47, 3.25)	0.59 (0.18, 1.69)	1.24 (0.45, 3.21)	0.87 (0.29, 2.33)	1.07 (0.42, 2.74)	1.18 (0.47, 2.87)	1.18 (0.45, 2.80)	0.97 (0.52, 1.75)	0.90 (0.20, 3.10)
NAT3mg/kgIVx2	**2.22 (1.01, 5.21)**	2.17 (0.99, 4.95)	0.99 (0.35, 2.85)	1.77 (0.63, 5.70)	1.02 (0.29, 3.61)	2.71 (0.97, 9.28)	0.87 (0.48, 1.54)	0.65 (0.38, 1.08)	NAT3mg/kgIVx2	0.92 (0.56, 1.48)	1.02 (0.33, 3.23)	0.79 (0.32, 2.04)	0.38 (0.12, 1.11)	0.80 (0.30, 2.13)	0.56 (0.19, 1.49)	0.69 (0.30, 1.68)	0.76 (0.33, 1.78)	0.76 (0.32, 1.75)	0.63 (0.37, 1.05)	0.59 (0.13, 1.96)
NAT6mg/kgIVx2	**2.43 (1.07, 5.69)**	**2.35 (1.03, 5.37)**	1.10 (0.38, 3.17)	1.94 (0.70, 6.44)	1.11 (0.31, 4.21)	**2.96 (1.08, 10.09)**	0.95 (0.52, 1.72)	0.71 (0.40, 1.25)	1.09 (0.67, 1.79)	NAT6mg/kgIVx2	1.12 (0.36, 3.53)	0.86 (0.35, 2.29)	0.41 (0.13, 1.23)	0.87 (0.32, 2.33)	0.61 (0.20, 1.67)	0.75 (0.31, 1.88)	0.84 (0.33, 2.02)	0.82 (0.34, 2.00)	0.69 (0.39, 1.19)	0.64 (0.14, 2.22)
NNC2mg/kgSC	2.21 (0.68, 7.19)	2.14 (0.65, 6.78)	0.99 (0.25, 3.48)	1.76 (0.48, 7.08)	1.01 (0.22, 4.50)	2.69 (0.67, 10.45)	0.86 (0.31, 2.18)	0.63 (0.20, 1.97)	0.98 (0.31, 3.00)	0.89 (0.28, 2.81)	NNC2mg/kgSC	0.79 (0.23, 2.69)	0.38 (0.09, 1.39)	0.81 (0.20, 2.73)	0.56 (0.14, 2.00)	0.69 (0.20, 2.19)	0.75 (0.23, 2.35)	0.74 (0.22, 2.32)	0.62 (0.22, 1.54)	0.56 (0.10, 2.41)
ONE10mgSC	**2.76 (1.05, 7.67)**	2.67 (1.00, 7.54)	1.22 (0.39, 4.13)	2.30 (0.67, 7.50)	1.28 (0.28, 5.14)	3.55 (0.96, 12.20)	1.10 (0.47, 2.43)	0.83 (0.31, 2.11)	1.27 (0.49, 3.08)	1.16 (0.44, 2.86)	1.27 (0.37, 4.41)	ONE10mgSC	0.48 (0.17, 1.12)	0.99 (0.49, 2.03)	0.70 (0.29, 1.47)	0.87 (0.30, 2.47)	0.94 (0.34, 2.71)	0.94 (0.35, 2.58)	0.79 (0.35, 1.64)	0.73 (0.16, 2.94)
ONE25mgSC	**5.83 (2.01, 19.54)**	**5.64 (1.87, 18.08)**	2.67 (0.72, 9.80)	**4.89 (1.33, 18.59)**	2.67 (0.63, 12.68)	**7.42 (1.97, 28.71)**	2.27 (0.86, 6.55)	1.70 (0.59, 5.57)	2.61 (0.90, 8.07)	2.43 (0.81, 7.42)	2.64 (0.72, 11.70)	2.10 (0.89, 5.82)	ONE25mgSC	2.09 (0.87, 5.80)	1.44 (0.54, 4.37)	1.81 (0.60, 6.35)	1.99 (0.68, 6.92)	1.94 (0.64, 6.91)	1.63 (0.65, 4.60)	1.57 (0.30, 6.88)
ONE35mgSC	**2.78 (1.02, 7.80)**	**2.71 (1.01, 7.61)**	1.26 (0.39, 4.36)	2.24 (0.71, 7.58)	1.27 (0.30, 5.26)	3.47 (0.99, 11.78)	1.10 (0.46, 2.52)	0.81 (0.31, 2.24)	1.25 (0.47, 3.30)	1.15 (0.43, 3.12)	1.24 (0.37, 4.97)	1.01 (0.49, 2.04)	0.48 (0.17, 1.15)	ONE35mgSC	0.71 (0.29, 1.55)	0.87 (0.32, 2.54)	0.95 (0.34, 2.78)	0.94 (0.35, 2.68)	0.79 (0.35, 1.80)	0.73 (0.14, 2.97)
ONE50mgSC	**3.99 (1.48, 12.15)**	**3.85 (1.41, 11.55)**	1.80 (0.54, 6.52)	3.19 (0.98, 12.88)	1.86 (0.42, 7.97)	**5.00 (1.37, 20.73)**	1.55 (0.66, 4.00)	1.15 (0.43, 3.51)	1.79 (0.67, 5.20)	1.64 (0.60, 4.94)	1.79 (0.50, 7.17)	1.42 (0.68, 3.43)	0.69 (0.23, 1.87)	1.42 (0.65, 3.43)	ONE50mgSC	1.26 (0.41, 3.95)	1.38 (0.47, 4.13)	1.37 (0.46, 4.16)	1.12 (0.49, 2.82)	1.03 (0.22, 4.37)
ONT22.5mgSC	**3.17 (1.29, 8.52)**	**3.02 (1.22, 8.43)**	1.42 (0.44, 4.63)	2.53 (0.90, 8.55)	1.47 (0.37, 5.63)	**3.92 (1.22, 13.58)**	1.25 (0.60, 2.63)	0.94 (0.36, 2.35)	1.44 (0.59, 3.38)	1.33 (0.53, 3.25)	1.44 (0.46, 4.91)	1.15 (0.41, 3.30)	0.55 (0.16, 1.68)	1.15 (0.39, 3.17)	0.80 (0.25, 2.46)	ONT22.5mgSC	1.09 (0.57, 2.07)	1.08 (0.56, 2.09)	0.89 (0.44, 1.80)	0.85 (0.18, 3.13)
ONT225mgSC	**2.87 (1.22, 7.76)**	**2.78 (1.19, 7.13)**	1.29 (0.43, 4.19)	2.30 (0.84, 7.59)	1.37 (0.34, 5.05)	**3.67 (1.21, 12.12)**	1.14 (0.56, 2.29)	0.84 (0.35, 2.12)	1.32 (0.56, 3.04)	1.19 (0.49, 3.07)	1.33 (0.43, 4.41)	1.06 (0.37, 2.97)	0.50 (0.14, 1.48)	1.06 (0.36, 2.98)	0.73 (0.24, 2.11)	0.92 (0.48, 1.75)	ONT225mgSC	0.98 (0.53, 2.10)	0.82 (0.42, 1.59)	0.76 (0.17, 2.77)
ONT75mgSC	**2.89 (1.22, 7.69)**	**2.81 (1.17, 7.87)**	1.31 (0.42, 4.10)	2.35 (0.83, 7.66)	1.37 (0.34, 5.06)	**3.64 (1.20, 11.89)**	1.16 (0.56, 2.43)	0.85 (0.36, 2.20)	1.32 (0.57, 3.15)	1.22 (0.50, 2.98)	1.34 (0.43, 4.49)	1.06 (0.39, 2.89)	0.52 (0.14, 1.57)	1.06 (0.37, 2.83)	0.73 (0.24, 2.16)	0.92 (0.48, 1.78)	1.02 (0.48, 1.88)	ONT75mgSC	0.82 (0.42, 1.66)	0.78 (0.17, 2.93)
PBO	**3.52 (1.98, 7.05)**	**3.41 (1.93, 6.74)**	1.59 (0.67, 3.94)	**2.83 (1.21, 8.02)**	1.64 (0.52, 5.18)	**4.37 (1.84, 12.83)**	**1.39 (1.11, 1.76)**	1.03 (0.57, 1.93)	1.59 (0.95, 2.73)	1.46 (0.84, 2.58)	1.61 (0.65, 4.47)	1.26 (0.61, 2.83)	0.61 (0.22, 1.54)	1.27 (0.56, 2.84)	0.89 (0.35, 2.06)	1.13 (0.56, 2.26)	1.21 (0.63, 2.38)	1.21 (0.60, 2.38)	PBO	0.91 (0.24, 2.81)
PF10mgSC	**3.85 (1.02, 18.11)**	**3.71 (1.02, 16.82)**	1.69 (0.40, 8.62)	3.21 (0.70, 15.16)	1.79 (0.33, 9.94)	**4.97 (1.08, 22.89)**	1.50 (0.49, 5.81)	1.11 (0.32, 5.00)	1.70 (0.51, 7.82)	1.57 (0.45, 7.04)	1.79 (0.42, 9.75)	1.38 (0.34, 6.25)	0.64 (0.15, 3.29)	1.38 (0.34, 6.95)	0.97 (0.23, 4.55)	1.18 (0.32, 5.66)	1.31 (0.36, 6.02)	1.28 (0.34, 6.00)	1.09 (0.36, 4.22)	PF10mgSC

	GUS200mgIV	GUS600mgIV	MED700IV	MIR1000mgIV	MIR200mgIV	MIR600mgIV	NAT300mgIV	NAT3mg/kgIV	NAT3mg/kgIVx2	NAT6mg/kgIVx2	NNC2mg/kgSC	ONE10mgSC	ONE25mgSC	ONE35mgSC	ONE50mgSC	ONT22.5mgSC	ONT225mgSC	ONT75mgSC	PBO	PF10mgSC
PF200mgSC	3.89 (0.92, 24.17)	3.76 (0.89, 22.51)	1.78 (0.34, 10.40)	3.25 (0.61, 21.05)	1.85 (0.32, 13.32)	5.05 (0.98, 32.50)	1.53 (0.38, 8.29)	1.15 (0.27, 6.65)	1.75 (0.41, 10.26)	1.60 (0.37, 9.21)	1.84 (0.34, 11.61)	1.44 (0.28, 8.46)	0.67 (0.13, 4.03)	1.43 (0.28, 8.61)	0.99 (0.19, 5.84)	1.23 (0.27, 7.20)	1.34 (0.31, 7.86)	1.36 (0.29, 7.94)	1.10 (0.28, 6.07)	1.01 (0.22, 5.42)
PF50mgSC	1.26 (0.42, 3.90)	1.21 (0.41, 3.81)	0.56 (0.15, 1.97)	1.03 (0.27, 3.65)	0.57 (0.13, 2.55)	1.55 (0.41, 6.03)	0.49 (0.18, 1.22)	0.36 (0.12, 1.07)	0.56 (0.19, 1.58)	0.50 (0.17, 1.48)	0.58 (0.15, 2.20)	0.45 (0.13, 1.45)	**0.21 (0.05, 0.76)**	0.44 (0.13, 1.51)	0.31 (0.09, 1.07)	0.38 (0.13, 1.26)	0.44 (0.14, 1.35)	0.43 (0.14, 1.31)	**0.35 (0.13, 0.85)**	**0.33 (0.10, 0.83)**
RIS1200mgIV	**1.99 (1.05, 4.12)**	**1.94 (1.06, 3.90)**	0.90 (0.36, 2.27)	1.60 (0.66, 4.53)	0.93 (0.28, 3.02)	2.46 (0.96, 7.35)	0.78 (0.58, 1.07)	0.58 (0.31, 1.11)	0.90 (0.51, 1.61)	0.82 (0.45, 1.51)	0.91 (0.35, 2.57)	0.72 (0.33, 1.63)	**0.34 (0.12, 0.90)**	0.72 (0.30, 1.67)	0.50 (0.20, 1.19)	0.64 (0.30, 1.31)	0.69 (0.34, 1.38)	0.69 (0.33, 1.37)	**0.57 (0.45, 0.70)**	0.52 (0.13, 1.63)
RIS200mgIV	**2.70 (1.03, 7.20)**	2.64 (0.99, 6.75)	1.22 (0.39, 3.73)	2.15 (0.71, 7.60)	1.23 (0.31, 5.09)	**3.31 (1.04, 11.52)**	1.04 (0.49, 2.37)	0.78 (0.29, 1.99)	1.20 (0.49, 3.04)	1.11 (0.42, 2.84)	1.24 (0.39, 4.07)	0.96 (0.32, 2.92)	0.45 (0.13, 1.64)	0.96 (0.32, 2.91)	0.67 (0.21, 2.12)	0.86 (0.30, 2.34)	0.91 (0.35, 2.57)	0.91 (0.33, 2.58)	0.75 (0.37, 1.67)	0.69 (0.15, 2.86)
RIS600mgIV	1.83 (0.98, 3.72)	1.79 (0.96, 3.57)	0.83 (0.34, 2.04)	1.46 (0.62, 4.13)	0.85 (0.26, 2.76)	2.27 (0.90, 6.87)	**0.72 (0.53, 0.98)**	0.53 (0.29, 1.02)	0.83 (0.47, 1.46)	0.76 (0.42, 1.38)	0.84 (0.33, 2.42)	0.66 (0.31, 1.50)	**0.32 (0.11, 0.82)**	0.66 (0.28, 1.56)	0.46 (0.18, 1.11)	0.59 (0.28, 1.21)	0.63 (0.31, 1.26)	0.63 (0.30, 1.26)	**0.52 (0.42, 0.63)**	0.48 (0.12, 1.50)
RIS600mgIV4/4	**2.52 (1.25, 5.46)**	**2.46 (1.21, 5.28)**	1.14 (0.44, 3.07)	2.04 (0.79, 5.63)	1.17 (0.35, 3.84)	**3.14 (1.20, 9.29)**	1.00 (0.64, 1.51)	0.73 (0.37, 1.46)	1.14 (0.61, 2.18)	1.04 (0.53, 2.01)	1.16 (0.42, 3.38)	0.90 (0.40, 2.14)	0.44 (0.15, 1.17)	0.91 (0.37, 2.20)	0.64 (0.24, 1.58)	0.80 (0.36, 1.73)	0.87 (0.39, 1.84)	0.86 (0.39, 1.84)	0.72 (0.49, 1.02)	0.66 (0.16, 2.18)
TES400mg200mgSC	1.76 (0.71, 4.45)	1.72 (0.69, 4.32)	0.78 (0.25, 2.32)	1.39 (0.48, 4.97)	0.82 (0.19, 3.23)	2.16 (0.68, 7.42)	0.70 (0.34, 1.32)	0.51 (0.21, 1.24)	0.80 (0.34, 1.86)	0.73 (0.31, 1.65)	0.79 (0.24, 2.54)	0.65 (0.22, 1.75)	**0.30 (0.09, 0.93)**	0.64 (0.21, 1.76)	0.44 (0.14, 1.25)	0.56 (0.21, 1.36)	0.61 (0.23, 1.48)	0.61 (0.22, 1.44)	**0.50 (0.25, 0.92)**	0.46 (0.11, 1.67)
UPA12mgPO_BID	2.78 (0.60, 12.13)	2.71 (0.59, 11.25)	1.22 (0.24, 6.11)	2.22 (0.38, 11.07)	1.29 (0.20, 7.22)	3.45 (0.56, 17.39)	1.07 (0.26, 4.07)	0.79 (0.18, 3.33)	1.23 (0.29, 5.22)	1.12 (0.25, 4.73)	1.27 (0.24, 5.82)	1.00 (0.21, 4.52)	0.45 (0.09, 2.43)	0.99 (0.20, 4.69)	0.69 (0.13, 3.25)	0.85 (0.18, 4.23)	0.93 (0.20, 4.48)	0.91 (0.20, 4.25)	0.77 (0.19, 2.92)	0.69 (0.13, 3.79)
UPA24mgPO	4.67 (0.87, 31.08)	4.48 (0.87, 30.28)	2.09 (0.33, 16.71)	3.89 (0.50, 24.04)	2.24 (0.27, 14.79)	6.12 (0.81, 33.95)	1.84 (0.38, 11.56)	1.34 (0.26, 8.71)	2.05 (0.40, 13.63)	1.90 (0.35, 12.55)	2.20 (0.33, 15.47)	1.71 (0.26, 11.56)	0.80 (0.11, 5.90)	1.71 (0.27, 11.99)	1.16 (0.18, 8.79)	1.45 (0.26, 10.57)	1.58 (0.29, 10.90)	1.57 (0.28, 11.66)	1.31 (0.27, 8.32)	1.17 (0.19, 9.91)
UPA24mgPO_BID	1.47 (0.32, 5.47)	1.42 (0.32, 5.26)	0.65 (0.13, 2.86)	1.20 (0.21, 4.79)	0.67 (0.11, 3.13)	1.87 (0.33, 7.46)	0.57 (0.14, 1.74)	0.42 (0.09, 1.57)	0.66 (0.15, 2.27)	0.60 (0.13, 2.18)	0.66 (0.12, 2.79)	0.51 (0.10, 2.05)	0.24 (0.04, 1.07)	0.53 (0.10, 2.08)	0.37 (0.07, 1.50)	0.45 (0.09, 1.74)	0.51 (0.10, 1.82)	0.49 (0.11, 1.81)	0.41 (0.10, 1.23)	0.37 (0.06, 1.81)
UPA3mgPO_BID	3.75 (0.82, 18.94)	3.64 (0.79, 18.27)	1.65 (0.32, 9.46)	3.05 (0.52, 17.45)	1.79 (0.28, 11.33)	4.64 (0.82, 28.53)	1.45 (0.35, 6.38)	1.08 (0.22, 5.65)	1.67 (0.36, 8.35)	1.53 (0.33, 7.78)	1.76 (0.30, 9.20)	1.36 (0.26, 7.10)	0.64 (0.11, 3.53)	1.34 (0.26, 7.32)	0.94 (0.17, 5.36)	1.17 (0.24, 6.21)	1.28 (0.28, 6.62)	1.25 (0.26, 6.80)	1.04 (0.24, 4.57)	0.97 (0.17, 6.39)
UPA45mgPO	**2.01 (1.09, 4.14)**	**1.95 (1.05, 4.05)**	0.92 (0.37, 2.33)	1.61 (0.68, 4.47)	0.94 (0.28, 3.01)	**2.50 (1.02, 7.45)**	0.79 (0.57, 1.09)	0.59 (0.32, 1.13)	0.92 (0.51, 1.65)	0.84 (0.45, 1.50)	0.92 (0.36, 2.60)	0.72 (0.33, 1.69)	**0.35 (0.12, 0.90)**	0.72 (0.31, 1.67)	0.51 (0.20, 1.21)	0.64 (0.30, 1.32)	0.69 (0.35, 1.41)	0.69 (0.33, 1.40)	**0.57 (0.45, 0.72)**	0.52 (0.13, 1.68)
UPA6mgPO_BID	1.23 (0.31, 4.33)	1.18 (0.31, 4.26)	0.54 (0.12, 2.41)	1.01 (0.20, 4.06)	0.57 (0.10, 2.56)	1.56 (0.31, 6.11)	0.48 (0.14, 1.43)	0.36 (0.09, 1.24)	0.55 (0.15, 1.86)	0.50 (0.13, 1.72)	0.56 (0.12, 2.26)	0.44 (0.10, 1.61)	**0.21 (0.04, 0.84)**	0.45 (0.10, 1.71)	0.31 (0.07, 1.17)	0.38 (0.09, 1.42)	0.42 (0.11, 1.48)	0.41 (0.10, 1.48)	0.34 (0.10, 1.02)	0.31 (0.06, 1.52)
UST6mg/kg90mgIV/SC	1.25 (0.84, 1.85)	1.20 (0.81, 1.81)	0.56 (0.19, 1.69)	1.00 (0.31, 3.49)	0.58 (0.14, 2.27)	1.55 (0.48, 5.68)	**0.49 (0.24, 0.96)**	**0.36 (0.15, 0.88)**	0.56 (0.24, 1.27)	0.51 (0.22, 1.21)	0.56 (0.18, 1.98)	0.45 (0.16, 1.25)	**0.21 (0.07, 0.63)**	0.45 (0.16, 1.25)	**0.31 (0.10, 0.88)**	0.39 (0.14, 1.02)	0.43 (0.16, 1.05)	0.43 (0.16, 1.04)	**0.35 (0.18, 0.67)**	0.33 (0.07, 1.19)
VED300mgIV	2.01 (0.68, 5.48)	1.95 (0.67, 5.34)	0.90 (0.27, 2.93)	1.60 (0.45, 5.77)	0.91 (0.21, 3.66)	2.48 (0.71, 8.41)	0.79 (0.31, 1.72)	0.58 (0.21, 1.56)	0.89 (0.32, 2.34)	0.83 (0.30, 2.11)	0.91 (0.25, 2.96)	0.72 (0.22, 2.18)	0.35 (0.09, 1.11)	0.72 (0.22, 2.16)	0.50 (0.14, 1.59)	0.64 (0.19, 1.77)	0.68 (0.23, 1.91)	0.68 (0.22, 1.92)	0.57 (0.23, 1.21)	0.50 (0.11, 2.13)

	PF200mgSC	PF50mgSC	RIS1200mgIV	RIS200mgIV	RIS600mgIV	RIS600mgIV4/4	TES400mg200mgSC	UPA12mgOR_BID	UPA24mgOR	UPA24mgOR_BID	UPA3mgOR_BID	UPA45mgOR	UPA6mgOR_BID	UST6mg/kg90mgIV/SC	VED300mgIV
ABA10mg/kgIV	1.50 (0.23, 8.49)	**4.71 (1.13, 26.89)**	2.98 (0.94, 9.30)	2.27 (0.55, 8.52)	**3.23 (1.02, 9.90)**	2.32 (0.69, 7.92)	3.42 (0.93, 12.46)	2.16 (0.39, 17.49)	1.29 (0.12, 11.11)	4.17 (0.82, 34.64)	1.60 (0.23, 9.33)	2.92 (0.88, 9.84)	4.96 (0.94, 32.89)	**4.83 (1.28, 18.62)**	3.02 (0.77, 12.52)
ABA30mg/kgIV	0.79 (0.11, 4.82)	2.58 (0.65, 11.90)	1.56 (0.53, 6.03)	1.17 (0.30, 5.66)	1.70 (0.57, 6.64)	1.24 (0.40, 4.86)	1.78 (0.54, 7.70)	1.20 (0.21, 7.25)	0.69 (0.09, 5.11)	2.22 (0.44, 13.68)	0.86 (0.13, 5.22)	1.55 (0.53, 5.85)	2.66 (0.55, 15.19)	2.55 (0.73, 11.38)	1.61 (0.43, 7.32)
ABA3mg/kgIV	1.26 (0.19, 7.16)	4.06 (0.99, 17.35)	2.52 (0.84, 8.31)	1.90 (0.53, 6.83)	2.75 (0.92, 9.01)	2.02 (0.64, 6.68)	2.88 (0.86, 11.02)	1.88 (0.34, 10.01)	1.12 (0.14, 6.34)	3.56 (0.77, 17.44)	1.36 (0.21, 7.06)	2.52 (0.81, 8.21)	4.33 (0.86, 19.66)	**4.07 (1.20, 15.34)**	2.56 (0.73, 10.49)
AMI0.4mgPO	1.33 (0.22, 6.13)	**4.17 (1.38, 13.89)**	**2.60 (1.28, 5.67)**	1.96 (0.73, 5.44)	**2.82 (1.40, 6.27)**	2.08 (0.95, 4.66)	**2.97 (1.20, 7.90)**	1.92 (0.43, 9.31)	1.14 (0.16, 6.07)	3.61 (0.96, 17.47)	1.42 (0.26, 6.88)	**2.60 (1.25, 5.73)**	**4.29 (1.17, 17.88)**	**4.23 (1.70, 10.97)**	2.57 (0.95, 8.38)
BRI400mgIV	0.32 (0.05, 1.60)	0.97 (0.27, 3.86)	0.63 (0.22, 1.68)	0.46 (0.13, 1.70)	0.69 (0.24, 1.83)	0.50 (0.17, 1.37)	0.72 (0.22, 2.30)	0.46 (0.08, 2.53)	0.26 (0.04, 1.75)	0.89 (0.19, 4.39)	0.32 (0.06, 1.80)	0.62 (0.22, 1.69)	1.01 (0.25, 4.68)	1.01 (0.30, 3.12)	0.63 (0.17, 2.28)
BRI700mgIV	0.41 (0.06, 1.93)	1.25 (0.34, 4.46)	0.81 (0.27, 1.90)	0.59 (0.16, 1.94)	0.88 (0.29, 2.08)	0.64 (0.21, 1.59)	0.91 (0.27, 2.72)	0.59 (0.10, 2.86)	0.34 (0.04, 2.11)	1.11 (0.23, 5.52)	0.41 (0.08, 2.30)	0.79 (0.27, 1.91)	1.29 (0.30, 5.88)	1.28 (0.38, 3.74)	0.81 (0.21, 2.77)
CDP10mg/kgIV	0.89 (0.16, 3.75)	**2.81 (1.04, 7.91)**	**1.75 (1.11, 2.76)**	1.31 (0.55, 3.01)	**1.89 (1.21, 3.01)**	1.39 (0.79, 2.40)	1.98 (0.95, 4.31)	1.28 (0.33, 5.36)	0.76 (0.12, 3.84)	2.40 (0.74, 10.36)	0.95 (0.20, 4.19)	**1.73 (1.08, 2.74)**	2.90 (0.91, 10.59)	**2.80 (1.32, 6.41)**	1.75 (0.73, 4.57)
CER100mgSC	0.82 (0.15, 4.14)	2.57 (0.86, 8.44)	1.63 (0.82, 3.09)	1.23 (0.46, 3.01)	1.77 (0.87, 3.39)	1.29 (0.65, 2.65)	1.85 (0.80, 4.55)	1.21 (0.27, 5.67)	0.70 (0.09, 3.99)	2.25 (0.65, 10.64)	0.86 (0.18, 4.27)	1.61 (0.86, 3.13)	2.70 (0.81, 11.26)	**2.62 (1.13, 7.10)**	1.63 (0.64, 4.93)
CER10mgIV	1.25 (0.20, 8.94)	4.05 (0.99, 20.93)	2.46 (0.90, 9.53)	1.91 (0.51, 9.31)	2.69 (0.97, 10.44)	1.98 (0.65, 8.07)	2.84 (0.86, 12.58)	1.90 (0.35, 11.21)	1.08 (0.15, 8.04)	3.50 (0.78, 20.41)	1.35 (0.25, 9.22)	2.46 (0.88, 9.41)	4.26 (0.96, 22.58)	**4.09 (1.17, 18.19)**	2.43 (0.71, 13.13)
CER200mgSC	1.15 (0.20, 5.35)	**3.63 (1.22, 11.68)**	**2.25 (1.14, 4.65)**	1.68 (0.60, 4.47)	**2.45 (1.26, 5.10)**	1.78 (0.87, 4.05)	**2.55 (1.07, 7.07)**	1.66 (0.39, 7.80)	0.98 (0.14, 5.65)	3.15 (0.91, 14.07)	1.23 (0.25, 6.06)	**2.23 (1.13, 4.72)**	**3.73 (1.04, 15.40)**	**3.67 (1.47, 9.42)**	2.28 (0.83, 7.10)
CER20mgIV	2.21 (0.33, 17.49)	**7.12 (1.63, 42.94)**	**4.39 (1.38, 18.38)**	3.29 (0.84, 16.07)	**4.76 (1.49, 19.69)**	**3.49 (1.06, 14.75)**	**5.01 (1.43, 24.74)**	3.26 (0.53, 24.50)	1.94 (0.20, 17.32)	**6.23 (1.33, 47.71)**	2.40 (0.40, 18.18)	**4.38 (1.33, 18.52)**	**7.44 (1.51, 47.27)**	**7.14 (1.91, 32.90)**	**4.43 (1.16, 23.65)**
CER400mgSC	0.80 (0.14, 3.56)	2.51 (0.90, 7.59)	1.59 (0.86, 2.93)	1.19 (0.47, 2.88)	1.73 (0.94, 3.22)	1.24 (0.63, 2.51)	1.77 (0.79, 4.31)	1.16 (0.27, 5.02)	0.68 (0.10, 3.76)	2.18 (0.66, 9.73)	0.85 (0.18, 4.00)	1.57 (0.83, 2.99)	2.60 (0.78, 10.04)	**2.53 (1.07, 6.29)**	1.58 (0.62, 4.52)
CER5mgIV	0.88 (0.15, 4.49)	2.81 (0.80, 11.38)	1.77 (0.73, 4.47)	1.31 (0.40, 4.21)	1.93 (0.79, 4.86)	1.40 (0.55, 3.59)	1.99 (0.67, 6.17)	1.31 (0.27, 6.43)	0.77 (0.11, 4.61)	2.44 (0.61, 12.03)	0.95 (0.18, 4.64)	1.75 (0.73, 4.37)	2.90 (0.73, 12.26)	2.86 (0.93, 8.65)	1.79 (0.55, 5.78)
ELD10mg/kgIV	0.78 (0.13, 3.77)	2.47 (0.74, 9.06)	1.55 (0.63, 3.95)	1.15 (0.37, 4.04)	1.67 (0.69, 4.29)	1.24 (0.47, 3.20)	1.77 (0.61, 5.16)	1.16 (0.22, 6.11)	0.66 (0.09, 4.31)	2.14 (0.50, 11.36)	0.81 (0.16, 4.82)	1.54 (0.62, 3.96)	2.56 (0.60, 12.77)	2.46 (0.85, 7.56)	1.58 (0.46, 5.24)
ELD20mg/kgIV	0.59 (0.10, 2.79)	1.91 (0.57, 6.55)	1.18 (0.50, 2.73)	0.88 (0.28, 2.56)	1.28 (0.55, 2.98)	0.93 (0.39, 2.32)	1.35 (0.48, 3.75)	0.87 (0.18, 4.12)	0.50 (0.07, 2.83)	1.65 (0.40, 7.61)	0.63 (0.12, 3.13)	1.16 (0.50, 2.73)	1.94 (0.47, 8.44)	1.91 (0.66, 5.64)	1.20 (0.38, 3.80)
FON1mg/kg0.1mgIV/SC	0.78 (0.13, 3.96)	2.45 (0.73, 8.65)	1.55 (0.66, 3.60)	1.18 (0.35, 3.33)	1.69 (0.73, 3.88)	1.21 (0.50, 3.04)	1.77 (0.62, 4.97)	1.16 (0.26, 4.87)	0.66 (0.11, 3.96)	2.18 (0.56, 9.49)	0.81 (0.16, 3.86)	1.52 (0.65, 3.51)	2.54 (0.70, 10.48)	2.49 (0.88, 7.52)	1.56 (0.51, 5.60)
FON1mg/kg1mgIV/SC	0.62 (0.10, 2.93)	2.01 (0.59, 7.00)	1.26 (0.56, 2.77)	0.94 (0.31, 2.61)	1.37 (0.61, 3.00)	0.99 (0.42, 2.33)	1.43 (0.54, 3.93)	0.92 (0.21, 3.92)	0.53 (0.08, 2.84)	1.75 (0.46, 7.41)	0.68 (0.13, 3.07)	1.25 (0.56, 2.71)	2.09 (0.55, 7.95)	2.03 (0.74, 5.44)	1.25 (0.43, 4.11)
FON4mg/kg0.1mgIV/SC	0.51 (0.09, 2.33)	1.62 (0.51, 5.10)	1.03 (0.46, 2.09)	0.77 (0.25, 2.07)	1.11 (0.50, 2.24)	0.82 (0.34, 1.74)	1.16 (0.44, 2.92)	0.75 (0.17, 3.08)	0.44 (0.07, 2.35)	1.39 (0.36, 5.87)	0.54 (0.11, 2.47)	1.02 (0.44, 2.07)	1.69 (0.47, 6.37)	1.66 (0.62, 4.33)	1.01 (0.35, 3.14)
FON4mg/kg1mgIV/SC	0.62 (0.11, 2.91)	1.98 (0.61, 6.33)	1.22 (0.53, 2.78)	0.92 (0.29, 2.61)	1.33 (0.60, 2.96)	0.96 (0.41, 2.27)	1.40 (0.53, 3.87)	0.90 (0.20, 3.82)	0.53 (0.08, 2.86)	1.72 (0.44, 7.26)	0.66 (0.13, 2.98)	1.21 (0.53, 2.72)	2.08 (0.54, 8.00)	1.97 (0.73, 5.55)	1.23 (0.41, 3.93)
GUS1200mgIV	0.32 (0.05, 1.39)	1.00 (0.33, 2.95)	0.63 (0.30, 1.18)	0.46 (0.17, 1.28)	0.68 (0.33, 1.28)	0.49 (0.23, 1.03)	0.71 (0.28, 1.76)	0.45 (0.11, 2.11)	0.27 (0.04, 1.41)	0.87 (0.23, 3.81)	0.33 (0.07, 1.57)	0.62 (0.30, 1.19)	1.03 (0.28, 4.00)	1.00 (0.66, 1.55)	0.62 (0.22, 1.74)
GUS200mgIV	0.26 (0.04, 1.09)	0.79 (0.26, 2.39)	**0.50 (0.24, 0.95)**	**0.37 (0.14, 0.97)**	0.55 (0.27, 1.02)	**0.40 (0.18, 0.80)**	0.57 (0.22, 1.41)	0.36 (0.08, 1.67)	0.21 (0.03, 1.15)	0.68 (0.18, 3.15)	0.27 (0.05, 1.23)	**0.50 (0.24, 0.92)**	0.81 (0.23, 3.25)	0.80 (0.54, 1.19)	0.50 (0.18, 1.46)
GUS600mgIV	0.27 (0.04, 1.13)	0.82 (0.26, 2.47)	**0.52 (0.26, 0.94)**	0.38 (0.15, 1.01)	0.56 (0.28, 1.04)	**0.41 (0.19, 0.83)**	0.58 (0.23, 1.44)	0.37 (0.09, 1.70)	0.22 (0.03, 1.15)	0.71 (0.19, 3.10)	0.27 (0.05, 1.27)	**0.51 (0.25, 0.95)**	0.85 (0.23, 3.20)	0.83 (0.55, 1.23)	0.51 (0.19, 1.48)
MED700IV	0.56 (0.10, 2.92)	1.80 (0.51, 6.75)	1.11 (0.44, 2.77)	0.82 (0.27, 2.58)	1.20 (0.49, 2.97)	0.88 (0.33, 2.28)	1.28 (0.43, 3.99)	0.82 (0.16, 4.18)	0.48 (0.06, 3.00)	1.55 (0.35, 7.63)	0.61 (0.11, 3.11)	1.09 (0.43, 2.74)	1.85 (0.41, 8.30)	1.79 (0.59, 5.20)	1.11 (0.34, 3.66)
MIR1000mgIV	0.31 (0.05, 1.65)	0.97 (0.27, 3.75)	0.63 (0.22, 1.52)	0.46 (0.13, 1.40)	0.69 (0.24, 1.61)	0.49 (0.18, 1.27)	0.72 (0.20, 2.09)	0.45 (0.09, 2.62)	0.26 (0.04, 2.01)	0.83 (0.21, 4.78)	0.33 (0.06, 1.92)	0.62 (0.22, 1.48)	0.99 (0.25, 4.94)	1.00 (0.29, 3.26)	0.62 (0.17, 2.23)
MIR200mgIV	0.54 (0.08, 3.17)	1.76 (0.39, 7.64)	1.08 (0.33, 3.57)	0.81 (0.20, 3.20)	1.18 (0.36, 3.81)	0.86 (0.26, 2.89)	1.23 (0.31, 5.14)	0.78 (0.14, 4.93)	0.45 (0.07, 3.68)	1.50 (0.32, 9.36)	0.56 (0.09, 3.52)	1.07 (0.33, 3.58)	1.74 (0.39, 10.09)	1.72 (0.44, 6.95)	1.10 (0.27, 4.68)
MIR600mgIV	0.20 (0.03, 1.02)	0.64 (0.17, 2.45)	0.41 (0.14, 1.05)	**0.30 (0.09, 0.97)**	0.44 (0.15, 1.12)	**0.32 (0.11, 0.84)**	0.46 (0.13, 1.46)	0.29 (0.06, 1.77)	0.16 (0.03, 1.24)	0.53 (0.13, 3.05)	0.22 (0.04, 1.22)	**0.40 (0.13, 0.98)**	0.64 (0.16, 3.23)	0.64 (0.18, 2.10)	0.40 (0.12, 1.42)
NAT300mgIV	0.65 (0.12, 2.62)	2.02 (0.82, 5.41)	1.27 (0.93, 1.74)	0.96 (0.42, 2.03)	**1.38 (1.02, 1.88)**	1.00 (0.66, 1.56)	1.44 (0.76, 2.97)	0.94 (0.25, 3.79)	0.54 (0.09, 2.64)	1.75 (0.57, 7.20)	0.69 (0.16, 2.87)	1.26 (0.91, 1.74)	2.10 (0.70, 7.34)	**2.03 (1.05, 4.25)**	1.27 (0.58, 3.25)
NAT3mg/kgIV	0.87 (0.15, 3.73)	2.78 (0.94, 8.08)	1.72 (0.90, 3.19)	1.28 (0.50, 3.44)	1.87 (0.98, 3.45)	1.37 (0.68, 2.70)	1.95 (0.80, 4.71)	1.27 (0.30, 5.70)	0.74 (0.11, 3.86)	2.36 (0.64, 10.66)	0.93 (0.18, 4.47)	1.69 (0.89, 3.17)	2.81 (0.81, 10.84)	**2.74 (1.13, 6.83)**	1.72 (0.64, 4.78)
NAT3mg/kgIVx2	0.57 (0.10, 2.44)	1.80 (0.63, 5.16)	1.11 (0.62, 1.96)	0.83 (0.33, 2.06)	1.21 (0.68, 2.12)	0.88 (0.46, 1.65)	1.25 (0.54, 2.94)	0.81 (0.19, 3.50)	0.49 (0.07, 2.49)	1.52 (0.44, 6.83)	0.60 (0.12, 2.75)	1.09 (0.61, 1.95)	1.81 (0.54, 6.72)	1.78 (0.79, 4.13)	1.12 (0.43, 3.09)
NAT6mg/kgIVx2	0.63 (0.11, 2.70)	1.99 (0.68, 5.79)	1.22 (0.66, 2.22)	0.90 (0.35, 2.36)	1.31 (0.72, 2.39)	0.96 (0.50, 1.87)	1.37 (0.61, 3.26)	0.89 (0.21, 4.06)	0.53 (0.08, 2.85)	1.67 (0.46, 7.87)	0.65 (0.13, 3.01)	1.20 (0.67, 2.20)	2.01 (0.58, 7.60)	1.96 (0.83, 4.58)	1.20 (0.47, 3.39)
NNC2mg/kgSC	0.54 (0.09, 2.97)	1.74 (0.45, 6.70)	1.10 (0.39, 2.84)	0.81 (0.25, 2.53)	1.20 (0.41, 3.04)	0.86 (0.30, 2.39)	1.26 (0.39, 4.15)	0.78 (0.17, 4.14)	0.45 (0.06, 3.06)	1.51 (0.36, 8.04)	0.57 (0.11, 3.33)	1.08 (0.38, 2.81)	1.80 (0.44, 8.16)	1.77 (0.50, 5.59)	1.10 (0.34, 3.97)
ONE10mgSC	0.69 (0.12, 3.62)	2.21 (0.69, 7.86)	1.39 (0.61, 3.04)	1.04 (0.34, 3.11)	1.51 (0.67, 3.27)	1.11 (0.47, 2.52)	1.55 (0.57, 4.47)	1.00 (0.22, 4.70)	0.59 (0.09, 3.80)	1.94 (0.49, 9.70)	0.74 (0.14, 3.82)	1.39 (0.59, 3.04)	2.25 (0.62, 9.58)	2.21 (0.80, 6.17)	1.39 (0.46, 4.47)
ONE25mgSC	1.49 (0.25, 7.75)	**4.68 (1.32, 18.51)**	**2.90 (1.11, 8.11)**	2.23 (0.61, 7.55)	**3.14 (1.23, 8.94)**	2.29 (0.85, 6.70)	**3.30 (1.08, 11.40)**	2.23 (0.41, 11.39)	1.26 (0.17, 8.92)	4.12 (0.94, 22.79)	1.56 (0.28, 8.71)	**2.89 (1.11, 8.26)**	**4.78 (1.19, 24.62)**	**4.71 (1.57, 15.16)**	2.89 (0.90, 11.34)
ONE35mgSC	0.70 (0.12, 3.55)	2.25 (0.66, 7.69)	1.40 (0.60, 3.31)	1.04 (0.34, 3.14)	1.50 (0.64, 3.54)	1.10 (0.46, 2.68)	1.56 (0.57, 4.67)	1.01 (0.21, 4.91)	0.59 (0.08, 3.72)	1.90 (0.48, 9.99)	0.75 (0.14, 3.80)	1.38 (0.60, 3.21)	2.23 (0.58, 9.77)	2.21 (0.80, 6.33)	1.40 (0.46, 4.64)
ONE50mgSC	1.01 (0.17, 5.16)	3.18 (0.93, 11.55)	2.00 (0.84, 5.13)	1.49 (0.47, 4.69)	2.17 (0.90, 5.61)	1.57 (0.63, 4.13)	2.26 (0.80, 7.18)	1.46 (0.31, 7.74)	0.86 (0.11, 5.43)	2.71 (0.67, 15.02)	1.07 (0.19, 5.78)	1.96 (0.83, 5.02)	3.22 (0.86, 15.32)	**3.18 (1.13, 9.92)**	2.00 (0.63, 7.24)
ONT22.5mgSC	0.81 (0.14, 3.68)	2.62 (0.79, 7.96)	1.57 (0.76, 3.34)	1.16 (0.43, 3.38)	1.70 (0.83, 3.62)	1.25 (0.58, 2.76)	1.77 (0.73, 4.78)	1.18 (0.24, 5.42)	0.69 (0.09, 3.92)	2.21 (0.57, 10.98)	0.85 (0.16, 4.15)	1.55 (0.76, 3.30)	2.61 (0.70, 10.85)	2.55 (0.98, 7.20)	1.57 (0.56, 5.18)
ONT225mgSC	0.75 (0.13, 3.22)	2.29 (0.74, 7.08)	1.45 (0.72, 2.93)	1.09 (0.39, 2.87)	1.58 (0.80, 3.20)	1.15 (0.54, 2.55)	1.64 (0.67, 4.32)	1.08 (0.22, 5.09)	0.63 (0.09, 3.50)	1.98 (0.55, 10.38)	0.78 (0.15, 3.63)	1.45 (0.71, 2.87)	2.40 (0.68, 9.14)	2.31 (0.95, 6.22)	1.47 (0.52, 4.37)
ONT75mgSC	0.74 (0.13, 3.43)	2.35 (0.76, 7.36)	1.46 (0.73, 3.07)	1.10 (0.39, 3.06)	1.59 (0.80, 3.36)	1.16 (0.54, 2.58)	1.65 (0.70, 4.48)	1.10 (0.24, 5.06)	0.64 (0.09, 3.62)	2.04 (0.55, 9.14)	0.80 (0.15, 3.80)	1.45 (0.72, 3.02)	2.45 (0.67, 9.67)	2.33 (0.96, 6.39)	1.47 (0.52, 4.47)
PBO	0.91 (0.16, 3.62)	**2.82 (1.17, 7.46)**	**1.77 (1.44, 2.20)**	1.33 (0.60, 2.72)	**1.92 (1.58, 2.38)**	1.40 (0.98, 2.06)	**1.99 (1.09, 4.01)**	1.29 (0.34, 5.21)	0.76 (0.12, 3.72)	2.41 (0.81, 9.66)	0.96 (0.22, 4.08)	**1.75 (1.39, 2.23)**	2.90 (0.98, 9.94)	**2.84 (1.49, 5.62)**	1.75 (0.83, 4.39)

	PF200mgSC	PF50mgSC	RIS1200mgIV	RIS200mgIV	RIS600mgIV	RIS600mgIV4/4	TES400mg200mg SC	UPA12mgOR_BID	UPA24mgOR	UPA24mgOR_BID	UPA3mgOR_BID	UPA45mgOR	UPA6mgOR_BID	UST6mg/kg90mgI V/SC	VED300mgIV
PF10mgSC	0.99 (0.18, 4.48)	**3.07 (1.21, 9.68)**	1.93 (0.61, 7.61)	1.45 (0.35, 6.83)	2.10 (0.67, 8.15)	1.51 (0.46, 6.25)	2.17 (0.60, 9.47)	1.45 (0.26, 7.78)	0.85 (0.10, 5.34)	2.70 (0.55, 15.51)	1.04 (0.16, 5.89)	1.92 (0.60, 7.46)	3.26 (0.66, 16.09)	3.07 (0.84, 14.37)	1.99 (0.47, 9.42)
PF200mgSC	PF200mgSC	**3.05 (1.04, 14.27)**	1.97 (0.49, 10.62)	1.52 (0.33, 8.19)	2.13 (0.53, 11.27)	1.57 (0.38, 8.39)	2.26 (0.52, 13.18)	1.46 (0.25, 10.94)	0.86 (0.11, 8.12)	2.71 (0.50, 19.77)	1.09 (0.15, 7.43)	1.94 (0.48, 10.53)	3.35 (0.59, 21.08)	3.15 (0.73, 19.14)	2.03 (0.38, 12.79)
PF50mgSC	**0.33 (0.07, 0.96)**	PF50mgSC	0.62 (0.24, 1.57)	0.47 (0.14, 1.51)	0.68 (0.26, 1.71)	0.50 (0.18, 1.27)	0.71 (0.23, 2.12)	0.45 (0.10, 2.15)	0.27 (0.04, 1.52)	0.85 (0.21, 4.06)	0.33 (0.06, 1.75)	0.62 (0.23, 1.53)	1.04 (0.25, 4.20)	1.01 (0.33, 3.21)	0.63 (0.18, 2.18)
RIS1200mgIV	0.51 (0.09, 2.02)	1.61 (0.64, 4.17)	RIS1200mgIV	0.75 (0.34, 1.52)	1.08 (0.93, 1.28)	0.79 (0.51, 1.23)	1.12 (0.59, 2.32)	0.73 (0.19, 3.06)	0.43 (0.06, 2.10)	1.36 (0.45, 5.56)	0.54 (0.12, 2.24)	0.99 (0.72, 1.36)	1.63 (0.54, 5.78)	1.60 (0.83, 3.28)	0.99 (0.45, 2.48)
RIS200mgIV	0.66 (0.12, 3.05)	2.14 (0.66, 7.13)	1.33 (0.66, 2.93)	RIS200mgIV	1.45 (0.72, 3.16)	1.06 (0.48, 2.57)	1.51 (0.61, 4.34)	0.97 (0.22, 4.99)	0.58 (0.08, 3.34)	1.83 (0.52, 9.38)	0.71 (0.14, 3.50)	1.31 (0.62, 3.09)	2.17 (0.63, 8.76)	2.19 (0.77, 5.75)	1.35 (0.47, 4.27)
RIS600mgIV	0.47 (0.09, 1.88)	1.47 (0.58, 3.85)	0.92 (0.78, 1.08)	0.69 (0.32, 1.39)	RIS600mgIV	0.73 (0.48, 1.13)	1.04 (0.55, 2.17)	0.67 (0.18, 2.78)	0.39 (0.06, 1.90)	1.25 (0.41, 5.14)	0.50 (0.11, 2.04)	0.91 (0.66, 1.24)	1.51 (0.50, 5.30)	1.47 (0.76, 3.02)	0.91 (0.42, 2.28)
RIS600mgIV4/4	0.64 (0.12, 2.64)	2.01 (0.79, 5.62)	1.27 (0.81, 1.94)	0.95 (0.39, 2.10)	1.37 (0.89, 2.09)	RIS600mgIV4/4	1.42 (0.69, 3.13)	0.92 (0.23, 3.83)	0.54 (0.08, 2.66)	1.72 (0.55, 6.91)	0.68 (0.15, 2.95)	1.25 (0.80, 1.91)	2.05 (0.66, 7.44)	2.05 (0.98, 4.37)	1.26 (0.53, 3.32)
TES400mg200mg SC	0.44 (0.08, 1.91)	1.41 (0.47, 4.28)	0.89 (0.43, 1.68)	0.66 (0.23, 1.64)	0.96 (0.46, 1.81)	0.70 (0.32, 1.45)	TES400mg200mg SC	0.63 (0.14, 2.93)	0.38 (0.05, 2.06)	1.21 (0.32, 5.70)	0.47 (0.09, 2.20)	0.88 (0.42, 1.69)	1.42 (0.42, 5.63)	1.41 (0.57, 3.61)	0.88 (0.34, 2.52)
UPA12mgPO_BID	0.68 (0.09, 3.95)	2.22 (0.47, 9.70)	1.38 (0.33, 5.38)	1.03 (0.20, 4.48)	1.49 (0.36, 5.68)	1.08 (0.26, 4.26)	1.59 (0.34, 7.01)	UPA12mgPO_BID	0.59 (0.10, 2.30)	1.90 (0.69, 6.03)	0.71 (0.18, 2.80)	1.35 (0.33, 5.26)	2.20 (0.85, 7.25)	2.24 (0.49, 9.66)	1.40 (0.27, 6.43)
UPA24mgPO	1.17 (0.12, 8.90)	3.71 (0.66, 25.86)	2.32 (0.48, 15.78)	1.74 (0.30, 12.41)	2.54 (0.53, 17.00)	1.85 (0.38, 11.97)	2.62 (0.49, 18.58)	1.70 (0.43, 9.55)	UPA24mgPO	**3.18 (1.03, 16.00)**	1.23 (0.31, 6.43)	2.30 (0.46, 15.13)	**3.79 (1.19, 20.59)**	3.72 (0.70, 26.03)	2.40 (0.36, 18.20)
UPA24mgPO_BID	0.37 (0.05, 2.00)	1.17 (0.25, 4.68)	0.74 (0.18, 2.22)	0.55 (0.11, 1.91)	0.80 (0.19, 2.41)	0.58 (0.14, 1.80)	0.83 (0.18, 3.09)	0.53 (0.17, 1.45)	**0.31 (0.06, 0.97)**	UPA24mgPO_BID	0.38 (0.11, 1.12)	0.72 (0.18, 2.26)	1.16 (0.53, 2.67)	1.17 (0.26, 4.34)	0.74 (0.15, 2.98)
UPA3mgPO_BID	0.92 (0.13, 6.51)	3.03 (0.57, 16.57)	1.85 (0.45, 8.59)	1.40 (0.29, 7.21)	2.00 (0.49, 9.34)	1.46 (0.34, 6.86)	2.11 (0.46, 11.55)	1.41 (0.36, 5.63)	0.81 (0.16, 3.19)	2.62 (0.89, 8.76)	UPA3mgPO_BID	1.83 (0.44, 8.10)	**3.04 (1.13, 10.59)**	2.99 (0.64, 15.41)	1.86 (0.37, 10.54)
UPA45mgPO	0.52 (0.09, 2.10)	1.61 (0.65, 4.31)	1.01 (0.73, 1.38)	0.76 (0.32, 1.61)	1.10 (0.80, 1.51)	0.80 (0.52, 1.25)	1.14 (0.59, 2.37)	0.74 (0.19, 3.00)	0.44 (0.07, 2.17)	1.39 (0.44, 5.54)	0.55 (0.12, 2.28)	UPA45mgPO	1.66 (0.55, 5.78)	1.63 (0.83, 3.32)	1.00 (0.45, 2.59)
UPA6mgPO_BID	0.30 (0.05, 1.69)	0.96 (0.24, 3.99)	0.62 (0.17, 1.85)	0.46 (0.11, 1.58)	0.66 (0.19, 1.99)	0.49 (0.13, 1.51)	0.70 (0.18, 2.38)	0.46 (0.14, 1.18)	**0.26 (0.05, 0.84)**	0.86 (0.38, 1.89)	**0.33 (0.09, 0.88)**	0.60 (0.17, 1.81)	UPA6mgPO_BID	0.99 (0.25, 3.55)	0.62 (0.14, 2.41)
UST6mg/kg90mgI V/SC	0.32 (0.05, 1.37)	0.99 (0.31, 3.06)	0.62 (0.30, 1.21)	0.46 (0.17, 1.30)	0.68 (0.33, 1.32)	0.49 (0.23, 1.02)	0.71 (0.28, 1.75)	0.45 (0.10, 2.04)	0.27 (0.04, 1.42)	0.85 (0.23, 3.87)	0.33 (0.06, 1.56)	0.61 (0.30, 1.20)	1.01 (0.28, 3.99)	UST6mg/kg90mgI V/SC	0.62 (0.22, 1.85)
VED300mgIV	0.49 (0.08, 2.63)	1.60 (0.46, 5.46)	1.01 (0.40, 2.21)	0.74 (0.23, 2.12)	1.10 (0.44, 2.41)	0.80 (0.30, 1.88)	1.14 (0.40, 2.98)	0.72 (0.16, 3.71)	0.42 (0.05, 2.74)	1.35 (0.34, 6.64)	0.54 (0.09, 2.71)	1.00 (0.39, 2.20)	1.60 (0.41, 7.37)	1.61 (0.54, 4.56)	VED300mgIV

TABLE S13: SUCRA PROBABILITYS FOR CDAI OUTCOMES. Higher probabilities are more closely associated with the chance of the drug treatment having the highest occurrence of the event in relation to the other drugs included in the network. Higher probabilities indicate better-performing interventions. For better visualization access the excel version using the LINK

	% SUCRA remission	% SUCRA remission in the naive population	% SUCRA remission in the prior population	% SUCRA remission in 4 weeks	% SUCRA remission in 6 weeks	% SUCRA remission in 8 weeks	% SUCRA remission in 12 weeks	%SUCRA remission approved	% SUCRA response with 100 points degree	% SUCRA response with 70 points degree
ABA10mg/kgIV	15.9	-	-	-	-	14.5	15.9	12.9	14.5	-
ABA30mg/kgIV	41.4	-	-	-	-	36.9	40.1	34.2	36.9	-
ABA3mg/kgIV	22.4	-	-	-	-	19.3	19.8	17.0	19.3	-
ADA160mg80mg40mgSC	81.3	-	-	-	-	-	-	74.3	-	-
ADA160mg80mg60mgSC	95.1	76.5	-	93.0	-	-	-	92.9	-	91.2
ADA160mg80mgSC	82.7	56.8	68.6	77.7	-	-	-	77.4	-	70.3
ADA40mg20mgSC	49.7	35.5	-	46.2	-	-	-	44.9	-	63.9
ADA80mg40mgSC	76.2	52.5	87.9	72.1	-	-	-	70.2	-	75.3
AMI0.4mgPO	17.0	-	-	-	-	-	15.7	-	-	16.0
AND150mgSC1/1	25.6	-	-	-	-	23.2	-	-	23.2	-
AND150mgSC2/2	34.2	-	-	-	-	30.2	-	-	30.2	-
AND300mgSC	13.6	-	-	-	-	12.3	-	-	12.3	-
API100mgPO	20.0	-	-	18.6	14.7	-	-	-	-	-
API50mgPO	11.5	-	-	15.7	7.4	-	-	-	-	-
BRI400mgIV	80.0	32.5	53.0	-	53.8	-	81.1	-	-	-
BRI700mgIV	70.9	-	64.3	-	69.6	-	72.9	-	-	-
BRO210mgIV	16.5	-	-	-	38.4	-	-	14.4	-	-
BRO350mgIV	72.5	-	-	-	90.0	-	-	65.7	-	-
BRO700mgIV	52.9	-	-	-	75.4	-	-	47.9	-	-
CDP10mg/kgIV	31.1	-	-	40.0	-	20.5	30.4	-	20.5	40.2
CER100mgSC	37.5	-	-	63.5	46.8	70.6	36.1	30.8	70.6	-
CER10mg/kgIV	20.7	-	-	68.2	-	31.6	19.5	16.8	31.6	-
CER200mgSC	22.0	-	-	55.1	24.4	52.2	20.0	17.4	52.2	-
CER20mg/kgIV	7.7	-	-	47.5	-	21.1	5.8	5.8	21.1	-
CER400mgSC	41.1	31.9	-	52.6	42.0	74.0	38.0	32.9	74.0	84.9
CER5mg/kgIV	32.9	-	-	61.3		34.0	32.1	27.0	34.0	-
ELD10mg/kgIV	38.1	-	-	-	-	-	39.9	-	-	-
ELD20mg/kgIV	52.3	-	-	-	-	-	54.1	-	-	-
ETA25mgSC	14.2	-	-	11.1	-	13.2	-	11.7	13.2	36.4
ETR105mgSC	27.8	-	-	-		-		-	-	-
ETR210mgSC	35.0	-	-	-		-		-	-	-
FIL100mgPO	64.8	-	-	-		-		30.2	-	-
FIL200mgPO	53.4	75.5	30.0	-	-	-	-	59.2	-	-
FON0.1mg/kgIV	15.4	-	-	14.4	-	-	-	-	-	20.2

	% SUCRA remission	% SUCRA remission in the naive population	% SUCRA remission in the prior population	% SUCRA remission in 4 weeks	% SUCRA remission in 6 weeks	% SUCRA remission in 8 weeks	% SUCRA remission in 12 weeks	%SUCRA remission approved	% SUCRA response with 100 points degree	% SUCRA response with 70 points degree
FON1mg/kg0.1mgIV/SC	36.8	-	-	30.2	39.7	50.9	37.7	-	50.9	-
FON1mg/kg1mgIV/SC	49.6	-	-	36.6	63.4	71.7	51.4	-	71.7	-
FON1mg/kgIV	29.0	-	-	27.7	-	-	-	-	-	41.4
FON4mg/kg0.1mgIV/SC	60.8	-	-	36.8	64.6	80.3	63.5	-	80.3	-
FON4mg/kg1mgIV/SC	50.2	-	-	4.8	47.1	49.7	52.9	-	49.7	-
FON4mg/kgIV	57.0	-	-	51.9	-	-	-	-	-	52.5
GUS1200mgIV	83.2	-	75.5	53.9	-	66.6	84.0	76.2	66.6	-
GUS200mgIV	90.1	-	93.1	53.8	-	73.4	91.5	85.4	73.4	-
GUS600mgIV	89.2	-	87.7	56.7	-	77.2	90.6	84.5	77.2	-
INF10mg/kgIV	92.0	77.8	-	90.1	-	-	-	90.8	-	85.3
INF20mg/kgIV	91.8	77.9	-	90.1	-	-	-	91.1	-	93.3
INF5mg/kgIV	98.6	89.5	-	98.0	-	-	-	98.3	-	98.2
MED700IV	54.9	-	36.7	-	-	56.5	55.2	-	56.5	-
MIR1000mgIV	82.4	83.7	37.1	-	-	-	82.7	75.9	-	-
MIR200mgIV	55.7	73.1	12.7	-	-	-	56.1	50.4	-	-
MIR600mgIV	91.5	78.1	74.1	-	-	-	93.6	88.1	-	-
NAT300mgIV	48.7	-	-	40.9	48.3	38.8	50.1	40.1	38.8	47.6
NAT3mg/kgIV	33.4	26.0	-	66.1	30.6	53.6	31.8	26.1	53.6	40.0
NAT3mg/kgIVx2	57.1	41.0	-	62.9	57.4	73.4	58.4	48.0	73.4	58.8
NAT6mg/kgIVx2	52.2	37.6	-	63.3	34.1	75.4	52.9	42.5	75.4	64.3
NNC2mg/kgSC	57.8	49.3	-	34.6	-	50.4	58.5	-	50.4	-
ONE10mgSC	43.9	34.6	-	-	-	-	43.3	-	-	48.9
ONE25mgSC	14.6	13.3	-	-	-	-	12.5	-	-	10.6
ONE35mgSC	45.1	35.1	-	-	-	-	44.9	-	-	49.1
ONE50mgSC	27.6	22.6	-	-	-	-	25.4	-	-	25.1
ONT22.5mgSC	39.0	-	-	55.0	-	-	38.4	-	-	33.6
ONT225mgSC	43.4	-	-	50.6	-	-	43.7	-	-	27.8
ONT75mgSC	40.0	-	-	41.5	-	-	40.8	-	-	33.4
PBO	29.4	21.5	15.1	23.8	22.2	24.9	28.5	22.9	24.9	25.4
PF10mgSC	31.9	-	18.2	-	-	15.8	31.9	-	15.8	47.2
PF200mgSC	32.5	-	20.6	-	-	7.2	34.3	-	7.2	58.3
PF50mgSC	79.7	-	64.7	-	-	44.9	82.5	-	44.9	73.3
RIS1200mgIV	64.1	50.9	43.7	58.8	-	-	65.2	54.8		-
RIS200mgIV	47.6	52.5	30.0	29.9	-	81.3	47.5	39.5	81.3	-
RIS600mgIV	69.5	-	50.4	60.7	-	91.2	70.8	60.9	91.2	-
RIS600mgIV4/4	49.1	-	-	-	-	-	50.3	40.5	-	-
SEC10mg/kgIV	26.2	-	-	16.8	21.7	-	-	21.5	-	-
SEM60mgIV	-	-	-	-	-	-	-	-	-	20.3
SEM60mgIVx3	-	-	-	-	-	-	-	-	-	31.4
TES400mg200mgSC	69.7	-	50.6	79.1	86.9	68.8	70.5	-	68.8	-
TOF15mgPO_BID	19.9	-	-	18.1	-	-	-	17.2	-	27.3
TOF1mgPO_BID	54.4	-	-	47.9	-	-	-	47.3	-	14.2
TOF5mgPO_BID	40.7	-	-	35.5	-	-	-	35.0	-	42.6
UPA12mgPO_BID	41.8	-	-	-	-	-	50.0	42.8	-	47.6
UPA24mgPO	24.0	-	-	-	-	-	28.5	23.8	-	54.5

	% SUCRA remission	% SUCRA remission in the naive population	% SUCRA remission in the prior population	% SUCRA remission in 4 weeks	% SUCRA remission in 6 weeks	% SUCRA remission in 8 weeks	% SUCRA remission in 12 weeks	%SUCRA remission approved	% SUCRA response with 100 points degree	% SUCRA response with 70 points degree
UPA24mgPO_BID	69.7	-	-	-	-	-	77.1	69.4	-	73.5
UPA3mgPO_BID	29.2	-	-	-	-	-	35.3	30.1	-	50.8
UPA45mgPO	63.1	-	41.3	42.6	-	-	65.1	54.3	-	-
UPA6mgPO_BID	77.0	-	-	-	-	-	83.9	76.9	-	64.0
UST130mgIV	60.9	-	47.0	35.4	56.8	50.8	-	51.3	50.8	55.8
UST1mg/kgIV	67.2	-	50.8	65.6	68.5	57.7	-	59.5	57.7	-
UST3mg/kgIV	69.4	-	54.6	-	68.2	59.7	-	62.8	59.7	-
UST4.5mg/kgIV	61.6	-	10.2	57.2	67.2	85.7	-	70.8	85.7	68.5
UST6mg/kg90mgIV/SC	83.3	-	81.0	75.2	-	81.8	83.9	76.8	81.8	-
UST6mg/kgIV	75.7	-	61.6	42.7	67.0	66.2	-	68.1	66.2	68.5
UST90mgSC	37.0	-	26.7	52.0	39.3	31.3	-	29.0	31.3	28.8
VED0.5mg/kgIV	50.3	37.0	62.8	56.0	53.8	41.7	-	41.7	41.7	41.1
VED2mg/kgIV	63.2	46.8	-	69.9	53.7	54.4	-	56.2	54.4	48.7
VED300mgIV	73.3	40.5	-	-	47.0	64.8	63.0	65.9	64.8	-

TABLE S14. LEAGUE TABLE FOR IBDQ OUTCOMES. Each table presents the results for main network (increase in the IBDQ score) and sensitive analysis for IBDQ remission and response. The table presented the multiple treatment comparisons based on consistency analysis of the networks. Treatments are depicted alphabetically. The upper quadrant is the inverse of the lower quadrant, i.e., the mirror image of the results. Values are presented as mean difference (MD) with 95% credible interval (CrI). For all comparisons an MD <0 favors the occurrence of the event for the row-defining treatment, while an MD>0 favors the column-defining treatment. Statistically significant results are represented in bold. For better visualization access the excel version using the LINK

Table S14a: League table for increase in the IBDQ score.

CER400mgSC	CER400mgSC	3.86 (-24.82, 32.97)	-11.91 (-39.84, 16.88)	-3.44 (-33.96, 25.12)	-8.96 (-36.61, 20.39)	-6.68 (-34.66, 24.66)	12.66 (-16.34, 43.08)	13.38 (-15.63, 42.90)	28.68 (-0.73, 57.43)	-0.75 (-26.36, 24.67)	-12.18 (-32.06, 6.90)	2.47 (-27.10, 29.75)	15.38 (-13.95, 42.21)	-14.33 (-43.14, 14.66)	-11.40 (-38.88, 16.61)	5.40 (-23.69, 33.72)	-4.43 (-31.63, 25.02)	17.71 (-10.78, 47.73)	-2.17 (-31.53, 27.14)	10.87 (-12.37, 34.19)	14.76 (-16.54, 46.28)	-2.42 (-25.05, 19.05)	-1.61 (-24.25, 20.94)
FIL200mgPO	-3.86 (-32.97, 24.82)	FIL200mgPO	-15.81 (-43.50, 14.07)	-7.64 (-35.78, 22.09)	-12.72 (-41.99, 16.98)	-10.29 (-40.09, 18.52)	8.99 (-22.84, 40.74)	9.36 (-22.55, 40.89)	24.79 (-5.60, 54.82)	-4.47 (-31.99, 20.89)	-16.18 (-35.69, 4.53)	-1.62 (-31.63, 26.24)	11.49 (-20.46, 39.87)	-17.80 (-46.77, 9.62)	-15.01 (-43.45, 13.67)	1.72 (-29.23, 31.73)	-8.28 (-37.34, 21.07)	14.16 (-15.82, 44.09)	-6.22 (-37.29, 22.71)	6.96 (-17.72, 30.65)	10.75 (-20.66, 42.00)	-6.18 (-30.12, 17.85)	-5.56 (-29.36, 18.32)
FON1mg/kg 0.1mgIV/SC	11.91 (-16.88, 39.84)	15.81 (-14.07, 43.50)	FON1mg/kg 0.1mgIV/SC	8.43 (-13.60, 30.67)	2.86 (-18.66, 24.77)	5.24 (-18.58, 28.46)	24.91 (-8.24, 56.18)	25.36 (-6.72, 55.59)	**40.87 (7.36, 71.41)**	10.82 (-17.87, 38.76)	-0.35 (-22.50, 20.43)	13.88 (-16.40, 43.13)	26.95 (-4.98, 57.24)	-1.97 (-32.64, 27.47)	0.64 (-27.74, 29.21)	17.18 (-13.63, 47.96)	7.51 (-22.22, 37.13)	29.74 (-0.72, 59.87)	9.50 (-21.13, 39.59)	22.71 (-2.77, 45.94)	26.31 (-5.09, 58.79)	9.30 (-15.54, 32.91)	10.20 (-14.58, 33.82)
FON1mg/kg 1mgIV/SC	3.44 (-25.12, 33.96)	7.64 (-22.09, 35.78)	-8.43 (-30.67, 13.60)	FON1mg/kg1mgIV/SC	-5.55 (-25.66, 17.83)	-3.16 (-24.53, 20.10)	16.63 (-13.29, 47.93)	17.30 (-14.86, 48.70)	**32.53 (1.60, 63.94)**	2.81 (-23.65, 30.76)	-8.62 (-29.77, 13.18)	6.00 (-25.29, 35.89)	19.03 (-13.54, 48.80)	-10.36 (-38.97, 20.27)	-7.91 (-35.07, 22.08)	8.83 (-22.81, 40.71)	-0.78 (-30.35, 28.58)	21.39 (-8.41, 54.68)	1.50 (-29.19, 32.36)	14.45 (-9.44, 40.69)	17.99 (-13.74, 53.07)	1.25 (-23.56, 26.50)	1.98 (-21.83, 25.41)
FON4mg/kg 0.1mgIV/SC	8.96 (-20.39, 36.61)	12.72 (-16.98, 41.99)	-2.86 (-24.77, 18.66)	5.55 (-17.83, 25.66)	FON4mg/kg 0.1mgIV/SC	2.32 (-20.29, 24.24)	21.97 (-10.52, 52.05)	22.44 (-9.47, 53.07)	**37.43 (6.91, 67.10)**	8.20 (-18.10, 32.97)	-3.14 (-25.94, 16.39)	11.02 (-19.81, 39.83)	24.01 (-7.85, 52.91)	-5.08 (-34.25, 23.30)	-2.40 (-31.37, 26.33)	14.16 (-17.10, 45.41)	4.68 (-24.09, 33.28)	26.57 (-3.01, 56.56)	6.57 (-24.24, 36.35)	20.04 (-6.37, 43.96)	23.41 (-8.39, 56.04)	6.35 (-18.96, 29.91)	7.26 (-17.57, 29.71)
FON4mg/kg 1mgIV/SC	6.68 (-24.66, 34.66)	10.29 (-18.52, 40.09)	-5.24 (-28.46, 18.58)	3.16 (-20.10, 24.53)	-2.32 (-24.24, 20.29)	FON4mg/kg1mgIV/SC	19.53 (-14.22, 51.78)	20.16 (-12.18, 52.16)	**35.48 (2.93, 66.62)**	5.86 (-22.85, 33.88)	-5.46 (-29.42, 16.50)	8.97 (-23.37, 39.67)	22.11 (-11.37, 52.32)	-7.55 (-38.51, 23.52)	-4.55 (-34.26, 24.91)	11.86 (-20.33, 43.83)	2.18 (-28.98, 32.73)	24.72 (-6.78, 56.34)	4.53 (-27.82, 36.43)	17.60 (-9.08, 42.72)	21.28 (-11.29, 55.08)	4.42 (-22.28, 28.23)	4.95 (-21.68, 29.72)
INF10mg/kgIV	-12.66 (-43.08, 16.34)	-8.99 (-40.74, 22.84)	-24.91 (-56.18, 8.24)	-16.63 (-47.93, 13.29)	-21.97 (-52.05, 10.52)	-19.53 (-51.78, 14.22)	INF10mg/kgIV	0.61 (-22.68, 23.03)	16.08 (-7.08, 39.07)	-13.56 (-42.64, 14.77)	**-24.91 (-48.22, -1.74)**	-10.61 (-43.31, 20.64)	2.39 (-30.64, 32.21)	-26.88 (-58.93, 4.16)	-24.17 (-52.93, 6.03)	-7.56 (-40.81, 24.16)	-16.68 (-48.95, 12.43)	4.90 (-27.01, 38.44)	-14.72 (-48.22, 17.80)	-1.78 (-27.90, 24.06)	2.17 (-32.74, 35.93)	-15.24 (-42.73, 10.91)	-14.44 (-40.68, 12.01)
INF20mg/kgIV	-13.38 (-42.90, 15.63)	-9.36 (-40.89, 22.55)	-25.36 (-55.59, 6.72)	-17.30 (-48.70, 14.86)	-22.44 (-53.07, 9.47)	-20.16 (-52.16, 12.18)	-0.61 (-23.03, 22.68)	INF20mg/kgIV	15.13 (-7.79, 38.01)	-14.21 (-41.48, 13.71)	**-25.54 (-48.17, -3.50)**	-11.17 (-42.62, 20.13)	1.76 (-29.53, 30.89)	-27.54 (-58.35, 3.26)	-24.87 (-54.14, 5.32)	-8.08 (-41.10, 21.56)	-17.53 (-47.75, 11.32)	4.37 (-26.09, 36.08)	-15.95 (-47.85, 17.48)	-2.75 (-27.47, 22.52)	1.75 (-33.56, 34.11)	-15.77 (-42.93, 9.34)	-15.34 (-41.27, 9.81)
INF5mg/kgIV	-28.68 (-57.43, 0.73)	-24.79 (-54.82, 5.60)	**-40.87 (-71.41, -7.36)**	**-32.53 (-63.94, -1.60)**	**-37.43 (-67.10, -6.91)**	**-35.48 (-66.62, -2.93)**	-16.08 (-39.07, 7.08)	-15.13 (-38.01, 7.79)	INF5mg/kgIV	-29.44 (-57.30, 0.21)	**-41.10 (-62.65, -18.45)**	-27.02 (-57.95, 3.94)	-13.69 (-44.67, 17.27)	**-43.05 (-71.88, -11.35)**	**-40.20 (-69.34, -8.57)**	-23.18 (-54.94, 9.57)	**-32.98 (-63.45, -2.71)**	-11.14 (-41.41, 20.14)	-30.90 (-62.68, 1.50)	-17.96 (-42.71, 7.82)	-13.67 (-48.03, 19.47)	**-31.28 (-55.98, -6.72)**	**-30.67 (-54.97, -5.60)**
NAT300mgIV	0.75 (-24.67, 26.36)	4.47 (-20.89, 31.99)	-10.82 (-38.76, 17.87)	-2.81 (-30.76, 23.65)	-8.20 (-32.97, 18.10)	-5.86 (-33.88, 22.85)	13.56 (-14.77, 42.64)	14.21 (-13.71, 41.48)	29.44 (-0.21, 57.30)	NAT300mgIV	-11.52 (-29.46, 6.02)	2.94 (-22.93, 29.06)	15.94 (-10.58, 41.74)	-13.24 (-40.96, 13.90)	-10.44 (-36.41, 15.89)	6.35 (-22.62, 33.63)	-3.41 (-30.43, 22.92)	18.58 (-8.67, 45.92)	-1.25 (-30.89, 25.47)	11.58 (-10.79, 32.89)	15.36 (-13.89, 44.15)	-1.59 (-23.03, 18.86)	-0.90 (-22.53, 19.46)
PBO	12.18 (-6.90, 32.06)	16.18 (-4.53, 35.69)	0.35 (-20.43, 22.50)	8.62 (-13.18, 29.77)	3.14 (-16.39, 25.94)	5.46 (-16.50, 29.42)	**24.91 (1.74, 48.22)**	**25.54 (3.50, 48.17)**	**41.10 (18.45, 62.65)**	11.52 (-6.02, 29.46)	PBO	14.45 (-7.27, 34.67)	**27.47 (4.97, 48.38)**	-1.93 (-22.24, 18.54)	0.92 (-18.93, 20.63)	17.50 (-4.79, 40.03)	7.90 (-11.66, 28.76)	**30.03 (8.65, 53.22)**	10.07 (-12.86, 32.96)	**23.09 (10.81, 36.38)**	**26.79 (3.00, 51.01)**	9.77 (-2.38, 21.36)	10.49 (-1.58, 21.70)
RIS200mgIV	-2.47 (-29.75, 27.10)	1.62 (-26.24, 31.63)	-13.88 (-43.13, 16.40)	-6.00 (-35.89, 25.29)	-11.02 (-39.83, 19.81)	-8.97 (-39.67, 23.37)	10.61 (-20.64, 43.31)	11.17 (-20.13, 42.62)	27.02 (-3.94, 57.95)	-2.94 (-29.06, 22.93)	-14.45 (-34.67, 7.27)	RIS200mgIV	13.06 (-7.53, 33.50)	-15.98 (-46.52, 12.91)	-13.45 (-41.12, 16.18)	3.28 (-26.97, 34.74)	-6.44 (-36.68, 24.17)	16.22 (-13.13, 46.16)	-4.19 (-34.50, 26.49)	8.74 (-13.92, 33.57)	12.55 (-18.53, 44.67)	-4.50 (-27.83, 20.05)	-4.08 (-28.39, 21.37)
RIS600mgIV	-15.38 (-42.21, 13.95)	-11.49 (-39.87, 20.46)	-26.95 (-57.24, 4.98)	-19.03 (-48.80, 13.54)	-24.01 (-52.91, 7.85)	-22.11 (-52.32, 11.37)	-2.39 (-32.21, 30.64)	-1.76 (-30.89, 29.53)	13.69 (-17.27, 44.67)	-15.94 (-41.74, 10.58)	**-27.47 (-48.38, -4.97)**	-13.06 (-33.50, 7.53)	RIS600mgIV	**-29.19 (-60.08, -0.24)**	-26.57 (-54.99, 2.97)	-9.88 (-40.01, 21.47)	-19.68 (-48.49, 10.51)	2.90 (-26.58, 35.35)	-17.36 (-48.00, 14.47)	-4.42 (-28.14, 20.49)	-0.47 (-32.03, 32.83)	-17.63 (-40.36, 7.12)	-16.96 (-41.29, 7.42)
SEM60mgIV	14.33 (-14.66, 43.14)	17.80 (-9.62, 46.77)	1.97 (-27.47, 32.64)	10.36 (-20.27, 38.97)	5.08 (-23.30, 34.25)	7.55 (-23.52, 38.51)	26.88 (-4.16, 58.93)	27.54 (-3.26, 58.35)	**43.05 (11.35, 71.88)**	13.24 (-13.90, 40.96)	1.93 (-18.54, 22.24)	15.98 (-12.91, 46.52)	**29.19 (0.24, 60.08)**	SEM60mgIV	3.03 (-18.40, 24.23)	19.53 (-11.69, 50.57)	9.72 (-19.08, 37.33)	**32.06 (0.96, 63.14)**	12.22 (-19.01, 42.99)	**24.86 (1.22, 49.27)**	28.38 (-2.87, 62.21)	11.53 (-12.23, 34.67)	12.48 (-11.93, 35.92)

	CER400mgSC	FIL200mgPO	FON1mg/kg0.1mgIV/SC	FON1mg/kg1mgIV/SC	FON4mg/kg0.1mgIV/SC	FON4mg/kg1mgIV/SC	INF10mg/kgIV	INF20mg/kgIV	INF5mg/kgIV	NAT300mgIV	PBO	RIS200mgIV	RIS600mgIV	SEM60mgIV	SEM60mgIVx3	UPA12mgPO_BID	UPA24mgPO	UPA24mgPO_BID	UPA3mgPO_BID	UPA45mgPO	UPA6mgPO_BID	UST130mgIV	UST6mg/kgIV
SEM60mgIVx3	11.40 (-16.61, 38.88)	15.01 (-13.67, 43.45)	-0.64 (-29.21, 27.74)	7.91 (-22.08, 35.07)	2.40 (-26.33, 31.37)	4.55 (-24.91, 34.26)	24.17 (-6.03, 52.93)	24.87 (-5.32, 54.14)	**40.20 (8.57, 69.34)**	10.44 (-15.89, 36.41)	-0.92 (-20.63, 18.93)	13.45 (-16.18, 41.12)	26.57 (-2.97, 54.99)	-3.03 (-24.23, 18.40)	SEM60mgIVx3	16.76 (-13.65, 45.37)	7.03 (-22.55, 33.58)	**28.97 (0.45, 58.66)**	9.18 (-22.43, 37.38)	22.18 (-0.80, 45.38)	26.25 (-5.02, 56.37)	8.77 (-15.46, 31.73)	9.28 (-15.16, 32.36)
UPA12mgPO_BID	-5.40 (-33.72, 23.69)	-1.72 (-31.73, 29.23)	-17.18 (-47.96, 13.63)	-8.83 (-40.71, 22.81)	-14.16 (-45.41, 17.10)	-11.86 (-43.83, 20.33)	7.56 (-24.16, 40.81)	8.08 (-21.56, 41.10)	23.18 (-9.57, 54.94)	-6.35 (-33.63, 22.62)	-17.50 (-40.03, 4.79)	-3.28 (-34.74, 26.97)	9.88 (-21.47, 40.01)	-19.53 (-50.57, 11.69)	-16.76 (-45.37, 13.65)	UPA12mgPO_BID	-9.71 (-31.13, 12.05)	12.37 (-10.38, 36.94)	-7.43 (-31.11, 16.39)	5.30 (-19.75, 32.33)	9.16 (-15.83, 35.71)	-7.92 (-32.29, 16.69)	-7.03 (-33.16, 17.70)
UPA24mgPO	4.43 (-25.02, 31.63)	8.28 (-21.07, 37.34)	-7.51 (-37.13, 22.22)	0.78 (-28.58, 30.35)	-4.68 (-33.28, 24.09)	-2.18 (-32.73, 28.98)	16.68 (-12.43, 48.95)	17.53 (-11.32, 47.75)	**32.98 (2.71, 63.45)**	3.41 (-22.92, 30.43)	-7.90 (-28.76, 11.66)	6.44 (-24.17, 36.68)	19.68 (-10.51, 48.49)	-9.72 (-37.33, 19.08)	-7.03 (-33.58, 22.55)	9.71 (-12.05, 31.13)	UPA24mgPO	22.16 (-0.23, 45.31)	2.18 (-20.63, 24.27)	15.15 (-9.27, 39.88)	18.93 (-4.46, 42.23)	1.93 (-22.06, 23.42)	2.60 (-21.14, 24.99)
UPA24mgPO_BID	-17.71 (-47.73, 10.78)	-14.16 (-44.09, 15.82)	-29.74 (-59.87, 0.72)	-21.39 (-54.68, 8.41)	-26.57 (-56.56, 3.01)	-24.72 (-56.34, 6.78)	-4.90 (-38.44, 27.01)	-4.37 (-36.08, 26.09)	11.14 (-20.14, 41.41)	-18.58 (-45.92, 8.67)	**-30.03 (-53.22, -8.65)**	-16.22 (-46.16, 13.13)	-2.90 (-35.35, 26.58)	**-32.06 (-63.14, -0.96)**	**-28.97 (-58.66, -0.45)**	-12.37 (-36.94, 10.38)	-22.16 (-45.31, 0.23)	UPA24mgPO_BID	-19.73 (-46.39, 4.30)	-6.84 (-33.06, 18.21)	-2.86 (-30.37, 21.62)	-20.18 (-44.62, 3.01)	-19.47 (-44.79, 4.05)
UPA3mgPO_BID	2.17 (-27.14, 31.53)	6.22 (-22.71, 37.29)	-9.50 (-39.59, 21.13)	-1.50 (-32.36, 29.19)	-6.57 (-36.35, 24.24)	-4.53 (-36.43, 27.82)	14.72 (-17.80, 48.22)	15.95 (-17.48, 47.85)	30.90 (-1.50, 62.68)	1.25 (-25.47, 30.89)	-10.07 (-32.96, 12.86)	4.19 (-26.49, 34.50)	17.36 (-14.47, 48.00)	-12.22 (-42.99, 19.01)	-9.18 (-37.38, 22.43)	7.43 (-16.39, 31.11)	-2.18 (-24.27, 20.63)	19.73 (-4.30, 46.39)	UPA3mgPO_BID	12.95 (-13.43, 38.73)	16.89 (-8.26, 42.21)	-0.30 (-26.19, 23.42)	0.22 (-24.63, 24.94)
UPA45mgPO	-10.87 (-34.19, 12.37)	-6.96 (-30.65, 17.72)	-22.71 (-45.94, 2.77)	-14.45 (-40.69, 9.44)	-20.04 (-43.96, 6.37)	-17.60 (-42.72, 9.08)	1.78 (-24.06, 27.90)	2.75 (-22.52, 27.47)	17.96 (-7.82, 42.71)	-11.58 (-32.89, 10.79)	**-23.09 (-36.38, -10.81)**	-8.74 (-33.57, 13.92)	4.42 (-20.49, 28.14)	**-24.86 (-49.27, -1.22)**	-22.18 (-45.38, 0.80)	-5.30 (-32.33, 19.75)	-15.15 (-39.88, 9.27)	6.84 (-18.21, 33.06)	-12.95 (-38.73, 13.43)	UPA45mgPO	3.80 (-22.76, 30.77)	-13.32 (-31.21, 3.10)	-12.58 (-30.37, 2.98)
UPA6mgPO_BID	-14.76 (-46.28, 16.54)	-10.75 (-42.00, 20.66)	-26.31 (-58.79, 5.09)	-17.99 (-53.07, 13.74)	-23.41 (-56.04, 8.39)	-21.28 (-55.08, 11.29)	-2.17 (-35.93, 32.74)	-1.75 (-34.11, 33.56)	13.67 (-19.47, 48.03)	-15.36 (-44.15, 13.89)	**-26.79 (-51.01, -3.00)**	-12.55 (-44.67, 18.53)	0.47 (-32.83, 32.03)	-28.38 (-62.21, 2.87)	-26.25 (-56.37, 5.02)	-9.16 (-35.71, 15.83)	-18.93 (-42.23, 4.46)	2.86 (-21.62, 30.37)	-16.89 (-42.21, 8.26)	-3.80 (-30.77, 22.76)	UPA6mgPO_BID	-17.00 (-44.71, 8.70)	-16.30 (-43.71, 10.06)
UST130mgIV	2.42 (-19.05, 25.05)	6.18 (-17.85, 30.12)	-9.30 (-32.91, 15.54)	-1.25 (-26.50, 23.56)	-6.35 (-29.91, 18.96)	-4.42 (-28.23, 22.28)	15.24 (-10.91, 42.73)	15.77 (-9.34, 42.93)	**31.28 (6.72, 55.98)**	1.59 (-18.86, 23.03)	-9.77 (-21.36, 2.38)	4.50 (-20.05, 27.83)	17.63 (-7.12, 40.36)	-11.53 (-34.67, 12.23)	-8.77 (-31.73, 15.46)	7.92 (-16.69, 32.29)	-1.93 (-23.42, 22.06)	20.18 (-3.01, 44.62)	0.30 (-23.42, 26.19)	13.32 (-3.10, 31.21)	17.00 (-8.70, 44.71)	UST130mgIV	0.60 (-11.85, 12.67)
UST6mg/kgIV	1.61 (-20.94, 24.25)	5.56 (-18.32, 29.36)	-10.20 (-33.82, 14.58)	-1.98 (-25.41, 21.83)	-7.26 (-29.71, 17.57)	-4.95 (-29.72, 21.68)	14.44 (-12.01, 40.68)	15.34 (-9.81, 41.27)	**30.67 (5.60, 54.97)**	0.90 (-19.46, 22.53)	-10.49 (-21.70, 1.58)	4.08 (-21.37, 28.39)	16.96 (-7.42, 41.29)	-12.48 (-35.92, 11.93)	-9.28 (-32.36, 15.16)	7.03 (-17.70, 33.16)	-2.60 (-24.99, 21.14)	19.47 (-4.05, 44.79)	-0.22 (-24.94, 24.63)	12.58 (-2.98, 30.37)	16.30 (-10.06, 43.71)	-0.60 (-12.67, 11.85)	UST6mg/kgIV

Table S14b: League table for IBDQ remission (IBDQ score ≥170)

	ADA160mg80mg40mgSC	GUS1200mgIV	GUS200mgIV	GUS600mgIV	PBO	TOF15mgPO_BID	TOF1PO_BID	TOF5mgPO_BID	UPA45mgPO	UST130mgIV	UST6mg/kgIV	UST6mg/kg90mgIV/SC
ADA160mg80mg40mgSC	ADA160mg80mg40mgSC	0.96 (0.21, 4.94)	1.17 (0.25, 5.96)	1.06 (0.21, 5.15)	0.46 (0.10, 2.51)	0.66 (0.09, 5.31)	0.85 (0.11, 6.21)	0.91 (0.12, 6.59)	1.04 (0.19, 6.12)	1.18 (0.16, 10.58)	2.03 (0.30, 17.83)	1.02 (0.36, 3.05)
GUS1200mgIV	1.04 (0.20, 4.67)	GUS1200mgIV	1.23 (0.44, 3.54)	1.11 (0.37, 3.38)	0.49 (0.16, 1.51)	0.70 (0.13, 3.81)	0.90 (0.17, 4.72)	0.94 (0.17, 4.79)	1.09 (0.29, 3.97)	1.24 (0.23, 8.20)	2.10 (0.40, 12.78)	1.06 (0.34, 2.99)
GUS200mgIV	0.85 (0.17, 3.94)	0.81 (0.28, 2.28)	GUS200mgIV	0.90 (0.28, 2.71)	0.40 (0.13, 1.24)	0.56 (0.11, 3.14)	0.73 (0.12, 3.69)	0.77 (0.15, 3.80)	0.89 (0.24, 3.33)	1.01 (0.20, 6.79)	1.72 (0.34, 10.73)	0.87 (0.29, 2.37)
GUS600mgIV	0.94 (0.19, 4.74)	0.90 (0.30, 2.73)	1.11 (0.37, 3.55)	GUS600mgIV	0.44 (0.15, 1.38)	0.63 (0.11, 3.58)	0.81 (0.15, 4.28)	0.86 (0.16, 4.18)	0.98 (0.28, 3.79)	1.11 (0.21, 7.92)	1.89 (0.37, 12.38)	0.97 (0.31, 2.88)
PBO	2.15 (0.40, 9.94)	2.03 (0.66, 6.09)	2.50 (0.80, 7.49)	2.25 (0.72, 6.58)	PBO	1.40 (0.38, 5.06)	1.78 (0.55, 6.20)	1.89 (0.53, 6.38)	2.22 (1.00, 4.66)	2.47 (0.65, 11.51)	**4.24 (1.32, 17.72)**	2.16 (0.71, 6.64)
TOF15mgPO_BID	1.52 (0.19, 10.71)	1.43 (0.26, 7.62)	1.77 (0.32, 9.43)	1.58 (0.28, 8.76)	0.71 (0.20, 2.62)	TOF15mgPO_BID	1.27 (0.38, 4.47)	1.37 (0.38, 4.39)	1.57 (0.35, 7.46)	1.75 (0.31, 12.36)	3.01 (0.52, 21.43)	1.54 (0.28, 8.24)
TOF1mgPO_BID	1.17 (0.16, 8.85)	1.11 (0.21, 5.92)	1.37 (0.27, 8.09)	1.24 (0.23, 6.62)	0.56 (0.16, 1.83)	0.78 (0.22, 2.62)	TOF1OmgR_BID	1.05 (0.32, 3.26)	1.23 (0.30, 5.34)	1.34 (0.23, 9.81)	2.37 (0.40, 15.80)	1.19 (0.22, 6.61)
TOF5mgPO_BID	1.10 (0.15, 8.07)	1.06 (0.21, 5.79)	1.30 (0.26, 6.49)	1.16 (0.24, 6.44)	0.53 (0.16, 1.88)	0.73 (0.23, 2.65)	0.95 (0.31, 3.17)	TOF5mgPO_BID	1.17 (0.29, 5.40)	1.29 (0.22, 10.17)	2.21 (0.41, 17.04)	1.13 (0.23, 5.94)
UPA45mgPO	0.96 (0.16, 5.24)	0.92 (0.25, 3.47)	1.13 (0.30, 4.13)	1.02 (0.26, 3.61)	0.45 (0.21, 1.00)	0.64 (0.13, 2.82)	0.82 (0.19, 3.37)	0.86 (0.19, 3.50)	UPA45mgPO	1.12 (0.27, 6.01)	1.94 (0.47, 9.89)	0.98 (0.25, 3.64)
UST130mgIV	0.85 (0.09, 6.41)	0.81 (0.12, 4.29)	0.99 (0.15, 4.98)	0.90 (0.13, 4.74)	0.41 (0.09, 1.53)	0.57 (0.08, 3.24)	0.74 (0.10, 4.44)	0.78 (0.10, 4.57)	0.89 (0.17, 3.74)	UST130mgIV	1.72 (0.56, 5.21)	0.88 (0.12, 4.75)
UST6mg/kgIV	0.49 (0.06, 3.35)	0.48 (0.08, 2.53)	0.58 (0.09, 2.95)	0.53 (0.08, 2.72)	**0.24 (0.06, 0.76)**	0.33 (0.05, 1.92)	0.42 (0.06, 2.47)	0.45 (0.06, 2.46)	0.52 (0.10, 2.12)	0.58 (0.19, 1.78)	UST6mg/kgIV	0.50 (0.08, 2.60)
UST6mg/kg90mgIV/SC	0.98 (0.33, 2.81)	0.94 (0.33, 2.94)	1.14 (0.42, 3.46)	1.03 (0.35, 3.21)	0.46 (0.15, 1.41)	0.65 (0.12, 3.61)	0.84 (0.15, 4.56)	0.89 (0.17, 4.41)	1.02 (0.27, 4.08)	1.14 (0.21, 8.31)	2.00 (0.39, 12.42)	UST6mg/kg90mgIV/SC

Table S14c: League table for IBDQ remission (improvement of 16 points compared to baseline)

	ADA160mg80mg40mgSC	GUS1200mgIV	GUS200mgIV	GUS600mgIV	PBO	TOF15mgPO_BID	TOF1PO_BID	TOF5mgPO_BID	UPA45mgPO	UST130mgIV	UST6mg/kgIV	UST6mg/kg90mgIV/SC
ADA160mg80mg40mgSC	ADA160mg80mg40mgSC	1.00 (0.66, 1.57)	0.96 (0.63, 1.48)	1.04 (0.69, 1.61)	**0.57 (0.34, 0.93)**	0.75 (0.35, 1.64)	0.72 (0.34, 1.62)	0.98 (0.48, 2.10)	0.73 (0.42, 1.27)	0.77 (0.45, 1.33)	0.90 (0.52, 1.55)	1.06 (0.82, 1.43)
GUS1200mgIV	1.00 (0.64, 1.52)	GUS1200mgIV	0.95 (0.66, 1.32)	1.03 (0.74, 1.42)	**0.57 (0.36, 0.84)**	0.75 (0.35, 1.58)	0.72 (0.35, 1.52)	0.97 (0.49, 1.95)	0.73 (0.45, 1.12)	0.77 (0.47, 1.21)	0.90 (0.54, 1.41)	1.05 (0.76, 1.45)
GUS200mgIV	1.05 (0.68, 1.59)	1.05 (0.76, 1.51)	GUS200mgIV	1.08 (0.79, 1.54)	**0.59 (0.38, 0.89)**	0.78 (0.36, 1.61)	0.76 (0.37, 1.62)	1.02 (0.52, 2.09)	0.76 (0.47, 1.20)	0.81 (0.50, 1.28)	0.94 (0.59, 1.48)	1.11 (0.79, 1.54)
GUS600mgIV	0.96 (0.62, 1.45)	0.97 (0.70, 1.36)	0.92 (0.65, 1.27)	GUS600mgIV	**0.54 (0.35, 0.81)**	0.72 (0.35, 1.55)	0.69 (0.34, 1.46)	0.93 (0.47, 1.89)	0.70 (0.42, 1.11)	0.74 (0.46, 1.17)	0.86 (0.54, 1.35)	1.02 (0.74, 1.42)
PBO	**1.76 (1.07, 2.91)**	**1.77 (1.18, 2.78)**	**1.69 (1.13, 2.65)**	**1.84 (1.24, 2.83)**	PBO	1.32 (0.73, 2.46)	1.27 (0.73, 2.33)	**1.72 (1.05, 3.06)**	**1.28 (1.04, 1.58)**	**1.36 (1.10, 1.70)**	**1.58 (1.28, 2.00)**	**1.88 (1.25, 2.90)**
TOF15mgPO_BID	1.33 (0.61, 2.88)	1.34 (0.63, 2.82)	1.28 (0.62, 2.76)	1.39 (0.65, 2.90)	0.76 (0.41, 1.37)	TOF15mgPO_BID	0.97 (0.58, 1.65)	1.30 (0.85, 2.10)	0.97 (0.51, 1.81)	1.03 (0.53, 1.91)	1.20 (0.62, 2.24)	1.42 (0.68, 2.97)
TOF1mgPO_BID	1.40 (0.62, 2.91)	1.39 (0.66, 2.82)	1.32 (0.62, 2.73)	1.44 (0.68, 2.93)	0.79 (0.43, 1.38)	1.03 (0.60, 1.72)	TOF1mgPO_BID	1.34 (0.87, 2.18)	1.01 (0.53, 1.81)	1.07 (0.56, 1.96)	1.25 (0.66, 2.30)	1.46 (0.70, 2.96)
TOF5mgPO_BID	1.02 (0.48, 2.08)	1.03 (0.51, 2.05)	0.98 (0.48, 1.94)	1.07 (0.53, 2.11)	**0.58 (0.33, 0.95)**	0.77 (0.48, 1.17)	0.75 (0.46, 1.14)	TOF5mgPO_BID	0.74 (0.41, 1.27)	0.79 (0.43, 1.39)	0.92 (0.50, 1.60)	1.09 (0.55, 2.12)
UPA45mgPO	1.38 (0.79, 2.36)	1.38 (0.89, 2.24)	1.31 (0.83, 2.11)	1.43 (0.90, 2.36)	**0.78 (0.63, 0.96)**	1.03 (0.55, 1.97)	0.99 (0.55, 1.87)	1.34 (0.79, 2.43)	UPA45mgPO	1.06 (0.79, 1.44)	1.24 (0.91, 1.69)	1.46 (0.93, 2.29)
UST130mgIV	1.30 (0.75, 2.23)	1.30 (0.83, 2.15)	1.24 (0.78, 2.01)	1.35 (0.85, 2.18)	**0.74 (0.59, 0.91)**	0.97 (0.52, 1.88)	0.94 (0.51, 1.77)	1.27 (0.72, 2.31)	0.94 (0.69, 1.27)	UST130mgIV	1.17 (0.95, 1.42)	1.38 (0.87, 2.21)
UST6mg/kgIV	1.11 (0.64, 1.91)	1.12 (0.71, 1.84)	1.06 (0.68, 1.69)	1.16 (0.74, 1.87)	**0.63 (0.50, 0.78)**	0.83 (0.45, 1.61)	0.80 (0.44, 1.52)	1.09 (0.63, 2.02)	0.81 (0.59, 1.10)	0.86 (0.70, 1.05)	UST6mg/kgIV	1.19 (0.75, 1.87)
UST6mg/kg90mgIV/SC	0.94 (0.70, 1.22)	0.95 (0.69, 1.31)	0.90 (0.65, 1.26)	0.98 (0.71, 1.36)	**0.53 (0.35, 0.80)**	0.71 (0.34, 1.46)	0.68 (0.34, 1.43)	0.92 (0.47, 1.81)	0.68 (0.44, 1.07)	0.73 (0.45, 1.15)	0.84 (0.53, 1.33)	UST6mg/kg90mgIV/SC

TABLE S15. SUCRA PROBABILITYS FOR IBDQ OUTCOMES. Higher probabilities are more closely associated with the chance of the drug treatment having the highest occurrence of the event in relation to the other drugs included in the network. Higher probabilities indicate better-performing interventions. For better visualization access the excel version using the LINK

	% SUCRA IBDQ score	% SUCRA IBDQ remission	% SUCRA IBDQ response
ADA160mg80mg40mgSC	-	52.04	66.49
CER400mgSC	48.29	-	-
FIL200mgPO	57.29	-	-
FON1mg/kg0.1mgIV_SC	18.75	-	-
FON1mg/kg1mgIV_SC	38.64	-	-
FON4mg/kg0.1mgIV_SC	26.20	-	-
FON4mg/kg1mgIV_SC	31.48	-	-
INF10mg/kgIV	74.12	-	-
INF20mg/kgIV	76.91	-	-
INF5mg/kgIV	95.14	-	-
GUS1200mgIV	-	47.93	68.46
GUS200mgIV	-	64.49	60.87
GUS600mgIV	-	57.02	74.59
NAT300mgIV	45.21	-	-
PBO	14.02	8.69	4.07
RIS200mgIV	52.52	-	-
RIS600mgIV	80.35	-	-
SEM60mgIV	14.43	-	-
SEM60mgIVx3	20.16	-	-
TOF15mgPO_BID	-	29.26	34.07
TOF1mgPO_BID	-	44.20	30.55
TOF5mgPO_BID	-	47.46	66.69
UPA12mgPO_BID	59.77	-	-
UPA24mgPO	35.89	55.09	-
UPA24mgPO_BID	83.87	-	-
UPA3mgPO_BID	40.78	-	-
UPA45mgPO	74.68	55.09	26.00
UPA6mgPO_BID	78.23	-	-
UST130mgIV	40.46	56.83	32.98
UST6mg/kgIV	42.82	83.31	56.71
UST6mg_mg90mgIV_SC	-	53.68	78.53

Table S16: League Tables for Safety Outcomes. Each table presents data from a network constructed for safety outcomes: main network (serious adverse events) and sensitive analysis of any adverse events and infections. The table presented the multiple treatment comparisons based on consistency analysis of the networks. Treatments are depicted alphabetically. The upper quadrant is the inverse of the lower quadrant, i.e., the mirror image of the results Values are presented as relative risk (RR) with 95% credible interval (CrI). For all comparisons an RR >1 favors the occurrence of the event for the row-defining treatment, while an RR<1 favors the column-defining treatment. Statistically significant results are represented in bold. Multiple treatment comparisons based on consistency analysis of the networks. Treatments are depicted alphabetically. Values are presented as relative risk (RR) with 95% credible interval (CrI). For all comparisons an RR <1 favors the occurrence of the event for the row-defining treatment, while an RR>1 favors the column-defining treatment. Statistically significant results are represented in bold. For a better visualization of the league tables, access the Excel version using the LINK

TABLE S16A. League table for serious adverse events (main network).

	ABA10mg_kgIV	ABA30mg_kgIV	ABA3mg_kgIV	ADA160mg80mg40mgSC	ADA160mg80mg60mgSC	ADA160mg80mgSC	ADA40mg20mgSC	ADA80mg40mgSC	AMI0_4mgPO	AND150mgSC1_1	AND150mgSC2_2	AND300mgSC	API100mgPR	API50mgPO	BRI400mgIV	BRI700mgIV	BRO210mgIV	BRO350mgIV	BRO700mgIV	CDP10mg_kgIV	CER100mgSC
ABA10mg_kgIV	ABA10mg_kgIV	0.96 (0.42, 2.12)	0.89 (0.44, 1.80)	1.15 (0.19, 6.65)	2.23 (0.15, 72.18)	0.51 (0.15, 1.56)	0.17 (0.01, 1.31)	0.20 (0.02, 1.00)	7.02 (0.81, 174.62)	1.00 (0.21, 6.24)	0.12 (0.00, 1.35)	1.42 (0.32, 7.98)	0.44 (0.09, 1.78)	0.67 (0.17, 2.46)	0.45 (0.05, 2.75)	0.30 (0.06, 1.60)	1.48 (0.22, 13.62)	4.11 (0.80, 34.32)	4.70 (0.99, 39.36)	0.69 (0.26, 1.93)	1.21 (0.33, 4.08)
ABA30mg_kgIV	1.04 (0.47, 2.39)	ABA30mg_kgIV	0.92 (0.43, 2.07)	1.21 (0.19, 7.24)	2.34 (0.15, 76.39)	0.53 (0.15, 1.77)	0.18 (0.01, 1.47)	0.21 (0.02, 1.09)	7.30 (0.85, 176.05)	1.08 (0.20, 6.54)	0.13 (0.00, 1.40)	1.49 (0.30, 8.90)	0.46 (0.09, 1.86)	0.70 (0.16, 2.66)	0.47 (0.05, 2.99)	0.32 (0.06, 1.74)	1.58 (0.21, 14.81)	4.38 (0.82, 36.15)	4.93 (0.97, 39.30)	0.73 (0.25, 2.17)	1.25 (0.33, 4.51)
ABA3mg_kgIV	1.13 (0.56, 2.25)	1.09 (0.48, 2.35)	ABA3mg_kgIV	1.30 (0.21, 7.70)	2.55 (0.17, 84.50)	0.58 (0.17, 1.83)	0.19 (0.01, 1.58)	0.23 (0.03, 1.16)	7.92 (0.98, 183.15)	1.15 (0.23, 6.72)	0.14 (0.00, 1.54)	1.62 (0.34, 9.48)	0.50 (0.10, 1.93)	0.76 (0.18, 2.83)	0.52 (0.06, 3.14)	0.34 (0.06, 1.82)	1.68 (0.24, 15.87)	4.67 (0.89, 35.96)	**5.35 (1.09, 40.96)**	0.79 (0.29, 2.17)	1.36 (0.37, 4.60)
ADA160mg80mg40mgSC	0.87 (0.15, 5.27)	0.83 (0.14, 5.22)	0.77 (0.13, 4.80)	ADA160mg80mg40mgSC	1.89 (0.09, 83.18)	0.44 (0.06, 2.90)	0.14 (0.00, 1.93)	0.18 (0.01, 1.57)	6.32 (0.45, 180.50)	0.90 (0.10, 9.98)	0.10 (0.00, 2.02)	1.22 (0.13, 13.03)	0.37 (0.05, 2.89)	0.57 (0.08, 4.17)	0.38 (0.03, 4.33)	0.26 (0.03, 2.55)	1.31 (0.10, 18.33)	3.71 (0.40, 45.30)	4.14 (0.44, 50.27)	0.61 (0.10, 3.58)	1.02 (0.14, 7.49)
ADA160mg80mg60mgSC	0.45 (0.01, 6.81)	0.43 (0.01, 6.72)	0.39 (0.01, 5.72)	0.53 (0.01, 10.67)	ADA160mg80mg60mgSC	0.23 (0.01, 3.34)	0.07 (0.00, 2.03)	0.09 (0.00, 1.73)	3.32 (0.05, 159.03)	0.46 (0.02, 8.83)	0.05 (0.00, 1.54)	0.65 (0.02, 12.18)	0.19 (0.01, 3.44)	0.28 (0.01, 4.69)	0.18 (0.00, 4.50)	0.13 (0.00, 2.90)	0.67 (0.02, 17.12)	1.86 (0.05, 44.34)	2.11 (0.06, 49.29)	0.31 (0.01, 4.36)	0.52 (0.01, 8.87)
ADA160mg80mgSC	1.97 (0.64, 6.54)	1.89 (0.57, 6.64)	1.72 (0.55, 5.83)	2.29 (0.35, 15.47)	4.34 (0.30, 142.81)	ADA160mg80mgSC	0.34 (0.01, 2.52)	0.40 (0.05, 1.75)	**14.03 (1.58, 331.79)**	2.03 (0.37, 13.74)	0.24 (0.01, 2.73)	2.80 (0.54, 18.04)	0.84 (0.16, 4.08)	1.30 (0.29, 5.67)	0.88 (0.09, 6.61)	0.59 (0.10, 3.70)	2.93 (0.39, 30.14)	**8.04 (1.52, 75.71)**	**9.15 (1.81, 82.70)**	1.35 (0.44, 4.71)	2.32 (0.57, 9.67)
ADA40mg20mgSC	5.81 (0.76, 165.21)	5.54 (0.68, 174.78)	5.16 (0.63, 149.04)	7.10 (0.52, 239.16)	14.50 (0.49, 1450.33)	2.91 (0.40, 87.32)	ADA40mg20mgSC	1.13 (0.08, 34.20)	**44.85 (2.61, 3280.75)**	6.23 (0.50, 231.29)	0.70 (0.01, 46.18)	8.80 (0.74, 344.97)	2.60 (0.22, 82.60)	3.97 (0.38, 120.25)	2.64 (0.14, 114.48)	1.82 (0.15, 62.71)	9.38 (0.51, 401.36)	**25.09 (2.05, 1018.56)**	**28.93 (2.31, 1200.59)**	4.07 (0.51, 122.73)	7.00 (0.74, 229.05)
ADA80mg40mgSC	4.94 (1.00, 44.22)	4.68 (0.91, 41.64)	4.38 (0.86, 39.13)	5.66 (0.64, 77.43)	11.73 (0.58, 463.46)	2.49 (0.57, 19.11)	0.88 (0.03, 12.54)	ADA80mg40mgSC	**37.83 (2.69, 1276.02)**	5.19 (0.66, 72.56)	0.61 (0.01, 12.30)	7.15 (0.89, 100.46)	2.09 (0.30, 23.14)	3.27 (0.51, 34.59)	2.16 (0.18, 28.16)	1.52 (0.17, 19.01)	7.41 (0.70, 145.22)	**20.83 (2.51, 405.85)**	**23.55 (3.03, 430.68)**	3.35 (0.68, 32.05)	5.94 (0.98, 60.22)
AMI0_4mgPO	0.14 (0.01, 1.23)	0.14 (0.01, 1.18)	0.13 (0.01, 1.02)	0.16 (0.01, 2.21)	0.30 (0.01, 18.50)	**0.07 (0.00, 0.63)**	**0.02 (0.00, 0.38)**	**0.03 (0.00, 0.37)**	AMI0_4mgPO	0.14 (0.00, 1.96)	**0.02 (0.00, 0.39)**	0.20 (0.01, 2.76)	**0.06 (0.00, 0.60)**	**0.09 (0.00, 0.95)**	**0.06 (0.00, 0.84)**	**0.04 (0.00, 0.54)**	0.21 (0.01, 4.21)	0.58 (0.02, 10.21)	0.66 (0.02, 11.52)	**0.10 (0.00, 0.82)**	0.17 (0.01, 1.61)
AND150mgSC1_1	1.00 (0.16, 4.88)	0.92 (0.15, 5.11)	0.87 (0.15, 4.35)	1.11 (0.10, 10.28)	2.18 (0.11, 64.05)	0.49 (0.07, 2.73)	0.16 (0.00, 2.02)	0.19 (0.01, 1.51)	6.97 (0.51, 219.45)	AND150mgSC1_1	**0.12 (0.00, 0.82)**	1.36 (0.48, 4.24)	0.42 (0.05, 2.82)	0.63 (0.09, 3.91)	0.41 (0.03, 4.03)	0.29 (0.03, 2.19)	1.44 (0.12, 17.59)	4.04 (0.49, 45.66)	4.45 (0.59, 48.76)	0.69 (0.11, 3.34)	1.15 (0.18, 6.72)
AND150mgSC2_2	8.23 (0.74, 304.09)	7.73 (0.72, 297.36)	7.16 (0.65, 274.61)	9.71 (0.50, 584.17)	19.79 (0.65, 2722.51)	4.09 (0.37, 143.86)	1.43 (0.02, 78.12)	1.63 (0.08, 79.34)	**65.49 (2.56, 4666.97)**	**8.01 (1.23, 281.70)**	AND150mgSC2_2	**11.13 (1.77, 389.94)**	3.68 (0.22, 148.60)	5.50 (0.41, 211.16)	3.63 (0.17, 180.77)	2.56 (0.15, 102.62)	13.06 (0.67, 676.55)	**36.46 (2.22, 1748.53)**	**41.71 (2.60, 1963.95)**	5.88 (0.49, 201.31)	9.59 (0.84, 324.12)
AND300mgSC	0.71 (0.13, 3.16)	0.67 (0.11, 3.36)	0.62 (0.11, 2.95)	0.82 (0.08, 7.64)	1.55 (0.08, 51.01)	0.36 (0.06, 1.85)	0.11 (0.00, 1.35)	0.14 (0.01, 1.12)	5.12 (0.36, 159.40)	0.73 (0.24, 2.10)	**0.09 (0.00, 0.56)**	AND300mgSC	0.30 (0.04, 1.99)	0.46 (0.07, 2.89)	0.30 (0.02, 2.80)	0.21 (0.02, 1.61)	1.04 (0.10, 12.35)	2.96 (0.33, 31.92)	3.29 (0.43, 34.21)	0.50 (0.09, 2.34)	0.84 (0.13, 4.87)
API100mgPR	2.29 (0.56, 11.02)	2.15 (0.54, 11.50)	1.99 (0.52, 10.35)	2.67 (0.35, 20.92)	5.26 (0.29, 192.89)	1.19 (0.25, 6.37)	0.38 (0.01, 4.50)	0.48 (0.04, 3.32)	**16.82 (1.67, 408.59)**	2.40 (0.35, 21.47)	0.27 (0.01, 4.61)	3.33 (0.50, 28.49)	API100mgPR	1.52 (0.44, 6.22)	1.01 (0.10, 8.87)	0.70 (0.10, 5.14)	3.44 (0.38, 48.16)	**9.64 (1.38, 101.13)**	**11.01 (1.67, 122.01)**	1.62 (0.38, 7.53)	2.75 (0.53, 16.21)
API50mgPO	1.49 (0.41, 6.03)	1.43 (0.38, 6.24)	1.32 (0.35, 5.62)	1.76 (0.24, 11.84)	3.53 (0.21, 119.37)	0.77 (0.18, 3.46)	0.25 (0.01, 2.60)	0.31 (0.03, 1.98)	**11.09 (1.05, 253.27)**	1.59 (0.26, 11.17)	0.18 (0.00, 2.44)	2.16 (0.35, 14.78)	0.66 (0.16, 2.29)	API50mgPO	0.67 (0.06, 5.00)	0.45 (0.07, 3.07)	2.24 (0.27, 25.45)	6.36 (0.94, 61.49)	**7.17 (1.11, 66.94)**	1.06 (0.28, 4.05)	1.78 (0.38, 8.55)
BRI400mgIV	2.22 (0.36, 19.49)	2.11 (0.33, 19.17)	1.93 (0.32, 16.61)	2.62 (0.23, 36.11)	5.64 (0.22, 229.99)	1.13 (0.15, 10.95)	0.38 (0.01, 7.39)	0.46 (0.04, 5.60)	**16.20 (1.19, 665.75)**	2.44 (0.25, 31.80)	0.28 (0.01, 5.91)	3.28 (0.36, 42.78)	0.99 (0.11, 10.25)	1.49 (0.20, 15.54)	BRI400mgIV	0.68 (0.12, 5.32)	3.58 (0.29, 59.65)	9.75 (0.98, 150.05)	**11.05 (1.14, 176.67)**	1.55 (0.26, 13.42)	2.75 (0.35, 25.74)
BRI700mgIV	3.32 (0.63, 17.21)	3.17 (0.57, 17.91)	2.90 (0.55, 15.55)	3.88 (0.39, 33.95)	7.69 (0.34, 271.49)	1.70 (0.27, 9.90)	0.55 (0.02, 6.61)	0.66 (0.05, 5.92)	**23.71 (1.86, 772.57)**	3.45 (0.46, 32.51)	0.39 (0.01, 6.74)	4.72 (0.62, 44.60)	1.43 (0.19, 9.54)	2.21 (0.33, 14.69)	1.48 (0.19, 8.20)	BRI700mgIV	4.89 (0.49, 67.84)	**13.96 (1.68, 170.02)**	**15.67 (1.96, 181.67)**	2.30 (0.43, 12.32)	3.95 (0.59, 24.54)
BRO210mgIV	0.67 (0.07, 4.64)	0.63 (0.07, 4.69)	0.59 (0.06, 4.11)	0.77 (0.05, 9.57)	1.50 (0.06, 53.03)	0.34 (0.03, 2.55)	0.11 (0.00, 1.97)	0.13 (0.01, 1.44)	4.66 (0.24, 171.28)	0.70 (0.06, 8.22)	0.08 (0.00, 1.49)	0.96 (0.08, 10.11)	0.29 (0.02, 2.62)	0.45 (0.04, 3.70)	0.28 (0.02, 3.47)	0.20 (0.01, 2.03)	BRO210mgIV	2.68 (0.79, 12.97)	3.04 (0.96, 14.44)	0.47 (0.05, 3.57)	0.78 (0.07, 6.41)
BRO350mgIV	0.24 (0.03, 1.25)	0.23 (0.03, 1.22)	0.21 (0.03, 1.12)	0.27 (0.02, 2.51)	0.54 (0.02, 18.48)	**0.12 (0.01, 0.66)**	**0.04 (0.00, 0.49)**	**0.05 (0.00, 0.40)**	1.73 (0.10, 57.23)	0.25 (0.02, 2.03)	**0.03 (0.00, 0.45)**	0.34 (0.03, 3.01)	**0.10 (0.01, 0.73)**	0.16 (0.02, 1.06)	0.10 (0.01, 1.02)	**0.07 (0.01, 0.60)**	0.37 (0.08, 1.26)	BRO350mgIV	1.13 (0.43, 3.00)	**0.17 (0.02, 0.88)**	0.29 (0.03, 1.75)
BRO700mgIV	0.21 (0.03, 1.01)	0.20 (0.03, 1.03)	**0.19 (0.02, 0.92)**	0.24 (0.02, 2.30)	0.47 (0.02, 16.83)	**0.11 (0.01, 0.55)**	**0.03 (0.00, 0.43)**	**0.04 (0.00, 0.33)**	1.51 (0.09, 45.88)	0.22 (0.02, 1.70)	**0.02 (0.00, 0.38)**	0.30 (0.03, 2.32)	**0.09 (0.01, 0.60)**	**0.14 (0.01, 0.90)**	**0.09 (0.01, 0.88)**	**0.06 (0.01, 0.51)**	0.33 (0.07, 1.04)	0.88 (0.33, 2.32)	BRO700mgIV	**0.15 (0.02, 0.72)**	0.25 (0.03, 1.46)
CDP10mg_kgIV	1.44 (0.52, 3.88)	1.37 (0.46, 4.05)	1.27 (0.46, 3.49)	1.63 (0.28, 9.68)	3.23 (0.23, 104.98)	0.74 (0.21, 2.28)	0.25 (0.01, 1.95)	0.30 (0.03, 1.46)	**10.04 (1.23, 247.34)**	1.46 (0.30, 8.71)	0.17 (0.00, 2.04)	2.02 (0.43, 11.52)	0.62 (0.13, 2.61)	0.94 (0.25, 3.63)	0.65 (0.07, 3.90)	0.43 (0.08, 2.35)	2.15 (0.28, 18.84)	**5.99 (1.14, 45.42)**	**6.75 (1.39, 52.18)**	CDP10mg_kgIV	1.73 (0.45, 5.96)
CER100mgSC	0.83 (0.25, 3.06)	0.80 (0.22, 3.01)	0.73 (0.22, 2.72)	0.98 (0.13, 7.34)	1.94 (0.11, 67.35)	0.43 (0.10, 1.76)	0.14 (0.00, 1.35)	0.17 (0.02, 1.02)	5.98 (0.62, 154.50)	0.87 (0.15, 5.55)	0.10 (0.00, 1.19)	1.19 (0.21, 7.73)	0.36 (0.06, 1.90)	0.56 (0.12, 2.63)	0.36 (0.04, 2.82)	0.25 (0.04, 1.68)	1.29 (0.16, 13.61)	3.45 (0.57, 33.03)	3.94 (0.68, 37.10)	0.58 (0.17, 2.21)	CER100mgSC

	ABA10mg_kgIV	ABA30mg_kgIV	ABA3mg_kgIV	ADA160mg80mg40mgSC	ADA160mg80mg60mgSC	ADA160mg80mgSC	ADA40mg20mgSC	ADA80mg40mgSC	AMI0_4mgPO	AND150mgSC1_1	AND150mgSC2_2	AND300mgSC	API100mgPR	API50mgPO	BRI400mgIV	BRI700mgIV	BRO210mgIV	BRO350mgIV	BRO700mgIV	CDP10mg_kgIV	CER100mgSC
CER10mgIV	1.76 (0.13, 62.39)	1.66 (0.13, 65.43)	1.57 (0.11, 56.96)	2.06 (0.10, 106.84)	4.30 (0.11, 703.45)	0.93 (0.06, 37.33)	0.30 (0.00, 17.91)	0.35 (0.02, 16.85)	14.87 (0.44, 1351.01)	1.94 (0.10, 93.79)	0.23 (0.00, 16.27)	2.63 (0.15, 134.96)	0.78 (0.04, 33.97)	1.19 (0.07, 50.33)	0.82 (0.03, 51.16)	0.53 (0.03, 27.52)	2.76 (0.13, 202.25)	8.08 (0.40, 493.83)	9.00 (0.46, 537.75)	1.21 (0.09, 44.58)	2.08 (0.13, 89.67)
CER200mgSC	0.56 (0.17, 1.83)	0.54 (0.16, 1.89)	0.49 (0.15, 1.67)	0.64 (0.09, 4.45)	1.27 (0.08, 43.88)	0.29 (0.07, 1.03)	**0.09 (0.00, 0.85)**	**0.11 (0.01, 0.64)**	3.94 (0.44, 104.34)	0.57 (0.11, 3.91)	**0.07 (0.00, 0.78)**	0.80 (0.15, 5.11)	0.24 (0.04, 1.17)	0.37 (0.08, 1.59)	0.24 (0.03, 1.75)	0.17 (0.03, 1.06)	0.84 (0.11, 8.52)	2.32 (0.39, 20.98)	2.67 (0.47, 22.36)	0.39 (0.12, 1.30)	0.67 (0.23, 1.83)
CER20mgIV	0.36 (0.04, 1.97)	0.34 (0.04, 1.93)	0.33 (0.04, 1.74)	0.41 (0.03, 4.01)	0.81 (0.03, 31.22)	0.18 (0.02, 1.08)	**0.06 (0.00, 0.75)**	**0.07 (0.00, 0.64)**	2.50 (0.15, 77.88)	0.36 (0.03, 3.43)	**0.04 (0.00, 0.64)**	0.52 (0.04, 5.07)	0.15 (0.01, 1.20)	0.24 (0.02, 1.56)	0.15 (0.01, 1.59)	**0.11 (0.01, 0.94)**	0.53 (0.04, 8.19)	1.46 (0.14, 18.44)	1.68 (0.16, 21.58)	0.25 (0.03, 1.35)	0.43 (0.05, 2.72)
CER400mgSC	0.82 (0.33, 2.07)	0.78 (0.29, 2.14)	0.72 (0.29, 1.89)	0.94 (0.16, 5.33)	1.84 (0.13, 58.53)	0.43 (0.13, 1.23)	0.14 (0.01, 1.08)	**0.17 (0.02, 0.79)**	5.73 (0.75, 145.47)	0.82 (0.19, 4.88)	0.10 (0.00, 1.13)	1.16 (0.27, 6.48)	0.36 (0.08, 1.37)	0.55 (0.15, 1.91)	0.37 (0.04, 2.22)	0.25 (0.05, 1.25)	1.22 (0.19, 10.30)	3.37 (0.68, 24.66)	3.88 (0.85, 28.83)	0.58 (0.23, 1.45)	0.99 (0.34, 2.73)
CER5mgIV	2.83 (0.20, 65.01)	2.77 (0.20, 63.37)	2.58 (0.18, 58.24)	3.23 (0.17, 110.57)	7.30 (0.18, 396.03)	1.43 (0.10, 35.22)	0.45 (0.01, 18.45)	0.57 (0.02, 17.45)	21.63 (0.74, 1379.03)	3.01 (0.15, 90.12)	0.35 (0.00, 16.78)	4.15 (0.21, 130.13)	1.22 (0.06, 34.86)	1.95 (0.11, 49.33)	1.32 (0.05, 41.91)	0.87 (0.05, 23.74)	4.45 (0.21, 191.94)	11.96 (0.66, 516.32)	13.96 (0.80, 546.82)	2.00 (0.15, 44.77)	3.41 (0.25, 88.92)
ELD10mg_kgIV	0.70 (0.08, 4.84)	0.67 (0.08, 4.97)	0.61 (0.07, 4.43)	0.77 (0.06, 10.50)	1.55 (0.05, 62.61)	0.34 (0.04, 2.90)	0.12 (0.00, 1.80)	0.14 (0.01, 1.80)	4.98 (0.29, 183.03)	0.73 (0.06, 9.05)	0.08 (0.00, 1.75)	1.00 (0.08, 12.13)	0.29 (0.03, 2.97)	0.46 (0.05, 4.44)	0.29 (0.02, 3.81)	0.21 (0.02, 2.32)	1.06 (0.06, 17.18)	3.00 (0.23, 43.61)	3.46 (0.26, 46.34)	0.49 (0.06, 3.55)	0.84 (0.08, 7.23)
ELD20mg_kgIV	0.53 (0.06, 3.29)	0.50 (0.06, 3.27)	0.48 (0.05, 2.87)	0.59 (0.05, 6.99)	1.20 (0.04, 50.62)	0.27 (0.03, 1.77)	0.09 (0.00, 1.29)	**0.10 (0.01, 0.97)**	3.85 (0.21, 121.62)	0.55 (0.05, 5.73)	0.06 (0.00, 1.17)	0.74 (0.07, 8.05)	0.23 (0.02, 1.87)	0.35 (0.03, 2.54)	0.23 (0.01, 2.35)	0.16 (0.01, 1.44)	0.79 (0.05, 14.01)	2.20 (0.19, 32.41)	2.47 (0.21, 34.46)	0.37 (0.04, 2.24)	0.62 (0.06, 4.97)
ETA25mgSC	3.18 (0.19, 73.87)	2.93 (0.18, 74.92)	2.74 (0.17, 63.31)	3.68 (0.15, 111.84)	7.32 (0.18, 587.83)	1.54 (0.09, 36.65)	0.54 (0.01, 20.18)	0.60 (0.02, 18.92)	22.88 (0.77, 2276.99)	3.44 (0.17, 95.89)	0.38 (0.00, 15.62)	4.78 (0.24, 127.04)	1.38 (0.07, 33.93)	2.12 (0.11, 53.03)	1.40 (0.05, 42.31)	0.97 (0.04, 28.51)	4.98 (0.18, 187.98)	13.56 (0.59, 480.21)	15.32 (0.72, 555.16)	2.15 (0.14, 53.06)	3.63 (0.22, 93.33)
ETR105mgSC	1.07 (0.32, 3.61)	1.03 (0.28, 3.52)	0.94 (0.28, 3.16)	1.23 (0.17, 8.05)	2.37 (0.16, 78.73)	0.55 (0.13, 1.93)	0.18 (0.01, 1.61)	0.22 (0.02, 1.26)	7.55 (0.83, 194.13)	1.08 (0.20, 7.52)	0.13 (0.00, 1.52)	1.52 (0.28, 10.01)	0.47 (0.08, 2.20)	0.71 (0.15, 2.99)	0.47 (0.05, 3.50)	0.32 (0.05, 2.05)	1.62 (0.18, 15.48)	4.46 (0.74, 38.23)	5.07 (0.87, 43.58)	0.75 (0.22, 2.43)	1.29 (0.29, 4.89)
ETR210mgSC	1.66 (0.45, 6.01)	1.58 (0.40, 5.85)	1.45 (0.40, 5.26)	1.89 (0.26, 13.75)	3.77 (0.23, 120.55)	0.83 (0.19, 3.47)	0.28 (0.01, 2.68)	0.33 (0.03, 2.01)	**11.77 (1.27, 265.32)**	1.69 (0.28, 12.30)	0.20 (0.01, 2.76)	2.32 (0.40, 16.70)	0.72 (0.12, 3.57)	1.10 (0.22, 5.19)	0.73 (0.07, 5.38)	0.49 (0.08, 3.15)	2.47 (0.28, 26.73)	**6.79 (1.13, 61.48)**	**7.66 (1.34, 66.98)**	1.14 (0.32, 4.09)	1.98 (0.42, 8.01)
FIL100mgPO	0.76 (0.27, 2.15)	0.73 (0.24, 2.19)	0.67 (0.23, 1.95)	0.88 (0.15, 5.17)	1.70 (0.12, 60.81)	0.39 (0.11, 1.34)	0.13 (0.00, 1.05)	**0.15 (0.02, 0.81)**	5.34 (0.64, 127.56)	0.77 (0.16, 5.00)	0.09 (0.00, 1.12)	1.09 (0.23, 6.71)	0.32 (0.07, 1.45)	0.51 (0.12, 2.02)	0.35 (0.04, 2.16)	0.23 (0.04, 1.22)	1.15 (0.16, 10.60)	3.15 (0.60, 27.16)	3.58 (0.73, 29.34)	0.53 (0.19, 1.55)	0.91 (0.24, 3.32)
FIL200mgPO	0.87 (0.32, 2.25)	0.83 (0.29, 2.29)	0.77 (0.28, 2.03)	0.99 (0.16, 5.69)	1.94 (0.14, 63.66)	0.44 (0.13, 1.30)	0.15 (0.01, 1.20)	**0.18 (0.02, 0.86)**	6.04 (0.72, 141.50)	0.87 (0.18, 5.28)	0.11 (0.00, 1.22)	1.22 (0.27, 7.14)	0.38 (0.08, 1.53)	0.58 (0.15, 2.07)	0.39 (0.04, 2.45)	0.26 (0.05, 1.36)	1.29 (0.19, 11.40)	3.60 (0.69, 27.50)	4.06 (0.85, 30.06)	0.61 (0.22, 1.58)	1.04 (0.28, 3.49)
FON0_1mg_kgIV	0.93 (0.02, 55.97)	0.87 (0.02, 55.85)	0.80 (0.01, 50.17)	1.04 (0.01, 72.98)	2.14 (0.02, 284.90)	0.45 (0.01, 29.87)	0.15 (0.00, 12.37)	0.18 (0.00, 11.78)	6.76 (0.06, 1018.03)	0.92 (0.01, 63.04)	0.10 (0.00, 10.82)	1.29 (0.02, 84.97)	0.37 (0.01, 25.70)	0.58 (0.01, 38.90)	0.39 (0.00, 29.73)	0.27 (0.00, 17.05)	1.45 (0.02, 107.47)	3.98 (0.05, 304.86)	4.38 (0.06, 325.36)	0.61 (0.01, 42.84)	1.09 (0.02, 61.90)
FON1mg_kgIV	0.58 (0.01, 6.85)	0.55 (0.01, 6.63)	0.50 (0.01, 6.23)	0.64 (0.01, 13.86)	1.32 (0.01, 58.54)	0.29 (0.01, 4.18)	0.09 (0.00, 2.48)	0.11 (0.00, 2.04)	4.38 (0.04, 161.69)	0.58 (0.01, 13.32)	0.06 (0.00, 2.38)	0.80 (0.02, 15.49)	0.24 (0.01, 3.37)	0.37 (0.01, 5.27)	0.24 (0.00, 5.28)	0.17 (0.00, 3.05)	0.86 (0.02, 20.04)	2.42 (0.04, 51.93)	2.78 (0.05, 57.24)	0.41 (0.01, 5.15)	0.70 (0.01, 8.79)
FON4mg_kgIV	0.66 (0.01, 10.37)	0.62 (0.01, 9.41)	0.59 (0.01, 9.56)	0.72 (0.01, 19.71)	1.39 (0.02, 76.74)	0.33 (0.01, 5.37)	0.10 (0.00, 3.25)	0.12 (0.00, 2.58)	4.88 (0.05, 260.38)	0.65 (0.01, 21.82)	0.07 (0.00, 2.76)	0.90 (0.02, 24.30)	0.28 (0.01, 5.00)	0.42 (0.01, 7.31)	0.27 (0.00, 6.21)	0.19 (0.00, 3.71)	0.97 (0.02, 27.97)	2.68 (0.04, 77.69)	2.98 (0.05, 86.33)	0.45 (0.01, 6.69)	0.75 (0.02, 13.90)
GUS1200mgIV	6.22 (0.67, 218.45)	5.90 (0.63, 200.01)	5.45 (0.59, 208.15)	7.21 (0.78, 223.09)	15.47 (0.51, 1942.11)	3.21 (0.28, 109.52)	1.04 (0.02, 71.25)	1.27 (0.06, 54.59)	**51.13 (2.48, 3287.44)**	6.83 (0.42, 379.62)	0.81 (0.01, 52.27)	9.10 (0.61, 445.20)	2.75 (0.20, 128.91)	4.20 (0.36, 199.23)	2.97 (0.14, 115.16)	1.92 (0.13, 74.35)	9.67 (0.59, 561.94)	**26.17 (1.94, 1609.18)**	**29.97 (2.31, 1761.08)**	4.24 (0.45, 155.71)	7.36 (0.67, 332.38)
GUS200mgIV	1.65 (0.28, 10.30)	1.59 (0.27, 10.42)	1.48 (0.25, 9.45)	1.89 (0.33, 12.65)	3.75 (0.17, 145.99)	0.84 (0.12, 6.04)	0.27 (0.01, 3.94)	0.33 (0.03, 3.33)	12.07 (0.94, 447.16)	1.75 (0.18, 18.88)	0.20 (0.00, 3.38)	2.39 (0.27, 24.74)	0.72 (0.09, 5.80)	1.10 (0.15, 8.61)	0.75 (0.05, 8.18)	0.50 (0.06, 4.80)	2.58 (0.21, 38.45)	7.10 (0.78, 93.98)	8.25 (0.88, 103.38)	1.18 (0.20, 7.78)	2.01 (0.28, 14.49)
GUS600mgIV	1.15 (0.22, 5.73)	1.10 (0.20, 5.76)	1.02 (0.20, 5.22)	1.34 (0.25, 6.77)	2.58 (0.14, 116.31)	0.60 (0.10, 3.20)	0.19 (0.01, 2.50)	0.23 (0.02, 1.87)	8.28 (0.68, 220.48)	1.24 (0.14, 11.41)	0.14 (0.00, 2.14)	1.68 (0.20, 14.47)	0.50 (0.06, 3.62)	0.78 (0.11, 4.94)	0.51 (0.04, 5.06)	0.34 (0.04, 2.94)	1.77 (0.17, 23.85)	4.84 (0.57, 59.54)	5.51 (0.67, 63.65)	0.80 (0.16, 4.02)	1.38 (0.22, 8.48)
MED700IV	1.10 (0.25, 4.84)	1.05 (0.22, 4.80)	0.98 (0.22, 4.29)	1.29 (0.16, 10.22)	2.48 (0.13, 82.25)	0.56 (0.11, 2.82)	0.18 (0.01, 2.12)	0.22 (0.02, 1.60)	7.82 (0.68, 241.60)	1.14 (0.17, 8.98)	0.13 (0.00, 1.89)	1.54 (0.24, 13.17)	0.47 (0.07, 2.75)	0.72 (0.12, 4.04)	0.48 (0.04, 3.96)	0.33 (0.05, 2.45)	1.68 (0.17, 19.29)	4.66 (0.58, 47.67)	5.25 (0.69, 53.86)	0.77 (0.17, 3.39)	1.30 (0.25, 7.10)
MIR1000mgIV	4.56 (0.89, 55.51)	4.42 (0.78, 53.67)	3.99 (0.77, 47.94)	5.29 (0.58, 90.16)	11.00 (0.56, 490.65)	2.37 (0.39, 26.71)	0.78 (0.02, 17.05)	0.92 (0.08, 12.67)	**34.77 (2.41, 1471.04)**	4.94 (0.59, 69.96)	0.56 (0.01, 12.37)	6.70 (0.83, 100.51)	1.99 (0.26, 25.46)	3.03 (0.44, 38.86)	2.09 (0.16, 31.93)	1.40 (0.15, 22.88)	6.85 (0.65, 165.85)	**19.83 (2.11, 476.53)**	**22.41 (2.33, 519.69)**	3.18 (0.58, 38.64)	5.55 (0.80, 74.21)
MIR200mgIV	5.44 (0.65, 146.62)	5.28 (0.58, 146.08)	4.86 (0.55, 128.27)	6.09 (0.47, 203.21)	13.11 (0.50, 1230.22)	2.79 (0.29, 80.62)	0.95 (0.02, 35.31)	1.09 (0.06, 39.62)	**44.19 (1.85, 2678.17)**	5.74 (0.42, 183.72)	0.66 (0.01, 37.73)	7.89 (0.73, 249.22)	2.33 (0.21, 80.06)	3.68 (0.35, 108.74)	2.44 (0.13, 101.39)	1.67 (0.12, 57.89)	8.61 (0.53, 367.22)	**23.87 (1.67, 982.32)**	**27.25 (1.92, 1050.49)**	3.71 (0.44, 96.77)	6.62 (0.62, 174.18)

	ABA10mg_kgIV	ABA30mg_kgIV	ABA3mg_kgIV	ADA160mg80mg40mgSC	ADA160mg80mg60mgSC	ADA160mg80mgSC	ADA40mg20mgSC	ADA80mg40mgSC	AMI0_4mgPO	AND150mgSC1_1	AND150mgSC2_2	AND300mgSC	API100mgPR	API50mgPO	BRI400mgIV	BRI700mgIV	BRO210mgIV	BRO350mgIV	BRO700mgIV	CDP10mg_kgIV	CER100mgSC
MIR600mgIV	1.39 (0.33, 9.22)	1.34 (0.28, 8.85)	1.22 (0.28, 8.36)	1.61 (0.20, 16.35)	3.19 (0.18, 112.43)	0.71 (0.14, 5.19)	0.23 (0.01, 3.22)	0.29 (0.02, 2.52)	10.67 (0.80, 279.69)	1.46 (0.20, 14.89)	0.17 (0.00, 3.17)	2.00 (0.29, 20.87)	0.60 (0.09, 5.07)	0.93 (0.15, 6.68)	0.63 (0.05, 6.97)	0.43 (0.06, 4.19)	2.18 (0.22, 29.70)	6.02 (0.82, 72.15)	6.89 (0.89, 78.98)	0.97 (0.22, 6.49)	1.66 (0.29, 12.06)
NAT300mgIV	1.42 (0.60, 3.40)	1.34 (0.52, 3.56)	1.25 (0.52, 3.08)	1.62 (0.29, 9.05)	3.11 (0.24, 102.63)	0.72 (0.23, 1.97)	0.24 (0.01, 1.82)	0.29 (0.03, 1.29)	**9.91 (1.29, 238.24)**	1.43 (0.32, 8.32)	0.17 (0.01, 1.80)	1.99 (0.46, 11.18)	0.61 (0.14, 2.32)	0.94 (0.25, 3.22)	0.63 (0.08, 3.70)	0.43 (0.09, 2.13)	2.10 (0.33, 18.75)	**5.82 (1.23, 43.56)**	**6.63 (1.46, 50.30)**	0.99 (0.40, 2.38)	1.70 (0.51, 5.29)
NAT3mg_kgIV	1.23 (0.32, 4.47)	1.19 (0.29, 4.42)	1.09 (0.29, 3.91)	1.39 (0.20, 9.46)	2.65 (0.17, 94.09)	0.61 (0.14, 2.65)	0.20 (0.01, 2.00)	0.24 (0.02, 1.58)	8.66 (0.87, 226.41)	1.26 (0.20, 9.77)	0.15 (0.00, 1.81)	1.73 (0.30, 12.37)	0.52 (0.09, 2.72)	0.80 (0.16, 4.04)	0.55 (0.05, 4.08)	0.37 (0.05, 2.33)	1.85 (0.21, 19.61)	5.12 (0.75, 47.15)	5.74 (0.96, 54.40)	0.84 (0.23, 3.15)	1.44 (0.30, 6.48)
NAT3mg_kgIVx2	1.40 (0.35, 5.46)	1.32 (0.32, 5.36)	1.24 (0.31, 4.92)	1.59 (0.21, 11.33)	3.10 (0.18, 96.62)	0.69 (0.15, 3.12)	0.23 (0.01, 2.26)	0.28 (0.03, 1.81)	9.80 (0.98, 261.50)	1.43 (0.21, 10.91)	0.16 (0.00, 2.40)	1.95 (0.33, 14.87)	0.60 (0.10, 3.11)	0.90 (0.18, 4.68)	0.61 (0.06, 4.96)	0.41 (0.06, 2.95)	2.06 (0.23, 21.42)	5.80 (0.85, 51.37)	**6.50 (1.01, 62.22)**	0.96 (0.25, 3.74)	1.64 (0.34, 8.11)
NAT6mg_kgIVx2	1.06 (0.27, 4.38)	1.02 (0.24, 4.30)	0.93 (0.24, 3.82)	1.22 (0.16, 9.52)	2.38 (0.15, 77.77)	0.53 (0.12, 2.68)	0.18 (0.01, 1.91)	0.21 (0.02, 1.37)	7.77 (0.78, 186.44)	1.11 (0.17, 8.48)	0.13 (0.00, 1.90)	1.48 (0.25, 11.63)	0.46 (0.08, 2.48)	0.70 (0.14, 3.65)	0.49 (0.04, 3.62)	0.32 (0.05, 2.24)	1.58 (0.17, 18.08)	4.48 (0.67, 43.43)	5.05 (0.77, 50.59)	0.74 (0.19, 2.97)	1.27 (0.27, 6.12)
NNC2mg_kgSC	0.41 (0.05, 2.48)	0.39 (0.05, 2.45)	0.36 (0.05, 2.23)	0.48 (0.04, 4.78)	0.94 (0.03, 42.71)	0.20 (0.02, 1.51)	0.06 (0.00, 1.02)	**0.08 (0.00, 0.80)**	3.04 (0.17, 92.71)	0.42 (0.04, 4.56)	**0.05 (0.00, 0.92)**	0.60 (0.05, 5.69)	0.18 (0.01, 1.49)	0.28 (0.03, 1.98)	0.18 (0.01, 1.84)	0.12 (0.01, 1.08)	0.61 (0.04, 9.05)	1.70 (0.14, 23.18)	1.96 (0.16, 24.72)	0.29 (0.04, 1.74)	0.48 (0.05, 3.48)
ONE10mgSC	1.46 (0.04, 36.82)	1.35 (0.04, 37.85)	1.30 (0.03, 33.91)	1.67 (0.03, 51.10)	3.34 (0.04, 302.64)	0.72 (0.02, 19.87)	0.23 (0.00, 8.90)	0.28 (0.00, 9.29)	11.13 (0.19, 638.46)	1.52 (0.03, 48.97)	0.16 (0.00, 11.29)	2.14 (0.05, 77.66)	0.64 (0.01, 17.34)	0.98 (0.02, 24.43)	0.62 (0.01, 22.24)	0.42 (0.01, 14.81)	2.34 (0.04, 102.77)	6.30 (0.12, 238.03)	7.21 (0.13, 274.50)	1.02 (0.03, 25.60)	1.79 (0.04, 50.63)
ONE25mgSC	1.19 (0.03, 45.18)	1.13 (0.03, 47.80)	1.06 (0.03, 41.55)	1.41 (0.03, 68.75)	2.90 (0.03, 271.93)	0.61 (0.02, 23.13)	0.18 (0.00, 11.50)	0.23 (0.00, 10.56)	9.19 (0.16, 848.24)	1.28 (0.02, 56.68)	0.14 (0.00, 9.27)	1.75 (0.04, 81.00)	0.51 (0.01, 22.39)	0.76 (0.02, 33.62)	0.51 (0.01, 27.32)	0.36 (0.01, 17.88)	1.80 (0.04, 108.00)	5.01 (0.11, 264.92)	5.88 (0.14, 300.28)	0.83 (0.02, 31.55)	1.45 (0.04, 56.18)
ONE35mgSC	0.30 (0.01, 3.46)	0.29 (0.01, 3.57)	0.27 (0.01, 3.16)	0.36 (0.01, 6.21)	0.69 (0.01, 41.35)	0.15 (0.01, 1.98)	0.04 (0.00, 1.35)	0.06 (0.00, 1.04)	2.23 (0.07, 84.78)	0.33 (0.01, 5.59)	0.03 (0.00, 1.02)	0.44 (0.01, 7.81)	0.13 (0.00, 1.90)	0.20 (0.01, 2.77)	0.13 (0.00, 2.55)	0.09 (0.00, 1.52)	0.45 (0.01, 12.05)	1.24 (0.04, 29.31)	1.45 (0.05, 33.66)	0.21 (0.01, 2.54)	0.36 (0.01, 4.59)
ONE50mgSC	1.39 (0.04, 104.09)	1.30 (0.04, 91.69)	1.25 (0.03, 87.37)	1.61 (0.03, 102.82)	3.18 (0.04, 789.28)	0.69 (0.02, 50.04)	0.21 (0.00, 26.04)	0.26 (0.01, 21.37)	10.94 (0.20, 1286.80)	1.50 (0.03, 152.41)	0.16 (0.00, 23.72)	2.05 (0.04, 182.19)	0.58 (0.01, 43.24)	0.89 (0.03, 56.54)	0.59 (0.01, 46.94)	0.41 (0.01, 26.60)	2.11 (0.05, 265.34)	5.89 (0.17, 537.95)	6.72 (0.19, 717.02)	0.96 (0.03, 55.90)	1.63 (0.05, 118.98)
ONT22_5mgSC	0.51 (0.12, 1.81)	0.48 (0.11, 1.84)	0.45 (0.11, 1.61)	0.57 (0.08, 4.07)	1.12 (0.07, 39.03)	0.26 (0.05, 1.01)	**0.09 (0.00, 0.83)**	**0.10 (0.01, 0.59)**	3.48 (0.35, 95.22)	0.52 (0.08, 3.83)	**0.06 (0.00, 0.90)**	0.72 (0.11, 5.23)	0.21 (0.04, 1.10)	0.33 (0.07, 1.52)	0.22 (0.02, 1.62)	0.15 (0.02, 1.00)	0.76 (0.08, 7.33)	2.12 (0.28, 18.24)	2.40 (0.33, 20.97)	0.35 (0.09, 1.27)	0.60 (0.12, 2.57)
ONT225mgSC	0.52 (0.13, 1.81)	0.50 (0.12, 1.80)	0.46 (0.12, 1.64)	0.59 (0.08, 4.10)	1.15 (0.07, 38.46)	0.27 (0.06, 1.02)	**0.09 (0.00, 0.86)**	**0.10 (0.01, 0.64)**	3.67 (0.35, 97.29)	0.54 (0.08, 3.58)	**0.06 (0.00, 0.83)**	0.73 (0.12, 4.88)	0.22 (0.04, 1.19)	0.34 (0.07, 1.61)	0.22 (0.02, 1.60)	**0.15 (0.02, 0.98)**	0.79 (0.09, 7.85)	2.19 (0.32, 19.70)	2.46 (0.36, 21.90)	0.36 (0.09, 1.31)	0.62 (0.12, 2.63)
ONT75mgSC	0.62 (0.14, 2.37)	0.59 (0.13, 2.25)	0.54 (0.13, 2.09)	0.70 (0.09, 5.01)	1.38 (0.08, 45.29)	0.31 (0.06, 1.27)	0.10 (0.00, 1.04)	**0.12 (0.01, 0.76)**	4.36 (0.40, 117.28)	0.63 (0.09, 4.84)	0.07 (0.00, 1.01)	0.87 (0.14, 6.37)	0.26 (0.04, 1.41)	0.40 (0.08, 1.90)	0.27 (0.03, 1.97)	0.19 (0.03, 1.18)	0.93 (0.10, 9.23)	2.57 (0.34, 22.99)	2.90 (0.41, 26.45)	0.43 (0.10, 1.56)	0.73 (0.14, 3.40)
PBO	1.11 (0.55, 2.25)	1.06 (0.48, 2.34)	0.98 (0.48, 2.04)	1.26 (0.24, 6.42)	2.48 (0.19, 77.67)	0.58 (0.21, 1.35)	0.19 (0.01, 1.34)	**0.23 (0.03, 0.95)**	**7.60 (1.08, 177.64)**	1.11 (0.27, 5.69)	0.13 (0.00, 1.41)	1.57 (0.40, 7.83)	0.48 (0.12, 1.62)	0.74 (0.22, 2.23)	0.50 (0.06, 2.76)	0.33 (0.07, 1.52)	1.64 (0.27, 13.37)	**4.54 (1.04, 32.37)**	**5.16 (1.29, 35.63)**	0.77 (0.38, 1.61)	1.34 (0.45, 3.63)
PF10mgSC	1.41 (0.41, 4.98)	1.35 (0.36, 5.08)	1.26 (0.36, 4.48)	1.62 (0.24, 11.55)	3.14 (0.20, 113.82)	0.72 (0.16, 2.82)	0.24 (0.01, 2.17)	0.29 (0.03, 1.75)	**10.04 (1.10, 278.92)**	1.45 (0.25, 10.46)	0.17 (0.00, 2.18)	1.99 (0.36, 13.12)	0.60 (0.11, 3.06)	0.93 (0.19, 4.55)	0.63 (0.06, 4.60)	0.43 (0.07, 2.81)	2.12 (0.26, 22.89)	5.94 (0.95, 55.07)	**6.73 (1.05, 62.61)**	0.99 (0.28, 3.46)	1.69 (0.34, 7.31)
PF200mgSC	0.53 (0.16, 1.71)	0.51 (0.14, 1.75)	0.47 (0.13, 1.51)	0.61 (0.09, 4.00)	1.21 (0.08, 36.98)	**0.27 (0.07, 0.97)**	**0.09 (0.00, 0.77)**	**0.11 (0.01, 0.61)**	3.74 (0.41, 99.26)	0.54 (0.10, 3.74)	**0.06 (0.00, 0.77)**	0.75 (0.14, 4.79)	0.23 (0.04, 1.12)	0.35 (0.07, 1.55)	0.23 (0.03, 1.61)	**0.16 (0.02, 0.94)**	0.78 (0.10, 7.73)	2.21 (0.38, 19.68)	2.51 (0.42, 22.64)	0.37 (0.11, 1.17)	0.63 (0.14, 2.45)
PF50mgSC	1.12 (0.34, 3.76)	1.09 (0.29, 3.81)	1.00 (0.29, 3.45)	1.30 (0.20, 8.42)	2.53 (0.16, 83.20)	0.58 (0.14, 2.11)	0.19 (0.01, 1.87)	0.22 (0.02, 1.37)	7.88 (0.90, 224.75)	1.15 (0.22, 8.42)	0.14 (0.00, 1.74)	1.59 (0.30, 10.86)	0.47 (0.09, 2.39)	0.74 (0.16, 3.45)	0.51 (0.05, 3.71)	0.35 (0.05, 2.12)	1.68 (0.20, 16.98)	4.61 (0.80, 44.31)	5.33 (0.88, 48.70)	0.79 (0.22, 2.66)	1.34 (0.30, 5.73)
RIS1200mgIV	**4.10 (1.61, 9.99)**	**3.92 (1.45, 10.38)**	**3.60 (1.43, 9.17)**	4.73 (0.82, 25.66)	9.12 (0.64, 294.62)	2.08 (0.66, 6.16)	0.71 (0.03, 5.25)	0.84 (0.10, 3.82)	**28.46 (3.57, 674.67)**	4.16 (0.88, 23.91)	0.50 (0.01, 5.50)	**5.81 (1.33, 31.05)**	1.78 (0.38, 6.87)	2.71 (0.70, 9.47)	1.85 (0.22, 10.44)	1.22 (0.26, 6.23)	6.04 (0.89, 54.12)	**16.85 (3.46, 132.40)**	**19.10 (4.13, 146.78)**	**2.84 (1.12, 7.03)**	**4.90 (1.42, 15.35)**
RIS200mgIV	1.68 (0.50, 6.32)	1.61 (0.45, 6.40)	1.48 (0.45, 5.44)	1.97 (0.29, 14.25)	3.83 (0.25, 124.64)	0.85 (0.21, 3.62)	0.29 (0.01, 2.80)	0.34 (0.03, 2.01)	**12.30 (1.27, 316.61)**	1.73 (0.31, 11.99)	0.20 (0.01, 2.80)	2.38 (0.44, 16.76)	0.74 (0.14, 3.66)	1.14 (0.23, 5.20)	0.75 (0.08, 5.77)	0.51 (0.09, 3.17)	2.52 (0.32, 28.66)	**7.05 (1.18, 63.51)**	**7.91 (1.40, 76.53)**	1.18 (0.34, 4.24)	2.01 (0.46, 9.40)
RIS600mgIV	**2.61 (1.12, 6.13)**	2.48 (0.99, 6.29)	2.30 (0.99, 5.56)	3.00 (0.53, 16.37)	5.80 (0.43, 185.09)	1.34 (0.44, 3.74)	0.45 (0.02, 3.35)	0.53 (0.06, 2.36)	**18.11 (2.34, 408.87)**	2.64 (0.60, 15.53)	0.32 (0.01, 3.36)	3.71 (0.87, 19.60)	1.15 (0.26, 4.57)	1.75 (0.46, 5.84)	1.15 (0.14, 6.80)	0.78 (0.17, 4.00)	3.85 (0.61, 35.22)	**10.61 (2.28, 81.14)**	**12.02 (2.82, 89.86)**	1.80 (0.77, 4.36)	3.14 (0.92, 9.80)
SEC10mg_kgIV	0.88 (0.16, 3.72)	0.84 (0.14, 3.65)	0.77 (0.14, 3.31)	0.99 (0.11, 8.24)	1.93 (0.10, 77.17)	0.44 (0.07, 2.09)	0.15 (0.00, 1.49)	0.17 (0.01, 1.16)	6.06 (0.40, 183.08)	0.90 (0.11, 7.49)	0.10 (0.00, 1.58)	1.22 (0.16, 9.82)	0.37 (0.05, 2.16)	0.57 (0.08, 3.07)	0.37 (0.03, 3.07)	0.26 (0.03, 1.89)	1.30 (0.12, 14.10)	3.58 (0.44, 36.83)	4.01 (0.50, 39.49)	0.61 (0.11, 2.58)	1.04 (0.14, 5.43)

	ABA10mg_kgIV	ABA30mg_kgIV	ABA3mg_kgIV	ADA160mg80mg40mgSC	ADA160mg80mg60mgSC	ADA160mg80mgSC	ADA40mg20mgSC	ADA80mg40mgSC	AMI0_4mgPO	AND150mgSC1_1	AND150mgSC2_2	AND300mgSC	API100mgPR	API50mgPO	BRI400mgIV	BRI700mgIV	BRO210mgIV	BRO350mgIV	BRO700mgIV	CDP10mg_kgIV	CER100mgSC
TES400mg200mgSC	**0.10 (0.00, 0.76)**	**0.09 (0.00, 0.75)**	**0.09 (0.00, 0.70)**	0.11 (0.00, 1.42)	0.22 (0.00, 8.94)	**0.05 (0.00, 0.41)**	**0.01 (0.00, 0.29)**	**0.02 (0.00, 0.22)**	0.69 (0.02, 25.29)	0.10 (0.00, 1.42)	**0.01 (0.00, 0.27)**	0.13 (0.01, 1.81)	**0.04 (0.00, 0.44)**	**0.06 (0.00, 0.65)**	**0.04 (0.00, 0.57)**	**0.03 (0.00, 0.37)**	0.14 (0.00, 2.48)	0.41 (0.02, 6.06)	0.46 (0.02, 6.84)	**0.07 (0.00, 0.54)**	0.12 (0.01, 1.05)
TOF15mgPO_BID	7.19 (0.94, 171.35)	6.84 (0.84, 173.67)	6.41 (0.83, 153.62)	8.76 (0.68, 261.03)	19.04 (0.59, 1387.38)	3.65 (0.43, 102.68)	1.31 (0.03, 45.28)	1.52 (0.08, 40.83)	**62.57 (3.06, 3195.41)**	8.09 (0.65, 238.95)	0.89 (0.01, 43.15)	10.94 (0.96, 360.63)	3.26 (0.29, 81.56)	4.94 (0.50, 116.82)	3.27 (0.20, 92.41)	2.29 (0.20, 62.87)	11.76 (0.76, 487.57)	**32.62 (2.60, 1025.67)**	**36.85 (2.96, 1358.64)**	5.13 (0.67, 122.59)	8.94 (0.92, 212.98)
TOF1mgPO_BID	1.53 (0.33, 6.94)	1.46 (0.29, 7.15)	1.35 (0.29, 6.20)	1.72 (0.21, 15.91)	3.48 (0.19, 131.04)	0.78 (0.14, 4.13)	0.26 (0.01, 2.99)	0.31 (0.03, 2.25)	10.73 (0.93, 309.84)	1.54 (0.21, 14.80)	0.19 (0.00, 2.63)	2.16 (0.31, 18.83)	0.67 (0.09, 4.37)	1.01 (0.17, 6.16)	0.67 (0.06, 5.83)	0.45 (0.06, 3.52)	2.24 (0.24, 28.32)	6.38 (0.79, 69.05)	7.28 (1.00, 82.66)	1.04 (0.22, 5.10)	1.80 (0.31, 10.13)
TOF5mgPO_BID	1.45 (0.32, 6.85)	1.37 (0.29, 6.81)	1.28 (0.28, 5.86)	1.61 (0.19, 14.20)	3.27 (0.17, 138.71)	0.73 (0.13, 3.93)	0.25 (0.01, 2.40)	0.29 (0.03, 2.13)	10.31 (0.88, 311.51)	1.50 (0.18, 13.01)	0.18 (0.00, 2.65)	2.07 (0.29, 17.76)	0.62 (0.09, 3.96)	0.93 (0.17, 5.48)	0.63 (0.05, 5.31)	0.43 (0.06, 3.24)	2.21 (0.22, 26.46)	6.11 (0.80, 64.77)	7.02 (0.96, 71.85)	1.00 (0.21, 4.86)	1.73 (0.32, 9.44)
UPA12mgPO_BID	**0.19 (0.03, 0.89)**	**0.18 (0.02, 0.90)**	**0.17 (0.02, 0.81)**	0.21 (0.02, 1.78)	0.41 (0.02, 16.86)	**0.10 (0.01, 0.50)**	**0.03 (0.00, 0.35)**	**0.04 (0.00, 0.30)**	1.38 (0.07, 40.10)	0.20 (0.02, 1.75)	**0.02 (0.00, 0.38)**	0.27 (0.03, 2.40)	**0.08 (0.01, 0.56)**	**0.13 (0.01, 0.76)**	**0.08 (0.01, 0.73)**	**0.06 (0.00, 0.44)**	0.27 (0.02, 3.68)	0.78 (0.07, 8.60)	0.88 (0.08, 10.27)	**0.13 (0.02, 0.65)**	0.23 (0.03, 1.24)
UPA24mgPO	0.28 (0.03, 1.43)	0.26 (0.03, 1.48)	0.24 (0.03, 1.27)	0.31 (0.02, 2.84)	0.60 (0.03, 24.21)	**0.14 (0.02, 0.80)**	**0.05 (0.00, 0.55)**	**0.05 (0.00, 0.44)**	1.97 (0.10, 57.95)	0.28 (0.02, 2.56)	**0.03 (0.00, 0.53)**	0.39 (0.04, 3.61)	**0.12 (0.01, 0.81)**	0.18 (0.02, 1.14)	0.12 (0.01, 1.18)	**0.08 (0.01, 0.70)**	0.40 (0.03, 5.94)	1.13 (0.10, 14.21)	1.29 (0.11, 16.24)	0.19 (0.02, 1.02)	0.33 (0.03, 1.88)
UPA24mgPO_BID	0.71 (0.09, 5.30)	0.66 (0.08, 5.19)	0.61 (0.07, 4.93)	0.79 (0.06, 9.06)	1.52 (0.06, 76.99)	0.34 (0.04, 2.85)	0.12 (0.00, 1.82)	0.14 (0.01, 1.47)	5.35 (0.23, 178.56)	0.74 (0.06, 8.68)	0.08 (0.00, 1.80)	0.97 (0.09, 11.66)	0.31 (0.03, 3.09)	0.47 (0.04, 4.23)	0.31 (0.02, 3.88)	0.21 (0.02, 2.30)	1.05 (0.06, 18.24)	2.87 (0.25, 42.63)	3.26 (0.28, 48.76)	0.48 (0.06, 3.72)	0.83 (0.09, 7.33)
UPA3mgPO_BID	0.45 (0.05, 2.74)	0.42 (0.05, 2.80)	0.39 (0.05, 2.51)	0.51 (0.04, 4.79)	0.95 (0.04, 43.64)	0.22 (0.03, 1.54)	0.07 (0.00, 1.06)	**0.09 (0.01, 0.75)**	3.30 (0.15, 98.35)	0.46 (0.04, 5.16)	0.05 (0.00, 1.03)	0.62 (0.06, 6.48)	0.19 (0.02, 1.42)	0.29 (0.03, 2.07)	0.19 (0.01, 2.13)	0.13 (0.01, 1.23)	0.63 (0.04, 11.85)	1.77 (0.16, 25.10)	2.05 (0.19, 30.48)	0.31 (0.04, 1.85)	0.52 (0.05, 3.94)
UPA45mgPO	1.14 (0.47, 2.75)	1.10 (0.41, 2.78)	1.01 (0.41, 2.53)	1.31 (0.23, 7.17)	2.53 (0.18, 81.12)	0.59 (0.19, 1.60)	0.19 (0.01, 1.49)	0.23 (0.03, 1.08)	**7.90 (1.02, 189.27)**	1.16 (0.26, 6.59)	0.14 (0.00, 1.49)	1.63 (0.37, 8.67)	0.49 (0.11, 1.85)	0.76 (0.20, 2.63)	0.52 (0.06, 3.05)	0.34 (0.07, 1.68)	1.71 (0.26, 14.26)	4.71 (0.99, 36.01)	**5.33 (1.22, 39.98)**	0.79 (0.32, 1.91)	1.38 (0.40, 4.21)
UPA6mgPO_BID	1.18 (0.11, 12.50)	1.13 (0.11, 13.77)	1.04 (0.10, 11.85)	1.36 (0.08, 21.08)	2.64 (0.09, 186.79)	0.59 (0.05, 7.84)	0.21 (0.00, 4.79)	0.24 (0.01, 3.56)	8.90 (0.33, 401.30)	1.27 (0.08, 23.70)	0.14 (0.00, 3.66)	1.73 (0.11, 28.94)	0.51 (0.04, 7.78)	0.79 (0.06, 10.92)	0.50 (0.03, 9.88)	0.35 (0.02, 5.73)	1.79 (0.11, 38.14)	5.07 (0.33, 100.36)	5.71 (0.40, 108.76)	0.82 (0.08, 10.17)	1.43 (0.12, 17.42)
UST130mgIV	1.39 (0.54, 3.52)	1.32 (0.49, 3.64)	1.22 (0.48, 3.16)	1.59 (0.27, 9.08)	3.11 (0.23, 99.17)	0.71 (0.23, 2.02)	0.24 (0.01, 1.83)	0.28 (0.03, 1.30)	**9.61 (1.18, 225.47)**	1.39 (0.30, 8.23)	0.17 (0.01, 1.86)	1.95 (0.44, 10.81)	0.61 (0.13, 2.33)	0.93 (0.25, 3.16)	0.62 (0.07, 3.79)	0.42 (0.08, 2.08)	2.06 (0.31, 18.50)	**5.75 (1.16, 44.60)**	**6.49 (1.41, 50.21)**	0.96 (0.38, 2.46)	1.67 (0.46, 5.32)
UST1mg_kgIV	2.06 (0.64, 7.46)	1.95 (0.57, 7.70)	1.82 (0.55, 6.69)	2.40 (0.34, 16.72)	4.73 (0.29, 160.13)	1.05 (0.26, 4.43)	0.35 (0.01, 3.58)	0.42 (0.04, 2.55)	**14.59 (1.63, 359.59)**	2.17 (0.38, 16.80)	0.25 (0.01, 3.59)	2.99 (0.54, 21.30)	0.90 (0.17, 4.68)	1.37 (0.30, 6.43)	0.94 (0.09, 6.34)	0.62 (0.11, 3.89)	3.08 (0.39, 31.98)	**8.59 (1.49, 83.18)**	**9.96 (1.63, 90.10)**	1.42 (0.44, 5.40)	2.44 (0.58, 11.15)
UST3mg_kgIV	1.56 (0.49, 5.17)	1.49 (0.44, 5.29)	1.39 (0.43, 4.62)	1.78 (0.27, 11.65)	3.52 (0.23, 119.73)	0.79 (0.22, 3.04)	0.27 (0.01, 2.33)	0.32 (0.03, 1.81)	**11.14 (1.30, 269.22)**	1.58 (0.30, 11.71)	0.19 (0.01, 2.52)	2.23 (0.42, 15.86)	0.67 (0.13, 3.17)	1.04 (0.24, 4.51)	0.71 (0.07, 4.63)	0.47 (0.08, 2.70)	2.34 (0.32, 23.89)	**6.54 (1.15, 56.05)**	**7.49 (1.35, 60.59)**	1.07 (0.35, 3.79)	1.87 (0.45, 7.75)
UST4_5mg_kgIV	0.43 (0.01, 6.37)	0.41 (0.01, 6.14)	0.37 (0.01, 5.63)	0.49 (0.01, 11.11)	0.95 (0.01, 62.07)	0.21 (0.01, 3.39)	0.07 (0.00, 1.78)	0.08 (0.00, 1.58)	3.14 (0.05, 176.33)	0.44 (0.01, 11.59)	0.05 (0.00, 1.90)	0.58 (0.02, 15.06)	0.18 (0.01, 3.21)	0.28 (0.01, 4.77)	0.17 (0.00, 4.55)	0.13 (0.00, 2.50)	0.62 (0.01, 19.14)	1.80 (0.04, 47.60)	2.00 (0.05, 56.33)	0.30 (0.01, 4.31)	0.51 (0.01, 7.67)
UST6mg_kg90mgIV_SC	1.09 (0.22, 5.74)	1.04 (0.20, 5.70)	0.97 (0.19, 5.18)	1.26 (0.65, 2.40)	2.42 (0.13, 99.15)	0.56 (0.09, 3.25)	0.18 (0.01, 2.20)	0.22 (0.02, 1.77)	8.04 (0.62, 209.25)	1.13 (0.13, 11.63)	0.13 (0.00, 2.58)	1.52 (0.18, 15.54)	0.47 (0.07, 3.36)	0.72 (0.12, 4.51)	0.48 (0.04, 4.93)	0.32 (0.04, 2.89)	1.66 (0.14, 21.46)	4.64 (0.53, 54.94)	5.31 (0.60, 59.31)	0.77 (0.15, 3.97)	1.30 (0.20, 8.47)
UST6mg_kgIV	1.30 (0.54, 3.16)	1.23 (0.48, 3.23)	1.14 (0.48, 2.80)	1.50 (0.27, 8.21)	2.91 (0.22, 87.47)	0.67 (0.22, 1.86)	0.23 (0.01, 1.76)	0.27 (0.03, 1.20)	**8.98 (1.16, 221.49)**	1.30 (0.31, 7.48)	0.16 (0.00, 1.79)	1.83 (0.45, 10.03)	0.58 (0.12, 2.17)	0.87 (0.24, 2.99)	0.59 (0.07, 3.42)	0.39 (0.08, 1.91)	1.94 (0.29, 16.52)	**5.42 (1.12, 39.76)**	**6.09 (1.38, 44.35)**	0.90 (0.38, 2.25)	1.56 (0.46, 4.94)
UST90mgSC	2.33 (0.13, 61.96)	2.20 (0.13, 64.88)	2.06 (0.12, 56.55)	2.67 (0.13, 106.61)	5.41 (0.12, 595.74)	1.20 (0.07, 33.78)	0.39 (0.01, 17.53)	0.47 (0.02, 14.30)	18.57 (0.64, 1391.20)	2.55 (0.11, 87.39)	0.27 (0.00, 13.28)	3.41 (0.16, 123.50)	1.00 (0.05, 28.85)	1.53 (0.08, 42.81)	1.09 (0.04, 38.43)	0.72 (0.03, 25.73)	3.66 (0.14, 208.18)	10.05 (0.43, 503.02)	11.24 (0.46, 593.50)	1.61 (0.10, 45.02)	2.80 (0.14, 92.56)
VED0_5mg_kgIV	1.98 (0.58, 7.83)	1.92 (0.52, 7.83)	1.76 (0.50, 7.19)	2.32 (0.35, 16.70)	4.53 (0.26, 188.53)	1.02 (0.24, 4.33)	0.35 (0.01, 3.71)	0.41 (0.04, 2.55)	**14.25 (1.53, 377.49)**	2.08 (0.34, 15.74)	0.24 (0.01, 3.05)	2.87 (0.52, 22.20)	0.86 (0.15, 5.01)	1.34 (0.26, 7.06)	0.86 (0.09, 6.70)	0.60 (0.10, 3.96)	2.93 (0.38, 33.17)	**8.31 (1.39, 93.64)**	**9.41 (1.68, 106.08)**	1.40 (0.41, 5.77)	2.38 (0.54, 11.78)
VED2mg_kgIV	1.24 (0.39, 3.84)	1.19 (0.33, 4.05)	1.10 (0.34, 3.51)	1.43 (0.21, 9.08)	2.82 (0.18, 94.37)	0.64 (0.16, 2.39)	0.21 (0.01, 1.82)	0.25 (0.03, 1.41)	**8.76 (1.02, 226.68)**	1.27 (0.22, 9.07)	0.15 (0.00, 1.95)	1.77 (0.33, 11.74)	0.53 (0.11, 2.53)	0.81 (0.19, 3.62)	0.54 (0.06, 3.95)	0.37 (0.07, 2.22)	1.83 (0.24, 17.84)	5.08 (0.88, 45.74)	**5.79 (1.10, 53.82)**	0.86 (0.28, 2.86)	1.48 (0.35, 6.00)
VED300mgIV	1.11 (0.47, 2.64)	1.06 (0.41, 2.72)	0.99 (0.41, 2.38)	1.27 (0.23, 6.88)	2.53 (0.17, 77.16)	0.57 (0.19, 1.64)	0.19 (0.01, 1.48)	0.23 (0.03, 1.05)	7.59 (0.99, 175.69)	1.12 (0.25, 6.52)	0.14 (0.00, 1.51)	1.57 (0.36, 8.73)	0.48 (0.11, 1.83)	0.75 (0.21, 2.43)	0.49 (0.06, 2.92)	0.33 (0.07, 1.64)	1.65 (0.25, 13.58)	4.61 (0.95, 33.75)	**5.20 (1.14, 38.93)**	0.77 (0.33, 1.90)	1.33 (0.40, 4.08)

	CER10mgIV	CER200mgSC	CER20mgIV	CER400mgSC	CER5mgIV	ELD10mg_kgIV	ELD20mg_kgIV	ETA25mgSC	ETR105mgSC	ETR210mgSC	FIL100mgPO	FIL200mgPO	FON0_1mg_kgIV	FON1mg_kgIV	FON4mg_kgIV	GUS1200mgIV	GUS200mgIV	GUS600mgIV	MED700IV	MIR1000mgIV	MIR200mgIV
ABA10mg_kgIV	0.57 (0.02, 7.63)	1.78 (0.55, 5.74)	2.77 (0.51, 24.98)	1.21 (0.48, 3.03)	0.35 (0.02, 4.89)	1.42 (0.21, 12.44)	1.88 (0.30, 15.92)	0.31 (0.01, 5.16)	0.93 (0.28, 3.11)	0.60 (0.17, 2.23)	1.31 (0.46, 3.69)	1.15 (0.44, 3.11)	1.08 (0.02, 60.94)	1.73 (0.15, 77.69)	1.51 (0.10, 71.59)	0.16 (0.00, 1.49)	0.61 (0.10, 3.54)	0.87 (0.17, 4.49)	0.91 (0.21, 4.07)	0.22 (0.02, 1.12)	0.18 (0.01, 1.54)
ABA30mg_kgIV	0.60 (0.02, 7.94)	1.87 (0.53, 6.43)	2.91 (0.52, 25.45)	1.28 (0.47, 3.45)	0.36 (0.02, 4.88)	1.49 (0.20, 12.92)	2.00 (0.31, 17.15)	0.34 (0.01, 5.57)	0.97 (0.28, 3.52)	0.63 (0.17, 2.49)	1.37 (0.46, 4.18)	1.21 (0.44, 3.44)	1.15 (0.02, 64.67)	1.82 (0.15, 81.66)	1.62 (0.11, 74.33)	0.17 (0.00, 1.59)	0.63 (0.10, 3.73)	0.91 (0.17, 4.91)	0.96 (0.21, 4.46)	0.23 (0.02, 1.28)	0.19 (0.01, 1.72)
ABA3mg_kgIV	0.64 (0.02, 8.75)	2.03 (0.60, 6.47)	3.07 (0.58, 27.04)	1.38 (0.53, 3.43)	0.39 (0.02, 5.61)	1.64 (0.23, 13.48)	2.09 (0.35, 18.30)	0.37 (0.02, 5.92)	1.06 (0.32, 3.52)	0.69 (0.19, 2.48)	1.49 (0.51, 4.28)	1.30 (0.49, 3.54)	1.25 (0.02, 67.34)	1.99 (0.16, 83.72)	1.71 (0.10, 82.06)	0.18 (0.00, 1.68)	0.68 (0.11, 4.01)	0.98 (0.19, 5.00)	1.03 (0.23, 4.54)	0.25 (0.02, 1.30)	0.21 (0.01, 1.82)
ADA160mg80mg40mgSC	0.49 (0.01, 10.25)	1.56 (0.22, 10.70)	2.46 (0.25, 33.95)	1.06 (0.19, 6.09)	0.31 (0.01, 6.00)	1.30 (0.10, 17.73)	1.71 (0.14, 20.73)	0.27 (0.01, 6.55)	0.81 (0.12, 5.86)	0.53 (0.07, 3.89)	1.14 (0.19, 6.86)	1.01 (0.18, 6.06)	0.96 (0.01, 76.77)	1.57 (0.07, 94.84)	1.38 (0.05, 96.04)	0.14 (0.00, 1.28)	0.53 (0.08, 3.05)	0.75 (0.15, 3.99)	0.78 (0.10, 6.30)	0.19 (0.01, 1.73)	0.16 (0.00, 2.11)
ADA160mg80mg60mgSC	0.23 (0.00, 9.48)	0.79 (0.02, 12.03)	1.24 (0.03, 32.58)	0.54 (0.02, 7.44)	0.14 (0.00, 5.59)	0.64 (0.02, 19.33)	0.83 (0.02, 23.54)	0.14 (0.00, 5.63)	0.42 (0.01, 6.38)	0.27 (0.01, 4.37)	0.59 (0.02, 8.36)	0.52 (0.02, 7.37)	0.47 (0.00, 42.92)	0.76 (0.02, 67.42)	0.72 (0.01, 53.50)	0.06 (0.00, 1.97)	0.27 (0.01, 5.83)	0.39 (0.01, 6.98)	0.40 (0.01, 7.77)	0.09 (0.00, 1.78)	0.08 (0.00, 2.02)
ADA160mg80mgSC	1.08 (0.03, 15.47)	3.45 (0.97, 13.75)	5.48 (0.92, 47.95)	2.35 (0.82, 7.49)	0.70 (0.03, 10.34)	2.92 (0.35, 26.06)	3.71 (0.56, 36.22)	0.65 (0.03, 11.18)	1.82 (0.52, 7.65)	1.20 (0.29, 5.16)	2.54 (0.75, 9.03)	2.25 (0.77, 7.60)	2.21 (0.03, 121.18)	3.39 (0.24, 168.31)	3.06 (0.19, 145.30)	0.31 (0.01, 3.56)	1.19 (0.17, 8.14)	1.67 (0.31, 10.41)	1.79 (0.35, 9.46)	0.42 (0.04, 2.54)	0.36 (0.01, 3.42)
ADA40mg20mgSC	3.30 (0.06, 205.89)	**10.76 (1.18, 329.54)**	**16.78 (1.33, 825.62)**	6.92 (0.93, 192.52)	2.20 (0.05, 128.81)	8.55 (0.56, 418.25)	11.60 (0.78, 504.82)	1.87 (0.05, 146.89)	5.43 (0.62, 183.82)	3.60 (0.37, 124.60)	7.63 (0.96, 233.26)	6.67 (0.83, 198.25)	6.88 (0.08, 751.76)	11.50 (0.40, 1137.98)	10.47 (0.31, 1090.86)	0.96 (0.01, 40.36)	3.71 (0.25, 135.87)	5.29 (0.40, 186.60)	5.55 (0.47, 164.98)	1.28 (0.06, 48.50)	1.06 (0.03, 43.49)
ADA80mg40mgSC	2.89 (0.06, 66.01)	**8.89 (1.55, 81.21)**	**14.45 (1.57, 235.83)**	**5.88 (1.27, 52.57)**	1.77 (0.06, 46.77)	7.32 (0.56, 143.80)	**9.53 (1.03, 180.56)**	1.67 (0.05, 41.17)	4.63 (0.80, 44.78)	3.00 (0.50, 29.99)	**6.53 (1.23, 58.24)**	**5.62 (1.17, 49.67)**	5.70 (0.08, 426.62)	9.09 (0.49, 536.57)	8.35 (0.39, 530.95)	0.79 (0.02, 16.74)	3.02 (0.30, 39.75)	4.36 (0.54, 52.29)	4.52 (0.62, 46.21)	1.09 (0.08, 13.01)	0.92 (0.03, 16.08)
AMI0_4mgPO	0.07 (0.00, 2.26)	0.25 (0.01, 2.27)	0.40 (0.01, 6.73)	0.17 (0.01, 1.34)	0.05 (0.00, 1.35)	0.20 (0.01, 3.46)	0.26 (0.01, 4.71)	0.04 (0.00, 1.29)	0.13 (0.01, 1.20)	**0.08 (0.00, 0.79)**	0.19 (0.01, 1.56)	0.17 (0.01, 1.38)	0.15 (0.00, 17.62)	0.23 (0.01, 25.18)	0.21 (0.00, 19.47)	**0.02 (0.00, 0.40)**	0.08 (0.00, 1.06)	0.12 (0.00, 1.46)	0.13 (0.00, 1.47)	**0.03 (0.00, 0.41)**	**0.02 (0.00, 0.54)**
AND150mgSC1_1	0.52 (0.01, 9.81)	1.76 (0.26, 9.28)	2.76 (0.29, 33.90)	1.22 (0.20, 5.34)	0.33 (0.01, 6.71)	1.37 (0.11, 16.37)	1.83 (0.17, 19.98)	0.29 (0.01, 6.05)	0.93 (0.13, 4.90)	0.59 (0.08, 3.60)	1.30 (0.20, 6.09)	1.14 (0.19, 5.47)	1.09 (0.02, 78.79)	1.73 (0.08, 87.03)	1.53 (0.05, 87.14)	0.15 (0.00, 2.40)	0.57 (0.05, 5.59)	0.81 (0.09, 6.97)	0.87 (0.11, 5.78)	0.20 (0.01, 1.70)	0.17 (0.01, 2.37)
AND150mgSC2_2	4.36 (0.06, 316.96)	**14.73 (1.28, 503.54)**	**23.17 (1.55, 1586.94)**	10.09 (0.88, 336.99)	2.88 (0.06, 280.36)	12.30 (0.57, 590.49)	15.92 (0.86, 903.25)	2.63 (0.06, 215.58)	7.79 (0.66, 281.31)	5.08 (0.36, 188.26)	11.01 (0.89, 390.89)	9.48 (0.82, 326.55)	10.29 (0.09, 2174.44)	16.16 (0.42, 2884.73)	14.39 (0.36, 2383.80)	1.24 (0.02, 106.70)	4.93 (0.30, 297.97)	7.17 (0.47, 352.86)	7.53 (0.53, 294.26)	1.78 (0.08, 82.85)	1.51 (0.03, 81.80)
AND300mgSC	0.38 (0.01, 6.71)	1.26 (0.20, 6.50)	1.94 (0.20, 24.76)	0.86 (0.15, 3.68)	0.24 (0.01, 4.69)	1.00 (0.08, 12.13)	1.35 (0.12, 14.64)	0.21 (0.01, 4.19)	0.66 (0.10, 3.55)	0.43 (0.06, 2.48)	0.92 (0.15, 4.39)	0.82 (0.14, 3.65)	0.78 (0.01, 51.53)	1.25 (0.06, 61.30)	1.12 (0.04, 63.30)	0.11 (0.00, 1.64)	0.42 (0.04, 3.75)	0.60 (0.07, 4.89)	0.65 (0.08, 4.16)	0.15 (0.01, 1.21)	0.13 (0.00, 1.36)
API100mgPR	1.28 (0.03, 24.04)	4.17 (0.85, 22.70)	6.54 (0.83, 77.16)	2.80 (0.73, 12.21)	0.82 (0.03, 15.70)	3.40 (0.34, 37.76)	4.36 (0.53, 45.85)	0.72 (0.03, 14.63)	2.13 (0.45, 12.55)	1.40 (0.28, 8.14)	3.09 (0.69, 14.10)	2.67 (0.65, 11.98)	2.70 (0.04, 171.34)	4.08 (0.30, 191.67)	3.59 (0.20, 195.00)	0.36 (0.01, 5.06)	1.39 (0.17, 11.62)	2.01 (0.28, 16.26)	2.11 (0.36, 14.78)	0.50 (0.04, 3.82)	0.43 (0.01, 4.87)
API50mgPO	0.84 (0.02, 13.52)	2.70 (0.63, 12.17)	4.20 (0.64, 45.27)	1.82 (0.52, 6.61)	0.51 (0.02, 8.81)	2.19 (0.23, 22.13)	2.84 (0.39, 29.05)	0.47 (0.02, 9.18)	1.40 (0.33, 6.85)	0.91 (0.19, 4.51)	1.96 (0.49, 8.26)	1.73 (0.48, 6.69)	1.72 (0.03, 92.76)	2.67 (0.19, 124.35)	2.38 (0.14, 106.59)	0.24 (0.01, 2.75)	0.91 (0.12, 6.52)	1.29 (0.20, 9.20)	1.39 (0.25, 8.18)	0.33 (0.03, 2.26)	0.27 (0.01, 2.83)
BRI400mgIV	1.22 (0.02, 34.73)	4.10 (0.57, 37.83)	6.57 (0.63, 111.44)	2.69 (0.45, 23.00)	0.76 (0.02, 21.53)	3.45 (0.26, 55.52)	4.34 (0.43, 71.79)	0.72 (0.02, 20.69)	2.11 (0.29, 20.99)	1.36 (0.19, 13.73)	2.90 (0.46, 26.19)	2.57 (0.41, 22.38)	2.59 (0.03, 228.81)	4.13 (0.19, 274.73)	3.77 (0.16, 226.00)	0.34 (0.01, 7.00)	1.33 (0.12, 18.53)	1.96 (0.20, 26.05)	2.10 (0.25, 23.02)	0.48 (0.03, 6.14)	0.41 (0.01, 7.79)
BRI700mgIV	1.87 (0.04, 35.05)	5.97 (0.94, 36.60)	**9.43 (1.06, 123.19)**	4.05 (0.80, 20.51)	1.15 (0.04, 19.83)	4.78 (0.43, 56.51)	6.31 (0.69, 70.88)	1.03 (0.04, 23.44)	3.17 (0.49, 18.54)	2.06 (0.32, 12.79)	4.31 (0.82, 23.65)	3.79 (0.73, 20.50)	3.75 (0.06, 259.94)	5.75 (0.33, 346.57)	5.15 (0.27, 299.10)	0.52 (0.01, 7.42)	2.00 (0.21, 17.69)	2.92 (0.34, 22.67)	3.03 (0.41, 21.34)	0.72 (0.04, 6.63)	0.60 (0.02, 8.39)
BRO210mgIV	0.36 (0.00, 7.66)	1.18 (0.12, 9.18)	1.89 (0.12, 28.33)	0.82 (0.10, 5.36)	0.22 (0.01, 4.66)	0.94 (0.06, 15.57)	1.27 (0.07, 18.90)	0.20 (0.01, 5.48)	0.62 (0.06, 5.50)	0.40 (0.04, 3.61)	0.87 (0.09, 6.17)	0.78 (0.09, 5.23)	0.69 (0.01, 55.07)	1.17 (0.05, 62.57)	1.03 (0.04, 56.82)	0.10 (0.00, 1.70)	0.39 (0.03, 4.67)	0.57 (0.04, 5.86)	0.60 (0.05, 5.84)	0.15 (0.01, 1.53)	0.12 (0.00, 1.89)
BRO350mgIV	0.12 (0.00, 2.52)	0.43 (0.05, 2.55)	0.68 (0.05, 7.35)	0.30 (0.04, 1.47)	0.08 (0.00, 1.51)	0.33 (0.02, 4.28)	0.46 (0.03, 5.26)	0.07 (0.00, 1.69)	0.22 (0.03, 1.35)	**0.15 (0.02, 0.89)**	0.32 (0.04, 1.68)	0.28 (0.04, 1.45)	0.25 (0.00, 18.66)	0.41 (0.02, 22.71)	0.37 (0.01, 22.52)	**0.04 (0.00, 0.51)**	0.14 (0.01, 1.29)	0.21 (0.02, 1.74)	0.21 (0.02, 1.72)	**0.05 (0.00, 0.47)**	**0.04 (0.00, 0.60)**
BRO700mgIV	0.11 (0.00, 2.19)	0.37 (0.04, 2.12)	0.60 (0.05, 6.25)	0.26 (0.03, 1.18)	0.07 (0.00, 1.25)	0.29 (0.02, 3.88)	0.40 (0.03, 4.83)	0.07 (0.00, 1.39)	0.20 (0.02, 1.15)	**0.13 (0.01, 0.75)**	0.28 (0.03, 1.37)	0.25 (0.03, 1.17)	0.23 (0.00, 16.05)	0.36 (0.02, 20.02)	0.34 (0.01, 19.17)	**0.03 (0.00, 0.43)**	0.12 (0.01, 1.14)	0.18 (0.02, 1.48)	0.19 (0.02, 1.45)	**0.04 (0.00, 0.43)**	**0.04 (0.00, 0.52)**
CDP10mg_kgIV	0.83 (0.02, 11.31)	2.56 (0.77, 8.29)	3.95 (0.74, 34.91)	1.74 (0.69, 4.40)	0.50 (0.02, 6.73)	2.05 (0.28, 17.50)	2.68 (0.45, 22.75)	0.47 (0.02, 7.13)	1.34 (0.41, 4.63)	0.88 (0.24, 3.11)	1.89 (0.64, 5.28)	1.65 (0.63, 4.45)	1.64 (0.02, 92.87)	2.47 (0.19, 111.88)	2.21 (0.15, 100.03)	0.24 (0.01, 2.21)	0.85 (0.13, 5.05)	1.24 (0.25, 6.29)	1.30 (0.30, 5.79)	0.31 (0.03, 1.72)	0.27 (0.01, 2.29)
CER100mgSC	0.48 (0.01, 7.63)	1.50 (0.55, 4.41)	2.34 (0.37, 21.03)	1.01 (0.37, 2.96)	0.29 (0.01, 4.05)	1.19 (0.14, 11.97)	1.61 (0.20, 16.27)	0.28 (0.01, 4.64)	0.77 (0.20, 3.50)	0.51 (0.12, 2.41)	1.10 (0.30, 4.14)	0.96 (0.29, 3.51)	0.92 (0.02, 59.08)	1.44 (0.11, 70.57)	1.34 (0.07, 65.71)	0.14 (0.00, 1.50)	0.50 (0.07, 3.62)	0.72 (0.12, 4.56)	0.77 (0.14, 4.06)	0.18 (0.01, 1.25)	0.15 (0.01, 1.60)

	CER10mgIV	CER200mgSC	CER20mgIV	CER400mgSC	CER5mgIV	ELD10mg_kgIV	ELD20mg_kgIV	ETA25mgSC	ETR105mgSC	ETR210mgSC	FIL100mgPO	FIL200mgPO	FON0_1mg_kgIV	FON1mg_kgIV	FON4mg_kgIV	GUS1200mgIV	GUS200mgIV	GUS600mgIV	MED700IV	MIR1000mgIV	MIR200mgIV
CER10mgIV	CER10mgIV	3.14 (0.21, 132.43)	4.86 (0.70, 151.04)	2.09 (0.17, 78.89)	0.63 (0.02, 27.59)	2.68 (0.12, 169.16)	3.66 (0.15, 192.66)	0.62 (0.01, 42.90)	1.68 (0.11, 65.90)	1.09 (0.07, 42.32)	2.27 (0.16, 85.58)	2.03 (0.16, 74.61)	2.11 (0.03, 297.22)	3.53 (0.10, 436.16)	3.08 (0.07, 357.75)	0.30 (0.00, 17.30)	1.10 (0.05, 52.83)	1.59 (0.08, 81.61)	1.62 (0.09, 74.26)	0.38 (0.01, 16.48)	0.33 (0.01, 15.06)
CER200mgSC	0.32 (0.01, 4.69)	CER200mgSC	1.53 (0.26, 13.75)	0.68 (0.26, 1.74)	0.19 (0.01, 3.05)	0.79 (0.10, 7.73)	1.03 (0.15, 10.55)	0.18 (0.01, 3.25)	0.52 (0.14, 2.19)	0.34 (0.08, 1.55)	0.73 (0.21, 2.52)	0.64 (0.21, 2.17)	0.63 (0.01, 36.75)	0.97 (0.08, 44.55)	0.86 (0.05, 42.37)	**0.09 (0.00, 0.93)**	0.34 (0.04, 2.20)	0.49 (0.08, 2.94)	0.52 (0.10, 2.52)	**0.12 (0.01, 0.76)**	**0.10 (0.00, 0.94)**
CER20mgIV	0.21 (0.01, 1.44)	0.65 (0.07, 3.87)	CER20mgIV	0.45 (0.05, 2.31)	**0.13 (0.01, 0.91)**	0.50 (0.03, 6.70)	0.67 (0.05, 8.31)	0.11 (0.00, 2.59)	0.34 (0.03, 2.15)	0.22 (0.02, 1.40)	0.47 (0.05, 2.74)	0.41 (0.05, 2.29)	0.39 (0.01, 25.00)	0.63 (0.03, 31.33)	0.57 (0.02, 29.11)	**0.06 (0.00, 0.78)**	0.21 (0.02, 2.06)	0.32 (0.02, 2.67)	0.33 (0.03, 2.55)	**0.08 (0.00, 0.71)**	**0.06 (0.00, 0.83)**
CER400mgSC	0.48 (0.01, 6.00)	1.48 (0.58, 3.79)	2.25 (0.43, 18.83)	CER400mgSC	0.29 (0.01, 3.76)	1.18 (0.17, 9.63)	1.53 (0.27, 13.36)	0.26 (0.01, 3.91)	0.77 (0.26, 2.39)	0.49 (0.15, 1.70)	1.08 (0.42, 2.90)	0.95 (0.40, 2.37)	0.91 (0.01, 49.45)	1.43 (0.12, 58.27)	1.23 (0.09, 57.84)	0.13 (0.00, 1.22)	0.49 (0.08, 2.84)	0.71 (0.15, 3.63)	0.75 (0.17, 3.21)	**0.18 (0.01, 0.94)**	0.15 (0.01, 1.23)
CER5mgIV	1.60 (0.04, 43.63)	5.18 (0.33, 124.68)	**7.74 (1.10, 151.18)**	3.48 (0.27, 79.82)	CER5mgIV	4.18 (0.18, 159.85)	5.52 (0.29, 226.72)	0.95 (0.02, 59.56)	2.68 (0.18, 65.03)	1.75 (0.12, 43.10)	3.82 (0.26, 87.19)	3.31 (0.23, 77.87)	3.17 (0.04, 524.43)	5.44 (0.18, 607.74)	5.03 (0.15, 545.01)	0.49 (0.01, 18.60)	1.72 (0.09, 50.26)	2.40 (0.13, 70.39)	2.60 (0.15, 69.40)	0.62 (0.02, 23.06)	0.50 (0.01, 25.80)
ELD10mg_kgIV	0.37 (0.01, 8.50)	1.27 (0.13, 10.00)	2.00 (0.15, 28.66)	0.85 (0.10, 5.81)	0.24 (0.01, 5.42)	ELD10mg_kgIV	1.32 (0.28, 6.87)	0.22 (0.01, 5.36)	0.65 (0.07, 5.31)	0.42 (0.04, 3.92)	0.92 (0.10, 6.74)	0.81 (0.09, 5.84)	0.78 (0.01, 51.94)	1.23 (0.06, 74.62)	1.03 (0.04, 69.07)	0.11 (0.00, 2.17)	0.41 (0.03, 5.07)	0.59 (0.05, 6.99)	0.62 (0.06, 6.53)	0.15 (0.01, 1.69)	0.13 (0.00, 2.16)
ELD20mg_kgIV	0.27 (0.01, 6.67)	0.97 (0.09, 6.51)	1.50 (0.12, 19.05)	0.65 (0.07, 3.74)	0.18 (0.00, 3.44)	0.76 (0.15, 3.61)	ELD20mg_kgIV	0.16 (0.00, 4.04)	0.50 (0.05, 3.49)	0.32 (0.03, 2.32)	0.70 (0.08, 4.33)	0.62 (0.08, 3.76)	0.58 (0.01, 46.02)	0.95 (0.05, 45.73)	0.82 (0.03, 52.53)	0.08 (0.00, 1.27)	0.32 (0.02, 3.40)	0.45 (0.04, 4.53)	0.47 (0.04, 4.09)	0.11 (0.01, 1.13)	0.09 (0.00, 1.51)
ETA25mgSC	1.62 (0.02, 84.84)	5.56 (0.31, 134.69)	9.31 (0.39, 333.27)	3.81 (0.26, 90.51)	1.05 (0.02, 65.45)	4.53 (0.19, 168.14)	6.15 (0.25, 211.11)	ETA25mgSC	2.96 (0.18, 74.33)	1.94 (0.11, 47.21)	4.01 (0.25, 93.48)	3.57 (0.24, 87.10)	3.66 (0.02, 484.47)	5.71 (0.14, 525.57)	5.09 (0.09, 482.93)	0.51 (0.01, 19.28)	1.86 (0.08, 56.39)	2.70 (0.13, 79.30)	2.78 (0.15, 81.96)	0.64 (0.02, 21.33)	0.53 (0.01, 21.67)
ETR105mgSC	0.60 (0.02, 9.16)	1.94 (0.46, 7.08)	2.94 (0.47, 28.78)	1.29 (0.42, 3.90)	0.37 (0.02, 5.59)	1.53 (0.19, 14.98)	2.00 (0.29, 19.43)	0.34 (0.01, 5.67)	ETR105mgSC	0.65 (0.23, 1.70)	1.40 (0.38, 4.77)	1.24 (0.37, 3.96)	1.19 (0.02, 68.20)	1.89 (0.13, 91.78)	1.64 (0.10, 80.41)	0.17 (0.00, 1.85)	0.64 (0.09, 4.06)	0.94 (0.15, 5.06)	0.96 (0.18, 4.96)	0.23 (0.02, 1.36)	0.20 (0.01, 1.91)
ETR210mgSC	0.91 (0.02, 14.36)	2.98 (0.65, 11.95)	4.53 (0.72, 44.42)	2.02 (0.59, 6.57)	0.57 (0.02, 8.65)	2.39 (0.26, 24.95)	3.10 (0.43, 31.61)	0.51 (0.02, 9.31)	1.53 (0.59, 4.35)	ETR210mgSC	2.16 (0.57, 7.78)	1.91 (0.54, 6.64)	1.84 (0.03, 108.83)	2.85 (0.21, 134.98)	2.57 (0.15, 123.90)	0.26 (0.01, 2.96)	0.99 (0.13, 6.79)	1.40 (0.22, 8.94)	1.49 (0.27, 8.13)	0.36 (0.03, 2.33)	0.30 (0.01, 3.07)
FIL100mgPO	0.44 (0.01, 6.10)	1.37 (0.40, 4.86)	2.12 (0.36, 18.73)	0.92 (0.34, 2.40)	0.26 (0.01, 3.82)	1.08 (0.15, 10.11)	1.42 (0.23, 12.35)	0.25 (0.01, 3.94)	0.71 (0.21, 2.62)	0.46 (0.13, 1.75)	FIL100mgPO	0.88 (0.42, 1.86)	0.86 (0.01, 47.08)	1.37 (0.11, 57.85)	1.19 (0.08, 53.69)	0.12 (0.00, 1.13)	0.46 (0.07, 2.82)	0.66 (0.13, 3.51)	0.69 (0.15, 3.12)	**0.17 (0.01, 0.95)**	0.14 (0.01, 1.25)
FIL200mgPO	0.49 (0.01, 6.44)	1.57 (0.46, 4.75)	2.43 (0.44, 21.44)	1.05 (0.42, 2.50)	0.30 (0.01, 4.33)	1.23 (0.17, 11.07)	1.62 (0.27, 12.97)	0.28 (0.01, 4.16)	0.81 (0.25, 2.72)	0.52 (0.15, 1.84)	1.14 (0.54, 2.38)	FIL200mgPO	1.00 (0.01, 50.79)	1.56 (0.12, 61.12)	1.32 (0.09, 63.04)	0.14 (0.00, 1.20)	0.52 (0.08, 2.89)	0.75 (0.15, 3.80)	0.79 (0.17, 3.43)	**0.19 (0.02, 0.99)**	0.16 (0.01, 1.29)
FON0_1mg_kgIV	0.47 (0.00, 37.77)	1.59 (0.03, 100.75)	2.59 (0.04, 162.69)	1.10 (0.02, 70.34)	0.32 (0.00, 24.73)	1.29 (0.02, 100.13)	1.71 (0.02, 129.66)	0.27 (0.00, 40.55)	0.84 (0.01, 55.75)	0.54 (0.01, 35.03)	1.16 (0.02, 77.58)	1.00 (0.02, 71.17)	FON0_1mg_kgIV	1.46 (0.15, 59.57)	1.39 (0.12, 58.18)	0.13 (0.00, 12.07)	0.54 (0.01, 40.39)	0.80 (0.01, 58.46)	0.81 (0.01, 57.25)	0.19 (0.00, 13.67)	0.16 (0.00, 13.84)
FON1mg_kgIV	0.28 (0.00, 9.89)	1.03 (0.02, 13.30)	1.58 (0.03, 31.86)	0.70 (0.02, 8.45)	0.18 (0.00, 5.70)	0.82 (0.01, 17.39)	1.05 (0.02, 21.72)	0.18 (0.00, 7.41)	0.53 (0.01, 7.42)	0.35 (0.01, 4.82)	0.73 (0.02, 9.24)	0.64 (0.02, 8.40)	0.68 (0.02, 6.57)	FON1mg_kgIV	0.90 (0.10, 6.71)	0.08 (0.00, 2.66)	0.32 (0.01, 6.99)	0.49 (0.01, 8.15)	0.52 (0.01, 8.57)	0.12 (0.00, 2.41)	0.10 (0.00, 2.98)
FON4mg_kgIV	0.32 (0.00, 14.03)	1.17 (0.02, 19.62)	1.76 (0.03, 55.09)	0.82 (0.02, 10.94)	0.20 (0.00, 6.64)	0.97 (0.01, 22.70)	1.21 (0.02, 32.62)	0.20 (0.00, 10.66)	0.61 (0.01, 10.00)	0.39 (0.01, 6.73)	0.84 (0.02, 12.79)	0.76 (0.02, 10.66)	0.72 (0.02, 8.62)	1.11 (0.15, 9.90)	FON4mg_kgIV	0.10 (0.00, 2.95)	0.38 (0.01, 10.23)	0.55 (0.01, 13.31)	0.58 (0.01, 11.37)	0.14 (0.00, 3.02)	0.11 (0.00, 3.22)
GUS1200mgIV	3.32 (0.06, 317.34)	**11.04 (1.08, 513.11)**	**17.40 (1.29, 871.28)**	7.55 (0.82, 248.76)	2.05 (0.05, 169.01)	9.15 (0.46, 498.05)	12.59 (0.79, 655.02)	1.97 (0.05, 148.09)	5.82 (0.54, 212.82)	3.80 (0.34, 135.45)	8.13 (0.89, 262.90)	7.28 (0.83, 235.43)	7.77 (0.08, 1100.46)	12.60 (0.38, 1458.10)	10.33 (0.34, 1416.96)	GUS1200mgIV	3.59 (0.40, 120.29)	5.37 (0.68, 164.24)	5.71 (0.45, 221.05)	1.32 (0.05, 56.46)	1.14 (0.03, 60.28)
GUS200mgIV	0.91 (0.02, 19.06)	2.96 (0.46, 22.33)	4.71 (0.49, 64.77)	2.04 (0.35, 12.32)	0.58 (0.02, 11.73)	2.45 (0.20, 36.00)	3.17 (0.29, 45.25)	0.54 (0.02, 13.19)	1.56 (0.25, 10.85)	1.01 (0.15, 7.49)	2.18 (0.36, 14.15)	1.93 (0.35, 12.13)	1.85 (0.02, 131.29)	3.12 (0.14, 177.43)	2.63 (0.10, 172.50)	0.28 (0.01, 2.52)	GUS200mgIV	1.42 (0.29, 7.88)	1.52 (0.18, 14.39)	0.37 (0.02, 3.30)	0.31 (0.01, 4.08)
GUS600mgIV	0.63 (0.01, 12.57)	2.05 (0.34, 13.23)	3.15 (0.37, 41.24)	1.40 (0.28, 6.73)	0.42 (0.01, 7.90)	1.70 (0.14, 19.35)	2.24 (0.22, 25.66)	0.37 (0.01, 7.83)	1.07 (0.20, 6.66)	0.71 (0.11, 4.46)	1.53 (0.28, 7.77)	1.34 (0.26, 6.57)	1.25 (0.02, 91.51)	2.04 (0.12, 116.50)	1.81 (0.08, 109.71)	0.19 (0.01, 1.47)	0.71 (0.13, 3.44)	GUS600mgIV	1.04 (0.14, 7.69)	0.26 (0.02, 2.11)	0.21 (0.01, 2.57)
MED700IV	0.62 (0.01, 10.74)	1.94 (0.40, 10.10)	3.07 (0.39, 34.02)	1.34 (0.31, 5.85)	0.38 (0.01, 6.87)	1.60 (0.15, 18.09)	2.12 (0.24, 24.26)	0.36 (0.01, 6.84)	1.04 (0.20, 5.42)	0.67 (0.12, 3.72)	1.44 (0.32, 6.63)	1.27 (0.29, 5.78)	1.23 (0.02, 82.41)	1.93 (0.12, 103.31)	1.73 (0.09, 89.67)	0.18 (0.00, 2.21)	0.66 (0.07, 5.52)	0.96 (0.13, 7.35)	MED700IV	0.23 (0.02, 2.09)	0.19 (0.01, 2.59)
MIR1000mgIV	2.62 (0.06, 71.57)	**8.23 (1.31, 99.18)**	**13.02 (1.41, 260.60)**	**5.54 (1.06, 67.65)**	1.62 (0.04, 53.01)	6.56 (0.59, 155.86)	9.10 (0.88, 187.58)	1.56 (0.05, 47.44)	4.30 (0.74, 61.24)	2.81 (0.43, 37.96)	**5.95 (1.06, 68.82)**	**5.34 (1.01, 62.26)**	5.22 (0.07, 470.69)	8.56 (0.42, 499.04)	7.39 (0.33, 465.86)	0.76 (0.02, 18.97)	2.68 (0.30, 48.52)	3.85 (0.47, 64.84)	4.32 (0.48, 52.62)	MIR1000mgIV	0.84 (0.03, 15.41)
MIR200mgIV	3.00 (0.07, 170.51)	**9.76 (1.06, 278.66)**	**16.30 (1.21, 696.30)**	6.50 (0.81, 168.84)	2.02 (0.04, 106.15)	7.94 (0.46, 374.27)	10.96 (0.66, 401.93)	1.89 (0.05, 97.35)	5.12 (0.52, 144.02)	3.30 (0.33, 100.11)	7.10 (0.80, 192.71)	6.10 (0.77, 166.48)	6.38 (0.07, 1026.97)	10.36 (0.34, 1464.61)	9.20 (0.31, 1375.91)	0.88 (0.02, 34.55)	3.28 (0.25, 112.40)	4.84 (0.39, 141.12)	5.18 (0.39, 148.87)	1.19 (0.06, 36.86)	MIR200mgIV

	CER10mgIV	CER200mgSC	CER20mgIV	CER400mgSC	CER5mgIV	ELD10mg_kgIV	ELD20mg_kgIV	ETA25mgSC	ETR105mgSC	ETR210mgSC	FIL100mgPO	FIL200mgPO	FON0_1mg_kgIV	FON1mg_kgIV	FON4mg_kgIV	GUS1200mgIV	GUS200mgIV	GUS600mgIV	MED700IV	MIR1000mgIV	MIR200mgIV
MIR600mgIV	0.78 (0.02, 15.85)	2.45 (0.49, 17.83)	3.93 (0.51, 54.93)	1.68 (0.41, 10.20)	0.49 (0.01, 11.50)	2.12 (0.19, 30.72)	2.76 (0.30, 32.48)	0.46 (0.01, 11.28)	1.33 (0.25, 9.39)	0.84 (0.16, 6.66)	1.78 (0.41, 12.81)	1.62 (0.38, 10.77)	1.59 (0.02, 118.76)	2.56 (0.15, 143.09)	2.21 (0.11, 129.42)	0.23 (0.01, 3.00)	0.83 (0.11, 8.73)	1.19 (0.16, 11.37)	1.28 (0.18, 11.77)	0.30 (0.03, 2.38)	0.27 (0.01, 2.22)
NAT300mgIV	0.80 (0.02, 10.40)	2.52 (0.85, 7.51)	3.87 (0.79, 33.10)	1.72 (0.76, 3.74)	0.50 (0.02, 6.68)	2.05 (0.29, 16.86)	2.67 (0.44, 22.15)	0.46 (0.02, 6.87)	1.32 (0.44, 4.07)	0.85 (0.26, 2.82)	1.85 (0.73, 4.64)	1.62 (0.71, 3.84)	1.58 (0.02, 86.11)	2.48 (0.20, 108.46)	2.18 (0.14, 98.53)	0.22 (0.01, 1.97)	0.85 (0.14, 4.78)	1.21 (0.26, 5.84)	1.27 (0.29, 5.42)	0.30 (0.03, 1.53)	0.26 (0.01, 2.04)
NAT3mg_kgIV	0.68 (0.02, 10.27)	2.17 (0.49, 9.16)	3.38 (0.51, 33.09)	1.49 (0.42, 5.13)	0.42 (0.02, 6.50)	1.78 (0.19, 17.08)	2.34 (0.31, 20.73)	0.39 (0.01, 6.86)	1.15 (0.27, 5.04)	0.73 (0.16, 3.34)	1.59 (0.40, 6.12)	1.40 (0.40, 5.02)	1.35 (0.02, 85.92)	2.13 (0.16, 103.12)	1.90 (0.10, 98.02)	0.19 (0.01, 1.95)	0.73 (0.09, 4.83)	1.04 (0.16, 6.69)	1.11 (0.19, 6.15)	0.27 (0.02, 1.79)	0.22 (0.01, 2.25)
NAT3mg_kgIVx2	0.76 (0.02, 13.34)	2.45 (0.52, 10.98)	3.81 (0.57, 42.02)	1.68 (0.45, 5.96)	0.49 (0.02, 7.68)	2.04 (0.22, 19.27)	2.60 (0.34, 26.25)	0.44 (0.02, 8.20)	1.30 (0.28, 5.92)	0.85 (0.17, 3.98)	1.81 (0.45, 6.93)	1.59 (0.43, 6.04)	1.60 (0.02, 88.97)	2.37 (0.19, 111.97)	2.12 (0.12, 101.58)	0.21 (0.01, 2.52)	0.81 (0.10, 5.75)	1.19 (0.19, 7.77)	1.25 (0.21, 7.30)	0.30 (0.02, 2.16)	0.24 (0.01, 2.84)
NAT6mg_kgIVx2	0.57 (0.01, 9.79)	1.89 (0.41, 8.84)	2.95 (0.44, 32.24)	1.30 (0.35, 5.07)	0.37 (0.01, 5.80)	1.55 (0.17, 16.20)	2.03 (0.25, 20.37)	0.34 (0.01, 6.36)	0.99 (0.23, 4.64)	0.65 (0.14, 3.24)	1.41 (0.34, 5.98)	1.22 (0.33, 4.93)	1.22 (0.02, 82.69)	1.87 (0.13, 93.15)	1.68 (0.09, 93.32)	0.17 (0.00, 1.86)	0.63 (0.08, 4.95)	0.91 (0.15, 5.93)	0.98 (0.16, 5.57)	0.23 (0.02, 1.73)	0.20 (0.01, 2.21)
NNC2mg_kgSC	0.21 (0.00, 4.87)	0.73 (0.08, 5.27)	1.15 (0.09, 16.30)	0.50 (0.06, 2.82)	0.14 (0.00, 2.67)	0.57 (0.04, 8.02)	0.77 (0.06, 10.21)	0.12 (0.00, 3.21)	0.38 (0.04, 2.61)	0.25 (0.03, 1.85)	0.55 (0.06, 3.31)	0.48 (0.06, 2.88)	0.44 (0.01, 35.85)	0.72 (0.03, 46.22)	0.63 (0.03, 40.71)	**0.06 (0.00, 0.96)**	0.24 (0.02, 2.38)	0.35 (0.03, 3.21)	0.37 (0.03, 3.24)	**0.09 (0.00, 0.81)**	**0.07 (0.00, 0.93)**
ONE10mgSC	0.75 (0.00, 37.54)	2.61 (0.07, 71.60)	4.07 (0.10, 163.61)	1.76 (0.05, 45.39)	0.48 (0.00, 26.74)	2.04 (0.03, 89.17)	2.69 (0.06, 117.25)	0.45 (0.00, 24.26)	1.39 (0.03, 37.32)	0.90 (0.02, 26.72)	1.93 (0.05, 45.80)	1.67 (0.05, 41.06)	1.53 (0.01, 244.17)	2.52 (0.04, 334.44)	2.31 (0.03, 330.30)	0.21 (0.00, 9.32)	0.84 (0.02, 26.82)	1.26 (0.02, 35.61)	1.30 (0.03, 39.01)	0.31 (0.01, 9.91)	0.24 (0.00, 10.61)
ONE25mgSC	0.64 (0.00, 45.77)	2.13 (0.06, 80.83)	3.36 (0.07, 186.53)	1.43 (0.04, 52.12)	0.38 (0.01, 32.16)	1.80 (0.03, 99.90)	2.37 (0.05, 125.06)	0.36 (0.00, 33.04)	1.16 (0.03, 45.40)	0.73 (0.02, 29.96)	1.56 (0.04, 60.73)	1.35 (0.04, 52.91)	1.29 (0.01, 276.65)	2.27 (0.03, 412.36)	2.04 (0.02, 380.91)	0.18 (0.00, 13.71)	0.74 (0.01, 37.45)	1.02 (0.02, 47.94)	1.08 (0.03, 45.85)	0.27 (0.00, 11.15)	0.22 (0.00, 12.38)
ONE35mgSC	0.16 (0.00, 5.00)	0.53 (0.02, 6.78)	0.84 (0.03, 16.29)	0.37 (0.02, 4.38)	0.10 (0.00, 3.82)	0.44 (0.01, 10.72)	0.57 (0.02, 13.16)	0.09 (0.00, 3.73)	0.29 (0.01, 4.02)	0.19 (0.01, 2.74)	0.40 (0.01, 4.83)	0.35 (0.01, 4.32)	0.32 (0.00, 32.14)	0.52 (0.01, 34.21)	0.48 (0.01, 30.82)	0.04 (0.00, 1.27)	0.18 (0.00, 3.35)	0.25 (0.01, 4.59)	0.27 (0.01, 3.98)	0.06 (0.00, 1.10)	0.05 (0.00, 1.21)
ONE50mgSC	0.67 (0.00, 86.33)	2.44 (0.08, 170.95)	3.89 (0.10, 380.61)	1.67 (0.05, 98.40)	0.45 (0.00, 61.96)	2.07 (0.04, 171.36)	2.74 (0.05, 229.87)	0.44 (0.00, 65.80)	1.30 (0.04, 87.91)	0.86 (0.03, 50.01)	1.81 (0.05, 116.03)	1.61 (0.04, 98.56)	1.57 (0.01, 510.59)	2.54 (0.04, 546.02)	2.35 (0.03, 618.95)	0.21 (0.00, 18.40)	0.80 (0.02, 68.62)	1.21 (0.03, 108.10)	1.24 (0.03, 100.28)	0.28 (0.01, 20.56)	0.24 (0.00, 22.34)
ONT22_5mgSC	0.27 (0.01, 4.33)	0.90 (0.19, 3.85)	1.41 (0.20, 12.91)	0.62 (0.15, 2.06)	0.17 (0.01, 2.93)	0.73 (0.08, 7.13)	0.92 (0.12, 9.57)	0.16 (0.01, 2.90)	0.48 (0.10, 2.05)	0.31 (0.06, 1.37)	0.66 (0.16, 2.50)	0.58 (0.15, 2.02)	0.55 (0.01, 32.51)	0.87 (0.06, 41.36)	0.77 (0.04, 40.60)	**0.08 (0.00, 0.89)**	0.30 (0.03, 2.12)	0.42 (0.07, 2.84)	0.45 (0.07, 2.54)	**0.11 (0.01, 0.72)**	**0.09 (0.00, 0.95)**
ONT225mgSC	0.28 (0.01, 4.33)	0.92 (0.20, 3.83)	1.44 (0.21, 13.18)	0.63 (0.17, 2.09)	0.17 (0.01, 2.84)	0.73 (0.08, 7.63)	0.96 (0.13, 9.85)	0.17 (0.01, 2.90)	0.49 (0.11, 2.07)	0.32 (0.07, 1.38)	0.69 (0.17, 2.46)	0.61 (0.16, 2.09)	0.56 (0.01, 33.50)	0.89 (0.06, 41.43)	0.82 (0.04, 40.56)	**0.08 (0.00, 0.90)**	0.31 (0.04, 2.15)	0.44 (0.07, 2.85)	0.46 (0.08, 2.64)	**0.11 (0.01, 0.76)**	0.09 (0.00, 1.03)
ONT75mgSC	0.34 (0.01, 5.14)	1.09 (0.23, 4.69)	1.70 (0.24, 16.34)	0.74 (0.19, 2.52)	0.21 (0.01, 3.35)	0.87 (0.10, 9.06)	1.10 (0.15, 12.06)	0.20 (0.01, 3.37)	0.57 (0.12, 2.53)	0.38 (0.07, 1.67)	0.80 (0.18, 3.06)	0.70 (0.18, 2.50)	0.66 (0.01, 39.52)	1.04 (0.08, 45.24)	0.95 (0.05, 41.99)	0.10 (0.00, 1.11)	0.38 (0.04, 2.60)	0.53 (0.08, 3.53)	0.56 (0.09, 3.18)	**0.13 (0.01, 0.92)**	0.11 (0.00, 1.17)
PBO	0.64 (0.02, 7.79)	1.99 (0.74, 5.18)	2.99 (0.66, 24.18)	1.34 (0.75, 2.40)	0.39 (0.02, 4.95)	1.59 (0.24, 12.51)	2.09 (0.39, 15.65)	0.36 (0.02, 4.94)	1.04 (0.39, 2.83)	0.67 (0.23, 1.96)	1.44 (0.68, 3.11)	1.28 (0.67, 2.56)	1.24 (0.02, 62.31)	1.95 (0.16, 82.21)	1.69 (0.12, 74.10)	0.18 (0.01, 1.48)	0.66 (0.12, 3.32)	0.96 (0.22, 4.26)	1.01 (0.27, 3.79)	0.24 (0.02, 1.12)	0.21 (0.01, 1.52)
PF10mgSC	0.79 (0.02, 11.77)	2.57 (0.60, 10.39)	3.95 (0.61, 37.87)	1.73 (0.51, 5.66)	0.50 (0.02, 8.22)	2.05 (0.25, 20.30)	2.66 (0.37, 26.57)	0.46 (0.02, 7.80)	1.33 (0.32, 5.63)	0.86 (0.20, 3.94)	1.85 (0.52, 6.73)	1.62 (0.50, 5.82)	1.56 (0.03, 88.30)	2.53 (0.18, 108.28)	2.28 (0.13, 111.54)	0.22 (0.01, 2.58)	0.85 (0.11, 6.06)	1.21 (0.19, 8.06)	1.29 (0.24, 7.11)	0.31 (0.03, 1.94)	0.27 (0.01, 2.47)
PF200mgSC	0.30 (0.01, 4.20)	0.97 (0.23, 3.65)	1.50 (0.23, 13.89)	0.65 (0.19, 1.88)	0.18 (0.01, 2.88)	0.76 (0.09, 7.38)	1.01 (0.15, 8.98)	0.17 (0.01, 2.85)	0.50 (0.13, 1.90)	0.32 (0.08, 1.36)	0.69 (0.20, 2.29)	0.61 (0.19, 1.93)	0.58 (0.01, 33.51)	0.93 (0.07, 39.94)	0.83 (0.05, 41.04)	**0.09 (0.00, 0.87)**	0.32 (0.04, 2.07)	0.46 (0.08, 2.66)	0.48 (0.09, 2.45)	**0.12 (0.01, 0.68)**	**0.10 (0.00, 0.95)**
PF50mgSC	0.63 (0.01, 9.47)	2.02 (0.50, 8.35)	3.14 (0.51, 29.00)	1.37 (0.40, 4.31)	0.40 (0.02, 6.39)	1.62 (0.20, 15.77)	2.10 (0.32, 21.09)	0.36 (0.01, 6.49)	1.07 (0.27, 4.26)	0.69 (0.17, 3.07)	1.47 (0.43, 5.26)	1.29 (0.40, 4.52)	1.24 (0.02, 76.81)	2.04 (0.15, 85.54)	1.77 (0.10, 85.27)	0.18 (0.00, 1.92)	0.67 (0.09, 4.68)	0.97 (0.17, 5.89)	1.03 (0.20, 5.54)	0.25 (0.02, 1.56)	0.21 (0.01, 1.93)
RIS1200mgIV	2.28 (0.06, 30.56)	**7.33 (2.34, 22.52)**	**11.14 (2.24, 93.55)**	**4.93 (2.18, 11.21)**	1.43 (0.06, 19.65)	5.85 (0.82, 49.98)	**7.62 (1.31, 64.21)**	1.33 (0.06, 19.66)	**3.82 (1.26, 12.31)**	2.48 (0.74, 8.45)	**5.28 (2.04, 13.90)**	**4.71 (2.02, 11.53)**	4.48 (0.07, 236.55)	7.19 (0.60, 291.27)	6.27 (0.43, 291.26)	0.65 (0.02, 5.79)	2.45 (0.39, 14.17)	3.50 (0.72, 17.05)	3.69 (0.85, 15.68)	0.88 (0.08, 4.53)	0.76 (0.03, 6.32)
RIS200mgIV	0.94 (0.02, 14.96)	3.04 (0.76, 13.17)	4.61 (0.74, 49.04)	2.04 (0.66, 6.81)	0.59 (0.02, 9.44)	2.45 (0.30, 24.30)	3.19 (0.46, 31.73)	0.54 (0.02, 9.37)	1.57 (0.39, 6.87)	1.04 (0.24, 4.53)	2.20 (0.62, 8.14)	1.94 (0.61, 6.85)	1.93 (0.03, 106.51)	2.94 (0.22, 132.71)	2.65 (0.16, 130.45)	0.27 (0.01, 2.73)	1.01 (0.14, 6.84)	1.44 (0.25, 9.31)	1.54 (0.29, 8.44)	0.37 (0.03, 2.61)	0.30 (0.01, 3.20)
RIS600mgIV	1.50 (0.04, 19.30)	**4.66 (1.56, 14.36)**	**7.10 (1.46, 65.12)**	**3.15 (1.51, 6.86)**	0.90 (0.04, 12.56)	3.75 (0.53, 31.67)	4.90 (0.87, 40.60)	0.84 (0.04, 12.03)	2.42 (0.85, 7.67)	1.59 (0.49, 5.24)	**3.40 (1.39, 8.66)**	**2.99 (1.37, 7.14)**	2.86 (0.05, 157.16)	4.49 (0.39, 191.92)	4.11 (0.28, 187.57)	0.41 (0.01, 3.67)	1.57 (0.27, 8.60)	2.23 (0.49, 11.08)	2.38 (0.58, 9.69)	0.57 (0.05, 2.89)	0.48 (0.02, 3.94)
SEC10mg_kgIV	0.46 (0.01, 7.97)	1.55 (0.25, 7.53)	2.44 (0.26, 26.09)	1.06 (0.19, 4.27)	0.30 (0.01, 4.43)	1.21 (0.10, 14.21)	1.61 (0.16, 17.98)	0.26 (0.01, 5.65)	0.81 (0.12, 4.05)	0.53 (0.08, 2.80)	1.15 (0.20, 5.07)	1.00 (0.19, 4.34)	0.98 (0.01, 58.55)	1.50 (0.08, 75.29)	1.32 (0.07, 63.41)	0.13 (0.00, 1.71)	0.51 (0.05, 4.18)	0.73 (0.08, 5.50)	0.79 (0.10, 5.00)	0.18 (0.01, 1.34)	0.15 (0.00, 1.84)

	CER10mgIV	CER200mgSC	CER20mgIV	CER400mgSC	CER5mgIV	ELD10mg_kgIV	ELD20mg_kgIV	ETA25mgSC	ETR105mgSC	ETR210mgSC	FIL100mgPO	FIL200mgPO	FON0_1mg_kgIV	FON1mg_kgIV	FON4mg_kgIV	GUS1200mgIV	GUS200mgIV	GUS600mgIV	MED700IV	MIR1000mgIV	MIR200mgIV
TES400mg200mgSC	0.05 (0.00, 1.46)	0.18 (0.01, 1.45)	0.27 (0.01, 4.15)	**0.12 (0.01, 0.88)**	**0.03 (0.00, 0.84)**	0.14 (0.00, 2.47)	0.18 (0.01, 3.36)	**0.03 (0.00, 0.89)**	**0.09 (0.00, 0.82)**	**0.06 (0.00, 0.56)**	0.13 (0.01, 1.04)	**0.11 (0.00, 0.89)**	0.10 (0.00, 8.08)	0.17 (0.00, 8.92)	0.14 (0.00, 9.12)	**0.01 (0.00, 0.29)**	**0.06 (0.00, 0.72)**	**0.08 (0.00, 0.98)**	**0.09 (0.00, 0.97)**	**0.02 (0.00, 0.27)**	**0.02 (0.00, 0.30)**
TOF15mgPO_BID	4.12 (0.08, 216.52)	**13.40 (1.44, 298.28)**	**21.28 (1.56, 845.19)**	**8.91 (1.18, 199.12)**	2.74 (0.06, 123.81)	11.15 (0.65, 489.13)	**14.21 (1.01, 590.98)**	2.52 (0.07, 128.47)	7.03 (0.79, 168.88)	4.66 (0.48, 104.78)	**9.76 (1.14, 235.40)**	**8.51 (1.10, 198.93)**	9.18 (0.12, 1031.62)	14.15 (0.58, 1468.01)	12.54 (0.39, 1175.52)	1.27 (0.02, 43.60)	4.65 (0.29, 124.07)	6.69 (0.54, 166.85)	6.81 (0.61, 187.04)	1.67 (0.07, 43.36)	1.38 (0.03, 60.19)
TOF1mgPO_BID	0.87 (0.02, 15.32)	2.76 (0.48, 15.26)	4.37 (0.50, 51.91)	1.86 (0.42, 8.26)	0.54 (0.02, 8.77)	2.17 (0.22, 24.82)	2.94 (0.35, 32.57)	0.48 (0.02, 9.98)	1.45 (0.26, 7.65)	0.94 (0.16, 5.10)	2.00 (0.39, 9.65)	1.77 (0.38, 8.49)	1.64 (0.03, 111.99)	2.63 (0.17, 140.95)	2.28 (0.12, 133.05)	0.23 (0.01, 3.17)	0.94 (0.09, 8.11)	1.29 (0.18, 10.75)	1.41 (0.20, 9.66)	0.33 (0.02, 2.43)	0.28 (0.01, 3.49)
TOF5mgPO_BID	0.78 (0.02, 12.88)	2.59 (0.48, 13.80)	4.18 (0.50, 39.86)	1.74 (0.39, 7.57)	0.51 (0.02, 8.44)	2.09 (0.22, 22.53)	2.76 (0.32, 29.96)	0.46 (0.02, 9.92)	1.37 (0.26, 7.63)	0.88 (0.15, 5.17)	1.88 (0.37, 9.45)	1.65 (0.36, 7.65)	1.59 (0.02, 109.49)	2.44 (0.14, 151.80)	2.23 (0.11, 129.15)	0.23 (0.01, 2.96)	0.86 (0.09, 7.55)	1.24 (0.16, 9.06)	1.35 (0.20, 9.15)	0.31 (0.02, 2.93)	0.26 (0.01, 3.05)
UPA12mgPO_BID	0.10 (0.00, 2.16)	0.34 (0.04, 1.81)	0.53 (0.04, 6.38)	0.23 (0.03, 1.03)	0.06 (0.00, 1.17)	0.26 (0.02, 3.74)	0.35 (0.03, 4.38)	0.06 (0.00, 1.24)	**0.18 (0.02, 0.97)**	**0.12 (0.01, 0.67)**	0.25 (0.03, 1.22)	0.22 (0.03, 1.02)	0.21 (0.00, 15.36)	0.33 (0.01, 16.43)	0.28 (0.01, 15.22)	**0.03 (0.00, 0.39)**	**0.11 (0.01, 0.94)**	0.16 (0.01, 1.23)	0.18 (0.02, 1.19)	**0.04 (0.00, 0.34)**	**0.03 (0.00, 0.41)**
UPA24mgPO	0.15 (0.00, 3.10)	0.50 (0.06, 2.77)	0.76 (0.06, 10.07)	0.34 (0.04, 1.68)	0.09 (0.00, 1.70)	0.38 (0.03, 5.61)	0.52 (0.04, 6.69)	0.08 (0.00, 1.81)	0.26 (0.03, 1.57)	0.17 (0.02, 1.03)	0.36 (0.05, 1.94)	0.32 (0.04, 1.70)	0.29 (0.00, 23.69)	0.48 (0.02, 23.73)	0.42 (0.01, 21.43)	**0.04 (0.00, 0.56)**	0.17 (0.01, 1.48)	0.24 (0.02, 1.84)	0.25 (0.02, 1.79)	**0.06 (0.00, 0.53)**	**0.05 (0.00, 0.64)**
UPA24mgPO_BID	0.37 (0.01, 10.75)	1.24 (0.14, 10.86)	2.01 (0.15, 30.68)	0.84 (0.10, 6.21)	0.24 (0.01, 4.91)	0.97 (0.07, 18.11)	1.33 (0.10, 20.72)	0.22 (0.01, 6.24)	0.64 (0.07, 5.54)	0.43 (0.04, 3.87)	0.90 (0.11, 7.20)	0.81 (0.09, 6.13)	0.77 (0.01, 62.44)	1.26 (0.05, 69.14)	1.12 (0.04, 62.64)	0.10 (0.00, 1.83)	0.42 (0.03, 4.82)	0.61 (0.05, 6.29)	0.64 (0.06, 6.25)	0.15 (0.01, 1.92)	0.12 (0.00, 2.38)
UPA3mgPO_BID	0.24 (0.00, 5.20)	0.78 (0.09, 5.33)	1.23 (0.10, 19.20)	0.54 (0.07, 3.07)	0.15 (0.00, 2.97)	0.62 (0.04, 10.38)	0.82 (0.06, 12.31)	0.14 (0.00, 3.53)	0.43 (0.05, 2.87)	0.27 (0.03, 1.80)	0.57 (0.07, 3.64)	0.51 (0.06, 2.93)	0.48 (0.01, 40.33)	0.81 (0.03, 44.54)	0.67 (0.02, 39.36)	0.06 (0.00, 1.02)	0.27 (0.02, 2.53)	0.37 (0.03, 3.69)	0.41 (0.04, 3.53)	**0.09 (0.00, 0.91)**	0.08 (0.00, 1.01)
UPA45mgPO	0.65 (0.02, 8.37)	2.06 (0.66, 6.06)	3.17 (0.61, 25.99)	1.38 (0.63, 3.06)	0.40 (0.02, 5.20)	1.64 (0.23, 13.43)	2.12 (0.36, 17.45)	0.36 (0.02, 5.54)	1.07 (0.36, 3.30)	0.69 (0.21, 2.27)	1.50 (0.58, 3.77)	1.30 (0.58, 3.14)	1.28 (0.02, 67.85)	2.00 (0.16, 85.91)	1.74 (0.12, 77.06)	0.18 (0.01, 1.58)	0.68 (0.11, 3.80)	0.99 (0.20, 4.69)	1.03 (0.25, 4.29)	0.25 (0.02, 1.26)	0.21 (0.01, 1.71)
UPA6mgPO_BID	0.61 (0.01, 21.04)	2.16 (0.20, 27.47)	3.38 (0.21, 70.10)	1.42 (0.14, 16.29)	0.42 (0.01, 10.92)	1.71 (0.09, 41.20)	2.32 (0.13, 48.70)	0.38 (0.01, 11.42)	1.14 (0.10, 13.51)	0.73 (0.06, 8.66)	1.55 (0.14, 18.17)	1.33 (0.13, 16.23)	1.31 (0.02, 125.57)	2.18 (0.07, 150.54)	1.94 (0.05, 145.14)	0.18 (0.00, 4.23)	0.73 (0.04, 10.38)	1.04 (0.07, 16.77)	1.06 (0.08, 16.29)	0.26 (0.01, 3.81)	0.21 (0.01, 5.06)
UST130mgIV	0.77 (0.02, 10.24)	2.47 (0.78, 7.73)	3.81 (0.74, 32.13)	1.68 (0.71, 3.90)	0.48 (0.02, 6.74)	2.01 (0.29, 16.64)	2.60 (0.43, 21.37)	0.44 (0.02, 6.57)	1.29 (0.40, 4.21)	0.85 (0.25, 2.79)	1.81 (0.68, 4.78)	1.58 (0.66, 4.03)	1.50 (0.02, 83.45)	2.41 (0.19, 104.22)	2.12 (0.14, 103.12)	0.22 (0.01, 2.00)	0.83 (0.14, 4.55)	1.21 (0.24, 5.87)	1.27 (0.29, 5.22)	0.30 (0.03, 1.55)	0.26 (0.01, 2.13)
UST1mg_kgIV	1.14 (0.03, 19.11)	3.69 (0.94, 15.54)	5.65 (0.90, 60.35)	2.48 (0.81, 8.92)	0.69 (0.03, 13.41)	2.95 (0.35, 31.74)	3.88 (0.58, 41.40)	0.66 (0.03, 11.78)	1.92 (0.49, 9.15)	1.24 (0.28, 6.16)	2.68 (0.78, 10.31)	2.38 (0.75, 8.73)	2.30 (0.03, 138.39)	3.68 (0.26, 160.39)	3.20 (0.21, 166.11)	0.33 (0.01, 3.62)	1.22 (0.18, 9.25)	1.78 (0.30, 12.36)	1.89 (0.35, 10.80)	0.45 (0.04, 2.84)	0.39 (0.01, 3.93)
UST3mg_kgIV	0.89 (0.02, 13.91)	2.79 (0.71, 10.57)	4.24 (0.74, 42.78)	1.88 (0.64, 5.98)	0.54 (0.02, 7.96)	2.24 (0.28, 21.97)	2.94 (0.45, 28.46)	0.50 (0.02, 8.30)	1.47 (0.39, 5.98)	0.95 (0.24, 4.03)	2.02 (0.63, 6.92)	1.79 (0.61, 5.92)	1.73 (0.03, 107.32)	2.69 (0.21, 126.55)	2.42 (0.16, 124.32)	0.25 (0.01, 2.76)	0.95 (0.14, 6.22)	1.35 (0.24, 7.91)	1.45 (0.28, 7.12)	0.33 (0.03, 2.04)	0.29 (0.01, 2.51)
UST4_5mg_kgIV	0.22 (0.00, 9.11)	0.78 (0.02, 11.68)	1.17 (0.03, 30.16)	0.52 (0.01, 7.40)	0.14 (0.00, 5.93)	0.59 (0.01, 16.73)	0.79 (0.02, 21.78)	0.13 (0.00, 5.49)	0.39 (0.01, 6.38)	0.25 (0.01, 4.44)	0.56 (0.02, 8.48)	0.49 (0.01, 7.44)	0.48 (0.00, 43.96)	0.77 (0.01, 57.80)	0.65 (0.01, 50.73)	0.06 (0.00, 2.17)	0.25 (0.01, 5.19)	0.35 (0.01, 7.03)	0.40 (0.01, 6.72)	0.09 (0.00, 1.82)	0.07 (0.00, 2.17)
UST6mg_kg90mgIV_SC	0.63 (0.01, 11.82)	1.95 (0.31, 12.08)	3.11 (0.35, 38.05)	1.32 (0.27, 6.61)	0.39 (0.01, 6.94)	1.61 (0.13, 19.86)	2.13 (0.20, 24.59)	0.35 (0.01, 7.82)	1.03 (0.18, 6.37)	0.66 (0.10, 4.50)	1.43 (0.27, 7.49)	1.28 (0.26, 6.60)	1.22 (0.02, 86.29)	1.97 (0.10, 114.33)	1.75 (0.07, 114.61)	0.18 (0.01, 1.53)	0.67 (0.11, 3.28)	0.94 (0.22, 4.65)	0.99 (0.14, 7.31)	0.24 (0.02, 2.00)	0.21 (0.01, 2.53)
UST6mg_kgIV	0.73 (0.02, 9.60)	2.34 (0.77, 6.80)	3.60 (0.73, 32.24)	1.57 (0.72, 3.58)	0.45 (0.02, 6.44)	1.88 (0.29, 15.06)	2.42 (0.42, 19.91)	0.42 (0.02, 6.24)	1.22 (0.41, 3.80)	0.79 (0.24, 2.63)	1.71 (0.67, 4.31)	1.50 (0.65, 3.61)	1.44 (0.02, 77.43)	2.30 (0.18, 96.56)	1.99 (0.13, 93.52)	0.21 (0.01, 1.92)	0.78 (0.13, 4.23)	1.13 (0.24, 5.41)	1.19 (0.28, 4.93)	0.28 (0.02, 1.40)	0.24 (0.01, 1.93)
UST90mgSC	1.21 (0.01, 98.79)	4.19 (0.24, 108.34)	6.81 (0.26, 331.67)	2.80 (0.17, 76.43)	0.79 (0.01, 60.03)	3.52 (0.13, 165.91)	4.73 (0.15, 212.48)	0.75 (0.01, 48.85)	2.23 (0.12, 63.65)	1.43 (0.07, 42.26)	2.99 (0.18, 88.69)	2.62 (0.15, 73.93)	2.53 (0.02, 477.59)	4.51 (0.10, 653.99)	3.64 (0.08, 514.39)	0.37 (0.01, 17.03)	1.43 (0.06, 46.59)	2.06 (0.10, 62.38)	2.11 (0.12, 69.74)	0.50 (0.02, 16.00)	0.42 (0.01, 17.25)
VED0_5mg_kgIV	1.09 (0.03, 18.91)	3.54 (0.90, 16.72)	5.48 (0.85, 61.99)	2.43 (0.76, 9.00)	0.72 (0.03, 11.13)	2.87 (0.33, 29.24)	3.79 (0.50, 39.33)	0.62 (0.02, 12.31)	1.85 (0.47, 8.32)	1.25 (0.26, 5.66)	2.62 (0.74, 10.33)	2.28 (0.71, 8.94)	2.26 (0.04, 127.78)	3.74 (0.24, 148.08)	3.22 (0.16, 146.94)	0.32 (0.01, 3.62)	1.17 (0.16, 8.59)	1.72 (0.30, 11.92)	1.78 (0.34, 10.39)	0.43 (0.04, 2.85)	0.36 (0.01, 3.98)
VED2mg_kgIV	0.69 (0.02, 10.79)	2.24 (0.57, 8.39)	3.46 (0.55, 34.19)	1.51 (0.49, 4.43)	0.43 (0.02, 6.34)	1.78 (0.24, 17.20)	2.30 (0.34, 21.23)	0.39 (0.02, 6.98)	1.16 (0.30, 4.60)	0.75 (0.18, 3.11)	1.58 (0.50, 5.40)	1.41 (0.47, 4.63)	1.36 (0.02, 88.39)	2.20 (0.16, 109.13)	1.94 (0.12, 94.58)	0.20 (0.01, 2.05)	0.74 (0.11, 4.70)	1.08 (0.20, 6.07)	1.13 (0.21, 5.58)	0.27 (0.02, 1.63)	0.22 (0.01, 2.04)
VED300mgIV	0.63 (0.02, 8.21)	1.99 (0.67, 5.80)	3.02 (0.61, 26.30)	1.35 (0.62, 2.87)	0.38 (0.02, 5.48)	1.61 (0.23, 12.75)	2.07 (0.36, 17.15)	0.36 (0.02, 5.19)	1.04 (0.36, 3.22)	0.67 (0.21, 2.23)	1.44 (0.57, 3.68)	1.28 (0.57, 3.07)	1.25 (0.02, 67.84)	1.99 (0.17, 79.54)	1.69 (0.12, 75.38)	0.18 (0.01, 1.55)	0.66 (0.11, 3.74)	0.96 (0.20, 4.75)	1.00 (0.25, 4.16)	0.25 (0.02, 1.21)	0.21 (0.01, 1.65)

	MIR600mgIV	NAT300mgIV	NAT3mg_kgIV	NAT3mg_kgIVx2	NAT6mg_kgIVx2	NNC2mg_kgSC	ONE10mgSC	ONE25mgSC	ONE35mgSC	ONE50mgSC	ONT22_5mgSC	ONT225mgSC	ONT75mgSC	PBO	PF10mgSC	PF200mgSC	PF50mgSC	RIS1200mgIV	RIS200mgIV	RIS600mgIV	SEC10mg_kgIV
ABA10mg_kgIV	0.72 (0.11, 3.06)	0.71 (0.29, 1.68)	0.81 (0.22, 3.08)	0.72 (0.18, 2.87)	0.94 (0.23, 3.71)	2.45 (0.40, 19.66)	0.69 (0.03, 26.09)	0.84 (0.02, 32.66)	3.29 (0.29, 83.69)	0.72 (0.01, 24.56)	1.97 (0.55, 8.26)	1.94 (0.55, 7.93)	1.62 (0.42, 6.98)	0.90 (0.44, 1.83)	0.71 (0.20, 2.47)	1.87 (0.59, 6.20)	0.90 (0.27, 2.96)	**0.24 (0.10, 0.62)**	0.60 (0.16, 2.00)	**0.38 (0.16, 0.89)**	1.14 (0.27, 6.43)
ABA30mg_kgIV	0.74 (0.11, 3.58)	0.74 (0.28, 1.91)	0.84 (0.23, 3.49)	0.76 (0.19, 3.13)	0.98 (0.23, 4.13)	2.54 (0.41, 21.58)	0.74 (0.03, 28.32)	0.89 (0.02, 34.11)	3.41 (0.28, 84.26)	0.77 (0.01, 25.54)	2.07 (0.54, 8.97)	2.00 (0.56, 8.52)	1.71 (0.45, 7.61)	0.95 (0.43, 2.10)	0.74 (0.20, 2.76)	1.96 (0.57, 7.39)	0.92 (0.26, 3.50)	**0.26 (0.10, 0.69)**	0.62 (0.16, 2.24)	0.40 (0.16, 1.01)	1.19 (0.27, 6.99)
ABA3mg_kgIV	0.82 (0.12, 3.51)	0.80 (0.32, 1.93)	0.92 (0.26, 3.50)	0.81 (0.20, 3.21)	1.08 (0.26, 4.15)	2.76 (0.45, 21.66)	0.77 (0.03, 29.45)	0.94 (0.02, 36.99)	3.69 (0.32, 106.11)	0.80 (0.01, 28.87)	2.22 (0.62, 9.16)	2.17 (0.61, 8.65)	1.84 (0.48, 7.82)	1.02 (0.49, 2.08)	0.79 (0.22, 2.76)	2.12 (0.66, 7.51)	1.00 (0.29, 3.43)	**0.28 (0.11, 0.70)**	0.68 (0.18, 2.21)	0.43 (0.18, 1.01)	1.30 (0.30, 7.29)
ADA160mg80mg40mgSC	0.62 (0.06, 5.03)	0.62 (0.11, 3.41)	0.72 (0.11, 4.99)	0.63 (0.09, 4.66)	0.82 (0.11, 6.11)	2.10 (0.21, 27.48)	0.60 (0.02, 29.15)	0.71 (0.01, 33.25)	2.80 (0.16, 98.59)	0.62 (0.01, 31.07)	1.76 (0.25, 12.86)	1.70 (0.24, 12.83)	1.43 (0.20, 10.54)	0.79 (0.16, 4.16)	0.62 (0.09, 4.16)	1.64 (0.25, 10.55)	0.77 (0.12, 5.10)	0.21 (0.04, 1.22)	0.51 (0.07, 3.50)	0.33 (0.06, 1.89)	1.01 (0.12, 9.45)
ADA160mg80mg60mgSC	0.31 (0.01, 5.70)	0.32 (0.01, 4.23)	0.38 (0.01, 5.98)	0.32 (0.01, 5.44)	0.42 (0.01, 6.68)	1.07 (0.02, 28.62)	0.30 (0.00, 25.97)	0.35 (0.00, 30.09)	1.44 (0.02, 90.50)	0.31 (0.00, 26.96)	0.89 (0.03, 15.26)	0.87 (0.03, 14.27)	0.72 (0.02, 12.75)	0.40 (0.01, 5.22)	0.32 (0.01, 5.01)	0.83 (0.03, 13.25)	0.40 (0.01, 6.07)	0.11 (0.00, 1.56)	0.26 (0.01, 3.96)	0.17 (0.01, 2.34)	0.52 (0.01, 9.60)
ADA160mg80mgSC	1.42 (0.19, 7.17)	1.39 (0.51, 4.35)	1.65 (0.38, 7.02)	1.46 (0.32, 6.52)	1.89 (0.37, 8.48)	4.89 (0.66, 42.27)	1.39 (0.05, 55.24)	1.63 (0.04, 65.17)	6.53 (0.50, 153.95)	1.46 (0.02, 54.53)	3.90 (0.99, 18.78)	3.76 (0.98, 17.48)	3.25 (0.79, 15.40)	1.74 (0.74, 4.75)	1.38 (0.35, 6.09)	**3.66 (1.03, 14.93)**	1.72 (0.47, 7.37)	0.48 (0.16, 1.51)	1.17 (0.28, 4.73)	0.75 (0.27, 2.28)	2.27 (0.48, 15.04)
ADA40mg20mgSC	4.31 (0.31, 136.65)	4.12 (0.55, 112.93)	4.90 (0.50, 145.65)	4.36 (0.44, 134.39)	5.49 (0.52, 176.08)	15.58 (0.98, 674.36)	4.27 (0.11, 440.42)	5.47 (0.09, 458.77)	22.40 (0.74, 1707.17)	4.77 (0.04, 478.13)	**11.67 (1.20, 377.10)**	**11.44 (1.16, 403.73)**	9.92 (0.96, 328.46)	5.24 (0.75, 139.01)	4.14 (0.46, 130.33)	**11.22 (1.29, 324.12)**	5.34 (0.54, 148.36)	1.41 (0.19, 39.67)	3.43 (0.36, 107.48)	2.20 (0.30, 64.14)	6.84 (0.67, 229.91)
ADA80mg40mgSC	3.48 (0.40, 44.59)	3.44 (0.77, 30.76)	4.19 (0.63, 45.29)	3.61 (0.55, 37.79)	4.69 (0.73, 52.01)	**12.28 (1.25, 212.00)**	3.55 (0.11, 205.77)	4.31 (0.09, 212.07)	16.74 (0.96, 636.82)	3.82 (0.05, 175.57)	**9.89 (1.69, 110.69)**	**9.56 (1.57, 102.18)**	**8.14 (1.31, 91.63)**	**4.30 (1.05, 35.51)**	3.46 (0.57, 36.90)	**9.33 (1.64, 93.49)**	4.45 (0.73, 43.04)	1.19 (0.26, 10.27)	2.98 (0.50, 29.99)	1.87 (0.42, 15.71)	5.75 (0.86, 83.73)
AMI0_4mgPO	0.09 (0.00, 1.25)	**0.10 (0.00, 0.78)**	0.12 (0.00, 1.15)	0.10 (0.00, 1.02)	0.13 (0.01, 1.28)	0.33 (0.01, 5.94)	0.09 (0.00, 5.34)	0.11 (0.00, 6.25)	0.45 (0.01, 15.19)	0.09 (0.00, 4.96)	0.29 (0.01, 2.88)	0.27 (0.01, 2.83)	0.23 (0.01, 2.47)	**0.13 (0.01, 0.92)**	**0.10 (0.00, 0.91)**	0.27 (0.01, 2.46)	0.13 (0.00, 1.11)	**0.04 (0.00, 0.28)**	**0.08 (0.00, 0.79)**	**0.06 (0.00, 0.43)**	0.17 (0.01, 2.47)
AND150mgSC1_1	0.68 (0.07, 4.91)	0.70 (0.12, 3.16)	0.79 (0.10, 4.98)	0.70 (0.09, 4.74)	0.90 (0.12, 5.83)	2.38 (0.22, 28.18)	0.66 (0.02, 28.90)	0.78 (0.02, 40.09)	3.08 (0.18, 104.18)	0.67 (0.01, 31.04)	1.94 (0.26, 12.89)	1.86 (0.28, 11.81)	1.60 (0.21, 10.57)	0.90 (0.18, 3.68)	0.69 (0.10, 3.96)	1.87 (0.27, 9.95)	0.87 (0.12, 4.63)	0.24 (0.04, 1.14)	0.58 (0.08, 3.27)	0.38 (0.06, 1.67)	1.11 (0.13, 8.97)
AND150mgSC2_2	6.00 (0.32, 249.09)	5.81 (0.56, 199.56)	6.57 (0.55, 290.14)	6.09 (0.42, 243.48)	7.62 (0.53, 356.10)	**20.97 (1.08, 1098.44)**	6.36 (0.09, 828.22)	7.07 (0.11, 949.81)	30.25 (0.98, 3312.06)	6.42 (0.04, 767.79)	**16.61 (1.11, 707.90)**	**16.02 (1.21, 661.09)**	13.51 (0.99, 565.96)	7.44 (0.71, 251.02)	5.77 (0.46, 229.35)	**15.54 (1.30, 629.27)**	7.28 (0.58, 289.97)	2.01 (0.18, 74.67)	4.91 (0.36, 185.29)	3.13 (0.30, 111.60)	9.87 (0.63, 405.86)
AND300mgSC	0.50 (0.05, 3.44)	0.50 (0.09, 2.16)	0.58 (0.08, 3.30)	0.51 (0.07, 3.08)	0.68 (0.09, 3.93)	1.67 (0.18, 19.47)	0.47 (0.01, 22.00)	0.57 (0.01, 28.24)	2.27 (0.13, 71.82)	0.49 (0.01, 22.87)	1.39 (0.19, 8.91)	1.36 (0.21, 8.27)	1.15 (0.16, 7.32)	0.64 (0.13, 2.49)	0.50 (0.08, 2.76)	1.34 (0.21, 7.01)	0.63 (0.09, 3.30)	**0.17 (0.03, 0.75)**	0.42 (0.06, 2.25)	0.27 (0.05, 1.16)	0.82 (0.10, 6.41)
API100mgPR	1.68 (0.20, 10.58)	1.63 (0.43, 7.37)	1.92 (0.37, 11.17)	1.67 (0.32, 10.07)	2.20 (0.40, 12.65)	5.67 (0.67, 67.04)	1.57 (0.06, 69.36)	1.98 (0.04, 95.08)	7.83 (0.53, 250.21)	1.73 (0.02, 70.82)	4.69 (0.91, 25.56)	4.49 (0.84, 25.52)	3.84 (0.71, 22.32)	2.08 (0.62, 8.61)	1.66 (0.33, 8.86)	4.36 (0.89, 23.01)	2.11 (0.42, 10.70)	0.56 (0.15, 2.62)	1.35 (0.27, 7.27)	0.87 (0.22, 3.91)	2.67 (0.46, 20.49)
API50mgPO	1.08 (0.15, 6.47)	1.07 (0.31, 3.99)	1.25 (0.25, 6.36)	1.11 (0.21, 5.57)	1.43 (0.27, 7.26)	3.63 (0.51, 37.08)	1.02 (0.04, 43.38)	1.31 (0.03, 53.22)	4.91 (0.36, 141.72)	1.12 (0.02, 39.58)	3.04 (0.66, 15.13)	2.97 (0.62, 14.45)	2.51 (0.53, 12.24)	1.35 (0.45, 4.45)	1.08 (0.22, 5.13)	2.82 (0.65, 13.75)	1.35 (0.29, 6.37)	0.37 (0.11, 1.42)	0.88 (0.19, 4.33)	0.57 (0.17, 2.17)	1.75 (0.33, 12.36)
BRI400mgIV	1.59 (0.14, 18.95)	1.59 (0.27, 12.95)	1.83 (0.25, 18.95)	1.64 (0.20, 17.70)	2.06 (0.28, 23.71)	5.62 (0.54, 86.64)	1.61 (0.04, 72.55)	1.96 (0.04, 108.02)	7.71 (0.39, 279.89)	1.68 (0.02, 77.68)	4.52 (0.62, 46.47)	4.45 (0.63, 46.18)	3.70 (0.51, 39.07)	1.99 (0.36, 15.42)	1.58 (0.22, 15.79)	4.29 (0.62, 39.31)	1.97 (0.27, 19.38)	0.54 (0.10, 4.59)	1.34 (0.17, 12.84)	0.87 (0.15, 6.90)	2.68 (0.33, 32.90)
BRI700mgIV	2.32 (0.24, 18.08)	2.34 (0.47, 11.25)	2.71 (0.43, 18.59)	2.43 (0.34, 16.00)	3.09 (0.45, 22.12)	8.08 (0.93, 91.83)	2.37 (0.07, 90.77)	2.81 (0.06, 130.21)	10.98 (0.66, 339.02)	2.42 (0.04, 100.96)	6.67 (1.00, 46.11)	**6.47 (1.02, 43.20)**	5.38 (0.85, 38.25)	3.00 (0.66, 13.36)	2.34 (0.36, 15.35)	**6.26 (1.06, 40.47)**	2.89 (0.47, 19.13)	0.82 (0.16, 3.92)	1.97 (0.32, 11.51)	1.28 (0.25, 6.02)	3.88 (0.53, 35.18)
BRO210mgIV	0.46 (0.03, 4.51)	0.48 (0.05, 3.07)	0.54 (0.05, 4.77)	0.49 (0.05, 4.30)	0.63 (0.06, 5.75)	1.65 (0.11, 23.20)	0.43 (0.01, 24.62)	0.56 (0.01, 27.23)	2.20 (0.08, 82.65)	0.47 (0.00, 18.92)	1.31 (0.14, 12.31)	1.26 (0.13, 11.20)	1.08 (0.11, 9.81)	0.61 (0.07, 3.69)	0.47 (0.04, 3.80)	1.28 (0.13, 9.72)	0.59 (0.06, 4.92)	0.17 (0.02, 1.12)	0.40 (0.03, 3.10)	0.26 (0.03, 1.65)	0.77 (0.07, 8.61)
BRO350mgIV	0.17 (0.01, 1.22)	**0.17 (0.02, 0.81)**	0.20 (0.02, 1.33)	0.17 (0.02, 1.17)	0.22 (0.02, 1.48)	0.59 (0.04, 6.91)	0.16 (0.00, 8.28)	0.20 (0.00, 9.51)	0.81 (0.03, 24.73)	0.17 (0.00, 5.95)	0.47 (0.05, 3.53)	0.46 (0.05, 3.17)	0.39 (0.04, 2.92)	**0.22 (0.03, 0.97)**	0.17 (0.02, 1.05)	0.45 (0.05, 2.65)	0.22 (0.02, 1.25)	**0.06 (0.01, 0.29)**	**0.14 (0.02, 0.85)**	**0.09 (0.01, 0.44)**	0.28 (0.03, 2.28)
BRO700mgIV	0.15 (0.01, 1.13)	**0.15 (0.02, 0.69)**	0.17 (0.02, 1.05)	**0.15 (0.02, 0.99)**	0.20 (0.02, 1.30)	0.51 (0.04, 6.27)	0.14 (0.00, 7.66)	0.17 (0.00, 7.27)	0.69 (0.03, 21.24)	0.15 (0.00, 5.35)	0.42 (0.05, 3.04)	0.41 (0.05, 2.76)	0.34 (0.04, 2.44)	**0.19 (0.03, 0.78)**	**0.15 (0.02, 0.95)**	0.40 (0.04, 2.37)	0.19 (0.02, 1.13)	**0.05 (0.01, 0.24)**	**0.13 (0.01, 0.71)**	**0.08 (0.01, 0.35)**	0.25 (0.03, 2.00)
CDP10mg_kgIV	1.03 (0.15, 4.57)	1.01 (0.42, 2.51)	1.19 (0.32, 4.36)	1.04 (0.27, 3.97)	1.35 (0.34, 5.17)	3.51 (0.57, 28.50)	0.98 (0.04, 37.16)	1.20 (0.03, 43.82)	4.70 (0.39, 119.12)	1.04 (0.02, 36.75)	2.83 (0.79, 11.72)	2.75 (0.77, 11.07)	2.34 (0.64, 9.58)	1.30 (0.62, 2.63)	1.01 (0.29, 3.57)	2.71 (0.86, 9.31)	1.26 (0.38, 4.55)	**0.35 (0.14, 0.89)**	0.85 (0.24, 2.91)	0.56 (0.23, 1.30)	1.64 (0.39, 9.18)
CER100mgSC	0.60 (0.08, 3.42)	0.59 (0.19, 1.95)	0.69 (0.15, 3.28)	0.61 (0.12, 2.95)	0.79 (0.16, 3.74)	2.08 (0.29, 19.07)	0.56 (0.02, 22.75)	0.69 (0.02, 25.74)	2.79 (0.22, 70.69)	0.61 (0.01, 20.68)	1.67 (0.39, 8.48)	1.62 (0.38, 8.36)	1.38 (0.29, 7.36)	0.75 (0.28, 2.23)	0.59 (0.14, 2.90)	1.58 (0.41, 7.28)	0.75 (0.17, 3.38)	**0.20 (0.07, 0.70)**	0.50 (0.11, 2.17)	0.32 (0.10, 1.08)	0.96 (0.18, 7.15)

(TABLE S16A. continued)

	MIR600mgIV	NAT300mgIV	NAT3mg_kgIV	NAT3mg_kgIVx2	NAT6mg_kgIVx2	NNC2mg_kgSC	ONE10mgSC	ONE25mgSC	ONE35mgSC	ONE50mgSC	ONT22_5mgSC	ONT225mgSC	ONT75mgSC	PBO	PF10mgSC	PF200mgSC	PF50mgSC	RIS1200mgIV	RIS200mgIV	RIS600mgIV	SEC10mg_kgIV
CER10mgIV	1.28 (0.06, 52.75)	1.26 (0.10, 43.82)	1.47 (0.10, 60.24)	1.32 (0.07, 55.66)	1.74 (0.10, 67.10)	4.69 (0.21, 263.62)	1.34 (0.03, 211.95)	1.56 (0.02, 305.81)	6.34 (0.20, 754.89)	1.48 (0.01, 233.75)	3.70 (0.23, 148.17)	3.53 (0.23, 141.82)	2.95 (0.19, 122.55)	1.56 (0.13, 54.67)	1.26 (0.08, 54.33)	3.36 (0.24, 137.61)	1.58 (0.11, 67.09)	0.44 (0.03, 15.90)	1.07 (0.07, 46.14)	0.67 (0.05, 23.44)	2.18 (0.13, 100.89)
CER200mgSC	0.41 (0.06, 2.06)	0.40 (0.13, 1.18)	0.46 (0.11, 2.04)	0.41 (0.09, 1.91)	0.53 (0.11, 2.42)	1.36 (0.19, 12.14)	0.38 (0.01, 13.92)	0.47 (0.01, 17.63)	1.87 (0.15, 50.24)	0.41 (0.01, 13.03)	1.12 (0.26, 5.29)	1.09 (0.26, 5.01)	0.91 (0.21, 4.34)	0.50 (0.19, 1.35)	0.39 (0.10, 1.66)	1.03 (0.27, 4.32)	0.50 (0.12, 1.99)	**0.14 (0.04, 0.43)**	0.33 (0.08, 1.32)	**0.21 (0.07, 0.64)**	0.64 (0.13, 3.98)
CER20mgIV	0.25 (0.02, 1.95)	0.26 (0.03, 1.27)	0.30 (0.03, 1.95)	0.26 (0.02, 1.75)	0.34 (0.03, 2.28)	0.87 (0.06, 11.14)	0.25 (0.01, 9.78)	0.30 (0.01, 14.02)	1.20 (0.06, 32.04)	0.26 (0.00, 9.63)	0.71 (0.08, 4.93)	0.69 (0.08, 4.71)	0.59 (0.06, 4.22)	0.33 (0.04, 1.51)	0.25 (0.03, 1.64)	0.67 (0.07, 4.27)	0.32 (0.03, 1.96)	**0.09 (0.01, 0.45)**	0.22 (0.02, 1.35)	**0.14 (0.02, 0.69)**	0.41 (0.04, 3.83)
CER400mgSC	0.59 (0.10, 2.45)	0.58 (0.27, 1.31)	0.67 (0.19, 2.40)	0.59 (0.17, 2.21)	0.77 (0.20, 2.89)	1.98 (0.35, 15.71)	0.57 (0.02, 20.96)	0.70 (0.02, 24.77)	2.69 (0.23, 66.02)	0.60 (0.01, 18.78)	1.62 (0.48, 6.51)	1.58 (0.48, 6.04)	1.35 (0.40, 5.24)	0.74 (0.42, 1.33)	0.58 (0.18, 1.95)	1.53 (0.53, 5.27)	0.73 (0.23, 2.53)	**0.20 (0.09, 0.46)**	0.49 (0.15, 1.52)	**0.32 (0.15, 0.66)**	0.95 (0.23, 5.13)
CER5mgIV	2.04 (0.09, 67.92)	2.02 (0.15, 44.68)	2.38 (0.15, 64.42)	2.04 (0.13, 54.77)	2.69 (0.17, 70.30)	7.03 (0.38, 273.17)	2.08 (0.04, 205.00)	2.64 (0.03, 188.48)	10.36 (0.26, 689.95)	2.24 (0.02, 207.41)	5.90 (0.34, 154.42)	5.73 (0.35, 140.10)	4.75 (0.30, 134.56)	2.59 (0.20, 53.93)	2.02 (0.12, 52.73)	5.45 (0.35, 133.94)	2.49 (0.16, 60.87)	0.70 (0.05, 15.50)	1.70 (0.11, 43.19)	1.11 (0.08, 24.29)	3.36 (0.23, 103.35)
ELD10mg_kgIV	0.47 (0.03, 5.40)	0.49 (0.06, 3.41)	0.56 (0.06, 5.13)	0.49 (0.05, 4.45)	0.65 (0.06, 5.97)	1.74 (0.12, 26.36)	0.49 (0.01, 29.04)	0.56 (0.01, 33.55)	2.30 (0.09, 88.56)	0.48 (0.01, 25.15)	1.37 (0.14, 13.00)	1.37 (0.13, 12.41)	1.15 (0.11, 10.32)	0.63 (0.08, 4.14)	0.49 (0.05, 4.07)	1.31 (0.14, 10.61)	0.62 (0.06, 5.08)	0.17 (0.02, 1.22)	0.41 (0.04, 3.33)	0.27 (0.03, 1.88)	0.83 (0.07, 9.93)
ELD20mg_kgIV	0.36 (0.03, 3.37)	0.37 (0.05, 2.29)	0.43 (0.05, 3.26)	0.38 (0.04, 2.97)	0.49 (0.05, 4.03)	1.31 (0.10, 17.70)	0.37 (0.01, 17.26)	0.42 (0.01, 20.77)	1.76 (0.08, 58.14)	0.37 (0.00, 20.02)	1.09 (0.10, 8.18)	1.04 (0.10, 7.67)	0.91 (0.08, 6.78)	0.48 (0.06, 2.60)	0.38 (0.04, 2.72)	0.99 (0.11, 6.71)	0.48 (0.05, 3.16)	**0.13 (0.02, 0.76)**	0.31 (0.03, 2.19)	0.20 (0.02, 1.15)	0.62 (0.06, 6.32)
ETA25mgSC	2.16 (0.09, 67.50)	2.19 (0.15, 48.74)	2.57 (0.15, 68.08)	2.28 (0.12, 61.18)	2.91 (0.16, 77.74)	8.04 (0.31, 286.71)	2.23 (0.04, 257.84)	2.75 (0.03, 403.24)	10.79 (0.27, 1011.21)	2.27 (0.02, 293.56)	6.32 (0.35, 155.04)	6.06 (0.34, 148.67)	5.11 (0.30, 124.95)	2.80 (0.20, 62.42)	2.16 (0.13, 60.08)	5.89 (0.35, 148.45)	2.76 (0.15, 75.60)	0.75 (0.05, 17.60)	1.85 (0.11, 47.85)	1.19 (0.08, 26.52)	3.80 (0.18, 114.89)
ETR105mgSC	0.75 (0.11, 3.96)	0.76 (0.25, 2.27)	0.87 (0.20, 3.76)	0.77 (0.17, 3.51)	1.01 (0.22, 4.29)	2.62 (0.38, 23.29)	0.72 (0.03, 31.42)	0.86 (0.02, 36.24)	3.47 (0.25, 94.12)	0.77 (0.01, 27.76)	2.10 (0.49, 10.24)	2.05 (0.48, 9.31)	1.74 (0.40, 8.20)	0.96 (0.35, 2.54)	0.75 (0.18, 3.09)	2.01 (0.53, 7.95)	0.93 (0.23, 3.68)	**0.26 (0.08, 0.80)**	0.64 (0.15, 2.54)	0.41 (0.13, 1.17)	1.23 (0.25, 8.02)
ETR210mgSC	1.20 (0.15, 6.23)	1.17 (0.35, 3.81)	1.36 (0.30, 6.27)	1.17 (0.25, 5.97)	1.55 (0.31, 7.13)	3.95 (0.54, 37.21)	1.12 (0.04, 48.57)	1.37 (0.03, 55.51)	5.31 (0.36, 152.09)	1.16 (0.02, 38.96)	3.19 (0.73, 16.38)	3.13 (0.72, 15.33)	2.66 (0.60, 13.54)	1.50 (0.51, 4.27)	1.16 (0.25, 4.97)	3.12 (0.74, 12.95)	1.45 (0.33, 5.96)	0.40 (0.12, 1.36)	0.96 (0.22, 4.19)	0.63 (0.19, 2.03)	1.88 (0.36, 12.14)
FIL100mgPO	0.56 (0.08, 2.46)	0.54 (0.22, 1.37)	0.63 (0.16, 2.49)	0.55 (0.14, 2.24)	0.71 (0.17, 2.93)	1.83 (0.30, 15.85)	0.52 (0.02, 18.94)	0.64 (0.02, 25.30)	2.49 (0.21, 71.13)	0.55 (0.01, 18.57)	1.51 (0.40, 6.31)	1.46 (0.41, 5.91)	1.25 (0.33, 5.53)	0.69 (0.32, 1.48)	0.54 (0.15, 1.94)	1.45 (0.44, 4.91)	0.68 (0.19, 2.34)	**0.19 (0.07, 0.49)**	0.45 (0.12, 1.60)	**0.29 (0.12, 0.72)**	0.87 (0.20, 5.07)
FIL200mgPO	0.62 (0.09, 2.62)	0.62 (0.26, 1.40)	0.71 (0.20, 2.51)	0.63 (0.17, 2.33)	0.82 (0.20, 3.02)	2.09 (0.35, 17.41)	0.60 (0.02, 21.91)	0.74 (0.02, 25.67)	2.82 (0.23, 69.96)	0.62 (0.01, 22.24)	1.71 (0.49, 6.66)	1.65 (0.48, 6.24)	1.42 (0.40, 5.44)	0.78 (0.39, 1.50)	0.62 (0.17, 2.01)	1.65 (0.52, 5.28)	0.77 (0.22, 2.51)	**0.21 (0.09, 0.50)**	0.52 (0.15, 1.63)	**0.33 (0.14, 0.73)**	1.00 (0.23, 5.32)
FON0_1mg_kgIV	0.63 (0.01, 43.35)	0.63 (0.01, 41.14)	0.74 (0.01, 50.21)	0.63 (0.01, 42.01)	0.82 (0.01, 56.03)	2.27 (0.03, 190.81)	0.65 (0.00, 95.79)	0.77 (0.00, 107.16)	3.08 (0.03, 337.33)	0.64 (0.00, 96.83)	1.83 (0.03, 118.34)	1.78 (0.03, 113.95)	1.51 (0.03, 100.93)	0.81 (0.02, 50.21)	0.64 (0.01, 39.67)	1.73 (0.03, 106.99)	0.80 (0.01, 53.06)	0.22 (0.00, 13.54)	0.52 (0.01, 36.64)	0.35 (0.01, 22.06)	1.02 (0.02, 77.09)
FON1mg_kgIV	0.39 (0.01, 6.67)	0.40 (0.01, 4.91)	0.47 (0.01, 6.35)	0.42 (0.01, 5.38)	0.53 (0.01, 7.74)	1.39 (0.02, 29.09)	0.40 (0.00, 23.12)	0.44 (0.00, 29.99)	1.92 (0.03, 89.10)	0.39 (0.00, 23.97)	1.15 (0.02, 16.60)	1.12 (0.02, 15.71)	0.96 (0.02, 12.70)	0.51 (0.01, 6.10)	0.39 (0.01, 5.68)	1.08 (0.03, 13.72)	0.49 (0.01, 6.75)	0.14 (0.00, 1.67)	0.34 (0.01, 4.53)	0.22 (0.01, 2.56)	0.67 (0.01, 12.35)
FON4mg_kgIV	0.45 (0.01, 9.29)	0.46 (0.01, 6.99)	0.53 (0.01, 9.85)	0.47 (0.01, 8.51)	0.60 (0.01, 11.29)	1.59 (0.02, 39.88)	0.43 (0.00, 34.18)	0.49 (0.00, 44.31)	2.10 (0.03, 152.28)	0.43 (0.00, 39.43)	1.29 (0.02, 22.97)	1.23 (0.02, 23.34)	1.05 (0.02, 19.52)	0.59 (0.01, 8.10)	0.44 (0.01, 8.00)	1.21 (0.02, 20.83)	0.56 (0.01, 9.79)	0.16 (0.00, 2.32)	0.38 (0.01, 6.39)	0.24 (0.01, 3.54)	0.76 (0.02, 14.43)
GUS1200mgIV	4.44 (0.33, 176.97)	4.49 (0.51, 144.02)	5.23 (0.51, 177.36)	4.66 (0.40, 159.67)	6.02 (0.54, 241.96)	**16.08 (1.05, 704.21)**	4.87 (0.11, 376.55)	5.59 (0.07, 527.03)	22.99 (0.79, 1507.47)	4.79 (0.05, 402.07)	**12.72 (1.12, 534.54)**	**12.16 (1.11, 526.50)**	10.36 (0.90, 456.34)	5.65 (0.68, 176.71)	4.60 (0.39, 164.57)	**11.72 (1.15, 420.28)**	5.47 (0.52, 200.53)	1.54 (0.17, 49.78)	3.67 (0.37, 139.38)	2.43 (0.27, 76.52)	7.60 (0.58, 370.51)
GUS200mgIV	1.21 (0.11, 9.28)	1.18 (0.21, 7.34)	1.38 (0.21, 10.71)	1.23 (0.17, 9.73)	1.58 (0.20, 12.84)	4.14 (0.42, 56.45)	1.19 (0.04, 60.96)	1.36 (0.03, 82.61)	5.61 (0.30, 223.39)	1.25 (0.01, 66.12)	3.30 (0.47, 28.84)	3.26 (0.46, 27.41)	2.66 (0.38, 23.36)	1.52 (0.30, 8.35)	1.17 (0.16, 8.76)	3.08 (0.48, 23.01)	1.50 (0.21, 11.12)	0.41 (0.07, 2.54)	0.99 (0.15, 7.12)	0.64 (0.12, 3.77)	1.97 (0.24, 18.93)
GUS600mgIV	0.84 (0.09, 6.09)	0.83 (0.17, 3.78)	0.96 (0.15, 6.11)	0.84 (0.13, 5.33)	1.10 (0.17, 6.83)	2.87 (0.31, 32.11)	0.79 (0.03, 40.09)	0.98 (0.02, 50.10)	3.93 (0.22, 138.89)	0.83 (0.01, 36.83)	2.37 (0.35, 14.82)	2.29 (0.35, 15.12)	1.88 (0.28, 12.67)	1.04 (0.23, 4.54)	0.83 (0.12, 5.15)	2.19 (0.38, 13.10)	1.04 (0.17, 5.96)	0.29 (0.06, 1.39)	0.69 (0.11, 4.07)	0.45 (0.09, 2.05)	1.36 (0.18, 11.97)
MED700IV	0.78 (0.08, 5.64)	0.78 (0.18, 3.41)	0.90 (0.16, 5.35)	0.80 (0.14, 4.70)	1.03 (0.18, 6.08)	2.74 (0.31, 31.40)	0.77 (0.03, 30.23)	0.92 (0.02, 37.74)	3.74 (0.25, 101.57)	0.80 (0.01, 31.02)	2.22 (0.39, 13.34)	2.17 (0.38, 12.26)	1.80 (0.31, 10.87)	0.99 (0.26, 3.73)	0.77 (0.14, 4.17)	2.07 (0.41, 11.23)	0.97 (0.18, 5.11)	0.27 (0.06, 1.17)	0.65 (0.12, 3.41)	0.42 (0.10, 1.74)	1.27 (0.20, 9.83)
MIR1000mgIV	3.31 (0.42, 35.64)	3.30 (0.65, 37.60)	3.77 (0.56, 54.33)	3.32 (0.46, 49.04)	4.37 (0.58, 64.17)	**10.88 (1.24, 237.30)**	3.26 (0.10, 181.65)	3.71 (0.09, 202.54)	15.63 (0.91, 615.16)	3.60 (0.05, 170.99)	**9.37 (1.38, 117.16)**	**9.07 (1.31, 111.89)**	**7.69 (1.09, 96.82)**	4.11 (0.89, 44.80)	3.24 (0.51, 38.20)	**8.61 (1.46, 98.79)**	4.08 (0.64, 46.66)	1.13 (0.22, 12.22)	2.73 (0.38, 33.99)	1.76 (0.35, 19.19)	5.48 (0.74, 86.55)
MIR200mgIV	3.73 (0.45, 100.90)	3.85 (0.49, 99.14)	4.49 (0.45, 128.15)	4.11 (0.35, 110.67)	5.11 (0.45, 150.48)	**13.64 (1.07, 493.51)**	4.11 (0.09, 395.23)	4.50 (0.08, 527.65)	18.59 (0.83, 1671.00)	4.14 (0.04, 399.46)	**10.71 (1.06, 323.70)**	10.61 (0.97, 306.85)	9.08 (0.85, 277.58)	4.80 (0.66, 120.49)	3.75 (0.40, 108.21)	**10.24 (1.05, 274.41)**	4.75 (0.52, 132.24)	1.31 (0.16, 37.39)	3.29 (0.31, 89.82)	2.06 (0.25, 53.73)	6.66 (0.54, 221.23)

(TABLE S16A. continued)

	MIR600mgIV	NAT300mgIV	NAT3mg_kgIV	NAT3mg_kgIVx2	NAT6mg_kgIVx2	NNC2mg_kgSC	ONE10mgSC	ONE25mgSC	ONE35mgSC	ONE50mgSC	ONT22_5mgSC	ONT225mgSC	ONT75mgSC	PBO	PF10mgSC	PF200mgSC	PF50mgSC	RIS1200mgIV	RIS200mgIV	RIS600mgIV	SEC10mg_kgIV
MIR600mgIV	MIR600mgIV	0.98 (0.25, 5.83)	1.16 (0.20, 8.98)	1.00 (0.17, 7.93)	1.34 (0.21, 10.07)	3.40 (0.43, 44.56)	1.00 (0.03, 41.24)	1.23 (0.03, 57.36)	4.83 (0.33, 148.95)	1.08 (0.02, 37.63)	2.84 (0.50, 23.52)	2.78 (0.46, 22.61)	2.30 (0.41, 17.78)	1.24 (0.35, 6.88)	0.98 (0.19, 7.00)	2.63 (0.55, 18.46)	1.24 (0.23, 8.73)	0.34 (0.08, 2.13)	0.82 (0.14, 5.90)	0.52 (0.14, 3.12)	1.66 (0.26, 16.13)
NAT300mgIV	1.02 (0.17, 4.01)	NAT300mgIV	1.15 (0.35, 3.97)	1.03 (0.27, 3.63)	1.33 (0.34, 4.78)	3.45 (0.59, 26.64)	0.95 (0.04, 38.48)	1.15 (0.03, 42.83)	4.61 (0.43, 115.10)	1.03 (0.01, 34.69)	2.77 (0.87, 10.61)	2.72 (0.86, 9.92)	2.30 (0.68, 8.79)	1.28 (0.74, 2.13)	0.99 (0.31, 3.15)	2.66 (0.94, 8.34)	1.24 (0.41, 3.91)	**0.35 (0.16, 0.75)**	0.84 (0.22, 2.61)	0.55 (0.26, 1.08)	1.61 (0.40, 8.58)
NAT3mg_kgIV	0.86 (0.11, 5.09)	0.87 (0.25, 2.84)	NAT3mg_kgIV	0.88 (0.26, 2.82)	1.14 (0.36, 3.64)	2.95 (0.42, 27.64)	0.85 (0.03, 35.24)	0.98 (0.03, 42.14)	3.92 (0.28, 117.97)	0.86 (0.01, 34.90)	2.44 (0.51, 12.14)	2.33 (0.52, 11.92)	1.99 (0.41, 10.70)	1.10 (0.36, 3.28)	0.86 (0.20, 3.79)	2.32 (0.57, 9.46)	1.07 (0.25, 4.52)	0.30 (0.09, 1.02)	0.71 (0.16, 3.16)	0.47 (0.14, 1.51)	1.40 (0.25, 9.24)
NAT3mg_kgIVx2	1.00 (0.13, 5.89)	0.97 (0.28, 3.69)	1.13 (0.35, 3.83)	NAT3mg_kgIVx2	1.29 (0.38, 4.43)	3.31 (0.46, 34.32)	0.96 (0.03, 40.33)	1.13 (0.03, 51.24)	4.41 (0.31, 148.01)	0.97 (0.02, 38.42)	2.76 (0.58, 14.20)	2.64 (0.57, 13.42)	2.25 (0.46, 12.29)	1.25 (0.39, 3.91)	0.96 (0.21, 4.54)	2.57 (0.63, 11.01)	1.21 (0.28, 5.33)	0.34 (0.09, 1.20)	0.81 (0.17, 3.81)	0.53 (0.16, 1.85)	1.61 (0.28, 10.87)
NAT6mg_kgIVx2	0.74 (0.10, 4.77)	0.75 (0.21, 2.95)	0.88 (0.27, 2.79)	0.77 (0.23, 2.61)	NAT6mg_kgIVx2	2.58 (0.33, 29.91)	0.72 (0.02, 29.76)	0.85 (0.02, 38.29)	3.43 (0.23, 103.15)	0.77 (0.01, 32.94)	2.14 (0.42, 11.31)	2.06 (0.42, 11.06)	1.76 (0.34, 9.74)	0.95 (0.30, 3.30)	0.76 (0.16, 3.84)	2.02 (0.48, 8.71)	0.95 (0.21, 4.47)	**0.26 (0.07, 0.97)**	0.63 (0.13, 3.03)	0.41 (0.12, 1.52)	1.23 (0.21, 8.83)
NNC2mg_kgSC	0.29 (0.02, 2.32)	0.29 (0.04, 1.70)	0.34 (0.04, 2.38)	0.30 (0.03, 2.19)	0.39 (0.03, 2.99)	NNC2mg_kgSC	0.29 (0.01, 12.93)	0.34 (0.00, 16.70)	1.35 (0.06, 46.65)	0.29 (0.00, 13.57)	0.81 (0.09, 6.43)	0.79 (0.09, 6.05)	0.67 (0.07, 5.39)	0.37 (0.05, 1.96)	0.29 (0.03, 2.05)	0.79 (0.09, 5.54)	0.36 (0.04, 2.45)	**0.10 (0.01, 0.58)**	0.25 (0.02, 1.69)	**0.16 (0.02, 0.87)**	0.48 (0.05, 4.55)
ONE10mgSC	1.00 (0.02, 32.07)	1.06 (0.03, 25.53)	1.18 (0.03, 34.61)	1.04 (0.02, 32.63)	1.39 (0.03, 40.48)	3.47 (0.08, 149.84)	ONE10mgSC	1.15 (0.04, 35.14)	4.40 (0.49, 91.79)	1.00 (0.01, 25.25)	2.90 (0.06, 80.30)	2.87 (0.07, 77.68)	2.37 (0.06, 63.71)	1.33 (0.04, 30.64)	1.03 (0.03, 29.73)	2.78 (0.07, 76.84)	1.26 (0.03, 36.54)	0.36 (0.01, 9.21)	0.88 (0.02, 23.85)	0.56 (0.02, 13.42)	1.73 (0.04, 61.22)
ONE25mgSC	0.81 (0.02, 34.07)	0.87 (0.02, 31.24)	1.02 (0.02, 39.60)	0.89 (0.02, 33.80)	1.18 (0.03, 45.79)	2.95 (0.06, 204.27)	0.87 (0.03, 26.96)	ONE25mgSC	3.84 (0.41, 103.58)	0.88 (0.02, 31.40)	2.43 (0.05, 103.60)	2.27 (0.05, 105.25)	1.90 (0.05, 90.37)	1.06 (0.03, 37.02)	0.87 (0.02, 34.55)	2.31 (0.06, 87.10)	1.06 (0.03, 41.19)	0.29 (0.01, 11.16)	0.70 (0.02, 28.49)	0.45 (0.01, 16.45)	1.47 (0.03, 73.57)
ONE35mgSC	0.21 (0.01, 2.99)	0.22 (0.01, 2.33)	0.26 (0.01, 3.56)	0.23 (0.01, 3.26)	0.29 (0.01, 4.31)	0.74 (0.02, 16.32)	0.23 (0.01, 2.04)	0.26 (0.01, 2.44)	ONE35mgSC	0.23 (0.01, 2.07)	0.60 (0.02, 8.80)	0.59 (0.02, 7.88)	0.51 (0.02, 6.72)	0.28 (0.01, 2.92)	0.22 (0.01, 2.70)	0.59 (0.02, 7.04)	0.27 (0.01, 3.37)	**0.08 (0.00, 0.85)**	0.18 (0.01, 2.34)	0.12 (0.00, 1.31)	0.35 (0.01, 6.18)
ONE50mgSC	0.93 (0.03, 61.19)	0.97 (0.03, 68.89)	1.17 (0.03, 75.80)	1.03 (0.03, 63.75)	1.29 (0.03, 80.67)	3.50 (0.07, 394.06)	1.00 (0.04, 69.49)	1.14 (0.03, 60.55)	4.39 (0.48, 188.45)	ONE50mgSC	2.75 (0.06, 179.96)	2.76 (0.06, 181.35)	2.27 (0.06, 157.26)	1.24 (0.04, 71.19)	0.95 (0.03, 56.68)	2.61 (0.08, 163.03)	1.19 (0.03, 72.59)	0.34 (0.01, 21.66)	0.82 (0.02, 54.42)	0.52 (0.02, 34.64)	1.68 (0.04, 129.92)
ONT22_5mgSC	0.35 (0.04, 2.00)	0.36 (0.09, 1.15)	0.41 (0.08, 1.95)	0.36 (0.07, 1.73)	0.47 (0.09, 2.38)	1.23 (0.16, 10.96)	0.34 (0.01, 15.43)	0.41 (0.01, 19.14)	1.67 (0.11, 48.06)	0.36 (0.01, 15.61)	ONT22_5mgSC	0.97 (0.40, 2.39)	0.83 (0.32, 2.04)	0.46 (0.13, 1.30)	0.35 (0.07, 1.54)	0.96 (0.20, 4.05)	0.45 (0.09, 1.88)	**0.12 (0.03, 0.41)**	0.29 (0.06, 1.31)	**0.19 (0.05, 0.61)**	0.57 (0.10, 3.94)
ONT225mgSC	0.36 (0.04, 2.15)	0.37 (0.10, 1.16)	0.43 (0.08, 1.91)	0.38 (0.07, 1.77)	0.49 (0.09, 2.39)	1.27 (0.17, 11.26)	0.35 (0.01, 14.61)	0.44 (0.01, 18.73)	1.69 (0.13, 48.25)	0.36 (0.01, 15.59)	1.03 (0.42, 2.52)	ONT225mgSC	0.86 (0.33, 2.11)	0.47 (0.14, 1.35)	0.37 (0.07, 1.58)	0.99 (0.22, 4.05)	0.46 (0.10, 2.00)	**0.13 (0.03, 0.43)**	0.30 (0.06, 1.28)	**0.20 (0.06, 0.64)**	0.60 (0.11, 4.05)
ONT75mgSC	0.43 (0.06, 2.46)	0.43 (0.11, 1.48)	0.50 (0.09, 2.41)	0.44 (0.08, 2.19)	0.57 (0.10, 2.97)	1.49 (0.19, 14.25)	0.42 (0.02, 16.97)	0.53 (0.01, 21.76)	1.95 (0.15, 56.47)	0.44 (0.01, 17.07)	1.21 (0.49, 3.14)	1.17 (0.47, 3.02)	ONT75mgSC	0.56 (0.16, 1.66)	0.43 (0.09, 1.91)	1.16 (0.25, 4.93)	0.54 (0.12, 2.38)	**0.15 (0.04, 0.52)**	0.36 (0.07, 1.63)	**0.24 (0.06, 0.77)**	0.69 (0.12, 4.78)
PBO	0.81 (0.15, 2.89)	0.78 (0.47, 1.35)	0.91 (0.30, 2.74)	0.80 (0.26, 2.58)	1.05 (0.30, 3.31)	2.70 (0.51, 19.28)	0.75 (0.03, 26.87)	0.94 (0.03, 32.24)	3.62 (0.34, 85.58)	0.81 (0.01, 25.99)	2.17 (0.77, 7.78)	2.12 (0.74, 7.07)	1.78 (0.60, 6.23)	PBO	0.78 (0.28, 2.23)	2.08 (0.83, 5.83)	0.98 (0.37, 2.72)	**0.27 (0.15, 0.48)**	0.67 (0.22, 1.74)	**0.43 (0.26, 0.68)**	1.26 (0.36, 6.01)
PF10mgSC	1.02 (0.14, 5.38)	1.01 (0.32, 3.24)	1.17 (0.26, 5.12)	1.05 (0.22, 4.74)	1.32 (0.26, 6.25)	3.44 (0.49, 33.36)	0.97 (0.03, 38.51)	1.16 (0.03, 49.71)	4.64 (0.37, 123.09)	1.05 (0.02, 38.18)	2.82 (0.65, 13.82)	2.69 (0.63, 13.57)	2.31 (0.52, 11.35)	1.28 (0.45, 3.62)	PF10mgSC	2.69 (1.00, 7.69)	1.24 (0.44, 3.65)	0.35 (0.10, 1.12)	0.84 (0.19, 3.56)	0.54 (0.17, 1.67)	1.63 (0.31, 10.92)
PF200mgSC	0.38 (0.05, 1.82)	0.38 (0.12, 1.06)	0.43 (0.11, 1.74)	0.39 (0.09, 1.60)	0.49 (0.11, 2.10)	1.27 (0.18, 11.47)	0.36 (0.01, 13.68)	0.43 (0.01, 17.87)	1.69 (0.14, 44.77)	0.38 (0.01, 12.96)	1.04 (0.25, 4.96)	1.01 (0.25, 4.64)	0.86 (0.20, 3.93)	0.48 (0.17, 1.20)	0.37 (0.13, 1.00)	PF200mgSC	0.46 (0.19, 1.21)	**0.13 (0.04, 0.39)**	0.32 (0.08, 1.18)	**0.20 (0.07, 0.57)**	0.62 (0.12, 4.03)
PF50mgSC	0.81 (0.11, 4.36)	0.80 (0.26, 2.46)	0.93 (0.22, 4.06)	0.83 (0.19, 3.58)	1.05 (0.22, 4.71)	2.76 (0.41, 26.62)	0.80 (0.03, 30.24)	0.94 (0.02, 39.48)	3.73 (0.30, 104.31)	0.84 (0.01, 32.71)	2.24 (0.53, 11.09)	2.16 (0.50, 10.24)	1.85 (0.42, 8.57)	1.02 (0.37, 2.73)	0.80 (0.27, 2.27)	2.16 (0.82, 5.40)	PF50mgSC	**0.28 (0.09, 0.85)**	0.68 (0.15, 2.80)	0.43 (0.15, 1.31)	1.29 (0.25, 8.14)
RIS1200mgIV	2.94 (0.47, 11.94)	**2.88 (1.34, 6.19)**	3.33 (0.98, 11.72)	2.94 (0.83, 10.65)	**3.84 (1.04, 14.10)**	**9.91 (1.72, 76.74)**	2.76 (0.11, 101.44)	3.46 (0.09, 126.92)	**13.18 (1.17, 360.23)**	2.94 (0.05, 93.36)	**8.07 (2.45, 31.62)**	**7.77 (2.35, 29.57)**	**6.66 (1.93, 27.50)**	**3.66 (2.10, 6.62)**	2.86 (0.90, 9.53)	**7.64 (2.57, 23.90)**	**3.62 (1.17, 11.74)**	RIS1200mgIV	2.45 (0.72, 7.27)	1.57 (0.86, 2.86)	**4.71 (1.12, 24.49)**
RIS200mgIV	1.22 (0.17, 7.11)	1.19 (0.38, 4.51)	1.40 (0.32, 6.32)	1.23 (0.26, 5.78)	1.60 (0.33, 7.55)	4.05 (0.59, 41.74)	1.14 (0.04, 44.17)	1.43 (0.04, 57.29)	5.56 (0.43, 154.70)	1.21 (0.02, 43.75)	3.42 (0.76, 16.19)	3.30 (0.78, 15.86)	2.78 (0.61, 14.07)	1.50 (0.57, 4.59)	1.18 (0.28, 5.36)	3.16 (0.85, 13.30)	1.48 (0.36, 6.60)	0.41 (0.14, 1.39)	RIS200mgIV	0.65 (0.23, 2.07)	1.94 (0.39, 13.25)
RIS600mgIV	1.91 (0.32, 7.37)	1.83 (0.92, 3.78)	2.12 (0.66, 7.24)	1.90 (0.54, 6.44)	2.47 (0.66, 8.61)	**6.29 (1.14, 48.26)**	1.78 (0.07, 62.12)	2.23 (0.06, 77.54)	8.51 (0.77, 211.07)	1.92 (0.03, 63.81)	**5.16 (1.65, 19.59)**	**4.99 (1.55, 17.94)**	**4.23 (1.30, 16.08)**	**2.34 (1.47, 3.84)**	1.85 (0.60, 5.74)	**4.91 (1.76, 14.68)**	2.31 (0.77, 6.87)	0.64 (0.35, 1.17)	1.55 (0.48, 4.30)	RIS600mgIV	2.97 (0.77, 15.34)
SEC10mg_kgIV	0.60 (0.06, 3.87)	0.62 (0.12, 2.52)	0.72 (0.11, 3.95)	0.62 (0.09, 3.59)	0.81 (0.11, 4.69)	2.08 (0.22, 21.13)	0.58 (0.02, 22.98)	0.68 (0.01, 32.58)	2.85 (0.16, 83.52)	0.59 (0.01, 22.93)	1.74 (0.25, 9.55)	1.68 (0.25, 9.45)	1.45 (0.21, 8.35)	0.79 (0.17, 2.77)	0.61 (0.09, 3.23)	1.63 (0.25, 8.30)	0.77 (0.12, 3.99)	**0.21 (0.04, 0.89)**	0.51 (0.08, 2.54)	0.34 (0.07, 1.30)	SEC10mg_kgIV

	MIR600mgIV	NAT300mgIV	NAT3mg_kgIV	NAT3mg_kgIVx2	NAT6mg_kgIVx2	NNC2mg_kgSC	ONE10mgSC	ONE25mgSC	ONE35mgSC	ONE50mgSC	ONT22_5mgSC	ONT225mgSC	ONT75mgSC	PBO	PF10mgSC	PF200mgSC	PF50mgSC	RIS1200mgIV	RIS200mgIV	RIS600mgIV	SEC10mg_kgIV
TES400mg200mgSC	**0.07 (0.00, 0.75)**	**0.07 (0.00, 0.51)**	**0.08 (0.00, 0.75)**	**0.07 (0.00, 0.71)**	**0.09 (0.00, 0.92)**	0.24 (0.01, 3.96)	0.06 (0.00, 3.65)	0.07 (0.00, 6.43)	0.31 (0.01, 13.98)	0.07 (0.00, 4.12)	0.19 (0.01, 2.00)	0.19 (0.01, 1.86)	0.16 (0.01, 1.60)	**0.09 (0.00, 0.60)**	**0.07 (0.00, 0.64)**	0.18 (0.01, 1.65)	**0.09 (0.00, 0.77)**	**0.02 (0.00, 0.18)**	**0.06 (0.00, 0.50)**	**0.04 (0.00, 0.27)**	0.11 (0.00, 1.34)
TOF15mgPO_BID	5.25 (0.41, 131.64)	5.14 (0.68, 116.94)	6.03 (0.63, 151.22)	5.45 (0.55, 127.74)	7.13 (0.69, 163.33)	**18.72 (1.28, 708.17)**	5.34 (0.12, 539.79)	6.70 (0.08, 670.95)	**25.68 (1.16, 2177.09)**	5.24 (0.07, 571.36)	**14.89 (1.58, 367.63)**	**14.44 (1.51, 351.29)**	**12.21 (1.33, 316.68)**	6.65 (0.98, 140.24)	5.21 (0.57, 135.13)	**14.09 (1.59, 370.68)**	6.61 (0.73, 166.51)	1.80 (0.24, 38.72)	4.29 (0.47, 103.16)	2.81 (0.39, 59.13)	8.91 (0.83, 247.65)
TOF1mgPO_BID	1.10 (0.11, 7.16)	1.08 (0.25, 4.71)	1.27 (0.22, 7.55)	1.10 (0.18, 6.71)	1.42 (0.24, 8.75)	3.80 (0.42, 44.75)	1.11 (0.03, 48.47)	1.29 (0.03, 56.65)	5.01 (0.33, 150.97)	1.08 (0.01, 41.79)	3.06 (0.53, 20.41)	2.91 (0.51, 18.58)	2.47 (0.41, 16.13)	1.37 (0.34, 5.42)	1.07 (0.19, 6.54)	2.90 (0.52, 16.45)	1.37 (0.24, 7.65)	0.37 (0.08, 1.67)	0.90 (0.15, 4.97)	0.59 (0.13, 2.47)	1.75 (0.27, 14.19)
TOF5mgPO_BID	1.01 (0.12, 7.78)	1.02 (0.23, 4.70)	1.19 (0.21, 6.85)	1.06 (0.17, 6.47)	1.35 (0.23, 8.09)	3.62 (0.40, 38.96)	1.03 (0.03, 43.05)	1.23 (0.02, 49.48)	4.83 (0.32, 137.92)	1.02 (0.01, 39.52)	2.85 (0.50, 18.43)	2.74 (0.48, 17.91)	2.35 (0.40, 15.76)	1.29 (0.34, 5.32)	1.01 (0.18, 5.74)	2.71 (0.53, 15.67)	1.26 (0.23, 6.95)	0.35 (0.08, 1.62)	0.86 (0.15, 4.56)	0.55 (0.13, 2.38)	1.69 (0.26, 12.76)
UPA12mgPO_BID	**0.13 (0.01, 0.97)**	**0.14 (0.02, 0.59)**	**0.16 (0.02, 0.94)**	**0.14 (0.02, 0.84)**	0.18 (0.02, 1.07)	0.46 (0.04, 5.04)	0.13 (0.00, 5.07)	0.16 (0.00, 7.33)	0.60 (0.03, 18.22)	0.13 (0.00, 5.54)	0.38 (0.04, 2.51)	0.37 (0.04, 2.32)	0.31 (0.03, 1.99)	**0.17 (0.02, 0.68)**	**0.14 (0.01, 0.74)**	0.36 (0.04, 1.88)	**0.17 (0.02, 0.92)**	**0.05 (0.01, 0.22)**	**0.11 (0.01, 0.61)**	**0.07 (0.01, 0.33)**	0.22 (0.02, 1.73)
UPA24mgPO	0.18 (0.02, 1.40)	**0.20 (0.03, 0.93)**	0.23 (0.02, 1.39)	0.20 (0.02, 1.25)	0.26 (0.03, 1.70)	0.66 (0.05, 7.53)	0.18 (0.00, 8.22)	0.22 (0.00, 10.92)	0.88 (0.04, 27.26)	0.19 (0.00, 7.84)	0.54 (0.06, 3.87)	0.53 (0.06, 3.51)	0.45 (0.05, 3.01)	0.25 (0.04, 1.12)	0.20 (0.02, 1.18)	0.53 (0.06, 2.95)	0.25 (0.03, 1.47)	**0.07 (0.01, 0.34)**	**0.16 (0.02, 0.96)**	**0.11 (0.01, 0.49)**	0.32 (0.03, 2.67)
UPA24mgPO_BID	0.48 (0.04, 5.19)	0.50 (0.06, 3.61)	0.57 (0.05, 4.96)	0.50 (0.05, 4.77)	0.64 (0.06, 5.82)	1.64 (0.13, 25.06)	0.48 (0.01, 22.65)	0.59 (0.01, 29.82)	2.27 (0.10, 79.30)	0.49 (0.00, 22.41)	1.38 (0.14, 13.41)	1.36 (0.13, 12.42)	1.16 (0.12, 11.03)	0.63 (0.08, 4.28)	0.50 (0.05, 4.35)	1.30 (0.14, 11.01)	0.62 (0.06, 5.11)	0.17 (0.02, 1.25)	0.41 (0.04, 3.47)	0.27 (0.03, 1.87)	0.81 (0.08, 9.14)
UPA3mgPO_BID	0.30 (0.03, 2.55)	0.31 (0.04, 1.83)	0.37 (0.04, 2.64)	0.32 (0.03, 2.24)	0.42 (0.04, 2.83)	1.08 (0.08, 15.04)	0.29 (0.01, 12.92)	0.34 (0.01, 17.87)	1.46 (0.07, 43.31)	0.30 (0.00, 13.39)	0.90 (0.09, 6.81)	0.86 (0.09, 6.34)	0.74 (0.07, 5.31)	0.40 (0.05, 2.11)	0.32 (0.03, 2.10)	0.84 (0.09, 5.45)	0.40 (0.04, 2.50)	**0.11 (0.01, 0.66)**	0.26 (0.03, 1.73)	**0.17 (0.02, 0.94)**	0.52 (0.05, 4.56)
UPA45mgPO	0.83 (0.13, 3.37)	0.80 (0.38, 1.71)	0.94 (0.27, 3.18)	0.83 (0.23, 2.97)	1.08 (0.28, 3.80)	2.79 (0.50, 20.85)	0.77 (0.03, 26.48)	0.96 (0.03, 34.86)	3.71 (0.33, 87.23)	0.83 (0.01, 27.03)	2.25 (0.69, 8.98)	2.19 (0.68, 8.14)	1.85 (0.54, 7.16)	1.03 (0.60, 1.73)	0.80 (0.25, 2.67)	2.17 (0.75, 6.80)	1.01 (0.33, 3.27)	**0.28 (0.13, 0.60)**	0.68 (0.20, 2.03)	**0.44 (0.21, 0.87)**	1.31 (0.32, 6.92)
UPA6mgPO_BID	0.81 (0.05, 11.39)	0.84 (0.08, 9.68)	0.97 (0.08, 14.50)	0.84 (0.07, 12.57)	1.10 (0.09, 15.04)	2.97 (0.18, 54.69)	0.78 (0.02, 53.73)	0.98 (0.01, 64.93)	4.14 (0.13, 176.30)	0.81 (0.01, 52.50)	2.28 (0.20, 36.10)	2.26 (0.19, 33.00)	1.95 (0.16, 29.84)	1.06 (0.11, 11.49)	0.84 (0.07, 11.48)	2.24 (0.19, 27.91)	1.07 (0.09, 13.06)	0.29 (0.03, 3.39)	0.69 (0.06, 8.17)	0.45 (0.05, 5.27)	1.39 (0.11, 22.19)
UST130mgIV	1.01 (0.16, 4.18)	0.98 (0.45, 2.21)	1.13 (0.33, 4.06)	1.00 (0.28, 3.73)	1.32 (0.34, 4.71)	3.36 (0.58, 26.44)	0.95 (0.04, 32.23)	1.19 (0.03, 42.43)	4.48 (0.39, 111.29)	0.99 (0.02, 33.45)	2.70 (0.82, 11.16)	2.62 (0.80, 10.20)	2.24 (0.64, 9.07)	1.25 (0.69, 2.32)	0.97 (0.29, 3.26)	2.58 (0.86, 8.73)	1.21 (0.39, 3.98)	**0.34 (0.15, 0.79)**	0.82 (0.25, 2.56)	0.53 (0.25, 1.14)	1.59 (0.39, 8.38)
UST1mg_kgIV	1.51 (0.21, 8.18)	1.46 (0.48, 4.88)	1.70 (0.40, 8.63)	1.48 (0.33, 7.20)	1.94 (0.40, 9.60)	5.00 (0.76, 49.62)	1.42 (0.05, 53.68)	1.75 (0.04, 67.70)	6.87 (0.56, 194.24)	1.53 (0.02, 53.43)	4.06 (0.99, 20.54)	3.96 (0.95, 19.01)	3.35 (0.79, 17.60)	1.85 (0.71, 5.61)	1.47 (0.35, 6.54)	**3.94 (1.07, 16.02)**	1.86 (0.47, 7.88)	0.51 (0.16, 1.68)	1.22 (0.30, 5.21)	0.79 (0.27, 2.49)	2.39 (0.49, 16.22)
UST3mg_kgIV	1.14 (0.16, 5.44)	1.10 (0.39, 3.38)	1.29 (0.32, 5.56)	1.13 (0.26, 5.18)	1.48 (0.32, 6.52)	3.74 (0.59, 37.12)	1.09 (0.04, 42.69)	1.29 (0.03, 51.97)	5.18 (0.39, 147.93)	1.13 (0.02, 39.94)	3.08 (0.79, 14.69)	3.00 (0.76, 14.32)	2.53 (0.62, 12.75)	1.39 (0.57, 3.73)	1.10 (0.28, 4.50)	2.89 (0.81, 12.07)	1.38 (0.37, 5.67)	0.38 (0.13, 1.18)	0.93 (0.23, 3.66)	0.60 (0.21, 1.78)	1.81 (0.38, 11.49)
UST4_5mg_kgIV	0.30 (0.01, 5.08)	0.30 (0.01, 4.23)	0.34 (0.01, 6.02)	0.31 (0.01, 5.41)	0.40 (0.01, 7.08)	1.06 (0.02, 28.66)	0.28 (0.00, 19.02)	0.34 (0.00, 28.34)	1.41 (0.03, 72.88)	0.30 (0.00, 21.16)	0.87 (0.02, 14.09)	0.84 (0.03, 14.23)	0.70 (0.02, 11.97)	0.38 (0.01, 5.10)	0.30 (0.01, 4.83)	0.79 (0.02, 12.28)	0.37 (0.01, 6.26)	0.10 (0.00, 1.46)	0.25 (0.01, 4.27)	0.16 (0.00, 2.30)	0.49 (0.01, 10.45)
UST6mg_kg90mgIV_SC	0.79 (0.08, 5.80)	0.78 (0.16, 3.80)	0.91 (0.15, 5.67)	0.80 (0.13, 5.15)	1.03 (0.15, 6.76)	2.69 (0.30, 30.46)	0.75 (0.03, 34.01)	0.90 (0.02, 42.82)	3.54 (0.22, 118.17)	0.79 (0.01, 36.91)	2.19 (0.35, 14.34)	2.15 (0.35, 13.81)	1.80 (0.27, 11.75)	0.99 (0.22, 4.37)	0.78 (0.12, 4.87)	2.06 (0.36, 12.59)	0.96 (0.16, 5.67)	0.27 (0.06, 1.35)	0.65 (0.11, 3.89)	0.42 (0.09, 2.09)	1.28 (0.17, 10.47)
UST6mg_kgIV	0.94 (0.16, 3.77)	0.92 (0.44, 1.95)	1.07 (0.32, 3.68)	0.94 (0.27, 3.40)	1.22 (0.33, 4.41)	3.20 (0.56, 24.26)	0.87 (0.04, 31.00)	1.09 (0.03, 41.62)	4.21 (0.39, 108.04)	0.94 (0.02, 35.99)	2.53 (0.83, 9.90)	2.47 (0.79, 9.43)	2.10 (0.63, 8.11)	1.18 (0.70, 1.99)	0.92 (0.29, 2.88)	2.44 (0.87, 7.82)	1.14 (0.39, 3.69)	**0.32 (0.15, 0.68)**	0.77 (0.23, 2.35)	0.50 (0.25, 1.04)	1.49 (0.38, 7.77)
UST90mgSC	1.59 (0.07, 51.17)	1.63 (0.10, 43.75)	1.98 (0.09, 57.11)	1.69 (0.09, 50.68)	2.23 (0.11, 66.35)	5.87 (0.29, 283.35)	1.78 (0.02, 152.30)	1.91 (0.03, 252.95)	8.09 (0.23, 639.70)	1.78 (0.01, 191.45)	4.77 (0.25, 145.53)	4.40 (0.23, 151.24)	3.83 (0.20, 139.67)	2.05 (0.13, 53.38)	1.60 (0.09, 47.71)	4.41 (0.25, 136.41)	2.04 (0.11, 60.96)	0.56 (0.03, 14.87)	1.35 (0.07, 44.60)	0.87 (0.05, 21.38)	2.86 (0.14, 104.38)
VED0_5mg_kgIV	1.44 (0.19, 8.06)	1.40 (0.46, 5.14)	1.60 (0.38, 8.33)	1.45 (0.30, 7.08)	1.88 (0.40, 9.43)	4.97 (0.65, 47.30)	1.36 (0.05, 55.16)	1.73 (0.05, 70.59)	6.81 (0.49, 173.82)	1.49 (0.02, 51.29)	3.98 (0.91, 21.70)	3.78 (0.90, 20.69)	3.26 (0.69, 18.01)	1.79 (0.66, 5.98)	1.40 (0.32, 6.68)	3.75 (0.95, 16.86)	1.78 (0.43, 8.30)	0.49 (0.15, 1.78)	1.19 (0.25, 5.45)	0.77 (0.25, 2.59)	2.31 (0.43, 16.64)
VED2mg_kgIV	0.88 (0.13, 4.32)	0.87 (0.31, 2.53)	1.03 (0.25, 4.56)	0.90 (0.22, 3.85)	1.17 (0.25, 5.15)	3.03 (0.47, 27.78)	0.86 (0.03, 32.89)	1.07 (0.03, 44.48)	4.10 (0.30, 103.77)	0.91 (0.01, 30.23)	2.47 (0.62, 11.32)	2.39 (0.59, 10.94)	2.03 (0.48, 9.35)	1.12 (0.43, 2.81)	0.88 (0.21, 3.68)	2.35 (0.63, 9.02)	1.10 (0.28, 4.42)	**0.30 (0.10, 0.91)**	0.73 (0.18, 2.89)	0.48 (0.17, 1.36)	1.43 (0.30, 8.66)
VED300mgIV	0.81 (0.12, 3.19)	0.78 (0.38, 1.66)	0.91 (0.27, 3.11)	0.80 (0.23, 2.86)	1.04 (0.29, 3.73)	2.70 (0.46, 21.23)	0.75 (0.03, 28.45)	0.95 (0.03, 34.40)	3.61 (0.31, 93.06)	0.81 (0.01, 24.97)	2.19 (0.69, 8.27)	2.12 (0.67, 7.65)	1.81 (0.55, 7.03)	1.00 (0.60, 1.68)	0.79 (0.24, 2.47)	2.11 (0.74, 6.20)	0.98 (0.33, 3.01)	**0.27 (0.12, 0.60)**	0.66 (0.20, 1.96)	**0.43 (0.21, 0.86)**	1.28 (0.32, 6.60)

	TES400mg200mgSC	TOF15mgPO_BID	TOF1mgPO_BID	TOF5mgPO_BID	UPA12mgPO_BID	UPA24mgPO	UPA24mgPO_BID	UPA3mgPO_BID	UPA45mgPO	UPA6mgPO_BID	UST130mgIV	UST1mg_kgIV	UST3mg_kgIV	UST4_5mg_kgIV	UST6mg_kg90mgIV_SC	UST6mg_kgIV	UST90mgSC	VED0_5mg_kgIV	VED2mg_kgIV	VED300mgIV
ABA10mg_kgIV	**9.97 (1.31, 231.53)**	0.14 (0.01, 1.06)	0.65 (0.14, 3.07)	0.69 (0.15, 3.12)	**5.21 (1.13, 38.72)**	3.63 (0.70, 28.67)	1.40 (0.19, 11.57)	2.24 (0.37, 18.92)	0.88 (0.36, 2.11)	0.85 (0.08, 9.15)	0.72 (0.28, 1.84)	0.48 (0.13, 1.57)	0.64 (0.19, 2.04)	2.34 (0.16, 82.16)	0.92 (0.17, 4.61)	0.77 (0.32, 1.85)	0.43 (0.02, 7.84)	0.50 (0.13, 1.72)	0.80 (0.26, 2.54)	0.90 (0.38, 2.15)
ABA30mg_kgIV	**10.70 (1.34, 255.91)**	0.15 (0.01, 1.19)	0.68 (0.14, 3.41)	0.73 (0.15, 3.46)	**5.58 (1.11, 42.59)**	3.79 (0.68, 30.50)	1.51 (0.19, 12.81)	2.38 (0.36, 18.73)	0.91 (0.36, 2.44)	0.88 (0.07, 9.13)	0.76 (0.27, 2.03)	0.51 (0.13, 1.75)	0.67 (0.19, 2.27)	2.45 (0.16, 89.44)	0.97 (0.18, 5.07)	0.81 (0.31, 2.10)	0.45 (0.02, 7.41)	0.52 (0.13, 1.91)	0.84 (0.25, 3.00)	0.94 (0.37, 2.45)
ABA3mg_kgIV	**11.31 (1.43, 265.55)**	0.16 (0.01, 1.21)	0.74 (0.16, 3.49)	0.78 (0.17, 3.58)	**6.01 (1.24, 44.35)**	4.11 (0.79, 31.03)	1.63 (0.20, 13.45)	2.57 (0.40, 20.70)	0.99 (0.40, 2.43)	0.96 (0.08, 9.85)	0.82 (0.32, 2.09)	0.55 (0.15, 1.80)	0.72 (0.22, 2.31)	2.70 (0.18, 93.91)	1.03 (0.19, 5.40)	0.87 (0.36, 2.08)	0.48 (0.02, 8.69)	0.57 (0.14, 2.00)	0.91 (0.29, 2.94)	1.01 (0.42, 2.43)
ADA160mg80mg40mgSC	9.10 (0.70, 274.16)	0.11 (0.00, 1.47)	0.58 (0.06, 4.76)	0.62 (0.07, 5.24)	4.70 (0.56, 59.09)	3.20 (0.35, 41.92)	1.27 (0.11, 17.69)	1.98 (0.21, 26.08)	0.76 (0.14, 4.33)	0.73 (0.05, 12.20)	0.63 (0.11, 3.72)	0.42 (0.06, 2.91)	0.56 (0.09, 3.70)	2.03 (0.09, 93.23)	0.79 (0.42, 1.54)	0.67 (0.12, 3.67)	0.37 (0.01, 7.79)	0.43 (0.06, 2.88)	0.70 (0.11, 4.69)	0.79 (0.15, 4.33)
ADA160mg80mg60mgSC	4.50 (0.11, 215.98)	0.05 (0.00, 1.69)	0.29 (0.01, 5.15)	0.31 (0.01, 5.78)	2.46 (0.06, 52.71)	1.67 (0.04, 37.80)	0.66 (0.01, 16.01)	1.05 (0.02, 24.10)	0.40 (0.01, 5.61)	0.38 (0.01, 10.78)	0.32 (0.01, 4.38)	0.21 (0.01, 3.43)	0.28 (0.01, 4.33)	1.05 (0.02, 69.41)	0.41 (0.01, 7.80)	0.34 (0.01, 4.65)	0.18 (0.00, 8.24)	0.22 (0.01, 3.82)	0.35 (0.01, 5.54)	0.40 (0.01, 5.82)
ADA160mg80mgSC	**20.12 (2.41, 448.47)**	0.27 (0.01, 2.33)	1.28 (0.24, 6.92)	1.37 (0.25, 7.41)	**10.22 (2.00, 87.67)**	**7.18 (1.25, 64.11)**	2.91 (0.35, 27.24)	4.49 (0.65, 39.13)	1.70 (0.62, 5.33)	1.69 (0.13, 19.26)	1.41 (0.50, 4.43)	0.95 (0.23, 3.84)	1.27 (0.33, 4.65)	4.67 (0.29, 179.68)	1.80 (0.31, 10.67)	1.49 (0.54, 4.53)	0.84 (0.03, 13.86)	0.98 (0.23, 4.09)	1.57 (0.42, 6.20)	1.75 (0.61, 5.31)
ADA40mg20mgSC	**67.63 (3.50, 4855.53)**	0.76 (0.02, 33.49)	3.79 (0.33, 140.33)	3.96 (0.42, 137.75)	**30.39 (2.84, 1498.14)**	**21.43 (1.82, 1109.41)**	8.20 (0.55, 424.06)	13.43 (0.94, 646.73)	5.13 (0.67, 140.29)	4.82 (0.21, 251.19)	4.13 (0.55, 125.32)	2.86 (0.28, 84.17)	3.76 (0.43, 115.02)	14.79 (0.56, 1495.47)	5.62 (0.45, 178.98)	4.37 (0.57, 126.43)	2.56 (0.06, 164.94)	2.83 (0.27, 97.85)	4.69 (0.55, 146.76)	5.22 (0.68, 144.08)
ADA80mg40mgSC	**52.75 (4.50, 1907.68)**	0.66 (0.02, 11.97)	3.25 (0.44, 37.60)	3.41 (0.47, 37.06)	**26.62 (3.29, 449.72)**	**18.46 (2.26, 332.43)**	7.17 (0.68, 122.37)	**11.34 (1.33, 190.27)**	4.26 (0.93, 36.68)	4.25 (0.28, 91.74)	3.51 (0.77, 32.17)	2.40 (0.39, 23.35)	3.15 (0.55, 30.39)	12.33 (0.63, 690.49)	4.48 (0.56, 56.56)	3.71 (0.83, 33.68)	2.13 (0.07, 58.38)	2.47 (0.39, 25.43)	3.98 (0.71, 39.14)	4.26 (0.95, 36.04)
AMI0_4mgPO	1.44 (0.04, 51.30)	**0.02 (0.00, 0.33)**	0.09 (0.00, 1.08)	0.10 (0.00, 1.14)	0.73 (0.02, 14.06)	0.51 (0.02, 10.28)	0.19 (0.01, 4.38)	0.30 (0.01, 6.64)	**0.13 (0.01, 0.98)**	0.11 (0.00, 3.01)	**0.10 (0.00, 0.85)**	**0.07 (0.00, 0.61)**	**0.09 (0.00, 0.77)**	0.32 (0.01, 18.18)	0.12 (0.00, 1.60)	**0.11 (0.00, 0.86)**	0.05 (0.00, 1.56)	**0.07 (0.00, 0.65)**	**0.11 (0.00, 0.98)**	0.13 (0.01, 1.01)
AND150mgSC1_1	10.28 (0.70, 240.93)	0.12 (0.00, 1.54)	0.65 (0.07, 4.67)	0.67 (0.08, 5.54)	5.09 (0.57, 55.06)	3.52 (0.39, 43.47)	1.35 (0.12, 16.57)	2.18 (0.19, 26.41)	0.86 (0.15, 3.80)	0.79 (0.04, 12.41)	0.72 (0.12, 3.30)	0.46 (0.06, 2.66)	0.63 (0.09, 3.31)	2.27 (0.09, 83.23)	0.89 (0.09, 7.52)	0.77 (0.13, 3.26)	0.39 (0.01, 8.98)	0.48 (0.06, 2.91)	0.79 (0.11, 4.51)	0.89 (0.15, 4.05)
AND150mgSC2_2	**96.77 (3.66, 7621.55)**	1.12 (0.02, 68.59)	5.35 (0.38, 240.58)	5.62 (0.38, 227.24)	**44.63 (2.65, 2139.48)**	**32.04 (1.89, 1436.71)**	12.12 (0.56, 671.55)	19.03 (0.98, 1042.62)	7.25 (0.67, 243.51)	7.02 (0.27, 459.54)	5.99 (0.54, 197.25)	4.06 (0.28, 154.32)	5.15 (0.40, 190.72)	21.22 (0.53, 2604.32)	7.78 (0.39, 418.89)	6.27 (0.56, 217.83)	3.74 (0.08, 230.13)	4.14 (0.33, 146.18)	6.61 (0.51, 250.06)	7.31 (0.66, 258.40)
AND300mgSC	7.57 (0.55, 162.16)	0.09 (0.00, 1.04)	0.46 (0.05, 3.18)	0.48 (0.06, 3.50)	3.71 (0.42, 39.35)	2.57 (0.28, 27.60)	1.03 (0.09, 11.43)	1.61 (0.15, 16.84)	0.61 (0.12, 2.69)	0.58 (0.03, 8.75)	0.51 (0.09, 2.26)	0.33 (0.05, 1.85)	0.45 (0.06, 2.37)	1.72 (0.07, 59.96)	0.66 (0.06, 5.42)	0.55 (0.10, 2.20)	0.29 (0.01, 6.34)	0.35 (0.05, 1.94)	0.57 (0.09, 3.02)	0.64 (0.11, 2.80)
API100mgPR	**24.61 (2.26, 667.71)**	0.31 (0.01, 3.39)	1.50 (0.23, 10.87)	1.61 (0.25, 11.15)	**12.19 (1.77, 129.40)**	**8.41 (1.24, 92.26)**	3.28 (0.32, 38.41)	5.21 (0.70, 58.63)	2.02 (0.54, 9.47)	1.95 (0.13, 26.94)	1.65 (0.43, 7.63)	1.12 (0.21, 5.98)	1.48 (0.32, 7.70)	5.67 (0.31, 199.01)	2.13 (0.30, 14.96)	1.73 (0.46, 8.17)	1.00 (0.03, 19.62)	1.16 (0.20, 6.64)	1.88 (0.40, 9.43)	2.09 (0.55, 8.86)
API50mgPO	**15.84 (1.54, 399.76)**	0.20 (0.01, 2.00)	0.99 (0.16, 5.92)	1.07 (0.18, 6.06)	**7.89 (1.32, 77.43)**	5.49 (0.88, 54.53)	2.13 (0.24, 22.47)	3.46 (0.48, 35.11)	1.32 (0.38, 4.91)	1.26 (0.09, 17.40)	1.08 (0.32, 4.04)	0.73 (0.16, 3.31)	0.96 (0.22, 4.22)	3.62 (0.21, 135.33)	1.38 (0.22, 8.59)	1.15 (0.33, 4.25)	0.65 (0.02, 12.24)	0.75 (0.14, 3.89)	1.23 (0.28, 5.16)	1.33 (0.41, 4.88)
BRI400mgIV	**24.78 (1.76, 771.36)**	0.31 (0.01, 4.98)	1.49 (0.17, 17.71)	1.60 (0.19, 18.28)	**12.07 (1.36, 191.47)**	8.48 (0.85, 134.63)	3.24 (0.26, 52.81)	5.23 (0.47, 79.38)	1.94 (0.33, 15.83)	2.02 (0.10, 37.38)	1.61 (0.26, 13.53)	1.07 (0.16, 10.60)	1.42 (0.22, 13.74)	5.78 (0.22, 264.73)	2.09 (0.20, 26.32)	1.70 (0.29, 14.49)	0.92 (0.03, 26.57)	1.16 (0.15, 10.89)	1.86 (0.25, 17.41)	2.02 (0.34, 16.24)
BRI700mgIV	**35.53 (2.72, 891.66)**	0.44 (0.02, 4.93)	2.22 (0.28, 15.99)	2.31 (0.31, 16.24)	**18.07 (2.29, 208.48)**	**12.57 (1.42, 148.17)**	4.71 (0.43, 61.62)	7.79 (0.81, 94.87)	2.91 (0.60, 14.24)	2.83 (0.17, 41.36)	2.40 (0.48, 12.03)	1.60 (0.26, 9.35)	2.13 (0.37, 12.12)	7.91 (0.40, 345.10)	3.09 (0.35, 23.95)	2.58 (0.52, 12.01)	1.38 (0.04, 30.55)	1.66 (0.25, 9.80)	2.71 (0.45, 15.38)	3.01 (0.61, 14.32)
BRO210mgIV	6.92 (0.40, 231.76)	0.08 (0.00, 1.31)	0.45 (0.04, 4.24)	0.45 (0.04, 4.48)	3.69 (0.27, 49.05)	2.50 (0.17, 35.15)	0.95 (0.05, 15.59)	1.59 (0.08, 22.64)	0.58 (0.07, 3.79)	0.56 (0.03, 9.52)	0.49 (0.05, 3.26)	0.32 (0.03, 2.55)	0.43 (0.04, 3.10)	1.62 (0.05, 82.15)	0.60 (0.05, 7.00)	0.52 (0.06, 3.45)	0.27 (0.00, 7.18)	0.34 (0.03, 2.64)	0.55 (0.06, 4.08)	0.61 (0.07, 3.95)
BRO350mgIV	2.45 (0.17, 65.54)	**0.03 (0.00, 0.38)**	0.16 (0.01, 1.26)	0.16 (0.02, 1.26)	1.28 (0.12, 13.76)	0.88 (0.07, 9.82)	0.35 (0.02, 4.06)	0.57 (0.04, 6.18)	0.21 (0.03, 1.01)	0.20 (0.01, 3.01)	**0.17 (0.02, 0.86)**	**0.12 (0.01, 0.67)**	**0.15 (0.02, 0.87)**	0.56 (0.02, 22.56)	0.22 (0.02, 1.89)	**0.18 (0.03, 0.89)**	0.10 (0.00, 2.35)	**0.12 (0.01, 0.72)**	0.20 (0.02, 1.14)	0.22 (0.03, 1.05)
BRO700mgIV	2.16 (0.15, 58.25)	**0.03 (0.00, 0.34)**	0.14 (0.01, 1.00)	0.14 (0.01, 1.04)	1.14 (0.10, 12.02)	0.77 (0.06, 8.88)	0.31 (0.02, 3.59)	0.49 (0.03, 5.23)	**0.19 (0.03, 0.82)**	0.18 (0.01, 2.47)	**0.15 (0.02, 0.71)**	**0.10 (0.01, 0.61)**	**0.13 (0.02, 0.74)**	0.50 (0.02, 20.83)	0.19 (0.02, 1.67)	**0.16 (0.02, 0.72)**	0.09 (0.00, 2.17)	**0.11 (0.01, 0.60)**	**0.17 (0.02, 0.91)**	**0.19 (0.03, 0.87)**
CDP10mg_kgIV	**14.64 (1.87, 329.96)**	0.20 (0.01, 1.49)	0.96 (0.20, 4.58)	1.00 (0.21, 4.71)	**7.52 (1.54, 61.19)**	5.22 (0.98, 43.72)	2.06 (0.27, 17.43)	3.25 (0.54, 27.53)	1.26 (0.52, 3.14)	1.22 (0.10, 13.09)	1.04 (0.41, 2.61)	0.70 (0.19, 2.25)	0.93 (0.26, 2.89)	3.33 (0.23, 122.57)	1.30 (0.25, 6.63)	1.11 (0.44, 2.63)	0.62 (0.02, 10.22)	0.71 (0.17, 2.45)	1.16 (0.35, 3.61)	1.30 (0.53, 3.07)
CER100mgSC	8.63 (0.96, 196.52)	0.11 (0.00, 1.08)	0.56 (0.10, 3.18)	0.58 (0.11, 3.14)	4.35 (0.81, 38.13)	3.01 (0.53, 28.80)	1.20 (0.14, 11.27)	1.91 (0.25, 18.63)	0.72 (0.24, 2.47)	0.70 (0.06, 8.26)	0.60 (0.19, 2.20)	0.41 (0.09, 1.73)	0.54 (0.13, 2.24)	1.97 (0.13, 95.42)	0.77 (0.12, 5.09)	0.64 (0.20, 2.20)	0.36 (0.01, 7.06)	0.42 (0.08, 1.86)	0.67 (0.17, 2.88)	0.75 (0.25, 2.51)

	TES400mg200mgSC	TOF15mgPO_BID	TOF1mgPO_BID	TOF5mgPO_BID	UPA12mgPO_BID	UPA24mgPO	UPA24mgPO_BID	UPA3mgPO_BID	UPA45mgPO	UPA6mgPO_BID	UST130mgIV	UST1mg_kgIV	UST3mg_kgIV	UST4_5mg_kgIV	UST6mg_kg90mgIV_SC	UST6mg_kgIV	UST90mgSC	VED0_5mg_kgIV	VED2mg_kgIV	VED300mgIV
CER10mgIV	19.41 (0.68, 1730.08)	0.24 (0.00, 12.17)	1.15 (0.07, 57.12)	1.28 (0.08, 58.32)	9.71 (0.46, 506.60)	6.82 (0.32, 360.49)	2.72 (0.09, 136.25)	4.22 (0.19, 244.83)	1.55 (0.12, 54.82)	1.63 (0.05, 85.07)	1.30 (0.10, 44.27)	0.88 (0.05, 35.70)	1.12 (0.07, 43.07)	4.64 (0.11, 689.44)	1.60 (0.08, 84.87)	1.36 (0.10, 48.58)	0.83 (0.01, 80.08)	0.92 (0.05, 36.14)	1.45 (0.09, 55.61)	1.58 (0.12, 56.42)
CER200mgSC	5.66 (0.69, 133.66)	**0.07 (0.00, 0.70)**	0.36 (0.07, 2.09)	0.39 (0.07, 2.09)	2.93 (0.55, 24.05)	2.01 (0.36, 17.56)	0.81 (0.09, 7.31)	1.28 (0.19, 11.10)	0.49 (0.16, 1.51)	0.46 (0.04, 5.02)	0.40 (0.13, 1.27)	0.27 (0.06, 1.06)	0.36 (0.09, 1.41)	1.28 (0.09, 48.41)	0.51 (0.08, 3.21)	0.43 (0.15, 1.30)	0.24 (0.01, 4.10)	0.28 (0.06, 1.12)	0.45 (0.12, 1.75)	0.50 (0.17, 1.50)
CER20mgIV	3.77 (0.24, 101.69)	**0.05 (0.00, 0.64)**	0.23 (0.02, 1.99)	0.24 (0.03, 2.02)	1.90 (0.16, 22.95)	1.32 (0.10, 15.78)	0.50 (0.03, 6.69)	0.81 (0.05, 10.02)	0.32 (0.04, 1.65)	0.30 (0.01, 4.76)	0.26 (0.03, 1.35)	0.18 (0.02, 1.11)	0.24 (0.02, 1.36)	0.85 (0.03, 38.93)	0.32 (0.03, 2.89)	0.28 (0.03, 1.38)	0.15 (0.00, 3.80)	0.18 (0.02, 1.17)	0.29 (0.03, 1.83)	0.33 (0.04, 1.64)
CER400mgSC	**8.32 (1.14, 184.25)**	**0.11 (0.01, 0.85)**	0.54 (0.12, 2.40)	0.58 (0.13, 2.54)	4.34 (0.97, 32.51)	2.93 (0.59, 23.55)	1.19 (0.16, 9.74)	1.87 (0.33, 14.45)	0.73 (0.33, 1.58)	0.70 (0.06, 6.91)	0.60 (0.26, 1.42)	0.40 (0.11, 1.23)	0.53 (0.17, 1.56)	1.91 (0.14, 68.81)	0.76 (0.15, 3.64)	0.64 (0.28, 1.40)	0.36 (0.01, 5.84)	0.41 (0.11, 1.32)	0.66 (0.23, 2.05)	0.74 (0.35, 1.62)
CER5mgIV	**30.92 (1.19, 2199.71)**	0.36 (0.01, 15.92)	1.85 (0.11, 55.31)	1.98 (0.12, 58.29)	16.16 (0.85, 486.23)	11.22 (0.59, 314.23)	4.18 (0.20, 127.22)	6.81 (0.34, 212.74)	2.51 (0.19, 53.78)	2.39 (0.09, 87.72)	2.07 (0.15, 45.81)	1.46 (0.07, 35.60)	1.86 (0.13, 43.42)	7.20 (0.17, 796.74)	2.54 (0.14, 75.09)	2.21 (0.16, 48.88)	1.26 (0.02, 71.73)	1.39 (0.09, 35.58)	2.32 (0.16, 54.36)	2.63 (0.18, 57.26)
ELD10mg_kgIV	7.38 (0.40, 250.38)	0.09 (0.00, 1.54)	0.46 (0.04, 4.53)	0.48 (0.04, 4.58)	3.80 (0.27, 51.58)	2.65 (0.18, 38.97)	1.03 (0.06, 15.31)	1.60 (0.10, 23.81)	0.61 (0.07, 4.34)	0.59 (0.02, 11.24)	0.50 (0.06, 3.50)	0.34 (0.03, 2.83)	0.45 (0.05, 3.62)	1.69 (0.06, 96.43)	0.62 (0.05, 7.56)	0.53 (0.07, 3.49)	0.28 (0.01, 7.98)	0.35 (0.03, 3.01)	0.56 (0.06, 4.19)	0.62 (0.08, 4.31)
ELD20mg_kgIV	5.62 (0.30, 153.84)	**0.07 (0.00, 0.99)**	0.34 (0.03, 2.88)	0.36 (0.03, 3.16)	2.83 (0.23, 36.14)	1.94 (0.15, 25.52)	0.75 (0.05, 10.32)	1.22 (0.08, 16.98)	0.47 (0.06, 2.81)	0.43 (0.02, 7.69)	0.39 (0.05, 2.33)	0.26 (0.02, 1.72)	0.34 (0.04, 2.22)	1.26 (0.05, 59.75)	0.47 (0.04, 4.90)	0.41 (0.05, 2.41)	0.21 (0.00, 6.79)	0.26 (0.03, 2.01)	0.44 (0.05, 2.93)	0.48 (0.06, 2.76)
ETA25mgSC	**34.74 (1.13, 2024.55)**	0.40 (0.01, 14.25)	2.10 (0.10, 57.35)	2.18 (0.10, 62.29)	17.52 (0.81, 633.52)	12.17 (0.55, 415.04)	4.62 (0.16, 184.50)	7.39 (0.28, 282.25)	2.76 (0.18, 60.80)	2.60 (0.09, 126.00)	2.25 (0.15, 51.51)	1.52 (0.08, 39.26)	1.99 (0.12, 50.03)	8.00 (0.18, 607.62)	2.82 (0.13, 80.99)	2.40 (0.16, 54.43)	1.33 (0.02, 67.27)	1.62 (0.08, 41.47)	2.55 (0.14, 63.46)	2.78 (0.19, 63.06)
ETR105mgSC	**11.02 (1.22, 238.94)**	0.14 (0.01, 1.26)	0.69 (0.13, 3.81)	0.73 (0.13, 3.84)	**5.52 (1.03, 47.56)**	3.86 (0.64, 33.96)	1.56 (0.18, 14.23)	2.34 (0.35, 21.89)	0.93 (0.30, 2.79)	0.88 (0.07, 10.37)	0.77 (0.24, 2.49)	0.52 (0.11, 2.06)	0.68 (0.17, 2.57)	2.54 (0.16, 87.03)	0.97 (0.16, 5.65)	0.82 (0.26, 2.46)	0.45 (0.02, 8.54)	0.54 (0.12, 2.14)	0.86 (0.22, 3.29)	0.96 (0.31, 2.82)
ETR210mgSC	**17.36 (1.79, 425.21)**	0.21 (0.01, 2.08)	1.06 (0.20, 6.26)	1.14 (0.19, 6.61)	**8.67 (1.49, 79.28)**	5.97 (0.97, 55.17)	2.35 (0.26, 23.32)	3.67 (0.56, 33.60)	1.45 (0.44, 4.71)	1.37 (0.12, 16.83)	1.18 (0.36, 4.04)	0.80 (0.16, 3.52)	1.05 (0.25, 4.21)	3.95 (0.23, 142.87)	1.52 (0.22, 9.62)	1.26 (0.38, 4.19)	0.70 (0.02, 13.36)	0.80 (0.18, 3.80)	1.34 (0.32, 5.53)	1.49 (0.45, 4.76)
FIL100mgPO	7.75 (0.96, 183.37)	**0.10 (0.00, 0.87)**	0.50 (0.10, 2.57)	0.53 (0.11, 2.67)	4.05 (0.82, 31.94)	2.79 (0.51, 22.16)	1.11 (0.14, 9.49)	1.76 (0.27, 15.01)	0.67 (0.27, 1.72)	0.64 (0.06, 7.25)	0.55 (0.21, 1.48)	0.37 (0.10, 1.29)	0.50 (0.14, 1.60)	1.80 (0.12, 62.94)	0.70 (0.13, 3.66)	0.58 (0.23, 1.50)	0.33 (0.01, 5.42)	0.38 (0.10, 1.35)	0.63 (0.19, 2.02)	0.69 (0.27, 1.74)
FIL200mgPO	**8.75 (1.13, 213.82)**	**0.12 (0.01, 0.91)**	0.57 (0.12, 2.60)	0.61 (0.13, 2.79)	4.48 (0.98, 34.61)	3.14 (0.59, 23.82)	1.24 (0.16, 10.63)	1.95 (0.34, 16.45)	0.77 (0.32, 1.72)	0.75 (0.06, 7.79)	0.63 (0.25, 1.52)	0.42 (0.11, 1.33)	0.56 (0.17, 1.65)	2.04 (0.13, 70.19)	0.78 (0.15, 3.91)	0.67 (0.28, 1.54)	0.38 (0.01, 6.52)	0.44 (0.11, 1.40)	0.71 (0.22, 2.13)	0.78 (0.33, 1.76)
FON0_1mg_kgIV	10.37 (0.12, 1022.55)	0.11 (0.00, 8.26)	0.61 (0.01, 37.45)	0.63 (0.01, 43.11)	4.86 (0.07, 355.00)	3.45 (0.04, 244.64)	1.29 (0.02, 97.72)	2.08 (0.02, 158.29)	0.78 (0.01, 50.28)	0.76 (0.01, 57.23)	0.66 (0.01, 42.23)	0.44 (0.01, 28.67)	0.58 (0.01, 36.07)	2.06 (0.02, 545.92)	0.82 (0.01, 57.32)	0.69 (0.01, 43.32)	0.40 (0.00, 43.78)	0.44 (0.01, 28.28)	0.73 (0.01, 44.18)	0.80 (0.01, 50.56)
FON1mg_kgIV	5.98 (0.11, 269.37)	0.07 (0.00, 1.71)	0.38 (0.01, 5.81)	0.41 (0.01, 6.90)	2.99 (0.06, 73.62)	2.08 (0.04, 54.26)	0.79 (0.01, 20.47)	1.24 (0.02, 33.44)	0.50 (0.01, 6.37)	0.46 (0.01, 14.43)	0.41 (0.01, 5.25)	0.27 (0.01, 3.78)	0.37 (0.01, 4.77)	1.30 (0.02, 105.54)	0.51 (0.01, 10.35)	0.44 (0.01, 5.59)	0.22 (0.00, 9.77)	0.27 (0.01, 4.08)	0.45 (0.01, 6.11)	0.50 (0.01, 5.97)
FON4mg_kgIV	6.92 (0.11, 316.27)	0.08 (0.00, 2.53)	0.44 (0.01, 8.47)	0.45 (0.01, 9.18)	3.55 (0.07, 101.00)	2.41 (0.05, 66.85)	0.89 (0.02, 26.75)	1.49 (0.03, 41.13)	0.58 (0.01, 8.12)	0.52 (0.01, 19.93)	0.47 (0.01, 7.09)	0.31 (0.01, 4.80)	0.41 (0.01, 6.08)	1.53 (0.02, 154.32)	0.57 (0.01, 13.95)	0.50 (0.01, 7.51)	0.27 (0.00, 11.87)	0.31 (0.01, 6.33)	0.52 (0.01, 8.54)	0.59 (0.01, 8.58)
GUS1200mgIV	**70.12 (3.40, 7460.68)**	0.79 (0.02, 46.03)	4.26 (0.32, 149.05)	4.35 (0.34, 179.58)	**34.91 (2.53, 1353.47)**	**24.49 (1.80, 1006.11)**	9.56 (0.55, 414.09)	15.51 (0.98, 640.76)	5.45 (0.63, 178.85)	5.53 (0.24, 281.65)	4.50 (0.50, 153.12)	3.08 (0.28, 117.51)	4.02 (0.36, 145.00)	16.50 (0.46, 1695.63)	5.57 (0.65, 168.66)	4.81 (0.52, 171.32)	2.67 (0.06, 158.22)	3.11 (0.28, 109.98)	5.02 (0.49, 164.54)	5.59 (0.64, 193.39)
GUS200mgIV	**17.39 (1.38, 497.86)**	0.22 (0.01, 3.39)	1.07 (0.12, 10.91)	1.17 (0.13, 11.17)	**8.82 (1.06, 115.68)**	5.96 (0.68, 82.30)	2.37 (0.21, 34.65)	3.74 (0.40, 53.98)	1.46 (0.26, 9.01)	1.37 (0.10, 25.14)	1.20 (0.22, 7.23)	0.82 (0.11, 5.70)	1.05 (0.16, 7.10)	3.98 (0.19, 192.31)	1.49 (0.30, 9.47)	1.29 (0.24, 7.62)	0.70 (0.02, 16.66)	0.85 (0.12, 6.20)	1.36 (0.21, 8.87)	1.52 (0.27, 8.80)
GUS600mgIV	**12.44 (1.03, 333.09)**	0.15 (0.01, 1.85)	0.77 (0.09, 5.56)	0.81 (0.11, 6.37)	6.14 (0.81, 68.32)	4.25 (0.54, 50.67)	1.64 (0.16, 20.40)	2.69 (0.27, 32.94)	1.01 (0.21, 4.90)	0.96 (0.06, 14.20)	0.83 (0.17, 4.22)	0.56 (0.08, 3.35)	0.74 (0.13, 4.17)	2.84 (0.14, 118.10)	1.06 (0.21, 4.60)	0.88 (0.18, 4.20)	0.49 (0.02, 10.24)	0.58 (0.08, 3.36)	0.93 (0.16, 4.98)	1.05 (0.21, 5.03)
MED700IV	**11.06 (1.03, 327.66)**	0.15 (0.01, 1.63)	0.71 (0.10, 5.12)	0.74 (0.11, 5.08)	5.67 (0.84, 62.77)	3.96 (0.56, 45.05)	1.55 (0.16, 17.45)	2.44 (0.28, 27.89)	0.97 (0.23, 3.94)	0.94 (0.06, 12.81)	0.79 (0.19, 3.43)	0.53 (0.09, 2.84)	0.69 (0.14, 3.55)	2.49 (0.15, 104.48)	1.01 (0.14, 7.14)	0.84 (0.20, 3.55)	0.47 (0.01, 8.16)	0.56 (0.10, 2.94)	0.88 (0.18, 4.77)	1.00 (0.24, 4.04)
MIR1000mgIV	**50.08 (3.66, 2018.72)**	0.60 (0.02, 15.21)	3.00 (0.41, 49.98)	3.24 (0.34, 47.85)	**25.04 (2.96, 510.23)**	**17.39 (1.89, 392.93)**	6.89 (0.52, 154.93)	**10.58 (1.09, 235.64)**	4.00 (0.79, 46.62)	3.83 (0.26, 112.50)	3.34 (0.64, 39.83)	2.24 (0.35, 23.36)	2.99 (0.49, 34.74)	11.36 (0.55, 615.68)	4.19 (0.50, 66.27)	3.55 (0.71, 40.82)	2.00 (0.06, 59.79)	2.30 (0.35, 27.59)	3.69 (0.61, 49.55)	4.07 (0.83, 45.72)
MIR200mgIV	**59.87 (3.33, 3880.83)**	0.72 (0.02, 32.55)	3.57 (0.29, 109.63)	3.81 (0.33, 111.13)	**29.91 (2.45, 1007.42)**	**20.83 (1.56, 694.98)**	8.11 (0.42, 284.20)	12.74 (0.99, 450.84)	4.76 (0.58, 124.32)	4.73 (0.20, 192.74)	3.89 (0.47, 99.96)	2.59 (0.25, 76.43)	3.46 (0.40, 96.78)	13.90 (0.46, 1628.44)	4.87 (0.39, 150.35)	4.13 (0.52, 108.23)	2.36 (0.06, 137.02)	2.76 (0.25, 69.73)	4.51 (0.49, 111.97)	4.83 (0.61, 128.31)

	TES400mg200mgSC	TOF15mgPO_BID	TOF1mgPO_BID	TOF5mgPO_BID	UPA12mgPO_BID	UPA24mgPO	UPA24mgPO_BID	UPA3mgPO_BID	UPA45mgPO	UPA6mgPO_BID	UST130mgIV	UST1mg_kgIV	UST3mg_kgIV	UST4_5mg_kgIV	UST6mg_kg90mgIV_SC	UST6mg_kgIV	UST90mgSC	VED0_5mg_kgIV	VED2mg_kgIV	VED300mgIV
MIR600mgIV	**14.51 (1.33, 454.33)**	0.19 (0.01, 2.42)	0.91 (0.14, 8.96)	0.99 (0.13, 8.48)	**7.88 (1.03, 90.73)**	5.44 (0.71, 62.14)	2.07 (0.19, 27.80)	3.31 (0.39, 38.94)	1.21 (0.30, 7.48)	1.24 (0.09, 20.51)	0.99 (0.24, 6.29)	0.66 (0.12, 4.69)	0.88 (0.18, 6.11)	3.32 (0.20, 136.71)	1.27 (0.17, 11.82)	1.06 (0.27, 6.31)	0.63 (0.02, 13.70)	0.69 (0.12, 5.39)	1.14 (0.23, 7.74)	1.23 (0.31, 8.05)
NAT300mgIV	**14.24 (1.95, 316.22)**	0.19 (0.01, 1.48)	0.93 (0.21, 4.04)	0.98 (0.21, 4.29)	**7.37 (1.70, 56.29)**	**5.09 (1.08, 38.26)**	2.01 (0.28, 17.01)	3.20 (0.55, 25.52)	1.24 (0.58, 2.60)	1.19 (0.10, 12.08)	1.02 (0.45, 2.24)	0.68 (0.21, 2.06)	0.91 (0.30, 2.57)	3.35 (0.24, 110.12)	1.28 (0.26, 6.24)	1.09 (0.51, 2.26)	0.61 (0.02, 9.79)	0.71 (0.19, 2.16)	1.14 (0.40, 3.26)	1.28 (0.60, 2.62)
NAT3mg_kgIV	**12.48 (1.34, 302.68)**	0.17 (0.01, 1.58)	0.79 (0.13, 4.47)	0.84 (0.15, 4.77)	**6.26 (1.06, 57.03)**	4.44 (0.72, 40.02)	1.75 (0.20, 18.42)	2.70 (0.38, 25.85)	1.06 (0.31, 3.67)	1.03 (0.07, 12.29)	0.88 (0.25, 3.04)	0.59 (0.12, 2.52)	0.78 (0.18, 3.13)	2.96 (0.17, 106.93)	1.10 (0.18, 6.86)	0.93 (0.27, 3.16)	0.50 (0.02, 10.70)	0.62 (0.12, 2.63)	0.98 (0.22, 3.96)	1.10 (0.32, 3.68)
NAT3mg_kgIVx2	**14.22 (1.41, 349.36)**	0.18 (0.01, 1.81)	0.91 (0.15, 5.67)	0.95 (0.15, 5.91)	**7.35 (1.19, 62.21)**	5.01 (0.80, 47.74)	1.99 (0.21, 20.09)	3.08 (0.45, 30.61)	1.20 (0.34, 4.37)	1.19 (0.08, 14.37)	1.00 (0.27, 3.56)	0.68 (0.14, 3.05)	0.89 (0.19, 3.82)	3.26 (0.18, 121.67)	1.26 (0.19, 7.98)	1.07 (0.29, 3.75)	0.59 (0.02, 11.55)	0.69 (0.14, 3.33)	1.11 (0.26, 4.63)	1.25 (0.35, 4.26)
NAT6mg_kgIVx2	**11.21 (1.08, 258.79)**	0.14 (0.01, 1.44)	0.70 (0.11, 4.23)	0.74 (0.12, 4.43)	5.59 (0.94, 54.61)	3.88 (0.59, 38.06)	1.56 (0.17, 16.73)	2.40 (0.35, 24.61)	0.92 (0.26, 3.53)	0.91 (0.07, 11.33)	0.76 (0.21, 2.91)	0.52 (0.10, 2.52)	0.67 (0.15, 3.12)	2.52 (0.14, 89.39)	0.97 (0.15, 6.74)	0.82 (0.23, 3.04)	0.45 (0.02, 8.81)	0.53 (0.11, 2.51)	0.86 (0.19, 4.05)	0.96 (0.27, 3.43)
NNC2mg_kgSC	4.25 (0.25, 126.59)	**0.05 (0.00, 0.78)**	0.26 (0.02, 2.35)	0.28 (0.03, 2.47)	2.19 (0.20, 25.35)	1.52 (0.13, 18.97)	0.61 (0.04, 7.59)	0.93 (0.07, 12.40)	0.36 (0.05, 2.02)	0.34 (0.02, 5.61)	0.30 (0.04, 1.73)	0.20 (0.02, 1.32)	0.27 (0.03, 1.69)	0.94 (0.03, 47.78)	0.37 (0.03, 3.36)	0.31 (0.04, 1.80)	0.17 (0.00, 3.44)	0.20 (0.02, 1.54)	0.33 (0.04, 2.14)	0.37 (0.05, 2.17)
ONE10mgSC	15.50 (0.27, 1151.54)	0.19 (0.00, 8.52)	0.90 (0.02, 32.20)	0.97 (0.02, 33.35)	7.88 (0.20, 307.61)	5.54 (0.12, 227.49)	2.10 (0.04, 92.14)	3.47 (0.08, 137.82)	1.30 (0.04, 30.60)	1.29 (0.02, 59.32)	1.05 (0.03, 26.96)	0.70 (0.02, 19.86)	0.92 (0.02, 24.14)	3.56 (0.05, 306.10)	1.33 (0.03, 35.72)	1.14 (0.03, 28.04)	0.56 (0.01, 43.66)	0.73 (0.02, 20.43)	1.17 (0.03, 32.59)	1.33 (0.04, 30.70)
ONE25mgSC	13.42 (0.16, 1208.47)	0.15 (0.00, 11.81)	0.77 (0.02, 37.61)	0.81 (0.02, 41.17)	6.40 (0.14, 357.66)	4.53 (0.09, 261.54)	1.70 (0.03, 113.36)	2.91 (0.06, 171.67)	1.04 (0.03, 38.15)	1.02 (0.02, 69.04)	0.84 (0.02, 32.03)	0.57 (0.01, 23.45)	0.77 (0.02, 30.53)	2.92 (0.04, 346.99)	1.11 (0.02, 48.68)	0.92 (0.02, 33.63)	0.52 (0.00, 39.79)	0.58 (0.01, 22.11)	0.93 (0.02, 35.03)	1.05 (0.03, 38.52)
ONE35mgSC	3.27 (0.07, 146.26)	**0.04 (0.00, 0.86)**	0.20 (0.01, 3.05)	0.21 (0.01, 3.12)	1.66 (0.05, 31.14)	1.13 (0.04, 23.32)	0.44 (0.01, 10.14)	0.69 (0.02, 14.42)	0.27 (0.01, 3.01)	0.24 (0.01, 7.67)	0.22 (0.01, 2.56)	0.15 (0.01, 1.80)	0.19 (0.01, 2.54)	0.71 (0.01, 39.30)	0.28 (0.01, 4.62)	0.24 (0.01, 2.58)	0.12 (0.00, 4.43)	0.15 (0.01, 2.05)	0.24 (0.01, 3.31)	0.28 (0.01, 3.23)
ONE50mgSC	15.06 (0.24, 2385.21)	0.19 (0.00, 15.27)	0.92 (0.02, 75.18)	0.98 (0.03, 69.90)	7.79 (0.18, 585.87)	5.28 (0.13, 410.95)	2.05 (0.04, 211.49)	3.30 (0.07, 257.91)	1.21 (0.04, 78.27)	1.23 (0.02, 98.15)	1.01 (0.03, 65.78)	0.65 (0.02, 42.31)	0.89 (0.03, 60.87)	3.34 (0.05, 721.68)	1.27 (0.03, 81.25)	1.07 (0.03, 66.33)	0.56 (0.01, 72.60)	0.67 (0.02, 56.13)	1.10 (0.03, 70.24)	1.23 (0.04, 72.05)
ONT22_5mgSC	5.16 (0.50, 125.70)	**0.07 (0.00, 0.63)**	0.33 (0.05, 1.88)	0.35 (0.05, 2.02)	2.66 (0.40, 23.05)	1.86 (0.26, 16.83)	0.72 (0.07, 7.13)	1.11 (0.15, 10.86)	0.44 (0.11, 1.45)	0.44 (0.03, 4.89)	0.37 (0.09, 1.22)	0.25 (0.05, 1.01)	0.33 (0.07, 1.26)	1.15 (0.07, 44.02)	0.46 (0.07, 2.87)	0.39 (0.10, 1.21)	0.21 (0.01, 4.02)	0.25 (0.05, 1.10)	0.41 (0.09, 1.62)	0.46 (0.12, 1.46)
ONT225mgSC	5.26 (0.54, 137.33)	**0.07 (0.00, 0.66)**	0.34 (0.05, 1.98)	0.36 (0.06, 2.10)	2.72 (0.43, 23.40)	1.89 (0.29, 17.94)	0.74 (0.08, 7.41)	1.17 (0.16, 10.90)	0.46 (0.12, 1.47)	0.44 (0.03, 5.31)	0.38 (0.10, 1.24)	0.25 (0.05, 1.06)	0.33 (0.07, 1.31)	1.18 (0.07, 39.33)	0.46 (0.07, 2.87)	0.40 (0.11, 1.26)	0.23 (0.01, 4.40)	0.26 (0.05, 1.11)	0.42 (0.09, 1.70)	0.47 (0.13, 1.49)
ONT75mgSC	6.09 (0.62, 166.17)	**0.08 (0.00, 0.75)**	0.40 (0.06, 2.44)	0.43 (0.06, 2.50)	3.18 (0.50, 29.39)	2.22 (0.33, 21.14)	0.86 (0.09, 8.56)	1.36 (0.19, 13.90)	0.54 (0.14, 1.85)	0.51 (0.03, 6.22)	0.45 (0.11, 1.56)	0.30 (0.06, 1.27)	0.40 (0.08, 1.62)	1.42 (0.08, 53.72)	0.56 (0.09, 3.71)	0.48 (0.12, 1.59)	0.26 (0.01, 4.94)	0.31 (0.06, 1.44)	0.49 (0.11, 2.08)	0.55 (0.14, 1.83)
PBO	**11.19 (1.65, 236.61)**	0.15 (0.01, 1.02)	0.73 (0.18, 2.91)	0.77 (0.19, 2.97)	**5.78 (1.47, 40.43)**	3.94 (0.89, 28.16)	1.59 (0.23, 12.49)	2.50 (0.47, 19.06)	0.97 (0.58, 1.66)	0.94 (0.09, 8.81)	0.80 (0.43, 1.45)	0.54 (0.18, 1.40)	0.72 (0.27, 1.75)	2.61 (0.20, 85.55)	1.01 (0.23, 4.45)	0.85 (0.50, 1.43)	0.49 (0.02, 7.47)	0.56 (0.17, 1.52)	0.89 (0.36, 2.30)	1.00 (0.60, 1.66)
PF10mgSC	**14.39 (1.57, 365.54)**	0.19 (0.01, 1.76)	0.93 (0.15, 5.30)	0.99 (0.17, 5.64)	**7.34 (1.35, 68.30)**	5.10 (0.85, 49.76)	2.01 (0.23, 19.46)	3.15 (0.48, 28.77)	1.25 (0.37, 4.03)	1.19 (0.09, 14.54)	1.03 (0.31, 3.40)	0.68 (0.15, 2.84)	0.91 (0.22, 3.56)	3.36 (0.21, 129.63)	1.29 (0.21, 8.32)	1.08 (0.35, 3.42)	0.62 (0.02, 11.66)	0.72 (0.15, 3.09)	1.13 (0.27, 4.84)	1.27 (0.41, 4.16)
PF200mgSC	5.49 (0.61, 125.71)	**0.07 (0.00, 0.63)**	0.34 (0.06, 1.93)	0.37 (0.06, 1.89)	2.76 (0.53, 23.50)	1.89 (0.34, 16.89)	0.77 (0.09, 7.01)	1.19 (0.18, 11.00)	0.46 (0.15, 1.34)	0.45 (0.04, 5.38)	0.39 (0.11, 1.16)	**0.25 (0.06, 0.94)**	0.35 (0.08, 1.24)	1.26 (0.08, 52.73)	0.48 (0.08, 2.76)	0.41 (0.13, 1.15)	0.23 (0.01, 4.06)	0.27 (0.06, 1.06)	0.43 (0.11, 1.58)	0.47 (0.16, 1.35)
PF50mgSC	**11.38 (1.30, 253.06)**	0.15 (0.01, 1.37)	0.73 (0.13, 4.20)	0.80 (0.14, 4.27)	**5.88 (1.09, 52.74)**	4.02 (0.68, 36.94)	1.61 (0.20, 16.07)	2.52 (0.40, 23.86)	0.99 (0.31, 3.05)	0.93 (0.08, 11.56)	0.82 (0.25, 2.58)	0.54 (0.13, 2.11)	0.72 (0.18, 2.70)	2.69 (0.16, 92.51)	1.04 (0.18, 6.10)	0.87 (0.27, 2.57)	0.49 (0.02, 9.10)	0.56 (0.12, 2.35)	0.91 (0.23, 3.57)	1.02 (0.33, 3.04)
RIS1200mgIV	**41.02 (5.59, 929.21)**	0.55 (0.03, 4.10)	2.69 (0.60, 12.35)	2.84 (0.62, 12.43)	**21.46 (4.64, 154.68)**	**14.92 (2.98, 114.88)**	5.86 (0.80, 47.13)	**9.27 (1.52, 71.44)**	**3.57 (1.67, 7.75)**	3.48 (0.30, 34.46)	**2.95 (1.27, 6.68)**	1.97 (0.60, 6.18)	2.61 (0.85, 7.55)	9.69 (0.69, 321.55)	3.72 (0.74, 17.96)	**3.13 (1.47, 6.82)**	1.78 (0.07, 29.30)	2.06 (0.56, 6.67)	**3.30 (1.09, 9.86)**	**3.64 (1.67, 8.04)**
RIS200mgIV	**17.67 (1.99, 424.51)**	0.23 (0.01, 2.11)	1.11 (0.20, 6.46)	1.17 (0.22, 6.64)	**8.99 (1.64, 80.97)**	**6.16 (1.04, 57.69)**	2.44 (0.29, 24.38)	3.82 (0.58, 36.27)	1.47 (0.49, 4.96)	1.46 (0.12, 17.18)	1.22 (0.39, 4.08)	0.82 (0.19, 3.34)	1.07 (0.27, 4.40)	4.07 (0.23, 156.77)	1.54 (0.26, 9.51)	1.29 (0.43, 4.34)	0.74 (0.02, 13.86)	0.84 (0.18, 3.97)	1.36 (0.35, 5.63)	1.51 (0.51, 4.98)
RIS600mgIV	**25.95 (3.72, 580.11)**	0.36 (0.02, 2.58)	1.71 (0.40, 7.75)	1.81 (0.42, 7.68)	**13.59 (3.07, 103.36)**	**9.40 (2.03, 72.79)**	3.71 (0.53, 30.75)	**5.88 (1.07, 47.03)**	**2.29 (1.15, 4.74)**	2.20 (0.19, 21.32)	1.90 (0.88, 4.07)	1.27 (0.40, 3.72)	1.67 (0.56, 4.72)	6.08 (0.43, 205.17)	2.40 (0.48, 11.24)	2.00 (0.97, 4.06)	1.15 (0.05, 18.33)	1.30 (0.39, 4.02)	2.10 (0.74, 5.83)	**2.34 (1.17, 4.80)**
SEC10mg_kgIV	9.07 (0.74, 237.24)	0.11 (0.00, 1.20)	0.57 (0.07, 3.77)	0.59 (0.08, 3.88)	4.57 (0.58, 44.04)	3.15 (0.37, 31.54)	1.23 (0.11, 13.03)	1.94 (0.22, 21.61)	0.76 (0.14, 3.15)	0.72 (0.05, 9.41)	0.63 (0.12, 2.56)	0.42 (0.06, 2.02)	0.55 (0.09, 2.62)	2.03 (0.10, 87.11)	0.78 (0.10, 5.81)	0.67 (0.13, 2.62)	0.35 (0.01, 7.28)	0.43 (0.06, 2.31)	0.70 (0.12, 3.38)	0.78 (0.15, 3.08)

	TES400mg200mgSC	TOF15mgPO_BID	TOF1mgPO_BID	TOF5mgPO_BID	UPA12mgPO_BID	UPA24mgPO	UPA24mgPO_BID	UPA3mgPO_BID	UPA45mgPO	UPA6mgPO_BID	UST130mgIV	UST1mg_kgIV	UST3mg_kgIV	UST4_5mg_kgIV	UST6mg_kg90mgIV_SC	UST6mg_kgIV	UST90mgSC	VED0_5mg_kgIV	VED2mg_kgIV	VED300mgIV
TES400mg200mgSC	TES400mg200mgSC	**0.01 (0.00, 0.20)**	**0.07 (0.00, 0.71)**	**0.07 (0.00, 0.77)**	0.50 (0.02, 7.99)	0.35 (0.01, 5.97)	0.14 (0.00, 2.33)	0.21 (0.01, 3.81)	**0.09 (0.00, 0.62)**	0.08 (0.00, 1.59)	**0.07 (0.00, 0.56)**	**0.05 (0.00, 0.42)**	**0.06 (0.00, 0.54)**	0.22 (0.01, 11.13)	0.09 (0.00, 1.05)	**0.08 (0.00, 0.55)**	0.04 (0.00, 1.06)	**0.05 (0.00, 0.45)**	**0.08 (0.00, 0.68)**	**0.09 (0.00, 0.64)**
TOF15mgPO_BID	**84.32 (4.93, 4583.48)**	TOF15mgPO_BID	4.79 (0.67, 102.00)	5.03 (0.67, 107.09)	**40.60 (3.43, 1493.22)**	**28.41 (2.20, 1024.70)**	11.03 (0.67, 442.38)	**17.23 (1.37, 626.09)**	6.47 (0.87, 143.61)	6.41 (0.29, 289.36)	5.30 (0.69, 117.82)	3.53 (0.40, 83.18)	4.78 (0.52, 106.82)	19.89 (0.64, 1791.90)	6.99 (0.58, 197.94)	5.63 (0.76, 117.71)	3.24 (0.07, 198.94)	3.70 (0.38, 87.09)	6.03 (0.64, 128.71)	6.58 (0.92, 136.38)
TOF1mgPO_BID	**15.38 (1.42, 419.41)**	0.21 (0.01, 1.49)	TOF1mgPO_BID	1.06 (0.23, 4.76)	**8.10 (1.06, 93.62)**	5.70 (0.70, 68.13)	2.24 (0.20, 28.22)	3.47 (0.41, 44.92)	1.34 (0.31, 5.72)	1.30 (0.08, 21.29)	1.10 (0.24, 4.96)	0.73 (0.12, 4.13)	0.98 (0.17, 5.12)	3.55 (0.17, 173.95)	1.37 (0.19, 11.23)	1.17 (0.27, 5.17)	0.66 (0.02, 12.39)	0.76 (0.13, 4.20)	1.24 (0.23, 6.18)	1.37 (0.31, 6.21)
TOF5mgPO_BID	**14.88 (1.30, 442.12)**	0.20 (0.01, 1.50)	0.94 (0.21, 4.35)	TOF5mgPO_BID	7.73 (0.99, 84.67)	5.42 (0.63, 62.76)	2.12 (0.19, 25.11)	3.32 (0.37, 39.70)	1.27 (0.29, 5.64)	1.25 (0.08, 17.08)	1.04 (0.23, 4.77)	0.69 (0.12, 3.83)	0.92 (0.17, 4.88)	3.28 (0.18, 161.09)	1.28 (0.17, 10.23)	1.11 (0.26, 4.91)	0.61 (0.02, 12.79)	0.71 (0.12, 4.00)	1.14 (0.22, 6.35)	1.28 (0.31, 5.74)
UPA12mgPO_BID	1.99 (0.13, 47.42)	**0.02 (0.00, 0.29)**	**0.12 (0.01, 0.95)**	0.13 (0.01, 1.01)	UPA12mgPO_BID	0.69 (0.26, 1.77)	**0.27 (0.06, 0.92)**	0.44 (0.13, 1.24)	**0.17 (0.02, 0.74)**	**0.17 (0.02, 0.64)**	**0.14 (0.02, 0.62)**	**0.09 (0.01, 0.52)**	**0.12 (0.01, 0.65)**	0.43 (0.02, 18.87)	0.17 (0.02, 1.25)	**0.15 (0.02, 0.64)**	0.08 (0.00, 1.88)	**0.10 (0.01, 0.53)**	**0.15 (0.02, 0.81)**	**0.17 (0.02, 0.73)**
UPA24mgPO	2.86 (0.17, 74.92)	**0.04 (0.00, 0.45)**	0.18 (0.01, 1.42)	0.18 (0.02, 1.60)	1.45 (0.56, 3.84)	UPA24mgPO	0.39 (0.08, 1.49)	0.62 (0.17, 2.00)	0.25 (0.03, 1.17)	0.24 (0.03, 1.04)	0.20 (0.03, 1.00)	**0.13 (0.01, 0.80)**	**0.18 (0.02, 0.99)**	0.63 (0.03, 25.36)	0.25 (0.02, 1.97)	0.21 (0.03, 1.01)	0.11 (0.00, 2.73)	**0.14 (0.01, 0.83)**	0.22 (0.02, 1.30)	0.25 (0.03, 1.22)
UPA24mgPO_BID	7.40 (0.43, 215.54)	0.09 (0.00, 1.49)	0.45 (0.04, 5.03)	0.47 (0.04, 5.27)	**3.66 (1.09, 16.68)**	2.57 (0.67, 12.05)	UPA24mgPO_BID	1.57 (0.34, 8.54)	0.62 (0.07, 4.29)	0.60 (0.07, 4.05)	0.50 (0.06, 3.78)	0.34 (0.03, 2.83)	0.45 (0.04, 3.59)	1.66 (0.07, 83.87)	0.64 (0.05, 6.69)	0.53 (0.06, 3.79)	0.29 (0.01, 7.88)	0.35 (0.03, 2.95)	0.56 (0.06, 4.65)	0.64 (0.08, 4.44)
UPA3mgPO_BID	4.73 (0.26, 125.38)	**0.06 (0.00, 0.73)**	0.29 (0.02, 2.46)	0.30 (0.03, 2.74)	2.29 (0.81, 7.84)	1.60 (0.50, 5.82)	0.64 (0.12, 2.92)	UPA3mgPO_BID	0.39 (0.05, 2.29)	0.39 (0.04, 2.03)	0.33 (0.04, 1.79)	0.21 (0.02, 1.49)	0.28 (0.03, 1.90)	1.01 (0.05, 48.29)	0.40 (0.03, 3.38)	0.35 (0.04, 1.93)	0.19 (0.00, 4.57)	0.22 (0.02, 1.58)	0.36 (0.04, 2.21)	0.40 (0.05, 2.19)
UPA45mgPO	**11.45 (1.62, 252.22)**	0.15 (0.01, 1.15)	0.75 (0.17, 3.27)	0.79 (0.18, 3.41)	**5.92 (1.35, 45.60)**	4.06 (0.85, 31.33)	1.62 (0.23, 14.00)	2.57 (0.44, 21.13)	UPA45mgPO	0.95 (0.09, 10.00)	0.83 (0.36, 1.81)	0.55 (0.17, 1.67)	0.73 (0.24, 2.06)	2.64 (0.19, 90.38)	1.03 (0.21, 5.01)	0.88 (0.42, 1.83)	0.49 (0.02, 8.11)	0.57 (0.16, 1.77)	0.92 (0.31, 2.67)	1.03 (0.49, 2.16)
UPA6mgPO_BID	12.84 (0.63, 435.67)	0.16 (0.00, 3.41)	0.77 (0.05, 12.49)	0.80 (0.06, 12.05)	**6.05 (1.57, 48.27)**	4.21 (0.96, 34.18)	1.66 (0.25, 14.92)	2.58 (0.49, 23.53)	1.05 (0.10, 11.33)	UPA6mgPO_BID	0.86 (0.08, 9.38)	0.58 (0.05, 7.01)	0.77 (0.06, 8.82)	2.68 (0.12, 170.28)	1.07 (0.07, 15.89)	0.90 (0.09, 9.78)	0.50 (0.01, 15.23)	0.59 (0.04, 8.16)	0.95 (0.08, 12.39)	1.06 (0.11, 12.51)
UST130mgIV	**14.11 (1.80, 314.75)**	0.19 (0.01, 1.45)	0.91 (0.20, 4.09)	0.96 (0.21, 4.30)	**7.33 (1.61, 55.84)**	4.99 (1.00, 39.63)	1.99 (0.26, 16.41)	3.07 (0.56, 26.35)	1.21 (0.55, 2.75)	1.16 (0.11, 12.48)	UST130mgIV	0.67 (0.20, 2.00)	0.89 (0.30, 2.42)	3.25 (0.24, 107.96)	1.26 (0.25, 6.26)	1.06 (0.58, 1.95)	0.61 (0.02, 10.17)	0.69 (0.19, 2.25)	1.13 (0.37, 3.35)	1.23 (0.56, 2.77)
UST1mg_kgIV	**21.43 (2.40, 562.15)**	0.28 (0.01, 2.49)	1.37 (0.24, 8.50)	1.45 (0.26, 8.19)	**10.99 (1.93, 103.21)**	**7.48 (1.25, 76.32)**	2.97 (0.35, 31.74)	4.79 (0.67, 45.93)	1.82 (0.60, 6.05)	1.72 (0.14, 21.35)	1.49 (0.50, 4.93)	UST1mg_kgIV	1.33 (0.43, 4.39)	5.02 (0.30, 191.56)	1.90 (0.31, 12.16)	1.59 (0.59, 4.87)	0.90 (0.03, 16.95)	1.03 (0.22, 4.50)	1.69 (0.42, 6.94)	1.85 (0.62, 6.01)
UST3mg_kgIV	**16.17 (1.84, 363.55)**	0.21 (0.01, 1.91)	1.02 (0.20, 5.73)	1.08 (0.20, 6.00)	**8.15 (1.54, 76.62)**	**5.59 (1.01, 55.79)**	2.23 (0.28, 22.29)	3.53 (0.53, 36.20)	1.36 (0.49, 4.17)	1.31 (0.11, 15.86)	1.12 (0.41, 3.28)	0.75 (0.23, 2.32)	UST3mg_kgIV	3.70 (0.24, 142.18)	1.41 (0.24, 8.30)	1.19 (0.48, 3.16)	0.68 (0.02, 12.54)	0.78 (0.18, 3.19)	1.24 (0.35, 4.78)	1.39 (0.50, 4.32)
UST4_5mg_kgIV	4.58 (0.09, 191.59)	0.05 (0.00, 1.57)	0.28 (0.01, 5.72)	0.30 (0.01, 5.71)	2.30 (0.05, 49.21)	1.58 (0.04, 36.34)	0.60 (0.01, 15.04)	0.99 (0.02, 20.45)	0.38 (0.01, 5.18)	0.37 (0.01, 8.23)	0.31 (0.01, 4.13)	0.20 (0.01, 3.34)	0.27 (0.01, 4.26)	UST4_5mg_kgIV	0.39 (0.01, 8.15)	0.33 (0.01, 4.46)	0.18 (0.00, 6.82)	0.21 (0.01, 3.58)	0.34 (0.01, 5.48)	0.38 (0.01, 5.45)
UST6mg_kg90mgIV_SC	11.26 (0.96, 327.04)	0.14 (0.01, 1.73)	0.73 (0.09, 5.35)	0.78 (0.10, 6.05)	5.90 (0.80, 62.30)	4.05 (0.51, 46.35)	1.56 (0.15, 20.57)	2.47 (0.30, 29.25)	0.97 (0.20, 4.68)	0.93 (0.06, 13.49)	0.79 (0.16, 4.07)	0.53 (0.08, 3.20)	0.71 (0.12, 4.09)	2.57 (0.12, 115.53)	UST6mg_kg90mgIV_SC	0.84 (0.18, 4.07)	0.47 (0.01, 9.39)	0.54 (0.08, 3.28)	0.88 (0.16, 5.36)	1.00 (0.21, 4.78)
UST6mg_kgIV	**13.04 (1.82, 282.09)**	0.18 (0.01, 1.32)	0.85 (0.19, 3.76)	0.90 (0.20, 3.91)	**6.80 (1.55, 55.04)**	4.67 (0.99, 37.73)	1.87 (0.26, 15.60)	2.88 (0.52, 24.22)	1.14 (0.55, 2.40)	1.11 (0.10, 11.29)	0.94 (0.51, 1.73)	0.63 (0.21, 1.70)	0.84 (0.32, 2.09)	2.99 (0.22, 103.13)	1.19 (0.25, 5.71)	UST6mg_kgIV	0.56 (0.02, 9.20)	0.66 (0.17, 2.05)	1.05 (0.37, 2.97)	1.16 (0.57, 2.43)
UST90mgSC	26.65 (0.94, 1946.82)	0.31 (0.01, 13.90)	1.52 (0.08, 47.88)	1.63 (0.08, 56.43)	12.55 (0.53, 655.92)	8.85 (0.37, 452.77)	3.46 (0.13, 182.44)	5.34 (0.22, 295.04)	2.03 (0.12, 54.06)	1.98 (0.07, 130.93)	1.64 (0.10, 45.74)	1.11 (0.06, 30.65)	1.48 (0.08, 40.45)	5.56 (0.15, 726.19)	2.11 (0.11, 75.92)	1.78 (0.11, 44.31)	UST90mgSC	1.16 (0.07, 34.06)	1.86 (0.11, 48.93)	2.07 (0.13, 51.54)
VED0_5mg_kgIV	**20.66 (2.22, 552.24)**	0.27 (0.01, 2.60)	1.32 (0.24, 7.61)	1.40 (0.25, 8.14)	**10.52 (1.88, 103.71)**	**7.31 (1.21, 74.68)**	2.87 (0.34, 31.07)	4.55 (0.63, 46.85)	1.74 (0.56, 6.40)	1.70 (0.12, 22.45)	1.44 (0.44, 5.30)	0.97 (0.22, 4.51)	1.28 (0.31, 5.45)	4.83 (0.28, 171.89)	1.84 (0.30, 11.80)	1.52 (0.49, 5.77)	0.87 (0.03, 15.08)	VED0_5mg_kgIV	1.61 (0.57, 5.21)	1.78 (0.58, 6.46)
VED2mg_kgIV	**12.57 (1.47, 302.50)**	0.17 (0.01, 1.56)	0.80 (0.16, 4.27)	0.88 (0.16, 4.45)	**6.52 (1.24, 57.67)**	4.51 (0.77, 43.08)	1.78 (0.21, 17.06)	2.75 (0.45, 26.14)	1.08 (0.37, 3.20)	1.05 (0.08, 12.50)	0.89 (0.30, 2.69)	0.59 (0.14, 2.37)	0.80 (0.21, 2.86)	2.94 (0.18, 109.40)	1.14 (0.19, 6.31)	0.95 (0.34, 2.74)	0.54 (0.02, 8.71)	0.62 (0.19, 1.77)	VED2mg_kgIV	1.11 (0.39, 3.15)
VED300mgIV	**11.18 (1.57, 241.92)**	0.15 (0.01, 1.09)	0.73 (0.16, 3.26)	0.78 (0.17, 3.19)	**5.86 (1.36, 45.09)**	3.99 (0.82, 31.47)	1.56 (0.23, 12.75)	2.49 (0.46, 19.48)	0.98 (0.46, 2.06)	0.95 (0.08, 9.47)	0.81 (0.36, 1.78)	0.54 (0.17, 1.62)	0.72 (0.23, 2.00)	2.65 (0.18, 84.81)	1.00 (0.21, 4.79)	0.86 (0.41, 1.75)	0.48 (0.02, 7.63)	0.56 (0.15, 1.73)	0.90 (0.32, 2.59)	VED300mgIV

TABLE S16B. League table for any adverse events.

	ABA10mg_kgIV	ABA30mg_kgIV	ABA3mg_kgIV	ADA160mg80mg40mgSC	ADA160mg80mg60mgSC	ADA160mg80mgSC	ADA40mg20mgSC	ADA80mg40mgSC	AMI0_4mgOR	AND150mgSC1_1	AND150mgSC2_2	AND300mgSC	API100mgOT	API50mgOR	BRI400mgIV	BRI700mgIV	BRO210mgIV	BRO350mgIV	BRO700mgIV	CDP10mg_kgIV	CER1_25mgIV	CER100mgSC
ABA10mg_kgIV	ABA10mg_kgIV	0.99 (0.78, 1.23)	0.98 (0.80, 1.19)	0.80 (0.52, 1.23)	0.98 (0.63, 1.54)	0.88 (0.68, 1.14)	0.84 (0.61, 1.14)	0.85 (0.63, 1.13)	1.15 (0.74, 1.80)	0.91 (0.60, 1.41)	0.88 (0.58, 1.36)	1.02 (0.69, 1.56)	0.96 (0.72, 1.31)	0.88 (0.64, 1.20)	0.94 (0.66, 1.33)	0.87 (0.63, 1.20)	0.90 (0.60, 1.32)	1.03 (0.71, 1.47)	1.07 (0.74, 1.53)	1.03 (0.78, 1.37)	0.48 (0.07, 1.60)	1.10 (0.82, 1.50)
ABA30mg_kgIV	1.01 (0.81, 1.28)	ABA30mg_kgIV	0.99 (0.79, 1.25)	0.81 (0.52, 1.27)	0.99 (0.63, 1.58)	0.89 (0.68, 1.19)	0.85 (0.62, 1.18)	0.86 (0.64, 1.18)	1.17 (0.75, 1.86)	0.92 (0.61, 1.45)	0.89 (0.59, 1.41)	1.04 (0.69, 1.61)	0.98 (0.72, 1.36)	0.89 (0.64, 1.24)	0.95 (0.67, 1.38)	0.88 (0.63, 1.24)	0.91 (0.61, 1.36)	1.04 (0.71, 1.52)	1.08 (0.74, 1.57)	1.05 (0.78, 1.43)	0.49 (0.07, 1.63)	1.12 (0.82, 1.54)
ABA3mg_kgIV	1.03 (0.84, 1.26)	1.01 (0.80, 1.26)	ABA3mg_kgIV	0.82 (0.53, 1.27)	1.00 (0.64, 1.57)	0.90 (0.70, 1.18)	0.86 (0.63, 1.17)	0.87 (0.65, 1.16)	1.18 (0.75, 1.86)	0.93 (0.62, 1.45)	0.90 (0.60, 1.40)	1.05 (0.71, 1.59)	0.99 (0.74, 1.34)	0.90 (0.66, 1.24)	0.97 (0.68, 1.36)	0.89 (0.64, 1.23)	0.92 (0.62, 1.35)	1.05 (0.73, 1.51)	1.09 (0.76, 1.56)	1.06 (0.80, 1.41)	0.50 (0.07, 1.64)	1.13 (0.84, 1.53)
ADA160mg80mg40mgSC	1.25 (0.81, 1.92)	1.23 (0.79, 1.91)	1.21 (0.79, 1.87)	ADA160mg80mg40mgSC	1.22 (0.72, 2.11)	1.09 (0.73, 1.66)	1.04 (0.68, 1.63)	1.06 (0.69, 1.63)	1.44 (0.83, 2.50)	1.13 (0.67, 1.94)	1.10 (0.65, 1.89)	1.27 (0.77, 2.17)	1.20 (0.78, 1.85)	1.09 (0.70, 1.70)	1.17 (0.72, 1.89)	1.08 (0.69, 1.70)	1.11 (0.68, 1.86)	1.27 (0.79, 2.08)	1.33 (0.82, 2.13)	1.29 (0.84, 1.97)	0.61 (0.08, 2.10)	1.37 (0.88, 2.14)
ADA160mg80mg60mgSC	1.02 (0.65, 1.58)	1.01 (0.63, 1.58)	1.00 (0.64, 1.56)	0.82 (0.47, 1.39)	ADA160mg80mg60mgSC	0.89 (0.59, 1.37)	0.86 (0.53, 1.33)	0.86 (0.55, 1.34)	1.18 (0.67, 2.05)	0.93 (0.54, 1.61)	0.91 (0.53, 1.55)	1.05 (0.62, 1.78)	0.99 (0.62, 1.55)	0.89 (0.56, 1.41)	0.96 (0.59, 1.54)	0.88 (0.55, 1.38)	0.92 (0.54, 1.51)	1.05 (0.64, 1.68)	1.09 (0.67, 1.75)	1.06 (0.68, 1.62)	0.49 (0.07, 1.70)	1.13 (0.72, 1.76)
ADA160mg80mgSC	1.14 (0.87, 1.46)	1.13 (0.84, 1.47)	1.12 (0.85, 1.44)	0.92 (0.60, 1.36)	1.12 (0.73, 1.71)	ADA160mg80mgSC	0.96 (0.74, 1.21)	0.97 (0.77, 1.20)	1.32 (0.85, 2.03)	1.04 (0.70, 1.56)	1.01 (0.67, 1.51)	1.16 (0.80, 1.73)	1.10 (0.83, 1.44)	1.00 (0.74, 1.32)	1.08 (0.77, 1.46)	0.99 (0.74, 1.32)	1.02 (0.70, 1.48)	1.17 (0.84, 1.63)	1.21 (0.87, 1.67)	1.18 (0.91, 1.52)	0.55 (0.08, 1.81)	1.26 (0.96, 1.64)
ADA40mg20mgSC	1.20 (0.87, 1.63)	1.18 (0.85, 1.63)	1.17 (0.85, 1.60)	0.96 (0.61, 1.48)	1.17 (0.75, 1.87)	1.04 (0.83, 1.35)	ADA40mg20mgSC	1.01 (0.79, 1.32)	1.38 (0.87, 2.20)	1.09 (0.71, 1.72)	1.05 (0.68, 1.65)	1.22 (0.81, 1.89)	1.15 (0.83, 1.59)	1.05 (0.75, 1.47)	1.12 (0.77, 1.62)	1.04 (0.74, 1.47)	1.07 (0.71, 1.61)	1.23 (0.84, 1.80)	1.27 (0.88, 1.85)	1.24 (0.92, 1.67)	0.58 (0.08, 1.93)	1.32 (0.96, 1.84)
ADA80mg40mgSC	1.18 (0.88, 1.58)	1.16 (0.85, 1.57)	1.15 (0.86, 1.54)	0.95 (0.61, 1.45)	1.16 (0.74, 1.82)	1.03 (0.84, 1.29)	0.99 (0.76, 1.27)	ADA80mg40mgSC	1.36 (0.87, 2.15)	1.07 (0.71, 1.66)	1.04 (0.69, 1.61)	1.21 (0.81, 1.84)	1.14 (0.84, 1.54)	1.03 (0.75, 1.43)	1.11 (0.78, 1.57)	1.02 (0.75, 1.41)	1.06 (0.71, 1.58)	1.21 (0.85, 1.74)	1.25 (0.88, 1.80)	1.22 (0.92, 1.63)	0.57 (0.08, 1.89)	1.30 (0.96, 1.77)
AMI0_4mgOR	0.87 (0.56, 1.36)	0.86 (0.54, 1.34)	0.85 (0.54, 1.33)	0.69 (0.40, 1.20)	0.85 (0.49, 1.50)	0.76 (0.49, 1.17)	0.73 (0.46, 1.15)	0.74 (0.47, 1.15)	AMI0_4mgOR	0.79 (0.45, 1.37)	0.76 (0.45, 1.31)	0.89 (0.52, 1.50)	0.84 (0.53, 1.32)	0.76 (0.48, 1.20)	0.82 (0.50, 1.32)	0.75 (0.47, 1.20)	0.78 (0.46, 1.31)	0.89 (0.54, 1.46)	0.92 (0.56, 1.51)	0.90 (0.57, 1.40)	0.42 (0.06, 1.43)	0.96 (0.60, 1.49)
AND150mgSC1_1	1.10 (0.71, 1.67)	1.09 (0.69, 1.65)	1.07 (0.69, 1.62)	0.88 (0.51, 1.50)	1.08 (0.62, 1.86)	0.96 (0.64, 1.43)	0.92 (0.58, 1.42)	0.93 (0.60, 1.41)	1.27 (0.73, 2.20)	AND150mgSC1_1	0.97 (0.68, 1.37)	1.12 (0.82, 1.56)	1.06 (0.68, 1.62)	0.96 (0.61, 1.48)	1.03 (0.64, 1.62)	0.95 (0.61, 1.47)	0.98 (0.59, 1.60)	1.12 (0.70, 1.82)	1.17 (0.74, 1.88)	1.14 (0.74, 1.71)	0.53 (0.07, 1.83)	1.21 (0.78, 1.86)
AND150mgSC2_2	1.14 (0.73, 1.71)	1.12 (0.71, 1.71)	1.11 (0.72, 1.67)	0.91 (0.53, 1.53)	1.10 (0.64, 1.89)	0.99 (0.66, 1.49)	0.95 (0.61, 1.47)	0.96 (0.62, 1.45)	1.31 (0.76, 2.25)	1.04 (0.73, 1.46)	AND150mgSC2_2	1.16 (0.84, 1.61)	1.10 (0.70, 1.66)	0.99 (0.63, 1.52)	1.07 (0.66, 1.69)	0.98 (0.63, 1.52)	1.02 (0.61, 1.66)	1.16 (0.72, 1.85)	1.20 (0.74, 1.92)	1.18 (0.77, 1.76)	0.55 (0.07, 1.91)	1.25 (0.80, 1.91)
AND300mgSC	0.98 (0.64, 1.45)	0.96 (0.62, 1.44)	0.96 (0.63, 1.41)	0.79 (0.46, 1.31)	0.96 (0.56, 1.62)	0.86 (0.58, 1.25)	0.82 (0.53, 1.24)	0.83 (0.54, 1.23)	1.13 (0.67, 1.93)	0.89 (0.64, 1.22)	0.86 (0.62, 1.19)	AND300mgSC	0.95 (0.62, 1.41)	0.86 (0.55, 1.30)	0.92 (0.58, 1.42)	0.85 (0.55, 1.29)	0.88 (0.53, 1.41)	1.00 (0.63, 1.57)	1.04 (0.65, 1.61)	1.01 (0.67, 1.48)	0.47 (0.06, 1.61)	1.08 (0.71, 1.61)
API100mgOT	1.04 (0.76, 1.39)	1.02 (0.74, 1.39)	1.02 (0.75, 1.36)	0.83 (0.54, 1.28)	1.01 (0.65, 1.60)	0.91 (0.69, 1.20)	0.87 (0.63, 1.20)	0.88 (0.65, 1.19)	1.20 (0.76, 1.89)	0.95 (0.62, 1.47)	0.91 (0.60, 1.43)	1.06 (0.71, 1.62)	API100mgOT	0.91 (0.71, 1.15)	0.98 (0.68, 1.39)	0.90 (0.65, 1.24)	0.93 (0.62, 1.38)	1.06 (0.74, 1.54)	1.10 (0.77, 1.58)	1.07 (0.80, 1.43)	0.50 (0.07, 1.66)	1.14 (0.84, 1.55)
API50mgOR	1.14 (0.83, 1.56)	1.13 (0.81, 1.55)	1.12 (0.81, 1.51)	0.92 (0.59, 1.42)	1.12 (0.71, 1.79)	1.00 (0.76, 1.35)	0.95 (0.68, 1.34)	0.97 (0.70, 1.33)	1.32 (0.83, 2.09)	1.04 (0.68, 1.64)	1.01 (0.66, 1.59)	1.17 (0.77, 1.81)	1.10 (0.87, 1.40)	API50mgOR	1.08 (0.73, 1.55)	0.99 (0.70, 1.40)	1.03 (0.68, 1.54)	1.17 (0.80, 1.70)	1.22 (0.84, 1.77)	1.18 (0.87, 1.61)	0.55 (0.08, 1.84)	1.26 (0.91, 1.75)
BRI400mgIV	1.06 (0.75, 1.51)	1.05 (0.72, 1.50)	1.04 (0.74, 1.47)	0.85 (0.53, 1.39)	1.04 (0.65, 1.69)	0.93 (0.68, 1.30)	0.89 (0.62, 1.29)	0.90 (0.64, 1.28)	1.22 (0.75, 2.01)	0.97 (0.62, 1.55)	0.94 (0.59, 1.51)	1.09 (0.71, 1.72)	1.02 (0.72, 1.48)	0.93 (0.64, 1.37)	BRI400mgIV	0.92 (0.72, 1.20)	0.95 (0.62, 1.48)	1.09 (0.72, 1.66)	1.13 (0.76, 1.68)	1.10 (0.78, 1.56)	0.51 (0.07, 1.74)	1.17 (0.82, 1.68)
BRI700mgIV	1.16 (0.83, 1.58)	1.14 (0.81, 1.59)	1.13 (0.81, 1.55)	0.93 (0.59, 1.46)	1.13 (0.72, 1.80)	1.01 (0.76, 1.35)	0.96 (0.68, 1.35)	0.98 (0.71, 1.33)	1.34 (0.84, 2.13)	1.05 (0.68, 1.64)	1.02 (0.66, 1.59)	1.18 (0.78, 1.82)	1.11 (0.80, 1.54)	1.01 (0.72, 1.42)	1.09 (0.83, 1.39)	BRI700mgIV	1.04 (0.68, 1.56)	1.19 (0.80, 1.74)	1.23 (0.84, 1.79)	1.19 (0.87, 1.63)	0.56 (0.08, 1.86)	1.27 (0.92, 1.76)
BRO210mgIV	1.11 (0.76, 1.66)	1.10 (0.74, 1.64)	1.08 (0.74, 1.61)	0.90 (0.54, 1.47)	1.09 (0.66, 1.85)	0.98 (0.67, 1.42)	0.93 (0.62, 1.40)	0.94 (0.63, 1.41)	1.29 (0.76, 2.19)	1.02 (0.63, 1.68)	0.98 (0.60, 1.65)	1.14 (0.71, 1.88)	1.07 (0.73, 1.61)	0.97 (0.65, 1.47)	1.05 (0.68, 1.62)	0.96 (0.64, 1.47)	BRO210mgIV	1.14 (0.83, 1.59)	1.18 (0.88, 1.65)	1.15 (0.79, 1.70)	0.54 (0.08, 1.87)	1.22 (0.83, 1.84)
BRO350mgIV	0.97 (0.68, 1.40)	0.96 (0.66, 1.40)	0.95 (0.66, 1.37)	0.78 (0.48, 1.27)	0.95 (0.59, 1.57)	0.86 (0.61, 1.18)	0.82 (0.56, 1.19)	0.83 (0.57, 1.18)	1.13 (0.69, 1.86)	0.89 (0.55, 1.43)	0.86 (0.54, 1.38)	1.00 (0.64, 1.58)	0.94 (0.65, 1.35)	0.86 (0.59, 1.25)	0.92 (0.60, 1.38)	0.84 (0.57, 1.24)	0.88 (0.63, 1.20)	BRO350mgIV	1.04 (0.79, 1.37)	1.01 (0.71, 1.44)	0.47 (0.07, 1.60)	1.08 (0.75, 1.55)
BRO700mgIV	0.94 (0.65, 1.35)	0.93 (0.64, 1.34)	0.92 (0.64, 1.31)	0.75 (0.47, 1.21)	0.92 (0.57, 1.50)	0.82 (0.60, 1.15)	0.79 (0.54, 1.14)	0.80 (0.56, 1.13)	1.09 (0.66, 1.78)	0.86 (0.53, 1.36)	0.83 (0.52, 1.34)	0.96 (0.62, 1.53)	0.91 (0.63, 1.30)	0.82 (0.57, 1.19)	0.89 (0.59, 1.32)	0.81 (0.56, 1.19)	0.85 (0.61, 1.14)	0.97 (0.73, 1.27)	BRO700mgIV	0.97 (0.69, 1.37)	0.45 (0.06, 1.54)	1.04 (0.73, 1.47)
CDP10mg_kgIV	0.97 (0.73, 1.28)	0.96 (0.70, 1.28)	0.94 (0.71, 1.25)	0.78 (0.51, 1.19)	0.95 (0.62, 1.47)	0.85 (0.66, 1.09)	0.81 (0.60, 1.09)	0.82 (0.61, 1.08)	1.11 (0.71, 1.77)	0.88 (0.59, 1.35)	0.85 (0.57, 1.30)	0.99 (0.67, 1.50)	0.93 (0.70, 1.25)	0.85 (0.62, 1.15)	0.91 (0.64, 1.28)	0.84 (0.61, 1.15)	0.87 (0.59, 1.27)	0.99 (0.69, 1.40)	1.03 (0.73, 1.46)	CDP10mg_kgIV	0.47 (0.07, 1.53)	1.07 (0.80, 1.43)
CER1_25mgIV	2.07 (0.63, 14.66)	2.05 (0.61, 14.48)	2.00 (0.61, 14.16)	1.65 (0.48, 11.96)	2.06 (0.59, 14.32)	1.80 (0.55, 12.87)	1.74 (0.52, 12.32)	1.76 (0.53, 12.54)	2.39 (0.70, 17.78)	1.87 (0.55, 14.39)	1.83 (0.52, 13.47)	2.13 (0.62, 15.62)	1.98 (0.60, 14.24)	1.81 (0.54, 12.88)	1.94 (0.57, 13.94)	1.80 (0.54, 12.90)	1.85 (0.53, 12.69)	2.13 (0.62, 15.06)	2.20 (0.65, 15.92)	2.14 (0.65, 15.14)	CER1_25mgIV	2.27 (0.69, 16.00)
CER100mgSC	0.91 (0.66, 1.23)	0.90 (0.65, 1.22)	0.88 (0.65, 1.19)	0.73 (0.47, 1.13)	0.89 (0.57, 1.40)	0.79 (0.61, 1.05)	0.76 (0.54, 1.04)	0.77 (0.57, 1.04)	1.04 (0.67, 1.66)	0.82 (0.54, 1.28)	0.80 (0.52, 1.25)	0.92 (0.62, 1.41)	0.88 (0.64, 1.19)	0.79 (0.57, 1.10)	0.85 (0.60, 1.23)	0.79 (0.57, 1.09)	0.82 (0.54, 1.20)	0.93 (0.64, 1.34)	0.97 (0.68, 1.38)	0.94 (0.70, 1.26)	0.44 (0.06, 1.46)	CER100mgSC

	ABA10mg_kgIV	ABA30mg_kgIV	ABA3mg_kgIV	ADA160mg80mg40mgSC	ADA160mg80mg60mgSC	ADA160mg80mgSC	ADA40mg20mgSC	ADA80mg40mgSC	AMI0_4mgOR	AND150mgSC1_1	AND150mgSC2_2	AND300mgSC	API100mgOT	API50mgOR	BRI400mgIV	BRI700mgIV	BRO210mgIV	BRO350mgIV	BRO700mgIV	CDP10mg_kgIV	CER1_25mgIV	CER100mgSC
CER10mgIV	0.96 (0.56, 1.75)	0.94 (0.54, 1.74)	0.94 (0.54, 1.70)	0.77 (0.42, 1.51)	0.94 (0.50, 1.89)	0.84 (0.50, 1.49)	0.79 (0.47, 1.48)	0.81 (0.48, 1.46)	1.10 (0.59, 2.19)	0.87 (0.47, 1.72)	0.84 (0.46, 1.66)	0.98 (0.53, 1.92)	0.92 (0.54, 1.69)	0.84 (0.48, 1.54)	0.90 (0.50, 1.66)	0.83 (0.48, 1.52)	0.86 (0.47, 1.63)	0.98 (0.55, 1.84)	1.01 (0.58, 1.92)	0.99 (0.58, 1.80)	0.47 (0.07, 1.56)	1.05 (0.61, 1.94)
CER200mgSC	0.91 (0.67, 1.24)	0.90 (0.65, 1.23)	0.89 (0.66, 1.20)	0.73 (0.47, 1.13)	0.89 (0.57, 1.40)	0.80 (0.61, 1.06)	0.76 (0.55, 1.05)	0.77 (0.57, 1.05)	1.05 (0.67, 1.67)	0.83 (0.54, 1.28)	0.80 (0.53, 1.26)	0.93 (0.62, 1.43)	0.88 (0.64, 1.21)	0.80 (0.57, 1.11)	0.86 (0.60, 1.23)	0.79 (0.57, 1.10)	0.82 (0.55, 1.21)	0.93 (0.65, 1.35)	0.97 (0.67, 1.40)	0.94 (0.71, 1.27)	0.44 (0.06, 1.44)	1.00 (0.80, 1.27)
CER20mgIV	1.00 (0.59, 1.73)	0.99 (0.57, 1.74)	0.98 (0.56, 1.69)	0.81 (0.44, 1.52)	0.98 (0.52, 1.86)	0.88 (0.52, 1.51)	0.84 (0.48, 1.46)	0.85 (0.50, 1.47)	1.16 (0.62, 2.23)	0.91 (0.49, 1.74)	0.88 (0.47, 1.68)	1.03 (0.56, 1.89)	0.96 (0.56, 1.68)	0.88 (0.51, 1.52)	0.95 (0.52, 1.68)	0.87 (0.50, 1.53)	0.90 (0.49, 1.63)	1.02 (0.58, 1.85)	1.07 (0.60, 1.91)	1.04 (0.61, 1.80)	0.49 (0.07, 1.56)	1.11 (0.64, 1.92)
CER400mgSC	1.01 (0.80, 1.31)	1.00 (0.76, 1.30)	0.99 (0.78, 1.28)	0.81 (0.54, 1.22)	0.99 (0.66, 1.51)	0.89 (0.73, 1.11)	0.85 (0.65, 1.12)	0.86 (0.67, 1.10)	1.17 (0.77, 1.79)	0.92 (0.62, 1.38)	0.89 (0.61, 1.34)	1.03 (0.73, 1.54)	0.97 (0.76, 1.27)	0.89 (0.67, 1.17)	0.95 (0.69, 1.31)	0.88 (0.67, 1.17)	0.91 (0.63, 1.31)	1.04 (0.75, 1.44)	1.08 (0.79, 1.50)	1.05 (0.83, 1.34)	0.49 (0.07, 1.61)	1.12 (0.90, 1.40)
CER5mgIV	0.90 (0.54, 1.49)	0.89 (0.52, 1.49)	0.88 (0.53, 1.45)	0.72 (0.40, 1.31)	0.89 (0.48, 1.63)	0.79 (0.48, 1.29)	0.76 (0.45, 1.27)	0.76 (0.46, 1.27)	1.04 (0.56, 1.92)	0.82 (0.46, 1.52)	0.80 (0.44, 1.43)	0.93 (0.52, 1.66)	0.87 (0.52, 1.45)	0.79 (0.47, 1.33)	0.85 (0.49, 1.44)	0.78 (0.46, 1.32)	0.81 (0.45, 1.42)	0.92 (0.54, 1.61)	0.96 (0.55, 1.66)	0.94 (0.56, 1.54)	0.44 (0.06, 1.36)	1.00 (0.60, 1.65)
ELD10mg_kgIV	1.23 (0.84, 1.84)	1.21 (0.82, 1.82)	1.20 (0.81, 1.77)	0.99 (0.60, 1.64)	1.20 (0.72, 2.01)	1.07 (0.76, 1.57)	1.03 (0.69, 1.56)	1.04 (0.71, 1.54)	1.42 (0.85, 2.39)	1.12 (0.68, 1.87)	1.09 (0.66, 1.79)	1.26 (0.78, 2.06)	1.18 (0.80, 1.77)	1.07 (0.72, 1.63)	1.15 (0.75, 1.79)	1.06 (0.71, 1.62)	1.10 (0.69, 1.78)	1.25 (0.81, 1.97)	1.30 (0.85, 2.05)	1.27 (0.87, 1.88)	0.59 (0.08, 2.07)	1.35 (0.92, 2.03)
ELD20mg_kgIV	1.37 (0.92, 2.09)	1.35 (0.90, 2.08)	1.34 (0.90, 2.03)	1.10 (0.66, 1.84)	1.35 (0.80, 2.29)	1.20 (0.83, 1.78)	1.14 (0.76, 1.77)	1.16 (0.78, 1.77)	1.57 (0.94, 2.69)	1.24 (0.76, 2.09)	1.21 (0.73, 2.03)	1.40 (0.86, 2.31)	1.32 (0.87, 2.03)	1.20 (0.78, 1.84)	1.29 (0.83, 2.05)	1.19 (0.78, 1.87)	1.23 (0.76, 2.02)	1.40 (0.89, 2.25)	1.45 (0.93, 2.34)	1.42 (0.96, 2.14)	0.66 (0.09, 2.31)	1.52 (1.00, 2.30)
ETA25mgSC	0.68 (0.35, 1.19)	0.67 (0.35, 1.19)	0.67 (0.34, 1.17)	0.55 (0.26, 1.05)	0.67 (0.32, 1.30)	0.60 (0.32, 1.03)	0.57 (0.29, 1.00)	0.58 (0.30, 1.01)	0.79 (0.38, 1.54)	0.62 (0.31, 1.19)	0.60 (0.30, 1.15)	0.70 (0.35, 1.32)	0.66 (0.34, 1.16)	0.60 (0.31, 1.05)	0.65 (0.32, 1.15)	0.59 (0.31, 1.05)	0.61 (0.31, 1.14)	0.70 (0.35, 1.27)	0.73 (0.37, 1.32)	0.71 (0.37, 1.23)	0.32 (0.04, 1.21)	0.76 (0.39, 1.32)
ETR105mgSC	0.81 (0.58, 1.14)	0.80 (0.56, 1.13)	0.80 (0.57, 1.10)	0.66 (0.41, 1.04)	0.80 (0.50, 1.28)	**0.71 (0.53, 0.97)**	**0.68 (0.47, 0.96)**	**0.69 (0.49, 0.96)**	0.94 (0.59, 1.53)	0.74 (0.48, 1.18)	0.72 (0.46, 1.15)	0.83 (0.54, 1.31)	0.79 (0.55, 1.10)	0.72 (0.49, 1.02)	0.77 (0.51, 1.11)	0.71 (0.49, 1.01)	0.73 (0.47, 1.11)	0.84 (0.55, 1.23)	0.87 (0.58, 1.28)	0.84 (0.60, 1.17)	0.39 (0.06, 1.32)	0.90 (0.64, 1.27)
ETR210mgSC	0.92 (0.65, 1.30)	0.91 (0.63, 1.30)	0.90 (0.64, 1.26)	0.74 (0.46, 1.17)	0.90 (0.57, 1.47)	0.81 (0.60, 1.11)	0.77 (0.54, 1.12)	0.78 (0.55, 1.11)	1.06 (0.66, 1.74)	0.84 (0.54, 1.35)	0.81 (0.52, 1.28)	0.94 (0.61, 1.49)	0.89 (0.63, 1.27)	0.81 (0.56, 1.16)	0.87 (0.59, 1.27)	0.80 (0.56, 1.15)	0.83 (0.54, 1.26)	0.95 (0.63, 1.42)	0.98 (0.66, 1.46)	0.96 (0.68, 1.33)	0.45 (0.06, 1.52)	1.02 (0.71, 1.44)
FIL100mgPO	1.03 (0.78, 1.37)	1.02 (0.75, 1.37)	1.01 (0.76, 1.34)	0.83 (0.54, 1.27)	1.01 (0.66, 1.58)	0.91 (0.71, 1.17)	0.87 (0.64, 1.18)	0.87 (0.66, 1.16)	1.19 (0.77, 1.86)	0.94 (0.62, 1.45)	0.91 (0.61, 1.41)	1.05 (0.71, 1.60)	1.00 (0.75, 1.35)	0.91 (0.66, 1.23)	0.97 (0.68, 1.37)	0.90 (0.66, 1.23)	0.93 (0.62, 1.37)	1.06 (0.74, 1.51)	1.10 (0.78, 1.56)	1.07 (0.81, 1.41)	0.50 (0.07, 1.65)	1.14 (0.85, 1.53)
FIL200mgPO	1.00 (0.76, 1.33)	0.99 (0.73, 1.32)	0.98 (0.74, 1.29)	0.80 (0.52, 1.23)	0.98 (0.64, 1.53)	0.87 (0.69, 1.13)	0.84 (0.61, 1.13)	0.85 (0.63, 1.13)	1.15 (0.74, 1.80)	0.91 (0.60, 1.39)	0.88 (0.59, 1.35)	1.02 (0.69, 1.55)	0.96 (0.72, 1.30)	0.88 (0.64, 1.19)	0.94 (0.66, 1.32)	0.87 (0.63, 1.19)	0.90 (0.60, 1.31)	1.03 (0.72, 1.46)	1.06 (0.75, 1.51)	1.03 (0.78, 1.36)	0.48 (0.07, 1.60)	1.10 (0.82, 1.48)
FON1mg_kg0_1mgIV_SC	1.17 (0.79, 1.73)	1.15 (0.76, 1.74)	1.13 (0.76, 1.69)	0.93 (0.57, 1.55)	1.15 (0.68, 1.94)	1.02 (0.70, 1.50)	0.97 (0.65, 1.46)	0.99 (0.67, 1.47)	1.34 (0.79, 2.29)	1.06 (0.65, 1.79)	1.03 (0.63, 1.73)	1.19 (0.74, 1.95)	1.12 (0.75, 1.69)	1.02 (0.67, 1.55)	1.10 (0.70, 1.71)	1.01 (0.67, 1.55)	1.05 (0.65, 1.71)	1.19 (0.77, 1.88)	1.24 (0.80, 1.95)	1.21 (0.82, 1.78)	0.56 (0.08, 1.95)	1.29 (0.86, 1.92)
FON1mg_kg1mgIV_SC	1.28 (0.85, 2.00)	1.26 (0.83, 1.99)	1.25 (0.83, 1.97)	1.03 (0.61, 1.78)	1.26 (0.74, 2.20)	1.13 (0.76, 1.73)	1.07 (0.71, 1.71)	1.08 (0.72, 1.71)	1.48 (0.86, 2.58)	1.17 (0.70, 2.01)	1.13 (0.69, 1.95)	1.31 (0.81, 2.22)	1.24 (0.81, 1.93)	1.12 (0.72, 1.77)	1.21 (0.76, 1.95)	1.11 (0.72, 1.77)	1.15 (0.71, 1.93)	1.32 (0.83, 2.15)	1.36 (0.86, 2.26)	1.33 (0.88, 2.05)	0.62 (0.09, 2.16)	1.42 (0.93, 2.23)
FON4mg_kg0_1mgIV_SC	0.99 (0.68, 1.44)	0.97 (0.66, 1.43)	0.97 (0.66, 1.40)	0.79 (0.49, 1.29)	0.97 (0.58, 1.61)	0.87 (0.60, 1.23)	0.83 (0.56, 1.21)	0.84 (0.57, 1.21)	1.14 (0.69, 1.91)	0.90 (0.56, 1.49)	0.87 (0.54, 1.44)	1.01 (0.64, 1.65)	0.95 (0.65, 1.39)	0.87 (0.58, 1.27)	0.93 (0.61, 1.40)	0.86 (0.58, 1.27)	0.88 (0.56, 1.40)	1.01 (0.66, 1.56)	1.05 (0.68, 1.62)	1.02 (0.70, 1.48)	0.47 (0.07, 1.63)	1.09 (0.74, 1.59)
FON4mg_kg1mgIV_SC	1.06 (0.73, 1.56)	1.04 (0.70, 1.56)	1.03 (0.71, 1.52)	0.85 (0.52, 1.40)	1.03 (0.62, 1.75)	0.92 (0.65, 1.35)	0.88 (0.60, 1.32)	0.89 (0.62, 1.32)	1.22 (0.73, 2.04)	0.96 (0.59, 1.58)	0.93 (0.58, 1.55)	1.08 (0.69, 1.77)	1.02 (0.69, 1.53)	0.93 (0.62, 1.40)	0.99 (0.65, 1.54)	0.92 (0.62, 1.38)	0.95 (0.59, 1.50)	1.08 (0.70, 1.68)	1.12 (0.73, 1.74)	1.09 (0.75, 1.60)	0.51 (0.07, 1.73)	1.16 (0.79, 1.74)
GUS1200mgIV	1.45 (0.96, 2.20)	1.42 (0.94, 2.22)	1.41 (0.93, 2.16)	1.16 (0.77, 1.78)	1.42 (0.85, 2.44)	1.26 (0.87, 1.90)	1.20 (0.80, 1.87)	1.22 (0.81, 1.88)	1.67 (0.97, 2.85)	1.32 (0.80, 2.25)	1.28 (0.77, 2.19)	1.48 (0.90, 2.51)	1.40 (0.92, 2.15)	1.26 (0.83, 1.97)	1.36 (0.85, 2.18)	1.26 (0.81, 1.99)	1.30 (0.80, 2.14)	1.48 (0.93, 2.39)	1.54 (0.97, 2.47)	1.49 (1.00, 2.28)	0.70 (0.10, 2.52)	**1.59 (1.05, 2.47)**
GUS200mgIV	1.40 (0.93, 2.11)	1.38 (0.90, 2.11)	1.36 (0.90, 2.04)	1.12 (0.74, 1.72)	1.37 (0.81, 2.36)	1.22 (0.84, 1.81)	1.17 (0.77, 1.81)	1.18 (0.78, 1.79)	1.62 (0.94, 2.74)	1.28 (0.76, 2.17)	1.23 (0.75, 2.09)	1.43 (0.88, 2.42)	1.35 (0.90, 2.04)	1.22 (0.80, 1.89)	1.32 (0.82, 2.09)	1.21 (0.78, 1.89)	1.25 (0.76, 2.03)	1.43 (0.91, 2.29)	1.49 (0.94, 2.37)	1.45 (0.96, 2.18)	0.68 (0.09, 2.31)	**1.54 (1.02, 2.36)**
GUS600mgIV	1.21 (0.82, 1.81)	1.19 (0.79, 1.80)	1.18 (0.79, 1.76)	0.97 (0.65, 1.43)	1.18 (0.71, 1.97)	1.06 (0.74, 1.53)	1.01 (0.68, 1.52)	1.02 (0.70, 1.52)	1.40 (0.82, 2.33)	1.10 (0.67, 1.83)	1.06 (0.66, 1.78)	1.23 (0.77, 2.05)	1.17 (0.79, 1.74)	1.06 (0.71, 1.60)	1.14 (0.73, 1.76)	1.05 (0.69, 1.60)	1.09 (0.68, 1.73)	1.23 (0.81, 1.96)	1.29 (0.83, 2.00)	1.25 (0.86, 1.86)	0.59 (0.08, 2.01)	1.33 (0.89, 2.01)
MED700IV	1.03 (0.72, 1.48)	1.02 (0.70, 1.47)	1.01 (0.71, 1.44)	0.83 (0.51, 1.34)	1.01 (0.63, 1.68)	0.90 (0.66, 1.26)	0.87 (0.59, 1.26)	0.87 (0.61, 1.25)	1.19 (0.74, 1.96)	0.94 (0.59, 1.53)	0.91 (0.57, 1.49)	1.05 (0.68, 1.69)	1.00 (0.69, 1.44)	0.91 (0.62, 1.32)	0.97 (0.65, 1.45)	0.90 (0.61, 1.32)	0.93 (0.59, 1.46)	1.06 (0.71, 1.60)	1.10 (0.72, 1.66)	1.07 (0.76, 1.52)	0.51 (0.07, 1.67)	1.14 (0.79, 1.63)
MIR1000mgIV	1.10 (0.77, 1.56)	1.08 (0.75, 1.56)	1.07 (0.75, 1.52)	0.88 (0.55, 1.43)	1.08 (0.67, 1.76)	0.97 (0.70, 1.33)	0.92 (0.63, 1.33)	0.93 (0.65, 1.32)	1.27 (0.78, 2.08)	1.00 (0.63, 1.60)	0.97 (0.62, 1.56)	1.12 (0.73, 1.77)	1.06 (0.74, 1.53)	0.96 (0.67, 1.39)	1.03 (0.70, 1.54)	0.95 (0.66, 1.38)	0.99 (0.63, 1.52)	1.13 (0.75, 1.69)	1.17 (0.79, 1.75)	1.14 (0.81, 1.61)	0.53 (0.07, 1.79)	1.22 (0.85, 1.74)
MIR200mgIV	1.27 (0.83, 1.98)	1.25 (0.81, 2.00)	1.24 (0.81, 1.95)	1.02 (0.60, 1.75)	1.25 (0.73, 2.21)	1.11 (0.75, 1.71)	1.06 (0.69, 1.72)	1.07 (0.72, 1.71)	1.46 (0.86, 2.61)	1.16 (0.69, 2.01)	1.12 (0.67, 1.94)	1.29 (0.80, 2.23)	1.23 (0.80, 1.97)	1.11 (0.72, 1.79)	1.20 (0.74, 1.96)	1.10 (0.71, 1.77)	1.14 (0.69, 1.91)	1.30 (0.82, 2.14)	1.35 (0.86, 2.20)	1.31 (0.88, 2.07)	0.60 (0.08, 2.16)	1.40 (0.91, 2.23)

	ABA10mg_kgIV	ABA30mg_kgIV	ABA3mg_kgIV	ADA160mg80mg40mgSC	ADA160mg80mg60mgSC	ADA160mg80mgSC	ADA40mg20mgSC	ADA80mg40mgSC	AMI0_4mgOR	AND150mgSC1_1	AND150mgSC2_2	AND300mgSC	API100mgOT	API50mgOR	BRI400mgIV	BRI700mgIV	BRO210mgIV	BRO350mgIV	BRO700mgIV	CDP10mg_kgIV	CER1_25mgIV	CER100mgSC
MIR600mgIV	1.11 (0.76, 1.69)	1.10 (0.73, 1.68)	1.09 (0.74, 1.66)	0.90 (0.54, 1.51)	1.09 (0.66, 1.85)	0.97 (0.68, 1.47)	0.93 (0.62, 1.45)	0.94 (0.64, 1.43)	1.29 (0.77, 2.21)	1.02 (0.63, 1.72)	0.98 (0.61, 1.67)	1.14 (0.72, 1.90)	1.07 (0.73, 1.66)	0.97 (0.66, 1.52)	1.05 (0.67, 1.64)	0.97 (0.64, 1.50)	1.01 (0.62, 1.63)	1.14 (0.74, 1.81)	1.18 (0.77, 1.88)	1.15 (0.79, 1.74)	0.54 (0.08, 1.86)	1.23 (0.83, 1.89)
NAT300mgIV	0.99 (0.78, 1.25)	0.98 (0.75, 1.26)	0.97 (0.77, 1.22)	0.80 (0.54, 1.18)	0.97 (0.65, 1.47)	0.87 (0.72, 1.06)	0.83 (0.64, 1.08)	0.84 (0.66, 1.07)	1.14 (0.76, 1.75)	0.90 (0.62, 1.35)	0.87 (0.60, 1.30)	1.01 (0.71, 1.50)	0.96 (0.75, 1.22)	0.87 (0.66, 1.13)	0.93 (0.68, 1.26)	0.86 (0.65, 1.13)	0.89 (0.62, 1.26)	1.02 (0.74, 1.40)	1.05 (0.77, 1.45)	1.02 (0.82, 1.30)	0.48 (0.07, 1.54)	1.09 (0.85, 1.41)
NAT3mg_kgIV	1.13 (0.82, 1.54)	1.11 (0.79, 1.54)	1.10 (0.79, 1.51)	0.90 (0.58, 1.41)	1.10 (0.69, 1.78)	0.99 (0.75, 1.32)	0.94 (0.67, 1.31)	0.95 (0.70, 1.31)	1.30 (0.82, 2.07)	1.02 (0.66, 1.61)	0.99 (0.64, 1.56)	1.15 (0.75, 1.79)	1.08 (0.78, 1.50)	0.99 (0.70, 1.38)	1.06 (0.72, 1.53)	0.97 (0.69, 1.37)	1.01 (0.66, 1.54)	1.15 (0.78, 1.70)	1.20 (0.81, 1.76)	1.17 (0.85, 1.60)	0.54 (0.08, 1.80)	1.24 (0.90, 1.72)
NAT3mg_kgIVx2	0.96 (0.70, 1.29)	0.95 (0.68, 1.29)	0.94 (0.69, 1.26)	0.77 (0.50, 1.20)	0.94 (0.60, 1.48)	0.84 (0.64, 1.10)	0.80 (0.58, 1.10)	0.81 (0.60, 1.10)	1.11 (0.70, 1.75)	0.87 (0.57, 1.36)	0.85 (0.55, 1.32)	0.98 (0.65, 1.50)	0.93 (0.68, 1.25)	0.84 (0.61, 1.15)	0.90 (0.63, 1.29)	0.83 (0.60, 1.15)	0.87 (0.57, 1.27)	0.99 (0.68, 1.42)	1.02 (0.70, 1.48)	0.99 (0.74, 1.33)	0.46 (0.07, 1.54)	1.06 (0.77, 1.43)
NAT6mg_kgIVx2	1.06 (0.77, 1.48)	1.05 (0.75, 1.46)	1.04 (0.75, 1.44)	0.85 (0.55, 1.35)	1.04 (0.65, 1.68)	0.93 (0.70, 1.27)	0.89 (0.63, 1.26)	0.90 (0.66, 1.25)	1.22 (0.77, 1.98)	0.97 (0.63, 1.53)	0.94 (0.60, 1.48)	1.09 (0.72, 1.69)	1.03 (0.74, 1.43)	0.93 (0.66, 1.31)	1.00 (0.68, 1.46)	0.92 (0.66, 1.32)	0.96 (0.63, 1.44)	1.09 (0.75, 1.61)	1.13 (0.77, 1.66)	1.10 (0.81, 1.52)	0.52 (0.07, 1.73)	1.18 (0.85, 1.63)
NNC2mg_kgSC	1.01 (0.68, 1.47)	0.99 (0.66, 1.46)	0.98 (0.66, 1.43)	0.81 (0.49, 1.34)	0.98 (0.59, 1.64)	0.88 (0.61, 1.26)	0.84 (0.55, 1.26)	0.85 (0.57, 1.24)	1.16 (0.69, 1.93)	0.91 (0.55, 1.51)	0.88 (0.54, 1.46)	1.02 (0.64, 1.66)	0.97 (0.65, 1.42)	0.88 (0.58, 1.31)	0.94 (0.61, 1.44)	0.87 (0.58, 1.30)	0.90 (0.56, 1.42)	1.02 (0.66, 1.60)	1.07 (0.68, 1.64)	1.04 (0.70, 1.51)	0.48 (0.07, 1.66)	1.11 (0.73, 1.64)
ONE10mgSC	0.98 (0.66, 1.45)	0.97 (0.64, 1.46)	0.96 (0.65, 1.41)	0.79 (0.48, 1.32)	0.97 (0.58, 1.63)	0.87 (0.60, 1.25)	0.83 (0.55, 1.23)	0.84 (0.57, 1.23)	1.14 (0.68, 1.95)	0.90 (0.55, 1.48)	0.87 (0.54, 1.43)	1.01 (0.62, 1.66)	0.95 (0.64, 1.40)	0.86 (0.57, 1.30)	0.93 (0.59, 1.43)	0.86 (0.56, 1.29)	0.89 (0.55, 1.42)	1.01 (0.64, 1.58)	1.05 (0.67, 1.64)	1.02 (0.69, 1.49)	0.48 (0.07, 1.63)	1.09 (0.73, 1.62)
ONE25mgSC	1.05 (0.69, 1.58)	1.03 (0.68, 1.58)	1.02 (0.68, 1.55)	0.84 (0.50, 1.42)	1.02 (0.62, 1.75)	0.92 (0.63, 1.37)	0.88 (0.58, 1.36)	0.89 (0.59, 1.34)	1.21 (0.71, 2.10)	0.96 (0.58, 1.61)	0.93 (0.56, 1.52)	1.08 (0.66, 1.80)	1.01 (0.67, 1.53)	0.92 (0.61, 1.41)	0.99 (0.63, 1.55)	0.91 (0.59, 1.40)	0.94 (0.58, 1.53)	1.07 (0.68, 1.69)	1.12 (0.70, 1.77)	1.09 (0.72, 1.63)	0.51 (0.07, 1.75)	1.16 (0.76, 1.77)
ONE35mgSC	0.93 (0.63, 1.36)	0.92 (0.61, 1.36)	0.91 (0.61, 1.32)	0.75 (0.45, 1.24)	0.92 (0.54, 1.51)	0.82 (0.56, 1.16)	0.78 (0.52, 1.16)	0.79 (0.54, 1.15)	1.08 (0.63, 1.80)	0.85 (0.52, 1.39)	0.82 (0.50, 1.34)	0.95 (0.59, 1.56)	0.90 (0.60, 1.31)	0.82 (0.54, 1.22)	0.88 (0.56, 1.34)	0.81 (0.53, 1.22)	0.84 (0.53, 1.33)	0.96 (0.62, 1.47)	0.99 (0.64, 1.53)	0.97 (0.66, 1.40)	0.45 (0.06, 1.54)	1.03 (0.68, 1.52)
ONE50mgSC	0.93 (0.63, 1.36)	0.92 (0.61, 1.37)	0.91 (0.61, 1.33)	0.75 (0.45, 1.23)	0.91 (0.55, 1.53)	0.82 (0.56, 1.16)	0.78 (0.52, 1.16)	0.79 (0.54, 1.15)	1.07 (0.64, 1.83)	0.85 (0.52, 1.39)	0.82 (0.51, 1.34)	0.96 (0.59, 1.56)	0.90 (0.61, 1.31)	0.82 (0.54, 1.21)	0.88 (0.56, 1.34)	0.81 (0.53, 1.20)	0.84 (0.53, 1.32)	0.95 (0.61, 1.48)	0.99 (0.63, 1.53)	0.96 (0.66, 1.39)	0.45 (0.06, 1.55)	1.03 (0.69, 1.52)
ONT22_5mgSC	1.02 (0.75, 1.36)	1.00 (0.73, 1.37)	0.99 (0.73, 1.32)	0.82 (0.53, 1.25)	1.00 (0.64, 1.56)	0.89 (0.69, 1.17)	0.85 (0.62, 1.16)	0.86 (0.64, 1.16)	1.17 (0.74, 1.83)	0.93 (0.60, 1.43)	0.89 (0.59, 1.39)	1.04 (0.70, 1.58)	0.98 (0.72, 1.33)	0.89 (0.64, 1.22)	0.96 (0.67, 1.34)	0.88 (0.64, 1.21)	0.91 (0.61, 1.35)	1.04 (0.73, 1.50)	1.08 (0.76, 1.54)	1.05 (0.79, 1.40)	0.49 (0.07, 1.61)	1.12 (0.82, 1.52)
ONT225mgSC	1.10 (0.81, 1.49)	1.09 (0.79, 1.49)	1.08 (0.79, 1.45)	0.89 (0.57, 1.37)	1.08 (0.70, 1.72)	0.97 (0.75, 1.28)	0.92 (0.67, 1.28)	0.94 (0.69, 1.26)	1.28 (0.81, 2.01)	1.01 (0.66, 1.57)	0.97 (0.64, 1.51)	1.13 (0.76, 1.73)	1.07 (0.78, 1.45)	0.97 (0.70, 1.33)	1.04 (0.72, 1.50)	0.96 (0.69, 1.35)	0.99 (0.66, 1.47)	1.13 (0.78, 1.64)	1.18 (0.82, 1.69)	1.14 (0.86, 1.54)	0.53 (0.08, 1.76)	1.22 (0.89, 1.67)
ONT75mgSC	1.12 (0.82, 1.52)	1.10 (0.79, 1.53)	1.09 (0.80, 1.49)	0.90 (0.57, 1.41)	1.09 (0.70, 1.73)	0.98 (0.75, 1.31)	0.93 (0.67, 1.31)	0.95 (0.70, 1.30)	1.28 (0.81, 2.03)	1.02 (0.66, 1.58)	0.98 (0.64, 1.54)	1.14 (0.76, 1.76)	1.08 (0.78, 1.48)	0.98 (0.70, 1.35)	1.05 (0.72, 1.52)	0.97 (0.69, 1.36)	1.00 (0.66, 1.50)	1.14 (0.79, 1.66)	1.19 (0.82, 1.71)	1.15 (0.86, 1.57)	0.54 (0.08, 1.79)	1.23 (0.90, 1.69)
PBO	1.02 (0.83, 1.26)	1.01 (0.80, 1.26)	1.00 (0.81, 1.23)	0.82 (0.57, 1.20)	1.00 (0.68, 1.50)	0.90 (0.77, 1.05)	0.86 (0.67, 1.08)	0.87 (0.70, 1.06)	1.18 (0.80, 1.78)	0.93 (0.65, 1.36)	0.90 (0.63, 1.32)	1.04 (0.75, 1.51)	0.99 (0.79, 1.23)	0.90 (0.70, 1.14)	0.96 (0.72, 1.27)	0.89 (0.70, 1.14)	0.92 (0.65, 1.28)	1.05 (0.78, 1.41)	1.09 (0.81, 1.46)	1.06 (0.88, 1.29)	0.49 (0.07, 1.60)	1.13 (0.90, 1.41)
PF10mgSC	1.04 (0.78, 1.37)	1.03 (0.75, 1.37)	1.02 (0.77, 1.35)	0.84 (0.54, 1.27)	1.02 (0.66, 1.58)	0.91 (0.72, 1.17)	0.87 (0.64, 1.17)	0.88 (0.67, 1.16)	1.20 (0.78, 1.88)	0.95 (0.63, 1.45)	0.91 (0.62, 1.40)	1.06 (0.73, 1.61)	1.01 (0.75, 1.34)	0.91 (0.67, 1.23)	0.98 (0.69, 1.38)	0.90 (0.66, 1.24)	0.94 (0.63, 1.36)	1.07 (0.76, 1.53)	1.11 (0.78, 1.56)	1.08 (0.82, 1.41)	0.50 (0.07, 1.67)	1.15 (0.86, 1.55)
PF200mgSC	1.14 (0.84, 1.58)	1.13 (0.81, 1.57)	1.12 (0.82, 1.53)	0.92 (0.59, 1.43)	1.12 (0.72, 1.78)	1.00 (0.78, 1.34)	0.96 (0.70, 1.33)	0.97 (0.72, 1.33)	1.32 (0.83, 2.13)	1.04 (0.69, 1.62)	1.01 (0.66, 1.58)	1.17 (0.79, 1.80)	1.10 (0.81, 1.52)	1.00 (0.72, 1.40)	1.08 (0.75, 1.55)	0.99 (0.71, 1.40)	1.03 (0.69, 1.53)	1.17 (0.81, 1.71)	1.22 (0.85, 1.77)	1.18 (0.89, 1.63)	0.55 (0.08, 1.81)	1.26 (0.92, 1.76)
PF50mgSC	1.15 (0.86, 1.52)	1.13 (0.82, 1.52)	1.12 (0.83, 1.49)	0.92 (0.60, 1.41)	1.12 (0.72, 1.75)	1.00 (0.79, 1.31)	0.96 (0.70, 1.30)	0.97 (0.73, 1.29)	1.31 (0.85, 2.08)	1.04 (0.69, 1.61)	1.00 (0.67, 1.56)	1.17 (0.79, 1.77)	1.10 (0.82, 1.48)	1.00 (0.73, 1.37)	1.08 (0.75, 1.52)	0.99 (0.72, 1.36)	1.03 (0.69, 1.51)	1.18 (0.82, 1.68)	1.22 (0.86, 1.73)	1.18 (0.89, 1.58)	0.55 (0.08, 1.84)	1.26 (0.94, 1.70)
RIS1200mgIV	1.14 (0.88, 1.47)	1.12 (0.85, 1.47)	1.11 (0.86, 1.43)	0.92 (0.61, 1.37)	1.12 (0.74, 1.71)	1.00 (0.81, 1.24)	0.95 (0.72, 1.25)	0.97 (0.75, 1.24)	1.31 (0.87, 2.02)	1.04 (0.70, 1.56)	1.00 (0.68, 1.51)	1.16 (0.81, 1.72)	1.10 (0.84, 1.44)	1.00 (0.75, 1.32)	1.07 (0.77, 1.47)	0.99 (0.74, 1.32)	1.03 (0.70, 1.49)	1.17 (0.83, 1.63)	1.21 (0.87, 1.68)	1.18 (0.92, 1.50)	0.55 (0.08, 1.80)	1.26 (0.96, 1.63)
RIS200mgIV	1.11 (0.81, 1.54)	1.09 (0.78, 1.54)	1.08 (0.79, 1.50)	0.89 (0.56, 1.40)	1.09 (0.69, 1.75)	0.97 (0.73, 1.32)	0.93 (0.66, 1.32)	0.94 (0.68, 1.30)	1.29 (0.80, 2.06)	1.01 (0.65, 1.60)	0.98 (0.63, 1.54)	1.13 (0.75, 1.77)	1.07 (0.77, 1.51)	0.97 (0.69, 1.39)	1.04 (0.71, 1.54)	0.96 (0.68, 1.37)	1.00 (0.65, 1.50)	1.14 (0.78, 1.68)	1.18 (0.81, 1.75)	1.15 (0.84, 1.59)	0.54 (0.08, 1.79)	1.23 (0.88, 1.72)
RIS600mgIV	1.17 (0.92, 1.50)	1.15 (0.88, 1.49)	1.14 (0.89, 1.46)	0.94 (0.63, 1.40)	1.15 (0.76, 1.74)	1.02 (0.84, 1.27)	0.98 (0.75, 1.28)	0.99 (0.78, 1.26)	1.35 (0.89, 2.06)	1.06 (0.73, 1.61)	1.03 (0.71, 1.55)	1.19 (0.84, 1.78)	1.13 (0.88, 1.46)	1.02 (0.78, 1.34)	1.10 (0.80, 1.51)	1.01 (0.77, 1.35)	1.05 (0.72, 1.50)	1.20 (0.87, 1.66)	1.25 (0.90, 1.71)	1.21 (0.96, 1.54)	0.57 (0.08, 1.87)	1.29 (0.99, 1.67)
RIS600mgIV4_4	1.47 (0.47, 4.97)	1.45 (0.46, 4.95)	1.43 (0.46, 4.83)	1.19 (0.37, 3.92)	1.44 (0.44, 5.09)	1.30 (0.42, 4.34)	1.24 (0.39, 4.17)	1.24 (0.40, 4.18)	1.70 (0.53, 6.00)	1.35 (0.41, 4.62)	1.31 (0.39, 4.58)	1.51 (0.46, 5.24)	1.42 (0.45, 4.74)	1.29 (0.41, 4.32)	1.38 (0.44, 4.72)	1.28 (0.41, 4.36)	1.32 (0.41, 4.59)	1.51 (0.48, 5.21)	1.55 (0.49, 5.30)	1.52 (0.49, 5.09)	0.69 (0.07, 3.92)	1.63 (0.52, 5.46)
SEC10mg_kgIV	0.67 (0.37, 1.10)	0.66 (0.35, 1.09)	0.65 (0.35, 1.08)	**0.54 (0.27, 0.98)**	0.66 (0.33, 1.22)	**0.59 (0.32, 0.95)**	**0.56 (0.30, 0.94)**	**0.57 (0.31, 0.93)**	0.78 (0.39, 1.41)	0.61 (0.31, 1.12)	0.60 (0.30, 1.07)	0.69 (0.35, 1.22)	0.65 (0.35, 1.07)	**0.59 (0.31, 0.99)**	0.63 (0.33, 1.08)	**0.58 (0.31, 0.98)**	0.60 (0.30, 1.06)	0.69 (0.36, 1.20)	0.72 (0.37, 1.23)	0.70 (0.38, 1.13)	0.32 (0.04, 1.17)	0.74 (0.40, 1.24)

	ABA10mg_kgIV	ABA30mg_kgIV	ABA3mg_kgIV	ADA160mg80mg40mgSC	ADA160mg80mg60mgSC	ADA160mg80mgSC	ADA40mg20mgSC	ADA80mg40mgSC	AMI0_4mgOR	AND150mgSC1_1	AND150mgSC2_2	AND300mgSC	API100mgOT	API50mgOR	BRI400mgIV	BRI700mgIV	BRO210mgIV	BRO350mgIV	BRO700mgIV	CDP10mg_kgIV	CER1_25mgIV	CER100mgSC
SEM60mgIV	0.89 (0.58, 1.34)	0.87 (0.57, 1.33)	0.87 (0.57, 1.30)	0.71 (0.42, 1.22)	0.87 (0.52, 1.49)	0.78 (0.52, 1.16)	0.74 (0.48, 1.14)	0.75 (0.49, 1.14)	1.03 (0.59, 1.77)	0.81 (0.48, 1.38)	0.78 (0.47, 1.33)	0.91 (0.55, 1.51)	0.85 (0.56, 1.30)	0.78 (0.50, 1.19)	0.84 (0.53, 1.31)	0.77 (0.50, 1.18)	0.80 (0.49, 1.29)	0.91 (0.57, 1.44)	0.94 (0.59, 1.50)	0.92 (0.61, 1.38)	0.42 (0.06, 1.51)	0.98 (0.64, 1.48)
SEM60mgIVx3	0.79 (0.53, 1.16)	0.78 (0.51, 1.16)	0.77 (0.52, 1.13)	0.63 (0.38, 1.04)	0.77 (0.46, 1.30)	**0.69 (0.48, 0.99)**	**0.66 (0.44, 0.99)**	**0.67 (0.45, 0.98)**	0.91 (0.54, 1.53)	0.72 (0.43, 1.19)	0.70 (0.42, 1.16)	0.80 (0.50, 1.32)	0.76 (0.51, 1.12)	0.69 (0.45, 1.03)	0.74 (0.47, 1.13)	0.68 (0.45, 1.03)	0.71 (0.44, 1.13)	0.81 (0.51, 1.26)	0.84 (0.53, 1.31)	0.82 (0.55, 1.20)	0.38 (0.05, 1.31)	0.87 (0.57, 1.29)
TES400mg200mgSC	0.80 (0.55, 1.19)	0.79 (0.53, 1.17)	0.78 (0.53, 1.15)	0.65 (0.40, 1.06)	0.79 (0.48, 1.33)	0.70 (0.49, 1.01)	0.67 (0.44, 1.00)	0.68 (0.46, 1.01)	0.93 (0.55, 1.57)	0.73 (0.45, 1.22)	0.70 (0.43, 1.17)	0.82 (0.51, 1.34)	0.77 (0.52, 1.15)	0.70 (0.47, 1.05)	0.76 (0.49, 1.16)	0.69 (0.46, 1.05)	0.72 (0.45, 1.14)	0.82 (0.53, 1.27)	0.85 (0.55, 1.32)	0.83 (0.57, 1.22)	0.39 (0.05, 1.32)	0.89 (0.59, 1.31)
TOF15mgOR_BID	1.06 (0.68, 1.68)	1.05 (0.65, 1.65)	1.04 (0.66, 1.63)	0.86 (0.49, 1.47)	1.04 (0.60, 1.80)	0.93 (0.60, 1.43)	0.89 (0.55, 1.42)	0.90 (0.57, 1.40)	1.22 (0.69, 2.19)	0.97 (0.56, 1.71)	0.94 (0.55, 1.65)	1.09 (0.64, 1.88)	1.03 (0.65, 1.63)	0.94 (0.58, 1.47)	1.00 (0.61, 1.61)	0.92 (0.58, 1.49)	0.95 (0.56, 1.61)	1.09 (0.66, 1.78)	1.13 (0.69, 1.87)	1.10 (0.70, 1.73)	0.51 (0.07, 1.78)	1.18 (0.75, 1.83)
TOF1mgOR_BID	1.33 (0.81, 2.19)	1.31 (0.79, 2.17)	1.30 (0.79, 2.13)	1.07 (0.59, 1.93)	1.31 (0.72, 2.40)	1.16 (0.72, 1.90)	1.12 (0.67, 1.84)	1.13 (0.68, 1.84)	1.52 (0.84, 2.80)	1.22 (0.68, 2.19)	1.17 (0.66, 2.15)	1.37 (0.78, 2.44)	1.28 (0.78, 2.12)	1.17 (0.70, 1.93)	1.26 (0.74, 2.10)	1.15 (0.69, 1.91)	1.20 (0.68, 2.07)	1.36 (0.80, 2.33)	1.42 (0.83, 2.40)	1.38 (0.85, 2.23)	0.63 (0.09, 2.31)	1.46 (0.89, 2.42)
TOF5mgOR_BID	1.08 (0.69, 1.70)	1.06 (0.67, 1.69)	1.06 (0.67, 1.65)	0.87 (0.49, 1.51)	1.06 (0.61, 1.88)	0.95 (0.62, 1.46)	0.90 (0.57, 1.43)	0.92 (0.58, 1.44)	1.25 (0.71, 2.20)	0.99 (0.57, 1.70)	0.96 (0.56, 1.65)	1.11 (0.65, 1.89)	1.05 (0.66, 1.65)	0.95 (0.59, 1.51)	1.02 (0.62, 1.65)	0.94 (0.58, 1.48)	0.97 (0.57, 1.62)	1.11 (0.67, 1.81)	1.15 (0.70, 1.85)	1.12 (0.71, 1.76)	0.52 (0.07, 1.85)	1.20 (0.75, 1.87)
UPA12mgOR_BID	0.92 (0.63, 1.34)	0.91 (0.61, 1.34)	0.90 (0.61, 1.31)	0.74 (0.45, 1.21)	0.91 (0.54, 1.48)	0.81 (0.57, 1.15)	0.77 (0.52, 1.15)	0.78 (0.54, 1.14)	1.07 (0.65, 1.76)	0.84 (0.53, 1.36)	0.81 (0.50, 1.33)	0.94 (0.60, 1.52)	0.89 (0.61, 1.29)	0.81 (0.54, 1.19)	0.86 (0.57, 1.32)	0.80 (0.54, 1.19)	0.83 (0.52, 1.29)	0.95 (0.61, 1.46)	0.99 (0.64, 1.49)	0.95 (0.66, 1.38)	0.45 (0.06, 1.53)	1.02 (0.70, 1.49)
UPA24mgOR	0.90 (0.62, 1.29)	0.89 (0.60, 1.29)	0.88 (0.61, 1.25)	0.72 (0.44, 1.17)	0.89 (0.54, 1.44)	0.79 (0.56, 1.12)	0.75 (0.51, 1.10)	0.76 (0.52, 1.09)	1.04 (0.63, 1.72)	0.82 (0.51, 1.33)	0.79 (0.50, 1.29)	0.92 (0.59, 1.47)	0.87 (0.59, 1.25)	0.79 (0.53, 1.15)	0.85 (0.56, 1.28)	0.78 (0.53, 1.14)	0.81 (0.51, 1.25)	0.92 (0.60, 1.42)	0.96 (0.62, 1.45)	0.93 (0.65, 1.33)	0.43 (0.06, 1.49)	0.99 (0.68, 1.44)
UPA24mgOR_BID	0.89 (0.62, 1.28)	0.88 (0.59, 1.29)	0.87 (0.60, 1.26)	0.72 (0.44, 1.16)	0.87 (0.53, 1.44)	0.78 (0.55, 1.11)	0.74 (0.50, 1.09)	0.75 (0.52, 1.09)	1.03 (0.63, 1.71)	0.81 (0.51, 1.32)	0.78 (0.49, 1.28)	0.91 (0.58, 1.46)	0.86 (0.59, 1.25)	0.78 (0.53, 1.15)	0.84 (0.55, 1.27)	0.77 (0.52, 1.14)	0.80 (0.51, 1.25)	0.91 (0.60, 1.40)	0.95 (0.62, 1.45)	0.92 (0.64, 1.33)	0.43 (0.06, 1.47)	0.99 (0.68, 1.43)
UPA3mgOR_BID	0.85 (0.59, 1.22)	0.84 (0.58, 1.22)	0.83 (0.57, 1.19)	0.68 (0.42, 1.11)	0.84 (0.51, 1.35)	0.75 (0.53, 1.04)	0.71 (0.48, 1.04)	0.72 (0.50, 1.02)	0.98 (0.60, 1.62)	0.77 (0.49, 1.27)	0.75 (0.47, 1.22)	0.87 (0.56, 1.41)	0.82 (0.56, 1.20)	0.74 (0.51, 1.09)	0.80 (0.53, 1.20)	0.74 (0.50, 1.08)	0.77 (0.48, 1.17)	0.87 (0.57, 1.32)	0.91 (0.59, 1.38)	0.88 (0.62, 1.24)	0.41 (0.06, 1.41)	0.94 (0.65, 1.34)
UPA45mgOR	0.97 (0.75, 1.24)	0.96 (0.72, 1.25)	0.94 (0.73, 1.22)	0.78 (0.52, 1.16)	0.95 (0.63, 1.44)	0.85 (0.69, 1.06)	0.81 (0.61, 1.07)	0.82 (0.64, 1.05)	1.12 (0.73, 1.71)	0.88 (0.60, 1.33)	0.85 (0.58, 1.28)	0.99 (0.69, 1.48)	0.93 (0.72, 1.22)	0.85 (0.64, 1.12)	0.91 (0.66, 1.24)	0.84 (0.63, 1.12)	0.87 (0.60, 1.25)	0.99 (0.71, 1.38)	1.03 (0.75, 1.43)	1.00 (0.79, 1.28)	0.47 (0.07, 1.54)	1.07 (0.82, 1.39)
UPA6mgOR_BID	0.95 (0.65, 1.37)	0.94 (0.63, 1.37)	0.93 (0.64, 1.35)	0.76 (0.47, 1.24)	0.94 (0.57, 1.53)	0.84 (0.58, 1.20)	0.80 (0.54, 1.18)	0.80 (0.55, 1.17)	1.09 (0.67, 1.82)	0.86 (0.54, 1.42)	0.84 (0.52, 1.38)	0.97 (0.62, 1.58)	0.92 (0.62, 1.35)	0.83 (0.55, 1.23)	0.89 (0.59, 1.36)	0.83 (0.56, 1.22)	0.86 (0.53, 1.35)	0.97 (0.64, 1.51)	1.01 (0.65, 1.56)	0.99 (0.68, 1.41)	0.45 (0.06, 1.59)	1.05 (0.72, 1.53)
UST130mgIV	1.07 (0.83, 1.38)	1.06 (0.80, 1.38)	1.04 (0.81, 1.35)	0.86 (0.58, 1.29)	1.05 (0.69, 1.61)	0.94 (0.77, 1.17)	0.90 (0.68, 1.18)	0.91 (0.71, 1.16)	1.24 (0.81, 1.91)	0.97 (0.66, 1.46)	0.94 (0.65, 1.43)	1.09 (0.76, 1.62)	1.03 (0.80, 1.36)	0.94 (0.71, 1.24)	1.01 (0.73, 1.38)	0.93 (0.70, 1.24)	0.96 (0.67, 1.39)	1.10 (0.79, 1.52)	1.14 (0.82, 1.57)	1.11 (0.87, 1.41)	0.52 (0.07, 1.69)	1.18 (0.91, 1.54)
UST1mg_kgIV	1.01 (0.76, 1.35)	0.99 (0.73, 1.35)	0.98 (0.74, 1.32)	0.81 (0.53, 1.25)	0.99 (0.63, 1.55)	0.88 (0.69, 1.15)	0.84 (0.62, 1.15)	0.85 (0.64, 1.13)	1.16 (0.75, 1.84)	0.92 (0.61, 1.40)	0.88 (0.59, 1.37)	1.03 (0.70, 1.56)	0.97 (0.72, 1.32)	0.88 (0.64, 1.21)	0.95 (0.67, 1.34)	0.88 (0.64, 1.20)	0.91 (0.61, 1.33)	1.03 (0.73, 1.47)	1.07 (0.76, 1.54)	1.04 (0.79, 1.38)	0.49 (0.07, 1.61)	1.11 (0.82, 1.50)
UST3mg_kgIV	1.05 (0.78, 1.40)	1.03 (0.75, 1.41)	1.02 (0.76, 1.37)	0.84 (0.55, 1.29)	1.03 (0.66, 1.61)	0.92 (0.72, 1.19)	0.88 (0.65, 1.20)	0.89 (0.66, 1.18)	1.21 (0.78, 1.90)	0.95 (0.63, 1.47)	0.92 (0.61, 1.43)	1.07 (0.73, 1.62)	1.01 (0.75, 1.36)	0.92 (0.67, 1.27)	0.99 (0.69, 1.39)	0.91 (0.66, 1.25)	0.94 (0.63, 1.38)	1.07 (0.75, 1.54)	1.11 (0.78, 1.60)	1.08 (0.82, 1.45)	0.51 (0.07, 1.67)	1.16 (0.85, 1.56)
UST4_5mg_kgIV	0.94 (0.60, 1.43)	0.92 (0.59, 1.41)	0.91 (0.59, 1.40)	0.75 (0.44, 1.27)	0.92 (0.54, 1.59)	0.82 (0.54, 1.22)	0.78 (0.50, 1.22)	0.79 (0.51, 1.21)	1.08 (0.63, 1.86)	0.86 (0.50, 1.47)	0.82 (0.49, 1.41)	0.96 (0.58, 1.61)	0.90 (0.58, 1.40)	0.82 (0.52, 1.28)	0.88 (0.55, 1.41)	0.81 (0.52, 1.27)	0.84 (0.50, 1.38)	0.96 (0.59, 1.55)	1.00 (0.62, 1.61)	0.97 (0.63, 1.49)	0.45 (0.06, 1.58)	1.04 (0.66, 1.60)
UST6mg_kg90mgIV_SC	1.21 (0.82, 1.80)	1.19 (0.80, 1.79)	1.18 (0.79, 1.76)	0.97 (0.81, 1.17)	1.19 (0.72, 2.01)	1.06 (0.74, 1.54)	1.01 (0.68, 1.52)	1.02 (0.70, 1.52)	1.40 (0.83, 2.33)	1.10 (0.67, 1.82)	1.07 (0.65, 1.78)	1.24 (0.77, 2.06)	1.17 (0.79, 1.74)	1.06 (0.70, 1.60)	1.14 (0.72, 1.77)	1.05 (0.69, 1.60)	1.09 (0.67, 1.73)	1.24 (0.79, 1.96)	1.29 (0.83, 1.99)	1.25 (0.85, 1.83)	0.59 (0.08, 2.02)	1.33 (0.90, 2.00)
UST6mg_kgIV	1.06 (0.82, 1.34)	1.04 (0.80, 1.35)	1.03 (0.81, 1.31)	0.85 (0.57, 1.27)	1.03 (0.69, 1.57)	0.92 (0.76, 1.14)	0.88 (0.67, 1.15)	0.89 (0.70, 1.13)	1.21 (0.81, 1.86)	0.96 (0.65, 1.43)	0.92 (0.64, 1.39)	1.08 (0.76, 1.60)	1.01 (0.79, 1.32)	0.92 (0.70, 1.21)	0.99 (0.72, 1.35)	0.91 (0.70, 1.20)	0.95 (0.66, 1.35)	1.08 (0.79, 1.49)	1.12 (0.82, 1.54)	1.09 (0.87, 1.39)	0.51 (0.07, 1.66)	1.16 (0.91, 1.50)
UST90mgSC	1.27 (0.85, 1.98)	1.26 (0.83, 1.95)	1.24 (0.83, 1.92)	1.02 (0.61, 1.74)	1.25 (0.75, 2.19)	1.11 (0.77, 1.69)	1.06 (0.70, 1.67)	1.08 (0.73, 1.66)	1.47 (0.87, 2.55)	1.16 (0.71, 1.99)	1.13 (0.68, 1.93)	1.31 (0.80, 2.21)	1.23 (0.82, 1.92)	1.11 (0.73, 1.75)	1.21 (0.76, 1.90)	1.10 (0.73, 1.72)	1.15 (0.71, 1.88)	1.30 (0.83, 2.10)	1.36 (0.87, 2.18)	1.32 (0.90, 2.01)	0.62 (0.09, 2.13)	1.41 (0.94, 2.18)
VED0_5mg_kgIV	0.94 (0.70, 1.25)	0.93 (0.68, 1.25)	0.92 (0.68, 1.22)	0.76 (0.49, 1.16)	0.92 (0.60, 1.43)	0.83 (0.64, 1.06)	0.79 (0.57, 1.06)	0.80 (0.60, 1.06)	1.09 (0.69, 1.70)	0.85 (0.56, 1.31)	0.83 (0.55, 1.29)	0.96 (0.65, 1.46)	0.91 (0.67, 1.22)	0.82 (0.60, 1.13)	0.89 (0.62, 1.24)	0.82 (0.59, 1.12)	0.85 (0.57, 1.24)	0.97 (0.68, 1.38)	1.00 (0.70, 1.42)	0.98 (0.73, 1.28)	0.46 (0.07, 1.48)	1.04 (0.76, 1.40)
VED2mg_kgIV	0.97 (0.72, 1.29)	0.96 (0.69, 1.30)	0.95 (0.71, 1.26)	0.78 (0.51, 1.19)	0.95 (0.62, 1.48)	0.85 (0.66, 1.10)	0.81 (0.59, 1.10)	0.82 (0.61, 1.09)	1.12 (0.72, 1.76)	0.88 (0.58, 1.36)	0.85 (0.57, 1.33)	0.99 (0.67, 1.50)	0.93 (0.69, 1.26)	0.85 (0.62, 1.16)	0.91 (0.64, 1.29)	0.84 (0.61, 1.16)	0.87 (0.58, 1.28)	1.00 (0.69, 1.42)	1.03 (0.72, 1.46)	1.00 (0.75, 1.33)	0.47 (0.07, 1.56)	1.07 (0.80, 1.44)
VED300mgIV	1.04 (0.81, 1.34)	1.03 (0.78, 1.34)	1.02 (0.79, 1.30)	0.84 (0.56, 1.26)	1.02 (0.67, 1.56)	0.92 (0.74, 1.13)	0.88 (0.66, 1.14)	0.88 (0.69, 1.13)	1.20 (0.79, 1.85)	0.95 (0.64, 1.42)	0.92 (0.63, 1.38)	1.07 (0.74, 1.57)	1.01 (0.77, 1.30)	0.91 (0.69, 1.20)	0.99 (0.71, 1.33)	0.90 (0.68, 1.21)	0.94 (0.65, 1.35)	1.07 (0.77, 1.48)	1.11 (0.81, 1.54)	1.08 (0.85, 1.37)	0.51 (0.07, 1.66)	1.15 (0.88, 1.50)

	CER10mgIV	CER200mgSC	CER20mgIV	CER400mgSC	CER5mgIV	ELD10mg_kgIV	ELD20mg_kgIV	ETA25mgSC	ETR105mgSC	ETR210mgSC	FIL100mgPO	FIL200mgPO	FON1mg_kg0_1mgIV_SC	FON1mg_kg1mgIV_SC	FON4mg_kg0_1mgIV_SC	FON4mg_kg1mgIV_SC	GUS1200mgIV	GUS200mgIV	GUS600mgIV	MED700IV	MIR1000mgIV	MIR200mgIV
ABA10mg_kgIV	1.05 (0.57, 1.79)	1.10 (0.81, 1.48)	1.00 (0.58, 1.70)	0.99 (0.76, 1.26)	1.11 (0.67, 1.84)	0.82 (0.54, 1.19)	0.73 (0.48, 1.09)	1.46 (0.84, 2.84)	1.23 (0.88, 1.74)	1.08 (0.77, 1.53)	0.97 (0.73, 1.28)	1.00 (0.75, 1.32)	0.86 (0.58, 1.27)	0.78 (0.50, 1.17)	1.01 (0.69, 1.47)	0.95 (0.64, 1.37)	0.69 (0.45, 1.04)	0.71 (0.47, 1.07)	0.83 (0.55, 1.23)	0.97 (0.67, 1.38)	0.91 (0.64, 1.30)	0.79 (0.50, 1.20)
ABA30mg_kgIV	1.06 (0.58, 1.86)	1.11 (0.81, 1.55)	1.01 (0.58, 1.75)	1.00 (0.77, 1.31)	1.12 (0.67, 1.91)	0.83 (0.55, 1.23)	0.74 (0.48, 1.12)	1.49 (0.84, 2.89)	1.24 (0.88, 1.78)	1.10 (0.77, 1.59)	0.98 (0.73, 1.33)	1.01 (0.76, 1.37)	0.87 (0.58, 1.31)	0.79 (0.50, 1.20)	1.03 (0.70, 1.52)	0.96 (0.64, 1.42)	0.70 (0.45, 1.06)	0.72 (0.47, 1.11)	0.84 (0.55, 1.26)	0.98 (0.68, 1.42)	0.92 (0.64, 1.33)	0.80 (0.50, 1.23)
ABA3mg_kgIV	1.07 (0.59, 1.86)	1.13 (0.83, 1.51)	1.02 (0.59, 1.77)	1.01 (0.78, 1.29)	1.13 (0.69, 1.89)	0.84 (0.56, 1.24)	0.75 (0.49, 1.12)	1.50 (0.86, 2.90)	1.26 (0.91, 1.77)	1.11 (0.79, 1.56)	0.99 (0.74, 1.31)	1.03 (0.78, 1.36)	0.88 (0.59, 1.31)	0.80 (0.51, 1.20)	1.04 (0.71, 1.51)	0.97 (0.66, 1.41)	0.71 (0.46, 1.07)	0.73 (0.49, 1.11)	0.85 (0.57, 1.26)	0.99 (0.70, 1.42)	0.93 (0.66, 1.33)	0.81 (0.51, 1.23)
ADA160mg80mg40mgSC	1.30 (0.66, 2.41)	1.37 (0.88, 2.13)	1.24 (0.66, 2.29)	1.23 (0.82, 1.84)	1.38 (0.77, 2.52)	1.01 (0.61, 1.66)	0.91 (0.54, 1.53)	1.82 (0.95, 3.78)	1.53 (0.96, 2.44)	1.35 (0.85, 2.16)	1.20 (0.79, 1.86)	1.25 (0.81, 1.92)	1.07 (0.64, 1.77)	0.97 (0.56, 1.65)	1.26 (0.77, 2.05)	1.18 (0.71, 1.92)	0.86 (0.56, 1.31)	0.89 (0.58, 1.36)	1.03 (0.70, 1.53)	1.20 (0.74, 1.97)	1.14 (0.70, 1.81)	0.98 (0.57, 1.66)
ADA160mg80mg60mgSC	1.07 (0.53, 1.99)	1.12 (0.71, 1.76)	1.02 (0.54, 1.91)	1.01 (0.66, 1.51)	1.13 (0.61, 2.09)	0.83 (0.50, 1.39)	0.74 (0.44, 1.25)	1.48 (0.77, 3.13)	1.25 (0.78, 2.00)	1.11 (0.68, 1.77)	0.99 (0.63, 1.51)	1.02 (0.65, 1.57)	0.87 (0.52, 1.47)	0.79 (0.45, 1.36)	1.03 (0.62, 1.72)	0.97 (0.57, 1.60)	0.70 (0.41, 1.18)	0.73 (0.42, 1.23)	0.84 (0.51, 1.41)	0.99 (0.59, 1.59)	0.93 (0.57, 1.50)	0.80 (0.45, 1.36)
ADA160mg80mgSC	1.19 (0.67, 1.99)	1.26 (0.94, 1.64)	1.13 (0.66, 1.92)	1.13 (0.90, 1.38)	1.26 (0.77, 2.08)	0.93 (0.64, 1.32)	0.83 (0.56, 1.21)	1.66 (0.97, 3.15)	**1.40 (1.03, 1.90)**	1.24 (0.90, 1.68)	1.10 (0.86, 1.40)	1.14 (0.88, 1.45)	0.98 (0.67, 1.42)	0.89 (0.58, 1.31)	1.15 (0.81, 1.65)	1.08 (0.74, 1.54)	0.79 (0.53, 1.15)	0.82 (0.55, 1.19)	0.94 (0.65, 1.35)	1.11 (0.79, 1.52)	1.04 (0.75, 1.42)	0.90 (0.58, 1.33)
ADA40mg20mgSC	1.26 (0.68, 2.14)	1.31 (0.95, 1.81)	1.19 (0.68, 2.10)	1.18 (0.89, 1.54)	1.32 (0.79, 2.23)	0.97 (0.64, 1.45)	0.88 (0.56, 1.32)	1.74 (1.00, 3.39)	**1.46 (1.04, 2.11)**	1.30 (0.90, 1.86)	1.15 (0.85, 1.57)	1.20 (0.88, 1.63)	1.03 (0.68, 1.55)	0.93 (0.59, 1.41)	1.21 (0.82, 1.80)	1.13 (0.76, 1.67)	0.83 (0.53, 1.26)	0.86 (0.55, 1.30)	0.99 (0.66, 1.48)	1.15 (0.79, 1.69)	1.09 (0.75, 1.58)	0.94 (0.58, 1.45)
ADA80mg40mgSC	1.24 (0.68, 2.09)	1.30 (0.95, 1.75)	1.18 (0.68, 2.02)	1.17 (0.91, 1.49)	1.31 (0.79, 2.19)	0.96 (0.65, 1.42)	0.86 (0.57, 1.29)	1.72 (0.99, 3.31)	**1.45 (1.04, 2.04)**	1.28 (0.90, 1.81)	1.14 (0.86, 1.51)	1.18 (0.89, 1.58)	1.02 (0.68, 1.50)	0.92 (0.59, 1.39)	1.20 (0.83, 1.75)	1.12 (0.76, 1.62)	0.82 (0.53, 1.23)	0.85 (0.56, 1.27)	0.98 (0.66, 1.43)	1.15 (0.80, 1.63)	1.08 (0.75, 1.53)	0.93 (0.59, 1.40)
AMI0_4mgOR	0.91 (0.46, 1.69)	0.95 (0.60, 1.50)	0.86 (0.45, 1.61)	0.86 (0.56, 1.29)	0.96 (0.52, 1.77)	0.70 (0.42, 1.17)	0.64 (0.37, 1.07)	1.27 (0.65, 2.66)	1.07 (0.65, 1.70)	0.94 (0.57, 1.51)	0.84 (0.54, 1.30)	0.87 (0.55, 1.35)	0.74 (0.44, 1.27)	0.67 (0.39, 1.16)	0.88 (0.52, 1.45)	0.82 (0.49, 1.36)	0.60 (0.35, 1.03)	0.62 (0.36, 1.06)	0.72 (0.43, 1.22)	0.84 (0.51, 1.36)	0.79 (0.48, 1.29)	0.68 (0.38, 1.17)
AND150mgSC1_1	1.15 (0.58, 2.11)	1.21 (0.78, 1.85)	1.10 (0.58, 2.03)	1.09 (0.72, 1.60)	1.21 (0.66, 2.17)	0.89 (0.53, 1.48)	0.80 (0.48, 1.32)	1.61 (0.84, 3.24)	1.34 (0.84, 2.10)	1.19 (0.74, 1.87)	1.06 (0.69, 1.60)	1.10 (0.72, 1.67)	0.94 (0.56, 1.55)	0.86 (0.50, 1.42)	1.11 (0.67, 1.79)	1.04 (0.63, 1.68)	0.76 (0.44, 1.25)	0.78 (0.46, 1.31)	0.91 (0.55, 1.49)	1.06 (0.65, 1.70)	1.00 (0.62, 1.58)	0.86 (0.50, 1.46)
AND150mgSC2_2	1.19 (0.60, 2.17)	1.25 (0.79, 1.90)	1.13 (0.60, 2.11)	1.12 (0.74, 1.63)	1.26 (0.70, 2.27)	0.92 (0.56, 1.51)	0.83 (0.49, 1.37)	1.66 (0.87, 3.38)	1.39 (0.87, 2.18)	1.23 (0.78, 1.92)	1.10 (0.71, 1.64)	1.14 (0.74, 1.71)	0.97 (0.58, 1.60)	0.88 (0.51, 1.45)	1.15 (0.69, 1.84)	1.08 (0.64, 1.72)	0.78 (0.46, 1.31)	0.81 (0.48, 1.34)	0.94 (0.56, 1.52)	1.10 (0.67, 1.76)	1.03 (0.64, 1.62)	0.89 (0.52, 1.50)
AND300mgSC	1.02 (0.52, 1.88)	1.07 (0.70, 1.62)	0.98 (0.53, 1.79)	0.97 (0.65, 1.38)	1.08 (0.60, 1.93)	0.80 (0.48, 1.27)	0.71 (0.43, 1.16)	1.43 (0.76, 2.88)	1.20 (0.76, 1.85)	1.06 (0.67, 1.64)	0.95 (0.63, 1.40)	0.98 (0.64, 1.44)	0.84 (0.51, 1.34)	0.76 (0.45, 1.23)	0.99 (0.61, 1.55)	0.93 (0.56, 1.46)	0.68 (0.40, 1.11)	0.70 (0.41, 1.14)	0.81 (0.49, 1.29)	0.95 (0.59, 1.47)	0.89 (0.56, 1.38)	0.77 (0.45, 1.25)
API100mgOT	1.08 (0.59, 1.85)	1.14 (0.83, 1.56)	1.04 (0.60, 1.79)	1.03 (0.79, 1.32)	1.15 (0.69, 1.94)	0.84 (0.56, 1.25)	0.76 (0.49, 1.15)	1.51 (0.86, 2.91)	1.27 (0.91, 1.80)	1.13 (0.79, 1.60)	1.00 (0.74, 1.33)	1.04 (0.77, 1.39)	0.89 (0.59, 1.33)	0.81 (0.52, 1.23)	1.05 (0.72, 1.54)	0.98 (0.65, 1.44)	0.72 (0.47, 1.09)	0.74 (0.49, 1.11)	0.86 (0.58, 1.27)	1.00 (0.70, 1.45)	0.94 (0.65, 1.34)	0.81 (0.51, 1.24)
API50mgOR	1.20 (0.65, 2.07)	1.25 (0.90, 1.75)	1.14 (0.66, 1.98)	1.13 (0.85, 1.48)	1.26 (0.75, 2.15)	0.93 (0.61, 1.39)	0.84 (0.54, 1.28)	1.66 (0.95, 3.23)	1.40 (0.98, 2.02)	1.24 (0.86, 1.79)	1.10 (0.81, 1.50)	1.14 (0.84, 1.56)	0.98 (0.65, 1.49)	0.89 (0.57, 1.39)	1.16 (0.79, 1.73)	1.08 (0.72, 1.61)	0.79 (0.51, 1.21)	0.82 (0.53, 1.25)	0.95 (0.63, 1.41)	1.10 (0.76, 1.60)	1.04 (0.72, 1.50)	0.90 (0.56, 1.40)
BRI400mgIV	1.11 (0.60, 2.01)	1.17 (0.81, 1.68)	1.06 (0.60, 1.91)	1.05 (0.77, 1.44)	1.17 (0.69, 2.05)	0.87 (0.56, 1.34)	0.77 (0.49, 1.21)	1.55 (0.87, 3.10)	1.30 (0.90, 1.94)	1.15 (0.79, 1.71)	1.03 (0.73, 1.46)	1.06 (0.76, 1.51)	0.91 (0.59, 1.43)	0.82 (0.51, 1.32)	1.08 (0.71, 1.65)	1.01 (0.65, 1.54)	0.74 (0.46, 1.17)	0.76 (0.48, 1.21)	0.88 (0.57, 1.37)	1.03 (0.69, 1.55)	0.97 (0.65, 1.44)	0.84 (0.51, 1.34)
BRI700mgIV	1.21 (0.66, 2.10)	1.27 (0.91, 1.76)	1.15 (0.65, 2.02)	1.14 (0.86, 1.49)	1.28 (0.76, 2.16)	0.94 (0.62, 1.41)	0.84 (0.54, 1.29)	1.69 (0.95, 3.26)	1.42 (0.99, 2.04)	1.25 (0.87, 1.80)	1.12 (0.81, 1.52)	1.15 (0.84, 1.58)	0.99 (0.65, 1.50)	0.90 (0.57, 1.38)	1.17 (0.79, 1.73)	1.09 (0.72, 1.62)	0.80 (0.50, 1.24)	0.83 (0.53, 1.28)	0.95 (0.63, 1.45)	1.12 (0.76, 1.63)	1.05 (0.72, 1.51)	0.91 (0.57, 1.40)
BRO210mgIV	1.17 (0.61, 2.11)	1.22 (0.82, 1.83)	1.11 (0.62, 2.04)	1.10 (0.77, 1.58)	1.24 (0.70, 2.20)	0.91 (0.56, 1.45)	0.81 (0.50, 1.32)	1.64 (0.88, 3.27)	1.36 (0.90, 2.12)	1.20 (0.79, 1.87)	1.07 (0.73, 1.61)	1.11 (0.77, 1.66)	0.96 (0.58, 1.54)	0.87 (0.52, 1.41)	1.13 (0.72, 1.78)	1.05 (0.67, 1.69)	0.77 (0.47, 1.25)	0.80 (0.49, 1.31)	0.92 (0.58, 1.46)	1.08 (0.68, 1.68)	1.01 (0.66, 1.58)	0.88 (0.52, 1.45)
BRO350mgIV	1.02 (0.54, 1.81)	1.07 (0.74, 1.55)	0.98 (0.54, 1.72)	0.96 (0.69, 1.34)	1.09 (0.62, 1.86)	0.80 (0.51, 1.24)	0.72 (0.44, 1.13)	1.43 (0.79, 2.84)	1.20 (0.81, 1.81)	1.06 (0.71, 1.59)	0.95 (0.66, 1.36)	0.98 (0.69, 1.39)	0.84 (0.53, 1.30)	0.76 (0.47, 1.21)	0.99 (0.64, 1.52)	0.92 (0.60, 1.43)	0.67 (0.42, 1.07)	0.70 (0.44, 1.09)	0.81 (0.51, 1.24)	0.94 (0.62, 1.42)	0.89 (0.59, 1.34)	0.77 (0.47, 1.22)
BRO700mgIV	0.99 (0.52, 1.73)	1.03 (0.71, 1.49)	0.94 (0.52, 1.68)	0.93 (0.67, 1.27)	1.04 (0.60, 1.80)	0.77 (0.49, 1.18)	0.69 (0.43, 1.07)	1.37 (0.76, 2.71)	1.15 (0.78, 1.73)	1.02 (0.68, 1.52)	0.91 (0.64, 1.29)	0.94 (0.66, 1.34)	0.81 (0.51, 1.26)	0.73 (0.44, 1.16)	0.95 (0.62, 1.47)	0.89 (0.57, 1.36)	0.65 (0.41, 1.03)	0.67 (0.42, 1.06)	0.78 (0.50, 1.20)	0.91 (0.60, 1.38)	0.86 (0.57, 1.27)	0.74 (0.45, 1.17)
CDP10mg_kgIV	1.01 (0.55, 1.73)	1.06 (0.79, 1.41)	0.96 (0.56, 1.65)	0.96 (0.75, 1.21)	1.07 (0.65, 1.79)	0.79 (0.53, 1.15)	0.71 (0.47, 1.05)	1.41 (0.81, 2.71)	1.18 (0.85, 1.66)	1.05 (0.75, 1.47)	0.94 (0.71, 1.23)	0.97 (0.74, 1.28)	0.83 (0.56, 1.23)	0.75 (0.49, 1.13)	0.98 (0.68, 1.42)	0.92 (0.62, 1.33)	0.67 (0.44, 1.00)	0.69 (0.46, 1.04)	0.80 (0.54, 1.17)	0.94 (0.66, 1.31)	0.88 (0.62, 1.24)	0.76 (0.48, 1.14)
CER1_25mgIV	2.13 (0.64, 15.17)	2.26 (0.69, 16.04)	2.04 (0.64, 13.89)	2.04 (0.62, 14.55)	2.25 (0.73, 15.74)	1.69 (0.48, 12.40)	1.51 (0.43, 11.02)	3.11 (0.82, 22.57)	2.55 (0.76, 18.07)	2.23 (0.66, 16.13)	2.01 (0.61, 13.80)	2.07 (0.63, 14.45)	1.79 (0.51, 13.00)	1.61 (0.46, 11.60)	2.12 (0.61, 14.83)	1.96 (0.58, 14.10)	1.43 (0.40, 10.48)	1.47 (0.43, 10.70)	1.70 (0.50, 12.31)	1.98 (0.60, 14.12)	1.90 (0.56, 13.38)	1.66 (0.46, 11.80)
CER100mgSC	0.95 (0.51, 1.63)	1.00 (0.78, 1.25)	0.90 (0.52, 1.56)	0.89 (0.71, 1.11)	1.00 (0.61, 1.67)	0.74 (0.49, 1.09)	0.66 (0.43, 1.00)	1.32 (0.76, 2.58)	1.11 (0.79, 1.57)	0.98 (0.69, 1.40)	0.88 (0.65, 1.17)	0.91 (0.68, 1.21)	0.78 (0.52, 1.16)	0.70 (0.45, 1.08)	0.92 (0.63, 1.35)	0.86 (0.57, 1.26)	**0.63 (0.40, 0.95)**	**0.65 (0.42, 0.98)**	0.75 (0.50, 1.12)	0.88 (0.61, 1.27)	0.82 (0.58, 1.18)	0.71 (0.45, 1.09)

	CER10mgIV	CER200mgSC	CER20mgIV	CER400mgSC	CER5mgIV	ELD10mg_kgIV	ELD20mg_kgIV	ETA25mgSC	ETR105mgSC	ETR210mgSC	FIL100mgPO	FIL200mgPO	FON1mg_kg0_1mgIV_SC	FON1mg_kg1mgIV_SC	FON4mg_kg0_1mgIV_SC	FON4mg_kg1mgIV_SC	GUS1200mgIV	GUS200mgIV	GUS600mgIV	MED700IV	MIR1000mgIV	MIR200mgIV
CER10mgIV	CER10mgIV	1.05 (0.61, 1.92)	0.96 (0.55, 1.68)	0.94 (0.56, 1.69)	1.06 (0.67, 1.81)	0.78 (0.42, 1.51)	0.70 (0.38, 1.36)	1.41 (0.68, 3.34)	1.18 (0.68, 2.19)	1.04 (0.58, 1.95)	0.92 (0.55, 1.68)	0.96 (0.56, 1.73)	0.82 (0.45, 1.56)	0.74 (0.39, 1.43)	0.97 (0.53, 1.83)	0.90 (0.49, 1.71)	0.66 (0.36, 1.28)	0.68 (0.37, 1.33)	0.79 (0.43, 1.53)	0.92 (0.52, 1.76)	0.87 (0.49, 1.61)	0.75 (0.40, 1.48)
CER200mgSC	0.95 (0.52, 1.64)	CER200mgSC	0.91 (0.53, 1.58)	0.90 (0.71, 1.13)	1.01 (0.60, 1.71)	0.74 (0.50, 1.11)	0.67 (0.43, 1.00)	1.33 (0.76, 2.58)	1.12 (0.80, 1.58)	0.99 (0.70, 1.41)	0.88 (0.65, 1.19)	0.91 (0.67, 1.23)	0.78 (0.52, 1.18)	0.71 (0.45, 1.08)	0.92 (0.63, 1.36)	0.86 (0.58, 1.28)	**0.63 (0.41, 0.95)**	**0.65 (0.43, 0.98)**	0.75 (0.50, 1.13)	0.88 (0.61, 1.27)	0.83 (0.58, 1.19)	0.72 (0.45, 1.10)
CER20mgIV	1.04 (0.59, 1.81)	1.10 (0.63, 1.90)	CER20mgIV	0.99 (0.58, 1.68)	1.11 (0.70, 1.83)	0.82 (0.44, 1.50)	0.73 (0.39, 1.34)	1.47 (0.70, 3.28)	1.22 (0.70, 2.21)	1.08 (0.62, 1.95)	0.97 (0.57, 1.67)	1.00 (0.59, 1.74)	0.86 (0.47, 1.57)	0.78 (0.41, 1.45)	1.02 (0.56, 1.83)	0.95 (0.52, 1.72)	0.69 (0.37, 1.30)	0.72 (0.39, 1.31)	0.83 (0.45, 1.53)	0.97 (0.55, 1.74)	0.91 (0.52, 1.62)	0.79 (0.42, 1.47)
CER400mgSC	1.06 (0.59, 1.77)	1.11 (0.89, 1.40)	1.01 (0.60, 1.71)	CER400mgSC	1.12 (0.70, 1.82)	0.83 (0.57, 1.19)	0.74 (0.50, 1.08)	1.48 (0.87, 2.79)	1.24 (0.93, 1.68)	1.10 (0.81, 1.50)	0.98 (0.77, 1.26)	1.01 (0.80, 1.30)	0.87 (0.61, 1.26)	0.79 (0.52, 1.16)	1.02 (0.73, 1.44)	0.96 (0.67, 1.35)	0.70 (0.47, 1.03)	0.73 (0.49, 1.06)	0.84 (0.58, 1.20)	0.98 (0.71, 1.35)	0.92 (0.67, 1.27)	0.80 (0.52, 1.18)
CER5mgIV	0.95 (0.55, 1.50)	0.99 (0.58, 1.66)	0.90 (0.55, 1.44)	0.89 (0.55, 1.44)	CER5mgIV	0.74 (0.40, 1.31)	0.66 (0.36, 1.17)	1.32 (0.65, 2.92)	1.11 (0.65, 1.87)	0.98 (0.56, 1.65)	0.88 (0.53, 1.45)	0.91 (0.54, 1.50)	0.78 (0.44, 1.35)	0.70 (0.38, 1.30)	0.92 (0.53, 1.59)	0.86 (0.48, 1.49)	0.63 (0.34, 1.10)	0.64 (0.36, 1.14)	0.74 (0.42, 1.37)	0.87 (0.50, 1.54)	0.82 (0.47, 1.40)	0.71 (0.38, 1.28)
ELD10mg_kgIV	1.29 (0.66, 2.35)	1.35 (0.90, 2.02)	1.22 (0.67, 2.28)	1.21 (0.84, 1.74)	1.36 (0.77, 2.47)	ELD10mg_kgIV	0.90 (0.59, 1.32)	1.80 (0.97, 3.64)	1.50 (1.00, 2.33)	1.33 (0.88, 2.06)	1.19 (0.82, 1.75)	1.23 (0.84, 1.81)	1.05 (0.66, 1.71)	0.96 (0.58, 1.55)	1.24 (0.80, 2.01)	1.16 (0.73, 1.86)	0.85 (0.52, 1.39)	0.88 (0.55, 1.43)	1.02 (0.63, 1.63)	1.19 (0.77, 1.87)	1.12 (0.72, 1.73)	0.97 (0.58, 1.59)
ELD20mg_kgIV	1.42 (0.73, 2.66)	1.50 (1.00, 2.32)	1.37 (0.74, 2.54)	1.35 (0.93, 1.99)	1.52 (0.86, 2.75)	1.11 (0.76, 1.68)	ELD20mg_kgIV	**2.00 (1.08, 4.17)**	**1.68 (1.09, 2.66)**	1.48 (0.96, 2.34)	1.33 (0.89, 2.00)	1.37 (0.93, 2.06)	1.17 (0.72, 1.93)	1.06 (0.63, 1.77)	1.39 (0.88, 2.24)	1.30 (0.80, 2.12)	0.95 (0.57, 1.56)	0.98 (0.59, 1.60)	1.13 (0.69, 1.87)	1.33 (0.84, 2.10)	1.24 (0.80, 1.99)	1.08 (0.64, 1.81)
ETA25mgSC	0.71 (0.30, 1.46)	0.75 (0.39, 1.31)	0.68 (0.30, 1.42)	0.68 (0.36, 1.14)	0.76 (0.34, 1.55)	0.56 (0.27, 1.03)	**0.50 (0.24, 0.93)**	ETA25mgSC	0.84 (0.43, 1.50)	0.74 (0.38, 1.34)	0.66 (0.34, 1.15)	0.69 (0.36, 1.19)	0.58 (0.29, 1.09)	0.53 (0.26, 1.00)	0.69 (0.34, 1.28)	0.65 (0.32, 1.19)	**0.47 (0.23, 0.88)**	**0.49 (0.23, 0.91)**	0.57 (0.28, 1.04)	0.67 (0.33, 1.18)	0.63 (0.31, 1.12)	0.54 (0.26, 1.01)
ETR105mgSC	0.85 (0.46, 1.47)	0.89 (0.63, 1.25)	0.82 (0.45, 1.43)	0.81 (0.59, 1.07)	0.90 (0.54, 1.54)	0.67 (0.43, 1.00)	**0.60 (0.38, 0.91)**	1.19 (0.67, 2.35)	ETR105mgSC	0.88 (0.69, 1.11)	0.79 (0.57, 1.09)	0.82 (0.58, 1.12)	0.70 (0.45, 1.08)	**0.63 (0.40, 0.99)**	0.82 (0.55, 1.25)	0.77 (0.50, 1.16)	**0.56 (0.36, 0.87)**	**0.58 (0.37, 0.90)**	0.67 (0.44, 1.02)	0.79 (0.53, 1.16)	0.74 (0.50, 1.08)	0.64 (0.39, 1.00)
ETR210mgSC	0.96 (0.51, 1.72)	1.01 (0.71, 1.44)	0.92 (0.51, 1.62)	0.91 (0.67, 1.23)	1.02 (0.60, 1.77)	0.75 (0.49, 1.14)	0.67 (0.43, 1.04)	1.35 (0.75, 2.66)	1.13 (0.90, 1.44)	ETR210mgSC	0.89 (0.64, 1.24)	0.92 (0.66, 1.30)	0.79 (0.51, 1.22)	0.72 (0.44, 1.12)	0.93 (0.62, 1.42)	0.88 (0.57, 1.33)	**0.64 (0.40, 0.99)**	0.66 (0.42, 1.03)	0.76 (0.49, 1.17)	0.89 (0.60, 1.32)	0.84 (0.56, 1.24)	0.73 (0.45, 1.14)
FIL100mgPO	1.08 (0.59, 1.83)	1.14 (0.84, 1.54)	1.03 (0.60, 1.75)	1.02 (0.80, 1.29)	1.14 (0.69, 1.89)	0.84 (0.57, 1.22)	0.75 (0.50, 1.12)	1.51 (0.87, 2.92)	1.27 (0.92, 1.76)	1.12 (0.80, 1.56)	FIL100mgPO	1.03 (0.85, 1.26)	0.88 (0.60, 1.31)	0.80 (0.52, 1.20)	1.05 (0.73, 1.51)	0.98 (0.67, 1.41)	0.72 (0.47, 1.07)	0.74 (0.49, 1.10)	0.85 (0.58, 1.25)	1.00 (0.71, 1.42)	0.94 (0.67, 1.33)	0.82 (0.51, 1.22)
FIL200mgPO	1.05 (0.58, 1.77)	1.10 (0.81, 1.49)	1.00 (0.58, 1.69)	0.99 (0.77, 1.25)	1.10 (0.67, 1.85)	0.81 (0.55, 1.19)	0.73 (0.49, 1.08)	1.46 (0.84, 2.82)	1.23 (0.89, 1.71)	1.09 (0.77, 1.51)	0.97 (0.79, 1.17)	FIL200mgPO	0.86 (0.59, 1.27)	0.78 (0.50, 1.17)	1.01 (0.70, 1.47)	0.95 (0.64, 1.37)	0.69 (0.45, 1.03)	0.71 (0.48, 1.07)	0.83 (0.56, 1.21)	0.97 (0.68, 1.37)	0.91 (0.64, 1.28)	0.79 (0.50, 1.17)
FON1mg_kg0_1mgIV_SC	1.22 (0.64, 2.21)	1.28 (0.85, 1.92)	1.17 (0.64, 2.11)	1.15 (0.80, 1.65)	1.29 (0.74, 2.27)	0.95 (0.58, 1.52)	0.85 (0.52, 1.40)	1.71 (0.91, 3.49)	1.43 (0.92, 2.21)	1.26 (0.82, 1.97)	1.13 (0.76, 1.66)	1.17 (0.79, 1.71)	FON1mg_kg0_1mgIV_SC	0.91 (0.60, 1.36)	1.18 (0.84, 1.66)	1.10 (0.78, 1.57)	0.81 (0.49, 1.32)	0.83 (0.51, 1.37)	0.96 (0.61, 1.56)	1.13 (0.72, 1.76)	1.06 (0.69, 1.65)	0.92 (0.53, 1.51)
FON1mg_kg1mgIV_SC	1.35 (0.70, 2.54)	1.41 (0.92, 2.21)	1.28 (0.69, 2.42)	1.27 (0.87, 1.92)	1.42 (0.77, 2.60)	1.04 (0.64, 1.73)	0.94 (0.56, 1.58)	1.88 (1.00, 3.91)	**1.58 (1.01, 2.51)**	1.39 (0.89, 2.25)	1.25 (0.83, 1.93)	1.28 (0.86, 2.00)	1.10 (0.73, 1.66)	FON1mg_kg1mgIV_SC	1.30 (0.90, 1.91)	1.21 (0.84, 1.80)	0.89 (0.53, 1.51)	0.92 (0.55, 1.55)	1.06 (0.65, 1.77)	1.24 (0.78, 2.01)	1.17 (0.72, 1.89)	1.01 (0.59, 1.73)
FON4mg_kg0_1mgIV_SC	1.03 (0.55, 1.87)	1.09 (0.73, 1.60)	0.98 (0.55, 1.78)	0.98 (0.69, 1.37)	1.09 (0.63, 1.90)	0.81 (0.50, 1.26)	0.72 (0.45, 1.14)	1.44 (0.78, 2.92)	1.21 (0.80, 1.83)	1.07 (0.70, 1.62)	0.96 (0.66, 1.37)	0.99 (0.68, 1.43)	0.85 (0.60, 1.19)	0.77 (0.52, 1.11)	FON4mg_kg0_1mgIV_SC	0.94 (0.68, 1.28)	0.68 (0.42, 1.09)	0.71 (0.43, 1.12)	0.82 (0.51, 1.28)	0.96 (0.61, 1.45)	0.90 (0.59, 1.38)	0.78 (0.46, 1.25)
FON4mg_kg1mgIV_SC	1.11 (0.58, 2.03)	1.16 (0.78, 1.74)	1.06 (0.58, 1.92)	1.04 (0.74, 1.49)	1.17 (0.67, 2.07)	0.86 (0.54, 1.37)	0.77 (0.47, 1.25)	1.55 (0.84, 3.13)	1.29 (0.86, 1.98)	1.14 (0.75, 1.77)	1.02 (0.71, 1.50)	1.05 (0.73, 1.55)	0.91 (0.64, 1.28)	0.82 (0.56, 1.19)	1.07 (0.78, 1.48)	FON4mg_kg1mgIV_SC	0.73 (0.45, 1.18)	0.76 (0.46, 1.23)	0.87 (0.55, 1.38)	1.03 (0.66, 1.57)	0.96 (0.63, 1.48)	0.83 (0.50, 1.35)
GUS1200mgIV	1.51 (0.78, 2.80)	**1.58 (1.05, 2.44)**	1.44 (0.77, 2.70)	1.42 (0.97, 2.12)	1.59 (0.91, 2.93)	1.18 (0.72, 1.91)	1.05 (0.64, 1.74)	**2.11 (1.14, 4.38)**	**1.77 (1.15, 2.81)**	**1.56 (1.01, 2.51)**	1.40 (0.93, 2.13)	1.45 (0.97, 2.21)	1.24 (0.76, 2.05)	1.12 (0.66, 1.89)	1.47 (0.91, 2.37)	1.37 (0.85, 2.24)	GUS1200mgIV	1.03 (0.70, 1.56)	1.20 (0.82, 1.77)	1.40 (0.87, 2.25)	1.32 (0.83, 2.10)	1.14 (0.66, 1.92)
GUS200mgIV	1.46 (0.75, 2.67)	**1.54 (1.02, 2.34)**	1.39 (0.76, 2.56)	1.38 (0.94, 2.03)	1.55 (0.88, 2.77)	1.14 (0.70, 1.83)	1.02 (0.63, 1.69)	**2.04 (1.10, 4.27)**	**1.72 (1.12, 2.68)**	1.52 (0.97, 2.40)	1.35 (0.91, 2.02)	1.40 (0.94, 2.11)	1.20 (0.73, 1.98)	1.09 (0.65, 1.82)	1.41 (0.89, 2.30)	1.32 (0.82, 2.17)	0.97 (0.64, 1.44)	GUS200mgIV	1.16 (0.80, 1.70)	1.35 (0.86, 2.17)	1.27 (0.80, 2.00)	1.10 (0.65, 1.86)
GUS600mgIV	1.27 (0.65, 2.34)	1.33 (0.89, 1.98)	1.20 (0.65, 2.22)	1.19 (0.83, 1.72)	1.35 (0.73, 2.40)	0.98 (0.61, 1.58)	0.89 (0.54, 1.44)	1.77 (0.96, 3.62)	1.49 (0.98, 2.30)	1.31 (0.85, 2.03)	1.17 (0.80, 1.73)	1.21 (0.83, 1.78)	1.04 (0.64, 1.65)	0.94 (0.57, 1.53)	1.22 (0.78, 1.95)	1.15 (0.72, 1.81)	0.84 (0.57, 1.21)	0.87 (0.59, 1.25)	GUS600mgIV	1.16 (0.75, 1.83)	1.10 (0.69, 1.71)	0.95 (0.57, 1.58)
MED700IV	1.09 (0.57, 1.91)	1.14 (0.79, 1.64)	1.03 (0.57, 1.83)	1.02 (0.74, 1.41)	1.15 (0.65, 2.02)	0.84 (0.54, 1.29)	0.75 (0.48, 1.19)	1.50 (0.84, 3.05)	1.27 (0.86, 1.90)	1.12 (0.76, 1.68)	1.00 (0.70, 1.42)	1.03 (0.73, 1.47)	0.89 (0.57, 1.40)	0.80 (0.50, 1.28)	1.04 (0.69, 1.63)	0.97 (0.64, 1.51)	0.72 (0.44, 1.14)	0.74 (0.46, 1.17)	0.86 (0.55, 1.33)	MED700IV	0.94 (0.62, 1.40)	0.81 (0.50, 1.30)
MIR1000mgIV	1.15 (0.62, 2.03)	1.21 (0.84, 1.72)	1.10 (0.62, 1.93)	1.09 (0.79, 1.49)	1.21 (0.71, 2.11)	0.90 (0.58, 1.38)	0.81 (0.50, 1.25)	1.60 (0.89, 3.20)	1.35 (0.92, 2.00)	1.19 (0.81, 1.77)	1.07 (0.75, 1.50)	1.10 (0.78, 1.56)	0.94 (0.61, 1.46)	0.86 (0.53, 1.38)	1.12 (0.73, 1.70)	1.05 (0.67, 1.59)	0.76 (0.48, 1.21)	0.79 (0.50, 1.24)	0.91 (0.58, 1.45)	1.06 (0.71, 1.61)	MIR1000mgIV	0.87 (0.57, 1.25)
MIR200mgIV	1.33 (0.68, 2.53)	1.39 (0.91, 2.22)	1.26 (0.68, 2.40)	1.25 (0.84, 1.93)	1.40 (0.78, 2.63)	1.03 (0.63, 1.73)	0.93 (0.55, 1.56)	1.86 (0.99, 3.90)	1.56 (1.00, 2.55)	1.38 (0.88, 2.25)	1.22 (0.82, 1.94)	1.27 (0.85, 2.00)	1.09 (0.66, 1.87)	0.99 (0.58, 1.69)	1.28 (0.80, 2.18)	1.20 (0.74, 2.01)	0.88 (0.52, 1.51)	0.91 (0.54, 1.55)	1.05 (0.63, 1.76)	1.23 (0.77, 2.01)	1.15 (0.80, 1.75)	MIR200mgIV

	CER10mgIV	CER200mgSC	CER20mgIV	CER400mgSC	CER5mgIV	ELD10mg_kgIV	ELD20mg_kgIV	ETA25mgSC	ETR105mgSC	ETR210mgSC	FIL100mgPO	FIL200mgPO	FON1mg_kg0_1mgIV_SC	FON1mg_kg1mgIV_SC	FON4mg_kg0_1mgIV_SC	FON4mg_kg1mgIV_SC	GUS1200mgIV	GUS200mgIV	GUS600mgIV	MED700IV	MIR1000mgIV	MIR200mgIV
MIR600mgIV	1.16 (0.61, 2.17)	1.22 (0.82, 1.88)	1.11 (0.61, 2.04)	1.10 (0.77, 1.62)	1.23 (0.70, 2.24)	0.91 (0.56, 1.47)	0.82 (0.50, 1.34)	1.63 (0.88, 3.28)	1.37 (0.90, 2.15)	1.20 (0.79, 1.93)	1.07 (0.74, 1.63)	1.11 (0.76, 1.70)	0.96 (0.59, 1.58)	0.87 (0.52, 1.45)	1.13 (0.71, 1.81)	1.05 (0.67, 1.72)	0.77 (0.47, 1.27)	0.80 (0.49, 1.32)	0.92 (0.57, 1.52)	1.08 (0.69, 1.74)	1.01 (0.72, 1.49)	0.88 (0.56, 1.36)
NAT300mgIV	1.04 (0.58, 1.72)	1.09 (0.84, 1.40)	0.99 (0.59, 1.64)	0.98 (0.81, 1.17)	1.09 (0.68, 1.78)	0.81 (0.57, 1.14)	0.72 (0.50, 1.04)	1.45 (0.86, 2.72)	1.21 (0.91, 1.63)	1.07 (0.79, 1.45)	0.96 (0.76, 1.21)	0.99 (0.79, 1.24)	0.85 (0.59, 1.21)	0.77 (0.51, 1.13)	1.00 (0.72, 1.41)	0.94 (0.66, 1.32)	**0.69 (0.47, 0.99)**	0.71 (0.48, 1.03)	0.82 (0.57, 1.17)	0.96 (0.70, 1.31)	0.90 (0.66, 1.22)	0.78 (0.51, 1.14)
NAT3mg_kgIV	1.17 (0.65, 2.05)	1.24 (0.89, 1.71)	1.13 (0.64, 1.97)	1.11 (0.84, 1.47)	1.25 (0.74, 2.12)	0.92 (0.61, 1.38)	0.82 (0.53, 1.25)	1.64 (0.94, 3.21)	1.38 (0.97, 1.97)	1.22 (0.85, 1.75)	1.09 (0.80, 1.49)	1.12 (0.83, 1.53)	0.96 (0.64, 1.47)	0.88 (0.55, 1.35)	1.14 (0.77, 1.70)	1.07 (0.70, 1.60)	0.78 (0.51, 1.19)	0.80 (0.52, 1.23)	0.93 (0.61, 1.39)	1.09 (0.75, 1.58)	1.02 (0.71, 1.47)	0.89 (0.56, 1.36)
NAT3mg_kgIVx2	1.01 (0.56, 1.72)	1.06 (0.77, 1.44)	0.96 (0.55, 1.64)	0.95 (0.72, 1.22)	1.06 (0.64, 1.77)	0.78 (0.52, 1.16)	0.70 (0.46, 1.06)	1.40 (0.80, 2.71)	1.18 (0.83, 1.66)	1.04 (0.73, 1.47)	0.93 (0.69, 1.24)	0.96 (0.72, 1.28)	0.82 (0.55, 1.23)	0.75 (0.48, 1.13)	0.97 (0.66, 1.44)	0.91 (0.61, 1.34)	0.66 (0.43, 1.00)	0.69 (0.45, 1.04)	0.79 (0.53, 1.18)	0.93 (0.64, 1.33)	0.87 (0.61, 1.24)	0.76 (0.48, 1.15)
NAT6mg_kgIVx2	1.11 (0.60, 1.93)	1.17 (0.84, 1.63)	1.06 (0.60, 1.86)	1.05 (0.79, 1.39)	1.18 (0.70, 1.99)	0.87 (0.58, 1.31)	0.78 (0.50, 1.19)	1.55 (0.88, 3.05)	1.31 (0.92, 1.89)	1.15 (0.80, 1.68)	1.03 (0.75, 1.42)	1.06 (0.78, 1.47)	0.91 (0.60, 1.39)	0.83 (0.52, 1.29)	1.08 (0.72, 1.62)	1.01 (0.66, 1.51)	0.74 (0.47, 1.13)	0.76 (0.49, 1.16)	0.88 (0.58, 1.33)	1.03 (0.70, 1.52)	0.97 (0.67, 1.40)	0.84 (0.53, 1.30)
NNC2mg_kgSC	1.05 (0.55, 1.89)	1.10 (0.72, 1.64)	1.00 (0.54, 1.81)	0.99 (0.69, 1.39)	1.11 (0.62, 1.98)	0.82 (0.51, 1.28)	0.73 (0.44, 1.16)	1.47 (0.80, 2.91)	1.23 (0.80, 1.87)	1.09 (0.71, 1.66)	0.97 (0.65, 1.41)	1.00 (0.68, 1.47)	0.86 (0.53, 1.37)	0.78 (0.47, 1.25)	1.02 (0.64, 1.59)	0.95 (0.60, 1.48)	0.69 (0.42, 1.12)	0.72 (0.44, 1.15)	0.83 (0.51, 1.30)	0.97 (0.63, 1.50)	0.91 (0.59, 1.41)	0.79 (0.46, 1.29)
ONE10mgSC	1.03 (0.53, 1.87)	1.09 (0.72, 1.62)	0.98 (0.53, 1.80)	0.98 (0.68, 1.39)	1.10 (0.62, 1.94)	0.81 (0.49, 1.29)	0.72 (0.44, 1.16)	1.45 (0.77, 2.87)	1.21 (0.78, 1.87)	1.07 (0.69, 1.63)	0.95 (0.65, 1.40)	0.99 (0.67, 1.45)	0.85 (0.53, 1.35)	0.77 (0.46, 1.25)	1.00 (0.64, 1.59)	0.94 (0.58, 1.50)	0.68 (0.40, 1.12)	0.70 (0.43, 1.15)	0.82 (0.51, 1.30)	0.96 (0.61, 1.48)	0.90 (0.57, 1.37)	0.78 (0.46, 1.27)
ONE25mgSC	1.09 (0.56, 2.05)	1.15 (0.75, 1.76)	1.04 (0.56, 1.93)	1.03 (0.70, 1.53)	1.16 (0.65, 2.09)	0.86 (0.52, 1.38)	0.77 (0.47, 1.26)	1.54 (0.82, 3.13)	1.29 (0.83, 2.00)	1.14 (0.72, 1.79)	1.02 (0.67, 1.52)	1.05 (0.70, 1.58)	0.90 (0.55, 1.50)	0.82 (0.48, 1.36)	1.06 (0.66, 1.72)	0.99 (0.60, 1.64)	0.72 (0.43, 1.22)	0.75 (0.45, 1.23)	0.87 (0.53, 1.44)	1.02 (0.64, 1.60)	0.95 (0.61, 1.49)	0.83 (0.47, 1.37)
ONE35mgSC	0.97 (0.51, 1.77)	1.03 (0.68, 1.52)	0.93 (0.49, 1.70)	0.92 (0.63, 1.30)	1.03 (0.58, 1.84)	0.76 (0.47, 1.20)	0.68 (0.41, 1.10)	1.37 (0.73, 2.74)	1.14 (0.74, 1.73)	1.01 (0.65, 1.53)	0.90 (0.61, 1.31)	0.94 (0.63, 1.36)	0.80 (0.50, 1.29)	0.72 (0.44, 1.19)	0.95 (0.59, 1.47)	0.88 (0.54, 1.40)	0.65 (0.39, 1.04)	0.67 (0.41, 1.06)	0.77 (0.48, 1.22)	0.90 (0.58, 1.39)	0.85 (0.55, 1.30)	0.74 (0.42, 1.18)
ONE50mgSC	0.98 (0.51, 1.75)	1.02 (0.69, 1.50)	0.93 (0.51, 1.67)	0.92 (0.64, 1.30)	1.03 (0.58, 1.83)	0.76 (0.47, 1.20)	0.68 (0.42, 1.09)	1.37 (0.72, 2.68)	1.14 (0.74, 1.75)	1.01 (0.65, 1.55)	0.90 (0.62, 1.31)	0.93 (0.64, 1.36)	0.80 (0.50, 1.28)	0.73 (0.43, 1.18)	0.95 (0.60, 1.48)	0.88 (0.55, 1.38)	0.64 (0.39, 1.04)	0.67 (0.41, 1.07)	0.77 (0.48, 1.21)	0.90 (0.58, 1.38)	0.85 (0.54, 1.28)	0.74 (0.43, 1.18)
ONT22_5mgSC	1.06 (0.58, 1.83)	1.12 (0.82, 1.51)	1.01 (0.58, 1.72)	1.00 (0.77, 1.29)	1.13 (0.67, 1.88)	0.83 (0.56, 1.21)	0.74 (0.49, 1.10)	1.49 (0.86, 2.84)	1.24 (0.89, 1.76)	1.10 (0.78, 1.56)	0.98 (0.74, 1.31)	1.02 (0.76, 1.36)	0.87 (0.59, 1.30)	0.79 (0.51, 1.19)	1.03 (0.71, 1.50)	0.96 (0.65, 1.40)	0.71 (0.46, 1.06)	0.73 (0.48, 1.10)	0.84 (0.56, 1.23)	0.98 (0.68, 1.40)	0.92 (0.64, 1.32)	0.80 (0.50, 1.23)
ONT225mgSC	1.16 (0.63, 1.98)	1.21 (0.89, 1.66)	1.10 (0.63, 1.91)	1.09 (0.84, 1.41)	1.22 (0.73, 2.08)	0.90 (0.60, 1.34)	0.81 (0.52, 1.20)	1.62 (0.93, 3.13)	1.35 (0.98, 1.92)	1.20 (0.85, 1.70)	1.07 (0.80, 1.44)	1.11 (0.83, 1.49)	0.95 (0.63, 1.41)	0.86 (0.55, 1.31)	1.12 (0.76, 1.65)	1.05 (0.71, 1.54)	0.76 (0.50, 1.16)	0.79 (0.52, 1.20)	0.91 (0.61, 1.36)	1.07 (0.73, 1.53)	1.00 (0.71, 1.44)	0.88 (0.55, 1.32)
ONT75mgSC	1.17 (0.63, 2.01)	1.23 (0.89, 1.69)	1.11 (0.64, 1.92)	1.10 (0.84, 1.44)	1.24 (0.74, 2.09)	0.91 (0.61, 1.36)	0.82 (0.53, 1.22)	1.63 (0.93, 3.16)	1.37 (0.97, 1.96)	1.21 (0.85, 1.73)	1.08 (0.80, 1.46)	1.12 (0.83, 1.51)	0.96 (0.63, 1.46)	0.87 (0.56, 1.33)	1.13 (0.77, 1.67)	1.06 (0.71, 1.56)	0.78 (0.50, 1.17)	0.80 (0.52, 1.22)	0.93 (0.62, 1.39)	1.08 (0.74, 1.57)	1.02 (0.71, 1.45)	0.88 (0.55, 1.35)
PBO	1.07 (0.61, 1.75)	1.13 (0.89, 1.41)	1.02 (0.62, 1.69)	1.01 (0.87, 1.16)	1.13 (0.72, 1.81)	0.84 (0.60, 1.16)	0.75 (0.52, 1.05)	1.50 (0.90, 2.78)	1.26 (0.97, 1.65)	1.11 (0.85, 1.47)	0.99 (0.81, 1.20)	1.02 (0.84, 1.24)	0.88 (0.63, 1.23)	0.80 (0.54, 1.14)	1.03 (0.76, 1.42)	0.97 (0.69, 1.34)	0.71 (0.49, 1.01)	0.73 (0.51, 1.04)	0.85 (0.60, 1.17)	0.99 (0.74, 1.32)	0.93 (0.70, 1.23)	0.81 (0.53, 1.15)
PF10mgSC	1.09 (0.60, 1.84)	1.15 (0.85, 1.53)	1.04 (0.60, 1.78)	1.03 (0.81, 1.29)	1.16 (0.70, 1.92)	0.85 (0.57, 1.24)	0.76 (0.50, 1.13)	1.52 (0.88, 2.92)	1.28 (0.93, 1.78)	1.13 (0.81, 1.57)	1.01 (0.77, 1.31)	1.04 (0.80, 1.37)	0.89 (0.61, 1.31)	0.81 (0.53, 1.22)	1.05 (0.73, 1.53)	0.99 (0.67, 1.42)	0.73 (0.47, 1.07)	0.75 (0.50, 1.11)	0.86 (0.59, 1.26)	1.01 (0.71, 1.42)	0.95 (0.67, 1.32)	0.82 (0.52, 1.23)
PF200mgSC	1.20 (0.65, 2.06)	1.26 (0.91, 1.74)	1.14 (0.66, 1.99)	1.13 (0.87, 1.49)	1.27 (0.75, 2.14)	0.93 (0.63, 1.39)	0.84 (0.55, 1.27)	1.67 (0.97, 3.21)	1.40 (1.00, 2.00)	1.24 (0.87, 1.77)	1.11 (0.83, 1.51)	1.14 (0.86, 1.56)	0.99 (0.66, 1.48)	0.89 (0.57, 1.37)	1.16 (0.80, 1.71)	1.08 (0.73, 1.61)	0.79 (0.52, 1.20)	0.82 (0.54, 1.25)	0.95 (0.63, 1.42)	1.11 (0.77, 1.60)	1.04 (0.72, 1.50)	0.91 (0.57, 1.38)
PF50mgSC	1.20 (0.66, 2.04)	1.26 (0.92, 1.70)	1.14 (0.66, 1.96)	1.13 (0.88, 1.44)	1.27 (0.76, 2.09)	0.93 (0.63, 1.36)	0.84 (0.55, 1.24)	1.67 (0.97, 3.23)	**1.40 (1.01, 1.96)**	1.24 (0.88, 1.75)	1.11 (0.83, 1.46)	1.14 (0.86, 1.51)	0.98 (0.66, 1.45)	0.89 (0.58, 1.34)	1.16 (0.80, 1.69)	1.09 (0.73, 1.58)	0.80 (0.52, 1.19)	0.82 (0.54, 1.22)	0.95 (0.64, 1.39)	1.11 (0.78, 1.58)	1.04 (0.73, 1.46)	0.90 (0.57, 1.35)
RIS1200mgIV	1.19 (0.67, 1.99)	1.25 (0.95, 1.63)	1.14 (0.67, 1.92)	1.13 (0.91, 1.37)	1.26 (0.78, 2.06)	0.93 (0.64, 1.32)	0.83 (0.57, 1.21)	1.67 (0.98, 3.19)	**1.40 (1.03, 1.89)**	1.24 (0.90, 1.69)	1.11 (0.86, 1.41)	1.14 (0.89, 1.45)	0.98 (0.68, 1.41)	0.89 (0.58, 1.31)	1.15 (0.82, 1.64)	1.08 (0.76, 1.52)	0.79 (0.53, 1.15)	0.81 (0.56, 1.19)	0.94 (0.65, 1.35)	1.11 (0.79, 1.52)	1.04 (0.75, 1.43)	0.90 (0.57, 1.32)
RIS200mgIV	1.16 (0.63, 2.05)	1.22 (0.87, 1.70)	1.10 (0.62, 1.95)	1.09 (0.82, 1.46)	1.23 (0.73, 2.11)	0.90 (0.59, 1.37)	0.81 (0.52, 1.24)	1.62 (0.92, 3.15)	1.36 (0.95, 1.95)	1.20 (0.84, 1.74)	1.07 (0.79, 1.47)	1.11 (0.81, 1.54)	0.95 (0.62, 1.45)	0.86 (0.54, 1.34)	1.12 (0.76, 1.69)	1.05 (0.70, 1.57)	0.77 (0.49, 1.17)	0.79 (0.51, 1.22)	0.92 (0.61, 1.38)	1.07 (0.73, 1.57)	1.01 (0.69, 1.47)	0.87 (0.54, 1.35)
RIS600mgIV	1.22 (0.69, 2.04)	1.28 (0.99, 1.66)	1.17 (0.69, 1.96)	1.16 (0.95, 1.39)	1.29 (0.80, 2.13)	0.95 (0.66, 1.36)	0.86 (0.58, 1.24)	**1.70 (1.01, 3.24)**	**1.43 (1.07, 1.95)**	1.27 (0.93, 1.72)	1.13 (0.89, 1.44)	1.17 (0.92, 1.48)	1.00 (0.70, 1.45)	0.91 (0.60, 1.34)	1.18 (0.84, 1.68)	1.11 (0.77, 1.56)	0.81 (0.54, 1.19)	0.83 (0.57, 1.21)	0.97 (0.67, 1.38)	1.13 (0.82, 1.56)	1.06 (0.77, 1.45)	0.92 (0.59, 1.35)
RIS600mgIV4_4	1.57 (0.44, 5.42)	1.62 (0.52, 5.41)	1.48 (0.42, 5.15)	1.45 (0.47, 4.86)	1.67 (0.50, 5.63)	1.21 (0.36, 4.07)	1.07 (0.33, 3.79)	2.17 (0.62, 8.52)	1.80 (0.58, 6.23)	1.60 (0.51, 5.50)	1.42 (0.46, 4.82)	1.48 (0.48, 4.92)	1.26 (0.39, 4.37)	1.14 (0.35, 3.91)	1.49 (0.47, 5.19)	1.40 (0.44, 4.88)	1.02 (0.31, 3.51)	1.06 (0.32, 3.66)	1.21 (0.38, 4.17)	1.41 (0.46, 4.76)	1.33 (0.43, 4.48)	1.16 (0.36, 4.01)
SEC10mg_kgIV	0.70 (0.32, 1.36)	0.74 (0.40, 1.23)	0.66 (0.32, 1.32)	0.66 (0.36, 1.07)	0.75 (0.36, 1.42)	**0.55 (0.28, 0.94)**	**0.49 (0.25, 0.86)**	0.98 (0.46, 2.11)	0.82 (0.44, 1.40)	0.73 (0.38, 1.23)	0.65 (0.35, 1.07)	0.67 (0.36, 1.11)	0.58 (0.29, 1.00)	**0.52 (0.26, 0.93)**	0.68 (0.35, 1.20)	0.64 (0.33, 1.10)	**0.46 (0.23, 0.84)**	**0.48 (0.24, 0.86)**	**0.56 (0.28, 0.98)**	0.65 (0.34, 1.13)	0.61 (0.32, 1.04)	**0.53 (0.27, 0.94)**

	CER10mgIV	CER200mgSC	CER20mgIV	CER400mgSC	CER5mgIV	ELD10mg_kgIV	ELD20mg_kgIV	ETA25mgSC	ETR105mgSC	ETR210mgSC	FIL100mgPO	FIL200mgPO	FON1mg_kg0_1mgIV_SC	FON1mg_kg1mgIV_SC	FON4mg_kg0_1mgIV_SC	FON4mg_kg1mgIV_SC	GUS1200mgIV	GUS200mgIV	GUS600mgIV	MED700IV	MIR1000mgIV	MIR200mgIV
SEM60mgIV	0.93 (0.47, 1.72)	0.97 (0.63, 1.49)	0.89 (0.46, 1.63)	0.87 (0.59, 1.28)	0.98 (0.54, 1.77)	0.72 (0.44, 1.17)	0.65 (0.39, 1.07)	1.30 (0.68, 2.66)	1.09 (0.70, 1.71)	0.96 (0.61, 1.51)	0.86 (0.57, 1.29)	0.89 (0.59, 1.33)	0.76 (0.46, 1.23)	0.69 (0.41, 1.14)	0.90 (0.56, 1.42)	0.84 (0.51, 1.33)	0.61 (0.36, 1.03)	0.63 (0.38, 1.04)	0.73 (0.45, 1.20)	0.86 (0.54, 1.34)	0.81 (0.51, 1.27)	0.70 (0.40, 1.16)
SEM60mgIVx3	0.82 (0.43, 1.52)	0.87 (0.57, 1.29)	0.79 (0.43, 1.43)	0.78 (0.53, 1.11)	0.87 (0.49, 1.56)	0.64 (0.40, 1.02)	**0.58 (0.35, 0.94)**	1.16 (0.61, 2.32)	0.97 (0.63, 1.48)	0.86 (0.55, 1.32)	0.77 (0.51, 1.12)	0.79 (0.53, 1.16)	0.68 (0.42, 1.08)	**0.62 (0.37, 0.98)**	0.80 (0.50, 1.24)	0.75 (0.46, 1.17)	**0.54 (0.32, 0.89)**	**0.56 (0.34, 0.93)**	0.65 (0.40, 1.04)	0.76 (0.48, 1.18)	0.72 (0.46, 1.11)	0.62 (0.36, 1.01)
TES400mg200mgSC	0.84 (0.45, 1.53)	0.88 (0.58, 1.30)	0.80 (0.43, 1.45)	0.79 (0.55, 1.12)	0.89 (0.50, 1.58)	0.65 (0.41, 1.03)	**0.59 (0.35, 0.95)**	1.18 (0.63, 2.34)	0.99 (0.65, 1.51)	0.87 (0.56, 1.34)	0.78 (0.53, 1.14)	0.80 (0.55, 1.17)	0.69 (0.43, 1.10)	0.62 (0.38, 1.01)	0.81 (0.52, 1.28)	0.76 (0.47, 1.21)	**0.56 (0.33, 0.90)**	**0.57 (0.35, 0.93)**	0.67 (0.42, 1.06)	0.78 (0.50, 1.21)	0.73 (0.47, 1.12)	0.64 (0.37, 1.03)
TOF15mgOR_BID	1.11 (0.54, 2.06)	1.17 (0.74, 1.87)	1.07 (0.55, 2.01)	1.05 (0.69, 1.59)	1.18 (0.64, 2.18)	0.87 (0.51, 1.45)	0.77 (0.46, 1.30)	1.55 (0.80, 3.30)	1.30 (0.80, 2.12)	1.16 (0.70, 1.90)	1.03 (0.66, 1.60)	1.07 (0.68, 1.65)	0.91 (0.54, 1.52)	0.82 (0.47, 1.40)	1.08 (0.65, 1.78)	1.01 (0.60, 1.65)	0.74 (0.43, 1.25)	0.76 (0.43, 1.28)	0.88 (0.52, 1.47)	1.03 (0.62, 1.67)	0.96 (0.59, 1.59)	0.84 (0.47, 1.42)
TOF1mgOR_BID	1.39 (0.67, 2.69)	1.46 (0.89, 2.40)	1.33 (0.67, 2.62)	1.31 (0.82, 2.11)	1.47 (0.78, 2.84)	1.09 (0.62, 1.88)	0.97 (0.55, 1.69)	1.95 (0.99, 4.22)	1.63 (0.97, 2.78)	1.44 (0.85, 2.46)	1.29 (0.78, 2.10)	1.34 (0.81, 2.16)	1.14 (0.65, 2.02)	1.04 (0.57, 1.85)	1.35 (0.79, 2.35)	1.26 (0.73, 2.19)	0.92 (0.51, 1.63)	0.95 (0.53, 1.69)	1.10 (0.63, 1.93)	1.29 (0.75, 2.20)	1.21 (0.71, 2.04)	1.05 (0.59, 1.87)
TOF5mgOR_BID	1.14 (0.55, 2.10)	1.19 (0.74, 1.89)	1.08 (0.56, 2.03)	1.07 (0.69, 1.62)	1.20 (0.64, 2.22)	0.88 (0.52, 1.45)	0.79 (0.46, 1.30)	1.58 (0.82, 3.33)	1.32 (0.82, 2.16)	1.17 (0.72, 1.91)	1.05 (0.67, 1.63)	1.08 (0.69, 1.68)	0.92 (0.55, 1.58)	0.84 (0.47, 1.44)	1.09 (0.65, 1.82)	1.02 (0.61, 1.70)	0.75 (0.43, 1.26)	0.77 (0.44, 1.31)	0.89 (0.53, 1.50)	1.05 (0.64, 1.70)	0.98 (0.60, 1.61)	0.85 (0.48, 1.46)
UPA12mgOR_BID	0.97 (0.51, 1.75)	1.01 (0.69, 1.49)	0.92 (0.51, 1.66)	0.91 (0.64, 1.28)	1.02 (0.58, 1.80)	0.75 (0.47, 1.16)	0.67 (0.42, 1.08)	1.35 (0.74, 2.72)	1.13 (0.75, 1.71)	1.00 (0.66, 1.51)	0.89 (0.62, 1.28)	0.92 (0.64, 1.34)	0.79 (0.50, 1.23)	0.72 (0.44, 1.14)	0.93 (0.61, 1.43)	0.87 (0.56, 1.36)	0.64 (0.39, 1.02)	0.65 (0.41, 1.06)	0.76 (0.47, 1.20)	0.89 (0.59, 1.36)	0.84 (0.55, 1.26)	0.72 (0.43, 1.18)
UPA24mgOR	0.94 (0.50, 1.67)	0.99 (0.67, 1.44)	0.89 (0.50, 1.60)	0.89 (0.63, 1.23)	1.00 (0.57, 1.72)	0.73 (0.46, 1.14)	0.66 (0.41, 1.05)	1.31 (0.72, 2.66)	1.11 (0.73, 1.66)	0.97 (0.64, 1.46)	0.87 (0.60, 1.23)	0.90 (0.62, 1.29)	0.78 (0.48, 1.20)	0.70 (0.43, 1.10)	0.91 (0.59, 1.40)	0.85 (0.55, 1.31)	**0.62 (0.38, 0.99)**	0.64 (0.40, 1.03)	0.75 (0.47, 1.18)	0.87 (0.57, 1.31)	0.82 (0.54, 1.24)	0.71 (0.42, 1.13)
UPA24mgOR_BID	0.94 (0.49, 1.68)	0.98 (0.67, 1.43)	0.88 (0.50, 1.58)	0.88 (0.63, 1.23)	0.99 (0.56, 1.73)	0.73 (0.46, 1.13)	0.65 (0.40, 1.04)	1.30 (0.72, 2.64)	1.10 (0.73, 1.64)	0.97 (0.64, 1.44)	0.86 (0.60, 1.24)	0.89 (0.62, 1.28)	0.77 (0.49, 1.20)	0.69 (0.43, 1.10)	0.90 (0.59, 1.39)	0.84 (0.54, 1.31)	**0.62 (0.38, 0.98)**	0.63 (0.40, 1.01)	0.74 (0.46, 1.16)	0.86 (0.57, 1.32)	0.81 (0.53, 1.23)	0.70 (0.42, 1.13)
UPA3mgOR_BID	0.89 (0.48, 1.57)	0.94 (0.64, 1.35)	0.85 (0.47, 1.49)	0.84 (0.60, 1.15)	0.95 (0.55, 1.65)	0.69 (0.44, 1.08)	**0.62 (0.39, 0.97)**	1.25 (0.68, 2.51)	1.05 (0.70, 1.56)	0.92 (0.61, 1.38)	0.82 (0.58, 1.17)	0.85 (0.60, 1.21)	0.73 (0.46, 1.14)	0.67 (0.41, 1.04)	0.86 (0.56, 1.31)	0.80 (0.52, 1.23)	**0.59 (0.37, 0.93)**	**0.61 (0.38, 0.96)**	0.71 (0.44, 1.09)	0.83 (0.54, 1.23)	0.77 (0.52, 1.15)	0.67 (0.40, 1.06)
UPA45mgOR	1.02 (0.57, 1.69)	1.07 (0.81, 1.39)	0.96 (0.57, 1.62)	0.96 (0.77, 1.16)	1.07 (0.66, 1.75)	0.79 (0.55, 1.12)	0.71 (0.48, 1.02)	1.41 (0.84, 2.66)	1.19 (0.89, 1.62)	1.05 (0.77, 1.43)	0.94 (0.73, 1.19)	0.97 (0.76, 1.23)	0.83 (0.57, 1.20)	0.75 (0.49, 1.11)	0.98 (0.70, 1.39)	0.92 (0.64, 1.29)	**0.67 (0.45, 0.98)**	0.69 (0.47, 1.01)	0.80 (0.55, 1.14)	0.94 (0.67, 1.29)	0.88 (0.63, 1.20)	0.77 (0.49, 1.12)
UPA6mgOR_BID	0.99 (0.53, 1.79)	1.04 (0.71, 1.53)	0.95 (0.53, 1.70)	0.94 (0.66, 1.31)	1.06 (0.61, 1.86)	0.78 (0.49, 1.21)	0.69 (0.43, 1.11)	1.39 (0.75, 2.85)	1.17 (0.77, 1.76)	1.03 (0.67, 1.56)	0.92 (0.63, 1.33)	0.95 (0.65, 1.38)	0.82 (0.52, 1.29)	0.74 (0.45, 1.18)	0.96 (0.63, 1.49)	0.90 (0.57, 1.38)	0.66 (0.41, 1.05)	0.68 (0.42, 1.09)	0.79 (0.49, 1.24)	0.92 (0.60, 1.39)	0.86 (0.56, 1.31)	0.75 (0.44, 1.21)
UST130mgIV	1.12 (0.63, 1.86)	1.18 (0.90, 1.54)	1.07 (0.63, 1.81)	1.06 (0.86, 1.29)	1.18 (0.73, 1.94)	0.87 (0.61, 1.25)	0.78 (0.53, 1.13)	1.56 (0.93, 2.95)	1.31 (0.98, 1.78)	1.16 (0.85, 1.60)	1.04 (0.81, 1.33)	1.07 (0.84, 1.38)	0.92 (0.64, 1.33)	0.83 (0.55, 1.23)	1.08 (0.77, 1.54)	1.02 (0.71, 1.44)	0.74 (0.50, 1.08)	0.77 (0.52, 1.12)	0.89 (0.61, 1.27)	1.04 (0.75, 1.44)	0.97 (0.71, 1.34)	0.84 (0.55, 1.24)
UST1mg_kgIV	1.05 (0.58, 1.80)	1.11 (0.81, 1.50)	1.01 (0.58, 1.73)	1.00 (0.78, 1.27)	1.11 (0.67, 1.87)	0.82 (0.56, 1.20)	0.74 (0.49, 1.10)	1.47 (0.85, 2.81)	1.24 (0.89, 1.73)	1.09 (0.77, 1.55)	0.98 (0.74, 1.29)	1.01 (0.76, 1.33)	0.86 (0.58, 1.28)	0.79 (0.51, 1.19)	1.02 (0.71, 1.48)	0.96 (0.65, 1.39)	0.70 (0.45, 1.04)	0.72 (0.48, 1.08)	0.83 (0.56, 1.23)	0.98 (0.68, 1.38)	0.92 (0.64, 1.31)	0.80 (0.50, 1.21)
UST3mg_kgIV	1.10 (0.60, 1.86)	1.15 (0.85, 1.56)	1.05 (0.60, 1.79)	1.03 (0.80, 1.32)	1.16 (0.69, 1.93)	0.85 (0.57, 1.25)	0.76 (0.51, 1.13)	1.52 (0.88, 2.93)	1.29 (0.93, 1.80)	1.14 (0.81, 1.58)	1.02 (0.76, 1.34)	1.05 (0.79, 1.38)	0.89 (0.61, 1.34)	0.82 (0.53, 1.23)	1.06 (0.73, 1.55)	0.99 (0.67, 1.45)	0.73 (0.47, 1.09)	0.75 (0.49, 1.12)	0.87 (0.58, 1.28)	1.01 (0.70, 1.45)	0.95 (0.67, 1.36)	0.82 (0.52, 1.24)
UST4_5mg_kgIV	0.98 (0.50, 1.84)	1.03 (0.66, 1.59)	0.93 (0.49, 1.75)	0.93 (0.61, 1.38)	1.04 (0.58, 1.91)	0.76 (0.45, 1.25)	0.68 (0.40, 1.14)	1.37 (0.71, 2.84)	1.15 (0.72, 1.82)	1.02 (0.64, 1.61)	0.91 (0.59, 1.40)	0.94 (0.61, 1.44)	0.80 (0.48, 1.34)	0.73 (0.42, 1.23)	0.94 (0.59, 1.53)	0.89 (0.54, 1.46)	0.65 (0.38, 1.08)	0.67 (0.40, 1.11)	0.78 (0.46, 1.27)	0.91 (0.56, 1.47)	0.85 (0.53, 1.36)	0.73 (0.42, 1.25)
UST6mg_kg90mgIV_SC	1.27 (0.66, 2.30)	1.33 (0.89, 1.98)	1.20 (0.66, 2.19)	1.20 (0.83, 1.71)	1.34 (0.75, 2.40)	0.99 (0.62, 1.56)	0.88 (0.55, 1.45)	1.77 (0.95, 3.59)	1.49 (0.97, 2.29)	1.31 (0.86, 2.02)	1.17 (0.80, 1.73)	1.21 (0.82, 1.78)	1.04 (0.65, 1.67)	0.94 (0.56, 1.55)	1.22 (0.77, 1.93)	1.15 (0.72, 1.81)	0.84 (0.56, 1.21)	0.87 (0.59, 1.27)	1.00 (0.71, 1.43)	1.17 (0.75, 1.84)	1.11 (0.70, 1.71)	0.95 (0.57, 1.56)
UST6mg_kgIV	1.10 (0.62, 1.83)	1.16 (0.90, 1.50)	1.05 (0.63, 1.77)	1.04 (0.86, 1.24)	1.17 (0.72, 1.90)	0.86 (0.60, 1.22)	0.77 (0.53, 1.11)	1.54 (0.92, 2.90)	1.29 (0.97, 1.75)	1.14 (0.85, 1.55)	1.02 (0.81, 1.28)	1.05 (0.84, 1.33)	0.90 (0.63, 1.31)	0.82 (0.54, 1.20)	1.07 (0.77, 1.50)	1.00 (0.70, 1.40)	0.73 (0.49, 1.06)	0.76 (0.52, 1.09)	0.87 (0.61, 1.24)	1.02 (0.74, 1.39)	0.96 (0.70, 1.31)	0.83 (0.54, 1.21)
UST90mgSC	1.34 (0.69, 2.48)	1.40 (0.92, 2.18)	1.27 (0.69, 2.38)	1.25 (0.87, 1.87)	1.42 (0.79, 2.60)	1.04 (0.65, 1.72)	0.93 (0.56, 1.57)	1.87 (1.00, 3.88)	**1.57 (1.02, 2.50)**	1.38 (0.89, 2.20)	1.23 (0.83, 1.89)	1.28 (0.87, 1.93)	1.10 (0.67, 1.82)	0.99 (0.59, 1.66)	1.29 (0.81, 2.13)	1.20 (0.75, 2.00)	0.88 (0.53, 1.47)	0.92 (0.56, 1.51)	1.05 (0.66, 1.76)	1.23 (0.79, 1.97)	1.15 (0.74, 1.84)	1.00 (0.59, 1.70)
VED0_5mg_kgIV	0.99 (0.54, 1.67)	1.04 (0.75, 1.39)	0.94 (0.54, 1.60)	0.93 (0.71, 1.17)	1.04 (0.63, 1.73)	0.77 (0.51, 1.12)	0.69 (0.45, 1.01)	1.37 (0.79, 2.66)	1.16 (0.83, 1.60)	1.02 (0.72, 1.43)	0.91 (0.68, 1.19)	0.94 (0.71, 1.23)	0.80 (0.55, 1.19)	0.73 (0.47, 1.11)	0.95 (0.66, 1.38)	0.89 (0.60, 1.30)	**0.65 (0.42, 0.97)**	0.67 (0.45, 1.01)	0.78 (0.52, 1.16)	0.91 (0.64, 1.29)	0.86 (0.61, 1.19)	0.74 (0.47, 1.11)
VED2mg_kgIV	1.02 (0.56, 1.71)	1.07 (0.78, 1.43)	0.97 (0.56, 1.64)	0.96 (0.74, 1.22)	1.07 (0.65, 1.79)	0.79 (0.53, 1.15)	0.71 (0.47, 1.05)	1.42 (0.81, 2.72)	1.19 (0.85, 1.66)	1.05 (0.75, 1.47)	0.94 (0.71, 1.24)	0.97 (0.73, 1.28)	0.83 (0.56, 1.23)	0.76 (0.49, 1.13)	0.98 (0.68, 1.42)	0.92 (0.62, 1.33)	0.67 (0.44, 1.01)	0.69 (0.46, 1.04)	0.80 (0.54, 1.17)	0.94 (0.65, 1.33)	0.88 (0.63, 1.24)	0.76 (0.49, 1.15)
VED300mgIV	1.09 (0.61, 1.82)	1.15 (0.87, 1.48)	1.04 (0.61, 1.75)	1.03 (0.84, 1.24)	1.16 (0.71, 1.88)	0.85 (0.59, 1.21)	0.76 (0.52, 1.10)	1.52 (0.90, 2.86)	1.28 (0.95, 1.75)	1.13 (0.83, 1.55)	1.01 (0.79, 1.28)	1.05 (0.82, 1.33)	0.90 (0.62, 1.29)	0.81 (0.53, 1.19)	1.06 (0.75, 1.50)	0.99 (0.68, 1.40)	0.72 (0.48, 1.05)	0.75 (0.51, 1.10)	0.86 (0.60, 1.22)	1.01 (0.73, 1.39)	0.95 (0.69, 1.30)	0.82 (0.53, 1.21)

(TABLE S16B. continued)

	MIR600mgIV	NAT300mgIV	NAT3mg_kgIV	NAT3mg_kgIVx2	NAT6mg_kgIVx2	NNC2mg_kgSC	ONE10mgSC	ONE25mgSC	ONE35mgSC	ONE50mgSC	ONT22_5mgSC	ONT225mgSC	ONT75mgSC	PBO	PF10mgSC	PF200mgSC	PF50mgSC	RIS1200mgIV	RIS200mgIV	RIS600mgIV	RIS600mgIV4_4	SEC10mg_kgIV
ABA10mg_kgIV	0.90 (0.59, 1.32)	1.01 (0.80, 1.29)	0.89 (0.65, 1.22)	1.04 (0.78, 1.42)	0.94 (0.68, 1.30)	0.99 (0.68, 1.48)	1.02 (0.69, 1.51)	0.95 (0.63, 1.44)	1.07 (0.74, 1.59)	1.07 (0.74, 1.59)	0.98 (0.73, 1.33)	0.91 (0.67, 1.23)	0.90 (0.66, 1.22)	0.98 (0.80, 1.21)	0.96 (0.73, 1.28)	0.87 (0.63, 1.19)	0.87 (0.66, 1.17)	0.88 (0.68, 1.14)	0.90 (0.65, 1.24)	0.86 (0.67, 1.09)	0.68 (0.20, 2.13)	1.49 (0.91, 2.73)
ABA30mg_kgIV	0.91 (0.59, 1.37)	1.02 (0.79, 1.33)	0.90 (0.65, 1.27)	1.06 (0.78, 1.47)	0.95 (0.68, 1.34)	1.01 (0.69, 1.52)	1.03 (0.69, 1.56)	0.97 (0.63, 1.48)	1.09 (0.73, 1.64)	1.09 (0.73, 1.65)	1.00 (0.73, 1.37)	0.92 (0.67, 1.27)	0.91 (0.65, 1.26)	0.99 (0.79, 1.26)	0.97 (0.73, 1.33)	0.89 (0.64, 1.23)	0.89 (0.66, 1.21)	0.89 (0.68, 1.18)	0.91 (0.65, 1.28)	0.87 (0.67, 1.14)	0.69 (0.20, 2.16)	1.51 (0.91, 2.83)
ABA3mg_kgIV	0.92 (0.60, 1.36)	1.04 (0.82, 1.31)	0.91 (0.66, 1.26)	1.07 (0.79, 1.45)	0.96 (0.70, 1.34)	1.02 (0.70, 1.51)	1.04 (0.71, 1.54)	0.98 (0.65, 1.47)	1.10 (0.76, 1.63)	1.10 (0.75, 1.64)	1.01 (0.76, 1.37)	0.93 (0.69, 1.26)	0.92 (0.67, 1.25)	1.00 (0.81, 1.23)	0.98 (0.74, 1.30)	0.90 (0.65, 1.22)	0.90 (0.67, 1.20)	0.90 (0.70, 1.16)	0.93 (0.67, 1.27)	0.88 (0.68, 1.12)	0.70 (0.21, 2.19)	1.53 (0.93, 2.82)
ADA160mg80mg40mgSC	1.11 (0.66, 1.85)	1.26 (0.85, 1.86)	1.11 (0.71, 1.72)	1.30 (0.84, 2.00)	1.17 (0.74, 1.82)	1.24 (0.75, 2.04)	1.26 (0.76, 2.09)	1.18 (0.70, 2.01)	1.34 (0.81, 2.23)	1.34 (0.81, 2.20)	1.22 (0.80, 1.90)	1.13 (0.73, 1.74)	1.11 (0.71, 1.75)	1.21 (0.84, 1.76)	1.19 (0.79, 1.84)	1.09 (0.70, 1.69)	1.09 (0.71, 1.67)	1.09 (0.73, 1.64)	1.12 (0.71, 1.77)	1.06 (0.71, 1.59)	0.84 (0.25, 2.70)	**1.85 (1.02, 3.73)**
ADA160mg80mg60mgSC	0.91 (0.54, 1.51)	1.03 (0.68, 1.55)	0.91 (0.56, 1.45)	1.06 (0.68, 1.67)	0.96 (0.60, 1.53)	1.02 (0.61, 1.68)	1.03 (0.61, 1.72)	0.98 (0.57, 1.62)	1.09 (0.66, 1.84)	1.10 (0.65, 1.82)	1.00 (0.64, 1.56)	0.92 (0.58, 1.44)	0.91 (0.58, 1.43)	1.00 (0.67, 1.46)	0.98 (0.63, 1.51)	0.89 (0.56, 1.39)	0.89 (0.57, 1.39)	0.89 (0.58, 1.35)	0.92 (0.57, 1.46)	0.87 (0.57, 1.31)	0.69 (0.20, 2.26)	1.52 (0.82, 3.00)
ADA160mg80mgSC	1.03 (0.68, 1.46)	1.15 (0.94, 1.38)	1.01 (0.76, 1.33)	1.19 (0.91, 1.56)	1.07 (0.79, 1.42)	1.14 (0.79, 1.65)	1.15 (0.80, 1.67)	1.09 (0.73, 1.60)	1.22 (0.86, 1.77)	1.22 (0.86, 1.77)	1.13 (0.86, 1.45)	1.03 (0.78, 1.33)	1.02 (0.76, 1.34)	1.11 (0.95, 1.30)	1.10 (0.85, 1.38)	1.00 (0.75, 1.29)	1.00 (0.77, 1.27)	1.00 (0.81, 1.23)	1.03 (0.76, 1.36)	0.98 (0.79, 1.19)	0.77 (0.23, 2.39)	**1.70 (1.06, 3.09)**
ADA40mg20mgSC	1.07 (0.69, 1.61)	1.21 (0.93, 1.57)	1.06 (0.76, 1.48)	1.25 (0.91, 1.72)	1.12 (0.79, 1.58)	1.19 (0.79, 1.82)	1.21 (0.81, 1.81)	1.14 (0.74, 1.74)	1.28 (0.86, 1.93)	1.28 (0.86, 1.94)	1.18 (0.86, 1.62)	1.08 (0.78, 1.49)	1.07 (0.76, 1.49)	1.17 (0.93, 1.48)	1.15 (0.85, 1.56)	1.04 (0.75, 1.44)	1.05 (0.77, 1.42)	1.05 (0.80, 1.39)	1.08 (0.76, 1.52)	1.02 (0.78, 1.34)	0.81 (0.24, 2.55)	**1.78 (1.06, 3.33)**
ADA80mg40mgSC	1.06 (0.70, 1.55)	1.19 (0.94, 1.51)	1.05 (0.76, 1.44)	1.23 (0.91, 1.67)	1.11 (0.80, 1.52)	1.17 (0.81, 1.76)	1.20 (0.81, 1.77)	1.13 (0.75, 1.70)	1.27 (0.87, 1.85)	1.27 (0.87, 1.86)	1.16 (0.87, 1.56)	1.07 (0.79, 1.44)	1.06 (0.77, 1.44)	1.15 (0.94, 1.42)	1.13 (0.86, 1.49)	1.03 (0.75, 1.38)	1.03 (0.77, 1.37)	1.04 (0.81, 1.34)	1.07 (0.77, 1.47)	1.01 (0.79, 1.29)	0.81 (0.24, 2.52)	**1.76 (1.07, 3.26)**
AMI0_4mgOR	0.78 (0.45, 1.31)	0.88 (0.57, 1.32)	0.77 (0.48, 1.22)	0.90 (0.57, 1.43)	0.82 (0.50, 1.29)	0.86 (0.52, 1.45)	0.88 (0.51, 1.47)	0.83 (0.48, 1.41)	0.93 (0.55, 1.58)	0.93 (0.55, 1.56)	0.85 (0.55, 1.35)	0.78 (0.50, 1.24)	0.78 (0.49, 1.23)	0.85 (0.56, 1.25)	0.83 (0.53, 1.29)	0.76 (0.47, 1.20)	0.76 (0.48, 1.18)	0.76 (0.49, 1.15)	0.78 (0.48, 1.24)	0.74 (0.48, 1.12)	0.59 (0.17, 1.89)	1.29 (0.71, 2.59)
AND150mgSC1_1	0.98 (0.58, 1.60)	1.11 (0.74, 1.62)	0.98 (0.62, 1.52)	1.15 (0.74, 1.75)	1.03 (0.65, 1.60)	1.10 (0.66, 1.82)	1.11 (0.67, 1.82)	1.05 (0.62, 1.73)	1.18 (0.72, 1.93)	1.18 (0.72, 1.93)	1.08 (0.70, 1.67)	0.99 (0.64, 1.52)	0.98 (0.63, 1.52)	1.07 (0.74, 1.54)	1.05 (0.69, 1.58)	0.96 (0.62, 1.45)	0.96 (0.62, 1.45)	0.96 (0.64, 1.43)	0.99 (0.62, 1.53)	0.94 (0.62, 1.37)	0.74 (0.22, 2.41)	1.63 (0.90, 3.22)
AND150mgSC2_2	1.02 (0.60, 1.65)	1.15 (0.77, 1.66)	1.01 (0.64, 1.57)	1.18 (0.76, 1.81)	1.06 (0.68, 1.65)	1.13 (0.69, 1.85)	1.15 (0.70, 1.87)	1.08 (0.66, 1.78)	1.22 (0.74, 1.99)	1.22 (0.75, 1.96)	1.12 (0.72, 1.70)	1.03 (0.66, 1.55)	1.02 (0.65, 1.56)	1.11 (0.76, 1.58)	1.09 (0.71, 1.63)	0.99 (0.63, 1.51)	1.00 (0.64, 1.49)	1.00 (0.66, 1.46)	1.02 (0.65, 1.58)	0.97 (0.65, 1.41)	0.76 (0.22, 2.54)	1.68 (0.94, 3.31)
AND300mgSC	0.88 (0.53, 1.40)	0.99 (0.67, 1.41)	0.87 (0.56, 1.33)	1.02 (0.67, 1.53)	0.92 (0.59, 1.39)	0.98 (0.60, 1.56)	0.99 (0.60, 1.60)	0.93 (0.55, 1.52)	1.05 (0.64, 1.69)	1.05 (0.64, 1.69)	0.96 (0.63, 1.44)	0.89 (0.58, 1.32)	0.88 (0.57, 1.31)	0.96 (0.66, 1.34)	0.94 (0.62, 1.37)	0.86 (0.56, 1.27)	0.86 (0.56, 1.26)	0.86 (0.58, 1.23)	0.88 (0.57, 1.33)	0.84 (0.56, 1.20)	0.66 (0.19, 2.16)	1.45 (0.82, 2.83)
API100mgOT	0.93 (0.60, 1.37)	1.05 (0.82, 1.34)	0.92 (0.67, 1.28)	1.08 (0.80, 1.48)	0.97 (0.70, 1.35)	1.03 (0.70, 1.55)	1.05 (0.71, 1.55)	0.99 (0.65, 1.50)	1.11 (0.76, 1.66)	1.11 (0.76, 1.65)	1.02 (0.75, 1.39)	0.94 (0.69, 1.28)	0.93 (0.68, 1.28)	1.01 (0.82, 1.26)	0.99 (0.75, 1.33)	0.91 (0.66, 1.24)	0.91 (0.67, 1.22)	0.91 (0.70, 1.19)	0.94 (0.66, 1.30)	0.89 (0.68, 1.14)	0.70 (0.21, 2.20)	1.55 (0.93, 2.89)
API50mgOR	1.03 (0.66, 1.53)	1.15 (0.89, 1.50)	1.01 (0.72, 1.42)	1.19 (0.87, 1.65)	1.07 (0.76, 1.51)	1.14 (0.76, 1.73)	1.16 (0.77, 1.74)	1.09 (0.71, 1.65)	1.22 (0.82, 1.86)	1.23 (0.82, 1.86)	1.12 (0.82, 1.56)	1.03 (0.75, 1.43)	1.02 (0.74, 1.43)	1.11 (0.88, 1.42)	1.10 (0.81, 1.50)	1.00 (0.72, 1.38)	1.00 (0.73, 1.37)	1.00 (0.76, 1.33)	1.03 (0.72, 1.45)	0.98 (0.75, 1.28)	0.78 (0.23, 2.43)	**1.71 (1.01, 3.19)**
BRI400mgIV	0.95 (0.61, 1.48)	1.07 (0.79, 1.47)	0.95 (0.65, 1.38)	1.11 (0.78, 1.59)	1.00 (0.68, 1.46)	1.06 (0.69, 1.64)	1.07 (0.70, 1.70)	1.01 (0.65, 1.60)	1.14 (0.74, 1.78)	1.14 (0.75, 1.77)	1.05 (0.75, 1.50)	0.96 (0.67, 1.39)	0.95 (0.66, 1.38)	1.04 (0.79, 1.39)	1.02 (0.73, 1.44)	0.93 (0.64, 1.34)	0.93 (0.66, 1.33)	0.93 (0.68, 1.29)	0.96 (0.65, 1.41)	0.91 (0.66, 1.25)	0.72 (0.21, 2.30)	1.58 (0.92, 3.03)
BRI700mgIV	1.03 (0.67, 1.55)	1.16 (0.89, 1.53)	1.03 (0.73, 1.45)	1.20 (0.87, 1.68)	1.08 (0.76, 1.52)	1.15 (0.77, 1.73)	1.17 (0.77, 1.77)	1.10 (0.71, 1.70)	1.24 (0.82, 1.88)	1.24 (0.83, 1.87)	1.13 (0.82, 1.56)	1.04 (0.74, 1.46)	1.03 (0.74, 1.44)	1.13 (0.88, 1.43)	1.11 (0.81, 1.52)	1.01 (0.71, 1.41)	1.01 (0.73, 1.39)	1.01 (0.76, 1.35)	1.04 (0.73, 1.47)	0.99 (0.74, 1.30)	0.78 (0.23, 2.43)	**1.72 (1.02, 3.24)**
BRO210mgIV	0.99 (0.61, 1.60)	1.12 (0.79, 1.62)	0.99 (0.65, 1.51)	1.15 (0.79, 1.75)	1.04 (0.70, 1.59)	1.11 (0.71, 1.79)	1.12 (0.70, 1.82)	1.06 (0.66, 1.73)	1.20 (0.75, 1.90)	1.20 (0.76, 1.90)	1.09 (0.74, 1.64)	1.01 (0.68, 1.51)	1.00 (0.67, 1.51)	1.08 (0.78, 1.53)	1.07 (0.73, 1.58)	0.97 (0.65, 1.46)	0.97 (0.66, 1.45)	0.97 (0.67, 1.42)	1.00 (0.67, 1.54)	0.95 (0.67, 1.38)	0.76 (0.22, 2.46)	1.66 (0.94, 3.30)
BRO350mgIV	0.87 (0.55, 1.34)	0.98 (0.71, 1.35)	0.87 (0.59, 1.29)	1.01 (0.70, 1.46)	0.92 (0.62, 1.34)	0.98 (0.63, 1.52)	0.99 (0.63, 1.56)	0.93 (0.59, 1.47)	1.05 (0.68, 1.62)	1.05 (0.68, 1.65)	0.96 (0.67, 1.37)	0.88 (0.61, 1.28)	0.87 (0.60, 1.27)	0.95 (0.71, 1.28)	0.94 (0.65, 1.32)	0.85 (0.58, 1.23)	0.85 (0.59, 1.22)	0.86 (0.61, 1.20)	0.88 (0.59, 1.29)	0.83 (0.60, 1.15)	0.66 (0.19, 2.09)	1.46 (0.84, 2.80)
BRO700mgIV	0.85 (0.53, 1.29)	0.95 (0.69, 1.29)	0.83 (0.57, 1.23)	0.98 (0.68, 1.42)	0.89 (0.60, 1.29)	0.93 (0.61, 1.47)	0.95 (0.61, 1.49)	0.89 (0.56, 1.42)	1.01 (0.65, 1.57)	1.01 (0.65, 1.58)	0.93 (0.65, 1.32)	0.85 (0.59, 1.22)	0.84 (0.59, 1.22)	0.92 (0.68, 1.23)	0.90 (0.64, 1.28)	0.82 (0.56, 1.18)	0.82 (0.58, 1.17)	0.82 (0.60, 1.15)	0.85 (0.57, 1.24)	0.80 (0.59, 1.11)	0.65 (0.19, 2.02)	1.40 (0.81, 2.67)
CDP10mg_kgIV	0.87 (0.57, 1.27)	0.98 (0.77, 1.22)	0.86 (0.62, 1.18)	1.01 (0.75, 1.35)	0.91 (0.66, 1.23)	0.96 (0.66, 1.43)	0.98 (0.67, 1.44)	0.92 (0.61, 1.39)	1.03 (0.71, 1.52)	1.04 (0.72, 1.52)	0.95 (0.72, 1.27)	0.87 (0.65, 1.17)	0.87 (0.64, 1.16)	0.94 (0.77, 1.14)	0.93 (0.71, 1.22)	0.84 (0.62, 1.13)	0.85 (0.63, 1.12)	0.85 (0.67, 1.09)	0.87 (0.63, 1.19)	0.83 (0.65, 1.05)	0.66 (0.20, 2.04)	1.44 (0.88, 2.63)
CER1_25mgIV	1.86 (0.54, 13.14)	2.09 (0.65, 14.76)	1.85 (0.56, 12.91)	2.15 (0.65, 15.16)	1.94 (0.58, 13.95)	2.06 (0.60, 15.16)	2.09 (0.61, 15.08)	1.97 (0.57, 14.42)	2.22 (0.65, 15.84)	2.21 (0.65, 16.15)	2.04 (0.62, 14.32)	1.87 (0.57, 13.23)	1.85 (0.56, 13.21)	2.02 (0.62, 14.06)	1.99 (0.60, 14.14)	1.81 (0.55, 12.61)	1.81 (0.54, 12.82)	1.81 (0.56, 12.78)	1.86 (0.56, 13.09)	1.76 (0.54, 12.48)	1.45 (0.25, 14.58)	3.14 (0.85, 23.51)
CER100mgSC	0.81 (0.53, 1.20)	0.91 (0.71, 1.18)	0.80 (0.58, 1.11)	0.94 (0.70, 1.30)	0.85 (0.61, 1.18)	0.90 (0.61, 1.37)	0.92 (0.62, 1.36)	0.86 (0.57, 1.31)	0.97 (0.66, 1.47)	0.97 (0.66, 1.44)	0.89 (0.66, 1.22)	0.82 (0.60, 1.12)	0.81 (0.59, 1.11)	0.88 (0.71, 1.11)	0.87 (0.65, 1.17)	0.79 (0.57, 1.08)	0.79 (0.59, 1.07)	0.80 (0.61, 1.04)	0.82 (0.58, 1.13)	0.77 (0.60, 1.01)	0.61 (0.18, 1.91)	1.35 (0.81, 2.52)

	MIR600mgIV	NAT300mgIV	NAT3mg_kgIV	NAT3mg_kgIVx2	NAT6mg_kgIVx2	NNC2mg_kgSC	ONE10mgSC	ONE25mgSC	ONE35mgSC	ONE50mgSC	ONT22_5mgSC	ONT225mgSC	ONT75mgSC	PBO	PF10mgSC	PF200mgSC	PF50mgSC	RIS1200mgIV	RIS200mgIV	RIS600mgIV	RIS600mgIV4_4	SEC10mg_kgIV
CER10mgIV	0.86 (0.46, 1.64)	0.96 (0.58, 1.72)	0.85 (0.49, 1.54)	0.99 (0.58, 1.80)	0.90 (0.52, 1.67)	0.96 (0.53, 1.81)	0.97 (0.53, 1.90)	0.92 (0.49, 1.79)	1.03 (0.56, 1.97)	1.02 (0.57, 1.98)	0.94 (0.55, 1.72)	0.86 (0.50, 1.58)	0.86 (0.50, 1.59)	0.93 (0.57, 1.64)	0.91 (0.54, 1.66)	0.83 (0.49, 1.53)	0.83 (0.49, 1.52)	0.84 (0.50, 1.50)	0.86 (0.49, 1.58)	0.82 (0.49, 1.46)	0.64 (0.18, 2.28)	1.43 (0.73, 3.16)
CER200mgSC	0.82 (0.53, 1.21)	0.92 (0.71, 1.19)	0.81 (0.58, 1.13)	0.95 (0.70, 1.30)	0.85 (0.61, 1.19)	0.91 (0.61, 1.39)	0.92 (0.62, 1.39)	0.87 (0.57, 1.33)	0.97 (0.66, 1.46)	0.98 (0.67, 1.46)	0.89 (0.66, 1.23)	0.82 (0.60, 1.12)	0.81 (0.59, 1.12)	0.89 (0.71, 1.12)	0.87 (0.65, 1.17)	0.79 (0.58, 1.09)	0.80 (0.59, 1.08)	0.80 (0.61, 1.05)	0.82 (0.59, 1.15)	0.78 (0.60, 1.01)	0.62 (0.18, 1.91)	1.36 (0.81, 2.51)
CER20mgIV	0.90 (0.49, 1.64)	1.01 (0.61, 1.70)	0.89 (0.51, 1.57)	1.04 (0.61, 1.83)	0.94 (0.54, 1.66)	1.00 (0.55, 1.85)	1.02 (0.56, 1.89)	0.96 (0.52, 1.80)	1.07 (0.59, 2.02)	1.08 (0.60, 1.98)	0.99 (0.58, 1.71)	0.91 (0.52, 1.59)	0.90 (0.52, 1.56)	0.98 (0.59, 1.62)	0.96 (0.56, 1.65)	0.88 (0.50, 1.51)	0.88 (0.51, 1.51)	0.88 (0.52, 1.50)	0.91 (0.51, 1.60)	0.86 (0.51, 1.45)	0.68 (0.19, 2.37)	1.51 (0.76, 3.16)
CER400mgSC	0.91 (0.62, 1.30)	1.02 (0.85, 1.24)	0.90 (0.68, 1.19)	1.05 (0.82, 1.39)	0.95 (0.72, 1.26)	1.01 (0.72, 1.45)	1.03 (0.72, 1.48)	0.97 (0.65, 1.43)	1.08 (0.77, 1.58)	1.09 (0.77, 1.57)	1.00 (0.78, 1.30)	0.92 (0.71, 1.19)	0.91 (0.70, 1.19)	0.99 (0.86, 1.14)	0.97 (0.78, 1.24)	0.89 (0.67, 1.15)	0.89 (0.70, 1.14)	0.89 (0.73, 1.10)	0.91 (0.68, 1.22)	0.87 (0.72, 1.06)	0.69 (0.21, 2.12)	1.51 (0.94, 2.75)
CER5mgIV	0.81 (0.45, 1.43)	0.91 (0.56, 1.46)	0.80 (0.47, 1.34)	0.94 (0.56, 1.57)	0.85 (0.50, 1.43)	0.90 (0.51, 1.60)	0.91 (0.52, 1.61)	0.86 (0.48, 1.54)	0.97 (0.54, 1.72)	0.97 (0.54, 1.73)	0.89 (0.53, 1.48)	0.82 (0.48, 1.37)	0.81 (0.48, 1.35)	0.88 (0.55, 1.40)	0.87 (0.52, 1.42)	0.79 (0.47, 1.33)	0.79 (0.48, 1.31)	0.79 (0.48, 1.28)	0.81 (0.47, 1.38)	0.77 (0.47, 1.25)	0.60 (0.18, 2.01)	1.34 (0.70, 2.80)
ELD10mg_kgIV	1.10 (0.68, 1.77)	1.24 (0.88, 1.76)	1.09 (0.72, 1.64)	1.28 (0.86, 1.91)	1.16 (0.76, 1.74)	1.22 (0.78, 1.96)	1.24 (0.78, 2.03)	1.17 (0.72, 1.91)	1.32 (0.83, 2.13)	1.31 (0.83, 2.13)	1.21 (0.82, 1.79)	1.11 (0.75, 1.67)	1.10 (0.74, 1.65)	1.20 (0.86, 1.67)	1.18 (0.81, 1.75)	1.07 (0.72, 1.59)	1.07 (0.74, 1.59)	1.07 (0.76, 1.56)	1.11 (0.73, 1.68)	1.05 (0.74, 1.51)	0.83 (0.25, 2.79)	**1.82 (1.06, 3.57)**
ELD20mg_kgIV	1.23 (0.75, 2.02)	1.38 (0.96, 2.02)	1.22 (0.80, 1.90)	1.42 (0.94, 2.20)	1.28 (0.84, 2.01)	1.37 (0.86, 2.25)	1.39 (0.86, 2.28)	1.30 (0.80, 2.15)	1.47 (0.91, 2.43)	1.47 (0.92, 2.40)	1.34 (0.91, 2.05)	1.24 (0.83, 1.91)	1.23 (0.82, 1.88)	1.33 (0.95, 1.93)	1.31 (0.89, 1.98)	1.20 (0.79, 1.83)	1.20 (0.81, 1.83)	1.20 (0.83, 1.77)	1.23 (0.81, 1.93)	1.17 (0.81, 1.73)	0.93 (0.26, 3.01)	**2.04 (1.16, 4.02)**
ETA25mgSC	0.61 (0.31, 1.13)	0.69 (0.37, 1.16)	0.61 (0.31, 1.07)	0.72 (0.37, 1.25)	0.64 (0.33, 1.14)	0.68 (0.34, 1.25)	0.69 (0.35, 1.29)	0.65 (0.32, 1.22)	0.73 (0.36, 1.38)	0.73 (0.37, 1.39)	0.67 (0.35, 1.17)	0.62 (0.32, 1.08)	0.61 (0.32, 1.08)	0.67 (0.36, 1.11)	0.66 (0.34, 1.13)	0.60 (0.31, 1.03)	0.60 (0.31, 1.03)	0.60 (0.31, 1.02)	0.62 (0.32, 1.09)	**0.59 (0.31, 0.99)**	0.46 (0.12, 1.62)	1.02 (0.47, 2.17)
ETR105mgSC	0.73 (0.46, 1.11)	0.82 (0.61, 1.09)	0.72 (0.51, 1.03)	0.85 (0.60, 1.20)	0.77 (0.53, 1.09)	0.81 (0.54, 1.25)	0.83 (0.54, 1.27)	0.77 (0.50, 1.21)	0.87 (0.58, 1.35)	0.88 (0.57, 1.34)	0.80 (0.57, 1.12)	0.74 (0.52, 1.02)	0.73 (0.51, 1.03)	0.80 (0.61, 1.03)	0.78 (0.56, 1.07)	0.71 (0.50, 1.00)	**0.71 (0.51, 0.99)**	**0.71 (0.53, 0.97)**	0.73 (0.51, 1.05)	**0.70 (0.51, 0.94)**	0.56 (0.16, 1.72)	1.21 (0.71, 2.28)
ETR210mgSC	0.83 (0.52, 1.27)	0.93 (0.69, 1.26)	0.82 (0.57, 1.18)	0.96 (0.68, 1.37)	0.87 (0.60, 1.25)	0.92 (0.60, 1.42)	0.93 (0.61, 1.45)	0.88 (0.56, 1.39)	0.99 (0.65, 1.53)	0.99 (0.65, 1.53)	0.91 (0.64, 1.28)	0.84 (0.59, 1.17)	0.83 (0.58, 1.17)	0.90 (0.68, 1.18)	0.88 (0.64, 1.24)	0.81 (0.56, 1.15)	0.81 (0.57, 1.14)	0.81 (0.59, 1.12)	0.83 (0.58, 1.20)	0.79 (0.58, 1.07)	0.62 (0.18, 1.95)	1.37 (0.81, 2.62)
FIL100mgPO	0.93 (0.61, 1.35)	1.04 (0.83, 1.32)	0.92 (0.67, 1.25)	1.07 (0.81, 1.45)	0.97 (0.71, 1.33)	1.03 (0.71, 1.53)	1.05 (0.71, 1.54)	0.98 (0.66, 1.48)	1.11 (0.76, 1.65)	1.11 (0.76, 1.62)	1.02 (0.76, 1.36)	0.94 (0.69, 1.25)	0.93 (0.69, 1.25)	1.01 (0.83, 1.23)	0.99 (0.76, 1.30)	0.90 (0.66, 1.20)	0.90 (0.69, 1.20)	0.90 (0.71, 1.16)	0.93 (0.68, 1.27)	0.88 (0.70, 1.12)	0.71 (0.21, 2.16)	1.54 (0.93, 2.84)
FIL200mgPO	0.90 (0.59, 1.31)	1.01 (0.81, 1.27)	0.89 (0.65, 1.21)	1.04 (0.78, 1.40)	0.94 (0.68, 1.28)	1.00 (0.68, 1.48)	1.01 (0.69, 1.49)	0.95 (0.63, 1.43)	1.07 (0.74, 1.60)	1.07 (0.74, 1.57)	0.98 (0.73, 1.32)	0.90 (0.67, 1.21)	0.90 (0.66, 1.20)	0.98 (0.80, 1.19)	0.96 (0.73, 1.25)	0.88 (0.64, 1.17)	0.87 (0.66, 1.16)	0.88 (0.69, 1.12)	0.90 (0.65, 1.23)	0.86 (0.67, 1.08)	0.68 (0.20, 2.09)	1.49 (0.90, 2.76)
FON1mg_kg0_1mgIV_SC	1.05 (0.63, 1.69)	1.18 (0.83, 1.69)	1.04 (0.68, 1.56)	1.22 (0.81, 1.82)	1.09 (0.72, 1.66)	1.16 (0.73, 1.88)	1.18 (0.74, 1.89)	1.12 (0.67, 1.81)	1.26 (0.77, 2.02)	1.26 (0.78, 2.00)	1.15 (0.77, 1.71)	1.05 (0.71, 1.58)	1.05 (0.68, 1.58)	1.14 (0.81, 1.60)	1.12 (0.76, 1.64)	1.01 (0.67, 1.52)	1.02 (0.69, 1.51)	1.02 (0.71, 1.48)	1.05 (0.69, 1.61)	1.00 (0.69, 1.44)	0.79 (0.23, 2.56)	1.73 (1.00, 3.40)
FON1mg_kg1mgIV_SC	1.15 (0.69, 1.92)	1.29 (0.88, 1.96)	1.14 (0.74, 1.82)	1.34 (0.89, 2.09)	1.21 (0.77, 1.92)	1.28 (0.80, 2.13)	1.30 (0.80, 2.16)	1.23 (0.74, 2.09)	1.38 (0.84, 2.27)	1.38 (0.85, 2.32)	1.26 (0.84, 1.96)	1.16 (0.76, 1.82)	1.15 (0.75, 1.79)	1.25 (0.88, 1.86)	1.23 (0.82, 1.90)	1.12 (0.73, 1.75)	1.12 (0.74, 1.74)	1.12 (0.76, 1.73)	1.16 (0.75, 1.85)	1.10 (0.74, 1.67)	0.88 (0.26, 2.88)	**1.92 (1.07, 3.88)**
FON4mg_kg0_1mgIV_SC	0.89 (0.55, 1.41)	1.00 (0.71, 1.39)	0.88 (0.59, 1.30)	1.03 (0.70, 1.50)	0.93 (0.62, 1.38)	0.98 (0.63, 1.56)	1.00 (0.63, 1.57)	0.94 (0.58, 1.51)	1.06 (0.68, 1.70)	1.06 (0.67, 1.67)	0.98 (0.67, 1.42)	0.90 (0.61, 1.31)	0.89 (0.60, 1.30)	0.97 (0.70, 1.32)	0.95 (0.65, 1.37)	0.87 (0.58, 1.26)	0.87 (0.59, 1.25)	0.87 (0.61, 1.22)	0.89 (0.59, 1.32)	0.85 (0.60, 1.19)	0.67 (0.19, 2.13)	1.47 (0.83, 2.85)
FON4mg_kg1mgIV_SC	0.95 (0.58, 1.49)	1.07 (0.76, 1.52)	0.94 (0.63, 1.43)	1.10 (0.75, 1.65)	0.99 (0.66, 1.52)	1.06 (0.67, 1.68)	1.07 (0.67, 1.72)	1.01 (0.61, 1.67)	1.13 (0.72, 1.84)	1.13 (0.72, 1.83)	1.04 (0.71, 1.54)	0.95 (0.65, 1.40)	0.94 (0.64, 1.41)	1.03 (0.75, 1.44)	1.01 (0.70, 1.49)	0.92 (0.62, 1.37)	0.92 (0.63, 1.37)	0.92 (0.66, 1.32)	0.95 (0.64, 1.44)	0.90 (0.64, 1.29)	0.71 (0.21, 2.25)	1.57 (0.91, 3.07)
GUS1200mgIV	1.29 (0.79, 2.12)	**1.46 (1.01, 2.15)**	1.28 (0.84, 1.98)	1.51 (1.00, 2.32)	1.36 (0.88, 2.12)	1.44 (0.90, 2.41)	1.47 (0.89, 2.49)	1.38 (0.82, 2.35)	1.55 (0.96, 2.55)	1.55 (0.96, 2.58)	1.41 (0.94, 2.17)	1.31 (0.86, 2.01)	1.29 (0.85, 1.99)	1.41 (0.99, 2.04)	1.38 (0.93, 2.11)	1.26 (0.83, 1.93)	1.26 (0.84, 1.92)	1.27 (0.87, 1.89)	1.30 (0.85, 2.03)	1.24 (0.84, 1.85)	0.98 (0.29, 3.24)	**2.16 (1.20, 4.32)**
GUS200mgIV	1.25 (0.76, 2.04)	1.41 (0.97, 2.07)	1.25 (0.81, 1.91)	1.45 (0.96, 2.21)	1.32 (0.86, 2.02)	1.40 (0.87, 2.27)	1.42 (0.87, 2.30)	1.34 (0.81, 2.20)	1.50 (0.94, 2.44)	1.50 (0.94, 2.43)	1.38 (0.91, 2.08)	1.26 (0.83, 1.93)	1.25 (0.82, 1.92)	1.37 (0.96, 1.96)	1.34 (0.90, 2.01)	1.23 (0.80, 1.86)	1.22 (0.82, 1.85)	1.23 (0.84, 1.80)	1.26 (0.82, 1.94)	1.20 (0.82, 1.75)	0.95 (0.27, 3.09)	**2.09 (1.17, 4.15)**
GUS600mgIV	1.08 (0.66, 1.74)	1.22 (0.86, 1.75)	1.07 (0.72, 1.63)	1.26 (0.85, 1.88)	1.14 (0.75, 1.71)	1.20 (0.77, 1.97)	1.22 (0.77, 1.96)	1.15 (0.70, 1.88)	1.30 (0.82, 2.07)	1.29 (0.83, 2.07)	1.19 (0.81, 1.77)	1.10 (0.74, 1.63)	1.08 (0.72, 1.62)	1.18 (0.85, 1.66)	1.16 (0.79, 1.71)	1.06 (0.71, 1.58)	1.06 (0.72, 1.56)	1.06 (0.74, 1.55)	1.09 (0.72, 1.65)	1.03 (0.72, 1.49)	0.82 (0.24, 2.65)	**1.79 (1.02, 3.58)**
MED700IV	0.93 (0.57, 1.44)	1.04 (0.76, 1.43)	0.92 (0.63, 1.33)	1.07 (0.75, 1.56)	0.97 (0.66, 1.42)	1.03 (0.67, 1.60)	1.04 (0.68, 1.63)	0.98 (0.62, 1.55)	1.11 (0.72, 1.74)	1.11 (0.72, 1.73)	1.02 (0.71, 1.47)	0.93 (0.65, 1.36)	0.92 (0.64, 1.34)	1.01 (0.76, 1.36)	0.99 (0.70, 1.42)	0.90 (0.62, 1.31)	0.90 (0.63, 1.29)	0.90 (0.66, 1.26)	0.93 (0.64, 1.36)	0.88 (0.64, 1.21)	0.71 (0.21, 2.18)	1.54 (0.89, 2.95)
MIR1000mgIV	0.99 (0.67, 1.38)	1.11 (0.82, 1.51)	0.98 (0.68, 1.41)	1.14 (0.80, 1.64)	1.04 (0.71, 1.50)	1.10 (0.71, 1.69)	1.11 (0.73, 1.75)	1.05 (0.67, 1.64)	1.18 (0.77, 1.83)	1.18 (0.78, 1.84)	1.08 (0.76, 1.55)	1.00 (0.69, 1.41)	0.98 (0.69, 1.41)	1.07 (0.81, 1.43)	1.05 (0.76, 1.49)	0.96 (0.67, 1.39)	0.96 (0.68, 1.37)	0.96 (0.70, 1.34)	0.99 (0.68, 1.44)	0.94 (0.69, 1.29)	0.75 (0.22, 2.35)	1.64 (0.96, 3.09)
MIR200mgIV	1.14 (0.73, 1.79)	1.28 (0.88, 1.97)	1.13 (0.74, 1.79)	1.32 (0.87, 2.09)	1.19 (0.77, 1.90)	1.27 (0.77, 2.16)	1.28 (0.79, 2.19)	1.20 (0.73, 2.13)	1.35 (0.85, 2.37)	1.36 (0.84, 2.33)	1.25 (0.82, 1.98)	1.14 (0.75, 1.83)	1.14 (0.74, 1.81)	1.24 (0.87, 1.88)	1.22 (0.81, 1.92)	1.10 (0.72, 1.77)	1.11 (0.74, 1.75)	1.11 (0.76, 1.74)	1.15 (0.74, 1.84)	1.08 (0.74, 1.68)	0.86 (0.25, 2.79)	**1.90 (1.06, 3.76)**

(TABLE S16B. continued)

	MIR600mgIV	NAT300mgIV	NAT3mg_kgIV	NAT3mg_kgIVx2	NAT6mg_kgIVx2	NNC2mg_kgSC	ONE10mgSC	ONE25mgSC	ONE35mgSC	ONE50mgSC	ONT22_5mgSC	ONT225mgSC	ONT75mgSC	PBO	PF10mgSC	PF200mgSC	PF50mgSC	RIS1200mgIV	RIS200mgIV	RIS600mgIV	RIS600mgIV4_4	SEC10mg_kgIV
MIR600mgIV	MIR600mgIV	1.12 (0.80, 1.66)	0.99 (0.66, 1.53)	1.16 (0.79, 1.79)	1.05 (0.69, 1.62)	1.11 (0.71, 1.83)	1.13 (0.71, 1.84)	1.06 (0.65, 1.77)	1.19 (0.76, 1.93)	1.20 (0.76, 1.94)	1.10 (0.74, 1.67)	1.01 (0.68, 1.55)	1.00 (0.67, 1.53)	1.08 (0.79, 1.58)	1.07 (0.74, 1.62)	0.97 (0.65, 1.51)	0.97 (0.67, 1.48)	0.98 (0.68, 1.46)	1.00 (0.66, 1.57)	0.95 (0.67, 1.41)	0.76 (0.22, 2.44)	1.67 (0.94, 3.23)
NAT300mgIV	0.89 (0.60, 1.25)	NAT300mgIV	0.88 (0.68, 1.15)	1.03 (0.81, 1.33)	0.93 (0.70, 1.22)	0.98 (0.70, 1.42)	1.00 (0.71, 1.42)	0.94 (0.65, 1.36)	1.06 (0.75, 1.52)	1.06 (0.76, 1.51)	0.97 (0.77, 1.24)	0.90 (0.70, 1.14)	0.89 (0.68, 1.14)	0.97 (0.86, 1.09)	0.95 (0.76, 1.20)	0.87 (0.66, 1.10)	0.87 (0.69, 1.10)	0.87 (0.72, 1.06)	0.89 (0.67, 1.17)	0.85 (0.71, 1.01)	0.67 (0.20, 2.05)	1.47 (0.93, 2.67)
NAT3mg_kgIV	1.01 (0.65, 1.52)	1.14 (0.87, 1.48)	NAT3mg_kgIV	1.17 (0.94, 1.48)	1.06 (0.81, 1.36)	1.12 (0.75, 1.70)	1.14 (0.74, 1.71)	1.07 (0.69, 1.64)	1.21 (0.81, 1.82)	1.21 (0.81, 1.81)	1.11 (0.80, 1.53)	1.02 (0.73, 1.41)	1.01 (0.72, 1.39)	1.10 (0.86, 1.40)	1.08 (0.79, 1.46)	0.98 (0.70, 1.35)	0.98 (0.72, 1.34)	0.99 (0.75, 1.31)	1.01 (0.71, 1.43)	0.96 (0.73, 1.26)	0.76 (0.23, 2.39)	1.69 (0.99, 3.09)
NAT3mg_kgIVx2	0.86 (0.56, 1.27)	0.97 (0.75, 1.23)	0.86 (0.67, 1.07)	NAT3mg_kgIVx2	0.90 (0.71, 1.13)	0.96 (0.64, 1.44)	0.97 (0.65, 1.45)	0.92 (0.60, 1.38)	1.03 (0.69, 1.53)	1.03 (0.69, 1.53)	0.95 (0.70, 1.28)	0.87 (0.63, 1.17)	0.86 (0.62, 1.17)	0.94 (0.75, 1.16)	0.92 (0.69, 1.22)	0.84 (0.61, 1.13)	0.84 (0.63, 1.12)	0.84 (0.65, 1.09)	0.87 (0.61, 1.19)	0.82 (0.63, 1.05)	0.65 (0.19, 2.05)	1.44 (0.86, 2.63)
NAT6mg_kgIVx2	0.96 (0.62, 1.44)	1.07 (0.82, 1.43)	0.94 (0.74, 1.23)	1.11 (0.88, 1.41)	NAT6mg_kgIVx2	1.06 (0.71, 1.62)	1.07 (0.72, 1.65)	1.02 (0.65, 1.58)	1.14 (0.77, 1.74)	1.14 (0.76, 1.73)	1.05 (0.77, 1.46)	0.96 (0.69, 1.35)	0.95 (0.68, 1.33)	1.04 (0.82, 1.34)	1.02 (0.75, 1.39)	0.93 (0.66, 1.29)	0.93 (0.68, 1.28)	0.93 (0.70, 1.25)	0.96 (0.67, 1.36)	0.91 (0.69, 1.22)	0.72 (0.21, 2.27)	1.59 (0.95, 2.96)
NNC2mg_kgSC	0.90 (0.55, 1.42)	1.02 (0.71, 1.42)	0.89 (0.59, 1.33)	1.05 (0.69, 1.55)	0.94 (0.62, 1.41)	NNC2mg_kgSC	1.02 (0.64, 1.63)	0.95 (0.58, 1.55)	1.07 (0.67, 1.73)	1.08 (0.68, 1.72)	0.99 (0.67, 1.46)	0.91 (0.60, 1.33)	0.90 (0.60, 1.32)	0.98 (0.69, 1.35)	0.96 (0.65, 1.40)	0.88 (0.58, 1.30)	0.87 (0.59, 1.29)	0.88 (0.61, 1.25)	0.90 (0.60, 1.36)	0.86 (0.59, 1.22)	0.68 (0.20, 2.19)	1.50 (0.84, 2.88)
ONE10mgSC	0.89 (0.54, 1.41)	1.00 (0.71, 1.41)	0.88 (0.58, 1.35)	1.03 (0.69, 1.54)	0.93 (0.61, 1.40)	0.99 (0.61, 1.57)	ONE10mgSC	0.94 (0.67, 1.31)	1.06 (0.77, 1.45)	1.06 (0.78, 1.44)	0.97 (0.66, 1.43)	0.90 (0.60, 1.32)	0.89 (0.59, 1.31)	0.96 (0.69, 1.33)	0.95 (0.65, 1.38)	0.86 (0.58, 1.27)	0.86 (0.58, 1.26)	0.87 (0.60, 1.23)	0.89 (0.59, 1.34)	0.84 (0.59, 1.19)	0.67 (0.20, 2.19)	1.48 (0.81, 2.84)
ONE25mgSC	0.94 (0.56, 1.54)	1.06 (0.73, 1.55)	0.93 (0.61, 1.44)	1.09 (0.73, 1.68)	0.98 (0.63, 1.53)	1.05 (0.65, 1.71)	1.06 (0.76, 1.50)	ONE25mgSC	1.13 (0.81, 1.59)	1.12 (0.81, 1.59)	1.03 (0.69, 1.57)	0.95 (0.63, 1.44)	0.94 (0.62, 1.43)	1.02 (0.72, 1.46)	1.01 (0.67, 1.50)	0.92 (0.60, 1.39)	0.92 (0.61, 1.38)	0.92 (0.63, 1.36)	0.95 (0.61, 1.45)	0.90 (0.62, 1.31)	0.71 (0.20, 2.29)	1.57 (0.84, 3.10)
ONE35mgSC	0.84 (0.52, 1.32)	0.94 (0.66, 1.33)	0.83 (0.55, 1.24)	0.97 (0.65, 1.44)	0.88 (0.57, 1.31)	0.93 (0.58, 1.48)	0.94 (0.69, 1.30)	0.89 (0.63, 1.24)	ONE35mgSC	1.00 (0.74, 1.36)	0.92 (0.61, 1.35)	0.85 (0.57, 1.24)	0.84 (0.55, 1.23)	0.91 (0.65, 1.26)	0.90 (0.61, 1.30)	0.81 (0.54, 1.20)	0.82 (0.55, 1.20)	0.82 (0.57, 1.17)	0.84 (0.55, 1.26)	0.80 (0.56, 1.13)	0.63 (0.18, 2.04)	1.40 (0.76, 2.73)
ONE50mgSC	0.83 (0.52, 1.32)	0.94 (0.66, 1.32)	0.83 (0.55, 1.24)	0.97 (0.65, 1.45)	0.88 (0.58, 1.31)	0.93 (0.58, 1.48)	0.94 (0.69, 1.29)	0.89 (0.63, 1.23)	1.00 (0.73, 1.36)	ONE50mgSC	0.92 (0.62, 1.34)	0.84 (0.57, 1.25)	0.83 (0.56, 1.24)	0.91 (0.65, 1.25)	0.89 (0.61, 1.29)	0.82 (0.54, 1.20)	0.82 (0.55, 1.18)	0.82 (0.57, 1.16)	0.84 (0.55, 1.25)	0.80 (0.56, 1.13)	0.63 (0.18, 2.02)	1.40 (0.77, 2.71)
ONT22_5mgSC	0.91 (0.60, 1.34)	1.03 (0.81, 1.30)	0.90 (0.65, 1.24)	1.06 (0.78, 1.44)	0.96 (0.69, 1.30)	1.01 (0.68, 1.50)	1.03 (0.70, 1.52)	0.97 (0.64, 1.46)	1.08 (0.74, 1.63)	1.09 (0.74, 1.62)	ONT22_5mgSC	0.92 (0.74, 1.15)	0.91 (0.72, 1.14)	0.99 (0.80, 1.23)	0.98 (0.73, 1.30)	0.89 (0.64, 1.20)	0.89 (0.66, 1.20)	0.89 (0.69, 1.16)	0.92 (0.66, 1.26)	0.87 (0.68, 1.12)	0.69 (0.20, 2.14)	1.51 (0.92, 2.83)
ONT225mgSC	0.99 (0.64, 1.47)	1.12 (0.87, 1.43)	0.98 (0.71, 1.36)	1.15 (0.85, 1.58)	1.04 (0.74, 1.44)	1.10 (0.75, 1.66)	1.12 (0.75, 1.66)	1.05 (0.69, 1.59)	1.18 (0.81, 1.77)	1.19 (0.80, 1.77)	1.09 (0.87, 1.36)	ONT225mgSC	0.99 (0.78, 1.25)	1.08 (0.87, 1.35)	1.06 (0.79, 1.43)	0.96 (0.70, 1.32)	0.97 (0.72, 1.31)	0.97 (0.75, 1.26)	0.99 (0.71, 1.39)	0.94 (0.73, 1.22)	0.75 (0.22, 2.34)	1.64 (0.99, 3.07)
ONT75mgSC	1.00 (0.65, 1.49)	1.13 (0.88, 1.46)	0.99 (0.72, 1.38)	1.16 (0.86, 1.62)	1.05 (0.75, 1.48)	1.12 (0.76, 1.67)	1.13 (0.76, 1.71)	1.07 (0.70, 1.62)	1.19 (0.81, 1.81)	1.20 (0.81, 1.80)	1.10 (0.88, 1.38)	1.01 (0.80, 1.28)	ONT75mgSC	1.09 (0.87, 1.37)	1.07 (0.80, 1.45)	0.98 (0.71, 1.34)	0.98 (0.72, 1.33)	0.98 (0.75, 1.30)	1.01 (0.71, 1.41)	0.95 (0.74, 1.25)	0.76 (0.22, 2.37)	1.66 (0.99, 3.12)
PBO	0.92 (0.63, 1.27)	1.03 (0.92, 1.16)	0.91 (0.72, 1.16)	1.06 (0.86, 1.34)	0.96 (0.75, 1.22)	1.02 (0.74, 1.44)	1.04 (0.75, 1.45)	0.98 (0.68, 1.39)	1.10 (0.79, 1.54)	1.10 (0.80, 1.54)	1.01 (0.82, 1.25)	0.93 (0.74, 1.15)	0.92 (0.73, 1.14)	PBO	0.98 (0.81, 1.19)	0.90 (0.70, 1.11)	0.90 (0.73, 1.09)	0.90 (0.78, 1.05)	0.92 (0.71, 1.17)	0.88 (0.76, 1.00)	0.70 (0.21, 2.11)	1.52 (0.97, 2.74)
PF10mgSC	0.94 (0.62, 1.35)	1.05 (0.84, 1.32)	0.93 (0.68, 1.26)	1.09 (0.82, 1.45)	0.98 (0.72, 1.33)	1.04 (0.72, 1.53)	1.06 (0.73, 1.55)	0.99 (0.67, 1.48)	1.11 (0.77, 1.63)	1.12 (0.78, 1.64)	1.03 (0.77, 1.37)	0.94 (0.70, 1.26)	0.93 (0.69, 1.25)	1.02 (0.84, 1.23)	PF10mgSC	0.91 (0.71, 1.14)	0.91 (0.74, 1.12)	0.92 (0.72, 1.17)	0.94 (0.68, 1.28)	0.89 (0.71, 1.12)	0.70 (0.21, 2.18)	1.55 (0.95, 2.86)
PF200mgSC	1.03 (0.66, 1.55)	1.15 (0.91, 1.51)	1.02 (0.74, 1.43)	1.19 (0.89, 1.64)	1.08 (0.77, 1.51)	1.14 (0.77, 1.72)	1.16 (0.79, 1.73)	1.09 (0.72, 1.67)	1.23 (0.83, 1.85)	1.22 (0.83, 1.86)	1.13 (0.83, 1.55)	1.04 (0.76, 1.43)	1.02 (0.74, 1.41)	1.11 (0.90, 1.42)	1.09 (0.88, 1.41)	PF200mgSC	1.00 (0.79, 1.29)	1.00 (0.77, 1.34)	1.03 (0.74, 1.45)	0.98 (0.76, 1.29)	0.78 (0.23, 2.38)	**1.70 (1.03, 3.21)**
PF50mgSC	1.03 (0.68, 1.50)	1.15 (0.91, 1.46)	1.02 (0.75, 1.39)	1.19 (0.89, 1.60)	1.08 (0.78, 1.48)	1.14 (0.78, 1.69)	1.16 (0.79, 1.72)	1.09 (0.72, 1.63)	1.22 (0.83, 1.83)	1.23 (0.85, 1.82)	1.12 (0.83, 1.51)	1.03 (0.76, 1.40)	1.02 (0.75, 1.39)	1.12 (0.91, 1.36)	1.10 (0.89, 1.36)	1.00 (0.77, 1.26)	PF50mgSC	1.00 (0.79, 1.29)	1.03 (0.75, 1.41)	0.98 (0.77, 1.24)	0.77 (0.23, 2.42)	**1.70 (1.04, 3.15)**
RIS1200mgIV	1.02 (0.68, 1.47)	1.15 (0.95, 1.39)	1.01 (0.76, 1.33)	1.19 (0.91, 1.54)	1.07 (0.80, 1.42)	1.14 (0.80, 1.64)	1.15 (0.81, 1.66)	1.09 (0.73, 1.59)	1.22 (0.85, 1.76)	1.22 (0.86, 1.75)	1.12 (0.86, 1.45)	1.03 (0.79, 1.33)	1.02 (0.77, 1.33)	1.11 (0.96, 1.28)	1.09 (0.85, 1.39)	1.00 (0.75, 1.29)	1.00 (0.77, 1.27)	RIS1200mgIV	1.03 (0.77, 1.34)	0.98 (0.83, 1.12)	0.77 (0.23, 2.37)	**1.69 (1.06, 3.08)**
RIS200mgIV	1.00 (0.64, 1.51)	1.12 (0.85, 1.49)	0.99 (0.70, 1.40)	1.15 (0.84, 1.63)	1.04 (0.74, 1.49)	1.11 (0.74, 1.67)	1.12 (0.75, 1.71)	1.06 (0.69, 1.64)	1.19 (0.80, 1.81)	1.19 (0.80, 1.81)	1.09 (0.80, 1.53)	1.01 (0.72, 1.41)	0.99 (0.71, 1.41)	1.08 (0.85, 1.40)	1.06 (0.78, 1.47)	0.97 (0.69, 1.35)	0.97 (0.71, 1.33)	0.97 (0.74, 1.29)	RIS200mgIV	0.95 (0.74, 1.24)	0.75 (0.22, 2.35)	1.65 (0.99, 3.07)
RIS600mgIV	1.05 (0.71, 1.49)	1.18 (0.99, 1.41)	1.04 (0.79, 1.36)	1.22 (0.95, 1.58)	1.10 (0.82, 1.45)	1.16 (0.82, 1.68)	1.19 (0.84, 1.69)	1.11 (0.76, 1.62)	1.25 (0.89, 1.80)	1.25 (0.89, 1.80)	1.15 (0.90, 1.48)	1.06 (0.82, 1.37)	1.05 (0.80, 1.36)	1.14 (1.00, 1.31)	1.12 (0.89, 1.41)	1.02 (0.78, 1.32)	1.02 (0.81, 1.31)	1.02 (0.89, 1.20)	1.06 (0.81, 1.35)	RIS600mgIV	0.79 (0.24, 2.42)	**1.74 (1.09, 3.16)**
RIS600mgIV4_4	1.32 (0.41, 4.52)	1.49 (0.49, 4.98)	1.32 (0.42, 4.44)	1.53 (0.49, 5.15)	1.38 (0.44, 4.74)	1.46 (0.46, 5.04)	1.50 (0.46, 5.12)	1.40 (0.44, 4.89)	1.58 (0.49, 5.55)	1.58 (0.50, 5.55)	1.46 (0.47, 4.96)	1.33 (0.43, 4.52)	1.32 (0.42, 4.48)	1.44 (0.47, 4.79)	1.42 (0.46, 4.80)	1.28 (0.42, 4.29)	1.30 (0.41, 4.31)	1.29 (0.42, 4.32)	1.33 (0.43, 4.46)	1.26 (0.41, 4.18)	RIS600mgIV4_4	2.24 (0.65, 8.54)
SEC10mg_kgIV	0.60 (0.31, 1.06)	0.68 (0.37, 1.08)	0.59 (0.32, 1.01)	0.70 (0.38, 1.17)	0.63 (0.34, 1.05)	0.67 (0.35, 1.19)	0.67 (0.35, 1.23)	0.64 (0.32, 1.19)	0.72 (0.37, 1.31)	0.72 (0.37, 1.29)	0.66 (0.35, 1.09)	0.61 (0.33, 1.01)	0.60 (0.32, 1.01)	0.66 (0.37, 1.03)	0.64 (0.35, 1.05)	**0.59 (0.31, 0.97)**	**0.59 (0.32, 0.96)**	**0.59 (0.32, 0.94)**	0.60 (0.33, 1.01)	**0.58 (0.32, 0.92)**	0.45 (0.12, 1.54)	SEC10mg_kgIV

	MIR600mgIV	NAT300mgIV	NAT3mg_kgIV	NAT3mg_kgIVx2	NAT6mg_kgIVx2	NNC2mg_kgSC	ONE10mgSC	ONE25mgSC	ONE35mgSC	ONE50mgSC	ONT22_5mgSC	ONT225mgSC	ONT75mgSC	PBO	PF10mgSC	PF200mgSC	PF50mgSC	RIS1200mgIV	RIS200mgIV	RIS600mgIV	RIS600mgIV4_4	SEC10mg_kgIV
SEM60mgIV	0.80 (0.48, 1.30)	0.90 (0.61, 1.30)	0.79 (0.51, 1.21)	0.92 (0.61, 1.42)	0.83 (0.54, 1.29)	0.88 (0.54, 1.46)	0.90 (0.55, 1.45)	0.85 (0.51, 1.38)	0.95 (0.59, 1.55)	0.96 (0.58, 1.53)	0.87 (0.58, 1.32)	0.80 (0.52, 1.21)	0.79 (0.52, 1.21)	0.87 (0.60, 1.25)	0.85 (0.56, 1.28)	0.77 (0.50, 1.18)	0.78 (0.51, 1.17)	0.78 (0.52, 1.15)	0.80 (0.51, 1.23)	0.76 (0.51, 1.10)	0.60 (0.18, 1.97)	1.32 (0.74, 2.65)
SEM60mgIVx3	0.71 (0.43, 1.14)	0.80 (0.55, 1.12)	0.70 (0.46, 1.06)	0.82 (0.54, 1.21)	0.74 (0.48, 1.11)	0.79 (0.49, 1.25)	0.80 (0.49, 1.26)	0.76 (0.46, 1.21)	0.85 (0.53, 1.34)	0.85 (0.53, 1.34)	0.78 (0.52, 1.15)	0.72 (0.47, 1.06)	0.71 (0.47, 1.06)	0.77 (0.54, 1.07)	0.76 (0.51, 1.11)	0.69 (0.45, 1.03)	0.69 (0.46, 1.02)	0.69 (0.48, 1.00)	0.71 (0.46, 1.06)	**0.68 (0.46, 0.96)**	0.53 (0.16, 1.73)	1.18 (0.65, 2.33)
TES400mg200mgSC	0.72 (0.44, 1.14)	0.81 (0.57, 1.15)	0.71 (0.47, 1.07)	0.84 (0.56, 1.24)	0.76 (0.50, 1.13)	0.80 (0.50, 1.28)	0.81 (0.50, 1.30)	0.76 (0.47, 1.25)	0.86 (0.53, 1.37)	0.86 (0.54, 1.38)	0.79 (0.53, 1.16)	0.72 (0.48, 1.08)	0.72 (0.48, 1.07)	0.78 (0.56, 1.08)	0.77 (0.53, 1.12)	0.70 (0.47, 1.04)	0.70 (0.48, 1.02)	0.70 (0.48, 1.01)	0.72 (0.47, 1.09)	**0.69 (0.48, 0.97)**	0.55 (0.16, 1.74)	1.19 (0.68, 2.32)
TOF15mgOR_BID	0.96 (0.55, 1.61)	1.08 (0.70, 1.63)	0.95 (0.59, 1.53)	1.11 (0.70, 1.75)	1.00 (0.62, 1.60)	1.06 (0.63, 1.78)	1.08 (0.64, 1.78)	1.02 (0.59, 1.72)	1.14 (0.68, 1.93)	1.14 (0.67, 1.89)	1.05 (0.66, 1.62)	0.96 (0.60, 1.53)	0.95 (0.60, 1.48)	1.04 (0.69, 1.55)	1.02 (0.65, 1.57)	0.93 (0.58, 1.46)	0.93 (0.59, 1.45)	0.93 (0.61, 1.42)	0.95 (0.60, 1.53)	0.91 (0.60, 1.39)	0.72 (0.20, 2.31)	1.59 (0.85, 3.16)
TOF1mgOR_BID	1.20 (0.67, 2.07)	1.34 (0.85, 2.14)	1.18 (0.71, 1.95)	1.39 (0.85, 2.29)	1.25 (0.75, 2.09)	1.33 (0.76, 2.34)	1.35 (0.76, 2.37)	1.27 (0.71, 2.25)	1.43 (0.82, 2.50)	1.44 (0.81, 2.50)	1.31 (0.80, 2.17)	1.20 (0.72, 2.01)	1.19 (0.71, 1.96)	1.30 (0.83, 2.04)	1.28 (0.78, 2.08)	1.16 (0.71, 1.91)	1.16 (0.71, 1.92)	1.17 (0.73, 1.87)	1.21 (0.72, 1.97)	1.14 (0.71, 1.83)	0.90 (0.25, 3.08)	**1.99 (1.05, 4.12)**
TOF5mgOR_BID	0.97 (0.56, 1.64)	1.09 (0.72, 1.65)	0.96 (0.60, 1.54)	1.13 (0.71, 1.80)	1.01 (0.64, 1.62)	1.09 (0.64, 1.83)	1.09 (0.66, 1.84)	1.03 (0.60, 1.75)	1.16 (0.69, 1.95)	1.16 (0.69, 1.96)	1.06 (0.68, 1.68)	0.98 (0.62, 1.55)	0.97 (0.61, 1.53)	1.05 (0.71, 1.58)	1.03 (0.67, 1.60)	0.95 (0.59, 1.49)	0.94 (0.61, 1.48)	0.95 (0.62, 1.46)	0.98 (0.60, 1.55)	0.92 (0.61, 1.41)	0.73 (0.21, 2.38)	1.62 (0.87, 3.18)
UPA12mgOR_BID	0.83 (0.52, 1.30)	0.93 (0.67, 1.29)	0.82 (0.55, 1.20)	0.96 (0.66, 1.40)	0.87 (0.58, 1.30)	0.92 (0.58, 1.45)	0.93 (0.59, 1.48)	0.88 (0.54, 1.40)	0.99 (0.63, 1.54)	0.99 (0.64, 1.55)	0.91 (0.62, 1.32)	0.83 (0.57, 1.22)	0.82 (0.56, 1.21)	0.90 (0.66, 1.23)	0.88 (0.62, 1.28)	0.81 (0.54, 1.18)	0.81 (0.56, 1.17)	0.81 (0.58, 1.13)	0.83 (0.55, 1.24)	0.79 (0.56, 1.10)	0.63 (0.18, 2.01)	1.37 (0.80, 2.60)
UPA24mgOR	0.81 (0.50, 1.26)	0.91 (0.65, 1.25)	0.80 (0.54, 1.18)	0.94 (0.64, 1.36)	0.84 (0.57, 1.25)	0.90 (0.57, 1.41)	0.91 (0.58, 1.43)	0.86 (0.53, 1.35)	0.96 (0.62, 1.50)	0.96 (0.63, 1.50)	0.88 (0.60, 1.27)	0.81 (0.56, 1.18)	0.81 (0.55, 1.16)	0.88 (0.65, 1.18)	0.86 (0.60, 1.23)	0.79 (0.53, 1.14)	0.79 (0.54, 1.13)	0.79 (0.56, 1.10)	0.81 (0.55, 1.18)	0.77 (0.55, 1.06)	0.61 (0.18, 1.93)	1.34 (0.78, 2.55)
UPA24mgOR_BID	0.80 (0.50, 1.25)	0.90 (0.65, 1.24)	0.79 (0.54, 1.16)	0.93 (0.63, 1.35)	0.84 (0.56, 1.23)	0.89 (0.57, 1.39)	0.90 (0.57, 1.41)	0.85 (0.53, 1.34)	0.96 (0.61, 1.49)	0.96 (0.62, 1.49)	0.88 (0.60, 1.26)	0.81 (0.55, 1.16)	0.80 (0.55, 1.16)	0.87 (0.64, 1.18)	0.85 (0.59, 1.23)	0.78 (0.53, 1.13)	0.78 (0.53, 1.12)	0.78 (0.56, 1.09)	0.80 (0.54, 1.17)	0.76 (0.54, 1.06)	0.60 (0.18, 1.94)	1.33 (0.77, 2.53)
UPA3mgOR_BID	0.77 (0.48, 1.18)	0.86 (0.62, 1.18)	0.76 (0.52, 1.10)	0.89 (0.62, 1.26)	0.80 (0.54, 1.17)	0.85 (0.54, 1.34)	0.86 (0.55, 1.33)	0.81 (0.51, 1.26)	0.92 (0.59, 1.40)	0.91 (0.60, 1.40)	0.84 (0.58, 1.20)	0.77 (0.53, 1.12)	0.76 (0.52, 1.10)	0.83 (0.61, 1.11)	0.82 (0.57, 1.15)	0.75 (0.51, 1.07)	0.74 (0.52, 1.06)	0.75 (0.54, 1.03)	0.77 (0.52, 1.12)	0.73 (0.52, 1.01)	0.58 (0.17, 1.84)	1.27 (0.73, 2.41)
UPA45mgOR	0.87 (0.58, 1.25)	0.98 (0.81, 1.17)	0.86 (0.65, 1.14)	1.01 (0.78, 1.32)	0.91 (0.68, 1.20)	0.97 (0.68, 1.39)	0.98 (0.68, 1.41)	0.92 (0.62, 1.34)	1.04 (0.73, 1.49)	1.03 (0.73, 1.50)	0.95 (0.74, 1.23)	0.88 (0.67, 1.14)	0.87 (0.66, 1.13)	0.95 (0.81, 1.09)	0.93 (0.74, 1.17)	0.85 (0.64, 1.09)	0.85 (0.66, 1.09)	0.85 (0.69, 1.04)	0.87 (0.65, 1.15)	0.83 (0.68, 1.00)	0.66 (0.20, 2.01)	1.44 (0.90, 2.62)
UPA6mgOR_BID	0.85 (0.53, 1.33)	0.96 (0.68, 1.34)	0.84 (0.57, 1.24)	0.99 (0.67, 1.44)	0.89 (0.59, 1.33)	0.95 (0.60, 1.50)	0.96 (0.61, 1.52)	0.91 (0.57, 1.44)	1.02 (0.65, 1.60)	1.02 (0.66, 1.61)	0.94 (0.64, 1.36)	0.86 (0.59, 1.26)	0.85 (0.58, 1.24)	0.93 (0.68, 1.27)	0.92 (0.63, 1.31)	0.83 (0.56, 1.21)	0.83 (0.57, 1.20)	0.84 (0.59, 1.17)	0.86 (0.57, 1.27)	0.82 (0.58, 1.13)	0.64 (0.19, 2.09)	1.42 (0.81, 2.69)
UST130mgIV	0.96 (0.65, 1.38)	1.08 (0.90, 1.32)	0.95 (0.72, 1.26)	1.11 (0.87, 1.45)	1.01 (0.76, 1.33)	1.07 (0.75, 1.55)	1.08 (0.76, 1.57)	1.02 (0.70, 1.49)	1.15 (0.81, 1.67)	1.14 (0.82, 1.68)	1.05 (0.82, 1.36)	0.97 (0.75, 1.26)	0.96 (0.73, 1.25)	1.04 (0.91, 1.22)	1.03 (0.81, 1.31)	0.94 (0.70, 1.22)	0.94 (0.73, 1.20)	0.94 (0.77, 1.17)	0.97 (0.72, 1.27)	0.92 (0.75, 1.11)	0.73 (0.22, 2.23)	1.59 (1.00, 2.92)
UST1mg_kgIV	0.91 (0.59, 1.32)	1.02 (0.81, 1.29)	0.90 (0.65, 1.23)	1.05 (0.78, 1.42)	0.95 (0.69, 1.30)	1.01 (0.69, 1.51)	1.02 (0.69, 1.51)	0.96 (0.63, 1.44)	1.08 (0.74, 1.60)	1.08 (0.74, 1.60)	0.99 (0.74, 1.34)	0.91 (0.67, 1.23)	0.90 (0.66, 1.22)	0.98 (0.81, 1.20)	0.97 (0.74, 1.28)	0.88 (0.64, 1.19)	0.88 (0.66, 1.17)	0.88 (0.69, 1.14)	0.91 (0.66, 1.25)	0.86 (0.67, 1.10)	0.68 (0.20, 2.10)	1.50 (0.93, 2.77)
UST3mg_kgIV	0.94 (0.62, 1.38)	1.06 (0.83, 1.34)	0.93 (0.68, 1.28)	1.09 (0.82, 1.48)	0.98 (0.72, 1.35)	1.04 (0.71, 1.56)	1.06 (0.71, 1.58)	1.00 (0.66, 1.52)	1.12 (0.76, 1.68)	1.12 (0.77, 1.66)	1.03 (0.77, 1.38)	0.95 (0.70, 1.27)	0.94 (0.68, 1.27)	1.02 (0.83, 1.27)	1.01 (0.76, 1.34)	0.91 (0.67, 1.23)	0.92 (0.68, 1.23)	0.92 (0.72, 1.19)	0.94 (0.68, 1.29)	0.90 (0.70, 1.15)	0.71 (0.21, 2.17)	1.55 (0.95, 2.88)
UST4_5mg_kgIV	0.84 (0.50, 1.38)	0.95 (0.63, 1.41)	0.83 (0.53, 1.31)	0.98 (0.63, 1.50)	0.88 (0.56, 1.36)	0.93 (0.57, 1.54)	0.95 (0.57, 1.58)	0.89 (0.54, 1.49)	1.00 (0.61, 1.67)	1.00 (0.61, 1.65)	0.92 (0.60, 1.41)	0.85 (0.54, 1.30)	0.84 (0.53, 1.29)	0.92 (0.62, 1.34)	0.90 (0.58, 1.36)	0.82 (0.52, 1.27)	0.82 (0.53, 1.26)	0.82 (0.54, 1.23)	0.84 (0.53, 1.32)	0.80 (0.53, 1.20)	0.63 (0.18, 2.08)	1.40 (0.78, 2.73)
UST6mg_kg90mgIV_SC	1.08 (0.67, 1.73)	1.22 (0.86, 1.74)	1.08 (0.72, 1.62)	1.26 (0.85, 1.88)	1.14 (0.75, 1.72)	1.21 (0.76, 1.93)	1.22 (0.76, 1.96)	1.15 (0.70, 1.88)	1.30 (0.81, 2.11)	1.30 (0.81, 2.08)	1.19 (0.81, 1.77)	1.09 (0.74, 1.64)	1.08 (0.72, 1.64)	1.18 (0.84, 1.65)	1.16 (0.79, 1.72)	1.06 (0.71, 1.58)	1.06 (0.72, 1.57)	1.06 (0.74, 1.53)	1.09 (0.72, 1.66)	1.03 (0.72, 1.48)	0.82 (0.25, 2.64)	**1.80 (1.03, 3.57)**
UST6mg_kgIV	0.95 (0.64, 1.34)	1.06 (0.90, 1.27)	0.94 (0.72, 1.23)	1.10 (0.86, 1.42)	0.99 (0.74, 1.30)	1.05 (0.74, 1.51)	1.07 (0.75, 1.54)	1.01 (0.68, 1.46)	1.12 (0.80, 1.63)	1.13 (0.80, 1.63)	1.04 (0.81, 1.33)	0.95 (0.74, 1.23)	0.94 (0.72, 1.22)	1.03 (0.91, 1.17)	1.01 (0.81, 1.28)	0.92 (0.70, 1.19)	0.92 (0.73, 1.17)	0.92 (0.77, 1.13)	0.95 (0.72, 1.25)	0.90 (0.75, 1.09)	0.72 (0.22, 2.18)	1.57 (0.98, 2.84)
UST90mgSC	1.15 (0.70, 1.89)	1.28 (0.90, 1.91)	1.13 (0.75, 1.77)	1.32 (0.89, 2.06)	1.20 (0.78, 1.87)	1.27 (0.79, 2.10)	1.29 (0.80, 2.14)	1.21 (0.74, 2.05)	1.36 (0.85, 2.26)	1.37 (0.87, 2.29)	1.25 (0.84, 1.94)	1.15 (0.77, 1.79)	1.14 (0.76, 1.77)	1.24 (0.89, 1.82)	1.22 (0.83, 1.87)	1.11 (0.73, 1.74)	1.11 (0.75, 1.72)	1.12 (0.77, 1.68)	1.15 (0.74, 1.80)	1.09 (0.76, 1.62)	0.87 (0.25, 2.84)	**1.90 (1.06, 3.78)**
VED0_5mg_kgIV	0.84 (0.55, 1.22)	0.95 (0.75, 1.19)	0.84 (0.61, 1.14)	0.98 (0.73, 1.32)	0.88 (0.64, 1.21)	0.94 (0.64, 1.38)	0.95 (0.65, 1.40)	0.90 (0.60, 1.35)	1.01 (0.68, 1.48)	1.01 (0.69, 1.48)	0.93 (0.69, 1.23)	0.85 (0.63, 1.14)	0.85 (0.62, 1.13)	0.92 (0.75, 1.12)	0.90 (0.68, 1.19)	0.83 (0.60, 1.10)	0.83 (0.61, 1.09)	0.83 (0.64, 1.06)	0.85 (0.61, 1.17)	0.81 (0.63, 1.02)	0.64 (0.19, 1.99)	1.40 (0.86, 2.61)
VED2mg_kgIV	0.87 (0.58, 1.28)	0.98 (0.77, 1.23)	0.86 (0.63, 1.17)	1.01 (0.76, 1.37)	0.91 (0.66, 1.25)	0.96 (0.66, 1.43)	0.98 (0.67, 1.45)	0.92 (0.61, 1.38)	1.04 (0.71, 1.53)	1.04 (0.71, 1.53)	0.95 (0.71, 1.28)	0.88 (0.65, 1.18)	0.87 (0.64, 1.17)	0.95 (0.77, 1.16)	0.93 (0.70, 1.22)	0.85 (0.61, 1.14)	0.85 (0.64, 1.13)	0.85 (0.66, 1.10)	0.88 (0.62, 1.21)	0.83 (0.65, 1.05)	0.65 (0.19, 2.04)	1.44 (0.89, 2.67)
VED300mgIV	0.94 (0.63, 1.33)	1.06 (0.88, 1.26)	0.93 (0.70, 1.22)	1.09 (0.84, 1.41)	0.98 (0.73, 1.29)	1.04 (0.73, 1.50)	1.05 (0.74, 1.53)	0.99 (0.68, 1.45)	1.12 (0.78, 1.61)	1.12 (0.79, 1.61)	1.03 (0.80, 1.32)	0.94 (0.73, 1.22)	0.93 (0.71, 1.22)	1.02 (0.88, 1.17)	1.00 (0.79, 1.26)	0.91 (0.69, 1.18)	0.91 (0.71, 1.16)	0.92 (0.75, 1.12)	0.94 (0.70, 1.24)	0.89 (0.74, 1.08)	0.71 (0.21, 2.15)	1.55 (0.97, 2.86)

	SEM60mgIV	SEM60mgIVx3	TES400mg200mgSC	TOF15mgOR_BID	TOF1mgOR_BID	TOF5mgOR_BID	UPA12mgOR_BID	UPA24mgOR	UPA24mgOR_BID	UPA3mgOR_BID	UPA45mgOR	UPA6mgOR_BID	UST130mgIV	UST1mg_kgIV	UST3mg_kgIV	UST4_5mg_kgIV	UST6mg_kg90mgIV_SC	UST6mg_kgIV	UST90mgSC	VED0_5mg_kgIV	VED2mg_kgIV	VED300mgIV
ABA10mg_kgIV	1.13 (0.75, 1.71)	1.26 (0.86, 1.90)	1.25 (0.84, 1.83)	0.94 (0.60, 1.47)	0.75 (0.46, 1.23)	0.92 (0.59, 1.45)	1.09 (0.75, 1.58)	1.11 (0.78, 1.62)	1.12 (0.78, 1.62)	1.18 (0.82, 1.69)	1.03 (0.80, 1.33)	1.05 (0.73, 1.53)	0.93 (0.72, 1.20)	0.99 (0.74, 1.31)	0.95 (0.72, 1.28)	1.07 (0.70, 1.66)	0.83 (0.56, 1.23)	0.95 (0.74, 1.21)	0.79 (0.50, 1.17)	1.06 (0.80, 1.43)	1.03 (0.77, 1.38)	0.96 (0.75, 1.24)
ABA30mg_kgIV	1.14 (0.75, 1.76)	1.28 (0.86, 1.96)	1.27 (0.85, 1.89)	0.95 (0.61, 1.54)	0.76 (0.46, 1.27)	0.94 (0.59, 1.50)	1.10 (0.75, 1.63)	1.13 (0.77, 1.67)	1.13 (0.78, 1.68)	1.19 (0.82, 1.74)	1.05 (0.80, 1.39)	1.06 (0.73, 1.58)	0.95 (0.72, 1.25)	1.01 (0.74, 1.36)	0.97 (0.71, 1.33)	1.08 (0.71, 1.70)	0.84 (0.56, 1.25)	0.96 (0.74, 1.26)	0.80 (0.51, 1.20)	1.07 (0.80, 1.48)	1.04 (0.77, 1.44)	0.97 (0.75, 1.29)
ABA3mg_kgIV	1.16 (0.77, 1.76)	1.30 (0.88, 1.94)	1.27 (0.87, 1.88)	0.96 (0.61, 1.52)	0.77 (0.47, 1.27)	0.95 (0.61, 1.49)	1.11 (0.76, 1.63)	1.14 (0.80, 1.65)	1.15 (0.79, 1.66)	1.20 (0.84, 1.74)	1.06 (0.82, 1.36)	1.08 (0.74, 1.56)	0.96 (0.74, 1.23)	1.02 (0.76, 1.35)	0.98 (0.73, 1.31)	1.10 (0.72, 1.71)	0.85 (0.57, 1.26)	0.97 (0.76, 1.24)	0.81 (0.52, 1.21)	1.09 (0.82, 1.46)	1.06 (0.79, 1.42)	0.98 (0.77, 1.26)
ADA160mg80mg40mgSC	1.41 (0.82, 2.39)	1.58 (0.96, 2.64)	1.55 (0.94, 2.53)	1.17 (0.68, 2.06)	0.93 (0.52, 1.69)	1.14 (0.66, 2.03)	1.35 (0.83, 2.21)	1.39 (0.85, 2.27)	1.40 (0.86, 2.26)	1.46 (0.90, 2.36)	1.29 (0.86, 1.93)	1.31 (0.80, 2.14)	1.16 (0.77, 1.74)	1.23 (0.80, 1.90)	1.19 (0.77, 1.83)	1.33 (0.78, 2.29)	1.03 (0.85, 1.23)	1.18 (0.79, 1.76)	0.98 (0.58, 1.65)	1.32 (0.86, 2.05)	1.28 (0.84, 1.97)	1.19 (0.80, 1.79)
ADA160mg80mg60mgSC	1.15 (0.67, 1.93)	1.30 (0.77, 2.16)	1.27 (0.75, 2.10)	0.96 (0.56, 1.67)	0.77 (0.42, 1.39)	0.94 (0.53, 1.64)	1.10 (0.68, 1.85)	1.13 (0.69, 1.86)	1.14 (0.70, 1.89)	1.19 (0.74, 1.97)	1.06 (0.69, 1.60)	1.07 (0.65, 1.75)	0.95 (0.62, 1.45)	1.01 (0.65, 1.58)	0.97 (0.62, 1.52)	1.09 (0.63, 1.87)	0.84 (0.50, 1.40)	0.97 (0.64, 1.45)	0.80 (0.46, 1.33)	1.08 (0.70, 1.67)	1.05 (0.68, 1.62)	0.98 (0.64, 1.49)
ADA160mg80mgSC	1.29 (0.86, 1.92)	**1.44 (1.01, 2.10)**	1.42 (0.99, 2.06)	1.07 (0.70, 1.66)	0.86 (0.53, 1.38)	1.06 (0.68, 1.62)	1.24 (0.87, 1.76)	1.27 (0.90, 1.80)	1.28 (0.90, 1.81)	1.34 (0.96, 1.90)	1.18 (0.95, 1.45)	1.20 (0.84, 1.71)	1.07 (0.85, 1.30)	1.13 (0.87, 1.44)	1.09 (0.84, 1.39)	1.22 (0.82, 1.85)	0.94 (0.65, 1.34)	1.08 (0.88, 1.31)	0.90 (0.59, 1.30)	1.21 (0.94, 1.55)	1.18 (0.91, 1.50)	1.09 (0.88, 1.34)
ADA40mg20mgSC	1.35 (0.88, 2.07)	**1.52 (1.01, 2.29)**	1.49 (1.00, 2.25)	1.12 (0.70, 1.81)	0.90 (0.54, 1.49)	1.11 (0.70, 1.76)	1.30 (0.87, 1.91)	1.33 (0.91, 1.98)	1.34 (0.91, 1.99)	1.40 (0.97, 2.06)	1.24 (0.93, 1.63)	1.26 (0.84, 1.86)	1.12 (0.85, 1.47)	1.19 (0.87, 1.61)	1.14 (0.83, 1.55)	1.28 (0.82, 2.00)	0.99 (0.66, 1.47)	1.13 (0.87, 1.48)	0.94 (0.60, 1.44)	1.27 (0.94, 1.74)	1.24 (0.91, 1.68)	1.14 (0.88, 1.51)
ADA80mg40mgSC	1.33 (0.88, 2.04)	**1.49 (1.02, 2.24)**	1.47 (0.99, 2.17)	1.11 (0.71, 1.76)	0.88 (0.54, 1.47)	1.09 (0.69, 1.71)	1.28 (0.88, 1.86)	1.32 (0.91, 1.91)	1.32 (0.92, 1.92)	1.39 (0.98, 2.00)	1.22 (0.95, 1.57)	1.24 (0.86, 1.81)	1.10 (0.86, 1.41)	1.17 (0.88, 1.55)	1.13 (0.85, 1.51)	1.26 (0.83, 1.96)	0.98 (0.66, 1.43)	1.12 (0.88, 1.43)	0.93 (0.60, 1.38)	1.25 (0.94, 1.68)	1.22 (0.92, 1.63)	1.13 (0.88, 1.46)
AMI0_4mgOR	0.97 (0.57, 1.69)	1.09 (0.65, 1.87)	1.08 (0.64, 1.83)	0.82 (0.46, 1.44)	0.66 (0.36, 1.19)	0.80 (0.46, 1.41)	0.94 (0.57, 1.54)	0.96 (0.58, 1.59)	0.97 (0.59, 1.59)	1.02 (0.62, 1.66)	0.90 (0.58, 1.36)	0.92 (0.55, 1.50)	0.81 (0.52, 1.23)	0.86 (0.54, 1.34)	0.83 (0.53, 1.29)	0.93 (0.54, 1.59)	0.72 (0.43, 1.21)	0.82 (0.54, 1.24)	0.68 (0.39, 1.15)	0.92 (0.59, 1.44)	0.89 (0.57, 1.39)	0.83 (0.54, 1.27)
AND150mgSC1_1	1.24 (0.73, 2.10)	1.39 (0.84, 2.30)	1.38 (0.82, 2.22)	1.03 (0.58, 1.79)	0.82 (0.46, 1.47)	1.01 (0.59, 1.77)	1.20 (0.73, 1.90)	1.22 (0.75, 1.94)	1.24 (0.76, 1.95)	1.29 (0.79, 2.05)	1.14 (0.75, 1.67)	1.16 (0.70, 1.86)	1.03 (0.69, 1.51)	1.09 (0.71, 1.65)	1.05 (0.68, 1.58)	1.17 (0.68, 1.98)	0.91 (0.55, 1.49)	1.04 (0.70, 1.53)	0.86 (0.50, 1.42)	1.17 (0.76, 1.78)	1.14 (0.73, 1.72)	1.05 (0.70, 1.55)
AND150mgSC2_2	1.28 (0.75, 2.12)	1.44 (0.86, 2.36)	1.42 (0.85, 2.32)	1.07 (0.61, 1.81)	0.85 (0.47, 1.51)	1.05 (0.60, 1.79)	1.23 (0.75, 1.98)	1.26 (0.78, 2.01)	1.28 (0.78, 2.03)	1.34 (0.82, 2.11)	1.18 (0.78, 1.71)	1.19 (0.72, 1.91)	1.06 (0.70, 1.55)	1.13 (0.73, 1.71)	1.09 (0.70, 1.65)	1.22 (0.71, 2.05)	0.94 (0.56, 1.54)	1.08 (0.72, 1.57)	0.89 (0.52, 1.46)	1.21 (0.78, 1.82)	1.18 (0.75, 1.77)	1.09 (0.72, 1.60)
AND300mgSC	1.10 (0.66, 1.81)	1.24 (0.76, 1.99)	1.22 (0.75, 1.95)	0.92 (0.53, 1.55)	0.73 (0.41, 1.29)	0.90 (0.53, 1.53)	1.06 (0.66, 1.66)	1.09 (0.68, 1.70)	1.10 (0.69, 1.73)	1.15 (0.71, 1.79)	1.01 (0.68, 1.46)	1.03 (0.63, 1.62)	0.92 (0.62, 1.32)	0.97 (0.64, 1.42)	0.93 (0.62, 1.38)	1.04 (0.62, 1.74)	0.81 (0.49, 1.30)	0.93 (0.63, 1.32)	0.77 (0.45, 1.25)	1.04 (0.68, 1.54)	1.01 (0.67, 1.50)	0.94 (0.64, 1.35)
API100mgOT	1.17 (0.77, 1.78)	1.31 (0.89, 1.98)	1.29 (0.87, 1.93)	0.97 (0.61, 1.54)	0.78 (0.47, 1.29)	0.96 (0.61, 1.52)	1.13 (0.77, 1.64)	1.15 (0.80, 1.69)	1.16 (0.80, 1.69)	1.22 (0.84, 1.77)	1.07 (0.82, 1.40)	1.09 (0.74, 1.60)	0.97 (0.74, 1.25)	1.03 (0.76, 1.39)	0.99 (0.73, 1.34)	1.11 (0.72, 1.72)	0.86 (0.58, 1.27)	0.99 (0.76, 1.26)	0.82 (0.52, 1.22)	1.10 (0.82, 1.48)	1.07 (0.79, 1.45)	0.99 (0.77, 1.30)
API50mgOR	1.28 (0.84, 2.01)	1.44 (0.97, 2.21)	1.42 (0.96, 2.15)	1.07 (0.68, 1.73)	0.86 (0.52, 1.43)	1.05 (0.66, 1.70)	1.24 (0.84, 1.84)	1.27 (0.87, 1.88)	1.28 (0.87, 1.90)	1.34 (0.92, 1.98)	1.18 (0.90, 1.56)	1.20 (0.81, 1.80)	1.07 (0.81, 1.41)	1.13 (0.83, 1.55)	1.09 (0.79, 1.50)	1.22 (0.78, 1.92)	0.94 (0.62, 1.42)	1.08 (0.83, 1.42)	0.90 (0.57, 1.36)	1.21 (0.89, 1.66)	1.18 (0.86, 1.61)	1.09 (0.83, 1.45)
BRI400mgIV	1.20 (0.77, 1.90)	1.34 (0.88, 2.12)	1.32 (0.86, 2.05)	1.00 (0.62, 1.65)	0.80 (0.48, 1.36)	0.98 (0.61, 1.62)	1.16 (0.76, 1.76)	1.18 (0.78, 1.79)	1.19 (0.79, 1.80)	1.25 (0.84, 1.87)	1.10 (0.80, 1.52)	1.12 (0.74, 1.70)	0.99 (0.73, 1.36)	1.05 (0.75, 1.50)	1.01 (0.72, 1.45)	1.14 (0.71, 1.83)	0.88 (0.56, 1.38)	1.01 (0.74, 1.38)	0.83 (0.53, 1.32)	1.13 (0.81, 1.61)	1.10 (0.78, 1.56)	1.02 (0.75, 1.41)
BRI700mgIV	1.30 (0.84, 2.01)	1.46 (0.97, 2.23)	1.44 (0.95, 2.17)	1.09 (0.67, 1.74)	0.87 (0.52, 1.45)	1.06 (0.67, 1.72)	1.25 (0.84, 1.87)	1.28 (0.87, 1.90)	1.29 (0.88, 1.92)	1.36 (0.93, 1.99)	1.19 (0.89, 1.58)	1.21 (0.82, 1.80)	1.08 (0.81, 1.42)	1.14 (0.83, 1.57)	1.10 (0.80, 1.52)	1.24 (0.79, 1.94)	0.95 (0.63, 1.45)	1.09 (0.83, 1.43)	0.91 (0.58, 1.37)	1.22 (0.89, 1.69)	1.19 (0.86, 1.64)	1.11 (0.82, 1.47)
BRO210mgIV	1.26 (0.77, 2.06)	1.41 (0.89, 2.28)	1.38 (0.88, 2.22)	1.05 (0.62, 1.78)	0.83 (0.48, 1.48)	1.03 (0.62, 1.74)	1.21 (0.78, 1.92)	1.24 (0.80, 1.97)	1.25 (0.80, 1.96)	1.30 (0.85, 2.07)	1.15 (0.80, 1.67)	1.17 (0.74, 1.88)	1.04 (0.72, 1.50)	1.10 (0.75, 1.64)	1.06 (0.73, 1.58)	1.19 (0.72, 1.99)	0.92 (0.58, 1.48)	1.05 (0.74, 1.52)	0.87 (0.53, 1.41)	1.18 (0.81, 1.77)	1.14 (0.78, 1.71)	1.07 (0.74, 1.53)
BRO350mgIV	1.10 (0.69, 1.77)	1.23 (0.79, 1.96)	1.22 (0.79, 1.89)	0.92 (0.56, 1.51)	0.73 (0.43, 1.25)	0.90 (0.55, 1.48)	1.06 (0.69, 1.64)	1.08 (0.71, 1.68)	1.09 (0.71, 1.67)	1.15 (0.76, 1.75)	1.01 (0.72, 1.41)	1.03 (0.66, 1.57)	0.91 (0.66, 1.26)	0.97 (0.68, 1.38)	0.93 (0.65, 1.34)	1.04 (0.65, 1.71)	0.81 (0.51, 1.26)	0.92 (0.67, 1.27)	0.77 (0.48, 1.20)	1.03 (0.73, 1.48)	1.00 (0.71, 1.44)	0.93 (0.67, 1.30)
BRO700mgIV	1.06 (0.66, 1.70)	1.19 (0.77, 1.88)	1.17 (0.76, 1.82)	0.89 (0.53, 1.45)	0.71 (0.42, 1.20)	0.87 (0.54, 1.42)	1.01 (0.67, 1.56)	1.04 (0.69, 1.62)	1.05 (0.69, 1.62)	1.10 (0.72, 1.69)	0.97 (0.70, 1.34)	0.99 (0.64, 1.53)	0.88 (0.64, 1.22)	0.93 (0.65, 1.32)	0.90 (0.63, 1.29)	1.00 (0.62, 1.62)	0.77 (0.50, 1.20)	0.89 (0.65, 1.22)	0.74 (0.46, 1.15)	1.00 (0.71, 1.42)	0.97 (0.68, 1.38)	0.90 (0.65, 1.24)
CDP10mg_kgIV	1.09 (0.73, 1.63)	1.22 (0.83, 1.82)	1.20 (0.82, 1.77)	0.91 (0.58, 1.43)	0.73 (0.45, 1.18)	0.89 (0.57, 1.40)	1.05 (0.73, 1.51)	1.08 (0.75, 1.53)	1.09 (0.75, 1.55)	1.14 (0.81, 1.61)	1.00 (0.78, 1.27)	1.01 (0.71, 1.47)	0.90 (0.71, 1.15)	0.96 (0.72, 1.26)	0.92 (0.69, 1.23)	1.03 (0.67, 1.59)	0.80 (0.55, 1.18)	0.92 (0.72, 1.16)	0.76 (0.50, 1.11)	1.02 (0.78, 1.37)	1.00 (0.75, 1.33)	0.93 (0.73, 1.18)
CER1_25mgIV	2.37 (0.66, 17.25)	2.65 (0.77, 19.34)	2.56 (0.76, 18.49)	1.96 (0.56, 14.13)	1.59 (0.43, 11.58)	1.93 (0.54, 14.18)	2.24 (0.65, 16.00)	2.31 (0.67, 16.78)	2.31 (0.68, 16.56)	2.44 (0.71, 17.27)	2.13 (0.65, 15.01)	2.20 (0.63, 15.43)	1.94 (0.59, 13.56)	2.05 (0.62, 14.54)	1.97 (0.60, 13.83)	2.23 (0.63, 15.78)	1.70 (0.49, 12.26)	1.96 (0.60, 13.78)	1.61 (0.47, 11.37)	2.18 (0.67, 15.38)	2.13 (0.64, 15.02)	1.97 (0.60, 13.93)
CER100mgSC	1.02 (0.67, 1.56)	1.15 (0.78, 1.74)	1.13 (0.76, 1.69)	0.85 (0.55, 1.34)	0.68 (0.41, 1.12)	0.84 (0.53, 1.34)	0.98 (0.67, 1.43)	1.01 (0.69, 1.46)	1.01 (0.70, 1.47)	1.06 (0.75, 1.53)	0.94 (0.72, 1.22)	0.95 (0.65, 1.40)	0.85 (0.65, 1.10)	0.90 (0.67, 1.22)	0.86 (0.64, 1.18)	0.97 (0.62, 1.52)	0.75 (0.50, 1.12)	0.86 (0.67, 1.10)	0.71 (0.46, 1.06)	0.96 (0.72, 1.31)	0.93 (0.69, 1.26)	0.87 (0.67, 1.13)

	SEM60mgIV	SEM60mgIVx3	TES400mg200mgSC	TOF15mgOR_BID	TOF1mgOR_BID	TOF5mgOR_BID	UPA12mgOR_BID	UPA24mgOR	UPA24mgOR_BID	UPA3mgOR_BID	UPA45mgOR	UPA6mgOR_BID	UST130mgIV	UST1mg_kgIV	UST3mg_kgIV	UST4_5mg_kgIV	UST6mg_kg90mgIV_SC	UST6mg_kgIV	UST90mgSC	VED0_5mg_kgIV	VED2mg_kgIV	VED300mgIV
CER10mgIV	1.08 (0.58, 2.12)	1.22 (0.66, 2.33)	1.19 (0.65, 2.24)	0.90 (0.48, 1.84)	0.72 (0.37, 1.49)	0.88 (0.48, 1.81)	1.03 (0.57, 1.95)	1.06 (0.60, 1.99)	1.07 (0.60, 2.03)	1.12 (0.64, 2.10)	0.98 (0.59, 1.77)	1.01 (0.56, 1.88)	0.89 (0.54, 1.58)	0.95 (0.55, 1.73)	0.91 (0.54, 1.66)	1.02 (0.54, 1.99)	0.79 (0.43, 1.52)	0.91 (0.55, 1.62)	0.75 (0.40, 1.46)	1.01 (0.60, 1.86)	0.98 (0.58, 1.79)	0.91 (0.55, 1.63)
CER200mgSC	1.03 (0.67, 1.58)	1.15 (0.78, 1.75)	1.14 (0.77, 1.71)	0.86 (0.53, 1.36)	0.68 (0.42, 1.12)	0.84 (0.53, 1.34)	0.99 (0.67, 1.45)	1.01 (0.69, 1.49)	1.02 (0.70, 1.49)	1.07 (0.74, 1.55)	0.94 (0.72, 1.23)	0.96 (0.65, 1.41)	0.85 (0.65, 1.11)	0.90 (0.67, 1.23)	0.87 (0.64, 1.18)	0.97 (0.63, 1.51)	0.75 (0.50, 1.12)	0.86 (0.67, 1.11)	0.71 (0.46, 1.08)	0.96 (0.72, 1.33)	0.94 (0.70, 1.28)	0.87 (0.67, 1.15)
CER20mgIV	1.13 (0.61, 2.16)	1.27 (0.70, 2.34)	1.25 (0.69, 2.31)	0.94 (0.50, 1.81)	0.75 (0.38, 1.49)	0.93 (0.49, 1.80)	1.09 (0.60, 1.96)	1.12 (0.62, 1.99)	1.13 (0.63, 2.02)	1.18 (0.67, 2.11)	1.04 (0.62, 1.75)	1.05 (0.59, 1.89)	0.94 (0.55, 1.58)	0.99 (0.58, 1.72)	0.96 (0.56, 1.67)	1.08 (0.57, 2.02)	0.83 (0.46, 1.50)	0.95 (0.57, 1.60)	0.79 (0.42, 1.44)	1.06 (0.63, 1.86)	1.03 (0.61, 1.79)	0.96 (0.57, 1.64)
CER400mgSC	1.14 (0.78, 1.70)	1.28 (0.90, 1.88)	1.26 (0.89, 1.83)	0.95 (0.63, 1.46)	0.76 (0.47, 1.22)	0.94 (0.62, 1.45)	1.10 (0.78, 1.56)	1.13 (0.81, 1.59)	1.13 (0.82, 1.59)	1.19 (0.87, 1.67)	1.04 (0.86, 1.29)	1.06 (0.76, 1.50)	0.95 (0.77, 1.16)	1.00 (0.79, 1.29)	0.97 (0.76, 1.25)	1.08 (0.72, 1.63)	0.84 (0.59, 1.20)	0.96 (0.80, 1.16)	0.80 (0.53, 1.15)	1.07 (0.85, 1.40)	1.04 (0.82, 1.35)	0.97 (0.80, 1.20)
CER5mgIV	1.02 (0.56, 1.86)	1.15 (0.64, 2.06)	1.12 (0.63, 2.02)	0.85 (0.46, 1.57)	0.68 (0.35, 1.29)	0.83 (0.45, 1.55)	0.98 (0.56, 1.72)	1.00 (0.58, 1.75)	1.01 (0.58, 1.78)	1.06 (0.61, 1.83)	0.93 (0.57, 1.53)	0.95 (0.54, 1.65)	0.84 (0.51, 1.37)	0.90 (0.54, 1.49)	0.86 (0.52, 1.44)	0.97 (0.52, 1.73)	0.75 (0.42, 1.34)	0.86 (0.53, 1.38)	0.71 (0.38, 1.26)	0.96 (0.58, 1.60)	0.93 (0.56, 1.53)	0.87 (0.53, 1.41)
ELD10mg_kgIV	1.39 (0.85, 2.25)	1.55 (0.98, 2.50)	1.53 (0.97, 2.45)	1.15 (0.69, 1.96)	0.92 (0.53, 1.61)	1.13 (0.69, 1.94)	1.33 (0.86, 2.11)	1.37 (0.88, 2.17)	1.38 (0.88, 2.19)	1.44 (0.92, 2.25)	1.27 (0.89, 1.82)	1.29 (0.83, 2.04)	1.15 (0.80, 1.65)	1.22 (0.83, 1.79)	1.17 (0.80, 1.74)	1.31 (0.80, 2.20)	1.01 (0.64, 1.62)	1.16 (0.82, 1.66)	0.97 (0.58, 1.55)	1.30 (0.89, 1.94)	1.27 (0.87, 1.88)	1.17 (0.83, 1.69)
ELD20mg_kgIV	1.54 (0.94, 2.59)	**1.73 (1.07, 2.89)**	**1.70 (1.05, 2.85)**	1.29 (0.77, 2.20)	1.03 (0.59, 1.83)	1.27 (0.77, 2.16)	1.49 (0.93, 2.39)	1.52 (0.96, 2.45)	1.53 (0.97, 2.48)	**1.60 (1.03, 2.55)**	1.41 (0.98, 2.10)	1.44 (0.90, 2.32)	1.28 (0.89, 1.90)	1.36 (0.91, 2.05)	1.31 (0.88, 1.97)	1.47 (0.87, 2.50)	1.13 (0.69, 1.83)	1.30 (0.90, 1.90)	1.08 (0.64, 1.78)	1.46 (0.99, 2.20)	1.41 (0.95, 2.12)	1.31 (0.91, 1.94)
ETA25mgSC	0.77 (0.38, 1.47)	0.86 (0.43, 1.63)	0.85 (0.43, 1.58)	0.64 (0.30, 1.24)	0.51 (0.24, 1.01)	0.63 (0.30, 1.22)	0.74 (0.37, 1.35)	0.76 (0.38, 1.39)	0.77 (0.38, 1.40)	0.80 (0.40, 1.46)	0.71 (0.38, 1.19)	0.72 (0.35, 1.34)	0.64 (0.34, 1.08)	0.68 (0.36, 1.17)	0.66 (0.34, 1.14)	0.73 (0.35, 1.41)	0.57 (0.28, 1.05)	0.65 (0.34, 1.09)	0.54 (0.26, 1.00)	0.73 (0.38, 1.26)	0.71 (0.37, 1.23)	0.66 (0.35, 1.11)
ETR105mgSC	0.92 (0.59, 1.44)	1.03 (0.68, 1.59)	1.01 (0.66, 1.55)	0.77 (0.47, 1.25)	0.61 (0.36, 1.03)	0.76 (0.46, 1.22)	0.88 (0.59, 1.33)	0.90 (0.60, 1.36)	0.91 (0.61, 1.38)	0.96 (0.64, 1.44)	0.84 (0.62, 1.13)	0.86 (0.57, 1.30)	0.76 (0.56, 1.02)	0.81 (0.58, 1.12)	0.78 (0.56, 1.08)	0.87 (0.55, 1.38)	0.67 (0.44, 1.03)	0.77 (0.57, 1.03)	**0.64 (0.40, 0.98)**	0.86 (0.62, 1.21)	0.84 (0.60, 1.18)	0.78 (0.57, 1.05)
ETR210mgSC	1.04 (0.66, 1.64)	1.17 (0.76, 1.81)	1.15 (0.74, 1.78)	0.86 (0.53, 1.43)	0.69 (0.41, 1.17)	0.86 (0.52, 1.39)	1.00 (0.66, 1.51)	1.03 (0.68, 1.56)	1.03 (0.69, 1.55)	1.08 (0.72, 1.63)	0.96 (0.70, 1.29)	0.97 (0.64, 1.49)	0.86 (0.63, 1.17)	0.91 (0.64, 1.29)	0.88 (0.63, 1.24)	0.98 (0.62, 1.57)	0.76 (0.49, 1.16)	0.88 (0.65, 1.18)	0.72 (0.46, 1.12)	0.98 (0.70, 1.38)	0.95 (0.68, 1.33)	0.88 (0.65, 1.20)
FIL100mgPO	1.16 (0.78, 1.75)	1.31 (0.90, 1.95)	1.29 (0.88, 1.87)	0.97 (0.62, 1.52)	0.78 (0.48, 1.28)	0.96 (0.61, 1.50)	1.12 (0.78, 1.62)	1.15 (0.81, 1.66)	1.16 (0.81, 1.68)	1.21 (0.86, 1.73)	1.07 (0.84, 1.36)	1.08 (0.75, 1.58)	0.97 (0.75, 1.23)	1.02 (0.77, 1.35)	0.98 (0.75, 1.31)	1.10 (0.72, 1.71)	0.86 (0.58, 1.25)	0.98 (0.78, 1.23)	0.81 (0.53, 1.20)	1.09 (0.84, 1.47)	1.07 (0.80, 1.41)	0.99 (0.78, 1.26)
FIL200mgPO	1.13 (0.75, 1.70)	1.26 (0.86, 1.87)	1.25 (0.85, 1.82)	0.94 (0.61, 1.47)	0.75 (0.46, 1.24)	0.92 (0.59, 1.44)	1.08 (0.75, 1.56)	1.11 (0.77, 1.61)	1.12 (0.78, 1.63)	1.17 (0.82, 1.67)	1.03 (0.81, 1.31)	1.05 (0.73, 1.53)	0.93 (0.73, 1.18)	0.99 (0.75, 1.31)	0.95 (0.72, 1.27)	1.07 (0.69, 1.64)	0.83 (0.56, 1.22)	0.95 (0.75, 1.20)	0.78 (0.52, 1.15)	1.06 (0.81, 1.40)	1.03 (0.78, 1.37)	0.96 (0.75, 1.22)
FON1mg_kg0_1mgIV_SC	1.31 (0.82, 2.16)	1.48 (0.93, 2.39)	1.45 (0.91, 2.33)	1.10 (0.66, 1.84)	0.88 (0.50, 1.53)	1.08 (0.63, 1.82)	1.26 (0.81, 2.00)	1.29 (0.84, 2.07)	1.30 (0.84, 2.06)	1.36 (0.88, 2.17)	1.21 (0.84, 1.75)	1.23 (0.78, 1.93)	1.09 (0.75, 1.57)	1.16 (0.78, 1.72)	1.12 (0.75, 1.65)	1.25 (0.75, 2.06)	0.96 (0.60, 1.55)	1.11 (0.76, 1.58)	0.91 (0.55, 1.49)	1.24 (0.84, 1.83)	1.21 (0.81, 1.77)	1.12 (0.77, 1.62)
FON1mg_kg1mgIV_SC	1.45 (0.88, 2.46)	**1.62 (1.02, 2.70)**	1.60 (0.99, 2.63)	1.21 (0.71, 2.13)	0.96 (0.54, 1.77)	1.19 (0.69, 2.11)	1.39 (0.88, 2.27)	1.43 (0.91, 2.34)	1.44 (0.91, 2.34)	1.50 (0.96, 2.43)	1.33 (0.90, 2.03)	1.35 (0.85, 2.21)	1.20 (0.81, 1.83)	1.27 (0.84, 1.97)	1.22 (0.81, 1.90)	1.37 (0.82, 2.36)	1.06 (0.65, 1.77)	1.22 (0.84, 1.85)	1.01 (0.60, 1.71)	1.37 (0.90, 2.12)	1.32 (0.89, 2.06)	1.23 (0.84, 1.87)
FON4mg_kg0_1mgIV_SC	1.11 (0.70, 1.79)	1.25 (0.81, 1.99)	1.23 (0.78, 1.93)	0.93 (0.56, 1.55)	0.74 (0.43, 1.27)	0.91 (0.55, 1.53)	1.07 (0.70, 1.64)	1.10 (0.72, 1.69)	1.11 (0.72, 1.71)	1.16 (0.76, 1.77)	1.02 (0.72, 1.44)	1.04 (0.67, 1.60)	0.92 (0.65, 1.29)	0.98 (0.68, 1.41)	0.94 (0.65, 1.37)	1.06 (0.65, 1.71)	0.82 (0.52, 1.29)	0.94 (0.67, 1.30)	0.77 (0.47, 1.24)	1.05 (0.73, 1.52)	1.02 (0.71, 1.48)	0.95 (0.67, 1.33)
FON4mg_kg1mgIV_SC	1.19 (0.75, 1.95)	1.33 (0.86, 2.18)	1.31 (0.83, 2.13)	0.99 (0.61, 1.66)	0.79 (0.46, 1.38)	0.98 (0.59, 1.63)	1.15 (0.74, 1.78)	1.18 (0.76, 1.82)	1.19 (0.76, 1.84)	1.24 (0.81, 1.93)	1.09 (0.78, 1.57)	1.11 (0.72, 1.75)	0.98 (0.70, 1.41)	1.04 (0.72, 1.54)	1.01 (0.69, 1.49)	1.13 (0.69, 1.86)	0.87 (0.55, 1.39)	1.00 (0.71, 1.43)	0.83 (0.50, 1.33)	1.12 (0.77, 1.67)	1.09 (0.75, 1.61)	1.01 (0.72, 1.46)
GUS1200mgIV	1.64 (0.97, 2.77)	**1.84 (1.13, 3.09)**	**1.80 (1.12, 3.00)**	1.36 (0.80, 2.35)	1.09 (0.61, 1.96)	1.33 (0.79, 2.34)	1.57 (0.98, 2.57)	**1.61 (1.01, 2.63)**	**1.62 (1.03, 2.64)**	**1.70 (1.08, 2.73)**	**1.49 (1.02, 2.23)**	1.52 (0.95, 2.46)	1.35 (0.92, 2.02)	1.43 (0.96, 2.22)	1.38 (0.92, 2.12)	1.54 (0.92, 2.63)	1.19 (0.82, 1.77)	1.37 (0.94, 2.03)	1.13 (0.68, 1.89)	**1.53 (1.03, 2.36)**	1.49 (0.99, 2.28)	1.38 (0.95, 2.07)
GUS200mgIV	1.58 (0.96, 2.61)	**1.77 (1.08, 2.91)**	**1.75 (1.07, 2.84)**	1.31 (0.78, 2.30)	1.05 (0.59, 1.87)	1.29 (0.76, 2.26)	1.53 (0.95, 2.44)	1.56 (0.98, 2.49)	1.58 (0.99, 2.50)	**1.65 (1.04, 2.60)**	1.45 (0.99, 2.13)	1.47 (0.92, 2.37)	1.31 (0.90, 1.91)	1.38 (0.92, 2.10)	1.33 (0.89, 2.03)	1.49 (0.90, 2.53)	1.15 (0.79, 1.70)	1.32 (0.92, 1.93)	1.09 (0.66, 1.80)	1.48 (0.99, 2.23)	1.44 (0.96, 2.19)	1.34 (0.91, 1.98)
GUS600mgIV	1.37 (0.84, 2.25)	1.53 (0.96, 2.48)	1.50 (0.94, 2.40)	1.14 (0.68, 1.93)	0.91 (0.52, 1.60)	1.12 (0.67, 1.88)	1.31 (0.83, 2.11)	1.34 (0.85, 2.15)	1.35 (0.86, 2.15)	1.42 (0.91, 2.25)	1.25 (0.88, 1.81)	1.27 (0.81, 2.04)	1.13 (0.79, 1.63)	1.20 (0.81, 1.79)	1.16 (0.78, 1.74)	1.29 (0.79, 2.17)	1.00 (0.70, 1.42)	1.15 (0.81, 1.65)	0.95 (0.57, 1.53)	1.28 (0.87, 1.91)	1.24 (0.85, 1.86)	1.16 (0.82, 1.67)
MED700IV	1.17 (0.74, 1.87)	1.31 (0.85, 2.09)	1.29 (0.83, 2.00)	0.97 (0.60, 1.61)	0.78 (0.46, 1.34)	0.96 (0.59, 1.56)	1.12 (0.73, 1.71)	1.15 (0.76, 1.74)	1.16 (0.76, 1.77)	1.21 (0.82, 1.84)	1.06 (0.78, 1.49)	1.08 (0.72, 1.67)	0.96 (0.70, 1.34)	1.02 (0.72, 1.47)	0.99 (0.69, 1.42)	1.10 (0.68, 1.79)	0.86 (0.54, 1.34)	0.98 (0.72, 1.35)	0.81 (0.51, 1.27)	1.10 (0.77, 1.57)	1.06 (0.75, 1.53)	0.99 (0.72, 1.38)
MIR1000mgIV	1.24 (0.79, 1.97)	1.40 (0.90, 2.18)	1.37 (0.89, 2.13)	1.04 (0.63, 1.69)	0.83 (0.49, 1.41)	1.02 (0.62, 1.68)	1.19 (0.79, 1.82)	1.22 (0.81, 1.86)	1.24 (0.82, 1.87)	1.29 (0.87, 1.94)	1.13 (0.83, 1.58)	1.16 (0.76, 1.78)	1.03 (0.74, 1.41)	1.09 (0.77, 1.55)	1.05 (0.74, 1.50)	1.17 (0.74, 1.89)	0.90 (0.59, 1.43)	1.04 (0.76, 1.42)	0.87 (0.54, 1.35)	1.17 (0.84, 1.65)	1.14 (0.81, 1.60)	1.05 (0.77, 1.45)
MIR200mgIV	1.43 (0.86, 2.47)	1.61 (0.99, 2.75)	1.57 (0.97, 2.73)	1.19 (0.71, 2.12)	0.96 (0.54, 1.71)	1.18 (0.68, 2.07)	1.38 (0.85, 2.32)	1.41 (0.88, 2.38)	1.42 (0.88, 2.39)	1.48 (0.94, 2.50)	1.31 (0.89, 2.03)	1.33 (0.83, 2.25)	1.18 (0.81, 1.82)	1.26 (0.83, 1.99)	1.21 (0.80, 1.93)	1.36 (0.80, 2.37)	1.05 (0.64, 1.76)	1.20 (0.83, 1.84)	1.00 (0.59, 1.68)	1.34 (0.90, 2.12)	1.31 (0.87, 2.05)	1.21 (0.83, 1.89)

	SEM60mgIV	SEM60mgIVx3	TES400mg200mgSC	TOF15mgOR_BID	TOF1mgOR_BID	TOF5mgOR_BID	UPA12mgOR_BID	UPA24mgOR	UPA24mgOR_BID	UPA3mgOR_BID	UPA45mgOR	UPA6mgOR_BID	UST130mgIV	UST1mg_kgIV	UST3mg_kgIV	UST4_5mg_kgIV	UST6mg_kg90mgIV_SC	UST6mg_kgIV	UST90mgSC	VED0_5mg_kgIV	VED2mg_kgIV	VED300mgIV
MIR600mgIV	1.25 (0.77, 2.08)	1.41 (0.88, 2.33)	1.39 (0.88, 2.27)	1.05 (0.62, 1.81)	0.84 (0.48, 1.50)	1.03 (0.61, 1.78)	1.21 (0.77, 1.93)	1.24 (0.79, 1.99)	1.25 (0.80, 1.99)	1.31 (0.85, 2.09)	1.15 (0.80, 1.71)	1.17 (0.75, 1.89)	1.04 (0.72, 1.55)	1.10 (0.76, 1.68)	1.06 (0.72, 1.63)	1.19 (0.73, 2.02)	0.93 (0.58, 1.50)	1.05 (0.74, 1.56)	0.87 (0.53, 1.44)	1.18 (0.82, 1.81)	1.15 (0.78, 1.74)	1.06 (0.75, 1.58)
NAT300mgIV	1.12 (0.77, 1.63)	1.25 (0.89, 1.81)	1.23 (0.87, 1.76)	0.93 (0.61, 1.43)	0.74 (0.47, 1.18)	0.92 (0.61, 1.39)	1.07 (0.78, 1.50)	1.10 (0.80, 1.54)	1.11 (0.80, 1.55)	1.16 (0.85, 1.61)	1.02 (0.85, 1.23)	1.04 (0.75, 1.46)	0.93 (0.76, 1.11)	0.98 (0.78, 1.24)	0.95 (0.75, 1.20)	1.06 (0.71, 1.59)	0.82 (0.57, 1.16)	0.94 (0.79, 1.12)	0.78 (0.52, 1.11)	1.05 (0.84, 1.34)	1.02 (0.81, 1.29)	0.95 (0.79, 1.14)
NAT3mg_kgIV	1.27 (0.83, 1.94)	1.43 (0.95, 2.16)	1.40 (0.94, 2.12)	1.06 (0.65, 1.70)	0.84 (0.51, 1.40)	1.04 (0.65, 1.67)	1.22 (0.83, 1.81)	1.25 (0.85, 1.85)	1.26 (0.86, 1.86)	1.32 (0.91, 1.94)	1.16 (0.88, 1.54)	1.18 (0.81, 1.75)	1.05 (0.79, 1.38)	1.11 (0.81, 1.53)	1.08 (0.78, 1.48)	1.20 (0.76, 1.88)	0.93 (0.62, 1.39)	1.07 (0.81, 1.40)	0.88 (0.56, 1.34)	1.20 (0.88, 1.64)	1.16 (0.85, 1.59)	1.08 (0.82, 1.43)
NAT3mg_kgIVx2	1.08 (0.71, 1.65)	1.21 (0.82, 1.85)	1.19 (0.81, 1.79)	0.90 (0.57, 1.42)	0.72 (0.44, 1.18)	0.89 (0.56, 1.40)	1.04 (0.71, 1.53)	1.07 (0.73, 1.56)	1.08 (0.74, 1.58)	1.13 (0.79, 1.63)	0.99 (0.76, 1.28)	1.01 (0.69, 1.49)	0.90 (0.69, 1.16)	0.95 (0.70, 1.28)	0.92 (0.67, 1.22)	1.03 (0.67, 1.59)	0.79 (0.53, 1.18)	0.91 (0.70, 1.16)	0.75 (0.49, 1.13)	1.02 (0.76, 1.37)	0.99 (0.73, 1.32)	0.92 (0.71, 1.19)
NAT6mg_kgIVx2	1.20 (0.77, 1.86)	1.34 (0.90, 2.09)	1.32 (0.88, 2.01)	1.00 (0.63, 1.60)	0.80 (0.48, 1.33)	0.99 (0.62, 1.56)	1.15 (0.77, 1.72)	1.18 (0.80, 1.76)	1.19 (0.81, 1.77)	1.25 (0.86, 1.85)	1.10 (0.83, 1.47)	1.12 (0.75, 1.69)	0.99 (0.75, 1.32)	1.05 (0.77, 1.45)	1.02 (0.74, 1.40)	1.13 (0.73, 1.79)	0.88 (0.58, 1.33)	1.01 (0.77, 1.35)	0.84 (0.53, 1.28)	1.13 (0.83, 1.56)	1.10 (0.80, 1.51)	1.02 (0.78, 1.36)
NNC2mg_kgSC	1.13 (0.69, 1.84)	1.27 (0.80, 2.03)	1.25 (0.78, 2.00)	0.94 (0.56, 1.59)	0.75 (0.43, 1.32)	0.92 (0.55, 1.56)	1.08 (0.69, 1.72)	1.12 (0.71, 1.75)	1.13 (0.72, 1.77)	1.18 (0.74, 1.84)	1.04 (0.72, 1.47)	1.05 (0.67, 1.66)	0.94 (0.64, 1.34)	0.99 (0.66, 1.45)	0.96 (0.64, 1.40)	1.07 (0.65, 1.76)	0.83 (0.52, 1.32)	0.95 (0.66, 1.35)	0.79 (0.48, 1.26)	1.07 (0.72, 1.57)	1.04 (0.70, 1.52)	0.96 (0.67, 1.37)
ONE10mgSC	1.11 (0.69, 1.82)	1.24 (0.79, 2.04)	1.23 (0.77, 2.01)	0.93 (0.56, 1.57)	0.74 (0.42, 1.32)	0.92 (0.54, 1.52)	1.07 (0.68, 1.69)	1.10 (0.70, 1.72)	1.11 (0.71, 1.74)	1.16 (0.75, 1.81)	1.02 (0.71, 1.47)	1.04 (0.66, 1.64)	0.92 (0.64, 1.31)	0.98 (0.66, 1.45)	0.94 (0.63, 1.40)	1.06 (0.63, 1.75)	0.82 (0.51, 1.32)	0.94 (0.65, 1.34)	0.78 (0.47, 1.25)	1.05 (0.71, 1.55)	1.02 (0.69, 1.50)	0.95 (0.66, 1.35)
ONE25mgSC	1.18 (0.72, 1.95)	1.32 (0.82, 2.18)	1.31 (0.80, 2.14)	0.98 (0.58, 1.69)	0.79 (0.44, 1.40)	0.97 (0.57, 1.66)	1.14 (0.71, 1.86)	1.17 (0.74, 1.87)	1.18 (0.75, 1.90)	1.23 (0.80, 1.97)	1.09 (0.75, 1.60)	1.10 (0.70, 1.76)	0.98 (0.67, 1.44)	1.04 (0.69, 1.58)	1.00 (0.66, 1.52)	1.12 (0.67, 1.86)	0.87 (0.53, 1.43)	0.99 (0.68, 1.46)	0.83 (0.49, 1.35)	1.11 (0.74, 1.68)	1.08 (0.73, 1.63)	1.01 (0.69, 1.48)
ONE35mgSC	1.05 (0.65, 1.70)	1.18 (0.74, 1.89)	1.16 (0.73, 1.88)	0.88 (0.52, 1.47)	0.70 (0.40, 1.22)	0.86 (0.51, 1.45)	1.01 (0.65, 1.58)	1.04 (0.67, 1.61)	1.04 (0.67, 1.63)	1.09 (0.71, 1.69)	0.96 (0.67, 1.37)	0.98 (0.62, 1.53)	0.87 (0.60, 1.23)	0.92 (0.63, 1.35)	0.89 (0.59, 1.31)	1.00 (0.60, 1.64)	0.77 (0.47, 1.23)	0.89 (0.61, 1.25)	0.73 (0.44, 1.17)	0.99 (0.67, 1.46)	0.97 (0.65, 1.40)	0.90 (0.62, 1.28)
ONE50mgSC	1.05 (0.65, 1.73)	1.18 (0.75, 1.90)	1.16 (0.73, 1.85)	0.88 (0.53, 1.49)	0.70 (0.40, 1.23)	0.86 (0.51, 1.45)	1.01 (0.65, 1.56)	1.04 (0.67, 1.59)	1.05 (0.67, 1.62)	1.09 (0.71, 1.67)	0.97 (0.67, 1.36)	0.98 (0.62, 1.52)	0.87 (0.60, 1.23)	0.92 (0.62, 1.35)	0.89 (0.60, 1.30)	1.00 (0.60, 1.63)	0.77 (0.48, 1.24)	0.89 (0.61, 1.25)	0.73 (0.44, 1.15)	0.99 (0.68, 1.45)	0.96 (0.65, 1.41)	0.89 (0.62, 1.27)
ONT22_5mgSC	1.15 (0.75, 1.74)	1.28 (0.87, 1.93)	1.27 (0.86, 1.89)	0.96 (0.62, 1.50)	0.76 (0.46, 1.25)	0.94 (0.59, 1.47)	1.10 (0.76, 1.61)	1.13 (0.79, 1.65)	1.14 (0.79, 1.66)	1.19 (0.84, 1.71)	1.05 (0.81, 1.36)	1.07 (0.73, 1.55)	0.95 (0.73, 1.22)	1.01 (0.75, 1.35)	0.97 (0.72, 1.30)	1.08 (0.71, 1.67)	0.84 (0.56, 1.24)	0.97 (0.75, 1.23)	0.80 (0.51, 1.19)	1.08 (0.81, 1.45)	1.05 (0.78, 1.41)	0.97 (0.76, 1.25)
ONT225mgSC	1.24 (0.83, 1.92)	1.40 (0.95, 2.13)	1.38 (0.93, 2.06)	1.04 (0.65, 1.66)	0.83 (0.50, 1.38)	1.02 (0.65, 1.62)	1.20 (0.82, 1.75)	1.23 (0.85, 1.80)	1.24 (0.86, 1.83)	1.30 (0.90, 1.89)	1.14 (0.88, 1.50)	1.16 (0.79, 1.70)	1.03 (0.79, 1.34)	1.09 (0.81, 1.48)	1.05 (0.79, 1.43)	1.18 (0.77, 1.84)	0.92 (0.61, 1.35)	1.05 (0.81, 1.35)	0.87 (0.56, 1.30)	1.17 (0.88, 1.59)	1.14 (0.84, 1.55)	1.06 (0.82, 1.38)
ONT75mgSC	1.27 (0.82, 1.92)	1.42 (0.95, 2.13)	1.39 (0.93, 2.09)	1.05 (0.68, 1.66)	0.84 (0.51, 1.40)	1.04 (0.65, 1.64)	1.22 (0.82, 1.78)	1.24 (0.86, 1.83)	1.25 (0.86, 1.82)	1.31 (0.91, 1.91)	1.15 (0.89, 1.52)	1.17 (0.80, 1.73)	1.05 (0.80, 1.36)	1.11 (0.82, 1.50)	1.07 (0.79, 1.47)	1.19 (0.78, 1.87)	0.92 (0.61, 1.39)	1.06 (0.82, 1.38)	0.87 (0.56, 1.32)	1.18 (0.89, 1.61)	1.15 (0.85, 1.57)	1.07 (0.82, 1.40)
PBO	1.15 (0.80, 1.67)	1.29 (0.94, 1.85)	1.27 (0.92, 1.79)	0.96 (0.65, 1.44)	0.77 (0.49, 1.21)	0.95 (0.63, 1.41)	1.11 (0.82, 1.52)	1.14 (0.84, 1.55)	1.15 (0.85, 1.57)	1.20 (0.90, 1.63)	1.06 (0.92, 1.23)	1.07 (0.79, 1.47)	0.96 (0.82, 1.10)	1.02 (0.83, 1.24)	0.98 (0.79, 1.21)	1.09 (0.75, 1.62)	0.85 (0.61, 1.19)	0.97 (0.86, 1.10)	0.81 (0.55, 1.12)	1.09 (0.90, 1.34)	1.05 (0.86, 1.29)	0.98 (0.85, 1.13)
PF10mgSC	1.18 (0.78, 1.78)	1.32 (0.90, 1.97)	1.30 (0.89, 1.90)	0.98 (0.64, 1.54)	0.78 (0.48, 1.28)	0.97 (0.62, 1.50)	1.13 (0.78, 1.61)	1.16 (0.81, 1.66)	1.17 (0.81, 1.69)	1.22 (0.87, 1.75)	1.08 (0.85, 1.36)	1.09 (0.76, 1.58)	0.97 (0.76, 1.23)	1.03 (0.78, 1.36)	0.99 (0.75, 1.31)	1.11 (0.74, 1.72)	0.86 (0.58, 1.26)	0.99 (0.78, 1.23)	0.82 (0.54, 1.21)	1.11 (0.84, 1.47)	1.07 (0.82, 1.43)	1.00 (0.79, 1.27)
PF200mgSC	1.29 (0.85, 2.01)	1.44 (0.97, 2.23)	1.43 (0.96, 2.12)	1.08 (0.69, 1.72)	0.86 (0.52, 1.41)	1.06 (0.67, 1.69)	1.24 (0.85, 1.84)	1.27 (0.88, 1.88)	1.28 (0.89, 1.90)	1.34 (0.94, 1.97)	1.18 (0.91, 1.56)	1.20 (0.83, 1.78)	1.07 (0.82, 1.42)	1.13 (0.84, 1.55)	1.09 (0.81, 1.49)	1.22 (0.79, 1.93)	0.95 (0.63, 1.42)	1.08 (0.84, 1.43)	0.90 (0.57, 1.36)	1.21 (0.91, 1.68)	1.18 (0.88, 1.63)	1.09 (0.85, 1.44)
PF50mgSC	1.29 (0.86, 1.95)	1.44 (0.99, 2.17)	1.42 (0.98, 2.08)	1.08 (0.69, 1.70)	0.86 (0.52, 1.40)	1.06 (0.68, 1.65)	1.24 (0.85, 1.79)	1.27 (0.88, 1.85)	1.28 (0.89, 1.87)	1.34 (0.94, 1.93)	1.18 (0.92, 1.52)	1.20 (0.83, 1.75)	1.07 (0.83, 1.37)	1.13 (0.85, 1.51)	1.09 (0.81, 1.47)	1.22 (0.80, 1.89)	0.94 (0.64, 1.39)	1.08 (0.85, 1.37)	0.90 (0.58, 1.34)	1.21 (0.92, 1.63)	1.18 (0.89, 1.57)	1.10 (0.86, 1.40)
RIS1200mgIV	1.28 (0.87, 1.92)	1.44 (1.00, 2.10)	1.42 (0.99, 2.07)	1.07 (0.70, 1.65)	0.86 (0.53, 1.37)	1.06 (0.69, 1.60)	1.24 (0.88, 1.73)	1.27 (0.91, 1.78)	1.28 (0.92, 1.80)	1.34 (0.97, 1.87)	1.18 (0.96, 1.45)	1.20 (0.85, 1.70)	1.07 (0.85, 1.30)	1.13 (0.87, 1.45)	1.09 (0.84, 1.40)	1.22 (0.81, 1.85)	0.94 (0.65, 1.35)	1.08 (0.89, 1.31)	0.89 (0.59, 1.30)	1.21 (0.94, 1.56)	1.18 (0.91, 1.51)	1.09 (0.89, 1.33)
RIS200mgIV	1.25 (0.81, 1.95)	1.40 (0.94, 2.17)	1.38 (0.92, 2.11)	1.05 (0.65, 1.68)	0.83 (0.51, 1.39)	1.03 (0.64, 1.66)	1.20 (0.80, 1.80)	1.24 (0.85, 1.82)	1.24 (0.85, 1.84)	1.30 (0.90, 1.92)	1.15 (0.87, 1.54)	1.16 (0.79, 1.75)	1.03 (0.79, 1.39)	1.10 (0.80, 1.51)	1.06 (0.77, 1.47)	1.18 (0.76, 1.88)	0.92 (0.60, 1.38)	1.05 (0.80, 1.40)	0.87 (0.56, 1.34)	1.18 (0.86, 1.64)	1.14 (0.83, 1.60)	1.06 (0.81, 1.43)
RIS600mgIV	1.32 (0.91, 1.95)	**1.48 (1.04, 2.15)**	**1.46 (1.03, 2.09)**	1.10 (0.72, 1.67)	0.88 (0.55, 1.40)	1.08 (0.71, 1.65)	1.27 (0.91, 1.78)	1.30 (0.94, 1.82)	1.31 (0.94, 1.84)	1.37 (0.99, 1.91)	1.21 (1.00, 1.48)	1.22 (0.88, 1.73)	1.09 (0.90, 1.33)	1.16 (0.91, 1.49)	1.12 (0.87, 1.43)	1.25 (0.83, 1.88)	0.97 (0.68, 1.38)	1.11 (0.92, 1.34)	0.92 (0.62, 1.32)	1.24 (0.98, 1.59)	1.20 (0.95, 1.55)	1.12 (0.93, 1.36)
RIS600mgIV4_4	1.68 (0.51, 5.55)	1.90 (0.58, 6.21)	1.82 (0.57, 6.27)	1.39 (0.43, 4.90)	1.11 (0.33, 3.98)	1.36 (0.42, 4.86)	1.59 (0.50, 5.48)	1.65 (0.52, 5.48)	1.66 (0.51, 5.45)	1.73 (0.54, 5.78)	1.52 (0.50, 5.12)	1.56 (0.48, 5.19)	1.38 (0.45, 4.55)	1.47 (0.48, 4.90)	1.41 (0.46, 4.65)	1.58 (0.48, 5.57)	1.23 (0.38, 4.06)	1.40 (0.46, 4.62)	1.15 (0.35, 3.93)	1.57 (0.50, 5.28)	1.53 (0.49, 5.14)	1.41 (0.46, 4.79)
SEC10mg_kgIV	0.75 (0.38, 1.35)	0.85 (0.43, 1.53)	0.84 (0.43, 1.47)	0.63 (0.32, 1.17)	**0.50 (0.24, 0.95)**	0.62 (0.31, 1.14)	0.73 (0.38, 1.25)	0.75 (0.39, 1.29)	0.75 (0.40, 1.29)	0.79 (0.42, 1.36)	0.70 (0.38, 1.11)	0.70 (0.37, 1.23)	0.63 (0.34, 1.00)	0.67 (0.36, 1.08)	0.64 (0.35, 1.05)	0.72 (0.37, 1.29)	**0.56 (0.28, 0.97)**	0.64 (0.35, 1.02)	**0.53 (0.26, 0.94)**	0.72 (0.38, 1.17)	0.69 (0.37, 1.12)	0.65 (0.35, 1.03)

(TABLE S16B. continued)

	SEM60mgIV	SEM60mgIVx3	TES400mg200mgSC	TOF15mgOR_BID	TOF1mgOR_BID	TOF5mgOR_BID	UPA12mgOR_BID	UPA24mgOR	UPA24mgOR_BID	UPA3mgOR_BID	UPA45mgOR	UPA6mgOR_BID	UST130mgIV	UST1mg_kgIV	UST3mg_kgIV	UST4_5mg_kgIV	UST6mg_kg90mgIV_SC	UST6mg_kgIV	UST90mgSC	VED0_5mg_kgIV	VED2mg_kgIV	VED300mgIV
SEM60mgIV	SEM60mgIV	1.12 (0.82, 1.58)	1.11 (0.67, 1.81)	0.83 (0.49, 1.45)	0.67 (0.37, 1.18)	0.82 (0.48, 1.40)	0.96 (0.60, 1.58)	0.98 (0.62, 1.58)	1.00 (0.62, 1.63)	1.04 (0.66, 1.67)	0.92 (0.62, 1.34)	0.93 (0.58, 1.50)	0.83 (0.56, 1.22)	0.88 (0.57, 1.34)	0.85 (0.56, 1.28)	0.95 (0.56, 1.60)	0.73 (0.44, 1.21)	0.84 (0.57, 1.23)	0.69 (0.41, 1.15)	0.94 (0.62, 1.42)	0.92 (0.60, 1.37)	0.85 (0.57, 1.24)
SEM60mgIVx3	0.89 (0.63, 1.22)	SEM60mgIVx3	0.99 (0.61, 1.56)	0.74 (0.43, 1.25)	0.59 (0.34, 1.04)	0.73 (0.43, 1.23)	0.86 (0.54, 1.35)	0.88 (0.56, 1.37)	0.89 (0.56, 1.38)	0.93 (0.59, 1.44)	0.82 (0.56, 1.16)	0.83 (0.52, 1.29)	0.74 (0.50, 1.06)	0.78 (0.53, 1.15)	0.76 (0.50, 1.10)	0.84 (0.51, 1.41)	0.65 (0.40, 1.05)	0.75 (0.52, 1.07)	**0.62 (0.37, 0.99)**	0.84 (0.56, 1.24)	0.82 (0.55, 1.19)	0.76 (0.52, 1.08)
TES400mg200mgSC	0.90 (0.55, 1.48)	1.01 (0.64, 1.65)	TES400mg200mgSC	0.76 (0.44, 1.27)	0.60 (0.35, 1.06)	0.74 (0.44, 1.26)	0.87 (0.54, 1.36)	0.89 (0.56, 1.42)	0.90 (0.57, 1.42)	0.94 (0.60, 1.49)	0.83 (0.58, 1.18)	0.84 (0.53, 1.33)	0.75 (0.52, 1.07)	0.80 (0.54, 1.16)	0.76 (0.52, 1.13)	0.86 (0.52, 1.42)	0.66 (0.42, 1.05)	0.76 (0.53, 1.08)	0.63 (0.39, 1.01)	0.85 (0.58, 1.25)	0.83 (0.56, 1.21)	0.77 (0.53, 1.09)
TOF15mgOR_BID	1.20 (0.69, 2.04)	1.35 (0.80, 2.31)	1.32 (0.79, 2.25)	TOF15mgOR_BID	0.80 (0.51, 1.23)	0.98 (0.65, 1.48)	1.15 (0.69, 1.88)	1.18 (0.72, 1.92)	1.19 (0.72, 1.96)	1.25 (0.76, 2.03)	1.10 (0.72, 1.67)	1.11 (0.68, 1.84)	1.00 (0.64, 1.50)	1.06 (0.66, 1.67)	1.02 (0.64, 1.59)	1.14 (0.66, 1.93)	0.88 (0.51, 1.46)	1.01 (0.66, 1.53)	0.83 (0.48, 1.43)	1.13 (0.71, 1.77)	1.10 (0.70, 1.70)	1.02 (0.66, 1.55)
TOF1mgOR_BID	1.50 (0.85, 2.68)	1.70 (0.96, 2.98)	1.66 (0.94, 2.89)	1.25 (0.81, 1.98)	TOF1mgOR_BID	1.23 (0.78, 1.94)	1.44 (0.84, 2.50)	1.48 (0.87, 2.55)	1.49 (0.87, 2.58)	1.56 (0.92, 2.68)	1.37 (0.86, 2.19)	1.39 (0.81, 2.42)	1.24 (0.78, 2.00)	1.32 (0.81, 2.17)	1.27 (0.78, 2.07)	1.42 (0.81, 2.54)	1.10 (0.63, 1.95)	1.26 (0.79, 2.03)	1.05 (0.58, 1.82)	1.41 (0.87, 2.34)	1.37 (0.84, 2.27)	1.27 (0.79, 2.05)
TOF5mgOR_BID	1.23 (0.71, 2.07)	1.37 (0.81, 2.34)	1.35 (0.80, 2.26)	1.02 (0.68, 1.53)	0.82 (0.51, 1.27)	TOF5mgOR_BID	1.17 (0.71, 1.95)	1.19 (0.74, 1.98)	1.21 (0.74, 2.02)	1.26 (0.78, 2.10)	1.12 (0.72, 1.71)	1.13 (0.69, 1.88)	1.01 (0.66, 1.54)	1.07 (0.68, 1.67)	1.04 (0.66, 1.62)	1.16 (0.67, 2.00)	0.90 (0.53, 1.49)	1.02 (0.67, 1.58)	0.85 (0.49, 1.44)	1.15 (0.73, 1.82)	1.12 (0.72, 1.75)	1.04 (0.68, 1.59)
UPA12mgOR_BID	1.04 (0.63, 1.68)	1.17 (0.74, 1.84)	1.15 (0.73, 1.84)	0.87 (0.53, 1.45)	0.69 (0.40, 1.19)	0.86 (0.51, 1.41)	UPA12mgOR_BID	1.03 (0.78, 1.36)	1.04 (0.79, 1.38)	1.08 (0.83, 1.43)	0.95 (0.68, 1.34)	0.97 (0.72, 1.30)	0.86 (0.61, 1.21)	0.91 (0.63, 1.32)	0.88 (0.60, 1.27)	0.98 (0.60, 1.61)	0.76 (0.48, 1.21)	0.88 (0.63, 1.22)	0.72 (0.45, 1.15)	0.98 (0.68, 1.42)	0.95 (0.66, 1.38)	0.88 (0.63, 1.24)
UPA24mgOR	1.02 (0.63, 1.62)	1.14 (0.73, 1.80)	1.12 (0.71, 1.78)	0.85 (0.52, 1.38)	0.68 (0.39, 1.14)	0.84 (0.50, 1.35)	0.98 (0.73, 1.29)	UPA24mgOR	1.01 (0.77, 1.33)	1.05 (0.81, 1.38)	0.93 (0.66, 1.30)	0.95 (0.71, 1.26)	0.84 (0.60, 1.17)	0.89 (0.61, 1.28)	0.86 (0.59, 1.24)	0.96 (0.60, 1.55)	0.75 (0.47, 1.17)	0.85 (0.61, 1.18)	0.70 (0.44, 1.11)	0.96 (0.66, 1.37)	0.93 (0.65, 1.34)	0.86 (0.61, 1.21)
UPA24mgOR_BID	1.00 (0.61, 1.61)	1.13 (0.72, 1.78)	1.11 (0.71, 1.77)	0.84 (0.51, 1.38)	0.67 (0.39, 1.15)	0.83 (0.50, 1.36)	0.96 (0.73, 1.27)	0.99 (0.75, 1.30)	UPA24mgOR_BID	1.04 (0.81, 1.36)	0.92 (0.65, 1.29)	0.94 (0.70, 1.23)	0.83 (0.59, 1.16)	0.88 (0.61, 1.26)	0.85 (0.58, 1.22)	0.95 (0.59, 1.56)	0.74 (0.47, 1.15)	0.85 (0.60, 1.17)	0.70 (0.43, 1.11)	0.95 (0.66, 1.37)	0.92 (0.64, 1.32)	0.85 (0.60, 1.20)
UPA3mgOR_BID	0.96 (0.60, 1.52)	1.08 (0.69, 1.70)	1.06 (0.67, 1.68)	0.80 (0.49, 1.31)	0.64 (0.37, 1.08)	0.79 (0.48, 1.27)	0.92 (0.70, 1.21)	0.95 (0.73, 1.23)	0.96 (0.73, 1.23)	UPA3mgOR_BID	0.88 (0.63, 1.22)	0.90 (0.68, 1.17)	0.80 (0.57, 1.10)	0.85 (0.59, 1.21)	0.81 (0.56, 1.16)	0.91 (0.56, 1.46)	0.70 (0.45, 1.10)	0.81 (0.59, 1.12)	0.67 (0.42, 1.04)	0.90 (0.64, 1.28)	0.88 (0.62, 1.25)	0.82 (0.59, 1.12)
UPA45mgOR	1.09 (0.75, 1.62)	1.22 (0.86, 1.79)	1.20 (0.85, 1.73)	0.91 (0.60, 1.39)	0.73 (0.46, 1.17)	0.89 (0.59, 1.38)	1.05 (0.75, 1.48)	1.08 (0.77, 1.52)	1.08 (0.78, 1.53)	1.13 (0.82, 1.59)	UPA45mgOR	1.01 (0.72, 1.45)	0.91 (0.73, 1.10)	0.96 (0.75, 1.23)	0.92 (0.71, 1.19)	1.03 (0.69, 1.56)	0.80 (0.55, 1.14)	0.92 (0.76, 1.10)	0.76 (0.50, 1.11)	1.03 (0.80, 1.33)	1.00 (0.78, 1.28)	0.93 (0.76, 1.13)
UPA6mgOR_BID	1.07 (0.67, 1.73)	1.20 (0.78, 1.91)	1.19 (0.75, 1.89)	0.90 (0.54, 1.47)	0.72 (0.41, 1.23)	0.88 (0.53, 1.44)	1.03 (0.77, 1.38)	1.06 (0.80, 1.41)	1.06 (0.81, 1.43)	1.12 (0.85, 1.47)	0.99 (0.69, 1.39)	UPA6mgOR_BID	0.89 (0.63, 1.24)	0.95 (0.65, 1.38)	0.91 (0.62, 1.32)	1.01 (0.63, 1.67)	0.78 (0.50, 1.24)	0.91 (0.64, 1.25)	0.75 (0.45, 1.19)	1.01 (0.70, 1.47)	0.98 (0.67, 1.42)	0.91 (0.65, 1.29)
UST130mgIV	1.21 (0.82, 1.78)	1.35 (0.94, 1.98)	1.34 (0.94, 1.91)	1.00 (0.67, 1.55)	0.81 (0.50, 1.28)	0.99 (0.65, 1.52)	1.16 (0.82, 1.64)	1.19 (0.85, 1.67)	1.20 (0.86, 1.70)	1.26 (0.91, 1.75)	1.10 (0.91, 1.37)	1.12 (0.80, 1.59)	UST130mgIV	1.06 (0.85, 1.34)	1.02 (0.80, 1.30)	1.15 (0.76, 1.72)	0.89 (0.61, 1.27)	1.01 (0.88, 1.18)	0.84 (0.56, 1.22)	1.14 (0.90, 1.47)	1.11 (0.87, 1.42)	1.03 (0.84, 1.26)
UST1mg_kgIV	1.14 (0.75, 1.74)	1.28 (0.87, 1.90)	1.26 (0.86, 1.85)	0.95 (0.60, 1.51)	0.76 (0.46, 1.24)	0.93 (0.60, 1.47)	1.10 (0.76, 1.58)	1.12 (0.78, 1.63)	1.13 (0.79, 1.63)	1.18 (0.83, 1.70)	1.04 (0.82, 1.33)	1.06 (0.73, 1.55)	0.94 (0.74, 1.18)	UST1mg_kgIV	0.96 (0.77, 1.20)	1.08 (0.70, 1.65)	0.83 (0.56, 1.23)	0.96 (0.78, 1.17)	0.79 (0.52, 1.18)	1.07 (0.81, 1.43)	1.04 (0.79, 1.38)	0.97 (0.76, 1.24)
UST3mg_kgIV	1.18 (0.78, 1.80)	1.32 (0.91, 1.98)	1.31 (0.89, 1.94)	0.98 (0.63, 1.56)	0.79 (0.48, 1.29)	0.97 (0.62, 1.53)	1.13 (0.79, 1.65)	1.17 (0.81, 1.69)	1.18 (0.82, 1.71)	1.23 (0.86, 1.78)	1.08 (0.84, 1.40)	1.10 (0.76, 1.62)	0.98 (0.77, 1.24)	1.04 (0.83, 1.30)	UST3mg_kgIV	1.12 (0.73, 1.73)	0.86 (0.58, 1.28)	0.99 (0.80, 1.23)	0.82 (0.53, 1.23)	1.11 (0.84, 1.49)	1.08 (0.81, 1.44)	1.00 (0.78, 1.29)
UST4_5mg_kgIV	1.05 (0.62, 1.77)	1.19 (0.71, 1.97)	1.17 (0.70, 1.92)	0.88 (0.52, 1.53)	0.70 (0.39, 1.24)	0.87 (0.50, 1.48)	1.02 (0.62, 1.66)	1.04 (0.64, 1.68)	1.05 (0.64, 1.70)	1.10 (0.68, 1.77)	0.97 (0.64, 1.44)	0.99 (0.60, 1.59)	0.87 (0.58, 1.32)	0.93 (0.61, 1.42)	0.89 (0.58, 1.37)	UST4_5mg_kgIV	0.77 (0.46, 1.27)	0.89 (0.59, 1.33)	0.74 (0.42, 1.22)	0.99 (0.64, 1.52)	0.97 (0.63, 1.48)	0.90 (0.60, 1.34)
UST6mg_kg90mgIV_SC	1.37 (0.83, 2.25)	1.54 (0.96, 2.48)	1.50 (0.95, 2.38)	1.14 (0.69, 1.94)	0.91 (0.51, 1.59)	1.12 (0.67, 1.89)	1.31 (0.83, 2.09)	1.34 (0.86, 2.14)	1.36 (0.87, 2.14)	1.42 (0.91, 2.23)	1.25 (0.88, 1.80)	1.28 (0.81, 2.01)	1.13 (0.79, 1.63)	1.20 (0.81, 1.78)	1.16 (0.78, 1.71)	1.29 (0.79, 2.16)	UST6mg_kg90mgIV_SC	1.15 (0.81, 1.64)	0.95 (0.57, 1.54)	1.28 (0.87, 1.92)	1.25 (0.85, 1.86)	1.16 (0.81, 1.67)
UST6mg_kgIV	1.19 (0.81, 1.75)	1.34 (0.94, 1.93)	1.31 (0.93, 1.90)	0.99 (0.65, 1.52)	0.79 (0.49, 1.26)	0.98 (0.63, 1.50)	1.14 (0.82, 1.59)	1.17 (0.85, 1.63)	1.18 (0.85, 1.66)	1.24 (0.89, 1.71)	1.09 (0.91, 1.32)	1.10 (0.80, 1.55)	0.99 (0.85, 1.14)	1.04 (0.85, 1.29)	1.01 (0.81, 1.24)	1.13 (0.75, 1.68)	0.87 (0.61, 1.24)	UST6mg_kgIV	0.83 (0.55, 1.20)	1.12 (0.90, 1.43)	1.09 (0.86, 1.38)	1.01 (0.84, 1.22)
UST90mgSC	1.44 (0.87, 2.44)	**1.61 (1.01, 2.71)**	1.59 (0.99, 2.60)	1.20 (0.70, 2.10)	0.96 (0.55, 1.72)	1.18 (0.69, 2.06)	1.38 (0.87, 2.24)	1.42 (0.90, 2.30)	1.44 (0.90, 2.31)	1.50 (0.96, 2.39)	1.32 (0.90, 1.98)	1.34 (0.84, 2.20)	1.19 (0.82, 1.78)	1.26 (0.85, 1.94)	1.22 (0.82, 1.88)	1.36 (0.82, 2.36)	1.05 (0.65, 1.74)	1.21 (0.83, 1.81)	UST90mgSC	1.35 (0.92, 2.08)	1.31 (0.88, 2.02)	1.22 (0.85, 1.83)
VED0_5mg_kgIV	1.06 (0.71, 1.60)	1.19 (0.81, 1.77)	1.17 (0.80, 1.72)	0.88 (0.57, 1.40)	0.71 (0.43, 1.15)	0.87 (0.55, 1.36)	1.02 (0.70, 1.47)	1.05 (0.73, 1.51)	1.06 (0.73, 1.52)	1.11 (0.78, 1.57)	0.97 (0.75, 1.25)	0.99 (0.68, 1.44)	0.88 (0.68, 1.11)	0.93 (0.70, 1.24)	0.90 (0.67, 1.20)	1.01 (0.66, 1.55)	0.78 (0.52, 1.14)	0.90 (0.70, 1.11)	0.74 (0.48, 1.09)	VED0_5mg_kgIV	0.97 (0.80, 1.17)	0.90 (0.70, 1.15)
VED2mg_kgIV	1.09 (0.73, 1.66)	1.22 (0.84, 1.83)	1.21 (0.83, 1.79)	0.91 (0.59, 1.43)	0.73 (0.44, 1.19)	0.90 (0.57, 1.40)	1.05 (0.72, 1.52)	1.08 (0.75, 1.55)	1.09 (0.76, 1.56)	1.14 (0.80, 1.62)	1.00 (0.78, 1.28)	1.02 (0.70, 1.49)	0.90 (0.71, 1.15)	0.96 (0.72, 1.27)	0.93 (0.70, 1.23)	1.03 (0.67, 1.59)	0.80 (0.54, 1.18)	0.92 (0.73, 1.16)	0.76 (0.49, 1.13)	1.03 (0.86, 1.25)	VED2mg_kgIV	0.93 (0.73, 1.19)
VED300mgIV	1.18 (0.81, 1.75)	1.32 (0.92, 1.93)	1.30 (0.92, 1.87)	0.98 (0.65, 1.51)	0.78 (0.49, 1.26)	0.97 (0.63, 1.48)	1.13 (0.81, 1.59)	1.16 (0.83, 1.63)	1.17 (0.83, 1.66)	1.22 (0.89, 1.71)	1.08 (0.88, 1.31)	1.10 (0.78, 1.54)	0.98 (0.79, 1.19)	1.04 (0.81, 1.32)	1.00 (0.78, 1.28)	1.11 (0.75, 1.68)	0.86 (0.60, 1.24)	0.99 (0.82, 1.19)	0.82 (0.55, 1.18)	1.11 (0.87, 1.42)	1.08 (0.84, 1.37)	VED300mgIV

TABLE S16B. League table for infections. The table presented the multiple treatment comparisons based on consistency analysis of the networks. Treatments are depicted alphabetically. The upper quadrant is the inverse of the lower quadrant, i.e., the mirror image of the results Values are presented as relative risk (RR) with 95% credible interval (CrI). For all comparisons an RR >1 favors the occurrence of the event for the row-defining treatment, while an RR<1 favors the column-defining treatment. Statistically significant results are represented in bold. Multiple treatment comparisons based on consistency analysis of the networks. Treatments are depicted alphabetically. Values are presented as relative risk (RR) with 95% credible interval (CrI). For all comparisons an RR <1 favors the occurrence of the event for the row-defining treatment, while an RR>1 favors the column-defining treatment. Statistically significant results are represented in bold.

	ABA10mg_kgIV	ABA30mg_kgIV	ABA3mg_kgIV	ADA160mg80mg40mgSC	ADA160mg80mg60mgSC	ADA160mg80mgSC	ADA40mg20mgSC	ADA80mg40mgSC	AND150mgSC1_1	AND150mgSC2_2	AND300mgSC	BRI400mgIV	BRI700mgIV	BRO210mgIV	BRO350mgIV	BRO700mgIV	CDP10mg_kgIV	CER400mgSC	ELD10mg_kgIV	ELD20mg_kgIV	ETR105mgSC	ETR210mgSC	FIL100mgOR
ABA10mg_kgIV	ABA10mg_kgIV	0.76 (0.37, 1.46)	0.92 (0.51, 1.61)	0.88 (0.29, 2.46)	1.15 (0.37, 3.50)	1.14 (0.60, 2.29)	0.67 (0.24, 1.75)	1.13 (0.50, 2.60)	0.79 (0.03, 87.19)	0.87 (0.03, 51.55)	0.71 (0.02, 34.53)	1.20 (0.52, 2.89)	1.18 (0.55, 2.66)	1.14 (0.43, 3.03)	1.12 (0.42, 2.84)	1.58 (0.66, 3.88)	1.28 (0.64, 2.59)	3.04 (0.74, 16.10)	0.81 (0.31, 1.98)	0.97 (0.37, 2.37)	2.15 (0.97, 4.92)	2.24 (0.99, 5.11)	1.13 (0.60, 2.17)
ABA30mg_kgIV	1.32 (0.68, 2.71)	ABA30mg_kgIV	1.21 (0.62, 2.49)	1.17 (0.36, 3.62)	1.54 (0.45, 5.09)	1.51 (0.74, 3.54)	0.89 (0.30, 2.51)	1.50 (0.63, 3.87)	1.03 (0.04, 108.04)	1.17 (0.04, 70.28)	0.90 (0.03, 50.24)	1.59 (0.65, 4.31)	1.57 (0.67, 4.00)	1.50 (0.54, 4.46)	1.46 (0.55, 4.44)	2.08 (0.82, 5.82)	1.67 (0.78, 4.00)	4.06 (0.94, 21.90)	1.06 (0.38, 2.97)	1.29 (0.48, 3.55)	**2.86 (1.16, 7.40)**	**2.96 (1.24, 7.49)**	1.50 (0.74, 3.34)
ABA3mg_kgIV	1.09 (0.62, 1.96)	0.82 (0.40, 1.61)	ABA3mg_kgIV	0.96 (0.32, 2.76)	1.27 (0.40, 3.97)	1.25 (0.65, 2.53)	0.73 (0.27, 1.87)	1.23 (0.55, 2.82)	0.84 (0.03, 91.48)	0.97 (0.03, 55.63)	0.75 (0.03, 39.54)	1.31 (0.55, 3.16)	1.28 (0.58, 2.91)	1.25 (0.47, 3.33)	1.23 (0.46, 3.20)	1.72 (0.71, 4.28)	1.39 (0.69, 2.86)	3.39 (0.81, 17.44)	0.88 (0.33, 2.21)	1.06 (0.42, 2.57)	**2.36 (1.05, 5.38)**	**2.45 (1.10, 5.55)**	1.24 (0.66, 2.36)
ADA160mg80mg40mgSC	1.14 (0.41, 3.43)	0.86 (0.28, 2.76)	1.04 (0.36, 3.15)	ADA160mg80mg40mgSC	1.30 (0.33, 5.34)	1.31 (0.48, 3.72)	0.77 (0.21, 2.66)	1.29 (0.43, 4.15)	0.90 (0.03, 95.59)	1.01 (0.03, 56.28)	0.78 (0.03, 42.94)	1.38 (0.45, 4.56)	1.35 (0.46, 4.29)	1.30 (0.36, 4.74)	1.29 (0.37, 4.51)	1.78 (0.56, 5.91)	1.47 (0.52, 4.20)	3.52 (0.70, 21.15)	0.92 (0.27, 3.08)	1.10 (0.33, 3.67)	2.46 (0.80, 7.94)	2.57 (0.83, 7.97)	1.29 (0.48, 3.79)
ADA160mg80mg60mgSC	0.87 (0.29, 2.71)	0.65 (0.20, 2.20)	0.79 (0.25, 2.52)	0.77 (0.19, 2.99)	ADA160mg80mg60mgSC	0.99 (0.34, 3.05)	0.60 (0.15, 2.15)	0.98 (0.30, 3.39)	0.69 (0.02, 68.59)	0.76 (0.02, 45.36)	0.60 (0.02, 35.98)	1.05 (0.31, 3.64)	1.04 (0.32, 3.37)	0.99 (0.27, 3.74)	0.98 (0.27, 3.80)	1.38 (0.41, 5.01)	1.12 (0.37, 3.52)	2.73 (0.52, 15.72)	0.70 (0.19, 2.54)	0.84 (0.23, 3.07)	1.91 (0.56, 6.29)	1.95 (0.60, 6.48)	0.99 (0.33, 2.94)
ADA160mg80mgSC	0.87 (0.44, 1.65)	0.66 (0.28, 1.36)	0.80 (0.40, 1.55)	0.77 (0.27, 2.07)	1.01 (0.33, 2.95)	ADA160mg80mgSC	0.58 (0.25, 1.27)	0.99 (0.53, 1.79)	0.70 (0.02, 70.22)	0.75 (0.02, 46.68)	0.61 (0.02, 31.05)	1.05 (0.46, 2.28)	1.03 (0.49, 2.07)	1.00 (0.40, 2.44)	0.99 (0.39, 2.30)	1.38 (0.58, 3.08)	1.12 (0.58, 2.05)	2.64 (0.68, 13.82)	0.70 (0.28, 1.64)	0.84 (0.35, 1.92)	1.88 (0.88, 3.98)	1.94 (0.93, 4.13)	0.99 (0.55, 1.69)
ADA40mg20mgSC	1.49 (0.57, 4.17)	1.12 (0.40, 3.36)	1.36 (0.53, 3.68)	1.30 (0.38, 4.71)	1.68 (0.47, 6.84)	1.71 (0.79, 4.07)	ADA40mg20mgSC	1.70 (0.73, 4.11)	1.20 (0.04, 131.75)	1.26 (0.04, 82.58)	1.04 (0.03, 58.99)	1.80 (0.64, 5.43)	1.77 (0.66, 4.92)	1.70 (0.55, 5.38)	1.66 (0.52, 5.39)	2.34 (0.80, 7.27)	1.90 (0.77, 5.03)	4.60 (0.99, 27.61)	1.20 (0.39, 3.74)	1.45 (0.47, 4.26)	**3.17 (1.19, 9.36)**	**3.32 (1.21, 9.52)**	1.68 (0.71, 4.26)
ADA80mg40mgSC	0.88 (0.38, 1.99)	0.67 (0.26, 1.58)	0.81 (0.35, 1.83)	0.78 (0.24, 2.34)	1.02 (0.30, 3.32)	1.01 (0.56, 1.89)	0.59 (0.24, 1.38)	ADA80mg40mgSC	0.68 (0.03, 66.56)	0.75 (0.02, 46.67)	0.62 (0.02, 33.55)	1.07 (0.43, 2.73)	1.05 (0.44, 2.48)	0.99 (0.35, 2.80)	0.99 (0.35, 2.60)	1.39 (0.54, 3.63)	1.12 (0.52, 2.46)	2.70 (0.65, 13.91)	0.71 (0.25, 1.88)	0.86 (0.32, 2.18)	1.91 (0.80, 4.57)	1.96 (0.83, 4.72)	1.00 (0.48, 2.06)
AND150mgSC1_1	1.26 (0.01, 36.90)	0.97 (0.01, 27.58)	1.19 (0.01, 33.52)	1.11 (0.01, 39.94)	1.44 (0.01, 48.33)	1.43 (0.01, 42.93)	0.83 (0.01, 24.44)	1.48 (0.02, 38.38)	AND150mgSC1_1	1.03 (0.04, 33.74)	0.80 (0.03, 31.61)	1.53 (0.02, 46.57)	1.50 (0.02, 46.11)	1.40 (0.01, 51.62)	1.42 (0.01, 49.31)	2.05 (0.02, 64.93)	1.62 (0.02, 46.78)	3.95 (0.04, 146.53)	1.01 (0.01, 32.11)	1.22 (0.01, 37.92)	2.78 (0.03, 80.76)	2.86 (0.03, 81.79)	1.43 (0.01, 40.38)
AND150mgSC2_2	1.15 (0.02, 34.83)	0.86 (0.01, 27.47)	1.03 (0.02, 31.90)	0.99 (0.02, 34.02)	1.31 (0.02, 43.32)	1.34 (0.02, 40.11)	0.80 (0.01, 24.75)	1.33 (0.02, 40.29)	0.97 (0.03, 26.72)	AND150mgSC2_2	0.77 (0.03, 25.55)	1.37 (0.02, 45.92)	1.35 (0.02, 41.28)	1.33 (0.02, 43.38)	1.31 (0.02, 39.37)	1.80 (0.03, 56.64)	1.51 (0.03, 45.40)	3.58 (0.05, 141.89)	0.90 (0.02, 29.13)	1.11 (0.02, 34.76)	2.43 (0.04, 78.50)	2.57 (0.04, 82.91)	1.30 (0.02, 38.32)
AND300mgSC	1.42 (0.03, 42.78)	1.11 (0.02, 32.29)	1.34 (0.03, 39.32)	1.29 (0.02, 38.48)	1.68 (0.03, 48.97)	1.63 (0.03, 50.76)	0.96 (0.02, 31.27)	1.62 (0.03, 46.54)	1.25 (0.03, 32.41)	1.30 (0.04, 32.76)	AND300mgSC	1.76 (0.04, 54.09)	1.72 (0.04, 49.92)	1.68 (0.03, 49.48)	1.67 (0.03, 49.14)	2.24 (0.05, 65.94)	1.83 (0.04, 53.43)	4.41 (0.07, 152.76)	1.15 (0.02, 32.84)	1.40 (0.03, 41.23)	3.17 (0.06, 90.56)	3.28 (0.06, 102.22)	1.63 (0.03, 45.41)
BRI400mgIV	0.83 (0.35, 1.91)	0.63 (0.23, 1.54)	0.77 (0.32, 1.81)	0.73 (0.22, 2.22)	0.95 (0.27, 3.19)	0.95 (0.44, 2.15)	0.56 (0.18, 1.55)	0.94 (0.37, 2.35)	0.65 (0.02, 64.53)	0.73 (0.02, 40.77)	0.57 (0.02, 27.98)	BRI400mgIV	0.98 (0.54, 1.81)	0.94 (0.32, 2.69)	0.92 (0.32, 2.69)	1.31 (0.48, 3.50)	1.06 (0.46, 2.37)	2.51 (0.59, 13.90)	0.66 (0.23, 1.81)	0.81 (0.29, 2.15)	1.80 (0.72, 4.40)	1.85 (0.76, 4.61)	0.94 (0.43, 2.08)
BRI700mgIV	0.85 (0.38, 1.82)	0.64 (0.25, 1.50)	0.78 (0.34, 1.71)	0.74 (0.23, 2.16)	0.96 (0.30, 3.14)	0.97 (0.48, 2.03)	0.56 (0.20, 1.53)	0.95 (0.40, 2.26)	0.67 (0.02, 66.08)	0.74 (0.02, 44.80)	0.58 (0.02, 28.41)	1.02 (0.55, 1.84)	BRI700mgIV	0.96 (0.34, 2.71)	0.94 (0.33, 2.62)	1.32 (0.52, 3.44)	1.08 (0.49, 2.30)	2.58 (0.62, 13.56)	0.67 (0.25, 1.75)	0.82 (0.31, 2.08)	1.82 (0.78, 4.37)	1.88 (0.81, 4.43)	0.96 (0.47, 1.96)
BRO210mgIV	0.87 (0.33, 2.33)	0.67 (0.22, 1.86)	0.80 (0.30, 2.14)	0.77 (0.21, 2.77)	1.01 (0.27, 3.76)	1.00 (0.41, 2.53)	0.59 (0.19, 1.82)	1.01 (0.36, 2.84)	0.71 (0.02, 71.56)	0.75 (0.02, 47.06)	0.60 (0.02, 30.87)	1.06 (0.37, 3.09)	1.05 (0.37, 2.95)	BRO210mgIV	0.99 (0.42, 2.28)	1.38 (0.66, 2.99)	1.12 (0.43, 2.87)	2.61 (0.58, 16.50)	0.71 (0.22, 2.19)	0.86 (0.27, 2.57)	1.90 (0.68, 5.30)	1.94 (0.70, 5.47)	0.99 (0.41, 2.46)
BRO350mgIV	0.90 (0.35, 2.36)	0.69 (0.23, 1.83)	0.82 (0.31, 2.15)	0.78 (0.22, 2.69)	1.02 (0.26, 3.68)	1.01 (0.43, 2.59)	0.60 (0.19, 1.94)	1.01 (0.38, 2.83)	0.71 (0.02, 77.61)	0.76 (0.03, 49.89)	0.60 (0.02, 31.73)	1.08 (0.37, 3.15)	1.07 (0.38, 2.99)	1.01 (0.44, 2.39)	BRO350mgIV	1.41 (0.67, 3.10)	1.15 (0.46, 2.95)	2.67 (0.60, 16.68)	0.72 (0.23, 2.19)	0.88 (0.28, 2.60)	1.94 (0.73, 5.42)	1.99 (0.74, 5.64)	1.02 (0.43, 2.46)
BRO700mgIV	0.63 (0.26, 1.52)	0.48 (0.17, 1.23)	0.58 (0.23, 1.40)	0.56 (0.17, 1.79)	0.73 (0.20, 2.46)	0.72 (0.32, 1.73)	0.43 (0.14, 1.26)	0.72 (0.28, 1.85)	0.49 (0.02, 53.08)	0.56 (0.02, 36.27)	0.45 (0.02, 22.15)	0.76 (0.29, 2.07)	0.76 (0.29, 1.92)	0.72 (0.33, 1.51)	0.71 (0.32, 1.48)	BRO700mgIV	0.81 (0.34, 1.91)	1.92 (0.44, 10.91)	0.51 (0.17, 1.47)	0.62 (0.21, 1.73)	1.37 (0.53, 3.53)	1.42 (0.54, 3.63)	0.72 (0.32, 1.60)
CDP10mg_kgIV	0.78 (0.39, 1.55)	0.60 (0.25, 1.29)	0.72 (0.35, 1.45)	0.68 (0.24, 1.92)	0.89 (0.28, 2.68)	0.89 (0.49, 1.72)	0.53 (0.20, 1.30)	0.89 (0.41, 1.93)	0.62 (0.02, 61.65)	0.66 (0.02, 38.28)	0.55 (0.02, 28.09)	0.94 (0.42, 2.16)	0.93 (0.43, 2.02)	0.89 (0.35, 2.33)	0.87 (0.34, 2.20)	1.24 (0.52, 2.91)	CDP10mg_kgIV	2.40 (0.61, 12.20)	0.63 (0.24, 1.50)	0.76 (0.31, 1.77)	1.67 (0.78, 3.66)	1.74 (0.81, 3.83)	0.89 (0.48, 1.63)
CER400mgSC	0.33 (0.06, 1.34)	0.25 (0.05, 1.07)	0.30 (0.06, 1.24)	0.28 (0.05, 1.43)	0.37 (0.06, 1.92)	0.38 (0.07, 1.47)	0.22 (0.04, 1.01)	0.37 (0.07, 1.54)	0.25 (0.01, 27.50)	0.28 (0.01, 19.37)	0.23 (0.01, 13.42)	0.40 (0.07, 1.70)	0.39 (0.07, 1.62)	0.38 (0.06, 1.73)	0.37 (0.06, 1.68)	0.52 (0.09, 2.26)	0.42 (0.08, 1.65)	CER400mgSC	0.26 (0.04, 1.20)	0.31 (0.05, 1.46)	0.72 (0.13, 3.17)	0.74 (0.14, 3.12)	0.37 (0.07, 1.47)
ELD10mg_kgIV	1.24 (0.51, 3.27)	0.94 (0.34, 2.66)	1.14 (0.45, 3.00)	1.09 (0.32, 3.72)	1.43 (0.39, 5.16)	1.43 (0.61, 3.53)	0.84 (0.27, 2.56)	1.41 (0.53, 3.98)	0.99 (0.03, 99.26)	1.11 (0.03, 64.07)	0.87 (0.03, 43.24)	1.51 (0.55, 4.39)	1.48 (0.57, 4.04)	1.41 (0.46, 4.62)	1.38 (0.46, 4.33)	1.95 (0.68, 5.79)	1.58 (0.67, 4.11)	3.79 (0.84, 22.51)	ELD10mg_kgIV	1.22 (0.53, 2.84)	**2.69 (1.03, 7.48)**	**2.79 (1.06, 7.69)**	1.40 (0.61, 3.52)
ELD20mg_kgIV	1.03 (0.42, 2.68)	0.77 (0.28, 2.10)	0.94 (0.39, 2.40)	0.91 (0.27, 2.99)	1.19 (0.33, 4.27)	1.19 (0.52, 2.90)	0.69 (0.23, 2.11)	1.17 (0.46, 3.16)	0.82 (0.03, 84.68)	0.90 (0.03, 55.72)	0.72 (0.02, 37.35)	1.24 (0.47, 3.42)	1.22 (0.48, 3.24)	1.16 (0.39, 3.68)	1.14 (0.38, 3.54)	1.62 (0.58, 4.71)	1.32 (0.57, 3.20)	3.19 (0.68, 18.51)	0.82 (0.35, 1.90)	ELD20mg_kgIV	2.22 (0.86, 6.09)	2.29 (0.91, 6.21)	1.16 (0.52, 2.77)
ETR105mgSC	0.46 (0.20, 1.03)	**0.35 (0.14, 0.86)**	**0.42 (0.19, 0.95)**	0.41 (0.13, 1.24)	0.52 (0.16, 1.78)	0.53 (0.25, 1.13)	**0.32 (0.11, 0.84)**	0.52 (0.22, 1.26)	0.36 (0.01, 38.64)	0.41 (0.01, 25.21)	0.32 (0.01, 16.41)	0.56 (0.23, 1.39)	0.55 (0.23, 1.28)	0.53 (0.19, 1.48)	0.52 (0.18, 1.37)	0.73 (0.28, 1.88)	0.60 (0.27, 1.28)	1.38 (0.32, 7.69)	**0.37 (0.13, 0.97)**	0.45 (0.16, 1.16)	ETR105mgSC	1.03 (0.63, 1.73)	0.53 (0.26, 1.08)
ETR210mgSC	0.45 (0.20, 1.01)	**0.34 (0.13, 0.81)**	**0.41 (0.18, 0.91)**	0.39 (0.13, 1.21)	0.51 (0.15, 1.67)	0.51 (0.24, 1.08)	**0.30 (0.11, 0.82)**	0.51 (0.21, 1.20)	0.35 (0.01, 35.08)	0.39 (0.01, 24.15)	0.30 (0.01, 15.48)	0.54 (0.22, 1.31)	0.53 (0.23, 1.24)	0.52 (0.18, 1.43)	0.50 (0.18, 1.35)	0.70 (0.28, 1.84)	0.57 (0.26, 1.23)	1.35 (0.32, 7.28)	**0.36 (0.13, 0.94)**	0.44 (0.16, 1.10)	0.97 (0.58, 1.59)	ETR210mgSC	0.51 (0.25, 1.03)

(TABLE S16C. continued)

	ABA10mg_kgIV	ABA30mg_kgIV	ABA3mg_kgIV	ADA160mg80mg40mgSC	ADA160mg80mg60mgSC	ADA160mg80mgSC	ADA40mg20mgSC	ADA80mg40mgSC	AND150mgSC1_1	AND150mgSC2_2	AND300mgSC	BRI400mgIV	BRI700mgIV	BRO210mgIV	BRO350mgIV	BRO700mgIV	CDP10mg_kgIV	CER400mgSC	ELD10mg_kgIV	ELD20mg_kgIV	ETR105mgSC	ETR210mgSC	FIL100mgOR
FIL100mgOR	0.88 (0.46, 1.66)	0.67 (0.30, 1.35)	0.81 (0.42, 1.51)	0.77 (0.26, 2.09)	1.01 (0.34, 2.99)	1.01 (0.59, 1.83)	0.59 (0.23, 1.41)	1.00 (0.49, 2.07)	0.70 (0.02, 73.28)	0.77 (0.03, 43.22)	0.61 (0.02, 30.73)	1.06 (0.48, 2.30)	1.04 (0.51, 2.11)	1.01 (0.41, 2.46)	0.98 (0.41, 2.30)	1.39 (0.63, 3.10)	1.13 (0.61, 2.07)	2.67 (0.68, 13.72)	0.71 (0.28, 1.63)	0.86 (0.36, 1.92)	1.88 (0.92, 3.87)	1.96 (0.97, 4.08)	FIL100mgOR
FIL200mgOR	0.81 (0.42, 1.47)	0.62 (0.27, 1.23)	0.74 (0.38, 1.38)	0.71 (0.25, 1.88)	0.93 (0.31, 2.69)	0.92 (0.54, 1.63)	0.54 (0.21, 1.28)	0.91 (0.44, 1.88)	0.64 (0.02, 65.97)	0.70 (0.02, 41.01)	0.56 (0.02, 28.09)	0.97 (0.44, 2.08)	0.95 (0.47, 1.90)	0.92 (0.37, 2.23)	0.90 (0.38, 2.14)	1.27 (0.57, 2.79)	1.03 (0.56, 1.84)	2.43 (0.63, 12.30)	0.65 (0.26, 1.49)	0.79 (0.33, 1.73)	1.73 (0.85, 3.56)	1.79 (0.88, 3.73)	0.92 (0.61, 1.32)
FON1mg_kg0_1mgIV_SC	0.69 (0.02, 39.05)	0.52 (0.01, 30.56)	0.62 (0.02, 36.44)	0.63 (0.01, 36.90)	0.79 (0.02, 52.15)	0.78 (0.02, 42.31)	0.46 (0.01, 26.86)	0.78 (0.02, 46.26)	0.67 (0.01, 120.90)	0.74 (0.00, 129.38)	0.60 (0.00, 62.61)	0.82 (0.02, 54.52)	0.81 (0.02, 50.67)	0.82 (0.02, 45.34)	0.81 (0.02, 39.21)	1.10 (0.03, 59.54)	0.88 (0.02, 52.19)	2.18 (0.04, 142.10)	0.55 (0.01, 34.45)	0.67 (0.02, 42.67)	1.56 (0.04, 85.97)	1.56 (0.04, 86.28)	0.76 (0.02, 42.46)
FON1mg_kg1mgIV_SC	0.81 (0.01, 25.35)	0.61 (0.01, 18.67)	0.73 (0.01, 22.65)	0.71 (0.01, 24.23)	0.92 (0.01, 29.54)	0.95 (0.02, 26.23)	0.53 (0.01, 16.44)	0.92 (0.01, 26.13)	0.57 (0.00, 222.53)	0.70 (0.00, 158.76)	0.56 (0.00, 95.36)	0.98 (0.02, 30.68)	0.96 (0.01, 28.30)	0.95 (0.01, 27.70)	0.91 (0.01, 25.10)	1.26 (0.02, 37.83)	1.04 (0.02, 29.66)	2.56 (0.04, 90.53)	0.64 (0.01, 20.35)	0.79 (0.01, 24.38)	1.79 (0.03, 52.37)	1.83 (0.03, 56.42)	0.92 (0.02, 26.72)
FON4mg_kg0_1mgIV_SC	0.82 (0.02, 26.01)	0.62 (0.01, 20.62)	0.75 (0.02, 23.27)	0.70 (0.01, 27.75)	0.94 (0.02, 34.38)	0.96 (0.02, 29.21)	0.57 (0.01, 17.74)	0.96 (0.02, 30.10)	0.62 (0.01, 156.49)	0.73 (0.01, 81.91)	0.57 (0.00, 62.68)	0.97 (0.02, 32.38)	0.96 (0.02, 31.77)	0.94 (0.02, 34.72)	0.92 (0.02, 34.23)	1.30 (0.03, 43.71)	1.07 (0.02, 32.00)	2.48 (0.05, 111.25)	0.64 (0.01, 19.88)	0.80 (0.02, 25.11)	1.78 (0.04, 61.23)	1.82 (0.04, 62.34)	0.94 (0.02, 29.28)
FON4mg_kg1mgIV_SC	0.74 (0.01, 32.18)	0.55 (0.01, 23.14)	0.67 (0.01, 29.18)	0.65 (0.01, 32.49)	0.84 (0.01, 37.06)	0.86 (0.01, 35.86)	0.52 (0.01, 20.32)	0.85 (0.01, 33.08)	0.53 (0.01, 82.55)	0.63 (0.00, 97.80)	0.53 (0.00, 53.19)	0.90 (0.01, 39.40)	0.89 (0.01, 35.90)	0.87 (0.01, 36.41)	0.84 (0.01, 37.60)	1.18 (0.02, 49.17)	0.95 (0.02, 38.82)	2.28 (0.03, 106.58)	0.61 (0.01, 28.17)	0.74 (0.01, 33.70)	1.60 (0.02, 73.23)	1.64 (0.03, 68.61)	0.84 (0.01, 35.58)
GUS1200mgIV	1.14 (0.45, 3.14)	0.87 (0.31, 2.50)	1.04 (0.41, 2.91)	0.99 (0.36, 2.86)	1.30 (0.36, 4.91)	1.29 (0.55, 3.62)	0.77 (0.24, 2.51)	1.29 (0.48, 3.73)	0.89 (0.03, 95.25)	0.97 (0.03, 55.24)	0.79 (0.03, 42.80)	1.37 (0.50, 4.17)	1.35 (0.51, 3.98)	1.30 (0.41, 4.43)	1.27 (0.41, 4.21)	1.81 (0.62, 5.40)	1.45 (0.59, 3.83)	3.49 (0.78, 20.14)	0.91 (0.29, 2.90)	1.09 (0.38, 3.45)	2.42 (0.90, 7.31)	2.52 (0.94, 7.43)	1.27 (0.56, 3.42)
GUS200mgIV	1.41 (0.54, 3.87)	1.06 (0.37, 3.18)	1.30 (0.48, 3.50)	1.23 (0.44, 3.49)	1.61 (0.44, 6.31)	1.62 (0.63, 4.31)	0.96 (0.28, 3.01)	1.61 (0.56, 4.68)	1.14 (0.03, 104.52)	1.23 (0.04, 64.29)	0.98 (0.03, 51.82)	1.70 (0.59, 5.11)	1.67 (0.62, 4.77)	1.61 (0.49, 5.45)	1.58 (0.48, 5.22)	2.22 (0.75, 7.19)	1.81 (0.71, 4.92)	4.40 (0.89, 23.69)	1.13 (0.35, 3.47)	1.37 (0.44, 4.14)	**3.04 (1.06, 8.99)**	**3.15 (1.12, 9.02)**	1.61 (0.64, 4.13)
GUS600mgIV	0.96 (0.38, 2.39)	0.72 (0.27, 1.96)	0.88 (0.35, 2.23)	0.86 (0.30, 2.15)	1.11 (0.31, 3.86)	1.09 (0.46, 2.68)	0.64 (0.21, 1.99)	1.09 (0.40, 2.88)	0.77 (0.02, 69.91)	0.85 (0.03, 46.41)	0.66 (0.02, 34.67)	1.16 (0.42, 3.23)	1.13 (0.43, 3.05)	1.10 (0.34, 3.37)	1.07 (0.35, 3.19)	1.50 (0.53, 4.33)	1.22 (0.50, 3.04)	2.97 (0.63, 15.76)	0.77 (0.25, 2.24)	0.93 (0.31, 2.64)	2.09 (0.80, 5.52)	2.17 (0.81, 5.71)	1.09 (0.47, 2.57)
MED700IV	2.20 (0.65, 8.96)	1.68 (0.47, 7.56)	2.01 (0.60, 8.71)	1.96 (0.48, 9.61)	2.53 (0.58, 13.56)	2.55 (0.79, 10.35)	1.51 (0.37, 7.28)	2.53 (0.70, 11.20)	1.84 (0.05, 210.79)	1.97 (0.06, 145.29)	1.60 (0.05, 95.13)	2.70 (0.77, 11.57)	2.68 (0.77, 10.95)	2.50 (0.65, 12.64)	2.48 (0.64, 11.65)	3.50 (0.95, 16.03)	2.82 (0.88, 11.61)	**6.91 (1.23, 47.34)**	1.79 (0.46, 8.33)	2.16 (0.58, 10.25)	**4.78 (1.36, 21.17)**	**4.93 (1.42, 21.79)**	2.47 (0.82, 10.08)
NAT300mgIV	0.69 (0.37, 1.26)	0.52 (0.24, 1.04)	0.63 (0.33, 1.14)	0.60 (0.22, 1.57)	0.79 (0.27, 2.26)	0.79 (0.48, 1.37)	0.46 (0.19, 1.06)	0.77 (0.40, 1.55)	0.54 (0.02, 56.80)	0.61 (0.02, 33.17)	0.48 (0.02, 23.85)	0.83 (0.40, 1.73)	0.81 (0.43, 1.58)	0.79 (0.33, 1.83)	0.77 (0.33, 1.77)	1.08 (0.51, 2.37)	0.88 (0.51, 1.52)	2.08 (0.56, 10.22)	0.56 (0.23, 1.24)	0.67 (0.29, 1.45)	1.47 (0.77, 2.99)	1.53 (0.79, 3.08)	0.78 (0.48, 1.28)
NAT3mg_kgIV	1.03 (0.31, 3.35)	0.77 (0.23, 2.76)	0.94 (0.29, 3.09)	0.89 (0.22, 3.72)	1.16 (0.27, 5.12)	1.17 (0.39, 3.80)	0.68 (0.18, 2.62)	1.16 (0.35, 3.95)	0.83 (0.03, 85.13)	0.93 (0.02, 51.92)	0.71 (0.02, 38.57)	1.24 (0.37, 4.50)	1.22 (0.38, 4.15)	1.18 (0.31, 4.26)	1.13 (0.30, 4.25)	1.63 (0.45, 5.88)	1.30 (0.42, 4.28)	3.15 (0.58, 20.04)	0.83 (0.22, 3.02)	1.01 (0.27, 3.69)	2.20 (0.65, 7.89)	2.28 (0.68, 8.08)	1.16 (0.40, 3.63)
NAT3mg_kgIVx2	0.86 (0.26, 2.50)	0.64 (0.18, 2.10)	0.78 (0.24, 2.37)	0.74 (0.18, 2.83)	0.97 (0.23, 3.99)	0.98 (0.33, 2.83)	0.57 (0.15, 2.01)	0.97 (0.29, 3.06)	0.68 (0.02, 69.73)	0.74 (0.02, 48.95)	0.59 (0.02, 33.74)	1.02 (0.30, 3.42)	1.02 (0.31, 3.18)	0.97 (0.26, 3.40)	0.95 (0.25, 3.39)	1.35 (0.36, 4.63)	1.10 (0.34, 3.22)	2.59 (0.46, 16.77)	0.69 (0.18, 2.36)	0.84 (0.23, 2.77)	1.84 (0.55, 6.01)	1.91 (0.58, 6.11)	0.97 (0.33, 2.78)
NAT6mg_kgIVx2	1.35 (0.37, 5.53)	1.02 (0.26, 4.62)	1.22 (0.33, 5.23)	1.21 (0.27, 5.99)	1.54 (0.34, 8.51)	1.51 (0.46, 6.12)	0.89 (0.22, 4.27)	1.51 (0.41, 6.95)	1.10 (0.03, 111.65)	1.23 (0.03, 80.10)	0.99 (0.02, 55.95)	1.64 (0.42, 6.77)	1.60 (0.44, 6.60)	1.53 (0.37, 7.25)	1.50 (0.35, 7.19)	2.11 (0.51, 9.51)	1.69 (0.47, 6.98)	4.22 (0.71, 30.39)	1.09 (0.26, 5.01)	1.31 (0.33, 5.71)	2.91 (0.75, 12.53)	3.02 (0.78, 12.87)	1.52 (0.45, 6.07)
NNC2mg_kgSC	1.70 (0.61, 5.34)	1.28 (0.42, 4.31)	1.56 (0.53, 5.00)	1.50 (0.40, 6.04)	1.98 (0.51, 7.95)	1.97 (0.74, 6.05)	1.15 (0.34, 4.25)	1.93 (0.67, 6.55)	1.34 (0.04, 149.38)	1.53 (0.05, 91.99)	1.22 (0.04, 69.17)	2.05 (0.69, 7.10)	2.01 (0.71, 6.67)	1.93 (0.60, 7.20)	1.93 (0.57, 6.69)	2.69 (0.86, 9.40)	2.19 (0.81, 6.74)	**5.29 (1.03, 33.31)**	1.37 (0.41, 5.01)	1.67 (0.51, 5.89)	**3.70 (1.26, 12.43)**	**3.79 (1.30, 13.12)**	1.95 (0.76, 5.85)
PBO	0.79 (0.47, 1.30)	0.60 (0.29, 1.11)	0.73 (0.42, 1.21)	0.69 (0.26, 1.72)	0.91 (0.33, 2.48)	0.90 (0.60, 1.43)	0.53 (0.22, 1.15)	0.89 (0.48, 1.66)	0.62 (0.02, 65.01)	0.69 (0.02, 38.06)	0.55 (0.02, 27.57)	0.94 (0.49, 1.90)	0.93 (0.52, 1.71)	0.90 (0.39, 2.01)	0.88 (0.39, 1.94)	1.24 (0.61, 2.59)	1.01 (0.64, 1.61)	2.39 (0.65, 11.80)	0.64 (0.28, 1.34)	0.77 (0.35, 1.56)	1.69 (0.93, 3.19)	1.75 (0.97, 3.35)	0.89 (0.61, 1.35)
PF10mgSC	0.33 (0.01, 3.67)	0.25 (0.00, 2.79)	0.31 (0.00, 3.50)	0.28 (0.00, 3.64)	0.37 (0.01, 5.09)	0.39 (0.01, 4.19)	0.22 (0.00, 2.83)	0.38 (0.01, 4.23)	0.20 (0.00, 38.16)	0.24 (0.00, 32.04)	0.20 (0.00, 21.32)	0.41 (0.01, 4.65)	0.40 (0.01, 4.27)	0.37 (0.01, 4.47)	0.36 (0.00, 4.33)	0.51 (0.01, 5.98)	0.43 (0.01, 4.70)	0.99 (0.01, 18.07)	0.27 (0.00, 3.18)	0.32 (0.00, 3.77)	0.73 (0.01, 7.89)	0.75 (0.01, 8.27)	0.39 (0.01, 4.03)
PF200mgSC	0.21 (0.00, 2.35)	0.15 (0.00, 1.83)	0.19 (0.00, 2.18)	0.17 (0.00, 2.37)	0.22 (0.00, 3.10)	0.24 (0.00, 2.58)	0.13 (0.00, 1.75)	0.23 (0.00, 2.76)	0.11 (0.00, 31.38)	0.14 (0.00, 22.04)	0.12 (0.00, 15.36)	0.24 (0.00, 3.05)	0.24 (0.00, 2.81)	0.22 (0.00, 2.92)	0.22 (0.00, 2.85)	0.31 (0.01, 3.98)	0.26 (0.00, 2.99)	0.59 (0.01, 10.40)	0.16 (0.00, 1.96)	0.19 (0.00, 2.35)	0.44 (0.01, 5.49)	0.45 (0.01, 5.69)	0.23 (0.00, 2.72)
PF50mgSC	0.35 (0.01, 4.33)	0.27 (0.01, 3.34)	0.32 (0.01, 3.82)	0.31 (0.01, 4.09)	0.38 (0.01, 5.90)	0.40 (0.01, 4.94)	0.23 (0.00, 3.13)	0.40 (0.01, 5.29)	0.21 (0.00, 50.74)	0.27 (0.00, 34.52)	0.20 (0.00, 24.30)	0.43 (0.01, 5.68)	0.42 (0.01, 5.18)	0.40 (0.01, 5.26)	0.38 (0.01, 5.28)	0.55 (0.01, 6.74)	0.45 (0.01, 5.70)	1.05 (0.02, 21.70)	0.27 (0.01, 3.45)	0.35 (0.01, 4.17)	0.76 (0.01, 9.83)	0.78 (0.02, 9.80)	0.40 (0.01, 4.71)
RIS1200mgIV	**4.18 (1.53, 13.62)**	**3.18 (1.09, 11.50)**	**3.84 (1.42, 12.49)**	3.79 (0.98, 15.01)	**4.87 (1.25, 21.73)**	**4.82 (1.89, 16.01)**	2.87 (0.82, 11.14)	**4.83 (1.63, 15.96)**	3.46 (0.10, 390.06)	3.65 (0.12, 215.00)	3.05 (0.10, 159.42)	**5.16 (1.63, 18.16)**	**5.07 (1.75, 17.12)**	**4.81 (1.45, 18.63)**	**4.85 (1.41, 17.57)**	**6.58 (2.13, 24.31)**	**5.37 (2.01, 17.68)**	**12.99 (2.63, 94.88)**	**3.38 (1.05, 12.49)**	**4.19 (1.26, 14.95)**	**9.11 (3.10, 32.06)**	**9.45 (3.21, 32.77)**	**4.77 (1.86, 15.13)**
RIS200mgIV	3.37 (0.46, 103.95)	2.52 (0.33, 84.65)	3.06 (0.41, 99.30)	3.01 (0.31, 97.91)	3.97 (0.41, 138.44)	3.89 (0.55, 121.24)	2.26 (0.28, 75.54)	3.82 (0.52, 115.25)	2.95 (0.06, 537.59)	3.22 (0.07, 537.38)	2.55 (0.05, 318.88)	4.22 (0.52, 126.88)	4.08 (0.53, 125.85)	3.83 (0.48, 137.45)	3.84 (0.46, 125.20)	5.24 (0.67, 171.82)	4.32 (0.59, 142.15)	10.78 (0.97, 408.08)	2.73 (0.33, 85.76)	3.29 (0.41, 103.97)	7.29 (0.88, 242.68)	7.53 (0.92, 249.86)	3.84 (0.53, 119.19)
RIS600mgIV	**3.11 (1.26, 8.53)**	2.36 (0.82, 6.98)	**2.85 (1.16, 7.85)**	2.72 (0.76, 9.63)	3.57 (0.99, 14.20)	**3.57 (1.51, 10.00)**	2.09 (0.66, 7.04)	**3.54 (1.30, 10.65)**	2.47 (0.07, 324.10)	2.68 (0.09, 197.29)	2.10 (0.07, 120.90)	**3.74 (1.33, 11.59)**	**3.68 (1.41, 10.80)**	**3.53 (1.19, 11.80)**	**3.50 (1.12, 10.85)**	**4.96 (1.67, 14.85)**	**3.96 (1.64, 10.72)**	**9.61 (2.12, 56.62)**	2.47 (0.80, 8.22)	**3.01 (1.04, 9.64)**	**6.75 (2.51, 19.53)**	**6.99 (2.63, 20.38)**	**3.51 (1.55, 9.60)**
SEC10mg_kgIV	**0.07 (0.00, 0.38)**	**0.05 (0.00, 0.29)**	**0.06 (0.00, 0.34)**	**0.06 (0.00, 0.39)**	**0.07 (0.00, 0.51)**	**0.08 (0.00, 0.43)**	**0.04 (0.00, 0.28)**	**0.07 (0.00, 0.44)**	0.05 (0.00, 7.53)	0.05 (0.00, 4.90)	0.04 (0.00, 3.39)	**0.08 (0.00, 0.49)**	**0.08 (0.00, 0.46)**	**0.07 (0.00, 0.46)**	**0.07 (0.00, 0.45)**	**0.10 (0.01, 0.63)**	**0.08 (0.00, 0.47)**	0.19 (0.01, 2.07)	**0.05 (0.00, 0.33)**	**0.06 (0.00, 0.39)**	**0.14 (0.01, 0.82)**	**0.15 (0.01, 0.87)**	**0.08 (0.00, 0.41)**

	ABA10mg_kgIV	ABA30mg_kgIV	ABA3mg_kgIV	ADA160mg80mg40mgSC	ADA160mg80mg60mgSC	ADA160mg80mgSC	ADA40mg20mgSC	ADA80mg40mgSC	AND150mgSC1_1	AND150mgSC2_2	AND300mgSC	BRI400mgIV	BRI700mgIV	BRO210mgIV	BRO350mgIV	BRO700mgIV	CDP10mg_kgIV	CER400mgSC	ELD10mg_kgIV	ELD20mg_kgIV	ETR105mgSC	ETR210mgSC	FIL100mgOR
SEM60mgIV	0.71 (0.28, 1.79)	0.54 (0.19, 1.49)	0.65 (0.26, 1.68)	0.63 (0.19, 2.07)	0.84 (0.23, 2.71)	0.82 (0.35, 2.03)	0.48 (0.15, 1.44)	0.80 (0.31, 2.20)	0.56 (0.02, 59.92)	0.64 (0.02, 37.32)	0.50 (0.02, 26.89)	0.86 (0.32, 2.43)	0.85 (0.32, 2.23)	0.80 (0.27, 2.44)	0.79 (0.27, 2.43)	1.12 (0.40, 3.16)	0.91 (0.38, 2.26)	2.19 (0.49, 12.00)	0.57 (0.18, 1.66)	0.69 (0.24, 1.98)	1.51 (0.59, 4.17)	1.58 (0.62, 4.30)	0.80 (0.36, 1.95)
SEM60mgIVx3	1.21 (0.45, 3.44)	0.93 (0.31, 2.79)	1.12 (0.38, 3.14)	1.05 (0.30, 3.71)	1.41 (0.36, 5.16)	1.38 (0.54, 3.94)	0.81 (0.25, 2.77)	1.36 (0.49, 4.29)	0.97 (0.03, 110.83)	1.09 (0.03, 70.36)	0.87 (0.03, 47.23)	1.48 (0.49, 4.49)	1.43 (0.51, 4.20)	1.37 (0.42, 4.55)	1.35 (0.42, 4.61)	1.93 (0.61, 5.97)	1.53 (0.60, 4.30)	3.74 (0.78, 22.92)	0.97 (0.29, 3.24)	1.17 (0.38, 3.71)	2.61 (0.91, 7.79)	2.71 (0.90, 8.13)	1.37 (0.55, 3.70)
TES400mg200mgSC	**0.28 (0.11, 0.70)**	**0.22 (0.07, 0.56)**	**0.26 (0.10, 0.65)**	**0.25 (0.07, 0.82)**	0.32 (0.09, 1.14)	**0.33 (0.13, 0.78)**	**0.19 (0.06, 0.56)**	**0.32 (0.11, 0.86)**	0.22 (0.01, 23.83)	0.25 (0.01, 14.62)	0.20 (0.01, 10.30)	**0.34 (0.12, 0.92)**	**0.34 (0.12, 0.87)**	0.32 (0.10, 1.00)	0.32 (0.10, 1.02)	0.45 (0.14, 1.30)	**0.37 (0.14, 0.87)**	0.86 (0.18, 4.94)	**0.23 (0.07, 0.63)**	**0.27 (0.09, 0.78)**	0.61 (0.21, 1.65)	0.63 (0.22, 1.67)	**0.32 (0.13, 0.75)**
TOF15mgOR_BID	1.34 (0.42, 4.71)	0.98 (0.29, 3.88)	1.22 (0.37, 4.40)	1.16 (0.28, 5.32)	1.52 (0.34, 7.14)	1.54 (0.49, 5.45)	0.89 (0.23, 3.67)	1.49 (0.44, 5.79)	1.05 (0.03, 100.51)	1.15 (0.04, 78.91)	0.92 (0.03, 51.02)	1.59 (0.46, 6.15)	1.58 (0.47, 5.77)	1.56 (0.39, 6.01)	1.50 (0.39, 5.76)	2.13 (0.58, 8.03)	1.70 (0.54, 5.97)	4.11 (0.73, 28.93)	1.07 (0.29, 4.12)	1.28 (0.35, 5.14)	2.88 (0.84, 10.53)	2.96 (0.87, 11.07)	1.52 (0.50, 5.19)
TOF1mgOR_BID	1.38 (0.43, 5.22)	1.04 (0.30, 4.00)	1.26 (0.39, 4.79)	1.24 (0.30, 5.50)	1.60 (0.35, 7.71)	1.61 (0.51, 5.68)	0.93 (0.25, 3.83)	1.58 (0.45, 5.90)	1.13 (0.03, 130.26)	1.21 (0.04, 82.30)	0.96 (0.03, 51.99)	1.67 (0.46, 6.87)	1.64 (0.48, 6.49)	1.62 (0.41, 6.68)	1.57 (0.40, 6.34)	2.19 (0.59, 8.96)	1.78 (0.54, 6.39)	4.34 (0.76, 27.94)	1.12 (0.29, 4.69)	1.34 (0.37, 5.48)	3.04 (0.85, 11.81)	3.13 (0.88, 11.91)	1.59 (0.52, 5.52)
TOF5mgOR_BID	0.79 (0.28, 2.31)	0.59 (0.19, 1.86)	0.72 (0.26, 2.10)	0.69 (0.18, 2.73)	0.91 (0.23, 3.74)	0.90 (0.34, 2.62)	0.52 (0.16, 1.92)	0.90 (0.29, 2.82)	0.63 (0.02, 63.76)	0.68 (0.02, 48.29)	0.54 (0.02, 29.78)	0.96 (0.31, 3.10)	0.94 (0.31, 2.88)	0.91 (0.27, 3.13)	0.89 (0.25, 2.99)	1.26 (0.39, 4.06)	1.01 (0.36, 2.98)	2.48 (0.49, 14.59)	0.63 (0.19, 2.11)	0.78 (0.23, 2.50)	1.70 (0.55, 5.26)	1.77 (0.58, 5.45)	0.89 (0.34, 2.47)
UPA12mgOR_BID	0.57 (0.23, 1.31)	0.43 (0.16, 1.08)	0.52 (0.21, 1.20)	0.50 (0.15, 1.52)	0.64 (0.19, 2.19)	0.65 (0.28, 1.48)	0.38 (0.12, 1.09)	0.65 (0.24, 1.61)	0.45 (0.01, 48.31)	0.50 (0.01, 29.31)	0.39 (0.01, 21.04)	0.68 (0.26, 1.81)	0.67 (0.26, 1.65)	0.64 (0.21, 1.88)	0.64 (0.21, 1.79)	0.89 (0.33, 2.37)	0.73 (0.30, 1.67)	1.71 (0.40, 9.59)	0.45 (0.16, 1.27)	0.56 (0.19, 1.51)	1.22 (0.47, 3.07)	1.27 (0.49, 3.18)	0.65 (0.28, 1.41)
UPA24mgOR	0.74 (0.29, 1.84)	0.56 (0.19, 1.49)	0.67 (0.26, 1.69)	0.65 (0.18, 2.02)	0.85 (0.25, 2.87)	0.85 (0.36, 2.01)	0.50 (0.16, 1.48)	0.83 (0.30, 2.20)	0.59 (0.02, 58.69)	0.64 (0.02, 36.25)	0.51 (0.02, 26.98)	0.89 (0.33, 2.44)	0.87 (0.34, 2.25)	0.84 (0.27, 2.61)	0.83 (0.27, 2.41)	1.15 (0.41, 3.24)	0.94 (0.37, 2.32)	2.24 (0.52, 12.66)	0.59 (0.19, 1.72)	0.71 (0.24, 2.09)	1.59 (0.61, 4.24)	1.64 (0.60, 4.43)	0.84 (0.36, 1.97)
UPA24mgOR_BID	0.45 (0.19, 1.02)	**0.34 (0.13, 0.83)**	**0.41 (0.17, 0.95)**	0.40 (0.12, 1.21)	0.51 (0.16, 1.70)	0.52 (0.23, 1.13)	**0.31 (0.10, 0.83)**	0.51 (0.20, 1.24)	0.36 (0.01, 38.90)	0.40 (0.01, 23.67)	0.31 (0.01, 16.56)	0.54 (0.21, 1.39)	0.53 (0.22, 1.30)	0.51 (0.17, 1.46)	0.50 (0.18, 1.37)	0.70 (0.27, 1.84)	0.58 (0.25, 1.26)	1.35 (0.33, 7.74)	**0.36 (0.13, 0.96)**	0.44 (0.16, 1.16)	0.96 (0.38, 2.41)	1.00 (0.40, 2.50)	0.51 (0.23, 1.10)
UPA3mgOR_BID	0.62 (0.24, 1.47)	0.46 (0.16, 1.18)	0.57 (0.22, 1.32)	0.54 (0.16, 1.68)	0.71 (0.21, 2.35)	0.70 (0.31, 1.61)	0.41 (0.13, 1.16)	0.69 (0.26, 1.76)	0.49 (0.02, 52.80)	0.55 (0.02, 30.41)	0.42 (0.01, 22.84)	0.73 (0.28, 1.90)	0.72 (0.29, 1.77)	0.69 (0.23, 2.09)	0.69 (0.23, 1.93)	0.96 (0.35, 2.59)	0.78 (0.32, 1.87)	1.85 (0.45, 10.43)	0.49 (0.17, 1.36)	0.59 (0.20, 1.71)	1.33 (0.50, 3.31)	1.36 (0.52, 3.48)	0.70 (0.30, 1.53)
UPA45mgOR	0.58 (0.18, 1.78)	0.44 (0.13, 1.46)	0.53 (0.16, 1.63)	0.51 (0.12, 1.90)	0.67 (0.16, 2.68)	0.67 (0.21, 1.95)	0.39 (0.10, 1.39)	0.66 (0.19, 2.13)	0.48 (0.01, 42.49)	0.52 (0.02, 27.10)	0.42 (0.01, 19.57)	0.71 (0.20, 2.37)	0.69 (0.20, 2.18)	0.66 (0.18, 2.29)	0.65 (0.17, 2.25)	0.92 (0.25, 3.09)	0.75 (0.23, 2.18)	1.79 (0.33, 11.55)	0.46 (0.12, 1.66)	0.57 (0.15, 1.93)	1.26 (0.37, 4.08)	1.29 (0.38, 4.13)	0.66 (0.21, 1.88)
UPA6mgOR_BID	0.49 (0.21, 1.10)	**0.37 (0.14, 0.92)**	0.45 (0.18, 1.02)	0.44 (0.13, 1.30)	0.57 (0.17, 1.82)	0.57 (0.25, 1.23)	**0.33 (0.11, 0.93)**	0.55 (0.22, 1.36)	0.39 (0.01, 40.91)	0.44 (0.01, 25.60)	0.34 (0.01, 17.57)	0.59 (0.23, 1.53)	0.58 (0.23, 1.41)	0.56 (0.19, 1.60)	0.55 (0.19, 1.48)	0.77 (0.29, 2.06)	0.63 (0.27, 1.40)	1.49 (0.36, 8.04)	0.39 (0.14, 1.09)	0.48 (0.17, 1.31)	1.06 (0.43, 2.62)	1.10 (0.43, 2.68)	0.56 (0.25, 1.16)
UST130mgIV	0.97 (0.52, 1.82)	0.73 (0.34, 1.50)	0.89 (0.48, 1.69)	0.85 (0.31, 2.26)	1.12 (0.37, 3.20)	1.10 (0.67, 1.99)	0.65 (0.26, 1.54)	1.09 (0.55, 2.29)	0.76 (0.03, 79.96)	0.85 (0.03, 45.70)	0.67 (0.02, 33.77)	1.16 (0.56, 2.52)	1.14 (0.60, 2.32)	1.11 (0.45, 2.67)	1.08 (0.46, 2.57)	1.53 (0.71, 3.40)	1.23 (0.71, 2.21)	2.93 (0.78, 15.10)	0.78 (0.33, 1.75)	0.95 (0.41, 2.11)	**2.08 (1.06, 4.37)**	**2.15 (1.10, 4.52)**	1.09 (0.68, 1.91)
UST1mg_kgIV	0.72 (0.36, 1.46)	0.55 (0.24, 1.20)	0.66 (0.33, 1.36)	0.64 (0.22, 1.82)	0.83 (0.27, 2.44)	0.83 (0.44, 1.62)	0.49 (0.18, 1.22)	0.81 (0.38, 1.83)	0.56 (0.02, 60.91)	0.63 (0.02, 36.13)	0.50 (0.02, 26.11)	0.87 (0.39, 2.00)	0.86 (0.41, 1.84)	0.82 (0.32, 2.15)	0.81 (0.32, 2.02)	1.15 (0.49, 2.68)	0.92 (0.48, 1.84)	2.19 (0.55, 11.51)	0.58 (0.23, 1.45)	0.71 (0.29, 1.71)	1.55 (0.72, 3.56)	1.61 (0.75, 3.68)	0.82 (0.45, 1.56)
UST3mg_kgIV	0.82 (0.40, 1.70)	0.62 (0.27, 1.42)	0.75 (0.36, 1.60)	0.72 (0.24, 2.11)	0.95 (0.31, 2.90)	0.93 (0.50, 1.88)	0.55 (0.20, 1.42)	0.92 (0.42, 2.13)	0.64 (0.02, 72.02)	0.71 (0.02, 41.62)	0.57 (0.02, 29.55)	0.99 (0.43, 2.32)	0.97 (0.46, 2.12)	0.94 (0.36, 2.47)	0.91 (0.36, 2.34)	1.29 (0.55, 3.16)	1.04 (0.53, 2.12)	2.48 (0.62, 13.31)	0.66 (0.26, 1.66)	0.80 (0.32, 2.00)	1.75 (0.81, 4.04)	1.81 (0.84, 4.26)	0.92 (0.51, 1.84)
UST4_5mg_kgIV	1.41 (0.40, 6.00)	1.06 (0.27, 4.74)	1.29 (0.34, 5.38)	1.26 (0.27, 5.87)	1.61 (0.34, 9.17)	1.60 (0.47, 6.77)	0.95 (0.23, 4.73)	1.61 (0.41, 6.84)	1.17 (0.03, 122.92)	1.29 (0.04, 86.16)	1.00 (0.03, 53.05)	1.70 (0.44, 7.43)	1.66 (0.45, 7.24)	1.63 (0.38, 7.23)	1.58 (0.36, 7.04)	2.24 (0.58, 9.98)	1.82 (0.50, 7.87)	4.29 (0.75, 34.44)	1.13 (0.27, 5.30)	1.36 (0.33, 6.16)	3.09 (0.81, 12.95)	3.17 (0.83, 13.70)	1.61 (0.47, 6.25)
UST6mg_kg90mgIV_SC	1.35 (0.53, 3.77)	1.03 (0.36, 2.96)	1.24 (0.47, 3.40)	1.20 (0.78, 1.82)	1.56 (0.43, 5.96)	1.57 (0.64, 4.21)	0.92 (0.28, 2.98)	1.53 (0.56, 4.77)	1.08 (0.03, 115.59)	1.22 (0.03, 67.25)	0.94 (0.03, 49.46)	1.64 (0.57, 4.87)	1.61 (0.60, 4.64)	1.56 (0.47, 5.23)	1.52 (0.48, 5.05)	2.11 (0.73, 6.65)	1.75 (0.69, 4.72)	4.14 (0.90, 23.63)	1.10 (0.35, 3.35)	1.32 (0.43, 4.00)	**2.97 (1.04, 8.62)**	**3.09 (1.07, 8.80)**	1.55 (0.63, 4.08)
UST6mg_kgIV	0.79 (0.43, 1.45)	0.60 (0.28, 1.19)	0.72 (0.40, 1.32)	0.69 (0.26, 1.82)	0.91 (0.33, 2.57)	0.90 (0.56, 1.57)	0.54 (0.22, 1.24)	0.90 (0.46, 1.81)	0.61 (0.02, 67.32)	0.69 (0.02, 39.24)	0.55 (0.02, 28.15)	0.95 (0.46, 2.01)	0.93 (0.50, 1.85)	0.91 (0.38, 2.18)	0.89 (0.38, 2.07)	1.24 (0.59, 2.70)	1.01 (0.58, 1.78)	2.38 (0.64, 12.18)	0.64 (0.27, 1.42)	0.78 (0.34, 1.66)	1.70 (0.88, 3.47)	1.76 (0.93, 3.56)	0.89 (0.57, 1.48)
UST90mgSC	0.93 (0.24, 3.97)	0.71 (0.17, 3.17)	0.87 (0.21, 3.78)	0.83 (0.16, 4.17)	1.07 (0.20, 5.78)	1.09 (0.29, 4.48)	0.63 (0.14, 3.02)	1.07 (0.26, 4.91)	0.77 (0.02, 84.27)	0.81 (0.02, 61.81)	0.65 (0.02, 43.89)	1.17 (0.27, 4.99)	1.15 (0.27, 4.86)	1.05 (0.24, 5.84)	1.05 (0.23, 5.30)	1.48 (0.34, 7.13)	1.20 (0.31, 5.01)	2.95 (0.42, 23.74)	0.75 (0.17, 3.58)	0.93 (0.20, 4.19)	2.05 (0.47, 9.01)	2.13 (0.50, 9.23)	1.07 (0.28, 4.41)
VED0_5mg_kgIV	0.63 (0.02, 19.64)	0.48 (0.02, 14.82)	0.59 (0.02, 17.39)	0.56 (0.02, 19.54)	0.71 (0.02, 25.34)	0.74 (0.02, 22.98)	0.43 (0.02, 14.72)	0.73 (0.02, 21.68)	0.55 (0.00, 92.46)	0.56 (0.00, 73.69)	0.46 (0.00, 59.01)	0.79 (0.02, 25.57)	0.75 (0.02, 24.28)	0.73 (0.02, 23.19)	0.69 (0.02, 23.12)	1.00 (0.03, 30.81)	0.82 (0.03, 25.24)	2.06 (0.05, 75.67)	0.51 (0.02, 16.22)	0.63 (0.02, 20.80)	1.39 (0.04, 41.75)	1.43 (0.05, 44.01)	0.72 (0.02, 21.98)
VED2mg_kgIV	0.86 (0.03, 21.92)	0.65 (0.02, 18.06)	0.77 (0.03, 21.32)	0.75 (0.02, 22.73)	0.99 (0.03, 30.14)	0.96 (0.03, 27.90)	0.55 (0.02, 17.44)	0.98 (0.03, 26.62)	0.70 (0.01, 195.50)	0.71 (0.01, 160.30)	0.58 (0.01, 135.31)	1.03 (0.03, 28.46)	1.02 (0.03, 27.76)	0.97 (0.03, 27.85)	0.94 (0.03, 27.56)	1.36 (0.04, 37.89)	1.09 (0.04, 30.99)	2.52 (0.07, 112.67)	0.69 (0.02, 21.06)	0.85 (0.03, 23.96)	1.83 (0.06, 52.76)	1.89 (0.06, 57.18)	0.96 (0.04, 27.28)
VED300mgIV	0.85 (0.39, 1.75)	0.65 (0.26, 1.47)	0.78 (0.36, 1.64)	0.75 (0.24, 2.19)	0.98 (0.30, 2.98)	0.97 (0.48, 1.99)	0.57 (0.20, 1.49)	0.95 (0.42, 2.22)	0.68 (0.02, 69.39)	0.73 (0.02, 44.69)	0.59 (0.02, 31.94)	1.03 (0.43, 2.54)	1.01 (0.44, 2.29)	0.96 (0.36, 2.63)	0.94 (0.35, 2.49)	1.33 (0.53, 3.37)	1.09 (0.52, 2.21)	2.61 (0.60, 13.72)	0.67 (0.26, 1.73)	0.83 (0.31, 2.07)	1.83 (0.79, 4.23)	1.88 (0.82, 4.40)	0.96 (0.50, 1.87)

	FIL200mgOR	FON1mg_kg0_1mgIV_SC	FON1mg_kg1mgIV_SC	FON4mg_kg0_1mgIV_SC	FON4mg_kg1mgIV_SC	GUS1200mgIV	GUS200mgIV	GUS600mgIV	MED700IV	NAT300mgIV	NAT3mg_kgIV	NAT3mg_kgIVx2	NAT6mg_kgIVx2	NNC2mg_kgSC	PBO	PF10mgSC	PF200mgSC	PF50mgSC	RIS1200mgIV	RIS200mgIV	RIS600mgIV	SEC10mg_kgIV
ABA10mg_kgIV	1.24 (0.68, 2.38)	1.45 (0.03, 55.25)	1.24 (0.04, 73.86)	1.22 (0.04, 61.25)	1.35 (0.03, 85.57)	0.88 (0.32, 2.23)	0.71 (0.26, 1.86)	1.04 (0.42, 2.64)	0.45 (0.11, 1.53)	1.45 (0.80, 2.67)	0.97 (0.30, 3.17)	1.16 (0.40, 3.86)	0.74 (0.18, 2.67)	0.59 (0.19, 1.63)	1.27 (0.77, 2.12)	2.99 (0.27, 193.17)	4.85 (0.43, 290.21)	2.83 (0.23, 140.38)	**0.24 (0.07, 0.65)**	0.30 (0.01, 2.19)	**0.32 (0.12, 0.80)**	**15.20 (2.61, 281.66)**
ABA30mg_kgIV	1.63 (0.81, 3.75)	1.93 (0.03, 75.13)	1.65 (0.05, 100.27)	1.62 (0.05, 81.85)	1.82 (0.04, 119.91)	1.15 (0.40, 3.26)	0.94 (0.31, 2.72)	1.40 (0.51, 3.76)	0.60 (0.13, 2.14)	1.92 (0.96, 4.11)	1.30 (0.36, 4.36)	1.56 (0.48, 5.43)	0.98 (0.22, 3.86)	0.78 (0.23, 2.39)	1.68 (0.90, 3.40)	4.07 (0.36, 264.51)	6.55 (0.55, 427.51)	3.74 (0.30, 192.40)	**0.31 (0.09, 0.92)**	0.40 (0.01, 3.06)	0.42 (0.14, 1.22)	**20.65 (3.40, 364.41)**
ABA3mg_kgIV	1.35 (0.73, 2.64)	1.61 (0.03, 59.48)	1.37 (0.04, 78.72)	1.34 (0.04, 66.54)	1.50 (0.03, 96.31)	0.96 (0.34, 2.45)	0.77 (0.29, 2.07)	1.14 (0.45, 2.82)	0.50 (0.11, 1.67)	1.59 (0.88, 3.08)	1.07 (0.32, 3.42)	1.27 (0.42, 4.17)	0.82 (0.19, 3.00)	0.64 (0.20, 1.90)	1.38 (0.83, 2.37)	3.26 (0.29, 211.08)	5.34 (0.46, 325.19)	3.14 (0.26, 147.25)	**0.26 (0.08, 0.70)**	0.33 (0.01, 2.45)	**0.35 (0.13, 0.86)**	**16.61 (2.92, 279.31)**
ADA160mg80mg40mgSC	1.41 (0.53, 4.06)	1.60 (0.03, 75.52)	1.41 (0.04, 83.72)	1.42 (0.04, 71.39)	1.55 (0.03, 111.53)	1.01 (0.35, 2.80)	0.81 (0.29, 2.29)	1.17 (0.46, 3.35)	0.51 (0.10, 2.10)	1.66 (0.64, 4.62)	1.12 (0.27, 4.55)	1.36 (0.35, 5.56)	0.83 (0.17, 3.66)	0.67 (0.17, 2.48)	1.44 (0.58, 3.84)	3.56 (0.27, 226.27)	5.93 (0.42, 348.29)	3.27 (0.24, 168.38)	0.26 (0.07, 1.02)	0.33 (0.01, 3.26)	0.37 (0.10, 1.32)	**17.57 (2.53, 342.98)**
ADA160mg80mg60mgSC	1.08 (0.37, 3.22)	1.26 (0.02, 57.96)	1.08 (0.03, 74.59)	1.06 (0.03, 64.02)	1.19 (0.03, 96.85)	0.77 (0.20, 2.79)	0.62 (0.16, 2.26)	0.90 (0.26, 3.21)	0.39 (0.07, 1.73)	1.26 (0.44, 3.66)	0.86 (0.20, 3.64)	1.03 (0.25, 4.42)	0.65 (0.12, 2.95)	0.51 (0.13, 1.97)	1.10 (0.40, 3.04)	2.73 (0.20, 162.11)	4.54 (0.32, 283.12)	2.60 (0.17, 121.19)	**0.21 (0.05, 0.80)**	0.25 (0.01, 2.43)	0.28 (0.07, 1.01)	**13.79 (1.94, 262.70)**
ADA160mg80mgSC	1.09 (0.61, 1.85)	1.28 (0.02, 45.54)	1.06 (0.04, 65.39)	1.04 (0.03, 53.03)	1.16 (0.03, 74.96)	0.78 (0.28, 1.82)	0.62 (0.23, 1.58)	0.92 (0.37, 2.16)	0.39 (0.10, 1.26)	1.27 (0.73, 2.07)	0.85 (0.26, 2.57)	1.02 (0.35, 3.08)	0.66 (0.16, 2.17)	0.51 (0.17, 1.34)	1.11 (0.70, 1.66)	2.55 (0.24, 168.65)	4.25 (0.39, 250.21)	2.48 (0.20, 120.75)	**0.21 (0.06, 0.53)**	0.26 (0.01, 1.82)	**0.28 (0.10, 0.66)**	**13.11 (2.32, 249.59)**
ADA40mg20mgSC	1.84 (0.78, 4.69)	2.16 (0.04, 86.93)	1.88 (0.06, 128.41)	1.75 (0.06, 101.02)	1.92 (0.05, 146.83)	1.31 (0.40, 4.19)	1.04 (0.33, 3.55)	1.56 (0.50, 4.87)	0.66 (0.14, 2.67)	2.16 (0.94, 5.31)	1.47 (0.38, 5.56)	1.75 (0.50, 6.52)	1.12 (0.23, 4.65)	0.87 (0.24, 2.95)	1.88 (0.87, 4.45)	4.52 (0.35, 284.21)	7.51 (0.57, 478.89)	4.37 (0.32, 213.92)	0.35 (0.09, 1.22)	0.44 (0.01, 3.58)	0.48 (0.14, 1.53)	**23.47 (3.55, 512.98)**
ADA80mg40mgSC	1.10 (0.53, 2.27)	1.28 (0.02, 50.05)	1.08 (0.04, 66.76)	1.05 (0.03, 56.62)	1.17 (0.03, 77.80)	0.77 (0.27, 2.10)	0.62 (0.21, 1.78)	0.92 (0.35, 2.47)	0.40 (0.09, 1.43)	1.29 (0.65, 2.53)	0.86 (0.25, 2.89)	1.03 (0.33, 3.50)	0.66 (0.14, 2.44)	0.52 (0.15, 1.50)	1.12 (0.60, 2.08)	2.65 (0.24, 171.09)	4.26 (0.36, 252.48)	2.53 (0.19, 118.98)	**0.21 (0.06, 0.61)**	0.26 (0.01, 1.93)	**0.28 (0.09, 0.77)**	**13.54 (2.25, 254.21)**
AND150mgSC1_1	1.56 (0.02, 44.91)	1.49 (0.01, 198.30)	1.74 (0.00, 203.79)	1.62 (0.01, 185.40)	1.88 (0.01, 198.36)	1.13 (0.01, 31.04)	0.88 (0.01, 28.71)	1.30 (0.01, 42.79)	0.54 (0.00, 18.33)	1.86 (0.02, 53.88)	1.21 (0.01, 37.68)	1.47 (0.01, 49.05)	0.91 (0.01, 37.29)	0.75 (0.01, 22.95)	1.61 (0.02, 45.71)	5.03 (0.03, 394.98)	8.76 (0.03, 610.09)	4.78 (0.02, 304.62)	0.29 (0.00, 10.06)	0.34 (0.00, 17.07)	0.41 (0.00, 13.37)	21.56 (0.13, 1120.81)
AND150mgSC2_2	1.42 (0.02, 43.22)	1.36 (0.01, 223.18)	1.43 (0.01, 211.58)	1.37 (0.01, 174.81)	1.58 (0.01, 243.33)	1.03 (0.02, 32.82)	0.81 (0.02, 25.66)	1.18 (0.02, 36.43)	0.51 (0.01, 17.27)	1.65 (0.03, 49.13)	1.07 (0.02, 41.17)	1.35 (0.02, 48.75)	0.81 (0.01, 30.66)	0.65 (0.01, 21.87)	1.44 (0.03, 42.17)	4.20 (0.03, 901.80)	7.05 (0.05, 1479.72)	3.67 (0.03, 642.33)	0.27 (0.00, 8.06)	0.31 (0.00, 15.25)	0.37 (0.01, 11.68)	18.70 (0.20, 1477.20)
AND300mgSC	1.80 (0.04, 51.85)	1.65 (0.02, 454.35)	1.79 (0.01, 281.88)	1.75 (0.02, 252.21)	1.87 (0.02, 308.23)	1.27 (0.02, 39.74)	1.02 (0.02, 29.79)	1.51 (0.03, 45.30)	0.63 (0.01, 20.82)	2.10 (0.04, 59.06)	1.40 (0.03, 45.74)	1.71 (0.03, 56.09)	1.01 (0.02, 40.64)	0.82 (0.01, 25.71)	1.82 (0.04, 51.68)	5.06 (0.05, 850.42)	8.67 (0.07, 1552.24)	4.92 (0.04, 713.72)	0.33 (0.01, 10.42)	0.39 (0.00, 18.94)	0.48 (0.01, 13.74)	25.18 (0.30, 1402.27)
BRI400mgIV	1.03 (0.48, 2.27)	1.22 (0.02, 50.00)	1.03 (0.03, 64.89)	1.03 (0.03, 49.76)	1.11 (0.03, 72.23)	0.73 (0.24, 1.98)	0.59 (0.20, 1.71)	0.86 (0.31, 2.39)	0.37 (0.09, 1.31)	1.21 (0.58, 2.50)	0.81 (0.22, 2.73)	0.98 (0.29, 3.35)	0.61 (0.15, 2.39)	0.49 (0.14, 1.45)	1.06 (0.53, 2.03)	2.43 (0.22, 164.80)	4.10 (0.33, 246.14)	2.33 (0.18, 114.20)	**0.19 (0.06, 0.61)**	0.24 (0.01, 1.91)	**0.27 (0.09, 0.75)**	**13.03 (2.04, 231.76)**
BRI700mgIV	1.05 (0.53, 2.11)	1.23 (0.02, 50.16)	1.04 (0.04, 69.77)	1.04 (0.03, 51.89)	1.12 (0.03, 72.97)	0.74 (0.25, 1.95)	0.60 (0.21, 1.61)	0.88 (0.33, 2.31)	0.37 (0.09, 1.29)	1.23 (0.63, 2.32)	0.82 (0.24, 2.65)	0.98 (0.31, 3.23)	0.62 (0.15, 2.28)	0.50 (0.15, 1.41)	1.08 (0.59, 1.92)	2.50 (0.23, 172.14)	4.17 (0.36, 252.44)	2.37 (0.19, 116.02)	**0.20 (0.06, 0.57)**	0.24 (0.01, 1.89)	**0.27 (0.09, 0.71)**	**13.01 (2.17, 224.21)**
BRO210mgIV	1.08 (0.45, 2.68)	1.22 (0.02, 54.74)	1.05 (0.04, 76.20)	1.07 (0.03, 57.42)	1.14 (0.03, 76.42)	0.77 (0.23, 2.42)	0.62 (0.18, 2.04)	0.91 (0.30, 2.90)	0.40 (0.08, 1.54)	1.27 (0.55, 3.04)	0.85 (0.23, 3.25)	1.03 (0.29, 3.92)	0.65 (0.14, 2.72)	0.52 (0.14, 1.68)	1.11 (0.50, 2.57)	2.70 (0.22, 171.82)	4.49 (0.34, 251.16)	2.50 (0.19, 133.66)	**0.21 (0.05, 0.69)**	0.26 (0.01, 2.09)	**0.28 (0.08, 0.84)**	**13.50 (2.16, 230.39)**
BRO350mgIV	1.11 (0.47, 2.66)	1.23 (0.03, 54.93)	1.10 (0.04, 72.86)	1.08 (0.03, 56.94)	1.19 (0.03, 78.66)	0.79 (0.24, 2.44)	0.63 (0.19, 2.07)	0.93 (0.31, 2.90)	0.40 (0.09, 1.57)	1.30 (0.56, 3.07)	0.88 (0.24, 3.29)	1.05 (0.29, 4.01)	0.67 (0.14, 2.89)	0.52 (0.15, 1.76)	1.14 (0.51, 2.56)	2.78 (0.23, 203.84)	4.58 (0.35, 275.32)	2.63 (0.19, 129.77)	**0.21 (0.06, 0.71)**	0.26 (0.01, 2.20)	**0.29 (0.09, 0.89)**	**13.60 (2.24, 242.03)**
BRO700mgIV	0.79 (0.36, 1.76)	0.91 (0.02, 37.89)	0.79 (0.03, 48.69)	0.77 (0.02, 36.59)	0.85 (0.02, 53.45)	0.55 (0.19, 1.61)	0.45 (0.14, 1.34)	0.67 (0.23, 1.90)	0.29 (0.06, 1.05)	0.93 (0.42, 1.98)	0.61 (0.17, 2.24)	0.74 (0.22, 2.76)	0.47 (0.11, 1.97)	0.37 (0.11, 1.16)	0.81 (0.39, 1.63)	1.95 (0.17, 132.01)	3.18 (0.25, 186.99)	1.82 (0.15, 92.25)	**0.15 (0.04, 0.47)**	0.19 (0.01, 1.49)	**0.20 (0.07, 0.60)**	**9.65 (1.59, 169.23)**
CDP10mg_kgIV	0.97 (0.54, 1.78)	1.14 (0.02, 46.32)	0.96 (0.03, 59.19)	0.93 (0.03, 44.97)	1.05 (0.03, 66.29)	0.69 (0.26, 1.69)	0.55 (0.20, 1.41)	0.82 (0.33, 1.98)	0.35 (0.09, 1.14)	1.14 (0.66, 1.96)	0.77 (0.23, 2.35)	0.91 (0.31, 2.93)	0.59 (0.14, 2.12)	0.46 (0.15, 1.23)	0.99 (0.62, 1.56)	2.31 (0.21, 154.58)	3.85 (0.33, 231.38)	2.24 (0.18, 115.66)	**0.19 (0.06, 0.50)**	0.23 (0.01, 1.69)	**0.25 (0.09, 0.61)**	**12.09 (2.15, 202.36)**
CER400mgSC	0.41 (0.08, 1.59)	0.46 (0.01, 23.57)	0.39 (0.01, 27.33)	0.40 (0.01, 19.95)	0.44 (0.01, 30.58)	0.29 (0.05, 1.28)	0.23 (0.04, 1.12)	0.34 (0.06, 1.59)	**0.14 (0.02, 0.81)**	0.48 (0.10, 1.79)	0.32 (0.05, 1.72)	0.39 (0.06, 2.18)	0.24 (0.03, 1.41)	**0.19 (0.03, 0.97)**	0.42 (0.08, 1.53)	1.01 (0.06, 76.85)	1.70 (0.10, 116.10)	0.96 (0.05, 63.03)	**0.08 (0.01, 0.38)**	0.09 (0.00, 1.03)	**0.10 (0.02, 0.47)**	5.14 (0.48, 109.50)
ELD10mg_kgIV	1.54 (0.67, 3.83)	1.81 (0.03, 75.37)	1.56 (0.05, 92.23)	1.56 (0.05, 77.17)	1.65 (0.04, 101.53)	1.10 (0.34, 3.47)	0.88 (0.29, 2.87)	1.30 (0.45, 3.97)	0.56 (0.12, 2.18)	1.80 (0.81, 4.34)	1.20 (0.33, 4.63)	1.44 (0.42, 5.62)	0.92 (0.20, 3.89)	0.73 (0.20, 2.44)	1.56 (0.74, 3.52)	3.77 (0.31, 265.00)	6.41 (0.51, 360.55)	3.66 (0.29, 187.64)	**0.30 (0.08, 0.96)**	0.37 (0.01, 3.07)	0.40 (0.12, 1.25)	**19.34 (3.02, 346.79)**
ELD20mg_kgIV	1.27 (0.58, 3.06)	1.50 (0.02, 62.81)	1.27 (0.04, 78.43)	1.25 (0.04, 62.44)	1.36 (0.03, 91.37)	0.92 (0.29, 2.66)	0.73 (0.24, 2.29)	1.07 (0.38, 3.21)	0.46 (0.10, 1.72)	1.49 (0.69, 3.40)	0.99 (0.27, 3.66)	1.19 (0.36, 4.43)	0.76 (0.18, 3.07)	0.60 (0.17, 1.95)	1.30 (0.64, 2.87)	3.13 (0.27, 215.05)	5.15 (0.43, 303.70)	2.89 (0.24, 137.67)	**0.24 (0.07, 0.80)**	0.30 (0.01, 2.41)	**0.33 (0.10, 0.96)**	**16.14 (2.58, 289.24)**
ETR105mgSC	0.58 (0.28, 1.18)	0.64 (0.01, 27.15)	0.56 (0.02, 39.00)	0.56 (0.02, 27.92)	0.63 (0.01, 41.57)	0.41 (0.14, 1.11)	**0.33 (0.11, 0.94)**	0.48 (0.18, 1.26)	**0.21 (0.05, 0.73)**	0.68 (0.33, 1.30)	0.46 (0.13, 1.54)	0.54 (0.17, 1.83)	0.34 (0.08, 1.34)	**0.27 (0.08, 0.79)**	0.59 (0.31, 1.08)	1.37 (0.13, 96.03)	2.27 (0.18, 137.71)	1.32 (0.10, 68.89)	**0.11 (0.03, 0.32)**	0.14 (0.00, 1.13)	**0.15 (0.05, 0.40)**	**7.06 (1.22, 123.89)**
ETR210mgSC	0.56 (0.27, 1.14)	0.64 (0.01, 24.41)	0.55 (0.02, 34.91)	0.55 (0.02, 27.85)	0.61 (0.01, 37.53)	0.40 (0.13, 1.06)	**0.32 (0.11, 0.89)**	0.46 (0.18, 1.23)	**0.20 (0.05, 0.70)**	0.65 (0.32, 1.27)	0.44 (0.12, 1.47)	0.52 (0.16, 1.71)	0.33 (0.08, 1.29)	**0.26 (0.08, 0.77)**	0.57 (0.30, 1.03)	1.33 (0.12, 91.37)	2.22 (0.18, 139.75)	1.28 (0.10, 63.68)	**0.11 (0.03, 0.31)**	0.13 (0.00, 1.09)	**0.14 (0.05, 0.38)**	**6.71 (1.14, 121.35)**

	FIL200mgOR	FON1mg_kg0_1mgIV_SC	FON1mg_kg1mgIV_SC		FON4mg_kg0_1mgIV_SC	FON4mg_kg1mgIV_SC	GUS1200mgIV	GUS200mgIV	GUS600mgIV	MED700IV	NAT300mgIV	NAT3mg_kgIV	NAT3mg_kgIVx2	NAT6mg_kgIVx2	NNC2mg_kgSC	PBO	PF10mgSC	PF200mgSC	PF50mgSC	RIS1200mgIV	RIS200mgIV	RIS600mgIV	SEC10mg_kgIV
FIL100mgOR	1.09 (0.76, 1.65)	1.32 (0.02, 47.13)	1.09 (0.04, 62.77)		1.06 (0.03, 52.16)	1.20 (0.03, 71.44)	0.78 (0.29, 1.80)	0.62 (0.24, 1.56)	0.92 (0.39, 2.12)	0.40 (0.10, 1.21)	1.28 (0.78, 2.09)	0.86 (0.28, 2.53)	1.03 (0.36, 3.06)	0.66 (0.16, 2.23)	0.51 (0.17, 1.31)	1.12 (0.74, 1.63)	2.59 (0.25, 177.19)	4.30 (0.37, 266.58)	2.52 (0.21, 124.59)	**0.21 (0.07, 0.54)**	0.26 (0.01, 1.89)	**0.28 (0.10, 0.65)**	**13.26 (2.43, 239.54)**
FIL200mgOR	FIL200mgOR	1.19 (0.02, 45.12)	0.98 (0.03, 60.73)		0.97 (0.03, 47.91)	1.08 (0.03, 66.24)	0.72 (0.27, 1.63)	0.57 (0.22, 1.41)	0.85 (0.35, 1.91)	0.37 (0.09, 1.09)	1.18 (0.71, 1.81)	0.79 (0.26, 2.27)	0.95 (0.33, 2.81)	0.61 (0.15, 2.03)	0.47 (0.16, 1.19)	1.03 (0.68, 1.43)	2.36 (0.23, 165.54)	3.92 (0.34, 233.29)	2.30 (0.19, 111.98)	**0.19 (0.06, 0.48)**	0.24 (0.01, 1.75)	**0.26 (0.10, 0.59)**	**12.19 (2.20, 219.82)**
FON1mg_kg0_1mgIV_SC	0.84 (0.02, 48.03)	FON1mg_kg0_1mgIV_SC	0.95 (0.02, 46.81)		0.98 (0.02, 36.96)	1.01 (0.03, 45.49)	0.62 (0.01, 34.22)	0.49 (0.01, 30.94)	0.72 (0.02, 42.19)	0.32 (0.01, 21.03)	0.98 (0.03, 56.36)	0.69 (0.01, 43.82)	0.83 (0.02, 51.06)	0.52 (0.01, 35.32)	0.42 (0.01, 26.05)	0.85 (0.02, 48.24)	2.51 (0.03, 1235.96)	4.04 (0.04, 1508.67)	2.23 (0.02, 1016.58)	0.17 (0.00, 9.98)	0.21 (0.00, 18.22)	0.22 (0.01, 15.75)	12.93 (0.22, 1365.26)
FON1mg_kg1mgIV_SC	1.02 (0.02, 29.71)	1.05 (0.02, 42.10)	FON1mg_kg1mgIV_SC		0.94 (0.03, 37.38)	1.11 (0.03, 33.35)	0.74 (0.01, 21.08)	0.56 (0.01, 19.55)	0.84 (0.01, 27.31)	0.36 (0.00, 13.36)	1.19 (0.02, 34.69)	0.74 (0.01, 31.98)	0.92 (0.01, 35.59)	0.59 (0.01, 25.75)	0.46 (0.01, 16.47)	1.03 (0.02, 29.23)	2.88 (0.03, 721.88)	4.91 (0.05, 687.62)	2.52 (0.03, 516.30)	0.19 (0.00, 6.08)	0.21 (0.00, 12.24)	0.26 (0.00, 8.10)	13.74 (0.18, 1064.90)
FON4mg_kg0_1mgIV_SC	1.04 (0.02, 32.11)	1.02 (0.03, 42.09)	1.07 (0.03, 36.00)		FON4mg_kg0_1mgIV_SC	1.16 (0.03, 29.84)	0.69 (0.01, 24.84)	0.58 (0.01, 18.86)	0.83 (0.02, 28.76)	0.35 (0.01, 13.92)	1.20 (0.02, 36.79)	0.81 (0.01, 26.30)	1.00 (0.02, 33.06)	0.59 (0.01, 22.81)	0.47 (0.01, 17.85)	1.04 (0.02, 32.13)	2.52 (0.03, 572.30)	4.25 (0.05, 1381.66)	2.43 (0.02, 687.86)	0.19 (0.00, 6.73)	0.21 (0.00, 12.42)	0.27 (0.01, 8.66)	13.42 (0.21, 1207.63)
FON4mg_kg1mgIV_SC	0.92 (0.02, 39.53)	0.99 (0.02, 30.86)	0.90 (0.03, 29.29)		0.86 (0.03, 30.80)	FON4mg_kg1mgIV_SC	0.67 (0.01, 25.04)	0.54 (0.01, 23.78)	0.76 (0.01, 35.40)	0.33 (0.00, 15.47)	1.08 (0.02, 44.45)	0.72 (0.01, 33.42)	0.88 (0.01, 39.38)	0.54 (0.01, 29.85)	0.42 (0.01, 19.87)	0.94 (0.02, 38.93)	2.44 (0.02, 701.56)	4.35 (0.03, 1011.67)	2.25 (0.02, 539.12)	0.17 (0.00, 7.77)	0.19 (0.00, 12.13)	0.24 (0.00, 10.26)	12.53 (0.17, 921.15)
GUS1200mgIV	1.39 (0.61, 3.74)	1.62 (0.03, 72.57)	1.36 (0.05, 85.70)		1.44 (0.04, 71.64)	1.49 (0.04, 106.42)	GUS1200mgIV	0.80 (0.32, 2.05)	1.18 (0.53, 2.85)	0.51 (0.11, 2.00)	1.63 (0.73, 4.26)	1.10 (0.29, 4.38)	1.34 (0.40, 5.18)	0.84 (0.19, 3.75)	0.67 (0.18, 2.26)	1.42 (0.67, 3.45)	3.39 (0.29, 258.50)	5.63 (0.46, 431.31)	3.15 (0.23, 178.99)	**0.27 (0.07, 0.88)**	0.34 (0.01, 2.67)	0.36 (0.11, 1.16)	**17.96 (2.79, 301.28)**
GUS200mgIV	1.77 (0.71, 4.51)	2.06 (0.03, 92.49)	1.79 (0.05, 103.20)		1.73 (0.05, 87.18)	1.84 (0.04, 128.51)	1.25 (0.49, 3.09)	GUS200mgIV	1.49 (0.61, 3.57)	0.64 (0.13, 2.54)	2.08 (0.84, 5.11)	1.40 (0.35, 5.14)	1.65 (0.46, 6.18)	1.07 (0.22, 4.53)	0.82 (0.22, 2.91)	1.81 (0.77, 4.26)	4.22 (0.36, 272.96)	7.23 (0.54, 440.02)	3.98 (0.30, 202.89)	0.33 (0.09, 1.15)	0.41 (0.01, 3.46)	0.45 (0.14, 1.50)	**22.55 (3.34, 421.81)**
GUS600mgIV	1.18 (0.52, 2.85)	1.39 (0.02, 56.37)	1.19 (0.04, 69.48)		1.21 (0.03, 59.41)	1.32 (0.03, 82.99)	0.85 (0.35, 1.88)	0.67 (0.28, 1.64)	GUS600mgIV	0.43 (0.09, 1.66)	1.41 (0.62, 3.11)	0.93 (0.26, 3.34)	1.12 (0.33, 4.09)	0.71 (0.16, 2.87)	0.57 (0.15, 1.78)	1.22 (0.57, 2.61)	2.89 (0.25, 207.34)	4.87 (0.40, 309.67)	2.66 (0.22, 138.27)	**0.23 (0.06, 0.74)**	0.28 (0.01, 2.50)	**0.31 (0.10, 0.91)**	**14.70 (2.36, 263.26)**
MED700IV	2.71 (0.91, 11.12)	3.08 (0.05, 163.26)	2.75 (0.07, 203.35)		2.82 (0.07, 161.15)	3.07 (0.06, 237.12)	1.95 (0.50, 9.25)	1.56 (0.39, 7.75)	2.30 (0.60, 10.71)	MED700IV	**3.20 (1.05, 12.71)**	2.18 (0.44, 11.34)	2.64 (0.60, 13.10)	1.64 (0.32, 9.16)	1.30 (0.30, 6.19)	2.77 (0.95, 10.68)	7.08 (0.49, 531.08)	11.64 (0.86, 824.96)	6.66 (0.43, 429.98)	0.53 (0.11, 2.53)	0.65 (0.02, 7.79)	0.72 (0.18, 3.43)	**35.91 (4.24, 678.06)**
NAT300mgIV	0.85 (0.55, 1.41)	1.02 (0.02, 37.14)	0.84 (0.03, 50.98)		0.84 (0.03, 40.71)	0.93 (0.02, 57.22)	0.61 (0.23, 1.37)	0.48 (0.20, 1.19)	0.71 (0.32, 1.61)	**0.31 (0.08, 0.95)**	NAT300mgIV	0.67 (0.22, 1.93)	0.80 (0.29, 2.35)	0.51 (0.13, 1.70)	0.40 (0.14, 1.01)	0.87 (0.64, 1.15)	2.04 (0.20, 134.81)	3.35 (0.29, 198.83)	1.96 (0.16, 94.82)	**0.16 (0.05, 0.41)**	0.21 (0.01, 1.44)	**0.22 (0.08, 0.49)**	**10.39 (1.93, 179.52)**
NAT3mg_kgIV	1.27 (0.44, 3.92)	1.46 (0.02, 69.95)	1.35 (0.03, 83.12)		1.23 (0.04, 71.04)	1.39 (0.03, 99.19)	0.91 (0.23, 3.42)	0.71 (0.19, 2.83)	1.07 (0.30, 3.81)	0.46 (0.09, 2.25)	1.49 (0.52, 4.55)	NAT3mg_kgIV	1.21 (0.42, 3.62)	0.77 (0.19, 2.61)	0.59 (0.15, 2.37)	1.28 (0.47, 3.83)	2.99 (0.23, 230.53)	5.15 (0.33, 343.56)	2.86 (0.22, 189.27)	0.24 (0.06, 1.00)	0.29 (0.01, 2.82)	0.33 (0.08, 1.24)	**17.15 (2.11, 306.93)**
NAT3mg_kgIVx2	1.06 (0.36, 3.00)	1.21 (0.02, 49.34)	1.09 (0.03, 68.21)		1.00 (0.03, 56.04)	1.14 (0.03, 77.99)	0.75 (0.19, 2.52)	0.60 (0.16, 2.18)	0.89 (0.24, 3.03)	0.38 (0.08, 1.67)	1.25 (0.43, 3.44)	0.83 (0.28, 2.38)	NAT3mg_kgIVx2	0.62 (0.17, 1.97)	0.49 (0.12, 1.88)	1.08 (0.38, 2.85)	2.52 (0.20, 164.22)	4.30 (0.29, 259.92)	2.34 (0.18, 130.29)	**0.20 (0.05, 0.74)**	0.24 (0.01, 2.43)	**0.28 (0.07, 0.96)**	**13.89 (1.73, 249.76)**
NAT6mg_kgIVx2	1.65 (0.49, 6.66)	1.94 (0.03, 84.00)	1.68 (0.04, 111.61)		1.69 (0.04, 87.75)	1.85 (0.03, 155.05)	1.19 (0.27, 5.38)	0.94 (0.22, 4.57)	1.41 (0.35, 6.27)	0.61 (0.11, 3.16)	1.96 (0.59, 7.45)	1.30 (0.38, 5.15)	1.61 (0.51, 5.99)	NAT6mg_kgIVx2	0.79 (0.16, 3.81)	1.69 (0.53, 6.35)	4.37 (0.28, 321.68)	7.36 (0.42, 417.91)	3.98 (0.27, 235.51)	0.32 (0.06, 1.64)	0.39 (0.01, 4.26)	0.43 (0.10, 2.16)	**22.28 (2.55, 566.57)**
NNC2mg_kgSC	2.11 (0.84, 6.30)	2.40 (0.04, 115.76)	2.16 (0.06, 145.85)		2.11 (0.06, 116.83)	2.38 (0.05, 151.35)	1.50 (0.44, 5.45)	1.23 (0.34, 4.58)	1.75 (0.56, 6.48)	0.77 (0.16, 3.33)	2.47 (0.99, 7.24)	1.68 (0.42, 6.86)	2.05 (0.53, 8.40)	1.27 (0.26, 6.21)	NNC2mg_kgSC	2.16 (0.90, 6.06)	5.19 (0.44, 340.37)	8.71 (0.64, 552.43)	4.97 (0.33, 266.00)	0.41 (0.10, 1.51)	0.50 (0.02, 4.51)	0.56 (0.14, 1.97)	**27.51 (4.01, 518.65)**
PBO	0.97 (0.70, 1.46)	1.18 (0.02, 41.58)	0.97 (0.03, 58.37)		0.96 (0.03, 45.93)	1.06 (0.03, 64.33)	0.70 (0.29, 1.49)	0.55 (0.23, 1.30)	0.82 (0.38, 1.74)	0.36 (0.09, 1.05)	1.14 (0.87, 1.56)	0.78 (0.26, 2.14)	0.92 (0.35, 2.61)	0.59 (0.16, 1.88)	0.46 (0.17, 1.11)	PBO	2.31 (0.23, 155.80)	3.85 (0.34, 231.87)	2.25 (0.20, 105.92)	**0.19 (0.06, 0.44)**	0.24 (0.01, 1.64)	**0.25 (0.11, 0.53)**	**11.79 (2.25, 201.65)**
PF10mgSC	0.42 (0.01, 4.41)	0.40 (0.00, 34.49)	0.35 (0.00, 39.36)		0.40 (0.00, 35.22)	0.41 (0.00, 47.51)	0.30 (0.00, 3.51)	0.24 (0.00, 2.75)	0.35 (0.00, 4.07)	0.14 (0.00, 2.04)	0.49 (0.01, 5.11)	0.33 (0.00, 4.32)	0.40 (0.01, 4.95)	0.23 (0.00, 3.56)	0.19 (0.00, 2.28)	0.43 (0.01, 4.32)	PF10mgSC	1.71 (0.18, 13.46)	0.94 (0.10, 7.07)	**0.08 (0.00, 0.99)**	0.08 (0.00, 2.62)	0.11 (0.00, 1.26)	5.39 (0.05, 180.69)
PF200mgSC	0.25 (0.00, 2.90)	0.25 (0.00, 22.85)	0.20 (0.00, 22.03)		0.24 (0.00, 21.29)	0.23 (0.00, 30.65)	0.18 (0.00, 2.18)	0.14 (0.00, 1.84)	0.21 (0.00, 2.50)	0.09 (0.00, 1.16)	0.30 (0.01, 3.44)	0.19 (0.00, 3.03)	0.23 (0.00, 3.45)	0.14 (0.00, 2.35)	0.11 (0.00, 1.55)	0.26 (0.00, 2.93)	0.58 (0.07, 5.49)	PF200mgSC	0.53 (0.06, 4.81)	**0.04 (0.00, 0.64)**	0.05 (0.00, 1.25)	**0.06 (0.00, 0.80)**	3.07 (0.04, 110.04)
PF50mgSC	0.44 (0.01, 5.18)	0.45 (0.00, 40.81)	0.40 (0.00, 37.38)		0.41 (0.00, 46.62)	0.44 (0.00, 43.59)	0.32 (0.01, 4.41)	0.25 (0.00, 3.32)	0.38 (0.01, 4.53)	0.15 (0.00, 2.34)	0.51 (0.01, 6.12)	0.35 (0.01, 4.56)	0.43 (0.01, 5.67)	0.25 (0.00, 3.73)	0.20 (0.00, 3.02)	0.44 (0.01, 5.13)	1.07 (0.14, 10.10)	1.90 (0.21, 16.24)	PF50mgSC	0.08 (0.00, 1.17)	0.09 (0.00, 2.57)	0.11 (0.00, 1.43)	5.58 (0.07, 216.99)
RIS1200mgIV	**5.21 (2.08, 16.57)**	6.00 (0.10, 319.62)	5.31 (0.16, 375.46)		5.18 (0.15, 283.30)	5.82 (0.13, 410.77)	**3.69 (1.14, 14.06)**	3.03 (0.87, 11.48)	**4.39 (1.34, 16.40)**	1.90 (0.39, 8.94)	**6.10 (2.43, 18.92)**	4.20 (1.00, 18.18)	**5.06 (1.35, 21.48)**	3.13 (0.61, 16.41)	2.44 (0.66, 10.22)	**5.29 (2.28, 15.89)**	**12.81 (1.01, 819.19)**	**22.49 (1.56, 1249.03)**	12.63 (0.86, 640.68)	RIS1200mgIV	1.25 (0.04, 11.25)	1.38 (0.47, 4.40)	**67.40 (9.65, 1432.49)**
RIS200mgIV	4.22 (0.57, 130.95)	4.69 (0.05, 699.98)	4.76 (0.08, 618.51)		4.73 (0.08, 634.45)	5.33 (0.08, 542.84)	2.97 (0.37, 99.60)	2.47 (0.29, 71.05)	3.59 (0.40, 110.74)	1.53 (0.13, 53.35)	4.86 (0.69, 147.69)	3.43 (0.36, 118.78)	4.10 (0.41, 142.50)	2.54 (0.23, 82.78)	1.99 (0.22, 64.15)	4.23 (0.61, 130.39)	11.82 (0.38, 3871.65)	19.36 (0.80, 4582.30)	11.17 (0.39, 1965.36)	0.80 (0.09, 22.27)	RIS200mgIV	1.09 (0.13, 32.03)	**57.30 (3.65, 5969.84)**
RIS600mgIV	**3.87 (1.71, 10.34)**	4.49 (0.06, 188.99)	3.87 (0.12, 245.99)		3.69 (0.12, 195.51)	4.18 (0.10, 285.50)	2.77 (0.86, 8.94)	2.24 (0.67, 7.26)	**3.26 (1.10, 9.98)**	1.39 (0.29, 5.50)	**4.50 (2.05, 12.02)**	3.03 (0.81, 11.80)	**3.61 (1.04, 14.39)**	2.33 (0.46, 10.18)	1.79 (0.51, 7.00)	**3.93 (1.88, 9.51)**	9.50 (0.79, 655.73)	**15.79 (1.25, 957.20)**	9.08 (0.70, 514.11)	0.73 (0.23, 2.11)	0.92 (0.03, 7.54)	RIS600mgIV	**48.84 (7.67, 936.50)**
SEC10mg_kgIV	**0.08 (0.00, 0.45)**	0.08 (0.00, 4.63)	0.07 (0.00, 5.67)		0.07 (0.00, 4.69)	0.08 (0.00, 5.92)	**0.06 (0.00, 0.36)**	**0.04 (0.00, 0.30)**	**0.07 (0.00, 0.42)**	**0.03 (0.00, 0.24)**	**0.10 (0.01, 0.52)**	**0.06 (0.00, 0.47)**	**0.07 (0.00, 0.58)**	**0.04 (0.00, 0.39)**	**0.04 (0.00, 0.25)**	**0.08 (0.00, 0.45)**	0.19 (0.01, 18.74)	0.33 (0.01, 26.62)	0.18 (0.00, 13.33)	**0.01 (0.00, 0.10)**	**0.02 (0.00, 0.27)**	**0.02 (0.00, 0.13)**	SEC10mg_kgIV

	FIL200mgOR	FON1mg_kg0_1mgIV_SC	FON1mg_kg1mgIV_SC	FON4mg_kg0_1mgIV_SC	FON4mg_kg1mgIV_SC	GUS1200mgIV	GUS200mgIV	GUS600mgIV	MED700IV	NAT300mgIV	NAT3mg_kgIV	NAT3mg_kgIVx2	NAT6mg_kgIVx2	NNC2mg_kgSC	PBO	PF10mgSC	PF200mgSC	PF50mgSC	RIS1200mgIV	RIS200mgIV	RIS600mgIV	SEC10mg_kgIV
SEM60mgIV	0.88 (0.39, 2.13)	1.04 (0.02, 41.08)	0.88 (0.03, 52.78)	0.88 (0.03, 43.39)	1.01 (0.02, 59.63)	0.63 (0.20, 1.86)	0.51 (0.16, 1.58)	0.74 (0.26, 2.14)	0.31 (0.07, 1.24)	1.03 (0.47, 2.43)	0.68 (0.19, 2.55)	0.83 (0.25, 3.15)	0.52 (0.12, 2.19)	0.42 (0.12, 1.37)	0.90 (0.43, 1.99)	2.12 (0.18, 144.20)	3.58 (0.29, 221.30)	1.96 (0.17, 108.53)	**0.17 (0.05, 0.55)**	0.21 (0.01, 1.67)	**0.23 (0.07, 0.66)**	**11.16 (1.68, 191.40)**
SEM60mgIVx3	1.49 (0.61, 4.08)	1.76 (0.03, 69.15)	1.45 (0.05, 86.48)	1.53 (0.04, 78.29)	1.70 (0.04, 102.95)	1.07 (0.31, 3.44)	0.85 (0.26, 2.95)	1.27 (0.40, 3.91)	0.54 (0.11, 2.36)	1.76 (0.73, 4.63)	1.18 (0.30, 4.76)	1.43 (0.40, 5.63)	0.91 (0.19, 4.00)	0.71 (0.19, 2.50)	1.54 (0.66, 3.81)	3.80 (0.30, 277.17)	6.26 (0.46, 384.50)	3.38 (0.27, 176.49)	0.28 (0.07, 1.04)	0.35 (0.01, 3.05)	0.39 (0.11, 1.29)	**19.31 (2.77, 372.88)**
TES400mg200mgSC	**0.35 (0.14, 0.81)**	0.41 (0.01, 17.10)	0.35 (0.01, 21.01)	0.35 (0.01, 19.05)	0.38 (0.01, 25.41)	**0.25 (0.07, 0.75)**	**0.20 (0.06, 0.62)**	**0.29 (0.09, 0.85)**	**0.13 (0.03, 0.48)**	**0.42 (0.17, 0.92)**	0.28 (0.07, 1.01)	0.33 (0.10, 1.21)	**0.21 (0.04, 0.84)**	**0.17 (0.04, 0.53)**	**0.36 (0.15, 0.76)**	0.84 (0.07, 60.82)	1.39 (0.11, 91.38)	0.80 (0.06, 39.90)	**0.07 (0.02, 0.21)**	**0.08 (0.00, 0.73)**	**0.09 (0.03, 0.27)**	4.51 (0.65, 80.81)
TOF15mgOR_BID	1.65 (0.55, 5.64)	1.94 (0.03, 89.77)	1.58 (0.05, 112.65)	1.63 (0.04, 91.72)	1.74 (0.04, 121.51)	1.18 (0.27, 4.84)	0.94 (0.24, 4.05)	1.36 (0.37, 5.78)	0.59 (0.12, 2.97)	1.93 (0.65, 6.40)	1.28 (0.27, 6.42)	1.54 (0.38, 7.38)	1.01 (0.17, 5.14)	0.78 (0.17, 3.30)	1.69 (0.59, 5.40)	4.04 (0.32, 299.52)	6.93 (0.50, 406.98)	3.88 (0.26, 211.63)	0.31 (0.07, 1.32)	0.39 (0.01, 3.82)	0.41 (0.11, 1.73)	**21.08 (2.71, 432.60)**
TOF1mgOR_BID	1.73 (0.56, 5.99)	2.10 (0.03, 97.05)	1.68 (0.05, 106.74)	1.67 (0.05, 92.61)	1.82 (0.04, 122.51)	1.25 (0.30, 4.91)	0.98 (0.24, 4.24)	1.44 (0.40, 6.02)	0.62 (0.12, 3.18)	2.01 (0.66, 6.91)	1.38 (0.27, 6.63)	1.62 (0.40, 8.15)	1.04 (0.20, 5.34)	0.81 (0.18, 3.61)	1.75 (0.60, 5.83)	4.09 (0.32, 303.09)	7.14 (0.49, 442.67)	3.94 (0.28, 261.21)	0.33 (0.07, 1.42)	0.39 (0.01, 3.89)	0.44 (0.11, 1.83)	**21.55 (2.77, 502.13)**
TOF5mgOR_BID	0.97 (0.37, 2.73)	1.15 (0.02, 46.15)	1.00 (0.03, 62.21)	0.96 (0.03, 52.98)	1.03 (0.02, 71.73)	0.70 (0.19, 2.48)	0.56 (0.15, 2.02)	0.81 (0.25, 2.89)	0.35 (0.07, 1.46)	1.14 (0.45, 3.11)	0.77 (0.18, 3.20)	0.92 (0.24, 3.76)	0.59 (0.12, 2.72)	0.47 (0.12, 1.71)	1.00 (0.40, 2.60)	2.42 (0.19, 171.80)	4.06 (0.30, 245.23)	2.26 (0.16, 143.79)	**0.18 (0.05, 0.72)**	0.23 (0.01, 2.07)	**0.25 (0.07, 0.85)**	**12.31 (1.73, 278.46)**
UPA12mgOR_BID	0.71 (0.31, 1.52)	0.80 (0.01, 34.45)	0.69 (0.02, 45.88)	0.70 (0.02, 36.46)	0.78 (0.02, 50.85)	0.50 (0.17, 1.40)	0.40 (0.13, 1.16)	0.58 (0.21, 1.63)	**0.25 (0.06, 0.94)**	0.83 (0.37, 1.73)	0.55 (0.16, 1.89)	0.67 (0.19, 2.26)	0.42 (0.10, 1.63)	0.33 (0.09, 1.01)	0.72 (0.34, 1.43)	1.68 (0.15, 119.01)	2.79 (0.24, 173.01)	1.57 (0.13, 83.80)	**0.13 (0.04, 0.40)**	0.17 (0.01, 1.33)	**0.18 (0.06, 0.50)**	**8.62 (1.41, 159.63)**
UPA24mgOR	0.91 (0.39, 2.14)	1.02 (0.02, 44.41)	0.90 (0.03, 61.63)	0.92 (0.02, 46.29)	1.02 (0.02, 71.65)	0.65 (0.21, 1.90)	0.52 (0.16, 1.61)	0.76 (0.27, 2.25)	0.33 (0.07, 1.23)	1.07 (0.47, 2.42)	0.72 (0.20, 2.65)	0.87 (0.23, 3.16)	0.53 (0.12, 2.29)	0.43 (0.12, 1.41)	0.94 (0.43, 2.01)	2.22 (0.20, 154.04)	3.66 (0.31, 229.16)	2.06 (0.17, 105.29)	**0.17 (0.05, 0.56)**	0.21 (0.01, 1.90)	**0.24 (0.07, 0.68)**	**11.38 (1.80, 202.39)**
UPA24mgOR_BID	0.56 (0.26, 1.18)	0.65 (0.01, 26.07)	0.56 (0.02, 34.58)	0.54 (0.02, 29.93)	0.60 (0.01, 39.48)	0.40 (0.13, 1.10)	**0.32 (0.10, 0.91)**	0.46 (0.17, 1.24)	**0.20 (0.05, 0.72)**	0.65 (0.30, 1.33)	0.44 (0.13, 1.49)	0.53 (0.16, 1.80)	0.33 (0.07, 1.27)	**0.27 (0.07, 0.78)**	0.57 (0.28, 1.09)	1.34 (0.13, 95.70)	2.19 (0.20, 135.47)	1.26 (0.10, 66.76)	**0.11 (0.03, 0.32)**	0.13 (0.00, 1.03)	**0.15 (0.05, 0.38)**	**6.86 (1.16, 128.47)**
UPA3mgOR_BID	0.76 (0.34, 1.67)	0.88 (0.02, 36.51)	0.75 (0.02, 48.86)	0.75 (0.02, 38.62)	0.85 (0.02, 55.14)	0.54 (0.18, 1.51)	0.44 (0.14, 1.27)	0.63 (0.23, 1.77)	**0.28 (0.06, 0.98)**	0.89 (0.40, 1.90)	0.59 (0.17, 2.05)	0.72 (0.20, 2.49)	0.44 (0.10, 1.81)	0.36 (0.10, 1.11)	0.78 (0.37, 1.57)	1.81 (0.16, 125.44)	3.07 (0.25, 179.60)	1.73 (0.14, 89.05)	**0.14 (0.04, 0.45)**	0.18 (0.01, 1.55)	**0.20 (0.06, 0.55)**	**9.36 (1.53, 165.24)**
UPA45mgOR	0.72 (0.24, 2.05)	0.81 (0.01, 35.54)	0.74 (0.02, 45.17)	0.73 (0.02, 36.65)	0.81 (0.02, 49.24)	0.51 (0.13, 1.82)	0.41 (0.11, 1.56)	0.61 (0.16, 2.09)	0.26 (0.05, 1.11)	0.86 (0.28, 2.33)	0.57 (0.12, 2.32)	0.69 (0.16, 2.71)	0.43 (0.08, 1.94)	0.34 (0.08, 1.29)	0.74 (0.25, 1.92)	1.74 (0.12, 115.40)	2.96 (0.19, 194.95)	1.72 (0.12, 105.29)	**0.14 (0.03, 0.51)**	0.16 (0.01, 1.63)	**0.19 (0.05, 0.64)**	**9.28 (1.20, 177.84)**
UPA6mgOR_BID	0.61 (0.28, 1.28)	0.72 (0.01, 29.33)	0.60 (0.02, 40.60)	0.59 (0.02, 30.49)	0.67 (0.02, 46.54)	0.43 (0.14, 1.18)	**0.35 (0.11, 0.97)**	0.51 (0.19, 1.39)	**0.22 (0.05, 0.77)**	0.71 (0.33, 1.43)	0.48 (0.14, 1.65)	0.58 (0.17, 1.99)	0.36 (0.08, 1.39)	**0.29 (0.08, 0.87)**	0.63 (0.31, 1.18)	1.46 (0.13, 101.12)	2.39 (0.21, 149.75)	1.39 (0.12, 78.15)	**0.11 (0.03, 0.34)**	0.14 (0.00, 1.20)	**0.16 (0.05, 0.42)**	**7.46 (1.24, 129.66)**
UST130mgIV	1.19 (0.76, 2.08)	1.45 (0.03, 53.27)	1.20 (0.04, 72.83)	1.18 (0.04, 58.75)	1.30 (0.03, 84.66)	0.86 (0.33, 1.98)	0.68 (0.27, 1.73)	1.01 (0.45, 2.31)	0.44 (0.11, 1.38)	1.40 (0.93, 2.20)	0.95 (0.31, 2.82)	1.13 (0.40, 3.43)	0.73 (0.18, 2.47)	0.57 (0.19, 1.46)	1.22 (0.89, 1.74)	2.86 (0.28, 191.08)	4.71 (0.42, 284.07)	2.74 (0.24, 135.01)	**0.23 (0.07, 0.58)**	0.29 (0.01, 2.13)	**0.31 (0.12, 0.73)**	**14.67 (2.71, 254.46)**
UST1mg_kgIV	0.89 (0.50, 1.70)	1.03 (0.02, 41.15)	0.88 (0.03, 54.56)	0.88 (0.03, 43.22)	0.97 (0.02, 63.95)	0.64 (0.24, 1.64)	0.51 (0.18, 1.38)	0.75 (0.30, 1.91)	0.33 (0.08, 1.07)	1.04 (0.60, 1.84)	0.71 (0.21, 2.24)	0.85 (0.29, 2.73)	0.54 (0.13, 1.97)	0.42 (0.14, 1.17)	0.91 (0.56, 1.51)	2.13 (0.20, 142.35)	3.56 (0.31, 213.11)	2.07 (0.17, 103.50)	**0.17 (0.05, 0.47)**	0.22 (0.01, 1.63)	**0.23 (0.09, 0.57)**	**11.14 (1.91, 188.75)**
UST3mg_kgIV	1.01 (0.56, 2.01)	1.19 (0.02, 44.24)	1.01 (0.04, 63.40)	0.99 (0.03, 50.89)	1.12 (0.03, 71.18)	0.72 (0.26, 1.84)	0.57 (0.21, 1.58)	0.85 (0.35, 2.18)	0.37 (0.09, 1.23)	1.19 (0.67, 2.20)	0.80 (0.24, 2.53)	0.96 (0.31, 3.10)	0.61 (0.15, 2.19)	0.48 (0.15, 1.34)	1.03 (0.64, 1.78)	2.40 (0.22, 164.93)	4.05 (0.36, 241.20)	2.30 (0.20, 115.73)	**0.20 (0.06, 0.53)**	0.24 (0.01, 1.98)	**0.27 (0.10, 0.66)**	**12.43 (2.17, 219.02)**
UST4_5mg_kgIV	1.76 (0.52, 7.07)	2.03 (0.03, 87.32)	1.70 (0.06, 112.44)	1.69 (0.04, 86.20)	1.89 (0.04, 123.43)	1.23 (0.28, 5.56)	0.99 (0.24, 4.87)	1.47 (0.36, 6.39)	0.64 (0.10, 3.41)	2.05 (0.61, 7.96)	1.37 (0.28, 7.19)	1.64 (0.36, 8.67)	1.06 (0.19, 5.75)	0.82 (0.17, 4.03)	1.78 (0.56, 6.81)	4.29 (0.28, 408.76)	7.28 (0.46, 516.69)	4.10 (0.27, 273.96)	0.33 (0.07, 1.68)	0.41 (0.01, 4.46)	0.45 (0.11, 2.28)	**22.37 (2.63, 548.36)**
UST6mg_kg90mgIV_SC	1.68 (0.70, 4.46)	1.93 (0.03, 87.96)	1.68 (0.05, 101.07)	1.69 (0.05, 82.07)	1.85 (0.04, 128.98)	1.20 (0.46, 3.17)	0.96 (0.38, 2.50)	1.39 (0.61, 3.56)	0.61 (0.13, 2.44)	1.97 (0.83, 4.99)	1.35 (0.34, 5.12)	1.62 (0.46, 6.13)	0.99 (0.21, 4.08)	0.80 (0.21, 2.73)	1.73 (0.77, 4.09)	4.22 (0.35, 274.97)	6.98 (0.53, 405.40)	3.83 (0.30, 199.42)	0.32 (0.09, 1.10)	0.40 (0.01, 3.66)	0.44 (0.14, 1.49)	**21.18 (3.22, 402.00)**
UST6mg_kgIV	0.98 (0.65, 1.63)	1.13 (0.02, 44.42)	0.99 (0.03, 60.95)	0.97 (0.03, 47.17)	1.06 (0.03, 66.61)	0.71 (0.28, 1.61)	0.56 (0.23, 1.39)	0.82 (0.37, 1.85)	0.36 (0.09, 1.09)	1.15 (0.78, 1.75)	0.78 (0.26, 2.28)	0.93 (0.32, 2.75)	0.59 (0.15, 1.99)	0.46 (0.16, 1.17)	1.00 (0.75, 1.34)	2.35 (0.23, 161.10)	3.87 (0.34, 233.64)	2.27 (0.19, 111.93)	**0.19 (0.06, 0.47)**	0.24 (0.01, 1.70)	**0.25 (0.10, 0.58)**	**11.91 (2.22, 204.61)**
UST90mgSC	1.18 (0.31, 4.88)	1.32 (0.02, 65.69)	1.15 (0.03, 68.09)	1.18 (0.02, 65.57)	1.31 (0.03, 86.84)	0.86 (0.17, 3.77)	0.66 (0.15, 3.18)	0.99 (0.23, 4.39)	0.42 (0.07, 2.30)	1.39 (0.36, 5.57)	0.93 (0.17, 5.14)	1.12 (0.20, 6.00)	0.70 (0.11, 4.25)	0.55 (0.10, 2.75)	1.20 (0.33, 4.70)	3.01 (0.18, 203.03)	5.06 (0.28, 323.04)	2.86 (0.15, 166.30)	0.22 (0.04, 1.09)	0.27 (0.01, 3.01)	0.30 (0.06, 1.58)	**15.12 (1.75, 325.75)**
VED0_5mg_kgIV	0.79 (0.03, 24.14)	0.84 (0.00, 163.75)	0.79 (0.00, 164.34)	0.87 (0.01, 104.92)	0.94 (0.01, 196.02)	0.56 (0.02, 17.24)	0.45 (0.01, 13.22)	0.67 (0.02, 20.61)	0.27 (0.01, 10.09)	0.93 (0.03, 27.32)	0.64 (0.02, 20.60)	0.77 (0.02, 27.50)	0.46 (0.01, 16.72)	0.37 (0.01, 12.71)	0.81 (0.03, 23.59)	2.54 (0.04, 249.86)	4.13 (0.07, 413.35)	2.31 (0.03, 187.95)	0.15 (0.00, 4.83)	0.18 (0.00, 10.41)	0.20 (0.01, 6.35)	11.22 (0.19, 1114.72)
VED2mg_kgIV	1.07 (0.04, 29.66)	0.99 (0.01, 154.42)	1.04 (0.01, 163.08)	1.05 (0.01, 131.42)	1.22 (0.01, 172.06)	0.77 (0.02, 20.46)	0.61 (0.02, 18.41)	0.91 (0.03, 25.06)	0.37 (0.01, 12.16)	1.27 (0.04, 34.37)	0.89 (0.02, 28.39)	1.00 (0.03, 34.99)	0.62 (0.02, 23.16)	0.49 (0.01, 16.07)	1.08 (0.04, 29.93)	3.30 (0.04, 318.36)	5.56 (0.08, 624.98)	2.89 (0.03, 300.08)	0.21 (0.01, 5.53)	0.23 (0.00, 10.16)	0.28 (0.01, 8.15)	15.08 (0.30, 1058.74)
VED300mgIV	1.05 (0.55, 2.07)	1.24 (0.02, 48.68)	1.03 (0.04, 62.83)	0.99 (0.03, 52.86)	1.13 (0.03, 75.15)	0.75 (0.24, 1.91)	0.60 (0.21, 1.63)	0.89 (0.34, 2.20)	0.38 (0.09, 1.29)	1.24 (0.64, 2.29)	0.82 (0.25, 2.72)	0.98 (0.33, 3.25)	0.63 (0.15, 2.31)	0.49 (0.15, 1.40)	1.08 (0.60, 1.84)	2.51 (0.23, 175.46)	4.17 (0.35, 241.48)	2.42 (0.20, 117.27)	**0.20 (0.06, 0.56)**	0.25 (0.01, 1.96)	**0.27 (0.09, 0.70)**	**13.18 (2.30, 223.31)**

(**TABLE S16C.** continued)

	SEM60mgIV	SEM60mgIVx3	TES400mg200mgSC	TOF15mgOR_BID	TOF1mgOR_BID	TOF5mgOR_BID	UPA12mgOR_BID	UPA24mgOR	UPA24mgOR_BID	UPA3mgOR_BID	UPA45mgOR	UPA6mgOR_BID	UST130mgIV	UST1mg_kgIV	UST3mg_kgIV	UST4_5mg_kgIV	UST6mg_kg90mgIV_SC	UST6mg_kgIV	UST90mgSC	VED0_5mg_kgIV	VED2mg_kgIV	VED300mgIV
ABA10mg_kgIV	1.40 (0.56, 3.52)	0.83 (0.29, 2.22)	**3.52 (1.43, 9.49)**	0.75 (0.21, 2.39)	0.72 (0.19, 2.32)	1.27 (0.43, 3.55)	1.76 (0.76, 4.30)	1.35 (0.54, 3.45)	2.24 (0.98, 5.28)	1.63 (0.68, 4.09)	1.72 (0.56, 5.60)	2.03 (0.91, 4.86)	1.03 (0.55, 1.91)	1.39 (0.69, 2.81)	1.23 (0.59, 2.49)	0.71 (0.17, 2.51)	0.74 (0.27, 1.89)	1.27 (0.69, 2.30)	1.07 (0.25, 4.15)	1.59 (0.05, 46.31)	1.17 (0.05, 36.58)	1.18 (0.57, 2.55)
ABA30mg_kgIV	1.86 (0.67, 5.16)	1.08 (0.36, 3.26)	**4.60 (1.77, 14.18)**	1.02 (0.26, 3.47)	0.96 (0.25, 3.29)	1.70 (0.54, 5.18)	2.33 (0.93, 6.35)	1.79 (0.67, 5.16)	**2.95 (1.20, 7.75)**	2.15 (0.85, 6.08)	2.29 (0.69, 7.92)	**2.69 (1.08, 7.27)**	1.37 (0.66, 2.94)	1.83 (0.84, 4.20)	1.62 (0.70, 3.71)	0.94 (0.21, 3.75)	0.98 (0.34, 2.75)	1.66 (0.84, 3.58)	1.41 (0.32, 5.86)	2.09 (0.07, 62.53)	1.53 (0.06, 45.70)	1.55 (0.68, 3.88)
ABA3mg_kgIV	1.53 (0.59, 3.78)	0.90 (0.32, 2.62)	**3.85 (1.55, 10.29)**	0.82 (0.23, 2.70)	0.79 (0.21, 2.59)	1.38 (0.48, 3.92)	1.91 (0.84, 4.81)	1.49 (0.59, 3.79)	**2.42 (1.05, 5.76)**	1.77 (0.76, 4.53)	1.87 (0.61, 6.19)	2.22 (0.98, 5.52)	1.13 (0.59, 2.09)	1.51 (0.73, 3.07)	1.33 (0.63, 2.77)	0.78 (0.19, 2.91)	0.81 (0.29, 2.12)	1.38 (0.76, 2.51)	1.15 (0.26, 4.78)	1.71 (0.06, 52.38)	1.29 (0.05, 37.84)	1.28 (0.61, 2.82)
ADA160mg80mg40mgSC	1.60 (0.48, 5.34)	0.96 (0.27, 3.30)	**4.07 (1.22, 14.06)**	0.86 (0.19, 3.55)	0.81 (0.18, 3.38)	1.45 (0.37, 5.51)	2.01 (0.66, 6.77)	1.54 (0.49, 5.44)	2.53 (0.83, 8.43)	1.86 (0.59, 6.40)	1.97 (0.53, 8.29)	2.30 (0.77, 7.76)	1.18 (0.44, 3.25)	1.57 (0.55, 4.62)	1.39 (0.47, 4.10)	0.79 (0.17, 3.73)	0.84 (0.55, 1.28)	1.45 (0.55, 3.88)	1.20 (0.24, 6.23)	1.78 (0.05, 63.84)	1.33 (0.04, 41.60)	1.34 (0.46, 4.22)
ADA160mg80mg60mgSC	1.20 (0.37, 4.35)	0.71 (0.19, 2.80)	3.09 (0.88, 11.32)	0.66 (0.14, 2.91)	0.63 (0.13, 2.88)	1.09 (0.27, 4.38)	1.55 (0.46, 5.30)	1.18 (0.35, 3.95)	1.95 (0.59, 6.40)	1.41 (0.43, 4.86)	1.50 (0.37, 6.15)	1.76 (0.55, 5.83)	0.89 (0.31, 2.67)	1.20 (0.41, 3.72)	1.05 (0.35, 3.26)	0.62 (0.11, 2.97)	0.64 (0.17, 2.33)	1.10 (0.39, 3.06)	0.93 (0.17, 4.94)	1.41 (0.04, 43.87)	1.01 (0.03, 32.32)	1.02 (0.34, 3.39)
ADA160mg80mgSC	1.22 (0.49, 2.85)	0.72 (0.25, 1.84)	**3.06 (1.28, 7.90)**	0.65 (0.18, 2.03)	0.62 (0.18, 1.97)	1.11 (0.38, 2.97)	1.53 (0.67, 3.53)	1.18 (0.50, 2.80)	1.94 (0.89, 4.29)	1.42 (0.62, 3.26)	1.49 (0.51, 4.74)	1.77 (0.81, 3.99)	0.91 (0.50, 1.50)	1.21 (0.62, 2.26)	1.07 (0.53, 1.99)	0.62 (0.15, 2.14)	0.64 (0.24, 1.57)	1.11 (0.64, 1.79)	0.92 (0.22, 3.47)	1.36 (0.04, 40.59)	1.05 (0.04, 31.01)	1.03 (0.50, 2.07)
ADA40mg20mgSC	2.08 (0.69, 6.66)	1.23 (0.36, 3.95)	**5.21 (1.79, 16.90)**	1.13 (0.27, 4.27)	1.08 (0.26, 4.00)	1.91 (0.52, 6.23)	2.61 (0.91, 8.11)	2.01 (0.68, 6.44)	**3.27 (1.21, 10.06)**	2.43 (0.86, 7.46)	2.57 (0.72, 9.78)	**3.02 (1.08, 9.09)**	1.54 (0.65, 3.86)	2.05 (0.82, 5.61)	1.81 (0.70, 4.91)	1.05 (0.21, 4.39)	1.09 (0.34, 3.58)	1.86 (0.81, 4.65)	1.59 (0.33, 7.32)	2.30 (0.07, 66.46)	1.82 (0.06, 55.25)	1.76 (0.67, 4.90)
ADA80mg40mgSC	1.25 (0.45, 3.21)	0.74 (0.23, 2.04)	**3.10 (1.17, 8.80)**	0.67 (0.17, 2.25)	0.63 (0.17, 2.21)	1.11 (0.35, 3.41)	1.55 (0.62, 4.10)	1.20 (0.45, 3.30)	1.96 (0.81, 5.03)	1.45 (0.57, 3.80)	1.51 (0.47, 5.38)	1.80 (0.74, 4.64)	0.91 (0.44, 1.82)	1.23 (0.55, 2.65)	1.08 (0.47, 2.40)	0.62 (0.15, 2.42)	0.65 (0.21, 1.79)	1.12 (0.55, 2.16)	0.93 (0.20, 3.87)	1.37 (0.05, 43.35)	1.02 (0.04, 32.85)	1.05 (0.45, 2.38)
AND150mgSC1_1	1.80 (0.02, 57.89)	1.03 (0.01, 35.76)	4.51 (0.04, 146.70)	0.96 (0.01, 36.57)	0.88 (0.01, 30.50)	1.59 (0.02, 52.31)	2.24 (0.02, 70.09)	1.70 (0.02, 52.52)	2.77 (0.03, 82.15)	2.05 (0.02, 64.80)	2.10 (0.02, 73.93)	2.54 (0.02, 77.78)	1.31 (0.01, 38.94)	1.79 (0.02, 53.01)	1.56 (0.01, 43.87)	0.85 (0.01, 32.69)	0.92 (0.01, 33.29)	1.63 (0.01, 47.32)	1.30 (0.01, 46.91)	1.82 (0.01, 233.58)	1.43 (0.01, 177.55)	1.46 (0.01, 46.74)
AND150mgSC2_2	1.56 (0.03, 48.88)	0.92 (0.01, 30.23)	4.05 (0.07, 133.27)	0.87 (0.01, 28.57)	0.83 (0.01, 28.01)	1.46 (0.02, 43.77)	1.98 (0.03, 68.65)	1.55 (0.03, 55.97)	2.51 (0.04, 86.10)	1.83 (0.03, 64.58)	1.94 (0.04, 66.42)	2.25 (0.04, 76.77)	1.17 (0.02, 34.90)	1.58 (0.03, 46.49)	1.40 (0.02, 42.66)	0.78 (0.01, 27.98)	0.82 (0.01, 28.66)	1.44 (0.03, 42.48)	1.24 (0.02, 43.33)	1.77 (0.01, 221.60)	1.41 (0.01, 197.56)	1.38 (0.02, 40.74)
AND300mgSC	2.01 (0.04, 61.25)	1.15 (0.02, 38.15)	5.09 (0.10, 153.95)	1.08 (0.02, 36.33)	1.04 (0.02, 36.65)	1.84 (0.03, 57.88)	2.54 (0.05, 74.47)	1.97 (0.04, 56.85)	3.18 (0.06, 90.60)	2.37 (0.04, 69.31)	2.37 (0.05, 83.26)	2.95 (0.06, 82.51)	1.50 (0.03, 43.67)	1.99 (0.04, 56.33)	1.77 (0.03, 50.03)	1.00 (0.02, 34.82)	1.07 (0.02, 30.60)	1.81 (0.04, 53.04)	1.55 (0.02, 58.05)	2.15 (0.02, 293.11)	1.74 (0.01, 194.26)	1.70 (0.03, 50.11)
BRI400mgIV	1.17 (0.41, 3.16)	0.68 (0.22, 2.05)	**2.92 (1.08, 8.58)**	0.63 (0.16, 2.19)	0.60 (0.15, 2.16)	1.04 (0.32, 3.27)	1.46 (0.55, 3.87)	1.12 (0.41, 3.04)	1.86 (0.72, 4.67)	1.37 (0.53, 3.58)	1.42 (0.42, 4.90)	1.70 (0.66, 4.41)	0.86 (0.40, 1.78)	1.15 (0.50, 2.56)	1.01 (0.43, 2.34)	0.59 (0.13, 2.29)	0.61 (0.21, 1.75)	1.06 (0.50, 2.16)	0.86 (0.20, 3.72)	1.27 (0.04, 40.34)	0.97 (0.04, 29.82)	0.97 (0.39, 2.35)
BRI700mgIV	1.18 (0.45, 3.13)	0.70 (0.24, 1.96)	**2.98 (1.14, 8.33)**	0.63 (0.17, 2.12)	0.61 (0.15, 2.09)	1.06 (0.35, 3.23)	1.48 (0.60, 3.78)	1.14 (0.44, 2.93)	1.87 (0.77, 4.59)	1.38 (0.56, 3.50)	1.44 (0.46, 4.91)	1.72 (0.71, 4.28)	0.88 (0.43, 1.66)	1.17 (0.54, 2.46)	1.04 (0.47, 2.20)	0.60 (0.14, 2.22)	0.62 (0.22, 1.68)	1.07 (0.54, 2.00)	0.87 (0.21, 3.75)	1.33 (0.04, 40.02)	0.98 (0.04, 29.83)	0.99 (0.44, 2.26)
BRO210mgIV	1.24 (0.41, 3.74)	0.73 (0.22, 2.39)	3.10 (1.00, 9.98)	0.64 (0.17, 2.59)	0.62 (0.15, 2.43)	1.10 (0.32, 3.76)	1.55 (0.53, 4.78)	1.19 (0.38, 3.65)	1.96 (0.69, 5.73)	1.45 (0.48, 4.35)	1.51 (0.44, 5.59)	1.77 (0.63, 5.32)	0.90 (0.37, 2.21)	1.21 (0.47, 3.14)	1.06 (0.41, 2.78)	0.61 (0.14, 2.65)	0.64 (0.19, 2.13)	1.10 (0.46, 2.63)	0.95 (0.17, 4.25)	1.36 (0.04, 48.18)	1.03 (0.04, 31.41)	1.04 (0.38, 2.80)
BRO350mgIV	1.27 (0.41, 3.72)	0.74 (0.22, 2.39)	3.16 (0.98, 10.31)	0.67 (0.17, 2.59)	0.64 (0.16, 2.52)	1.13 (0.33, 3.98)	1.57 (0.56, 4.75)	1.21 (0.41, 3.65)	1.99 (0.73, 5.71)	1.46 (0.52, 4.36)	1.54 (0.45, 5.80)	1.81 (0.68, 5.21)	0.93 (0.39, 2.18)	1.24 (0.49, 3.16)	1.10 (0.43, 2.81)	0.63 (0.14, 2.76)	0.66 (0.20, 2.10)	1.13 (0.48, 2.64)	0.95 (0.19, 4.43)	1.44 (0.04, 47.49)	1.06 (0.04, 32.78)	1.06 (0.40, 2.82)
BRO700mgIV	0.90 (0.32, 2.51)	0.52 (0.17, 1.63)	2.24 (0.77, 7.00)	0.47 (0.12, 1.73)	0.46 (0.11, 1.69)	0.80 (0.25, 2.55)	1.13 (0.42, 3.07)	0.87 (0.31, 2.46)	1.42 (0.54, 3.76)	1.05 (0.39, 2.85)	1.09 (0.32, 3.94)	1.30 (0.49, 3.48)	0.66 (0.29, 1.41)	0.87 (0.37, 2.04)	0.78 (0.32, 1.83)	0.45 (0.10, 1.73)	0.47 (0.15, 1.36)	0.80 (0.37, 1.71)	0.68 (0.14, 2.97)	1.00 (0.03, 33.69)	0.73 (0.03, 22.63)	0.75 (0.30, 1.88)
CDP10mg_kgIV	1.10 (0.44, 2.66)	0.65 (0.23, 1.66)	**2.73 (1.14, 6.99)**	0.59 (0.17, 1.86)	0.56 (0.16, 1.84)	0.99 (0.34, 2.81)	1.37 (0.60, 3.32)	1.06 (0.43, 2.67)	1.73 (0.79, 3.99)	1.28 (0.54, 3.12)	1.34 (0.46, 4.32)	1.58 (0.72, 3.73)	0.81 (0.45, 1.40)	1.09 (0.54, 2.09)	0.96 (0.47, 1.88)	0.55 (0.13, 2.00)	0.57 (0.21, 1.45)	0.99 (0.56, 1.71)	0.83 (0.20, 3.24)	1.22 (0.04, 36.10)	0.92 (0.03, 27.09)	0.92 (0.45, 1.94)
CER400mgSC	0.46 (0.08, 2.05)	0.27 (0.04, 1.28)	1.16 (0.20, 5.56)	0.24 (0.03, 1.38)	0.23 (0.04, 1.32)	0.40 (0.07, 2.05)	0.59 (0.10, 2.50)	0.45 (0.08, 1.94)	0.74 (0.13, 3.08)	0.54 (0.10, 2.24)	0.56 (0.09, 3.05)	0.67 (0.12, 2.77)	0.34 (0.07, 1.28)	0.46 (0.09, 1.81)	0.40 (0.08, 1.60)	0.23 (0.03, 1.34)	0.24 (0.04, 1.11)	0.42 (0.08, 1.57)	0.34 (0.04, 2.38)	0.49 (0.01, 19.51)	0.40 (0.01, 15.10)	0.38 (0.07, 1.66)
ELD10mg_kgIV	1.76 (0.60, 5.45)	1.03 (0.31, 3.46)	**4.40 (1.58, 13.97)**	0.93 (0.24, 3.45)	0.89 (0.21, 3.47)	1.59 (0.47, 5.32)	2.21 (0.79, 6.42)	1.70 (0.58, 5.14)	**2.78 (1.05, 7.92)**	2.06 (0.73, 5.99)	2.17 (0.60, 8.48)	2.55 (0.92, 7.32)	1.28 (0.57, 3.04)	1.73 (0.69, 4.38)	1.52 (0.60, 3.91)	0.88 (0.19, 3.71)	0.91 (0.30, 2.88)	1.57 (0.70, 3.65)	1.34 (0.28, 5.87)	1.97 (0.06, 62.88)	1.45 (0.05, 45.68)	1.49 (0.58, 3.91)
ELD20mg_kgIV	1.44 (0.50, 4.23)	0.85 (0.27, 2.66)	**3.64 (1.28, 11.41)**	0.78 (0.19, 2.87)	0.75 (0.18, 2.72)	1.29 (0.40, 4.34)	1.79 (0.66, 5.30)	1.40 (0.48, 4.19)	2.28 (0.86, 6.33)	1.68 (0.58, 4.97)	1.77 (0.52, 6.60)	2.09 (0.76, 5.87)	1.05 (0.47, 2.43)	1.42 (0.58, 3.49)	1.26 (0.50, 3.15)	0.73 (0.16, 3.04)	0.76 (0.25, 2.32)	1.29 (0.60, 2.96)	1.08 (0.24, 4.88)	1.60 (0.05, 50.19)	1.18 (0.04, 36.93)	1.21 (0.48, 3.26)
ETR105mgSC	0.66 (0.24, 1.69)	0.38 (0.13, 1.10)	1.64 (0.61, 4.75)	0.35 (0.09, 1.19)	0.33 (0.08, 1.17)	0.59 (0.19, 1.80)	0.82 (0.33, 2.12)	0.63 (0.24, 1.65)	1.04 (0.42, 2.62)	0.75 (0.30, 1.98)	0.79 (0.25, 2.69)	0.94 (0.38, 2.34)	**0.48 (0.23, 0.94)**	0.65 (0.28, 1.40)	0.57 (0.25, 1.24)	0.32 (0.08, 1.23)	**0.34 (0.12, 0.96)**	0.59 (0.29, 1.13)	0.49 (0.11, 2.13)	0.72 (0.02, 22.81)	0.55 (0.02, 16.29)	0.55 (0.24, 1.26)
ETR210mgSC	0.63 (0.23, 1.62)	0.37 (0.12, 1.11)	1.58 (0.60, 4.54)	0.34 (0.09, 1.15)	0.32 (0.08, 1.14)	0.57 (0.18, 1.72)	0.79 (0.31, 2.04)	0.61 (0.23, 1.66)	1.00 (0.40, 2.53)	0.73 (0.29, 1.93)	0.77 (0.24, 2.65)	0.91 (0.37, 2.33)	**0.46 (0.22, 0.91)**	0.62 (0.27, 1.34)	0.55 (0.23, 1.19)	0.32 (0.07, 1.20)	**0.32 (0.11, 0.94)**	0.57 (0.28, 1.08)	0.47 (0.11, 2.01)	0.70 (0.02, 21.29)	0.53 (0.02, 15.67)	0.53 (0.23, 1.22)

	SEM60mgIV	SEM60mgIVx3	TES400mg200mgSC	TOF15mgOR_BID	TOF1mgOR_BID	TOF5mgOR_BID	UPA12mgOR_BID	UPA24mgOR	UPA24mgOR_BID	UPA3mgOR_BID	UPA45mgOR	UPA6mgOR_BID	UST130mgIV	UST1mg_kgIV	UST3mg_kgIV	UST4_5mg_kgIV	UST6mg_kg90mgIV_SC	UST6mg_kgIV	UST90mgSC	VED0_5mg_kgIV	VED2mg_kgIV	VED300mgIV
FIL100mgOR	1.24 (0.51, 2.80)	0.73 (0.27, 1.82)	**3.11 (1.34, 7.80)**	0.66 (0.19, 2.01)	0.63 (0.18, 1.93)	1.12 (0.40, 2.95)	1.54 (0.71, 3.60)	1.19 (0.51, 2.76)	1.95 (0.91, 4.30)	1.43 (0.65, 3.32)	1.51 (0.53, 4.66)	1.79 (0.86, 3.94)	0.92 (0.52, 1.47)	1.23 (0.64, 2.24)	1.09 (0.54, 1.95)	0.62 (0.16, 2.13)	0.65 (0.25, 1.58)	1.12 (0.67, 1.76)	0.93 (0.23, 3.53)	1.39 (0.05, 40.07)	1.04 (0.04, 28.09)	1.04 (0.53, 2.01)
FIL200mgOR	1.14 (0.47, 2.55)	0.67 (0.25, 1.64)	**2.83 (1.23, 6.96)**	0.61 (0.18, 1.82)	0.58 (0.17, 1.78)	1.03 (0.37, 2.69)	1.41 (0.66, 3.22)	1.10 (0.47, 2.55)	1.80 (0.85, 3.80)	1.32 (0.60, 2.96)	1.38 (0.49, 4.22)	1.64 (0.78, 3.61)	0.84 (0.48, 1.32)	1.12 (0.59, 2.00)	0.99 (0.50, 1.78)	0.57 (0.14, 1.93)	0.59 (0.22, 1.43)	1.02 (0.61, 1.53)	0.85 (0.20, 3.22)	1.26 (0.04, 35.96)	0.94 (0.03, 27.60)	0.96 (0.48, 1.82)
FON1mg_kg0_1mgIV_SC	0.96 (0.02, 57.78)	0.57 (0.01, 33.48)	2.45 (0.06, 136.44)	0.51 (0.01, 32.61)	0.48 (0.01, 30.88)	0.87 (0.02, 53.27)	1.25 (0.03, 70.89)	0.98 (0.02, 51.66)	1.54 (0.04, 83.66)	1.14 (0.03, 65.34)	1.24 (0.03, 74.24)	1.39 (0.03, 77.60)	0.69 (0.02, 39.10)	0.97 (0.02, 52.74)	0.84 (0.02, 49.69)	0.49 (0.01, 36.22)	0.52 (0.01, 28.85)	0.88 (0.02, 49.13)	0.76 (0.02, 42.52)	1.19 (0.01, 306.48)	1.01 (0.01, 118.41)	0.81 (0.02, 47.43)
FON1mg_kg1mgIV_SC	1.14 (0.02, 34.00)	0.69 (0.01, 20.34)	2.82 (0.05, 88.17)	0.63 (0.01, 19.80)	0.60 (0.01, 21.26)	1.00 (0.02, 35.58)	1.45 (0.02, 44.62)	1.11 (0.02, 34.19)	1.80 (0.03, 53.80)	1.33 (0.02, 41.66)	1.35 (0.02, 47.53)	1.66 (0.02, 49.46)	0.83 (0.01, 24.35)	1.14 (0.02, 30.31)	0.99 (0.02, 27.45)	0.59 (0.01, 17.78)	0.59 (0.01, 19.54)	1.01 (0.02, 29.06)	0.87 (0.01, 31.59)	1.26 (0.01, 252.40)	0.96 (0.01, 119.19)	0.97 (0.02, 28.27)
FON4mg_kg0_1mgIV_SC	1.14 (0.02, 37.01)	0.65 (0.01, 22.62)	2.89 (0.05, 100.43)	0.61 (0.01, 22.88)	0.60 (0.01, 21.50)	1.04 (0.02, 36.71)	1.43 (0.03, 55.76)	1.09 (0.02, 41.90)	1.84 (0.03, 63.56)	1.34 (0.03, 51.24)	1.38 (0.03, 49.03)	1.69 (0.03, 59.69)	0.85 (0.02, 27.35)	1.14 (0.02, 36.07)	1.01 (0.02, 31.07)	0.59 (0.01, 23.21)	0.59 (0.01, 21.83)	1.03 (0.02, 33.66)	0.85 (0.02, 41.73)	1.15 (0.01, 151.83)	0.95 (0.01, 106.00)	1.01 (0.02, 32.19)
FON4mg_kg1mgIV_SC	0.99 (0.02, 47.58)	0.59 (0.01, 28.55)	2.62 (0.04, 101.35)	0.57 (0.01, 25.30)	0.55 (0.01, 23.64)	0.97 (0.01, 41.67)	1.28 (0.02, 54.15)	0.98 (0.01, 45.68)	1.66 (0.03, 70.51)	1.17 (0.02, 53.76)	1.23 (0.02, 63.46)	1.49 (0.02, 64.64)	0.77 (0.01, 31.03)	1.04 (0.02, 40.78)	0.90 (0.01, 34.43)	0.53 (0.01, 23.27)	0.54 (0.01, 26.62)	0.94 (0.02, 38.16)	0.77 (0.01, 32.51)	1.07 (0.01, 198.79)	0.82 (0.01, 151.95)	0.88 (0.01, 38.53)
GUS1200mgIV	1.58 (0.54, 4.97)	0.94 (0.29, 3.18)	**3.93 (1.34, 13.70)**	0.85 (0.21, 3.64)	0.80 (0.20, 3.34)	1.43 (0.40, 5.29)	2.01 (0.71, 6.02)	1.55 (0.53, 4.84)	2.52 (0.91, 7.62)	1.86 (0.66, 5.69)	1.98 (0.55, 7.70)	2.30 (0.85, 7.00)	1.16 (0.51, 3.04)	1.56 (0.61, 4.20)	1.39 (0.54, 3.86)	0.81 (0.18, 3.53)	0.83 (0.32, 2.18)	1.41 (0.62, 3.60)	1.17 (0.26, 5.97)	1.79 (0.06, 59.45)	1.30 (0.05, 41.33)	1.33 (0.52, 4.11)
GUS200mgIV	1.96 (0.63, 6.26)	1.17 (0.34, 3.89)	**5.03 (1.62, 16.49)**	1.07 (0.25, 4.22)	1.02 (0.24, 4.16)	1.78 (0.50, 6.52)	2.50 (0.86, 7.71)	1.91 (0.62, 6.16)	**3.16 (1.10, 9.57)**	2.29 (0.79, 7.15)	2.43 (0.64, 9.03)	**2.85 (1.03, 8.96)**	1.47 (0.58, 3.69)	1.97 (0.72, 5.49)	1.74 (0.63, 4.82)	1.01 (0.21, 4.19)	1.04 (0.40, 2.65)	1.80 (0.72, 4.40)	1.51 (0.31, 6.71)	2.21 (0.08, 68.33)	1.65 (0.05, 51.72)	1.66 (0.62, 4.84)
GUS600mgIV	1.35 (0.47, 3.79)	0.79 (0.26, 2.51)	**3.40 (1.18, 10.79)**	0.73 (0.17, 2.67)	0.70 (0.17, 2.52)	1.23 (0.35, 4.01)	1.71 (0.61, 4.86)	1.31 (0.44, 3.70)	2.16 (0.80, 5.85)	1.59 (0.56, 4.35)	1.64 (0.48, 6.09)	1.97 (0.72, 5.35)	0.99 (0.43, 2.23)	1.33 (0.52, 3.29)	1.18 (0.46, 2.83)	0.68 (0.16, 2.75)	0.72 (0.28, 1.64)	1.22 (0.54, 2.67)	1.01 (0.23, 4.43)	1.50 (0.05, 46.93)	1.09 (0.04, 31.75)	1.12 (0.45, 2.95)
MED700IV	3.18 (0.80, 14.81)	1.86 (0.42, 8.92)	**7.90 (2.08, 37.11)**	1.71 (0.34, 8.43)	1.62 (0.31, 8.38)	2.87 (0.69, 13.70)	**3.95 (1.07, 17.95)**	3.07 (0.81, 13.86)	**5.01 (1.38, 22.12)**	**3.63 (1.02, 16.63)**	3.80 (0.90, 21.57)	**4.57 (1.30, 20.00)**	2.29 (0.73, 8.79)	3.05 (0.93, 12.52)	2.74 (0.81, 11.52)	1.56 (0.29, 10.04)	1.64 (0.41, 7.45)	2.77 (0.92, 10.79)	2.38 (0.44, 14.91)	3.72 (0.10, 118.62)	2.71 (0.08, 97.38)	2.65 (0.77, 11.27)
NAT300mgIV	0.97 (0.41, 2.13)	0.57 (0.22, 1.38)	**2.41 (1.08, 5.87)**	0.52 (0.16, 1.53)	0.50 (0.14, 1.51)	0.88 (0.32, 2.24)	1.21 (0.58, 2.70)	0.93 (0.41, 2.14)	1.53 (0.75, 3.30)	1.12 (0.53, 2.50)	1.17 (0.43, 3.59)	1.40 (0.70, 3.01)	0.71 (0.46, 1.07)	0.96 (0.54, 1.66)	0.84 (0.46, 1.49)	0.49 (0.13, 1.63)	0.51 (0.20, 1.20)	0.87 (0.57, 1.29)	0.72 (0.18, 2.74)	1.07 (0.04, 31.02)	0.79 (0.03, 24.37)	0.81 (0.44, 1.55)
NAT3mg_kgIV	1.46 (0.39, 5.21)	0.85 (0.21, 3.31)	3.64 (0.99, 13.63)	0.78 (0.16, 3.71)	0.73 (0.15, 3.68)	1.29 (0.31, 5.56)	1.82 (0.53, 6.27)	1.40 (0.38, 5.00)	2.27 (0.67, 7.64)	1.69 (0.49, 5.80)	1.75 (0.43, 8.04)	2.10 (0.61, 7.03)	1.05 (0.35, 3.21)	1.40 (0.45, 4.77)	1.25 (0.40, 4.09)	0.73 (0.14, 3.52)	0.74 (0.20, 2.90)	1.28 (0.44, 3.88)	1.07 (0.19, 5.99)	1.57 (0.05, 65.72)	1.13 (0.04, 49.44)	1.22 (0.37, 3.98)
NAT3mg_kgIVx2	1.21 (0.32, 4.04)	0.70 (0.18, 2.50)	3.05 (0.83, 10.31)	0.65 (0.14, 2.60)	0.62 (0.12, 2.53)	1.09 (0.27, 4.09)	1.49 (0.44, 5.29)	1.15 (0.32, 4.26)	1.89 (0.55, 6.28)	1.38 (0.40, 4.96)	1.45 (0.37, 6.16)	1.74 (0.50, 5.73)	0.88 (0.29, 2.52)	1.18 (0.37, 3.48)	1.04 (0.32, 3.18)	0.61 (0.12, 2.81)	0.62 (0.16, 2.19)	1.08 (0.36, 3.14)	0.89 (0.17, 5.07)	1.29 (0.04, 42.92)	1.00 (0.03, 31.20)	1.02 (0.31, 3.03)
NAT6mg_kgIVx2	1.91 (0.46, 8.56)	1.10 (0.25, 5.31)	**4.73 (1.19, 22.81)**	0.99 (0.19, 5.75)	0.96 (0.19, 5.10)	1.70 (0.37, 8.65)	2.41 (0.61, 10.49)	1.88 (0.44, 8.41)	3.07 (0.79, 13.41)	2.26 (0.55, 9.64)	2.30 (0.52, 11.88)	2.79 (0.72, 12.17)	1.37 (0.41, 5.45)	1.86 (0.51, 7.95)	1.64 (0.46, 6.85)	0.94 (0.17, 5.36)	1.01 (0.24, 4.74)	1.69 (0.50, 6.57)	1.43 (0.24, 9.25)	2.18 (0.06, 82.11)	1.61 (0.04, 58.34)	1.58 (0.43, 6.88)
NNC2mg_kgSC	2.40 (0.73, 8.36)	1.42 (0.40, 5.30)	**6.04 (1.87, 22.48)**	1.27 (0.30, 5.73)	1.23 (0.28, 5.63)	2.14 (0.59, 8.60)	3.01 (0.99, 10.75)	2.35 (0.71, 8.21)	**3.77 (1.27, 13.44)**	2.77 (0.90, 9.93)	2.94 (0.78, 12.75)	**3.46 (1.15, 11.93)**	1.75 (0.68, 5.19)	2.37 (0.86, 7.34)	2.08 (0.75, 6.63)	1.21 (0.25, 5.73)	1.25 (0.37, 4.67)	2.15 (0.85, 6.22)	1.83 (0.36, 9.87)	2.72 (0.08, 88.85)	2.02 (0.06, 67.17)	2.03 (0.71, 6.74)
PBO	1.11 (0.50, 2.32)	0.65 (0.26, 1.53)	**2.75 (1.32, 6.47)**	0.59 (0.19, 1.70)	0.57 (0.17, 1.65)	1.00 (0.38, 2.47)	1.38 (0.70, 2.97)	1.07 (0.50, 2.32)	1.75 (0.92, 3.59)	1.28 (0.64, 2.73)	1.35 (0.52, 3.98)	1.60 (0.85, 3.26)	0.82 (0.58, 1.12)	1.10 (0.66, 1.77)	0.97 (0.56, 1.57)	0.56 (0.15, 1.79)	0.58 (0.24, 1.31)	1.00 (0.74, 1.33)	0.83 (0.21, 2.99)	1.23 (0.04, 35.48)	0.92 (0.03, 25.84)	0.92 (0.54, 1.66)
PF10mgSC	0.47 (0.01, 5.62)	0.26 (0.00, 3.37)	1.20 (0.02, 14.61)	0.25 (0.00, 3.17)	0.24 (0.00, 3.09)	0.41 (0.01, 5.22)	0.59 (0.01, 6.71)	0.45 (0.01, 5.05)	0.75 (0.01, 7.90)	0.55 (0.01, 6.20)	0.58 (0.01, 8.03)	0.69 (0.01, 7.41)	0.35 (0.01, 3.57)	0.47 (0.01, 5.04)	0.42 (0.01, 4.57)	0.23 (0.00, 3.58)	0.24 (0.00, 2.87)	0.43 (0.01, 4.40)	0.33 (0.00, 5.58)	0.39 (0.00, 22.32)	0.30 (0.00, 22.76)	0.40 (0.01, 4.27)
PF200mgSC	0.28 (0.00, 3.41)	0.16 (0.00, 2.18)	0.72 (0.01, 8.83)	0.14 (0.00, 2.00)	0.14 (0.00, 2.05)	0.25 (0.00, 3.34)	0.36 (0.01, 4.22)	0.27 (0.00, 3.21)	0.46 (0.01, 5.07)	0.33 (0.01, 4.01)	0.34 (0.01, 5.16)	0.42 (0.01, 4.80)	0.21 (0.00, 2.37)	0.28 (0.00, 3.20)	0.25 (0.00, 2.81)	0.14 (0.00, 2.17)	0.14 (0.00, 1.90)	0.26 (0.00, 2.92)	0.20 (0.00, 3.54)	0.24 (0.00, 14.79)	0.18 (0.00, 12.28)	0.24 (0.00, 2.82)
PF50mgSC	0.51 (0.01, 6.01)	0.30 (0.01, 3.64)	1.25 (0.03, 16.93)	0.26 (0.00, 3.80)	0.25 (0.00, 3.54)	0.44 (0.01, 6.42)	0.64 (0.01, 7.64)	0.48 (0.01, 5.93)	0.79 (0.01, 10.12)	0.58 (0.01, 7.36)	0.58 (0.01, 8.31)	0.72 (0.01, 8.55)	0.36 (0.01, 4.21)	0.48 (0.01, 5.85)	0.43 (0.01, 4.91)	0.24 (0.00, 3.76)	0.26 (0.01, 3.33)	0.44 (0.01, 5.14)	0.35 (0.01, 6.78)	0.43 (0.01, 33.35)	0.35 (0.00, 28.71)	0.41 (0.01, 5.06)
RIS1200mgIV	**5.97 (1.82, 22.20)**	3.55 (0.97, 13.49)	**15.03 (4.76, 58.35)**	3.24 (0.76, 13.78)	3.04 (0.70, 13.56)	**5.50 (1.40, 20.17)**	**7.59 (2.50, 27.05)**	**5.85 (1.79, 21.95)**	**9.46 (3.16, 34.17)**	**7.07 (2.25, 25.59)**	**7.39 (1.97, 33.48)**	**8.70 (2.91, 30.69)**	**4.35 (1.73, 13.81)**	**5.83 (2.14, 19.41)**	**5.13 (1.88, 17.16)**	3.03 (0.59, 14.32)	3.13 (0.91, 11.65)	**5.35 (2.15, 16.62)**	4.48 (0.92, 23.61)	6.71 (0.21, 210.31)	4.79 (0.18, 152.20)	**5.02 (1.78, 16.24)**
RIS200mgIV	4.76 (0.60, 148.98)	2.89 (0.33, 90.73)	**12.23 (1.36, 415.84)**	2.56 (0.26, 89.95)	2.55 (0.26, 69.51)	4.36 (0.48, 127.14)	6.05 (0.75, 186.63)	4.71 (0.53, 136.71)	7.56 (0.97, 234.06)	5.58 (0.64, 161.87)	6.12 (0.62, 165.63)	6.95 (0.83, 202.06)	3.45 (0.47, 106.53)	4.62 (0.61, 140.48)	4.11 (0.51, 132.92)	2.43 (0.22, 89.67)	2.49 (0.27, 79.23)	4.21 (0.59, 129.94)	3.72 (0.33, 133.92)	5.69 (0.10, 788.92)	4.35 (0.10, 554.41)	4.02 (0.51, 129.44)
RIS600mgIV	**4.34 (1.52, 13.55)**	2.56 (0.78, 9.44)	**10.91 (3.75, 36.84)**	2.41 (0.58, 9.46)	2.28 (0.55, 8.96)	**3.94 (1.17, 13.85)**	**5.45 (1.98, 16.83)**	**4.20 (1.48, 13.50)**	**6.85 (2.61, 21.50)**	**5.05 (1.82, 16.22)**	**5.29 (1.57, 20.97)**	**6.25 (2.36, 19.65)**	**3.21 (1.37, 8.10)**	**4.30 (1.75, 11.37)**	**3.76 (1.52, 10.47)**	2.25 (0.44, 9.51)	2.27 (0.67, 7.40)	**3.93 (1.74, 9.76)**	3.34 (0.63, 16.80)	4.98 (0.16, 153.35)	3.61 (0.12, 121.47)	**3.64 (1.43, 10.53)**
SEC10mg_kgIV	**0.09 (0.01, 0.59)**	**0.05 (0.00, 0.36)**	0.22 (0.01, 1.54)	**0.05 (0.00, 0.37)**	**0.05 (0.00, 0.36)**	**0.08 (0.00, 0.58)**	**0.12 (0.01, 0.71)**	**0.09 (0.00, 0.56)**	**0.15 (0.01, 0.87)**	**0.11 (0.01, 0.65)**	**0.11 (0.01, 0.83)**	**0.13 (0.01, 0.80)**	**0.07 (0.00, 0.37)**	**0.09 (0.01, 0.52)**	**0.08 (0.00, 0.46)**	**0.04 (0.00, 0.38)**	**0.05 (0.00, 0.31)**	**0.08 (0.00, 0.45)**	**0.07 (0.00, 0.57)**	0.09 (0.00, 5.40)	0.07 (0.00, 3.30)	**0.08 (0.00, 0.43)**

	SEM60mgIV	SEM60mgIVx3	TES400mg200mgSC	TOF15mgOR_BID	TOF1mgOR_BID	TOF5mgOR_BID	UPA12mgOR_BID	UPA24mgOR	UPA24mgOR_BID	UPA3mgOR_BID	UPA45mgOR	UPA6mgOR_BID	UST130mgIV	UST1mg_kgIV	UST3mg_kgIV	UST4_5mg_kgIV	UST6mg_kg90mgIV_SC	UST6mg_kgIV	UST90mgSC	VED0_5mg_kgIV	VED2mg_kgIV	VED300mgIV
SEM60mgIV	SEM60mgIV	0.59 (0.24, 1.42)	2.48 (0.87, 8.22)	0.53 (0.13, 2.01)	0.51 (0.12, 2.04)	0.89 (0.27, 3.16)	1.24 (0.46, 3.68)	0.97 (0.33, 2.93)	1.57 (0.59, 4.51)	1.16 (0.42, 3.44)	1.21 (0.35, 4.44)	1.44 (0.54, 4.15)	0.73 (0.32, 1.72)	0.99 (0.40, 2.48)	0.87 (0.35, 2.22)	0.51 (0.11, 1.99)	0.52 (0.17, 1.59)	0.89 (0.41, 2.07)	0.75 (0.16, 3.36)	1.15 (0.04, 33.98)	0.82 (0.03, 24.89)	0.84 (0.33, 2.23)
SEM60mgIVx3	1.69 (0.70, 4.20)	SEM60mgIVx3	**4.22 (1.36, 15.30)**	0.92 (0.22, 3.60)	0.87 (0.20, 3.46)	1.53 (0.43, 5.52)	2.13 (0.73, 6.96)	1.65 (0.52, 5.36)	2.67 (0.95, 8.44)	1.97 (0.66, 6.39)	2.11 (0.57, 8.35)	2.46 (0.86, 7.61)	1.25 (0.49, 3.27)	1.67 (0.62, 4.70)	1.48 (0.54, 4.13)	0.87 (0.19, 3.78)	0.87 (0.28, 2.91)	1.52 (0.63, 3.97)	1.26 (0.25, 6.24)	1.96 (0.06, 64.11)	1.38 (0.05, 48.20)	1.42 (0.52, 4.14)
TES400mg200mgSC	0.40 (0.12, 1.16)	**0.24 (0.07, 0.73)**	TES400mg200mgSC	**0.21 (0.05, 0.81)**	**0.20 (0.05, 0.75)**	0.36 (0.10, 1.21)	0.50 (0.17, 1.49)	0.38 (0.12, 1.17)	0.63 (0.22, 1.81)	0.46 (0.15, 1.40)	0.48 (0.14, 1.82)	0.58 (0.20, 1.61)	**0.30 (0.12, 0.66)**	**0.40 (0.15, 0.96)**	**0.35 (0.13, 0.86)**	**0.20 (0.04, 0.80)**	**0.21 (0.06, 0.64)**	**0.36 (0.15, 0.82)**	0.30 (0.06, 1.34)	0.44 (0.01, 13.18)	0.33 (0.01, 9.91)	**0.34 (0.12, 0.87)**
TOF15mgOR_BID	1.87 (0.50, 7.47)	1.09 (0.28, 4.64)	**4.67 (1.23, 20.07)**	TOF15mgOR_BID	0.95 (0.27, 3.24)	1.64 (0.57, 5.51)	2.32 (0.66, 9.08)	1.79 (0.49, 7.07)	2.96 (0.85, 11.31)	2.16 (0.62, 8.84)	2.27 (0.53, 10.73)	2.70 (0.79, 10.72)	1.37 (0.46, 4.58)	1.85 (0.57, 6.41)	1.63 (0.51, 5.68)	0.93 (0.18, 4.75)	0.97 (0.25, 4.27)	1.68 (0.56, 5.52)	1.41 (0.24, 7.92)	2.05 (0.07, 78.94)	1.57 (0.05, 51.45)	1.57 (0.49, 5.62)
TOF1mgOR_BID	1.95 (0.49, 8.09)	1.15 (0.29, 4.99)	**4.89 (1.33, 20.39)**	1.05 (0.31, 3.71)	TOF1mgOR_BID	1.72 (0.59, 5.62)	2.48 (0.65, 9.82)	1.90 (0.50, 7.93)	3.10 (0.88, 11.98)	2.28 (0.62, 9.19)	2.40 (0.59, 11.45)	2.83 (0.79, 11.64)	1.44 (0.47, 5.02)	1.93 (0.59, 7.11)	1.71 (0.51, 6.08)	0.98 (0.19, 5.31)	1.03 (0.27, 4.32)	1.76 (0.58, 6.04)	1.49 (0.26, 8.21)	2.15 (0.06, 75.74)	1.60 (0.05, 52.75)	1.64 (0.49, 6.00)
TOF5mgOR_BID	1.13 (0.32, 3.72)	0.65 (0.18, 2.33)	2.79 (0.82, 10.30)	0.61 (0.18, 1.74)	0.58 (0.18, 1.69)	TOF5mgOR_BID	1.40 (0.44, 4.69)	1.07 (0.32, 3.71)	1.77 (0.56, 5.60)	1.29 (0.41, 4.25)	1.35 (0.36, 5.51)	1.62 (0.50, 5.25)	0.81 (0.31, 2.26)	1.09 (0.38, 3.22)	0.96 (0.34, 2.82)	0.56 (0.11, 2.57)	0.58 (0.17, 2.04)	0.99 (0.39, 2.69)	0.85 (0.16, 4.14)	1.23 (0.04, 39.84)	0.90 (0.03, 29.14)	0.94 (0.31, 2.85)
UPA12mgOR_BID	0.81 (0.27, 2.16)	0.47 (0.14, 1.36)	2.00 (0.67, 5.90)	0.43 (0.11, 1.52)	0.40 (0.10, 1.55)	0.71 (0.21, 2.30)	UPA12mgOR_BID	0.77 (0.38, 1.55)	1.25 (0.70, 2.34)	0.92 (0.49, 1.77)	0.96 (0.29, 3.49)	1.14 (0.64, 2.12)	0.59 (0.26, 1.26)	0.79 (0.32, 1.83)	0.69 (0.28, 1.64)	0.40 (0.09, 1.60)	0.42 (0.14, 1.17)	0.72 (0.32, 1.51)	0.60 (0.13, 2.55)	0.89 (0.03, 27.17)	0.65 (0.02, 20.81)	0.68 (0.26, 1.60)
UPA24mgOR	1.03 (0.34, 3.01)	0.60 (0.19, 1.91)	2.60 (0.86, 8.26)	0.56 (0.14, 2.04)	0.53 (0.13, 2.02)	0.94 (0.27, 3.11)	1.30 (0.64, 2.62)	UPA24mgOR	1.62 (0.87, 3.25)	1.20 (0.61, 2.46)	1.25 (0.36, 4.65)	1.48 (0.79, 3.01)	0.76 (0.32, 1.73)	1.03 (0.40, 2.47)	0.89 (0.36, 2.26)	0.52 (0.11, 2.06)	0.54 (0.17, 1.56)	0.93 (0.40, 2.11)	0.78 (0.17, 3.46)	1.18 (0.04, 36.89)	0.85 (0.03, 25.52)	0.87 (0.34, 2.23)
UPA24mgOR_BID	0.64 (0.22, 1.68)	0.37 (0.12, 1.06)	1.58 (0.55, 4.64)	0.34 (0.09, 1.18)	0.32 (0.08, 1.13)	0.56 (0.18, 1.78)	0.80 (0.43, 1.43)	0.62 (0.31, 1.15)	UPA24mgOR_BID	0.74 (0.40, 1.32)	0.76 (0.24, 2.69)	0.92 (0.52, 1.59)	**0.46 (0.21, 0.96)**	0.62 (0.26, 1.42)	0.55 (0.24, 1.25)	0.32 (0.07, 1.21)	**0.33 (0.11, 0.92)**	0.57 (0.27, 1.13)	0.47 (0.11, 1.97)	0.70 (0.02, 20.59)	0.52 (0.02, 15.02)	0.53 (0.21, 1.28)
UPA3mgOR_BID	0.86 (0.29, 2.39)	0.51 (0.16, 1.52)	2.16 (0.71, 6.82)	0.46 (0.11, 1.60)	0.44 (0.11, 1.61)	0.77 (0.24, 2.47)	1.08 (0.57, 2.03)	0.83 (0.41, 1.63)	1.36 (0.76, 2.49)	UPA3mgOR_BID	1.04 (0.32, 3.92)	1.23 (0.68, 2.32)	0.64 (0.27, 1.37)	0.85 (0.34, 2.03)	0.74 (0.30, 1.78)	0.43 (0.10, 1.73)	0.45 (0.14, 1.25)	0.77 (0.34, 1.63)	0.64 (0.14, 2.71)	0.95 (0.03, 30.04)	0.71 (0.03, 20.65)	0.73 (0.29, 1.82)
UPA45mgOR	0.83 (0.23, 2.84)	0.47 (0.12, 1.75)	2.07 (0.55, 7.28)	0.44 (0.09, 1.87)	0.42 (0.09, 1.70)	0.74 (0.18, 2.77)	1.04 (0.29, 3.43)	0.80 (0.22, 2.74)	1.31 (0.37, 4.24)	0.96 (0.25, 3.12)	UPA45mgOR	1.19 (0.34, 3.78)	0.61 (0.20, 1.65)	0.81 (0.25, 2.34)	0.72 (0.21, 2.05)	0.41 (0.07, 1.89)	0.42 (0.11, 1.48)	0.74 (0.24, 1.95)	0.63 (0.11, 3.18)	0.91 (0.03, 30.40)	0.66 (0.02, 19.97)	0.69 (0.21, 2.18)
UPA6mgOR_BID	0.69 (0.24, 1.86)	0.41 (0.13, 1.17)	1.74 (0.62, 5.08)	0.37 (0.09, 1.27)	0.35 (0.09, 1.26)	0.62 (0.19, 1.98)	0.87 (0.47, 1.56)	0.68 (0.33, 1.27)	1.09 (0.63, 1.91)	0.81 (0.43, 1.47)	0.84 (0.26, 2.95)	UPA6mgOR_BID	0.51 (0.23, 1.02)	0.69 (0.28, 1.53)	0.60 (0.25, 1.38)	0.35 (0.08, 1.34)	**0.37 (0.11, 0.99)**	0.62 (0.29, 1.25)	0.52 (0.12, 2.16)	0.77 (0.02, 24.10)	0.57 (0.02, 17.15)	0.58 (0.23, 1.39)
UST130mgIV	1.37 (0.58, 3.10)	0.80 (0.31, 2.02)	**3.38 (1.51, 8.51)**	0.73 (0.22, 2.18)	0.69 (0.20, 2.13)	1.23 (0.44, 3.26)	1.70 (0.79, 3.89)	1.32 (0.58, 3.10)	**2.15 (1.05, 4.72)**	1.57 (0.73, 3.65)	1.65 (0.61, 5.09)	1.96 (0.98, 4.39)	UST130mgIV	1.34 (0.77, 2.36)	1.18 (0.65, 2.09)	0.69 (0.17, 2.38)	0.71 (0.29, 1.71)	1.22 (0.88, 1.72)	1.03 (0.25, 3.92)	1.52 (0.05, 43.46)	1.15 (0.04, 32.97)	1.14 (0.60, 2.25)
UST1mg_kgIV	1.01 (0.40, 2.51)	0.60 (0.21, 1.61)	**2.53 (1.05, 6.69)**	0.54 (0.16, 1.75)	0.52 (0.14, 1.71)	0.92 (0.31, 2.61)	1.27 (0.55, 3.11)	0.97 (0.40, 2.47)	1.60 (0.70, 3.84)	1.17 (0.49, 2.91)	1.24 (0.43, 4.01)	1.46 (0.66, 3.54)	0.75 (0.42, 1.30)	UST1mg_kgIV	0.88 (0.50, 1.54)	0.51 (0.12, 1.82)	0.53 (0.20, 1.38)	0.91 (0.56, 1.51)	0.77 (0.18, 3.11)	1.13 (0.04, 34.47)	0.84 (0.03, 24.22)	0.85 (0.41, 1.81)
UST3mg_kgIV	1.15 (0.45, 2.87)	0.67 (0.24, 1.86)	**2.89 (1.17, 7.82)**	0.61 (0.18, 1.97)	0.58 (0.16, 1.95)	1.05 (0.35, 2.97)	1.44 (0.61, 3.62)	1.12 (0.44, 2.79)	1.83 (0.80, 4.23)	1.35 (0.56, 3.31)	1.39 (0.49, 4.70)	1.67 (0.72, 3.94)	0.85 (0.48, 1.53)	1.13 (0.65, 1.99)	UST3mg_kgIV	0.57 (0.14, 2.12)	0.60 (0.22, 1.59)	1.03 (0.63, 1.76)	0.87 (0.20, 3.55)	1.28 (0.04, 39.23)	0.95 (0.03, 29.43)	0.97 (0.46, 2.08)
UST4_5mg_kgIV	1.96 (0.50, 8.94)	1.15 (0.26, 5.37)	**5.05 (1.24, 22.98)**	1.07 (0.21, 5.54)	1.02 (0.19, 5.20)	1.78 (0.39, 8.79)	2.50 (0.62, 11.30)	1.92 (0.48, 8.90)	3.13 (0.83, 14.53)	2.31 (0.58, 10.20)	2.42 (0.53, 13.46)	2.87 (0.75, 12.98)	1.45 (0.42, 5.83)	1.96 (0.55, 8.25)	1.74 (0.47, 7.00)	UST4_5mg_kgIV	1.05 (0.24, 4.58)	1.79 (0.52, 6.99)	1.53 (0.23, 9.29)	2.25 (0.06, 94.47)	1.71 (0.05, 52.06)	1.64 (0.46, 7.25)
UST6mg_kg90mgIV_SC	1.91 (0.63, 5.88)	1.15 (0.34, 3.59)	**4.83 (1.57, 15.70)**	1.03 (0.23, 3.97)	0.97 (0.23, 3.73)	1.72 (0.49, 6.00)	2.38 (0.86, 7.37)	1.84 (0.64, 5.88)	**3.02 (1.09, 9.31)**	2.20 (0.80, 7.03)	2.35 (0.67, 9.26)	**2.73 (1.01, 8.71)**	1.41 (0.58, 3.47)	1.88 (0.73, 4.93)	1.65 (0.63, 4.45)	0.95 (0.22, 4.23)	UST6mg_kg90mgIV_SC	1.73 (0.72, 4.22)	1.42 (0.31, 6.90)	2.11 (0.07, 73.75)	1.59 (0.05, 47.12)	1.61 (0.59, 4.72)
UST6mg_kgIV	1.12 (0.48, 2.46)	0.66 (0.25, 1.60)	**2.79 (1.21, 6.82)**	0.60 (0.18, 1.78)	0.57 (0.17, 1.72)	1.01 (0.37, 2.57)	1.39 (0.66, 3.15)	1.08 (0.47, 2.49)	1.76 (0.88, 3.76)	1.29 (0.61, 2.91)	1.35 (0.51, 4.14)	1.61 (0.80, 3.49)	0.82 (0.58, 1.13)	1.10 (0.66, 1.79)	0.97 (0.57, 1.58)	0.56 (0.14, 1.91)	0.58 (0.24, 1.38)	UST6mg_kgIV	0.84 (0.21, 3.16)	1.24 (0.04, 34.78)	0.94 (0.03, 27.97)	0.93 (0.50, 1.81)
UST90mgSC	1.33 (0.30, 6.38)	0.79 (0.16, 3.94)	3.35 (0.75, 17.50)	0.71 (0.13, 4.10)	0.67 (0.12, 3.90)	1.18 (0.24, 6.29)	1.67 (0.39, 7.76)	1.29 (0.29, 5.96)	2.11 (0.51, 9.18)	1.56 (0.37, 6.91)	1.59 (0.31, 9.42)	1.93 (0.46, 8.52)	0.97 (0.26, 3.93)	1.29 (0.32, 5.64)	1.15 (0.28, 5.11)	0.65 (0.11, 4.36)	0.70 (0.14, 3.27)	1.19 (0.32, 4.78)	UST90mgSC	1.50 (0.04, 49.08)	1.07 (0.03, 37.73)	1.14 (0.27, 5.14)
VED0_5mg_kgIV	0.87 (0.03, 26.67)	0.51 (0.02, 17.32)	2.25 (0.08, 79.50)	0.49 (0.01, 14.65)	0.46 (0.01, 17.46)	0.81 (0.03, 28.23)	1.13 (0.04, 35.01)	0.85 (0.03, 28.44)	1.42 (0.05, 44.48)	1.05 (0.03, 32.47)	1.10 (0.03, 36.73)	1.30 (0.04, 40.48)	0.66 (0.02, 19.15)	0.89 (0.03, 26.96)	0.78 (0.03, 22.87)	0.44 (0.01, 16.03)	0.47 (0.01, 15.08)	0.81 (0.03, 22.84)	0.67 (0.02, 24.69)	VED0_5mg_kgIV	0.73 (0.03, 23.64)	0.74 (0.03, 22.30)
VED2mg_kgIV	1.23 (0.04, 33.63)	0.73 (0.02, 21.22)	3.02 (0.10, 99.82)	0.64 (0.02, 20.49)	0.63 (0.02, 19.69)	1.12 (0.03, 33.95)	1.54 (0.05, 44.97)	1.18 (0.04, 32.41)	1.93 (0.07, 52.56)	1.41 (0.05, 38.58)	1.51 (0.05, 44.11)	1.76 (0.06, 50.59)	0.87 (0.03, 24.70)	1.19 (0.04, 33.14)	1.05 (0.03, 29.25)	0.59 (0.02, 18.74)	0.63 (0.02, 18.34)	1.06 (0.04, 29.19)	0.94 (0.03, 29.53)	1.37 (0.04, 37.22)	VED2mg_kgIV	1.01 (0.03, 27.76)
VED300mgIV	1.20 (0.45, 3.07)	0.70 (0.24, 1.93)	**2.97 (1.15, 8.19)**	0.64 (0.18, 2.05)	0.61 (0.17, 2.03)	1.07 (0.35, 3.21)	1.47 (0.62, 3.84)	1.15 (0.45, 2.97)	1.88 (0.78, 4.66)	1.37 (0.55, 3.51)	1.45 (0.46, 4.87)	1.72 (0.72, 4.31)	0.88 (0.44, 1.66)	1.17 (0.55, 2.43)	1.03 (0.48, 2.17)	0.61 (0.14, 2.16)	0.62 (0.21, 1.69)	1.08 (0.55, 1.99)	0.88 (0.19, 3.67)	1.34 (0.04, 37.28)	0.99 (0.04, 28.97)	VED300mgIV

TABLE S17. SUCRA PROBABILITYS FOR SAFETY OUTCOMES. Higher probabilities are more closely associated with the chance of the drug treatment having the highest occurrence of the event in relation to the other drugs included in the network. Lowers probabilities indicate better-performing interventions. For better visualization access the excel version using the LINK

	% SUCRA serious adverse events	% SUCRA adverse events	% SUCRA infections
ABA10mg/kgIV	54.9	58.2	41.4
ABA30mg/kgIV	52.7	-	28.0
ABA3mg/kgIV	49.7	32.7	36.5
ADA160mg80mg40mgSC	57.9	-	37.4
ADA160mg80mg60mgSC	70.5	21.3	49.9
ADA160mg80mgSC	30.1	-	49.6
ADA40mg20mgSC	14.0	58.8	24.9
ADA80mg40mgSC	12.9	47.8	49.1
AMI0.4mgPO	90.7	10.9	-
AND150mgSC1/1	54.4	13.0	42.1
AND150mgSC2/2	11.2	27.9	43.9
AND300mgSC	64.4	51.4	39.2
API100mgPO	27.1	40.6	-
API50mgPO	39.5	22.8	-
BRI400mgIV	29.9	39.3	52.6
BRI700mgIV	19.8	61.1	51.9
BRO210mgIV	64.1	35.7	49.4
BRO350mgIV	87.1	65.4	48.3
BRO700mgIV	89.1	45.8	66.8
CDP10mg/kgIV	40.1	56.1	56.6
CER1.25mgIV	-	58.1	-
CER100mgSC	60.0	48.6	-
CER10mgIV	38.4	67.4	-
CER200mgSC	73.4	67.6	-
CER20mgIV	80.4	54.8	-
CER400mgSC	62.6	38.5	82.9
CER5mgIV	28.5	36.7	-
ELD10mg/kgIV	62.7	53.7	31.8
ELD20mg/kgIV	70.8	49.6	41.1
ETA25mgSC	27.4	32.2	-
ETR105mgSC	52.1	31.8	80.2
ETR210mgSC	36.3	31.5	81.3
FIL100mgPO	64.6	38.3	48.4
FIL200mgPO	60.5	26.7	54.9
FON0.1mg/kgIV	54.0	28.7	-
FON1mg/kg0.1mgIV/SC	-	92.1	54.0
FON1mg/kg1mgIV/SC	-	86.4	51.4
FON1mg/kgIV	65.6	73.0	-
FON4mg/kg0.1mgIV/SC	-	47.0	51.2
FON4mg/kg1mgIV/SC	-	20.3	53.5
FON4mg/kgIV	62.7	84.7	51.2
GUS1200mgIV	13.7	44.6	36.5
GUS200mgIV	37.7	69.1	27.1

	% SUCRA serious adverse events	% SUCRA adverse events	% SUCRA infections
GUS600mgIV	48.9	73.0	44.9
MED700IV	50.6	74.2	15.9
MIR1000mgIV	14.4	80.5	-
MIR200mgIV	14.8	65.3	-
MIR600mgIV	42.5	64.2	-
NAT300mgIV	40.3	43.7	65.8
NAT3mg/kgIV	47.1	56.2	42.4
NAT3mg/kgIVx2	42.5	48.7	51.3
NAT6mg/kgIVx2	51.8	65.8	31.7
NNC2mg/kgSC	76.5	27.9	21.3
ONE10mgSC	44.6	47.0	-
ONE25mgSC	48.4	21.7	-
ONE35mgSC	78.4	69.3	-
ONE50mgSC	45.5	63.8	-
ONT22.5mgSC	75.5	49.3	-
ONT225mgSC	74.7	30.4	-
ONT75mgSC	69.9	27.8	-
PBO	50.8	54.1	56.9
PF10mgSC	41.3	42.8	74.3
PF200mgSC	75.1	19.6	84.5
PF50mgSC	49.6	15.9	72.1
RIS1200mgIV	12.0	36.4	5.3
RIS200mgIV	35.2	42.3	14.6
RIS600mgIV	20.2	31.2	8.1
RIS600mgIV4/4	-	35.1	-
SEC10mg/kgIV	58.6	89.6	97.3
SEM60mgIV	-	70.4	60.7
SEM60mgIVx3	-	83.1	33.7
TES400mg200mgSC	94.0	78.8	89.6
TOF15mgPO_BID	10.5	48.9	31.7
TOF1mgPO_BID	40.0	24.3	29.8
TOF5mgPO_BID	41.8	46.2	54.7
UPA12mgPO_BID	91.0	71.6	71.9
UPA24mgPO	85.4	77.8	58.2
UPA24mgPO_BID	62.8	78.9	81.4
UPA3mgPO_BID	75.1	87.4	68.1
UPA45mgPO	49.4	62.6	68.2
UPA6mgPO_BID	47.8	66.3	78.2
UST130mgIV	41.2	45.9	42.2
UST1mg/kgIV	28.7	57.3	61.5
UST3mg/kgIV	37.6	51.2	53.5
UST4.5mg/kgIV	71.2	67.6	30.1
UST6mg/kg90mgIV/SC	50.3	33.0	28.1
UST6mg/kgIV	43.8	48.7	56.2
UST90mgSC	32.9	16.4	46.4
VED0.5mg/kgIV	29.9	84.6	56.4
VED2mg/kgIV	46.2	71.8	50.2
VED300mgIV	50.4	51.4	51.5

TABLE S18. SENSITIVE ANALYSIS OF RISK OF BIAS Each table presents the results for gelman rubin, geometric analysis, SUCRA (for remission, SAE and IBDQ) and league tables (for remission, SAE and IBDQ). Lowers probabilities indicate better-performing interventions. For better visualization access the excel version using the LINK

Table S18a: Geometric metrics for sensitivity analysis by risk of bias. Nodes represent the number of interventions in the network. Edges represent direct comparisons, and "Edges more than 1study" represent comparisons with more than one study. Density measures the connectedness of a graph. The mean thickness represents the total number of studies divided by the total number of edges, and the percentage of common comparators represents the directly connected nodes that can be considered "common comparators." The percentage of strong edges represents the number of studies on an edge, which is proportional to the direct evidence between two nodes. The greater the number of studies in a comparison, the more robust the evidence.

Outcome	Nodes	Edges	Studies	Edges more than 1study	Density	Mean thickness	Common comparator	strong edges
Remission low risk of bias	38	68	22	5	0.1	0.3	73.7	7.4
Remission some concerns	52	93	30	8	0.1	0.3	88.5	8.6
Remission high risk of bias	6	11	2	0	0.7	0.2	83.3	0.0
SAE low risk of bias	41	77	22	5	0.1	0.3	78.0	6.5
SAE some concerns	44	75	24	8	0.1	0.3	86.4	10.7
SAE high risk of bias		2	2	0	0.7	1.0	33.3	0.0
IBDQ low risk of bias	12	23	7	4	0.3	0.3	83.3	17.4
IBDQ some concerns	10	18	4	0	0.4	0.2	80	0

Table S18b: Gelman-rubin brooks for sensitivity analysis by risk of bias. A potential scale reduction factor (PSRF) between 1 and 1.05 indicates convergence of the Bayesian model.

Outcome	PSRF Point est. (sd)	PSRF Upper C.I. (sd)
Remission low risk of bias	1.01	1.02
Remission some concerns	1.00	1.00
Remission high risk of bias	1.00	1.00
SAE low risk of bias	1.00	1.01
SAE some concerns	1.00	1.00
SAE high risk of bias	1.00	1.00
IBDQ low risk of bias	1.00	1.01
IBDQ some concerns	1.00	1.00

Table S18c. The results for the SUCRA analysis for sensitivity analysis by risk of bias for clinical remisison. Higher probabilities are more closely associated with the chance of the drug treatment having the highest occurrence of the event in relation to the other drugs included in the network. Higher probabilities indicate better-performing interventions

	% SUCRA remission (original analysis)	% SUCRA remission (low risk of bias)	% SUCRA remission (some concerns)
ABA10mg/kgIV	15.94	13.58	16.19
ABA30mg/kgIV	41.41	36.72	44.99
ABA3mg/kgIV	22.44	18.96	22.58
ADA160mg80mg40mgSC	81.31	76.09	-
ADA160mg80mg60mgSC	95.14	92.83	-
ADA160mg80mgSC	82.73	77.71	80.99
ADA40mg20mgSC	49.73	43.5	50.57
ADA80mg40mgSC	76.16	70.44	76.36
AMI0.4mgPO	17.04	-	18.91
AND150mgSC1/1	25.64	-	27.29
AND150mgSC2/2	34.23	-	37.74
AND300mgSC	13.62	-	14.78
API100mgPO	19.95	-	20.83
API50mgPO	11.53	-	11.98
BRI400mgIV	80.04	-	81.8
BRI700mgIV	70.92	-	74.77
BRO210mgIV	16.45	20.22	42.46
BRO350mgIV	72.48	71.97	87.95
BRO700mgIV	52.92	50.2	77.17
CDP10mg/kgIV	31.13	-	33.26
CER100mgSC	37.49	29.92	40.21
CER10mg/kgIV	20.72	18.8	20.56
CER200mgSC	21.96	17.58	23.76
CER20mg/kgIV	7.66	7.45	8.03
CER400mgSC	41.06	32.81	44.07
CER5mg/kgIV	32.93	29.57	36.21
ELD10mg/kgIV	38.08	-	42.61
ELD20mg/kgIV	52.25	-	55.49
ETA25mgSC	14.15	12.33	-
	27.75	-	-
	35.01	-	-
FIL100mgPO	64.83	30.02	-
FIL200mgPO	53.42	58.85	-
FON0.1mg/kgIV	15.39	-	14.11
FON1mg/kg0.1mgIV/SC	36.81	-	41.38
FON1mg/kg1mgIV/SC	49.57	-	53.78
FON1mg/kgIV	28.99	-	31.73
FON4mg/kg0.1mgIV/SC	60.78	-	65.67
FON4mg/kg1mgIV/SC	50.17	-	55.95
FON4mg/kgIV	56.97	-	58.19

	% SUCRA remission (original analysis)	% SUCRA remission (low risk of bias)	% SUCRA remission (some concerns)
GUS1200mgIV	83.17	78.03	-
GUS200mgIV	90.05	86.55	-
GUS600mgIV	89.17	85.58	-
INF10mg/kgIV	92.03	89.5	90.95
INF20mg/kgIV	91.81	90.7	91.2
INF5mg/kgIV	98.59	98.17	98.33
MED700IV	54.93	-	-
MIR1000mgIV	82.36	76.18	-
MIR200mgIV	55.66	50.5	-
MIR600mgIV	91.51	87.75	-
NAT300mgIV	48.73	39.87	52.77
NAT3mg/kgIV	33.44	25.86	35.94
NAT3mg/kgIVx2	57.13	48.68	61.23
NAT6mg/kgIVx2	52.17	43.2	56.27
NNC2mg/kgSC	57.82	-	-
ONE10mgSC	43.91	-	-
ONE25mgSC	14.56	-	-
ONE35mgSC	45.13	-	-
ONE50mgSC	27.58	-	-
ONT22.5mgSC	38.99	-	-
ONT225mgSC	43.37	-	-
ONT75mgSC	40.02	-	-
PBO	29.43	22.75	31.17
PF10mgSC	31.88	-	-
PF200mgSC	32.45	-	-
PF50mgSC	79.66	-	-
RIS1200mgIV	64.05	55.56	68.04
RIS200mgIV	47.59	38.32	73.69
RIS600mgIV	69.54	61.84	53.18
RIS600mgIV4/4	49.11	40.65	-
SEC10mg/kgIV	26.21	22.07	-
SEM60mgIV	-	-	-
SEM60mgIVx3	-	-	-
TES400mg200mgSC	69.66	-	-
TOF15mgPO_BID	19.9	16.37	-
TOF1mgPO_BID	54.35	46.95	-
TOF5mgPO_BID	40.7	33.55	-
UPA12mgPO_BID	41.84	40.11	-
UPA24mgPO	23.98	20.55	-
UPA24mgPO_BID	69.67	66.23	-
UPA3mgPO_BID	29.17	26.26	-
UPA45mgPO	63.14	54.31	-
UPA6mgPO_BID	77.03	74.18	-
UST130mgIV	60.85	52.42	-
UST1mg/kgIV	67.24	60.34	62.06
UST3mg/kgIV	69.36	62.63	64.89

	% SUCRA remission (original analysis)	% SUCRA remission (low risk of bias)	% SUCRA remission (some concerns)
UST4.5mg/kgIV	61.59	53.32	65.52
UST6mg/kg90mgIV/SC	83.32	78.25	64.49
UST6mg/kgIV	75.68	69.18	-
UST90mgSC	37	28.92	36.96
VED0.5mg/kgIV	50.26	42.57	50.72
VED2mg/kgIV	63.16	56.38	66.1
VED300mgIV	73.27	66.18	64.13

Table S18d. The results for the SUCRA analysis for sensitivity analysis by risk of bias for IBDQ score. Higher probabilities are more closely associated with the chance of the drug treatment having the highest occurrence of the event in relation to the other drugs included in the network. Higher probabilities indicate better-performing interventions

	% SUCRA IBDQ score (original analysis)	%SUCRA IBDQ low (risk of bias)	%SUCRA IBDQ some concerns
CER400mgSC	48.29	-	50.6
FIL200mgPO	57.29	51.78	-
FON1mg/kg0.1mgIV_SC	18.75	-	30.66
FON1mg/kg1mgIV_SC	38.64	-	45.84
FON4mg/kg0.1mgIV_SC	26.20	-	35.33
FON4mg/kg1mgIV_SC	31.48	-	39.59
INF10mg/kgIV	74.12	-	67.56
INF20mg/kgIV	76.91	-	68.81
INF5mg/kgIV	95.14	-	85.67
NAT300mgIV	45.21	-	49.38
PBO	14.02	5.49	26.58
RIS200mgIV	52.52	45.76	-
RIS600mgIV	80.35	80.11	-
SEM60mgIV	14.43	-	-
SEM60mgIVx3	20.16	-	-
UPA12mgPO_BID	59.77	55.01	-
UPA24mgPO	35.89	26.55	-
UPA24mgPO_BID	83.87	84.31	-
UPA3mgPO_BID	40.78	33.6	-
UPA45mgPO	74.68	72.44	-
UPA6mgPO_BID	78.23	78.84	-
UST130mgIV	40.46	31.85	-
UST6mg/kgIV	42.82	34.28	-

Table S18e. The results for the SUCRA analysis for sensitivity analysis by risk of bias for IBDQ score. Higher probabilities are more closely associated with the chance of the drug treatment having the highest occurrence of the event in relation to the other drugs included in the network. Lower probabilities indicate better-performing interventions

	% SUCRA serious adverse events (original analysis)	% SUCRA serious adverse events low (risk of bias)	% SUCRA serious adverse events some concerns	% SUCRA serious adverse events high (risk of bias)
ABA10mg/kgIV	54.88	-	57.04	-
ABA30mg/kgIV	52.74	-	55.53	-
ABA3mg/kgIV	49.67	-	52.72	-
ADA160mg80mg40mgSC	57.85	57.01	-	-
ADA160mg80mg60mgSC	70.5	70.02	-	-
ADA160mg80mgSC	30.06	15.43	53.59	-
ADA40mg20mgSC	14.02	-	18.79	-
ADA80mg40mgSC	12.87	-	19.68	-
AMI0.4mgPO	90.72	-	91.69	-
AND150mgSC1/1	54.42	-	57.02	-
AND150mgSC2/2	11.18	-	10.96	-
AND300mgSC	64.44	-	66.42	-
API100mgPO	27.07	-	28.65	-
API50mgPO	39.52	-	42.47	-
BRI400mgIV	29.86	-	29.82	-
BRI700mgIV	19.75	-	20.37	-
BRO210mgIV	64.06	-	63.93	-
BRO350mgIV	87.11	-	87.95	-
BRO700mgIV	89.06	-	89.93	-
CDP10mg/kgIV	40.06	-	42.85	-
CER100mgSC	59.98	-	62.07	-
CER10mgIV	38.37	-	41.71	-
CER200mgSC	73.43	-	74.77	-
CER20mgIV	80.35	-	81.43	-
CER400mgSC	62.62	-	64.65	-
CER5mgIV	28.51	-	31.46	-
ELD10mg/kgIV	62.65	-	64.25	-
ELD20mg/kgIV	70.77	-	72.63	-
ETA25mgSC	27.4	-	-	24.48
ETR105mgSC	52.06	51.35	-	-
ETR210mgSC	36.34	37.5	-	-
FIL100mgPO	64.63	-	-	-
FIL200mgPO	60.46	73.99	-	-
FON0.1mg/kgIV	53.96	-	57.34	-
FON1mg/kgIV	65.59	-	66.37	-
FON4mg/kgIV	62.71	-	65.9	-
GUS1200mgIV	13.66	14.16	-	-
GUS200mgIV	37.74	39.65	-	-
GUS600mgIV	48.86	49.48	-	-

	% SUCRA serious adverse events (original analysis)	% SUCRA serious adverse events low (risk of bias)	% SUCRA serious adverse events some concerns	% SUCRA serious adverse events high (risk of bias)
MED700IV	50.55	-	-	62.94
MIR1000mgIV	14.42	16.69	-	-
MIR200mgIV	14.82	16.38	-	-
MIR600mgIV	42.5	43.66	-	-
NAT300mgIV	40.25	-	42.95	-
NAT3mg/kgIV	47.05	-	49.75	-
NAT3mg/kgIVx2	42.47	-	45.34	-
NAT6mg/kgIVx2	51.76	-	54.97	-
NNC2mg/kgSC	76.52	76.23	-	-
ONE10mgSC	44.6	30.54	-	-
ONE25mgSC	48.37	30.87	-	-
ONE35mgSC	78.36	63.46	-	-
ONE50mgSC	45.45	29.6	-	-
ONT22.5mgSC	75.54	73.63	-	-
ONT225mgSC	74.7	73.09	-	-
ONT75mgSC	69.89	67.97	-	-
PBO	50.83	50.25	53.88	62.58
PF10mgSC	41.34	42.59	-	-
PF200mgSC	75.07	73.6	-	-
PF50mgSC	49.61	48.96	-	-
RIS1200mgIV	11.96	-	12.91	-
RIS200mgIV	35.2	35.4	-	-
RIS600mgIV	20.21	18.66	23.15	-
SEC10mg/kgIV	58.59	58.49	-	-
TES400mg200mgSC	94.02	93.47	-	-
TOF15mgPO_BID	10.48	12.01	-	-
TOF1mgPO_BID	40.01	40.91	-	-
TOF5mgPO_BID	41.84	42.8	-	-
UPA12mgPO_BID	90.98	90.84	-	-
UPA24mgPO	85.35	84.89	-	-
UPA24mgPO_BID	62.81	63.03	-	-
UPA3mgPO_BID	75.13	75.36	-	-
UPA45mgPO	49.38	49.16	-	-
UPA6mgPO_BID	47.76	49.24	-	-
UST130mgIV	41.24	42.45	-	-
UST1mg/kgIV	28.73	-	31.65	-
UST3mg/kgIV	37.56	-	40.07	-
UST4.5mg/kgIV	71.17	-	71.97	-
UST6mg/kg90mgIV/SC	50.31	49.75	-	-
UST6mg/kgIV	43.75	44.03	45.64	-
UST90mgSC	32.93	-	31.6	-
VED0.5mg/kgIV	29.91	-	31.5	-
VED2mg/kgIV	46.21	-	48.85	-
VED300mgIV	50.44	53.4	43.76	-

Table S18f: League table for clinical remission for the low risk of bias studies. The table presented the multiple treatment comparisons based on consistency analysis of the networks. Treatments are depicted alphabetically. Values are presented as relative risk (RR) with 95% credible interval (CrI). For all comparisons an RR <1 favors the occurrence of the event for the row-defining treatment, while an RR>1 favors the column-defining treatment. Statistically significant results are represented in bold. Multiple treatment comparisons based on consistency analysis of the networks. Treatments are depicted alphabetically. Values are presented as relative risk (RR) with 95% credible interval (CrI). For all comparisons an RR <1 favors the occurrence of the event for the row-defining treatment, while an RR>1 favors the column-defining treatment. Statistically significant results are represented in bold.

	ABA10mg_kgIV	ABA30mg_kgIV	ABA3mg_kgIV	ADA160mg80mg40mgSC	ADA160mg80mg60mgSC	ADA160mg80mgSC	ADA40mg20mgSC	ADA80mg40mgSC	BRO210mgIV	BRO350mgIV	BRO700mgIV	CER100mgSC	CER10mg_kgIV	CER200mgSC	CER20mg_kgIV	CER400mgSC	CER5mg_kgIV	ETA25mgSC	FIL100mgPO	FIL200mgPO	GUS1200mgIV
ABA10mg_kgIV	ABA10mg_kgIV	1.97 (0.59, 8.10)	1.23 (0.41, 4.10)	**4.62 (1.35, 22.52)**	**9.88 (2.72, 42.81)**	**4.46 (1.40, 18.32)**	2.39 (0.64, 11.03)	**3.89 (1.14, 17.64)**	0.78 (0.04, 12.45)	5.14 (0.73, 49.96)	2.82 (0.34, 27.85)	1.85 (0.59, 8.29)	1.24 (0.24, 5.64)	1.32 (0.39, 6.00)	0.67 (0.11, 3.70)	2.00 (0.71, 8.21)	1.67 (0.44, 8.26)	0.82 (0.12, 6.00)	1.84 (0.61, 7.96)	**3.04 (1.03, 13.00)**	**4.75 (1.45, 22.22)**
ABA30mg_kgIV	0.51 (0.12, 1.70)	ABA30mg_kgIV	0.61 (0.18, 2.10)	2.38 (0.65, 9.73)	**5.02 (1.31, 21.72)**	2.29 (0.72, 8.27)	1.22 (0.32, 4.72)	1.98 (0.60, 7.35)	0.43 (0.03, 6.92)	2.57 (0.41, 23.82)	1.40 (0.16, 13.95)	0.94 (0.28, 4.03)	0.64 (0.12, 2.69)	0.68 (0.19, 2.67)	0.35 (0.05, 1.87)	1.01 (0.34, 3.60)	0.89 (0.22, 3.69)	0.42 (0.07, 2.60)	0.94 (0.28, 3.66)	1.55 (0.49, 5.57)	2.47 (0.68, 9.59)
ABA3mg_kgIV	0.81 (0.24, 2.46)	1.63 (0.48, 5.42)	ABA3mg_kgIV	**3.94 (1.11, 13.89)**	**7.96 (2.09, 31.53)**	**3.76 (1.12, 12.07)**	1.98 (0.52, 7.36)	3.26 (0.93, 11.03)	0.67 (0.03, 10.29)	4.18 (0.60, 39.60)	2.24 (0.28, 22.77)	1.58 (0.46, 5.31)	1.03 (0.19, 4.36)	1.12 (0.32, 3.81)	0.55 (0.09, 2.94)	1.72 (0.54, 5.08)	1.42 (0.34, 5.76)	0.67 (0.11, 4.64)	1.59 (0.48, 5.03)	2.61 (0.79, 8.12)	**4.09 (1.17, 13.70)**
ADA160mg80mg40mgSC	**0.22 (0.04, 0.74)**	0.42 (0.10, 1.54)	**0.25 (0.07, 0.90)**	ADA160mg80mg40mgSC	2.07 (0.76, 6.10)	0.97 (0.42, 2.14)	0.52 (0.19, 1.33)	0.84 (0.34, 1.96)	0.17 (0.01, 2.13)	1.08 (0.19, 8.47)	0.59 (0.09, 4.96)	**0.40 (0.17, 0.91)**	**0.26 (0.07, 0.87)**	**0.29 (0.12, 0.67)**	**0.15 (0.03, 0.58)**	**0.44 (0.21, 0.88)**	0.37 (0.12, 1.13)	**0.18 (0.03, 0.82)**	**0.41 (0.17, 0.91)**	0.67 (0.30, 1.40)	1.04 (0.64, 1.66)
ADA160mg80mg60mgSC	**0.10 (0.02, 0.37)**	**0.20 (0.05, 0.76)**	**0.13 (0.03, 0.48)**	0.48 (0.16, 1.31)	ADA160mg80mg60mgSC	0.46 (0.17, 1.23)	**0.25 (0.08, 0.73)**	0.40 (0.14, 1.07)	0.08 (0.01, 1.07)	0.52 (0.09, 3.97)	0.28 (0.04, 2.42)	**0.19 (0.07, 0.47)**	**0.13 (0.03, 0.43)**	**0.14 (0.05, 0.35)**	**0.07 (0.01, 0.28)**	**0.21 (0.08, 0.46)**	**0.18 (0.05, 0.60)**	**0.09 (0.01, 0.40)**	**0.20 (0.07, 0.48)**	**0.32 (0.12, 0.73)**	0.51 (0.17, 1.31)
ADA160mg80mgSC	**0.22 (0.05, 0.72)**	0.44 (0.12, 1.39)	**0.27 (0.08, 0.89)**	1.03 (0.47, 2.36)	2.16 (0.82, 5.82)	ADA160mg80mgSC	**0.54 (0.29, 0.91)**	0.87 (0.56, 1.30)	0.17 (0.01, 2.06)	1.12 (0.22, 8.24)	0.60 (0.10, 5.41)	**0.42 (0.21, 0.82)**	**0.27 (0.08, 0.81)**	**0.30 (0.14, 0.62)**	**0.15 (0.03, 0.52)**	**0.45 (0.27, 0.74)**	0.39 (0.14, 1.05)	**0.18 (0.03, 0.77)**	**0.42 (0.22, 0.76)**	0.69 (0.39, 1.18)	1.07 (0.51, 2.43)
ADA40mg20mgSC	0.42 (0.09, 1.55)	0.82 (0.21, 3.11)	0.51 (0.14, 1.93)	1.92 (0.75, 5.24)	**4.00 (1.37, 12.14)**	**1.85 (1.09, 3.45)**	ADA40mg20mgSC	1.62 (0.88, 3.06)	0.32 (0.03, 4.66)	2.15 (0.38, 16.17)	1.16 (0.18, 11.24)	0.77 (0.34, 1.86)	0.52 (0.13, 1.68)	0.56 (0.23, 1.38)	0.28 (0.06, 1.10)	0.84 (0.42, 1.73)	0.72 (0.23, 2.15)	0.34 (0.06, 1.59)	0.78 (0.36, 1.76)	1.29 (0.62, 2.81)	2.00 (0.80, 5.54)
ADA80mg40mgSC	**0.26 (0.06, 0.88)**	0.50 (0.14, 1.66)	0.31 (0.09, 1.08)	1.19 (0.51, 2.94)	2.50 (0.94, 6.91)	1.15 (0.77, 1.79)	0.62 (0.33, 1.13)	ADA80mg40mgSC	0.20 (0.02, 2.58)	1.30 (0.24, 9.83)	0.69 (0.11, 6.17)	0.48 (0.23, 1.03)	0.32 (0.09, 1.00)	**0.34 (0.15, 0.75)**	**0.17 (0.04, 0.61)**	**0.52 (0.30, 0.93)**	0.44 (0.16, 1.26)	**0.21 (0.04, 0.95)**	**0.48 (0.24, 0.96)**	0.79 (0.43, 1.53)	1.22 (0.56, 2.94)
BRO210mgIV	1.28 (0.08, 24.04)	2.35 (0.14, 38.83)	1.50 (0.10, 29.19)	5.99 (0.47, 82.55)	12.31 (0.93, 166.58)	5.77 (0.48, 72.15)	3.10 (0.21, 38.69)	4.96 (0.39, 64.61)	BRO210mgIV	5.67 (0.91, 71.54)	3.23 (0.37, 42.06)	2.35 (0.20, 30.89)	1.50 (0.10, 22.55)	1.66 (0.13, 24.19)	0.80 (0.04, 13.97)	2.59 (0.22, 31.92)	2.11 (0.14, 28.43)	0.99 (0.06, 15.02)	2.41 (0.19, 30.13)	3.98 (0.35, 48.29)	6.00 (0.48, 80.69)
BRO350mgIV	0.19 (0.02, 1.37)	0.39 (0.04, 2.45)	0.24 (0.03, 1.66)	0.93 (0.12, 5.15)	1.93 (0.25, 11.57)	0.90 (0.12, 4.47)	0.46 (0.06, 2.66)	0.77 (0.10, 4.21)	0.18 (0.01, 1.10)	BRO350mgIV	0.54 (0.12, 2.14)	0.36 (0.05, 1.93)	0.23 (0.03, 1.65)	0.27 (0.04, 1.44)	0.13 (0.01, 1.04)	0.40 (0.06, 2.07)	0.33 (0.04, 2.23)	0.16 (0.01, 1.37)	0.37 (0.05, 2.01)	0.60 (0.09, 3.19)	0.96 (0.12, 5.39)
BRO700mgIV	0.35 (0.04, 2.94)	0.72 (0.07, 6.14)	0.45 (0.04, 3.54)	1.70 (0.20, 11.15)	3.63 (0.41, 25.36)	1.68 (0.18, 9.92)	0.87 (0.09, 5.70)	1.46 (0.16, 9.15)	0.31 (0.02, 2.69)	1.84 (0.47, 8.35)	BRO700mgIV	0.69 (0.08, 4.53)	0.45 (0.04, 3.41)	0.50 (0.06, 3.14)	0.24 (0.02, 2.23)	0.75 (0.09, 4.48)	0.64 (0.07, 5.13)	0.30 (0.02, 3.09)	0.70 (0.08, 4.42)	1.15 (0.13, 7.01)	1.80 (0.19, 11.19)
CER100mgSC	0.54 (0.12, 1.68)	1.06 (0.25, 3.58)	0.63 (0.19, 2.19)	**2.48 (1.10, 5.92)**	**5.15 (2.13, 14.37)**	**2.41 (1.22, 4.66)**	1.30 (0.54, 2.95)	2.10 (0.97, 4.30)	0.43 (0.03, 5.08)	2.75 (0.52, 18.63)	1.44 (0.22, 12.97)	CER100mgSC	0.66 (0.17, 2.09)	0.72 (0.38, 1.33)	0.36 (0.08, 1.33)	1.08 (0.67, 1.78)	0.92 (0.34, 2.58)	0.44 (0.08, 1.89)	1.00 (0.53, 1.94)	1.66 (0.92, 3.03)	**2.57 (1.18, 5.88)**
CER10mg_kgIV	0.81 (0.18, 4.16)	1.57 (0.37, 8.21)	0.97 (0.23, 5.16)	**3.83 (1.15, 14.55)**	**7.97 (2.31, 31.98)**	**3.68 (1.24, 12.70)**	1.93 (0.59, 7.77)	3.16 (1.00, 11.74)	0.67 (0.04, 9.75)	4.29 (0.60, 34.98)	2.24 (0.29, 22.41)	1.52 (0.48, 6.03)	CER10mg_kgIV	1.12 (0.32, 4.08)	0.56 (0.12, 2.19)	1.65 (0.59, 5.68)	1.39 (0.53, 4.73)	0.66 (0.11, 4.23)	1.53 (0.50, 5.72)	2.50 (0.87, 8.86)	**3.90 (1.22, 14.69)**
CER200mgSC	0.76 (0.17, 2.54)	1.48 (0.37, 5.18)	0.90 (0.26, 3.12)	**3.43 (1.49, 8.64)**	**7.20 (2.87, 21.15)**	**3.34 (1.62, 7.06)**	1.80 (0.73, 4.30)	**2.90 (1.33, 6.59)**	0.60 (0.04, 7.68)	3.74 (0.70, 27.43)	2.01 (0.32, 17.23)	1.39 (0.75, 2.66)	0.89 (0.25, 3.15)	CER200mgSC	0.50 (0.10, 1.96)	1.50 (0.88, 2.74)	1.27 (0.44, 3.85)	0.61 (0.11, 2.74)	1.41 (0.70, 2.95)	**2.27 (1.22, 4.64)**	**3.57 (1.63, 8.51)**
CER20mg_kgIV	1.49 (0.27, 9.27)	2.90 (0.54, 19.92)	1.82 (0.34, 11.47)	**6.87 (1.71, 37.44)**	**14.52 (3.59, 80.63)**	**6.66 (1.93, 31.49)**	3.53 (0.91, 17.50)	**5.75 (1.64, 26.94)**	1.24 (0.07, 25.00)	7.72 (0.97, 78.59)	4.18 (0.45, 49.84)	2.75 (0.75, 13.12)	1.79 (0.46, 8.45)	2.01 (0.51, 9.99)	CER20mg_kgIV	3.01 (0.91, 13.29)	2.51 (0.82, 10.60)	1.26 (0.17, 8.32)	2.76 (0.77, 13.01)	**4.58 (1.32, 21.47)**	**7.16 (1.86, 36.50)**
CER400mgSC	0.50 (0.12, 1.41)	0.99 (0.28, 2.95)	0.58 (0.20, 1.86)	**2.28 (1.14, 4.83)**	**4.71 (2.19, 11.93)**	**2.21 (1.36, 3.69)**	1.19 (0.58, 2.37)	**1.92 (1.07, 3.38)**	0.39 (0.03, 4.52)	2.49 (0.48, 17.79)	1.34 (0.22, 11.06)	0.92 (0.56, 1.49)	0.61 (0.18, 1.69)	0.67 (0.37, 1.13)	0.33 (0.08, 1.09)	CER400mgSC	0.85 (0.33, 2.23)	0.41 (0.08, 1.68)	0.93 (0.58, 1.49)	**1.52 (1.03, 2.28)**	**2.36 (1.26, 4.79)**
CER5mg_kgIV	0.60 (0.12, 2.26)	1.12 (0.27, 4.58)	0.71 (0.17, 2.92)	2.68 (0.89, 8.50)	**5.60 (1.67, 19.78)**	2.59 (0.96, 7.05)	1.38 (0.47, 4.26)	2.28 (0.80, 6.27)	0.47 (0.04, 7.29)	3.04 (0.45, 22.98)	1.57 (0.19, 14.91)	1.08 (0.39, 2.97)	0.72 (0.21, 1.87)	0.79 (0.26, 2.27)	0.40 (0.09, 1.22)	1.18 (0.45, 3.02)	CER5mg_kgIV	0.48 (0.08, 2.54)	1.10 (0.41, 2.97)	1.81 (0.67, 4.66)	2.74 (0.97, 8.55)
ETA25mgSC	1.22 (0.17, 8.16)	2.39 (0.38, 15.35)	1.49 (0.22, 9.04)	**5.70 (1.21, 33.37)**	**11.71 (2.47, 69.32)**	**5.53 (1.30, 28.73)**	2.91 (0.63, 17.13)	**4.70 (1.06, 25.85)**	1.01 (0.07, 17.94)	6.45 (0.73, 76.35)	3.38 (0.32, 46.47)	2.26 (0.53, 12.00)	1.51 (0.24, 9.51)	1.64 (0.36, 8.95)	0.79 (0.12, 5.73)	2.44 (0.59, 12.13)	2.10 (0.39, 12.90)	ETA25mgSC	2.29 (0.52, 12.16)	3.78 (0.88, 19.40)	**5.90 (1.24, 33.46)**
FIL100mgPO	0.54 (0.13, 1.64)	1.06 (0.27, 3.52)	0.63 (0.20, 2.07)	**2.44 (1.09, 5.81)**	**5.09 (2.10, 13.66)**	**2.38 (1.32, 4.46)**	1.28 (0.57, 2.80)	**2.07 (1.04, 4.13)**	0.41 (0.03, 5.32)	2.74 (0.50, 18.56)	1.42 (0.23, 12.29)	1.00 (0.52, 1.89)	0.66 (0.17, 2.01)	0.71 (0.34, 1.44)	0.36 (0.08, 1.30)	1.08 (0.67, 1.73)	0.91 (0.34, 2.44)	0.44 (0.08, 1.93)	FIL100mgPO	**1.63 (1.11, 2.44)**	**2.54 (1.19, 5.80)**
FIL200mgPO	**0.33 (0.08, 0.97)**	0.65 (0.18, 2.06)	0.38 (0.12, 1.27)	1.49 (0.72, 3.32)	**3.10 (1.37, 8.22)**	1.45 (0.85, 2.57)	0.78 (0.36, 1.63)	1.26 (0.66, 2.33)	0.25 (0.02, 2.87)	1.66 (0.31, 11.47)	0.87 (0.14, 7.51)	0.60 (0.33, 1.09)	0.40 (0.11, 1.15)	**0.44 (0.22, 0.82)**	**0.22 (0.05, 0.76)**	**0.66 (0.44, 0.97)**	0.55 (0.21, 1.49)	0.26 (0.05, 1.14)	**0.61 (0.41, 0.90)**	FIL200mgPO	1.54 (0.77, 3.37)
GUS1200mgIV	**0.21 (0.05, 0.69)**	0.40 (0.10, 1.47)	**0.24 (0.07, 0.86)**	0.97 (0.60, 1.57)	1.98 (0.77, 5.92)	0.93 (0.41, 1.97)	0.50 (0.18, 1.25)	0.82 (0.34, 1.78)	0.17 (0.01, 2.10)	1.04 (0.19, 8.41)	0.55 (0.09, 5.14)	**0.39 (0.17, 0.84)**	**0.26 (0.07, 0.82)**	**0.28 (0.12, 0.61)**	**0.14 (0.03, 0.54)**	**0.42 (0.21, 0.80)**	0.37 (0.12, 1.03)	**0.17 (0.03, 0.80)**	**0.39 (0.17, 0.84)**	0.65 (0.30, 1.30)	GUS1200mgIV

(**TABLE S18F.** continued)

	ABA10mg_kgIV	ABA30mg_kgIV	ABA3mg_kgIV	ADA160mg80mg40mgSC	ADA160mg80mg60mgSC	ADA160mg80mgSC	ADA40mg20mgSC	ADA80mg40mgSC	BRO210mgIV	BRO350mgIV	BRO700mgIV	CER100mgSC	CER10mg_kgIV	CER200mgSC	CER20mg_kgIV	CER400mgSC	CER5mg_kgIV	ETA25mgSC	FIL100mgPO	FIL200mgPO	GUS1200mgIV
GUS200mgIV	**0.17 (0.04, 0.55)**	0.33 (0.08, 1.17)	**0.19 (0.06, 0.68)**	0.77 (0.50, 1.19)	1.60 (0.62, 4.57)	0.75 (0.34, 1.54)	**0.40 (0.15, 0.99)**	0.65 (0.28, 1.42)	0.13 (0.01, 1.68)	0.83 (0.15, 6.58)	0.45 (0.07, 3.92)	**0.31 (0.14, 0.66)**	**0.20 (0.05, 0.65)**	**0.22 (0.09, 0.49)**	**0.11 (0.02, 0.42)**	**0.34 (0.17, 0.62)**	**0.29 (0.09, 0.81)**	**0.14 (0.02, 0.61)**	**0.31 (0.14, 0.65)**	0.51 (0.24, 1.01)	0.80 (0.54, 1.15)
GUS600mgIV	**0.17 (0.04, 0.57)**	0.33 (0.08, 1.17)	**0.20 (0.06, 0.71)**	0.79 (0.51, 1.24)	1.64 (0.63, 4.65)	0.77 (0.35, 1.60)	0.41 (0.15, 1.01)	0.66 (0.28, 1.46)	0.14 (0.01, 1.68)	0.85 (0.15, 6.71)	0.46 (0.07, 3.91)	**0.32 (0.14, 0.67)**	**0.21 (0.06, 0.66)**	**0.23 (0.09, 0.51)**	**0.11 (0.02, 0.43)**	**0.35 (0.17, 0.65)**	**0.30 (0.10, 0.85)**	**0.14 (0.03, 0.64)**	**0.32 (0.14, 0.67)**	0.53 (0.25, 1.07)	0.82 (0.55, 1.18)
INF10mg_kgIV	**0.07 (0.01, 0.63)**	0.13 (0.01, 1.31)	**0.08 (0.01, 0.83)**	0.32 (0.04, 2.69)	0.70 (0.07, 5.57)	0.32 (0.04, 2.40)	0.17 (0.02, 1.40)	0.26 (0.03, 2.20)	0.06 (0.00, 1.35)	0.36 (0.02, 5.11)	0.18 (0.01, 3.18)	**0.13 (0.01, 0.95)**	**0.08 (0.01, 0.79)**	**0.09 (0.01, 0.74)**	**0.05 (0.00, 0.45)**	0.14 (0.02, 1.06)	0.12 (0.01, 1.05)	**0.06 (0.00, 0.59)**	0.13 (0.02, 1.01)	0.22 (0.03, 1.65)	0.34 (0.04, 2.77)
INF20mg_kgIV	**0.07 (0.01, 0.60)**	0.14 (0.01, 1.16)	**0.08 (0.01, 0.70)**	0.35 (0.03, 2.37)	0.72 (0.08, 4.93)	0.34 (0.04, 2.03)	0.17 (0.02, 1.17)	0.28 (0.03, 1.84)	0.06 (0.00, 1.44)	0.36 (0.02, 4.74)	0.18 (0.01, 3.18)	**0.14 (0.01, 0.81)**	**0.08 (0.01, 0.68)**	**0.10 (0.01, 0.60)**	**0.05 (0.00, 0.40)**	**0.15 (0.02, 0.82)**	**0.13 (0.01, 0.91)**	**0.06 (0.00, 0.56)**	**0.14 (0.01, 0.85)**	0.23 (0.03, 1.32)	0.35 (0.04, 2.33)
INF5mg_kgIV	**0.03 (0.00, 0.27)**	**0.07 (0.01, 0.59)**	**0.04 (0.00, 0.35)**	0.17 (0.02, 1.15)	0.37 (0.04, 2.31)	**0.17 (0.02, 0.95)**	**0.09 (0.01, 0.55)**	**0.14 (0.02, 0.90)**	**0.03 (0.00, 0.60)**	0.18 (0.01, 2.90)	0.09 (0.01, 1.90)	**0.07 (0.01, 0.41)**	**0.04 (0.00, 0.35)**	**0.05 (0.01, 0.29)**	**0.02 (0.00, 0.21)**	**0.07 (0.01, 0.42)**	**0.06 (0.01, 0.46)**	**0.03 (0.00, 0.25)**	**0.07 (0.01, 0.40)**	**0.11 (0.01, 0.64)**	0.18 (0.02, 1.17)
MIR1000mgIV	**0.21 (0.04, 0.78)**	0.41 (0.10, 1.51)	0.25 (0.06, 1.01)	0.98 (0.33, 2.72)	2.00 (0.63, 7.03)	0.94 (0.35, 2.47)	0.49 (0.16, 1.56)	0.81 (0.29, 2.22)	0.16 (0.01, 2.97)	1.02 (0.18, 10.91)	0.55 (0.08, 6.23)	0.39 (0.15, 1.02)	0.25 (0.06, 1.04)	**0.28 (0.09, 0.76)**	**0.14 (0.02, 0.61)**	0.42 (0.17, 1.05)	0.36 (0.11, 1.26)	**0.17 (0.03, 0.95)**	0.39 (0.14, 1.07)	0.65 (0.25, 1.70)	1.01 (0.35, 2.87)
MIR200mgIV	0.36 (0.07, 1.75)	0.71 (0.14, 3.78)	0.44 (0.09, 2.30)	1.72 (0.45, 6.33)	3.59 (0.87, 17.57)	1.65 (0.48, 5.57)	0.87 (0.23, 3.40)	1.41 (0.41, 5.06)	0.28 (0.02, 4.74)	1.78 (0.26, 19.26)	0.95 (0.12, 11.72)	0.69 (0.20, 2.29)	0.44 (0.09, 1.95)	0.49 (0.14, 1.85)	0.25 (0.04, 1.24)	0.74 (0.23, 2.54)	0.64 (0.16, 2.53)	0.30 (0.04, 1.86)	0.68 (0.21, 2.45)	1.14 (0.34, 3.90)	1.75 (0.50, 6.32)
MIR600mgIV	**0.13 (0.03, 0.57)**	0.27 (0.06, 1.09)	**0.17 (0.04, 0.70)**	0.65 (0.21, 2.01)	1.32 (0.42, 4.87)	0.62 (0.23, 1.73)	0.33 (0.11, 1.10)	0.54 (0.19, 1.55)	0.11 (0.01, 1.79)	0.69 (0.11, 6.02)	0.36 (0.05, 3.62)	**0.26 (0.10, 0.77)**	**0.17 (0.04, 0.63)**	**0.19 (0.06, 0.54)**	**0.09 (0.02, 0.42)**	**0.28 (0.11, 0.75)**	**0.24 (0.07, 0.80)**	**0.11 (0.02, 0.58)**	**0.26 (0.10, 0.73)**	0.43 (0.16, 1.16)	0.67 (0.23, 2.03)
NAT300mgIV	0.43 (0.11, 1.25)	0.86 (0.25, 2.57)	0.52 (0.17, 1.60)	**2.01 (1.02, 4.22)**	**4.13 (1.95, 10.42)**	**1.93 (1.24, 3.25)**	1.04 (0.52, 2.09)	1.69 (0.93, 2.97)	0.34 (0.03, 3.94)	2.19 (0.44, 16.02)	1.18 (0.20, 9.62)	0.81 (0.47, 1.38)	0.54 (0.16, 1.52)	0.59 (0.31, 1.04)	**0.29 (0.07, 0.96)**	0.88 (0.65, 1.18)	0.75 (0.30, 1.92)	0.36 (0.07, 1.49)	0.82 (0.51, 1.32)	1.35 (0.91, 1.99)	**2.07 (1.12, 4.21)**
NAT3mg_kgIV	0.59 (0.14, 1.96)	1.15 (0.31, 3.87)	0.70 (0.22, 2.48)	**2.75 (1.14, 6.62)**	**5.67 (2.18, 15.67)**	**2.63 (1.30, 5.59)**	1.42 (0.56, 3.41)	**2.32 (1.03, 5.04)**	0.46 (0.04, 5.56)	2.99 (0.53, 23.55)	1.59 (0.25, 13.44)	1.10 (0.51, 2.25)	0.73 (0.19, 2.40)	0.79 (0.35, 1.69)	0.40 (0.08, 1.42)	1.20 (0.65, 2.12)	1.02 (0.35, 2.95)	0.49 (0.09, 2.18)	1.11 (0.55, 2.21)	1.83 (0.96, 3.39)	**2.79 (1.25, 6.61)**
NAT3mg_kgIVx2	0.39 (0.09, 1.22)	0.76 (0.20, 2.49)	0.46 (0.14, 1.53)	1.78 (0.77, 4.21)	**3.66 (1.44, 10.21)**	1.72 (0.88, 3.43)	0.92 (0.38, 2.15)	1.50 (0.70, 3.07)	0.30 (0.02, 3.65)	1.92 (0.37, 15.32)	1.02 (0.16, 8.77)	0.72 (0.34, 1.44)	0.47 (0.13, 1.59)	0.51 (0.23, 1.08)	**0.26 (0.05, 0.90)**	0.78 (0.44, 1.32)	0.66 (0.24, 1.76)	0.32 (0.06, 1.39)	0.72 (0.36, 1.39)	1.18 (0.64, 2.13)	1.83 (0.85, 4.13)
NAT6mg_kgIVx2	0.41 (0.10, 1.38)	0.82 (0.23, 2.87)	0.50 (0.15, 1.64)	1.95 (0.83, 4.62)	**3.99 (1.57, 11.59)**	1.88 (0.92, 3.92)	1.01 (0.41, 2.41)	1.64 (0.74, 3.54)	0.33 (0.03, 4.40)	2.10 (0.39, 16.51)	1.13 (0.18, 10.10)	0.79 (0.36, 1.65)	0.52 (0.14, 1.64)	0.56 (0.25, 1.23)	**0.28 (0.06, 0.99)**	0.86 (0.47, 1.53)	0.73 (0.25, 2.00)	0.35 (0.06, 1.54)	0.79 (0.40, 1.58)	1.30 (0.68, 2.43)	2.03 (0.92, 4.63)
PBO	0.61 (0.15, 1.71)	1.20 (0.35, 3.66)	0.72 (0.24, 2.20)	**2.79 (1.45, 5.74)**	**5.76 (2.78, 14.51)**	**2.69 (1.78, 4.41)**	1.45 (0.74, 2.82)	**2.35 (1.35, 3.95)**	0.47 (0.04, 5.51)	3.06 (0.62, 21.75)	1.63 (0.28, 13.12)	1.13 (0.68, 1.84)	0.75 (0.22, 2.04)	0.82 (0.44, 1.40)	0.41 (0.10, 1.30)	1.23 (0.98, 1.52)	1.04 (0.43, 2.60)	0.50 (0.10, 2.01)	1.13 (0.75, 1.75)	**1.87 (1.34, 2.64)**	**2.88 (1.62, 5.67)**
RIS1200mgIV	**0.34 (0.09, 0.98)**	0.68 (0.19, 2.03)	0.40 (0.14, 1.24)	1.58 (0.78, 3.29)	**3.24 (1.53, 8.43)**	1.52 (0.96, 2.52)	0.82 (0.40, 1.65)	1.32 (0.74, 2.32)	0.26 (0.02, 3.11)	1.73 (0.34, 12.15)	0.91 (0.16, 7.88)	0.64 (0.37, 1.07)	0.42 (0.12, 1.18)	**0.46 (0.24, 0.82)**	**0.23 (0.05, 0.75)**	**0.69 (0.51, 0.93)**	0.58 (0.24, 1.48)	0.28 (0.06, 1.15)	0.64 (0.40, 1.03)	1.05 (0.71, 1.56)	1.63 (0.87, 3.33)
RIS200mgIV	0.46 (0.11, 1.62)	0.91 (0.23, 3.33)	0.55 (0.16, 2.05)	2.15 (0.83, 5.88)	**4.40 (1.62, 14.06)**	2.05 (0.97, 4.82)	1.08 (0.44, 3.03)	1.79 (0.79, 4.47)	0.36 (0.03, 4.94)	2.40 (0.43, 17.49)	1.26 (0.18, 11.77)	0.87 (0.37, 2.05)	0.57 (0.14, 1.94)	0.62 (0.25, 1.54)	0.30 (0.06, 1.23)	0.94 (0.47, 1.94)	0.79 (0.27, 2.59)	0.38 (0.07, 1.91)	0.87 (0.41, 2.03)	1.43 (0.69, 3.13)	2.20 (0.90, 6.05)
RIS600mgIV	**0.31 (0.08, 0.90)**	0.63 (0.18, 1.90)	0.37 (0.13, 1.13)	1.45 (0.71, 3.05)	**2.99 (1.40, 7.67)**	1.40 (0.88, 2.31)	0.76 (0.37, 1.50)	1.21 (0.68, 2.16)	0.24 (0.02, 2.92)	1.60 (0.32, 11.20)	0.84 (0.15, 7.20)	**0.59 (0.34, 0.97)**	0.39 (0.11, 1.07)	**0.43 (0.22, 0.75)**	**0.21 (0.05, 0.69)**	**0.63 (0.47, 0.85)**	0.54 (0.22, 1.35)	0.26 (0.05, 1.05)	**0.59 (0.37, 0.95)**	0.97 (0.66, 1.44)	1.50 (0.80, 3.04)
RIS600mgIV4_4	0.43 (0.10, 1.30)	0.86 (0.24, 2.65)	0.51 (0.16, 1.62)	1.99 (0.96, 4.37)	**4.12 (1.83, 10.93)**	**1.93 (1.13, 3.39)**	1.04 (0.49, 2.20)	1.69 (0.88, 3.12)	0.33 (0.03, 4.04)	2.19 (0.41, 16.32)	1.15 (0.19, 9.90)	0.81 (0.43, 1.45)	0.53 (0.15, 1.57)	0.59 (0.29, 1.10)	**0.29 (0.06, 0.97)**	0.88 (0.57, 1.31)	0.74 (0.28, 1.92)	0.36 (0.07, 1.49)	0.81 (0.47, 1.43)	1.34 (0.82, 2.15)	**2.06 (1.05, 4.39)**
SEC10mg_kgIV	0.87 (0.10, 5.14)	1.70 (0.22, 8.73)	1.02 (0.14, 6.33)	4.10 (0.69, 21.08)	**8.39 (1.32, 46.82)**	3.99 (0.65, 18.09)	2.13 (0.30, 9.98)	3.45 (0.54, 15.71)	0.71 (0.03, 14.42)	4.30 (0.48, 42.74)	2.38 (0.20, 24.99)	1.63 (0.26, 7.68)	1.05 (0.13, 5.97)	1.16 (0.20, 5.84)	0.60 (0.06, 3.65)	1.81 (0.31, 7.65)	1.49 (0.20, 8.54)	0.67 (0.07, 6.03)	1.68 (0.28, 7.43)	2.73 (0.48, 11.75)	4.26 (0.72, 21.63)
TOF15mgPO_BID	0.90 (0.14, 3.97)	1.78 (0.33, 9.27)	1.08 (0.22, 5.43)	**4.19 (1.19, 15.11)**	**8.47 (2.31, 39.63)**	**4.03 (1.24, 13.72)**	2.18 (0.60, 7.66)	**3.52 (1.04, 12.16)**	0.73 (0.04, 10.72)	4.42 (0.65, 47.62)	2.49 (0.28, 29.87)	1.70 (0.51, 5.86)	1.08 (0.20, 5.58)	1.19 (0.36, 4.24)	0.61 (0.10, 2.89)	1.81 (0.62, 5.60)	1.55 (0.38, 6.37)	0.75 (0.11, 4.15)	1.71 (0.52, 5.30)	2.77 (0.90, 8.67)	**4.35 (1.23, 15.62)**
TOF1mgPO_BID	0.40 (0.07, 1.56)	0.80 (0.16, 3.22)	0.48 (0.11, 2.02)	1.87 (0.60, 5.58)	**3.76 (1.20, 13.79)**	1.81 (0.64, 4.99)	0.95 (0.33, 2.93)	1.57 (0.52, 4.28)	0.33 (0.02, 4.60)	2.01 (0.29, 17.26)	1.09 (0.14, 9.56)	0.74 (0.26, 2.10)	0.48 (0.10, 2.08)	0.54 (0.19, 1.53)	0.27 (0.05, 1.14)	0.81 (0.31, 2.08)	0.68 (0.19, 2.54)	0.32 (0.05, 1.74)	0.75 (0.28, 1.92)	1.24 (0.48, 3.14)	1.92 (0.62, 6.03)
TOF5mgPO_BID	0.54 (0.09, 2.32)	1.05 (0.21, 4.78)	0.65 (0.14, 3.11)	2.49 (0.74, 8.66)	**5.13 (1.49, 21.26)**	2.41 (0.81, 6.98)	1.29 (0.40, 3.94)	2.09 (0.67, 6.34)	0.43 (0.03, 7.46)	2.75 (0.39, 25.15)	1.46 (0.16, 14.86)	1.00 (0.32, 3.05)	0.64 (0.13, 2.79)	0.72 (0.23, 2.23)	0.36 (0.06, 1.52)	1.09 (0.39, 2.86)	0.92 (0.25, 3.37)	0.44 (0.07, 2.45)	1.02 (0.36, 2.71)	1.67 (0.60, 4.51)	2.58 (0.78, 8.28)
UPA12mgPO_BID	0.48 (0.06, 2.39)	0.93 (0.14, 5.20)	0.56 (0.10, 3.00)	2.22 (0.49, 9.05)	4.48 (0.99, 21.05)	2.13 (0.54, 8.03)	1.12 (0.27, 4.77)	1.85 (0.45, 7.17)	0.37 (0.02, 6.19)	2.43 (0.28, 24.77)	1.24 (0.15, 14.70)	0.89 (0.21, 3.26)	0.57 (0.09, 3.31)	0.62 (0.15, 2.62)	0.31 (0.05, 1.86)	0.96 (0.24, 3.46)	0.80 (0.14, 3.89)	0.38 (0.05, 2.38)	0.90 (0.22, 3.35)	1.45 (0.36, 5.36)	2.24 (0.52, 9.52)

(TABLE S18F. continued)

	ABA10mg_kgIV	ABA30mg_kgIV	ABA3mg_kgIV	ADA160mg80mg40mgSC	ADA160mg80mg60mgSC	ADA160mg80mgSC	ADA40mg20mgSC	ADA80mg40mgSC	BRO210mgIV	BRO350mgIV	BRO700mgIV	CER100mgSC	CER10mg_kgIV	CER200mgSC	CER20mg_kgIV	CER400mgSC	CER5mg_kgIV	ETA25mgSC	FIL100mgPO	FIL200mgPO	GUS1200mgIV
UPA24mgPO	0.90 (0.12, 6.54)	1.81 (0.22, 18.56)	1.06 (0.16, 10.12)	4.16 (0.79, 28.82)	**8.43 (1.64, 75.90)**	3.92 (0.77, 32.13)	2.09 (0.37, 17.42)	3.44 (0.64, 30.81)	0.67 (0.04, 18.72)	4.50 (0.50, 102.76)	2.38 (0.23, 49.75)	1.65 (0.32, 12.61)	1.09 (0.15, 9.90)	1.20 (0.24, 8.63)	0.57 (0.08, 6.24)	1.79 (0.38, 12.75)	1.58 (0.26, 12.32)	0.74 (0.08, 7.62)	1.68 (0.33, 11.91)	2.72 (0.59, 17.78)	4.26 (0.84, 33.66)
UPA24mgPO_BID	0.25 (0.04, 1.12)	0.51 (0.09, 2.43)	0.30 (0.06, 1.51)	1.19 (0.30, 4.33)	2.46 (0.60, 9.85)	1.18 (0.30, 3.75)	0.62 (0.14, 2.20)	1.02 (0.25, 3.27)	0.20 (0.01, 3.18)	1.29 (0.18, 12.11)	0.68 (0.10, 7.71)	0.49 (0.13, 1.57)	0.32 (0.05, 1.46)	0.35 (0.09, 1.20)	**0.17 (0.03, 0.86)**	0.54 (0.14, 1.52)	0.45 (0.09, 1.78)	0.21 (0.03, 1.21)	0.50 (0.13, 1.53)	0.81 (0.22, 2.44)	1.24 (0.30, 4.54)
UPA3mgPO_BID	0.66 (0.11, 4.58)	1.39 (0.22, 9.68)	0.81 (0.15, 5.94)	3.21 (0.74, 16.52)	**6.73 (1.42, 35.55)**	3.11 (0.69, 16.15)	1.68 (0.32, 9.20)	2.68 (0.60, 14.08)	0.55 (0.03, 7.53)	3.66 (0.41, 32.59)	1.89 (0.21, 21.17)	1.31 (0.30, 6.38)	0.87 (0.13, 5.53)	0.93 (0.21, 4.76)	0.45 (0.07, 3.32)	1.45 (0.33, 6.63)	1.21 (0.23, 6.69)	0.56 (0.07, 4.24)	1.33 (0.30, 6.54)	2.18 (0.50, 10.53)	3.22 (0.76, 16.98)
UPA45mgPO	0.35 (0.08, 1.01)	0.69 (0.19, 2.13)	0.41 (0.14, 1.32)	1.61 (0.79, 3.36)	**3.30 (1.53, 8.58)**	1.55 (0.96, 2.61)	0.83 (0.41, 1.67)	1.35 (0.76, 2.39)	0.27 (0.02, 3.18)	1.76 (0.35, 12.66)	0.94 (0.16, 7.53)	0.65 (0.38, 1.11)	0.43 (0.13, 1.21)	**0.47 (0.25, 0.83)**	**0.24 (0.05, 0.77)**	**0.70 (0.52, 0.95)**	0.60 (0.24, 1.56)	0.29 (0.06, 1.16)	0.65 (0.40, 1.07)	1.07 (0.71, 1.62)	1.66 (0.89, 3.40)
UPA6mgPO_BID	**0.21 (0.03, 0.89)**	0.42 (0.07, 2.04)	0.25 (0.05, 1.21)	1.01 (0.25, 3.38)	2.06 (0.50, 7.70)	0.97 (0.23, 2.85)	0.51 (0.12, 1.71)	0.84 (0.21, 2.59)	0.16 (0.01, 2.32)	1.08 (0.15, 9.51)	0.58 (0.07, 6.56)	0.41 (0.11, 1.20)	0.27 (0.05, 1.16)	**0.29 (0.07, 0.90)**	**0.14 (0.02, 0.71)**	0.45 (0.12, 1.16)	0.38 (0.08, 1.44)	**0.18 (0.03, 0.95)**	0.42 (0.11, 1.13)	0.69 (0.18, 1.82)	1.05 (0.26, 3.50)
UST130mgIV	0.36 (0.09, 1.07)	0.71 (0.20, 2.18)	0.42 (0.14, 1.40)	1.66 (0.79, 3.49)	**3.40 (1.53, 8.84)**	1.59 (0.95, 2.78)	0.86 (0.40, 1.76)	1.39 (0.75, 2.54)	0.28 (0.02, 3.10)	1.82 (0.34, 12.70)	0.96 (0.16, 7.89)	0.67 (0.37, 1.18)	0.44 (0.13, 1.24)	**0.48 (0.25, 0.86)**	**0.24 (0.06, 0.81)**	0.73 (0.50, 1.02)	0.61 (0.24, 1.63)	0.29 (0.06, 1.23)	0.67 (0.40, 1.12)	1.10 (0.73, 1.73)	1.71 (0.86, 3.51)
UST1mg_kgIV	0.31 (0.07, 1.01)	0.62 (0.16, 2.06)	0.37 (0.11, 1.24)	1.45 (0.60, 3.50)	**3.00 (1.18, 8.69)**	1.40 (0.70, 2.80)	0.74 (0.32, 1.79)	1.21 (0.58, 2.57)	0.24 (0.02, 3.11)	1.60 (0.30, 11.90)	0.82 (0.13, 7.47)	0.58 (0.28, 1.17)	0.38 (0.10, 1.18)	**0.42 (0.19, 0.88)**	**0.21 (0.04, 0.76)**	0.63 (0.36, 1.10)	0.54 (0.19, 1.52)	0.25 (0.05, 1.11)	0.58 (0.30, 1.17)	0.96 (0.52, 1.80)	1.48 (0.66, 3.50)
UST3mg_kgIV	**0.30 (0.07, 0.96)**	0.59 (0.15, 2.07)	0.35 (0.10, 1.22)	1.39 (0.58, 3.33)	**2.86 (1.17, 8.44)**	1.34 (0.68, 2.67)	0.72 (0.30, 1.69)	1.16 (0.56, 2.43)	0.23 (0.02, 3.25)	1.52 (0.28, 11.87)	0.79 (0.13, 7.07)	0.56 (0.27, 1.16)	0.37 (0.10, 1.15)	**0.40 (0.18, 0.88)**	**0.20 (0.04, 0.74)**	0.60 (0.35, 1.11)	0.52 (0.18, 1.42)	0.25 (0.05, 1.06)	0.57 (0.28, 1.09)	0.92 (0.50, 1.73)	1.43 (0.62, 3.30)
UST4_5mg_kgIV	0.35 (0.09, 1.04)	0.70 (0.19, 2.20)	0.42 (0.14, 1.33)	1.62 (0.76, 3.57)	**3.34 (1.50, 8.84)**	1.57 (0.93, 2.80)	0.84 (0.40, 1.78)	1.36 (0.73, 2.55)	0.27 (0.02, 3.27)	1.80 (0.34, 12.45)	0.94 (0.16, 8.15)	0.66 (0.36, 1.17)	0.44 (0.12, 1.21)	**0.48 (0.24, 0.88)**	**0.24 (0.05, 0.78)**	0.71 (0.49, 1.04)	0.61 (0.23, 1.58)	0.29 (0.06, 1.23)	0.66 (0.40, 1.13)	1.08 (0.70, 1.74)	1.69 (0.85, 3.58)
UST6mg_kg90mgIV_SC	**0.21 (0.04, 0.71)**	0.41 (0.10, 1.45)	**0.24 (0.07, 0.84)**	0.96 (0.76, 1.21)	1.99 (0.76, 5.77)	0.93 (0.41, 1.97)	0.50 (0.19, 1.25)	0.80 (0.34, 1.79)	0.16 (0.01, 2.03)	1.03 (0.19, 8.08)	0.56 (0.09, 4.62)	**0.39 (0.16, 0.84)**	**0.25 (0.07, 0.81)**	**0.28 (0.12, 0.62)**	**0.14 (0.03, 0.55)**	**0.42 (0.21, 0.80)**	0.36 (0.11, 1.03)	**0.17 (0.03, 0.77)**	**0.39 (0.17, 0.83)**	0.65 (0.30, 1.29)	1.00 (0.66, 1.49)
UST6mg_kgIV	**0.28 (0.07, 0.82)**	0.55 (0.15, 1.69)	0.33 (0.11, 1.03)	1.27 (0.61, 2.73)	**2.60 (1.20, 6.91)**	1.23 (0.75, 2.12)	0.66 (0.32, 1.34)	1.07 (0.59, 1.92)	0.21 (0.02, 2.43)	1.40 (0.27, 9.87)	0.74 (0.13, 6.15)	**0.51 (0.29, 0.90)**	**0.34 (0.10, 0.95)**	**0.37 (0.19, 0.66)**	**0.18 (0.04, 0.62)**	**0.56 (0.40, 0.77)**	0.47 (0.19, 1.20)	**0.23 (0.04, 0.92)**	**0.52 (0.32, 0.86)**	0.85 (0.57, 1.32)	1.32 (0.68, 2.71)
UST90mgSC	0.59 (0.11, 2.69)	1.20 (0.24, 5.54)	0.71 (0.16, 3.34)	2.83 (0.77, 9.63)	**5.71 (1.55, 23.55)**	2.73 (0.85, 8.03)	1.46 (0.40, 4.73)	2.37 (0.69, 7.31)	0.46 (0.04, 7.15)	3.18 (0.45, 26.91)	1.69 (0.19, 16.83)	1.15 (0.35, 3.49)	0.73 (0.15, 3.28)	0.81 (0.24, 2.55)	0.41 (0.06, 1.84)	1.24 (0.42, 3.49)	1.03 (0.26, 3.93)	0.49 (0.08, 2.84)	1.14 (0.38, 3.34)	1.91 (0.62, 5.37)	2.91 (0.80, 9.61)
VED0_5mg_kgIV	0.42 (0.09, 1.46)	0.84 (0.21, 2.85)	0.50 (0.14, 1.97)	1.99 (0.75, 5.34)	**4.13 (1.49, 12.02)**	1.91 (0.90, 4.09)	1.01 (0.40, 2.55)	1.67 (0.71, 3.72)	0.34 (0.03, 4.15)	2.15 (0.38, 14.58)	1.12 (0.18, 8.75)	0.80 (0.35, 1.79)	0.54 (0.13, 1.67)	0.57 (0.22, 1.32)	0.29 (0.06, 1.08)	0.86 (0.42, 1.66)	0.74 (0.23, 2.20)	0.35 (0.06, 1.64)	0.80 (0.37, 1.70)	1.32 (0.62, 2.70)	2.05 (0.81, 5.25)
VED2mg_kgIV	0.34 (0.08, 1.12)	0.66 (0.17, 2.24)	0.40 (0.11, 1.46)	1.57 (0.61, 4.03)	**3.19 (1.19, 9.47)**	1.50 (0.72, 3.04)	0.81 (0.33, 1.95)	1.30 (0.57, 2.85)	0.26 (0.02, 3.17)	1.70 (0.29, 12.58)	0.90 (0.14, 7.79)	0.63 (0.28, 1.35)	0.42 (0.11, 1.31)	0.45 (0.18, 1.00)	**0.23 (0.05, 0.81)**	0.68 (0.35, 1.26)	0.57 (0.20, 1.68)	0.27 (0.05, 1.30)	0.63 (0.30, 1.26)	1.04 (0.51, 2.00)	1.61 (0.67, 3.93)
VED300mgIV	**0.28 (0.07, 0.88)**	0.57 (0.15, 1.90)	0.34 (0.11, 1.13)	1.32 (0.63, 2.91)	**2.72 (1.20, 7.47)**	1.28 (0.76, 2.27)	0.69 (0.32, 1.46)	1.13 (0.59, 2.05)	0.23 (0.02, 2.85)	1.48 (0.29, 9.93)	0.78 (0.13, 6.72)	**0.54 (0.29, 0.95)**	0.36 (0.10, 1.02)	**0.39 (0.19, 0.74)**	**0.19 (0.04, 0.66)**	**0.58 (0.39, 0.88)**	0.49 (0.19, 1.30)	0.23 (0.05, 1.00)	**0.54 (0.32, 0.94)**	0.89 (0.55, 1.43)	1.37 (0.69, 2.94)

(TABLE S18F. continued)

	GUS200mgIV	GUS600mgIV	INF10mg_kgIV	INF20mg_kgIV	INF5mg_kgIV	MIR1000mgIV	MIR200mgIV	MIR600mgIV	NAT300mgIV	NAT3mg_kgIV	NAT3mg_kgIVx2	NAT6mg_kgIVx2	PBO	RIS1200mgIV	RIS200mgIV	RIS600mgIV	RIS600mgIV4_4	SEC10mg_kgIV	TOF15mgPO_BID	TOF1mgPO_BID	TOF5mgPO_BID	UPA12mgPO_BID
ABA10mg_kgIV	**5.99 (1.83, 27.92)**	**5.90 (1.74, 26.12)**	**14.55 (1.58, 157.98)**	**14.40 (1.68, 158.95)**	**29.61 (3.64, 288.42)**	**4.85 (1.28, 23.26)**	2.81 (0.57, 15.36)	**7.41 (1.76, 36.66)**	2.30 (0.80, 9.19)	1.70 (0.51, 7.27)	2.58 (0.82, 10.86)	2.42 (0.72, 10.13)	1.65 (0.58, 6.62)	**2.95 (1.02, 11.53)**	2.17 (0.62, 9.28)	**3.18 (1.11, 12.52)**	2.32 (0.77, 9.75)	1.15 (0.19, 9.87)	1.12 (0.25, 7.11)	2.48 (0.64, 14.49)	1.86 (0.43, 10.93)	2.10 (0.42, 16.44)
ABA30mg_kgIV	3.08 (0.86, 12.07)	2.99 (0.85, 11.98)	7.64 (0.76, 74.90)	7.14 (0.87, 76.87)	**14.64 (1.70, 144.04)**	2.43 (0.66, 10.48)	1.40 (0.26, 7.05)	3.71 (0.92, 15.81)	1.16 (0.39, 4.03)	0.87 (0.26, 3.28)	1.32 (0.40, 5.00)	1.23 (0.35, 4.39)	0.83 (0.27, 2.90)	1.47 (0.49, 5.24)	1.09 (0.30, 4.31)	1.60 (0.53, 5.64)	1.16 (0.38, 4.24)	0.59 (0.11, 4.55)	0.56 (0.11, 3.06)	1.25 (0.31, 6.08)	0.95 (0.21, 4.73)	1.08 (0.19, 7.31)
ABA3mg_kgIV	**5.14 (1.47, 17.80)**	**4.92 (1.41, 17.29)**	**12.63 (1.20, 129.50)**	**11.89 (1.42, 130.08)**	**23.89 (2.83, 241.62)**	4.06 (0.99, 15.71)	2.28 (0.43, 10.91)	**6.02 (1.43, 23.61)**	1.94 (0.63, 5.73)	1.42 (0.40, 4.63)	2.19 (0.65, 7.05)	1.99 (0.61, 6.57)	1.39 (0.45, 4.09)	2.49 (0.80, 7.32)	1.82 (0.49, 6.26)	2.69 (0.89, 7.99)	1.95 (0.62, 6.25)	0.98 (0.16, 7.01)	0.92 (0.18, 4.47)	2.07 (0.50, 8.97)	1.54 (0.32, 7.00)	1.78 (0.33, 9.58)
ADA160mg80mg40mgSC	1.30 (0.84, 2.01)	1.26 (0.81, 1.95)	3.08 (0.37, 27.37)	2.89 (0.42, 29.71)	5.81 (0.87, 55.44)	1.02 (0.37, 3.07)	0.58 (0.16, 2.21)	1.53 (0.50, 4.74)	**0.50 (0.24, 0.98)**	**0.36 (0.15, 0.88)**	0.56 (0.24, 1.30)	0.51 (0.22, 1.21)	**0.36 (0.17, 0.69)**	0.63 (0.30, 1.28)	0.47 (0.17, 1.21)	0.69 (0.33, 1.40)	0.50 (0.23, 1.05)	0.24 (0.05, 1.45)	**0.24 (0.07, 0.84)**	0.53 (0.18, 1.68)	0.40 (0.12, 1.35)	0.45 (0.11, 2.04)
ADA160mg80mg60mgSC	0.63 (0.22, 1.61)	0.61 (0.21, 1.59)	1.44 (0.18, 14.37)	1.40 (0.20, 13.31)	2.70 (0.43, 26.80)	0.50 (0.14, 1.59)	0.28 (0.06, 1.14)	0.76 (0.21, 2.39)	**0.24 (0.10, 0.51)**	**0.18 (0.06, 0.46)**	**0.27 (0.10, 0.69)**	**0.25 (0.09, 0.64)**	**0.17 (0.07, 0.36)**	**0.31 (0.12, 0.65)**	**0.23 (0.07, 0.62)**	**0.33 (0.13, 0.71)**	**0.24 (0.09, 0.55)**	**0.12 (0.02, 0.76)**	**0.12 (0.03, 0.43)**	**0.27 (0.07, 0.84)**	**0.19 (0.05, 0.67)**	0.22 (0.05, 1.01)
ADA160mg80mgSC	1.34 (0.65, 2.91)	1.31 (0.63, 2.88)	3.15 (0.42, 27.39)	2.96 (0.49, 27.07)	**6.00 (1.05, 52.46)**	1.06 (0.41, 2.89)	0.60 (0.18, 2.08)	1.61 (0.58, 4.38)	**0.52 (0.31, 0.81)**	**0.38 (0.18, 0.77)**	0.58 (0.29, 1.14)	0.53 (0.26, 1.08)	**0.37 (0.23, 0.56)**	0.66 (0.40, 1.05)	0.49 (0.21, 1.03)	0.71 (0.43, 1.14)	**0.52 (0.29, 0.89)**	0.25 (0.06, 1.54)	**0.25 (0.07, 0.81)**	0.55 (0.20, 1.56)	0.41 (0.14, 1.24)	0.47 (0.12, 1.85)
ADA40mg20mgSC	**2.50 (1.01, 6.71)**	2.42 (0.99, 6.58)	6.05 (0.72, 53.96)	5.77 (0.86, 54.63)	**11.25 (1.83, 104.47)**	2.03 (0.64, 6.07)	1.15 (0.29, 4.26)	3.04 (0.91, 9.42)	0.96 (0.48, 1.93)	0.70 (0.29, 1.79)	1.09 (0.47, 2.63)	0.99 (0.41, 2.47)	0.69 (0.35, 1.35)	1.22 (0.61, 2.52)	0.92 (0.33, 2.29)	1.32 (0.66, 2.73)	0.96 (0.45, 2.03)	0.47 (0.10, 3.32)	0.46 (0.13, 1.67)	1.05 (0.34, 3.04)	0.77 (0.25, 2.49)	0.89 (0.21, 3.75)
ADA80mg40mgSC	1.55 (0.70, 3.59)	1.51 (0.69, 3.53)	3.79 (0.45, 33.82)	3.53 (0.54, 34.06)	**7.12 (1.11, 63.71)**	1.23 (0.45, 3.42)	0.71 (0.20, 2.45)	1.86 (0.64, 5.26)	0.59 (0.34, 1.07)	**0.43 (0.20, 0.97)**	0.66 (0.33, 1.44)	0.61 (0.28, 1.34)	**0.43 (0.25, 0.74)**	0.76 (0.43, 1.35)	0.56 (0.22, 1.27)	0.82 (0.46, 1.48)	0.59 (0.32, 1.14)	0.29 (0.06, 1.86)	**0.28 (0.08, 0.96)**	0.64 (0.23, 1.92)	0.48 (0.16, 1.49)	0.54 (0.14, 2.22)
BRO210mgIV	7.65 (0.60, 105.44)	7.37 (0.59, 107.07)	17.47 (0.74, 444.24)	17.54 (0.70, 498.21)	**34.48 (1.67, 979.47)**	6.20 (0.34, 74.63)	3.58 (0.21, 47.66)	9.34 (0.56, 111.69)	2.92 (0.25, 35.23)	2.18 (0.18, 27.44)	3.35 (0.27, 42.29)	3.04 (0.23, 39.96)	2.11 (0.18, 25.37)	3.81 (0.32, 44.39)	2.77 (0.20, 31.14)	4.10 (0.34, 48.62)	3.00 (0.25, 37.44)	1.42 (0.07, 32.99)	1.38 (0.09, 23.14)	3.06 (0.22, 42.68)	2.33 (0.13, 32.80)	2.69 (0.16, 43.11)
BRO350mgIV	1.21 (0.15, 6.62)	1.17 (0.15, 6.52)	2.81 (0.20, 42.14)	2.76 (0.21, 41.20)	5.41 (0.34, 80.72)	0.98 (0.09, 5.70)	0.56 (0.05, 3.79)	1.45 (0.17, 9.13)	0.46 (0.06, 2.29)	0.33 (0.04, 1.89)	0.52 (0.07, 2.70)	0.48 (0.06, 2.57)	0.33 (0.05, 1.62)	0.58 (0.08, 2.93)	0.42 (0.06, 2.33)	0.63 (0.09, 3.16)	0.46 (0.06, 2.43)	0.23 (0.02, 2.08)	0.23 (0.02, 1.54)	0.50 (0.06, 3.48)	0.36 (0.04, 2.56)	0.41 (0.04, 3.60)
BRO700mgIV	2.24 (0.26, 14.10)	2.19 (0.26, 13.85)	5.48 (0.31, 92.04)	5.51 (0.31, 87.57)	11.20 (0.53, 169.13)	1.82 (0.16, 12.31)	1.05 (0.09, 8.42)	2.75 (0.28, 19.00)	0.85 (0.10, 4.94)	0.63 (0.07, 3.95)	0.98 (0.11, 6.13)	0.89 (0.10, 5.69)	0.61 (0.08, 3.53)	1.10 (0.13, 6.19)	0.80 (0.08, 5.60)	1.19 (0.14, 6.77)	0.87 (0.10, 5.18)	0.42 (0.04, 5.04)	0.40 (0.03, 3.62)	0.92 (0.10, 7.27)	0.68 (0.07, 6.11)	0.80 (0.07, 6.85)
CER100mgSC	**3.24 (1.51, 7.25)**	**3.14 (1.49, 7.25)**	**7.60 (1.05, 69.05)**	**7.33 (1.24, 68.37)**	**14.84 (2.45, 131.85)**	2.56 (0.98, 6.86)	1.45 (0.44, 5.10)	**3.89 (1.31, 10.50)**	1.23 (0.73, 2.12)	0.91 (0.44, 1.97)	1.39 (0.69, 2.96)	1.27 (0.60, 2.76)	0.88 (0.54, 1.48)	1.56 (0.94, 2.72)	1.14 (0.49, 2.73)	**1.70 (1.03, 2.97)**	1.24 (0.69, 2.32)	0.61 (0.13, 3.78)	0.59 (0.17, 1.95)	1.35 (0.48, 3.88)	1.00 (0.33, 3.08)	1.13 (0.31, 4.87)
CER10mg_kgIV	**4.90 (1.55, 18.72)**	**4.81 (1.51, 17.53)**	**11.94 (1.27, 114.03)**	**11.99 (1.47, 120.68)**	**22.78 (2.85, 224.32)**	3.98 (0.96, 17.72)	2.27 (0.51, 10.97)	**5.93 (1.58, 26.15)**	1.85 (0.66, 6.35)	1.38 (0.42, 5.28)	2.13 (0.63, 7.76)	1.93 (0.61, 7.08)	1.33 (0.49, 4.59)	2.38 (0.84, 8.39)	1.75 (0.52, 7.09)	2.56 (0.93, 9.22)	1.88 (0.64, 6.61)	0.96 (0.17, 7.43)	0.93 (0.18, 5.09)	2.09 (0.48, 10.26)	1.57 (0.36, 7.74)	1.77 (0.30, 10.94)
CER200mgSC	**4.50 (2.03, 10.77)**	**4.34 (1.97, 10.57)**	**10.97 (1.35, 93.20)**	**10.27 (1.66, 97.22)**	**20.63 (3.50, 184.73)**	**3.55 (1.31, 10.77)**	2.04 (0.54, 7.26)	**5.40 (1.85, 15.89)**	1.70 (0.97, 3.23)	1.26 (0.59, 2.85)	1.94 (0.93, 4.31)	1.78 (0.81, 3.99)	1.22 (0.72, 2.25)	**2.17 (1.23, 4.16)**	1.62 (0.65, 4.03)	**2.35 (1.33, 4.53)**	1.71 (0.91, 3.48)	0.86 (0.17, 5.09)	0.84 (0.24, 2.79)	1.85 (0.65, 5.33)	1.39 (0.45, 4.32)	1.62 (0.38, 6.56)
CER20mg_kgIV	**9.00 (2.37, 46.18)**	**8.80 (2.31, 44.89)**	**21.00 (2.24, 361.35)**	**20.55 (2.49, 283.68)**	**41.32 (4.84, 627.94)**	**7.11 (1.65, 41.62)**	4.08 (0.81, 27.76)	**10.74 (2.37, 60.68)**	**3.43 (1.04, 14.37)**	2.53 (0.70, 12.73)	**3.85 (1.12, 19.06)**	**3.54 (1.01, 17.18)**	2.45 (0.77, 10.34)	**4.30 (1.33, 19.30)**	3.29 (0.81, 16.12)	**4.68 (1.45, 20.77)**	**3.41 (1.03, 15.93)**	1.66 (0.27, 16.31)	1.64 (0.35, 10.40)	3.68 (0.88, 21.04)	2.77 (0.66, 15.66)	3.27 (0.54, 21.64)
CER400mgSC	**2.96 (1.61, 5.99)**	**2.87 (1.54, 5.89)**	7.07 (0.94, 59.53)	**6.65 (1.22, 60.21)**	**13.40 (2.39, 118.25)**	2.36 (0.95, 6.01)	1.35 (0.39, 4.28)	**3.58 (1.34, 9.05)**	1.13 (0.85, 1.55)	0.83 (0.47, 1.55)	1.28 (0.76, 2.29)	1.17 (0.65, 2.15)	0.82 (0.66, 1.02)	**1.45 (1.08, 1.95)**	1.07 (0.51, 2.11)	**1.58 (1.18, 2.11)**	1.14 (0.76, 1.74)	0.55 (0.13, 3.21)	0.55 (0.18, 1.63)	1.23 (0.48, 3.22)	0.91 (0.35, 2.58)	1.05 (0.29, 4.20)
CER5mg_kgIV	**3.48 (1.24, 10.60)**	**3.34 (1.18, 10.34)**	8.03 (0.95, 88.57)	**7.81 (1.09, 90.12)**	**15.47 (2.17, 172.12)**	2.77 (0.80, 9.45)	1.56 (0.39, 6.42)	**4.23 (1.25, 13.98)**	1.34 (0.52, 3.33)	0.98 (0.34, 2.88)	1.52 (0.57, 4.19)	1.38 (0.50, 3.97)	0.96 (0.38, 2.32)	1.71 (0.68, 4.15)	1.26 (0.39, 3.75)	1.85 (0.74, 4.56)	1.35 (0.52, 3.53)	0.67 (0.12, 4.92)	0.64 (0.16, 2.63)	1.48 (0.39, 5.24)	1.09 (0.30, 3.95)	1.24 (0.26, 6.97)
ETA25mgSC	**7.41 (1.63, 40.94)**	**7.13 (1.57, 39.61)**	**17.77 (1.70, 239.17)**	**17.79 (1.79, 248.62)**	**34.72 (4.06, 462.49)**	**5.89 (1.06, 33.64)**	3.35 (0.54, 24.77)	**8.84 (1.72, 51.96)**	2.76 (0.67, 14.30)	2.05 (0.46, 11.36)	3.17 (0.72, 18.00)	2.89 (0.65, 17.40)	2.00 (0.50, 10.00)	3.56 (0.87, 18.10)	2.63 (0.52, 14.19)	3.84 (0.95, 19.42)	2.80 (0.67, 14.03)	1.48 (0.17, 14.54)	1.34 (0.24, 9.33)	3.13 (0.57, 18.65)	2.29 (0.41, 14.25)	2.64 (0.42, 18.85)
FIL100mgPO	**3.21 (1.54, 7.18)**	**3.12 (1.48, 7.28)**	7.46 (0.99, 66.15)	**7.16 (1.17, 70.09)**	**14.21 (2.52, 128.22)**	2.53 (0.93, 6.93)	1.46 (0.41, 4.86)	**3.85 (1.38, 10.30)**	1.22 (0.76, 1.96)	0.90 (0.45, 1.82)	1.39 (0.72, 2.74)	1.26 (0.63, 2.51)	0.88 (0.57, 1.34)	1.57 (0.97, 2.53)	1.15 (0.49, 2.44)	**1.70 (1.06, 2.72)**	1.24 (0.70, 2.12)	0.60 (0.13, 3.59)	0.58 (0.19, 1.91)	1.33 (0.52, 3.55)	0.99 (0.37, 2.79)	1.11 (0.30, 4.58)
FIL200mgPO	1.94 (0.99, 4.14)	1.89 (0.94, 4.05)	4.60 (0.61, 37.91)	4.33 (0.76, 39.61)	**8.75 (1.57, 72.75)**	1.55 (0.59, 4.08)	0.88 (0.26, 2.92)	2.35 (0.86, 6.09)	0.74 (0.50, 1.10)	0.55 (0.29, 1.05)	0.85 (0.47, 1.56)	0.77 (0.41, 1.47)	**0.53 (0.38, 0.75)**	0.95 (0.64, 1.40)	0.70 (0.32, 1.44)	1.03 (0.70, 1.52)	0.75 (0.46, 1.22)	0.37 (0.09, 2.09)	0.36 (0.12, 1.11)	0.80 (0.32, 2.08)	0.60 (0.22, 1.67)	0.69 (0.19, 2.81)
GUS1200mgIV	1.26 (0.87, 1.85)	1.21 (0.84, 1.82)	2.94 (0.36, 26.24)	2.85 (0.43, 27.78)	5.68 (0.86, 53.19)	0.99 (0.35, 2.88)	0.57 (0.16, 2.02)	1.50 (0.49, 4.40)	**0.48 (0.24, 0.89)**	**0.36 (0.15, 0.80)**	0.55 (0.24, 1.17)	0.49 (0.22, 1.09)	**0.35 (0.18, 0.62)**	0.61 (0.30, 1.15)	0.46 (0.17, 1.11)	0.67 (0.33, 1.25)	**0.49 (0.23, 0.96)**	0.23 (0.05, 1.39)	**0.23 (0.06, 0.81)**	0.52 (0.17, 1.60)	0.39 (0.12, 1.28)	0.45 (0.11, 1.91)

(TABLE S18F. continued)

	GUS200mgIV	GUS600mgIV	INF10mg_kgIV	INF20mg_kgIV	INF5mg_kgIV	MIR1000mgIV	MIR200mgIV	MIR600mgIV	NAT300mgIV	NAT3mg_kgIV	NAT3mg_kgIVx2	NAT6mg_kgIVx2	PBO	RIS1200mgIV	RIS200mgIV	RIS600mgIV	RIS600mgIV4_4	SEC10mg_kgIV	TOF15mgPO_BID	TOF1mgPO_BID	TOF5mgPO_BID	UPA12mgPO_BID
GUS200mgIV	GUS200mgIV	0.97 (0.69, 1.35)	2.37 (0.29, 20.55)	2.28 (0.34, 22.27)	4.58 (0.68, 41.12)	0.80 (0.28, 2.36)	0.45 (0.12, 1.62)	1.19 (0.40, 3.63)	**0.38 (0.19, 0.70)**	**0.28 (0.12, 0.63)**	**0.43 (0.19, 0.94)**	**0.39 (0.17, 0.86)**	**0.28 (0.14, 0.49)**	**0.49 (0.24, 0.90)**	**0.36 (0.13, 0.89)**	**0.53 (0.27, 0.97)**	**0.39 (0.18, 0.76)**	0.19 (0.04, 1.10)	**0.18 (0.05, 0.63)**	0.41 (0.14, 1.25)	0.31 (0.09, 1.02)	0.35 (0.09, 1.50)
GUS600mgIV	1.03 (0.74, 1.44)	GUS600mgIV	2.44 (0.30, 21.49)	2.32 (0.34, 23.38)	4.67 (0.69, 43.12)	0.82 (0.29, 2.41)	0.46 (0.13, 1.62)	1.22 (0.40, 3.61)	**0.40 (0.20, 0.73)**	**0.29 (0.12, 0.66)**	**0.45 (0.20, 0.98)**	**0.41 (0.18, 0.90)**	**0.28 (0.15, 0.51)**	**0.51 (0.25, 0.93)**	**0.37 (0.14, 0.91)**	0.55 (0.27, 1.03)	**0.40 (0.19, 0.78)**	0.20 (0.04, 1.14)	**0.19 (0.05, 0.66)**	0.43 (0.14, 1.28)	0.32 (0.09, 1.03)	0.36 (0.09, 1.49)
INF10mg_kgIV	0.42 (0.05, 3.49)	0.41 (0.05, 3.37)	INF10mg_kgIV	1.02 (0.39, 2.61)	1.97 (0.96, 4.68)	0.33 (0.04, 3.18)	0.19 (0.02, 1.97)	0.50 (0.05, 4.65)	0.16 (0.02, 1.20)	**0.12 (0.01, 0.92)**	0.18 (0.02, 1.47)	0.17 (0.02, 1.33)	**0.12 (0.01, 0.85)**	0.21 (0.02, 1.53)	0.16 (0.02, 1.18)	0.22 (0.03, 1.67)	0.16 (0.02, 1.23)	0.08 (0.01, 1.02)	**0.08 (0.01, 0.78)**	0.18 (0.02, 1.51)	0.13 (0.01, 1.10)	0.15 (0.01, 1.52)
INF20mg_kgIV	0.44 (0.04, 2.94)	0.43 (0.04, 2.90)	0.98 (0.38, 2.57)	INF20mg_kgIV	1.93 (0.92, 4.71)	0.34 (0.03, 2.74)	0.19 (0.02, 1.64)	0.53 (0.04, 3.91)	**0.17 (0.02, 0.94)**	**0.13 (0.01, 0.78)**	0.20 (0.02, 1.18)	0.18 (0.02, 1.08)	**0.12 (0.01, 0.66)**	0.22 (0.02, 1.23)	0.16 (0.02, 1.02)	0.24 (0.03, 1.33)	**0.17 (0.02, 0.99)**	**0.09 (0.01, 0.84)**	**0.08 (0.01, 0.73)**	0.18 (0.02, 1.30)	**0.14 (0.01, 0.93)**	0.15 (0.01, 1.34)
INF5mg_kgIV	0.22 (0.02, 1.48)	0.21 (0.02, 1.45)	0.51 (0.21, 1.04)	0.52 (0.21, 1.09)	INF5mg_kgIV	0.17 (0.02, 1.22)	**0.10 (0.01, 0.85)**	0.26 (0.03, 1.90)	**0.08 (0.01, 0.47)**	**0.06 (0.01, 0.39)**	**0.10 (0.01, 0.59)**	**0.09 (0.01, 0.54)**	**0.06 (0.01, 0.33)**	**0.11 (0.01, 0.61)**	**0.08 (0.01, 0.50)**	**0.12 (0.01, 0.65)**	**0.08 (0.01, 0.49)**	**0.04 (0.00, 0.45)**	**0.04 (0.00, 0.32)**	**0.09 (0.01, 0.63)**	**0.07 (0.01, 0.46)**	**0.08 (0.01, 0.70)**
MIR1000mgIV	1.26 (0.42, 3.59)	1.23 (0.42, 3.48)	3.05 (0.31, 27.83)	2.96 (0.37, 29.41)	5.87 (0.82, 56.22)	MIR1000mgIV	0.58 (0.20, 1.40)	1.50 (0.78, 2.81)	0.48 (0.19, 1.19)	0.35 (0.12, 1.03)	0.54 (0.19, 1.54)	0.50 (0.17, 1.37)	**0.35 (0.14, 0.83)**	0.61 (0.25, 1.51)	0.46 (0.13, 1.37)	0.67 (0.27, 1.64)	0.48 (0.18, 1.22)	0.24 (0.04, 1.81)	**0.23 (0.06, 0.87)**	0.52 (0.15, 1.80)	0.38 (0.11, 1.44)	0.46 (0.09, 2.11)
MIR200mgIV	2.24 (0.62, 8.13)	2.16 (0.62, 7.99)	5.15 (0.51, 60.30)	5.17 (0.61, 58.76)	**10.17 (1.18, 116.53)**	1.72 (0.71, 5.04)	MIR200mgIV	**2.60 (1.08, 7.45)**	0.84 (0.27, 2.82)	0.62 (0.17, 2.32)	0.96 (0.28, 3.40)	0.86 (0.24, 3.16)	0.60 (0.20, 1.99)	1.07 (0.34, 3.47)	0.78 (0.20, 3.23)	1.16 (0.37, 3.77)	0.84 (0.25, 2.99)	0.43 (0.06, 3.32)	0.41 (0.09, 2.00)	0.93 (0.21, 3.84)	0.69 (0.15, 3.10)	0.78 (0.15, 5.10)
MIR600mgIV	0.84 (0.28, 2.50)	0.82 (0.28, 2.51)	2.00 (0.22, 20.56)	1.89 (0.26, 22.61)	3.82 (0.53, 40.00)	0.67 (0.36, 1.29)	**0.39 (0.13, 0.92)**	MIR600mgIV	**0.32 (0.13, 0.84)**	**0.23 (0.08, 0.71)**	0.36 (0.13, 1.05)	**0.33 (0.11, 0.98)**	**0.23 (0.09, 0.60)**	0.41 (0.16, 1.06)	**0.29 (0.09, 0.96)**	0.44 (0.18, 1.15)	**0.32 (0.12, 0.88)**	0.16 (0.03, 1.15)	**0.15 (0.04, 0.63)**	0.34 (0.10, 1.29)	0.25 (0.07, 1.04)	0.30 (0.06, 1.48)
NAT300mgIV	**2.61 (1.43, 5.16)**	**2.53 (1.38, 5.12)**	6.16 (0.83, 51.48)	**5.78 (1.06, 53.26)**	**11.77 (2.14, 99.89)**	2.07 (0.84, 5.27)	1.19 (0.35, 3.71)	**3.15 (1.18, 7.94)**	NAT300mgIV	0.73 (0.42, 1.36)	1.13 (0.68, 1.99)	1.03 (0.58, 1.89)	**0.72 (0.58, 0.87)**	1.28 (0.97, 1.68)	0.94 (0.45, 1.85)	**1.39 (1.06, 1.81)**	1.01 (0.68, 1.51)	0.49 (0.11, 2.80)	0.48 (0.15, 1.42)	1.08 (0.44, 2.84)	0.80 (0.29, 2.26)	0.91 (0.26, 3.54)
NAT3mg_kgIV	**3.56 (1.60, 8.22)**	**3.45 (1.53, 8.07)**	**8.18 (1.09, 74.11)**	**7.96 (1.28, 79.95)**	**15.64 (2.58, 148.41)**	2.85 (0.98, 8.22)	1.61 (0.43, 5.73)	**4.29 (1.40, 12.41)**	1.36 (0.73, 2.37)	NAT3mg_kgIV	1.53 (0.97, 2.50)	1.41 (0.82, 2.33)	0.98 (0.55, 1.66)	1.74 (0.95, 3.07)	1.28 (0.50, 2.97)	**1.88 (1.03, 3.33)**	1.38 (0.70, 2.58)	0.69 (0.14, 3.79)	0.66 (0.19, 2.21)	1.46 (0.54, 4.35)	1.10 (0.34, 3.39)	1.22 (0.29, 5.17)
NAT3mg_kgIVx2	**2.31 (1.07, 5.19)**	**2.23 (1.02, 5.01)**	5.43 (0.68, 46.17)	5.12 (0.85, 50.28)	**10.38 (1.68, 92.34)**	1.85 (0.65, 5.13)	1.05 (0.29, 3.60)	2.80 (0.95, 7.69)	0.88 (0.50, 1.48)	0.65 (0.40, 1.03)	NAT3mg_kgIVx2	0.91 (0.57, 1.42)	0.63 (0.37, 1.02)	1.13 (0.64, 1.90)	0.82 (0.34, 1.93)	1.23 (0.69, 2.05)	0.89 (0.47, 1.61)	0.44 (0.09, 2.43)	0.43 (0.12, 1.41)	0.95 (0.33, 2.74)	0.72 (0.24, 2.08)	0.79 (0.21, 3.41)
NAT6mg_kgIVx2	**2.55 (1.16, 5.74)**	**2.46 (1.11, 5.53)**	6.00 (0.75, 51.04)	5.71 (0.93, 53.66)	**11.34 (1.84, 100.88)**	2.01 (0.73, 5.78)	1.16 (0.32, 4.09)	**3.05 (1.02, 8.73)**	0.97 (0.53, 1.72)	0.71 (0.43, 1.22)	1.09 (0.70, 1.75)	NAT6mg_kgIVx2	0.70 (0.39, 1.19)	1.24 (0.68, 2.22)	0.92 (0.36, 2.17)	1.35 (0.74, 2.41)	0.97 (0.50, 1.89)	0.48 (0.10, 2.81)	0.47 (0.14, 1.56)	1.05 (0.37, 3.06)	0.79 (0.27, 2.36)	0.88 (0.22, 3.74)
PBO	**3.62 (2.06, 7.10)**	**3.51 (1.97, 6.89)**	**8.67 (1.18, 71.78)**	**8.05 (1.51, 71.70)**	**16.40 (3.00, 139.81)**	**2.89 (1.20, 7.10)**	1.66 (0.50, 5.09)	**4.39 (1.67, 10.87)**	**1.39 (1.15, 1.71)**	1.02 (0.60, 1.82)	1.58 (0.98, 2.71)	1.43 (0.84, 2.55)	PBO	**1.78 (1.46, 2.17)**	1.32 (0.64, 2.51)	**1.93 (1.60, 2.34)**	1.39 (1.00, 2.00)	0.68 (0.16, 3.90)	0.68 (0.22, 1.96)	1.51 (0.62, 3.85)	1.12 (0.42, 3.04)	1.27 (0.37, 4.79)
RIS1200mgIV	**2.05 (1.11, 4.10)**	**1.98 (1.07, 4.05)**	4.87 (0.65, 40.08)	4.56 (0.81, 41.99)	**9.28 (1.65, 78.32)**	1.63 (0.66, 4.05)	0.94 (0.29, 2.97)	2.47 (0.94, 6.22)	0.78 (0.60, 1.03)	0.57 (0.33, 1.05)	0.89 (0.53, 1.57)	0.81 (0.45, 1.46)	**0.56 (0.46, 0.68)**	RIS1200mgIV	0.74 (0.36, 1.40)	1.08 (0.94, 1.25)	0.79 (0.54, 1.18)	0.38 (0.09, 2.24)	0.38 (0.12, 1.12)	0.84 (0.35, 2.18)	0.63 (0.24, 1.77)	0.72 (0.20, 2.78)
RIS200mgIV	**2.79 (1.12, 7.41)**	**2.70 (1.10, 7.18)**	6.40 (0.85, 65.56)	6.18 (0.98, 63.08)	**12.39 (2.02, 120.66)**	2.20 (0.73, 7.52)	1.28 (0.31, 4.94)	**3.40 (1.04, 10.80)**	1.06 (0.54, 2.23)	0.78 (0.34, 1.99)	1.22 (0.52, 2.95)	1.09 (0.46, 2.75)	0.76 (0.40, 1.56)	1.35 (0.71, 2.77)	RIS200mgIV	1.47 (0.78, 2.98)	1.07 (0.51, 2.39)	0.52 (0.11, 3.34)	0.51 (0.14, 1.99)	1.15 (0.39, 3.74)	0.88 (0.26, 2.81)	0.99 (0.23, 4.49)
RIS600mgIV	**1.88 (1.03, 3.77)**	1.82 (0.97, 3.72)	4.50 (0.60, 37.12)	4.20 (0.75, 38.16)	**8.55 (1.53, 73.26)**	1.50 (0.61, 3.73)	0.86 (0.27, 2.71)	2.26 (0.87, 5.63)	**0.72 (0.55, 0.95)**	**0.53 (0.30, 0.97)**	0.82 (0.49, 1.45)	0.74 (0.41, 1.36)	**0.52 (0.43, 0.62)**	0.92 (0.80, 1.06)	0.68 (0.34, 1.28)	RIS600mgIV	0.72 (0.50, 1.08)	0.35 (0.08, 2.05)	0.35 (0.11, 1.02)	0.78 (0.32, 2.01)	0.58 (0.22, 1.65)	0.66 (0.18, 2.55)
RIS600mgIV4_4	**2.59 (1.32, 5.48)**	**2.52 (1.28, 5.36)**	6.15 (0.81, 52.62)	**5.82 (1.02, 53.95)**	**11.78 (2.02, 104.61)**	2.07 (0.82, 5.46)	1.20 (0.33, 3.96)	**3.14 (1.14, 8.21)**	0.99 (0.66, 1.48)	0.73 (0.39, 1.43)	1.12 (0.62, 2.14)	1.03 (0.53, 2.02)	0.72 (0.50, 1.00)	1.27 (0.85, 1.86)	0.93 (0.42, 1.96)	1.38 (0.93, 1.99)	RIS600mgIV4_4	0.49 (0.11, 2.73)	0.48 (0.15, 1.47)	1.07 (0.41, 2.99)	0.80 (0.29, 2.32)	0.90 (0.25, 3.59)
SEC10mg_kgIV	5.32 (0.91, 26.00)	5.12 (0.88, 26.17)	12.43 (0.98, 146.85)	**11.63 (1.19, 151.67)**	**23.83 (2.21, 285.15)**	4.16 (0.55, 25.93)	2.35 (0.30, 16.15)	6.30 (0.87, 39.82)	2.04 (0.36, 8.80)	1.44 (0.26, 7.21)	2.26 (0.41, 10.54)	2.09 (0.36, 9.70)	1.47 (0.26, 6.17)	2.62 (0.45, 10.98)	1.92 (0.30, 9.19)	2.83 (0.49, 11.99)	2.03 (0.37, 9.35)	SEC10mg_kgIV	0.93 (0.12, 6.47)	2.13 (0.30, 12.22)	1.58 (0.22, 9.49)	1.78 (0.23, 17.01)
TOF15mgPO_BID	**5.43 (1.58, 18.69)**	**5.26 (1.51, 18.54)**	**12.83 (1.28, 123.05)**	**12.73 (1.37, 131.27)**	**25.19 (3.08, 247.13)**	**4.31 (1.16, 16.58)**	2.45 (0.50, 11.69)	**6.49 (1.59, 24.71)**	2.07 (0.70, 6.67)	1.52 (0.45, 5.39)	2.35 (0.71, 8.04)	2.14 (0.64, 6.94)	1.47 (0.51, 4.59)	2.63 (0.90, 8.29)	1.96 (0.50, 7.16)	2.85 (0.98, 8.98)	2.07 (0.68, 6.60)	1.07 (0.15, 8.62)	TOF15mgPO_BID	2.21 (0.86, 6.48)	1.66 (0.63, 4.86)	1.92 (0.35, 10.18)
TOF1mgPO_BID	2.41 (0.80, 7.13)	2.32 (0.78, 7.11)	5.64 (0.66, 55.69)	5.50 (0.77, 60.41)	**11.22 (1.58, 109.69)**	1.92 (0.56, 6.84)	1.07 (0.26, 4.72)	2.93 (0.77, 10.37)	0.93 (0.35, 2.28)	0.69 (0.23, 1.85)	1.05 (0.37, 3.03)	0.95 (0.33, 2.74)	0.66 (0.26, 1.62)	1.19 (0.46, 2.87)	0.87 (0.27, 2.57)	1.29 (0.50, 3.11)	0.94 (0.33, 2.42)	0.47 (0.08, 3.37)	0.45 (0.15, 1.16)	TOF1mgPO_BID	0.76 (0.34, 1.64)	0.86 (0.17, 4.11)
TOF5mgPO_BID	3.23 (0.98, 10.76)	3.13 (0.97, 10.83)	7.47 (0.91, 75.43)	**7.07 (1.08, 83.89)**	**14.20 (2.16, 157.38)**	2.66 (0.69, 9.49)	1.44 (0.32, 6.47)	3.95 (0.96, 14.12)	1.25 (0.44, 3.41)	0.91 (0.29, 2.92)	1.39 (0.48, 4.16)	1.27 (0.42, 3.72)	0.89 (0.33, 2.38)	1.59 (0.57, 4.11)	1.14 (0.36, 3.88)	1.72 (0.61, 4.50)	1.25 (0.43, 3.46)	0.63 (0.11, 4.46)	0.60 (0.21, 1.60)	1.32 (0.61, 2.98)	TOF5mgPO_BID	1.16 (0.22, 6.06)
UPA12mgPO_BID	2.83 (0.67, 11.55)	2.77 (0.67, 11.28)	6.59 (0.66, 74.29)	6.80 (0.75, 70.51)	**12.84 (1.44, 153.68)**	2.17 (0.47, 11.59)	1.29 (0.20, 6.87)	3.31 (0.67, 16.48)	1.10 (0.28, 3.91)	0.82 (0.19, 3.45)	1.26 (0.29, 4.87)	1.14 (0.27, 4.59)	0.79 (0.21, 2.73)	1.39 (0.36, 4.96)	1.01 (0.22, 4.32)	1.51 (0.39, 5.41)	1.12 (0.28, 4.01)	0.56 (0.06, 4.40)	0.52 (0.10, 2.86)	1.16 (0.24, 5.97)	0.86 (0.17, 4.49)	UPA12mgPO_BID

(**TABLE S18F.** continued)

	GUS200mgIV	GUS600mgIV	INF10mg_kgIV	INF20mg_kgIV	INF5mg_kgIV	MIR1000mgIV	MIR200mgIV	MIR600mgIV	NAT300mgIV	NAT3mg_kgIV	NAT3mg_kgIVx2	NAT6mg_kgIVx2	PBO	RIS1200mgIV	RIS200mgIV	RIS600mgIV	RIS600mgIV4_4	SEC10mg_kgIV	TOF15mgPO_BID	TOF1mgPO_BID	TOF5mgPO_BID	UPA12mgPO_BID
UPA24mgPO	**5.32 (1.09, 40.97)**	**5.10 (1.06, 39.53)**	12.39 (0.92, 201.96)	**12.31 (1.13, 206.39)**	**24.86 (2.32, 365.17)**	4.24 (0.77, 40.72)	2.51 (0.35, 19.54)	**6.51 (1.04, 53.95)**	2.05 (0.43, 14.24)	1.49 (0.28, 10.99)	2.34 (0.45, 18.35)	2.13 (0.41, 15.44)	1.46 (0.31, 10.78)	2.60 (0.56, 18.75)	1.91 (0.36, 15.18)	2.81 (0.60, 19.96)	2.03 (0.42, 14.85)	1.06 (0.11, 14.97)	1.04 (0.15, 6.69)	2.23 (0.36, 15.78)	1.70 (0.26, 12.68)	1.79 (0.45, 23.53)
UPA24mgPO_BID	1.54 (0.39, 5.57)	1.50 (0.38, 5.39)	3.64 (0.36, 34.95)	3.74 (0.44, 34.68)	7.11 (0.86, 69.34)	1.23 (0.29, 5.20)	0.71 (0.12, 3.32)	1.88 (0.40, 8.32)	0.61 (0.16, 1.73)	0.45 (0.11, 1.47)	0.69 (0.16, 2.24)	0.63 (0.15, 2.04)	0.44 (0.12, 1.25)	0.78 (0.21, 2.21)	0.55 (0.13, 2.00)	0.85 (0.23, 2.42)	0.61 (0.16, 1.81)	0.31 (0.04, 2.45)	0.29 (0.05, 1.27)	0.66 (0.14, 2.56)	0.48 (0.10, 1.95)	0.55 (0.18, 1.55)
UPA3mgPO_BID	4.17 (0.96, 21.16)	4.07 (0.95, 19.79)	9.26 (0.98, 125.48)	**9.50 (1.02, 122.99)**	**18.75 (2.12, 236.99)**	3.22 (0.66, 19.67)	1.93 (0.29, 12.12)	4.95 (0.94, 32.80)	1.62 (0.38, 7.41)	1.17 (0.28, 5.93)	1.81 (0.42, 9.41)	1.67 (0.37, 8.44)	1.16 (0.28, 5.36)	2.06 (0.48, 9.70)	1.49 (0.30, 8.32)	2.24 (0.52, 10.71)	1.62 (0.38, 7.68)	0.84 (0.09, 7.78)	0.79 (0.12, 5.01)	1.71 (0.33, 10.05)	1.32 (0.22, 7.77)	1.44 (0.39, 7.69)
UPA45mgPO	**2.08 (1.14, 4.16)**	**2.01 (1.08, 4.14)**	4.92 (0.66, 42.44)	4.64 (0.82, 43.11)	**9.32 (1.67, 81.70)**	1.66 (0.68, 4.27)	0.96 (0.28, 3.01)	2.52 (0.95, 6.46)	0.80 (0.59, 1.07)	0.59 (0.33, 1.07)	0.90 (0.53, 1.59)	0.82 (0.46, 1.51)	**0.58 (0.46, 0.71)**	1.02 (0.76, 1.36)	0.75 (0.35, 1.49)	1.11 (0.83, 1.47)	0.80 (0.54, 1.22)	0.39 (0.09, 2.22)	0.39 (0.12, 1.16)	0.86 (0.35, 2.29)	0.64 (0.23, 1.78)	0.73 (0.21, 2.83)
UPA6mgPO_BID	1.32 (0.32, 4.31)	1.28 (0.32, 4.31)	2.98 (0.32, 34.58)	2.89 (0.36, 29.06)	5.71 (0.71, 60.61)	1.01 (0.24, 4.06)	0.60 (0.11, 2.75)	1.56 (0.34, 6.13)	0.51 (0.13, 1.30)	0.37 (0.09, 1.14)	0.57 (0.14, 1.69)	0.52 (0.12, 1.55)	**0.37 (0.10, 0.91)**	0.65 (0.18, 1.66)	0.46 (0.11, 1.49)	0.71 (0.19, 1.81)	0.52 (0.13, 1.36)	0.25 (0.03, 1.88)	0.24 (0.04, 1.12)	0.53 (0.12, 2.06)	0.40 (0.08, 1.69)	0.46 (0.15, 1.14)
UST130mgIV	**2.16 (1.12, 4.33)**	**2.08 (1.07, 4.28)**	5.05 (0.69, 42.46)	4.75 (0.87, 43.51)	**9.75 (1.75, 82.63)**	1.69 (0.66, 4.50)	0.98 (0.29, 3.16)	2.60 (0.95, 6.75)	0.82 (0.58, 1.17)	0.60 (0.33, 1.15)	0.93 (0.54, 1.70)	0.84 (0.47, 1.59)	**0.59 (0.44, 0.78)**	1.05 (0.74, 1.49)	0.78 (0.36, 1.59)	1.13 (0.81, 1.61)	0.82 (0.54, 1.29)	0.40 (0.09, 2.38)	0.40 (0.12, 1.23)	0.88 (0.36, 2.38)	0.66 (0.24, 1.87)	0.75 (0.21, 2.89)
UST1mg_kgIV	1.87 (0.84, 4.36)	1.82 (0.81, 4.28)	4.42 (0.57, 39.46)	4.19 (0.70, 40.61)	**8.29 (1.40, 79.21)**	1.48 (0.52, 4.39)	0.85 (0.24, 3.08)	2.25 (0.79, 6.41)	0.72 (0.41, 1.26)	0.53 (0.25, 1.17)	0.80 (0.41, 1.74)	0.73 (0.36, 1.61)	**0.51 (0.31, 0.87)**	0.91 (0.53, 1.59)	0.67 (0.28, 1.61)	0.99 (0.58, 1.74)	0.72 (0.39, 1.34)	0.35 (0.07, 2.15)	0.35 (0.10, 1.23)	0.77 (0.28, 2.31)	0.57 (0.19, 1.88)	0.65 (0.18, 2.67)
UST3mg_kgIV	1.81 (0.81, 4.16)	1.75 (0.76, 4.15)	4.25 (0.55, 39.74)	4.04 (0.66, 39.19)	**8.12 (1.36, 73.03)**	1.42 (0.52, 4.34)	0.83 (0.23, 2.85)	2.19 (0.74, 6.17)	0.69 (0.40, 1.21)	0.51 (0.24, 1.11)	0.78 (0.39, 1.65)	0.71 (0.34, 1.54)	**0.50 (0.30, 0.84)**	0.88 (0.51, 1.55)	0.64 (0.26, 1.50)	0.95 (0.56, 1.67)	0.69 (0.38, 1.28)	0.34 (0.07, 2.26)	0.34 (0.10, 1.17)	0.74 (0.27, 2.20)	0.55 (0.19, 1.72)	0.63 (0.17, 2.60)
UST4_5mg_kgIV	**2.12 (1.10, 4.43)**	**2.05 (1.03, 4.36)**	5.04 (0.66, 42.62)	4.73 (0.84, 43.91)	**9.56 (1.69, 83.48)**	1.69 (0.67, 4.43)	0.96 (0.28, 3.16)	2.56 (0.97, 6.58)	0.81 (0.57, 1.16)	0.60 (0.32, 1.17)	0.92 (0.51, 1.73)	0.84 (0.44, 1.61)	**0.58 (0.42, 0.79)**	1.04 (0.77, 1.36)	0.76 (0.36, 1.52)	1.12 (0.87, 1.45)	0.81 (0.52, 1.30)	0.40 (0.09, 2.38)	0.39 (0.12, 1.17)	0.88 (0.35, 2.33)	0.66 (0.24, 1.88)	0.74 (0.20, 2.91)
UST6mg_kg90mgIV_SC	1.25 (0.86, 1.80)	1.21 (0.83, 1.76)	2.96 (0.36, 26.53)	2.78 (0.41, 28.61)	5.63 (0.83, 52.00)	0.98 (0.36, 2.84)	0.56 (0.16, 2.06)	1.48 (0.49, 4.40)	**0.48 (0.24, 0.90)**	**0.35 (0.15, 0.82)**	0.54 (0.24, 1.20)	0.49 (0.21, 1.12)	**0.34 (0.17, 0.63)**	0.61 (0.30, 1.17)	0.45 (0.17, 1.13)	0.66 (0.33, 1.28)	**0.48 (0.23, 0.98)**	0.23 (0.05, 1.40)	**0.23 (0.06, 0.80)**	0.52 (0.18, 1.58)	0.38 (0.12, 1.28)	0.43 (0.11, 1.89)
UST6mg_kgIV	1.65 (0.88, 3.37)	1.60 (0.84, 3.27)	3.88 (0.53, 34.04)	3.65 (0.67, 34.52)	**7.50 (1.34, 64.33)**	1.31 (0.51, 3.48)	0.75 (0.23, 2.47)	1.99 (0.73, 5.15)	**0.63 (0.46, 0.88)**	**0.47 (0.26, 0.88)**	0.72 (0.42, 1.30)	0.65 (0.36, 1.23)	**0.45 (0.35, 0.58)**	0.81 (0.59, 1.12)	0.60 (0.28, 1.20)	0.87 (0.64, 1.21)	**0.63 (0.42, 0.98)**	0.31 (0.07, 1.81)	**0.31 (0.09, 0.95)**	0.68 (0.28, 1.83)	0.50 (0.18, 1.42)	0.58 (0.17, 2.23)
UST90mgSC	**3.71 (1.02, 11.99)**	**3.58 (1.01, 12.14)**	8.62 (0.87, 90.33)	8.34 (0.99, 97.81)	**16.80 (1.96, 185.30)**	2.94 (0.79, 11.08)	1.62 (0.36, 8.02)	**4.44 (1.10, 17.00)**	1.39 (0.48, 3.91)	1.03 (0.31, 3.30)	1.58 (0.50, 4.85)	1.42 (0.45, 4.57)	1.01 (0.35, 2.70)	1.78 (0.61, 4.87)	1.31 (0.38, 4.29)	1.92 (0.68, 5.27)	1.40 (0.47, 4.11)	0.71 (0.12, 4.65)	0.68 (0.16, 2.89)	1.50 (0.41, 5.63)	1.12 (0.31, 4.43)	1.27 (0.25, 6.66)
VED0_5mg_kgIV	**2.58 (1.04, 6.55)**	2.49 (0.99, 6.40)	6.18 (0.73, 60.41)	5.82 (0.90, 60.85)	**11.77 (1.80, 121.98)**	2.04 (0.65, 6.07)	1.16 (0.28, 4.47)	3.06 (0.92, 9.52)	0.98 (0.49, 1.88)	0.71 (0.31, 1.76)	1.11 (0.47, 2.63)	1.02 (0.40, 2.40)	0.71 (0.35, 1.30)	1.25 (0.63, 2.40)	0.92 (0.35, 2.36)	1.36 (0.68, 2.60)	0.99 (0.46, 2.00)	0.48 (0.10, 3.33)	0.47 (0.14, 1.63)	1.07 (0.35, 3.33)	0.79 (0.25, 2.65)	0.90 (0.21, 3.96)
VED2mg_kgIV	2.04 (0.85, 5.05)	1.98 (0.81, 4.91)	4.74 (0.57, 45.32)	4.55 (0.71, 46.69)	**9.17 (1.43, 93.84)**	1.59 (0.53, 4.63)	0.91 (0.24, 3.45)	2.42 (0.74, 7.09)	0.77 (0.40, 1.42)	0.57 (0.24, 1.32)	0.88 (0.39, 1.94)	0.80 (0.33, 1.79)	**0.56 (0.29, 0.98)**	0.99 (0.51, 1.80)	0.73 (0.27, 1.77)	1.08 (0.56, 1.95)	0.78 (0.38, 1.52)	0.38 (0.08, 2.49)	0.37 (0.11, 1.26)	0.84 (0.29, 2.53)	0.62 (0.21, 1.97)	0.70 (0.17, 3.11)
VED300mgIV	1.72 (0.88, 3.63)	1.67 (0.83, 3.62)	4.11 (0.53, 34.28)	3.89 (0.68, 36.27)	**7.70 (1.33, 69.43)**	1.36 (0.53, 3.57)	0.79 (0.23, 2.64)	2.07 (0.77, 5.38)	**0.66 (0.45, 0.99)**	**0.49 (0.25, 0.93)**	0.75 (0.41, 1.43)	0.68 (0.35, 1.32)	**0.48 (0.34, 0.66)**	0.84 (0.57, 1.23)	0.62 (0.28, 1.30)	0.92 (0.62, 1.33)	0.66 (0.41, 1.08)	0.32 (0.07, 1.94)	**0.32 (0.10, 0.98)**	0.71 (0.28, 1.87)	0.53 (0.20, 1.47)	0.61 (0.16, 2.41)

	UPA24mgPO	UPA24mgPO_BID	UPA3mgPO_BID	UPA45mgPO	UPA6mgPO_BID	UST130mgIV	UST1mg_kgIV	UST3mg_kgIV	UST4_5mg_kgIV	UST6mg_kg90mgIV_SC	UST6mg_kgIV	UST90mgSC	VED0_5mg_kgIV	VED2mg_kgIV	VED300mgIV
ABA10mg_kgIV	1.11 (0.15, 8.22)	3.96 (0.89, 22.82)	1.51 (0.22, 9.28)	2.88 (0.99, 11.81)	**4.79 (1.13, 30.15)**	2.80 (0.94, 11.39)	3.22 (0.99, 14.14)	**3.39 (1.04, 14.86)**	2.85 (0.96, 11.63)	**4.81 (1.41, 22.89)**	**3.62 (1.22, 15.03)**	1.69 (0.37, 8.94)	2.38 (0.69, 10.54)	2.96 (0.90, 13.06)	**3.52 (1.14, 15.02)**
ABA30mg_kgIV	0.55 (0.05, 4.61)	1.97 (0.41, 11.33)	0.72 (0.10, 4.59)	1.45 (0.47, 5.17)	2.38 (0.49, 13.71)	1.41 (0.46, 5.06)	1.62 (0.49, 6.15)	1.69 (0.48, 6.47)	1.43 (0.45, 5.24)	2.46 (0.69, 9.76)	1.81 (0.59, 6.56)	0.84 (0.18, 4.20)	1.19 (0.35, 4.67)	1.51 (0.45, 5.84)	1.76 (0.53, 6.54)
ABA3mg_kgIV	0.94 (0.10, 6.36)	3.34 (0.66, 15.89)	1.23 (0.17, 6.47)	2.42 (0.76, 7.39)	3.96 (0.82, 19.15)	2.37 (0.72, 7.28)	2.71 (0.81, 9.39)	2.83 (0.82, 9.76)	2.40 (0.75, 7.36)	**4.10 (1.18, 14.11)**	3.07 (0.97, 9.42)	1.40 (0.30, 6.20)	1.98 (0.51, 6.93)	2.49 (0.68, 8.78)	2.95 (0.89, 8.99)
ADA160mg80mg40mg SC	0.24 (0.03, 1.26)	0.84 (0.23, 3.39)	0.31 (0.06, 1.35)	0.62 (0.30, 1.26)	0.99 (0.30, 4.06)	0.60 (0.29, 1.27)	0.69 (0.29, 1.66)	0.72 (0.30, 1.72)	0.62 (0.28, 1.31)	1.04 (0.82, 1.32)	0.79 (0.37, 1.64)	0.35 (0.10, 1.29)	0.50 (0.19, 1.33)	0.64 (0.25, 1.65)	0.76 (0.34, 1.60)
ADA160mg80mg60mg SC	**0.12 (0.01, 0.61)**	0.41 (0.10, 1.66)	**0.15 (0.03, 0.70)**	**0.30 (0.12, 0.65)**	0.48 (0.13, 2.01)	**0.29 (0.11, 0.65)**	**0.33 (0.12, 0.84)**	**0.35 (0.12, 0.86)**	**0.30 (0.11, 0.66)**	0.50 (0.17, 1.32)	**0.38 (0.14, 0.83)**	**0.18 (0.04, 0.65)**	**0.24 (0.08, 0.67)**	**0.31 (0.11, 0.84)**	**0.37 (0.13, 0.83)**
ADA160mg80mgSC	0.25 (0.03, 1.30)	0.85 (0.27, 3.34)	0.32 (0.06, 1.45)	0.65 (0.38, 1.04)	1.03 (0.35, 4.26)	0.63 (0.36, 1.05)	0.71 (0.36, 1.42)	0.74 (0.37, 1.47)	0.64 (0.36, 1.07)	1.07 (0.51, 2.41)	0.82 (0.47, 1.33)	0.37 (0.12, 1.17)	0.52 (0.24, 1.11)	0.67 (0.33, 1.39)	0.78 (0.44, 1.32)
ADA40mg20mgSC	0.48 (0.06, 2.69)	1.62 (0.46, 7.13)	0.60 (0.11, 3.11)	1.20 (0.60, 2.44)	1.95 (0.58, 8.49)	1.17 (0.57, 2.47)	1.35 (0.56, 3.10)	1.39 (0.59, 3.28)	1.19 (0.56, 2.51)	2.00 (0.80, 5.40)	1.51 (0.75, 3.16)	0.68 (0.21, 2.50)	0.99 (0.39, 2.49)	1.24 (0.51, 3.01)	1.46 (0.69, 3.10)
ADA80mg40mgSC	0.29 (0.03, 1.55)	0.98 (0.31, 3.92)	0.37 (0.07, 1.67)	0.74 (0.42, 1.32)	1.19 (0.39, 4.76)	0.72 (0.39, 1.33)	0.83 (0.39, 1.73)	0.86 (0.41, 1.79)	0.73 (0.39, 1.38)	1.24 (0.56, 2.93)	0.94 (0.52, 1.69)	0.42 (0.14, 1.45)	0.60 (0.27, 1.40)	0.77 (0.35, 1.75)	0.88 (0.49, 1.68)
BRO210mgIV	1.48 (0.05, 27.44)	5.02 (0.31, 76.18)	1.81 (0.13, 28.67)	3.67 (0.31, 44.96)	6.24 (0.43, 87.34)	3.62 (0.32, 43.63)	4.22 (0.32, 55.70)	4.32 (0.31, 56.65)	3.66 (0.31, 43.35)	6.21 (0.49, 85.06)	4.69 (0.41, 58.98)	2.16 (0.14, 26.71)	2.97 (0.24, 36.73)	3.84 (0.32, 47.44)	4.44 (0.35, 54.09)
BRO350mgIV	0.22 (0.01, 2.01)	0.78 (0.08, 5.64)	0.27 (0.03, 2.44)	0.57 (0.08, 2.86)	0.92 (0.11, 6.48)	0.55 (0.08, 2.90)	0.63 (0.08, 3.36)	0.66 (0.08, 3.53)	0.56 (0.08, 2.92)	0.97 (0.12, 5.35)	0.72 (0.10, 3.69)	0.31 (0.04, 2.21)	0.47 (0.07, 2.62)	0.59 (0.08, 3.45)	0.68 (0.10, 3.45)
BRO700mgIV	0.42 (0.02, 4.38)	1.46 (0.13, 10.33)	0.53 (0.05, 4.83)	1.07 (0.13, 6.35)	1.71 (0.15, 14.17)	1.04 (0.13, 6.12)	1.21 (0.13, 7.51)	1.26 (0.14, 7.62)	1.06 (0.12, 6.18)	1.79 (0.22, 11.39)	1.35 (0.16, 7.95)	0.59 (0.06, 5.15)	0.89 (0.11, 5.45)	1.11 (0.13, 7.16)	1.28 (0.15, 7.71)
CER100mgSC	0.60 (0.08, 3.15)	2.06 (0.64, 7.81)	0.76 (0.16, 3.38)	1.54 (0.90, 2.66)	2.44 (0.83, 9.43)	1.50 (0.85, 2.68)	1.73 (0.85, 3.59)	1.80 (0.86, 3.67)	1.51 (0.86, 2.77)	**2.58 (1.19, 6.07)**	**1.96 (1.11, 3.40)**	0.87 (0.29, 2.88)	1.25 (0.56, 2.84)	1.58 (0.74, 3.57)	**1.84 (1.05, 3.44)**
CER10mg_kgIV	0.92 (0.10, 6.69)	3.15 (0.68, 19.35)	1.15 (0.18, 7.61)	2.31 (0.83, 7.84)	3.67 (0.86, 21.31)	2.26 (0.81, 7.77)	2.61 (0.85, 9.64)	2.73 (0.87, 10.18)	2.30 (0.83, 8.11)	**3.98 (1.23, 14.82)**	**2.95 (1.05, 10.22)**	1.36 (0.31, 6.74)	1.87 (0.60, 7.74)	2.40 (0.76, 9.35)	2.81 (0.98, 10.10)
CER200mgSC	0.84 (0.12, 4.17)	2.87 (0.83, 11.19)	1.08 (0.21, 4.71)	**2.13 (1.20, 4.08)**	**3.50 (1.12, 13.44)**	**2.06 (1.16, 4.05)**	**2.39 (1.14, 5.39)**	**2.48 (1.13, 5.45)**	**2.09 (1.13, 4.14)**	**3.57 (1.61, 8.67)**	**2.67 (1.53, 5.22)**	1.23 (0.39, 4.19)	1.74 (0.76, 4.51)	2.20 (1.00, 5.44)	**2.59 (1.36, 5.13)**
CER20mg_kgIV	1.76 (0.16, 12.77)	**5.92 (1.16, 37.59)**	2.22 (0.30, 14.70)	**4.25 (1.29, 18.62)**	**7.02 (1.41, 49.44)**	**4.19 (1.24, 17.86)**	**4.74 (1.31, 23.08)**	**4.92 (1.35, 22.67)**	**4.25 (1.28, 19.33)**	**7.13 (1.83, 37.71)**	**5.42 (1.62, 22.98)**	2.41 (0.54, 15.80)	3.49 (0.93, 17.43)	**4.39 (1.24, 22.11)**	**5.13 (1.52, 23.26)**
CER400mgSC	0.56 (0.08, 2.62)	1.86 (0.66, 7.01)	0.69 (0.15, 3.02)	**1.42 (1.05, 1.94)**	2.22 (0.87, 8.34)	1.38 (0.98, 1.99)	1.59 (0.91, 2.77)	1.65 (0.90, 2.89)	1.40 (0.96, 2.05)	**2.37 (1.24, 4.84)**	**1.79 (1.29, 2.50)**	0.81 (0.29, 2.41)	1.16 (0.60, 2.37)	1.48 (0.79, 2.88)	**1.73 (1.14, 2.55)**
CER5mg_kgIV	0.63 (0.08, 3.87)	2.24 (0.56, 10.67)	0.82 (0.15, 4.38)	1.68 (0.64, 4.21)	2.66 (0.70, 12.15)	1.64 (0.62, 4.16)	1.86 (0.66, 5.39)	1.93 (0.71, 5.67)	1.64 (0.63, 4.28)	2.81 (0.97, 8.83)	2.12 (0.83, 5.35)	0.97 (0.25, 3.85)	1.36 (0.45, 4.43)	1.75 (0.59, 5.05)	2.02 (0.77, 5.23)
ETA25mgSC	1.34 (0.13, 12.54)	4.86 (0.82, 32.19)	1.77 (0.24, 13.56)	3.48 (0.86, 17.60)	**5.68 (1.06, 37.85)**	3.39 (0.81, 17.47)	3.93 (0.90, 20.85)	4.08 (0.95, 21.57)	3.44 (0.82, 17.66)	**5.96 (1.30, 33.34)**	**4.40 (1.09, 22.81)**	2.04 (0.35, 13.06)	2.86 (0.61, 15.50)	3.66 (0.77, 19.81)	4.26 (1.00, 21.90)
FIL100mgPO	0.60 (0.08, 3.03)	2.01 (0.65, 7.85)	0.75 (0.15, 3.31)	1.53 (0.94, 2.48)	2.40 (0.89, 9.38)	1.49 (0.89, 2.51)	1.72 (0.86, 3.33)	1.76 (0.92, 3.53)	1.52 (0.88, 2.53)	**2.55 (1.20, 5.93)**	**1.94 (1.16, 3.14)**	0.88 (0.30, 2.63)	1.24 (0.59, 2.72)	1.58 (0.80, 3.36)	**1.86 (1.07, 3.11)**
FIL200mgPO	0.37 (0.06, 1.69)	1.23 (0.41, 4.62)	0.46 (0.09, 1.99)	0.93 (0.62, 1.40)	1.45 (0.55, 5.50)	0.91 (0.58, 1.38)	1.04 (0.56, 1.91)	1.08 (0.58, 1.99)	0.92 (0.57, 1.43)	1.55 (0.78, 3.36)	1.18 (0.76, 1.77)	0.52 (0.19, 1.60)	0.76 (0.37, 1.60)	0.96 (0.50, 1.95)	1.13 (0.70, 1.81)
GUS1200mgIV	0.23 (0.03, 1.19)	0.81 (0.22, 3.30)	0.31 (0.06, 1.32)	0.60 (0.29, 1.13)	0.95 (0.29, 3.87)	0.58 (0.29, 1.16)	0.67 (0.29, 1.52)	0.70 (0.30, 1.61)	0.59 (0.28, 1.18)	1.00 (0.67, 1.53)	0.76 (0.37, 1.47)	0.34 (0.10, 1.24)	0.49 (0.19, 1.23)	0.62 (0.25, 1.50)	0.73 (0.34, 1.46)

(**TABLE S18F.** continued)

	UPA24mgPO	UPA24mgPO_BID	UPA3mgPO_BID	UPA45mgPO	UPA6mgPO_BID	UST130mgIV	UST1mg_kgIV	UST3mg_kgIV	UST4_5mg_kgIV	UST6mg_kg90mgIV_SC	UST6mg_kgIV	UST90mgSC	VED0_5mg_kgIV	VED2mg_kgIV	VED300mgIV
GUS200mgIV	**0.19 (0.02, 0.92)**	0.65 (0.18, 2.56)	0.24 (0.05, 1.04)	**0.48 (0.24, 0.88)**	0.76 (0.23, 3.11)	**0.46 (0.23, 0.90)**	0.53 (0.23, 1.20)	0.55 (0.24, 1.24)	**0.47 (0.23, 0.91)**	0.80 (0.55, 1.16)	0.61 (0.30, 1.14)	**0.27 (0.08, 0.98)**	**0.39 (0.15, 0.97)**	0.49 (0.20, 1.17)	0.58 (0.28, 1.14)
GUS600mgIV	**0.20 (0.03, 0.95)**	0.67 (0.19, 2.60)	0.25 (0.05, 1.05)	**0.50 (0.24, 0.93)**	0.78 (0.23, 3.16)	**0.48 (0.23, 0.94)**	0.55 (0.23, 1.24)	0.57 (0.24, 1.31)	**0.49 (0.23, 0.97)**	0.82 (0.57, 1.21)	0.62 (0.31, 1.19)	**0.28 (0.08, 0.99)**	0.40 (0.16, 1.01)	0.51 (0.20, 1.24)	0.60 (0.28, 1.20)
INF10mg_kgIV	0.08 (0.00, 1.09)	0.27 (0.03, 2.78)	0.11 (0.01, 1.02)	0.20 (0.02, 1.52)	0.34 (0.03, 3.15)	0.20 (0.02, 1.46)	0.23 (0.03, 1.75)	0.24 (0.03, 1.82)	0.20 (0.02, 1.52)	0.34 (0.04, 2.77)	0.26 (0.03, 1.89)	0.12 (0.01, 1.15)	0.16 (0.02, 1.38)	0.21 (0.02, 1.75)	0.24 (0.03, 1.87)
INF20mg_kgIV	**0.08 (0.00, 0.88)**	0.27 (0.03, 2.27)	**0.11 (0.01, 0.98)**	0.22 (0.02, 1.22)	0.35 (0.03, 2.77)	0.21 (0.02, 1.15)	0.24 (0.02, 1.43)	0.25 (0.03, 1.53)	0.21 (0.02, 1.19)	0.36 (0.03, 2.45)	0.27 (0.03, 1.48)	0.12 (0.01, 1.01)	0.17 (0.02, 1.12)	0.22 (0.02, 1.42)	0.26 (0.03, 1.47)
INF5mg_kgIV	**0.04 (0.00, 0.43)**	0.14 (0.01, 1.16)	**0.05 (0.00, 0.47)**	**0.11 (0.01, 0.60)**	0.17 (0.02, 1.42)	**0.10 (0.01, 0.57)**	**0.12 (0.01, 0.71)**	**0.12 (0.01, 0.74)**	**0.10 (0.01, 0.59)**	0.18 (0.02, 1.21)	**0.13 (0.02, 0.74)**	**0.06 (0.01, 0.51)**	**0.08 (0.01, 0.56)**	**0.11 (0.01, 0.70)**	**0.13 (0.01, 0.75)**
MIR1000mgIV	0.24 (0.02, 1.30)	0.81 (0.19, 3.47)	0.31 (0.05, 1.51)	0.60 (0.23, 1.48)	0.99 (0.25, 4.25)	0.59 (0.22, 1.51)	0.68 (0.23, 1.94)	0.70 (0.23, 1.94)	0.59 (0.23, 1.50)	1.02 (0.35, 2.79)	0.76 (0.29, 1.97)	0.34 (0.09, 1.26)	0.49 (0.16, 1.54)	0.63 (0.22, 1.87)	0.74 (0.28, 1.90)
MIR200mgIV	0.40 (0.05, 2.89)	1.42 (0.30, 8.12)	0.52 (0.08, 3.43)	1.04 (0.33, 3.57)	1.67 (0.36, 9.33)	1.03 (0.32, 3.41)	1.18 (0.32, 4.20)	1.21 (0.35, 4.33)	1.04 (0.32, 3.64)	1.78 (0.48, 6.30)	1.33 (0.41, 4.43)	0.62 (0.12, 2.81)	0.86 (0.22, 3.62)	1.10 (0.29, 4.15)	1.27 (0.38, 4.30)
MIR600mgIV	**0.15 (0.02, 0.96)**	0.53 (0.12, 2.48)	0.20 (0.03, 1.06)	0.40 (0.15, 1.06)	0.64 (0.16, 2.95)	0.38 (0.15, 1.05)	0.44 (0.16, 1.26)	0.46 (0.16, 1.36)	0.39 (0.15, 1.03)	0.67 (0.23, 2.05)	0.50 (0.19, 1.37)	**0.23 (0.06, 0.91)**	0.33 (0.11, 1.08)	0.41 (0.14, 1.35)	0.48 (0.19, 1.30)
NAT300mgIV	0.49 (0.07, 2.34)	1.64 (0.58, 6.39)	0.62 (0.13, 2.61)	1.25 (0.94, 1.68)	1.94 (0.77, 7.43)	1.22 (0.85, 1.72)	1.40 (0.80, 2.41)	1.45 (0.82, 2.50)	1.24 (0.86, 1.76)	**2.08 (1.11, 4.22)**	**1.58 (1.14, 2.18)**	0.72 (0.26, 2.08)	1.02 (0.53, 2.04)	1.29 (0.71, 2.53)	**1.52 (1.01, 2.23)**
NAT3mg_kgIV	0.67 (0.09, 3.62)	2.22 (0.68, 9.19)	0.86 (0.17, 3.62)	1.70 (0.93, 2.99)	2.71 (0.88, 11.17)	1.66 (0.87, 3.06)	1.90 (0.86, 4.03)	1.98 (0.90, 4.15)	1.68 (0.85, 3.16)	**2.85 (1.22, 6.79)**	**2.15 (1.14, 3.90)**	0.97 (0.30, 3.20)	1.40 (0.57, 3.26)	1.76 (0.76, 4.19)	**2.05 (1.07, 3.99)**
NAT3mg_kgIVx2	0.43 (0.05, 2.25)	1.44 (0.45, 6.12)	0.55 (0.11, 2.40)	1.11 (0.63, 1.87)	1.76 (0.59, 7.03)	1.07 (0.59, 1.87)	1.25 (0.57, 2.43)	1.28 (0.61, 2.59)	1.09 (0.58, 1.95)	1.85 (0.83, 4.25)	1.40 (0.77, 2.39)	0.63 (0.21, 1.99)	0.90 (0.38, 2.11)	1.14 (0.52, 2.57)	1.33 (0.70, 2.45)
NAT6mg_kgIVx2	0.47 (0.06, 2.46)	1.58 (0.49, 6.65)	0.60 (0.12, 2.68)	1.21 (0.66, 2.16)	1.92 (0.64, 8.00)	1.19 (0.63, 2.15)	1.38 (0.62, 2.76)	1.41 (0.65, 2.98)	1.19 (0.62, 2.29)	2.03 (0.89, 4.71)	1.55 (0.82, 2.76)	0.71 (0.22, 2.23)	0.98 (0.42, 2.48)	1.25 (0.56, 3.05)	1.47 (0.76, 2.83)
PBO	0.68 (0.09, 3.26)	2.27 (0.80, 8.53)	0.86 (0.19, 3.58)	**1.74 (1.41, 2.17)**	**2.69 (1.10, 9.83)**	**1.70 (1.28, 2.25)**	**1.95 (1.16, 3.25)**	**2.02 (1.19, 3.32)**	**1.72 (1.27, 2.36)**	**2.90 (1.58, 5.81)**	**2.21 (1.71, 2.85)**	0.99 (0.37, 2.83)	1.42 (0.77, 2.82)	**1.80 (1.02, 3.42)**	**2.10 (1.51, 2.97)**
RIS1200mgIV	0.38 (0.05, 1.79)	1.28 (0.45, 4.76)	0.49 (0.10, 2.08)	0.98 (0.73, 1.32)	1.53 (0.60, 5.63)	0.95 (0.67, 1.35)	1.10 (0.63, 1.88)	1.14 (0.65, 1.96)	0.96 (0.73, 1.30)	1.64 (0.85, 3.36)	1.24 (0.89, 1.71)	0.56 (0.21, 1.64)	0.80 (0.42, 1.59)	1.01 (0.56, 1.95)	1.19 (0.81, 1.74)
RIS200mgIV	0.52 (0.07, 2.76)	1.81 (0.50, 7.57)	0.67 (0.12, 3.35)	1.33 (0.67, 2.83)	2.15 (0.67, 9.14)	1.29 (0.63, 2.77)	1.49 (0.62, 3.55)	1.55 (0.67, 3.80)	1.31 (0.66, 2.79)	2.23 (0.89, 6.05)	1.67 (0.83, 3.53)	0.77 (0.23, 2.66)	1.09 (0.42, 2.87)	1.38 (0.56, 3.73)	1.60 (0.77, 3.61)
RIS600mgIV	0.36 (0.05, 1.67)	1.17 (0.41, 4.43)	0.45 (0.09, 1.91)	0.90 (0.68, 1.20)	1.41 (0.55, 5.33)	0.88 (0.62, 1.23)	1.01 (0.58, 1.73)	1.05 (0.60, 1.80)	0.89 (0.69, 1.15)	1.51 (0.78, 3.07)	1.14 (0.83, 1.57)	0.52 (0.19, 1.48)	0.73 (0.38, 1.47)	0.93 (0.51, 1.79)	1.09 (0.75, 1.61)
RIS600mgIV4_4	0.49 (0.07, 2.38)	1.63 (0.55, 6.34)	0.62 (0.13, 2.66)	1.25 (0.82, 1.86)	1.94 (0.74, 7.46)	1.22 (0.77, 1.87)	1.40 (0.75, 2.59)	1.44 (0.78, 2.62)	1.23 (0.77, 1.91)	**2.07 (1.02, 4.43)**	**1.58 (1.03, 2.37)**	0.71 (0.24, 2.14)	1.01 (0.50, 2.15)	1.29 (0.66, 2.66)	1.51 (0.93, 2.44)
SEC10mg_kgIV	0.94 (0.07, 9.01)	3.27 (0.41, 26.98)	1.19 (0.13, 11.24)	2.56 (0.45, 10.82)	4.04 (0.53, 32.09)	2.48 (0.42, 10.78)	2.82 (0.47, 13.45)	2.91 (0.44, 13.52)	2.51 (0.42, 10.91)	4.28 (0.72, 20.89)	3.25 (0.55, 13.82)	1.42 (0.21, 8.68)	2.08 (0.30, 10.09)	2.65 (0.40, 13.06)	3.10 (0.51, 13.69)
TOF15mgPO_BID	0.96 (0.15, 6.74)	3.48 (0.79, 18.31)	1.26 (0.20, 8.63)	2.59 (0.87, 8.13)	4.22 (0.89, 22.37)	2.50 (0.81, 8.01)	2.87 (0.82, 9.92)	2.97 (0.86, 10.03)	2.55 (0.85, 8.12)	**4.37 (1.26, 15.46)**	**3.23 (1.05, 10.64)**	1.46 (0.35, 6.18)	2.11 (0.61, 7.30)	2.71 (0.79, 9.50)	**3.14 (1.02, 10.32)**
TOF1mgPO_BID	0.45 (0.06, 2.76)	1.52 (0.39, 7.23)	0.58 (0.10, 3.07)	1.16 (0.44, 2.89)	1.88 (0.49, 8.18)	1.13 (0.42, 2.80)	1.30 (0.43, 3.63)	1.35 (0.45, 3.69)	1.14 (0.43, 2.88)	1.93 (0.63, 5.69)	1.47 (0.55, 3.60)	0.66 (0.18, 2.46)	0.94 (0.30, 2.88)	1.20 (0.40, 3.40)	1.40 (0.53, 3.58)
TOF5mgPO_BID	0.59 (0.08, 3.82)	2.09 (0.51, 10.06)	0.76 (0.13, 4.61)	1.56 (0.56, 4.26)	2.50 (0.59, 12.24)	1.52 (0.53, 4.22)	1.76 (0.53, 5.24)	1.81 (0.58, 5.19)	1.52 (0.53, 4.16)	2.61 (0.78, 8.46)	1.99 (0.70, 5.47)	0.89 (0.23, 3.26)	1.27 (0.38, 3.96)	1.62 (0.51, 4.86)	1.88 (0.68, 5.11)
UPA12mgPO_BID	0.56 (0.04, 2.23)	1.81 (0.65, 5.55)	0.70 (0.13, 2.57)	1.37 (0.35, 4.86)	2.16 (0.87, 6.58)	1.33 (0.35, 4.73)	1.55 (0.37, 5.46)	1.59 (0.38, 5.73)	1.35 (0.34, 4.96)	2.32 (0.53, 9.26)	1.73 (0.45, 6.06)	0.79 (0.15, 3.99)	1.11 (0.25, 4.71)	1.43 (0.32, 5.73)	1.64 (0.41, 6.15)

	UPA24mgPO	UPA24mgPO_BID	UPA3mgPO_BID	UPA45mgPO	UPA6mgPO_BID	UST130mgIV	UST1mg_kgIV	UST3mg_kgIV	UST4_5mg_kgIV	UST6mg_kg90mgIV_SC	UST6mg_kgIV	UST90mgSC	VED0_5mg_kgIV	VED2mg_kgIV	VED300mgIV
UPA24mgPO	UPA24mgPO	**3.22 (1.07, 33.11)**	1.27 (0.23, 11.29)	2.54 (0.53, 18.16)	**3.91 (1.34, 39.78)**	2.46 (0.52, 18.40)	2.79 (0.56, 22.96)	2.93 (0.60, 24.37)	2.50 (0.52, 18.11)	4.30 (0.85, 30.64)	3.20 (0.67, 25.15)	1.47 (0.22, 12.04)	2.05 (0.40, 17.68)	2.61 (0.51, 20.01)	3.13 (0.61, 23.65)
UPA24mgPO_BID	**0.31 (0.03, 0.93)**	UPA24mgPO_BID	0.39 (0.08, 1.07)	0.76 (0.20, 2.16)	1.20 (0.57, 2.60)	0.74 (0.20, 2.19)	0.83 (0.20, 2.66)	0.87 (0.22, 2.77)	0.75 (0.20, 2.20)	1.24 (0.31, 4.43)	0.95 (0.27, 2.82)	0.43 (0.09, 1.76)	0.60 (0.15, 2.14)	0.77 (0.19, 2.57)	0.92 (0.22, 2.77)
UPA3mgPO_BID	0.78 (0.09, 4.26)	2.58 (0.93, 11.80)	UPA3mgPO_BID	2.01 (0.49, 9.45)	**3.12 (1.21, 14.21)**	1.98 (0.48, 9.15)	2.25 (0.50, 11.12)	2.32 (0.54, 11.36)	1.98 (0.46, 9.72)	3.32 (0.79, 17.14)	2.57 (0.62, 11.82)	1.16 (0.20, 6.54)	1.64 (0.35, 8.18)	2.06 (0.44, 10.85)	2.42 (0.54, 12.16)
UPA45mgPO	0.39 (0.06, 1.89)	1.31 (0.46, 4.93)	0.50 (0.11, 2.06)	UPA45mgPO	1.55 (0.61, 5.90)	0.98 (0.68, 1.40)	1.12 (0.63, 1.96)	1.16 (0.65, 2.01)	0.99 (0.68, 1.44)	1.66 (0.87, 3.41)	1.26 (0.90, 1.75)	0.57 (0.21, 1.69)	0.82 (0.42, 1.68)	1.04 (0.56, 2.08)	1.21 (0.81, 1.81)
UPA6mgPO_BID	**0.26 (0.03, 0.75)**	0.83 (0.39, 1.76)	**0.32 (0.07, 0.82)**	0.64 (0.17, 1.64)	UPA6mgPO_BID	0.62 (0.16, 1.61)	0.70 (0.18, 2.02)	0.72 (0.19, 2.15)	0.63 (0.17, 1.67)	1.05 (0.26, 3.47)	0.81 (0.22, 2.07)	0.36 (0.07, 1.44)	0.51 (0.13, 1.66)	0.65 (0.16, 2.08)	0.76 (0.19, 2.09)
UST130mgIV	0.41 (0.05, 1.92)	1.35 (0.46, 4.90)	0.51 (0.11, 2.09)	1.03 (0.72, 1.47)	1.61 (0.62, 6.10)	UST130mgIV	1.16 (0.66, 1.89)	1.20 (0.69, 2.02)	1.01 (0.66, 1.58)	1.72 (0.85, 3.51)	**1.30 (1.03, 1.64)**	0.58 (0.20, 1.81)	0.84 (0.42, 1.72)	1.06 (0.56, 2.13)	1.25 (0.79, 1.92)
UST1mg_kgIV	0.36 (0.04, 1.79)	1.20 (0.38, 4.91)	0.44 (0.09, 2.00)	0.89 (0.51, 1.59)	1.43 (0.49, 5.71)	0.87 (0.53, 1.51)	UST1mg_kgIV	1.04 (0.61, 1.77)	0.89 (0.49, 1.62)	1.50 (0.65, 3.62)	1.13 (0.70, 1.87)	0.52 (0.17, 1.64)	0.73 (0.33, 1.69)	0.93 (0.43, 2.17)	1.08 (0.58, 2.04)
UST3mg_kgIV	0.34 (0.04, 1.67)	1.15 (0.36, 4.58)	0.43 (0.09, 1.84)	0.86 (0.50, 1.54)	1.39 (0.46, 5.34)	0.83 (0.49, 1.46)	0.96 (0.56, 1.64)	UST3mg_kgIV	0.84 (0.47, 1.60)	1.45 (0.62, 3.35)	1.09 (0.67, 1.81)	0.49 (0.16, 1.59)	0.70 (0.31, 1.68)	0.88 (0.42, 2.00)	1.04 (0.57, 1.92)
UST4_5mg_kgIV	0.40 (0.06, 1.92)	1.33 (0.46, 4.89)	0.51 (0.10, 2.16)	1.01 (0.70, 1.48)	1.59 (0.60, 5.94)	0.99 (0.63, 1.53)	1.13 (0.62, 2.06)	1.19 (0.63, 2.13)	UST4_5mg_kgIV	1.68 (0.84, 3.59)	1.28 (0.84, 1.91)	0.58 (0.21, 1.79)	0.83 (0.41, 1.74)	1.04 (0.54, 2.15)	1.23 (0.77, 1.96)
UST6mg_kg90mgIV_SC	0.23 (0.03, 1.17)	0.80 (0.23, 3.19)	0.30 (0.06, 1.26)	0.60 (0.29, 1.15)	0.95 (0.29, 3.81)	0.58 (0.28, 1.17)	0.66 (0.28, 1.55)	0.69 (0.30, 1.60)	0.60 (0.28, 1.19)	UST6mg_kg90mgIV_SC	0.76 (0.36, 1.49)	0.34 (0.10, 1.23)	0.49 (0.19, 1.23)	0.62 (0.24, 1.53)	0.73 (0.34, 1.47)
UST6mg_kgIV	0.31 (0.04, 1.50)	1.05 (0.36, 3.77)	0.39 (0.08, 1.60)	0.79 (0.57, 1.11)	1.24 (0.48, 4.64)	**0.77 (0.61, 0.97)**	0.89 (0.53, 1.42)	0.92 (0.55, 1.49)	0.78 (0.52, 1.19)	1.32 (0.67, 2.75)	UST6mg_kgIV	0.45 (0.16, 1.36)	0.64 (0.33, 1.29)	0.81 (0.44, 1.63)	0.96 (0.62, 1.45)
UST90mgSC	0.68 (0.08, 4.63)	2.32 (0.57, 11.39)	0.86 (0.15, 5.11)	1.74 (0.59, 4.85)	2.76 (0.70, 13.75)	1.72 (0.55, 4.97)	1.91 (0.61, 5.92)	2.02 (0.63, 6.32)	1.72 (0.56, 4.84)	2.95 (0.81, 9.76)	2.21 (0.74, 6.25)	UST90mgSC	1.42 (0.43, 4.67)	1.82 (0.55, 5.70)	2.10 (0.70, 6.03)
VED0_5mg_kgIV	0.49 (0.06, 2.53)	1.66 (0.47, 6.67)	0.61 (0.12, 2.88)	1.23 (0.60, 2.40)	1.97 (0.60, 7.96)	1.19 (0.58, 2.37)	1.37 (0.59, 3.07)	1.43 (0.60, 3.20)	1.21 (0.57, 2.44)	2.06 (0.81, 5.40)	1.55 (0.77, 3.03)	0.71 (0.21, 2.35)	VED0_5mg_kgIV	1.26 (0.76, 2.19)	1.49 (0.68, 3.07)
VED2mg_kgIV	0.38 (0.05, 1.97)	1.30 (0.39, 5.28)	0.48 (0.09, 2.26)	0.97 (0.48, 1.79)	1.54 (0.48, 6.12)	0.94 (0.47, 1.78)	1.08 (0.46, 2.34)	1.13 (0.50, 2.38)	0.96 (0.47, 1.84)	1.62 (0.65, 4.10)	1.23 (0.61, 2.27)	0.55 (0.18, 1.82)	0.79 (0.46, 1.31)	VED2mg_kgIV	1.17 (0.55, 2.33)
VED300mgIV	0.32 (0.04, 1.63)	1.09 (0.36, 4.47)	0.41 (0.08, 1.86)	0.82 (0.55, 1.23)	1.31 (0.48, 5.14)	0.80 (0.52, 1.27)	0.92 (0.49, 1.71)	0.96 (0.52, 1.75)	0.82 (0.51, 1.29)	1.37 (0.68, 2.96)	1.05 (0.69, 1.60)	0.48 (0.17, 1.42)	0.67 (0.33, 1.47)	0.86 (0.43, 1.80)	VED300mgIV

Table S18g: League table for clinical remission for the some concerns studies.

	ABA10mg_kgIV	ABA30mg_kgIV	ABA3mg_kgIV	ADA160mg80mgSC	ADA40mg20mgSC	ADA80mg40mgSC	AMI0_4mgPO	AND150mgSC1_1	AND150mgSC2_2	AND300mgSC	API100mgPO	API50mgPO	BRI400mgIV	BRI700mgIV	BRO210mgIV	BRO350mgIV	BRO700mgIV	CDP10mg_kgIV	CER100mgSC	CER10mg_kgIV
ABA10mg_kgIV	ABA10mg_kgIV	2.07 (0.59, 7.38)	1.22 (0.39, 4.09)	**4.37 (1.25, 15.47)**	2.42 (0.63, 9.37)	**3.92 (1.13, 14.40)**	1.22 (0.32, 4.49)	1.44 (0.31, 6.38)	1.80 (0.41, 7.83)	0.96 (0.20, 4.38)	1.30 (0.33, 4.57)	0.93 (0.24, 3.48)	**5.17 (1.22, 20.44)**	4.04 (1.00, 16.30)	1.80 (0.08, 55.26)	**10.57 (1.25, 276.69)**	6.66 (0.59, 166.90)	1.79 (0.55, 5.82)	2.00 (0.59, 6.99)	1.15 (0.23, 5.32)
ABA30mg_kgIV	0.48 (0.14, 1.70)	ABA30mg_kgIV	0.58 (0.17, 1.96)	2.06 (0.65, 7.13)	1.14 (0.33, 4.24)	1.86 (0.55, 6.64)	0.59 (0.16, 2.04)	0.68 (0.17, 3.04)	0.87 (0.20, 3.70)	0.45 (0.10, 2.17)	0.61 (0.16, 2.22)	0.44 (0.12, 1.66)	2.36 (0.59, 10.99)	1.87 (0.51, 8.27)	0.82 (0.04, 28.01)	4.77 (0.57, 129.49)	2.89 (0.31, 89.77)	0.85 (0.27, 2.73)	0.96 (0.29, 3.21)	0.56 (0.11, 2.66)
ABA3mg_kgIV	0.82 (0.24, 2.60)	1.73 (0.51, 5.90)	ABA3mg_kgIV	**3.48 (1.08, 12.51)**	1.91 (0.53, 7.44)	3.13 (0.92, 11.18)	1.02 (0.28, 3.33)	1.14 (0.28, 5.03)	1.46 (0.38, 6.08)	0.76 (0.17, 3.54)	1.03 (0.27, 3.74)	0.74 (0.19, 2.86)	4.07 (1.00, 18.31)	3.23 (0.83, 13.77)	1.39 (0.07, 42.89)	**8.36 (1.07, 252.19)**	5.16 (0.56, 146.09)	1.46 (0.46, 4.41)	1.63 (0.48, 5.19)	0.96 (0.19, 4.29)
ADA160mg80mgSC	**0.23 (0.06, 0.80)**	0.48 (0.14, 1.53)	**0.29 (0.08, 0.92)**	ADA160mg80mgSC	0.56 (0.29, 1.01)	0.90 (0.56, 1.41)	**0.29 (0.11, 0.67)**	0.34 (0.10, 1.10)	0.42 (0.14, 1.45)	**0.22 (0.06, 0.86)**	**0.30 (0.11, 0.75)**	**0.22 (0.07, 0.56)**	1.17 (0.38, 4.14)	0.93 (0.32, 2.98)	0.41 (0.02, 11.45)	2.43 (0.36, 55.80)	1.47 (0.18, 33.94)	**0.42 (0.20, 0.87)**	0.46 (0.21, 1.04)	**0.27 (0.07, 0.91)**
ADA40mg20mgSC	0.41 (0.11, 1.58)	0.88 (0.24, 3.07)	0.52 (0.13, 1.87)	1.79 (0.99, 3.41)	ADA40mg20mgSC	1.63 (0.89, 3.07)	0.52 (0.19, 1.34)	0.59 (0.17, 2.27)	0.76 (0.23, 2.67)	0.40 (0.11, 1.61)	0.54 (0.19, 1.52)	0.39 (0.13, 1.16)	2.05 (0.63, 7.72)	1.70 (0.54, 5.61)	0.75 (0.04, 26.13)	4.37 (0.62, 97.90)	2.72 (0.32, 65.11)	0.75 (0.32, 1.76)	0.84 (0.34, 2.04)	0.48 (0.13, 1.82)
ADA80mg40mgSC	**0.25 (0.07, 0.89)**	0.54 (0.15, 1.81)	0.32 (0.09, 1.09)	1.11 (0.71, 1.78)	0.61 (0.33, 1.12)	ADA80mg40mgSC	**0.32 (0.12, 0.76)**	0.37 (0.11, 1.29)	0.47 (0.15, 1.54)	**0.25 (0.07, 0.96)**	**0.33 (0.12, 0.86)**	**0.24 (0.08, 0.68)**	1.27 (0.41, 4.60)	1.04 (0.35, 3.32)	0.45 (0.03, 14.34)	2.69 (0.38, 59.57)	1.66 (0.19, 37.74)	0.46 (0.21, 1.00)	0.51 (0.22, 1.17)	0.30 (0.08, 1.04)
AMI0_4mgPO	0.82 (0.22, 3.09)	1.70 (0.49, 6.10)	0.98 (0.30, 3.55)	**3.50 (1.49, 8.80)**	1.93 (0.75, 5.24)	**3.16 (1.32, 8.14)**	AMI0_4mgPO	1.15 (0.38, 3.74)	1.46 (0.50, 4.87)	0.78 (0.22, 2.78)	1.04 (0.39, 2.80)	0.76 (0.26, 2.06)	**4.03 (1.30, 15.16)**	**3.20 (1.13, 10.94)**	1.50 (0.08, 42.58)	**8.16 (1.18, 185.83)**	4.87 (0.66, 123.75)	1.44 (0.69, 3.22)	1.61 (0.72, 3.80)	0.95 (0.25, 3.29)
AND150mgSC1_1	0.69 (0.16, 3.18)	1.47 (0.33, 5.95)	0.88 (0.20, 3.54)	2.97 (0.91, 10.21)	1.68 (0.44, 5.90)	2.73 (0.78, 9.26)	0.87 (0.27, 2.65)	AND150mgSC1_1	1.27 (0.55, 2.89)	0.68 (0.24, 1.74)	0.90 (0.27, 2.85)	0.66 (0.17, 2.21)	3.44 (0.87, 15.97)	2.80 (0.76, 11.91)	1.25 (0.07, 38.51)	7.52 (0.75, 169.06)	4.47 (0.47, 120.67)	1.26 (0.44, 3.45)	1.41 (0.41, 4.31)	0.81 (0.18, 3.33)
AND150mgSC2_2	0.55 (0.13, 2.42)	1.16 (0.27, 4.88)	0.68 (0.16, 2.65)	2.38 (0.69, 7.19)	1.32 (0.37, 4.27)	2.13 (0.65, 6.57)	0.69 (0.21, 1.99)	0.79 (0.35, 1.82)	AND150mgSC2_2	0.53 (0.20, 1.29)	0.70 (0.23, 2.25)	0.50 (0.15, 1.70)	2.69 (0.72, 11.53)	2.16 (0.62, 8.78)	1.01 (0.05, 34.67)	5.43 (0.62, 136.34)	3.49 (0.42, 98.15)	0.98 (0.35, 2.61)	1.10 (0.32, 3.02)	0.64 (0.15, 2.67)
AND300mgSC	1.04 (0.23, 5.12)	2.20 (0.46, 10.19)	1.31 (0.28, 5.90)	**4.49 (1.16, 15.48)**	2.51 (0.62, 9.15)	**4.04 (1.04, 14.50)**	1.29 (0.36, 4.47)	1.46 (0.58, 4.21)	1.90 (0.78, 4.92)	AND300mgSC	1.31 (0.38, 4.83)	0.97 (0.25, 3.88)	**5.01 (1.24, 25.98)**	**4.18 (1.05, 19.79)**	1.93 (0.10, 59.91)	**10.36 (1.13, 263.54)**	6.80 (0.72, 191.55)	1.85 (0.60, 5.81)	2.07 (0.57, 6.68)	1.23 (0.26, 5.49)
API100mgPO	0.77 (0.22, 3.01)	1.63 (0.45, 6.07)	0.97 (0.27, 3.77)	**3.35 (1.34, 8.77)**	1.87 (0.66, 5.34)	**3.05 (1.17, 8.31)**	0.96 (0.36, 2.59)	1.11 (0.35, 3.76)	1.42 (0.44, 4.39)	0.76 (0.21, 2.66)	API100mgPO	0.73 (0.30, 1.66)	**3.87 (1.17, 14.18)**	**3.17 (1.01, 10.39)**	1.41 (0.08, 42.54)	**8.15 (1.12, 189.84)**	4.98 (0.60, 128.59)	1.41 (0.61, 3.23)	1.56 (0.65, 3.83)	0.91 (0.26, 3.17)
API50mgPO	1.07 (0.29, 4.25)	2.27 (0.60, 8.56)	1.34 (0.35, 5.19)	**4.53 (1.77, 13.54)**	2.55 (0.86, 7.80)	**4.09 (1.48, 12.24)**	1.32 (0.48, 3.91)	1.52 (0.45, 5.89)	1.98 (0.59, 6.79)	1.03 (0.26, 4.01)	1.36 (0.60, 3.36)	API50mgPO	**5.37 (1.48, 20.72)**	**4.32 (1.30, 16.04)**	1.94 (0.10, 57.20)	**10.59 (1.49, 254.69)**	6.98 (0.78, 164.99)	1.87 (0.81, 5.33)	2.10 (0.85, 5.93)	1.28 (0.31, 4.57)
BRI400mgIV	**0.19 (0.05, 0.82)**	0.42 (0.09, 1.70)	0.25 (0.05, 1.00)	0.86 (0.24, 2.65)	0.49 (0.13, 1.58)	0.79 (0.22, 2.43)	**0.25 (0.07, 0.77)**	0.29 (0.06, 1.14)	0.37 (0.09, 1.39)	**0.20 (0.04, 0.80)**	**0.26 (0.07, 0.86)**	**0.19 (0.05, 0.68)**	BRI400mgIV	0.80 (0.45, 1.47)	0.34 (0.02, 11.01)	2.04 (0.23, 49.56)	1.27 (0.12, 29.47)	0.36 (0.11, 1.00)	0.41 (0.11, 1.13)	**0.23 (0.05, 0.94)**
BRI700mgIV	0.25 (0.06, 1.00)	0.53 (0.12, 1.97)	0.31 (0.07, 1.21)	1.07 (0.34, 3.08)	0.59 (0.18, 1.85)	0.97 (0.30, 2.86)	**0.31 (0.09, 0.89)**	0.36 (0.08, 1.31)	0.46 (0.11, 1.61)	**0.24 (0.05, 0.95)**	**0.32 (0.10, 0.99)**	**0.23 (0.06, 0.77)**	1.25 (0.68, 2.24)	BRI700mgIV	0.42 (0.02, 14.26)	2.49 (0.31, 56.14)	1.57 (0.17, 38.70)	0.45 (0.15, 1.14)	0.50 (0.16, 1.37)	0.29 (0.07, 1.11)
BRO210mgIV	0.55 (0.02, 12.23)	1.22 (0.04, 24.18)	0.72 (0.02, 13.50)	2.45 (0.09, 41.22)	1.33 (0.04, 22.85)	2.22 (0.07, 36.35)	0.67 (0.02, 11.94)	0.80 (0.03, 15.13)	0.99 (0.03, 19.17)	0.52 (0.02, 10.41)	0.71 (0.02, 12.44)	0.52 (0.02, 9.53)	2.94 (0.09, 53.84)	2.36 (0.07, 42.40)	BRO210mgIV	5.69 (0.91, 85.68)	3.60 (0.38, 57.15)	0.98 (0.04, 17.27)	1.08 (0.04, 20.79)	0.66 (0.02, 12.77)
BRO350mgIV	**0.09 (0.00, 0.80)**	0.21 (0.01, 1.76)	**0.12 (0.00, 0.93)**	0.41 (0.02, 2.80)	0.23 (0.01, 1.62)	0.37 (0.02, 2.63)	**0.12 (0.01, 0.85)**	0.13 (0.01, 1.34)	0.18 (0.01, 1.62)	**0.10 (0.00, 0.88)**	**0.12 (0.01, 0.89)**	**0.09 (0.00, 0.67)**	0.49 (0.02, 4.37)	0.40 (0.02, 3.26)	0.18 (0.01, 1.09)	BRO350mgIV	0.63 (0.14, 2.42)	0.18 (0.01, 1.16)	0.20 (0.01, 1.27)	**0.11 (0.00, 0.99)**
BRO700mgIV	0.15 (0.01, 1.68)	0.35 (0.01, 3.19)	0.19 (0.01, 1.80)	0.68 (0.03, 5.52)	0.37 (0.02, 3.17)	0.60 (0.03, 5.34)	0.21 (0.01, 1.51)	0.22 (0.01, 2.12)	0.29 (0.01, 2.40)	0.15 (0.01, 1.40)	0.20 (0.01, 1.65)	0.14 (0.01, 1.29)	0.79 (0.03, 8.20)	0.64 (0.03, 5.78)	0.28 (0.02, 2.62)	1.58 (0.41, 7.35)	BRO700mgIV	0.28 (0.01, 2.04)	0.33 (0.01, 2.35)	0.18 (0.01, 1.84)
CDP10mg_kgIV	0.56 (0.17, 1.82)	1.17 (0.37, 3.66)	0.68 (0.23, 2.20)	**2.41 (1.15, 5.01)**	1.34 (0.57, 3.08)	2.17 (1.00, 4.69)	0.69 (0.31, 1.45)	0.80 (0.29, 2.29)	1.02 (0.38, 2.84)	0.54 (0.17, 1.66)	0.71 (0.31, 1.63)	0.53 (0.19, 1.24)	2.76 (1.00, 8.83)	2.24 (0.88, 6.56)	1.02 (0.06, 27.86)	5.55 (0.86, 119.08)	3.53 (0.49, 82.10)	CDP10mg_kgIV	1.13 (0.58, 2.09)	0.66 (0.19, 1.96)
CER100mgSC	0.50 (0.14, 1.71)	1.04 (0.31, 3.46)	0.61 (0.19, 2.06)	2.16 (0.97, 4.83)	1.18 (0.49, 2.97)	1.95 (0.85, 4.54)	0.62 (0.26, 1.40)	0.71 (0.23, 2.45)	0.91 (0.33, 3.08)	0.48 (0.15, 1.74)	0.64 (0.26, 1.54)	0.48 (0.17, 1.18)	2.46 (0.88, 8.79)	2.01 (0.73, 6.15)	0.92 (0.05, 23.75)	5.01 (0.79, 136.01)	3.04 (0.43, 80.67)	0.88 (0.48, 1.73)	CER100mgSC	0.58 (0.16, 1.87)
CER10mg_kgIV	0.87 (0.19, 4.33)	1.80 (0.38, 8.97)	1.04 (0.23, 5.32)	**3.67 (1.10, 13.34)**	2.08 (0.55, 7.99)	3.30 (0.96, 12.65)	1.06 (0.30, 4.03)	1.24 (0.30, 5.49)	1.57 (0.37, 6.79)	0.81 (0.18, 3.83)	1.10 (0.32, 3.87)	0.78 (0.22, 3.21)	**4.28 (1.06, 19.71)**	3.42 (0.90, 14.18)	1.50 (0.08, 51.71)	**9.22 (1.01, 222.23)**	5.60 (0.54, 138.26)	1.52 (0.51, 5.20)	1.73 (0.53, 6.10)	CER10mg_kgIV

(**TABLE S18F.** continued)

	ABA10mg_kgIV	ABA30mg_kgIV	ABA3mg_kgIV	ADA160mg80mgSC	ADA40mg20mgSC	ADA80mg40mgSC	AMI0_4mgPO	AND150mgSC1_1	AND150mgSC2_2	AND300mgSC	API100mgPO	API50mgPO	BRI400mgIV	BRI700mgIV	BRO210mgIV	BRO350mgIV	BRO700mgIV	CDP10mg_kgIV	CER100mgSC	CER10mg_kgIV
CER200mgSC	0.70 (0.20, 2.67)	1.46 (0.42, 5.12)	0.88 (0.26, 3.00)	**3.03 (1.30, 7.39)**	1.67 (0.65, 4.54)	**2.72 (1.14, 6.94)**	0.88 (0.34, 2.03)	1.00 (0.31, 3.29)	1.28 (0.44, 4.16)	0.66 (0.20, 2.34)	0.90 (0.34, 2.45)	0.65 (0.23, 1.82)	**3.44 (1.18, 11.80)**	**2.81 (1.01, 8.95)**	1.27 (0.07, 35.85)	**7.15 (1.08, 162.60)**	4.28 (0.61, 109.37)	1.25 (0.61, 2.67)	1.39 (0.75, 2.82)	0.81 (0.23, 2.75)
CER20mg_kgIV	1.48 (0.32, 8.54)	3.14 (0.67, 17.01)	1.86 (0.35, 10.10)	**6.33 (1.92, 27.71)**	3.54 (0.92, 16.33)	**5.69 (1.66, 25.30)**	1.85 (0.48, 7.90)	2.18 (0.48, 10.55)	2.80 (0.61, 12.94)	1.47 (0.27, 7.79)	1.88 (0.52, 8.41)	1.41 (0.34, 6.19)	**7.52 (1.76, 41.68)**	**6.10 (1.49, 32.26)**	2.65 (0.14, 85.38)	**15.90 (1.74, 352.66)**	9.98 (0.98, 213.84)	2.69 (0.79, 10.36)	2.98 (0.88, 12.85)	1.77 (0.44, 7.55)
CER400mgSC	0.46 (0.15, 1.45)	0.96 (0.31, 2.84)	0.58 (0.19, 1.72)	**1.98 (1.08, 3.87)**	1.10 (0.51, 2.40)	1.79 (0.90, 3.63)	0.57 (0.28, 1.10)	0.65 (0.24, 1.85)	0.84 (0.34, 2.33)	0.44 (0.15, 1.37)	0.59 (0.27, 1.24)	**0.44 (0.17, 0.97)**	2.27 (0.89, 7.04)	1.84 (0.77, 5.15)	0.85 (0.05, 24.57)	4.75 (0.79, 110.43)	2.79 (0.41, 70.38)	0.83 (0.52, 1.33)	0.93 (0.56, 1.48)	0.54 (0.17, 1.56)
CER5mg_kgIV	0.56 (0.14, 2.33)	1.19 (0.28, 4.80)	0.70 (0.18, 2.74)	2.41 (0.88, 7.30)	1.33 (0.44, 4.41)	2.16 (0.73, 6.69)	0.71 (0.22, 2.06)	0.80 (0.22, 3.14)	1.03 (0.29, 3.51)	0.53 (0.14, 2.13)	0.72 (0.22, 2.23)	0.52 (0.16, 1.75)	2.76 (0.84, 11.34)	2.25 (0.71, 8.58)	1.03 (0.05, 32.89)	5.71 (0.80, 121.38)	3.62 (0.40, 79.55)	0.99 (0.40, 2.73)	1.10 (0.43, 3.19)	0.67 (0.20, 1.83)
ELD10mg_kgIV	0.49 (0.12, 2.01)	1.02 (0.25, 4.27)	0.60 (0.15, 2.54)	2.11 (0.74, 6.57)	1.18 (0.36, 3.72)	1.92 (0.64, 5.83)	0.60 (0.20, 1.76)	0.71 (0.19, 2.56)	0.91 (0.25, 3.04)	0.49 (0.12, 1.81)	0.63 (0.19, 1.95)	0.46 (0.13, 1.48)	2.51 (0.63, 10.16)	1.97 (0.57, 7.30)	0.84 (0.05, 25.87)	5.10 (0.62, 125.06)	3.09 (0.32, 84.16)	0.88 (0.33, 2.41)	0.99 (0.36, 2.72)	0.56 (0.13, 2.16)
ELD20mg_kgIV	0.39 (0.10, 1.48)	0.79 (0.21, 3.29)	0.46 (0.11, 2.00)	1.66 (0.59, 4.72)	0.92 (0.30, 2.73)	1.49 (0.51, 4.38)	0.48 (0.15, 1.47)	0.55 (0.16, 1.92)	0.70 (0.19, 2.41)	0.37 (0.09, 1.45)	0.50 (0.16, 1.46)	0.36 (0.10, 1.12)	1.92 (0.52, 7.37)	1.56 (0.44, 5.46)	0.68 (0.03, 20.93)	3.94 (0.49, 95.52)	2.45 (0.24, 64.91)	0.69 (0.26, 1.69)	0.77 (0.28, 2.07)	0.44 (0.11, 1.64)
FON0_1mg_kgIV	1.56 (0.17, 56.55)	3.36 (0.33, 98.36)	1.85 (0.22, 61.44)	6.54 (0.92, 185.24)	3.68 (0.46, 98.59)	5.97 (0.74, 171.59)	1.83 (0.24, 53.21)	2.18 (0.28, 62.91)	2.78 (0.35, 77.53)	1.48 (0.17, 41.92)	1.90 (0.27, 54.59)	1.41 (0.20, 37.41)	7.99 (0.91, 219.07)	6.33 (0.76, 159.48)	3.10 (0.08, 293.49)	**16.76 (1.17, 1101.01)**	9.90 (0.77, 812.91)	2.62 (0.41, 71.24)	2.97 (0.46, 83.56)	1.73 (0.21, 63.64)
FON1mg_kg0_1mgIV_SC	0.49 (0.12, 1.97)	1.05 (0.25, 4.37)	0.61 (0.16, 2.41)	2.18 (0.77, 6.33)	1.20 (0.38, 3.77)	1.97 (0.67, 5.52)	0.61 (0.21, 1.72)	0.71 (0.21, 2.60)	0.89 (0.27, 3.12)	0.47 (0.13, 1.85)	0.63 (0.23, 1.87)	0.46 (0.14, 1.48)	2.55 (0.68, 9.51)	2.00 (0.59, 7.31)	0.92 (0.04, 30.86)	5.21 (0.68, 125.75)	3.12 (0.36, 80.57)	0.89 (0.35, 2.26)	0.99 (0.38, 2.64)	0.57 (0.15, 2.34)
FON1mg_kg1mgIV_SC	0.40 (0.10, 1.63)	0.83 (0.20, 3.38)	0.49 (0.13, 1.96)	1.71 (0.61, 4.77)	0.95 (0.31, 2.87)	1.54 (0.54, 4.38)	0.49 (0.18, 1.30)	0.56 (0.17, 2.12)	0.71 (0.22, 2.53)	0.38 (0.10, 1.47)	0.51 (0.17, 1.48)	0.37 (0.11, 1.14)	1.99 (0.57, 7.33)	1.57 (0.50, 5.54)	0.71 (0.04, 22.60)	4.02 (0.56, 101.81)	2.48 (0.29, 65.99)	0.70 (0.29, 1.68)	0.79 (0.31, 2.01)	0.47 (0.12, 1.71)
FON1mg_kgIV	0.68 (0.10, 4.81)	1.43 (0.20, 10.33)	0.85 (0.13, 5.55)	2.93 (0.54, 17.63)	1.61 (0.27, 9.82)	2.64 (0.44, 15.99)	0.86 (0.15, 4.54)	0.96 (0.16, 6.56)	1.24 (0.21, 7.37)	0.64 (0.10, 4.39)	0.86 (0.16, 4.59)	0.63 (0.12, 3.68)	3.33 (0.58, 24.00)	2.77 (0.49, 19.13)	1.22 (0.05, 48.58)	6.76 (0.65, 195.34)	4.21 (0.39, 143.93)	1.20 (0.25, 6.41)	1.37 (0.27, 7.01)	0.79 (0.12, 4.85)
FON4mg_kg0_1mgIV_SC	0.32 (0.08, 1.23)	0.68 (0.17, 2.45)	0.40 (0.11, 1.46)	1.40 (0.51, 3.56)	0.77 (0.26, 2.12)	1.26 (0.44, 3.24)	0.40 (0.14, 1.01)	0.46 (0.14, 1.58)	0.58 (0.19, 1.88)	0.31 (0.08, 1.11)	0.42 (0.14, 1.14)	**0.30 (0.10, 0.86)**	1.60 (0.46, 5.87)	1.31 (0.39, 4.54)	0.60 (0.03, 17.00)	3.25 (0.45, 78.43)	2.00 (0.25, 50.89)	0.57 (0.24, 1.29)	0.65 (0.26, 1.55)	0.38 (0.10, 1.31)
FON4mg_kg1mgIV_SC	0.38 (0.10, 1.52)	0.82 (0.19, 3.16)	0.47 (0.12, 1.84)	1.66 (0.56, 4.66)	0.92 (0.29, 2.59)	1.49 (0.48, 4.15)	0.47 (0.17, 1.32)	0.54 (0.16, 1.94)	0.70 (0.21, 2.31)	0.37 (0.10, 1.37)	0.49 (0.17, 1.38)	0.36 (0.11, 1.09)	1.97 (0.54, 6.99)	1.57 (0.48, 5.40)	0.69 (0.04, 22.75)	3.87 (0.53, 104.78)	2.44 (0.29, 64.09)	0.70 (0.27, 1.63)	0.77 (0.28, 1.93)	0.45 (0.11, 1.58)
FON4mg_kgIV	0.35 (0.06, 1.96)	0.75 (0.12, 3.78)	0.45 (0.07, 2.05)	1.56 (0.34, 5.99)	0.85 (0.17, 3.63)	1.40 (0.28, 5.50)	0.46 (0.09, 1.69)	0.51 (0.09, 2.48)	0.66 (0.13, 3.08)	0.34 (0.06, 1.73)	0.46 (0.10, 1.83)	0.33 (0.07, 1.47)	1.74 (0.31, 9.62)	1.45 (0.28, 6.61)	0.62 (0.03, 22.89)	3.51 (0.40, 93.59)	2.19 (0.22, 65.73)	0.64 (0.15, 2.32)	0.72 (0.16, 2.55)	0.42 (0.07, 1.88)
INF10mg_kgIV	**0.08 (0.00, 0.62)**	0.15 (0.01, 1.25)	**0.09 (0.00, 0.71)**	0.33 (0.01, 2.16)	0.18 (0.01, 1.22)	0.29 (0.01, 1.96)	**0.09 (0.00, 0.61)**	**0.11 (0.00, 0.90)**	0.14 (0.01, 1.09)	**0.07 (0.00, 0.62)**	**0.10 (0.00, 0.67)**	**0.07 (0.00, 0.51)**	0.38 (0.01, 3.15)	0.30 (0.01, 2.32)	0.12 (0.00, 4.19)	0.79 (0.02, 24.57)	0.48 (0.01, 15.11)	**0.14 (0.01, 0.83)**	0.15 (0.01, 1.00)	**0.09 (0.00, 0.79)**
INF20mg_kgIV	**0.07 (0.00, 0.63)**	0.15 (0.01, 1.25)	**0.09 (0.00, 0.67)**	0.33 (0.01, 2.01)	0.18 (0.01, 1.18)	0.29 (0.01, 1.91)	**0.09 (0.00, 0.62)**	**0.11 (0.00, 0.86)**	**0.14 (0.01, 0.98)**	**0.07 (0.00, 0.59)**	**0.09 (0.00, 0.66)**	**0.07 (0.00, 0.50)**	0.37 (0.01, 3.07)	0.29 (0.01, 2.19)	0.12 (0.00, 5.16)	0.75 (0.02, 23.79)	0.44 (0.01, 15.26)	**0.13 (0.01, 0.82)**	**0.15 (0.01, 0.94)**	**0.08 (0.00, 0.71)**
INF5mg_kgIV	**0.04 (0.00, 0.27)**	**0.08 (0.00, 0.55)**	**0.04 (0.00, 0.34)**	**0.16 (0.01, 0.90)**	**0.09 (0.00, 0.56)**	**0.14 (0.01, 0.82)**	**0.04 (0.00, 0.28)**	**0.05 (0.00, 0.40)**	**0.07 (0.00, 0.47)**	**0.03 (0.00, 0.27)**	**0.05 (0.00, 0.29)**	**0.03 (0.00, 0.22)**	0.18 (0.01, 1.42)	0.15 (0.01, 1.08)	0.06 (0.00, 2.33)	0.38 (0.01, 11.48)	0.23 (0.01, 6.93)	**0.07 (0.00, 0.37)**	**0.07 (0.00, 0.42)**	**0.04 (0.00, 0.34)**
NAT300mgIV	0.41 (0.13, 1.27)	0.85 (0.28, 2.50)	0.50 (0.17, 1.53)	1.74 (0.97, 3.41)	0.97 (0.44, 2.13)	1.58 (0.78, 3.14)	**0.50 (0.24, 0.96)**	0.58 (0.22, 1.66)	0.74 (0.30, 2.08)	0.39 (0.13, 1.26)	0.52 (0.24, 1.08)	**0.38 (0.15, 0.85)**	1.99 (0.78, 5.99)	1.62 (0.69, 4.48)	0.76 (0.04, 21.48)	4.02 (0.68, 96.43)	2.53 (0.35, 59.98)	0.73 (0.46, 1.18)	0.81 (0.47, 1.42)	0.48 (0.15, 1.36)
NAT3mg_kgIV	0.54 (0.15, 1.96)	1.14 (0.31, 3.76)	0.66 (0.20, 2.38)	2.35 (0.99, 5.37)	1.29 (0.46, 3.32)	2.12 (0.81, 5.01)	0.67 (0.27, 1.56)	0.76 (0.25, 2.40)	0.98 (0.33, 2.95)	0.52 (0.15, 1.77)	0.69 (0.27, 1.74)	0.50 (0.17, 1.33)	2.61 (0.89, 9.40)	2.14 (0.75, 7.14)	0.98 (0.06, 27.67)	5.31 (0.83, 120.88)	3.36 (0.43, 82.67)	0.96 (0.46, 1.99)	1.09 (0.50, 2.39)	0.64 (0.17, 2.04)
NAT3mg_kgIVx2	0.36 (0.10, 1.23)	0.76 (0.21, 2.43)	0.44 (0.13, 1.48)	1.54 (0.68, 3.42)	0.84 (0.33, 2.13)	1.40 (0.57, 3.17)	**0.44 (0.19, 0.98)**	0.50 (0.17, 1.56)	0.66 (0.23, 1.86)	0.34 (0.10, 1.11)	0.45 (0.19, 1.12)	**0.33 (0.12, 0.86)**	1.74 (0.59, 5.92)	1.43 (0.52, 4.47)	0.64 (0.04, 17.68)	3.56 (0.55, 84.67)	2.22 (0.29, 54.87)	0.64 (0.32, 1.24)	0.72 (0.33, 1.46)	0.42 (0.12, 1.27)
NAT6mg_kgIVx2	0.39 (0.11, 1.39)	0.81 (0.24, 2.69)	0.47 (0.14, 1.61)	1.66 (0.72, 3.94)	0.90 (0.35, 2.46)	1.50 (0.61, 3.63)	0.47 (0.20, 1.09)	0.54 (0.18, 1.81)	0.70 (0.24, 2.16)	0.37 (0.11, 1.29)	0.49 (0.20, 1.24)	**0.36 (0.13, 0.95)**	1.90 (0.64, 6.60)	1.54 (0.56, 5.05)	0.70 (0.04, 19.43)	3.79 (0.57, 96.68)	2.44 (0.30, 65.56)	0.68 (0.34, 1.40)	0.77 (0.35, 1.65)	0.46 (0.13, 1.49)

	ABA10mg_kgIV	ABA30mg_kgIV	ABA3mg_kgIV	ADA160mg80mgSC	ADA40mg20mgSC	ADA80mg40mgSC	AMI0_4mgPO	AND150mgSC1_1	AND150mgSC2_2	AND300mgSC	API100mgPO	API50mgPO	BRI400mgIV	BRI700mgIV	BRO210mgIV	BRO350mgIV	BRO700mgIV	CDP10mg_kgIV	CER100mgSC	CER10mg_kgIV
PBO	0.57 (0.19, 1.74)	1.19 (0.40, 3.41)	0.70 (0.24, 2.06)	**2.43 (1.39, 4.55)**	1.35 (0.63, 2.83)	**2.21 (1.14, 4.28)**	0.70 (0.35, 1.29)	0.80 (0.31, 2.20)	1.03 (0.42, 2.72)	0.54 (0.18, 1.62)	0.72 (0.35, 1.47)	0.53 (0.21, 1.17)	**2.77 (1.11, 8.18)**	2.24 (0.98, 6.10)	1.06 (0.06, 29.35)	5.63 (0.97, 127.75)	3.51 (0.50, 83.41)	1.01 (0.68, 1.52)	1.14 (0.67, 1.87)	0.67 (0.20, 1.86)
RIS1200mgIV	0.32 (0.10, 1.00)	0.67 (0.23, 2.01)	0.40 (0.13, 1.18)	1.37 (0.75, 2.67)	0.77 (0.35, 1.66)	1.25 (0.62, 2.54)	**0.40 (0.19, 0.76)**	0.45 (0.17, 1.27)	0.58 (0.24, 1.55)	**0.31 (0.10, 0.92)**	**0.41 (0.19, 0.87)**	**0.30 (0.12, 0.68)**	1.58 (0.62, 4.83)	1.28 (0.54, 3.60)	0.60 (0.03, 16.26)	3.19 (0.56, 72.47)	1.99 (0.28, 47.95)	**0.57 (0.37, 0.92)**	0.64 (0.36, 1.11)	0.38 (0.11, 1.10)
RIS600mgIV	**0.30 (0.09, 0.93)**	0.62 (0.21, 1.84)	0.37 (0.12, 1.08)	1.27 (0.69, 2.47)	0.71 (0.33, 1.52)	1.15 (0.58, 2.34)	**0.37 (0.18, 0.70)**	0.42 (0.15, 1.17)	0.54 (0.22, 1.46)	**0.28 (0.10, 0.88)**	**0.38 (0.18, 0.80)**	**0.28 (0.11, 0.63)**	1.45 (0.57, 4.45)	1.17 (0.50, 3.33)	0.55 (0.03, 15.15)	2.95 (0.51, 69.57)	1.85 (0.26, 44.18)	**0.53 (0.34, 0.84)**	0.59 (0.34, 1.02)	0.35 (0.11, 1.02)
RIS600mgIV4_4	0.41 (0.13, 1.31)	0.85 (0.26, 2.56)	0.50 (0.16, 1.57)	1.74 (0.87, 3.56)	0.96 (0.41, 2.23)	1.58 (0.73, 3.33)	0.50 (0.22, 1.01)	0.57 (0.20, 1.65)	0.74 (0.28, 2.09)	0.39 (0.12, 1.17)	0.51 (0.22, 1.15)	**0.38 (0.14, 0.89)**	1.99 (0.74, 6.25)	1.62 (0.65, 4.62)	0.74 (0.04, 20.33)	4.12 (0.65, 91.40)	2.48 (0.34, 60.79)	0.72 (0.42, 1.24)	0.81 (0.42, 1.50)	0.48 (0.15, 1.41)
UST1mg_kgIV	0.34 (0.09, 1.27)	0.71 (0.20, 2.45)	0.42 (0.11, 1.51)	1.51 (0.60, 3.60)	0.83 (0.30, 2.21)	1.35 (0.52, 3.28)	0.43 (0.16, 1.02)	0.50 (0.15, 1.61)	0.62 (0.21, 1.95)	0.33 (0.09, 1.15)	0.44 (0.17, 1.13)	**0.33 (0.11, 0.86)**	1.74 (0.53, 5.95)	1.39 (0.43, 4.51)	0.59 (0.03, 16.91)	3.59 (0.51, 76.68)	2.11 (0.26, 53.15)	0.62 (0.28, 1.31)	0.68 (0.30, 1.55)	0.40 (0.11, 1.35)
UST3mg_kgIV	0.33 (0.09, 1.18)	0.69 (0.19, 2.40)	0.41 (0.11, 1.44)	1.42 (0.58, 3.43)	0.79 (0.29, 2.10)	1.29 (0.52, 3.11)	**0.41 (0.15, 0.97)**	0.47 (0.14, 1.46)	0.60 (0.20, 1.81)	0.32 (0.09, 1.05)	0.42 (0.16, 1.08)	**0.31 (0.10, 0.86)**	1.66 (0.49, 5.49)	1.33 (0.42, 4.14)	0.57 (0.03, 16.58)	3.41 (0.47, 75.15)	2.03 (0.24, 50.55)	0.59 (0.27, 1.25)	0.65 (0.29, 1.46)	0.38 (0.10, 1.26)
UST4_5mg_kgIV	0.33 (0.10, 1.05)	0.70 (0.22, 2.12)	0.41 (0.14, 1.26)	1.43 (0.74, 2.87)	0.79 (0.35, 1.76)	1.29 (0.62, 2.68)	**0.41 (0.19, 0.82)**	0.46 (0.17, 1.33)	0.60 (0.23, 1.65)	0.32 (0.10, 1.00)	**0.42 (0.18, 0.92)**	**0.31 (0.12, 0.73)**	1.63 (0.62, 5.08)	1.33 (0.54, 3.81)	0.61 (0.03, 17.02)	3.32 (0.56, 76.85)	2.06 (0.28, 50.68)	0.59 (0.35, 1.02)	0.67 (0.35, 1.22)	0.39 (0.12, 1.17)
UST6mg_kgIV	0.33 (0.09, 1.20)	0.69 (0.18, 2.46)	0.41 (0.11, 1.45)	1.43 (0.59, 3.37)	0.80 (0.29, 2.02)	1.30 (0.51, 3.07)	**0.41 (0.16, 0.97)**	0.48 (0.14, 1.54)	0.60 (0.18, 1.88)	0.32 (0.08, 1.10)	0.43 (0.16, 1.09)	**0.31 (0.10, 0.83)**	1.67 (0.50, 5.63)	1.33 (0.42, 4.16)	0.57 (0.03, 17.17)	3.40 (0.49, 77.52)	2.02 (0.24, 50.72)	0.60 (0.26, 1.24)	0.66 (0.29, 1.44)	0.38 (0.10, 1.30)
UST90mgSC	0.56 (0.12, 2.54)	1.16 (0.26, 5.20)	0.70 (0.15, 2.95)	2.45 (0.72, 7.87)	1.35 (0.36, 4.82)	2.24 (0.62, 7.33)	0.69 (0.19, 2.36)	0.80 (0.19, 3.35)	1.02 (0.25, 3.90)	0.53 (0.12, 2.24)	0.72 (0.19, 2.56)	0.52 (0.13, 1.96)	2.82 (0.62, 12.93)	2.27 (0.53, 9.98)	0.97 (0.04, 32.66)	5.94 (0.65, 127.70)	3.70 (0.36, 92.15)	1.01 (0.31, 3.07)	1.12 (0.34, 3.62)	0.66 (0.13, 2.79)
VED0_5mg_kgIV	0.43 (0.11, 1.50)	0.87 (0.23, 3.23)	0.51 (0.14, 1.84)	1.82 (0.73, 4.44)	1.00 (0.36, 2.71)	1.64 (0.64, 4.12)	0.52 (0.19, 1.43)	0.60 (0.19, 1.92)	0.75 (0.25, 2.39)	0.40 (0.11, 1.39)	0.54 (0.19, 1.44)	0.39 (0.13, 1.09)	2.10 (0.63, 7.16)	1.67 (0.55, 5.40)	0.75 (0.04, 20.40)	4.45 (0.61, 100.04)	2.58 (0.35, 62.59)	0.75 (0.33, 1.57)	0.84 (0.37, 1.87)	0.49 (0.12, 1.64)
VED2mg_kgIV	0.33 (0.09, 1.11)	0.66 (0.19, 2.40)	0.39 (0.12, 1.33)	1.39 (0.58, 3.40)	0.76 (0.29, 1.97)	1.24 (0.51, 3.12)	0.39 (0.15, 1.08)	0.46 (0.14, 1.59)	0.58 (0.20, 1.92)	0.31 (0.09, 1.16)	0.41 (0.15, 1.05)	**0.30 (0.10, 0.81)**	1.61 (0.49, 5.68)	1.29 (0.44, 4.11)	0.58 (0.03, 15.36)	3.35 (0.49, 73.49)	1.98 (0.26, 48.32)	0.57 (0.27, 1.22)	0.64 (0.28, 1.40)	0.38 (0.10, 1.22)
VED300mgIV	0.32 (0.08, 1.33)	0.66 (0.16, 2.67)	0.39 (0.10, 1.55)	1.40 (0.46, 4.02)	0.78 (0.23, 2.35)	1.27 (0.39, 3.58)	0.40 (0.13, 1.10)	0.46 (0.12, 1.71)	0.57 (0.17, 2.09)	0.30 (0.08, 1.22)	0.41 (0.12, 1.31)	**0.30 (0.09, 0.99)**	1.56 (0.44, 6.62)	1.26 (0.37, 5.04)	0.58 (0.03, 16.12)	3.13 (0.44, 79.03)	1.93 (0.25, 54.48)	0.57 (0.21, 1.43)	0.64 (0.23, 1.72)	0.38 (0.09, 1.38)

(**TABLE S18G.** continued)

	CER200mgSC	CER20mg_kgIV	CER400mgSC	CER5mg_kgIV	ELD10mg_kgIV	ELD20mg_kgIV	FON0_1mg_kgIV	FON1mg_kg0_1mgIV_SC	FON1mg_kg1mgIV_SC	FON1mg_kgIV	FON4mg_kg0_1mgIV_SC	FON4mg_kg1mgIV_SC	FON4mg_kgIV	INF10mg_kgIV	INF20mg_kgIV	INF5mg_kgIV	NAT300mgIV	NAT3mg_kgIV	NAT3mg_kgIVx2	NAT6mg_kgIVx2
ABA10mg_kgIV	1.42 (0.37, 4.94)	0.68 (0.12, 3.13)	2.17 (0.69, 6.65)	1.78 (0.43, 7.39)	2.05 (0.50, 8.60)	2.55 (0.68, 10.50)	0.64 (0.02, 5.73)	2.04 (0.51, 8.03)	2.50 (0.61, 10.38)	1.48 (0.21, 9.83)	3.13 (0.82, 12.11)	2.62 (0.66, 10.49)	2.83 (0.51, 15.65)	**12.95 (1.60, 323.02)**	**13.48 (1.59, 344.17)**	**26.90 (3.67, 628.66)**	2.45 (0.79, 7.69)	1.86 (0.51, 6.58)	2.79 (0.82, 9.83)	2.59 (0.72, 8.80)
ABA30mg_kgIV	0.69 (0.20, 2.39)	0.32 (0.06, 1.48)	1.04 (0.35, 3.18)	0.84 (0.21, 3.55)	0.98 (0.23, 3.99)	1.26 (0.30, 4.87)	0.30 (0.01, 3.01)	0.95 (0.23, 3.95)	1.20 (0.30, 4.92)	0.70 (0.10, 4.94)	1.47 (0.41, 5.98)	1.23 (0.32, 5.36)	1.34 (0.26, 8.07)	6.50 (0.80, 149.82)	6.57 (0.80, 152.35)	**13.26 (1.82, 288.42)**	1.18 (0.40, 3.58)	0.88 (0.27, 3.22)	1.32 (0.41, 4.68)	1.23 (0.37, 4.22)
ABA3mg_kgIV	1.14 (0.33, 3.92)	0.54 (0.10, 2.86)	1.73 (0.58, 5.22)	1.42 (0.36, 5.58)	1.66 (0.39, 6.60)	2.16 (0.50, 8.77)	0.54 (0.02, 4.65)	1.64 (0.41, 6.36)	2.04 (0.51, 7.95)	1.18 (0.18, 7.85)	2.51 (0.68, 9.37)	2.14 (0.54, 8.16)	2.23 (0.49, 13.65)	**10.95 (1.42, 273.68)**	**11.09 (1.49, 271.35)**	**22.74 (2.93, 532.91)**	1.99 (0.66, 5.93)	1.51 (0.42, 5.09)	2.28 (0.67, 7.62)	2.12 (0.62, 7.08)
ADA160mg80mgSC	**0.33 (0.14, 0.77)**	**0.16 (0.04, 0.52)**	**0.50 (0.26, 0.93)**	0.42 (0.14, 1.14)	0.47 (0.15, 1.36)	0.60 (0.21, 1.68)	0.15 (0.01, 1.09)	0.46 (0.16, 1.30)	0.59 (0.21, 1.63)	0.34 (0.06, 1.85)	0.71 (0.28, 1.95)	0.60 (0.21, 1.79)	0.64 (0.17, 2.97)	3.01 (0.46, 71.50)	3.06 (0.50, 74.04)	**6.31 (1.11, 141.83)**	0.57 (0.29, 1.03)	0.42 (0.19, 1.01)	0.65 (0.29, 1.46)	0.60 (0.25, 1.39)
ADA40mg20mgSC	0.60 (0.22, 1.53)	0.28 (0.06, 1.08)	0.91 (0.42, 1.97)	0.75 (0.23, 2.29)	0.85 (0.27, 2.77)	1.08 (0.37, 3.32)	0.27 (0.01, 2.16)	0.84 (0.27, 2.64)	1.05 (0.35, 3.22)	0.62 (0.10, 3.71)	1.31 (0.47, 3.90)	1.09 (0.39, 3.44)	1.17 (0.28, 5.81)	5.63 (0.82, 117.83)	5.58 (0.85, 124.60)	**11.43 (1.78, 252.20)**	1.03 (0.47, 2.25)	0.77 (0.30, 2.15)	1.19 (0.47, 3.01)	1.11 (0.41, 2.85)
ADA80mg40mgSC	**0.37 (0.14, 0.88)**	**0.18 (0.04, 0.60)**	0.56 (0.28, 1.11)	0.46 (0.15, 1.37)	0.52 (0.17, 1.56)	0.67 (0.23, 1.95)	0.17 (0.01, 1.35)	0.51 (0.18, 1.49)	0.65 (0.23, 1.87)	0.38 (0.06, 2.28)	0.79 (0.31, 2.25)	0.67 (0.24, 2.07)	0.71 (0.18, 3.51)	3.40 (0.51, 77.21)	3.47 (0.52, 75.93)	**7.00 (1.21, 152.69)**	0.63 (0.32, 1.28)	0.47 (0.20, 1.24)	0.71 (0.32, 1.75)	0.67 (0.28, 1.64)
AMI0_4mgPO	1.13 (0.49, 2.91)	0.54 (0.13, 2.08)	1.74 (0.91, 3.62)	1.41 (0.48, 4.47)	1.65 (0.57, 5.09)	2.10 (0.68, 6.54)	0.55 (0.02, 4.10)	1.65 (0.58, 4.67)	2.02 (0.77, 5.69)	1.17 (0.22, 6.62)	2.49 (0.99, 6.90)	2.11 (0.76, 5.89)	2.20 (0.59, 10.70)	**11.02 (1.64, 247.92)**	**11.15 (1.62, 246.33)**	**22.28 (3.57, 483.09)**	**1.99 (1.04, 4.18)**	1.48 (0.64, 3.64)	**2.27 (1.03, 5.38)**	2.11 (0.92, 5.02)
AND150mgSC1_1	1.00 (0.30, 3.19)	0.46 (0.09, 2.09)	1.53 (0.54, 4.15)	1.26 (0.32, 4.52)	1.41 (0.39, 5.18)	1.83 (0.52, 6.43)	0.46 (0.02, 3.63)	1.40 (0.38, 4.82)	1.78 (0.47, 6.01)	1.05 (0.15, 6.37)	2.19 (0.63, 7.06)	1.86 (0.51, 6.07)	1.95 (0.40, 10.74)	**9.44 (1.11, 225.85)**	**9.50 (1.17, 222.61)**	**19.40 (2.50, 424.13)**	1.73 (0.60, 4.63)	1.31 (0.42, 3.99)	2.00 (0.64, 5.98)	1.85 (0.55, 5.47)
AND150mgSC2_2	0.78 (0.24, 2.26)	0.36 (0.08, 1.63)	1.18 (0.43, 2.96)	0.97 (0.28, 3.45)	1.10 (0.33, 3.98)	1.43 (0.42, 5.18)	0.36 (0.01, 2.87)	1.12 (0.32, 3.72)	1.41 (0.40, 4.62)	0.80 (0.14, 4.69)	1.72 (0.53, 5.31)	1.44 (0.43, 4.68)	1.52 (0.33, 7.96)	7.29 (0.92, 166.21)	**7.39 (1.02, 164.22)**	**14.92 (2.15, 316.88)**	1.35 (0.48, 3.39)	1.02 (0.34, 3.01)	1.53 (0.54, 4.31)	1.43 (0.46, 4.14)
AND300mgSC	1.51 (0.43, 5.08)	0.68 (0.13, 3.71)	2.25 (0.73, 6.62)	1.88 (0.47, 7.34)	2.04 (0.55, 8.48)	2.70 (0.69, 10.54)	0.67 (0.02, 5.76)	2.11 (0.54, 7.92)	2.65 (0.68, 9.92)	1.57 (0.23, 10.38)	3.26 (0.90, 11.81)	2.71 (0.73, 10.10)	2.91 (0.58, 17.85)	**14.12 (1.62, 339.63)**	**14.02 (1.71, 366.30)**	**28.86 (3.74, 685.41)**	2.54 (0.79, 7.86)	1.93 (0.57, 6.79)	2.93 (0.90, 9.55)	2.70 (0.78, 9.12)
API100mgPO	1.11 (0.41, 2.92)	0.53 (0.12, 1.94)	1.69 (0.81, 3.69)	1.39 (0.45, 4.56)	1.58 (0.51, 5.22)	2.01 (0.68, 6.25)	0.53 (0.02, 3.70)	1.59 (0.54, 4.44)	1.95 (0.68, 5.93)	1.16 (0.22, 6.25)	2.40 (0.88, 7.05)	2.05 (0.72, 5.86)	2.18 (0.55, 10.49)	**10.31 (1.48, 240.36)**	**10.68 (1.51, 235.07)**	**21.18 (3.51, 443.84)**	1.94 (0.93, 4.16)	1.45 (0.57, 3.74)	2.23 (0.89, 5.28)	2.04 (0.81, 5.01)
API50mgPO	1.55 (0.55, 4.37)	0.71 (0.16, 2.96)	**2.29 (1.03, 5.83)**	1.94 (0.57, 6.42)	2.19 (0.67, 7.81)	2.78 (0.89, 9.56)	0.71 (0.03, 5.01)	2.16 (0.67, 7.11)	2.69 (0.88, 8.73)	1.60 (0.27, 8.57)	**3.30 (1.16, 10.41)**	2.79 (0.92, 9.16)	3.07 (0.68, 14.83)	**14.21 (1.96, 393.92)**	**14.85 (2.02, 374.78)**	**29.09 (4.57, 737.84)**	**2.60 (1.17, 6.65)**	2.00 (0.75, 5.73)	**3.02 (1.17, 8.40)**	**2.75 (1.05, 7.99)**
BRI400mgIV	**0.29 (0.08, 0.85)**	**0.13 (0.02, 0.57)**	0.44 (0.14, 1.13)	0.36 (0.09, 1.19)	0.40 (0.10, 1.59)	0.52 (0.14, 1.92)	0.13 (0.00, 1.10)	0.39 (0.11, 1.46)	0.50 (0.14, 1.77)	0.30 (0.04, 1.74)	0.63 (0.17, 2.18)	0.51 (0.14, 1.86)	0.58 (0.10, 3.22)	2.66 (0.32, 71.29)	2.73 (0.33, 68.81)	5.52 (0.71, 144.16)	0.50 (0.17, 1.29)	0.38 (0.11, 1.13)	0.57 (0.17, 1.71)	0.53 (0.15, 1.55)
BRI700mgIV	**0.36 (0.11, 0.99)**	**0.16 (0.03, 0.67)**	0.54 (0.19, 1.30)	0.44 (0.12, 1.40)	0.51 (0.14, 1.77)	0.64 (0.18, 2.29)	0.16 (0.01, 1.31)	0.50 (0.14, 1.69)	0.64 (0.18, 2.01)	0.36 (0.05, 2.03)	0.76 (0.22, 2.54)	0.64 (0.19, 2.10)	0.69 (0.15, 3.53)	3.35 (0.43, 82.46)	3.42 (0.46, 85.84)	6.81 (0.93, 177.32)	0.62 (0.22, 1.45)	0.47 (0.14, 1.33)	0.70 (0.22, 1.93)	0.65 (0.20, 1.78)
BRO210mgIV	0.78 (0.03, 13.47)	0.38 (0.01, 7.26)	1.18 (0.04, 20.15)	0.97 (0.03, 18.43)	1.20 (0.04, 21.77)	1.48 (0.05, 29.99)	0.32 (0.00, 11.79)	1.09 (0.03, 22.43)	1.40 (0.04, 27.39)	0.82 (0.02, 18.34)	1.66 (0.06, 33.04)	1.45 (0.04, 25.79)	1.61 (0.04, 33.76)	8.35 (0.24, 659.68)	8.56 (0.19, 506.27)	16.50 (0.43, 1111.75)	1.32 (0.05, 23.03)	1.02 (0.04, 17.22)	1.55 (0.06, 27.99)	1.43 (0.05, 26.73)
BRO350mgIV	**0.14 (0.01, 0.92)**	**0.06 (0.00, 0.57)**	0.21 (0.01, 1.27)	0.18 (0.01, 1.25)	0.20 (0.01, 1.61)	0.25 (0.01, 2.03)	**0.06 (0.00, 0.86)**	0.19 (0.01, 1.48)	0.25 (0.01, 1.77)	0.15 (0.01, 1.54)	0.31 (0.01, 2.22)	0.26 (0.01, 1.87)	0.29 (0.01, 2.49)	1.26 (0.04, 43.35)	1.33 (0.04, 44.68)	2.60 (0.09, 87.58)	0.25 (0.01, 1.46)	0.19 (0.01, 1.21)	0.28 (0.01, 1.83)	0.26 (0.01, 1.76)
BRO700mgIV	0.23 (0.01, 1.64)	0.10 (0.00, 1.02)	0.36 (0.01, 2.43)	0.28 (0.01, 2.53)	0.32 (0.01, 3.17)	0.41 (0.02, 4.14)	0.10 (0.00, 1.29)	0.32 (0.01, 2.79)	0.40 (0.02, 3.40)	0.24 (0.01, 2.56)	0.50 (0.02, 3.96)	0.41 (0.02, 3.45)	0.46 (0.02, 4.59)	2.07 (0.07, 83.76)	2.27 (0.07, 78.95)	4.34 (0.14, 161.23)	0.39 (0.02, 2.83)	0.30 (0.01, 2.35)	0.45 (0.02, 3.46)	0.41 (0.02, 3.37)
CDP10mg_kgIV	0.80 (0.37, 1.64)	0.37 (0.10, 1.26)	1.21 (0.75, 1.91)	1.01 (0.37, 2.52)	1.13 (0.42, 3.02)	1.45 (0.59, 3.84)	0.38 (0.01, 2.41)	1.12 (0.44, 2.87)	1.42 (0.60, 3.50)	0.84 (0.16, 3.97)	1.74 (0.78, 4.10)	1.44 (0.61, 3.73)	1.56 (0.43, 6.65)	**7.40 (1.21, 172.82)**	**7.41 (1.22, 154.29)**	**15.26 (2.67, 310.99)**	1.38 (0.85, 2.17)	1.04 (0.50, 2.18)	1.57 (0.80, 3.11)	1.47 (0.72, 2.93)
CER100mgSC	0.72 (0.35, 1.33)	0.34 (0.08, 1.14)	1.08 (0.68, 1.77)	0.91 (0.31, 2.32)	1.01 (0.37, 2.82)	1.30 (0.48, 3.56)	0.34 (0.01, 2.16)	1.01 (0.38, 2.62)	1.26 (0.50, 3.26)	0.73 (0.14, 3.76)	1.54 (0.65, 3.87)	1.29 (0.52, 3.57)	1.38 (0.39, 6.30)	6.56 (1.00, 180.24)	**6.66 (1.06, 161.14)**	**13.65 (2.36, 335.97)**	1.23 (0.70, 2.14)	0.92 (0.42, 2.02)	1.39 (0.68, 3.08)	1.31 (0.61, 2.84)
CER10mg_kgIV	1.23 (0.36, 4.39)	0.56 (0.13, 2.28)	1.84 (0.64, 6.00)	1.50 (0.55, 4.95)	1.77 (0.46, 7.53)	2.27 (0.61, 9.51)	0.58 (0.02, 4.84)	1.77 (0.43, 6.70)	2.14 (0.59, 8.43)	1.27 (0.21, 8.34)	2.66 (0.76, 9.99)	2.24 (0.63, 8.95)	2.39 (0.53, 13.78)	**11.67 (1.27, 309.44)**	**12.06 (1.41, 301.34)**	**23.44 (2.97, 584.78)**	2.08 (0.74, 6.88)	1.56 (0.49, 5.94)	2.38 (0.79, 8.60)	2.18 (0.67, 7.92)

(**TABLE S18G.** continued)

	CER200mg SC	CER20mg_kgIV	CER400mg SC	CER5mg_kg IV	ELD10mg_kgIV	ELD20mg_kgIV	FON0_1mg_kgIV	FON1mg_kg0_1mgIV_SC	FON1mg_kg1mgIV_SC	FON1mg_kg IV	FON4mg_kg0_1mgIV_SC	FON4mg_kg1mgIV_SC	FON4mg_kg IV	INF10mg_kg IV	INF20mg_kg IV	INF5mg_kgIV	NAT300mgIV	NAT3mg_kgIV	NAT3mg_kgIVx2	NAT6mg_kgIVx2
CER200mg SC	CER200mg SC	0.47 (0.11, 1.68)	1.51 (0.85, 2.84)	1.26 (0.42, 3.60)	1.42 (0.48, 4.37)	1.81 (0.68, 5.37)	0.47 (0.02, 3.22)	1.40 (0.50, 4.00)	1.79 (0.64, 4.94)	1.03 (0.18, 5.33)	2.18 (0.86, 5.98)	1.85 (0.68, 5.04)	1.91 (0.53, 9.73)	**9.36 (1.43, 215.61)**	**9.39 (1.50, 224.08)**	**18.66 (3.25, 450.23)**	1.72 (0.91, 3.44)	1.28 (0.56, 3.21)	1.95 (0.90, 4.65)	1.81 (0.80, 4.29)
CER20mg_kgIV	2.13 (0.60, 9.17)	CER20mg_kgIV	**3.17 (1.01, 12.64)**	2.70 (0.83, 10.11)	3.03 (0.73, 16.69)	3.87 (1.00, 20.72)	0.98 (0.03, 10.43)	2.95 (0.75, 14.45)	3.76 (0.97, 18.15)	2.30 (0.29, 18.32)	**4.65 (1.17, 21.27)**	**3.95 (1.01, 18.84)**	4.38 (0.73, 31.44)	**20.56 (2.04, 451.83)**	**21.26 (2.03, 438.85)**	**42.22 (4.37, 873.78)**	**3.65 (1.18, 13.74)**	2.76 (0.82, 11.67)	**4.16 (1.26, 17.88)**	**3.86 (1.12, 16.55)**
CER400mg SC	0.66 (0.35, 1.18)	**0.32 (0.08, 0.99)**	CER400mg SC	0.84 (0.32, 1.98)	0.95 (0.37, 2.40)	1.19 (0.51, 3.00)	0.32 (0.01, 2.03)	0.93 (0.40, 2.26)	1.17 (0.52, 2.68)	0.68 (0.13, 3.20)	1.42 (0.68, 3.21)	1.22 (0.54, 2.85)	1.29 (0.39, 5.26)	6.15 (0.99, 138.53)	**6.16 (1.04, 128.32)**	**12.41 (2.19, 253.99)**	1.14 (0.83, 1.58)	0.86 (0.46, 1.72)	1.31 (0.74, 2.40)	1.21 (0.63, 2.25)
CER5mg_kg IV	0.79 (0.28, 2.35)	0.37 (0.10, 1.21)	1.19 (0.51, 3.13)	CER5mg_kg IV	1.15 (0.32, 4.05)	1.46 (0.45, 5.17)	0.38 (0.01, 3.00)	1.13 (0.36, 3.99)	1.39 (0.45, 5.11)	0.82 (0.13, 4.97)	1.71 (0.59, 5.99)	1.42 (0.47, 5.18)	1.58 (0.34, 8.64)	7.57 (0.90, 190.14)	7.80 (0.90, 171.62)	**15.49 (2.10, 359.80)**	1.36 (0.57, 3.57)	1.02 (0.37, 3.12)	1.56 (0.59, 4.56)	1.45 (0.53, 4.32)
ELD10mg_kgIV	0.71 (0.23, 2.07)	0.33 (0.06, 1.37)	1.06 (0.42, 2.69)	0.87 (0.25, 3.17)	ELD10mg_kgIV	1.28 (0.57, 2.81)	0.31 (0.01, 2.53)	0.97 (0.28, 3.25)	1.24 (0.36, 4.13)	0.74 (0.12, 3.96)	1.51 (0.47, 5.24)	1.29 (0.38, 4.43)	1.39 (0.30, 6.77)	6.35 (0.78, 157.20)	6.51 (0.85, 155.96)	**12.89 (1.89, 296.33)**	1.21 (0.47, 3.03)	0.91 (0.31, 2.63)	1.39 (0.48, 3.89)	1.28 (0.45, 3.63)
ELD20mg_kgIV	0.55 (0.19, 1.46)	0.26 (0.05, 1.00)	0.84 (0.33, 1.96)	0.68 (0.19, 2.23)	0.78 (0.36, 1.74)	ELD20mg_kgIV	0.24 (0.01, 1.83)	0.79 (0.23, 2.33)	0.96 (0.29, 3.04)	0.55 (0.09, 3.33)	1.20 (0.36, 3.63)	1.00 (0.30, 3.13)	1.09 (0.24, 5.13)	4.89 (0.62, 127.90)	5.12 (0.68, 129.26)	**10.23 (1.47, 238.23)**	0.95 (0.38, 2.25)	0.71 (0.26, 1.99)	1.09 (0.39, 2.96)	1.00 (0.36, 2.71)
FON0_1mg_kgIV	2.11 (0.31, 61.09)	1.02 (0.10, 36.53)	3.14 (0.49, 90.98)	2.65 (0.33, 91.02)	3.17 (0.40, 87.51)	4.08 (0.55, 120.03)	FON0_1mg_kgIV	3.03 (0.41, 84.40)	3.83 (0.51, 95.34)	2.38 (0.31, 57.50)	4.59 (0.67, 129.66)	4.05 (0.53, 106.18)	4.37 (0.87, 99.75)	**25.33 (1.02, 1134.17)**	**24.99 (1.14, 1236.75)**	**49.80 (2.53, 2441.13)**	3.62 (0.56, 98.73)	2.87 (0.38, 82.35)	4.29 (0.59, 116.63)	4.01 (0.53, 102.48)
FON1mg_kg0_1mgIV_SC	0.71 (0.25, 2.00)	0.34 (0.07, 1.34)	1.08 (0.44, 2.53)	0.89 (0.25, 2.77)	1.03 (0.31, 3.56)	1.27 (0.43, 4.37)	0.33 (0.01, 2.46)	FON1mg_kg0_1mgIV_SC	1.26 (0.56, 2.67)	0.73 (0.12, 4.36)	1.53 (0.78, 3.18)	1.31 (0.61, 2.76)	1.40 (0.34, 6.94)	6.41 (0.93, 153.66)	6.67 (0.98, 160.59)	**13.03 (1.87, 329.52)**	1.22 (0.52, 2.97)	0.91 (0.33, 2.64)	1.39 (0.55, 4.06)	1.28 (0.48, 3.76)
FON1mg_kg1mgIV_SC	0.56 (0.20, 1.57)	0.27 (0.06, 1.03)	0.85 (0.37, 1.94)	0.72 (0.20, 2.23)	0.81 (0.24, 2.80)	1.04 (0.33, 3.45)	0.26 (0.01, 1.95)	0.80 (0.38, 1.78)	FON1mg_kg1mgIV_SC	0.58 (0.09, 3.12)	1.23 (0.64, 2.50)	1.03 (0.51, 2.14)	1.10 (0.28, 5.24)	5.16 (0.73, 128.92)	5.36 (0.74, 132.00)	**10.42 (1.49, 256.34)**	0.98 (0.42, 2.19)	0.74 (0.28, 2.06)	1.12 (0.42, 2.93)	1.03 (0.39, 2.78)
FON1mg_kg IV	0.97 (0.19, 5.71)	0.44 (0.05, 3.44)	1.47 (0.31, 7.52)	1.22 (0.20, 7.58)	1.35 (0.25, 8.29)	1.81 (0.30, 10.96)	0.42 (0.02, 3.25)	1.38 (0.23, 8.22)	1.73 (0.32, 10.54)	FON1mg_kg IV	2.09 (0.39, 12.68)	1.76 (0.32, 11.30)	1.81 (0.59, 8.46)	9.50 (0.80, 293.44)	9.74 (0.81, 291.27)	**19.36 (1.71, 533.40)**	1.65 (0.35, 8.35)	1.26 (0.24, 7.20)	1.94 (0.38, 10.57)	1.80 (0.33, 9.63)
FON4mg_kg0_1mgIV_SC	0.46 (0.17, 1.17)	**0.21 (0.05, 0.85)**	0.70 (0.31, 1.47)	0.58 (0.17, 1.69)	0.66 (0.19, 2.13)	0.84 (0.28, 2.76)	0.22 (0.01, 1.50)	0.65 (0.31, 1.28)	0.81 (0.40, 1.57)	0.48 (0.08, 2.58)	FON4mg_kg0_1mgIV_SC	0.84 (0.42, 1.61)	0.91 (0.23, 4.23)	4.22 (0.62, 98.05)	4.36 (0.63, 97.61)	**8.47 (1.28, 190.11)**	0.80 (0.35, 1.68)	0.60 (0.23, 1.54)	0.91 (0.35, 2.20)	0.85 (0.32, 2.11)
FON4mg_kg1mgIV_SC	0.54 (0.20, 1.47)	**0.25 (0.05, 0.99)**	0.82 (0.35, 1.85)	0.71 (0.19, 2.14)	0.77 (0.23, 2.62)	1.00 (0.32, 3.37)	0.25 (0.01, 1.89)	0.77 (0.36, 1.63)	0.97 (0.47, 1.94)	0.57 (0.09, 3.12)	1.19 (0.62, 2.36)	FON4mg_kg1mgIV_SC	1.08 (0.27, 5.12)	4.98 (0.61, 116.12)	5.20 (0.67, 117.56)	**10.10 (1.39, 223.87)**	0.95 (0.40, 2.14)	0.71 (0.26, 1.94)	1.06 (0.42, 2.78)	1.00 (0.38, 2.70)
FON4mg_kg IV	0.52 (0.10, 1.90)	0.23 (0.03, 1.38)	0.78 (0.19, 2.56)	0.63 (0.12, 2.98)	0.72 (0.15, 3.31)	0.92 (0.19, 4.18)	0.23 (0.01, 1.15)	0.71 (0.14, 2.92)	0.91 (0.19, 3.59)	0.55 (0.12, 1.69)	1.10 (0.24, 4.40)	0.93 (0.20, 3.68)	FON4mg_kg IV	4.95 (0.42, 102.70)	5.04 (0.45, 112.05)	9.80 (0.96, 222.18)	0.88 (0.21, 2.97)	0.66 (0.14, 2.51)	1.01 (0.22, 3.65)	0.96 (0.20, 3.44)
INF10mg_kg IV	**0.11 (0.00, 0.70)**	**0.05 (0.00, 0.49)**	0.16 (0.01, 1.01)	0.13 (0.01, 1.11)	0.16 (0.01, 1.28)	0.20 (0.01, 1.61)	**0.04 (0.00, 0.98)**	0.16 (0.01, 1.07)	0.19 (0.01, 1.37)	0.11 (0.00, 1.25)	0.24 (0.01, 1.60)	0.20 (0.01, 1.64)	0.20 (0.01, 2.40)	INF10mg_kg IV	1.01 (0.37, 2.75)	2.00 (0.94, 4.88)	0.19 (0.01, 1.17)	**0.14 (0.01, 0.96)**	0.21 (0.01, 1.45)	0.19 (0.01, 1.38)
INF20mg_kg IV	**0.11 (0.00, 0.67)**	**0.05 (0.00, 0.49)**	**0.16 (0.01, 0.96)**	0.13 (0.01, 1.11)	0.15 (0.01, 1.18)	0.20 (0.01, 1.46)	**0.04 (0.00, 0.87)**	0.15 (0.01, 1.02)	0.19 (0.01, 1.35)	0.10 (0.00, 1.23)	0.23 (0.01, 1.59)	0.19 (0.01, 1.49)	0.20 (0.01, 2.20)	0.99 (0.36, 2.67)	INF20mg_kg IV	1.97 (0.93, 4.92)	0.19 (0.01, 1.10)	**0.14 (0.01, 0.93)**	0.21 (0.01, 1.36)	0.19 (0.01, 1.27)
INF5mg_kgIV	**0.05 (0.00, 0.31)**	**0.02 (0.00, 0.23)**	**0.08 (0.00, 0.46)**	**0.06 (0.00, 0.48)**	**0.08 (0.00, 0.53)**	**0.10 (0.00, 0.68)**	**0.02 (0.00, 0.40)**	**0.08 (0.00, 0.53)**	**0.10 (0.00, 0.67)**	**0.05 (0.00, 0.59)**	**0.12 (0.01, 0.78)**	**0.10 (0.00, 0.72)**	0.10 (0.00, 1.05)	0.50 (0.21, 1.06)	0.51 (0.20, 1.08)	INF5mg_kgIV	**0.09 (0.00, 0.51)**	**0.07 (0.00, 0.43)**	**0.11 (0.00, 0.64)**	**0.10 (0.00, 0.61)**
NAT300mgIV	0.58 (0.29, 1.10)	**0.27 (0.07, 0.85)**	0.88 (0.63, 1.20)	0.74 (0.28, 1.74)	0.83 (0.33, 2.11)	1.05 (0.44, 2.63)	0.28 (0.01, 1.78)	0.82 (0.34, 1.94)	1.02 (0.46, 2.39)	0.61 (0.12, 2.82)	1.26 (0.60, 2.82)	1.05 (0.47, 2.52)	1.13 (0.34, 4.68)	5.32 (0.85, 121.69)	5.39 (0.91, 112.84)	**10.74 (1.96, 224.82)**	NAT300mgIV	0.76 (0.41, 1.45)	1.14 (0.65, 2.09)	1.06 (0.58, 1.95)
NAT3mg_kgIV	0.78 (0.31, 1.80)	0.36 (0.09, 1.22)	1.16 (0.58, 2.16)	0.98 (0.32, 2.68)	1.10 (0.38, 3.19)	1.41 (0.50, 3.92)	0.35 (0.01, 2.63)	1.10 (0.38, 3.03)	1.36 (0.49, 3.58)	0.80 (0.14, 4.24)	1.68 (0.65, 4.34)	1.40 (0.52, 3.80)	1.52 (0.40, 7.04)	**7.10 (1.04, 167.18)**	**7.20 (1.07, 158.88)**	**14.59 (2.34, 317.68)**	1.32 (0.69, 2.47)	NAT3mg_kgIV	1.50 (0.90, 2.62)	1.41 (0.81, 2.50)
NAT3mg_kgIVx2	0.51 (0.22, 1.12)	**0.24 (0.06, 0.79)**	0.76 (0.42, 1.36)	0.64 (0.22, 1.68)	0.72 (0.26, 2.09)	0.92 (0.34, 2.58)	0.23 (0.01, 1.69)	0.72 (0.25, 1.82)	0.89 (0.34, 2.39)	0.52 (0.09, 2.64)	1.10 (0.45, 2.86)	0.94 (0.36, 2.37)	0.99 (0.27, 4.58)	4.74 (0.69, 114.29)	4.73 (0.73, 106.26)	**9.50 (1.56, 211.86)**	0.88 (0.48, 1.53)	0.67 (0.38, 1.12)	NAT3mg_kgIVx2	0.93 (0.56, 1.49)
NAT6mg_kgIVx2	0.55 (0.23, 1.24)	**0.26 (0.06, 0.89)**	0.82 (0.44, 1.58)	0.69 (0.23, 1.88)	0.78 (0.28, 2.24)	1.00 (0.37, 2.80)	0.25 (0.01, 1.88)	0.78 (0.27, 2.08)	0.97 (0.36, 2.57)	0.56 (0.10, 3.05)	1.18 (0.47, 3.12)	1.00 (0.37, 2.63)	1.04 (0.29, 5.07)	5.18 (0.72, 113.58)	5.19 (0.79, 112.99)	**10.49 (1.65, 218.91)**	0.94 (0.51, 1.74)	0.71 (0.40, 1.24)	1.07 (0.67, 1.79)	NAT6mg_kgIVx2

(**TABLE S18G.** continued)

	CER200mgSC	CER20mg_kgIV	CER400mgSC	CER5mg_kgIV	ELD10mg_kgIV	ELD20mg_kgIV	FON0_1mg_kgIV	FON1mg_kg0_1mgIV_SC	FON1mg_kg1mgIV_SC	FON1mg_kgIV	FON4mg_kg0_1mgIV_SC	FON4mg_kg1mgIV_SC	FON4mg_kgIV	INF10mg_kgIV	INF20mg_kgIV	INF5mg_kgIV	NAT300mgIV	NAT3mg_kgIV	NAT3mg_kgIVx2	NAT6mg_kgIVx2
PBO	0.81 (0.42, 1.48)	0.38 (0.10, 1.15)	1.22 (0.97, 1.55)	1.02 (0.40, 2.36)	1.15 (0.47, 2.86)	1.46 (0.64, 3.62)	0.39 (0.01, 2.43)	1.14 (0.50, 2.61)	1.42 (0.66, 3.22)	0.84 (0.16, 3.87)	1.74 (0.86, 3.83)	1.47 (0.67, 3.36)	1.57 (0.48, 6.34)	**7.49 (1.22, 172.89)**	**7.47 (1.27, 150.86)**	**15.03 (2.70, 309.74)**	**1.39 (1.12, 1.74)**	1.05 (0.59, 2.00)	1.59 (0.95, 2.80)	1.48 (0.82, 2.63)
RIS1200mgIV	**0.46 (0.23, 0.87)**	**0.21 (0.06, 0.66)**	**0.69 (0.51, 0.96)**	0.57 (0.22, 1.36)	0.65 (0.27, 1.66)	0.83 (0.35, 2.10)	0.22 (0.01, 1.41)	0.65 (0.27, 1.53)	0.81 (0.36, 1.89)	0.48 (0.09, 2.19)	0.98 (0.48, 2.24)	0.83 (0.37, 1.99)	0.89 (0.27, 3.70)	4.21 (0.69, 95.80)	4.25 (0.73, 86.82)	**8.54 (1.52, 172.97)**	0.79 (0.58, 1.09)	0.59 (0.32, 1.16)	0.90 (0.51, 1.67)	0.84 (0.45, 1.58)
RIS600mgIV	**0.42 (0.22, 0.80)**	**0.20 (0.05, 0.62)**	**0.64 (0.47, 0.88)**	0.53 (0.21, 1.25)	0.60 (0.24, 1.54)	0.76 (0.32, 1.94)	0.20 (0.01, 1.28)	0.60 (0.25, 1.39)	0.75 (0.33, 1.74)	0.44 (0.09, 2.00)	0.90 (0.45, 2.06)	0.77 (0.34, 1.79)	0.82 (0.25, 3.38)	3.90 (0.63, 87.05)	3.92 (0.68, 78.76)	**7.89 (1.41, 161.93)**	**0.73 (0.54, 0.99)**	0.54 (0.29, 1.08)	0.83 (0.47, 1.53)	0.77 (0.42, 1.44)
RIS600mgIV4_4	0.58 (0.27, 1.18)	**0.28 (0.07, 0.86)**	0.88 (0.56, 1.34)	0.72 (0.27, 1.80)	0.82 (0.30, 2.20)	1.05 (0.42, 2.75)	0.27 (0.01, 1.80)	0.81 (0.32, 1.99)	1.02 (0.44, 2.41)	0.60 (0.11, 2.86)	1.26 (0.56, 2.87)	1.07 (0.44, 2.53)	1.13 (0.32, 4.75)	5.31 (0.85, 124.61)	5.36 (0.88, 114.54)	**10.85 (1.94, 224.05)**	1.00 (0.65, 1.50)	0.75 (0.37, 1.51)	1.15 (0.59, 2.17)	1.06 (0.52, 2.07)
UST1mg_kgIV	0.49 (0.19, 1.17)	**0.23 (0.05, 0.84)**	0.74 (0.37, 1.47)	0.61 (0.20, 1.69)	0.70 (0.24, 2.06)	0.89 (0.32, 2.55)	0.23 (0.01, 1.64)	0.69 (0.24, 1.93)	0.86 (0.31, 2.40)	0.51 (0.08, 2.62)	1.06 (0.41, 2.91)	0.90 (0.30, 2.71)	0.98 (0.24, 4.36)	4.48 (0.65, 104.94)	4.57 (0.68, 105.38)	**9.19 (1.55, 197.65)**	0.85 (0.42, 1.66)	0.64 (0.26, 1.54)	0.98 (0.41, 2.22)	0.90 (0.36, 2.12)
UST3mg_kgIV	0.47 (0.18, 1.11)	**0.22 (0.05, 0.75)**	0.72 (0.35, 1.38)	0.59 (0.19, 1.64)	0.66 (0.22, 2.03)	0.84 (0.29, 2.45)	0.22 (0.01, 1.56)	0.65 (0.23, 1.89)	0.83 (0.29, 2.29)	0.49 (0.08, 2.58)	1.02 (0.39, 2.70)	0.87 (0.30, 2.47)	0.93 (0.23, 4.21)	4.35 (0.62, 103.36)	4.41 (0.65, 104.67)	**8.85 (1.50, 193.98)**	0.81 (0.40, 1.54)	0.61 (0.25, 1.51)	0.93 (0.39, 2.15)	0.85 (0.35, 2.02)
UST4_5mg_kgIV	**0.48 (0.23, 0.95)**	**0.22 (0.06, 0.73)**	0.72 (0.47, 1.08)	0.60 (0.22, 1.47)	0.68 (0.26, 1.80)	0.86 (0.35, 2.25)	0.23 (0.01, 1.50)	0.67 (0.27, 1.65)	0.84 (0.36, 1.98)	0.49 (0.10, 2.34)	1.01 (0.47, 2.42)	0.86 (0.36, 2.05)	0.92 (0.27, 3.87)	4.42 (0.69, 96.92)	4.42 (0.76, 89.35)	**8.79 (1.60, 178.66)**	0.81 (0.54, 1.23)	0.61 (0.31, 1.27)	0.93 (0.50, 1.82)	0.87 (0.45, 1.72)
UST6mg_kgIV	0.47 (0.18, 1.12)	**0.23 (0.05, 0.75)**	0.72 (0.35, 1.38)	0.60 (0.19, 1.60)	0.67 (0.23, 1.97)	0.86 (0.29, 2.44)	0.22 (0.01, 1.55)	0.66 (0.23, 1.79)	0.83 (0.29, 2.29)	0.48 (0.08, 2.54)	1.03 (0.39, 2.69)	0.87 (0.30, 2.49)	0.94 (0.22, 4.21)	4.33 (0.60, 99.03)	4.37 (0.61, 96.56)	**8.86 (1.44, 191.29)**	0.82 (0.39, 1.53)	0.62 (0.24, 1.51)	0.94 (0.39, 2.13)	0.87 (0.34, 1.97)
UST90mgSC	0.81 (0.22, 2.64)	0.39 (0.07, 1.69)	1.22 (0.40, 3.53)	1.01 (0.25, 3.65)	1.18 (0.27, 4.72)	1.46 (0.38, 5.67)	0.36 (0.01, 3.19)	1.13 (0.28, 4.37)	1.42 (0.37, 5.36)	0.83 (0.12, 5.04)	1.76 (0.48, 6.41)	1.48 (0.37, 5.69)	1.56 (0.32, 8.93)	7.70 (0.77, 213.56)	8.03 (0.77, 203.05)	**15.50 (1.93, 413.59)**	1.39 (0.45, 4.08)	1.04 (0.30, 3.40)	1.60 (0.48, 5.17)	1.46 (0.44, 4.89)
VED0_5mg_kgIV	0.60 (0.23, 1.46)	0.29 (0.06, 1.03)	0.91 (0.43, 1.77)	0.76 (0.24, 2.15)	0.86 (0.28, 2.59)	1.08 (0.38, 3.27)	0.28 (0.01, 1.80)	0.84 (0.28, 2.49)	1.04 (0.37, 2.91)	0.60 (0.11, 3.20)	1.30 (0.47, 3.54)	1.10 (0.39, 3.18)	1.19 (0.30, 5.26)	5.54 (0.80, 122.07)	5.67 (0.83, 119.91)	**10.98 (1.86, 229.64)**	1.03 (0.50, 2.05)	0.77 (0.31, 1.95)	1.19 (0.49, 2.83)	1.09 (0.44, 2.66)
VED2mg_kgIV	0.46 (0.18, 1.07)	**0.22 (0.05, 0.74)**	0.69 (0.35, 1.33)	0.58 (0.18, 1.54)	0.66 (0.21, 1.94)	0.82 (0.30, 2.43)	0.21 (0.01, 1.47)	0.65 (0.22, 1.86)	0.81 (0.30, 2.28)	0.46 (0.09, 2.49)	1.01 (0.37, 2.69)	0.85 (0.30, 2.31)	0.89 (0.24, 3.95)	4.16 (0.59, 98.83)	4.28 (0.66, 94.55)	**8.56 (1.37, 200.66)**	0.79 (0.40, 1.51)	0.60 (0.25, 1.43)	0.91 (0.40, 2.07)	0.84 (0.35, 2.00)
VED300mgIV	0.46 (0.15, 1.28)	**0.21 (0.05, 0.85)**	0.70 (0.27, 1.62)	0.57 (0.16, 1.82)	0.65 (0.18, 2.28)	0.84 (0.25, 2.85)	0.21 (0.01, 1.75)	0.65 (0.19, 2.12)	0.80 (0.24, 2.55)	0.45 (0.07, 2.81)	1.00 (0.31, 3.09)	0.82 (0.26, 2.76)	0.88 (0.20, 4.72)	4.29 (0.57, 100.52)	4.40 (0.59, 90.35)	**8.86 (1.37, 178.57)**	0.80 (0.31, 1.87)	0.60 (0.19, 1.70)	0.91 (0.32, 2.44)	0.83 (0.28, 2.24)

(**TABLE S18G.** continued)

	PBO	RIS1200mgIV	RIS600mgIV	RIS600mgIV4_4	UST1mg_kgIV	UST3mg_kgIV	UST4_5mg_kgIV	UST6mg_kgIV	UST90mgSC	VED0_5mg_kgIV	VED2mg_kgIV	VED300mgIV
ABA10mg_kgIV	1.76 (0.58, 5.39)	3.11 (1.00, 9.90)	**3.38 (1.08, 10.69)**	2.46 (0.77, 7.98)	2.90 (0.79, 10.62)	3.07 (0.85, 10.67)	3.00 (0.95, 9.57)	3.05 (0.83, 10.63)	1.80 (0.39, 8.05)	2.34 (0.67, 9.02)	3.08 (0.90, 11.50)	3.13 (0.75, 12.33)
ABA30mg_kgIV	0.84 (0.29, 2.50)	1.49 (0.50, 4.43)	1.60 (0.54, 4.79)	1.18 (0.39, 3.83)	1.40 (0.41, 5.09)	1.46 (0.42, 5.23)	1.43 (0.47, 4.47)	1.45 (0.41, 5.43)	0.86 (0.19, 3.87)	1.15 (0.31, 4.35)	1.51 (0.42, 5.40)	1.51 (0.37, 6.09)
ABA3mg_kgIV	1.42 (0.49, 4.17)	2.52 (0.85, 7.46)	2.72 (0.92, 8.19)	1.99 (0.64, 6.27)	2.36 (0.66, 8.76)	2.46 (0.69, 9.07)	2.44 (0.79, 7.25)	2.45 (0.69, 8.93)	1.43 (0.34, 6.69)	1.94 (0.54, 6.99)	2.54 (0.75, 8.67)	2.54 (0.65, 10.45)
ADA160mg80mgSC	**0.41 (0.22, 0.72)**	0.73 (0.37, 1.34)	0.79 (0.40, 1.45)	0.58 (0.28, 1.14)	0.66 (0.28, 1.66)	0.70 (0.29, 1.73)	0.70 (0.35, 1.36)	0.70 (0.30, 1.68)	0.41 (0.13, 1.39)	0.55 (0.23, 1.38)	0.72 (0.29, 1.72)	0.71 (0.25, 2.16)
ADA40mg20mgSC	0.74 (0.35, 1.59)	1.30 (0.60, 2.86)	1.41 (0.66, 3.06)	1.04 (0.45, 2.43)	1.21 (0.45, 3.29)	1.26 (0.48, 3.43)	1.26 (0.57, 2.85)	1.25 (0.50, 3.41)	0.74 (0.21, 2.81)	1.00 (0.37, 2.77)	1.31 (0.51, 3.44)	1.29 (0.43, 4.34)
ADA80mg40mgSC	**0.45 (0.23, 0.88)**	0.80 (0.39, 1.61)	0.87 (0.43, 1.73)	0.63 (0.30, 1.36)	0.74 (0.30, 1.91)	0.77 (0.32, 1.93)	0.77 (0.37, 1.61)	0.77 (0.33, 1.97)	0.45 (0.14, 1.60)	0.61 (0.24, 1.56)	0.80 (0.32, 1.97)	0.79 (0.28, 2.60)
AMI0_4mgPO	1.43 (0.78, 2.87)	**2.50 (1.31, 5.14)**	**2.71 (1.43, 5.60)**	1.99 (0.99, 4.50)	2.34 (0.99, 6.39)	**2.44 (1.03, 6.61)**	**2.42 (1.21, 5.22)**	**2.42 (1.04, 6.45)**	1.44 (0.42, 5.21)	1.94 (0.70, 5.19)	2.56 (0.92, 6.51)	2.48 (0.91, 7.98)
AND150mgSC1_1	1.25 (0.46, 3.27)	2.22 (0.79, 6.02)	2.41 (0.85, 6.49)	1.74 (0.61, 4.88)	2.02 (0.62, 6.65)	2.11 (0.68, 6.95)	2.16 (0.75, 5.95)	2.09 (0.65, 7.06)	1.25 (0.30, 5.31)	1.67 (0.52, 5.32)	2.19 (0.63, 7.06)	2.17 (0.59, 8.47)
AND150mgSC2_2	0.97 (0.37, 2.37)	1.72 (0.64, 4.24)	1.87 (0.69, 4.58)	1.36 (0.48, 3.51)	1.62 (0.51, 4.83)	1.68 (0.55, 5.12)	1.65 (0.61, 4.27)	1.66 (0.53, 5.51)	0.98 (0.26, 4.05)	1.33 (0.42, 4.00)	1.73 (0.52, 5.09)	1.74 (0.48, 5.94)
AND300mgSC	1.84 (0.62, 5.45)	**3.23 (1.08, 9.59)**	**3.51 (1.14, 10.31)**	2.55 (0.85, 8.09)	3.04 (0.87, 10.73)	3.14 (0.96, 11.48)	3.12 (1.00, 9.76)	3.12 (0.91, 11.93)	1.87 (0.45, 8.61)	2.50 (0.72, 8.79)	3.26 (0.87, 11.29)	3.30 (0.82, 12.49)
API100mgPO	1.39 (0.68, 2.90)	**2.45 (1.15, 5.28)**	**2.66 (1.25, 5.68)**	1.94 (0.87, 4.46)	2.26 (0.88, 5.86)	2.36 (0.92, 6.18)	**2.36 (1.09, 5.44)**	2.33 (0.92, 6.35)	1.38 (0.39, 5.28)	1.86 (0.70, 5.19)	2.45 (0.96, 6.75)	2.44 (0.76, 8.24)
API50mgPO	1.87 (0.86, 4.66)	**3.29 (1.47, 8.44)**	**3.58 (1.59, 9.02)**	**2.64 (1.12, 6.98)**	**3.07 (1.16, 9.41)**	**3.27 (1.16, 9.60)**	**3.19 (1.37, 8.33)**	**3.24 (1.21, 9.64)**	1.93 (0.51, 7.69)	2.58 (0.92, 7.79)	**3.33 (1.24, 9.93)**	**3.34 (1.01, 11.72)**
BRI400mgIV	**0.36 (0.12, 0.90)**	0.63 (0.21, 1.61)	0.69 (0.22, 1.74)	0.50 (0.16, 1.34)	0.58 (0.17, 1.90)	0.60 (0.18, 2.06)	0.61 (0.20, 1.62)	0.60 (0.18, 2.01)	0.35 (0.08, 1.62)	0.48 (0.14, 1.58)	0.62 (0.18, 2.02)	0.64 (0.15, 2.28)
BRI700mgIV	0.45 (0.16, 1.02)	0.78 (0.28, 1.84)	0.85 (0.30, 2.00)	0.62 (0.22, 1.53)	0.72 (0.22, 2.30)	0.75 (0.24, 2.38)	0.75 (0.26, 1.84)	0.75 (0.24, 2.38)	0.44 (0.10, 1.88)	0.60 (0.19, 1.83)	0.77 (0.24, 2.30)	0.79 (0.20, 2.70)
BRO210mgIV	0.94 (0.03, 16.47)	1.68 (0.06, 28.88)	1.83 (0.07, 31.78)	1.36 (0.05, 22.31)	1.71 (0.06, 30.47)	1.77 (0.06, 31.19)	1.64 (0.06, 29.06)	1.76 (0.06, 32.86)	1.03 (0.03, 22.98)	1.33 (0.05, 26.86)	1.73 (0.07, 33.06)	1.71 (0.06, 34.43)
BRO350mgIV	0.18 (0.01, 1.03)	0.31 (0.01, 1.79)	0.34 (0.01, 1.97)	0.24 (0.01, 1.53)	0.28 (0.01, 1.96)	0.29 (0.01, 2.12)	0.30 (0.01, 1.78)	0.29 (0.01, 2.05)	0.17 (0.01, 1.54)	0.22 (0.01, 1.63)	0.30 (0.01, 2.06)	0.32 (0.01, 2.30)
BRO700mgIV	0.29 (0.01, 2.02)	0.50 (0.02, 3.57)	0.54 (0.02, 3.86)	0.40 (0.02, 2.93)	0.47 (0.02, 3.83)	0.49 (0.02, 4.11)	0.49 (0.02, 3.52)	0.50 (0.02, 4.11)	0.27 (0.01, 2.81)	0.39 (0.02, 2.89)	0.50 (0.02, 3.88)	0.52 (0.02, 4.08)
CDP10mg_kgIV	0.99 (0.66, 1.48)	**1.75 (1.09, 2.70)**	**1.90 (1.19, 2.94)**	1.38 (0.81, 2.37)	1.62 (0.77, 3.56)	1.69 (0.80, 3.76)	1.70 (0.98, 2.84)	1.68 (0.81, 3.78)	0.99 (0.33, 3.18)	1.33 (0.64, 2.99)	1.74 (0.82, 3.75)	1.74 (0.70, 4.83)
CER100mgSC	0.88 (0.54, 1.49)	1.56 (0.90, 2.74)	1.69 (0.98, 2.95)	1.23 (0.67, 2.36)	1.47 (0.64, 3.36)	1.53 (0.69, 3.49)	1.50 (0.82, 2.82)	1.52 (0.69, 3.50)	0.90 (0.28, 2.92)	1.19 (0.53, 2.74)	1.57 (0.72, 3.53)	1.56 (0.58, 4.38)
CER10mg_kgIV	1.50 (0.54, 4.92)	2.66 (0.91, 8.75)	2.88 (0.98, 9.45)	2.10 (0.71, 6.89)	2.52 (0.74, 9.29)	2.64 (0.79, 10.02)	2.56 (0.86, 8.62)	2.60 (0.77, 9.94)	1.50 (0.36, 7.71)	2.04 (0.61, 8.07)	2.64 (0.82, 9.96)	2.63 (0.72, 11.19)
CER200mgSC	1.23 (0.68, 2.38)	**2.18 (1.14, 4.28)**	**2.37 (1.25, 4.63)**	1.74 (0.85, 3.69)	2.04 (0.85, 5.14)	2.13 (0.90, 5.50)	**2.09 (1.05, 4.34)**	2.11 (0.89, 5.42)	1.23 (0.38, 4.57)	1.66 (0.68, 4.30)	2.20 (0.93, 5.45)	2.18 (0.78, 6.67)
CER20mg_kgIV	2.63 (0.87, 9.80)	**4.68 (1.50, 17.33)**	**5.06 (1.62, 18.79)**	**3.63 (1.17, 14.52)**	**4.33 (1.19, 18.93)**	**4.48 (1.34, 19.87)**	**4.49 (1.36, 17.14)**	**4.42 (1.33, 20.11)**	2.58 (0.59, 15.22)	3.50 (0.97, 16.40)	**4.64 (1.34, 20.54)**	**4.77 (1.18, 22.06)**
CER400mgSC	0.82 (0.65, 1.03)	**1.45 (1.04, 1.98)**	**1.57 (1.14, 2.13)**	1.14 (0.75, 1.79)	1.34 (0.68, 2.72)	1.39 (0.72, 2.87)	1.39 (0.92, 2.12)	1.39 (0.73, 2.87)	0.82 (0.28, 2.48)	1.10 (0.56, 2.32)	1.44 (0.75, 2.88)	1.43 (0.62, 3.74)
CER5mg_kgIV	0.98 (0.42, 2.49)	1.74 (0.73, 4.53)	1.88 (0.80, 4.86)	1.38 (0.56, 3.76)	1.63 (0.59, 5.04)	1.68 (0.61, 5.37)	1.67 (0.68, 4.58)	1.68 (0.63, 5.20)	0.99 (0.27, 4.08)	1.32 (0.46, 4.25)	1.72 (0.65, 5.50)	1.77 (0.55, 6.17)
ELD10mg_kgIV	0.87 (0.35, 2.12)	1.53 (0.60, 3.77)	1.66 (0.65, 4.09)	1.22 (0.45, 3.35)	1.44 (0.48, 4.16)	1.51 (0.49, 4.49)	1.47 (0.55, 3.88)	1.48 (0.51, 4.40)	0.85 (0.21, 3.75)	1.17 (0.39, 3.59)	1.51 (0.51, 4.66)	1.53 (0.44, 5.62)
ELD20mg_kgIV	0.69 (0.28, 1.57)	1.21 (0.48, 2.88)	1.31 (0.52, 3.12)	0.96 (0.36, 2.37)	1.12 (0.39, 3.17)	1.19 (0.41, 3.39)	1.16 (0.44, 2.86)	1.16 (0.41, 3.41)	0.69 (0.18, 2.62)	0.93 (0.31, 2.64)	1.22 (0.41, 3.31)	1.19 (0.35, 4.07)
FON0_1mg_kgIV	2.59 (0.41, 70.02)	4.54 (0.71, 127.69)	4.92 (0.78, 140.20)	3.66 (0.56, 97.30)	4.39 (0.61, 123.67)	4.55 (0.64, 126.53)	4.41 (0.67, 127.49)	4.53 (0.65, 132.65)	2.75 (0.31, 78.72)	3.62 (0.56, 99.96)	4.67 (0.68, 134.72)	4.74 (0.57, 134.88)
FON1mg_kg0_1mgIV_SC	0.87 (0.38, 2.00)	1.54 (0.65, 3.69)	1.67 (0.72, 4.02)	1.23 (0.50, 3.11)	1.46 (0.52, 4.09)	1.53 (0.53, 4.27)	1.49 (0.61, 3.77)	1.52 (0.56, 4.32)	0.88 (0.23, 3.51)	1.19 (0.40, 3.54)	1.54 (0.54, 4.50)	1.54 (0.47, 5.35)
FON1mg_kg1mgIV_SC	0.70 (0.31, 1.52)	1.23 (0.53, 2.75)	1.34 (0.58, 3.01)	0.98 (0.42, 2.29)	1.17 (0.42, 3.26)	1.20 (0.44, 3.41)	1.19 (0.50, 2.80)	1.21 (0.44, 3.45)	0.71 (0.19, 2.72)	0.96 (0.34, 2.67)	1.24 (0.44, 3.33)	1.25 (0.39, 4.14)

(**TABLE S18G.** continued)

	PBO	RIS1200mgIV	RIS600mgIV	RIS600mgIV4_4	UST1mg_kgIV	UST3mg_kgIV	UST4_5mg_kgIV	UST6mg_kgIV	UST90mgSC	VED0_5mg_kgIV	VED2mg_kgIV	VED300mgIV
FON1mg_kgIV	1.19 (0.26, 6.11)	2.08 (0.46, 10.61)	2.27 (0.50, 11.47)	1.66 (0.35, 8.94)	1.96 (0.38, 11.99)	2.05 (0.39, 12.08)	2.04 (0.43, 10.35)	2.07 (0.39, 12.60)	1.21 (0.20, 8.34)	1.66 (0.31, 9.23)	2.17 (0.40, 11.74)	2.20 (0.36, 13.74)
FON4mg_kg0_1mgIV_SC	0.58 (0.26, 1.16)	1.02 (0.45, 2.08)	1.12 (0.48, 2.24)	0.79 (0.35, 1.77)	0.94 (0.34, 2.43)	0.98 (0.37, 2.58)	0.99 (0.41, 2.11)	0.97 (0.37, 2.58)	0.57 (0.16, 2.10)	0.77 (0.28, 2.11)	0.99 (0.37, 2.69)	1.00 (0.32, 3.23)
FON4mg_kg1mgIV_SC	0.68 (0.30, 1.48)	1.20 (0.50, 2.73)	1.30 (0.56, 2.95)	0.94 (0.40, 2.25)	1.11 (0.37, 3.28)	1.15 (0.40, 3.34)	1.16 (0.49, 2.74)	1.15 (0.40, 3.39)	0.67 (0.18, 2.69)	0.91 (0.31, 2.58)	1.18 (0.43, 3.31)	1.21 (0.36, 3.89)
FON4mg_kgIV	0.64 (0.16, 2.10)	1.12 (0.27, 3.73)	1.22 (0.30, 3.99)	0.89 (0.21, 3.08)	1.02 (0.23, 4.10)	1.08 (0.24, 4.33)	1.08 (0.26, 3.75)	1.06 (0.24, 4.50)	0.64 (0.11, 3.09)	0.84 (0.19, 3.34)	1.12 (0.25, 4.18)	1.13 (0.21, 4.95)
INF10mg_kgIV	**0.13 (0.01, 0.82)**	0.24 (0.01, 1.44)	0.26 (0.01, 1.58)	0.19 (0.01, 1.18)	0.22 (0.01, 1.53)	0.23 (0.01, 1.62)	0.23 (0.01, 1.44)	0.23 (0.01, 1.66)	0.13 (0.00, 1.30)	0.18 (0.01, 1.26)	0.24 (0.01, 1.68)	0.23 (0.01, 1.75)
INF20mg_kgIV	**0.13 (0.01, 0.79)**	0.24 (0.01, 1.37)	0.26 (0.01, 1.47)	0.19 (0.01, 1.14)	0.22 (0.01, 1.48)	0.23 (0.01, 1.55)	0.23 (0.01, 1.32)	0.23 (0.01, 1.64)	0.12 (0.00, 1.30)	0.18 (0.01, 1.20)	0.23 (0.01, 1.51)	0.23 (0.01, 1.69)
INF5mg_kgIV	**0.07 (0.00, 0.37)**	**0.12 (0.01, 0.66)**	**0.13 (0.01, 0.71)**	**0.09 (0.00, 0.51)**	**0.11 (0.01, 0.65)**	**0.11 (0.01, 0.67)**	**0.11 (0.01, 0.63)**	**0.11 (0.01, 0.70)**	**0.06 (0.00, 0.52)**	**0.09 (0.00, 0.54)**	**0.12 (0.00, 0.73)**	**0.11 (0.01, 0.73)**
NAT300mgIV	**0.72 (0.57, 0.89)**	1.27 (0.92, 1.72)	**1.38 (1.01, 1.85)**	1.00 (0.67, 1.54)	1.18 (0.60, 2.37)	1.23 (0.65, 2.48)	1.23 (0.81, 1.84)	1.22 (0.65, 2.55)	0.72 (0.24, 2.23)	0.97 (0.49, 2.02)	1.27 (0.66, 2.50)	1.26 (0.53, 3.20)
NAT3mg_kgIV	0.95 (0.50, 1.70)	1.69 (0.86, 3.13)	1.84 (0.92, 3.44)	1.33 (0.66, 2.73)	1.56 (0.65, 3.89)	1.65 (0.66, 4.00)	1.65 (0.79, 3.18)	1.62 (0.66, 4.09)	0.96 (0.29, 3.32)	1.29 (0.51, 3.24)	1.67 (0.70, 4.03)	1.68 (0.59, 5.19)
NAT3mg_kgIVx2	0.63 (0.36, 1.05)	1.11 (0.60, 1.94)	1.20 (0.65, 2.12)	0.87 (0.46, 1.70)	1.02 (0.45, 2.44)	1.08 (0.47, 2.56)	1.08 (0.55, 1.98)	1.07 (0.47, 2.54)	0.62 (0.19, 2.09)	0.84 (0.35, 2.04)	1.10 (0.48, 2.52)	1.10 (0.41, 3.14)
NAT6mg_kgIVx2	0.68 (0.38, 1.22)	1.19 (0.63, 2.22)	1.29 (0.69, 2.41)	0.95 (0.48, 1.91)	1.11 (0.47, 2.76)	1.17 (0.50, 2.86)	1.15 (0.58, 2.24)	1.15 (0.51, 2.93)	0.68 (0.20, 2.30)	0.91 (0.38, 2.26)	1.19 (0.50, 2.88)	1.20 (0.45, 3.57)
PBO	PBO	**1.77 (1.41, 2.18)**	**1.91 (1.55, 2.35)**	1.39 (0.99, 2.06)	1.64 (0.87, 3.23)	1.71 (0.93, 3.36)	**1.71 (1.21, 2.40)**	1.70 (0.95, 3.44)	1.00 (0.36, 2.99)	1.35 (0.69, 2.73)	1.76 (0.96, 3.37)	1.75 (0.78, 4.40)
RIS1200mgIV	**0.57 (0.46, 0.71)**	RIS1200mgIV	1.08 (0.92, 1.28)	0.79 (0.53, 1.23)	0.94 (0.48, 1.89)	0.98 (0.51, 1.94)	0.97 (0.70, 1.35)	0.97 (0.52, 2.01)	0.57 (0.20, 1.75)	0.77 (0.38, 1.60)	1.00 (0.53, 1.97)	1.00 (0.42, 2.60)
RIS600mgIV	**0.52 (0.43, 0.65)**	0.92 (0.78, 1.08)	RIS600mgIV	0.73 (0.48, 1.13)	0.86 (0.44, 1.75)	0.90 (0.47, 1.80)	0.89 (0.67, 1.19)	0.89 (0.48, 1.84)	0.53 (0.18, 1.58)	0.71 (0.35, 1.45)	0.92 (0.49, 1.80)	0.92 (0.39, 2.37)
RIS600mgIV4_4	0.72 (0.49, 1.01)	1.26 (0.82, 1.90)	1.37 (0.89, 2.08)	RIS600mgIV4_4	1.17 (0.56, 2.53)	1.21 (0.59, 2.66)	1.22 (0.72, 2.00)	1.20 (0.60, 2.65)	0.71 (0.24, 2.23)	0.96 (0.47, 2.11)	1.26 (0.62, 2.67)	1.25 (0.50, 3.39)
UST1mg_kgIV	0.61 (0.31, 1.15)	1.06 (0.53, 2.08)	1.16 (0.57, 2.27)	0.85 (0.40, 1.78)	UST1mg_kgIV	1.05 (0.60, 1.83)	1.03 (0.50, 2.19)	1.04 (0.61, 1.82)	0.61 (0.17, 2.24)	0.82 (0.31, 2.05)	1.07 (0.43, 2.63)	1.07 (0.36, 3.33)
UST3mg_kgIV	0.59 (0.30, 1.08)	1.03 (0.51, 1.96)	1.11 (0.56, 2.14)	0.83 (0.38, 1.69)	0.95 (0.55, 1.66)	UST3mg_kgIV	0.99 (0.47, 2.03)	0.99 (0.59, 1.76)	0.59 (0.17, 2.05)	0.77 (0.31, 2.03)	1.02 (0.42, 2.55)	1.01 (0.35, 3.13)
UST4_5mg_kgIV	**0.59 (0.42, 0.83)**	1.04 (0.74, 1.43)	1.12 (0.84, 1.49)	0.82 (0.50, 1.39)	0.97 (0.46, 2.01)	1.01 (0.49, 2.11)	UST4_5mg_kgIV	1.00 (0.50, 2.12)	0.59 (0.20, 1.85)	0.80 (0.37, 1.69)	1.04 (0.52, 2.14)	1.03 (0.42, 2.77)
UST6mg_kgIV	0.59 (0.29, 1.06)	1.04 (0.50, 1.93)	1.12 (0.54, 2.08)	0.83 (0.38, 1.66)	0.96 (0.55, 1.64)	1.01 (0.57, 1.70)	1.00 (0.47, 1.99)	UST6mg_kgIV	0.59 (0.17, 2.10)	0.79 (0.31, 1.96)	1.02 (0.42, 2.47)	1.01 (0.36, 3.06)
UST90mgSC	1.00 (0.33, 2.81)	1.74 (0.57, 5.13)	1.89 (0.63, 5.54)	1.41 (0.45, 4.16)	1.64 (0.45, 5.74)	1.71 (0.49, 5.86)	1.70 (0.54, 5.12)	1.70 (0.48, 5.86)	UST90mgSC	1.34 (0.37, 4.71)	1.72 (0.49, 6.26)	1.73 (0.44, 6.91)
VED0_5mg_kgIV	0.74 (0.37, 1.45)	1.30 (0.62, 2.66)	1.41 (0.69, 2.88)	1.04 (0.47, 2.14)	1.22 (0.49, 3.18)	1.30 (0.49, 3.22)	1.25 (0.59, 2.74)	1.26 (0.51, 3.26)	0.75 (0.21, 2.69)	VED0_5mg_kgIV	1.30 (0.74, 2.34)	1.28 (0.45, 4.05)
VED2mg_kgIV	0.57 (0.30, 1.04)	1.00 (0.51, 1.89)	1.08 (0.56, 2.06)	0.79 (0.37, 1.62)	0.94 (0.38, 2.31)	0.98 (0.39, 2.36)	0.96 (0.47, 1.91)	0.98 (0.40, 2.37)	0.58 (0.16, 2.06)	0.77 (0.43, 1.35)	VED2mg_kgIV	0.98 (0.35, 3.07)
VED300mgIV	0.57 (0.23, 1.29)	1.00 (0.39, 2.36)	1.09 (0.42, 2.55)	0.80 (0.30, 2.00)	0.94 (0.30, 2.80)	0.99 (0.32, 2.84)	0.97 (0.36, 2.38)	0.99 (0.33, 2.78)	0.58 (0.14, 2.25)	0.78 (0.25, 2.21)	1.02 (0.33, 2.82)	VED300mgIV

Table S18h: League table for clinical remission for the some concerns studies. The table presented the multiple treatment comparisons based on consistency analysis of the networks. Treatments are depicted alphabetically. Values are presented as relative risk (RR) with 95% credible interval (CrI). For all comparisons an RR <1 favors the occurrence of the event for the row-defining treatment, while an RR>1 favors the column-defining treatment. Statistically significant results are represented in bold. Multiple treatment comparisons based on consistency analysis of the networks. Treatments are depicted alphabetically. Values are presented as relative risk (RR) with 95% credible interval (CrI). For all comparisons an RR <1 favors the occurrence of the event for the row-defining treatment, while an RR>1 favors the column-defining treatment. Statistically significant results are represented in bold.

	ETA25mgSC	ONE10mgSC	ONE25mgSC	ONE35mgSC	ONE50mgSC	PBO
ETA25mgSC	ETA25mgSC	2.53 (0.37, 20.85)	1.19 (0.17, 10.40)	2.57 (0.39, 20.33)	1.76 (0.27, 14.01)	1.97 (0.43, 13.00)
ONE10mgSC	0.40 (0.05, 2.70)	ONE10mgSC	0.47 (0.13, 1.44)	1.01 (0.35, 2.84)	0.69 (0.22, 2.18)	0.80 (0.24, 2.53)
ONE25mgSC	0.84 (0.10, 5.93)	2.12 (0.69, 7.65)	ONE25mgSC	2.13 (0.68, 7.81)	1.48 (0.44, 5.45)	1.66 (0.49, 6.01)
ONE35mgSC	0.39 (0.05, 2.55)	0.99 (0.35, 2.87)	0.47 (0.13, 1.46)	ONE35mgSC	0.69 (0.22, 2.16)	0.78 (0.25, 2.45)
ONE50mgSC	0.57 (0.07, 3.65)	1.45 (0.46, 4.55)	0.68 (0.18, 2.27)	1.46 (0.46, 4.59)	ONE50mgSC	1.14 (0.35, 3.61)
PBO	0.51 (0.08, 2.33)	1.26 (0.40, 4.11)	0.60 (0.17, 2.06)	1.29 (0.41, 3.98)	0.88 (0.28, 2.85)	PBO

Table S18j: League table for increse in the IBDQ score for the low risk of bias studies. The table presented the Multiple treatment comparisons based on consistency analysis of the networks. Treatments are depicted alphabetically. Values are presented as mean difference (MD) with 95% credible interval (CrI). For all comparisons an MD <0 favors the occurrence of the event for the row-defining treatment, while an MD>0 favors the column-defining treatment. Statistically significant results are represented in bold. Each tab of the spreadsheet presents data from a network constructed for the IBDQ outcome.

	FIL200mgOR	PBO	RIS200mgIV	RIS600mgIV	UPA12mgOR_BID	UPA24mgOR	UPA24mgOR_BID	UPA3mgOR_BID	UPA45mgOR	UPA6mgOR_BID	UST130mgIV	UST6mg_kgIV
FIL200mgOR	FIL200mgOR	-16.05 (-35.24, 2.66)	-1.76 (-30.09, 25.84)	11.36 (-17.45, 40.36)	1.36 (-26.53, 30.17)	-8.14 (-35.62, 19.82)	13.61 (-14.92, 43.00)	-5.92 (-35.01, 22.42)	6.91 (-14.90, 29.52)	11.35 (-18.30, 41.17)	-6.21 (-27.85, 16.08)	-5.70 (-27.31, 16.34)
PBO	16.05 (-2.66, 35.24)	PBO	14.35 (-6.21, 34.84)	**27.44 (6.76, 48.19)**	17.38 (-4.12, 39.02)	8.00 (-11.47, 28.44)	**29.75 (8.12, 51.74)**	10.25 (-11.97, 31.74)	23.14 (11.49, 34.49)	**27.49 (4.40, 50.81)**	9.87 (-1.31, 21.56)	10.48 (-1.23, 22.23)
RIS200mgIV	1.76 (-25.84, 30.09)	-14.35 (-34.84, 6.21)	RIS200mgIV	13.16 (-6.40, 32.66)	3.13 (-26.44, 32.41)	-6.35 (-33.84, 21.60)	15.16 (-14.00, 45.85)	-4.54 (-33.12, 25.34)	8.68 (-14.50, 32.56)	12.88 (-17.18, 43.89)	-4.40 (-27.28, 19.38)	-4.00 (-27.20, 19.64)
RIS600mgIV	-11.36 (-40.36, 17.45)	**-27.44 (-48.19, -6.76)**	-13.16 (-32.66, 6.40)	RIS600mgIV	-9.92 (-39.92, 20.21)	-19.44 (-47.40, 8.89)	2.54 (-27.51, 32.61)	-17.55 (-47.67, 12.39)	-4.34 (-27.73, 19.46)	0.18 (-30.86, 30.37)	-17.46 (-40.67, 6.10)	-16.90 (-40.56, 6.78)
UPA12mgOR_BID	-1.36 (-30.17, 26.53)	-17.38 (-39.02, 4.12)	-3.13 (-32.41, 26.44)	9.92 (-20.21, 39.92)	UPA12mgOR_BID	-9.38 (-31.23, 12.25)	12.35 (-10.92, 35.55)	-7.37 (-31.04, 15.97)	5.66 (-18.71, 29.60)	9.99 (-14.30, 34.51)	-7.40 (-32.41, 16.66)	-6.79 (-31.72, 16.30)
UPA24mgOR	8.14 (-19.82, 35.62)	-8.00 (-28.44, 11.47)	6.35 (-21.60, 33.84)	19.44 (-8.89, 47.40)	9.38 (-12.25, 31.23)	UPA24mgOR	21.72 (0.31, 43.45)	1.95 (-19.78, 24.10)	15.12 (-7.77, 37.56)	19.41 (-3.60, 42.13)	2.03 (-20.79, 24.97)	2.47 (-19.97, 25.59)
UPA24mgOR_BID	-13.61 (-43.00, 14.92)	**-29.75 (-51.74, -8.12)**	-15.16 (-45.85, 14.00)	-2.54 (-32.61, 27.51)	-12.35 (-35.55, 10.92)	**-21.72 (-43.45, -0.31)**	UPA24mgOR_BID	-19.81 (-43.56, 3.70)	-6.67 (-31.37, 17.17)	-2.42 (-27.43, 22.48)	-19.81 (-44.56, 4.72)	-19.25 (-43.96, 4.52)
UPA3mgOR_BID	5.92 (-22.42, 35.01)	-10.25 (-31.74, 11.97)	4.54 (-25.34, 33.12)	17.55 (-12.39, 47.67)	7.37 (-15.97, 31.04)	-1.95 (-24.10, 19.78)	19.81 (-3.70, 43.56)	UPA3mgOR_BID	12.92 (-11.67, 37.20)	17.33 (-7.86, 42.69)	-0.27 (-25.06, 24.74)	0.38 (-24.28, 24.53)
UPA45mgOR	-6.91 (-29.52, 14.90)	**-23.14 (-34.49, -11.49)**	-8.68 (-32.56, 14.50)	4.34 (-19.46, 27.73)	-5.66 (-29.60, 18.71)	-15.12 (-37.56, 7.77)	6.67 (-17.17, 31.37)	-12.92 (-37.20, 11.67)	UPA45mgOR	4.17 (-21.06, 30.24)	-13.33 (-28.80, 3.52)	-12.65 (-29.13, 3.95)
UPA6mgOR_BID	-11.35 (-41.17, 18.30)	**-27.49 (-50.81, -4.40)**	-12.88 (-43.89, 17.18)	-0.18 (-30.37, 30.86)	-9.99 (-34.51, 14.30)	-19.41 (-42.13, 3.60)	2.42 (-22.48, 27.43)	-17.33 (-42.69, 7.86)	-4.17 (-30.24, 21.06)	UPA6mgOR_BID	-17.55 (-43.77, 8.01)	-17.01 (-42.54, 8.35)
UST130mgIV	6.21 (-16.08, 27.85)	-9.87 (-21.56, 1.31)	4.40 (-19.38, 27.28)	17.46 (-6.10, 40.67)	7.40 (-16.66, 32.41)	-2.03 (-24.97, 20.79)	19.81 (-4.72, 44.56)	0.27 (-24.74, 25.06)	13.33 (-3.52, 28.80)	17.55 (-8.01, 43.77)	UST130mgIV	0.65 (-10.95, 11.34)
UST6mg_kgIV	5.70 (-16.34, 27.31)	-10.48 (-22.23, 1.23)	4.00 (-19.64, 27.20)	16.90 (-6.78, 40.56)	6.79 (-16.30, 31.72)	-2.47 (-25.59, 19.97)	19.25 (-4.52, 43.96)	-0.38 (-24.53, 24.28)	12.65 (-3.95, 29.13)	17.01 (-8.35, 42.54)	-0.65 (-11.34, 10.95)	UST136mg_kgIV

Table S18k: League table for increse in the IBDQ score for the low risk of bias studies. The table presented the Multiple treatment comparisons based on consistency analysis of the networks. Treatments are depicted alphabetically. Values are presented as mean difference (MD) with 95% credible interval (CrI). For all comparisons an MD <0 favors the occurrence of the event for the row-defining treatment, while an MD>0 favors the column-defining treatment. Statistically significant results are represented in bold. Each tab of the spreadsheet presents data from a network constructed for the IBDQ outcome.

	CER400mgSC	FON1mg_kg0_1mgIV_SC	FON1mg_kg1mgIV_SC	FON4mg_kg0_1mgIV_SC	FON4mg_kg1mgIV_SC	INF10mg_kgIV	INF20mg_kgIV	INF5mg_kgIV	NAT300mgIV	PBO
CER400mgSC	CER400mgSC	-12.12 (-85.37, 61.76)	-3.76 (-78.25, 70.91)	-9.25 (-81.36, 66.07)	-7.10 (-78.68, 68.16)	13.06 (-60.86, 87.16)	13.98 (-61.43, 88.77)	28.63 (-44.31, 104.95)	-0.36 (-73.15, 71.44)	-12.23 (-62.58, 39.32)
FON1mg_kg0_1mgIV_SC	12.12 (-61.76, 85.37)	FON1mg_kg0_1mgIV_SC	8.86 (-46.44, 61.45)	3.12 (-49.91, 55.71)	5.45 (-49.82, 60.29)	25.03 (-51.92, 100.48)	26.05 (-48.67, 100.94)	40.74 (-33.28, 118.46)	11.51 (-61.94, 84.91)	-0.02 (-54.20, 52.94)
FON1mg_kg1mgIV_SC	3.76 (-70.91, 78.25)	-8.86 (-61.45, 46.44)	FON1mg_kg1mgIV_SC	-5.52 (-58.35, 49.60)	-3.13 (-54.85, 49.24)	16.66 (-58.48, 93.73)	17.89 (-59.14, 93.40)	32.39 (-44.51, 107.65)	3.01 (-71.35, 79.20)	-8.39 (-61.87, 43.93)
FON4mg_kg0_1mgIV_SC	9.25 (-66.07, 81.36)	-3.12 (-55.71, 49.91)	5.52 (-49.60, 58.35)	FON4mg_kg0_1mgIV_SC	2.23 (-50.75, 54.69)	22.03 (-55.11, 100.16)	23.00 (-54.14, 99.19)	37.62 (-38.59, 113.46)	8.28 (-66.32, 84.15)	-3.00 (-57.46, 50.56)
FON4mg_kg1mgIV_SC	7.10 (-68.16, 78.68)	-5.45 (-60.29, 49.82)	3.13 (-49.24, 54.85)	-2.23 (-54.69, 50.75)	FON4mg_kg1mgIV_SC	19.67 (-55.68, 95.28)	20.89 (-54.66, 94.32)	35.51 (-39.07, 112.10)	6.38 (-66.07, 80.57)	-5.25 (-59.34, 47.48)
INF10mg_kgIV	-13.06 (-87.16, 60.86)	-25.03 (-100.48, 51.92)	-16.66 (-93.73, 58.48)	-22.03 (-100.16, 55.11)	-19.67 (-95.28, 55.68)	INF10mg_kgIV	0.81 (-53.45, 54.00)	15.71 (-36.00, 70.32)	-13.49 (-87.76, 61.16)	-25.13 (-77.99, 29.14)
INF20mg_kgIV	-13.98 (-88.77, 61.43)	-26.05 (-100.94, 48.67)	-17.89 (-93.40, 59.14)	-23.00 (-99.19, 54.14)	-20.89 (-94.32, 54.66)	-0.81 (-54.00, 53.45)	INF20mg_kgIV	15.04 (-37.68, 68.30)	-14.47 (-88.87, 59.29)	-26.02 (-79.60, 27.69)
INF5mg_kgIV	-28.63 (-104.95, 44.31)	-40.74 (-118.46, 33.28)	-32.39 (-107.65, 44.51)	-37.62 (-113.46, 38.59)	-35.51 (-112.10, 39.07)	-15.71 (-70.32, 36.00)	-15.04 (-68.30, 37.68)	INF5mg_kgIV	-29.23 (-106.72, 42.98)	-40.87 (-95.89, 12.61)
NAT300mgIV	0.36 (-71.44, 73.15)	-11.51 (-84.91, 61.94)	-3.01 (-79.20, 71.35)	-8.28 (-84.15, 66.32)	-6.38 (-80.57, 66.07)	13.49 (-61.16, 87.76)	14.47 (-59.29, 88.87)	29.23 (-42.98, 106.72)	NAT300mgIV	-11.65 (-63.32, 39.39)
PBO	12.23 (-39.32, 62.58)	0.02 (-52.94, 54.20)	8.39 (-43.93, 61.87)	3.00 (-50.56, 57.46)	5.25 (-47.48, 59.34)	25.13 (-29.14, 77.99)	26.02 (-27.69, 79.60)	40.87 (-12.61, 95.89)	11.65 (-39.39, 63.32)	PBO

Table S18l: League table for serious adverse events for the low risk of bias studies. The table presented the multiple treatment comparisons based on consistency analysis of the networks. Treatments are depicted alphabetically. Values are presented as relative risk (RR) with 95% credible interval (CrI). For all comparisons an RR >1 favors the occurrence of the event for the row-defining treatment, while an RR<1 favors the column-defining treatment. Statistically significant results are represented in bold. Multiple treatment comparisons based on consistency analysis of the networks. Treatments are depicted alphabetically. Values are presented as relative risk (RR) with 95% credible interval (CrI). For all comparisons an RR <1 favors the occurrence of the event for the row-defining treatment, while an RR>1 favors the column-defining treatment. Statistically significant results are represented in bold Each tab of the spreadsheet presents data from a network constructed for the safety outcomes.

	ADA160mg80mg40mgSC	ADA160mg80mg60mgSC	ADA160mg80mgSC	ETR105mgSC	ETR210mgSC	FIL200mgPO	GUS1200mgIV	GUS200mgIV	GUS600mgIV	MIR1000mgIV	MIR200mgIV	MIR600mgIV	NNC2mg_kgSC	ONE10mgSC	ONE25mgSC	ONE35mgSC	ONE50mgSC	ONT22_5mgSC	ONT225mgSC
ADA160mg80mg40mgSC	ADA160mg80mg40mgSC	2.03 (0.06, 109.48)	0.17 (0.01, 2.63)	0.82 (0.07, 10.47)	0.53 (0.05, 6.81)	1.85 (0.14, 27.33)	0.13 (0.00, 1.74)	0.55 (0.06, 4.73)	0.78 (0.10, 6.53)	0.18 (0.01, 2.86)	0.15 (0.00, 3.19)	0.63 (0.04, 8.75)	2.30 (0.13, 43.98)	0.32 (0.00, 10.62)	0.32 (0.00, 9.51)	1.34 (0.07, 28.96)	0.29 (0.01, 10.22)	1.76 (0.14, 21.63)	1.77 (0.14, 21.66)
ADA160mg80mg60mgSC	0.49 (0.01, 17.25)	ADA160mg80mg60mgSC	0.08 (0.00, 2.58)	0.41 (0.01, 9.80)	0.27 (0.01, 6.26)	0.92 (0.02, 26.62)	0.06 (0.00, 2.79)	0.28 (0.01, 7.76)	0.39 (0.01, 10.69)	0.09 (0.00, 3.00)	0.07 (0.00, 2.85)	0.31 (0.01, 8.88)	1.10 (0.02, 39.99)	0.15 (0.00, 10.67)	0.15 (0.00, 10.02)	0.65 (0.01, 27.04)	0.14 (0.00, 10.85)	0.86 (0.02, 20.93)	0.85 (0.02, 20.74)
ADA160mg80mgSC	5.96 (0.38, 124.56)	12.29 (0.39, 728.68)	ADA160mg80mgSC	4.77 (0.52, 63.65)	3.12 (0.34, 42.43)	**11.04 (1.04, 167.56)**	0.76 (0.01, 16.75)	3.24 (0.24, 55.57)	4.60 (0.38, 76.64)	1.10 (0.07, 18.92)	0.90 (0.02, 18.36)	3.87 (0.28, 55.36)	13.44 (0.99, 279.78)	1.93 (0.02, 73.83)	1.86 (0.03, 58.05)	7.92 (0.45, 170.77)	1.78 (0.04, 67.45)	**10.39 (1.02, 149.08)**	10.07 (0.99, 141.69)
ETR105mgSC	1.21 (0.10, 14.04)	2.44 (0.10, 112.34)	0.21 (0.02, 1.91)	ETR105mgSC	0.65 (0.17, 2.57)	2.23 (0.29, 20.56)	0.17 (0.00, 2.24)	0.66 (0.06, 6.57)	0.95 (0.10, 8.00)	0.23 (0.02, 2.07)	0.19 (0.01, 2.26)	0.77 (0.08, 5.98)	2.71 (0.27, 34.58)	0.39 (0.01, 9.29)	0.39 (0.01, 8.53)	1.57 (0.13, 24.82)	0.37 (0.01, 9.55)	2.12 (0.31, 15.90)	2.09 (0.30, 15.30)
ETR210mgSC	1.88 (0.15, 21.10)	3.73 (0.16, 189.86)	0.32 (0.02, 2.94)	1.53 (0.39, 5.81)	ETR210mgSC	3.43 (0.43, 32.45)	0.25 (0.00, 3.62)	1.02 (0.09, 10.05)	1.46 (0.14, 12.30)	0.34 (0.03, 3.18)	0.30 (0.01, 3.71)	1.20 (0.11, 9.99)	4.18 (0.40, 51.76)	0.60 (0.01, 15.14)	0.59 (0.01, 15.67)	2.41 (0.19, 38.39)	0.55 (0.02, 14.18)	3.30 (0.44, 25.03)	3.24 (0.42, 24.50)
FIL200mgPO	0.54 (0.04, 7.07)	1.09 (0.04, 50.19)	**0.09 (0.01, 0.96)**	0.45 (0.05, 3.45)	0.29 (0.03, 2.34)	FIL200mgPO	0.07 (0.00, 1.11)	0.29 (0.02, 3.08)	0.41 (0.04, 3.92)	0.10 (0.01, 1.09)	0.08 (0.00, 1.19)	0.34 (0.03, 3.03)	1.18 (0.09, 16.47)	0.17 (0.00, 4.74)	0.17 (0.00, 4.66)	0.69 (0.04, 11.74)	0.16 (0.00, 4.77)	0.95 (0.10, 8.24)	0.94 (0.10, 7.99)
GUS1200mgIV	7.60 (0.58, 398.61)	17.34 (0.36, 2174.28)	1.32 (0.06, 85.25)	6.03 (0.45, 371.37)	4.03 (0.28, 240.49)	14.35 (0.90, 921.45)	GUS1200mgIV	4.18 (0.35, 198.34)	5.85 (0.57, 248.22)	1.46 (0.06, 86.42)	1.20 (0.02, 94.12)	4.93 (0.27, 298.98)	17.96 (0.81, 1387.51)	2.57 (0.02, 229.82)	2.51 (0.03, 313.59)	10.44 (0.45, 907.85)	2.47 (0.04, 226.36)	13.45 (0.88, 782.47)	13.31 (0.90, 769.92)
GUS200mgIV	1.80 (0.21, 17.80)	3.63 (0.13, 191.33)	0.31 (0.02, 4.20)	1.51 (0.15, 15.63)	0.98 (0.10, 10.62)	3.42 (0.33, 42.79)	0.24 (0.01, 2.83)	GUS200mgIV	1.40 (0.23, 9.88)	0.34 (0.02, 4.79)	0.28 (0.01, 5.51)	1.17 (0.09, 13.58)	4.13 (0.28, 72.36)	0.58 (0.01, 16.37)	0.58 (0.01, 15.45)	2.36 (0.15, 51.82)	0.55 (0.01, 16.91)	3.21 (0.31, 35.68)	3.21 (0.29, 36.45)
GUS600mgIV	1.28 (0.15, 10.35)	2.59 (0.09, 125.53)	0.22 (0.01, 2.65)	1.05 (0.13, 10.05)	0.68 (0.08, 7.18)	2.42 (0.26, 27.73)	0.17 (0.00, 1.74)	0.72 (0.10, 4.34)	GUS600mgIV	0.24 (0.02, 2.85)	0.20 (0.00, 3.45)	0.82 (0.07, 8.63)	2.95 (0.22, 44.28)	0.42 (0.00, 12.56)	0.41 (0.01, 10.81)	1.70 (0.11, 31.78)	0.38 (0.01, 12.50)	2.27 (0.26, 23.73)	2.23 (0.25, 22.05)
MIR1000mgIV	5.46 (0.35, 103.79)	11.54 (0.33, 631.46)	0.91 (0.05, 14.62)	4.34 (0.48, 53.03)	2.90 (0.31, 38.53)	10.15 (0.92, 150.51)	0.69 (0.01, 16.06)	2.95 (0.21, 51.36)	4.22 (0.35, 61.08)	MIR1000mgIV	0.87 (0.03, 12.16)	3.41 (0.40, 35.50)	12.13 (0.80, 253.25)	1.73 (0.02, 66.90)	1.65 (0.03, 69.89)	7.21 (0.41, 187.11)	1.65 (0.03, 62.17)	9.38 (0.98, 126.78)	9.25 (0.94, 126.80)
MIR200mgIV	6.52 (0.31, 323.57)	14.32 (0.35, 1406.20)	1.11 (0.05, 49.13)	5.18 (0.44, 188.53)	3.31 (0.27, 130.64)	11.94 (0.84, 510.66)	0.83 (0.01, 50.83)	3.58 (0.18, 168.44)	5.10 (0.29, 205.56)	1.15 (0.08, 38.36)	MIR200mgIV	3.99 (0.38, 126.53)	15.24 (0.78, 725.99)	2.04 (0.02, 178.98)	2.02 (0.03, 169.83)	8.55 (0.42, 520.57)	1.95 (0.04, 173.03)	11.05 (0.86, 443.36)	10.93 (0.86, 442.34)
MIR600mgIV	1.58 (0.11, 23.18)	3.20 (0.11, 150.86)	0.26 (0.02, 3.58)	1.29 (0.17, 12.03)	0.83 (0.10, 8.76)	2.96 (0.33, 35.05)	0.20 (0.00, 3.76)	0.86 (0.07, 11.36)	1.23 (0.12, 14.38)	0.29 (0.03, 2.48)	0.25 (0.01, 2.64)	MIR600mgIV	3.61 (0.28, 58.31)	0.52 (0.01, 15.45)	0.50 (0.01, 13.57)	2.09 (0.14, 39.30)	0.46 (0.01, 15.35)	2.76 (0.33, 27.30)	2.69 (0.32, 27.37)
NNC2mg_kgSC	0.44 (0.02, 7.42)	0.91 (0.03, 45.65)	0.07 (0.00, 1.01)	0.37 (0.03, 3.65)	0.24 (0.02, 2.48)	0.85 (0.06, 10.55)	0.06 (0.00, 1.24)	0.24 (0.01, 3.52)	0.34 (0.02, 4.45)	0.08 (0.00, 1.25)	0.07 (0.00, 1.29)	0.28 (0.02, 3.52)	NNC2mg_kgSC	0.14 (0.00, 4.26)	0.14 (0.00, 3.81)	0.58 (0.03, 10.97)	0.13 (0.00, 3.90)	0.80 (0.06, 8.13)	0.78 (0.06, 7.95)
ONE10mgSC	3.13 (0.09, 307.97)	6.71 (0.09, 1364.91)	0.52 (0.01, 53.95)	2.55 (0.11, 193.57)	1.67 (0.07, 120.60)	5.93 (0.21, 518.96)	0.39 (0.00, 55.01)	1.73 (0.06, 137.07)	2.39 (0.08, 216.17)	0.58 (0.01, 53.53)	0.49 (0.01, 47.59)	1.92 (0.06, 163.59)	7.03 (0.23, 832.71)	ONE10mgSC	1.01 (0.02, 70.08)	3.99 (0.34, 204.72)	0.94 (0.03, 59.55)	5.64 (0.22, 486.67)	5.48 (0.22, 494.84)
ONE25mgSC	3.12 (0.11, 229.48)	6.76 (0.10, 993.03)	0.54 (0.02, 38.79)	2.58 (0.12, 139.30)	1.70 (0.06, 98.26)	5.91 (0.21, 386.78)	0.40 (0.00, 34.42)	1.73 (0.06, 121.55)	2.45 (0.09, 157.30)	0.61 (0.01, 32.20)	0.49 (0.01, 39.90)	2.01 (0.07, 115.06)	7.34 (0.26, 542.01)	0.99 (0.01, 46.31)	ONE25mgSC	3.95 (0.38, 169.24)	0.98 (0.02, 51.46)	5.53 (0.25, 337.49)	5.41 (0.22, 325.44)
ONE35mgSC	0.75 (0.03, 15.36)	1.53 (0.04, 106.72)	0.13 (0.01, 2.22)	0.64 (0.04, 7.95)	0.42 (0.03, 5.32)	1.44 (0.09, 23.18)	0.10 (0.00, 2.22)	0.42 (0.02, 6.64)	0.59 (0.03, 9.09)	0.14 (0.01, 2.43)	0.12 (0.00, 2.38)	0.48 (0.03, 7.22)	1.73 (0.09, 37.48)	0.25 (0.00, 2.92)	0.25 (0.01, 2.65)	ONE35mgSC	0.23 (0.01, 2.94)	1.38 (0.08, 17.57)	1.33 (0.08, 17.16)
ONE50mgSC	3.40 (0.10, 173.39)	7.11 (0.09, 805.37)	0.56 (0.01, 22.50)	2.74 (0.10, 98.79)	1.82 (0.07, 65.73)	6.41 (0.21, 267.01)	0.40 (0.00, 24.71)	1.82 (0.06, 83.54)	2.61 (0.08, 101.24)	0.61 (0.02, 29.77)	0.51 (0.01, 28.24)	2.16 (0.07, 90.12)	7.45 (0.26, 417.76)	1.06 (0.02, 37.45)	1.02 (0.02, 40.81)	4.26 (0.34, 120.82)	ONE50mgSC	5.89 (0.23, 211.64)	5.83 (0.23, 208.01)
ONT22_5mgSC	0.57 (0.05, 7.19)	1.17 (0.05, 51.98)	**0.10 (0.01, 0.98)**	0.47 (0.06, 3.26)	0.30 (0.04, 2.25)	1.05 (0.12, 10.21)	0.07 (0.00, 1.14)	0.31 (0.03, 3.27)	0.44 (0.04, 3.89)	0.11 (0.01, 1.02)	0.09 (0.00, 1.16)	0.36 (0.04, 3.00)	1.25 (0.12, 16.71)	0.18 (0.00, 4.59)	0.18 (0.00, 4.01)	0.72 (0.06, 13.05)	0.17 (0.00, 4.33)	ONT22_5mgSC	0.98 (0.27, 3.43)
ONT225mgSC	0.56 (0.05, 7.35)	1.18 (0.05, 51.74)	0.10 (0.01, 1.01)	0.48 (0.07, 3.39)	0.31 (0.04, 2.36)	1.06 (0.13, 10.26)	0.08 (0.00, 1.11)	0.31 (0.03, 3.40)	0.45 (0.05, 4.05)	0.11 (0.01, 1.06)	0.09 (0.00, 1.17)	0.37 (0.04, 3.15)	1.28 (0.13, 17.07)	0.18 (0.00, 4.48)	0.18 (0.00, 4.65)	0.75 (0.06, 12.55)	0.17 (0.00, 4.33)	1.02 (0.29, 3.77)	ONT225mgSC

(**TABLE S18L.** continued)

	ADA160mg80mg40mgSC	ADA160mg80mg60mgSC	ADA160mg80mgSC	ETR105mgSC	ETR210mgSC	FIL200mgPO	GUS1200mgIV	GUS200mgIV	GUS600mgIV	MIR1000mgIV	MIR200mgIV	MIR600mgIV	NNC2mg_kgSC	ONE10mgSC	ONE25mgSC	ONE35mgSC	ONE50mgSC	ONT22_5mgSC	ONT225mgSC
ONT75mgSC	0.68 (0.05, 8.36)	1.43 (0.06, 68.18)	0.12 (0.01, 1.15)	0.57 (0.08, 4.05)	0.36 (0.05, 2.80)	1.28 (0.15, 12.33)	0.09 (0.00, 1.39)	0.38 (0.03, 3.96)	0.53 (0.05, 4.79)	0.13 (0.01, 1.33)	0.11 (0.00, 1.42)	0.44 (0.04, 3.75)	1.55 (0.15, 19.67)	0.22 (0.00, 5.38)	0.22 (0.00, 5.30)	0.90 (0.07, 13.86)	0.21 (0.01, 5.43)	1.22 (0.33, 4.50)	1.18 (0.32, 4.36)
PBO	1.24 (0.16, 10.22)	2.49 (0.15, 91.52)	0.22 (0.02, 1.28)	1.03 (0.27, 4.05)	0.67 (0.17, 2.82)	2.28 (0.53, 13.00)	0.17 (0.00, 1.62)	0.69 (0.10, 4.38)	0.97 (0.17, 5.58)	0.24 (0.03, 1.39)	0.20 (0.01, 1.63)	0.82 (0.13, 3.82)	2.74 (0.44, 24.34)	0.41 (0.01, 6.74)	0.41 (0.01, 6.78)	1.64 (0.18, 17.65)	0.38 (0.01, 7.37)	2.20 (0.54, 9.97)	2.16 (0.52, 9.35)
PF10mgSC	1.61 (0.13, 19.55)	3.12 (0.14, 137.71)	0.27 (0.02, 2.74)	1.29 (0.18, 9.36)	0.84 (0.12, 6.74)	2.92 (0.38, 27.49)	0.21 (0.00, 3.09)	0.87 (0.08, 8.83)	1.23 (0.14, 11.40)	0.29 (0.02, 2.89)	0.25 (0.01, 3.29)	1.01 (0.10, 8.47)	3.60 (0.34, 44.59)	0.49 (0.01, 13.37)	0.51 (0.01, 10.89)	2.06 (0.16, 34.90)	0.46 (0.01, 13.10)	2.75 (0.38, 20.39)	2.73 (0.40, 20.55)
PF200mgSC	0.59 (0.05, 6.94)	1.18 (0.05, 50.15)	**0.10 (0.01, 0.91)**	0.48 (0.07, 3.43)	0.31 (0.05, 2.33)	1.09 (0.14, 10.06)	0.08 (0.00, 1.05)	0.32 (0.03, 3.20)	0.45 (0.06, 3.88)	0.11 (0.01, 1.05)	0.09 (0.00, 1.12)	0.38 (0.04, 2.95)	1.32 (0.14, 15.97)	0.19 (0.00, 4.58)	0.19 (0.00, 4.38)	0.76 (0.06, 11.80)	0.17 (0.01, 4.47)	1.05 (0.16, 6.99)	1.03 (0.15, 7.18)
PF50mgSC	1.29 (0.10, 16.44)	2.59 (0.12, 106.72)	0.22 (0.02, 1.98)	1.06 (0.15, 7.28)	0.69 (0.10, 5.01)	2.38 (0.31, 22.16)	0.17 (0.00, 2.53)	0.70 (0.07, 6.71)	0.99 (0.12, 8.91)	0.24 (0.02, 2.27)	0.21 (0.01, 2.54)	0.83 (0.09, 6.33)	2.88 (0.28, 35.97)	0.41 (0.01, 11.09)	0.42 (0.01, 9.35)	1.68 (0.14, 25.76)	0.38 (0.01, 10.58)	2.29 (0.32, 16.04)	2.24 (0.33, 16.63)
RIS200mgIV	2.06 (0.16, 24.55)	4.18 (0.17, 230.89)	0.35 (0.03, 3.31)	1.63 (0.25, 12.63)	1.07 (0.15, 8.40)	3.77 (0.53, 35.43)	0.26 (0.00, 4.00)	1.11 (0.11, 11.11)	1.56 (0.17, 14.50)	0.39 (0.03, 3.71)	0.31 (0.01, 4.48)	1.28 (0.13, 10.76)	4.50 (0.46, 59.89)	0.65 (0.01, 15.63)	0.65 (0.01, 13.60)	2.63 (0.21, 42.63)	0.60 (0.02, 15.99)	3.52 (0.52, 28.96)	3.46 (0.48, 26.31)
RIS600mgIV	4.41 (0.33, 63.76)	8.85 (0.33, 523.73)	0.74 (0.05, 8.30)	3.54 (0.46, 32.06)	2.35 (0.29, 21.80)	8.10 (1.00, 93.98)	0.57 (0.01, 10.15)	2.42 (0.21, 30.42)	3.42 (0.33, 38.98)	0.83 (0.06, 9.49)	0.68 (0.02, 10.64)	2.80 (0.26, 28.54)	9.94 (0.87, 151.89)	1.40 (0.02, 40.35)	1.38 (0.02, 40.63)	5.74 (0.39, 107.40)	1.29 (0.03, 41.98)	7.77 (0.98, 76.27)	7.53 (0.91, 73.17)
SEC10mg_kgIV	0.98 (0.06, 12.04)	1.87 (0.07, 100.32)	0.16 (0.01, 1.80)	0.78 (0.09, 6.16)	0.52 (0.05, 4.26)	1.80 (0.17, 18.70)	0.12 (0.00, 1.97)	0.53 (0.04, 5.68)	0.75 (0.06, 7.10)	0.17 (0.01, 2.01)	0.15 (0.00, 2.24)	0.60 (0.05, 5.76)	2.18 (0.17, 30.89)	0.30 (0.00, 8.47)	0.30 (0.00, 7.64)	1.25 (0.08, 22.53)	0.28 (0.01, 8.77)	1.65 (0.16, 15.16)	1.63 (0.17, 14.65)
TES400mg200mgSC	0.11 (0.00, 2.24)	0.21 (0.00, 15.10)	**0.02 (0.00, 0.33)**	0.09 (0.00, 1.19)	**0.06 (0.00, 0.77)**	0.22 (0.01, 3.03)	**0.01 (0.00, 0.36)**	0.06 (0.00, 1.03)	0.09 (0.00, 1.38)	**0.02 (0.00, 0.38)**	**0.02 (0.00, 0.40)**	0.07 (0.00, 1.05)	0.25 (0.01, 5.18)	0.03 (0.00, 1.13)	0.03 (0.00, 1.12)	0.14 (0.00, 3.49)	0.03 (0.00, 1.10)	0.20 (0.01, 2.67)	0.20 (0.01, 2.68)
TOF15mgPO_BID	9.74 (0.42, 674.87)	21.68 (0.48, 2386.56)	1.65 (0.07, 101.55)	7.69 (0.54, 418.61)	5.18 (0.33, 222.33)	**18.69 (1.07, 740.01)**	1.21 (0.02, 97.48)	5.44 (0.25, 307.07)	7.60 (0.37, 427.85)	1.80 (0.07, 76.60)	1.48 (0.03, 95.83)	6.01 (0.31, 342.18)	**21.94 (1.11, 1294.79)**	3.07 (0.03, 324.33)	2.99 (0.04, 302.54)	12.83 (0.51, 1017.74)	2.81 (0.05, 270.50)	**16.88 (1.07, 806.51)**	**16.33 (1.10, 762.99)**
TOF1mgPO_BID	1.74 (0.13, 25.87)	3.49 (0.13, 162.21)	0.29 (0.02, 3.53)	1.40 (0.17, 12.63)	0.92 (0.11, 8.67)	3.20 (0.36, 35.30)	0.22 (0.00, 4.06)	0.96 (0.07, 10.98)	1.34 (0.13, 14.65)	0.32 (0.02, 3.57)	0.27 (0.01, 4.18)	1.11 (0.09, 10.51)	3.88 (0.33, 62.42)	0.56 (0.01, 15.63)	0.55 (0.01, 13.05)	2.35 (0.15, 42.19)	0.51 (0.01, 16.02)	3.03 (0.33, 29.51)	2.96 (0.34, 28.33)
TOF5mgPO_BID	1.61 (0.11, 22.55)	3.27 (0.12, 157.70)	0.27 (0.02, 3.24)	1.34 (0.16, 11.35)	0.88 (0.10, 7.74)	3.04 (0.34, 30.31)	0.21 (0.00, 3.62)	0.90 (0.07, 10.27)	1.26 (0.12, 13.52)	0.30 (0.02, 3.45)	0.26 (0.01, 3.92)	1.06 (0.09, 9.66)	3.67 (0.32, 53.98)	0.51 (0.01, 15.04)	0.50 (0.01, 12.77)	2.16 (0.14, 38.40)	0.48 (0.01, 15.74)	2.90 (0.34, 25.51)	2.77 (0.35, 25.72)
UPA12mgPO_BID	0.20 (0.01, 3.05)	0.41 (0.01, 21.33)	**0.03 (0.00, 0.41)**	0.17 (0.01, 1.43)	0.11 (0.01, 1.07)	0.39 (0.03, 4.26)	**0.03 (0.00, 0.48)**	0.11 (0.01, 1.43)	0.16 (0.01, 1.87)	**0.04 (0.00, 0.48)**	**0.03 (0.00, 0.52)**	0.13 (0.01, 1.34)	0.45 (0.03, 6.75)	0.06 (0.00, 1.99)	0.06 (0.00, 2.03)	0.26 (0.01, 5.17)	0.06 (0.00, 1.85)	0.37 (0.03, 3.43)	0.36 (0.03, 3.20)
UPA24mgPO	0.28 (0.02, 4.94)	0.58 (0.02, 33.97)	**0.05 (0.00, 0.63)**	0.24 (0.02, 2.37)	0.16 (0.01, 1.61)	0.55 (0.04, 6.73)	**0.04 (0.00, 0.76)**	0.16 (0.01, 2.23)	0.23 (0.02, 2.86)	**0.05 (0.00, 0.75)**	**0.05 (0.00, 0.76)**	0.19 (0.01, 2.01)	0.66 (0.04, 10.40)	0.09 (0.00, 2.98)	0.09 (0.00, 3.05)	0.38 (0.02, 7.47)	0.09 (0.00, 2.86)	0.52 (0.04, 5.26)	0.51 (0.04, 4.99)
UPA24mgPO_BID	0.72 (0.04, 15.40)	1.52 (0.04, 99.60)	0.12 (0.01, 2.07)	0.62 (0.05, 7.24)	0.40 (0.03, 5.27)	1.40 (0.09, 21.05)	0.09 (0.00, 2.13)	0.41 (0.02, 6.50)	0.59 (0.04, 9.03)	0.14 (0.01, 2.39)	0.11 (0.00, 2.42)	0.47 (0.03, 6.96)	1.70 (0.10, 34.58)	0.24 (0.00, 8.98)	0.23 (0.00, 7.43)	1.00 (0.04, 21.56)	0.21 (0.00, 8.83)	1.32 (0.09, 16.85)	1.27 (0.10, 15.85)
UPA3mgPO_BID	0.45 (0.02, 8.09)	0.95 (0.03, 54.67)	0.08 (0.00, 1.11)	0.39 (0.03, 3.72)	0.25 (0.02, 2.65)	0.86 (0.06, 10.95)	0.06 (0.00, 1.22)	0.25 (0.02, 3.31)	0.36 (0.02, 4.57)	0.09 (0.00, 1.20)	0.07 (0.00, 1.26)	0.29 (0.02, 3.34)	1.03 (0.06, 16.79)	0.14 (0.00, 4.55)	0.14 (0.00, 4.66)	0.60 (0.03, 12.50)	0.13 (0.00, 5.01)	0.83 (0.07, 8.93)	0.81 (0.06, 8.86)
UPA45mgPO	1.29 (0.13, 12.47)	2.54 (0.14, 101.36)	0.22 (0.02, 1.57)	1.05 (0.23, 5.57)	0.68 (0.14, 3.88)	2.36 (0.44, 15.93)	0.17 (0.00, 2.01)	0.70 (0.09, 5.56)	0.99 (0.14, 6.60)	0.25 (0.02, 1.71)	0.20 (0.01, 1.96)	0.83 (0.11, 4.74)	2.84 (0.37, 29.66)	0.42 (0.01, 8.21)	0.42 (0.01, 7.08)	1.69 (0.16, 20.94)	0.38 (0.01, 8.41)	2.25 (0.44, 12.44)	2.22 (0.43, 12.38)
UPA6mgPO_BID	1.22 (0.06, 29.51)	2.57 (0.07, 163.52)	0.21 (0.01, 4.72)	1.03 (0.07, 16.53)	0.67 (0.05, 11.49)	2.35 (0.15, 47.94)	0.16 (0.00, 4.73)	0.69 (0.03, 13.95)	0.99 (0.05, 18.15)	0.23 (0.01, 5.15)	0.19 (0.00, 5.10)	0.79 (0.04, 14.02)	2.87 (0.16, 66.12)	0.39 (0.00, 18.49)	0.38 (0.01, 15.83)	1.69 (0.07, 47.22)	0.37 (0.01, 18.06)	2.20 (0.15, 37.00)	2.13 (0.14, 35.80)
UST130mgIV	1.55 (0.17, 14.62)	3.05 (0.16, 127.42)	0.26 (0.03, 1.99)	1.26 (0.26, 6.43)	0.83 (0.16, 4.39)	2.84 (0.49, 20.86)	0.21 (0.00, 2.35)	0.85 (0.10, 6.98)	1.19 (0.17, 8.34)	0.29 (0.03, 2.13)	0.24 (0.01, 2.44)	0.99 (0.13, 6.01)	3.42 (0.43, 36.63)	0.49 (0.01, 10.35)	0.50 (0.01, 8.61)	2.02 (0.19, 24.28)	0.47 (0.01, 10.10)	2.73 (0.52, 15.56)	2.66 (0.51, 14.87)
UST6mg_kg90mgIV_SC	1.27 (0.40, 3.93)	2.57 (0.09, 120.04)	0.21 (0.01, 2.68)	1.05 (0.12, 9.68)	0.68 (0.08, 6.55)	2.37 (0.23, 28.69)	0.17 (0.00, 1.70)	0.70 (0.10, 4.52)	0.98 (0.17, 5.82)	0.24 (0.01, 2.93)	0.20 (0.00, 3.34)	0.79 (0.07, 8.70)	2.87 (0.22, 46.23)	0.41 (0.00, 11.26)	0.41 (0.01, 9.61)	1.69 (0.10, 30.69)	0.38 (0.01, 12.23)	2.24 (0.21, 22.08)	2.25 (0.22, 22.00)
UST6mg_kgIV	1.48 (0.16, 15.23)	3.01 (0.16, 124.02)	0.26 (0.02, 1.96)	1.21 (0.26, 6.52)	0.79 (0.17, 4.72)	2.74 (0.50, 19.75)	0.20 (0.00, 2.41)	0.81 (0.10, 6.88)	1.13 (0.16, 8.21)	0.28 (0.03, 2.13)	0.24 (0.01, 2.42)	0.96 (0.14, 6.23)	3.33 (0.43, 35.89)	0.47 (0.01, 10.27)	0.48 (0.01, 9.07)	1.94 (0.19, 26.58)	0.44 (0.01, 10.44)	2.59 (0.53, 16.12)	2.55 (0.49, 14.89)
VED300mgIV	1.15 (0.12, 11.67)	2.31 (0.12, 94.07)	0.20 (0.02, 1.44)	0.94 (0.19, 4.85)	0.61 (0.12, 3.34)	2.09 (0.39, 14.89)	0.15 (0.00, 1.77)	0.63 (0.08, 5.05)	0.89 (0.13, 6.06)	0.22 (0.02, 1.52)	0.19 (0.01, 1.77)	0.74 (0.10, 4.38)	2.52 (0.33, 26.81)	0.37 (0.00, 7.28)	0.37 (0.01, 7.35)	1.52 (0.14, 18.28)	0.34 (0.01, 7.75)	2.03 (0.39, 11.49)	1.97 (0.37, 11.12)

(**TABLE S18L.** continued)

	ONT75mgSC	PBO	PF10mgSC	PF200mgSC	PF50mgSC	RIS200mgIV	RIS600mgIV	SEC10mg_kgIV	TES400mg200mgSC	TOF15mgPO_BID	TOF1mgPO_BID	TOF5mgPO_BID	UPA12mgPO_BID	UPA24mgPO	UPA24mgPO_BID	UPA3mgPO_BID	UPA45mgPO	UPA6mgPO_BID	UST130mgIV	UST6mg_kg90mgIV_SC	UST6mg_kgIV	VED300mgIV
ADA160mg80mg40mgSC	1.48 (0.12, 18.45)	0.80 (0.10, 6.30)	0.62 (0.05, 7.70)	1.69 (0.14, 19.79)	0.77 (0.06, 9.54)	0.49 (0.04, 6.15)	0.23 (0.02, 3.04)	1.02 (0.08, 16.94)	8.94 (0.45, 383.53)	0.10 (0.00, 2.40)	0.58 (0.04, 7.63)	0.62 (0.04, 8.91)	4.95 (0.33, 95.46)	3.56 (0.20, 62.81)	1.39 (0.06, 27.46)	2.24 (0.12, 40.84)	0.77 (0.08, 7.43)	0.82 (0.03, 18.17)	0.65 (0.07, 6.02)	0.79 (0.25, 2.51)	0.68 (0.07, 6.20)	0.87 (0.09, 8.61)
ADA160mg80mg60mgSC	0.70 (0.01, 17.09)	0.40 (0.01, 6.82)	0.32 (0.01, 7.00)	0.85 (0.02, 18.37)	0.39 (0.01, 8.46)	0.24 (0.00, 6.00)	0.11 (0.00, 3.00)	0.53 (0.01, 15.12)	4.66 (0.07, 322.26)	0.05 (0.00, 2.07)	0.29 (0.01, 7.75)	0.31 (0.01, 8.55)	2.42 (0.05, 84.01)	1.73 (0.03, 56.76)	0.66 (0.01, 25.43)	1.05 (0.02, 37.99)	0.39 (0.01, 7.33)	0.39 (0.01, 14.59)	0.33 (0.01, 6.16)	0.39 (0.01, 10.81)	0.33 (0.01, 6.36)	0.43 (0.01, 8.07)
ADA160mg80mgSC	8.54 (0.87, 116.92)	4.60 (0.78, 41.88)	3.75 (0.36, 48.89)	**9.88 (1.10, 129.39)**	4.50 (0.51, 60.49)	2.85 (0.30, 38.81)	1.34 (0.12, 18.84)	6.33 (0.56, 111.50)	**56.51 (3.07, 2324.92)**	0.61 (0.01, 14.47)	3.44 (0.28, 53.11)	3.66 (0.31, 57.22)	**28.81 (2.42, 595.38)**	**20.04 (1.58, 399.93)**	8.01 (0.48, 169.44)	12.70 (0.90, 274.41)	4.49 (0.64, 47.28)	4.84 (0.21, 115.88)	3.80 (0.50, 39.88)	4.67 (0.37, 76.30)	3.92 (0.51, 40.68)	5.02 (0.69, 54.00)
ETR105mgSC	1.75 (0.25, 13.14)	0.97 (0.25, 3.69)	0.77 (0.11, 5.42)	2.06 (0.29, 13.45)	0.94 (0.14, 6.49)	0.61 (0.08, 4.07)	0.28 (0.03, 2.20)	1.28 (0.16, 11.19)	10.86 (0.84, 360.41)	0.13 (0.00, 1.86)	0.71 (0.08, 5.90)	0.75 (0.09, 6.12)	5.87 (0.70, 68.59)	4.20 (0.42, 47.20)	1.62 (0.14, 21.63)	2.55 (0.27, 31.42)	0.96 (0.18, 4.41)	0.97 (0.06, 14.06)	0.80 (0.16, 3.92)	0.95 (0.10, 8.66)	0.83 (0.15, 3.87)	1.07 (0.21, 5.23)
ETR210mgSC	2.74 (0.36, 19.81)	1.50 (0.36, 5.91)	1.19 (0.15, 8.40)	3.20 (0.43, 20.29)	1.45 (0.20, 9.82)	0.94 (0.12, 6.62)	0.42 (0.05, 3.48)	1.94 (0.23, 18.37)	**16.44 (1.29, 534.48)**	0.19 (0.00, 3.04)	1.08 (0.12, 9.30)	1.13 (0.13, 9.98)	9.06 (0.93, 103.49)	6.38 (0.62, 75.23)	2.50 (0.19, 31.56)	4.05 (0.38, 45.68)	1.47 (0.26, 7.28)	1.49 (0.09, 21.25)	1.21 (0.23, 6.09)	1.46 (0.15, 13.14)	1.27 (0.21, 6.05)	1.64 (0.30, 8.47)
FIL200mgPO	0.78 (0.08, 6.48)	0.44 (0.08, 1.90)	0.34 (0.04, 2.64)	0.92 (0.10, 6.97)	0.42 (0.05, 3.19)	0.27 (0.03, 1.90)	0.12 (0.01, 1.00)	0.55 (0.05, 5.78)	4.62 (0.33, 192.45)	**0.05 (0.00, 0.94)**	0.31 (0.03, 2.77)	0.33 (0.03, 2.92)	2.56 (0.23, 36.45)	1.81 (0.15, 24.49)	0.71 (0.05, 10.71)	1.16 (0.09, 15.87)	0.42 (0.06, 2.27)	0.43 (0.02, 6.88)	0.35 (0.05, 2.04)	0.42 (0.03, 4.39)	0.36 (0.05, 1.98)	0.48 (0.07, 2.58)
GUS1200mgIV	11.08 (0.72, 623.54)	5.84 (0.62, 257.74)	4.78 (0.32, 272.40)	12.84 (0.95, 682.91)	5.93 (0.40, 307.95)	3.82 (0.25, 208.79)	1.75 (0.10, 106.70)	8.21 (0.51, 513.27)	**74.20 (2.78, 11774.38)**	0.82 (0.01, 64.03)	4.65 (0.25, 250.55)	4.86 (0.28, 277.46)	**39.02 (2.06, 2877.58)**	**26.99 (1.31, 1879.84)**	10.78 (0.47, 710.24)	17.26 (0.82, 1204.21)	5.80 (0.50, 264.06)	6.40 (0.21, 477.97)	4.87 (0.43, 254.22)	5.95 (0.59, 245.46)	4.98 (0.42, 239.79)	6.59 (0.57, 327.17)
GUS200mgIV	2.64 (0.25, 29.31)	1.45 (0.23, 10.19)	1.15 (0.11, 12.56)	3.11 (0.31, 31.43)	1.43 (0.15, 14.90)	0.90 (0.09, 9.41)	0.41 (0.03, 4.66)	1.88 (0.18, 26.05)	16.19 (0.97, 673.21)	0.18 (0.00, 3.96)	1.04 (0.09, 13.65)	1.11 (0.10, 14.45)	9.08 (0.70, 154.45)	6.25 (0.45, 96.37)	2.46 (0.15, 43.27)	3.97 (0.30, 66.32)	1.43 (0.18, 11.27)	1.45 (0.07, 29.28)	1.18 (0.14, 10.29)	1.42 (0.22, 10.47)	1.24 (0.15, 10.11)	1.58 (0.20, 13.31)
GUS600mgIV	1.88 (0.21, 18.55)	1.03 (0.18, 5.80)	0.81 (0.09, 7.36)	2.20 (0.26, 17.99)	1.01 (0.11, 8.62)	0.64 (0.07, 6.04)	0.29 (0.03, 3.01)	1.33 (0.14, 16.81)	11.63 (0.72, 456.95)	0.13 (0.00, 2.72)	0.74 (0.07, 7.79)	0.80 (0.07, 8.37)	6.34 (0.53, 93.38)	4.44 (0.35, 63.12)	1.70 (0.11, 27.53)	2.80 (0.22, 41.38)	1.01 (0.15, 7.02)	1.01 (0.06, 18.22)	0.84 (0.12, 6.06)	1.02 (0.17, 5.92)	0.88 (0.12, 6.11)	1.13 (0.17, 7.90)
MIR1000mgIV	7.74 (0.75, 99.57)	4.15 (0.72, 37.24)	3.40 (0.35, 44.17)	9.18 (0.95, 109.88)	4.12 (0.44, 53.90)	2.59 (0.27, 36.16)	1.20 (0.11, 17.43)	5.78 (0.50, 83.29)	**47.88 (2.64, 2641.45)**	0.56 (0.01, 15.06)	3.10 (0.28, 46.45)	3.28 (0.29, 47.60)	**26.84 (2.09, 516.31)**	**18.43 (1.34, 381.97)**	7.27 (0.42, 162.18)	11.72 (0.84, 258.97)	4.05 (0.59, 41.71)	4.37 (0.19, 95.99)	3.39 (0.47, 34.06)	4.23 (0.34, 67.12)	3.55 (0.47, 36.16)	4.55 (0.66, 46.28)
MIR200mgIV	9.29 (0.71, 357.01)	5.01 (0.61, 152.18)	4.02 (0.30, 156.42)	10.74 (0.89, 391.87)	4.86 (0.39, 190.98)	3.18 (0.22, 126.38)	1.48 (0.09, 60.50)	6.81 (0.45, 307.90)	**61.85 (2.47, 5172.16)**	0.68 (0.01, 37.03)	3.69 (0.24, 157.65)	3.91 (0.26, 151.61)	**32.66 (1.93, 1456.60)**	**22.17 (1.31, 1022.97)**	8.92 (0.41, 425.13)	14.07 (0.79, 646.23)	4.88 (0.51, 158.78)	5.35 (0.20, 273.86)	4.09 (0.41, 139.43)	5.13 (0.30, 219.10)	4.22 (0.41, 134.09)	5.39 (0.57, 174.52)
MIR600mgIV	2.27 (0.27, 23.85)	1.23 (0.26, 7.49)	0.99 (0.12, 9.74)	2.64 (0.34, 23.82)	1.21 (0.16, 11.00)	0.78 (0.09, 7.71)	0.36 (0.04, 3.82)	1.67 (0.17, 21.35)	14.21 (0.95, 556.69)	0.17 (0.00, 3.22)	0.90 (0.10, 10.62)	0.94 (0.10, 11.39)	7.76 (0.75, 110.95)	5.37 (0.50, 79.33)	2.13 (0.14, 32.63)	3.43 (0.30, 51.34)	1.20 (0.21, 8.91)	1.26 (0.07, 22.31)	1.01 (0.17, 7.58)	1.26 (0.11, 14.22)	1.04 (0.16, 7.40)	1.35 (0.23, 10.18)
NNC2mg_kgSC	0.64 (0.05, 6.47)	0.36 (0.04, 2.27)	0.28 (0.02, 2.95)	0.76 (0.06, 7.30)	0.35 (0.03, 3.54)	0.22 (0.02, 2.17)	0.10 (0.01, 1.15)	0.46 (0.03, 6.04)	3.93 (0.19, 176.99)	**0.05 (0.00, 0.90)**	0.26 (0.02, 3.03)	0.27 (0.02, 3.12)	2.21 (0.15, 34.02)	1.51 (0.10, 22.82)	0.59 (0.03, 9.91)	0.97 (0.06, 15.83)	0.35 (0.03, 2.67)	0.35 (0.02, 6.40)	0.29 (0.03, 2.34)	0.35 (0.02, 4.64)	0.30 (0.03, 2.34)	0.40 (0.04, 3.00)
ONE10mgSC	4.55 (0.19, 390.37)	2.43 (0.15, 158.83)	2.04 (0.07, 163.53)	5.39 (0.22, 396.38)	2.45 (0.09, 191.50)	1.55 (0.06, 107.53)	0.71 (0.02, 64.46)	3.30 (0.12, 369.83)	29.60 (0.89, 5092.80)	0.33 (0.00, 32.79)	1.80 (0.06, 161.91)	1.95 (0.07, 168.95)	15.75 (0.50, 1329.56)	10.58 (0.34, 1040.46)	4.22 (0.11, 412.68)	6.97 (0.22, 710.69)	2.40 (0.12, 170.82)	2.57 (0.05, 293.95)	2.03 (0.10, 149.63)	2.44 (0.09, 221.84)	2.13 (0.10, 151.65)	2.69 (0.14, 200.91)
ONE25mgSC	4.56 (0.19, 276.70)	2.45 (0.15, 108.83)	1.96 (0.09, 115.95)	5.30 (0.23, 292.01)	2.40 (0.11, 146.14)	1.55 (0.07, 90.44)	0.72 (0.02, 44.16)	3.37 (0.13, 214.11)	30.59 (0.89, 3700.41)	0.33 (0.00, 24.58)	1.82 (0.08, 113.94)	1.99 (0.08, 121.77)	16.30 (0.49, 1044.64)	11.17 (0.33, 709.49)	4.35 (0.13, 284.27)	7.03 (0.21, 460.78)	2.40 (0.14, 112.67)	2.62 (0.06, 173.79)	1.99 (0.12, 97.40)	2.46 (0.10, 162.86)	2.09 (0.11, 108.12)	2.67 (0.14, 140.47)
ONE35mgSC	1.12 (0.07, 15.26)	0.61 (0.06, 5.50)	0.48 (0.03, 6.08)	1.31 (0.08, 16.42)	0.59 (0.04, 7.17)	0.38 (0.02, 4.76)	0.17 (0.01, 2.55)	0.80 (0.04, 12.84)	7.01 (0.29, 329.23)	0.08 (0.00, 1.97)	0.43 (0.02, 6.63)	0.46 (0.03, 7.03)	3.79 (0.19, 70.90)	2.64 (0.13, 51.80)	1.00 (0.05, 23.71)	1.65 (0.08, 35.81)	0.59 (0.05, 6.39)	0.59 (0.02, 14.28)	0.49 (0.04, 5.27)	0.59 (0.03, 9.81)	0.52 (0.04, 5.21)	0.66 (0.05, 6.97)
ONE50mgSC	4.87 (0.18, 187.09)	2.61 (0.14, 73.50)	2.15 (0.08, 73.06)	5.73 (0.22, 194.67)	2.66 (0.09, 86.71)	1.68 (0.06, 62.10)	0.77 (0.02, 30.82)	3.63 (0.11, 145.95)	31.15 (0.91, 3113.94)	0.36 (0.00, 18.67)	1.95 (0.06, 80.32)	2.09 (0.06, 78.00)	17.00 (0.54, 753.72)	11.63 (0.35, 531.89)	4.67 (0.11, 228.87)	7.57 (0.20, 338.93)	2.61 (0.12, 80.74)	2.67 (0.06, 132.62)	2.13 (0.10, 67.22)	2.66 (0.08, 116.14)	2.27 (0.10, 72.29)	2.91 (0.13, 95.60)
ONT22_5mgSC	0.82 (0.22, 2.99)	0.45 (0.10, 1.85)	0.36 (0.05, 2.62)	0.95 (0.14, 6.43)	0.44 (0.06, 3.11)	0.28 (0.03, 1.93)	0.13 (0.01, 1.02)	0.61 (0.07, 6.14)	4.96 (0.37, 180.38)	**0.06 (0.00, 0.94)**	0.33 (0.03, 3.01)	0.34 (0.04, 2.91)	2.73 (0.29, 34.55)	1.93 (0.19, 23.58)	0.76 (0.06, 10.88)	1.21 (0.11, 14.90)	0.44 (0.08, 2.27)	0.45 (0.03, 6.85)	0.37 (0.06, 1.92)	0.45 (0.05, 4.71)	0.39 (0.06, 1.90)	0.49 (0.09, 2.56)
ONT225mgSC	0.85 (0.23, 3.10)	0.46 (0.11, 1.91)	0.37 (0.05, 2.49)	0.98 (0.14, 6.82)	0.45 (0.06, 3.07)	0.29 (0.04, 2.07)	0.13 (0.01, 1.10)	0.61 (0.07, 5.82)	4.94 (0.37, 189.27)	**0.06 (0.00, 0.91)**	0.34 (0.04, 2.91)	0.36 (0.04, 2.84)	2.81 (0.31, 33.47)	1.97 (0.20, 22.76)	0.79 (0.06, 10.53)	1.24 (0.11, 15.41)	0.45 (0.08, 2.30)	0.47 (0.03, 7.01)	0.38 (0.07, 1.96)	0.44 (0.05, 4.49)	0.39 (0.07, 2.03)	0.51 (0.09, 2.67)

(**TABLE S18L.** continued)

	ONT75mgSC	PBO	PF10mgSC	PF200mgSC	PF50mgSC	RIS200mgIV	RIS600mgIV	SEC10mg_kgIV	TES400mg200mgSC	TOF15mgPO_BID	TOF1mgPO_BID	TOF5mgPO_BID	UPA12mgPO_BID	UPA24mgPO	UPA24mgPO_BID	UPA3mgPO_BID	UPA45mgPO	UPA6mgPO_BID	UST130mgIV	UST6mg_kg90mgIV_SC	UST6mg_kgIV	VED300mgIV
ONT75mgSC	ONT75mgSC	0.55 (0.12, 2.29)	0.44 (0.06, 3.13)	1.15 (0.17, 8.31)	0.53 (0.07, 3.78)	0.34 (0.04, 2.50)	0.16 (0.02, 1.35)	0.73 (0.08, 7.04)	6.05 (0.44, 231.45)	0.07 (0.00, 1.16)	0.40 (0.04, 3.35)	0.43 (0.05, 3.49)	3.35 (0.36, 40.01)	2.36 (0.23, 26.69)	0.95 (0.07, 12.59)	1.48 (0.14, 18.61)	0.54 (0.10, 2.77)	0.56 (0.03, 8.11)	0.45 (0.08, 2.51)	0.54 (0.06, 5.12)	0.47 (0.08, 2.49)	0.59 (0.11, 3.26)
PBO	1.81 (0.44, 8.05)	PBO	0.80 (0.19, 3.07)	2.14 (0.57, 8.13)	0.96 (0.25, 3.79)	0.62 (0.15, 2.57)	0.29 (0.05, 1.38)	1.31 (0.26, 8.05)	**10.57 (1.35, 302.24)**	0.14 (0.00, 1.32)	0.74 (0.13, 3.66)	0.78 (0.15, 3.90)	**5.90 (1.17, 48.18)**	4.19 (0.75, 34.58)	1.66 (0.20, 15.98)	2.63 (0.43, 22.95)	0.98 (0.42, 2.34)	0.99 (0.08, 10.56)	0.81 (0.33, 2.02)	0.99 (0.17, 5.66)	0.86 (0.33, 1.95)	1.09 (0.45, 2.63)
PF10mgSC	2.30 (0.32, 17.93)	1.25 (0.33, 5.35)	PF10mgSC	2.65 (0.72, 10.34)	1.22 (0.30, 5.24)	0.78 (0.10, 5.59)	0.36 (0.04, 3.15)	1.67 (0.21, 15.96)	**13.69 (1.16, 482.89)**	0.16 (0.00, 2.65)	0.92 (0.10, 8.15)	0.99 (0.11, 8.32)	7.76 (0.86, 93.91)	5.41 (0.56, 65.69)	2.10 (0.18, 30.76)	3.39 (0.35, 42.23)	1.22 (0.25, 6.60)	1.27 (0.07, 19.93)	1.02 (0.20, 5.63)	1.25 (0.13, 11.84)	1.06 (0.21, 5.52)	1.37 (0.27, 7.58)
PF200mgSC	0.87 (0.12, 5.84)	0.47 (0.12, 1.74)	0.38 (0.10, 1.39)	PF200mgSC	0.46 (0.12, 1.76)	0.29 (0.04, 1.98)	0.13 (0.02, 1.07)	0.61 (0.08, 5.62)	5.09 (0.44, 177.73)	**0.06 (0.00, 0.88)**	0.35 (0.04, 2.84)	0.37 (0.05, 2.83)	2.85 (0.35, 35.07)	1.99 (0.23, 23.55)	0.78 (0.07, 10.18)	1.25 (0.13, 14.73)	0.46 (0.10, 2.14)	0.48 (0.03, 6.97)	0.38 (0.08, 1.88)	0.46 (0.05, 4.13)	0.40 (0.08, 1.92)	0.51 (0.10, 2.54)
PF50mgSC	1.89 (0.26, 13.35)	1.04 (0.26, 3.96)	0.82 (0.19, 3.36)	2.18 (0.57, 8.31)	PF50mgSC	0.63 (0.09, 4.34)	0.29 (0.03, 2.38)	1.37 (0.17, 13.01)	10.96 (0.91, 385.22)	0.13 (0.00, 1.87)	0.76 (0.08, 6.01)	0.81 (0.09, 6.29)	6.23 (0.75, 73.43)	4.34 (0.49, 48.91)	1.75 (0.15, 22.43)	2.76 (0.28, 32.22)	1.01 (0.20, 5.11)	1.05 (0.07, 14.82)	0.83 (0.17, 4.17)	1.02 (0.11, 9.30)	0.88 (0.16, 4.16)	1.13 (0.22, 5.54)
RIS200mgIV	2.94 (0.40, 22.49)	1.61 (0.39, 6.79)	1.28 (0.18, 9.62)	3.44 (0.50, 23.58)	1.58 (0.23, 10.91)	RIS200mgIV	0.47 (0.08, 2.54)	2.13 (0.26, 20.46)	**17.95 (1.45, 639.47)**	0.22 (0.00, 3.18)	1.18 (0.13, 10.55)	1.26 (0.15, 10.32)	**9.75 (1.12, 124.66)**	6.86 (0.72, 84.16)	2.73 (0.22, 37.09)	4.31 (0.43, 58.14)	1.58 (0.30, 8.12)	1.58 (0.11, 25.60)	1.32 (0.25, 6.75)	1.61 (0.17, 14.68)	1.37 (0.24, 6.80)	1.76 (0.35, 9.58)
RIS600mgIV	6.35 (0.74, 60.24)	3.44 (0.72, 20.29)	2.79 (0.32, 23.76)	7.51 (0.93, 60.22)	3.43 (0.42, 29.03)	2.14 (0.39, 12.88)	RIS600mgIV	4.63 (0.49, 51.56)	**39.39 (2.76, 1614.01)**	0.45 (0.01, 8.79)	2.54 (0.25, 27.39)	2.69 (0.27, 28.12)	**21.91 (2.12, 303.86)**	**15.20 (1.38, 220.28)**	5.94 (0.43, 98.11)	9.58 (0.79, 141.17)	3.35 (0.58, 23.33)	3.48 (0.19, 62.28)	2.79 (0.47, 19.94)	3.45 (0.33, 41.23)	2.94 (0.47, 20.41)	3.75 (0.62, 26.67)
SEC10mg_kgIV	1.37 (0.14, 12.43)	0.76 (0.12, 3.79)	0.60 (0.06, 4.83)	1.63 (0.18, 12.65)	0.73 (0.08, 5.91)	0.47 (0.05, 3.84)	0.22 (0.02, 2.03)	SEC10mg_kgIV	8.48 (0.52, 296.67)	0.10 (0.00, 1.86)	0.56 (0.05, 5.42)	0.59 (0.05, 5.63)	4.60 (0.40, 68.49)	3.21 (0.26, 45.85)	1.26 (0.08, 19.06)	2.03 (0.16, 31.37)	0.74 (0.11, 4.39)	0.74 (0.04, 12.05)	0.61 (0.08, 3.73)	0.76 (0.06, 7.55)	0.65 (0.08, 3.82)	0.83 (0.11, 5.10)
TES400mg200mgSC	0.17 (0.00, 2.30)	**0.09 (0.00, 0.74)**	**0.07 (0.00, 0.86)**	0.20 (0.01, 2.26)	0.09 (0.00, 1.10)	**0.06 (0.00, 0.69)**	**0.03 (0.00, 0.36)**	0.12 (0.00, 1.92)	TES400mg200mgSC	**0.01 (0.00, 0.28)**	**0.06 (0.00, 0.94)**	0.07 (0.00, 1.02)	0.53 (0.02, 11.32)	0.37 (0.01, 7.42)	0.14 (0.00, 3.48)	0.24 (0.01, 4.90)	**0.09 (0.00, 0.81)**	0.09 (0.00, 2.16)	**0.08 (0.00, 0.74)**	0.09 (0.00, 1.47)	**0.08 (0.00, 0.72)**	**0.10 (0.00, 0.97)**
TOF15mgPO_BID	13.86 (0.86, 651.44)	7.37 (0.76, 282.61)	6.12 (0.38, 280.21)	**16.16 (1.14, 719.52)**	7.42 (0.53, 387.65)	4.63 (0.31, 224.86)	2.23 (0.11, 95.31)	10.27 (0.54, 589.04)	**90.67 (3.54, 12993.75)**	TOF15mgPO_BID	5.33 (0.55, 208.21)	5.82 (0.57, 217.31)	**48.31 (2.65, 2517.54)**	**34.28 (1.71, 1658.19)**	13.44 (0.54, 723.65)	**21.33 (1.03, 1108.79)**	7.22 (0.63, 319.68)	7.67 (0.27, 508.62)	6.13 (0.50, 264.62)	7.64 (0.39, 451.72)	6.27 (0.53, 269.11)	8.25 (0.69, 351.92)
TOF1mgPO_BID	2.48 (0.30, 24.13)	1.35 (0.27, 7.62)	1.08 (0.12, 9.74)	2.89 (0.35, 23.39)	1.32 (0.17, 11.81)	0.85 (0.09, 7.86)	0.39 (0.04, 4.05)	1.79 (0.18, 20.92)	**15.43 (1.06, 557.04)**	0.19 (0.00, 1.83)	TOF1mgPO_BID	1.06 (0.19, 6.51)	8.34 (0.83, 119.07)	5.84 (0.55, 85.87)	2.32 (0.17, 35.73)	3.67 (0.32, 55.85)	1.33 (0.20, 9.05)	1.35 (0.07, 25.41)	1.10 (0.17, 7.34)	1.34 (0.12, 15.94)	1.15 (0.17, 7.65)	1.48 (0.25, 9.70)
TOF5mgPO_BID	2.35 (0.29, 21.19)	1.28 (0.26, 6.82)	1.01 (0.12, 8.88)	2.73 (0.35, 22.04)	1.24 (0.16, 10.55)	0.79 (0.10, 6.76)	0.37 (0.04, 3.66)	1.71 (0.18, 19.55)	14.25 (0.98, 559.30)	0.17 (0.00, 1.77)	0.94 (0.15, 5.35)	TOF5mgPO_BID	7.90 (0.76, 111.56)	5.59 (0.49, 76.42)	2.20 (0.15, 32.56)	3.51 (0.30, 48.05)	1.26 (0.20, 7.87)	1.30 (0.07, 22.05)	1.05 (0.17, 7.05)	1.27 (0.12, 13.55)	1.09 (0.17, 6.81)	1.40 (0.23, 8.94)
UPA12mgPO_BID	0.30 (0.02, 2.76)	**0.17 (0.02, 0.85)**	0.13 (0.01, 1.16)	0.35 (0.03, 2.86)	0.16 (0.01, 1.34)	**0.10 (0.01, 0.89)**	**0.05 (0.00, 0.47)**	0.22 (0.01, 2.51)	1.87 (0.09, 64.06)	**0.02 (0.00, 0.38)**	0.12 (0.01, 1.21)	0.13 (0.01, 1.31)	UPA12mgPO_BID	0.69 (0.17, 2.61)	0.28 (0.05, 1.31)	0.45 (0.10, 1.82)	0.16 (0.02, 1.02)	**0.17 (0.02, 0.84)**	**0.14 (0.01, 0.87)**	0.16 (0.01, 2.03)	**0.14 (0.01, 0.86)**	0.18 (0.02, 1.17)
UPA24mgPO	0.42 (0.04, 4.40)	0.24 (0.03, 1.34)	0.19 (0.02, 1.78)	0.50 (0.04, 4.39)	0.23 (0.02, 2.04)	0.15 (0.01, 1.38)	**0.07 (0.00, 0.72)**	0.31 (0.02, 3.87)	2.68 (0.13, 101.42)	**0.03 (0.00, 0.58)**	0.17 (0.01, 1.81)	0.18 (0.01, 2.03)	1.44 (0.38, 5.83)	UPA24mgPO	0.40 (0.07, 1.99)	0.65 (0.14, 2.73)	0.23 (0.02, 1.60)	0.24 (0.02, 1.34)	0.19 (0.02, 1.30)	0.23 (0.02, 3.05)	0.20 (0.02, 1.35)	0.26 (0.03, 1.77)
UPA24mgPO_BID	1.05 (0.08, 14.11)	0.60 (0.06, 4.92)	0.48 (0.03, 5.69)	1.28 (0.10, 14.86)	0.57 (0.04, 6.72)	0.37 (0.03, 4.55)	0.17 (0.01, 2.32)	0.79 (0.05, 12.07)	6.95 (0.29, 289.39)	0.07 (0.00, 1.84)	0.43 (0.03, 5.85)	0.46 (0.03, 6.61)	3.62 (0.77, 21.97)	2.50 (0.50, 14.99)	UPA24mgPO_BID	1.59 (0.29, 9.92)	0.59 (0.05, 5.56)	0.60 (0.06, 4.76)	0.49 (0.04, 4.79)	0.57 (0.03, 9.44)	0.51 (0.05, 4.66)	0.66 (0.06, 6.08)
UPA3mgPO_BID	0.67 (0.05, 7.25)	0.38 (0.04, 2.34)	0.29 (0.02, 2.89)	0.80 (0.07, 7.74)	0.36 (0.03, 3.54)	0.23 (0.02, 2.31)	0.10 (0.01, 1.27)	0.49 (0.03, 6.17)	4.16 (0.20, 154.62)	**0.05 (0.00, 0.97)**	0.27 (0.02, 3.17)	0.28 (0.02, 3.37)	2.24 (0.55, 10.20)	1.54 (0.37, 7.02)	0.63 (0.10, 3.41)	UPA3mgPO_BID	0.37 (0.04, 2.72)	0.37 (0.04, 2.33)	0.31 (0.03, 2.31)	0.36 (0.02, 5.09)	0.32 (0.03, 2.25)	0.42 (0.04, 3.16)
UPA45mgPO	1.86 (0.36, 10.44)	1.02 (0.43, 2.37)	0.82 (0.15, 4.07)	2.17 (0.47, 10.51)	0.99 (0.20, 4.96)	0.63 (0.12, 3.29)	0.30 (0.04, 1.71)	1.35 (0.23, 9.40)	**10.94 (1.24, 342.15)**	0.14 (0.00, 1.59)	0.75 (0.11, 4.96)	0.79 (0.13, 5.00)	6.13 (0.98, 61.96)	4.29 (0.63, 41.94)	1.71 (0.18, 18.47)	2.71 (0.37, 27.49)	UPA45mgPO	1.01 (0.08, 12.16)	0.83 (0.24, 2.84)	1.02 (0.14, 7.26)	0.87 (0.24, 2.89)	1.12 (0.33, 3.81)
UPA6mgPO_BID	1.80 (0.12, 29.17)	1.01 (0.09, 11.79)	0.79 (0.05, 13.73)	2.11 (0.14, 34.25)	0.96 (0.07, 14.77)	0.63 (0.04, 9.34)	0.29 (0.02, 5.17)	1.35 (0.08, 25.19)	11.70 (0.46, 635.65)	0.13 (0.00, 3.68)	0.74 (0.04, 13.50)	0.77 (0.05, 15.26)	**6.02 (1.19, 54.21)**	4.16 (0.75, 41.32)	1.65 (0.21, 17.78)	2.69 (0.43, 24.65)	0.99 (0.08, 12.59)	UPA6mgPO_BID	0.82 (0.07, 11.19)	0.99 (0.06, 19.96)	0.85 (0.07, 10.70)	1.09 (0.09, 14.57)
UST130mgIV	2.23 (0.40, 12.61)	1.24 (0.50, 3.07)	0.98 (0.18, 4.88)	2.63 (0.53, 13.22)	1.20 (0.24, 5.78)	0.76 (0.15, 3.98)	0.36 (0.05, 2.14)	1.63 (0.27, 12.08)	**13.26 (1.35, 404.63)**	0.16 (0.00, 1.99)	0.91 (0.14, 5.72)	0.95 (0.14, 5.96)	**7.37 (1.14, 75.00)**	5.21 (0.77, 48.81)	2.05 (0.21, 22.29)	3.26 (0.43, 34.02)	1.20 (0.35, 4.08)	1.22 (0.09, 14.83)	UST130mgIV	1.22 (0.17, 8.95)	1.05 (0.39, 2.45)	1.34 (0.38, 4.76)
UST6mg_kg90mgIV_SC	1.84 (0.20, 18.00)	1.01 (0.18, 6.03)	0.80 (0.08, 7.71)	2.16 (0.24, 19.67)	0.98 (0.11, 9.01)	0.62 (0.07, 5.94)	0.29 (0.02, 3.00)	1.31 (0.13, 16.44)	11.30 (0.68, 423.84)	0.13 (0.00, 2.57)	0.75 (0.06, 8.01)	0.79 (0.07, 8.54)	6.34 (0.49, 94.27)	4.44 (0.33, 65.37)	1.76 (0.11, 28.60)	2.80 (0.20, 44.38)	0.98 (0.14, 7.00)	1.01 (0.05, 18.14)	0.82 (0.11, 5.77)	UST6mg_kg90mgIV_SC	0.86 (0.11, 6.28)	1.12 (0.16, 7.86)
UST6mg_kgIV	2.13 (0.40, 12.86)	1.17 (0.51, 3.04)	0.94 (0.18, 4.86)	2.52 (0.52, 13.20)	1.14 (0.24, 6.11)	0.73 (0.15, 4.10)	0.34 (0.05, 2.11)	1.53 (0.26, 12.40)	**12.75 (1.39, 391.01)**	0.16 (0.00, 1.90)	0.87 (0.13, 5.90)	0.92 (0.15, 5.96)	**7.08 (1.16, 71.56)**	5.00 (0.74, 47.69)	1.95 (0.21, 21.71)	3.10 (0.44, 32.09)	1.15 (0.35, 4.10)	1.18 (0.09, 15.02)	0.95 (0.41, 2.58)	1.16 (0.16, 8.94)	UST6mg_kgIV	1.27 (0.38, 4.95)

VED300m gIV	1.69 (0.31, 9.01)	0.92 (0.38, 2.21)	0.73 (0.13, 3.68)	1.96 (0.39, 9.66)	0.88 (0.18, 4.58)	0.57 (0.10, 2.88)	0.27 (0.04, 1.62)	1.21 (0.20, 8.90)	**9.95 (1.03, 302.41)**	0.12 (0.00, 1.46)	0.67 (0.10, 4.05)	0.72 (0.11, 4.43)	5.45 (0.85, 54.11)	3.88 (0.57, 37.19)	1.52 (0.16, 16.81)	2.40 (0.32, 24.11)	0.89 (0.26, 3.07)	0.91 (0.07, 11.16)	0.75 (0.21, 2.65)	0.90 (0.13, 6.42)	0.79 (0.20, 2.63)	VED300m gIV

Table S18m: League table for serious adverse events for the some concerns studies. The table presented the multiple treatment comparisons based on consistency analysis of the networks. Treatments are depicted alphabetically. Values are presented as relative risk (RR) with 95% credible interval (CrI). For all comparisons an RR >1 favors the occurrence of the event for the row-defining treatment, while an RR<1 favors the column-defining treatment. Statistically significant results are represented in bold. Multiple treatment comparisons based on consistency analysis of the networks. Treatments are depicted alphabetically. Values are presented as relative risk (RR) with 95% credible interval (CrI). For all comparisons an RR <1 favors the occurrence of the event for the row-defining treatment, while an RR>1 favors the column-defining treatment. Statistically significant results are represented in bold.

	ABA10mg_kgIV	ABA30mg_kgIV	ABA3mg_kgIV	ADA160mg80mgSC	ADA40mg20mgSC	ADA80mg40mgSC	AMI0_4mgPO	AND150mgSC1_1	AND150mgSC2_2	AND300mgSC	API100mgPR	API50mgPO	BRI400mgIV	BRI700mgIV	BRO210mgIV	BRO350mgIV	BRO700mgIV	CDP10mg_kgIV	CER100mgSC	CER10mgIV	CER200mgSC	CER20mgIV
ABA10mg_kgIV	ABA10mg_kgIV	0.96 (0.32, 2.72)	0.89 (0.34, 2.41)	0.94 (0.17, 4.89)	0.23 (0.00, 2.62)	0.28 (0.02, 1.85)	6.90 (0.71, 212.72)	1.03 (0.17, 7.57)	0.12 (0.00, 1.56)	1.41 (0.24, 9.93)	0.42 (0.07, 2.48)	0.66 (0.12, 3.51)	0.43 (0.04, 3.36)	0.31 (0.04, 2.03)	1.43 (0.15, 15.33)	4.07 (0.59, 37.33)	4.58 (0.68, 41.74)	0.69 (0.17, 2.84)	1.18 (0.25, 5.56)	0.55 (0.02, 8.96)	1.84 (0.40, 7.86)	2.76 (0.37, 27.72)
ABA30mg_kgIV	1.04 (0.37, 3.17)	ABA30mg_kgIV	0.92 (0.33, 2.83)	0.95 (0.17, 5.57)	0.24 (0.00, 2.84)	0.29 (0.03, 2.13)	7.17 (0.69, 249.28)	1.09 (0.17, 7.99)	0.12 (0.00, 1.67)	1.46 (0.23, 10.86)	0.44 (0.07, 2.60)	0.69 (0.12, 3.81)	0.44 (0.04, 3.69)	0.32 (0.04, 2.18)	1.48 (0.15, 16.70)	4.20 (0.59, 42.20)	4.74 (0.71, 46.65)	0.72 (0.16, 3.17)	1.21 (0.24, 5.99)	0.58 (0.02, 9.32)	1.89 (0.38, 8.90)	2.86 (0.35, 30.77)
ABA3mg_kgIV	1.12 (0.41, 2.98)	1.08 (0.35, 3.07)	ABA3mg_kgIV	1.04 (0.19, 5.66)	0.26 (0.00, 2.95)	0.31 (0.03, 2.22)	7.77 (0.79, 251.46)	1.17 (0.19, 8.22)	0.13 (0.00, 1.74)	1.55 (0.26, 11.32)	0.47 (0.07, 2.70)	0.74 (0.14, 3.90)	0.48 (0.04, 3.72)	0.35 (0.05, 2.27)	1.60 (0.16, 17.16)	4.48 (0.66, 45.02)	5.08 (0.75, 50.85)	0.77 (0.19, 3.27)	1.33 (0.25, 6.27)	0.63 (0.03, 9.87)	2.04 (0.42, 9.34)	3.00 (0.42, 32.24)
ADA160mg80mgSC	1.07 (0.20, 5.76)	1.05 (0.18, 5.74)	0.96 (0.18, 5.17)	ADA160mg80mgSC	0.25 (0.01, 2.18)	0.30 (0.04, 1.50)	7.53 (0.63, 269.17)	1.13 (0.14, 10.28)	0.13 (0.00, 2.13)	1.52 (0.20, 13.08)	0.45 (0.06, 3.47)	0.73 (0.11, 4.67)	0.45 (0.03, 4.80)	0.33 (0.04, 2.85)	1.55 (0.14, 19.91)	4.39 (0.55, 49.78)	4.96 (0.62, 54.41)	0.74 (0.14, 4.14)	1.28 (0.21, 7.59)	0.60 (0.02, 11.92)	1.96 (0.32, 11.10)	2.98 (0.33, 39.69)
ADA40mg20mgSC	4.28 (0.38, 228.49)	4.22 (0.35, 206.06)	3.87 (0.34, 202.86)	3.93 (0.46, 169.81)	ADA40mg20mgSC	1.20 (0.08, 62.77)	**33.13 (1.42, 5503.52)**	4.65 (0.30, 281.60)	0.50 (0.01, 47.72)	6.36 (0.40, 398.90)	1.81 (0.13, 112.59)	2.90 (0.22, 177.97)	1.92 (0.07, 117.43)	1.37 (0.08, 80.79)	6.34 (0.32, 491.42)	**18.76 (1.15, 1134.33)**	**21.30 (1.28, 1420.96)**	2.96 (0.27, 157.88)	5.14 (0.40, 256.57)	2.46 (0.06, 303.89)	7.93 (0.62, 425.33)	12.55 (0.72, 966.19)
ADA80mg40mgSC	3.57 (0.54, 40.08)	3.47 (0.47, 37.78)	3.19 (0.45, 34.86)	3.28 (0.66, 27.09)	0.84 (0.02, 13.30)	ADA80mg40mgSC	**26.26 (1.70, 1101.44)**	3.77 (0.35, 57.66)	0.42 (0.01, 11.21)	5.16 (0.51, 74.26)	1.50 (0.16, 19.98)	2.39 (0.28, 29.38)	1.48 (0.09, 28.93)	1.08 (0.10, 17.61)	5.20 (0.38, 105.78)	**15.10 (1.41, 279.38)**	**16.99 (1.58, 305.29)**	2.47 (0.37, 27.80)	4.25 (0.52, 49.59)	2.04 (0.06, 57.36)	6.49 (0.84, 74.12)	10.30 (0.86, 187.76)
AMI0_4mgPO	0.14 (0.00, 1.40)	0.14 (0.00, 1.45)	0.13 (0.00, 1.27)	0.13 (0.00, 1.59)	**0.03 (0.00, 0.71)**	**0.04 (0.00, 0.59)**	AMI0_4mgPO	0.15 (0.00, 2.12)	**0.02 (0.00, 0.42)**	0.20 (0.01, 3.02)	**0.06 (0.00, 0.73)**	0.10 (0.00, 1.17)	**0.06 (0.00, 0.96)**	**0.04 (0.00, 0.63)**	0.20 (0.00, 4.21)	0.59 (0.01, 10.40)	0.65 (0.02, 12.02)	0.10 (0.00, 1.00)	0.17 (0.00, 1.92)	0.08 (0.00, 2.32)	0.26 (0.01, 2.88)	0.41 (0.01, 7.43)
AND150mgSC1_1	0.97 (0.13, 5.85)	0.92 (0.13, 5.82)	0.85 (0.12, 5.38)	0.88 (0.10, 7.06)	0.21 (0.00, 3.38)	0.27 (0.02, 2.84)	6.66 (0.47, 244.21)	AND150mgSC1_1	**0.12 (0.00, 0.94)**	1.36 (0.37, 4.97)	0.40 (0.04, 3.27)	0.63 (0.08, 4.66)	0.40 (0.02, 4.49)	0.30 (0.03, 2.88)	1.38 (0.10, 19.94)	3.88 (0.36, 49.69)	4.42 (0.40, 54.82)	0.67 (0.10, 4.09)	1.13 (0.13, 8.37)	0.53 (0.02, 10.33)	1.74 (0.22, 11.56)	2.70 (0.23, 35.98)
AND150mgSC2_2	8.32 (0.64, 275.07)	8.04 (0.60, 257.13)	7.52 (0.57, 228.88)	7.90 (0.47, 272.98)	2.00 (0.02, 97.43)	2.37 (0.09, 91.29)	**64.26 (2.38, 6207.35)**	**8.57 (1.07, 229.35)**	AND150mgSC2_2	**11.53 (1.47, 306.36)**	3.51 (0.21, 127.78)	5.63 (0.37, 200.88)	3.59 (0.15, 149.76)	2.65 (0.13, 100.45)	13.08 (0.47, 590.37)	**36.57 (1.69, 1572.81)**	**41.68 (2.01, 1810.32)**	5.94 (0.43, 181.93)	9.98 (0.70, 337.11)	4.84 (0.10, 288.66)	**15.13 (1.02, 489.05)**	**24.08 (1.05, 1045.68)**
AND300mgSC	0.71 (0.10, 4.18)	0.68 (0.09, 4.36)	0.64 (0.09, 3.86)	0.66 (0.08, 4.96)	0.16 (0.00, 2.48)	0.19 (0.01, 1.95)	4.97 (0.33, 193.56)	0.74 (0.20, 2.67)	**0.09 (0.00, 0.68)**	AND300mgSC	0.30 (0.03, 2.27)	0.47 (0.06, 3.30)	0.30 (0.02, 2.94)	0.22 (0.02, 1.95)	1.05 (0.07, 13.09)	2.89 (0.28, 35.02)	3.29 (0.31, 39.89)	0.50 (0.07, 2.84)	0.83 (0.10, 5.89)	0.40 (0.01, 7.96)	1.28 (0.16, 8.43)	2.01 (0.17, 25.18)
API100mgPR	2.36 (0.40, 14.83)	2.27 (0.38, 14.94)	2.11 (0.37, 13.84)	2.22 (0.29, 15.93)	0.55 (0.01, 7.80)	0.67 (0.05, 6.11)	**17.16 (1.38, 662.15)**	2.51 (0.31, 23.67)	0.29 (0.01, 4.68)	3.38 (0.44, 31.49)	API100mgPR	1.59 (0.35, 7.64)	0.99 (0.08, 11.01)	0.73 (0.08, 6.96)	3.51 (0.30, 47.86)	**9.77 (1.13, 124.75)**	**11.10 (1.35, 135.49)**	1.63 (0.29, 9.97)	2.80 (0.42, 19.22)	1.31 (0.05, 25.47)	4.26 (0.68, 28.81)	6.55 (0.69, 83.04)
API50mgPO	1.51 (0.28, 8.17)	1.44 (0.26, 8.15)	1.35 (0.26, 7.34)	1.38 (0.21, 9.04)	0.34 (0.01, 4.63)	0.42 (0.03, 3.56)	10.50 (0.85, 369.65)	1.58 (0.21, 12.85)	0.18 (0.00, 2.68)	2.13 (0.30, 17.15)	0.63 (0.13, 2.82)	API50mgPO	0.63 (0.04, 6.08)	0.46 (0.05, 3.96)	2.21 (0.18, 28.27)	6.20 (0.72, 71.46)	6.80 (0.87, 76.80)	1.04 (0.20, 5.37)	1.76 (0.27, 10.35)	0.83 (0.03, 15.05)	2.75 (0.45, 15.74)	4.17 (0.45, 49.79)
BRI400mgIV	2.35 (0.30, 28.54)	2.25 (0.27, 28.00)	2.10 (0.27, 25.48)	2.23 (0.21, 32.95)	0.52 (0.01, 15.08)	0.68 (0.03, 11.15)	**17.09 (1.04, 812.20)**	2.51 (0.22, 43.70)	0.28 (0.01, 6.88)	3.38 (0.34, 55.56)	1.01 (0.09, 12.65)	1.59 (0.16, 23.66)	BRI400mgIV	0.72 (0.11, 7.25)	3.53 (0.22, 82.04)	9.93 (0.89, 186.91)	11.06 (0.99, 206.06)	1.62 (0.21, 18.70)	2.80 (0.30, 34.01)	1.33 (0.04, 45.74)	4.26 (0.49, 54.82)	6.69 (0.52, 117.97)
BRI700mgIV	3.24 (0.49, 23.42)	3.14 (0.46, 23.03)	2.89 (0.44, 20.55)	3.00 (0.35, 26.04)	0.73 (0.01, 12.55)	0.92 (0.06, 9.86)	**23.37 (1.59, 875.95)**	3.39 (0.35, 38.01)	0.38 (0.01, 7.78)	4.50 (0.51, 46.63)	1.37 (0.14, 12.00)	2.17 (0.25, 18.67)	1.40 (0.14, 8.91)	BRI700mgIV	4.80 (0.34, 71.13)	**13.47 (1.33, 169.82)**	**15.38 (1.47, 195.41)**	2.24 (0.33, 16.06)	3.80 (0.50, 29.67)	1.80 (0.06, 38.63)	5.94 (0.80, 44.30)	9.01 (0.75, 132.17)
BRO210mgIV	0.70 (0.07, 6.65)	0.68 (0.06, 6.67)	0.63 (0.06, 6.43)	0.65 (0.05, 7.15)	0.16 (0.00, 3.16)	0.19 (0.01, 2.61)	5.00 (0.24, 219.28)	0.73 (0.05, 10.32)	0.08 (0.00, 2.12)	0.95 (0.08, 13.98)	0.28 (0.02, 3.34)	0.45 (0.04, 5.46)	0.28 (0.01, 4.59)	0.21 (0.01, 2.92)	BRO210mgIV	2.80 (0.66, 16.12)	3.12 (0.78, 17.67)	0.47 (0.04, 4.77)	0.81 (0.07, 9.12)	0.37 (0.01, 10.11)	1.25 (0.10, 13.94)	1.89 (0.12, 34.33)
BRO350mgIV	0.25 (0.03, 1.69)	0.24 (0.02, 1.68)	0.22 (0.02, 1.51)	0.23 (0.02, 1.81)	**0.05 (0.00, 0.87)**	**0.07 (0.00, 0.71)**	1.69 (0.10, 67.17)	0.26 (0.02, 2.75)	**0.03 (0.00, 0.59)**	0.35 (0.03, 3.60)	**0.10 (0.01, 0.89)**	0.16 (0.01, 1.39)	0.10 (0.01, 1.12)	**0.07 (0.01, 0.75)**	0.36 (0.06, 1.53)	BRO350mgIV	1.12 (0.35, 3.79)	0.17 (0.02, 1.16)	0.29 (0.03, 2.20)	0.13 (0.00, 2.94)	0.45 (0.04, 3.06)	0.67 (0.05, 10.07)
BRO700mgIV	0.22 (0.02, 1.46)	0.21 (0.02, 1.42)	0.20 (0.02, 1.33)	0.20 (0.02, 1.61)	**0.05 (0.00, 0.78)**	**0.06 (0.00, 0.63)**	1.53 (0.08, 53.53)	0.23 (0.02, 2.49)	**0.02 (0.00, 0.50)**	0.30 (0.03, 3.21)	**0.09 (0.01, 0.74)**	0.15 (0.01, 1.15)	0.09 (0.00, 1.01)	**0.07 (0.01, 0.68)**	0.32 (0.06, 1.29)	0.89 (0.26, 2.86)	BRO700mgIV	**0.15 (0.02, 0.99)**	0.26 (0.02, 1.96)	0.12 (0.00, 2.50)	0.40 (0.04, 2.76)	0.59 (0.04, 9.01)
CDP10mg_kgIV	1.45 (0.35, 5.94)	1.39 (0.32, 6.16)	1.30 (0.31, 5.33)	1.35 (0.24, 7.05)	0.34 (0.01, 3.73)	0.41 (0.04, 2.69)	9.96 (1.00, 316.15)	1.50 (0.24, 10.32)	0.17 (0.01, 2.31)	2.00 (0.35, 13.87)	0.61 (0.10, 3.45)	0.96 (0.19, 5.09)	0.62 (0.05, 4.80)	0.45 (0.06, 3.00)	2.12 (0.21, 22.30)	5.88 (0.86, 54.48)	**6.61 (1.01, 59.50)**	CDP10mg_kgIV	1.70 (0.35, 7.66)	0.81 (0.03, 12.31)	2.62 (0.56, 11.48)	3.99 (0.54, 40.38)
CER100mgSC	0.85 (0.18, 4.06)	0.82 (0.17, 4.14)	0.75 (0.16, 3.94)	0.78 (0.13, 4.85)	0.19 (0.00, 2.50)	0.24 (0.02, 1.92)	5.95 (0.52, 207.02)	0.89 (0.12, 7.42)	0.10 (0.00, 1.43)	1.21 (0.17, 9.75)	0.36 (0.05, 2.40)	0.57 (0.10, 3.64)	0.36 (0.03, 3.32)	0.26 (0.03, 1.98)	1.23 (0.11, 14.81)	3.46 (0.45, 37.27)	3.89 (0.51, 43.08)	0.59 (0.13, 2.89)	CER100mgSC	0.47 (0.02, 8.22)	1.53 (0.45, 5.57)	2.30 (0.29, 26.72)
CER10mgIV	1.81 (0.11, 42.70)	1.71 (0.11, 42.63)	1.59 (0.10, 39.70)	1.66 (0.08, 43.95)	0.41 (0.00, 15.82)	0.49 (0.02, 15.64)	13.26 (0.43, 1134.91)	1.90 (0.10, 56.61)	0.21 (0.00, 9.72)	2.53 (0.13, 71.44)	0.76 (0.04, 20.85)	1.20 (0.07, 32.47)	0.75 (0.02, 26.56)	0.55 (0.03, 16.93)	2.72 (0.10, 104.57)	7.59 (0.34, 262.95)	8.68 (0.40, 304.82)	1.24 (0.08, 29.95)	2.14 (0.12, 54.00)	CER10mgIV	3.17 (0.20, 78.30)	4.94 (0.56, 91.56)

CER200mg SC	0.54 (0.13, 2.48)	0.53 (0.11, 2.62)	0.49 (0.11, 2.38)	0.51 (0.09, 3.11)	0.13 (0.00, 1.62)	0.15 (0.01, 1.18)	3.86 (0.35, 129.34)	0.57 (0.09, 4.46)	**0.07 (0.00, 0.98)**	0.78 (0.12, 6.09)	0.23 (0.03, 1.47)	0.36 (0.06, 2.25)	0.23 (0.02, 2.03)	0.17 (0.02, 1.25)	0.80 (0.07, 10.28)	2.21 (0.33, 24.69)	2.51 (0.36, 28.30)	0.38 (0.09, 1.79)	0.66 (0.18, 2.24)	0.32 (0.01, 5.12)	CER200mg SC	1.50 (0.20, 16.37)
CER20mgIV	0.36 (0.04, 2.69)	0.35 (0.03, 2.86)	0.33 (0.03, 2.37)	0.34 (0.03, 2.99)	0.08 (0.00, 1.38)	0.10 (0.01, 1.16)	2.46 (0.13, 109.63)	0.37 (0.03, 4.30)	**0.04 (0.00, 0.95)**	0.50 (0.04, 5.82)	0.15 (0.01, 1.45)	0.24 (0.02, 2.22)	0.15 (0.01, 1.94)	0.11 (0.01, 1.33)	0.53 (0.03, 8.07)	1.50 (0.10, 20.86)	1.70 (0.11, 23.24)	0.25 (0.02, 1.85)	0.43 (0.04, 3.44)	0.20 (0.01, 1.78)	0.67 (0.06, 4.98)	CER20mgIV

(**TABLE S18M.** continued)

	ABA10mg_kgIV	ABA30mg_kgIV	ABA3mg_kgIV	ADA160mg80mgSC	ADA40mg20mgSC	ADA80mg40mgSC	AMI0_4mgPO	AND150mgSC1_1	AND150mgSC2_2	AND300mgSC	API100mgPR	API50mgPO	BRI400mgIV	BRI700mgIV	BRO210mgIV	BRO350mgIV	BRO700mgIV	CDP10mg_kgIV	CER100mgSC	CER10mgIV	CER200mgSC	CER20mgIV
CER400mgSC	0.82 (0.25, 3.14)	0.80 (0.21, 3.26)	0.74 (0.21, 2.82)	0.77 (0.16, 3.83)	0.19 (0.00, 2.06)	0.24 (0.02, 1.55)	5.64 (0.63, 176.24)	0.87 (0.16, 5.64)	0.10 (0.00, 1.22)	1.17 (0.22, 7.69)	0.35 (0.06, 1.83)	0.55 (0.12, 2.67)	0.35 (0.04, 2.70)	0.25 (0.04, 1.62)	1.20 (0.13, 12.23)	3.29 (0.59, 30.68)	3.80 (0.65, 34.37)	0.57 (0.17, 2.20)	0.99 (0.29, 3.28)	0.47 (0.02, 6.89)	1.51 (0.48, 4.76)	2.27 (0.35, 21.89)
CER5mgIV	2.72 (0.18, 101.31)	2.60 (0.17, 97.13)	2.40 (0.17, 85.22)	2.44 (0.14, 97.94)	0.57 (0.01, 38.30)	0.73 (0.03, 33.76)	20.49 (0.72, 1889.02)	2.87 (0.15, 119.17)	0.32 (0.00, 19.07)	3.90 (0.21, 157.79)	1.15 (0.06, 45.19)	1.81 (0.11, 75.50)	1.16 (0.04, 53.25)	0.82 (0.04, 35.02)	4.10 (0.16, 224.54)	11.65 (0.56, 570.73)	13.24 (0.61, 609.98)	1.87 (0.13, 63.29)	3.20 (0.21, 125.78)	1.50 (0.07, 57.50)	4.92 (0.31, 181.12)	7.43 (0.94, 219.76)
ELD10mg_kgIV	0.68 (0.06, 6.67)	0.66 (0.05, 6.65)	0.61 (0.05, 5.90)	0.64 (0.05, 6.89)	0.15 (0.00, 3.43)	0.19 (0.01, 2.60)	4.73 (0.24, 219.78)	0.71 (0.05, 10.22)	0.08 (0.00, 1.97)	0.97 (0.06, 14.15)	0.29 (0.02, 3.29)	0.45 (0.04, 4.94)	0.28 (0.01, 4.38)	0.21 (0.01, 2.95)	0.99 (0.05, 17.92)	2.83 (0.18, 47.26)	3.18 (0.22, 53.33)	0.47 (0.04, 4.31)	0.80 (0.07, 7.79)	0.38 (0.01, 9.71)	1.25 (0.10, 12.06)	1.88 (0.12, 33.03)
ELD20mg_kgIV	0.51 (0.05, 4.49)	0.49 (0.04, 4.53)	0.46 (0.04, 4.14)	0.47 (0.04, 4.86)	0.11 (0.00, 2.33)	0.14 (0.01, 1.81)	3.58 (0.18, 154.91)	0.52 (0.04, 6.41)	0.06 (0.00, 1.31)	0.71 (0.05, 8.73)	0.21 (0.02, 2.38)	0.33 (0.03, 3.61)	0.21 (0.01, 3.16)	0.16 (0.01, 2.05)	0.73 (0.04, 12.31)	2.10 (0.14, 31.68)	2.35 (0.17, 34.43)	0.36 (0.03, 2.97)	0.61 (0.05, 5.62)	0.28 (0.01, 6.60)	0.94 (0.08, 8.59)	1.42 (0.09, 21.44)
FON0_1mg_kgIV	0.81 (0.02, 40.84)	0.78 (0.02, 40.02)	0.71 (0.02, 37.09)	0.74 (0.02, 43.82)	0.18 (0.00, 15.18)	0.22 (0.00, 14.91)	6.20 (0.09, 919.38)	0.85 (0.02, 49.56)	0.10 (0.00, 9.01)	1.13 (0.02, 73.46)	0.34 (0.01, 19.48)	0.53 (0.01, 29.88)	0.33 (0.01, 22.34)	0.25 (0.01, 14.29)	1.17 (0.02, 87.15)	3.43 (0.06, 215.89)	3.80 (0.07, 250.91)	0.56 (0.01, 28.86)	0.94 (0.02, 49.71)	0.45 (0.00, 44.13)	1.46 (0.04, 75.38)	2.26 (0.04, 158.04)
FON1mg_kgIV	0.54 (0.01, 7.97)	0.52 (0.01, 7.88)	0.49 (0.01, 7.19)	0.49 (0.01, 8.50)	0.12 (0.00, 3.55)	0.14 (0.00, 3.39)	3.82 (0.07, 230.32)	0.55 (0.01, 10.76)	0.06 (0.00, 2.14)	0.76 (0.02, 14.14)	0.22 (0.01, 4.49)	0.34 (0.01, 6.67)	0.21 (0.00, 5.20)	0.16 (0.00, 3.39)	0.76 (0.01, 20.87)	2.24 (0.05, 55.97)	2.53 (0.06, 62.93)	0.37 (0.01, 5.70)	0.64 (0.02, 9.99)	0.27 (0.00, 10.37)	0.97 (0.03, 15.43)	1.48 (0.03, 37.96)
FON4mg_kgIV	0.57 (0.02, 9.04)	0.54 (0.01, 8.95)	0.51 (0.01, 8.04)	0.53 (0.01, 9.20)	0.12 (0.00, 4.03)	0.15 (0.00, 3.69)	3.98 (0.07, 272.13)	0.59 (0.02, 12.90)	0.06 (0.00, 2.29)	0.76 (0.02, 17.37)	0.23 (0.01, 4.72)	0.37 (0.01, 7.07)	0.23 (0.00, 5.49)	0.17 (0.00, 3.70)	0.80 (0.02, 24.28)	2.31 (0.05, 59.30)	2.61 (0.06, 67.87)	0.39 (0.01, 6.41)	0.66 (0.02, 12.44)	0.30 (0.00, 14.38)	1.03 (0.03, 18.37)	1.51 (0.03, 48.64)
NAT300mgIV	1.41 (0.41, 5.06)	1.36 (0.38, 5.10)	1.27 (0.36, 4.38)	1.31 (0.28, 6.12)	0.33 (0.01, 3.28)	0.40 (0.04, 2.45)	**9.68 (1.12, 305.04)**	1.47 (0.26, 9.63)	0.17 (0.01, 2.05)	1.97 (0.37, 12.91)	0.60 (0.11, 3.01)	0.94 (0.21, 4.31)	0.60 (0.06, 4.29)	0.44 (0.07, 2.57)	2.07 (0.23, 20.39)	5.67 (0.99, 50.09)	**6.45 (1.14, 56.63)**	0.98 (0.28, 3.46)	1.68 (0.39, 6.73)	0.79 (0.04, 10.86)	2.60 (0.64, 9.56)	3.88 (0.60, 36.05)
NAT3mg_kgIV	1.18 (0.22, 6.22)	1.13 (0.20, 6.43)	1.06 (0.20, 5.80)	1.10 (0.14, 7.27)	0.27 (0.00, 3.71)	0.33 (0.02, 2.89)	7.96 (0.69, 297.82)	1.21 (0.15, 10.62)	0.14 (0.00, 2.16)	1.64 (0.22, 14.01)	0.50 (0.06, 3.57)	0.79 (0.12, 5.22)	0.50 (0.04, 4.81)	0.36 (0.04, 3.11)	1.69 (0.15, 21.72)	4.81 (0.62, 54.50)	5.32 (0.70, 62.06)	0.82 (0.15, 4.40)	1.38 (0.22, 8.64)	0.66 (0.02, 12.85)	2.14 (0.36, 12.46)	3.23 (0.36, 39.27)
NAT3mg_kgIVx2	1.34 (0.25, 7.72)	1.30 (0.23, 7.41)	1.20 (0.22, 7.07)	1.28 (0.18, 8.83)	0.32 (0.01, 4.31)	0.38 (0.03, 3.55)	9.29 (0.83, 365.14)	1.41 (0.18, 12.09)	0.16 (0.00, 2.46)	1.93 (0.27, 17.06)	0.57 (0.08, 4.40)	0.89 (0.13, 6.26)	0.57 (0.04, 5.38)	0.41 (0.05, 3.48)	1.95 (0.18, 25.70)	5.58 (0.68, 60.57)	6.33 (0.76, 67.65)	0.95 (0.18, 5.03)	1.63 (0.25, 9.73)	0.76 (0.03, 13.98)	2.48 (0.41, 14.66)	3.82 (0.43, 46.53)
NAT6mg_kgIVx2	1.07 (0.20, 6.25)	1.03 (0.19, 6.01)	0.95 (0.17, 5.59)	1.00 (0.13, 7.33)	0.25 (0.00, 3.57)	0.30 (0.02, 2.86)	7.61 (0.63, 270.58)	1.13 (0.14, 10.28)	0.13 (0.00, 2.10)	1.52 (0.19, 13.42)	0.46 (0.06, 3.51)	0.72 (0.10, 5.20)	0.45 (0.03, 4.34)	0.33 (0.04, 2.82)	1.57 (0.14, 20.20)	4.45 (0.52, 49.60)	4.98 (0.61, 56.08)	0.75 (0.14, 4.24)	1.28 (0.20, 8.04)	0.60 (0.02, 11.24)	1.98 (0.31, 11.39)	3.03 (0.33, 35.67)
PBO	1.10 (0.41, 3.04)	1.06 (0.36, 3.12)	0.98 (0.35, 2.72)	1.02 (0.26, 3.99)	0.26 (0.01, 2.37)	0.31 (0.03, 1.67)	7.42 (0.96, 211.10)	1.15 (0.25, 6.32)	0.13 (0.00, 1.43)	1.54 (0.36, 8.56)	0.47 (0.10, 1.91)	0.73 (0.19, 2.83)	0.47 (0.05, 2.89)	0.34 (0.06, 1.75)	1.59 (0.20, 13.50)	4.40 (0.91, 34.84)	**4.99 (1.01, 37.12)**	0.76 (0.28, 2.11)	1.31 (0.37, 4.16)	0.62 (0.03, 7.80)	2.01 (0.61, 6.04)	2.98 (0.56, 24.80)
RIS1200mgIV	**3.91 (1.15, 13.88)**	3.75 (0.99, 14.09)	3.51 (0.98, 12.29)	3.64 (0.75, 16.45)	0.90 (0.02, 9.29)	1.09 (0.11, 6.85)	**26.19 (3.01, 865.91)**	4.02 (0.74, 25.89)	0.47 (0.02, 5.60)	**5.44 (1.07, 33.80)**	1.66 (0.30, 8.14)	2.62 (0.55, 12.34)	1.67 (0.16, 11.95)	1.21 (0.19, 7.30)	5.62 (0.62, 54.83)	**15.84 (2.62, 139.81)**	**17.77 (2.97, 152.02)**	2.70 (0.77, 9.39)	**4.59 (1.06, 19.38)**	2.20 (0.10, 32.51)	**7.14 (1.77, 27.54)**	**10.78 (1.68, 99.77)**
RIS600mgIV	2.46 (0.76, 8.91)	2.40 (0.67, 9.01)	2.21 (0.66, 7.98)	2.31 (0.51, 11.04)	0.58 (0.01, 6.00)	0.71 (0.07, 4.31)	**17.15 (1.90, 503.60)**	2.57 (0.50, 16.22)	0.30 (0.01, 3.60)	3.52 (0.71, 22.07)	1.06 (0.20, 5.40)	1.67 (0.37, 8.27)	1.07 (0.10, 7.65)	0.76 (0.13, 4.79)	3.61 (0.42, 35.65)	**10.09 (1.74, 84.69)**	**11.40 (2.01, 99.06)**	1.72 (0.52, 6.20)	3.00 (0.69, 12.21)	1.41 (0.06, 18.96)	**4.51 (1.15, 16.84)**	**6.89 (1.08, 65.90)**
UST1mg_kgIV	2.06 (0.43, 10.58)	1.99 (0.39, 11.01)	1.87 (0.38, 9.55)	1.94 (0.30, 12.79)	0.47 (0.01, 6.36)	0.59 (0.05, 5.23)	**14.61 (1.25, 484.22)**	2.17 (0.30, 19.15)	0.24 (0.01, 3.93)	2.89 (0.43, 24.67)	0.88 (0.12, 6.01)	1.38 (0.21, 8.90)	0.89 (0.07, 8.06)	0.64 (0.08, 5.11)	3.01 (0.26, 37.07)	**8.46 (1.15, 91.42)**	**9.58 (1.21, 100.72)**	1.44 (0.30, 7.42)	2.47 (0.40, 14.37)	1.16 (0.05, 21.02)	3.79 (0.66, 20.55)	5.79 (0.70, 67.82)
UST3mg_kgIV	1.55 (0.34, 7.59)	1.48 (0.31, 7.66)	1.39 (0.31, 6.89)	1.42 (0.25, 8.26)	0.36 (0.01, 4.51)	0.43 (0.04, 3.47)	**10.59 (1.02, 363.85)**	1.59 (0.24, 13.89)	0.18 (0.01, 2.87)	2.13 (0.35, 18.47)	0.66 (0.10, 4.28)	1.03 (0.18, 6.68)	0.66 (0.05, 5.77)	0.47 (0.06, 3.49)	2.25 (0.22, 26.30)	6.25 (0.86, 66.01)	**7.03 (1.02, 78.65)**	1.07 (0.23, 5.29)	1.86 (0.32, 9.69)	0.85 (0.03, 15.19)	2.84 (0.53, 14.28)	4.17 (0.55, 48.00)
UST4_5mg_kgIV	0.45 (0.01, 6.97)	0.42 (0.01, 6.78)	0.39 (0.01, 6.26)	0.40 (0.01, 7.78)	0.09 (0.00, 3.17)	0.12 (0.00, 2.78)	3.17 (0.06, 176.35)	0.47 (0.01, 9.41)	0.05 (0.00, 1.71)	0.61 (0.02, 12.97)	0.18 (0.01, 3.51)	0.29 (0.01, 5.35)	0.18 (0.00, 4.30)	0.13 (0.00, 2.78)	0.63 (0.02, 16.91)	1.82 (0.05, 46.64)	2.05 (0.05, 49.84)	0.30 (0.01, 4.89)	0.51 (0.01, 8.53)	0.23 (0.00, 8.95)	0.81 (0.02, 14.08)	1.21 (0.03, 31.16)
UST6mg_kgIV	1.35 (0.30, 6.10)	1.29 (0.25, 6.43)	1.20 (0.25, 5.60)	1.27 (0.21, 6.85)	0.31 (0.01, 3.91)	0.37 (0.03, 2.89)	9.60 (0.87, 341.64)	1.41 (0.20, 11.45)	0.16 (0.00, 2.39)	1.91 (0.29, 14.59)	0.57 (0.08, 3.47)	0.90 (0.15, 5.10)	0.57 (0.05, 5.06)	0.41 (0.05, 3.03)	1.95 (0.18, 22.41)	5.51 (0.73, 57.94)	6.23 (0.82, 65.39)	0.93 (0.20, 4.41)	1.62 (0.28, 7.91)	0.74 (0.03, 12.92)	2.46 (0.46, 12.21)	3.70 (0.46, 42.51)
UST90mgSC	2.59 (0.15, 103.36)	2.48 (0.15, 98.32)	2.33 (0.14, 91.40)	2.46 (0.12, 93.65)	0.59 (0.01, 30.93)	0.70 (0.02, 33.36)	19.98 (0.65, 1975.02)	2.75 (0.12, 144.15)	0.31 (0.00, 22.67)	3.69 (0.16, 188.69)	1.10 (0.04, 50.21)	1.74 (0.08, 72.63)	1.11 (0.03, 52.46)	0.81 (0.03, 37.80)	3.77 (0.14, 228.38)	11.05 (0.46, 574.71)	12.57 (0.47, 670.50)	1.78 (0.09, 72.62)	3.08 (0.14, 119.13)	1.42 (0.03, 118.41)	4.63 (0.26, 183.35)	7.25 (0.26, 415.74)
VED0_5mg_kgIV	2.02 (0.41, 10.11)	1.93 (0.38, 10.61)	1.80 (0.37, 9.54)	1.88 (0.29, 12.73)	0.46 (0.01, 5.93)	0.56 (0.04, 4.81)	**14.15 (1.21, 486.80)**	2.11 (0.29, 18.13)	0.24 (0.01, 3.79)	2.91 (0.42, 23.07)	0.86 (0.12, 6.06)	1.36 (0.21, 9.33)	0.85 (0.07, 8.30)	0.62 (0.08, 4.96)	3.01 (0.27, 34.07)	**8.30 (1.06, 90.62)**	**9.36 (1.19, 98.20)**	1.40 (0.29, 7.06)	2.39 (0.43, 13.65)	1.13 (0.04, 21.57)	3.66 (0.66, 20.07)	5.57 (0.64, 67.28)
VED2mg_kgIV	1.21 (0.27, 5.81)	1.18 (0.24, 5.71)	1.09 (0.23, 5.57)	1.15 (0.18, 6.91)	0.29 (0.01, 3.49)	0.34 (0.03, 2.65)	8.45 (0.79, 285.87)	1.28 (0.19, 9.92)	0.15 (0.00, 2.12)	1.74 (0.27, 13.20)	0.51 (0.08, 3.28)	0.82 (0.13, 5.03)	0.52 (0.04, 4.57)	0.38 (0.05, 2.74)	1.78 (0.17, 20.89)	5.01 (0.67, 53.22)	5.59 (0.78, 58.29)	0.84 (0.18, 4.17)	1.47 (0.27, 7.46)	0.68 (0.03, 12.15)	2.23 (0.40, 11.20)	3.35 (0.42, 37.43)
VED300mgIV	1.39 (0.30, 6.84)	1.34 (0.27, 7.09)	1.24 (0.26, 6.14)	1.31 (0.20, 7.82)	0.32 (0.01, 3.92)	0.39 (0.03, 3.06)	9.55 (0.88, 324.81)	1.48 (0.20, 11.07)	0.17 (0.01, 2.51)	2.01 (0.29, 15.02)	0.59 (0.08, 3.89)	0.93 (0.15, 5.75)	0.59 (0.05, 5.55)	0.43 (0.06, 3.31)	2.04 (0.19, 24.07)	5.66 (0.79, 57.37)	6.43 (0.88, 67.84)	0.97 (0.20, 4.70)	1.65 (0.29, 9.08)	0.78 (0.03, 13.81)	2.55 (0.47, 13.42)	3.82 (0.48, 42.40)

	CER400mgSC	CER5mgIV	ELD10mg_kgIV	ELD20mg_kgIV	FON0_1mg_kgIV	FON1mg_kgIV	FON4mg_kgIV	NAT300mgIV	NAT3mg_kgIV	NAT3mg_kgIVx2	NAT6mg_kgIVx2	PBO	RIS1200mgIV	RIS600mgIV	UST1mg_kgIV	UST3mg_kgIV	UST4_5mg_kgIV	UST6mg_kgIV	UST90mgSC	VED0_5mg_kgIV	VED2mg_kgIV	VED300mgIV
ABA10mg_kgIV	1.22 (0.32, 4.06)	0.37 (0.01, 5.47)	1.48 (0.15, 16.93)	1.96 (0.22, 20.91)	1.24 (0.02, 48.32)	1.85 (0.13, 68.81)	1.76 (0.11, 64.91)	0.71 (0.20, 2.43)	0.85 (0.16, 4.47)	0.75 (0.13, 4.03)	0.93 (0.16, 4.98)	0.91 (0.33, 2.44)	**0.26 (0.07, 0.87)**	0.41 (0.11, 1.31)	0.49 (0.09, 2.34)	0.64 (0.13, 2.95)	2.24 (0.14, 75.90)	0.74 (0.16, 3.38)	0.39 (0.01, 6.47)	0.50 (0.10, 2.43)	0.82 (0.17, 3.75)	0.72 (0.15, 3.39)
ABA30mg_kgIV	1.25 (0.31, 4.80)	0.38 (0.01, 5.87)	1.52 (0.15, 19.28)	2.02 (0.22, 23.52)	1.28 (0.02, 51.02)	1.94 (0.13, 74.21)	1.86 (0.11, 73.19)	0.73 (0.20, 2.65)	0.89 (0.16, 4.90)	0.77 (0.13, 4.35)	0.97 (0.17, 5.38)	0.94 (0.32, 2.82)	0.27 (0.07, 1.01)	0.42 (0.11, 1.50)	0.50 (0.09, 2.59)	0.68 (0.13, 3.20)	2.39 (0.15, 71.94)	0.78 (0.16, 4.06)	0.40 (0.01, 6.86)	0.52 (0.09, 2.66)	0.85 (0.18, 4.22)	0.74 (0.14, 3.67)
ABA3mg_kgIV	1.36 (0.35, 4.66)	0.42 (0.01, 5.94)	1.63 (0.17, 18.45)	2.16 (0.24, 24.32)	1.41 (0.03, 57.76)	2.05 (0.14, 77.23)	1.95 (0.12, 74.90)	0.79 (0.23, 2.79)	0.94 (0.17, 5.02)	0.83 (0.14, 4.54)	1.05 (0.18, 5.89)	1.02 (0.37, 2.82)	0.28 (0.08, 1.02)	0.45 (0.13, 1.51)	0.53 (0.10, 2.63)	0.72 (0.15, 3.24)	2.54 (0.16, 81.39)	0.83 (0.18, 3.95)	0.43 (0.01, 7.04)	0.55 (0.10, 2.72)	0.92 (0.18, 4.33)	0.81 (0.16, 3.84)
ADA160mg80mgSC	1.30 (0.26, 6.24)	0.41 (0.01, 7.12)	1.56 (0.15, 20.50)	2.14 (0.21, 27.10)	1.35 (0.02, 61.34)	2.02 (0.12, 93.91)	1.87 (0.11, 79.53)	0.76 (0.16, 3.53)	0.91 (0.14, 6.92)	0.78 (0.11, 5.69)	1.00 (0.14, 7.45)	0.98 (0.25, 3.83)	0.27 (0.06, 1.34)	0.43 (0.09, 1.96)	0.52 (0.08, 3.30)	0.70 (0.12, 3.96)	2.48 (0.13, 92.24)	0.79 (0.15, 4.72)	0.41 (0.01, 8.51)	0.53 (0.08, 3.42)	0.87 (0.14, 5.68)	0.76 (0.13, 4.92)
ADA40mg20mgSC	5.29 (0.49, 239.99)	1.74 (0.03, 137.81)	6.79 (0.29, 487.28)	8.93 (0.43, 628.65)	5.53 (0.07, 1388.31)	8.64 (0.28, 1854.85)	8.36 (0.25, 1590.98)	3.05 (0.30, 135.52)	3.72 (0.27, 212.05)	3.13 (0.23, 165.99)	4.07 (0.28, 211.35)	3.86 (0.42, 168.20)	1.11 (0.11, 50.69)	1.72 (0.17, 80.47)	2.12 (0.16, 101.40)	2.78 (0.22, 139.40)	10.92 (0.32, 1375.47)	3.26 (0.26, 164.09)	1.71 (0.03, 150.25)	2.19 (0.17, 131.35)	3.49 (0.29, 188.73)	3.13 (0.26, 179.55)
ADA80mg40mgSC	4.22 (0.65, 43.30)	1.37 (0.03, 34.43)	5.31 (0.38, 117.88)	7.31 (0.55, 148.26)	4.49 (0.07, 265.12)	7.17 (0.29, 407.64)	6.60 (0.27, 360.05)	2.51 (0.41, 25.29)	3.05 (0.35, 40.00)	2.62 (0.28, 33.59)	3.37 (0.35, 43.26)	3.20 (0.60, 29.52)	0.92 (0.15, 9.44)	1.42 (0.23, 14.18)	1.70 (0.19, 22.11)	2.31 (0.29, 28.20)	8.66 (0.36, 392.17)	2.68 (0.35, 32.36)	1.43 (0.03, 42.77)	1.78 (0.21, 23.01)	2.94 (0.38, 37.71)	2.53 (0.33, 29.82)
AMI0_4mgPO	0.18 (0.01, 1.59)	0.05 (0.00, 1.38)	0.21 (0.00, 4.21)	0.28 (0.01, 5.43)	0.16 (0.00, 10.98)	0.26 (0.00, 14.80)	0.25 (0.00, 13.84)	**0.10 (0.00, 0.89)**	0.13 (0.00, 1.44)	0.11 (0.00, 1.21)	0.13 (0.00, 1.58)	0.13 (0.00, 1.04)	**0.04 (0.00, 0.33)**	**0.06 (0.00, 0.53)**	**0.07 (0.00, 0.80)**	**0.09 (0.00, 0.98)**	0.32 (0.01, 16.18)	0.10 (0.00, 1.15)	0.05 (0.00, 1.53)	**0.07 (0.00, 0.82)**	0.12 (0.00, 1.26)	0.10 (0.00, 1.14)
AND150mgSC1_1	1.15 (0.18, 6.34)	0.35 (0.01, 6.69)	1.41 (0.10, 20.85)	1.92 (0.16, 26.47)	1.18 (0.02, 53.95)	1.81 (0.09, 79.97)	1.71 (0.08, 61.91)	0.68 (0.10, 3.78)	0.83 (0.09, 6.49)	0.71 (0.08, 5.42)	0.88 (0.10, 7.17)	0.87 (0.16, 3.99)	0.25 (0.04, 1.35)	0.39 (0.06, 2.01)	0.46 (0.05, 3.33)	0.63 (0.07, 4.20)	2.14 (0.11, 76.87)	0.71 (0.09, 5.02)	0.36 (0.01, 8.09)	0.47 (0.06, 3.44)	0.78 (0.10, 5.40)	0.67 (0.09, 5.03)
AND150mgSC2_2	10.03 (0.82, 303.48)	3.16 (0.05, 203.53)	13.18 (0.51, 542.88)	17.45 (0.76, 725.67)	10.42 (0.11, 1408.93)	16.79 (0.47, 2064.65)	16.48 (0.44, 1441.95)	5.88 (0.49, 168.18)	7.12 (0.46, 228.22)	6.28 (0.41, 209.66)	7.93 (0.48, 277.89)	7.47 (0.70, 202.84)	2.13 (0.18, 60.80)	3.31 (0.28, 98.80)	4.10 (0.25, 131.69)	5.57 (0.35, 178.63)	20.63 (0.58, 1689.43)	6.24 (0.42, 203.07)	3.19 (0.04, 209.36)	4.23 (0.26, 153.17)	6.82 (0.47, 228.98)	6.00 (0.40, 192.02)
AND300mgSC	0.86 (0.13, 4.54)	0.26 (0.01, 4.69)	1.03 (0.07, 15.44)	1.40 (0.11, 19.12)	0.88 (0.01, 41.37)	1.32 (0.07, 58.29)	1.31 (0.06, 49.12)	0.51 (0.08, 2.70)	0.61 (0.07, 4.53)	0.52 (0.06, 3.72)	0.66 (0.07, 5.15)	0.65 (0.12, 2.81)	**0.18 (0.03, 0.94)**	0.28 (0.05, 1.41)	0.35 (0.04, 2.34)	0.47 (0.05, 2.89)	1.63 (0.08, 56.09)	0.52 (0.07, 3.48)	0.27 (0.01, 6.35)	0.34 (0.04, 2.36)	0.57 (0.08, 3.76)	0.50 (0.07, 3.47)
API100mgPR	2.86 (0.55, 16.15)	0.87 (0.02, 16.00)	3.50 (0.30, 49.21)	4.76 (0.42, 56.85)	2.94 (0.05, 128.20)	4.56 (0.22, 173.50)	4.34 (0.21, 170.92)	1.67 (0.33, 9.08)	2.00 (0.28, 15.42)	1.74 (0.23, 13.11)	2.19 (0.29, 17.03)	2.13 (0.52, 10.07)	0.60 (0.12, 3.36)	0.95 (0.19, 5.12)	1.13 (0.17, 8.37)	1.53 (0.23, 10.47)	5.50 (0.28, 195.31)	1.75 (0.29, 12.02)	0.91 (0.02, 22.52)	1.17 (0.17, 8.58)	1.94 (0.30, 13.29)	1.70 (0.26, 12.06)
API50mgPO	1.82 (0.37, 8.36)	0.55 (0.01, 9.32)	2.23 (0.20, 28.52)	2.99 (0.28, 35.07)	1.89 (0.03, 79.81)	2.94 (0.15, 107.48)	2.73 (0.14, 93.02)	1.06 (0.23, 4.67)	1.27 (0.19, 8.62)	1.12 (0.16, 7.52)	1.39 (0.19, 9.72)	1.36 (0.35, 5.18)	0.38 (0.08, 1.82)	0.60 (0.12, 2.67)	0.73 (0.11, 4.67)	0.97 (0.15, 5.66)	3.46 (0.19, 116.86)	1.11 (0.20, 6.74)	0.57 (0.01, 12.91)	0.74 (0.11, 4.87)	1.23 (0.20, 7.64)	1.08 (0.17, 6.53)
BRI400mgIV	2.83 (0.37, 28.33)	0.87 (0.02, 26.79)	3.52 (0.23, 97.64)	4.70 (0.32, 112.49)	3.06 (0.04, 172.09)	4.72 (0.19, 226.20)	4.40 (0.18, 204.83)	1.67 (0.23, 17.89)	2.01 (0.21, 27.51)	1.76 (0.19, 24.48)	2.20 (0.23, 31.93)	2.14 (0.35, 19.12)	0.60 (0.08, 6.44)	0.93 (0.13, 10.24)	1.13 (0.12, 15.31)	1.51 (0.17, 19.39)	5.61 (0.23, 230.67)	1.76 (0.20, 21.89)	0.90 (0.02, 31.27)	1.17 (0.12, 15.22)	1.93 (0.22, 24.46)	1.70 (0.18, 21.66)
BRI700mgIV	3.96 (0.62, 24.21)	1.23 (0.03, 25.56)	4.85 (0.34, 80.15)	6.36 (0.49, 95.30)	4.05 (0.07, 196.31)	6.18 (0.30, 256.91)	5.98 (0.27, 231.04)	2.28 (0.39, 14.28)	2.80 (0.32, 23.22)	2.41 (0.29, 20.77)	3.04 (0.35, 26.92)	2.98 (0.57, 15.59)	0.83 (0.14, 5.24)	1.32 (0.21, 7.99)	1.55 (0.20, 12.62)	2.11 (0.29, 15.50)	7.58 (0.36, 293.99)	2.45 (0.33, 18.37)	1.24 (0.03, 28.78)	1.61 (0.20, 13.04)	2.65 (0.36, 20.56)	2.31 (0.30, 17.99)
BRO210mgIV	0.83 (0.08, 7.58)	0.24 (0.00, 6.27)	1.01 (0.06, 19.98)	1.36 (0.08, 26.34)	0.85 (0.01, 50.49)	1.32 (0.05, 70.69)	1.24 (0.04, 63.02)	0.48 (0.05, 4.32)	0.59 (0.05, 6.56)	0.51 (0.04, 5.43)	0.64 (0.05, 7.22)	0.63 (0.07, 5.02)	0.18 (0.02, 1.63)	0.28 (0.03, 2.38)	0.33 (0.03, 3.82)	0.44 (0.04, 4.57)	1.58 (0.06, 64.61)	0.51 (0.04, 5.53)	0.26 (0.00, 7.37)	0.33 (0.03, 3.71)	0.56 (0.05, 5.88)	0.49 (0.04, 5.18)
BRO350mgIV	0.30 (0.03, 1.69)	0.09 (0.00, 1.78)	0.35 (0.02, 5.61)	0.48 (0.03, 6.92)	0.29 (0.00, 15.47)	0.45 (0.02, 19.29)	0.43 (0.02, 19.07)	0.18 (0.02, 1.01)	0.21 (0.02, 1.61)	0.18 (0.02, 1.46)	0.22 (0.02, 1.92)	0.23 (0.03, 1.09)	**0.06 (0.01, 0.38)**	**0.10 (0.01, 0.57)**	**0.12 (0.01, 0.87)**	0.16 (0.02, 1.16)	0.55 (0.02, 20.98)	0.18 (0.02, 1.37)	0.09 (0.00, 2.17)	**0.12 (0.01, 0.94)**	0.20 (0.02, 1.49)	0.18 (0.02, 1.27)
BRO700mgIV	0.26 (0.03, 1.54)	0.08 (0.00, 1.63)	0.31 (0.02, 4.54)	0.43 (0.03, 5.97)	0.26 (0.00, 13.53)	0.39 (0.02, 16.99)	0.38 (0.01, 16.47)	**0.16 (0.02, 0.87)**	0.19 (0.02, 1.43)	0.16 (0.01, 1.32)	0.20 (0.02, 1.63)	**0.20 (0.03, 0.99)**	**0.06 (0.01, 0.34)**	**0.09 (0.01, 0.50)**	**0.10 (0.01, 0.83)**	**0.14 (0.01, 0.98)**	0.49 (0.02, 18.56)	0.16 (0.02, 1.22)	0.08 (0.00, 2.12)	**0.11 (0.01, 0.84)**	0.18 (0.02, 1.28)	0.16 (0.01, 1.14)
CDP10mg_kgIV	1.74 (0.45, 5.93)	0.53 (0.02, 7.97)	2.12 (0.23, 24.60)	2.78 (0.34, 31.52)	1.79 (0.03, 70.55)	2.72 (0.18, 96.72)	2.54 (0.16, 96.33)	1.02 (0.29, 3.51)	1.22 (0.23, 6.66)	1.06 (0.20, 5.42)	1.33 (0.24, 7.36)	1.31 (0.47, 3.51)	0.37 (0.11, 1.31)	0.58 (0.16, 1.93)	0.70 (0.13, 3.35)	0.93 (0.19, 4.31)	3.32 (0.20, 102.85)	1.07 (0.23, 5.07)	0.56 (0.01, 11.07)	0.71 (0.14, 3.51)	1.19 (0.24, 5.59)	1.03 (0.21, 4.88)
CER100mgSC	1.01 (0.30, 3.43)	0.31 (0.01, 4.84)	1.25 (0.13, 15.03)	1.64 (0.18, 20.85)	1.06 (0.02, 45.56)	1.57 (0.10, 64.84)	1.53 (0.08, 60.89)	0.60 (0.15, 2.56)	0.72 (0.12, 4.45)	0.61 (0.10, 3.99)	0.78 (0.12, 5.12)	0.76 (0.24, 2.74)	**0.22 (0.05, 0.94)**	0.33 (0.08, 1.46)	0.40 (0.07, 2.50)	0.54 (0.10, 3.12)	1.96 (0.12, 67.38)	0.62 (0.13, 3.55)	0.32 (0.01, 6.96)	0.42 (0.07, 2.33)	0.68 (0.13, 3.77)	0.61 (0.11, 3.51)
CER10mgIV	2.14 (0.15, 46.51)	0.67 (0.02, 13.79)	2.62 (0.10, 111.75)	3.55 (0.15, 140.70)	2.24 (0.02, 232.96)	3.64 (0.10, 325.50)	3.36 (0.07, 315.79)	1.26 (0.09, 28.21)	1.52 (0.08, 41.28)	1.32 (0.07, 33.42)	1.67 (0.09, 40.93)	1.62 (0.13, 34.32)	0.45 (0.03, 10.37)	0.71 (0.05, 15.53)	0.86 (0.05, 20.47)	1.17 (0.07, 29.24)	4.35 (0.11, 282.33)	1.36 (0.08, 32.01)	0.70 (0.01, 33.90)	0.89 (0.05, 23.00)	1.47 (0.08, 36.81)	1.28 (0.07, 31.99)
CER200mgSC	0.66 (0.21, 2.06)	0.20 (0.01, 3.22)	0.80 (0.08, 9.91)	1.07 (0.12, 13.32)	0.68 (0.01, 28.31)	1.03 (0.06, 39.97)	0.97 (0.05, 39.30)	0.38 (0.10, 1.57)	0.47 (0.08, 2.80)	0.40 (0.07, 2.43)	0.51 (0.09, 3.22)	0.50 (0.17, 1.63)	**0.14 (0.04, 0.56)**	**0.22 (0.06, 0.87)**	0.26 (0.05, 1.50)	0.35 (0.07, 1.89)	1.24 (0.07, 41.43)	0.41 (0.08, 2.18)	0.22 (0.01, 3.79)	0.27 (0.05, 1.51)	0.45 (0.09, 2.49)	0.39 (0.07, 2.12)
CER20mgIV	0.44 (0.05, 2.83)	0.13 (0.00, 1.06)	0.53 (0.03, 8.53)	0.70 (0.05, 11.06)	0.44 (0.01, 23.14)	0.68 (0.03, 31.25)	0.66 (0.02, 28.68)	0.26 (0.03, 1.65)	0.31 (0.03, 2.74)	0.26 (0.02, 2.32)	0.33 (0.03, 3.07)	0.34 (0.04, 1.78)	**0.09 (0.01, 0.59)**	**0.15 (0.02, 0.93)**	0.17 (0.01, 1.43)	0.24 (0.02, 1.82)	0.83 (0.03, 30.74)	0.27 (0.02, 2.19)	0.14 (0.00, 3.84)	0.18 (0.01, 1.55)	0.30 (0.03, 2.36)	0.26 (0.02, 2.09)

(**TABLE S18M.** continued)

	CER400mgSC	CER5mgIV	ELD10mg_kgIV	ELD20mg_kgIV	FON0_1mg_kgIV	FON1mg_kgIV	FON4mg_kgIV	NAT300mgIV	NAT3mg_kgIV	NAT3mg_kgIVx2	NAT6mg_kgIVx2	PBO	RIS1200mgIV	RIS600mgIV	UST1mg_kgIV	UST3mg_kgIV	UST4_5mg_kgIV	UST6mg_kgIV	UST90mgSC	VED0_5mg_kgIV	VED2mg_kgIV	VED300mgIV
CER400mgSC	CER400mgSC	0.31 (0.01, 4.41)	1.20 (0.15, 13.02)	1.61 (0.21, 16.15)	1.05 (0.02, 39.45)	1.57 (0.11, 57.43)	1.45 (0.10, 52.78)	0.58 (0.21, 1.82)	0.70 (0.16, 3.35)	0.61 (0.13, 2.97)	0.77 (0.16, 3.78)	0.75 (0.36, 1.78)	**0.21 (0.07, 0.67)**	0.33 (0.11, 1.02)	0.40 (0.08, 1.79)	0.53 (0.13, 2.13)	1.90 (0.13, 54.91)	0.62 (0.15, 2.61)	0.32 (0.01, 5.01)	0.41 (0.09, 1.77)	0.67 (0.17, 2.92)	0.59 (0.14, 2.63)
CER5mgIV	3.24 (0.23, 106.02)	CER5mgIV	4.16 (0.15, 230.17)	5.41 (0.22, 259.01)	3.49 (0.04, 414.60)	5.46 (0.15, 606.12)	5.32 (0.13, 544.39)	1.91 (0.14, 65.62)	2.33 (0.13, 90.00)	1.97 (0.11, 76.65)	2.57 (0.14, 97.19)	2.44 (0.21, 76.60)	0.70 (0.05, 22.78)	1.07 (0.08, 34.50)	1.29 (0.08, 49.59)	1.77 (0.11, 66.07)	6.28 (0.17, 680.92)	1.99 (0.13, 77.66)	1.07 (0.01, 72.50)	1.37 (0.08, 54.45)	2.22 (0.14, 80.72)	1.97 (0.12, 74.56)
ELD10mg_kgIV	0.83 (0.08, 6.84)	0.24 (0.00, 6.86)	ELD10mg_kgIV	1.32 (0.23, 8.28)	0.85 (0.01, 45.21)	1.31 (0.04, 56.52)	1.29 (0.04, 49.50)	0.48 (0.05, 3.93)	0.58 (0.04, 6.68)	0.50 (0.04, 5.42)	0.64 (0.05, 6.83)	0.62 (0.07, 4.68)	0.18 (0.02, 1.54)	0.27 (0.03, 2.27)	0.33 (0.02, 3.46)	0.44 (0.03, 4.50)	1.51 (0.05, 73.37)	0.51 (0.04, 5.01)	0.26 (0.00, 7.35)	0.34 (0.03, 3.53)	0.56 (0.05, 5.77)	0.48 (0.04, 4.96)
ELD20mg_kgIV	0.62 (0.06, 4.83)	0.18 (0.00, 4.52)	0.76 (0.12, 4.35)	ELD20mg_kgIV	0.62 (0.01, 35.80)	1.00 (0.03, 47.38)	0.94 (0.03, 43.11)	0.36 (0.04, 2.72)	0.43 (0.04, 4.35)	0.37 (0.03, 3.96)	0.46 (0.04, 4.93)	0.47 (0.05, 3.13)	0.13 (0.01, 1.05)	0.21 (0.02, 1.54)	0.24 (0.02, 2.53)	0.33 (0.03, 3.11)	1.11 (0.04, 50.41)	0.38 (0.03, 3.48)	0.19 (0.00, 5.06)	0.25 (0.02, 2.42)	0.41 (0.04, 4.09)	0.36 (0.03, 3.43)
FON0_1mg_kgIV	0.95 (0.03, 45.43)	0.29 (0.00, 28.41)	1.17 (0.02, 100.99)	1.62 (0.03, 129.73)	FON0_1mg_kgIV	1.52 (0.11, 69.04)	1.44 (0.11, 51.96)	0.56 (0.02, 26.22)	0.69 (0.01, 42.11)	0.60 (0.01, 34.07)	0.74 (0.02, 43.42)	0.72 (0.02, 31.58)	0.20 (0.01, 9.94)	0.32 (0.01, 14.92)	0.38 (0.01, 20.03)	0.51 (0.01, 26.44)	2.10 (0.02, 235.71)	0.61 (0.01, 31.63)	0.29 (0.00, 31.69)	0.39 (0.01, 21.65)	0.66 (0.02, 35.29)	0.57 (0.01, 32.41)
FON1mg_kgIV	0.64 (0.02, 9.08)	0.18 (0.00, 6.84)	0.76 (0.02, 22.88)	1.00 (0.02, 30.94)	0.66 (0.01, 9.36)	FON1mg_kgIV	0.93 (0.09, 9.12)	0.38 (0.01, 5.29)	0.45 (0.01, 7.36)	0.39 (0.01, 6.41)	0.50 (0.01, 8.42)	0.48 (0.02, 6.32)	0.14 (0.00, 1.98)	0.21 (0.01, 3.03)	0.26 (0.01, 4.58)	0.34 (0.01, 5.68)	1.21 (0.02, 90.94)	0.39 (0.01, 6.66)	0.19 (0.00, 10.83)	0.26 (0.01, 4.42)	0.43 (0.01, 7.13)	0.37 (0.01, 6.44)
FON4mg_kgIV	0.69 (0.02, 10.36)	0.19 (0.00, 7.83)	0.77 (0.02, 27.58)	1.07 (0.02, 33.46)	0.70 (0.02, 9.26)	1.07 (0.11, 11.02)	FON4mg_kgIV	0.40 (0.01, 5.87)	0.49 (0.01, 9.14)	0.42 (0.01, 7.60)	0.52 (0.01, 9.93)	0.53 (0.02, 6.98)	0.15 (0.00, 2.18)	0.23 (0.01, 3.28)	0.27 (0.01, 4.87)	0.37 (0.01, 6.44)	1.34 (0.02, 75.91)	0.42 (0.01, 7.42)	0.20 (0.00, 9.63)	0.27 (0.01, 5.09)	0.46 (0.01, 8.23)	0.41 (0.01, 7.21)
NAT300mgIV	1.72 (0.55, 4.83)	0.52 (0.02, 7.23)	2.07 (0.25, 21.71)	2.75 (0.37, 25.83)	1.80 (0.04, 65.06)	2.64 (0.19, 89.99)	2.52 (0.17, 86.77)	NAT300mgIV	1.21 (0.26, 5.59)	1.04 (0.21, 4.71)	1.32 (0.26, 6.33)	1.29 (0.62, 2.63)	0.36 (0.13, 1.02)	0.56 (0.21, 1.57)	0.68 (0.15, 3.01)	0.93 (0.22, 3.53)	3.22 (0.22, 92.38)	1.05 (0.26, 4.24)	0.55 (0.01, 9.29)	0.71 (0.16, 2.91)	1.16 (0.29, 4.59)	1.02 (0.24, 4.09)
NAT3mg_kgIV	1.43 (0.30, 6.31)	0.43 (0.01, 7.43)	1.73 (0.15, 23.01)	2.31 (0.23, 27.61)	1.46 (0.02, 68.49)	2.22 (0.14, 102.00)	2.05 (0.11, 84.61)	0.83 (0.18, 3.83)	NAT3mg_kgIV	0.86 (0.22, 3.20)	1.08 (0.27, 4.27)	1.08 (0.28, 4.09)	0.30 (0.06, 1.41)	0.47 (0.10, 2.14)	0.56 (0.09, 3.49)	0.76 (0.12, 4.41)	2.76 (0.15, 92.03)	0.87 (0.15, 5.34)	0.45 (0.01, 8.98)	0.58 (0.09, 3.67)	0.96 (0.17, 5.63)	0.84 (0.14, 5.15)
NAT3mg_kgIVx2	1.64 (0.34, 7.76)	0.51 (0.01, 8.85)	2.01 (0.18, 26.78)	2.70 (0.25, 35.06)	1.68 (0.03, 75.90)	2.55 (0.16, 119.39)	2.40 (0.13, 97.36)	0.96 (0.21, 4.66)	1.16 (0.31, 4.51)	NAT3mg_kgIVx2	1.27 (0.31, 5.06)	1.24 (0.31, 4.98)	0.35 (0.07, 1.67)	0.54 (0.12, 2.54)	0.66 (0.10, 4.27)	0.88 (0.14, 5.33)	3.12 (0.17, 103.92)	1.00 (0.16, 6.34)	0.53 (0.01, 9.93)	0.67 (0.10, 4.31)	1.10 (0.19, 6.80)	0.97 (0.16, 5.71)
NAT6mg_kgIVx2	1.30 (0.26, 6.34)	0.39 (0.01, 6.95)	1.57 (0.15, 21.82)	2.15 (0.20, 27.27)	1.34 (0.02, 61.50)	2.02 (0.12, 85.61)	1.91 (0.10, 81.40)	0.76 (0.16, 3.91)	0.93 (0.23, 3.66)	0.79 (0.20, 3.26)	NAT6mg_kgIVx2	0.98 (0.25, 4.11)	0.28 (0.06, 1.40)	0.44 (0.09, 2.14)	0.52 (0.08, 3.57)	0.69 (0.10, 4.22)	2.48 (0.13, 76.08)	0.80 (0.13, 5.11)	0.41 (0.01, 8.27)	0.53 (0.08, 3.56)	0.87 (0.15, 5.63)	0.77 (0.13, 4.99)
PBO	1.34 (0.56, 2.81)	0.41 (0.01, 4.79)	1.62 (0.21, 14.78)	2.14 (0.32, 18.65)	1.40 (0.03, 47.66)	2.08 (0.16, 63.17)	1.90 (0.14, 62.77)	0.78 (0.38, 1.62)	0.93 (0.24, 3.55)	0.81 (0.20, 3.20)	1.02 (0.24, 4.00)	PBO	**0.28 (0.13, 0.60)**	**0.44 (0.21, 0.89)**	0.53 (0.14, 1.88)	0.72 (0.21, 2.22)	2.49 (0.19, 66.98)	0.82 (0.25, 2.66)	0.43 (0.01, 5.99)	0.55 (0.15, 1.90)	0.90 (0.28, 2.98)	0.79 (0.23, 2.63)
RIS1200mgIV	**4.68 (1.50, 13.92)**	1.43 (0.04, 19.61)	5.70 (0.65, 62.71)	7.55 (0.95, 78.02)	4.88 (0.10, 193.01)	7.29 (0.50, 249.67)	6.82 (0.46, 240.78)	2.76 (0.98, 7.66)	3.32 (0.71, 15.48)	2.88 (0.60, 13.71)	3.62 (0.72, 17.70)	**3.53 (1.67, 7.55)**	RIS1200mgIV	1.56 (0.70, 3.36)	1.88 (0.42, 8.01)	2.53 (0.61, 9.89)	9.05 (0.57, 269.56)	2.91 (0.73, 12.00)	1.52 (0.04, 24.44)	1.94 (0.43, 8.34)	3.19 (0.80, 12.56)	2.81 (0.65, 11.60)
RIS600mgIV	3.01 (0.98, 8.80)	0.93 (0.03, 12.19)	3.67 (0.44, 39.32)	4.88 (0.65, 51.15)	3.14 (0.07, 117.17)	4.66 (0.33, 158.96)	4.33 (0.30, 150.33)	1.79 (0.64, 4.81)	2.11 (0.47, 9.64)	1.84 (0.39, 8.69)	2.29 (0.47, 11.40)	**2.27 (1.12, 4.70)**	0.64 (0.30, 1.43)	RIS600mgIV	1.20 (0.27, 5.05)	1.62 (0.40, 6.25)	5.72 (0.39, 179.55)	1.86 (0.48, 7.33)	0.98 (0.03, 14.69)	1.24 (0.28, 5.20)	2.03 (0.54, 8.30)	1.78 (0.44, 7.24)
UST1mg_kgIV	2.49 (0.56, 11.93)	0.77 (0.02, 12.95)	3.05 (0.29, 40.88)	4.10 (0.40, 48.72)	2.63 (0.05, 107.14)	3.91 (0.22, 158.03)	3.65 (0.21, 140.37)	1.47 (0.33, 6.50)	1.79 (0.29, 11.61)	1.52 (0.23, 10.39)	1.93 (0.28, 13.33)	1.87 (0.53, 7.06)	0.53 (0.12, 2.38)	0.83 (0.20, 3.72)	UST1mg_kgIV	1.35 (0.36, 5.28)	4.71 (0.28, 174.43)	1.53 (0.42, 6.30)	0.82 (0.02, 14.73)	1.03 (0.17, 6.23)	1.70 (0.30, 10.20)	1.48 (0.26, 9.15)
UST3mg_kgIV	1.88 (0.47, 7.63)	0.57 (0.02, 9.11)	2.25 (0.22, 32.73)	3.03 (0.32, 36.49)	1.95 (0.04, 75.22)	2.92 (0.18, 105.45)	2.73 (0.16, 99.93)	1.07 (0.28, 4.45)	1.31 (0.23, 8.02)	1.14 (0.19, 7.13)	1.44 (0.24, 9.55)	1.39 (0.45, 4.83)	0.39 (0.10, 1.64)	0.62 (0.16, 2.49)	0.74 (0.19, 2.80)	UST3mg_kgIV	3.54 (0.20, 128.40)	1.14 (0.33, 4.16)	0.60 (0.02, 10.98)	0.77 (0.13, 4.54)	1.26 (0.24, 6.75)	1.10 (0.21, 6.40)
UST4_5mg_kgIV	0.53 (0.02, 7.89)	0.16 (0.00, 5.72)	0.66 (0.01, 18.88)	0.90 (0.02, 24.49)	0.48 (0.00, 48.77)	0.83 (0.01, 62.42)	0.75 (0.01, 63.69)	0.31 (0.01, 4.57)	0.36 (0.01, 6.72)	0.32 (0.01, 5.94)	0.40 (0.01, 7.62)	0.40 (0.01, 5.32)	0.11 (0.00, 1.74)	0.17 (0.01, 2.59)	0.21 (0.01, 3.56)	0.28 (0.01, 5.00)	UST4_5mg_kgIV	0.32 (0.01, 5.82)	0.16 (0.00, 6.28)	0.21 (0.01, 3.88)	0.36 (0.01, 6.10)	0.31 (0.01, 5.23)
UST6mg_kgIV	1.62 (0.38, 6.59)	0.50 (0.01, 7.53)	1.96 (0.20, 24.90)	2.63 (0.29, 29.69)	1.65 (0.03, 68.61)	2.57 (0.15, 97.84)	2.39 (0.13, 85.92)	0.96 (0.24, 3.81)	1.14 (0.19, 6.48)	1.00 (0.16, 6.08)	1.26 (0.20, 7.49)	1.23 (0.38, 4.02)	0.34 (0.08, 1.38)	0.54 (0.14, 2.08)	0.66 (0.16, 2.36)	0.87 (0.24, 3.05)	3.16 (0.17, 98.20)	UST6mg_kgIV	0.52 (0.01, 9.87)	0.67 (0.11, 3.97)	1.10 (0.21, 5.52)	0.96 (0.18, 5.00)
UST90mgSC	3.13 (0.20, 108.71)	0.93 (0.01, 71.63)	3.89 (0.14, 220.35)	5.15 (0.20, 290.92)	3.41 (0.03, 430.80)	5.39 (0.09, 747.84)	5.08 (0.10, 642.25)	1.81 (0.11, 69.91)	2.24 (0.11, 97.19)	1.90 (0.10, 82.89)	2.44 (0.12, 118.33)	2.34 (0.17, 79.63)	0.66 (0.04, 25.38)	1.03 (0.07, 38.72)	1.22 (0.07, 52.91)	1.66 (0.09, 64.50)	6.24 (0.16, 685.33)	1.93 (0.10, 78.86)	UST90mgSC	1.25 (0.07, 53.22)	2.08 (0.12, 88.74)	1.85 (0.11, 75.17)
VED0_5mg_kgIV	2.44 (0.56, 10.72)	0.73 (0.02, 12.72)	2.94 (0.28, 38.88)	3.93 (0.41, 51.23)	2.55 (0.05, 111.09)	3.89 (0.23, 146.58)	3.66 (0.20, 139.14)	1.42 (0.34, 6.37)	1.72 (0.27, 10.87)	1.50 (0.23, 9.95)	1.88 (0.28, 12.78)	1.82 (0.53, 6.77)	0.51 (0.12, 2.33)	0.81 (0.19, 3.62)	0.97 (0.16, 6.01)	1.30 (0.22, 7.53)	4.73 (0.26, 140.11)	1.49 (0.25, 8.93)	0.80 (0.02, 14.94)	VED0_5mg_kgIV	1.65 (0.47, 6.28)	1.44 (0.26, 9.17)
VED2mg_kgIV	1.49 (0.34, 5.77)	0.45 (0.01, 6.93)	1.79 (0.17, 21.92)	2.42 (0.24, 27.99)	1.52 (0.03, 64.69)	2.34 (0.14, 86.79)	2.18 (0.12, 81.71)	0.87 (0.22, 3.46)	1.04 (0.18, 5.85)	0.91 (0.15, 5.30)	1.15 (0.18, 6.70)	1.11 (0.34, 3.56)	0.31 (0.08, 1.25)	0.49 (0.12, 1.85)	0.59 (0.10, 3.35)	0.79 (0.15, 4.16)	2.80 (0.16, 90.43)	0.91 (0.18, 4.87)	0.48 (0.01, 8.10)	0.61 (0.16, 2.11)	VED2mg_kgIV	0.87 (0.16, 4.85)
VED300mgIV	1.71 (0.38, 7.02)	0.51 (0.01, 8.30)	2.07 (0.20, 26.03)	2.77 (0.29, 31.54)	1.76 (0.03, 76.17)	2.67 (0.16, 104.69)	2.44 (0.14, 92.10)	0.98 (0.24, 4.13)	1.19 (0.19, 7.26)	1.03 (0.18, 6.15)	1.29 (0.20, 7.99)	1.27 (0.38, 4.31)	0.36 (0.09, 1.54)	0.56 (0.14, 2.26)	0.68 (0.11, 3.84)	0.91 (0.16, 4.80)	3.21 (0.19, 103.40)	1.04 (0.20, 5.62)	0.54 (0.01, 9.08)	0.70 (0.11, 3.83)	1.15 (0.21, 6.31)	VED300mgIV

Table S18n: League table for serious adverse events for the some concerns studies. The table presented the multiple treatment comparisons based on consistency analysis of the networks. Treatments are depicted alphabetically. Values are presented as relative risk (RR) with 95% credible interval (CrI). For all comparisons an RR >1 favors the occurrence of the event for the row-defining treatment, while an RR<1 favors the column-defining treatment. Statistically significant results are represented in bold. Multiple treatment comparisons based on consistency analysis of the networks. Treatments are depicted alphabetically. Values are presented as relative risk (RR) with 95% credible interval (CrI). For all comparisons an RR <1 favors the occurrence of the event for the row-defining treatment, while an RR>1 favors the column-defining treatment. Statistically significant results are represented in bold.

	ETA25mgSC	MED700IV	PBO
ETA25mgSC	ETA25mgSC	2.80 (0.15, 85.60)	2.54 (0.21, 65.66)
MED700IV	0.36 (0.01, 6.83)	MED700IV	0.97 (0.23, 4.14)
PBO	0.39 (0.02, 4.74)	1.03 (0.24, 4.34)	PBO

TABLE S19. CINEMA ANALYSIS. For better visualization access the excel version using the LINK

TABLE S19A. CINEMA ANALYSIS FOR CDAI

treatment with dose	Overall
ABA10mg/kgIV	Low
ABA30mg/kgIV	Low
ABA3mg/kgIV	Low
ADA160mg80mg40mgSC	Moderate
ADA160mg80mg60mgSC	Moderate
ADA160mg80mgSC	Moderate
ADA40mg20mgSC	Low
ADA80mg40mgSC	Low
AMI0.4mgPO	Low
AND150mgSC1/1	Low
AND150mgSC2/2	Low
AND300mgSC	Low
API100mgPO	Low
API50mgPO	Low
BRI400mgIV	Low
BRI700mgIV	Low
BRO210mgIV	Low
BRO350mgIV	Low
BRO700mgIV	Low
CDP10mg/kgIV	Low
CER100mgSC	Low
CER10mg/kgIV	Low
CER200mgSC	Low
CER20mg/kgIV	Low
CER400mgSC	Low
CER5mg/kgIV	Low
ELD10mg/kgIV	Low
ELD20mg/kgIV	Low
ETA25mgSC	Low
ETR105mgSC	Moderate
ETR210mgSC	Low
FIL200mgPO	Moderate
FON0.1mg/kgIV	Low
FON1mg/kg0.1mgIV/SC	Low
FON1mg/kg1mgIV/SC	Low
FON1mg/kgIV	Low

treatment with dose	Overall
FON4mg/kg0.1mgIV/SC	Low
FON4mg/kg1mgIV/SC	Low
FON4mg/kgIV	Low
GUS1200mgIV	Moderate
GUS200mgIV	Moderate
GUS600mgIV	Moderate
INF10mg/kgIV	Low
INF20mg/kgIV	Low
INF5mg/kgIV	Low
MED700IV	Moderate
MIR1000mgIV	Moderate
MIR200mgIV	Moderate
MIR600mgIV	Moderate
NAT300mgIV	Moderate
NAT3mg/kgIV	Low
NAT3mg/kgIVx2	Low
NAT6mg/kgIVx2	Low
NNC2mg/kgSC	Low
ONE10mgSC	Low
ONE25mgSC	Low
ONE35mgSC	Low
ONE50mgSC	Low
ONT225mgSC	Low
ONT22.5mgSC	Moderate
ONT75mgSC	Moderate
PBO	Moderate
PF10mgSC	Low
PF200mgSC	Moderate
RIS1200mgIV	Moderate
RIS200mgIV	Low
RIS600mgIV	Low
TOF15mgPO_BID	Moderate
TOF1mgPO_BID	Moderate
UPA12mgPO_BID	Moderate
UPA24mgPO	Moderate
UPA24mgPO_BID	Moderate
UPA3mgPO_BID	Moderate
UST130mgIV	Moderate
UST1mg/kgIV	Low

treatment with dose	Overall
UST3mg/kgIV	Low
VED0.5mg/kgIV	Low
PF50mgSC	Low
RIS600mgIV4/4	Moderate
SEC10mg/kgIV	Moderate
TES400mg200mgSC	Low
TOF5mgPO_BID	Moderate
UPA45mgPO	Low
UPA6mgPO_BID	Moderate
UST4.5mg/kgIV	Moderate
UST6mg/kg90mgIV/SC	Low
UST6mg/kgIV	Moderate
UST90mgSC	Low
VED2mg/kgIV	Low

TABLE S19B. CINEMA ANALYSIS FOR IBDQ

treatment with dose	Overall
CER400mgSC	Very low
FIL200mgOR	Very low
FON1mg/kg0.1mgIV/SC	Very low
FON1mg/kg1mgIV/SC	Very low
FON4mg/kg0.1mgIV/SC	Very low
FON4mg/kg1mgIV/SC	Very low
INF10mg/kgIV	Very low
INF20mg/kgIV	Very low
INF5mg/kgIV	Very low
NAT300mgIV	Very low
PBO	Very low
RIS200mgIV	Low
SEM60mgIV	Very low
UPA12mgOR_BID	Low
UPA24mgOR	Low
UPA24mgOR_BID	Low
UPA3mgOR_BID	Low
UST130mgIV	Low
RIS600mgIV	Low
SEM60mgmgIVx3	Very low
UPA45mgOR	Very low
UPA6mgOR_BID	Low

TABLE S19C. CINEMA ANALYSIS FOR SAFETY

treatment with dose	Overall
ABA10mg/kgIV	Low
ABA30mg/kgIV	Low
ABA3mg/kgIV	Low
ADA160mg80mg40mgSC	Moderate
ADA160mg80mg60mgSC	Moderate
ADA160mg80mgSC	Low
ADA40mg20mgSC	Low
ADA80mg40mgSC	Low
AMI0.4mgPO	Low
AND150mgSC1/1	Low
AND150mgSC2/2	Low
AND300mgSC	Low
API100mgPR	Low
API50mgPO	Low
BRI400mgIV	Low
BRI700mgIV	Low
BRO210mgIV	Low
BRO350mgIV	Low
BRO700mgIV	Low
CDP10mg/kgIV	Low
CER100mgSC	Low
CER10mgIV	Low
CER200mgSC	Low
CER20mgIV	Low
CER400mgSC	Low
CER5mgIV	Low
ELD10mg/kgIV	Low
ELD20mg/kgIV	Low
ETA25mgSC	Low
ETR105mgSC	Moderate
ETR210mgSC	Moderate
FIL200mgPO	Moderate
FON0.1mg/kgIV	Low
FON1mg/kgIV	Low
FON4mg/kgIV	Low
GUS1200mgIV	Moderate
GUS200mgIV	Moderate

treatment with dose	Overall
GUS600mgIV	Moderate
MED700IV	Low
MIR1000mgIV	Moderate
MIR200mgIV	Moderate
MIR600mgIV	Moderate
NAT300mgIV	Low
NAT3mg/kgIV	Low
NAT3mg/kgIVx2	Low
NAT6mg/kgIVx2	Low
NNC2mg/kgSC	Moderate
ONE10mgSC	Moderate
ONE25mgSC	Moderate
ONE35mgSC	Moderate
ONE50mgSC	Moderate
ONT225mgSC	Moderate
ONT22.5mgSC	Moderate
ONT75mgSC	Moderate
PBO	Moderate
PF10mgSC	Moderate
PF200mgSC	Moderate
RIS1200mgIV	Moderate
RIS200mgIV	Low
TOF15mgPO_BID	Moderate
TOF1mgPO_BID	Moderate
UPA12mgPO_BID	Moderate
UPA24mgPO	Moderate
UPA24mgPO_BID	Moderate
UPA3mgPO_BID	Moderate
UST130mgIV	Moderate
UST1mg/kgIV	Low
UST3mg/kgIV	Low
VED0.5mg/kgIV	Moderate
PF50mgSC	Moderate
RIS600mgIV	Moderate
SEC10mg/kgIV	Moderate
TES400mg200mgSC	Moderate
TOF5mgPO_BID	Moderate
UPA45mgPO	Moderate
UPA6mgPO_BID	Moderate
UST4.5mg/kgIV	Low
UST6mg/kg90mgIV/SC	Moderate
UST6mg/kgIV	Moderate
UST90mgSC	Low

treatment with dose	Overall
VED2mg/kgIV	Moderate
